# SLIT2 and ANGPTL3 as putative mediators linking obesity to atrial fibrillation: A Mendelian randomization study

**DOI:** 10.1097/MD.0000000000049568

**Published:** 2026-07-03

**Authors:** Yanping Wu, Ying Peng, Qing Zhang, Wen Xu, Qianyuan Li, Xiaodi Chen, Zhiyang Lv

**Affiliations:** a Department of Cardiology, The First College of Clinical Medical Science, China Three Gorges University and Yichang Central People’s Hospital, Yichang, Hubei, China; b Institute of Cardiovascular Diseases, China Three Gorges University, Yichang, Hubei, China; c Hubei Key Laboratory of Ischemic Cardiovascular Disease, Yichang, Hubei, China; d Hubei Provincial Clinical Research Center for Ischemic Cardiovascular Disease, Yichang, Hubei, China; e Department of Cardiology, Zhongxiang People’s Hospital, Zhongxiang, Hubei, China; f Department of Medical Record, The First College of Clinical Medical Science, China Three Gorges University and Yichang Central People’s Hospital, Yichang, Hubei, China; g Clinical Laboratory, The First College of Clinical Medical Science, China Three Gorges University and Yichang Central People’s Hospital, Yichang, Hubei, China; h Department of Ultrasound, The First College of Clinical Medical Science, China Three Gorges University and Yichang Central People’s Hospital, Yichang, Hubei, China.

**Keywords:** ANGPTL3, atrial fibrillation, BMI, Mendelian randomization, SLIT2

## Abstract

The observational association between obesity and atrial fibrillation has been well-established. However, its causal mediators underlying this association remain unclear. This study aimed to investigate the causal relationship between body mass index (BMI), as well as potential mediating factors underlying this association, using Mendelian randomization (MR). Genetically predicted BMI was significantly associated with an increased risk of atrial fibrillation (inverse-variance weighted odds ratio [OR] = 1.350, 95% confidence interval [CI] = 1.268–1.436, *P* < .001). Sensitivity analyses using multiple methods yielded consistent results, and no evidence of directional pleiotropy was detected after outlier correction. A total of 91 inflammatory cytokines and 4907 plasma proteins were included as potential mediators. Among the screened mediators, SLIT2 exhibited a significant inverse mediating effect on the association between BMI and atrial fibrillation (OR = 1.019, 95% CI = 1.005–1.034), accounting for 7% of the total effect. These results suggest that higher BMI is associated with reduced SLIT2 expression, which in turn increases the risk of atrial fibrillation. In contrast, ANGPTL3 showed a significant positive mediating effect (OR = 1.010, 95% CI = 1.003–1.017), accounting for 3.64% of the total effect, indicating that elevated BMI increases ANGPTL3 levels, thereby further increasing the risk of atrial fibrillation. This MR study provides genetic evidence supporting a causal association between obesity and atrial fibrillation and suggests that SLIT2 and ANGPTL3 may be involved as mediators linking obesity-related inflammation and lipid dysregulation to atrial fibrillation susceptibility.

First, a 2-sample MR analysis was conducted using genome-wide association studies meta-analysis of European ancestry to estimate the causal effect of genetically proxied BMI on atrial fibrillation. Subsequently, inflammatory cytokines and plasma proteins were considered as candidate mediators, with their cis-protein quantitative trait locus used as instrumental variables. The inverse-variance weighted method was applied as the primary MR approach, while MR-Egger, weighted median, weighted mode, MR-Pleiotropy Residual Sum and Outlier, and leave-one-out methods were used for sensitivity analyses. Shared causal variants were further evaluated using false discovery rate correction and colocalization analysis within a ± 500 kb window (PPH4 > 0.75).

## 1. Introduction

Atrial fibrillation is the most common sustained arrhythmia in clinical practice.^[1]^ The global burden of atrial fibrillation has increased substantially in recent decades, with an estimated 50 million individuals affected worldwide, and both incidence and prevalence continuing to rise. Atrial fibrillation is associated with a markedly increased risk of mortality, stroke, and peripheral embolism.^[2]^ This rising burden is driven by multiple factors, including population aging, increasing prevalence of obesity, advances in diagnostic technology, and improved survival from other cardiovascular diseases.

Obesity has emerged as a major global public health challenge, with its prevalence nearly tripling since 1975 and projections suggesting that more than half of the global population may be affected by 2030.^[3]^ Obesity is a well-established risk factor for numerous chronic conditions, including type 2 diabetes and cardiovascular disease,^[4]^ and has been consistently associated with an increased risk of atrial fibrillation.^[5]^ Data from the Framingham Heart Study demonstrated that each 1-standard deviation (SD) increase in body mass index (BMI) was associated with a 4% higher risk of atrial fibrillation.^[6]^ Although obesity independently predicts the development and progression of atrial fibrillation,^[7]^ the underlying pathophysiological mechanisms remain incompletely understood.

Several obesity-related systemic and molecular mechanisms have been proposed, including hemodynamic alterations,^[8,9]^ hypertension, diabetes, and obstructive sleep apnea syndrome.^[10–12]^ From a molecular biology perspective, this may be related to the secretion of multiple pro-inflammatory factors^[13]^ and protein-mediated processes by adipose tissue,^[14–16]^ as well as abnormalities in plasma proteins caused by metabolic disorders in obese individuals. These processes may contribute to atrial remodeling, inflammatory activation, myocardial fibrosis, and electrical conduction abnormalities,^[3,17]^ ultimately promoting the initiation and persistence of atrial fibrillation. However, the specific circulating inflammatory factors and plasma proteins that play pivotal causal roles in obesity-related atrial fibrillation have not yet been clearly elucidated. This study aims to identify and validate potential plasma protein mediators linking obesity and atrial fibrillation through Mendelian randomization (MR) analysis.

Traditional methods may fail to adequately establish observed associations due to potential confounding factors or the ease of establishing bidirectional causality. However, MR effectively overcomes the limitations of confounding and bidirectional causality in traditional observational studies by utilizing genetic variants as instrumental variables (IVs) to infer causality. A 2-sample MR (TSMR) study by the Mi Ma team found a positive causal relationship between BMI and atrial fibrillation, and pointed out that for every 1-SD increase in BMI, the risk of atrial fibrillation increased by an average of 42.5%.^[18]^ However, this study focused primarily on the total causal effect of BMI on atrial fibrillation and did not investigate potential mediating pathways or underlying molecular mechanisms.

In the present study, we conducted a 2-step MR analysis to further clarify the causal relationship between obesity and atrial fibrillation. First, we confirmed the causal effect of genetically predicted BMI on atrial fibrillation using large-scale genome-wide association study (GWAS) data. We then performed mediation analyses to identify circulating inflammatory cytokines and plasma proteins that may act as intermediate mediators linking obesity to atrial fibrillation. This work aims to provide mechanistic insights into obesity-related atrial fibrillation and to improve understanding of its potential biological pathways.

## 2. Methods

### 2.1. Study design

A TSMR study was conducted using publicly available pooled data from GWAS. Based on prior epidemiological evidence regarding risk factors for atrial fibrillation, potential mediators were categorized into 2 groups: inflammatory cytokines and plasma-derived regulatory proteins. The inflammatory cytokines included CD40, FLT3L, GDNF, PD-L1, SCF, TNFSF12, CXCL5, IL-33, and NT-3, while plasma proteins included SLIT2, PPBP, ANGPTL3, and FAM177A1.

First, univariable MR analyses were performed to estimate the total causal effect of BMI on atrial fibrillation. Subsequently, causal associations between BMI and each potential mediator, as well as between each mediator and atrial fibrillation, were evaluated to identify significant mediators. Finally, a 2-step MR mediation analysis was conducted to assess the mediating effects of these significant mediators on the association between BMI and atrial fibrillation. All mediators were assessed individually rather than jointly.

### 2.2. Data sources

All GWAS summary statistics used in this study were obtained from the GWAS Catalog (http://www.ebi.ac.uk/gwas/), which integrates standardized GWAS results from published literature, the United Kingdom Biobank, and the Finnish Gene Pool. Detailed information on all datasets is provided in Table S1, Supplemental Digital Content 1.

The GWAS dataset for BMI included 532,396 individuals and identified 396 genome-wide significant single nucleotide polymorphisms (SNPs) (Data S1, Supplemental Digital Content 2). The atrial fibrillation GWAS dataset comprised 537,409 individuals. Plasma protein quantitative trait locus (pQTL) data included 4907 circulating proteins measured in 35,559 individuals (Data S2, Supplemental Digital Content 3). The inflammatory cytokine GWAS dataset comprised 91 circulating inflammatory biomarkers measured in 8293 individuals.

All datasets were restricted to individuals of European ancestry to minimize population stratification bias. All data were obtained from publicly available sources, were anonymized, and had received appropriate ethical approval; therefore, no additional ethical approval was required for this study.

### 2.3. Genetic tool selection

This study adheres to the 3 core assumptions of MR^[19]^: genetic instruments are robustly associated with the exposure, geneticinstruments are independent of confounding factors, genetic instruments influence the outcome only through the exposure..

For BMI, SNPs significantly associated with BMI were selected from GWAS datasets of European ancestry populations using a genome-wide significance threshold of *P* < 5 × 10^−8^. For candidate mediators (91 inflammatory cytokines and 4907 plasma proteins), cis-pQTLs meeting the same significance threshold were selected as IVs. Cis-pQTLs were required to be located within ± 500 kb of the transcription start site of the corresponding gene to minimize confounding from long-range regulatory effects.

For inflammatory cytokines lacking sufficient independent cis-pQTLs at the genome-wide significance threshold, the threshold was relaxed to *P* < 5 × 10^−5^ to maintain statistical power, as supported by previous MR studies. Linkage disequilibrium pruning was applied to ensure independence among instruments (*R*^2^ < 0.05).

### 2.4. Statistical analyses

#### 2.4.1. Primary analyses

All analyses were performed using the TSMR R package (MRC Integrative Epidemiology Unit [MRC IEU], University of Bristol). BMI-associated SNPs were used as IVs, and the inverse-variance weighted (IVW) method served as the primary analysis to estimate odds ratio (OR) and 95% confidence interval (CI) for atrial fibrillation per 1-SD increase in genetically predicted BMI.

Univariable IVW analyses were subsequently conducted for each candidate mediator. Mediators that showed significant associations in both the BMI → mediator and mediator → atrial fibrillation analyses (*P* < .05) were included in mediation analyses.^[20]^ Mediation effects were estimated using a 2-step MR framework: the causal effect of BMI on each mediator (*β*_1_) and the causal effect of each mediator on atrial fibrillation (*β*_2_) were estimated separately. The indirect effect was calculated as *β*_1_ × *β*_2_, and the mediation proportion was calculated as (*β*_1_ × *β*_2_)/*β*(total).

Where *β* (total) represents the total causal effect of BMI on atrial fibrillation estimated using the IVW method. Each mediator was assessed individually rather than jointly in the mediation analyses.

#### 2.4.2. Sensitivity analysis

The Cochran *Q* test was employed to assess heterogeneity among IVs, with *P* < .05 indicating significant heterogeneity. When heterogeneity was detected, the random-effects IVW method was applied instead of the fixed-effects IVW method to reestimate the causal effect. Concurrently, leave-one-out analyses were conducted by sequentially excluding individual IVs and repeating the IVW analysis. Directional horizontal pleiotropy was assessed using the MR-Egger test. Finally, colocalization analysis was employed to rule out the possibility of spurious associations arising from horizontal pleiotropy.

## 3. Results

### 3.1. Univariate MR study: causal association between BMI and atrial fibrillation

**Figure 1.**
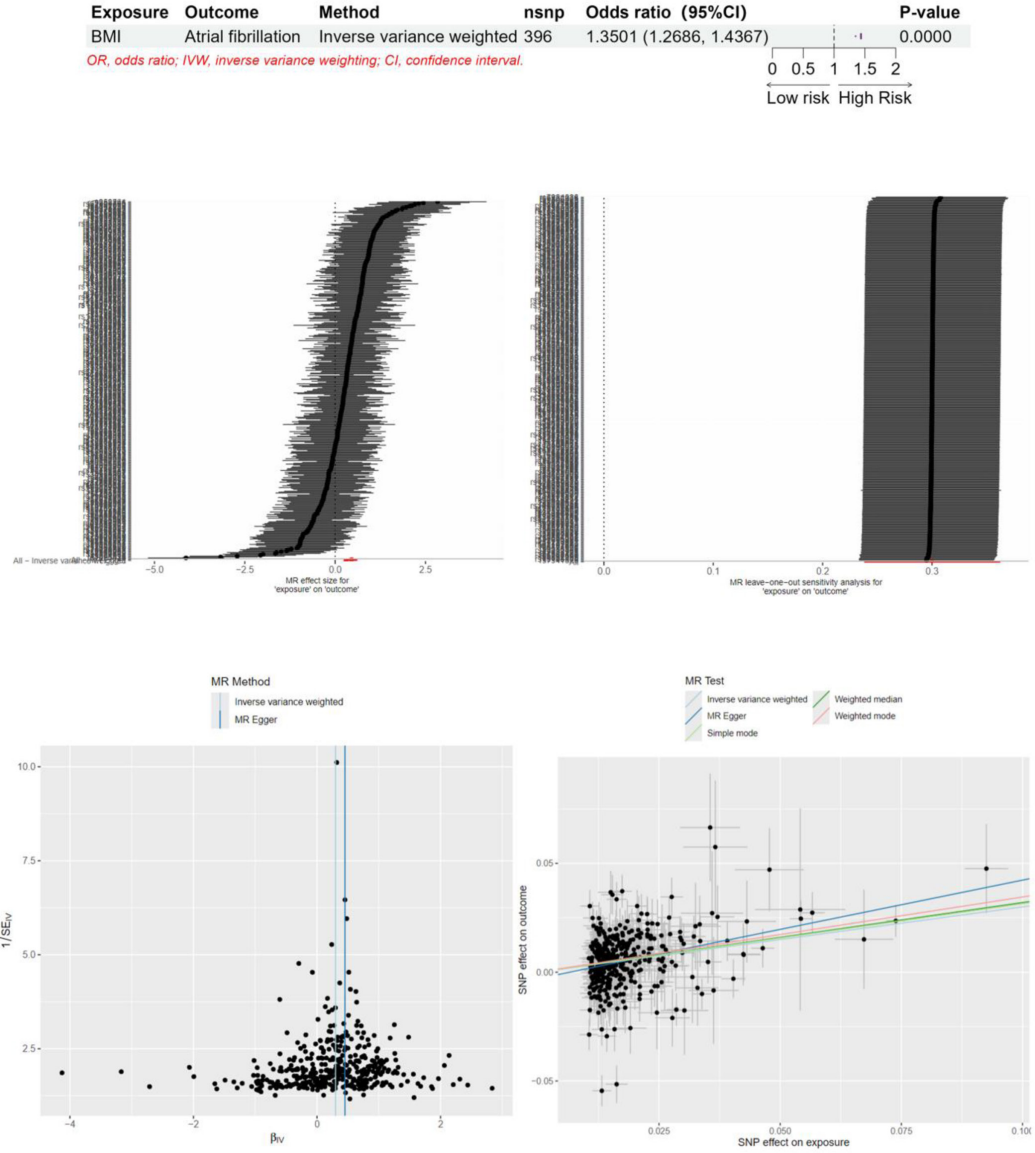
Analysis of the significant correlation between BMI and AF: A total of 396 SNPs were included as IVs. The IVW method was used as the primary MR approach, while the weighted median, MR-Egger, and weighted mode were applied for sensitivity analyses. The results demonstrated a significant genetic causal association between elevated BMI and an increased risk of AF, with consistent findings across multiple analytical methods. AF = atrial fibrillation, BMI = body mass index, CI = confidence interval, IV = instrumental variable, IVW = inverse-variance weighted, MR = Mendelian randomization, nSNP = number of single nucleotide polymorphism, OR = odds ratio, SNP = single nucleotide polymorphism.

As shown in Figure 1, IVW analysis demonstrated that each 1-SD increase in BMI was associated with a significantly increased risk of atrial fibrillation (OR = 1.350, 95% CI = 1.268–1.436, *P* < .001). Consistent results were observed using the MR-Egger method (OR = 1.572, 95% CI = 1.337–1.849, *P* < .001) and the weighted median method (OR = 1.377, 95% CI = 1.263–1.501, *P* < .001), supporting a causal association between BMI and atrial fibrillation. No evidence of reverse causality was detected (OR = 0.995, 95% CI = 0.986–1.005, *P* = .58), although heterogeneity was observed.

### 3.2. Univariate MR study: association between potential mediating factors and atrial fibrillation

**Figure 2.**
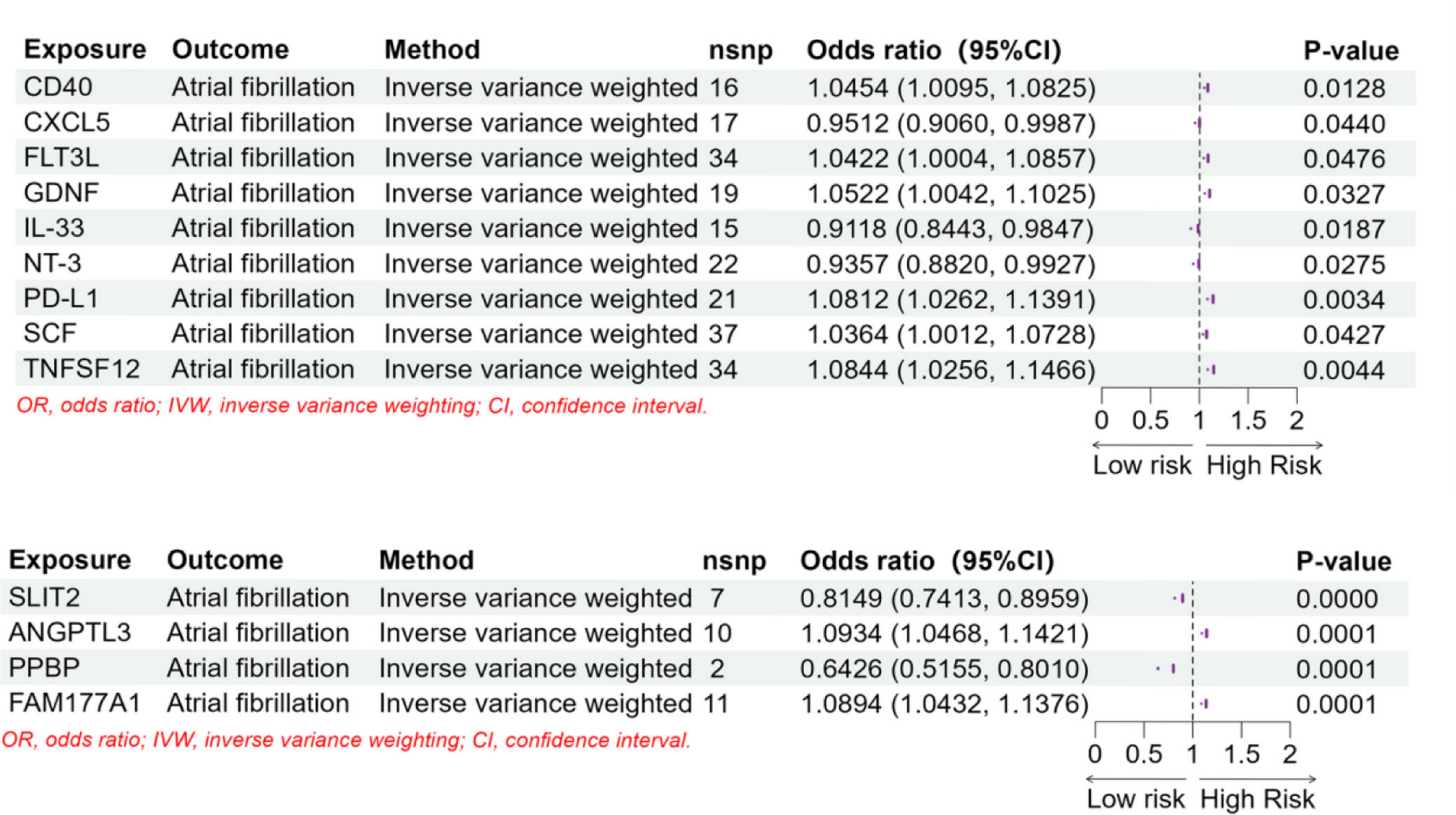
Mediating moderating correlation analysis forest plot. The forest plot illustrates the genetic associations between AF and various potential biomarkers (e.g., cytokines, metabolism-related proteins) beyond BMI. This analysis aims to identify key intermediate regulatory factors that may mediate the obesity-induced AF pathway and to provide candidate molecular targets for subsequent mechanism studies. Molecular Mechanism Diagram of Obesity-induced AF (Dual Mediator Regulation by SLIT2/ANGPTL3). AF = atrial fibrillation, BMI = body mass index, CI = confidence interval, IVW = inverse-variance weighted, nSNP = number of single nucleotide polymorphism, OR = odds ratio.

Figure 2 presents the results of univariable MR analyses evaluating the associations between potential mediators and atrial fibrillation. Genetically predicted levels of ANGPTL3 (OR = 1.093, 95% CI = 1.046–1.142, *P* < .001), FAM177A1 (OR = 1.089, 95% CI = 1.043–1.137, *P* < .001), TNFSF12 (OR = 1.084, 95%CI = 1.025–1.146, *P* = .004), PD-L1 (OR = 1.081, 95% CI = 1.026–1.139, *P* = .003), GDNF (OR = 1.052, 95% CI= 1.004–1.102, *P* = .032), CD40 (OR = 1.045, 95% CI = 1.009–1.082, *P* = .012), FLT3L (OR = 1.042, 95% CI = 1.000–1.085, *P* = .047), and SCF (OR = 1.036, 95% CI = 1.001–1.072, *P* = .042) were positively associated with the risk of atrial fibrillation.

In contrast, genetically predicted levels of CXCL5 (OR = 0.951, 95% CI = 0.906–0.998, *P* = .044), NT-3 (OR= 0.935, 95% CI = 0.882–0.992, *P* = .027), IL-33 (OR = 0.911, 95% CI = 0.844–0.984, *P* = .018), SLIT2 (OR = 0.814, 95% CI = 0.741–0.895, *P* < .001), and PPBP (OR = 0.642, 95% CI = 0.515–0.801, *P* < .001) were inversely associated with atrial fibrillation risk.

### 3.3. TSMR study: the association between BMI and atrial fibrillation

Following preliminary screening, univariable MR analyses identified several factors associated with both BMI and atrial fibrillation, including CD40, FLT3L, GDNF, PD-L1, SCF, TNFSF12, ANGPTL3, FAM177A1, CXCL5, IL-33, NT-3, SLIT2, and PPBP. To further evaluate their potential mediating roles, TSMR mediation analyses were performed.

As summarized in Table 1, SLIT2 (OR = 1.019, 95% CI = 1.005–1.034, *P* < .001) and ANGPTL3 (OR = 1.010, 95% CI = 1.003–1.017, *P* < .001) showed significant mediating effects, accounting for approximately 7% and 3.64% of the total effect, respectively. These results suggest that genetically predicted higher BMI is associated with reduced SLIT2 levels, which in turn are associated with an increased risk of atrial fibrillation, indicating a negative mediating role of SLIT2. In contrast, ANGPTL3 exhibited a positive mediating effect in the association between BMI and atrial fibrillation.

**Table 1:** Mediation analysis of the causal pathway from BMI to AF.

Exposure	Mediator	Outcome	Mediation effect	*P*	OR (95% CI)	Proportion of the direct effect	Proportion of the total effect mediated
BMI	CD40	AF	0.0013	.4337	-	-	-
BMI	FLT3L	AF	−0.0004	.8104	-	-	-
BMI	GDNF	AF	0.0028	.2521	-	-	-
BMI	PD-L1	AF	0.0015	.6251	-	-	-
BMI	SCF	AF	−0.0042	.0986	-	-	-
BMI	TNFSF12	AF	−0.0047 (−0.0187–−0.0004)	.0409	0.9905 (0.9815–0.9996)	0.3097	Suppression effect observed
BMI	CXCL5	AF	0.0015	.5251	-	-	-
BMI	IL-33	AF	−0.0023	.5653	-	-	-
BMI	NT-3	AF	0.0036	.2209	-	-	-
BMI	SLIT2	AF	0.0197 (0.0053–0.0340)	.0071	1.0198 (1.0054–1.0345)	0.2805	7%
BMI	PPBP	AF	−0.0150	.2699	-	-	-
BMI	ANGPTL3	AF	0.0106 (0.0034–0.0177)	.0039	1.0106 (1.0034–1.0179)	0.2896	3.64%
BMI	FAM177A1	AF	−0.0110 (−0.0188–−0.0034)	.0049	0.9890 (0.9814–0.9966)	0.3112	Suppression effect observed

AF = atrial fibrillation, BMI = body mass index, CI = confidence interval, OR = odds ratio, SNP = single nucleotide polymorphism.

*P* serves as a statistical measure of the significance of the association between a SNP and a particular trait.“-” indicates no significant analytical meaning.

## 4. Discussion

Our study aimed to validate the significant association between obesity and atrial fibrillation using MR methods and to identify potential mediating factors. The findings confirmed a significant causal relationship between elevated BMI and an increased risk of atrial fibrillation. Furthermore, mediation analysis suggested that SLIT2 and ANGPTL3 may mediate this pathway. Specifically, SLIT2 exhibited a negative mediating effect, while ANGPTL3 demonstrated a positive mediating effect, accounting for approximately 7% and 3.64% of the mediating effect, respectively. These findings suggest that inflammation-related and lipid metabolism-related pathways may contribute to the development of obesity-associated atrial fibrillation.

As a member of the SLIT family of secreted proteins, Darrell Pilling et al identified SLIT2 as a key signal preventing monocytes from entering fibroblasts in normal tissues. Their study revealed that SLIT2 is derived from fibroblasts, and its upregulation inhibits human fibroblast differentiation, thereby suppressing fibrosis.^[21]^ However, in liver fibrosis, SLIT2 exhibits the opposite function compared to its role in inhibiting pulmonary fibrosis.^[22]^ Liu team demonstrated that SLIT2-Robo1 signaling is activated in fibrotic cardiac tissue. SLIT2 exerts its pro-fibrotic effects at least partially by activating TGF-*β*1-Smad, PI3K/Akt, and periostein signaling pathways, indicating that this signaling pathway can promote cardiac fibrosis.^[23]^ Atrial tissue fibrosis affecting the left atrium is a primary determinant of atrial fibrillation progression. Chronic low-grade inflammation and adipose tissue dysfunction in obesity, particularly abnormal visceral fat hyperplasia, lead to the secretion of substantial pro-inflammatorycytokines (such as TNF-α and IL-6). This process is also intrinsically linked to fibrosis. In the present study, genetically predicted circulating SLIT2 levels were associated with atrial fibrillation risk, which differs from previous reports describing “local pro-fibrotic effects of SLIT2 in cardiac tissue.” However, this reflects its tissue-specific functions: local myocardial SLIT2 directly promotes fibrosis via the Robo1-TGF-*β*1 pathway; circulating SLIT2 suppresses monocyte infiltration into the myocardium, whereas reduced circulating SLIT2 in obesity enhances monocyte-mediated myocardial inflammation. Genetic evidence from this study suggests that reduced circulating SLIT2 levels in obesity may be associated with inflammatory processes that contribute to atrial remodeling. This mechanism is not opposed to local pro-fibrotic effects but rather reflects a “systemic-local” synergistic regulatory process.

ANGPTL3, as a liver-derived circulating protein,^[24]^ has been shown to play a significant role in cardiovascular diseases and holds promise as a therapeutic target for reducing cardiovascular risk.^[25]^ Current research on this plasma-derived regulatory protein has primarily focused on hepatic steatosis and coronary atherosclerotic heart disease.^[26–28]^ A higher BMI is often accompanied by systemic low-grade inflammation, insulin resistance, and lipid metabolism disorders. Research has demonstrated that these conditions can disrupt hepatic lipid homeostasis. Furthermore, elevated levels of ANGPTL3 (a key inhibitor of lipoprotein lipase and endothelial lipase) significantly suppress the hydrolysis and clearance of triglyceride-rich lipoproteins. This leads to increased circulating triglyceride and very low-density lipoprotein levels while simultaneously reducing high-density lipoprotein cholesterol levels. This process is closely associated with atrial fibrosis, inflammation, and oxidative stress. TSMR analysis identified the positive mediating role of ANGPTL3, revealing a “metabolic-electrical remodeling” pathway in obesity-associated atrial fibrillation. Obesity upregulates hepatic ANGPTL3 expression via insulin resistance. Acting as a lipoprotein lipase/endothelial lipase inhibitor, ANGPTL3 significantly reduces triglyceride hydrolysis and clearance, leading to elevated circulating triglycerides. Conversely, elevated triglyceride levels activate endoplasmic reticulum stress in atrial myocytes via lipotoxicity, downregulating Kv1.5 potassium channel expression and inducing electrical conduction abnormalities. Together, these findings support a potential role of ANGPTL3 in metabolic pathways linking obesity to atrial fibrillation.

In summary, the SLIT2-mediated “inflammation-fibrosis”axis and the ANGPTL3-mediated “metabolism-lipotoxicity” axis do not regulate pathological physiological processes in isolation. Instead, they are intertwined and act synergistically, collectively forming a complex network underlying atrial fibrillation (mechanism diagram). ANGPTL3-induced lipotoxicity promotes TNF-α secretion from visceral fat, which further suppresses SLIT2 expression, creating a positive feedback loop where metabolic abnormalities exacerbate inflammation. Low SLIT2 levels lead to monocyte infiltration, which heightens myocardial sensitivity to lipotoxicity. This cross-regulation may represent a conceptual framework linking obesity to atrial fibrillation through interacting inflammatory and metabolic pathways.

## 5. Conclusion

This study reveals that obese individuals often exhibit susceptibility to atrial fibrillation. Furthermore, SLIT2 and ANGPTL3 have been identified as effective mediating factors in the pathogenesis of atrial fibrillation. These findings hold potential implications for the prevention and treatment of obesity-related atrial fibrillation.

## 6. Translational Significance

This study demonstrates significant innovation. First, we are the first to suggest the key mediating roles of SLIT2 and ANGPTL3 through genetic methods. Second, the MR study design mitigates biases arising from confounding factors and reverse causality to a certain extent. Furthermore, the large pool of GWAS meta-data enhances statistical power and result stability. Additionally, the systematic screening of inflammatory factors and plasma proteins lends greater biological plausibility. Collectively, this mechanistic summary holds significant translational medical implications. SLIT2 and ANGPTL3 show promise as novel biomarkers for predicting atrial fibrillation risk in obese populations. As potential therapeutic targets, they provide new theoretical foundations for developing precision intervention strategies against obesity-related atrial fibrillation. However, at this stage, the lack of substantial foundational research means these findings cannot be directly applied in clinical practice. Therefore, future studies must further validate these pathways in animal models and clinical cohorts to make significant contributions to the prevention and targeted treatment of atrial fibrillation.

## 7. Limitations

We must acknowledge that this study has several limitations. GWAS databases primarily focus on European populations, and their findings may not be applicable to other ethnic groups. Furthermore, limited data sources may have led to the exclusion of other mediating variables that could potentially exhibit significant correlations. It is important to consider that the MR hypothesis and horizontal pleiotropy cannot be entirely ruled out, and the proportion of mediating effects is relatively small. Therefore, this field still requires contributions from more researchers and additional research data.

## Acknowledgments

We thank the participants in all the GWASs used in this study and the investigators who made these GWAS data publicly available.

## Author contributions

**Conceptualization:** Zhiyang Lv.

**Software:** Ying Peng.

**Supervision:** Xiaodi Chen, Zhiyang Lv.

**Visualization:** Qing Zhang, Wen Xu, Xiaodi Chen.

**Writing – original draft:** Yanping Wu.

**Writing – review & editing:** Qianyuan Li, Zhiyang Lv.







## References

[1] Joglar JA Chung MK Armbruster AL. 2023 ACC/AHA/ACCP/HRS guideline for the diagnosis and management of atrial fibrillation: a report of the American college of cardiology/American heart association joint committee on clinical practice guidelines. Circulation. 2024;149:e1–e156. 38033089 10.1161/CIR.0000000000001193 PMC11095842

[2] Sagris M Vardas EP Theofilis P Antonopoulos AS Oikonomou E Tousoulis D. Atrial fibrillation: pathogenesis, predisposing factors, and genetics. Int J Mol Sci. 2021;23:6. 35008432 10.3390/ijms23010006 PMC8744894

[3] Shu H Cheng J Li N. Obesity and atrial fibrillation: a narrative review from arrhythmogenic mechanisms to clinical significance. Cardiovasc Diabetol. 2023;22:192. 37516824 10.1186/s12933-023-01913-5 PMC10387211

[4] Gallo G Desideri G Savoia C. Update on obesity and cardiovascular risk: from pathophysiology to clinical management. Nutrients. 2024;16:2781. 39203917 10.3390/nu16162781 PMC11356794

[5] Goudis CA Korantzopoulos P Ntalas IV Kallergis EM Ketikoglou DG. Obesity and atrial fibrillation: a comprehensive review of the pathophysiological mechanisms and links. J Cardiol. 2015;66:361–9. 25959929 10.1016/j.jjcc.2015.04.002

[6] Hu YF Chen YJ Lin YJ Chen SA. Inflammation and the pathogenesis of atrial fibrillation. Nat Rev Cardiol. 2015;12:230–43. 25622848 10.1038/nrcardio.2015.2

[7] Wang J Zhou S Xie X Liu W. Elucidating the linkage between obesity-related body fat indicators and atrial fibrillation: supported by evidence from Mendelian randomization and mediation analyses. Clin Cardiol. 2025;48:e70103. 40045506 10.1002/clc.70103 PMC11882476

[8] Alpert MA Omran J Bostick BP. Effects of obesity on cardiovascular hemodynamics, cardiac morphology, and ventricular function. Curr Obes Rep. 2016;5:424–34. 27744513 10.1007/s13679-016-0235-6

[9] Upadhyay K Frishman WH. An exploration of the relationship between atrial fibrillation and obesity. Cardiol Rev. 2023;31:185–92. 36727745 10.1097/CRD.0000000000000490

[10] Čarná Z Osmančík P. The effect of obesity, hypertension, diabetes mellitus, alcohol, and sleep apnea on the risk of atrial fibrillation. Physiol Res. 2021;70(Suppl4):S511–25. 35199540 10.33549/physiolres.934744 PMC9054178

[11] Sultan D Brundel B Kurakula K. The interplay between pulmonary hypertension and atrial fibrillation: a comprehensive overview. Cells. 2025;14. 10.3390/cells14110839 PMC12155084 40498015

[12] Pouwels S Topal B Knook MT. Interaction of obesity and atrial fibrillation: an overview of pathophysiology and clinical management. Expert Rev Cardiovasc Ther. 2019;17:209–23. 30757925 10.1080/14779072.2019.1581064

[13] Vyas V Hunter RJ Longhi MP Finlay MC. Inflammation and adiposity: new frontiers in atrial fibrillation. Europace. 2020;22:1609–18. 33006596 10.1093/europace/euaa214

[14] Willar B Tran KV Fitzgibbons TP. Epicardial adipocytes in the pathogenesis of atrial fibrillation: an update on basic and translational studies. Front Endocrinol. 2023;14:1154824. 10.3389/fendo.2023.1154824 PMC10067711 37020587

[15] Conte M Petraglia L Cabaro S. Epicardial adipose tissue and cardiac arrhythmias: focus on atrial fibrillation. Front Cardiovasc Med. 2022;9:932262. 35845044 10.3389/fcvm.2022.932262 PMC9280076

[16] Raggi P Stillman AE. Clinical role of epicardial adipose tissue. Can J Cardiol. 2025;41:1753–63. 39971003 10.1016/j.cjca.2025.02.021

[17] Scridon A Dobreanu D Chevalier P Şerban RC. Inflammation, a link between obesity and atrial fibrillation. Inflam Res. 2015;64:383–93. 10.1007/s00011-015-0827-8 25929437

[18] Ma M Zhi H Yang S Yu EY Wang L. Body mass index and the risk of atrial fibrillation: a Mendelian randomization study. Nutrients. 2022;14:1878. 35565843 10.3390/nu14091878 PMC9101688

[19] Burgess S Thompson SG. Interpreting findings from Mendelian randomization using the MR-Egger method. Eur J Epidemiol. 2017;32:377–89. 28527048 10.1007/s10654-017-0255-x PMC5506233

[20] Larsson SC Butterworth AS Burgess S. Mendelian randomization for cardiovascular diseases: principles and applications. Eur Heart J. 2023;44:4913–24. 37935836 10.1093/eurheartj/ehad736 PMC10719501

[21] Pilling D Zheng Z Vakil V Gomer RH. Fibroblasts secrete Slit2 to inhibit fibrocyte differentiation and fibrosis. Proc Natl Acad Sci USA. 2014;111:18291–6. 25489114 10.1073/pnas.1417426112 PMC4280645

[22] Chang J Lan T Li C. Activation of Slit2-Robo1 signaling promotes liver fibrosis. J Hepatol. 2015;63:1413–20. 26264936 10.1016/j.jhep.2015.07.033

[23] Liu Y Yin Z Xu X. Crosstalk between the activated Slit2-Robo1 pathway and TGF-*β*1 signalling promotes cardiac fibrosis. ESC Heart Fail. 2021;8:447–60. 33236535 10.1002/ehf2.13095 PMC7835586

[24] Fappi A Patterson BW Burks KH. Effect of complete, lifelong ANGPTL3 deficiency on triglyceride-rich lipoprotein kinetics. Cell Rep Med. 2025;6:102152. 40446802 10.1016/j.xcrm.2025.102152 PMC12208341

[25] Luo F Das A Khetarpal SA. ANGPTL3 inhibition, dyslipidemia, and cardiovascular diseases. Trends Cardiovasc Med. 2024;34:215–22. 36746257 10.1016/j.tcm.2023.01.008

[26] D’Erasmo L Di Martino M Neufeld T. ANGPTL3 deficiency and risk of hepatic steatosis. Circulation. 2023;148:1479–89. 37712257 10.1161/CIRCULATIONAHA.123.065866 PMC10805521

[27] Zhang Y Yan C Dong Y. ANGPTL3 accelerates atherosclerotic progression via direct regulation of M1 macrophage activation in plaque. J Adv Res. 2025;70:125–38. 38740260 10.1016/j.jare.2024.05.011 PMC11976407

[28] Mohamed F Mansfield BS Raal FJ. ANGPTL3 as a drug target in hyperlipidemia and atherosclerosis. Curr Atheroscler Rep. 2022;24:959–67. 36367663 10.1007/s11883-022-01071-1 PMC9650658

