# Supplementary material for: SLIT2 and ANGPTL3 as putative mediators linking obesity to atrial fibrillation: A Mendelian randomization study
Source: Medicine (Baltimore). 2026 Jul 3;105(27):e49568. doi: 10.1097/MD.0000000000049568 (PMC13336997; doi:10.1097/MD.0000000000049568)
Supplement: Supplementary file 1 [file medi-105-e49568-s001.docx]

**Supplementary Table1:** The genome-wide association study (GWAS) data information of each core variable.

|  | **ID** | **Sample size** | **SNP（5×10-8）** |
| --- | --- | --- | --- |
| Obesity | GCST90029007 | 532396 | 474 |
| AF | GCST006061 | 537409 | 103 |
|  | **ID** | **Sample size** | **SNP（5×10-5）** |
| TNFSF12 | GCST90274846 | 14736 | 50 |
| SCF | GCST90274833 | 14736 | 47 |
| PD-L1 | GCST90274832 | 14736 | 33 |
| NT-3 | GCST90274829 | 14744 | 34 |
| IL-33 | GCST90274812 | 11793 | 29 |
| GDNF | GCST90274792 | 14736 | 28 |
| FLT3L | GCST90274791 | 14734 | 51 |
| CXCL5 | GCST90274782 | 14736 | 27 |
| CD40 | GCST90274772 | 14736 | 26 |
|  | DOI | | |
| SLIT2 | [10.1038/s41586-023-06563-x](https://doi.org/10.1038/s41586-023-06563-x) | | |
| ANGPTL3 |  |  |  |
| PPBP |  |  |  |
| FAM177A1 |  |  |  |

GWAS = genome-wide association studies, ID = GWAS Dataset Identifier, SNP = Single Nucleotide Polymorphism, AF = Atrial Fibrillation, DOI indicates the bibliographic link to the source of the resource. 5×10⁻⁸ is the conventional significance threshold, while 5×10⁻⁵ represents a more lenient threshold designed to detect more potential associations in scenarios with smaller sample sizes.
