# Supplementary material for: SLIT2 and ANGPTL3 as putative mediators linking obesity to atrial fibrillation: A Mendelian randomization study
Source: Medicine (Baltimore). 2026 Jul 3;105(27):e49568. doi: 10.1097/MD.0000000000049568 (PMC13336997; doi:10.1097/MD.0000000000049568)
Supplement: Supplementary file 2 [file medi-105-e49568-s002.pdf]

1	SNP	CHR.exposure	BP.exposure	A1.exposure	A2.exposure	REF.exposure	FRQ.exposure	BETA.exposure	SE.exposure	P.exposure	N.exposure	INFO.exposure	id.exposure	samplesize.exposure	n.exposure	R2.exposure	F.exposure
2	rs72634826	1	1601052	G	A	G	0.260093	-0.0199809	0.00220625	4.8e-20	457824	0.968513	GCST90029007	532396	12007571	0.000154034839914206	82.0198584770541
3	rs4648450	1	2723214	C	A	C	0.466683	-0.0140325	0.00192484	8.4e-14	457824	0.982788	GCST90029007	532396	12007571	9.98165823111089e-05	53.1470544802787
4	rs3866805	1	6657424	C	A	C	0.355815	0.0107244	0.00199923	2.5e-08	457824	0.995388	GCST90029007	532396	12007571	5.40458350559217e-05	28.7752334902853
5	rs4908671	1	7725284	C	A	C	0.393291	0.0110809	0.00195717	1.3e-08	457824	0.995534	GCST90029007	532396	12007571	6.02049277465783e-05	32.0546721519326
6	rs2791643	1	11207269	C	T	C	0.762026	-0.0150464	0.00224164	1e-11	457824	0.999122	GCST90029007	532396	12007571	8.46178402793848e-05	45.0538428165782
7	rs9435731	1	17306029	C	A	C	0.519466	-0.0116296	0.00190916	5.4e-10	457824	1	GCST90029007	532396	12007571	6.96915060882244e-05	37.1059256601782
8	rs10799778	1	23313353	T	G	T	0.834181	-0.0181364	0.00256859	1.6e-12	457824	0.994485	GCST90029007	532396	12007571	9.3634769463412e-05	49.8552576392594
9	rs3766823	1	32197257	G	A	G	0.171726	0.0144516	0.00253246	1.4e-08	457824	1	GCST90029007	532396	12007571	6.11625140728116e-05	32.5645472468597
10	rs6682438	1	33784146	T	C	T	0.673136	0.0123749	0.00203443	1.9e-09	457824	0.998456	GCST90029007	532396	12007571	6.94916988196219e-05	36.999534661893
11	rs112646560	1	39560250	C	T	C	0.21802	0.018399	0.00233164	4e-16	457824	0.982614	GCST90029007	532396	12007571	0.000116944518282883	62.2678417494549
12	rs12144626	1	47670525	T	C	T	0.58266	-0.0163986	0.00194104	1.9e-17	457824	0.99404	GCST90029007	532396	12007571	0.000134045294059575	71.3744776984049
13	rs1167311	1	49996959	G	A	G	0.681205	-0.0187351	0.00206281	2.4e-20	457824	0.980207	GCST90029007	532396	12007571	0.000154914299426169	82.4882221338387
14	rs3820110	1	54727371	A	C	A	0.456283	0.011386	0.00191939	3.6e-09	457824	0.997456	GCST90029007	532396	12007571	6.60925357924158e-05	35.1895952702523
15	rs12071132	1	57884383	C	G	C	0.147699	0.0155704	0.00270389	3.4e-08	457824	0.9868	GCST90029007	532396	12007571	6.22815864959762e-05	33.160408243773
16	rs1013293	1	62570321	G	A	G	0.430849	-0.0181966	0.00193498	4e-21	457824	0.987131	GCST90029007	532396	12007571	0.000166081254984643	88.4353511203932
17	rs2186120	1	66453163	G	A	G	0.528536	0.0144714	0.00192383	1.3e-14	457824	0.987622	GCST90029007	532396	12007571	0.000106269043551662	56.5830141954434
18	rs2613499	1	72751552	A	G	A	0.191421	-0.0321891	0.00242841	3.1e-40	457824	0.999743	GCST90029007	532396	12007571	0.000329910058922328	175.700101140614
19	rs6604866	1	74992546	G	C	G	0.565305	-0.0172952	0.00192477	1.5e-19	457824	0.998518	GCST90029007	532396	12007571	0.000151632694668715	80.7405797571334
20	rs12049202	1	77967523	C	T	C	0.197501	0.0254907	0.00239671	5.2e-26	457824	0.996576	GCST90029007	532396	12007571	0.000212424937468105	113.117791198116
21	rs1328448	1	78832137	A	T	A	0.691523	-0.0120619	0.00207707	2.6e-09	457824	0.986826	GCST90029007	532396	12007571	6.33383708894025e-05	33.7231045977991
22	rs28726372	1	84353839	T	C	T	0.307595	0.0114264	0.00206825	4.6e-08	457824	1	GCST90029007	532396	12007571	5.73261927171473e-05	30.5218707480975
23	rs4472800	1	96280456	G	T	G	0.389804	0.0133168	0.00196015	8.4e-12	457824	0.993927	GCST90029007	532396	12007571	8.66859490030842e-05	46.1550801304704
24	rs2181375	1	96940119	A	G	A	0.596927	0.0196627	0.00194481	1.1e-23	457824	0.998496	GCST90029007	532396	12007571	0.000191961346985151	102.218691404502
25	rs75641275	1	98327133	A	C	A	0.143238	0.019534	0.00272764	1.2e-12	457824	0.996373	GCST90029007	532396	12007571	9.63233652094509e-05	51.286921826222

26	rs41279738	1	110082551	T	G	T	0.026047	0.0672871	0.00601767	6.6e-29	457824	0.98765	GCST90029007	532396	12007571	0.00023478487966592	125.027415771625
27	rs2618039	1	112324111	A	T	A	0.381337	0.0145444	0.00196654	2.5e-14	457824	0.99397	GCST90029007	532396	12007571	0.000102732214240877	54.6996338830646
28	rs61813324	1	156049877	C	T	C	0.135797	0.028448	0.00282604	7.3e-24	457824	0.966354	GCST90029007	532396	12007571	0.000190295900811119	101.331678819537
29	rs1778830	1	156489974	G	A	G	0.362155	0.0137895	0.00199043	1.6e-12	457824	0.991388	GCST90029007	532396	12007571	9.01424352788712e-05	47.9956181297603
30	rs61828641	1	174321997	G	A	G	0.109101	0.0226429	0.00306166	1.7e-13	457824	0.996355	GCST90029007	532396	12007571	0.000102723729382284	54.6951156670122
31	rs539515	1	177889025	A	C	A	0.20524	0.0463952	0.00236489	1.6e-87	457824	0.999777	GCST90029007	532396	12007571	0.000722396416042637	384.877551686557
32	rs815163	1	190294726	T	C	T	0.562933	-0.015407	0.00192281	1.2e-15	457824	0.998709	GCST90029007	532396	12007571	0.000120580274040656	64.2039561482281
33	rs672313	1	195142845	G	A	G	0.212128	-0.0171507	0.00234245	1.1e-13	457824	0.989992	GCST90029007	532396	12007571	0.000100680306856491	53.6069884566023
34	rs2678204	1	201800511	T	G	T	0.34058	0.0240227	0.00201823	4.2e-33	457824	0.994817	GCST90029007	532396	12007571	0.000266043088137176	141.677436168341
35	rs7539903	1	209208033	T	A	T	0.615266	-0.0116795	0.0019621	3.4e-09	457824	0.994611	GCST90029007	532396	12007571	6.65491541195412e-05	35.4327283764206
36	rs4844809	1	209519772	G	C	G	0.131927	0.0173583	0.00281904	2e-10	457824	0.996774	GCST90029007	532396	12007571	7.1210853647341e-05	37.9149311713371
37	rs7518221	1	225561346	T	C	T	0.64723	-0.0122934	0.00199897	1e-09	457824	0.997489	GCST90029007	532396	12007571	7.10339292304201e-05	37.8207242733517
38	rs4658403	1	243832560	C	T	C	0.833927	-0.0176739	0.00256699	2.1e-12	457824	0.997673	GCST90029007	532396	12007571	8.90313547147213e-05	47.4039795025033
39	rs6710091	2	239597	C	G	C	0.348501	-0.0113725	0.00200006	4e-09	457824	0.999241	GCST90029007	532396	12007571	6.07246031638064e-05	32.3313776868713
40	rs62107261	2	422144	T	C	T	0.048326	-0.0925382	0.00445924	4.1e-97	457824	0.99281	GCST90029007	532396	12007571	0.000808229068055105	430.644365752525
41	rs6744646	2	628504	A	G	A	0.828453	0.0566218	0.00252825	8.7e-113	457824	0.996505	GCST90029007	532396	12007571	0.000941203590227015	501.563217316185
42	rs10929925	2	6155557	C	A	C	0.427436	-0.0137564	0.00193003	5.7e-13	457824	0.995122	GCST90029007	532396	12007571	9.54124941347163e-05	50.8018865370594
43	rs4133529	2	12854006	G	A	G	0.110127	0.0175437	0.00305079	8.7e-09	457824	0.991658	GCST90029007	532396	12007571	6.21092089219657e-05	33.0686240408803
44	rs6752378	2	25150116	C	A	C	0.486186	0.0338571	0.00190891	1.1e-71	457824	0.995937	GCST90029007	532396	12007571	0.000590523636645587	314.577006165977
45	rs935166	2	26949366	G	A	G	0.506872	-0.0170148	0.00190811	6.8e-19	457824	0.99615	GCST90029007	532396	12007571	0.000149330060141805	79.514301914623
46	rs13012070	2	35447243	G	A	G	0.228288	-0.0133015	0.00227206	4.8e-09	457824	0.997372	GCST90029007	532396	12007571	6.43722818889426e-05	34.2736229152974
47	rs6713781	2	40291940	G	C	G	0.401309	-0.0130184	0.00196404	1.5e-11	457824	0.975725	GCST90029007	532396	12007571	8.25170975761802e-05	43.9352330548859
48	rs10169594	2	41637688	T	C	T	0.363327	0.012114	0.00198738	1.2e-09	457824	0.989258	GCST90029007	532396	12007571	6.97827720696874e-05	37.1545218988022
49	rs35809007	2	47019521	G	A	G	0.363322	-0.0167517	0.00198894	4e-17	457824	0.987561	GCST90029007	532396	12007571	0.000133223783541942	70.9369935096969
50	rs6724631	2	50713331	G	C	G	0.537028	-0.0132161	0.0019125	4.9e-12							

457824 0.997556 GCST90029007 532396 12007571 8.96871055364623e-05 47.7531597076537
51 rs6705567 2 55320173 T C T 0.376102 -0.0147751 0.0019826 3.6e-14 457824 0.983007 GCST90029007 532396 12007571 0.000104306306749397 55.5378448230171
52 rs4671328 2 58935282 T G T 0.551365 -0.0210575 0.00193182 2.3e-28 457824 0.980863 GCST90029007 532396 12007571 0.000223125191426729 118.817024235719
53 rs10172678 2 59294558 T C T 0.603769 -0.0200264 0.00194942 9e-25 457824 1 GCST90029007 532396 12007571 0.000198186502834681 105.534220448247
54 rs4672338 2 60217457 C T C 0.336359 0.0147594 0.00201939 2.3e-13 457824 0.996575 GCST90029007 532396 12007571 0.000100327189308149 53.418952997933
55 rs2861685 2 67837553 T C T 0.411851 -0.0175389 0.00193301 5e-20 457824 0.997983 GCST90029007 532396 12007571 0.000154608927468887 82.3255936026007
56 rs4832298 2 86764004 C T C 0.686079 -0.0162453 0.002053 1.2e-15 457824 0.999248 GCST90029007 532396 12007571 0.00011759577397327 62.6146477066842
57 rs6542924 2 100893113 C A C 0.682785 -0.0179702 0.00206183 7.1e-19 457824 0.98365 GCST90029007 532396 12007571 0.00014266037706334 75.9623656059797
58 rs2198234 2 104159202 G T G 0.528054 0.0126132 0.00191082 5.5e-11 457824 0.996626 GCST90029007 532396 12007571 8.18353005561624e-05 43.5721887474596
59 rs1451533 2 105466005 G A G 0.273791 0.0178122 0.0021545 1.6e-16 457824 0.983165 GCST90029007 532396 12007571 0.0001283664682367 68.3503113784844
60 rs752590 2 113972945 A G A 0.210166 0.0127707 0.0023387 2.4e-08 457824 1 GCST90029007 532396 12007571 5.60042863597912e-05 29.8180159689397
61 rs13033310 2 133523605 G A G 0.252819 0.01334 0.0022083 2.8e-09 457824 0.984294 GCST90029007 532396 12007571 6.85379268600925e-05 36.4916820969906
62 rs36118164 2 143473080 G T G 0.283121 -0.0121554 0.00212252 2.6e-09 457824 0.987949 GCST90029007 532396 12007571 6.15989615072911e-05 32.79693777002
63 rs778094 2 147903802 G A G 0.576606 -0.0175127 0.00193177 4.3e-20 457824 0.996266 GCST90029007 532396 12007571 0.0001543453540234 82.1852253176312
64 rs10803762 2 161105876 G A G 0.677736 0.0131741 0.00204747 1.4e-10 457824 0.990402 GCST90029007 532396 12007571 7.77567791101873e-05 41.4004618241542
65 rs1432544 2 166291814 T C T 0.235266 -0.0122191 0.00225448 2.3e-08 457824 0.989863 GCST90029007 532396 12007571 5.51730405553173e-05 29.3754164844526
66 rs788163 2 172931559 A C A 0.275606 0.013699 0.00214084 1.4e-10 457824 0.989256 GCST90029007 532396 12007571 7.69026183147522e-05 40.9456414020966
67 rs34234296 2 175166636 G A G 0.39262 -0.0164511 0.00197338 2.8e-17 457824 0.971528 GCST90029007 532396 12007571 0.000130519970865666 69.4971201311499
68 rs1019612 2 181602526 C T C 0.662155 0.0171431 0.00201654 5.5e-18 457824 0.995325 GCST90029007 532396 12007571 0.000135728578528034 72.2708900620314
69 rs62190049 2 182566998 G C G 0.39069 -0.0109299 0.00197036 2.2e-08 457824 0.980377 GCST90029007 532396 12007571 5.77938103079044e-05 30.7708562100933
70 rs12478299 2 193811641 T C T 0.251908 -0.0122855 0.0022022 1.9e-08 457824 0.991797 GCST90029007 532396 12007571 5.84536971548374e-05 31.1222168516911
71 rs1064213 2 198950240 G A G 0.47814 0.0142337 0.00190816 7.4e-14 457824 1 GCST90029007 532396 12007571 0.000104502305011091 55.6422149137893
72 rs11675464 2 204053742 A G A 0.5629 0.0117081 0.00192178 1.6e-09 457824 0.998287 GCST90029007 532396 12007571 6.97108611314062e-05 37.1162316056611
73 rs4482463 2 205375909 C A C 0.9231982 -0.0334898 0.0035915 3.9e-21 457824 0.985924 GCST90029007 532396 12007571 0.000163293034413982 86.9504301633827
74 rs73985439 2 212299249 A C A 0.307376 0.0125669 0.00207069 1.3e-09 457824 0.993132 GCST90029007 532396 12007571 6.91769353365266e-05 36.8319332318183

75	rs13427822	2	213414265	A	G	A	0.271377	-0.0178913	0.00216803	2.7e-16	457824	0.973061	GCST90029007	532396	12007571	0.000127897745301059	68.1007021380688
76	rs6725931	2	220205146	C	T	C	0.848244	0.019811	0.00266267	6.4e-14	457824	0.993955	GCST90029007	532396	12007571	0.00010396763544409	55.3575006930721
77	rs4605363	2	229010960	A	C	A	0.341463	0.0172107	0.00201044	1.3e-17	457824	0.998286	GCST90029007	532396	12007571	0.000137632270717534	73.284681473511
78	rs62190394	2	230624929	C	T	C	0.316877	0.0171349	0.00205116	2.1e-16	457824	0.998751	GCST90029007	532396	12007571	0.000131060664191134	69.7850573273384
79	rs7568228	2	236848488	G	C	G	0.526971	-0.0123935	0.00190911	4.9e-11	457824	0.998632	GCST90029007	532396	12007571	7.91510858340135e-05	42.1428988477178
80	rs62246311	3	9498143	G	A	G	0.102357	0.0228787	0.00315315	2e-13	457824	0.998691	GCST90029007	532396	12007571	9.88770946550648e-05	52.6467774921899
81	rs17776482	3	11633951	G	A	G	0.140417	0.0158341	0.00276867	8.2e-09	457824	0.985088	GCST90029007	532396	12007571	6.14303392028871e-05	32.7071532210986
82	rs2920503	3	12324230	C	T	C	0.285439	-0.0141505	0.0021283	1e-11	457824	0.989016	GCST90029007	532396	12007571	8.30246471955959e-05	44.2054941646092
83	rs2569993	3	12926096	T	C	T	0.320247	0.0124889	0.0020558	8.1e-10	457824	0.990233	GCST90029007	532396	12007571	6.93141281980492e-05	36.9049840046647
84	rs7619139	3	25110415	T	A	T	0.58888	0.0148354	0.0019476	1.4e-14	457824	0.991144	GCST90029007	532396	12007571	0.000108972490521576	58.0226229884776
85	rs13085002	3	35552535	T	C	T	0.160148	0.0175506	0.00260687	2.6e-11	457824	0.998328	GCST90029007	532396	12007571	8.51281963460379e-05	45.3255994519822
86	rs4377469	3	42303074	G	T	G	0.886889	0.0210003	0.00301313	4.4e-13	457824	1	GCST90029007	532396	12007571	9.12306674370974e-05	48.5750914975242
87	rs28350	3	42418446	A	G	A	0.820797	-0.0176526	0.00250449	2e-12	457824	0.988403	GCST90029007	532396	12007571	9.33046846529148e-05	49.6794896102157
88	rs7637852	3	44041777	A	G	A	0.703561	-0.0129601	0.00209323	6.2e-10	457824	1	GCST90029007	532396	12007571	7.19973897200511e-05	38.3337382317078
89	rs35979968	3	48092335	C	T	C	0.276993	0.0135731	0.00213507	1.6e-10	457824	0.999271	GCST90029007	532396	12007571	7.59042530814248e-05	40.4140365122875
90	rs9843653	3	49920571	T	C	T	0.512102	0.0276498	0.00191651	3.7e-48	457824	0.996642	GCST90029007	532396	12007571	0.000390802441976966	208.142217780872
91	rs17668356	3	61208619	C	G	C	0.146126	-0.0235749	0.00270554	2.1e-18	457824	0.997304	GCST90029007	532396	12007571	0.000142591975210361	75.9259384796826
92	rs9827532	3	62100592	A	G	A	0.774362	-0.0132211	0.0022873	5.7e-09	457824	0.997631	GCST90029007	532396	12007571	6.27519004063296e-05	33.4108318581207
93	rs6445264	3	62354425	G	A	G	0.667458	0.0122439	0.00212758	2.7e-09	457824	0.904763	GCST90029007	532396	12007571	6.22022294210227e-05	33.1181537533737
94	rs76267866	3	70540347	A	T	A	0.206521	0.016358	0.00236351	4.4e-12	457824	0.997593	GCST90029007	532396	12007571	8.99646019326052e-05	47.9009236688375
95	rs4564925	3	78504037	A	T	A	0.173043	0.0141281	0.0025334	2.4e-08	457824	0.992056	GCST90029007	532396	12007571	5.84116985877478e-05	31.0998544532498
96	rs78605811	3	83631491	A	C	A	0.054067	-0.0329443	0.00430743	2.3e-14	457824	0.963089	GCST90029007	532396	12007571	0.000109860510549719	58.4955029994315
97	rs9835772	3	85766025	A	T	A	0.243682	0.0180171	0.00222725	3.5e-16	457824	0.996986	GCST90029007	532396	12007571	0.000122897679234837	65.4380292204767
98	rs9876664	3	85806313	G	T	G	0.375387	-0.0180472	0.00197744	2.1e-20	457824	0.992666	GCST90029007	532396	12007571	0.000156426492847518	

83.2935555518332
99 rs9851777 3 88267467 T C T 0.88489 0.0246851 0.00299504 9.5e-17 457824 0.999364 GCST90029007 532396 12007571 0.000127577572489818 67.9302004982227
100 rs1454687 3 94038085 C G C 0.515465 -0.0220363 0.00191237 4.1e-31 457824 0.998402 GCST90029007 532396 12007571 0.000249339120981575 132.779759164299
101 rs9830592 3 104631603 C A C 0.582486 0.015486 0.00193894 2.9e-15 457824 0.997709 GCST90029007 532396 12007571 0.000119801692429349 63.789344310638
102 rs9854637 3 107406630 G A G 0.290636 0.0126981 0.00211524 2.2e-09 457824 0.990117 GCST90029007 532396 12007571 6.76852448803715e-05 36.037657480512
103 rs1471093 3 108031094 G A G 0.616747 0.0129697 0.00197682 4.3e-11 457824 0.985774 GCST90029007 532396 12007571 8.08454741749913e-05 43.0451253814927
104 rs1870931 3 119757013 C G C 0.173082 -0.0145388 0.00252805 8.4e-09 457824 0.997709 GCST90029007 532396 12007571 6.2118941039892e-05 33.0738060057975
105 rs11709402 3 131551027 A G A 0.278942 0.0234542 0.00213983 1.9e-28 457824 0.993504 GCST90029007 532396 12007571 0.00022560562841698 120.138186786556
106 rs2203254 3 131889481 C T C 0.743169 -0.0123318 0.00219589 1e-08 457824 0.990699 GCST90029007 532396 12007571 5.92340290075229e-05 31.537709745045
107 rs1471740 3 136328270 T C T 0.740533 0.0188093 0.00218384 3.1e-18 457824 0.99523 GCST90029007 532396 12007571 0.000139318355834758 74.1825917330022
108 rs3821709 3 141280273 T C T 0.066507 0.0334 0.00383886 1.5e-18 457824 0.998125 GCST90029007 532396 12007571 0.000142164727165248 75.6984093981342
109 rs355748 3 153964496 G T G 0.377487 0.0155831 0.0019781 3.7e-15 457824 0.994751 GCST90029007 532396 12007571 0.000116553635371532 62.059689432405
110 rs7633365 3 156330873 G A G 0.153412 -0.0143177 0.00265725 4.4e-08 457824 0.995821 GCST90029007 532396 12007571 5.45284583554227e-05 29.0322071391749
111 rs56143236 3 157020444 C T C 0.256429 0.0120174 0.00219428 1.4e-08 457824 0.99751 GCST90029007 532396 12007571 5.6334921560933e-05 29.9940639427509
112 rs4679819 3 157804409 T G T 0.366416 -0.0113759 0.00198675 3.7e-09 457824 0.998126 GCST90029007 532396 12007571 6.15777137634739e-05 32.7856242051738
113 rs2555502 3 168228135 C T C 0.375438 0.0113038 0.00197497 4.4e-09 457824 0.997756 GCST90029007 532396 12007571 6.15270960933978e-05 32.7586723435296
114 rs6444950 3 170602073 G A G 0.237322 0.0145306 0.00224833 5.2e-11 457824 0.997453 GCST90029007 532396 12007571 7.84473233665685e-05 41.7681608768436
115 rs12631813 3 171126134 C G C 0.503018 -0.0109349 0.00192344 2.8e-08 457824 0.986147 GCST90029007 532396 12007571 6.07031456581142e-05 32.3199524522875
116 rs529200 3 173114305 A G A 0.527729 0.0163907 0.00191699 1.3e-17 457824 0.997553 GCST90029007 532396 12007571 0.000137296951010009 73.1061101820511
117 rs2606228 3 183537759 A C A 0.645741 -0.0129081 0.00202086 6.2e-11 457824 0.979161 GCST90029007 532396 12007571 7.66274058218806e-05 40.7990974241298
118 rs869400 3 185826740 T G T 0.814941 0.0300953 0.00246649 2.4e-33 457824 0.990326 GCST90029007 532396 12007571 0.00027956475682151 148.880220805956
119 rs6583310 3 196170985 G C G 0.43855 0.0132039 0.00193354 7.7e-12 457824 0.988358 GCST90029007 532396 12007571 8.75841074121607e-05 46.6333376208411
120 rs2051559 4 3298800 T C T 0.132646 0.0192056 0.00283481 1.3e-11 457824 0.991953 GCST90029007 532396 12007571 8.62056285183029e-05 45.8993161687722
121 rs75631642 4 18049216 T C T 0.218733 0.0135602 0.00237201 7.5e-09 457824 0.950491 GCST90029007 532396 12007571 6.13815309840079e-05 32.6811648266313
122 rs66679256 4 18351898 C T C 0.445771 0.014705 0.00193152 8.2e-15

457824 0.997394 GCST90029007 532396 12007571 0.000108855299524954
57.9602176121371
123 rs34811474 4 25408838 G A G 0.231142 -0.0270893 0.00227383 5e-34
457824 1 GCST90029007 532396 12007571 0.000266519527203595 141.931224608909
124 rs73213484 4 28489339 A T A 0.140982 -0.0206867 0.00275855 2.7e-13
457824 0.995438 GCST90029007 532396 12007571 0.00010561851488096
56.2366032380495
125 rs2242189 4 38691024 T C T 0.381636 -0.0141233 0.00199288 3.8e-12
457824 0.978199 GCST90029007 532396 12007571 9.43266302294088e-05
50.2236694038485
126 rs12641981 4 45179883 C T C 0.435131 0.0293836 0.00193588 2.9e-52
457824 0.997779 GCST90029007 532396 12007571 0.000432544171639544
230.383572787514
127 rs13106834 4 55501728 A G A 0.487921 -0.0141504 0.0019152 1.6e-14
457824 1 GCST90029007 532396 12007571 0.000102525018139117 54.5893012762608
128 rs9917946 4 65651232 T G T 0.404777 -0.012387 0.00196112 2.3e-10
457824 0.98916 GCST90029007 532396 12007571 7.49301515254691e-05
39.8953524560553
129 rs2318543 4 67803263 A G A 0.782965 -0.0131564 0.00233251 8.2e-09
457824 0.990781 GCST90029007 532396 12007571 5.97539162424418e-05
31.8145275265917
130 rs11097236 4 77028450 G A G 0.20079 0.0134151 0.00239565 4.1e-08 457824
1 GCST90029007 532396 12007571 5.88953122613723e-05 31.3573576774531
131 rs7442137 4 80718632 C T C 0.634553 -0.0119452 0.00199331 7.7e-10
457824 0.991903 GCST90029007 532396 12007571 6.74486222193865e-05
35.9116639701245
132 rs4148155 4 89054667 A G A 0.113467 -0.0203776 0.00301768 5.1e-12
457824 0.999679 GCST90029007 532396 12007571 8.56421807889312e-05
45.5992884214451
133 rs2865397 4 96061112 G A G 0.594043 -0.0110681 0.00195764 2e-08
457824 0.994698 GCST90029007 532396 12007571 6.00370894284992e-05
31.9653052930889
134 rs1229984 4 100239319 T C T 0.9734712 0.0431133 0.00604223 6.8e-14
457824 1 GCST90029007 532396 12007571 9.56206547497047e-05 50.9127311735042
135 rs13135092 4 103198082 A G A 0.08353 0.0423916 0.00348253 5.1e-36 457824
0.989709 GCST90029007 532396 12007571 0.00027823659332215 148.172719937971
136 rs9991259 4 112686354 G A G 0.633001 0.012775 0.0019883 6e-11
457824 0.996746 GCST90029007 532396 12007571 7.75335260275376e-05
41.2815847627315
137 rs11099020 4 130724902 C T C 0.641196 -0.0142048 0.00200313 4.7e-13
457824 0.99274 GCST90029007 532396 12007571 9.44443916977399e-05
50.2863767397884
138 rs1296328 4 137083193 A C A 0.559206 -0.0190974 0.00193929 1.8e-23
457824 0.990364 GCST90029007 532396 12007571 0.000182116413914414
96.9753468719609
139 rs34236292 4 140786038 G T G 0.327008 -0.0123413 0.00204698 4.4e-10
457824 0.994406 GCST90029007 532396 12007571 6.82700445214965e-05
36.3490436338048
140 rs113079574 4 147354089 C T C 0.192408 -0.0168026 0.00244452 1.6e-12
457824 0.987469 GCST90029007 532396 12007571 8.87344916058677e-05
47.245903265221
141 rs6536575 4 162091639 T C T 0.519018 0.0131888 0.00191862 5.8e-12
457824 0.995395 GCST90029007 532396 12007571 8.87481486160713e-05
47.2531754661435
142 rs28885461 4 171609988 C T C 0.181035 -0.0152784 0.00249335 1.7e-09
457824 0.996421 GCST90029007 532396 12007571 7.05218744887007e-05
37.5480708068741
143 rs1037702 4 180168024 G A G 0.621777 -0.0136161 0.00198227 4.6e-12
457824 0.988017 GCST90029007 532396 12007571 8.86148644387131e-05
47.1822031825232
144 rs698147 5 3513485 A G A 0.543503 -0.0125664 0.00192515 1.9e-11 457824
0.994274 GCST90029007 532396 12007571 8.0024518995151e-05 42.6079834492853
145 rs72740698 5 27188816 A G A 0.281664 -0.0129824 0.00214042 1.7e-09
457824 0.987303 GCST90029007 532396 12007571 6.90950887213295e-05
36.7883525591875
146 rs13176429 5 43152216 T C T 0.687315 0.0159856 0.00206677 2.6e-14

457824 0.997191 GCST90029007 532396 12007571 0.000112354379043666 59.8235187108707
147 rs116374395 5 50723410 G A G 0.035409 0.0325297 0.00520216 3.4e-10 457824 0.990505 GCST90029007 532396 12007571 7.34388768330456e-05 39.1012889473955
148 rs150215901 5 50935903 T A T 0.043127 -0.028561 0.00478766 6.8e-10 457824 0.970231 GCST90029007 532396 12007571 6.68399941703809e-05 35.5875905306894
149 rs1503526 5 63020706 T C T 0.480117 0.0143412 0.00191707 6.6e-14 457824 0.998736 GCST90029007 532396 12007571 0.000105102878637893 55.9620237393317
150 rs9291822 5 64076515 C T C 0.51497 -0.0142736 0.00193462 2.1e-14 457824 0.981244 GCST90029007 532396 12007571 0.000102234272709306 54.4344784541135
151 rs7707394 5 74472939 G A G 0.356549 -0.0202023 0.00199868 2.6e-24 457824 0.999953 GCST90029007 532396 12007571 0.000191865540151442 102.167664837587
152 rs6874626 5 74999576 G A G 0.367758 -0.0272891 0.00198797 1.5e-43 457824 0.999624 GCST90029007 532396 12007571 0.000353810163445313 188.433077695322
153 rs252761 5 77380723 G T G 0.587811 -0.0107191 0.00195602 3.7e-08 457824 0.985959 GCST90029007 532396 12007571 5.64041140900537e-05 30.0309057834961
154 rs7707981 5 80922749 A G A 0.327846 -0.0132614 0.00204762 8.4e-11 457824 0.990833 GCST90029007 532396 12007571 7.87791018716412e-05 41.9448255375346
155 rs7442885 5 87682877 C G C 0.213287 -0.0233892 0.00234912 1.2e-22 457824 0.993203 GCST90029007 532396 12007571 0.000186167851705705 99.1331026377578
156 rs1477290 5 87988934 T C T 0.13676 0.0351045 0.00281205 1.7e-35 457824 0.976383 GCST90029007 532396 12007571 0.000292629170854163 155.839618005935
157 rs1919243 5 88778861 T C T 0.487156 0.011873 0.00194094 3e-10 457824 0.968818 GCST90029007 532396 12007571 7.02799441735178e-05 37.4192504211464
158 rs159037 5 94197477 T C T 0.254184 0.012979 0.00219953 7.3e-09 457824 1 GCST90029007 532396 12007571 6.53972518739328e-05 34.8193816060396
159 rs10515237 5 95751549 A G A 0.281976 0.014337 0.00212998 3.9e-11 457824 0.996422 GCST90029007 532396 12007571 8.50929888968529e-05 45.3068520262075
160 rs11955324 5 95834624 C A C 0.396542 -0.0110138 0.00197474 1.6e-08 457824 0.977832 GCST90029007 532396 12007571 5.84244142802301e-05 31.1066250026548
161 rs12659431 5 103903766 G A G 0.534923 -0.0112024 0.00191904 5.2e-09 457824 0.99925 GCST90029007 532396 12007571 6.40017010562912e-05 34.0763025734935
162 rs62379271 5 105870033 T G T 0.578875 0.0120408 0.00194555 3e-10 457824 0.994978 GCST90029007 532396 12007571 7.19382493867409e-05 38.3022477406546
163 rs40071 5 107496102 T C T 0.179566 -0.0255212 0.00250248 1.6e-24 457824 0.989006 GCST90029007 532396 12007571 0.000195317579625588 104.00622173068
164 rs6594682 5 112444220 C T C 0.42242 0.0116367 0.00194562 1.1e-09 457824 0.990538 GCST90029007 532396 12007571 6.71861366547808e-05 35.7718994139078
165 rs4434411 5 119326790 A G A 0.545754 -0.0121473 0.00192862 3.4e-10 457824 0.994904 GCST90029007 532396 12007571 7.45073366268337e-05 39.6702146981471
166 rs1582931 5 122657199 G A G 0.473151 -0.0139465 0.001935 1.6e-12 457824 0.979289 GCST90029007 532396 12007571 9.7564397287308e-05 51.9477679820527
167 rs329124 5 133865452 A G A 0.420663 -0.0154652 0.00194395 3.2e-16 457824 0.995677 GCST90029007 532396 12007571 0.000118865135977286 63.2906082502469
168 rs2133561 5 139086651 A T A 0.611522 -0.0140795 0.00198653 3.5e-13 457824 0.976015 GCST90029007 532396 12007571 9.43427323030179e-05 50.2322436688504
169 rs10063055 5 140990108 C T C 0.253639 0.0121658 0.00220305 1e-08 457824 0.999486 GCST90029007 532396 12007571 5.72760392562541e-05 30.4951662861355

170 rs2579040 5 152515823 T A T 0.720992 -0.0146771 0.00213917 7.5e-12 457824 0.993186 GCST90029007 532396 12007571 8.84131216794157e-05 47.0747775314248

171 rs4958702 5 153544512 T C T 0.572455 -0.0161113 0.00193507 4.1e-17 457824 0.999242 GCST90029007 532396 12007571 0.00013018965640657 69.3212168383214

172 rs17056301 5 158271680 T C T 0.256186 0.0129589 0.00220255 4.1e-09 457824 0.991639 GCST90029007 532396 12007571 6.50162181498432e-05 34.6164950792633

173 rs34774377 5 167001890 T C T 0.119299 -0.0162429 0.00295617 1.5e-08 457824 1 GCST90029007 532396 12007571 5.67033764266262e-05 30.1902492783465

174 rs864789 5 167358292 C G C 0.203507 0.0136784 0.00239015 1.6e-08 457824 0.991463 GCST90029007 532396 12007571 6.15118496516347e-05 32.7505542306003

175 rs779655 5 170495158 G C G 0.728901 0.0183994 0.00215432 3.1e-18 457824 0.998653 GCST90029007 532396 12007571 0.000136991182002596 72.9432759366796

176 rs28710014 5 176728817 A G A 0.026924 0.0318871 0.0059268 4e-08 457824 0.993099 GCST90029007 532396 12007571 5.43664665219709e-05 28.9459542667518

177 rs9378999 6 5979069 T C T 0.659219 0.0121518 0.00203118 3.3e-09 457824 0.985214 GCST90029007 532396 12007571 6.72233921200626e-05 35.7917366663172

178 rs10947793 6 12142817 A G A 0.372098 -0.0141429 0.00199562 2.9e-12 457824 0.987553 GCST90029007 532396 12007571 9.43290571026579e-05 50.2249617003927

179 rs9463511 6 13178259 G A G 0.295199 -0.0148031 0.00210396 6.8e-12 457824 0.998402 GCST90029007 532396 12007571 9.2972653235271e-05 49.5026851425189

180 rs3806114 6 20482335 G A G 0.667853 -0.012236 0.00206157 9e-10 457824 0.976095 GCST90029007 532396 12007571 6.61636180453531e-05 35.2274440407899

181 rs2183947 6 26159356 G A G 0.225127 -0.015297 0.0025041 3.1e-10 457824 1 GCST90029007 532396 12007571 7.0088049353509e-05 37.3170724283262

182 rs2269553 6 28876509 G A G 0.214821 -0.0252082 0.0035013 4.4e-13 457824 0.999663 GCST90029007 532396 12007571 9.73526904997595e-05 51.835034586008

183 rs9380362 6 33467734 T C T 0.618756 -0.0134935 0.0020243 1.6e-11 457824 0.99884 GCST90029007 532396 12007571 8.34504050755008e-05 44.432202845092

184 rs9366863 6 34688946 T C T 0.672278 -0.0285762 0.00204334 2.6e-45 457824 0.999071 GCST90029007 532396 12007571 0.000367225966638101 195.580723602606

185 rs34045288 6 40369081 C T C 0.33463 0.0233165 0.00203303 1.7e-30 457824 0.998649 GCST90029007 532396 12007571 0.000247000070451785 131.533844376936

186 rs9349235 6 42516718 C T C 0.41075 0.0115478 0.0019511 6.8e-09 457824 0.994219 GCST90029007 532396 12007571 6.57924548681778e-05 35.0298129144739

187 rs72892910 6 50816887 G T G 0.172221 0.0390886 0.00254546 6.7e-54 457824 0.994623 GCST90029007 532396 12007571 0.000442731656173652 235.812078829121

188 rs1775255 6 51243035 G T G 0.477132 0.0148086 0.001926 5.2e-15 457824 0.995946 GCST90029007 532396 12007571 0.000111027978812102 59.1171934141883

189 rs7761673 6 70357368 T A T 0.219727 -0.0152658 0.00232179 1.1e-10 457824 0.993124 GCST90029007 532396 12007571 8.11938652512443e-05 43.2306367590667

190 rs7776021 6 73742152 G A G 0.287456 0.0119457 0.00211901 1e-08 457824 0.996113 GCST90029007 532396 12007571 5.9689300281103e-05 31.7801222671185

191 rs9294260 6 83433228 G A G 0.476668 0.0145811 0.00193069 1.9e-14 457824 0.986212 GCST90029007 532396 12007571 0.000107120890467894 57.0366291742701

192 rs6909685 6 97753952 C T C 0.326626 -0.0147096 0.00205263 1.4e-12 457824 0.989878 GCST90029007 532396 12007571 9.64503278878168e-05 51.3545290266695

193 rs6938973 6 98421721 T C T 0.60123 0.0181625 0.00195995 4.5e-21 457824 0.998867 GCST90029007 532396 12007571 0.000161271060345022 85.8735938268595

194 rs57989773 6 100629078 T C T 0.244673 0.0153918 0.00229705 6.1e-11 457824 0.937577 GCST90029007 532396 12007571 8.43269742674817e-05 44.8989613317185
195 rs34240187 6 104847826 A C A 0.75358 0.0129298 0.00222267 6.8e-09 457824 0.997421 GCST90029007 532396 12007571 6.35581505249646e-05 33.8401288065885
196 rs2253310 6 108888593 C G C 0.62654 0.0180543 0.00198188 3.7e-20 457824 0.996197 GCST90029007 532396 12007571 0.000155849037942036 82.9860259984267
197 rs13218383 6 120173501 C G C 0.335111 -0.0130984 0.00203141 7.5e-11 457824 0.996723 GCST90029007 532396 12007571 7.80859014172343e-05 41.575711876066
198 rs9388346 6 124925864 C T C 0.163086 0.0150336 0.00260758 3.1e-09 457824 0.988927 GCST90029007 532396 12007571 6.24293482997433e-05 33.2390855531427
199 rs1159974 6 126090277 T C T 0.524764 0.0129451 0.00191934 6.8e-12 457824 1 GCST90029007 532396 12007571 8.54348578934599e-05 45.4888920703604
200 rs9375702 6 130384187 C T C 0.691191 -0.0114904 0.00207613 1.7e-08 457824 0.99608 GCST90029007 532396 12007571 5.75309356127435e-05 30.6308871582078
201 rs6922607 6 142703483 A G A 0.189856 0.0139516 0.00244117 2.5e-08 457824 0.998426 GCST90029007 532396 12007571 6.13466083530579e-05 32.6625699454042
202 rs765875 6 143185683 C T C 0.489544 -0.0124348 0.00191838 4.2e-11 457824 0.996785 GCST90029007 532396 12007571 7.89113143275502e-05 42.015225756788
203 rs7749708 6 153375907 C T C 0.293367 0.0147625 0.00211125 2.1e-12 457824 0.995032 GCST90029007 532396 12007571 9.18260347310582e-05 48.8921195040707
204 rs9478496 6 154333183 T C T 0.164291 0.0175505 0.00259644 9.3e-12 457824 0.992115 GCST90029007 532396 12007571 8.58124599277906e-05 45.6899595586199
205 rs36007635 6 163009335 G A G 0.1377 -0.0208323 0.00278385 5.7e-14 457824 0.998743 GCST90029007 532396 12007571 0.000105172529296645 55.9991131307245
206 rs6950388 7 1270699 G A G 0.795627 0.0143021 0.00238011 9.7e-10 457824 1 GCST90029007 532396 12007571 6.78174295531724e-05 36.1080413440818
207 rs4721089 7 1872921 C T C 0.783091 0.0175773 0.0023334 6e-14 457824 0.996414 GCST90029007 532396 12007571 0.000106572431120385 56.7445703007159
208 rs2396962 7 3104205 C A C 0.170767 0.0155452 0.00256799 3.2e-09 457824 0.98133 GCST90029007 532396 12007571 6.88242197453552e-05 36.6441236503271
209 rs9638713 7 14645949 A G A 0.9748179 -0.0355542 0.00617066 3.2e-09 457824 0.985638 GCST90029007 532396 12007571 6.23529094008573e-05 33.1983848634437
210 rs10263780 7 19778086 G A G 0.136795 -0.0175223 0.0028007 2.3e-10 457824 0.990721 GCST90029007 532396 12007571 7.35160776507966e-05 39.1423962402596
211 rs215634 7 32369148 A G A 0.612388 -0.0161456 0.00197618 2.9e-16 457824 0.994362 GCST90029007 532396 12007571 0.000125362067771451 66.7503806748599
212 rs56408610 7 39441027 G A G 0.349326 -0.0118846 0.00201438 1e-09 457824 0.994116 GCST90029007 532396 12007571 6.53767247235515e-05 34.8084516450317
213 rs799449 7 44784697 C T C 0.557398 0.0148436 0.00193733 5e-15 457824 0.989403 GCST90029007 532396 12007571 0.000110252541469456 58.704263857375
214 rs10259490 7 49616420 A G A 0.383807 0.0128566 0.00197522 4.6e-11 457824 0.99496 GCST90029007 532396 12007571 7.9570479157394e-05 42.366216780691
215 rs1451371 7 50553051 T C T 0.439939 -0.0111613 0.00193397 7.3e-09 457824 0.995708 GCST90029007 532396 12007571 6.25558809834153e-05 33.306459215183
216 rs73124396 7 71579606 T C T 0.205162 -0.0146755 0.0023817 3.1e-10 457824 0.992147 GCST90029007 532396 12007571 7.13092626816446e-05 37.9673310185124
217 rs58862095 7 75081418 C T C 0.419731 -0.0225517 0.00194762 3.3e-31 457824 0.994674 GCST90029007 532396 12007571 0.00025177115916802 134.075210785334
218 rs17149254 7 76634463 T C T 0.805243 -0.0247001 0.00248538 1.8e-23 457824 0.951483 GCST90029007 532396 12007571 0.000185479740858913

98.7666203619906
219 rs3729628 7 77825224 C T C 0.755736 0.0140456 0.00223311 1.7e-10
457824 1 GCST90029007 532396 12007571 7.43007685537742e-05 39.5602227283709
220 rs16868443 7 93206264 G C G 0.360635 0.012566 0.00199771 6e-10
457824 0.995623 GCST90029007 532396 12007571 7.43125501418699e-05
39.5664961074566
221 rs3901286 7 99107727 C A C 0.152218 -0.0240784 0.0026752 2e-18
457824 0.996016 GCST90029007 532396 12007571 0.000152139461929861
81.0104615827225
222 rs2103123 7 109228343 A T A 0.471526 0.0106046 0.00195178 4.9e-09
457824 0.962744 GCST90029007 532396 12007571 5.54457155241561e-05
29.520603061727
223 rs1524445 7 113035833 C T C 0.421341 -0.0184608 0.00195755 2.7e-21
457824 0.979259 GCST90029007 532396 12007571 0.000167019808534019
88.9351978843833
224 rs6962980 7 113452183 A C A 0.55654 -0.0164094 0.00192995 4.4e-17 457824
0.998891 GCST90029007 532396 12007571 0.0001357686614746 72.2922357771945
225 rs7802342 7 137435925 T G T 0.288355 0.0122593 0.00211699 1.3e-09
457824 0.996972 GCST90029007 532396 12007571 6.29841966289933e-05
33.5345205249309
226 rs11525873 7 138817193 T C T 0.097774 -0.0235895 0.00323551 3.2e-13
457824 0.991504 GCST90029007 532396 12007571 9.98329341937647e-05
53.1557618628313
227 rs2710082 7 147660103 G A G 0.50043 0.010864 0.0019178 1.9e-08 457824
0.998641 GCST90029007 532396 12007571 6.02714895911118e-05 32.0901135483151
228 rs1805123 7 150645534 T G T 0.245498 -0.0162508 0.00222824 3.3e-13
457824 1 GCST90029007 532396 12007571 9.98958365983201e-05 53.1892574152936
229 rs56211164 7 158016764 G A G 0.239774 -0.0121209 0.00224608 1.9e-08
457824 0.995892 GCST90029007 532396 12007571 5.46966535323088e-05
29.1217630236623
230 rs78686130 8 4128311 C T C 0.313544 -0.0120571 0.00208387 5.7e-09 457824
0.981473 GCST90029007 532396 12007571 6.28756357868368e-05 33.4767161089066
231 rs1658820 8 4288577 G T G 0.245455 0.0147004 0.00224628 9.7e-11 457824
0.982671 GCST90029007 532396 12007571 8.04379149371335e-05 42.8281082887717
232 rs13263601 8 14095900 A C A 0.348596 0.0153821 0.00201137 1.4e-14
457824 0.998884 GCST90029007 532396 12007571 0.000109841090126503
58.4851614106951
233 rs13278290 8 15560370 C T C 0.645969 0.0132725 0.00201385 3.7e-11
457824 0.995371 GCST90029007 532396 12007571 8.15794953582654e-05
43.4359773368799
234 rs117176448 8 27261138 C G C 0.096083 0.0224302 0.00325492 1.6e-11
457824 1 GCST90029007 532396 12007571 8.91891724528136e-05 47.4880156956645
235 rs142583374 8 27985864 G A G 0.132714 0.0178483 0.00283792 1.4e-10
457824 0.993438 GCST90029007 532396 12007571 7.42893174738866e-05
39.5541253361669
236 rs10954772 8 30863938 T C T 0.685511 -0.0153055 0.00208173 7.1e-14
457824 0.981932 GCST90029007 532396 12007571 0.000101523682976324
54.0560876476025
237 rs2468964 8 38333062 T G T 0.600516 -0.0118493 0.00195739 1.7e-09
457824 0.998092 GCST90029007 532396 12007571 6.88281320191858e-05
36.6462068081826
238 rs13264909 8 64702385 A T A 0.428985 -0.0126211 0.00194242 1.8e-10
457824 0.992497 GCST90029007 532396 12007571 7.9293741610674e-05
42.2188599624462
239 rs1808629 8 73435964 G A G 0.685937 -0.021082 0.00207613 3.9e-24
457824 0.994078 GCST90029007 532396 12007571 0.000193640252834842
103.112875571049
240 rs10217047 8 74809210 C A C 0.095958 0.0194062 0.00331268 1.7e-09
457824 0.9562 GCST90029007 532396 12007571 6.44553924442981e-05
34.3178761771665
241 rs10102172 8 77238392 A G A 0.566283 0.0210695 0.00193841 4.3e-28
457824 0.998666 GCST90029007 532396 12007571 0.00022186358540832
118.145053775106
242 rs1465809 8 85080457 G A G 0.749105 0.0128129 0.00221797 1.9e-09
457824 0.997833 GCST90029007 532396 12007571 6.26789239711757e-05
33.3719747681794

243 rs11778219 8 87762607 A G A 0.163401 0.015578 0.00260681 3.1e-09 457824 0.991861 GCST90029007 532396 12007571 6.70719184423253e-05 35.7110821579733
244 rs2920939 8 93182601 G A G 0.606491 0.0106937 0.00196311 4.6e-08 457824 1 GCST90029007 532396 12007571 5.57323984109379e-05 29.6732482808882
245 rs12680855 8 95582655 A G A 0.31763 -0.0137678 0.00206486 5.5e-12 457824 0.993599 GCST90029007 532396 12007571 8.3498144806904e-05 44.4576234354062
246 rs2694047 8 116750548 A G A 0.752758 0.0213461 0.00222862 7.5e-22 457824 0.995312 GCST90029007 532396 12007571 0.000172288154064727 91.7409853801596
247 rs2954021 8 126482077 A G A 0.504879 0.0118064 0.00192021 8.2e-10 457824 0.99957 GCST90029007 532396 12007571 7.10022172499298e-05 37.8038386069208
248 rs7828631 8 132875320 C T C 0.109635 0.0176218 0.00307688 7.5e-09 457824 0.995026 GCST90029007 532396 12007571 6.16052718723108e-05 32.8002977844496
249 rs11782074 8 142617096 G T G 0.384209 0.0131166 0.00200603 1.6e-11 457824 0.966652 GCST90029007 532396 12007571 8.02967641961867e-05 42.7529484008811
250 rs10099330 8 143383694 A G A 0.452834 0.0109593 0.00193039 9.3e-09 457824 0.996807 GCST90029007 532396 12007571 6.05360998822739e-05 32.2310075002127
251 rs12375985 9 11813799 G A G 0.354645 -0.0147559 0.00200775 2e-13 457824 0.994217 GCST90029007 532396 12007571 0.000101445618473442 54.0145181377486
252 rs13299961 9 13945051 C G C 0.19231 -0.0147385 0.00243901 8.8e-10 457824 0.994379 GCST90029007 532396 12007571 6.85827412198142e-05 36.5155442651046
253 rs13292699 9 15910044 A C A 0.433865 -0.0202312 0.00193938 3.1e-25 457824 0.99793 GCST90029007 532396 12007571 0.000204359069554469 108.821781194334
254 rs1411432 9 16728532 A C A 0.185967 0.0213607 0.00247641 9.2e-18 457824 0.990638 GCST90029007 532396 12007571 0.000139730235470167 74.4019351828264
255 rs1111817 9 23226898 C G C 0.364805 -0.013124 0.00201785 3.3e-11 457824 0.974912 GCST90029007 532396 12007571 7.94484439835223e-05 42.3012356535146
256 rs1412239 9 28425515 C G C 0.323556 0.024347 0.00205133 1.9e-32 457824 0.997925 GCST90029007 532396 12007571 0.000264527097564629 140.869903487527
257 rs1384131 9 29634777 T A T 0.420312 -0.0115862 0.00195763 5.4e-09 457824 0.984169 GCST90029007 532396 12007571 6.57896326550934e-05 35.0283101874355
258 rs2275003 9 34124860 A G A 0.521136 -0.0116943 0.00192023 1.7e-10 457824 0.999407 GCST90029007 532396 12007571 6.9658949916268e-05 37.0885905339921
259 rs7034554 9 37081301 A G A 0.373686 -0.0143124 0.00198271 4.5e-13 457824 0.998569 GCST90029007 532396 12007571 9.78654163875225e-05 52.1080600692145
260 rs7038966 9 73777777 C T C 0.411405 0.0135059 0.00196051 2e-12 457824 0.986199 GCST90029007 532396 12007571 8.91323671654488e-05 47.4577675078403
261 rs10780248 9 81370555 G A G 0.559887 -0.0135155 0.00193605 4.2e-12 457824 0.994769 GCST90029007 532396 12007571 9.15285451899223e-05 48.7337088133129
262 rs3949781 9 92178472 T A T 0.537995 0.0128229 0.00193812 3.2e-11 457824 0.984924 GCST90029007 532396 12007571 8.22130375398942e-05 43.7733266461613
263 rs10820852 9 94186973 C A C 0.27587 -0.0153833 0.0021522 3e-12 457824 0.993291 GCST90029007 532396 12007571 9.59527237857204e-05 51.0895566092885
264 rs7873003 9 96392945 T C T 0.680089 -0.0159945 0.00206875 1.1e-14 457824 0.988183 GCST90029007 532396 12007571 0.000112264325629252 59.7755640424464
265 rs2417084 9 103119277 T C T 0.315375 0.0169484 0.00206584 1.4e-16 457824 0.999681 GCST90029007 532396 12007571 0.000126407941892075 67.3073379977598
266 rs7024334 9 109072075 T G T 0.779198 -0.0127382 0.00231432 1.3e-08

457824 0.999089 GCST90029007 532396 12007571 5.68996786972559e-05 30.2947713031002
267 rs7038943 9 120377178 T C T 0.338488 -0.013402 0.00202798 1.3e-11 457824 1 GCST90029007 532396 12007571 8.20241019075648e-05 43.67272192677
268 rs10760277 9 126093999 C T C 0.385223 0.013193 0.0019781 7.1e-12 457824 0.991506 GCST90029007 532396 12007571 8.35448282480721e-05 44.4824815715871
269 rs871998 9 127057420 T C T 0.46257 0.0105876 0.00192714 1.1e-08 457824 0.996964 GCST90029007 532396 12007571 5.66903468445467e-05 30.1833116203603
270 rs10733682 9 129460914 A G A 0.526727 -0.0137195 0.00195223 5.2e-12 457824 0.961882 GCST90029007 532396 12007571 9.27554708818698e-05 49.3870370825623
271 rs113132247 9 131026108 G A G 0.152898 0.0186388 0.00267628 5.6e-13 457824 0.989351 GCST90029007 532396 12007571 9.10958203603833e-05 48.5032866316318
272 rs7893571 10 16750129 G T G 0.665628 0.0139566 0.00204009 9e-12 457824 0.993715 GCST90029007 532396 12007571 8.78997519109034e-05 46.8014143515641
273 rs73601548 10 18549889 C T C 0.114465 0.0180319 0.00302615 1.8e-09 457824 0.99386 GCST90029007 532396 12007571 6.66865625073927e-05 35.5058935255488
274 rs76638898 10 21099584 G A G 0.024277 -0.0359599 0.00642938 2.9e-08 457824 0.938754 GCST90029007 532396 12007571 5.87541380871778e-05 31.2821885508107
275 rs11012732 10 21830104 A G A 0.331473 0.0212449 0.00204152 3.1e-25 457824 0.996658 GCST90029007 532396 12007571 0.000203366301705317 108.293021981482
276 rs12762034 10 33969931 T C T 0.076917 0.0277533 0.00360092 6.7e-15 457824 0.998067 GCST90029007 532396 12007571 0.000111562715546627 59.4019474232943
277 rs3004929 10 34414865 G A G 0.426732 0.0110641 0.00195823 9e-09 457824 0.981965 GCST90029007 532396 12007571 5.9957561494807e-05 31.9229600173046
278 rs4595495 10 53673286 A G A 0.419653 0.012465 0.00194885 4.2e-11 457824 0.997408 GCST90029007 532396 12007571 7.68350865166549e-05 40.9096823499307
279 rs7070670 10 61842645 C T C 0.328147 -0.0116127 0.00205632 1.2e-08 457824 0.982719 GCST90029007 532396 12007571 5.98996404629888e-05 31.8921195111448
280 rs7924036 10 65191645 G T G 0.503449 -0.0132044 0.00192036 1.4e-12 457824 0.999646 GCST90029007 532396 12007571 8.87970814097503e-05 47.2792316178416
281 rs10824211 10 76363107 C T C 0.139286 0.019386 0.00278373 4.2e-12 457824 0.986304 GCST90029007 532396 12007571 9.10852078487613e-05 48.497635564649
282 rs7918784 10 77577699 T C T 0.514862 0.010804 0.00192204 2.6e-08 457824 0.998895 GCST90029007 532396 12007571 5.93449474980399e-05 31.596769086874
283 rs17399739 10 87490850 A G A 0.068909 0.02694 0.00379661 1.3e-12 457824 0.995203 GCST90029007 532396 12007571 9.45643308080587e-05 50.3502436733246
284 rs11201992 10 88117318 C A C 0.458595 -0.0124598 0.00192894 6.9e-11 457824 1 GCST90029007 532396 12007571 7.8363871931368e-05 41.7237248656606
285 rs2439823 10 99778226 A G A 0.545648 0.0189092 0.00193329 1.6e-22 457824 0.991052 GCST90029007 532396 12007571 0.000179655074001731 95.6644700755286
286 rs117597828 10 102416055 C T C 0.217854 0.0161257 0.00233475 1.4e-11 457824 0.98979 GCST90029007 532396 12007571 8.95947759379249e-05 47.7039951694542
287 rs10883553 10 102635475 C A C 0.445833 0.0147099 0.00193329 2.8e-14 457824 1 GCST90029007 532396 12007571 0.000108728507094807 57.8926993930094
288 rs7096249 10 104618524 G A G 0.346403 0.0122487 0.00201779 2.8e-09 457824 0.999511 GCST90029007 532396 12007571 6.9209103385505e-05 36.8490616783419
289 rs35198068 10 114754784 T C T 0.291274 -0.0163559 0.00211271 5.2e-15 457824 0.996163 GCST90029007 532396 12007571 0.000112560352988336 59.9332026713203

290	rs4751614	10	118696266	A	T	A	0.234003	0.0159072	0.00227864	1.1e-12	457824	0.986066	GCST90029007	532396	12007571	9.15295891013111e-05	48.7342646872254
291	rs12414412	10	120465796	C	G	C	0.085391	0.0187503	0.0034487	4.2e-08	457824	0.990653	GCST90029007	532396	12007571	5.55196488898581e-05	29.5599691101733
292	rs845084	10	125220036	G	A	G	0.258328	0.0164274	0.00220195	5.2e-14	457824	0.98939	GCST90029007	532396	12007571	0.000104530390968971	55.6571708353619
293	rs72828935	10	126587488	G	C	G	0.282798	0.0173133	0.00213786	2.7e-16	457824	0.993408	GCST90029007	532396	12007571	0.000123172258708607	65.5842496630766
294	rs11017772	10	132954247	C	T	C	0.20621	-0.0131679	0.00237873	3.1e-08	457824	0.998154	GCST90029007	532396	12007571	5.75549869046369e-05	30.6436933954793
295	rs4880341	10	133992689	C	T	C	0.576891	-0.0137701	0.00194525	2.4e-12	457824	0.998582	GCST90029007	532396	12007571	9.41125805771394e-05	50.1096891759458
296	rs7946756	11	851623	T	A	T	0.630523	0.0136424	0.002003	3.8e-11	457824	0.97996	GCST90029007	532396	12007571	8.71258515269087e-05	46.3893223070248
297	rs10766451	11	2917510	C	T	C	0.32404	0.0117012	0.00206567	2.1e-08	457824	0.980393	GCST90029007	532396	12007571	6.02667764159579e-05	32.0876039796523
298	rs11042030	11	8690718	T	C	T	0.276298	-0.0181168	0.00215019	1.2e-17	457824	0.99439	GCST90029007	532396	12007571	0.000133326541541851	70.9917158375832
299	rs900144	11	13294268	C	T	C	0.566605	0.0168892	0.00193549	1e-18	457824	0.999577	GCST90029007	532396	12007571	0.00014300110740105	76.1438202242886
300	rs111909661	11	27468595	G	C	G	0.215735	0.0145538	0.0023424	2.6e-10	457824	0.988474	GCST90029007	532396	12007571	7.25044083499141e-05	38.6037109182644
301	rs6265	11	27679916	C	T	C	0.188647	-0.0403447	0.00245205	9.2e-61	457824	1	GCST90029007	532396	12007571	0.000508228220817879	270.715240519151
302	rs7941828	11	30430331	C	T	C	0.360534	-0.0173979	0.00199701	1.1e-18	457824	0.99756	GCST90029007	532396	12007571	0.000142539912792595	75.8982128559424
303	rs59227842	11	43692423	A	G	A	0.311403	0.0229843	0.00208999	9.4e-29	457824	0.982138	GCST90029007	532396	12007571	0.000227112332806605	120.940710439119
304	rs7115013	11	43934592	C	T	C	0.442667	-0.0127172	0.00193548	4.4e-11	457824	0.994974	GCST90029007	532396	12007571	8.10840862916524e-05	43.1721816140574
305	rs10742752	11	45438374	T	C	T	0.611991	0.0113481	0.00196977	4.4e-09	457824	0.995667	GCST90029007	532396	12007571	6.2338078711626e-05	33.1904881088576
306	rs7124681	11	47529947	C	A	C	0.408423	0.0257954	0.0019568	9.3e-40	457824	1	GCST90029007	532396	12007571	0.000326298545116267	173.776090514141
307	rs34292685	11	64049021	C	T	C	0.161894	-0.0193086	0.00260199	2.8e-13	457824	0.999869	GCST90029007	532396	12007571	0.000103421450282763	55.06665467513
308	rs2234458	11	65639374	C	T	C	0.639363	-0.0189865	0.00199492	1e-21	457824	0.998813	GCST90029007	532396	12007571	0.000170110135484186	90.5810242212701
309	rs597539	11	68601974	C	G	C	0.314618	0.0126434	0.0020692	6.8e-10	457824	0.994883	GCST90029007	532396	12007571	7.01225301211558e-05	37.3354323563026
310	rs7951796	11	69296607	G	A	G	0.420065	-0.012564	0.00196139	1.2e-10	457824	0.97727	GCST90029007	532396	12007571	7.70654445005936e-05	41.0323424351577
311	rs10160769	11	76474827	G	C	G	0.217001	-0.0151329	0.00235573	1.8e-10	457824	0.973449	GCST90029007	532396	12007571	7.75041315539035e-05	41.2659328948007
312	rs349071	11	84776849	G	A	G	0.500484	-0.0137451	0.00192323	2.6e-12	457824	0.993804	GCST90029007	532396	12007571	9.5930544606807e-05	51.0777462814145
313	rs61903695	11	89922417	A	G	A	0.255165	0.014945	0.00220365	5.1e-12	457824	0.998989	GCST90029007	532396	12007571	8.63841971889774e-05	45.9944014676744

314 rs680071 11 103088414 T C T 0.880656 0.0169141 0.00295936 1.3e-08 457824 0.998774 GCST90029007 532396 12007571 6.1353700045659e-05 32.6663459833016
315 rs1048932 11 115044850 C A C 0.413264 -0.016143 0.0019478 1.1e-16 457824 1 GCST90029007 532396 12007571 0.000128999776886763 68.687567896785
316 rs7925100 11 118941596 G A G 0.396229 0.0137098 0.00196196 9.4e-12 457824 0.999505 GCST90029007 532396 12007571 9.17080220970436e-05 48.8292787529083
317 rs7944782 11 130795698 T G T 0.51004 0.0154735 0.00192924 1.4e-15 457824 0.988343 GCST90029007 532396 12007571 0.000120814023221398 64.3284328557137
318 rs2512896 11 131454621 A G A 0.567338 0.0127742 0.0019443 3.1e-11 457824 0.988548 GCST90029007 532396 12007571 8.10719979747818e-05 43.1657448229628
319 rs1793636 11 131934926 G C G 0.309094 -0.012628 0.00207735 5.2e-10 457824 0.997125 GCST90029007 532396 12007571 6.94040591233761e-05 36.9528693320586
320 rs10894670 11 133221987 C A C 0.550726 -0.0111054 0.00193162 6.4e-09 457824 0.990867 GCST90029007 532396 12007571 6.20816538984226e-05 33.0539520896104
321 rs329651 11 133767622 G T G 0.803914 0.0164258 0.00242732 8.2e-12 457824 0.985862 GCST90029007 532396 12007571 8.60056248215776e-05 45.7928170611027
322 rs12364470 11 134601012 T G T 0.164778 0.0196763 0.00258654 1.6e-14 457824 1 GCST90029007 532396 12007571 0.000108684218178651 57.8691151125373
323 rs55726687 12 991306 G A G 0.210044 0.0249288 0.00235487 7.3e-27 457824 0.996748 GCST90029007 532396 12007571 0.000210446972910571 112.064289286202
324 rs2429150 12 2152655 A C A 0.406099 0.0119658 0.00196161 9.6e-10 457824 0.991639 GCST90029007 532396 12007571 6.98864546827214e-05 37.2097296104349
325 rs117855597 12 17168386 G C G 0.030977 0.0300866 0.00553865 3.4e-08 457824 1 GCST90029007 532396 12007571 5.54216595942101e-05 29.507794408937
326 rs10505836 12 19288508 A C A 0.860466 0.0184643 0.00279097 4.3e-11 457824 0.982597 GCST90029007 532396 12007571 8.22024711712075e-05 43.7677002498419
327 rs78086698 12 24024639 T C T 0.039789 0.0302486 0.00492816 2.8e-10 457824 0.983955 GCST90029007 532396 12007571 7.07579829593586e-05 37.6737913011477
328 rs1458156 12 41887940 C T C 0.488349 0.0151943 0.00192087 4.7e-15 457824 0.993447 GCST90029007 532396 12007571 0.000117511298411983 62.569662849072
329 rs1126930 12 49399132 G C G 0.035349 0.0323902 0.00519667 2.2e-10 457824 1 GCST90029007 532396 12007571 7.2964314765807e-05 38.8485979567566
330 rs7132908 12 50263148 G A G 0.384578 0.0293526 0.00197356 4.6e-51 457824 1 GCST90029007 532396 12007571 0.000415314691004282 221.202918424249
331 rs2292238 12 56493822 A C A 0.40797 -0.016157 0.00195478 1.9e-16 457824 0.994541 GCST90029007 532396 12007571 0.00012830251439514 68.3162539960477
332 rs1154752 12 68089328 C T C 0.605996 -0.0105336 0.00196828 4.9e-08 457824 0.992398 GCST90029007 532396 12007571 5.37925013823971e-05 28.6403456168111
333 rs317656 12 69681101 T A T 0.724606 -0.0151836 0.00215085 2.4e-12 457824 0.996 GCST90029007 532396 12007571 9.35952539290083e-05 49.8342158663688
334 rs11115160 12 82424100 G A G 0.23799 -0.0127384 0.0022662 5.3e-09 457824 0.986029 GCST90029007 532396 12007571 5.93434425771985e-05 31.5959677809447
335 rs704061 12 89771903 T C T 0.454775 0.0152705 0.00192761 7.3e-16 457824 0.995572 GCST90029007 532396 12007571 0.000117864277587037 62.7576310845238
336 rs4294611 12 97498717 T G T 0.604396 -0.0121611 0.00196612 2.9e-10 457824 0.996334 GCST90029007 532396 12007571 7.18554454323824e-05 38.2581570724457
337 rs4764949 12 103658096 A G A 0.325355 -0.019241 0.00205136 7.9e-21 457824 0.998616 GCST90029007 532396 12007571 0.000165220894535107 87.9771485883124
338 rs10861861 12 108436396 T G T 0.228453 -0.0171231 0.00228534 3.6e-14 457824 0.998613 GCST90029007 532396 12007571 0.000105434457532958 56.138591525746
339 rs16942944 12 114066309 G A G 0.293789 0.012309 0.00210452 5.2e-09

457824 0.999785 GCST90029007 532396 12007571 6.42505338081654e-05
34.2087966297088
340 rs11610621 12 121671133 T A T 0.148257 0.0163745 0.00270062 2.4e-09
457824 0.996753 GCST90029007 532396 12007571 6.90469285392302e-05
36.7627088248441
341 rs147730268 12 123024476 G T G 0.087465 -0.0370716 0.00347759 5.1e-26
457824 0.953867 GCST90029007 532396 12007571 0.000213401973398692
113.638180837668
342 rs4148866 12 123425575 C T C 0.410106 0.0111961 0.00195266 7.3e-09
457824 1 GCST90029007 532396 12007571 6.17474001560654e-05 32.8759753646947
343 rs77927866 12 133487774 G A G 0.312302 0.0131447 0.00208356 2.2e-10
457824 0.986921 GCST90029007 532396 12007571 7.47518741170423e-05
39.800424424985
344 rs4771123 13 28022914 C T C 0.276394 -0.017296 0.00215984 4.5e-15
457824 0.989445 GCST90029007 532396 12007571 0.000120437324995
64.1278325880026
345 rs4771218 13 28655311 G A G 0.625821 -0.0134704 0.0019954 4.4e-12
457824 0.98889 GCST90029007 532396 12007571 8.55911952806957e-05
45.572139394153
346 rs12323184 13 33143248 T A T 0.370146 -0.0169598 0.00199348 1.8e-17
457824 0.998868 GCST90029007 532396 12007571 0.000135932675358728
72.3795795148197
347 rs1336486 13 40784814 T G T 0.328728 0.014685 0.00204917 7.5e-13
457824 0.993082 GCST90029007 532396 12007571 9.64528908245464e-05
51.3558937820597
348 rs2806946 13 53645933 A T A 0.627128 -0.0109226 0.00200385 1.3e-08
457824 0.983869 GCST90029007 532396 12007571 5.58036519631603e-05
29.7111874760401
349 rs4477562 13 54104968 C T C 0.128631 0.029633 0.00289581 2.9e-24
457824 0.979334 GCST90029007 532396 12007571 0.00019664862737023
104.715141408969
350 rs6561937 13 58257667 T A T 0.753308 -0.01419 0.0022378 1.2e-10
457824 0.993205 GCST90029007 532396 12007571 7.55187285394355e-05
40.2087544760384
351 rs4055791 13 59266053 C T C 0.417002 -0.0176995 0.00195083 4.7e-20
457824 0.999128 GCST90029007 532396 12007571 0.000154589936285027
82.31547968329
352 rs9571687 13 67472713 C A C 0.329168 -0.0126929 0.00204942 2.3e-10
457824 0.996301 GCST90029007 532396 12007571 7.2043317581145e-05
38.3581934718102
353 rs1441264 13 79580919 G A G 0.593256 0.0189714 0.00200178 1.6e-20
457824 0.952647 GCST90029007 532396 12007571 0.000168677837389171
89.818218901893
354 rs61969510 13 86484025 T C T 0.279266 0.0158259 0.0021627 6e-13
457824 0.982912 GCST90029007 532396 12007571 0.000100569388882589
53.5479245068007
355 rs7996639 13 97019090 G A G 0.448634 0.0147807 0.00194832 7e-15
457824 0.981556 GCST90029007 532396 12007571 0.000108090542793282
57.5529773724501
356 rs7339274 13 99120272 C T C 0.28932 -0.0130535 0.00211763 1.4e-09 457824
1 GCST90029007 532396 12007571 7.13654496738843e-05 37.9972489044324
357 rs9888533 13 107854612 C T C 0.538351 0.012773 0.00196135 8.9e-11
457824 0.962994 GCST90029007 532396 12007571 7.96537536078532e-05
42.4105586584891
358 rs4773337 13 111879786 C T C 0.393561 -0.0128892 0.00196538 5e-11
457824 0.999508 GCST90029007 532396 12007571 8.077723705819e-05
43.008790477622
359 rs2528787 13 112188236 C T C 0.373637 -0.015846 0.00200199 6.3e-16
457824 0.981082 GCST90029007 532396 12007571 0.000117660207364898
62.6489597294205
360 rs12879423 14 25927832 A G A 0.679056 0.0218435 0.00207395 2.4e-26
457824 0.987864 GCST90029007 532396 12007571 0.000208315999208239
110.929296429698
361 rs11161044 14 29675957 G C G 0.807557 0.016886 0.00244161 7.8e-12
457824 0.992824 GCST90029007 532396 12007571 8.98311958348776e-05
47.829886291196

362	rs1191600	14	30101641	C	A	C	0.593777	-0.0112891	0.00196888	6.9e-09	457824	0.987832	GCST90029007	532396	12007571	6.17473813224580e-05	32.8759653365567
363	rs76746358	14	35825531	G	T	G	0.096019	-0.0189274	0.00327007	8.3e-09	457824	0.996161	GCST90029007	532396	12007571	6.29224624548718e-05	33.5016494824808
364	rs67272968	14	40845125	A	G	A	0.188776	-0.0160161	0.00246517	2.7e-11	457824	0.989995	GCST90029007	532396	12007571	7.92776157856791e-05	42.2102733084304
365	rs35697587	14	47298505	G	A	G	0.508498	-0.0170097	0.00192421	7.5e-19	457824	0.997954	GCST90029007	532396	12007571	0.000146753967224951	78.1423993338715
366	rs217669	14	62360075	T	C	T	0.271731	0.0177008	0.00216324	1.7e-15	457824	1	GCST90029007	532396	12007571	0.000125743916587491	66.9537257513868
367	rs3902951	14	69789755	T	G	T	0.2373	0.0140233	0.00228501	3.5e-10	457824	0.975453	GCST90029007	532396	12007571	7.07389702354712e-05	37.6636676086054
368	rs1860750	14	73309738	T	A	T	0.510626	0.0117539	0.00192772	2e-09	457824	0.997418	GCST90029007	532396	12007571	6.98250036544443e-05	37.1770088803851
369	rs10145154	14	79939525	C	T	C	0.221974	0.0276238	0.00231813	1.4e-33	457824	0.996252	GCST90029007	532396	12007571	0.000266649432359269	142.000422223461
370	rs113624107	14	88326386	G	A	G	0.226172	0.0142157	0.00230061	1e-09	457824	0.995772	GCST90029007	532396	12007571	7.17107995237235e-05	38.1811374015231
371	rs1286058	14	91458523	T	A	T	0.703761	0.014859	0.0021071	7.2e-13	457824	0.997399	GCST90029007	532396	12007571	9.33971284031995e-05	49.728715298301
372	rs6575340	14	94023972	G	A	G	0.636098	0.0206865	0.00200317	1.2e-25	457824	0.992755	GCST90029007	532396	12007571	0.000200270347422746	106.644088994539
373	rs12881629	14	101146413	A	G	A	0.082391	0.0208396	0.00349274	6.4e-10	457824	1	GCST90029007	532396	12007571	6.68624704346464e-05	35.5995583590014
374	rs61992671	14	101531854	A	G	A	0.492333	-0.0174396	0.00200997	1.1e-17	457824	0.91185	GCST90029007	532396	12007571	0.000141383164605811	75.2821921717141
375	rs7159965	14	102780553	C	G	C	0.16399	0.0146103	0.00260219	2e-08	457824	0.996021	GCST90029007	532396	12007571	5.92079141831111e-05	31.523804722328
376	rs3803286	14	103246470	A	G	A	0.666928	-0.0192862	0.00203813	6.4e-22	457824	0.997594	GCST90029007	532396	12007571	0.0001681596320077	89.5422365126484
377	rs12889639	14	103860206	G	A	G	0.651678	0.0151732	0.00202041	2.1e-14	457824	0.997453	GCST90029007	532396	12007571	0.000105924045066144	56.3993000909492
378	rs4284600	15	31843528	T	C	T	0.466808	0.0112032	0.00193805	3e-09	457824	0.991343	GCST90029007	532396	12007571	6.2761336310463e-05	33.4158561074559
379	rs8024137	15	35837297	A	T	A	0.848023	0.015542	0.00268965	3.3e-09	457824	0.992028	GCST90029007	532396	12007571	6.27133859331997e-05	33.3903244108211
380	rs34153025	15	41339697	T	C	T	0.022215	-0.0366207	0.00657997	1.3e-08	457824	0.984075	GCST90029007	532396	12007571	5.81762074369345e-05	30.9744657591245
381	rs7162533	15	46586095	T	C	T	0.433479	0.0145663	0.00194382	3e-14	457824	0.995834	GCST90029007	532396	12007571	0.000105464383668473	56.1545273814135
382	rs117632017	15	52260107	G	A	G	0.038296	0.0362607	0.00516702	2.2e-12	457824	0.937212	GCST90029007	532396	12007571	9.24947593176891e-05	49.2482100935212
383	rs340025	15	60908307	T	C	T	0.579982	0.012542	0.00195518	1.7e-10	457824	0.989995	GCST90029007	532396	12007571	7.72843842947601e-05	41.1489226613768
384	rs72749744	15	62291924	G	A	G	0.018767	-0.0477815	0.00709875	1.8e-11	457824	0.995036	GCST90029007	532396	12007571	8.50910838186328e-05	45.305837601362
385	rs3784710	15	68072458	T	C	T	0.226245	-0.0297996	0.00229758	3.4e-41							

457824 0.997454 GCST90029007 532396 12007571 0.000315869431680778
168.220125805792
386 rs7171864 15 73227249 G A G 0.660171 0.0149763 0.00204207 1.8e-13
457824 0.986737 GCST90029007 532396 12007571 0.000101015764558332
53.7856201518106
387 rs35107470 15 74817689 A G A 0.322376 0.0123245 0.00212074 3e-09
457824 0.942692 GCST90029007 532396 12007571 6.34309813042168e-05
33.77241607797
388 rs62007782 15 78029797 G A G 0.265176 -0.0161401 0.00217781 5.9e-14
457824 0.99592 GCST90029007 532396 12007571 0.000103155616978115
54.925097377754
389 rs2870111 15 79403585 C T C 0.41208 -0.0158475 0.00196201 2.8e-16 457824
0.991709 GCST90029007 532396 12007571 0.00012252679904139 65.2405263617045
390 rs11634851 15 81028965 C G C 0.465756 0.0115349 0.00192881 3.5e-09
457824 0.99843 GCST90029007 532396 12007571 6.71714638111829e-05
35.7640866303417
391 rs66615128 15 92561941 G C G 0.215665 -0.013904 0.00233935 1.5e-09
457824 0.998553 GCST90029007 532396 12007571 6.63476450182295e-05
35.3254318810498
392 rs8025516 15 95271872 T G T 0.645693 -0.0154438 0.00201663 1e-14
457824 0.994938 GCST90029007 532396 12007571 0.000110147150786764
58.6481421217223
393 rs34303684 15 99519259 G C G 0.35746 -0.0120308 0.00201438 3.1e-10 457824
0.990802 GCST90029007 532396 12007571 6.69949908589424e-05 35.6701208827782
394 rs7201895 16 407723 G A G 0.354259 -0.0150043 0.00201818 4.3e-14 457824
0.985077 GCST90029007 532396 12007571 0.000103808236265032 55.2726198912699
395 rs3211995 16 2089006 G A G 0.159902 -0.0160011 0.00263085 2.5e-10 457824
0.98718 GCST90029007 532396 12007571 6.94772381266293e-05 36.9918348057042
396 rs879620 16 4015729 C T C 0.613649 0.0230719 0.00197669 3.1e-32 457824
0.993348 GCST90029007 532396 12007571 0.000255825416196187 136.234768946819
397 rs249293 16 9412222 G C G 0.696235 0.0133513 0.00209536 1.1e-10 457824
0.990379 GCST90029007 532396 12007571 7.62538734072105e-05 40.6002006013154
398 rs12445679 16 19838883 C G C 0.211096 -0.0208146 0.00235386 1.1e-18
457824 0.997308 GCST90029007 532396 12007571 0.000146850799724864
78.1939675153823
399 rs35154326 16 24862414 A G A 0.273986 -0.0123427 0.00216784 1.1e-08
457824 0.977586 GCST90029007 532396 12007571 6.08842091557544e-05
32.4163612937898
400 rs35626515 16 28649651 C A C 0.405823 0.0259059 0.00195545 1.9e-40
457824 0.991127 GCST90029007 532396 12007571 0.000329553559913689
175.510177980643
401 rs8059619 16 29925445 C T C 0.54756 0.0232727 0.00192641 2e-34 457824
1 GCST90029007 532396 12007571 0.000274057866452743 145.946761610344
402 rs2193101 16 49035172 C G C 0.813086 -0.0149264 0.00249156 2e-09
457824 0.970527 GCST90029007 532396 12007571 6.74067506158812e-05
35.8893687731221
403 rs16952396 16 53650663 C A C 0.162476 0.0182309 0.00260291 1.1e-12
457824 0.99683 GCST90029007 532396 12007571 9.21346763641397e-05
49.0564686900761
404 rs62033399 16 53810943 C T C 0.394797 0.073833 0.00196337 1e-306
457824 0.999216 GCST90029007 532396 12007571 0.00264917042587996
1414.14876079082
405 rs5011579 16 69187318 C G C 0.715152 0.0129765 0.00212773 1e-09
457824 0.994326 GCST90029007 532396 12007571 6.9858134541892e-05
37.1946500301621
406 rs862320 16 69651866 C T C 0.409857 -0.0229755 0.00195438 4.3e-33
457824 0.996355 GCST90029007 532396 12007571 0.000259516167246311
138.200715664973
407 rs12149660 16 70309237 G A G 0.115069 -0.0226301 0.003022 2.6e-14
457824 0.988949 GCST90029007 532396 12007571 0.000105318217646961
56.0766930633013
408 rs34270657 16 72042635 A G A 0.106901 0.0200256 0.00314568 8.3e-11
457824 0.970667 GCST90029007 532396 12007571 7.61156832612538e-05
40.526617785395
409 rs4888164 16 81726108 A C A 0.438399 0.0114825 0.00194397 4.7e-09
457824 0.985505 GCST90029007 532396 12007571 6.55285484404241e-05

34.8892922630692
410 rs11150461 16 82448195 C G C 0.72773 -0.0150547 0.00216401 4.6e-12 457824 0.990903 GCST90029007 532396 12007571 9.08973919746516e-05 48.3976253208721
411 rs72817412 16 89141490 C T C 0.048141 -0.0256312 0.00455335 7e-09 457824 0.958905 GCST90029007 532396 12007571 5.95134571552065e-05 31.6864932814493
412 rs4790292 17 1824305 C A C 0.153833 -0.0258933 0.00267562 5.9e-22 457824 0.991526 GCST90029007 532396 12007571 0.000175879314793655 93.6535636448552
413 rs118136827 17 2168104 G T G 0.281214 -0.0136563 0.00213903 8.1e-11 457824 0.996479 GCST90029007 532396 12007571 7.65533701091869e-05 40.7596752164128
414 rs12450045 17 4742112 A G A 0.552119 -0.0112577 0.00193914 5.4e-09 457824 0.989883 GCST90029007 532396 12007571 6.33021901287881e-05 33.7038397382972
415 rs6502482 17 15899241 C G C 0.561367 0.0132667 0.00193942 1.9e-11 457824 0.998149 GCST90029007 532396 12007571 8.78838601396856e-05 46.7929521804727
416 rs1320251 17 21264396 C T C 0.454489 -0.0182073 0.00193799 1.4e-20 457824 0.990754 GCST90029007 532396 12007571 0.000165760578567263 88.2645682516399
417 rs2306593 17 34866546 C T C 0.488201 -0.0162008 0.00192759 2.9e-17 457824 0.996003 GCST90029007 532396 12007571 0.000132663420130976 70.6385800528337
418 rs6503600 17 39270542 G C G 0.694117 -0.0139973 0.00208856 9.5e-12 457824 0.999581 GCST90029007 532396 12007571 8.43574043013064e-05 44.9151648323087
419 rs56161855 17 46288649 A T A 0.132734 0.023922 0.00283515 5e-17 457824 0.998584 GCST90029007 532396 12007571 0.000133705734575368 71.1936498527692
420 rs11079849 17 47090785 C T C 0.328516 -0.0210387 0.00205215 1.8e-24 457824 0.994153 GCST90029007 532396 12007571 0.000197378155083823 105.103690670254
421 rs2332306 17 52937056 A G A 0.157115 0.014899 0.00265052 1.4e-08 457824 0.991452 GCST90029007 532396 12007571 5.93460079978435e-05 31.5973337576258
422 rs62058023 17 55336891 T C T 0.13409 0.0158115 0.00283265 4.2e-08 457824 0.992937 GCST90029007 532396 12007571 5.85194333279849e-05 31.1572184899893
423 rs7218014 17 65832016 T C T 0.197125 0.0188576 0.0024212 2e-15 457824 0.996759 GCST90029007 532396 12007571 0.000113927152758149 60.661043505492
424 rs1285245 17 77796889 G C G 0.374277 -0.0117453 0.00198999 3.9e-09 457824 0.995081 GCST90029007 532396 12007571 6.54279390278694e-05 34.8357214002593
425 rs11150745 17 78757626 A G A 0.31799 -0.0209819 0.00206819 1e-24 457824 0.994449 GCST90029007 532396 12007571 0.000193281383150105 102.921741557382
426 rs3935190 17 79084367 G A G 0.536759 -0.0162788 0.00193982 4.1e-17 457824 0.985972 GCST90029007 532396 12007571 0.000132260354368188 70.4239333978845
427 rs9955276 18 1839339 C T C 0.144379 0.0207338 0.00274889 1.4e-14 457824 0.98291 GCST90029007 532396 12007571 0.00010684689298948 56.8907233432703
428 rs512121 18 7548501 T C T 0.191366 -0.0145389 0.00245454 4.6e-09 457824 0.990304 GCST90029007 532396 12007571 6.58960629668687e-05 35.0849805092679
429 rs1788808 18 21090023 A G A 0.495036 -0.0204963 0.00192804 1.1e-26 457824 0.996571 GCST90029007 532396 12007571 0.000212222730507151 113.010091695859
430 rs7232171 18 31251221 G T G 0.582724 0.013249 0.00195071 1.5e-11 457824 0.997445 GCST90029007 532396 12007571 8.66379899088169e-05 46.1295425703584
431 rs559231 18 39644247 G T G 0.392677 0.014086 0.00197769 5.9e-13 457824 0.990924 GCST90029007 532396 12007571 9.52758362551888e-05 50.7291168262731
432 rs1834144 18 40744790 C A C 0.373306 -0.0142834 0.0019935 3.9e-13 457824 0.994061 GCST90029007 532396 12007571 9.64170984624462e-05 51.3368344674392
433 rs784257 18 53397199 T C T 0.812578 0.0170235 0.00247665 1.7e-11 457824 0.987731 GCST90029007 532396 12007571 8.87350328681188e-05 47.2461914811432
434 rs9951619 18 56882326 T G T 0.767715 0.0153225 0.00229204 9.8e-12

457824 0.983447 GCST90029007 532396 12007571 8.39351047993431e-05 44.6902972693263
435 rs6567160 18 57829135 T C T 0.232618 0.0542686 0.0022763 1.8e-126 457824 0.997773 GCST90029007 532396 12007571 0.00106644891140892 568.377146930252
436 rs57636386 18 58048295 T C T 0.083662 -0.0424548 0.00348359 5.2e-34 457824 0.991133 GCST90029007 532396 12007571 0.000278896846752302 148.524430825268
437 rs9955040 18 63349078 A T A 0.25138 0.0154183 0.00222055 1.4e-12 457824 0.996018 GCST90029007 532396 12007571 9.05477702148528e-05 48.2114550155168
438 rs11150911 18 73498528 A C A 0.722814 -0.0117042 0.00215667 1.7e-08 457824 0.991862 GCST90029007 532396 12007571 5.53168276604357e-05 29.4519763353492
439 rs12974458 19 1866115 C T C 0.543327 0.0148572 0.00194031 9.9e-15 457824 0.988773 GCST90029007 532396 12007571 0.00011015660020319 58.6313729331862
440 rs45486197 19 2244849 G A G 0.065776 0.0245605 0.00392042 2e-10 457824 0.981249 GCST90029007 532396 12007571 7.37127166997898e-05 39.247101105113
441 rs72976986 19 4050424 G A G 0.189988 -0.0220969 0.00247738 2.7e-19 457824 0.983024 GCST90029007 532396 12007571 0.000149409347736496 79.5565267675968
442 rs273512 19 18224729 C T C 0.405161 0.0167506 0.00196263 8.7e-18 457824 0.996362 GCST90029007 532396 12007571 0.000136801125318437 72.8420631889989
443 rs9636202 19 18449238 G A G 0.266996 -0.0180417 0.00218454 9.7e-17 457824 0.986616 GCST90029007 532396 12007571 0.000128098631927139 68.2076803567579
444 rs8112818 19 18812785 A G A 0.400386 -0.0201737 0.00196928 5.6e-25 457824 0.991414 GCST90029007 532396 12007571 0.000197076932666185 104.943258385348
445 rs10416080 19 19356992 G A G 0.093923 0.0190892 0.0033122 2e-09 457824 0.988842 GCST90029007 532396 12007571 6.23850420876779e-05 33.2154942472338
446 rs113701136 19 30277729 C T C 0.319261 0.0211416 0.0020757 4.3e-24 457824 0.989916 GCST90029007 532396 12007571 0.000194817126186711 103.739679345249
447 rs11084553 19 31019780 A G A 0.154353 -0.0234458 0.00266447 6.1e-19 457824 0.996992 GCST90029007 532396 12007571 0.000145415433021262 77.4295635014271
448 rs429358 19 45411941 T C T 0.154421 -0.0256068 0.00266543 9.9e-22 457824 1 GCST90029007 532396 12007571 0.000173326908390102 92.2942031343318
449 rs10423928 19 46182304 T A T 0.194362 -0.034031 0.00242863 2e-44 457824 1 GCST90029007 532396 12007571 0.000368664897982088 196.34736607796
450 rs7259070 19 47562509 T C T 0.596469 0.0226076 0.00197988 2.3e-30 457824 0.977562 GCST90029007 532396 12007571 0.000244844330668644 130.385576751271
451 rs3764625 19 49649051 T G T 0.58732 -0.012037 0.0019582 7.9e-10 457824 0.997612 GCST90029007 532396 12007571 7.09670615082607e-05 37.7851192435104
452 rs1884389 20 1410582 C T C 0.438292 -0.0107362 0.00194767 3.2e-08 457824 0.990338 GCST90029007 532396 12007571 5.70703934589361e-05 30.3856691772723
453 rs1884897 20 6612832 A G A 0.627453 0.0208349 0.00199887 2.1e-25 457824 0.992545 GCST90029007 532396 12007571 0.000204028266449708 108.64559165995
454 rs16996657 20 15816236 T C T 0.127928 0.0184519 0.0028911 1.4e-10 457824 0.99107 GCST90029007 532396 12007571 7.65046700506249e-05 40.7337436285486
455 rs852042 20 17091233 A G A 0.758723 -0.0127478 0.00224871 1.3e-08 457824 0.996882 GCST90029007 532396 12007571 6.03590758534707e-05 32.1367495744379
456 rs6138524 20 25150535 A T A 0.207241 0.016759 0.00238833 4.1e-12 457824 0.991092 GCST90029007 532396 12007571 9.24767559150416e-05 49.238623416792
457 rs6142059 20 32544327 T C T 0.492684 0.0137647 0.00192966 2.9e-13 457824 0.995693 GCST90029007 532396 12007571 9.55642868259194e-05 50.8827154908162
458 rs117435593 20 36562529 G A G 0.0454790000000001 -0.0261584 0.0046734 2.3e-08 457824 0.974744 GCST90029007 532396 12007571 5.88431759118262e-05 31.3295973294077
459 rs3904162 20 44913138 C G C 0.542977 -0.0117024 0.00193289 3.9e-10

457824 0.99774 GCST90029007 532396 12007571 6.8844763935408e-05 36.6550627597703
460 rs79186842 20 47689036 A G A 0.146605 -0.0209216 0.00273505 4e-14
457824 0.991443 GCST90029007 532396 12007571 0.0001098946969279
58.5137076223974
461 rs35679975 20 51194941 T C T 0.192442 -0.0263938 0.00245104 2.2e-26
457824 0.99057 GCST90029007 532396 12007571 0.00021775777991536 115.958186277465
462 rs6023649 20 53470583 A G A 0.742498 -0.0148445 0.00223656 5.8e-11
457824 0.967086 GCST90029007 532396 12007571 8.27369150542532e-05
44.0522819032999
463 rs4341996 20 54377866 A C A 0.212804 -0.0135364 0.00236095 8.8e-09
457824 0.990364 GCST90029007 532396 12007571 6.17406476371764e-05
32.8723799201725
464 rs55886426 20 62567684 C G C 0.052804 -0.0259305 0.004473 6.9e-09
457824 0.924594 GCST90029007 532396 12007571 6.31192440200666e-05
33.6064280131497
465 rs17193211 21 38885506 C T C 0.066723 -0.0225903 0.00393022 1.3e-08
457824 0.96796 GCST90029007 532396 12007571 6.20509651573163e-05
33.037611559648
466 rs8132491 21 40288577 G A G 0.312847 -0.0143253 0.00213438 6.9e-12
457824 0.945086 GCST90029007 532396 12007571 8.46043001551112e-05
45.0466329156324
467 rs8134638 21 40644170 T C T 0.375807 0.0131914 0.0019938 1.7e-11
457824 0.998467 GCST90029007 532396 12007571 8.22144404189285e-05
43.7740736533653
468 rs394608 21 46581798 T C T 0.537623 0.018109 0.00194099 6.3e-21
457824 0.993709 GCST90029007 532396 12007571 0.000163469433694517
87.0443747770096
469 rs140733155 21 48048773 A G A 0.0115189999999999 0.0541353 0.00929932
6.8e-09 457824 0.944831 GCST90029007 532396 12007571 6.36496885870623e-05
33.8888693215993
470 rs406388 22 18226997 C G C 0.177566 0.0151133 0.00253134 4.4e-10
457824 0.992648 GCST90029007 532396 12007571 6.69504933479924e-05
35.6464275014184
471 rs2238799 22 20109325 A G A 0.389625 -0.0112777 0.00198899 3.5e-08
457824 0.988747 GCST90029007 532396 12007571 6.03830251884372e-05
32.1495015963375
472 rs6000329 22 36959219 G A G 0.560503 -0.0116332 0.00198561 9e-09
457824 0.955674 GCST90029007 532396 12007571 6.44685204689079e-05
34.3248663598733
473 rs4820410 22 40690385 A G A 0.345518 -0.0169691 0.00202896 3.3e-16
457824 0.998978 GCST90029007 532396 12007571 0.000131364739514995
69.9469876971648
474 rs28489620 22 41804716 G A G 0.290573 -0.0148085 0.00213974 4.5e-12
457824 0.981569 GCST90029007 532396 12007571 8.9955200502516e-05
47.8959175031987
475 rs79966207 22 50722408 T C T 0.176026 0.0148195 0.00253397 2.3e-09
457824 1 GCST90029007 532396 12007571 6.42394007123853e-05 34.202868674656
476
