# Supplementary material for: SLIT2 and ANGPTL3 as putative mediators linking obesity to atrial fibrillation: A Mendelian randomization study
Source: Medicine (Baltimore). 2026 Jul 3;105(27):e49568. doi: 10.1097/MD.0000000000049568 (PMC13336997; doi:10.1097/MD.0000000000049568)
Supplement: Supplementary file 3 [file medi-105-e49568-s003.pdf]

```
1 SNP effect_allele.exposure other_allele.exposure effect_allele.outcome other_allele.outcome beta.exposure beta.outcome eaf.exposure eaf.outcome remove palindromic ambiguous id.outcome se.outcome pval.outcome samplesize.outcome outcome mr_keep.outcome pval_origin.outcome id.exposure exposure chr.exposure pos.exposure se.exposure pval.exposure samplesize.exposure mr_keep.exposure pval_origin.exposure data_source.exposure action SNP_index mr_keep
2 rs10000798 T C T C -0.0646 5e-04 0.14406 NA FALSE FALSE FALSE GCST006061 0.0094 0.9551 537409 outcome TRUE reported 12549_33_HPGDS_PTGD2 HPGDS chr4 26173177 0.011352 1.264e-08 35349 TRUE reported textfile 2 1 TRUE
3 rs1047891 A C A C -0.0571 0.0155 0.26363 NA FALSE FALSE FALSE GCST006061 0.0079 0.05042 537409 outcome TRUE reported 12549_33_HPGDS_PTGD2 HPGDS chr2 210675783 0.008901 1.408e-10 35344 TRUE reported textfile 2 1 TRUE
4 rs115074597 A G A G 0.1103 -0.0055 0.04401 NA FALSE FALSE FALSE GCST006061 0.0233 0.8145 537409 outcome TRUE reported 12549_33_HPGDS_PTGD2 HPGDS chr4 91684191 0.019394 1.29e-08 35341 TRUE reported textfile 2 1 TRUE
5 rs11729397 A G A G -0.259 -0.0094 0.10547 NA FALSE FALSE FALSE GCST006061 0.0139 0.5002 537409 outcome TRUE reported 12549_33_HPGDS_PTGD2 HPGDS chr4 94160524 0.013109 6.881e-87 35334 TRUE reported textfile 2 1 TRUE
6 rs12342201 A G A G -0.0659 -0.0063 0.49613 NA FALSE FALSE FALSE GCST006061 0.0074 0.3926 537409 outcome TRUE reported 12549_33_HPGDS_PTGD2 HPGDS chr9 93132682 0.007789 2.661e-17 35348 TRUE reported textfile 2 1 TRUE
7 rs138042382 G A G A -0.2121 -0.0125 0.01472 NA FALSE FALSE FALSE GCST006061 0.0372 0.7362 537409 outcome TRUE reported 12549_33_HPGDS_PTGD2 HPGDS chr4 91760587 0.03285 1.07e-10 35341 TRUE reported textfile 2 1 TRUE
8 rs17022003 C A C A -0.2019 -0.0161 0.03783 NA FALSE FALSE FALSE GCST006061 0.0178 0.3638 537409 outcome TRUE reported 12549_33_HPGDS_PTGD2 HPGDS chr4 94681399 0.021343 3.085e-21 35322 TRUE reported textfile 2 1 TRUE
9 rs181514001 T C T C -0.2094 0.0405 0.01463 NA FALSE FALSE FALSE GCST006061 0.0391 0.3001 537409 outcome TRUE reported 12549_33_HPGDS_PTGD2 HPGDS chr4 93589240 0.033206 2.86e-10 35336 TRUE reported textfile 2 1 TRUE
10 rs282451 T C T C -0.2565 -0.0138 0.01727 NA FALSE FALSE FALSE GCST006061 0.0275 0.6164 537409 outcome TRUE reported 12549_33_HPGDS_PTGD2 HPGDS chr4 94171961 0.030674 6.153e-17 35334 TRUE reported textfile 2 1 TRUE
11 rs28649197 G T G T 0.3632 -0.0279 0.02021 NA FALSE FALSE FALSE GCST006061 0.0286 0.3301 537409 outcome TRUE reported 12549_33_HPGDS_PTGD2 HPGDS chr4 94228903 0.028589 5.62e-37 35334 TRUE reported textfile 2 1 TRUE
12 rs4657825 G A G A 0.0748 0.0023 0.24343 NA FALSE FALSE FALSE GCST006061 0.0083 0.7823 537409 outcome TRUE reported 12549_33_HPGDS_PTGD2 HPGDS chr1 196615191 0.009081 1.764e-16 35329 TRUE reported textfile 2 1 TRUE
13 rs56028059 G T G T 0.3274 -0.0529 0.0147 NA FALSE FALSE FALSE GCST006061 0.0481 0.2705 537409 outcome TRUE reported 12549_33_HPGDS_PTGD2 HPGDS chr4 94130304 0.032996 3.325e-23 35334 TRUE reported textfile 2 1 TRUE
14 rs62310110 A G A G 0.2634 0.0142 0.02231 NA FALSE FALSE FALSE GCST006061 0.0402 0.7235 537409 outcome TRUE reported 12549_33_HPGDS_PTGD2 HPGDS chr4 93489784 0.025937 3.131e-24 35336 TRUE reported textfile 2 1 TRUE
15 rs74354031 C T C T -0.8504 -0.0133 0.02323 NA FALSE FALSE FALSE GCST006061 0.0274 0.6263 537409 outcome TRUE reported 12549_33_HPGDS_PTGD2 HPGDS chr4 93906083 0.026855 1e-200 35333 TRUE reported textfile 2 1 TRUE
16 rs78621898 A G A G 0.2108 -0.0167 0.02344 NA FALSE FALSE FALSE GCST006061 0.0683 0.8069 537409 outcome TRUE reported 12549_33_HPGDS_PTGD2 HPGDS chr4 93786882 0.026767 3.394e-15 35334 TRUE reported
```

textfile 2 1 TRUE
17 rs78736235 G A G A 0.1084 -0.0014 0.04373 NA FALSE FALSE FALSE
GCST006061 0.0261 0.9582 537409 outcome TRUE reported 12549_33_HPGDS_PTGD2
HPGDS chr4 97027710 0.019551 2.948e-08 35329 TRUE reported
textfile 2 1 TRUE
18 rs10414043 A G A G -0.0862 0.0012 0.14467 NA FALSE FALSE FALSE
GCST006061 0.0112 0.9167 537409 outcome TRUE reported 3169_70_IDUA_IDUA
IDUA chr19 44912456 0.011322 2.664e-14 35367 TRUE reported
textfile 2 1 TRUE
19 rs114480470 T C T C -0.2392 -0.0133 0.03792 NA FALSE FALSE FALSE
GCST006061 0.028 0.6361 537409 outcome TRUE reported 3169_70_IDUA_IDUA
IDUA chr4 731515 0.021738 3.673e-28 35276 TRUE reported textfile
2 1 TRUE
20 rs139974673 C T C T 0.242 0.0351 0.02734 NA FALSE FALSE FALSE
GCST006061 0.0241 0.1443 537409 outcome TRUE reported 3169_70_IDUA_IDUA
IDUA chr15 43735687 0.024051 8.143e-24 35362 TRUE reported
textfile 2 1 TRUE
21 rs140624338 C T C T 0.3243 0.0021 0.01047 NA FALSE FALSE FALSE
GCST006061 0.0539 0.9695 537409 outcome TRUE reported 3169_70_IDUA_IDUA
IDUA chr12 101963654 0.039031 9.664e-17 35362 TRUE reported
textfile 2 1 TRUE
22 rs141617671 G A G A -0.3038 -0.0061 0.01759 NA FALSE FALSE FALSE
GCST006061 0.0298 0.8374 537409 outcome TRUE reported 3169_70_IDUA_IDUA
IDUA chr4 2279597 0.031433 4.242e-22 35280 TRUE reported textfile
2 1 TRUE
23 rs148339463 T C T C 0.2589 0.0494 0.0161 NA FALSE FALSE FALSE
GCST006061 0.0515 0.3371 537409 outcome TRUE reported 3169_70_IDUA_IDUA
IDUA chr4 160640 0.033829 1.962e-14 35279 TRUE reported textfile
2 1 TRUE
24 rs185408056 A G A G -0.1119 0.0218 0.04323 NA FALSE FALSE FALSE
GCST006061 0.0307 0.4784 537409 outcome TRUE reported 3169_70_IDUA_IDUA
IDUA chr4 5595772 0.020295 3.515e-08 35378 TRUE reported textfile
2 1 TRUE
25 rs2244608 G A G A 0.0475 -0.0109 0.31579 NA FALSE FALSE FALSE
GCST006061 0.0076 0.1491 537409 outcome TRUE reported 3169_70_IDUA_IDUA
IDUA chr12 120979185 0.008553 2.8e-08 35365 TRUE reported textfile
2 1 TRUE
26 rs3999089 G A G A 0.054 -0.0523 0.48195 NA FALSE FALSE FALSE
GCST006061 0.0071 1.572e-13 537409 outcome TRUE reported 3169_70_IDUA_IDUA
IDUA chr10 63444048 0.007897 8.046e-12 35351 TRUE reported
textfile 2 1 TRUE
27 rs4678322 T G T G -0.0603 0.0226 0.23022 NA FALSE FALSE FALSE
GCST006061 0.0086 0.008492 537409 outcome TRUE reported 3169_70_IDUA_IDUA
IDUA chr3 136093681 0.009417 1.518e-10 35361 TRUE reported
textfile 2 1 TRUE
28 rs56083715 T C T C 0.2962 -0.0082 0.24029 NA FALSE FALSE FALSE
GCST006061 0.009 0.3613 537409 outcome TRUE reported 3169_70_IDUA_IDUA
IDUA chr4 878279 0.009717 1e-200 35276 TRUE reported textfile 2
1 TRUE
29 rs56252442 T G T G 0.0618 -0.0144 0.2494 NA FALSE FALSE FALSE
GCST006061 0.0084 0.08523 537409 outcome TRUE reported 3169_70_IDUA_IDUA
IDUA chr19 18118398 0.009097 1.095e-11 35353 TRUE reported
textfile 2 1 TRUE
30 rs825038 A C A C 0.1184 0.0218 0.05118 NA FALSE FALSE FALSE
GCST006061 0.0239 0.3624 537409 outcome TRUE reported 3169_70_IDUA_IDUA
IDUA chr12 101570303 0.017995 4.72e-11 35363 TRUE reported
textfile 2 1 TRUE
31 rs10013653 A C A C 0.2694 -0.0177 0.40733 NA FALSE FALSE FALSE
GCST006061 0.0075 0.01761 537409 outcome TRUE reported
19631_13_KNG1_Kininostatin KNG1 chr4 186194478 0.008445 1e-200 35392
TRUE reported textfile 2 1 TRUE
32 rs10935742 C A C A 0.0885 0.0017 0.08569 NA FALSE FALSE FALSE
GCST006061 0.0138 0.9027 537409 outcome TRUE reported
19631_13_KNG1_Kininostatin KNG1 chr3 149183074 0.014787 2.163e-09 35358
TRUE reported textfile 2 1 TRUE
33 rs112260799 G A G A -0.1283 0.0329 0.04358 NA FALSE FALSE FALSE

GCST006061 0.0185 0.0759 537409 outcome TRUE reported
19631_13_KNG1_Kininostatin KNG1 chr5 178139341 0.0206 4.721e-10 35364
TRUE reported textfile 2 1 TRUE
34 rs1260326 C T C T -0.0523 -0.0069 0.34132 NA FALSE FALSE FALSE
GCST006061 0.0073 0.3401 537409 outcome TRUE reported
19631_13_KNG1_Kininostatin KNG1 chr2 27508073 0.008685 1.724e-09 35373
TRUE reported textfile 2 1 TRUE
35 rs1391867 A G A G 0.0715 0.0231 0.16425 NA FALSE FALSE FALSE
GCST006061 0.0095 0.01527 537409 outcome TRUE reported
19631_13_KNG1_Kininostatin KNG1 chr4 185863168 0.011353 3.019e-10 35392
TRUE reported textfile 2 1 TRUE
36 rs1846899 T C T C -0.1467 -0.015 0.08575 NA FALSE FALSE FALSE
GCST006061 0.0114 0.1874 537409 outcome TRUE reported
19631_13_KNG1_Kininostatin KNG1 chr3 186755027 0.014869 5.842e-23 35366
TRUE reported textfile 2 1 TRUE
37 rs185903163 A C A C -0.2408 -0.0503 0.02347 NA FALSE FALSE FALSE
GCST006061 0.0287 0.0799 537409 outcome TRUE reported
19631_13_KNG1_Kininostatin KNG1 chr5 177656170 0.027973 7.411e-18 35356
TRUE reported textfile 2 1 TRUE
38 rs1970871 G A G A -0.0802 0.0036 0.18204 NA FALSE FALSE FALSE
GCST006061 0.0088 0.6798 537409 outcome TRUE reported
19631_13_KNG1_Kininostatin KNG1 chr4 185996569 0.010934 2.224e-13 35383
TRUE reported textfile 2 1 TRUE
39 rs5030062 C A C A 0.2433 -0.0031 0.35548 NA FALSE FALSE FALSE
GCST006061 0.0073 0.6701 537409 outcome TRUE reported
19631_13_KNG1_Kininostatin KNG1 chr3 186736391 0.008557 7.605e-178 35367
TRUE reported textfile 2 1 TRUE
40 rs6875461 C T C T 0.0862 -0.0089 0.3813 NA FALSE FALSE FALSE
GCST006061 0.0079 0.2569 537409 outcome TRUE reported
19631_13_KNG1_Kininostatin KNG1 chr5 177327664 0.008565 7.94e-24 35369
TRUE reported textfile 2 1 TRUE
41 rs75061705 G A G A 0.1176 -0.0042 0.17017 NA FALSE FALSE FALSE
GCST006061 0.0102 0.6808 537409 outcome TRUE reported
19631_13_KNG1_Kininostatin KNG1 chr4 186399882 0.011123 4.007e-26 35376
TRUE reported textfile 2 1 TRUE
42 rs76484380 A G A G 0.3176 0.0056 0.01098 NA FALSE FALSE FALSE
GCST006061 0.0481 0.9074 537409 outcome TRUE reported
19631_13_KNG1_Kininostatin KNG1 chr4 186170655 0.041499 1.961e-14 35393
TRUE reported textfile 2 1 TRUE
43 rs10013413 T C T C 0.0461 0.0108 0.26452 NA FALSE FALSE FALSE
GCST006061 0.0082 0.1852 537409 outcome TRUE reported 5648_28_CTRB2_CTRB2
CTRB2 chr4 148061127 0.008241 2.222e-08 35362 TRUE reported
textfile 2 1 TRUE
44 rs11166273 G A G A -0.0586 0.0172 0.14077 NA FALSE FALSE FALSE
GCST006061 0.0098 0.07923 537409 outcome TRUE reported 5648_28_CTRB2_CTRB2
CTRB2 chr1 99559271 0.010377 1.63e-08 35361 TRUE reported
textfile 2 1 TRUE
45 rs112716238 T C T C 0.3067 0.0164 0.02396 NA FALSE FALSE FALSE
GCST006061 0.0275 0.5492 537409 outcome TRUE reported 5648_28_CTRB2_CTRB2
CTRB2 chr16 75680975 0.026797 2.479e-30 35362 TRUE reported
textfile 2 1 TRUE
46 rs112963922 T C T C 0.3068 0.0542 0.01119 NA FALSE FALSE FALSE
GCST006061 0.0399 0.1741 537409 outcome TRUE reported 5648_28_CTRB2_CTRB2
CTRB2 chr14 94411531 0.033886 1.38e-19 35371 TRUE reported
textfile 2 1 TRUE
47 rs113400434 A G A G 0.6272 -0.0287 0.03811 NA FALSE FALSE FALSE
GCST006061 0.0216 0.1846 537409 outcome TRUE reported 5648_28_CTRB2_CTRB2
CTRB2 chr16 74983615 0.020718 1e-200 35364 TRUE reported textfile
2 1 TRUE
48 rs117187218 T C T C 0.161 -0.0254 0.03053 NA FALSE FALSE FALSE
GCST006061 0.0288 0.3787 537409 outcome TRUE reported 5648_28_CTRB2_CTRB2
CTRB2 chr16 73538219 0.024092 2.344e-11 35363 TRUE reported
textfile 2 1 TRUE
49 rs11724862 C T C T -0.0517 0.0096 0.21985 NA FALSE FALSE FALSE
GCST006061 0.0093 0.298 537409 outcome TRUE reported 5648_28_CTRB2_CTRB2
CTRB2 chr4 185651349 0.00873 3.173e-09 35383 TRUE reported textfile

2 1 TRUE
50 rs117274901 T C T C 0.5364 0.0058 0.01497 NA FALSE FALSE FALSE
GCST006061 0.0297 0.8459 537409 outcome TRUE reported 5648_28_CTRB2_CTRB2
CTRB2 chr16 74943388 0.034013 4.969e-56 35363 TRUE reported
textfile 2 1 TRUE
51 rs117323918 G A G A 0.1831 8e-04 0.03317 NA FALSE FALSE FALSE
GCST006061 0.0222 0.9711 537409 outcome TRUE reported 5648_28_CTRB2_CTRB2
CTRB2 chr16 77851019 0.023124 2.411e-15 35360 TRUE reported
textfile 2 1 TRUE
52 rs117445981 T C T C 0.1455 0.0464 0.02485 NA FALSE FALSE FALSE
GCST006061 0.0291 0.1115 537409 outcome TRUE reported 5648_28_CTRB2_CTRB2
CTRB2 chr16 78376897 0.025671 1.446e-08 35366 TRUE reported
textfile 2 1 TRUE
53 rs118003726 T C T C -0.1787 -0.0153 0.02617 NA FALSE FALSE FALSE
GCST006061 0.0231 0.5077 537409 outcome TRUE reported 5648_28_CTRB2_CTRB2
CTRB2 chr16 77395250 0.025533 2.581e-12 35360 TRUE reported
textfile 2 1 TRUE
54 rs13405197 C T C T -0.0588 0.0483 0.13135 NA FALSE FALSE FALSE
GCST006061 0.0103 2.458e-06 537409 outcome TRUE reported 5648_28_CTRB2_CTRB2
CTRB2 chr2 86357127 0.010738 4.354e-08 35345 TRUE reported
textfile 2 1 TRUE
55 rs148793797 A G A G 0.5313 0.0361 0.02636 NA FALSE FALSE FALSE
GCST006061 0.0358 0.314 537409 outcome TRUE reported 5648_28_CTRB2_CTRB2
CTRB2 chr16 75455734 0.026072 2.599e-92 35362 TRUE reported
textfile 2 1 TRUE
56 rs149263900 A G A G -0.5918 -0.0151 0.01569 NA FALSE FALSE FALSE
GCST006061 0.0278 0.5881 537409 outcome TRUE reported 5648_28_CTRB2_CTRB2
CTRB2 chr16 75030927 0.033292 1.088e-70 35365 TRUE reported
textfile 2 1 TRUE
57 rs17032925 C T C T -0.0746 -9.00E-04 0.08221 NA FALSE FALSE FALSE
GCST006061 0.0134 0.9475 537409 outcome TRUE reported 5648_28_CTRB2_CTRB2
CTRB2 chr2 67039351 0.013196 1.573e-08 35359 TRUE reported
textfile 2 1 TRUE
58 rs2641346 T C T C -0.0718 -0.0154 0.11627 NA FALSE FALSE FALSE
GCST006061 0.0115 0.1812 537409 outcome TRUE reported 5648_28_CTRB2_CTRB2
CTRB2 chr1 119900917 0.011266 1.854e-10 35361 TRUE reported
textfile 2 1 TRUE
59 rs75213011 A C A C -0.573 0.039 0.01182 NA FALSE FALSE FALSE
GCST006061 0.0527 0.4595 537409 outcome TRUE reported 5648_28_CTRB2_CTRB2
CTRB2 chr16 76303837 0.037278 2.561e-53 35359 TRUE reported
textfile 2 1 TRUE
60 rs76708035 G T G T 0.1384 0.0042 0.02833 NA FALSE FALSE FALSE
GCST006061 0.0277 0.8802 537409 outcome TRUE reported 5648_28_CTRB2_CTRB2
CTRB2 chr16 73270390 0.024677 2.042e-08 35368 TRUE reported
textfile 2 1 TRUE
61 rs79500103 G A G A 0.3275 -0.0745 0.01514 NA FALSE FALSE FALSE
GCST006061 0.0347 0.0318 537409 outcome TRUE reported 5648_28_CTRB2_CTRB2
CTRB2 chr16 76409370 0.032752 1.533e-23 35358 TRUE reported
textfile 2 1 TRUE
62 rs8099628 C A C A 0.0788 -0.0077 0.06761 NA FALSE FALSE FALSE
GCST006061 0.0134 0.5654 537409 outcome TRUE reported 5648_28_CTRB2_CTRB2
CTRB2 chr18 44795613 0.014439 4.829e-08 35349 TRUE reported
textfile 2 1 TRUE
63 rs10013413 T C T C 0.0604 0.0108 0.26452 NA FALSE FALSE FALSE
GCST006061 0.0082 0.1852 537409 outcome TRUE reported 15476_6_REG3G_REG3G
REG3G chr4 148061127 0.009171 4.526e-11 35362 TRUE reported
textfile 2 1 TRUE
64 rs10182495 T C T C 0.1183 7e-04 0.14917 NA FALSE FALSE FALSE
GCST006061 0.0102 0.9469 537409 outcome TRUE reported 15476_6_REG3G_REG3G
REG3G chr2 80325951 0.011781 9.994e-24 35352 TRUE reported
textfile 2 1 TRUE
65 rs112834521 A G A G 0.2034 -0.0436 0.06261 NA FALSE FALSE FALSE
GCST006061 0.0185 0.01833 537409 outcome TRUE reported 15476_6_REG3G_REG3G
REG3G chr2 78955172 0.017271 5.137e-32 35350 TRUE reported
textfile 2 1 TRUE
66 rs115154697 G A G A 0.2541 -0.006 0.01145 NA FALSE FALSE FALSE

GCST006061 0.056 0.9145 537409 outcome TRUE reported 15476_6_REG3G_REG3G
REG3G chr2 78235870 0.038617 4.706e-11 35354 TRUE reported
textfile 2 1 TRUE
67 rs13256317 G A G A 0.0497 0.0027 0.41078 NA FALSE FALSE FALSE
GCST006061 0.0071 0.7097 537409 outcome TRUE reported 15476_6_REG3G_REG3G
REG3G chr8 115982906 0.008172 1.187e-09 35349 TRUE reported
textfile 2 1 TRUE
68 rs1882682 T G T G 0.0963 -0.0198 0.08605 NA FALSE FALSE FALSE
GCST006061 0.0135 0.1442 537409 outcome TRUE reported 15476_6_REG3G_REG3G
REG3G chr2 77475425 0.015085 1.728e-10 35348 TRUE reported
textfile 2 1 TRUE
69 rs2523442 C T C T 0.059 -4.00E-04 0.28164 NA FALSE FALSE FALSE
GCST006061 0.0087 0.9621 537409 outcome TRUE reported 15476_6_REG3G_REG3G
REG3G chr6 29450040 0.00897 4.79e-11 35448 TRUE reported textfile
2 1 TRUE
70 rs2793831 C T C T -0.091 -0.0164 0.11439 NA FALSE FALSE FALSE
GCST006061 0.0117 0.1586 537409 outcome TRUE reported 15476_6_REG3G_REG3G
REG3G chr1 119981279 0.012649 6.269e-13 35361 TRUE reported
textfile 2 1 TRUE
71 rs31863 G A G A -0.0498 -0.0159 0.30462 NA FALSE FALSE FALSE GCST006061
0.0076 0.0357 537409 outcome TRUE reported 15476_6_REG3G_REG3G REG3G chr5
158793484 0.008798 1.51e-08 35369 TRUE reported textfile 2 1 TRUE
72 rs3806561 G A G A -0.0932 0 0.49672 NA FALSE FALSE FALSE GCST006061
0.0072 0.9999 537409 outcome TRUE reported 15476_6_REG3G_REG3G REG3G chr2
79139474 0.008401 1.341e-28 35350 TRUE reported textfile 2 1 TRUE
73 rs499241 T G T G -0.0613 0.0042 0.25357 NA FALSE FALSE FALSE
GCST006061 0.0082 0.6095 537409 outcome TRUE reported 15476_6_REG3G_REG3G
REG3G chr11 100775231 0.009225 3.03e-11 35357 TRUE reported
textfile 2 1 TRUE
74 rs6137646 C T C T 0.0899 0.0044 0.08536 NA FALSE FALSE FALSE
GCST006061 0.0129 0.7331 537409 outcome TRUE reported 15476_6_REG3G_REG3G
REG3G chr20 22409174 0.014228 2.644e-10 35369 TRUE reported
textfile 2 1 TRUE
75 rs718768 A G A G -0.0507 0.002 0.28191 NA FALSE FALSE FALSE
GCST006061 0.0082 0.8081 537409 outcome TRUE reported 15476_6_REG3G_REG3G
REG3G chr4 109902959 0.008962 1.538e-08 35356 TRUE reported
textfile 2 1 TRUE
76 rs7766106 T C T C 0.0565 0.006 0.47133 NA FALSE FALSE FALSE
GCST006061 0.007 0.3902 537409 outcome TRUE reported 15476_6_REG3G_REG3G
REG3G chr6 127133993 0.008096 2.973e-12 35355 TRUE reported
textfile 2 1 TRUE
77 rs7938011 A G A G 0.0569 -0.0088 0.18166 NA FALSE FALSE FALSE
GCST006061 0.0091 0.3337 537409 outcome TRUE reported 15476_6_REG3G_REG3G
REG3G chr11 65837391 0.010394 4.396e-08 35366 TRUE reported
textfile 2 1 TRUE
78 rs9983358 A C A C 0.0488 0.0054 0.40996 NA FALSE FALSE FALSE
GCST006061 0.0072 0.4523 537409 outcome TRUE reported 15476_6_REG3G_REG3G
REG3G chr21 38298850 0.008169 2.318e-09 35378 TRUE reported
textfile 2 1 TRUE
79 rs10013413 T C T C -0.0486 0.0108 0.26452 NA FALSE FALSE FALSE
GCST006061 0.0082 0.1852 537409 outcome TRUE reported
6580_29_PZP_Pregnancy_zone_protein PZP chr4 148061127 0.008757 2.859e-08
35362 TRUE reported textfile 2 1 TRUE
80 rs11045856 G T G T -0.0543 -0.017 0.22646 NA FALSE FALSE FALSE
GCST006061 0.0088 0.05224 537409 outcome TRUE reported
6580_29_PZP_Pregnancy_zone_protein PZP chr12 21197755 0.009828 3.299e-08
35423 TRUE reported textfile 2 1 TRUE
81 rs117006594 C T C T -0.3619 -0.0082 0.02166 NA FALSE FALSE FALSE
GCST006061 0.0331 0.8041 537409 outcome TRUE reported
6580_29_PZP_Pregnancy_zone_protein PZP chr12 9765670 0.027883 1.6e-38 35387
TRUE reported textfile 2 1 TRUE
82 rs12810163 G A G A -0.0607 0.0069 0.32606 NA FALSE FALSE FALSE
GCST006061 0.0078 0.3784 537409 outcome TRUE reported
6580_29_PZP_Pregnancy_zone_protein PZP chr12 9828566 0.008711 3.205e-12 35386
TRUE reported textfile 2 1 TRUE
83 rs2297441 A G A G 0.0455 -0.0154 0.31895 NA FALSE FALSE FALSE

GCST006061 0.0085 0.06834 537409 outcome TRUE reported
6580_29_PZP_Pregnancy_zone_protein PZP chr20 63696229 0.008278 3.869e-08
35256 TRUE reported textfile 2 1 TRUE
84 rs34022782 A G A G -0.1849 0.0075 0.08039 NA FALSE FALSE FALSE
GCST006061 0.0167 0.6541 537409 outcome TRUE reported
6580_29_PZP_Pregnancy_zone_protein PZP chr12 8663938 0.015063 1.231e-34 35384
TRUE reported textfile 2 1 TRUE
85 rs3782676 T C T C -0.6193 0.0143 0.09214 NA FALSE FALSE FALSE
GCST006061 0.014 0.3069 537409 outcome TRUE reported
6580_29_PZP_Pregnancy_zone_protein PZP chr12 9181311 0.010186 1e-200 35385
TRUE reported textfile 2 1 TRUE
86 rs4271437 A G A G 0.2179 0.001 0.4025 NA FALSE FALSE FALSE
GCST006061 0.0076 0.8989 537409 outcome TRUE reported
6580_29_PZP_Pregnancy_zone_protein PZP chr12 9320047 0.008319 3.282e-151 35386
TRUE reported textfile 2 1 TRUE
87 rs55680021 T C T C -0.0468 0.0034 0.37015 NA FALSE FALSE FALSE
GCST006061 0.0076 0.6549 537409 outcome TRUE reported
6580_29_PZP_Pregnancy_zone_protein PZP chr12 8042981 0.008498 3.652e-08 35383
TRUE reported textfile 2 1 TRUE
88 rs62385693 C T C T -0.0599 -0.0105 0.19556 NA FALSE FALSE FALSE
GCST006061 0.0087 0.2246 537409 outcome TRUE reported
6580_29_PZP_Pregnancy_zone_protein PZP chr5 132437982 0.009732 7.501e-10
35352 TRUE reported textfile 2 1 TRUE
89 rs7075901 A C A C -0.0725 -0.0542 0.48567 NA FALSE FALSE FALSE
GCST006061 0.0072 4.556e-14 537409 outcome TRUE reported
6580_29_PZP_Pregnancy_zone_protein PZP chr10 63521234 0.007692 4.302e-21
35351 TRUE reported textfile 2 1 TRUE
90 rs7222851 C T C T -0.0572 0.007 0.42039 NA FALSE FALSE FALSE
GCST006061 0.0075 0.3501 537409 outcome TRUE reported
6580_29_PZP_Pregnancy_zone_protein PZP chr17 47514803 0.007786 2.04e-13
35364 TRUE reported textfile 2 1 TRUE
91 rs75718176 T G T G -0.107 0.0129 0.05396 NA FALSE FALSE FALSE
GCST006061 0.0229 0.5741 537409 outcome TRUE reported
6580_29_PZP_Pregnancy_zone_protein PZP chr12 12113503 0.018263 4.662e-09
35376 TRUE reported textfile 2 1 TRUE
92 rs79391862 C A C A 0.1362 0.0467 0.06536 NA FALSE FALSE FALSE
GCST006061 0.0414 0.2586 537409 outcome TRUE reported
6580_29_PZP_Pregnancy_zone_protein PZP chr15 53447229 0.015538 1.855e-18
35362 TRUE reported textfile 2 1 TRUE
93 rs10013413 T C T C 0.0519 0.0108 0.26452 NA FALSE FALSE FALSE
GCST006061 0.0082 0.1852 537409 outcome TRUE reported 15503_20_LEFTY2_Lefty_A
LEFTY2 chr4 148061127 0.009022 8.779e-09 35362 TRUE reported
textfile 2 1 TRUE
94 rs114468433 A G A G 0.2104 -0.0371 0.02701 NA FALSE FALSE FALSE
GCST006061 0.0312 0.2354 537409 outcome TRUE reported 15503_20_LEFTY2_Lefty_A
LEFTY2 chr1 224523810 0.026518 2.119e-15 35365 TRUE reported
textfile 2 1 TRUE
95 rs141415111 A G A G -0.33 0.0297 0.02806 NA FALSE FALSE FALSE
GCST006061 0.022 0.1769 537409 outcome TRUE reported 15503_20_LEFTY2_Lefty_A
LEFTY2 chr1 226624199 0.026196 2.182e-36 35358 TRUE reported
textfile 2 1 TRUE
96 rs144135113 T G T G 0.1392 0.0224 0.03227 NA FALSE FALSE FALSE
GCST006061 0.0328 0.4946 537409 outcome TRUE reported 15503_20_LEFTY2_Lefty_A
LEFTY2 chr1 223992676 0.024584 1.494e-08 35362 TRUE reported
textfile 2 1 TRUE
97 rs145643169 T G T G -0.5162 0.0071 0.03459 NA FALSE FALSE FALSE
GCST006061 0.0287 0.804 537409 outcome TRUE reported 15503_20_LEFTY2_Lefty_A
LEFTY2 chr1 226208398 0.02358 3.109e-106 35357 TRUE reported textfile
2 1 TRUE
98 rs186015966 C A C A -0.2129 -0.0228 0.03265 NA FALSE FALSE FALSE
GCST006061 0.0391 0.5606 537409 outcome TRUE reported 15503_20_LEFTY2_Lefty_A
LEFTY2 chr1 222625498 0.024274 1.777e-18 35356 TRUE reported
textfile 2 1 TRUE
99 rs2615068 A G A G 0.1925 0.0183 0.28663 NA FALSE FALSE FALSE
GCST006061 0.0087 0.03649 537409 outcome TRUE reported 15503_20_LEFTY2_Lefty_A
LEFTY2 chr1 225750690 0.009481 1.192e-91 35355 TRUE reported

textfile 2 1 TRUE
100 rs2740168 A G A G 0.5252 0.0034 0.38154 NA FALSE FALSE FALSE
GCST006061 0.0073 0.6431 537409 outcome TRUE reported 15503_20_LEFTY2_Lefty_A
LEFTY2 chr1 225833287 0.006212 1e-200 35354 TRUE reported textfile
2 1 TRUE
101 rs56301120 A G A G 0.4541 -0.003 0.08876 NA FALSE FALSE FALSE
GCST006061 0.0122 0.8035 537409 outcome TRUE reported 15503_20_LEFTY2_Lefty_A
LEFTY2 chr1 225844317 0.01501 1e-200 35357 TRUE reported textfile 2
1 TRUE
102 rs58012510 A G A G 0.1961 -0.0097 0.04389 NA FALSE FALSE FALSE
GCST006061 0.015 0.5206 537409 outcome TRUE reported 15503_20_LEFTY2_Lefty_A
LEFTY2 chr1 224634051 0.02111 1.548e-20 35362 TRUE reported textfile
2 1 TRUE
103 rs61824945 G T G T -0.1813 0.0268 0.03228 NA FALSE FALSE FALSE
GCST006061 0.0406 0.5096 537409 outcome TRUE reported 15503_20_LEFTY2_Lefty_A
LEFTY2 chr1 227694048 0.024684 2.062e-13 35360 TRUE reported
textfile 2 1 TRUE
104 rs635634 C T C T 0.0908 0.0285 0.12995 NA FALSE FALSE FALSE
GCST006061 0.0089 0.001351 537409 outcome TRUE reported
15503_20_LEFTY2_Lefty_A LEFTY2 chr9 133279427 0.011783 1.299e-14 35371
TRUE reported textfile 2 1 TRUE
105 rs7532346 T C T C 0.1218 0.0067 0.05217 NA FALSE FALSE FALSE
GCST006061 0.0205 0.7444 537409 outcome TRUE reported 15503_20_LEFTY2_Lefty_A
LEFTY2 chr1 230174408 0.019367 3.193e-10 35356 TRUE reported
textfile 2 1 TRUE
106 rs77722489 G T G T -0.2163 0.0435 0.02157 NA FALSE FALSE FALSE
GCST006061 0.0248 0.07949 537409 outcome TRUE reported 15503_20_LEFTY2_Lefty_A
LEFTY2 chr1 227193770 0.030044 6.046e-13 35360 TRUE reported
textfile 2 1 TRUE
107 rs10013613 T G T G 0.0856 -0.0142 0.41912 NA FALSE FALSE FALSE
GCST006061 0.0073 0.05099 537409 outcome TRUE reported 5087_5_IL22RA2_IL_22BP
IL22RA2 chr4 102502336 0.00841 2.466e-24 35341 TRUE reported textfile
2 1 TRUE
108 rs10519515 A C A C 0.0465 2e-04 0.4067 NA FALSE FALSE FALSE
GCST006061 0.0075 0.9789 537409 outcome TRUE reported 5087_5_IL22RA2_IL_22BP
IL22RA2 chr4 139980600 0.008466 3.955e-08 35356 TRUE reported
textfile 2 1 TRUE
109 rs10849448 G A G A 0.113 0.0056 0.28422 NA FALSE FALSE FALSE
GCST006061 0.009 0.532 537409 outcome TRUE reported 5087_5_IL22RA2_IL_22BP
IL22RA2 chr12 6384185 0.009216 1.468e-34 35380 TRUE reported textfile
2 1 TRUE
110 rs111470468 A G A G -0.1845 -0.0272 0.02199 NA FALSE FALSE FALSE
GCST006061 0.0379 0.4741 537409 outcome TRUE reported 5087_5_IL22RA2_IL_22BP
IL22RA2 chr6 139374714 0.02902 2.049e-10 35351 TRUE reported textfile
2 1 TRUE
111 rs11641991 A G A G -0.0533 -0.0104 0.46213 NA FALSE FALSE FALSE
GCST006061 0.0072 0.148 537409 outcome TRUE reported 5087_5_IL22RA2_IL_22BP
IL22RA2 chr16 11610960 0.0084 2.222e-10 35379 TRUE reported textfile
2 1 TRUE
112 rs12232384 A C A C 0.0713 0.0044 0.23225 NA FALSE FALSE FALSE
GCST006061 0.0085 0.6014 537409 outcome TRUE reported 5087_5_IL22RA2_IL_22BP
IL22RA2 chr16 85976154 0.009922 6.662e-13 35379 TRUE reported
textfile 2 1 TRUE
113 rs139795227 C A C A -0.1507 0.0043 0.02759 NA FALSE FALSE FALSE
GCST006061 0.0384 0.9115 537409 outcome TRUE reported 5087_5_IL22RA2_IL_22BP
IL22RA2 chr1 92376810 0.025402 2.983e-09 35352 TRUE reported
textfile 2 1 TRUE
114 rs55714927 T C T C 0.0567 1e-04 0.21166 NA FALSE FALSE FALSE
GCST006061 0.0098 0.9926 537409 outcome TRUE reported 5087_5_IL22RA2_IL_22BP
IL22RA2 chr17 7176997 0.010243 3.108e-08 35370 TRUE reported textfile
2 1 TRUE
115 rs6600249 C T C T 0.1101 -0.0132 0.47274 NA FALSE FALSE FALSE
GCST006061 0.0071 0.06391 537409 outcome TRUE reported 5087_5_IL22RA2_IL_22BP
IL22RA2 chr1 24978669 0.008398 2.902e-39 35371 TRUE reported
textfile 2 1 TRUE
116 rs71560620 A G A G -0.2362 0.0281 0.01186 NA FALSE FALSE FALSE

GCST006061 0.0317 0.3753 537409 outcome TRUE reported 5087_5_IL22RA2_IL_22BP
IL22RA2 chr6 137012344 0.038218 6.394e-10 35364 TRUE reported
textfile 2 1 TRUE
117 rs7334951 G A G A 0.2113 -0.0044 0.09621 NA FALSE FALSE FALSE
GCST006061 0.0141 0.7578 537409 outcome TRUE reported 5087_5_IL22RA2_IL_22BP
IL22RA2 chr13 28041982 0.014159 2.332e-50 35363 TRUE reported
textfile 2 1 TRUE
118 rs74648690 T G T G -0.2018 -0.0163 0.0193 NA FALSE FALSE FALSE
GCST006061 0.0311 0.5996 537409 outcome TRUE reported 5087_5_IL22RA2_IL_22BP
IL22RA2 chr6 137690898 0.031045 8.02e-11 35364 TRUE reported
textfile 2 1 TRUE
119 rs75489957 T C T C 0.0802 -0.0295 0.12019 NA FALSE FALSE FALSE
GCST006061 0.0128 0.02159 537409 outcome TRUE reported 5087_5_IL22RA2_IL_22BP
IL22RA2 chr19 20837621 0.012735 3.021e-10 35355 TRUE reported
textfile 2 1 TRUE
120 rs763948 A G A G -0.0465 0.0062 0.45936 NA FALSE FALSE FALSE
GCST006061 0.0072 0.3931 537409 outcome TRUE reported 5087_5_IL22RA2_IL_22BP
IL22RA2 chr12 67762146 0.008356 2.619e-08 35367 TRUE reported
textfile 2 1 TRUE
121 rs76488192 A C A C 0.1088 0.0331 0.08325 NA FALSE FALSE FALSE
GCST006061 0.0193 0.08667 537409 outcome TRUE reported 5087_5_IL22RA2_IL_22BP
IL22RA2 chr6 137042781 0.015183 7.714e-13 35364 TRUE reported
textfile 2 1 TRUE
122 rs10013945 G A G A -0.3006 -0.0198 0.23676 NA FALSE FALSE FALSE
GCST006061 0.0089 0.02532 537409 outcome TRUE reported 3320_49_IGFBP7_IGFBP_7
IGFBP7 chr4 57073488 0.009658 1e-200 35358 TRUE reported textfile
2 1 TRUE
123 rs11133472 T C T C 0.1088 0.0217 0.36371 NA FALSE FALSE FALSE
GCST006061 0.0078 0.005748 537409 outcome TRUE reported
3320_49_IGFBP7_IGFBP_7 IGFBP7 chr4 57038221 0.008613 1.406e-36 35358
TRUE reported textfile 2 1 TRUE
124 rs139826977 G A G A 0.2894 0.002 0.03207 NA FALSE FALSE FALSE
GCST006061 0.033 0.9511 537409 outcome TRUE reported 3320_49_IGFBP7_IGFBP_7
IGFBP7 chr4 56540903 0.023581 1.272e-34 35356 TRUE reported
textfile 2 1 TRUE
125 rs143745420 C T C T 0.2373 0.0533 0.0155 NA FALSE FALSE FALSE
GCST006061 0.0613 0.3843 537409 outcome TRUE reported 3320_49_IGFBP7_IGFBP_7
IGFBP7 chr4 58649592 0.032725 4.128e-13 35362 TRUE reported
textfile 2 1 TRUE
126 rs145552690 G A G A 0.2061 -0.059 0.01531 NA FALSE FALSE FALSE
GCST006061 0.0311 0.05754 537409 outcome TRUE reported 3320_49_IGFBP7_IGFBP_7
IGFBP7 chr4 47252884 0.034188 1.655e-09 35346 TRUE reported
textfile 2 1 TRUE
127 rs36221780 A G A G -0.087 0.0063 0.08267 NA FALSE FALSE FALSE
GCST006061 0.0147 0.6694 537409 outcome TRUE reported 3320_49_IGFBP7_IGFBP_7
IGFBP7 chr21 45019995 0.014709 3.321e-09 35256 TRUE reported
textfile 2 1 TRUE
128 rs76023148 A G A G 0.4028 0.0308 0.02313 NA FALSE FALSE FALSE
GCST006061 0.0333 0.3553 537409 outcome TRUE reported 3320_49_IGFBP7_IGFBP_7
IGFBP7 chr4 56621832 0.027551 2.087e-48 35352 TRUE reported
textfile 2 1 TRUE
129 rs77202557 T C T C 0.1867 0.0127 0.0198 NA FALSE FALSE FALSE
GCST006061 0.0292 0.6639 537409 outcome TRUE reported 3320_49_IGFBP7_IGFBP_7
IGFBP7 chr4 67095343 0.029449 2.3e-10 35349 TRUE reported textfile
2 1 TRUE
130 rs77542162 G A G A 0.1993 0.0177 0.025 NA FALSE FALSE FALSE
GCST006061 0.0302 0.557 537409 outcome TRUE reported 3320_49_IGFBP7_IGFBP_7
IGFBP7 chr17 69085137 0.025809 1.144e-14 35371 TRUE reported
textfile 2 1 TRUE
131 rs142799933 G A G A -0.2024 0.005 0.01631 NA FALSE FALSE FALSE
GCST006061 0.0426 0.9058 537409 outcome TRUE reported
3708_62_A2M_a2_Macroglobulin A2M chr12 9530722 0.029368 5.51e-12 35385
TRUE reported textfile 2 1 TRUE
132 rs190543502 C T C T 0.2556 0.0395 0.02719 NA FALSE FALSE FALSE
GCST006061 0.0261 0.1297 537409 outcome TRUE reported
3708_62_A2M_a2_Macroglobulin A2M chr15 43464986 0.025269 4.74e-24 35363

TRUE reported textfile 2 1 TRUE
133 rs1973612 T C T C 0.2444 -0.0191 0.49071 NA FALSE FALSE FALSE
GCST006061 0.0071 0.007232 537409 outcome TRUE reported
3708_62_A2M_a2_Macroglobulin A2M chr4 186248013 0.00821 1.002e-194 35392
TRUE reported textfile 2 1 TRUE
134 rs205757 A G A G 0.0533 0.0126 0.33881 NA FALSE FALSE FALSE
GCST006061 0.0078 0.1073 537409 outcome TRUE reported
3708_62_A2M_a2_Macroglobulin A2M chr7 130897730 0.008729 1.019e-09 35355
TRUE reported textfile 2 1 TRUE
135 rs226379 C T C T -0.0982 6e-04 0.34878 NA FALSE FALSE FALSE
GCST006061 0.0077 0.9413 537409 outcome TRUE reported
3708_62_A2M_a2_Macroglobulin A2M chr12 9115989 0.008654 7.595e-30 35387
TRUE reported textfile 2 1 TRUE
136 rs2731672 C T C T 0.1769 -0.0025 0.25436 NA FALSE FALSE FALSE
GCST006061 0.0083 0.7666 537409 outcome TRUE reported
3708_62_A2M_a2_Macroglobulin A2M chr5 177415473 0.009507 2.852e-77 35362
TRUE reported textfile 2 1 TRUE
137 rs6440008 C T C T 0.0602 -0.0199 0.39039 NA FALSE FALSE FALSE
GCST006061 0.0074 0.007142 537409 outcome TRUE reported
3708_62_A2M_a2_Macroglobulin A2M chr3 141435700 0.008457 1.089e-12 35367
TRUE reported textfile 2 1 TRUE
138 rs80177247 G A G A -0.0663 0.0094 0.19547 NA FALSE FALSE FALSE
GCST006061 0.0103 0.3589 537409 outcome TRUE reported
3708_62_A2M_a2_Macroglobulin A2M chr10 5254280 0.010425 2.023e-10 35374
TRUE reported textfile 2 1 TRUE
139 rs1001581 T C T C -0.0588 -0.0031 0.36564 NA FALSE FALSE FALSE
GCST006061 0.0073 0.6736 537409 outcome TRUE reported 2652_15_PLAUR_suPAR
PLAUR chr19 43561236 0.008586 7.478e-12 35372 TRUE reported
textfile 2 1 TRUE
140 rs12045503 T C T C -0.0542 -0.0097 0.39965 NA FALSE FALSE FALSE
GCST006061 0.0075 0.1936 537409 outcome TRUE reported 2652_15_PLAUR_suPAR
PLAUR chr1 196703343 0.008368 9.332e-11 35339 TRUE reported
textfile 2 1 TRUE
141 rs143501783 C T C T 0.2894 -0.0333 0.01116 NA FALSE FALSE FALSE
GCST006061 0.0443 0.4525 537409 outcome TRUE reported 2652_15_PLAUR_suPAR
PLAUR chr17 7897001 0.037289 8.424e-15 35373 TRUE reported textfile
2 1 TRUE
142 rs2227576 G T G T 0.1307 -0.0272 0.42138 NA FALSE FALSE FALSE
GCST006061 0.0074 0.0002281 537409 outcome TRUE reported 2652_15_PLAUR_suPAR
PLAUR chr10 73918518 0.008251 1.614e-56 35370 TRUE reported
textfile 2 1 TRUE
143 rs2302524 C T C T -0.2438 0.0208 0.17537 NA FALSE FALSE FALSE
GCST006061 0.0098 0.03386 537409 outcome TRUE reported 2652_15_PLAUR_suPAR
PLAUR chr19 43652320 0.010782 3.293e-113 35369 TRUE reported
textfile 2 1 TRUE
144 rs2470854 G A G A -0.0628 -0.0016 0.45355 NA FALSE FALSE FALSE
GCST006061 0.0073 0.8314 537409 outcome TRUE reported 2652_15_PLAUR_suPAR
PLAUR chr3 98985392 0.008266 3.022e-14 35331 TRUE reported
textfile 2 1 TRUE
145 rs3184504 C T C T -0.0468 0.0074 0.37862 NA FALSE FALSE FALSE
GCST006061 0.0075 0.3235 537409 outcome TRUE reported 2652_15_PLAUR_suPAR
PLAUR chr12 111446804 0.008454 3.101e-08 35371 TRUE reported
textfile 2 1 TRUE
146 rs41556717 T C T C 0.1797 -0.0483 0.01931 NA FALSE FALSE FALSE
GCST006061 0.0325 0.1371 537409 outcome TRUE reported 2652_15_PLAUR_suPAR
PLAUR chr17 7496000 0.028714 3.895e-10 35374 TRUE reported textfile
2 1 TRUE
147 rs4251824 T C T C -0.2892 0.0016 0.02862 NA FALSE FALSE FALSE
GCST006061 0.0221 0.9421 537409 outcome TRUE reported 2652_15_PLAUR_suPAR
PLAUR chr19 43666441 0.024358 1.639e-32 35367 TRUE reported
textfile 2 1 TRUE
148 rs62061425 G A G A 0.1238 0.001 0.21627 NA FALSE FALSE FALSE
GCST006061 0.0097 0.9151 537409 outcome TRUE reported 2652_15_PLAUR_suPAR
PLAUR chr17 7169772 0.010003 3.528e-35 35370 TRUE reported textfile
2 1 TRUE
149 rs6717518 G T G T -0.0764 0.0021 0.10681 NA FALSE FALSE FALSE

GCST006061 0.0127 0.8677 537409 outcome TRUE reported 2652_15_PLAUR_suPAR
PLAUR chr2 161443201 0.013357 1.066e-08 35343 TRUE reported
textfile 2 1 TRUE
150 rs72957552 G A G A -0.1313 -0.0037 0.47037 NA FALSE FALSE FALSE
GCST006061 0.0072 0.6087 537409 outcome TRUE reported 2652_15_PLAUR_suPAR
PLAUR chr2 159877871 0.008204 1.209e-57 35348 TRUE reported
textfile 2 1 TRUE
151 rs8176741 A G A G 0.1578 0.0181 0.06497 NA FALSE FALSE FALSE
GCST006061 0.0128 0.1561 537409 outcome TRUE reported 2652_15_PLAUR_suPAR
PLAUR chr9 133256074 0.016661 2.759e-21 35370 TRUE reported
textfile 2 1 TRUE
152 rs9948409 G A G A 0.0586 -0.0013 0.34814 NA FALSE FALSE FALSE
GCST006061 0.0079 0.8709 537409 outcome TRUE reported 2652_15_PLAUR_suPAR
PLAUR chr18 27181964 0.008566 7.857e-12 35367 TRUE reported
textfile 2 1 TRUE
153 rs10015837 C T C T -0.0861 0.0021 0.30399 NA FALSE FALSE FALSE
GCST006061 0.008 0.793 537409 outcome TRUE reported 5660_51_SOD3_SOD3
SOD3 chr4 25491785 0.009484 1.098e-19 35352 TRUE reported
textfile 2 1 TRUE
154 rs10805206 T C T C 0.0504 0.004 0.36349 NA FALSE FALSE FALSE
GCST006061 0.0075 0.5965 537409 outcome TRUE reported 5660_51_SOD3_SOD3
SOD3 chr4 8514905 0.009028 2.368e-08 35365 TRUE reported textfile
2 1 TRUE
155 rs112992178 T C T C 0.212 0.0082 0.03892 NA FALSE FALSE FALSE
GCST006061 0.0184 0.6566 537409 outcome TRUE reported 5660_51_SOD3_SOD3
SOD3 chr4 17621287 0.022299 1.962e-21 35355 TRUE reported
textfile 2 1 TRUE
156 rs184693375 A G A G 0.3724 -0.0063 0.01711 NA FALSE FALSE FALSE
GCST006061 0.0428 0.8832 537409 outcome TRUE reported 5660_51_SOD3_SOD3
SOD3 chr4 22752935 0.032632 3.638e-30 35357 TRUE reported
textfile 2 1 TRUE
157 rs2695234 G A G A -0.5368 -0.0059 0.07858 NA FALSE FALSE FALSE
GCST006061 0.0134 0.6571 537409 outcome TRUE reported 5660_51_SOD3_SOD3
SOD3 chr4 24802616 0.015862 1e-200 35354 TRUE reported textfile
2 1 TRUE
158 rs333947 A G A G -0.0703 -0.0071 0.14937 NA FALSE FALSE FALSE
GCST006061 0.01 0.4808 537409 outcome TRUE reported 5660_51_SOD3_SOD3
SOD3 chr1 109928142 0.01151 1.009e-09 35349 TRUE reported textfile
2 1 TRUE
159 rs4510458 A C A C 0.1552 0.0043 0.24126 NA FALSE FALSE FALSE
GCST006061 0.0087 0.6217 537409 outcome TRUE reported 5660_51_SOD3_SOD3
SOD3 chr4 25022020 0.010191 2.29e-52 35360 TRUE reported
textfile 2 1 TRUE
160 rs4696870 C A C A -0.0857 0.0075 0.24698 NA FALSE FALSE FALSE
GCST006061 0.0093 0.4178 537409 outcome TRUE reported 5660_51_SOD3_SOD3
SOD3 chr4 8617320 0.0101 2.147e-17 35357 TRUE reported textfile 2
1 TRUE
161 rs4697596 T C T C 0.0787 -0.0042 0.1966 NA FALSE FALSE FALSE
GCST006061 0.009 0.6408 537409 outcome TRUE reported 5660_51_SOD3_SOD3
SOD3 chr4 25659535 0.010877 4.633e-13 35347 TRUE reported
textfile 2 1 TRUE
162 rs56344867 C T C T 0.0508 -0.005 0.35465 NA FALSE FALSE FALSE
GCST006061 0.008 0.5298 537409 outcome TRUE reported 5660_51_SOD3_SOD3
SOD3 chr11 126361219 0.00851 2.381e-09 35374 TRUE reported textfile
2 1 TRUE
163 rs62410642 T C T C 0.1328 0.0425 0.04535 NA FALSE FALSE FALSE
GCST006061 0.033 0.1974 537409 outcome TRUE reported 5660_51_SOD3_SOD3
SOD3 chr4 25543497 0.020952 2.321e-10 35350 TRUE reported
textfile 2 1 TRUE
164 rs78842674 T C T C 0.1974 -0.0166 0.02488 NA FALSE FALSE FALSE
GCST006061 0.0551 0.7633 537409 outcome TRUE reported 5660_51_SOD3_SOD3
SOD3 chr4 14555392 0.02835 3.329e-12 35366 TRUE reported textfile
2 1 TRUE
165 rs800454 C T C T 0.3285 0.0109 0.1003 NA FALSE FALSE FALSE
GCST006061 0.012 0.3616 537409 outcome TRUE reported 5660_51_SOD3_SOD3
SOD3 chr4 24820354 0.014397 3.038e-115 35353 TRUE reported

textfile 2 1 TRUE
166 rs1001652 C T C T -0.0594 -0.0011 0.20649 NA FALSE FALSE FALSE
GCST006061 0.0086 0.9023 537409 outcome TRUE reported 11441_11_PYGL_PYGL
PYGL chr12 7223650 0.009765 1.181e-09 35379 TRUE reported textfile
2 1 TRUE
167 rs17720606 A C A C -0.0635 -0.0079 0.28657 NA FALSE FALSE FALSE
GCST006061 0.008 0.3226 537409 outcome TRUE reported 11441_11_PYGL_PYGL
PYGL chr14 50864597 0.00874 3.71e-13 35358 TRUE reported textfile
2 1 TRUE
168 rs3748136 A G A G -0.1396 -0.0088 0.17278 NA FALSE FALSE FALSE
GCST006061 0.0093 0.3444 537409 outcome TRUE reported 11441_11_PYGL_PYGL
PYGL chr8 9172650 0.010377 2.989e-41 35375 TRUE reported textfile
2 1 TRUE
169 rs4759076 C T C T 0.0468 -0.0011 0.4756 NA FALSE FALSE FALSE
GCST006061 0.0073 0.8799 537409 outcome TRUE reported 11441_11_PYGL_PYGL
PYGL chr12 54336088 0.007891 3.017e-09 35363 TRUE reported
textfile 2 1 TRUE
170 rs73284790 A G A G -0.1914 -6.00E-04 0.08333 NA FALSE FALSE FALSE
GCST006061 0.0124 0.964 537409 outcome TRUE reported 11441_11_PYGL_PYGL
PYGL chr14 50902292 0.014063 3.501e-42 35361 TRUE reported
textfile 2 1 TRUE
171 rs79419800 G A G A -0.148 0.0053 0.02513 NA FALSE FALSE FALSE
GCST006061 0.0214 0.8048 537409 outcome TRUE reported 11441_11_PYGL_PYGL
PYGL chr14 42265331 0.024832 2.522e-09 35353 TRUE reported
textfile 2 1 TRUE
172 rs10019833 C T C T 0.0444 0.02 0.40021 NA FALSE FALSE FALSE
GCST006061 0.0075 0.007312 537409 outcome TRUE reported 2687_2_MIA_MIA MIA
chr4 76436439 0.008065 3.682e-08 35356 TRUE reported textfile 2
1 TRUE
173 rs111613270 T C T C 1.2361 0.0739 0.01242 NA FALSE FALSE FALSE
GCST006061 0.0413 0.07371 537409 outcome TRUE reported 2687_2_MIA_MIA MIA
chr19 40574767 0.037663 1e-200 35373 TRUE reported textfile 2 1
TRUE
174 rs117030537 T C T C 0.3589 -0.0757 0.01304 NA FALSE FALSE FALSE
GCST006061 0.0482 0.1158 537409 outcome TRUE reported 2687_2_MIA_MIA MIA
chr19 40590210 0.036035 2.283e-23 35374 TRUE reported textfile 2
1 TRUE
175 rs117613652 G A G A 0.2611 0.0066 0.01577 NA FALSE FALSE FALSE
GCST006061 0.0394 0.8675 537409 outcome TRUE reported 2687_2_MIA_MIA MIA
chr19 41738819 0.033291 4.398e-15 35375 TRUE reported textfile 2
1 TRUE
176 rs184589612 C T C T 0.893 0.0049 0.02063 NA FALSE FALSE FALSE
GCST006061 0.0341 0.8863 537409 outcome TRUE reported 2687_2_MIA_MIA MIA
chr19 40906287 0.028912 1e-200 35375 TRUE reported textfile 2 1
TRUE
177 rs191048002 C T C T 0.1833 -0.1571 0.02037 NA FALSE FALSE FALSE
GCST006061 0.0636 0.01347 537409 outcome TRUE reported 2687_2_MIA_MIA MIA
chr19 33337433 0.029541 5.471e-10 35368 TRUE reported textfile 2
1 TRUE
178 rs34262842 G A G A -0.0649 0.012 0.18967 NA FALSE FALSE FALSE
GCST006061 0.0101 0.233 537409 outcome TRUE reported 2687_2_MIA_MIA MIA
chr16 20344489 0.010185 1.865e-10 35357 TRUE reported textfile 2
1 TRUE
179 rs60530219 G A G A 0.1271 0.0186 0.04942 NA FALSE FALSE FALSE
GCST006061 0.0164 0.2566 537409 outcome TRUE reported 2687_2_MIA_MIA MIA
chr19 38507228 0.019367 5.28e-11 35375 TRUE reported textfile 2
1 TRUE
180 rs74746550 C T C T 0.8936 -0.0065 0.03467 NA FALSE FALSE FALSE
GCST006061 0.0151 0.669 537409 outcome TRUE reported 2687_2_MIA_MIA MIA
chr19 40709027 0.016773 1e-200 35374 TRUE reported textfile 2 1
TRUE
181 rs77542162 G A G A 0.2181 0.0177 0.025 NA FALSE FALSE FALSE
GCST006061 0.0302 0.557 537409 outcome TRUE reported 2687_2_MIA_MIA MIA
chr17 69085137 0.024914 2.056e-18 35371 TRUE reported textfile 2
1 TRUE
182 rs10031141 G A G A 0.4927 -0.0125 0.41213 NA FALSE FALSE FALSE

GCST006061 0.0075 0.0979 537409 outcome TRUE reported 3495_15_CXCL6_GCP_2
CXCL6 chr4 73728326 0.006009 1e-200 35350 TRUE reported textfile
2 1 TRUE
183 rs116119339 A G A G 0.341 -0.0327 0.01188 NA FALSE FALSE FALSE
GCST006061 0.0421 0.4373 537409 outcome TRUE reported 3495_15_CXCL6_GCP_2
CXCL6 chr4 71643740 0.037466 8.901e-20 35352 TRUE reported
textfile 2 1 TRUE
184 rs116608301 T C T C -0.1751 0.0173 0.03591 NA FALSE FALSE FALSE
GCST006061 0.0237 0.4649 537409 outcome TRUE reported 3495_15_CXCL6_GCP_2
CXCL6 chr4 59546223 0.021941 1.457e-15 35354 TRUE reported
textfile 2 1 TRUE
185 rs653178 T C T C -0.0616 0.0076 0.38124 NA FALSE FALSE FALSE
GCST006061 0.0075 0.3129 537409 outcome TRUE reported 3495_15_CXCL6_GCP_2
CXCL6 chr12 111569952 0.008036 1.783e-14 35371 TRUE reported
textfile 2 1 TRUE
186 rs72647456 T C T C -0.2159 -0.0415 0.02486 NA FALSE FALSE FALSE
GCST006061 0.0276 0.1318 537409 outcome TRUE reported 3495_15_CXCL6_GCP_2
CXCL6 chr4 75540247 0.025733 4.86e-17 35352 TRUE reported
textfile 2 1 TRUE
187 rs72653982 A G A G -0.1156 -0.006 0.06904 NA FALSE FALSE FALSE
GCST006061 0.0237 0.8017 537409 outcome TRUE reported 3495_15_CXCL6_GCP_2
CXCL6 chr4 72289631 0.016104 7.061e-13 35350 TRUE reported
textfile 2 1 TRUE
188 rs77858271 A G A G -0.2662 0.0142 0.01948 NA FALSE FALSE FALSE
GCST006061 0.0407 0.7272 537409 outcome TRUE reported 3495_15_CXCL6_GCP_2
CXCL6 chr4 74822692 0.028932 3.545e-20 35345 TRUE reported
textfile 2 1 TRUE
189 rs79190329 C A C A 0.1575 7e-04 0.1362 NA FALSE FALSE FALSE
GCST006061 0.0131 0.9576 537409 outcome TRUE reported 3495_15_CXCL6_GCP_2
CXCL6 chr4 72185275 0.01199 2.042e-39 35353 TRUE reported textfile
2 1 TRUE
190 rs9494145 C T C T 0.0575 -0.0073 0.24334 NA FALSE FALSE FALSE
GCST006061 0.0083 0.3799 537409 outcome TRUE reported 3495_15_CXCL6_GCP_2
CXCL6 chr6 135111414 0.009092 2.548e-10 35372 TRUE reported
textfile 2 1 TRUE
191 rs10517405 T C T C 0.1532 0.0032 0.02885 NA FALSE FALSE FALSE
GCST006061 0.0185 0.8636 537409 outcome TRUE reported 17755_5_UGDH_UGDH
UGDH chr4 37258081 0.024245 2.634e-10 35363 TRUE reported
textfile 2 1 TRUE
192 rs1354034 C T C T 0.0811 0.0088 0.38119 NA FALSE FALSE FALSE
GCST006061 0.0073 0.2248 537409 outcome TRUE reported 17755_5_UGDH_UGDH
UGDH chr3 56815721 0.008428 6.384e-22 35356 TRUE reported
textfile 2 1 TRUE
193 rs4734879 G A G A -0.066 0.0194 0.21866 NA FALSE FALSE FALSE
GCST006061 0.0079 0.01412 537409 outcome TRUE reported 17755_5_UGDH_UGDH
UGDH chr8 105570896 0.009941 3.151e-11 35365 TRUE reported
textfile 2 1 TRUE
194 rs7667751 T C T C 0.273 0.0145 0.37142 NA FALSE FALSE FALSE
GCST006061 0.0079 0.06668 537409 outcome TRUE reported 17755_5_UGDH_UGDH
UGDH chr4 39502649 0.008431 1e-200 35364 TRUE reported textfile
2 1 TRUE
195 rs10025052 T C T C -0.064 0.0022 0.39135 NA FALSE FALSE FALSE
GCST006061 0.0076 0.7664 537409 outcome TRUE reported
6342_10_NPNT_Nephronectin NPNT chr4 106040689 0.008186 5.339e-15 35350
TRUE reported textfile 2 1 TRUE
196 rs1250258 T C T C 0.1856 0.0164 0.32665 NA FALSE FALSE FALSE
GCST006061 0.0083 0.04845 537409 outcome TRUE reported
6342_10_NPNT_Nephronectin NPNT chr2 215435462 0.008448 5.553e-107 35355
TRUE reported textfile 2 1 TRUE
197 rs34712979 A G A G -0.1964 0.0048 0.24399 NA FALSE FALSE FALSE
GCST006061 0.0095 0.6133 537409 outcome TRUE reported
6342_10_NPNT_Nephronectin NPNT chr4 105897896 0.009233 2.055e-100 35350
TRUE reported textfile 2 1 TRUE
198 rs10026650 C T C T -0.1065 0.0013 0.44014 NA FALSE FALSE FALSE
GCST006061 0.0072 0.8576 537409 outcome TRUE reported 15385_116_FABP2_FABP2
FABP2 chr4 117736014 0.007709 2.044e-43 35351 TRUE reported

textfile 2 1 TRUE
199 rs116089146 T G T G 0.1384 -0.017 0.02477 NA FALSE FALSE FALSE
GCST006061 0.0259 0.5124 537409 outcome TRUE reported 15385_116_FABP2_FABP2
FABP2 chr4 146607421 0.025077 3.411e-08 35362 TRUE reported
textfile 2 1 TRUE
200 rs12502424 A G A G 0.2271 0.0137 0.32334 NA FALSE FALSE FALSE
GCST006061 0.0074 0.06562 537409 outcome TRUE reported 15385_116_FABP2_FABP2
FABP2 chr4 119945301 0.007865 2.546e-183 35351 TRUE reported
textfile 2 1 TRUE
201 rs281379 A G A G -0.0445 0.0023 0.37357 NA FALSE FALSE FALSE
GCST006061 0.0076 0.766 537409 outcome TRUE reported 15385_116_FABP2_FABP2
FABP2 chr19 48711017 0.007535 3.517e-09 35378 TRUE reported
textfile 2 1 TRUE
202 rs58289215 G T G T 0.0548 -0.0067 0.1897 NA FALSE FALSE FALSE
GCST006061 0.0093 0.4701 537409 outcome TRUE reported 15385_116_FABP2_FABP2
FABP2 chr4 108391041 0.009777 2.08e-08 35354 TRUE reported
textfile 2 1 TRUE
203 rs76350290 G A G A -0.2297 -8.00E-04 0.09274 NA FALSE FALSE FALSE
GCST006061 0.0148 0.9592 537409 outcome TRUE reported 15385_116_FABP2_FABP2
FABP2 chr4 120516218 0.013218 1.205e-67 35361 TRUE reported
textfile 2 1 TRUE
204 rs77657103 A G A G 0.1273 0.002 0.13667 NA FALSE FALSE FALSE
GCST006061 0.0108 0.8527 537409 outcome TRUE reported 15385_116_FABP2_FABP2
FABP2 chr4 120833497 0.011162 3.952e-30 35362 TRUE reported
textfile 2 1 TRUE
205 rs79551293 C T C T -0.1119 -0.003 0.09409 NA FALSE FALSE FALSE
GCST006061 0.0158 0.8503 537409 outcome TRUE reported 15385_116_FABP2_FABP2
FABP2 chr4 123664238 0.01306 1.051e-17 35351 TRUE reported textfile
2 1 TRUE
206 rs10029917 T C T C 0.0543 -0.0018 0.27408 NA FALSE FALSE FALSE
GCST006061 0.0082 0.8284 537409 outcome TRUE reported 15558_63_ENPEP_AMPE
ENPEP chr4 113025527 0.009292 5.107e-09 35355 TRUE reported
textfile 2 1 TRUE
207 rs114702284 A G A G -0.1633 -0.0311 0.04207 NA FALSE FALSE FALSE
GCST006061 0.0225 0.1663 537409 outcome TRUE reported 15558_63_ENPEP_AMPE
ENPEP chr4 111983924 0.020736 3.398e-15 35358 TRUE reported
textfile 2 1 TRUE
208 rs144496825 G T G T 0.3284 0.0434 0.02315 NA FALSE FALSE FALSE
GCST006061 0.0346 0.2093 537409 outcome TRUE reported 15558_63_ENPEP_AMPE
ENPEP chr4 111394075 0.02739 4.03e-33 35359 TRUE reported textfile
2 1 TRUE
209 rs6833731 A G A G 0.287 -0.0303 0.28437 NA FALSE FALSE FALSE
GCST006061 0.0078 9.507e-05 537409 outcome TRUE reported 15558_63_ENPEP_AMPE
ENPEP chr4 110489326 0.009076 1e-200 35355 TRUE reported textfile
2 1 TRUE
210 rs76716443 C A C A 0.2359 -0.0165 0.02264 NA FALSE FALSE FALSE
GCST006061 0.033 0.6178 537409 outcome TRUE reported 15558_63_ENPEP_AMPE
ENPEP chr4 111092586 0.027887 2.693e-17 35354 TRUE reported
textfile 2 1 TRUE
211 rs79797272 A G A G -0.151 0.0641 0.07082 NA FALSE FALSE FALSE
GCST006061 0.0223 0.004067 537409 outcome TRUE reported 15558_63_ENPEP_AMPE
ENPEP chr4 111470329 0.016123 7.582e-21 35359 TRUE reported
textfile 2 1 TRUE
212 rs80008037 A G A G 0.1453 0.0107 0.07967 NA FALSE FALSE FALSE
GCST006061 0.0137 0.4347 537409 outcome TRUE reported 15558_63_ENPEP_AMPE
ENPEP chr4 109608797 0.015318 2.416e-21 35361 TRUE reported
textfile 2 1 TRUE
213 rs10031270 G T G T 0.447 0.0172 0.19216 NA FALSE FALSE FALSE
GCST006061 0.0102 0.09262 537409 outcome TRUE reported 8402_22_CYTL1_CYTL1
CYTL1 chr4 5015781 0.008707 1e-200 35344 TRUE reported textfile 2
1 TRUE
214 rs151316626 A G A G -0.2829 0.0257 0.01782 NA FALSE FALSE FALSE
GCST006061 0.0455 0.5726 537409 outcome TRUE reported 8402_22_CYTL1_CYTL1
CYTL1 chr4 4508475 0.032088 1.181e-18 35341 TRUE reported textfile
2 1 TRUE
215 rs56724509 G T G T -0.1827 -0.0108 0.06091 NA FALSE FALSE FALSE

GCST006061 0.0141 0.4446 537409 outcome TRUE reported 8402_22_CYTL1_CYTL1
CYTL1 chr4 4776529 0.017455 1.226e-25 35342 TRUE reported textfile
2 1 TRUE
216 rs77924615 A G A G -0.0796 0.0136 0.19701 NA FALSE FALSE FALSE
GCST006061 0.0092 0.1375 537409 outcome TRUE reported 8402_22_CYTL1_CYTL1
CYTL1 chr16 20381010 0.01037 1.638e-14 35355 TRUE reported textfile
2 1 TRUE
217 rs10032216 C T C T -0.0548 -0.0187 0.21066 NA FALSE FALSE FALSE
GCST006061 0.009 0.03829 537409 outcome TRUE reported
18839_24_TG_Thyroglobulin TG chr4 148748354 0.009783 2.125e-08 35370
TRUE reported textfile 2 1 TRUE
218 rs116909374 T C T C -0.1234 0.0141 0.04906 NA FALSE FALSE FALSE
GCST006061 0.023 0.539 537409 outcome TRUE reported
18839_24_TG_Thyroglobulin TG chr14 36269155 0.018212 1.239e-11 35355
TRUE reported textfile 2 1 TRUE
219 rs3872724 T C T C -0.1374 0.0014 0.40614 NA FALSE FALSE FALSE
GCST006061 0.0074 0.8498 537409 outcome TRUE reported
18839_24_TG_Thyroglobulin TG chr3 187023433 0.008113 2.413e-64 35363
TRUE reported textfile 2 1 TRUE
220 rs9295128 T G T G 0.2472 -0.0818 0.01256 NA FALSE FALSE FALSE
GCST006061 0.0355 0.02112 537409 outcome TRUE reported
18839_24_TG_Thyroglobulin TG chr6 160330499 0.035179 2.112e-12 35390
TRUE reported textfile 2 1 TRUE
221 rs1354034 C T C T 0.0592 0.0088 0.38119 NA FALSE FALSE FALSE
GCST006061 0.0073 0.2248 537409 outcome TRUE reported 9482_110_NUDT9_NUDT9
NUDT9 chr3 56815721 0.008337 1.242e-12 35356 TRUE reported
textfile 2 1 TRUE
222 rs149062332 C T C T 0.2266 0.0424 0.02045 NA FALSE FALSE FALSE
GCST006061 0.0435 0.33 537409 outcome TRUE reported 9482_110_NUDT9_NUDT9
NUDT9 chr3 186623236 0.027886 4.44e-16 35367 TRUE reported
textfile 2 1 TRUE
223 rs28529046 T C T C -0.1379 0.007 0.16738 NA FALSE FALSE FALSE
GCST006061 0.0102 0.49 537409 outcome TRUE reported 9482_110_NUDT9_NUDT9
NUDT9 chr4 87440564 0.010909 1.247e-36 35364 TRUE reported
textfile 2 1 TRUE
224 rs8178824 T C T C 0.3059 -0.0854 0.01066 NA FALSE FALSE FALSE
GCST006061 0.023 0.0002035 537409 outcome TRUE reported
9482_110_NUDT9_NUDT9 NUDT9 chr17 66228657 0.039149 5.556e-15 35369
TRUE reported textfile 2 1 TRUE
225 rs10071051 A G A G -0.0683 -0.0082 0.31444 NA FALSE FALSE FALSE
GCST006061 0.0086 0.3389 537409 outcome TRUE reported 3196_6_HAPLN1_HPLN1
HAPLN1 chr5 132416928 0.008664 3.194e-15 35352 TRUE reported
textfile 2 1 TRUE
226 rs10922098 T C T C -0.0554 -0.009 0.40011 NA FALSE FALSE FALSE
GCST006061 0.0075 0.2275 537409 outcome TRUE reported 3196_6_HAPLN1_HPLN1
HAPLN1 chr1 196695521 0.008213 1.527e-11 35338 TRUE reported
textfile 2 1 TRUE
227 rs173053 C T C T 0.0642 -0.0063 0.38645 NA FALSE FALSE FALSE
GCST006061 0.0075 0.4009 537409 outcome TRUE reported 3196_6_HAPLN1_HPLN1
HAPLN1 chr5 83670235 0.008318 1.183e-14 35340 TRUE reported
textfile 2 1 TRUE
228 rs191703636 C T C T -0.247 0.0119 0.03111 NA FALSE FALSE FALSE
GCST006061 0.0332 0.7196 537409 outcome TRUE reported 3196_6_HAPLN1_HPLN1
HAPLN1 chr5 83609723 0.023635 1.454e-25 35342 TRUE reported
textfile 2 1 TRUE
229 rs224916 G A G A 0.0519 -0.0047 0.2588 NA FALSE FALSE FALSE
GCST006061 0.0081 0.5645 537409 outcome TRUE reported 3196_6_HAPLN1_HPLN1
HAPLN1 chr5 82426928 0.009173 1.533e-08 35340 TRUE reported
textfile 2 1 TRUE
230 rs2544663 C T C T 0.0513 -0.0042 0.38724 NA FALSE FALSE FALSE
GCST006061 0.0074 0.5731 537409 outcome TRUE reported 3196_6_HAPLN1_HPLN1
HAPLN1 chr5 87125654 0.008252 5.072e-10 35343 TRUE reported
textfile 2 1 TRUE
231 rs10476544 T C T C 0.1162 -0.0264 0.09215 NA FALSE FALSE FALSE
GCST006061 0.0147 0.07274 537409 outcome TRUE reported 13388_57_PCSK1_NEC1
PCSK1 chr5 95850229 0.014071 1.477e-16 35343 TRUE reported

textfile 2 1 TRUE

232 rs27373 T C T C -0.2698 -9.00E-04 0.04944 NA FALSE FALSE FALSE GCST006061 0.0175 0.9585 537409 outcome TRUE reported 13388_57_PCSK1_NEC1 PCSK1 chr5 96771318 0.019053 1.611e-45 35343 TRUE reported textfile 2 1 TRUE

233 rs6232 C T C T -0.6235 -0.0118 0.08679 NA FALSE FALSE FALSE GCST006061 0.0176 0.5023 537409 outcome TRUE reported 13388_57_PCSK1_NEC1 PCSK1 chr5 96416081 0.010783 1e-200 35342 TRUE reported textfile 2 1 TRUE

234 rs6872684 T C T C -0.0687 -0.0144 0.12818 NA FALSE FALSE FALSE GCST006061 0.0104 0.1664 537409 outcome TRUE reported 13388_57_PCSK1_NEC1 PCSK1 chr5 81387048 0.012292 2.282e-08 35333 TRUE reported textfile 2 1 TRUE

235 rs72772040 C T C T 0.2321 0.0031 0.06384 NA FALSE FALSE FALSE GCST006061 0.0151 0.8384 537409 outcome TRUE reported 13388_57_PCSK1_NEC1 PCSK1 chr5 96617136 0.016803 2.126e-43 35343 TRUE reported textfile 2 1 TRUE

236 rs76239650 A C A C -0.4909 0.0029 0.10806 NA FALSE FALSE FALSE GCST006061 0.0132 0.8266 537409 outcome TRUE reported 13388_57_PCSK1_NEC1 PCSK1 chr5 96022520 0.009712 1e-200 35342 TRUE reported textfile 2 1 TRUE

237 rs10040716 C A C A 0.0523 -0.0234 0.32942 NA FALSE FALSE FALSE GCST006061 0.0075 0.001891 537409 outcome TRUE reported 17447_52_SFRP4_SFRP4 SFRP4 chr5 109864333 0.008897 4.145e-09 35351 TRUE reported textfile 2 1 TRUE

238 rs1052981 A G A G 0.1241 0.0025 0.25366 NA FALSE FALSE FALSE GCST006061 0.0081 0.761 537409 outcome TRUE reported 17447_52_SFRP4_SFRP4 SFRP4 chr7 37906899 0.009614 4.063e-38 35361 TRUE reported textfile 2 1 TRUE

239 rs215223 A G A G 0.0992 -0.0193 0.41963 NA FALSE FALSE FALSE GCST006061 0.0073 0.00813 537409 outcome TRUE reported 17447_52_SFRP4_SFRP4 SFRP4 chr12 481093 0.008467 1.056e-31 35311 TRUE reported textfile 2 1 TRUE

240 rs2598105 C T C T -0.2693 -0.0019 0.15511 NA FALSE FALSE FALSE GCST006061 0.0098 0.8423 537409 outcome TRUE reported 17447_52_SFRP4_SFRP4 SFRP4 chr7 37937514 0.011389 1.293e-123 35361 TRUE reported textfile 2 1 TRUE

241 rs35887873 C T C T 0.1491 -0.0114 0.26311 NA FALSE FALSE FALSE GCST006061 0.0091 0.2089 537409 outcome TRUE reported 17447_52_SFRP4_SFRP4 SFRP4 chr11 126349501 0.009391 9.156e-57 35374 TRUE reported textfile 2 1 TRUE

242 rs763665 T C T C 0.0656 0.0096 0.18297 NA FALSE FALSE FALSE GCST006061 0.01 0.3355 537409 outcome TRUE reported 17447_52_SFRP4_SFRP4 SFRP4 chr16 72044144 0.010679 8.089e-10 35375 TRUE reported textfile 2 1 TRUE

243 rs780094 C T C T 0.05 -0.0076 0.33917 NA FALSE FALSE FALSE GCST006061 0.0073 0.2951 537409 outcome TRUE reported 17447_52_SFRP4_SFRP4 SFRP4 chr2 27518370 0.008824 1.456e-08 35373 TRUE reported textfile 2 1 TRUE

244 rs10045921 A G A G -0.0505 0.0053 0.39281 NA FALSE FALSE FALSE GCST006061 0.0073 0.4702 537409 outcome TRUE reported 7227_75_COCH_COCH COCH chr5 89453265 0.008623 4.737e-09 35342 TRUE reported textfile 2 1 TRUE

245 rs10822155 A C A C 0.0647 -0.0472 0.43479 NA FALSE FALSE FALSE GCST006061 0.0072 4.622e-11 537409 outcome TRUE reported 7227_75_COCH_COCH COCH chr10 63311455 0.008477 2.302e-14 35351 TRUE reported textfile 2 1 TRUE

246 rs11083114 T C T C -0.0552 2e-04 0.25877 NA FALSE FALSE FALSE GCST006061 0.0077 0.9783 537409 outcome TRUE reported 7227_75_COCH_COCH COCH chr18 25272750 0.009634 1.005e-08 35368 TRUE reported textfile 2 1 TRUE

247 rs11154799 T C T C 0.0495 -0.0128 0.45988 NA FALSE FALSE FALSE GCST006061 0.0073 0.07819 537409 outcome TRUE reported 7227_75_COCH_COCH COCH chr6 135311511 0.008464 4.963e-09 35364 TRUE reported textfile 2 1 TRUE

248 rs11171914 C T C T -0.0605 -0.0437 0.31333 NA FALSE FALSE FALSE GCST006061 0.0075 6.028e-09 537409 outcome TRUE reported 7227_75_COCH_COCH

COCH chr12 56649462 0.009149 3.774e-11 35369 TRUE reported
textfile 2 1 TRUE
249 rs114694170 C T C T -0.1974 0.0053 0.08378 NA FALSE FALSE FALSE
GCST006061 0.0175 0.7624 537409 outcome TRUE reported 7227_75_COCH_COCH
COCH chr5 88884379 0.015256 2.697e-38 35344 TRUE reported
textfile 2 1 TRUE
250 rs11708890 C T C T -0.0486 0.0091 0.4683 NA FALSE FALSE FALSE
GCST006061 0.0073 0.2172 537409 outcome TRUE reported 7227_75_COCH_COCH
COCH chr3 179057947 0.008454 8.973e-09 35354 TRUE reported
textfile 2 1 TRUE
251 rs117309559 G A G A 0.4934 -0.0122 0.02722 NA FALSE FALSE FALSE
GCST006061 0.0251 0.6276 537409 outcome TRUE reported 7227_75_COCH_COCH
COCH chr14 30561098 0.026153 2.182e-79 35362 TRUE reported
textfile 2 1 TRUE
252 rs12825574 T C T C 0.0543 0.0018 0.41502 NA FALSE FALSE FALSE
GCST006061 0.0075 0.813 537409 outcome TRUE reported 7227_75_COCH_COCH
COCH chr12 8021806 0.008614 2.9e-10 35381 TRUE reported textfile 2
1 TRUE
253 rs142183746 A C A C 0.1619 0.001 0.03748 NA FALSE FALSE FALSE
GCST006061 0.0325 0.9745 537409 outcome TRUE reported 7227_75_COCH_COCH
COCH chr14 31471737 0.021931 1.555e-13 35358 TRUE reported
textfile 2 1 TRUE
254 rs1434282 T C T C 0.0982 -0.0021 0.26524 NA FALSE FALSE FALSE
GCST006061 0.008 0.7916 537409 outcome TRUE reported 7227_75_COCH_COCH
COCH chr1 199041592 0.009638 2.228e-24 35343 TRUE reported
textfile 2 1 TRUE
255 rs154513 T C T C -0.0472 0.0056 0.49159 NA FALSE FALSE FALSE
GCST006061 0.0071 0.4326 537409 outcome TRUE reported 7227_75_COCH_COCH
COCH chr5 122858030 0.008434 2.192e-08 35354 TRUE reported
textfile 2 1 TRUE
256 rs1769758 T G T G 0.0667 -0.031 0.45567 NA FALSE FALSE FALSE
GCST006061 0.0082 0.0001497 537409 outcome TRUE reported 7227_75_COCH_COCH
COCH chr10 79139212 0.008454 3.036e-15 35363 TRUE reported
textfile 2 1 TRUE
257 rs342299 T C T C 0.0802 1e-04 0.45394 NA FALSE FALSE FALSE
GCST006061 0.0074 0.9852 537409 outcome TRUE reported 7227_75_COCH_COCH
COCH chr7 106733272 0.008414 1.541e-21 35354 TRUE reported
textfile 2 1 TRUE
258 rs35140383 C T C T -0.219 0.0133 0.01155 NA FALSE FALSE FALSE
GCST006061 0.0296 0.6519 537409 outcome TRUE reported 7227_75_COCH_COCH
COCH chr9 132646885 0.039907 4.071e-08 35373 TRUE reported
textfile 2 1 TRUE
259 rs3759217 T C T C 0.0795 -0.0219 0.09927 NA FALSE FALSE FALSE
GCST006061 0.0113 0.05339 537409 outcome TRUE reported 7227_75_COCH_COCH
COCH chr12 12715518 0.01417 2.017e-08 35371 TRUE reported textfile
2 1 TRUE
260 rs432002 C T C T 0.0622 0.0042 0.18348 NA FALSE FALSE FALSE
GCST006061 0.01 0.6768 537409 outcome TRUE reported 7227_75_COCH_COCH
COCH chr7 102043205 0.010878 1.077e-08 35366 TRUE reported
textfile 2 1 TRUE
261 rs4414033 A G A G 0.0555 -0.0207 0.39306 NA FALSE FALSE FALSE
GCST006061 0.0075 0.005726 537409 outcome TRUE reported 7227_75_COCH_COCH
COCH chr1 156437061 0.008614 1.169e-10 35358 TRUE reported
textfile 2 1 TRUE
262 rs4671605 T C T C -0.0659 -0.0027 0.27763 NA FALSE FALSE FALSE
GCST006061 0.0083 0.7416 537409 outcome TRUE reported 7227_75_COCH_COCH
COCH chr2 64660248 0.0094 2.374e-12 35349 TRUE reported textfile
2 1 TRUE
263 rs4759076 C T C T 0.1288 -0.0011 0.4756 NA FALSE FALSE FALSE
GCST006061 0.0073 0.8799 537409 outcome TRUE reported 7227_75_COCH_COCH
COCH chr12 54336088 0.008429 1.036e-52 35372 TRUE reported
textfile 2 1 TRUE
264 rs60377712 C A C A 0.061 0.0018 0.27928 NA FALSE FALSE FALSE
GCST006061 0.0079 0.8241 537409 outcome TRUE reported 7227_75_COCH_COCH
COCH chr19 57861683 0.009364 7.295e-11 35164 TRUE reported
textfile 2 1 TRUE

265 rs61269268 C A C A -0.0832 -0.002 0.20448 NA FALSE FALSE FALSE
GCST006061 0.0098 0.835 537409 outcome TRUE reported 7227_75_COCH_COCH
COCH chr14 91775008 0.010556 3.229e-15 35360 TRUE reported
textfile 2 1 TRUE
266 rs690686 T C T C 0.165 -8.00E-04 0.05901 NA FALSE FALSE FALSE
GCST006061 0.013 0.9534 537409 outcome TRUE reported 7227_75_COCH_COCH
COCH chr7 129571648 0.017707 1.181e-20 35354 TRUE reported
textfile 2 1 TRUE
267 rs7159420 G T G T 0.4286 -0.0048 0.1198 NA FALSE FALSE FALSE
GCST006061 0.0111 0.6668 537409 outcome TRUE reported 7227_75_COCH_COCH
COCH chr14 30845149 0.012891 1e-200 35362 TRUE reported textfile
2 1 TRUE
268 rs73000929 A G A G -0.2059 0.0131 0.04815 NA FALSE FALSE FALSE
GCST006061 0.0245 0.593 537409 outcome TRUE reported 7227_75_COCH_COCH
COCH chr11 114082900 0.019606 8.478e-26 35365 TRUE reported
textfile 2 1 TRUE
269 rs7618405 A C A C -0.0562 -0.0082 0.21773 NA FALSE FALSE FALSE
GCST006061 0.009 0.3631 537409 outcome TRUE reported 7227_75_COCH_COCH
COCH chr3 18209017 0.010187 3.448e-08 35369 TRUE reported
textfile 2 1 TRUE
270 rs76539348 A G A G 0.1939 -0.06 0.01692 NA FALSE FALSE FALSE
GCST006061 0.0524 0.2523 537409 outcome TRUE reported 7227_75_COCH_COCH
COCH chr14 31599846 0.03325 5.49e-09 35357 TRUE reported textfile
2 1 TRUE
271 rs10046898 T C T C -0.2659 0.0161 0.41992 NA FALSE FALSE FALSE
GCST006061 0.0075 0.03112 537409 outcome TRUE reported 6451_64_ASPN_ASPN
ASPN chr9 92176794 0.008205 1e-200 35354 TRUE reported textfile
2 1 TRUE
272 rs143501783 C T C T 0.208 -0.0333 0.01116 NA FALSE FALSE FALSE
GCST006061 0.0443 0.4525 537409 outcome TRUE reported 6451_64_ASPN_ASPN
ASPN chr17 7897001 0.036549 1.263e-08 35373 TRUE reported textfile
2 1 TRUE
273 rs2073333 T C T C 0.1043 0.0103 0.20754 NA FALSE FALSE FALSE
GCST006061 0.0081 0.2059 537409 outcome TRUE reported 6451_64_ASPN_ASPN
ASPN chr14 94378225 0.009928 8.106e-26 35371 TRUE reported
textfile 2 1 TRUE
274 rs2229498 A G A G -0.0746 0.0047 0.16514 NA FALSE FALSE FALSE
GCST006061 0.0092 0.6113 537409 outcome TRUE reported 6451_64_ASPN_ASPN
ASPN chr10 69097096 0.010878 6.987e-12 35362 TRUE reported
textfile 2 1 TRUE
275 rs3199918 C T C T 0.0556 6e-04 0.20519 NA FALSE FALSE FALSE
GCST006061 0.0097 0.9503 537409 outcome TRUE reported 6451_64_ASPN_ASPN
ASPN chr3 52547419 0.009925 2.118e-08 35352 TRUE reported
textfile 2 1 TRUE
276 rs333947 A G A G -0.0756 -0.0071 0.14937 NA FALSE FALSE FALSE
GCST006061 0.01 0.4808 537409 outcome TRUE reported 6451_64_ASPN_ASPN
ASPN chr1 109928142 0.011356 2.792e-11 35349 TRUE reported
textfile 2 1 TRUE
277 rs35887873 C T C T 0.0993 -0.0114 0.26311 NA FALSE FALSE FALSE
GCST006061 0.0091 0.2089 537409 outcome TRUE reported 6451_64_ASPN_ASPN
ASPN chr11 126349501 0.00906 5.948e-28 35374 TRUE reported textfile
2 1 TRUE
278 rs55714927 T C T C 0.0935 1e-04 0.21166 NA FALSE FALSE FALSE
GCST006061 0.0098 0.9926 537409 outcome TRUE reported 6451_64_ASPN_ASPN
ASPN chr17 7176997 0.009894 3.374e-21 35370 TRUE reported textfile
2 1 TRUE
279 rs73505430 T C T C -0.1525 -0.0178 0.03003 NA FALSE FALSE FALSE
GCST006061 0.0198 0.3691 537409 outcome TRUE reported 6451_64_ASPN_ASPN
ASPN chr9 90602534 0.023571 9.813e-11 35358 TRUE reported
textfile 2 1 TRUE
280 rs10048885 G A G A -0.0582 0.0018 0.25188 NA FALSE FALSE FALSE
GCST006061 0.0086 0.8353 537409 outcome TRUE reported 2665_26_TNFRSF17_BCMA
TNFRSF17 chr22 30184263 0.00961 1.396e-09 35366 TRUE reported
textfile 2 1 TRUE
281 rs1140809 A C A C -0.0621 -0.0082 0.45749 NA FALSE FALSE FALSE
GCST006061 0.0078 0.2959 537409 outcome TRUE reported 2665_26_TNFRSF17_BCMA

TNFRSF17 chr6 30643899 0.008389 1.334e-13 35442 TRUE reported
textfile 2 1 TRUE
282 rs12758915 G A G A -0.0503 9e-04 0.48594 NA FALSE FALSE FALSE
GCST006061 0.0072 0.8975 537409 outcome TRUE reported 2665_26_TNFRSF17_BCMA
TNFRSF17 chr1 226870643 0.008396 2.084e-09 35358 TRUE reported
textfile 2 1 TRUE
283 rs1418708 A G A G 0.0748 0.004 0.12213 NA FALSE FALSE FALSE
GCST006061 0.0104 0.7031 537409 outcome TRUE reported 2665_26_TNFRSF17_BCMA
TNFRSF17 chr6 205610 0.012841 5.712e-09 35316 TRUE reported
textfile 2 1 TRUE
284 rs17334923 T C T C -0.0602 -0.0391 0.18787 NA FALSE FALSE FALSE
GCST006061 0.0094 3.273e-05 537409 outcome TRUE reported
2665_26_TNFRSF17_BCMA TNFRSF17 chr17 45749878 0.010774 2.305e-08 35363
TRUE reported textfile 2 1 TRUE
285 rs2532744 G T G T -0.0764 -0.0168 0.28804 NA FALSE FALSE FALSE
GCST006061 0.0079 0.03298 537409 outcome TRUE reported 2665_26_TNFRSF17_BCMA
TNFRSF17 chr10 28613689 0.00926 1.582e-16 35351 TRUE reported
textfile 2 1 TRUE
286 rs34562254 A G A G 0.2207 -0.0013 0.07695 NA FALSE FALSE FALSE
GCST006061 0.0109 0.902 537409 outcome TRUE reported 2665_26_TNFRSF17_BCMA
TNFRSF17 chr17 16939677 0.015664 4.395e-45 35357 TRUE reported
textfile 2 1 TRUE
287 rs4690163 C T C T -0.0744 -0.0051 0.33124 NA FALSE FALSE FALSE
GCST006061 0.0075 0.4943 537409 outcome TRUE reported 2665_26_TNFRSF17_BCMA
TNFRSF17 chr4 981349 0.008916 7.129e-17 35274 TRUE reported
textfile 2 1 TRUE
288 rs4851257 T C T C -0.0511 0.0028 0.3606 NA FALSE FALSE FALSE
GCST006061 0.0074 0.7053 537409 outcome TRUE reported 2665_26_TNFRSF17_BCMA
TNFRSF17 chr2 100158835 0.008697 4.208e-09 35360 TRUE reported
textfile 2 1 TRUE
289 rs510432 C T C T 0.0499 -0.0123 0.46309 NA FALSE FALSE FALSE
GCST006061 0.0071 0.08437 537409 outcome TRUE reported 2665_26_TNFRSF17_BCMA
TNFRSF17 chr6 106326155 0.008363 2.422e-09 35374 TRUE reported
textfile 2 1 TRUE
290 rs6728684 G T G T 0.0696 -0.0448 0.216 NA FALSE FALSE FALSE
GCST006061 0.0089 5.469e-07 537409 outcome TRUE reported
2665_26_TNFRSF17_BCMA TNFRSF17 chr2 25927904 0.010183 8.209e-12 35369
TRUE reported textfile 2 1 TRUE
291 rs78377363 C T C T 0.195 0.0218 0.0156 NA FALSE FALSE FALSE
GCST006061 0.0289 0.4502 537409 outcome TRUE reported 2665_26_TNFRSF17_BCMA
TNFRSF17 chr6 137804744 0.033913 8.922e-09 35362 TRUE reported
textfile 2 1 TRUE
292 rs894872 G T G T 0.0507 0.0083 0.37062 NA FALSE FALSE FALSE
GCST006061 0.0077 0.282 537409 outcome TRUE reported 2665_26_TNFRSF17_BCMA
TNFRSF17 chr17 79937175 0.00869 5.409e-09 35376 TRUE reported
textfile 2 1 TRUE
293 rs10051765 C T C T 0.0494 0.0044 0.39217 NA FALSE FALSE FALSE
GCST006061 0.0076 0.5641 537409 outcome TRUE reported 9312_8_AZGP1_AZGP1
AZGP1 chr5 177372991 0.008467 5.392e-09 35365 TRUE reported
textfile 2 1 TRUE
294 rs10922098 T C T C -0.0772 -0.009 0.40011 NA FALSE FALSE FALSE
GCST006061 0.0075 0.2275 537409 outcome TRUE reported 9312_8_AZGP1_AZGP1
AZGP1 chr1 196695521 0.008397 3.789e-20 35338 TRUE reported
textfile 2 1 TRUE
295 rs1128097 G A G A -0.0609 -0.0037 0.20775 NA FALSE FALSE FALSE
GCST006061 0.0109 0.733 537409 outcome TRUE reported 9312_8_AZGP1_AZGP1
AZGP1 chr7 99119190 0.010157 2.022e-09 35368 TRUE reported
textfile 2 1 TRUE
296 rs150844304 C A C A -0.2192 0.0439 0.0276 NA FALSE FALSE FALSE
GCST006061 0.0244 0.07203 537409 outcome TRUE reported 9312_8_AZGP1_AZGP1
AZGP1 chr15 43434427 0.024972 1.667e-18 35363 TRUE reported
textfile 2 1 TRUE
297 rs6535576 A G A G 0.0463 0.0163 0.4143 NA FALSE FALSE FALSE
GCST006061 0.0073 0.02527 537409 outcome TRUE reported 9312_8_AZGP1_AZGP1
AZGP1 chr4 148065653 0.00835 2.938e-08 35362 TRUE reported textfile
2 1 TRUE

298 rs72704449 C T C T 0.1588 0.013 0.04582 NA FALSE FALSE FALSE
GCST006061 0.0199 0.5128 537409 outcome TRUE reported 9312_8_AZGP1_AZGP1
AZGP1 chr1 179504138 0.019578 5.008e-16 35358 TRUE reported
textfile 2 1 TRUE
299 rs77924615 A G A G -0.0676 0.0136 0.19701 NA FALSE FALSE FALSE
GCST006061 0.0092 0.1375 537409 outcome TRUE reported 9312_8_AZGP1_AZGP1
AZGP1 chr16 20381010 0.010395 7.863e-11 35355 TRUE reported
textfile 2 1 TRUE
300 rs10051765 C T C T 0.0498 0.0044 0.39217 NA FALSE FALSE FALSE
GCST006061 0.0076 0.5641 537409 outcome TRUE reported 14131_37_EFNB2_EFNB2
EFNB2 chr5 177372991 0.008428 3.448e-09 35365 TRUE reported
textfile 2 1 TRUE
301 rs10253736 T C T C 0.054 -0.0035 0.26815 NA FALSE FALSE FALSE
GCST006061 0.0082 0.6665 537409 outcome TRUE reported 14131_37_EFNB2_EFNB2
EFNB2 chr7 151718170 0.009203 4.428e-09 35372 TRUE reported
textfile 2 1 TRUE
302 rs11568581 A G A G -0.098 -0.0393 0.05565 NA FALSE FALSE FALSE
GCST006061 0.0174 0.02416 537409 outcome TRUE reported 14131_37_EFNB2_EFNB2
EFNB2 chr17 50661130 0.017545 2.33e-08 35360 TRUE reported
textfile 2 1 TRUE
303 rs1260326 C T C T 0.0512 -0.0069 0.34132 NA FALSE FALSE FALSE
GCST006061 0.0073 0.3401 537409 outcome TRUE reported 14131_37_EFNB2_EFNB2
EFNB2 chr2 27508073 0.008669 3.507e-09 35373 TRUE reported
textfile 2 1 TRUE
304 rs13108218 G A G A 0.0487 -0.01 0.36204 NA FALSE FALSE FALSE
GCST006061 0.0074 0.1774 537409 outcome TRUE reported 14131_37_EFNB2_EFNB2
EFNB2 chr4 3442204 0.008505 1.027e-08 35280 TRUE reported textfile
2 1 TRUE
305 rs635634 C T C T 0.1867 0.0285 0.12995 NA FALSE FALSE FALSE
GCST006061 0.0089 0.001351 537409 outcome TRUE reported
14131_37_EFNB2_EFNB2 EFNB2 chr9 133279427 0.012157 3.176e-53 35371
TRUE reported textfile 2 1 TRUE
306 rs72632701 G T G T 0.0912 -0.0034 0.12726 NA FALSE FALSE FALSE
GCST006061 0.0098 0.7312 537409 outcome TRUE reported 14131_37_EFNB2_EFNB2
EFNB2 chr13 106643719 0.012174 6.802e-14 35358 TRUE reported
textfile 2 1 TRUE
307 rs72704449 C T C T 0.1398 0.013 0.04582 NA FALSE FALSE FALSE
GCST006061 0.0199 0.5128 537409 outcome TRUE reported 14131_37_EFNB2_EFNB2
EFNB2 chr1 179504138 0.019493 7.403e-13 35358 TRUE reported
textfile 2 1 TRUE
308 rs77924615 A G A G -0.0815 0.0136 0.19701 NA FALSE FALSE FALSE
GCST006061 0.0092 0.1375 537409 outcome TRUE reported 14131_37_EFNB2_EFNB2
EFNB2 chr16 20381010 0.01036 3.641e-15 35355 TRUE reported textfile
2 1 TRUE
309 rs10051765 C T C T -0.0854 0.0044 0.39217 NA FALSE FALSE FALSE
GCST006061 0.0076 0.5641 537409 outcome TRUE reported 5954_62_PTH_PTH PTH
chr5 177372991 0.008351 1.513e-24 35365 TRUE reported textfile 2
1 TRUE
310 rs219771 T C T C -0.0585 0.0053 0.24559 NA FALSE FALSE FALSE
GCST006061 0.0086 0.5374 537409 outcome TRUE reported 5954_62_PTH_PTH PTH
chr21 36463203 0.009416 5.214e-10 35384 TRUE reported textfile 2
1 TRUE
311 rs138605513 T C T C 0.2225 -0.0148 0.02349 NA FALSE FALSE FALSE
GCST006061 0.0246 0.548 537409 outcome TRUE reported 3283_21_TGFBI_BGH3
TGFBI chr5 135905791 0.026972 1.593e-16 35356 TRUE reported
textfile 2 1 TRUE
312 rs17750709 T C T C 0.1847 0.0253 0.03996 NA FALSE FALSE FALSE
GCST006061 0.023 0.2698 537409 outcome TRUE reported 3283_21_TGFBI_BGH3
TGFBI chr5 136230740 0.021043 1.678e-18 35356 TRUE reported
textfile 2 1 TRUE
313 rs2255531 A G A G 0.0513 -0.004 0.3496 NA FALSE FALSE FALSE
GCST006061 0.0074 0.5835 537409 outcome TRUE reported 3283_21_TGFBI_BGH3
TGFBI chr12 120977112 0.008457 1.312e-09 35365 TRUE reported
textfile 2 1 TRUE
314 rs6892697 A G A G -0.2622 -0.0099 0.39238 NA FALSE FALSE FALSE
GCST006061 0.0078 0.2041 537409 outcome TRUE reported 3283_21_TGFBI_BGH3

TGFBI chr5 136008474 0.00841 1e-200 35354 TRUE reported textfile 2 1 TRUE

315 rs699 G A G A 0.0955 -0.0081 0.42489 NA FALSE FALSE FALSE GCST006061 0.0073 0.2711 537409 outcome TRUE reported 3283_21_TGFBI_BGH3 TGFBI chr1 230710048 0.008183 1.792e-31 35364 TRUE reported textfile 2 1 TRUE

316 rs76221198 T G T G -0.1985 -0.0348 0.01585 NA FALSE FALSE FALSE GCST006061 0.0288 0.228 537409 outcome TRUE reported 3283_21_TGFBI_BGH3 TGFBI chr5 135789109 0.032618 1.161e-09 35355 TRUE reported textfile 2 1 TRUE

317 rs76926676 C T C T 0.1568 -0.014 0.02741 NA FALSE FALSE FALSE GCST006061 0.0247 0.5708 537409 outcome TRUE reported 3283_21_TGFBI_BGH3 TGFBI chr5 141805409 0.025392 6.606e-10 35371 TRUE reported textfile 2 1 TRUE

318 rs7810066 G A G A -0.0832 0.006 0.27436 NA FALSE FALSE FALSE GCST006061 0.0082 0.4625 537409 outcome TRUE reported 3283_21_TGFBI_BGH3 TGFBI chr7 77222006 0.009014 2.707e-20 35351 TRUE reported textfile 2 1 TRUE

319 rs10055076 A G A G -0.0666 -4.00E-04 0.14101 NA FALSE FALSE FALSE GCST006061 0.0106 0.9719 537409 outcome TRUE reported 6927_7_NDST1_NDST1 NDST1 chr5 61188260 0.012004 2.89e-08 35355 TRUE reported textfile 2 1 TRUE

320 rs140624338 C T C T 0.2693 0.0021 0.01047 NA FALSE FALSE FALSE GCST006061 0.0539 0.9695 537409 outcome TRUE reported 6927_7_NDST1_NDST1 NDST1 chr12 101963654 0.040811 4.149e-11 35362 TRUE reported textfile 2 1 TRUE

321 rs1560661 T C T C -0.0589 -0.0021 0.43381 NA FALSE FALSE FALSE GCST006061 0.0076 0.7831 537409 outcome TRUE reported 6927_7_NDST1_NDST1 NDST1 chr5 150413894 0.008387 2.17e-12 35353 TRUE reported textfile 2 1 TRUE

322 rs2272662 C T C T -0.0501 0.0109 0.35723 NA FALSE FALSE FALSE GCST006061 0.0083 0.1873 537409 outcome TRUE reported 6927_7_NDST1_NDST1 NDST1 chr8 144414342 0.008754 1.047e-08 34529 TRUE reported textfile 2 1 TRUE

323 rs635634 C T C T -0.1395 0.0285 0.12995 NA FALSE FALSE FALSE GCST006061 0.0089 0.001351 537409 outcome TRUE reported 6927_7_NDST1_NDST1 NDST1 chr9 133279427 0.01231 9.103e-30 35371 TRUE reported textfile 2 1 TRUE

324 rs6863373 T C T C 0.1343 -0.0132 0.44916 NA FALSE FALSE FALSE GCST006061 0.0074 0.07607 537409 outcome TRUE reported 6927_7_NDST1_NDST1 NDST1 chr5 150530369 0.008357 4.078e-58 35355 TRUE reported textfile 2 1 TRUE

325 rs77542162 G A G A 0.2454 0.0177 0.025 NA FALSE FALSE FALSE GCST006061 0.0302 0.557 537409 outcome TRUE reported 6927_7_NDST1_NDST1 NDST1 chr17 69085137 0.026076 4.925e-21 35371 TRUE reported textfile 2 1 TRUE

326 rs950419 G A G A 0.0835 0.0029 0.08025 NA FALSE FALSE FALSE GCST006061 0.0153 0.8477 537409 outcome TRUE reported 6927_7_NDST1_NDST1 NDST1 chr15 82105876 0.015296 4.787e-08 35365 TRUE reported textfile 2 1 TRUE

327 rs9554252 A G A G 0.0568 -0.002 0.20156 NA FALSE FALSE FALSE GCST006061 0.0082 0.8116 537409 outcome TRUE reported 6927_7_NDST1_NDST1 NDST1 chr13 28095902 0.010342 3.969e-08 35359 TRUE reported textfile 2 1 TRUE

328 rs2706538 A G A G -0.0499 -0.0116 0.482 NA FALSE FALSE FALSE GCST006061 0.0071 0.1018 537409 outcome TRUE reported 12560_9_NT5C_NT5C NT5C chr17 74555513 0.008174 1.029e-09 35371 TRUE reported textfile 2 1 TRUE

329 rs80155227 A G A G -0.7497 -0.0321 0.03137 NA FALSE FALSE FALSE GCST006061 0.0251 0.2009 537409 outcome TRUE reported 12560_9_NT5C_NT5C NT5C chr17 74796149 0.023933 1e-200 35369 TRUE reported textfile 2 1 TRUE

330 rs113745074 C T C T 0.1932 -0.0233 0.15059 NA FALSE FALSE FALSE GCST006061 0.0116 0.04577 537409 outcome TRUE reported 13686_2_IL5RA_IL_5_Ra IL5RA chr7 151245263 0.011725 5.267e-61 35374 TRUE reported textfile 2 1 TRUE

331 rs12521868 T G T G -0.0557 0.014 0.39184 NA FALSE FALSE FALSE

GCST006061 0.0076 0.06431 537409 outcome TRUE reported 13686_2_IL5RA_IL_5_Ra
IL5RA chr5 132448701 0.008617 1.021e-10 35352 TRUE reported
textfile 2 1 TRUE
332 rs12887521 A C A C -0.0577 0.0076 0.34316 NA FALSE FALSE FALSE
GCST006061 0.0074 0.308 537409 outcome TRUE reported 13686_2_IL5RA_IL_5_Ra
IL5RA chr14 102781507 0.00887 7.776e-11 35376 TRUE reported textfile
2 1 TRUE
333 rs16830188 T C T C -0.3108 0.0318 0.01152 NA FALSE FALSE FALSE
GCST006061 0.0224 0.1559 537409 outcome TRUE reported 13686_2_IL5RA_IL_5_Ra
IL5RA chr1 24972350 0.040076 8.815e-15 35371 TRUE reported
textfile 2 1 TRUE
334 rs1999148 T C T C -0.0511 -0.0048 0.37985 NA FALSE FALSE FALSE
GCST006061 0.0072 0.5027 537409 outcome TRUE reported 13686_2_IL5RA_IL_5_Ra
IL5RA chr9 87477230 0.008644 3.392e-09 35356 TRUE reported
textfile 2 1 TRUE
335 rs2511737 C A C A -0.0757 -0.0263 0.27692 NA FALSE FALSE FALSE
GCST006061 0.0084 0.001647 537409 outcome TRUE reported
13686_2_IL5RA_IL_5_Ra IL5RA chr8 102568299 0.00942 9.249e-16 35360 TRUE
reported textfile 2 1 TRUE
336 rs2872507 A G A G -0.0804 0.0243 0.49389 NA FALSE FALSE FALSE
GCST006061 0.0071 0.000663 537409 outcome TRUE reported
13686_2_IL5RA_IL_5_Ra IL5RA chr17 39884510 0.008446 1.737e-21 35379
TRUE reported textfile 2 1 TRUE
337 rs340829 G T G T 0.1924 -0.0122 0.4698 NA FALSE FALSE FALSE
GCST006061 0.0073 0.0967 537409 outcome TRUE reported 13686_2_IL5RA_IL_5_Ra
IL5RA chr3 3066558 0.008758 5.895e-107 35368 TRUE reported textfile
2 1 TRUE
338 rs34562254 A G A G 0.1962 -0.0013 0.07695 NA FALSE FALSE FALSE
GCST006061 0.0109 0.902 537409 outcome TRUE reported 13686_2_IL5RA_IL_5_Ra
IL5RA chr17 16939677 0.015724 9.918e-36 35357 TRUE reported
textfile 2 1 TRUE
339 rs62127753 T C T C 0.0525 -0.0383 0.29111 NA FALSE FALSE FALSE
GCST006061 0.008 1.582e-06 537409 outcome TRUE reported
13686_2_IL5RA_IL_5_Ra IL5RA chr2 25920200 0.009277 1.522e-08 35369
TRUE reported textfile 2 1 TRUE
340 rs62328135 C T C T 0.0778 -0.0103 0.17108 NA FALSE FALSE FALSE
GCST006061 0.01 0.3015 537409 outcome TRUE reported 13686_2_IL5RA_IL_5_Ra
IL5RA chr4 146031340 0.011159 3.13e-12 35360 TRUE reported
textfile 2 1 TRUE
341 rs6766682 C T C T -0.2858 -0.0644 0.02048 NA FALSE FALSE FALSE
GCST006061 0.025 0.009946 537409 outcome TRUE reported
13686_2_IL5RA_IL_5_Ra IL5RA chr3 3103052 0.031443 9.965e-20 35369 TRUE
reported textfile 2 1 TRUE
342 rs7635810 T C T C 0.2918 7e-04 0.37746 NA FALSE FALSE FALSE
GCST006061 0.0081 0.9347 537409 outcome TRUE reported 13686_2_IL5RA_IL_5_Ra
IL5RA chr3 3095016 0.009023 1e-200 35366 TRUE reported textfile 2
1 TRUE
343 rs7758383 G A G A -0.0564 -0.0051 0.49007 NA FALSE FALSE FALSE
GCST006061 0.0072 0.4746 537409 outcome TRUE reported 13686_2_IL5RA_IL_5_Ra
IL5RA chr6 142848586 0.008401 1.899e-11 35350 TRUE reported
textfile 2 1 TRUE
344 rs7766960 G T G T 0.0505 0.0133 0.42824 NA FALSE FALSE FALSE
GCST006061 0.0071 0.06321 537409 outcome TRUE reported 13686_2_IL5RA_IL_5_Ra
IL5RA chr6 41950452 0.008522 3.108e-09 35364 TRUE reported
textfile 2 1 TRUE
345 rs885107 C T C T 0.0654 -0.0155 0.26534 NA FALSE FALSE FALSE
GCST006061 0.0083 0.06053 537409 outcome TRUE reported 13686_2_IL5RA_IL_5_Ra
IL5RA chr16 30661398 0.009483 5.341e-12 35368 TRUE reported
textfile 2 1 TRUE
346 rs980455 C T C T 0.0494 -0.0138 0.41985 NA FALSE FALSE FALSE
GCST006061 0.0073 0.05708 537409 outcome TRUE reported 13686_2_IL5RA_IL_5_Ra
IL5RA chr4 102497800 0.008501 6.196e-09 35341 TRUE reported
textfile 2 1 TRUE
347 rs9831674 A G A G -0.2704 0.0079 0.19935 NA FALSE FALSE FALSE
GCST006061 0.0091 0.3832 537409 outcome TRUE reported 13686_2_IL5RA_IL_5_Ra
IL5RA chr3 3117289 0.010944 8.75e-135 35369 TRUE reported textfile

```
    2   1   TRUE
348 rs9858020   G   A   G   A   0.0673  -0.0064 0.35872 NA  FALSE   FALSE   FALSE
    GCST006061  0.0075  0.3891  537409  outcome TRUE    reported    13686_2_IL5RA_IL_5_Ra
    IL5RA   chr3    196492923   0.009193    2.464e-13   35206   TRUE    reported
    textfile    2   1   TRUE
349 rs9901675   A   G   A   G   0.0951  -0.0272 0.07646 NA  FALSE   FALSE   FALSE
    GCST006061  0.0161  0.0905  537409  outcome TRUE    reported    13686_2_IL5RA_IL_5_Ra
    IL5RA   chr17   7581494 0.015786    1.697e-09   35379   TRUE    reported    textfile
    2   1   TRUE
350 rs1006026   G   A   G   A   -0.1372 -0.0143 0.45689 NA  FALSE   FALSE   FALSE
    GCST006061  0.0072  0.04605 537409  outcome TRUE    reported
    3421_54_TNFSF8_CD30_Ligand  TNFSF8  chr9    114928990   0.008187    4.844e-63   35362
    TRUE    reported    textfile    2   1   TRUE
351 rs10922098  T   C   T   C   -0.1456 -0.009  0.40011 NA  FALSE   FALSE   FALSE
    GCST006061  0.0075  0.2275  537409  outcome TRUE    reported
    3421_54_TNFSF8_CD30_Ligand  TNFSF8  chr1    196695521   0.008308    9.153e-69   35338
    TRUE    reported    textfile    2   1   TRUE
352 rs10982299  C   T   C   T   -0.0547 0.0202  0.35539 NA  FALSE   FALSE   FALSE
    GCST006061  0.0075  0.007311    537409  outcome TRUE    reported
    3421_54_TNFSF8_CD30_Ligand  TNFSF8  chr9    114573441   0.008538    1.486e-10   35362
    TRUE    reported    textfile    2   1   TRUE
353 rs117633773 A   G   A   G   0.1425  0.0067  0.04197 NA  FALSE   FALSE   FALSE
    GCST006061  0.0224  0.7662  537409  outcome TRUE    reported
    3421_54_TNFSF8_CD30_Ligand  TNFSF8  chr17   6953365 0.02013 1.452e-12   35374   TRUE
    reported    textfile    2   1   TRUE
354 rs143501783 C   T   C   T   0.336   -0.0333 0.01116 NA  FALSE   FALSE   FALSE
    GCST006061  0.0443  0.4525  537409  outcome TRUE    reported
    3421_54_TNFSF8_CD30_Ligand  TNFSF8  chr17   7897001 0.037116    1.394e-19   35373
    TRUE    reported    textfile    2   1   TRUE
355 rs1555405   A   G   A   G   -0.0514 -0.0047 0.30381 NA  FALSE   FALSE   FALSE
    GCST006061  0.0081  0.5592  537409  outcome TRUE    reported
    3421_54_TNFSF8_CD30_Ligand  TNFSF8  chr14   100710432   0.00891 7.995e-09   35380
    TRUE    reported    textfile    2   1   TRUE
356 rs2062059   G   A   G   A   -0.0472 -0.0013 0.45355 NA  FALSE   FALSE   FALSE
    GCST006061  0.0073  0.8592  537409  outcome TRUE    reported
    3421_54_TNFSF8_CD30_Ligand  TNFSF8  chr3    98978545    0.00823 9.75e-09    35331
    TRUE    reported    textfile    2   1   TRUE
357 rs3184504   C   T   C   T   -0.0654 0.0074  0.37862 NA  FALSE   FALSE   FALSE
    GCST006061  0.0075  0.3235  537409  outcome TRUE    reported
    3421_54_TNFSF8_CD30_Ligand  TNFSF8  chr12   111446804   0.008419    7.939e-15   35371
    TRUE    reported    textfile    2   1   TRUE
358 rs55714927  T   C   T   C   0.1307  1e-04   0.21166 NA  FALSE   FALSE   FALSE
    GCST006061  0.0098  0.9926  537409  outcome TRUE    reported
    3421_54_TNFSF8_CD30_Ligand  TNFSF8  chr17   7176997 0.010047    1.091e-38   35370
    TRUE    reported    textfile    2   1   TRUE
359 rs681343    T   C   T   C   0.0563  0.0054  0.3954  NA  FALSE   FALSE   FALSE
    GCST006061  0.0076  0.4742  537409  outcome TRUE    reported
    3421_54_TNFSF8_CD30_Ligand  TNFSF8  chr19   48703205    0.008385    1.888e-11   35377
    TRUE    reported    textfile    2   1   TRUE
360 rs697357    G   A   G   A   0.0524  0.0156  0.38884 NA  FALSE   FALSE   FALSE
    GCST006061  0.0072  0.03066 537409  outcome TRUE    reported
    3421_54_TNFSF8_CD30_Ligand  TNFSF8  chr3    165718484   0.008346    3.414e-10   35339
    TRUE    reported    textfile    2   1   TRUE
361 rs10060626  G   T   G   T   -0.0785 -0.0056 0.31712 NA  FALSE   FALSE   FALSE
    GCST006061  0.0088  0.5216  537409  outcome TRUE    reported
    13666_222_CA10_Carbonic_Anhydrase_X CA10    chr5    132468275   0.008907    1.214e-18
    35348   TRUE    reported    textfile    2   1   TRUE
362 rs10157710  T   C   T   C   0.0649  -0.007  0.19506 NA  FALSE   FALSE   FALSE
    GCST006061  0.0087  0.4225  537409  outcome TRUE    reported
    13666_222_CA10_Carbonic_Anhydrase_X CA10    chr1    47496019    0.010668    1.173e-09
    35368   TRUE    reported    textfile    2   1   TRUE
363 rs10420053  T   C   T   C   0.0843  0.0072  0.10162 NA  FALSE   FALSE   FALSE
    GCST006061  0.0111  0.5166  537409  outcome TRUE    reported
    13666_222_CA10_Carbonic_Anhydrase_X CA10    chr19   48889291    0.013715    7.927e-10
    35379   TRUE    reported    textfile    2   1   TRUE
364 rs117399000 A   G   A   G   -0.5585 -0.0295 0.02683 NA  FALSE   FALSE   FALSE
```

GCST006061 0.0241 0.2203 537409 outcome TRUE reported
13666_222_CA10_Carbonic_Anhydrase_X CA10 chr17 52136371 0.025551 6.546e-106
35360 TRUE reported textfile 2 1 TRUE
365 rs1250565 A G A G 0.0679 -0.0195 0.37923 NA FALSE FALSE FALSE
GCST006061 0.0074 0.008136 537409 outcome TRUE reported
13666_222_CA10_Carbonic_Anhydrase_X CA10 chr10 79287258 0.008608 3.071e-15
35368 TRUE reported textfile 2 1 TRUE
366 rs1263963 G A G A -0.0605 -0.0047 0.37085 NA FALSE FALSE FALSE
GCST006061 0.0073 0.5236 537409 outcome TRUE reported
13666_222_CA10_Carbonic_Anhydrase_X CA10 chr17 51701707 0.008722 4.009e-12
35362 TRUE reported textfile 2 1 TRUE
367 rs12945886 G A G A 0.0595 0 0.24063 NA FALSE FALSE FALSE GCST006061
0.0094 0.9973 537409 outcome TRUE reported 13666_222_CA10_Carbonic_Anhydrase_X
CA10 chr17 7169695 0.009848 1.523e-09 35370 TRUE reported textfile
2 1 TRUE
368 rs17879496 T C T C 0.1464 0.0172 0.12179 NA FALSE FALSE FALSE
GCST006061 0.0127 0.1781 537409 outcome TRUE reported
13666_222_CA10_Carbonic_Anhydrase_X CA10 chr6 29944936 0.012651 5.687e-31
35445 TRUE reported textfile 2 1 TRUE
369 rs2106331 A G A G -0.0889 -0.014 0.31975 NA FALSE FALSE FALSE
GCST006061 0.0076 0.06573 537409 outcome TRUE reported
13666_222_CA10_Carbonic_Anhydrase_X CA10 chr17 51660546 0.008999 5.154e-23
35362 TRUE reported textfile 2 1 TRUE
370 rs2938140 T C T C -0.1423 0.0116 0.46038 NA FALSE FALSE FALSE
GCST006061 0.0073 0.1098 537409 outcome TRUE reported
13666_222_CA10_Carbonic_Anhydrase_X CA10 chr17 52099141 0.008394 1.845e-64
35360 TRUE reported textfile 2 1 TRUE
371 rs57880821 A G A G 0.1036 -0.0269 0.06174 NA FALSE FALSE FALSE
GCST006061 0.0143 0.05978 537409 outcome TRUE reported
13666_222_CA10_Carbonic_Anhydrase_X CA10 chr3 186922204 0.017438 2.831e-09
35368 TRUE reported textfile 2 1 TRUE
372 rs669 C T C T -0.0495 -0.0018 0.34586 NA FALSE FALSE FALSE GCST006061
0.0077 0.8114 537409 outcome TRUE reported 13666_222_CA10_Carbonic_Anhydrase_X
CA10 chr12 9079672 0.008777 1.7e-08 35387 TRUE reported textfile 2
1 TRUE
373 rs7528419 G A G A -0.3256 0.0149 0.21011 NA FALSE FALSE FALSE
GCST006061 0.0087 0.0864 537409 outcome TRUE reported
13666_222_CA10_Carbonic_Anhydrase_X CA10 chr1 109274570 0.01018 1e-200 35344
TRUE reported textfile 2 1 TRUE
374 rs7756288 C T C T -0.0599 -0.0214 0.2673 NA FALSE FALSE FALSE
GCST006061 0.0082 0.009152 537409 outcome TRUE reported
13666_222_CA10_Carbonic_Anhydrase_X CA10 chr6 15110481 0.009445 2.27e-10
35384 TRUE reported textfile 2 1 TRUE
375 rs79811137 A G A G -0.2837 0.0339 0.0106 NA FALSE FALSE FALSE
GCST006061 0.0475 0.4747 537409 outcome TRUE reported
13666_222_CA10_Carbonic_Anhydrase_X CA10 chr17 54255149 0.039781 9.92e-13
35374 TRUE reported textfile 2 1 TRUE
376 rs10065637 T C T C -0.0765 0.0029 0.22548 NA FALSE FALSE FALSE
GCST006061 0.0092 0.7556 537409 outcome TRUE reported 6366_38_TXNDC15_TXD15
TXNDC15 chr5 56143024 0.009813 6.415e-15 35359 TRUE reported
textfile 2 1 TRUE
377 rs116078841 G A G A -0.2843 0.0073 0.02153 NA FALSE FALSE FALSE
GCST006061 0.0472 0.8775 537409 outcome TRUE reported 6366_38_TXNDC15_TXD15
TXNDC15 chr6 33556992 0.028712 4.086e-23 35367 TRUE reported
textfile 2 1 TRUE
378 rs1468512 T C T C 0.0636 0.0076 0.1514 NA FALSE FALSE FALSE
GCST006061 0.0109 0.4855 537409 outcome TRUE reported 6366_38_TXNDC15_TXD15
TXNDC15 chr17 69223627 0.011383 2.309e-08 35371 TRUE reported
textfile 2 1 TRUE
379 rs149670015 G A G A 0.2651 0.014 0.02041 NA FALSE FALSE FALSE
GCST006061 0.0347 0.6857 537409 outcome TRUE reported 6366_38_TXNDC15_TXD15
TXNDC15 chr5 133821002 0.030065 1.169e-18 35355 TRUE reported
textfile 2 1 TRUE
380 rs16830188 T C T C -0.3368 0.0318 0.01152 NA FALSE FALSE FALSE
GCST006061 0.0224 0.1559 537409 outcome TRUE reported 6366_38_TXNDC15_TXD15
TXNDC15 chr1 24972350 0.038926 5.048e-18 35371 TRUE reported

textfile 2 1 TRUE
381 rs27302 A G A G -0.0588 0.018 0.44907 NA FALSE FALSE FALSE GCST006061 0.0072 0.01299 537409 outcome TRUE reported 6366_38_TXNDC15_TXD15 TXNDC15 chr5 97038046 0.008262 1.106e-12 35347 TRUE reported textfile 2 1 TRUE
382 rs329122 A G A G 0.0684 0.0159 0.45972 NA FALSE FALSE FALSE GCST006061 0.0072 0.02704 537409 outcome TRUE reported 6366_38_TXNDC15_TXD15 TXNDC15 chr5 134528909 0.008254 1.159e-16 35359 TRUE reported textfile 2 1 TRUE
383 rs35668054 T C T C -0.0975 0.0074 0.07578 NA FALSE FALSE FALSE GCST006061 0.0157 0.6377 537409 outcome TRUE reported 6366_38_TXNDC15_TXD15 TXNDC15 chr2 71827707 0.015368 2.236e-10 35351 TRUE reported textfile 2 1 TRUE
384 rs3733897 G A G A 0.4729 0.012 0.12569 NA FALSE FALSE FALSE GCST006061 0.01 0.2342 537409 outcome TRUE reported 6366_38_TXNDC15_TXD15 TXNDC15 chr5 134887903 0.009435 1e-200 35362 TRUE reported textfile 2 1 TRUE
385 rs59307279 T C T C 0.0617 -0.0103 0.24266 NA FALSE FALSE FALSE GCST006061 0.0087 0.2399 537409 outcome TRUE reported 6366_38_TXNDC15_TXD15 TXNDC15 chr5 95935774 0.009601 1.307e-10 35348 TRUE reported textfile 2 1 TRUE
386 rs651249 C T C T 0.0612 -0.0015 0.32605 NA FALSE FALSE FALSE GCST006061 0.0085 0.8574 537409 outcome TRUE reported 6366_38_TXNDC15_TXD15 TXNDC15 chr5 135234001 0.008759 2.803e-12 35359 TRUE reported textfile 2 1 TRUE
387 rs73169668 T C T C -0.0844 -0.0233 0.14975 NA FALSE FALSE FALSE GCST006061 0.0116 0.0452 537409 outcome TRUE reported 6366_38_TXNDC15_TXD15 TXNDC15 chr7 151233277 0.01142 1.46e-13 35374 TRUE reported textfile 2 1 TRUE
388 rs76035204 G T G T -0.1957 0.0217 0.03087 NA FALSE FALSE FALSE GCST006061 0.0253 0.3915 537409 outcome TRUE reported 6366_38_TXNDC15_TXD15 TXNDC15 chr6 25283509 0.024268 7.383e-16 35376 TRUE reported textfile 2 1 TRUE
389 rs76520335 A G A G 0.1887 -0.0068 0.03258 NA FALSE FALSE FALSE GCST006061 0.0267 0.7976 537409 outcome TRUE reported 6366_38_TXNDC15_TXD15 TXNDC15 chr5 133615156 0.023364 6.666e-16 35353 TRUE reported textfile 2 1 TRUE
390 rs8178824 T C T C 0.2669 -0.0854 0.01066 NA FALSE FALSE FALSE GCST006061 0.023 0.0002035 537409 outcome TRUE reported 6366_38_TXNDC15_TXD15 TXNDC15 chr17 66228657 0.039443 1.318e-11 35369 TRUE reported textfile 2 1 TRUE
391 rs10075736 T C T C 0.4737 0.0079 0.13755 NA FALSE FALSE FALSE GCST006061 0.0095 0.405 537409 outcome TRUE reported 4964_67_ERAP1_ARTS1 ERAP1 chr5 96654353 0.006181 1e-200 35349 TRUE reported textfile 2 1 TRUE
392 rs10474346 T C T C -0.1013 -0.0041 0.11438 NA FALSE FALSE FALSE GCST006061 0.0101 0.6864 537409 outcome TRUE reported 4964_67_ERAP1_ARTS1 ERAP1 chr5 91268322 0.012605 9.254e-16 35340 TRUE reported textfile 2 1 TRUE
393 rs111755236 G A G A 0.368 -0.0166 0.03851 NA FALSE FALSE FALSE GCST006061 0.025 0.5073 537409 outcome TRUE reported 4964_67_ERAP1_ARTS1 ERAP1 chr5 97857486 0.020991 8.221e-69 35344 TRUE reported textfile 2 1 TRUE
394 rs114068443 T C T C 0.1705 -0.0086 0.03146 NA FALSE FALSE FALSE GCST006061 0.0304 0.7769 537409 outcome TRUE reported 4964_67_ERAP1_ARTS1 ERAP1 chr5 102102425 0.023349 2.831e-13 35344 TRUE reported textfile 2 1 TRUE
395 rs1422251 T C T C 0.0668 0.0113 0.4996 NA FALSE FALSE FALSE GCST006061 0.0073 0.1192 537409 outcome TRUE reported 4964_67_ERAP1_ARTS1 ERAP1 chr5 86808103 0.008093 1.536e-16 35344 TRUE reported textfile 2 1 TRUE
396 rs174528 C T C T 0.0371 0.0111 0.40583 NA FALSE FALSE FALSE GCST006061 0.0074 0.1332 537409 outcome TRUE reported 4964_67_ERAP1_ARTS1 ERAP1 chr11 61776027 0.005967 5.035e-10 35363 TRUE reported textfile 2 1 TRUE
397 rs17530412 G A G A -0.1586 0.0235 0.02345 NA FALSE FALSE FALSE

GCST006061 0.0373 0.5291 537409 outcome TRUE reported 4964_67_ERAP1_ARTS1
ERAP1 chr5 85479592 0.027742 1.084e-08 35343 TRUE reported
textfile 2 1 TRUE
398 rs2115459 C T C T 0.0815 0.0167 0.46538 NA FALSE FALSE FALSE
GCST006061 0.0073 0.02135 537409 outcome TRUE reported 4964_67_ERAP1_ARTS1
ERAP1 chr5 89134816 0.0081 8.14e-24 35345 TRUE reported textfile
2 1 TRUE
399 rs62355918 T C T C 0.1013 -0.0516 0.05383 NA FALSE FALSE FALSE
GCST006061 0.0214 0.01574 537409 outcome TRUE reported 4964_67_ERAP1_ARTS1
ERAP1 chr5 107755557 0.01762 8.97e-09 35357 TRUE reported textfile
2 1 TRUE
400 rs62377124 G T G T 0.0732 0.0128 0.3157 NA FALSE FALSE FALSE
GCST006061 0.0082 0.1171 537409 outcome TRUE reported 4964_67_ERAP1_ARTS1
ERAP1 chr5 109007059 0.008714 4.448e-17 35351 TRUE reported
textfile 2 1 TRUE
401 rs66941665 A C A C -0.1517 -0.0169 0.02767 NA FALSE FALSE FALSE
GCST006061 0.0223 0.4476 537409 outcome TRUE reported 4964_67_ERAP1_ARTS1
ERAP1 chr5 84846084 0.025486 2.644e-09 35343 TRUE reported
textfile 2 1 TRUE
402 rs6871839 T C T C 0.0986 -0.0035 0.39841 NA FALSE FALSE FALSE
GCST006061 0.0083 0.6693 537409 outcome TRUE reported 4964_67_ERAP1_ARTS1
ERAP1 chr5 103694668 0.008203 2.808e-33 35342 TRUE reported
textfile 2 1 TRUE
403 rs72774481 T C T C 0.1511 -0.0114 0.0218 NA FALSE FALSE FALSE
GCST006061 0.0335 0.7327 537409 outcome TRUE reported 4964_67_ERAP1_ARTS1
ERAP1 chr5 74085843 0.027445 3.678e-08 35358 TRUE reported
textfile 2 1 TRUE
404 rs76200405 T C T C 0.8343 0.0086 0.02277 NA FALSE FALSE FALSE
GCST006061 0.022 0.6944 537409 outcome TRUE reported 4964_67_ERAP1_ARTS1
ERAP1 chr5 96789627 0.027413 1e-200 35349 TRUE reported textfile
2 1 TRUE
405 rs78174795 T C T C 0.0828 -0.0078 0.18114 NA FALSE FALSE FALSE
GCST006061 0.0098 0.4236 537409 outcome TRUE reported 4964_67_ERAP1_ARTS1
ERAP1 chr5 80162214 0.010555 4.35e-15 35342 TRUE reported
textfile 2 1 TRUE
406 rs10067548 A G A G -0.309 0.0079 0.02341 NA FALSE FALSE FALSE
GCST006061 0.0208 0.7061 537409 outcome TRUE reported 4127_75_C6_C6 C6
chr5 40186614 0.027134 4.813e-30 35360 TRUE reported textfile 2
1 TRUE
407 rs10922098 T C T C -0.123 -0.009 0.40011 NA FALSE FALSE FALSE
GCST006061 0.0075 0.2275 537409 outcome TRUE reported 4127_75_C6_C6 C6
chr1 196695521 0.008289 8.108e-50 35338 TRUE reported textfile 2
1 TRUE
408 rs114815454 A G A G -0.1885 0.0303 0.01864 NA FALSE FALSE FALSE
GCST006061 0.0406 0.4548 537409 outcome TRUE reported 4127_75_C6_C6 C6
chr5 52731861 0.030782 9.137e-10 35361 TRUE reported textfile 2
1 TRUE
409 rs61319746 T C T C -0.2183 -0.0034 0.01173 NA FALSE FALSE FALSE
GCST006061 0.0376 0.9272 537409 outcome TRUE reported 4127_75_C6_C6 C6
chr5 43845111 0.037932 8.66e-09 35361 TRUE reported textfile 2
1 TRUE
410 rs77444140 A G A G -0.5174 0.0241 0.02383 NA FALSE FALSE FALSE
GCST006061 0.0329 0.4631 537409 outcome TRUE reported 4127_75_C6_C6 C6
chr5 41171293 0.026362 9.093e-86 35357 TRUE reported textfile 2
1 TRUE
411 rs10068466 T C T C -0.0511 -0.0165 0.31261 NA FALSE FALSE FALSE
GCST006061 0.0077 0.03178 537409 outcome TRUE reported 4148_49_PAPPA_PAPP_A
PAPPA chr5 142145047 0.009061 1.706e-08 35373 TRUE reported
textfile 2 1 TRUE
412 rs1234212 T C T C -0.048 -0.0033 0.36283 NA FALSE FALSE FALSE
GCST006061 0.0075 0.6657 537409 outcome TRUE reported 4148_49_PAPPA_PAPP_A
PAPPA chr10 87849135 0.008777 4.535e-08 35357 TRUE reported
textfile 2 1 TRUE
413 rs12431998 A G A G -0.0837 -0.0046 0.20834 NA FALSE FALSE FALSE
GCST006061 0.0089 0.6026 537409 outcome TRUE reported 4148_49_PAPPA_PAPP_A
PAPPA chr14 20964296 0.010219 2.592e-16 35342 TRUE reported

textfile 2 1 TRUE
414 rs1260326 C T C T 0.0564 -0.0069 0.34132 NA FALSE FALSE FALSE
GCST006061 0.0073 0.3401 537409 outcome TRUE reported 4148_49_PAPPA_PAPP_A
PAPPA chr2 27508073 0.008898 2.322e-10 35373 TRUE reported
textfile 2 1 TRUE
415 rs13001714 G A G A 0.054 -0.0098 0.42436 NA FALSE FALSE FALSE
GCST006061 0.0072 0.1764 537409 outcome TRUE reported 4148_49_PAPPA_PAPP_A
PAPPA chr2 102344025 0.008516 2.284e-10 35366 TRUE reported
textfile 2 1 TRUE
416 rs13089722 A G A G -0.2361 0.007 0.13554 NA FALSE FALSE FALSE
GCST006061 0.0108 0.5139 537409 outcome TRUE reported 4148_49_PAPPA_PAPP_A
PAPPA chr3 128587914 0.0123 4.015e-82 35363 TRUE reported textfile
2 1 TRUE
417 rs13256023 T C T C -0.1311 -0.0216 0.18528 NA FALSE FALSE FALSE
GCST006061 0.009 0.01682 537409 outcome TRUE reported 4148_49_PAPPA_PAPP_A
PAPPA chr8 60735992 0.01073 2.472e-34 35360 TRUE reported textfile
2 1 TRUE
418 rs17354348 G A G A 0.064 -0.0058 0.30374 NA FALSE FALSE FALSE
GCST006061 0.0085 0.4958 537409 outcome TRUE reported 4148_49_PAPPA_PAPP_A
PAPPA chr2 212971253 0.0091 2.019e-12 35359 TRUE reported textfile
2 1 TRUE
419 rs193607 G A G A -0.0856 0.0023 0.18723 NA FALSE FALSE FALSE
GCST006061 0.0092 0.8047 537409 outcome TRUE reported 4148_49_PAPPA_PAPP_A
PAPPA chr2 201774475 0.010679 1.094e-15 35366 TRUE reported
textfile 2 1 TRUE
420 rs2242892 A G A G -0.1029 0.0126 0.07683 NA FALSE FALSE FALSE
GCST006061 0.0152 0.4068 537409 outcome TRUE reported 4148_49_PAPPA_PAPP_A
PAPPA chr21 35023677 0.015828 7.977e-11 35377 TRUE reported
textfile 2 1 TRUE
421 rs2305637 T C T C 0.1288 5e-04 0.15762 NA FALSE FALSE FALSE
GCST006061 0.0097 0.961 537409 outcome TRUE reported 4148_49_PAPPA_PAPP_A
PAPPA chr3 47004356 0.011425 1.764e-29 35343 TRUE reported
textfile 2 1 TRUE
422 rs2694616 C T C T 0.1253 0.0293 0.48571 NA FALSE FALSE FALSE
GCST006061 0.0071 3.743e-05 537409 outcome TRUE reported
4148_49_PAPPA_PAPP_A PAPPA chr2 61429868 0.008343 5.535e-51 35353
TRUE reported textfile 2 1 TRUE
423 rs35829912 T C T C -0.0504 0.0117 0.28585 NA FALSE FALSE FALSE
GCST006061 0.0081 0.1495 537409 outcome TRUE reported 4148_49_PAPPA_PAPP_A
PAPPA chr15 50052479 0.009185 4.089e-08 35359 TRUE reported
textfile 2 1 TRUE
424 rs3785496 G A G A 0.0541 0.0025 0.28451 NA FALSE FALSE FALSE
GCST006061 0.0085 0.7673 537409 outcome TRUE reported 4148_49_PAPPA_PAPP_A
PAPPA chr17 58196772 0.009257 5.094e-09 35367 TRUE reported
textfile 2 1 TRUE
425 rs4807601 G A G A -0.1124 -0.0126 0.49735 NA FALSE FALSE FALSE
GCST006061 0.0081 0.1185 537409 outcome TRUE reported 4148_49_PAPPA_PAPP_A
PAPPA chr19 4547905 0.008409 9.52e-41 35350 TRUE reported textfile
2 1 TRUE
426 rs7323613 A C A C -0.0784 -0.0011 0.21819 NA FALSE FALSE FALSE
GCST006061 0.0089 0.903 537409 outcome TRUE reported 4148_49_PAPPA_PAPP_A
PAPPA chr13 41007530 0.010156 1.169e-14 35358 TRUE reported
textfile 2 1 TRUE
427 rs78198540 A G A G -0.4003 0.0196 0.01752 NA FALSE FALSE FALSE
GCST006061 0.0401 0.6257 537409 outcome TRUE reported 4148_49_PAPPA_PAPP_A
PAPPA chr19 39727055 0.032099 1.077e-35 35376 TRUE reported
textfile 2 1 TRUE
428 rs7840212 T C T C -0.072 -0.0045 0.32442 NA FALSE FALSE FALSE
GCST006061 0.0075 0.5503 537409 outcome TRUE reported 4148_49_PAPPA_PAPP_A
PAPPA chr8 129587001 0.008924 7.127e-16 35372 TRUE reported
textfile 2 1 TRUE
429 rs10068466 T C T C -0.0503 -0.0165 0.31261 NA FALSE FALSE FALSE
GCST006061 0.0077 0.03178 537409 outcome TRUE reported 15576_158_RNASE3_ECP
RNASE3 chr5 142145047 0.008806 1.118e-08 35373 TRUE reported
textfile 2 1 TRUE
430 rs11156734 T C T C -0.284 -0.0068 0.26296 NA FALSE FALSE FALSE

GCST006061 0.0083 0.4134 537409 outcome TRUE reported 15576_158_RNASE3_ECP
RNASE3 chr14 20879073 0.009328 1e-200 35337 TRUE reported textfile
2 1 TRUE
431 rs13089722 A G A G -0.1081 0.007 0.13554 NA FALSE FALSE FALSE
GCST006061 0.0108 0.5139 537409 outcome TRUE reported 15576_158_RNASE3_ECP
RNASE3 chr3 128587914 0.011989 1.945e-19 35363 TRUE reported
textfile 2 1 TRUE
432 rs13390874 T C T C 0.0574 -0.0015 0.2456 NA FALSE FALSE FALSE
GCST006061 0.0085 0.8576 537409 outcome TRUE reported 15576_158_RNASE3_ECP
RNASE3 chr2 43126091 0.009489 1.455e-09 35360 TRUE reported
textfile 2 1 TRUE
433 rs143057962 A G A G -0.129 -0.0236 0.04091 NA FALSE FALSE FALSE
GCST006061 0.0334 0.4812 537409 outcome TRUE reported 15576_158_RNASE3_ECP
RNASE3 chr14 21115737 0.021147 1.06e-09 35341 TRUE reported
textfile 2 1 TRUE
434 rs147599917 T C T C -0.208 0.0165 0.01746 NA FALSE FALSE FALSE
GCST006061 0.0366 0.6528 537409 outcome TRUE reported 15576_158_RNASE3_ECP
RNASE3 chr14 21053797 0.032508 1.569e-10 35341 TRUE reported
textfile 2 1 TRUE
435 rs17354348 G A G A 0.0496 -0.0058 0.30374 NA FALSE FALSE FALSE
GCST006061 0.0085 0.4958 537409 outcome TRUE reported 15576_158_RNASE3_ECP
RNASE3 chr2 212971253 0.008849 2.079e-08 35359 TRUE reported
textfile 2 1 TRUE
436 rs2006079 A G A G -0.1555 6e-04 0.03461 NA FALSE FALSE FALSE
GCST006061 0.0242 0.9811 537409 outcome TRUE reported 15576_158_RNASE3_ECP
RNASE3 chr14 20799388 0.022481 4.617e-12 35336 TRUE reported
textfile 2 1 TRUE
437 rs2593620 G A G A 0.062 0.0268 0.48569 NA FALSE FALSE FALSE
GCST006061 0.0071 0.000167 537409 outcome TRUE reported
15576_158_RNASE3_ECP RNASE3 chr2 61386126 0.008125 2.334e-14 35353
TRUE reported textfile 2 1 TRUE
438 rs4580194 G A G A 0.0505 -0.0221 0.43092 NA FALSE FALSE FALSE
GCST006061 0.0073 0.002422 537409 outcome TRUE reported
15576_158_RNASE3_ECP RNASE3 chr17 39970224 0.008242 8.956e-10 35379
TRUE reported textfile 2 1 TRUE
439 rs72659967 A G A G -0.3699 -0.0221 0.01054 NA FALSE FALSE FALSE
GCST006061 0.0414 0.5937 537409 outcome TRUE reported 15576_158_RNASE3_ECP
RNASE3 chr14 20675809 0.040484 6.418e-20 35337 TRUE reported
textfile 2 1 TRUE
440 rs76428106 C T C T 0.2138 -0.0446 0.01359 NA FALSE FALSE FALSE
GCST006061 0.0438 0.3085 537409 outcome TRUE reported 15576_158_RNASE3_ECP
RNASE3 chr13 28029870 0.035379 1.512e-09 35363 TRUE reported
textfile 2 1 TRUE
441 rs10068466 T C T C -0.051 -0.0165 0.31261 NA FALSE FALSE FALSE
GCST006061 0.0077 0.03178 537409 outcome TRUE reported 9015_1_PRG3_PRG3
PRG3 chr5 142145047 0.008806 6.972e-09 35373 TRUE reported
textfile 2 1 TRUE
442 rs117024075 A G A G 0.1501 0.0047 0.02463 NA FALSE FALSE FALSE
GCST006061 0.0431 0.9125 537409 outcome TRUE reported 9015_1_PRG3_PRG3
PRG3 chr11 58448182 0.02655 1.572e-08 35368 TRUE reported textfile
2 1 TRUE
443 rs1234212 T C T C -0.0473 -0.0033 0.36283 NA FALSE FALSE FALSE
GCST006061 0.0075 0.6657 537409 outcome TRUE reported 9015_1_PRG3_PRG3
PRG3 chr10 87849135 0.008554 3.213e-08 35357 TRUE reported
textfile 2 1 TRUE
444 rs13089722 A G A G -0.1896 0.007 0.13554 NA FALSE FALSE FALSE
GCST006061 0.0108 0.5139 537409 outcome TRUE reported 9015_1_PRG3_PRG3
PRG3 chr3 128587914 0.011982 2.131e-56 35363 TRUE reported
textfile 2 1 TRUE
445 rs13256023 T C T C -0.123 -0.0216 0.18528 NA FALSE FALSE FALSE
GCST006061 0.009 0.01682 537409 outcome TRUE reported 9015_1_PRG3_PRG3
PRG3 chr8 60735992 0.010444 5.121e-32 35360 TRUE reported
textfile 2 1 TRUE
446 rs17354348 G A G A 0.0734 -0.0058 0.30374 NA FALSE FALSE FALSE
GCST006061 0.0085 0.4958 537409 outcome TRUE reported 9015_1_PRG3_PRG3
PRG3 chr2 212971253 0.008859 1.177e-16 35359 TRUE reported

textfile 2 1 TRUE
447 rs1870028 T C T C -0.1002 0.0076 0.10976 NA FALSE FALSE FALSE
GCST006061 0.0134 0.5688 537409 outcome TRUE reported 9015_1_PRG3_PRG3
PRG3 chr7 50218717 0.013109 2.109e-14 35337 TRUE reported
textfile 2 1 TRUE
448 rs2242886 T C T C -0.0966 0.011 0.07405 NA FALSE FALSE FALSE
GCST006061 0.0151 0.4674 537409 outcome TRUE reported 9015_1_PRG3_PRG3
PRG3 chr21 35015509 0.01572 8.003e-10 35377 TRUE reported textfile
2 1 TRUE
449 rs2694616 C T C T 0.1391 0.0293 0.48571 NA FALSE FALSE FALSE
GCST006061 0.0071 3.743e-05 537409 outcome TRUE reported 9015_1_PRG3_PRG3
PRG3 chr2 61429868 0.00811 6.161e-66 35353 TRUE reported textfile
2 1 TRUE
450 rs61886960 T G T G 0.0864 -0.0076 0.30897 NA FALSE FALSE FALSE
GCST006061 0.0081 0.3495 537409 outcome TRUE reported 9015_1_PRG3_PRG3
PRG3 chr11 57411820 0.008807 1.014e-22 35362 TRUE reported
textfile 2 1 TRUE
451 rs7323613 A C A C -0.1008 -0.0011 0.21819 NA FALSE FALSE FALSE
GCST006061 0.0089 0.903 537409 outcome TRUE reported 9015_1_PRG3_PRG3
PRG3 chr13 41007530 0.009886 2.062e-24 35358 TRUE reported
textfile 2 1 TRUE
452 rs78198540 A G A G -0.2764 0.0196 0.01752 NA FALSE FALSE FALSE
GCST006061 0.0401 0.6257 537409 outcome TRUE reported 9015_1_PRG3_PRG3
PRG3 chr19 39727055 0.031256 9.298e-19 35376 TRUE reported
textfile 2 1 TRUE
453 rs7840212 T C T C -0.078 -0.0045 0.32442 NA FALSE FALSE FALSE
GCST006061 0.0075 0.5503 537409 outcome TRUE reported 9015_1_PRG3_PRG3
PRG3 chr8 129587001 0.008691 2.826e-19 35372 TRUE reported
textfile 2 1 TRUE
454 rs10068638 A G A G 0.0509 0.0084 0.48364 NA FALSE FALSE FALSE
GCST006061 0.0072 0.2437 537409 outcome TRUE reported 5708_1_LEAP2_LEAP2
LEAP2 chr5 115965294 0.008232 6.275e-10 35347 TRUE reported
textfile 2 1 TRUE
455 rs10761737 C T C T -0.0476 -0.0474 0.43543 NA FALSE FALSE FALSE
GCST006061 0.0072 4.478e-11 537409 outcome TRUE reported 5708_1_LEAP2_LEAP2
LEAP2 chr10 63292445 0.008203 6.531e-09 35351 TRUE reported
textfile 2 1 TRUE
456 rs113414093 A G A G 0.0896 0.0533 0.08768 NA FALSE FALSE FALSE
GCST006061 0.0237 0.02426 537409 outcome TRUE reported 5708_1_LEAP2_LEAP2
LEAP2 chr2 218994449 0.01457 7.757e-10 35363 TRUE reported textfile
2 1 TRUE
457 rs1260326 C T C T -0.1049 -0.0069 0.34132 NA FALSE FALSE FALSE
GCST006061 0.0073 0.3401 537409 outcome TRUE reported 5708_1_LEAP2_LEAP2
LEAP2 chr2 27508073 0.008635 5.865e-34 35373 TRUE reported
textfile 2 1 TRUE
458 rs13360043 T C T C 0.1449 0.0024 0.04039 NA FALSE FALSE FALSE
GCST006061 0.0184 0.896 537409 outcome TRUE reported 5708_1_LEAP2_LEAP2
LEAP2 chr5 133307970 0.020954 4.673e-12 35349 TRUE reported
textfile 2 1 TRUE
459 rs150844304 C A C A 0.1486 0.0439 0.0276 NA FALSE FALSE FALSE
GCST006061 0.0244 0.07203 537409 outcome TRUE reported 5708_1_LEAP2_LEAP2
LEAP2 chr15 43434427 0.024794 2.056e-09 35363 TRUE reported
textfile 2 1 TRUE
460 rs152784 G A G A -0.1335 0.0066 0.06195 NA FALSE FALSE FALSE
GCST006061 0.0213 0.7565 537409 outcome TRUE reported 5708_1_LEAP2_LEAP2
LEAP2 chr5 133436739 0.017054 4.958e-15 35351 TRUE reported
textfile 2 1 TRUE
461 rs2954038 A C A C -0.0581 0.0046 0.31787 NA FALSE FALSE FALSE
GCST006061 0.0078 0.5552 537409 outcome TRUE reported 5708_1_LEAP2_LEAP2
LEAP2 chr8 125495147 0.008746 3.073e-11 35367 TRUE reported
textfile 2 1 TRUE
462 rs757537 G A G A 0.3696 -0.009 0.12655 NA FALSE FALSE FALSE
GCST006061 0.0104 0.3831 537409 outcome TRUE reported 5708_1_LEAP2_LEAP2
LEAP2 chr5 132815379 0.012189 1e-200 35348 TRUE reported textfile
2 1 TRUE
463 rs7717377 G A G A 0.1 -0.0068 0.42237 NA FALSE FALSE FALSE GCST006061

0.0073 0.3527 537409 outcome TRUE reported 5708_1_LEAP2_LEAP2 LEAP2 chr5
133155870 0.008281 1.423e-33 35347 TRUE reported textfile 2 1 TRUE

464 rs10069277 G A G A -0.136 -0.0231 0.10271 NA FALSE FALSE FALSE
GCST006061 0.0154 0.1328 537409 outcome TRUE reported 3340_53_THBS4_TSP4
THBS4 chr5 79888253 0.013598 1.496e-23 35341 TRUE reported
textfile 2 1 TRUE

465 rs11899888 G A G A -0.0612 -0.0129 0.20608 NA FALSE FALSE FALSE
GCST006061 0.0104 0.2167 537409 outcome TRUE reported 3340_53_THBS4_TSP4
THBS4 chr2 55875609 0.010253 2.389e-09 35349 TRUE reported
textfile 2 1 TRUE

466 rs13167730 T G T G 0.3753 0.0057 0.08121 NA FALSE FALSE FALSE
GCST006061 0.0131 0.6647 537409 outcome TRUE reported 3340_53_THBS4_TSP4
THBS4 chr5 80074424 0.015294 5.645e-133 35343 TRUE reported
textfile 2 1 TRUE

467 rs2404710 A C A C 0.0928 0.0125 0.4189 NA FALSE FALSE FALSE
GCST006061 0.0072 0.08311 537409 outcome TRUE reported 3340_53_THBS4_TSP4
THBS4 chr5 79960739 0.008421 3.059e-28 35341 TRUE reported
textfile 2 1 TRUE

468 rs3890787 C T C T 0.0562 -0.0049 0.24277 NA FALSE FALSE FALSE
GCST006061 0.0079 0.5382 537409 outcome TRUE reported 3340_53_THBS4_TSP4
THBS4 chr2 69143639 0.009634 5.419e-09 35361 TRUE reported
textfile 2 1 TRUE

469 rs10922098 T C T C -0.0774 -0.009 0.40011 NA FALSE FALSE FALSE
GCST006061 0.0075 0.2275 537409 outcome TRUE reported 11638_42_B3GALT2_B3GT2
B3GALT2 chr1 196695521 0.008218 4.595e-21 35329 TRUE reported
textfile 2 1 TRUE

470 rs148840367 A G A G 0.2034 0.0153 0.01586 NA FALSE FALSE FALSE
GCST006061 0.0354 0.6645 537409 outcome TRUE reported 11638_42_B3GALT2_B3GT2
B3GALT2 chr17 65985133 0.033018 7.262e-10 35361 TRUE reported
textfile 2 1 TRUE

471 rs182233516 T C T C 0.1926 -0.0153 0.01573 NA FALSE FALSE FALSE
GCST006061 0.057 0.7883 537409 outcome TRUE reported 11638_42_B3GALT2_B3GT2
B3GALT2 chr17 66244612 0.032832 4.46e-09 35360 TRUE reported
textfile 2 1 TRUE

472 rs210142 C T C T 0.0502 0.0108 0.28614 NA FALSE FALSE FALSE
GCST006061 0.0079 0.1725 537409 outcome TRUE reported 11638_42_B3GALT2_B3GT2
B3GALT2 chr6 33579060 0.008929 1.884e-08 35358 TRUE reported
textfile 2 1 TRUE

473 rs72850959 C T C T -0.1671 -0.0063 0.09785 NA FALSE FALSE FALSE
GCST006061 0.0137 0.6437 537409 outcome TRUE reported 11638_42_B3GALT2_B3GT2
B3GALT2 chr11 1760578 0.013527 4.71e-35 35350 TRUE reported textfile
2 1 TRUE

474 rs8178824 T C T C 0.4364 -0.0854 0.01066 NA FALSE FALSE FALSE
GCST006061 0.023 0.0002035 537409 outcome TRUE reported
11638_42_B3GALT2_B3GT2 B3GALT2 chr17 66228657 0.038879 3.084e-29 35360
TRUE reported textfile 2 1 TRUE

475 rs9635249 G A G A 0.0723 -0.0086 0.1706 NA FALSE FALSE FALSE
GCST006061 0.0085 0.3087 537409 outcome TRUE reported 11638_42_B3GALT2_B3GT2
B3GALT2 chr14 65331066 0.010661 1.185e-11 35350 TRUE reported
textfile 2 1 TRUE

476 rs10073440 G A G A 0.0491 -0.0103 0.46303 NA FALSE FALSE FALSE
GCST006061 0.0071 0.1497 537409 outcome TRUE reported 9360_33_EDIL3_EDIL3
EDIL3 chr5 84366827 0.008205 2.179e-09 35342 TRUE reported
textfile 2 1 TRUE

477 rs10434639 G A G A -0.0639 0.0013 0.35296 NA FALSE FALSE FALSE
GCST006061 0.0074 0.8573 537409 outcome TRUE reported 9360_33_EDIL3_EDIL3
EDIL3 chr5 83914528 0.008561 8.405e-14 35342 TRUE reported
textfile 2 1 TRUE

478 rs1803274 T C T C 0.0999 0.0149 0.1903 NA FALSE FALSE FALSE
GCST006061 0.0089 0.09298 537409 outcome TRUE reported 9360_33_EDIL3_EDIL3
EDIL3 chr3 165773492 0.010424 9.401e-22 35339 TRUE reported
textfile 2 1 TRUE

479 rs3184504 C T C T 0.0616 0.0074 0.37862 NA FALSE FALSE FALSE
GCST006061 0.0075 0.3235 537409 outcome TRUE reported 9360_33_EDIL3_EDIL3
EDIL3 chr12 111446804 0.008466 3.438e-13 35371 TRUE reported
textfile 2 1 TRUE

480 rs34931250 T C T C 0.1504 0.0028 0.08188 NA FALSE FALSE FALSE
GCST006061 0.0174 0.8738 537409 outcome TRUE reported 9360_33_EDIL3_EDIL3
EDIL3 chr17 68883786 0.015016 1.298e-23 35372 TRUE reported
textfile 2 1 TRUE
481 rs10075805 G A G A -0.0537 0.0023 0.25777 NA FALSE FALSE FALSE
GCST006061 0.0081 0.7801 537409 outcome TRUE reported 15431_31_OTC_OTCase OTC
chr5 31021251 0.009476 1.452e-08 35386 TRUE reported textfile 2
1 TRUE
482 rs151068477 T G T G 0.1891 0.0663 0.01772 NA FALSE FALSE FALSE
GCST006061 0.0574 0.2481 537409 outcome TRUE reported 15431_31_OTC_OTCase OTC
chr10 50882756 0.032274 4.651e-09 35353 TRUE reported textfile 2
1 TRUE
483 rs4835265 A C A C 0.123 -0.0033 0.16265 NA FALSE FALSE FALSE
GCST006061 0.0094 0.7227 537409 outcome TRUE reported 15431_31_OTC_OTCase OTC
chr4 145900258 0.011289 1.216e-27 35360 TRUE reported textfile 2
1 TRUE
484 rs4940689 G A G A -0.0852 0.0053 0.21998 NA FALSE FALSE FALSE
GCST006061 0.0088 0.5452 537409 outcome TRUE reported 15431_31_OTC_OTCase OTC
chr18 58421884 0.010048 2.266e-17 35367 TRUE reported textfile 2
1 TRUE
485 rs7519043 T C T C 0.0763 0.0013 0.43222 NA FALSE FALSE FALSE
GCST006061 0.0073 0.8569 537409 outcome TRUE reported 15431_31_OTC_OTCase OTC
chr1 16174028 0.008459 1.881e-19 35386 TRUE reported textfile 2
1 TRUE
486 rs9645500 G T G T 0.0555 0.0078 0.27524 NA FALSE FALSE FALSE
GCST006061 0.0077 0.3114 537409 outcome TRUE reported 15431_31_OTC_OTCase OTC
chr10 69226967 0.00933 2.71e-09 35362 TRUE reported textfile 2 1
TRUE
487 rs10082476 G A G A 0.0829 0.0084 0.23196 NA FALSE FALSE FALSE
GCST006061 0.0084 0.3173 537409 outcome TRUE reported 12459_13_PLEKHA1_PKHA1
PLEKHA1 chr10 122405138 0.00953 3.36e-18 35373 TRUE reported textfile
2 1 TRUE
488 rs10749451 T C T C -0.2428 -0.008 0.4463 NA FALSE FALSE FALSE
GCST006061 0.0073 0.2758 537409 outcome TRUE reported 12459_13_PLEKHA1_PKHA1
PLEKHA1 chr10 122268525 0.008009 1e-200 35372 TRUE reported textfile
2 1 TRUE
489 rs2420998 C T C T 0.0967 -0.0032 0.07049 NA FALSE FALSE FALSE
GCST006061 0.0135 0.8103 537409 outcome TRUE reported 12459_13_PLEKHA1_PKHA1
PLEKHA1 chr10 122134897 0.015763 8.541e-10 35366 TRUE reported
textfile 2 1 TRUE
490 rs1008369 A G A G 0.0647 0.0012 0.4616 NA FALSE FALSE FALSE
GCST006061 0.0075 0.8691 537409 outcome TRUE reported 14634_13_LHCGR_LSHR
LHCGR chr3 44063667 0.008145 1.969e-15 35353 TRUE reported
textfile 2 1 TRUE
491 rs113685194 G A G A 0.232 -0.008 0.05245 NA FALSE FALSE FALSE
GCST006061 0.0242 0.7403 537409 outcome TRUE reported 14634_13_LHCGR_LSHR
LHCGR chr3 53662786 0.018126 1.652e-37 35350 TRUE reported
textfile 2 1 TRUE
492 rs1329424 G T G T -0.0553 -0.0095 0.39735 NA FALSE FALSE FALSE
GCST006061 0.0075 0.2071 537409 outcome TRUE reported 14634_13_LHCGR_LSHR
LHCGR chr1 196677046 0.007995 4.617e-12 35338 TRUE reported
textfile 2 1 TRUE
493 rs138404620 T G T G -0.1422 -9.00E-04 0.07024 NA FALSE FALSE FALSE
GCST006061 0.0167 0.9581 537409 outcome TRUE reported 14634_13_LHCGR_LSHR
LHCGR chr3 51523225 0.015835 2.71e-19 35342 TRUE reported
textfile 2 1 TRUE
494 rs145663802 T C T C 0.7369 0.059 0.01216 NA FALSE FALSE FALSE
GCST006061 0.0386 0.1267 537409 outcome TRUE reported 14634_13_LHCGR_LSHR
LHCGR chr3 47656167 0.037666 3.136e-85 35344 TRUE reported
textfile 2 1 TRUE
495 rs147367607 T G T G 0.4447 0.0011 0.01885 NA FALSE FALSE FALSE
GCST006061 0.0411 0.9787 537409 outcome TRUE reported 14634_13_LHCGR_LSHR
LHCGR chr3 50407244 0.029862 3.727e-50 35341 TRUE reported
textfile 2 1 TRUE
496 rs187979386 T C T C 0.4078 0.0045 0.03103 NA FALSE FALSE FALSE
GCST006061 0.0242 0.8509 537409 outcome TRUE reported 14634_13_LHCGR_LSHR

LHCGR chr3 48401516 0.023584 5.485e-67 35344 TRUE reported
textfile 2 1 TRUE
497 rs34537989 A G A G 0.1525 -0.0379 0.03973 NA FALSE FALSE FALSE
GCST006061 0.0214 0.07632 537409 outcome TRUE reported 14634_13_LHCGR_LSHR
LHCGR chr3 52667950 0.020937 3.249e-13 35353 TRUE reported
textfile 2 1 TRUE
498 rs10253461 A G A G -0.0782 -2.00E-04 0.40893 NA FALSE FALSE FALSE
GCST006061 0.0074 0.9747 537409 outcome TRUE reported 13929_27_CROT_OCTC
CROT chr7 88967620 0.008258 2.817e-21 35350 TRUE reported
textfile 2 1 TRUE
499 rs10264865 A C A C -0.2042 -0.0574 0.02343 NA FALSE FALSE FALSE
GCST006061 0.0343 0.09366 537409 outcome TRUE reported 13929_27_CROT_OCTC
CROT chr7 85105713 0.026593 1.608e-14 35348 TRUE reported
textfile 2 1 TRUE
500 rs10876550 A G A G 0.0562 -1.00E-04 0.43699 NA FALSE FALSE FALSE
GCST006061 0.0073 0.991 537409 outcome TRUE reported 13929_27_CROT_OCTC
CROT chr12 54318524 0.008064 3.189e-12 35374 TRUE reported
textfile 2 1 TRUE
501 rs114694170 C T C T 0.0907 0.0053 0.08378 NA FALSE FALSE FALSE
GCST006061 0.0175 0.7624 537409 outcome TRUE reported 13929_27_CROT_OCTC
CROT chr5 88884379 0.014486 3.825e-10 35344 TRUE reported
textfile 2 1 TRUE
502 rs190125718 G A G A -0.1078 -0.0168 0.04335 NA FALSE FALSE FALSE
GCST006061 0.0297 0.5705 537409 outcome TRUE reported 13929_27_CROT_OCTC
CROT chr2 61076886 0.01958 3.68e-08 35353 TRUE reported textfile
2 1 TRUE
503 rs2371656 T C T C -0.0771 -0.0096 0.12411 NA FALSE FALSE FALSE
GCST006061 0.0104 0.3579 537409 outcome TRUE reported 13929_27_CROT_OCTC
CROT chr7 83361661 0.012072 1.696e-10 35350 TRUE reported
textfile 2 1 TRUE
504 rs7810499 C T C T 0.106 -0.0044 0.06598 NA FALSE FALSE FALSE
GCST006061 0.0167 0.7912 537409 outcome TRUE reported 13929_27_CROT_OCTC
CROT chr7 87657130 0.016541 1.473e-10 35354 TRUE reported
textfile 2 1 TRUE
505 rs7812191 C T C T 0.0578 -0.0068 0.18836 NA FALSE FALSE FALSE
GCST006061 0.0088 0.442 537409 outcome TRUE reported 13929_27_CROT_OCTC
CROT chr7 87138373 0.010446 3.143e-08 35351 TRUE reported
textfile 2 1 TRUE
506 rs809066 G A G A 0.5317 0.0141 0.10785 NA FALSE FALSE FALSE
GCST006061 0.0121 0.2416 537409 outcome TRUE reported 13929_27_CROT_OCTC
CROT chr7 87358339 0.010636 1e-200 35347 TRUE reported textfile
2 1 TRUE
507 rs1008764 C T C T 0.051 0.0087 0.25345 NA FALSE FALSE FALSE
GCST006061 0.0086 0.3128 537409 outcome TRUE reported 17792_158_ALDH5A1_SSDH
ALDH5A1 chr12 89493872 0.008246 6.232e-10 35357 TRUE reported
textfile 2 1 TRUE
508 rs112049074 T C T C 0.1112 -0.0429 0.05227 NA FALSE FALSE FALSE
GCST006061 0.0276 0.1202 537409 outcome TRUE reported 17792_158_ALDH5A1_SSDH
ALDH5A1 chr6 161236625 0.016218 7.049e-12 35389 TRUE reported
textfile 2 1 TRUE
509 rs12212507 A G A G -0.1065 0.0435 0.03928 NA FALSE FALSE FALSE
GCST006061 0.0182 0.01691 537409 outcome TRUE reported 17792_158_ALDH5A1_SSDH
ALDH5A1 chr6 160650950 0.018479 8.247e-09 35389 TRUE reported
textfile 2 1 TRUE
510 rs150683617 C T C T 0.1907 -0.0177 0.03324 NA FALSE FALSE FALSE
GCST006061 0.0274 0.5186 537409 outcome TRUE reported 17792_158_ALDH5A1_SSDH
ALDH5A1 chr6 160784251 0.02005 1.881e-21 35390 TRUE reported textfile
2 1 TRUE
511 rs17530689 A G A G -0.2052 0.0052 0.02708 NA FALSE FALSE FALSE
GCST006061 0.0319 0.8707 537409 outcome TRUE reported 17792_158_ALDH5A1_SSDH
ALDH5A1 chr13 51539720 0.024966 2.05e-16 35368 TRUE reported
textfile 2 1 TRUE
512 rs180756495 T C T C -0.1898 0.0205 0.02563 NA FALSE FALSE FALSE
GCST006061 0.0222 0.3559 537409 outcome TRUE reported 17792_158_ALDH5A1_SSDH
ALDH5A1 chr13 45715256 0.025648 1.359e-13 35358 TRUE reported
textfile 2 1 TRUE

513 rs3803195 G T G T 0.1258 -0.0071 0.24121 NA FALSE FALSE FALSE
GCST006061 0.0088 0.4192 537409 outcome TRUE reported 17792_158_ALDH5A1_SSDH
ALDH5A1 chr13 45777596 0.009512 6.282e-40 35357 TRUE reported
textfile 2 1 TRUE
514 rs4418728 T G T G -0.0403 0.0179 0.42967 NA FALSE FALSE FALSE
GCST006061 0.0071 0.01164 537409 outcome TRUE reported 17792_158_ALDH5A1_SSDH
ALDH5A1 chr10 93079967 0.007233 2.527e-08 35375 TRUE reported
textfile 2 1 TRUE
515 rs4942469 C T C T 0.3165 -0.0053 0.07497 NA FALSE FALSE FALSE
GCST006061 0.0133 0.6879 537409 outcome TRUE reported 17792_158_ALDH5A1_SSDH
ALDH5A1 chr13 46052106 0.015523 2.115e-92 35361 TRUE reported
textfile 2 1 TRUE
516 rs73186684 T C T C -0.8628 -0.0097 0.02376 NA FALSE FALSE FALSE
GCST006061 0.0283 0.7324 537409 outcome TRUE reported 17792_158_ALDH5A1_SSDH
ALDH5A1 chr13 46011166 0.026756 1e-200 35361 TRUE reported textfile
2 1 TRUE
517 rs780093 C T C T -0.0525 -0.0074 0.34157 NA FALSE FALSE FALSE
GCST006061 0.0073 0.3116 537409 outcome TRUE reported 17792_158_ALDH5A1_SSDH
ALDH5A1 chr2 27519736 0.007603 5.013e-12 35373 TRUE reported
textfile 2 1 TRUE
518 rs9316176 G A G A -0.4918 0.005 0.29884 NA FALSE FALSE FALSE
GCST006061 0.0084 0.5476 537409 outcome TRUE reported 17792_158_ALDH5A1_SSDH
ALDH5A1 chr13 45923055 0.005922 1e-200 35356 TRUE reported textfile
2 1 TRUE
519 rs9317599 C T C T -0.0818 0.0039 0.12943 NA FALSE FALSE FALSE
GCST006061 0.0115 0.7339 537409 outcome TRUE reported 17792_158_ALDH5A1_SSDH
ALDH5A1 chr13 66660505 0.012167 1.779e-11 35351 TRUE reported
textfile 2 1 TRUE
520 rs9534213 T C T C 0.1102 -0.0074 0.27866 NA FALSE FALSE FALSE
GCST006061 0.0087 0.3936 537409 outcome TRUE reported 17792_158_ALDH5A1_SSDH
ALDH5A1 chr13 45791555 0.00907 5.72e-34 35356 TRUE reported textfile
2 1 TRUE
521 rs9534515 A G A G 0.1011 -0.0054 0.4737 NA FALSE FALSE FALSE
GCST006061 0.0071 0.4507 537409 outcome TRUE reported 17792_158_ALDH5A1_SSDH
ALDH5A1 chr13 46902823 0.008105 1.034e-35 35367 TRUE reported
textfile 2 1 TRUE
522 rs1008764 C T C T 0.0529 0.0087 0.25345 NA FALSE FALSE FALSE
GCST006061 0.0086 0.3128 537409 outcome TRUE reported 10666_7_GNPTG_GNPTG
GNPTG chr12 89493872 0.009343 1.497e-08 35345 TRUE reported
textfile 2 1 TRUE
523 rs1132368 A G A G 0.1162 0.0162 0.04066 NA FALSE FALSE FALSE
GCST006061 0.0187 0.3861 537409 outcome TRUE reported 10666_7_GNPTG_GNPTG
GNPTG chr16 2037561 0.020548 1.557e-08 35306 TRUE reported textfile
2 1 TRUE
524 rs143501783 C T C T 0.2136 -0.0333 0.01116 NA FALSE FALSE FALSE
GCST006061 0.0443 0.4525 537409 outcome TRUE reported 10666_7_GNPTG_GNPTG
GNPTG chr17 7897001 0.037064 8.262e-09 35361 TRUE reported textfile
2 1 TRUE
525 rs4764823 A G A G -0.0622 0.0052 0.27829 NA FALSE FALSE FALSE
GCST006061 0.0078 0.503 537409 outcome TRUE reported 10666_7_GNPTG_GNPTG
GNPTG chr12 101825988 0.009167 1.162e-11 35352 TRUE reported
textfile 2 1 TRUE
526 rs58894215 T C T C 0.1725 0.0067 0.02552 NA FALSE FALSE FALSE
GCST006061 0.0302 0.8234 537409 outcome TRUE reported 10666_7_GNPTG_GNPTG
GNPTG chr16 1048778 0.026133 4.09e-11 35301 TRUE reported textfile
2 1 TRUE
527 rs6600145 G A G A -0.1272 -0.0215 0.07929 NA FALSE FALSE FALSE
GCST006061 0.0131 0.1004 537409 outcome TRUE reported 10666_7_GNPTG_GNPTG
GNPTG chr16 1436397 0.015258 7.632e-17 35305 TRUE reported textfile
2 1 TRUE
528 rs660240 C T C T 0.0617 -0.0136 0.205 NA FALSE FALSE FALSE
GCST006061 0.0088 0.1214 537409 outcome TRUE reported 10666_7_GNPTG_GNPTG
GNPTG chr1 109275216 0.010114 1.059e-09 35333 TRUE reported
textfile 2 1 TRUE
529 rs9928413 A G A G 0.0608 -0.0149 0.18655 NA FALSE FALSE FALSE
GCST006061 0.0081 0.06695 537409 outcome TRUE reported 10666_7_GNPTG_GNPTG

GNPTG chr16 71967211 0.010578 9.041e-09 35364 TRUE reported
textfile 2 1 TRUE
530 rs1047891 A C A C 0.0799 0.0155 0.26363 NA FALSE FALSE FALSE
GCST006061 0.0079 0.05042 537409 outcome TRUE reported 9185_15_TFF1_TFF1
TFF1 chr2 210675783 0.009452 2.824e-17 35353 TRUE reported
textfile 2 1 TRUE
531 rs117396825 C T C T 0.4401 -0.0384 0.01462 NA FALSE FALSE FALSE
GCST006061 0.0429 0.3708 537409 outcome TRUE reported 9185_15_TFF1_TFF1
TFF1 chr21 42389187 0.035395 1.712e-35 35333 TRUE reported
textfile 2 1 TRUE
532 rs12990985 C T C T 0.6029 0.0084 0.01902 NA FALSE FALSE FALSE
GCST006061 0.0404 0.8358 537409 outcome TRUE reported 9185_15_TFF1_TFF1
TFF1 chr2 68944477 0.031264 7.279e-83 35358 TRUE reported
textfile 2 1 TRUE
533 rs143227779 G A G A 0.1564 0.0053 0.02726 NA FALSE FALSE FALSE
GCST006061 0.0397 0.8931 537409 outcome TRUE reported 9185_15_TFF1_TFF1
TFF1 chr2 68303206 0.026019 1.843e-09 35362 TRUE reported
textfile 2 1 TRUE
534 rs220250 C T C T -0.063 0.0057 0.20961 NA FALSE FALSE FALSE
GCST006061 0.0085 0.4987 537409 outcome TRUE reported 9185_15_TFF1_TFF1
TFF1 chr21 42052058 0.010241 7.652e-10 35373 TRUE reported
textfile 2 1 TRUE
535 rs225358 T C T C 0.2895 0.0035 0.30886 NA FALSE FALSE FALSE
GCST006061 0.008 0.6582 537409 outcome TRUE reported 9185_15_TFF1_TFF1
TFF1 chr21 42366403 0.008981 1e-200 35341 TRUE reported textfile
2 1 TRUE
536 rs62133344 A C A C 0.5418 -0.0088 0.05058 NA FALSE FALSE FALSE
GCST006061 0.0182 0.6286 537409 outcome TRUE reported 9185_15_TFF1_TFF1
TFF1 chr2 68950137 0.018542 1.079e-187 35358 TRUE reported
textfile 2 1 TRUE
537 rs71469854 T C T C 0.1347 -0.0073 0.16077 NA FALSE FALSE FALSE
GCST006061 0.0114 0.5222 537409 outcome TRUE reported 9185_15_TFF1_TFF1
TFF1 chr11 1193912 0.011253 5.095e-33 35344 TRUE reported textfile
2 1 TRUE
538 rs79188300 C T C T 0.171 -0.0233 0.03283 NA FALSE FALSE FALSE
GCST006061 0.0296 0.4323 537409 outcome TRUE reported 9185_15_TFF1_TFF1
TFF1 chr2 70736005 0.023185 1.638e-13 35359 TRUE reported
textfile 2 1 TRUE
539 rs80334578 C T C T 0.1782 0.0494 0.0245 NA FALSE FALSE FALSE
GCST006061 0.0445 0.2675 537409 outcome TRUE reported 9185_15_TFF1_TFF1
TFF1 chr11 1198256 0.027008 4.166e-11 35344 TRUE reported textfile
2 1 TRUE
540 rs10096633 T C T C -0.0835 0.0162 0.09986 NA FALSE FALSE FALSE
GCST006061 0.0106 0.1274 537409 outcome TRUE reported 6289_78_ARHGAP36_RHG36
ARHGAP36 chr8 19973410 0.013451 5.382e-10 35368 TRUE reported
textfile 2 1 TRUE
541 rs10922098 T C T C -0.0614 -0.009 0.40011 NA FALSE FALSE FALSE
GCST006061 0.0075 0.2275 537409 outcome TRUE reported 6289_78_ARHGAP36_RHG36
ARHGAP36 chr1 196695521 0.008273 1.158e-13 35338 TRUE reported
textfile 2 1 TRUE
542 rs6445035 A G A G 0.1008 0.0154 0.1904 NA FALSE FALSE FALSE
GCST006061 0.0089 0.08256 537409 outcome TRUE reported 6289_78_ARHGAP36_RHG36
ARHGAP36 chr3 165762312 0.010277 1.036e-22 35339 TRUE reported
textfile 2 1 TRUE
543 rs67769959 G A G A 0.1578 -5.00E-04 0.02069 NA FALSE FALSE FALSE
GCST006061 0.0241 0.9843 537409 outcome TRUE reported 6289_78_ARHGAP36_RHG36
ARHGAP36 chr2 174785314 0.028384 2.705e-08 35360 TRUE reported
textfile 2 1 TRUE
544 rs10096633 T C T C -0.1356 0.0162 0.09986 NA FALSE FALSE FALSE
GCST006061 0.0106 0.1274 537409 outcome TRUE reported 13691_10_SLC5A8_SC5A8
SLC5A8 chr8 19973410 0.013973 2.88e-22 35368 TRUE reported
textfile 2 1 TRUE
545 rs111696008 A G A G -0.2475 -0.0259 0.02443 NA FALSE FALSE FALSE
GCST006061 0.0401 0.519 537409 outcome TRUE reported 13691_10_SLC5A8_SC5A8
SLC5A8 chr2 216622986 0.028085 1.223e-18 35359 TRUE reported
textfile 2 1 TRUE

546 rs112875651 A G A G -0.062 -0.0016 0.40253 NA FALSE FALSE FALSE
GCST006061 0.0076 0.8301 537409 outcome TRUE reported 13691_10_SLC5A8_SC5A8
SLC5A8 chr8 125494452 0.008647 7.478e-13 35367 TRUE reported
textfile 2 1 TRUE
547 rs11591147 T G T G -0.2876 0.0511 0.01208 NA FALSE FALSE FALSE
GCST006061 0.0356 0.1511 537409 outcome TRUE reported 13691_10_SLC5A8_SC5A8
SLC5A8 chr1 55039974 0.03985 5.31e-13 35363 TRUE reported textfile
2 1 TRUE
548 rs11897611 T C T C -0.0698 -0.0223 0.17244 NA FALSE FALSE FALSE
GCST006061 0.0096 0.02028 537409 outcome TRUE reported 13691_10_SLC5A8_SC5A8
SLC5A8 chr2 20575557 0.011189 4.418e-10 35372 TRUE reported
textfile 2 1 TRUE
549 rs12026141 T C T C -0.0546 0.0094 0.31246 NA FALSE FALSE FALSE
GCST006061 0.0076 0.2137 537409 outcome TRUE reported 13691_10_SLC5A8_SC5A8
SLC5A8 chr1 201980465 0.009154 2.449e-09 35357 TRUE reported
textfile 2 1 TRUE
550 rs144202860 A G A G -0.2395 -0.0629 0.01274 NA FALSE FALSE FALSE
GCST006061 0.0538 0.2423 537409 outcome TRUE reported 13691_10_SLC5A8_SC5A8
SLC5A8 chr2 216625740 0.037618 1.932e-10 35359 TRUE reported
textfile 2 1 TRUE
551 rs148077724 G A G A 0.2204 -0.0095 0.02611 NA FALSE FALSE FALSE
GCST006061 0.0214 0.6574 537409 outcome TRUE reported 13691_10_SLC5A8_SC5A8
SLC5A8 chr3 165952844 0.026219 4.242e-17 35340 TRUE reported
textfile 2 1 TRUE
552 rs261290 C T C T -0.0756 0.0145 0.36352 NA FALSE FALSE FALSE
GCST006061 0.0074 0.05053 537409 outcome TRUE reported 13691_10_SLC5A8_SC5A8
SLC5A8 chr15 58386521 0.008744 5.317e-18 35364 TRUE reported
textfile 2 1 TRUE
553 rs3764261 A C A C -0.1066 0.0132 0.32148 NA FALSE FALSE FALSE
GCST006061 0.0076 0.08361 537409 outcome TRUE reported 13691_10_SLC5A8_SC5A8
SLC5A8 chr16 56959412 0.008984 1.798e-32 35369 TRUE reported
textfile 2 1 TRUE
554 rs473224 G T G T -0.0775 -0.0043 0.1252 NA FALSE FALSE FALSE
GCST006061 0.0103 0.677 537409 outcome TRUE reported 13691_10_SLC5A8_SC5A8
SLC5A8 chr15 58445142 0.012766 1.271e-09 35366 TRUE reported
textfile 2 1 TRUE
555 rs483465 G A G A 0.0723 -0.0204 0.24663 NA FALSE FALSE FALSE
GCST006061 0.0083 0.01415 537409 outcome TRUE reported 13691_10_SLC5A8_SC5A8
SLC5A8 chr3 136329135 0.00984 2.022e-13 35361 TRUE reported textfile
2 1 TRUE
556 rs62290472 A G A G 0.107 0.011 0.08266 NA FALSE FALSE FALSE
GCST006061 0.0106 0.3013 537409 outcome TRUE reported 13691_10_SLC5A8_SC5A8
SLC5A8 chr3 162193597 0.015436 4.156e-12 35340 TRUE reported
textfile 2 1 TRUE
557 rs6445035 A G A G 0.2386 0.0154 0.1904 NA FALSE FALSE FALSE
GCST006061 0.0089 0.08256 537409 outcome TRUE reported 13691_10_SLC5A8_SC5A8
SLC5A8 chr3 165762312 0.01067 9.494e-111 35339 TRUE reported textfile
2 1 TRUE
558 rs660240 C T C T 0.1077 -0.0136 0.205 NA FALSE FALSE FALSE
GCST006061 0.0088 0.1214 537409 outcome TRUE reported 13691_10_SLC5A8_SC5A8
SLC5A8 chr1 109275216 0.010461 7.391e-25 35345 TRUE reported
textfile 2 1 TRUE
559 rs673548 A G A G 0.214 -0.009 0.23304 NA FALSE FALSE FALSE
GCST006061 0.0085 0.2884 537409 outcome TRUE reported 13691_10_SLC5A8_SC5A8
SLC5A8 chr2 21014672 0.009986 7.017e-102 35373 TRUE reported
textfile 2 1 TRUE
560 rs73013176 C T C T -0.1524 -0.0519 0.02391 NA FALSE FALSE FALSE
GCST006061 0.0478 0.2776 537409 outcome TRUE reported 13691_10_SLC5A8_SC5A8
SLC5A8 chr19 11036850 0.027748 3.966e-08 35379 TRUE reported
textfile 2 1 TRUE
561 rs73169103 T C T C 0.1427 0.0178 0.03563 NA FALSE FALSE FALSE
GCST006061 0.0249 0.4756 537409 outcome TRUE reported 13691_10_SLC5A8_SC5A8
SLC5A8 chr3 167190675 0.02275 3.551e-10 35354 TRUE reported textfile
2 1 TRUE
562 rs7412 T C T C -0.3052 -0.0027 0.05941 NA FALSE FALSE FALSE GCST006061
0.0138 0.8459 537409 outcome TRUE reported 13691_10_SLC5A8_SC5A8 SLC5A8

chr19 44908822 0.017752 3.048e-66 35367 TRUE reported textfile 2
1 TRUE
563 rs74717300 T G T G -0.094 0.0033 0.08971 NA FALSE FALSE FALSE
GCST006061 0.0178 0.8533 537409 outcome TRUE reported 13691_10_SLC5A8_SC5A8
SLC5A8 chr2 21313631 0.014731 1.759e-10 35372 TRUE reported
textfile 2 1 TRUE
564 rs780093 C T C T -0.0516 -0.0074 0.34157 NA FALSE FALSE FALSE
GCST006061 0.0073 0.3116 537409 outcome TRUE reported 13691_10_SLC5A8_SC5A8
SLC5A8 chr2 27519736 0.008931 7.573e-09 35373 TRUE reported
textfile 2 1 TRUE
565 rs78058190 A G A G 0.0959 0.0697 0.09004 NA FALSE FALSE FALSE
GCST006061 0.0227 0.002119 537409 outcome TRUE reported
13691_10_SLC5A8_SC5A8 SLC5A8 chr2 218835276 0.014844 1.043e-10 35362
TRUE reported textfile 2 1 TRUE
566 rs8106503 C T C T -0.1348 0.0102 0.08927 NA FALSE FALSE FALSE
GCST006061 0.0116 0.3814 537409 outcome TRUE reported 13691_10_SLC5A8_SC5A8
SLC5A8 chr19 11086210 0.014768 6.993e-20 35379 TRUE reported
textfile 2 1 TRUE
567 rs10096633 T C T C -0.0855 0.0162 0.09986 NA FALSE FALSE FALSE
GCST006061 0.0106 0.1274 537409 outcome TRUE reported 11218_84_TPMT_TPMT
TPMT chr8 19973410 0.013359 1.552e-10 35359 TRUE reported
textfile 2 1 TRUE
568 rs10822155 A C A C 0.0656 -0.0472 0.43479 NA FALSE FALSE FALSE
GCST006061 0.0072 4.622e-11 537409 outcome TRUE reported 11218_84_TPMT_TPMT
TPMT chr10 63311455 0.008088 5.044e-16 35342 TRUE reported
textfile 2 1 TRUE
569 rs12995461 C A C A 0.0605 0.0086 0.21643 NA FALSE FALSE FALSE
GCST006061 0.008 0.2842 537409 outcome TRUE reported 11218_84_TPMT_TPMT
TPMT chr2 27555300 0.009676 4.036e-10 35364 TRUE reported
textfile 2 1 TRUE
570 rs1355538 G A G A -0.0816 -0.0076 0.39847 NA FALSE FALSE FALSE
GCST006061 0.0072 0.2923 537409 outcome TRUE reported 11218_84_TPMT_TPMT
TPMT chr3 165787389 0.008184 2.041e-23 35329 TRUE reported
textfile 2 1 TRUE
571 rs261290 C T C T -0.047 0.0145 0.36352 NA FALSE FALSE FALSE
GCST006061 0.0074 0.05053 537409 outcome TRUE reported 11218_84_TPMT_TPMT
TPMT chr15 58386521 0.008361 1.898e-08 35355 TRUE reported
textfile 2 1 TRUE
572 rs3816117 C T C T -0.067 0.0137 0.49421 NA FALSE FALSE FALSE
GCST006061 0.0071 0.05455 537409 outcome TRUE reported 11218_84_TPMT_TPMT
TPMT chr16 56962246 0.008033 7.408e-17 35360 TRUE reported
textfile 2 1 TRUE
573 rs775824 C T C T 0.0729 0.0102 0.16338 NA FALSE FALSE FALSE
GCST006061 0.0101 0.3143 537409 outcome TRUE reported 11218_84_TPMT_TPMT
TPMT chr19 55050202 0.010843 1.781e-11 35372 TRUE reported
textfile 2 1 TRUE
574 rs9332701 G A G A 0.3568 -0.0115 0.07189 NA FALSE FALSE FALSE
GCST006061 0.0196 0.5575 537409 outcome TRUE reported 11218_84_TPMT_TPMT
TPMT chr1 169515529 0.015367 2.964e-119 35355 TRUE reported
textfile 2 1 TRUE
575 rs10096633 T C T C -0.1259 0.0162 0.09986 NA FALSE FALSE FALSE
GCST006061 0.0106 0.1274 537409 outcome TRUE reported 8905_20_KCNMB3_KCMB3
KCNMB3 chr8 19973410 0.013578 1.819e-20 35368 TRUE reported
textfile 2 1 TRUE
576 rs111266377 T G T G 0.1586 0.037 0.02883 NA FALSE FALSE FALSE
GCST006061 0.0258 0.1519 537409 outcome TRUE reported 8905_20_KCNMB3_KCMB3
KCNMB3 chr3 167239870 0.024383 7.787e-11 35354 TRUE reported
textfile 2 1 TRUE
577 rs111696008 A G A G -0.1987 -0.0259 0.02443 NA FALSE FALSE FALSE
GCST006061 0.0401 0.519 537409 outcome TRUE reported 8905_20_KCNMB3_KCMB3
KCNMB3 chr2 216622986 0.027254 3.083e-13 35359 TRUE reported
textfile 2 1 TRUE
578 rs116843064 A G A G -0.1725 0.0449 0.02426 NA FALSE FALSE FALSE
GCST006061 0.0321 0.1613 537409 outcome TRUE reported 8905_20_KCNMB3_KCMB3
KCNMB3 chr19 8364439 0.026273 5.183e-11 35375 TRUE reported textfile
2 1 TRUE

579 rs12485327 T G T G 0.0872 0.0109 0.07334 NA FALSE FALSE FALSE
GCST006061 0.0118 0.3548 537409 outcome TRUE reported 8905_20_KCNMB3_KCMB3
KCNMB3 chr3 162196930 0.015827 3.599e-08 35340 TRUE reported
textfile 2 1 TRUE
580 rs148077724 G A G A 0.1818 -0.0095 0.02611 NA FALSE FALSE FALSE
GCST006061 0.0214 0.6574 537409 outcome TRUE reported 8905_20_KCNMB3_KCMB3
KCNMB3 chr3 165952844 0.025451 9.114e-13 35340 TRUE reported
textfile 2 1 TRUE
581 rs3764261 A C A C -0.0778 0.0132 0.32148 NA FALSE FALSE FALSE
GCST006061 0.0076 0.08361 537409 outcome TRUE reported 8905_20_KCNMB3_KCMB3
KCNMB3 chr16 56959412 0.00873 5.027e-19 35369 TRUE reported textfile
2 1 TRUE
582 rs571513 G A G A 0.0667 -0.0143 0.23433 NA FALSE FALSE FALSE
GCST006061 0.0084 0.08851 537409 outcome TRUE reported 8905_20_KCNMB3_KCMB3
KCNMB3 chr3 136376193 0.009718 6.728e-12 35361 TRUE reported
textfile 2 1 TRUE
583 rs602633 G T G T 0.0669 -0.0162 0.20391 NA FALSE FALSE FALSE
GCST006061 0.0088 0.06452 537409 outcome TRUE reported 8905_20_KCNMB3_KCMB3
KCNMB3 chr1 109278889 0.010171 4.792e-11 35344 TRUE reported
textfile 2 1 TRUE
584 rs6445035 A G A G 0.2082 0.0154 0.1904 NA FALSE FALSE FALSE
GCST006061 0.0089 0.08256 537409 outcome TRUE reported 8905_20_KCNMB3_KCMB3
KCNMB3 chr3 165762312 0.010367 1.023e-89 35339 TRUE reported
textfile 2 1 TRUE
585 rs679899 A G A G 0.2091 -0.0016 0.48235 NA FALSE FALSE FALSE
GCST006061 0.0073 0.8278 537409 outcome TRUE reported 8905_20_KCNMB3_KCMB3
KCNMB3 chr2 21028042 0.008169 1.606e-144 35373 TRUE reported
textfile 2 1 TRUE
586 rs7412 T C T C -0.1915 -0.0027 0.05941 NA FALSE FALSE FALSE GCST006061
0.0138 0.8459 537409 outcome TRUE reported 8905_20_KCNMB3_KCMB3 KCNMB3
chr19 44908822 0.017261 1.332e-28 35367 TRUE reported textfile 2
1 TRUE
587 rs74717300 T G T G -0.1281 0.0033 0.08971 NA FALSE FALSE FALSE
GCST006061 0.0178 0.8533 537409 outcome TRUE reported 8905_20_KCNMB3_KCMB3
KCNMB3 chr2 21313631 0.014293 3.17e-19 35372 TRUE reported
textfile 2 1 TRUE
588 rs77804075 T C T C 0.1151 -0.0144 0.04071 NA FALSE FALSE FALSE
GCST006061 0.0223 0.5185 537409 outcome TRUE reported 8905_20_KCNMB3_KCMB3
KCNMB3 chr2 22956435 0.020429 1.758e-08 35369 TRUE reported
textfile 2 1 TRUE
589 rs10096633 T C T C -0.1315 0.0162 0.09986 NA FALSE FALSE FALSE
GCST006061 0.0106 0.1274 537409 outcome TRUE reported 7144_234_KAZALD1_KAZD1
KAZALD1 chr8 19973410 0.013898 3.025e-21 35368 TRUE reported
textfile 2 1 TRUE
590 rs141469619 G A G A 0.2295 -0.0376 0.01201 NA FALSE FALSE FALSE
GCST006061 0.0531 0.4786 537409 outcome TRUE reported 7144_234_KAZALD1_KAZD1
KAZALD1 chr11 116843577 0.037344 7.97e-10 35377 TRUE reported
textfile 2 1 TRUE
591 rs150026080 T C T C -0.2654 0.0368 0.04294 NA FALSE FALSE FALSE
GCST006061 0.0381 0.3344 537409 outcome TRUE reported 7144_234_KAZALD1_KAZD1
KAZALD1 chr10 73596323 0.020818 3.163e-37 35370 TRUE reported
textfile 2 1 TRUE
592 rs1707652 T C T C 0.0647 0.0081 0.3102 NA FALSE FALSE FALSE
GCST006061 0.0074 0.2701 537409 outcome TRUE reported 7144_234_KAZALD1_KAZD1
KAZALD1 chr3 165761011 0.009023 7.454e-13 35339 TRUE reported
textfile 2 1 TRUE
593 rs2070895 A G A G 0.0919 0.0021 0.19622 NA FALSE FALSE FALSE
GCST006061 0.0084 0.7984 537409 outcome TRUE reported 7144_234_KAZALD1_KAZD1
KAZALD1 chr15 58431740 0.010507 2.195e-18 35366 TRUE reported
textfile 2 1 TRUE
594 rs2414577 C T C T 0.0831 -0.0167 0.41557 NA FALSE FALSE FALSE
GCST006061 0.0076 0.02713 537409 outcome TRUE reported 7144_234_KAZALD1_KAZD1
KAZALD1 chr15 58388439 0.008469 9.932e-23 35364 TRUE reported
textfile 2 1 TRUE
595 rs2633320 C T C T -0.1343 -0.0019 0.27163 NA FALSE FALSE FALSE
GCST006061 0.0079 0.806 537409 outcome TRUE reported 7144_234_KAZALD1_KAZD1

KAZALD1 chr10 73933548 0.009397 2.451e-46 35368 TRUE reported
textfile 2 1 TRUE
596 rs2954038 A C A C -0.0678 0.0046 0.31787 NA FALSE FALSE FALSE
GCST006061 0.0078 0.5552 537409 outcome TRUE reported 7144_234_KAZALD1_KAZD1
KAZALD1 chr8 125495147 0.008985 4.492e-14 35367 TRUE reported
textfile 2 1 TRUE
597 rs344773 A G A G 0.0566 -0.0101 0.24735 NA FALSE FALSE FALSE
GCST006061 0.0084 0.2312 537409 outcome TRUE reported 7144_234_KAZALD1_KAZD1
KAZALD1 chr19 43675608 0.00968 5e-09 35367 TRUE reported textfile 2
1 TRUE
598 rs3816117 C T C T -0.1136 0.0137 0.49421 NA FALSE FALSE FALSE
GCST006061 0.0071 0.05455 537409 outcome TRUE reported 7144_234_KAZALD1_KAZD1
KAZALD1 chr16 56962246 0.008364 5.143e-42 35369 TRUE reported
textfile 2 1 TRUE
599 rs673548 A G A G 0.1052 -0.009 0.23304 NA FALSE FALSE FALSE
GCST006061 0.0085 0.2884 537409 outcome TRUE reported 7144_234_KAZALD1_KAZD1
KAZALD1 chr2 21014672 0.00993 3.183e-26 35373 TRUE reported textfile
2 1 TRUE
600 rs75425987 T C T C -0.153 0.0091 0.04572 NA FALSE FALSE FALSE
GCST006061 0.018 0.6139 537409 outcome TRUE reported 7144_234_KAZALD1_KAZD1
KAZALD1 chr19 43683342 0.019876 1.383e-14 35367 TRUE reported
textfile 2 1 TRUE
601 rs77908245 A G A G -0.2518 -0.0113 0.03351 NA FALSE FALSE FALSE
GCST006061 0.0369 0.7587 537409 outcome TRUE reported 7144_234_KAZALD1_KAZD1
KAZALD1 chr10 72228064 0.023447 6.672e-27 35373 TRUE reported
textfile 2 1 TRUE
602 rs10096633 T C T C -0.1783 0.0162 0.09986 NA FALSE FALSE FALSE
GCST006061 0.0106 0.1274 537409 outcome TRUE reported 13268_45_WNT5A_WNT5A
WNT5A chr8 19973410 0.01405 6.695e-37 35359 TRUE reported textfile
2 1 TRUE
603 rs115365379 C T C T -0.2312 -0.0666 0.01178 NA FALSE FALSE FALSE
GCST006061 0.05 0.1828 537409 outcome TRUE reported 13268_45_WNT5A_WNT5A
WNT5A chr1 63830105 0.03901 3.092e-09 35344 TRUE reported textfile
2 1 TRUE
604 rs116843064 A G A G -0.2069 0.0449 0.02426 NA FALSE FALSE FALSE
GCST006061 0.0321 0.1613 537409 outcome TRUE reported 13268_45_WNT5A_WNT5A
WNT5A chr19 8364439 0.027178 2.684e-14 35366 TRUE reported textfile
2 1 TRUE
605 rs141469619 G A G A 0.2779 -0.0376 0.01201 NA FALSE FALSE FALSE
GCST006061 0.0531 0.4786 537409 outcome TRUE reported 13268_45_WNT5A_WNT5A
WNT5A chr11 116843577 0.037778 1.893e-13 35368 TRUE reported
textfile 2 1 TRUE
606 rs150844304 C A C A 0.1759 0.0439 0.0276 NA FALSE FALSE FALSE
GCST006061 0.0244 0.07203 537409 outcome TRUE reported 13268_45_WNT5A_WNT5A
WNT5A chr15 43434427 0.025768 8.709e-12 35354 TRUE reported
textfile 2 1 TRUE
607 rs17123220 A G A G -0.2259 -0.03 0.01048 NA FALSE FALSE FALSE
GCST006061 0.0282 0.2867 537409 outcome TRUE reported 13268_45_WNT5A_WNT5A
WNT5A chr1 62238014 0.040891 3.305e-08 35348 TRUE reported
textfile 2 1 TRUE
608 rs261290 C T C T -0.1218 0.0145 0.36352 NA FALSE FALSE FALSE
GCST006061 0.0074 0.05053 537409 outcome TRUE reported 13268_45_WNT5A_WNT5A
WNT5A chr15 58386521 0.008791 1.181e-43 35355 TRUE reported
textfile 2 1 TRUE
609 rs34250990 G A G A 0.117 -0.0122 0.04399 NA FALSE FALSE FALSE
GCST006061 0.022 0.5798 537409 outcome TRUE reported 13268_45_WNT5A_WNT5A
WNT5A chr16 57614249 0.020343 8.859e-09 35362 TRUE reported
textfile 2 1 TRUE
610 rs3816117 C T C T -0.1488 0.0137 0.49421 NA FALSE FALSE FALSE
GCST006061 0.0071 0.05455 537409 outcome TRUE reported 13268_45_WNT5A_WNT5A
WNT5A chr16 56962246 0.008444 1.699e-69 35360 TRUE reported
textfile 2 1 TRUE
611 rs473224 G T G T -0.1062 -0.0043 0.1252 NA FALSE FALSE FALSE
GCST006061 0.0103 0.677 537409 outcome TRUE reported 13268_45_WNT5A_WNT5A
WNT5A chr15 58445142 0.012845 1.368e-16 35357 TRUE reported
textfile 2 1 TRUE

612 rs6445035 A G A G 0.1295 0.0154 0.1904 NA FALSE FALSE FALSE
GCST006061 0.0089 0.08256 537409 outcome TRUE reported 13268_45_WNT5A_WNT5A
WNT5A chr3 165762312 0.010741 1.795e-33 35330 TRUE reported
textfile 2 1 TRUE
613 rs673548 A G A G 0.0919 -0.009 0.23304 NA FALSE FALSE FALSE
GCST006061 0.0085 0.2884 537409 outcome TRUE reported 13268_45_WNT5A_WNT5A
WNT5A chr2 21014672 0.010043 5.68e-20 35364 TRUE reported
textfile 2 1 TRUE
614 rs77391147 G A G A 0.2055 0.0022 0.01924 NA FALSE FALSE FALSE
GCST006061 0.0358 0.9503 537409 outcome TRUE reported 13268_45_WNT5A_WNT5A
WNT5A chr9 104765088 0.030658 2.041e-11 35354 TRUE reported
textfile 2 1 TRUE
615 rs780093 C T C T -0.0929 -0.0074 0.34157 NA FALSE FALSE FALSE
GCST006061 0.0073 0.3116 537409 outcome TRUE reported 13268_45_WNT5A_WNT5A
WNT5A chr2 27519736 0.008931 2.438e-25 35364 TRUE reported
textfile 2 1 TRUE
616 rs78058190 A G A G 0.0846 0.0697 0.09004 NA FALSE FALSE FALSE
GCST006061 0.0227 0.002119 537409 outcome TRUE reported
13268_45_WNT5A_WNT5A WNT5A chr2 218835276 0.014863 1.257e-08 35353
TRUE reported textfile 2 1 TRUE
617 rs10096633 T C T C -0.0792 0.0162 0.09986 NA FALSE FALSE FALSE
GCST006061 0.0106 0.1274 537409 outcome TRUE reported 12514_16_METTL1_METTL1
METTL1 chr8 19973410 0.013408 3.486e-09 35359 TRUE reported
textfile 2 1 TRUE
618 rs1707652 T C T C 0.1229 0.0081 0.3102 NA FALSE FALSE FALSE
GCST006061 0.0074 0.2701 537409 outcome TRUE reported 12514_16_METTL1_METTL1
METTL1 chr3 165761011 0.008692 2.154e-45 35330 TRUE reported
textfile 2 1 TRUE
619 rs429358 C T C T -0.1387 0.005 0.17407 NA FALSE FALSE FALSE
GCST006061 0.0103 0.6319 537409 outcome TRUE reported 12514_16_METTL1_METTL1
METTL1 chr19 44908684 0.010743 3.906e-38 35359 TRUE reported
textfile 2 1 TRUE
620 rs704 A G A G 0.0451 -0.0155 0.49189 NA FALSE FALSE FALSE GCST006061
0.0072 0.03144 537409 outcome TRUE reported 12514_16_METTL1_METTL1 METTL1
chr17 28367840 0.008082 2.397e-08 35351 TRUE reported textfile 2
1 TRUE
621 rs780093 C T C T -0.0503 -0.0074 0.34157 NA FALSE FALSE FALSE
GCST006061 0.0073 0.3116 537409 outcome TRUE reported 12514_16_METTL1_METTL1
METTL1 chr2 27519736 0.008519 3.535e-09 35364 TRUE reported
textfile 2 1 TRUE
622 rs10096633 T C T C -0.0995 0.0162 0.09986 NA FALSE FALSE FALSE
GCST006061 0.0106 0.1274 537409 outcome TRUE reported
2943_5_CYP3A4_Cytochrome_P450_3A4 CYP3A4 chr8 19973410 0.013791 5.391e-13
35368 TRUE reported textfile 2 1 TRUE
623 rs112875651 A G A G -0.0583 -0.0016 0.40253 NA FALSE FALSE FALSE
GCST006061 0.0076 0.8301 537409 outcome TRUE reported
2943_5_CYP3A4_Cytochrome_P450_3A4 CYP3A4 chr8 125494452 0.008527 8.08e-12
35367 TRUE reported textfile 2 1 TRUE
624 rs11591147 T G T G -0.2317 0.0511 0.01208 NA FALSE FALSE FALSE
GCST006061 0.0356 0.1511 537409 outcome TRUE reported
2943_5_CYP3A4_Cytochrome_P450_3A4 CYP3A4 chr1 55039974 0.03926 3.599e-09
35363 TRUE reported textfile 2 1 TRUE
625 rs1707652 T C T C 0.0599 0.0081 0.3102 NA FALSE FALSE FALSE
GCST006061 0.0074 0.2701 537409 outcome TRUE reported
2943_5_CYP3A4_Cytochrome_P450_3A4 CYP3A4 chr3 165761011 0.008942 2.104e-11
35339 TRUE reported textfile 2 1 TRUE
626 rs3764261 A C A C -0.0635 0.0132 0.32148 NA FALSE FALSE FALSE
GCST006061 0.0076 0.08361 537409 outcome TRUE reported
2943_5_CYP3A4_Cytochrome_P450_3A4 CYP3A4 chr16 56959412 0.008872 8.216e-13
35369 TRUE reported textfile 2 1 TRUE
627 rs4665972 C T C T -0.0631 -0.0046 0.33903 NA FALSE FALSE FALSE
GCST006061 0.0075 0.5416 537409 outcome TRUE reported
2943_5_CYP3A4_Cytochrome_P450_3A4 CYP3A4 chr2 27375230 0.008787 6.922e-13
35374 TRUE reported textfile 2 1 TRUE
628 rs602633 G T G T 0.0674 -0.0162 0.20391 NA FALSE FALSE FALSE
GCST006061 0.0088 0.06452 537409 outcome TRUE reported

2943_5_CYP3A4_Cytochrome_P450_3A4 CYP3A4 chr1 109278889 0.010336 6.975e-11 35344 TRUE reported textfile 2 1 TRUE
629 rs62122484 A G A G 0.095 0.0026 0.16998 NA FALSE FALSE FALSE GCST006061 0.0095 0.7806 537409 outcome TRUE reported 2943_5_CYP3A4_Cytochrome_P450_3A4 CYP3A4 chr2 21000700 0.011051 8.209e-18 35373 TRUE reported textfile 2 1 TRUE
630 rs6511720 T G T G -0.1105 0.0068 0.08895 NA FALSE FALSE FALSE GCST006061 0.0119 0.5645 537409 outcome TRUE reported 2943_5_CYP3A4_Cytochrome_P450_3A4 CYP3A4 chr19 11091630 0.014555 3.158e-14 35379 TRUE reported textfile 2 1 TRUE
631 rs7412 T C T C -0.1932 -0.0027 0.05941 NA FALSE FALSE FALSE GCST006061 0.0138 0.8459 537409 outcome TRUE reported 2943_5_CYP3A4_Cytochrome_P450_3A4 CYP3A4 chr19 44908822 0.01752 2.812e-28 35367 TRUE reported textfile 2 1 TRUE
632 rs10096633 T C T C -0.1588 0.0162 0.09986 NA FALSE FALSE FALSE GCST006061 0.0106 0.1274 537409 outcome TRUE reported 8086_49_ITM2B_ITM2B ITM2B chr8 19973410 0.013745 7.076e-31 35368 TRUE reported textfile 2 1 TRUE
633 rs10189499 C T C T 0.047 -0.0061 0.37801 NA FALSE FALSE FALSE GCST006061 0.0074 0.4067 537409 outcome TRUE reported 8086_49_ITM2B_ITM2B ITM2B chr2 202885326 0.008581 4.314e-08 35364 TRUE reported textfile 2 1 TRUE
634 rs111266377 T G T G 0.1884 0.037 0.02883 NA FALSE FALSE FALSE GCST006061 0.0258 0.1519 537409 outcome TRUE reported 8086_49_ITM2B_ITM2B ITM2B chr3 167239870 0.024745 2.663e-14 35354 TRUE reported textfile 2 1 TRUE
635 rs111696008 A G A G -0.2543 -0.0259 0.02443 NA FALSE FALSE FALSE GCST006061 0.0401 0.519 537409 outcome TRUE reported 8086_49_ITM2B_ITM2B ITM2B chr2 216622986 0.027557 2.754e-20 35359 TRUE reported textfile 2 1 TRUE
636 rs113414093 A G A G 0.0817 0.0533 0.08768 NA FALSE FALSE FALSE GCST006061 0.0237 0.02426 537409 outcome TRUE reported 8086_49_ITM2B_ITM2B ITM2B chr2 218994449 0.01481 3.458e-08 35363 TRUE reported textfile 2 1 TRUE
637 rs116843064 A G A G -0.2055 0.0449 0.02426 NA FALSE FALSE FALSE GCST006061 0.0321 0.1613 537409 outcome TRUE reported 8086_49_ITM2B_ITM2B ITM2B chr19 8364439 0.02662 1.165e-14 35375 TRUE reported textfile 2 1 TRUE
638 rs12485327 T G T G 0.0911 0.0109 0.07334 NA FALSE FALSE FALSE GCST006061 0.0118 0.3548 537409 outcome TRUE reported 8086_49_ITM2B_ITM2B ITM2B chr3 162196930 0.016079 1.463e-08 35340 TRUE reported textfile 2 1 TRUE
639 rs1321257 A G A G -0.0493 -0.003 0.36703 NA FALSE FALSE FALSE GCST006061 0.0073 0.6839 537409 outcome TRUE reported 8086_49_ITM2B_ITM2B ITM2B chr1 230169566 0.008619 1.065e-08 35356 TRUE reported textfile 2 1 TRUE
640 rs141469619 G A G A 0.2022 -0.0376 0.01201 NA FALSE FALSE FALSE GCST006061 0.0531 0.4786 537409 outcome TRUE reported 8086_49_ITM2B_ITM2B ITM2B chr11 116843577 0.036933 4.38e-08 35377 TRUE reported textfile 2 1 TRUE
641 rs144202860 A G A G -0.248 -0.0629 0.01274 NA FALSE FALSE FALSE GCST006061 0.0538 0.2423 537409 outcome TRUE reported 8086_49_ITM2B_ITM2B ITM2B chr2 216625740 0.036902 1.81e-11 35359 TRUE reported textfile 2 1 TRUE
642 rs148077724 G A G A 0.2157 -0.0095 0.02611 NA FALSE FALSE FALSE GCST006061 0.0214 0.6574 537409 outcome TRUE reported 8086_49_ITM2B_ITM2B ITM2B chr3 165952844 0.025819 6.58e-17 35340 TRUE reported textfile 2 1 TRUE
643 rs1618069 G A G A 0.0649 -0.02 0.24647 NA FALSE FALSE FALSE GCST006061 0.0083 0.01621 537409 outcome TRUE reported 8086_49_ITM2B_ITM2B ITM2B chr3 136308303 0.0097 2.219e-11 35361 TRUE reported textfile 2 1 TRUE
644 rs2954038 A C A C -0.0695 0.0046 0.31787 NA FALSE FALSE FALSE GCST006061 0.0078 0.5552 537409 outcome TRUE reported 8086_49_ITM2B_ITM2B ITM2B chr8 125495147 0.008884 5.149e-15 35367 TRUE reported textfile 2 1 TRUE

645 rs3816117 C T C T -0.0942 0.0137 0.49421 NA FALSE FALSE FALSE
GCST006061 0.0071 0.05455 537409 outcome TRUE reported 8086_49_ITM2B_ITM2B
ITM2B chr16 56962246 0.008276 5.084e-30 35369 TRUE reported
textfile 2 1 TRUE
646 rs520354 G A G A 0.1364 -0.0013 0.48258 NA FALSE FALSE FALSE
GCST006061 0.0073 0.8551 537409 outcome TRUE reported 8086_49_ITM2B_ITM2B
ITM2B chr2 21036740 0.008298 1.012e-60 35373 TRUE reported
textfile 2 1 TRUE
647 rs6445035 A G A G 0.2713 0.0154 0.1904 NA FALSE FALSE FALSE
GCST006061 0.0089 0.08256 537409 outcome TRUE reported 8086_49_ITM2B_ITM2B
ITM2B chr3 165762312 0.010484 1.175e-147 35339 TRUE reported
textfile 2 1 TRUE
648 rs660240 C T C T 0.0679 -0.0136 0.205 NA FALSE FALSE FALSE
GCST006061 0.0088 0.1214 537409 outcome TRUE reported 8086_49_ITM2B_ITM2B
ITM2B chr1 109275216 0.010284 4.04e-11 35345 TRUE reported
textfile 2 1 TRUE
649 rs7412 T C T C -0.2061 -0.0027 0.05941 NA FALSE FALSE FALSE GCST006061
0.0138 0.8459 537409 outcome TRUE reported 8086_49_ITM2B_ITM2B ITM2B chr19
44908822 0.017494 4.866e-32 35367 TRUE reported textfile 2 1 TRUE
650 rs1010163 T C T C -0.066 -7.00E-04 0.15673 NA FALSE FALSE FALSE
GCST006061 0.0093 0.9371 537409 outcome TRUE reported 14153_8_EFNA3_Ephrin_A3
EFNA3 chr3 24430558 0.011008 2.029e-09 35359 TRUE reported
textfile 2 1 TRUE
651 rs10922098 T C T C -0.0564 -0.009 0.40011 NA FALSE FALSE FALSE
GCST006061 0.0075 0.2275 537409 outcome TRUE reported 14153_8_EFNA3_Ephrin_A3
EFNA3 chr1 196695521 0.008206 6.302e-12 35338 TRUE reported
textfile 2 1 TRUE
652 rs67333363 G A G A -0.0914 -0.0024 0.08685 NA FALSE FALSE FALSE
GCST006061 0.0151 0.8729 537409 outcome TRUE reported 14153_8_EFNA3_Ephrin_A3
EFNA3 chr1 155085570 0.014312 1.698e-10 35365 TRUE reported
textfile 2 1 TRUE
653 rs117325986 T C T C 0.1553 2e-04 0.03741 NA FALSE FALSE FALSE
GCST006061 0.0384 0.9948 537409 outcome TRUE reported
13676_46_INHBB_Inhibin_bB_chain INHBB chr12 56603971 0.021519 5.319e-13
35369 TRUE reported textfile 2 1 TRUE
654 rs17608972 A G A G 0.0944 0.0158 0.07992 NA FALSE FALSE FALSE
GCST006061 0.0108 0.1458 537409 outcome TRUE reported
13676_46_INHBB_Inhibin_bB_chain INHBB chr2 119673087 0.015375 8.269e-10
35361 TRUE reported textfile 2 1 TRUE
655 rs1880544 C T C T -0.1198 0.0233 0.14217 NA FALSE FALSE FALSE
GCST006061 0.011 0.03353 537409 outcome TRUE reported
13676_46_INHBB_Inhibin_bB_chain INHBB chr2 120465218 0.011955 1.23e-23
35357 TRUE reported textfile 2 1 TRUE
656 rs2311597 G A G A -0.2705 -0.0065 0.42374 NA FALSE FALSE FALSE
GCST006061 0.0073 0.3704 537409 outcome TRUE reported
13676_46_INHBB_Inhibin_bB_chain INHBB chr2 120548195 0.00836 1e-200 35358
TRUE reported textfile 2 1 TRUE
657 rs28416518 T C T C -0.109 -0.0128 0.06424 NA FALSE FALSE FALSE
GCST006061 0.0143 0.3714 537409 outcome TRUE reported
13676_46_INHBB_Inhibin_bB_chain INHBB chr2 120544823 0.016985 1.387e-10
35358 TRUE reported textfile 2 1 TRUE
658 rs3741414 T C T C 0.2037 -0.0052 0.31233 NA FALSE FALSE FALSE
GCST006061 0.0087 0.548 537409 outcome TRUE reported
13676_46_INHBB_Inhibin_bB_chain INHBB chr12 57450266 0.008808 2.495e-118
35367 TRUE reported textfile 2 1 TRUE
659 rs4738679 A G A G 0.0696 0.005 0.31095 NA FALSE FALSE FALSE
GCST006061 0.0076 0.5108 537409 outcome TRUE reported
13676_46_INHBB_Inhibin_bB_chain INHBB chr8 58457761 0.008875 4.415e-15
35361 TRUE reported textfile 2 1 TRUE
660 rs10107182 T C T C 0.0897 0.0044 0.30751 NA FALSE FALSE FALSE
GCST006061 0.0076 0.5641 537409 outcome TRUE reported 13724_27_FGF19_FGF_19
FGF19 chr8 58480178 0.00854 8.363e-26 35361 TRUE reported textfile
2 1 TRUE
661 rs1260326 C T C T -0.051 -0.0069 0.34132 NA FALSE FALSE FALSE
GCST006061 0.0073 0.3401 537409 outcome TRUE reported 13724_27_FGF19_FGF_19
FGF19 chr2 27508073 0.008381 1.165e-09 35373 TRUE reported

textfile 2 1 TRUE
662 rs3795054 A G A G 0.0915 -0.0125 0.07719 NA FALSE FALSE FALSE
GCST006061 0.0149 0.4027 537409 outcome TRUE reported 13724_27_FGF19_FGF_19
FGF19 chr19 49100573 0.015056 1.223e-09 35378 TRUE reported
textfile 2 1 TRUE
663 rs492602 G A G A -0.2344 0.0055 0.39439 NA FALSE FALSE FALSE
GCST006061 0.0076 0.4635 537409 outcome TRUE reported 13724_27_FGF19_FGF_19
FGF19 chr19 48703160 0.00806 6.135e-186 35377 TRUE reported textfile
2 1 TRUE
664 rs708686 T C T C 0.0923 -4.00E-04 0.23081 NA FALSE FALSE FALSE
GCST006061 0.0081 0.9647 537409 outcome TRUE reported 13724_27_FGF19_FGF_19
FGF19 chr19 5840608 0.009398 9.123e-23 35362 TRUE reported textfile
2 1 TRUE
665 rs10102164 A G A G -0.049 -0.0109 0.244 NA FALSE FALSE FALSE
GCST006061 0.0087 0.2115 537409 outcome TRUE reported
5363_51_SEMA3E_Semaphorin_3E SEMA3E chr8 54509054 0.00817 2.006e-09 35355
TRUE reported textfile 2 1 TRUE
666 rs112429006 T C T C -0.6775 -0.0448 0.018 NA FALSE FALSE FALSE
GCST006061 0.0408 0.2724 537409 outcome TRUE reported
5363_51_SEMA3E_Semaphorin_3E SEMA3E chr7 82819130 0.030369 3.002e-110
35358 TRUE reported textfile 2 1 TRUE
667 rs118161729 G A G A -0.5813 0.0016 0.09215 NA FALSE FALSE FALSE
GCST006061 0.0147 0.915 537409 outcome TRUE reported
5363_51_SEMA3E_Semaphorin_3E SEMA3E chr7 83324282 0.008924 1e-200 35352
TRUE reported textfile 2 1 TRUE
668 rs12563740 C T C T 0.0399 0.0176 0.46895 NA FALSE FALSE FALSE
GCST006061 0.0071 0.01386 537409 outcome TRUE reported
5363_51_SEMA3E_Semaphorin_3E SEMA3E chr1 183875625 0.007074 1.7e-08 35359
TRUE reported textfile 2 1 TRUE
669 rs141830128 C T C T -0.2289 0.0093 0.02337 NA FALSE FALSE FALSE
GCST006061 0.0357 0.794 537409 outcome TRUE reported
5363_51_SEMA3E_Semaphorin_3E SEMA3E chr7 93654187 0.027105 3.045e-17
35357 TRUE reported textfile 2 1 TRUE
670 rs148081846 A G A G -0.2439 -0.0128 0.02596 NA FALSE FALSE FALSE
GCST006061 0.0375 0.7332 537409 outcome TRUE reported
5363_51_SEMA3E_Semaphorin_3E SEMA3E chr7 84214832 0.025067 2.248e-22
35347 TRUE reported textfile 2 1 TRUE
671 rs149394237 A G A G -0.2569 -0.1057 0.01876 NA FALSE FALSE FALSE
GCST006061 0.045 0.01882 537409 outcome TRUE reported
5363_51_SEMA3E_Semaphorin_3E SEMA3E chr7 84279393 0.030462 3.356e-17
35348 TRUE reported textfile 2 1 TRUE
672 rs149656509 T C T C -0.5382 -0.0148 0.0233 NA FALSE FALSE FALSE
GCST006061 0.0567 0.7939 537409 outcome TRUE reported
5363_51_SEMA3E_Semaphorin_3E SEMA3E chr7 88117754 0.026208 1.028e-93
35352 TRUE reported textfile 2 1 TRUE
673 rs17284837 G T G T -0.7622 0.0511 0.02426 NA FALSE FALSE FALSE
GCST006061 0.0435 0.2408 537409 outcome TRUE reported
5363_51_SEMA3E_Semaphorin_3E SEMA3E chr7 82813929 0.025085 1e-200 35358
TRUE reported textfile 2 1 TRUE
674 rs1733609 G A G A 0.1611 0.003 0.26295 NA FALSE FALSE FALSE
GCST006061 0.0089 0.7345 537409 outcome TRUE reported
5363_51_SEMA3E_Semaphorin_3E SEMA3E chr7 81907059 0.009186 7.308e-69
35347 TRUE reported textfile 2 1 TRUE
675 rs34779581 G A G A -0.3385 -0.0054 0.0578 NA FALSE FALSE FALSE
GCST006061 0.0157 0.7312 537409 outcome TRUE reported
5363_51_SEMA3E_Semaphorin_3E SEMA3E chr7 84363575 0.017269 1.513e-85
35346 TRUE reported textfile 2 1 TRUE
676 rs3761986 C T C T 0.0469 0.009 0.38748 NA FALSE FALSE FALSE
GCST006061 0.0074 0.2222 537409 outcome TRUE reported
5363_51_SEMA3E_Semaphorin_3E SEMA3E chr6 6680277 0.007271 1.114e-10 35375
TRUE reported textfile 2 1 TRUE
677 rs4577905 A G A G 0.401 -0.0104 0.05959 NA FALSE FALSE FALSE
GCST006061 0.0193 0.5902 537409 outcome TRUE reported
5363_51_SEMA3E_Semaphorin_3E SEMA3E chr7 83153419 0.016936 6.209e-124
35351 TRUE reported textfile 2 1 TRUE
678 rs79983584 T C T C 0.1303 0.0112 0.08155 NA FALSE FALSE FALSE

GCST006061 0.0149 0.451 537409 outcome TRUE reported
5363_51_SEMA3E_Semaphorin_3E SEMA3E chr7 85428659 0.014875 1.957e-18
35349 TRUE reported textfile 2 1 TRUE
679 rs9389269 C T C T 0.0646 -0.0053 0.27891 NA FALSE FALSE FALSE
GCST006061 0.0079 0.508 537409 outcome TRUE reported
5363_51_SEMA3E_Semaphorin_3E SEMA3E chr6 135106021 0.007872 2.286e-16
35372 TRUE reported textfile 2 1 TRUE
680 rs10102164 A G A G 0.0683 -0.0109 0.244 NA FALSE FALSE FALSE
GCST006061 0.0087 0.2115 537409 outcome TRUE reported 19492_5_KAAG1_KAAG1
KAAG1 chr8 54509054 0.009546 8.37e-13 35355 TRUE reported
textfile 2 1 TRUE
681 rs113855303 A C A C -0.1933 -0.039 0.04252 NA FALSE FALSE FALSE
GCST006061 0.0247 0.1143 537409 outcome TRUE reported 19492_5_KAAG1_KAAG1
KAAG1 chr1 11464306 0.021006 3.51e-20 35387 TRUE reported
textfile 2 1 TRUE
682 rs114159428 T C T C -0.4099 -0.0124 0.01002 NA FALSE FALSE FALSE
GCST006061 0.0427 0.7706 537409 outcome TRUE reported 19492_5_KAAG1_KAAG1
KAAG1 chr1 10144401 0.044054 1.347e-20 35391 TRUE reported
textfile 2 1 TRUE
683 rs115102542 C T C T -1.1903 -0.0025 0.03569 NA FALSE FALSE FALSE
GCST006061 0.0299 0.934 537409 outcome TRUE reported 19492_5_KAAG1_KAAG1
KAAG1 chr1 11036895 0.01675 1e-200 35388 TRUE reported textfile 2
1 TRUE
684 rs115388123 A G A G -0.2961 -0.029 0.01147 NA FALSE FALSE FALSE
GCST006061 0.0417 0.4866 537409 outcome TRUE reported 19492_5_KAAG1_KAAG1
KAAG1 chr1 11921245 0.040952 4.818e-13 35389 TRUE reported
textfile 2 1 TRUE
685 rs11579159 A G A G 0.1815 0.0156 0.01805 NA FALSE FALSE FALSE
GCST006061 0.034 0.6464 537409 outcome TRUE reported 19492_5_KAAG1_KAAG1
KAAG1 chr1 10751231 0.032445 2.217e-08 35386 TRUE reported
textfile 2 1 TRUE
686 rs1260326 C T C T -0.0528 -0.0069 0.34132 NA FALSE FALSE FALSE
GCST006061 0.0073 0.3401 537409 outcome TRUE reported 19492_5_KAAG1_KAAG1
KAAG1 chr2 27508073 0.008726 1.439e-09 35373 TRUE reported
textfile 2 1 TRUE
687 rs12748809 A G A G 0.141 -0.0298 0.0541 NA FALSE FALSE FALSE
GCST006061 0.0323 0.3557 537409 outcome TRUE reported 19492_5_KAAG1_KAAG1
KAAG1 chr1 11400312 0.018678 4.389e-14 35389 TRUE reported
textfile 2 1 TRUE
688 rs138200736 A G A G -0.7701 0.0029 0.01808 NA FALSE FALSE FALSE
GCST006061 0.0385 0.94 537409 outcome TRUE reported 19492_5_KAAG1_KAAG1
KAAG1 chr1 11307205 0.031598 3.398e-131 35388 TRUE reported
textfile 2 1 TRUE
689 rs148414479 A G A G 0.1628 0.0476 0.0474 NA FALSE FALSE FALSE
GCST006061 0.0246 0.05274 537409 outcome TRUE reported 19492_5_KAAG1_KAAG1
KAAG1 chr1 11022862 0.020085 5.254e-16 35388 TRUE reported
textfile 2 1 TRUE
690 rs1486236 A C A C -0.0489 0.0029 0.34055 NA FALSE FALSE FALSE
GCST006061 0.0076 0.7053 537409 outcome TRUE reported 19492_5_KAAG1_KAAG1
KAAG1 chr2 179874723 0.008681 1.77e-08 35351 TRUE reported
textfile 2 1 TRUE
691 rs150814911 T C T C -0.4129 -0.0265 0.01353 NA FALSE FALSE FALSE
GCST006061 0.0233 0.256 537409 outcome TRUE reported 19492_5_KAAG1_KAAG1
KAAG1 chr1 27360610 0.037133 1.01e-28 35368 TRUE reported
textfile 2 1 TRUE
692 rs1800450 T C T C 0.1896 -0.0064 0.13736 NA FALSE FALSE FALSE
GCST006061 0.01 0.5249 537409 outcome TRUE reported 19492_5_KAAG1_KAAG1
KAAG1 chr10 52771475 0.011868 1.897e-57 35361 TRUE reported
textfile 2 1 TRUE
693 rs215225 G A G A -0.0728 -0.0192 0.43203 NA FALSE FALSE FALSE
GCST006061 0.0072 0.008012 537409 outcome TRUE reported 19492_5_KAAG1_KAAG1
KAAG1 chr12 481379 0.008322 2.167e-18 35311 TRUE reported textfile
2 1 TRUE
694 rs2781083 G A G A -0.2694 0.023 0.01629 NA FALSE FALSE FALSE
GCST006061 0.0309 0.4563 537409 outcome TRUE reported 19492_5_KAAG1_KAAG1
KAAG1 chr1 8935492 0.033324 6.257e-16 35389 TRUE reported textfile

2 1 TRUE
695 rs3774282 A G A G 0.0542 -5.00E-04 0.28575 NA FALSE FALSE FALSE
GCST006061 0.0076 0.9441 537409 outcome TRUE reported 19492_5_KAAG1_KAAG1
KAAG1 chr3 187264420 0.009137 2.999e-09 35368 TRUE reported
textfile 2 1 TRUE
696 rs4379693 T C T C 0.202 -0.0031 0.01406 NA FALSE FALSE FALSE
GCST006061 0.0252 0.9019 537409 outcome TRUE reported 19492_5_KAAG1_KAAG1
KAAG1 chr1 27962473 0.0359 1.836e-08 35371 TRUE reported textfile
2 1 TRUE
697 rs56410663 T C T C 0.1852 -0.0967 0.02688 NA FALSE FALSE FALSE
GCST006061 0.0326 0.003016 537409 outcome TRUE reported 19492_5_KAAG1_KAAG1
KAAG1 chr1 11212560 0.025706 5.821e-13 35389 TRUE reported
textfile 2 1 TRUE
698 rs62467349 T C T C -0.0649 -0.0056 0.15309 NA FALSE FALSE FALSE
GCST006061 0.0108 0.6067 537409 outcome TRUE reported 19492_5_KAAG1_KAAG1
KAAG1 chr7 95317483 0.011449 1.441e-08 35349 TRUE reported
textfile 2 1 TRUE
699 rs6542680 T C T C -0.0634 -0.0012 0.18362 NA FALSE FALSE FALSE
GCST006061 0.0091 0.8935 537409 outcome TRUE reported 19492_5_KAAG1_KAAG1
KAAG1 chr2 3592552 0.010656 2.687e-09 35332 TRUE reported textfile
2 1 TRUE
700 rs698088 G A G A -0.1134 -0.0064 0.36622 NA FALSE FALSE FALSE
GCST006061 0.0077 0.4066 537409 outcome TRUE reported 19492_5_KAAG1_KAAG1
KAAG1 chr3 187241180 0.008538 2.941e-40 35369 TRUE reported
textfile 2 1 TRUE
701 rs73167083 A C A C 0.0731 0.0036 0.24974 NA FALSE FALSE FALSE
GCST006061 0.0079 0.6452 537409 outcome TRUE reported 19492_5_KAAG1_KAAG1
KAAG1 chr3 165984008 0.009534 1.752e-14 35341 TRUE reported
textfile 2 1 TRUE
702 rs77639220 A G A G -0.2278 -0.0642 0.03077 NA FALSE FALSE FALSE
GCST006061 0.0262 0.01435 537409 outcome TRUE reported 19492_5_KAAG1_KAAG1
KAAG1 chr1 10658320 0.024237 5.517e-21 35390 TRUE reported
textfile 2 1 TRUE
703 rs903107 C T C T 0.0503 0.008 0.42128 NA FALSE FALSE FALSE
GCST006061 0.0075 0.2835 537409 outcome TRUE reported 19492_5_KAAG1_KAAG1
KAAG1 chr17 73065795 0.008333 1.58e-09 35363 TRUE reported
textfile 2 1 TRUE
704 rs10103048 C A C A -0.0589 0.0056 0.38989 NA FALSE FALSE FALSE
GCST006061 0.0072 0.437 537409 outcome TRUE reported 18392_19_MAT2B_MAT2B
MAT2B chr8 129590035 0.008364 1.899e-12 35372 TRUE reported
textfile 2 1 TRUE
705 rs10799536 G A G A 0.0607 -0.0116 0.1903 NA FALSE FALSE FALSE
GCST006061 0.0088 0.1851 537409 outcome TRUE reported 18392_19_MAT2B_MAT2B
MAT2B chr1 229525044 0.010376 4.915e-09 35361 TRUE reported
textfile 2 1 TRUE
706 rs113753171 A G A G -0.2058 -0.0091 0.01453 NA FALSE FALSE FALSE
GCST006061 0.0314 0.7727 537409 outcome TRUE reported 18392_19_MAT2B_MAT2B
MAT2B chr17 57732095 0.034781 3.277e-09 35368 TRUE reported
textfile 2 1 TRUE
707 rs145433937 G A G A -0.4251 -0.1611 0.01131 NA FALSE FALSE FALSE
GCST006061 0.0682 0.01807 537409 outcome TRUE reported 18392_19_MAT2B_MAT2B
MAT2B chr17 59868859 0.038819 6.589e-28 35370 TRUE reported
textfile 2 1 TRUE
708 rs190383363 T C T C -0.3796 0.0078 0.01537 NA FALSE FALSE FALSE
GCST006061 0.0374 0.8348 537409 outcome TRUE reported 18392_19_MAT2B_MAT2B
MAT2B chr17 59235408 0.033883 3.931e-29 35369 TRUE reported
textfile 2 1 TRUE
709 rs214085 T C T C 0.0573 -0.0109 0.43872 NA FALSE FALSE FALSE
GCST006061 0.0073 0.1326 537409 outcome TRUE reported 18392_19_MAT2B_MAT2B
MAT2B chr11 17277109 0.008248 3.732e-12 35371 TRUE reported
textfile 2 1 TRUE
710 rs2305637 T C T C 0.0771 5e-04 0.15762 NA FALSE FALSE FALSE
GCST006061 0.0097 0.961 537409 outcome TRUE reported 18392_19_MAT2B_MAT2B
MAT2B chr3 47004356 0.011125 4.192e-12 35343 TRUE reported
textfile 2 1 TRUE
711 rs6131985 T G T G 0.0485 -3.00E-04 0.46023 NA FALSE FALSE FALSE

GCST006061 0.0073 0.9626 537409 outcome TRUE reported 18392_19_MAT2B_MAT2B
MAT2B chr20 17689784 0.008168 2.893e-09 35376 TRUE reported
textfile 2 1 TRUE
712 rs62083752 T C T C -0.1674 -0.0104 0.12173 NA FALSE FALSE FALSE
GCST006061 0.0133 0.4366 537409 outcome TRUE reported 18392_19_MAT2B_MAT2B
MAT2B chr17 58290785 0.012491 5.883e-41 35369 TRUE reported
textfile 2 1 TRUE
713 rs744233 A G A G 0.0543 -0.0028 0.31736 NA FALSE FALSE FALSE
GCST006061 0.0077 0.721 537409 outcome TRUE reported 18392_19_MAT2B_MAT2B
MAT2B chr3 126530422 0.008795 6.649e-10 35371 TRUE reported
textfile 2 1 TRUE
714 rs76428106 C T C T 0.2145 -0.0446 0.01359 NA FALSE FALSE FALSE
GCST006061 0.0438 0.3085 537409 outcome TRUE reported 18392_19_MAT2B_MAT2B
MAT2B chr13 28029870 0.035494 1.51e-09 35363 TRUE reported
textfile 2 1 TRUE
715 rs10103048 C A C A -0.072 0.0056 0.38989 NA FALSE FALSE FALSE
GCST006061 0.0072 0.437 537409 outcome TRUE reported 3046_31_RETN_resistin
RETN chr8 129590035 0.008819 3.239e-16 35372 TRUE reported
textfile 2 1 TRUE
716 rs11078930 T C T C -0.105 0.0266 0.37546 NA FALSE FALSE FALSE
GCST006061 0.0074 0.0003261 537409 outcome TRUE reported
3046_31_RETN_resistin RETN chr17 39985702 0.008895 3.706e-32 35379
TRUE reported textfile 2 1 TRUE
717 rs112736139 T C T C -0.0824 -0.0157 0.13453 NA FALSE FALSE FALSE
GCST006061 0.0113 0.1677 537409 outcome TRUE reported 3046_31_RETN_resistin
RETN chr19 7999978 0.012814 1.274e-10 35378 TRUE reported textfile
2 1 TRUE
718 rs1144700 T C T C -0.0668 -0.0052 0.17953 NA FALSE FALSE FALSE
GCST006061 0.0089 0.5579 537409 outcome TRUE reported 3046_31_RETN_resistin
RETN chr6 16744456 0.011326 3.679e-09 35385 TRUE reported
textfile 2 1 TRUE
719 rs117029024 A G A G -0.5646 -0.005 0.0332 NA FALSE FALSE FALSE
GCST006061 0.0356 0.8878 537409 outcome TRUE reported 3046_31_RETN_resistin
RETN chr19 7613602 0.023973 1.209e-122 35373 TRUE reported textfile
2 1 TRUE
720 rs13390874 T C T C 0.0907 -0.0015 0.2456 NA FALSE FALSE FALSE
GCST006061 0.0085 0.8576 537409 outcome TRUE reported 3046_31_RETN_resistin
RETN chr2 43126091 0.010023 1.438e-19 35360 TRUE reported
textfile 2 1 TRUE
721 rs137964046 A G A G -0.5023 -0.002 0.01392 NA FALSE FALSE FALSE
GCST006061 0.0535 0.9697 537409 outcome TRUE reported 3046_31_RETN_resistin
RETN chr19 7258078 0.036159 7.136e-44 35368 TRUE reported textfile
2 1 TRUE
722 rs138604055 A C A C 0.2196 -0.0466 0.01608 NA FALSE FALSE FALSE
GCST006061 0.0411 0.257 537409 outcome TRUE reported 3046_31_RETN_resistin
RETN chr6 144362043 0.03362 6.497e-11 35358 TRUE reported textfile
2 1 TRUE
723 rs139719552 C T C T -0.19 0.0594 0.01912 NA FALSE FALSE FALSE
GCST006061 0.0459 0.1958 537409 outcome TRUE reported 3046_31_RETN_resistin
RETN chr6 130033828 0.032158 3.456e-09 35367 TRUE reported
textfile 2 1 TRUE
724 rs141094656 C T C T 0.1954 0.018 0.02759 NA FALSE FALSE FALSE
GCST006061 0.0394 0.6484 537409 outcome TRUE reported 3046_31_RETN_resistin
RETN chr1 92287779 0.026185 8.496e-14 35352 TRUE reported
textfile 2 1 TRUE
725 rs149110519 T C T C 0.3487 -0.0345 0.04822 NA FALSE FALSE FALSE
GCST006061 0.0217 0.1121 537409 outcome TRUE reported 3046_31_RETN_resistin
RETN chr6 144064640 0.019998 4.371e-68 35361 TRUE reported
textfile 2 1 TRUE
726 rs2074038 T G T G 0.0919 -0.0098 0.0923 NA FALSE FALSE FALSE
GCST006061 0.0113 0.3879 537409 outcome TRUE reported 3046_31_RETN_resistin
RETN chr11 44066439 0.014833 5.807e-10 35377 TRUE reported
textfile 2 1 TRUE
727 rs243596 A G A G -0.0636 -3.00E-04 0.48652 NA FALSE FALSE FALSE
GCST006061 0.0073 0.9695 537409 outcome TRUE reported 3046_31_RETN_resistin
RETN chr21 17784538 0.008632 1.737e-13 35353 TRUE reported

textfile 2 1 TRUE
728 rs3745368 A G A G -0.6658 -0.0188 0.03859 NA FALSE FALSE FALSE
GCST006061 0.0246 0.4442 537409 outcome TRUE reported 3046_31_RETN_resistin
RETN chr19 7670411 0.022532 6.634e-192 35368 TRUE reported textfile
2 1 TRUE
729 rs445 T C T C -0.0885 0.049 0.0895 NA FALSE FALSE FALSE GCST006061
0.0112 1.271e-05 537409 outcome TRUE reported 3046_31_RETN_resistin RETN
chr7 92779056 0.015324 7.681e-09 35347 TRUE reported textfile 2
1 TRUE
730 rs4811689 A G A G -0.0872 0.0026 0.45752 NA FALSE FALSE FALSE
GCST006061 0.0074 0.7269 537409 outcome TRUE reported 3046_31_RETN_resistin
RETN chr20 38311185 0.008654 7.07e-24 35374 TRUE reported
textfile 2 1 TRUE
731 rs10103048 C A C A -0.0553 0.0056 0.38989 NA FALSE FALSE FALSE
GCST006061 0.0072 0.437 537409 outcome TRUE reported 15481_45_CAMP_LL_37
CAMP chr8 129590035 0.008479 6.937e-11 35372 TRUE reported
textfile 2 1 TRUE
732 rs1144700 T C T C -0.0638 -0.0052 0.17953 NA FALSE FALSE FALSE
GCST006061 0.0089 0.5579 537409 outcome TRUE reported 15481_45_CAMP_LL_37
CAMP chr6 16744456 0.010888 4.64e-09 35385 TRUE reported
textfile 2 1 TRUE
733 rs12180 A G A G -0.0782 0 0.30227 NA FALSE FALSE FALSE GCST006061
0.0077 0.9981 537409 outcome TRUE reported 15481_45_CAMP_LL_37 CAMP chr11
134246231 0.009062 6.173e-18 35153 TRUE reported textfile 2 1 TRUE
734 rs12444012 A G A G 0.0462 0.0113 0.43711 NA FALSE FALSE FALSE
GCST006061 0.0072 0.119 537409 outcome TRUE reported 15481_45_CAMP_LL_37
CAMP chr16 56967526 0.008337 2.999e-08 35369 TRUE reported
textfile 2 1 TRUE
735 rs1532085 G A G A -0.0544 0.0148 0.41111 NA FALSE FALSE FALSE
GCST006061 0.0073 0.04221 537409 outcome TRUE reported 15481_45_CAMP_LL_37
CAMP chr15 58391167 0.008388 8.844e-11 35364 TRUE reported
textfile 2 1 TRUE
736 rs17019087 C T C T -0.0595 -0.0023 0.34772 NA FALSE FALSE FALSE
GCST006061 0.0073 0.7478 537409 outcome TRUE reported 15481_45_CAMP_LL_37
CAMP chr2 36556456 0.008793 1.317e-11 35362 TRUE reported
textfile 2 1 TRUE
737 rs1707652 T C T C 0.1327 0.0081 0.3102 NA FALSE FALSE FALSE
GCST006061 0.0074 0.2701 537409 outcome TRUE reported 15481_45_CAMP_LL_37
CAMP chr3 165761011 0.008943 8.354e-50 35339 TRUE reported
textfile 2 1 TRUE
738 rs17703558 C T C T 0.4189 0.0379 0.04157 NA FALSE FALSE FALSE
GCST006061 0.0242 0.1172 537409 outcome TRUE reported 15481_45_CAMP_LL_37
CAMP chr17 69226872 0.021897 1.412e-81 35371 TRUE reported
textfile 2 1 TRUE
739 rs2041037 T C T C -0.0689 0.0054 0.21006 NA FALSE FALSE FALSE
GCST006061 0.0086 0.527 537409 outcome TRUE reported 15481_45_CAMP_LL_37
CAMP chr17 71249048 0.010635 9.25e-11 35368 TRUE reported
textfile 2 1 TRUE
740 rs2074038 T G T G 0.2484 -0.0098 0.0923 NA FALSE FALSE FALSE
GCST006061 0.0113 0.3879 537409 outcome TRUE reported 15481_45_CAMP_LL_37
CAMP chr11 44066439 0.014267 6.803e-68 35377 TRUE reported
textfile 2 1 TRUE
741 rs2521350 A G A G 0.0512 -0.0055 0.41595 NA FALSE FALSE FALSE
GCST006061 0.0072 0.4467 537409 outcome TRUE reported 15481_45_CAMP_LL_37
CAMP chr17 69508121 0.008737 4.623e-09 35363 TRUE reported
textfile 2 1 TRUE
742 rs2734031 C T C T -0.097 -0.0107 0.12145 NA FALSE FALSE FALSE
GCST006061 0.0114 0.3477 537409 outcome TRUE reported 15481_45_CAMP_LL_37
CAMP chr3 128582547 0.012803 3.557e-14 35363 TRUE reported
textfile 2 1 TRUE
743 rs34216592 A G A G 0.5112 0.0105 0.13279 NA FALSE FALSE FALSE
GCST006061 0.0127 0.4071 537409 outcome TRUE reported 15481_45_CAMP_LL_37
CAMP chr17 68781934 0.008561 1e-200 35370 TRUE reported textfile
2 1 TRUE
744 rs35362691 G T G T 0.116 0.0338 0.0677 NA FALSE FALSE FALSE
GCST006061 0.0152 0.026 537409 outcome TRUE reported 15481_45_CAMP_LL_37

CAMP chr17 67487610 0.017146 1.328e-11 35367 TRUE reported
textfile 2 1 TRUE
745 rs4147974 A C A C -0.1718 -7.00E-04 0.47677 NA FALSE FALSE FALSE
GCST006061 0.0074 0.9223 537409 outcome TRUE reported 15481_45_CAMP_LL_37
CAMP chr17 68930480 0.008685 4.241e-87 35368 TRUE reported
textfile 2 1 TRUE
746 rs4619875 T C T C -0.0512 -0.0138 0.37238 NA FALSE FALSE FALSE
GCST006061 0.0072 0.0568 537409 outcome TRUE reported 15481_45_CAMP_LL_37
CAMP chr4 152779978 0.008567 2.285e-09 35377 TRUE reported
textfile 2 1 TRUE
747 rs4657179 G T G T 0.0684 0.0207 0.16816 NA FALSE FALSE FALSE
GCST006061 0.0094 0.02808 537409 outcome TRUE reported 15481_45_CAMP_LL_37
CAMP chr1 162248149 0.011119 7.669e-10 35358 TRUE reported
textfile 2 1 TRUE
748 rs55771110 A G A G 0.109 0.0077 0.14938 NA FALSE FALSE FALSE
GCST006061 0.0102 0.4518 537409 outcome TRUE reported 15481_45_CAMP_LL_37
CAMP chr3 47305227 0.011549 3.807e-21 35343 TRUE reported
textfile 2 1 TRUE
749 rs60642791 G A G A -0.1066 6e-04 0.29722 NA FALSE FALSE FALSE
GCST006061 0.0079 0.9358 537409 outcome TRUE reported 15481_45_CAMP_LL_37
CAMP chr17 68727210 0.009493 2.946e-29 35367 TRUE reported
textfile 2 1 TRUE
750 rs61863078 G A G A -0.0508 0.014 0.46924 NA FALSE FALSE FALSE
GCST006061 0.0074 0.05914 537409 outcome TRUE reported 15481_45_CAMP_LL_37
CAMP chr10 93017351 0.008273 8.216e-10 35373 TRUE reported
textfile 2 1 TRUE
751 rs6507939 C A C A 0.0653 -0.0068 0.16934 NA FALSE FALSE FALSE
GCST006061 0.0101 0.5053 537409 outcome TRUE reported 15481_45_CAMP_LL_37
CAMP chr18 49649891 0.010974 2.677e-09 35365 TRUE reported
textfile 2 1 TRUE
752 rs7502514 G A G A -0.071 0.0189 0.37292 NA FALSE FALSE FALSE
GCST006061 0.0073 0.009352 537409 outcome TRUE reported 15481_45_CAMP_LL_37
CAMP chr17 40032591 0.008966 2.406e-15 35378 TRUE reported
textfile 2 1 TRUE
753 rs7518497 C A C A -0.0631 -0.0248 0.34836 NA FALSE FALSE FALSE
GCST006061 0.0076 0.001156 537409 outcome TRUE reported 15481_45_CAMP_LL_37
CAMP chr1 62712025 0.008697 4.015e-13 35357 TRUE reported
textfile 2 1 TRUE
754 rs9840812 T C T C 0.0563 -0.0222 0.23118 NA FALSE FALSE FALSE
GCST006061 0.0086 0.009709 537409 outcome TRUE reported 15481_45_CAMP_LL_37
CAMP chr3 136124320 0.009896 1.277e-08 35361 TRUE reported
textfile 2 1 TRUE
755 rs1010366 C T C T -0.0573 0 0.27123 NA FALSE FALSE FALSE GCST006061
0.0076 0.9962 537409 outcome TRUE reported 5066_134_CD300C_CLM6 CD300C
chr7 7190195 0.0092 4.71e-10 35364 TRUE reported textfile 2 1 TRUE
756 rs11105271 T C T C -0.2787 0.0104 0.30691 NA FALSE FALSE FALSE
GCST006061 0.0077 0.1801 537409 outcome TRUE reported 5066_134_CD300C_CLM6
CD300C chr12 89385419 0.00887 1e-200 35357 TRUE reported textfile 2
1 TRUE
757 rs11230289 T C T C 0.0608 -0.003 0.27375 NA FALSE FALSE FALSE
GCST006061 0.008 0.7093 537409 outcome TRUE reported 5066_134_CD300C_CLM6
CD300C chr11 60366513 0.009172 3.377e-11 35374 TRUE reported
textfile 2 1 TRUE
758 rs117027254 T G T G -0.3839 -0.0721 0.01691 NA FALSE FALSE FALSE
GCST006061 0.0546 0.1865 537409 outcome TRUE reported 5066_134_CD300C_CLM6
CD300C chr17 74437210 0.0313 1.39e-34 35374 TRUE reported textfile
2 1 TRUE
759 rs11888817 C T C T 0.1406 0.0137 0.32877 NA FALSE FALSE FALSE
GCST006061 0.0077 0.07488 537409 outcome TRUE reported 5066_134_CD300C_CLM6
CD300C chr2 62303845 0.008686 6.272e-59 35352 TRUE reported
textfile 2 1 TRUE
760 rs13205436 C T C T 0.1097 -0.0345 0.10488 NA FALSE FALSE FALSE
GCST006061 0.013 0.008041 537409 outcome TRUE reported
5066_134_CD300C_CLM6 CD300C chr6 95586174 0.013307 1.669e-16 35343
TRUE reported textfile 2 1 TRUE
761 rs139524107 C A C A -0.1715 -0.0247 0.02794 NA FALSE FALSE FALSE

GCST006061 0.0395 0.5321 537409 outcome TRUE reported 5066_134_CD300C_CLM6
CD300C chr12 90535309 0.025186 9.804e-12 35359 TRUE reported
textfile 2 1 TRUE
762 rs143501783 C T C T 0.2614 -0.0333 0.01116 NA FALSE FALSE FALSE
GCST006061 0.0443 0.4525 537409 outcome TRUE reported 5066_134_CD300C_CLM6
CD300C chr17 7897001 0.03751 3.196e-12 35373 TRUE reported textfile 2
1 TRUE
763 rs145767181 G A G A -0.3434 0.0519 0.01163 NA FALSE FALSE FALSE
GCST006061 0.0404 0.1994 537409 outcome TRUE reported 5066_134_CD300C_CLM6
CD300C chr17 74472902 0.039051 1.448e-18 35376 TRUE reported
textfile 2 1 TRUE
764 rs3184504 C T C T -0.0669 0.0074 0.37862 NA FALSE FALSE FALSE
GCST006061 0.0075 0.3235 537409 outcome TRUE reported 5066_134_CD300C_CLM6
CD300C chr12 111446804 0.008602 7.408e-15 35371 TRUE reported
textfile 2 1 TRUE
765 rs35234681 G A G A 0.1467 0.0099 0.07752 NA FALSE FALSE FALSE
GCST006061 0.0143 0.4894 537409 outcome TRUE reported 5066_134_CD300C_CLM6
CD300C chr3 98990066 0.01542 1.84e-21 35331 TRUE reported textfile
2 1 TRUE
766 rs35415187 C T C T 0.119 0.0274 0.06365 NA FALSE FALSE FALSE
GCST006061 0.019 0.1487 537409 outcome TRUE reported 5066_134_CD300C_CLM6
CD300C chr12 91503553 0.017087 3.303e-12 35357 TRUE reported
textfile 2 1 TRUE
767 rs58440339 T C T C 0.1485 0.0071 0.11302 NA FALSE FALSE FALSE
GCST006061 0.0115 0.5375 537409 outcome TRUE reported 5066_134_CD300C_CLM6
CD300C chr17 74543335 0.012928 1.533e-30 35380 TRUE reported
textfile 2 1 TRUE
768 rs62064540 C T C T -0.2703 -0.0544 0.01416 NA FALSE FALSE FALSE
GCST006061 0.0651 0.4028 537409 outcome TRUE reported 5066_134_CD300C_CLM6
CD300C chr17 74175749 0.034472 4.461e-15 35377 TRUE reported
textfile 2 1 TRUE
769 rs6846475 A C A C 0.0515 0.0018 0.38696 NA FALSE FALSE FALSE
GCST006061 0.0075 0.8127 537409 outcome TRUE reported 5066_134_CD300C_CLM6
CD300C chr4 98870240 0.008429 9.994e-10 35344 TRUE reported
textfile 2 1 TRUE
770 rs72844326 G T G T -0.6071 0.0459 0.04222 NA FALSE FALSE FALSE
GCST006061 0.0177 0.00935 537409 outcome TRUE reported 5066_134_CD300C_CLM6
CD300C chr17 74462952 0.020092 1.447e-200 35377 TRUE reported
textfile 2 1 TRUE
771 rs76081401 A G A G -0.3093 -0.0419 0.01252 NA FALSE FALSE FALSE
GCST006061 0.0656 0.5235 537409 outcome TRUE reported 5066_134_CD300C_CLM6
CD300C chr17 74646399 0.03727 1.051e-16 35380 TRUE reported textfile
2 1 TRUE
772 rs78124338 A G A G 0.1356 -0.0108 0.0281 NA FALSE FALSE FALSE
GCST006061 0.0253 0.6676 537409 outcome TRUE reported 5066_134_CD300C_CLM6
CD300C chr12 90492835 0.024627 3.667e-08 35359 TRUE reported
textfile 2 1 TRUE
773 rs9852537 T C T C -0.1246 -0.0341 0.0361 NA FALSE FALSE FALSE
GCST006061 0.0225 0.1294 537409 outcome TRUE reported 5066_134_CD300C_CLM6
CD300C chr3 98939241 0.02208 1.67e-08 35331 TRUE reported textfile
2 1 TRUE
774 rs10105155 T C T C 0.2156 -0.0227 0.12733 NA FALSE FALSE FALSE
GCST006061 0.0105 0.03132 537409 outcome TRUE reported 7156_2_FUT10_FUT10
FUT10 chr8 32742333 0.011422 1.797e-79 35357 TRUE reported
textfile 2 1 TRUE
775 rs10225433 A G A G -0.0683 0.0085 0.08534 NA FALSE FALSE FALSE
GCST006061 0.0171 0.6212 537409 outcome TRUE reported 7156_2_FUT10_FUT10
FUT10 chr7 28152053 0.012404 3.669e-08 35374 TRUE reported
textfile 2 1 TRUE
776 rs11774682 T C T C 0.1738 -0.0114 0.05428 NA FALSE FALSE FALSE
GCST006061 0.0209 0.5859 537409 outcome TRUE reported 7156_2_FUT10_FUT10
FUT10 chr8 31176759 0.01677 3.626e-25 35360 TRUE reported textfile
2 1 TRUE
777 rs12945886 G A G A 0.0479 0 0.24063 NA FALSE FALSE FALSE GCST006061
0.0094 0.9973 537409 outcome TRUE reported 7156_2_FUT10_FUT10 FUT10 chr17
7169695 0.008179 4.737e-09 35370 TRUE reported textfile 2 1 TRUE

778 rs146637002 T C T C 0.2278 0.0026 0.04562 NA FALSE FALSE FALSE GCST006061 0.0246 0.9171 537409 outcome TRUE reported 7156_2_FUT10_FUT10 FUT10 chr8 33177036 0.018312 1.593e-35 35356 TRUE reported textfile 2 1 TRUE
779 rs189826607 A G A G 0.1542 0.0045 0.02476 NA FALSE FALSE FALSE GCST006061 0.0357 0.9003 537409 outcome TRUE reported 7156_2_FUT10_FUT10 FUT10 chr8 49335576 0.024406 2.649e-10 35353 TRUE reported textfile 2 1 TRUE
780 rs2186296 A G A G 0.2546 -0.0411 0.02175 NA FALSE FALSE FALSE GCST006061 0.0218 0.05962 537409 outcome TRUE reported 7156_2_FUT10_FUT10 FUT10 chr8 36782715 0.026612 1.1e-21 35368 TRUE reported textfile 2 1 TRUE
781 rs2288369 A G A G 0.0512 0.0055 0.25514 NA FALSE FALSE FALSE GCST006061 0.0082 0.5018 537409 outcome TRUE reported 7156_2_FUT10_FUT10 FUT10 chr12 51354542 0.007994 1.507e-10 35370 TRUE reported textfile 2 1 TRUE
782 rs4733407 C T C T 0.3897 -0.0142 0.27664 NA FALSE FALSE FALSE GCST006061 0.008 0.07752 537409 outcome TRUE reported 7156_2_FUT10_FUT10 FUT10 chr8 33208165 0.006119 1e-200 35358 TRUE reported textfile 2 1 TRUE
783 rs635634 C T C T -0.1994 0.0285 0.12995 NA FALSE FALSE FALSE GCST006061 0.0089 0.001351 537409 outcome TRUE reported 7156_2_FUT10_FUT10 FUT10 chr9 133279427 0.01035 1.026e-82 35371 TRUE reported textfile 2 1 TRUE
784 rs73183043 C T C T 0.0626 -0.0063 0.17029 NA FALSE FALSE FALSE GCST006061 0.0088 0.4747 537409 outcome TRUE reported 7156_2_FUT10_FUT10 FUT10 chr12 101803173 0.009179 9.09e-12 35364 TRUE reported textfile 2 1 TRUE
785 rs7838624 C A C A 0.2344 -0.0021 0.44433 NA FALSE FALSE FALSE GCST006061 0.0073 0.7703 537409 outcome TRUE reported 7156_2_FUT10_FUT10 FUT10 chr8 33608880 0.007567 1e-200 35356 TRUE reported textfile 2 1 TRUE
786 rs78930932 T C T C 0.301 0.0093 0.07417 NA FALSE FALSE FALSE GCST006061 0.0177 0.6018 537409 outcome TRUE reported 7156_2_FUT10_FUT10 FUT10 chr8 34956358 0.014211 1.429e-99 35369 TRUE reported textfile 2 1 TRUE
787 rs79558899 G A G A 0.1495 0.0462 0.03159 NA FALSE FALSE FALSE GCST006061 0.0331 0.1626 537409 outcome TRUE reported 7156_2_FUT10_FUT10 FUT10 chr8 36569086 0.021711 5.74e-12 35368 TRUE reported textfile 2 1 TRUE
788 rs10107182 T C T C 0.047 0.0044 0.30751 NA FALSE FALSE FALSE GCST006061 0.0076 0.5641 537409 outcome TRUE reported 15305_7_SCGN_Secretagogin SCGN chr8 58480178 0.008551 3.873e-08 35361 TRUE reported textfile 2 1 TRUE
789 rs11240503 A G A G -0.0638 2e-04 0.32657 NA FALSE FALSE FALSE GCST006061 0.0079 0.9765 537409 outcome TRUE reported 15305_7_SCGN_Secretagogin SCGN chr1 205514945 0.008466 4.861e-14 35373 TRUE reported textfile 2 1 TRUE
790 rs601338 A G A G -0.1136 0.0056 0.39439 NA FALSE FALSE FALSE GCST006061 0.0076 0.4559 537409 outcome TRUE reported 15305_7_SCGN_Secretagogin SCGN chr19 48703417 0.008116 1.607e-44 35377 TRUE reported textfile 2 1 TRUE
791 rs6445035 A G A G 0.0572 0.0154 0.1904 NA FALSE FALSE FALSE GCST006061 0.0089 0.08256 537409 outcome TRUE reported 15305_7_SCGN_Secretagogin SCGN chr3 165762312 0.010048 1.251e-08 35339 TRUE reported textfile 2 1 TRUE
792 rs6859236 A G A G -0.0686 8e-04 0.48811 NA FALSE FALSE FALSE GCST006061 0.0072 0.9104 537409 outcome TRUE reported 15305_7_SCGN_Secretagogin SCGN chr5 151156083 0.007952 6.292e-18 35352 TRUE reported textfile 2 1 TRUE
793 rs7480143 T C T C 0.0837 -0.0224 0.11386 NA FALSE FALSE FALSE GCST006061 0.0108 0.03888 537409 outcome TRUE reported 15305_7_SCGN_Secretagogin SCGN chr11 2203793 0.012548 2.547e-11 35361 TRUE reported textfile 2 1 TRUE
794 rs10107630 T C T C -0.0563 0.0063 0.40285 NA FALSE FALSE FALSE GCST006061 0.0071 0.3806 537409 outcome TRUE reported

7849_3_QPCT_Glutaminyl_cyclase QPCT chr8 129591389 0.008456 2.775e-11
35372 TRUE reported textfile 2 1 TRUE
795 rs10896045 G A G A -0.1546 0.0065 0.31686 NA FALSE FALSE FALSE
GCST006061 0.0077 0.398 537409 outcome TRUE reported
7849_3_QPCT_Glutaminyl_cyclase QPCT chr11 65788053 0.008931 3.877e-67
35366 TRUE reported textfile 2 1 TRUE
796 rs16830188 T C T C -0.2802 0.0318 0.01152 NA FALSE FALSE FALSE
GCST006061 0.0224 0.1559 537409 outcome TRUE reported
7849_3_QPCT_Glutaminyl_cyclase QPCT chr1 24972350 0.039504 1.313e-12
35371 TRUE reported textfile 2 1 TRUE
797 rs182664662 T C T C 0.2137 0.0878 0.01185 NA FALSE FALSE FALSE
GCST006061 0.0611 0.1507 537409 outcome TRUE reported
7849_3_QPCT_Glutaminyl_cyclase QPCT chr2 35949621 0.038745 3.477e-08
35367 TRUE reported textfile 2 1 TRUE
798 rs2227319 A G A G -0.0598 0.0273 0.37661 NA FALSE FALSE FALSE
GCST006061 0.0074 0.0002196 537409 outcome TRUE reported
7849_3_QPCT_Glutaminyl_cyclase QPCT chr17 40014592 0.008555 2.755e-12
35379 TRUE reported textfile 2 1 TRUE
799 rs2518136 C T C T 0.0771 -0.0027 0.45877 NA FALSE FALSE FALSE
GCST006061 0.0071 0.7029 537409 outcome TRUE reported
7849_3_QPCT_Glutaminyl_cyclase QPCT chr3 186620038 0.008341 2.382e-20
35367 TRUE reported textfile 2 1 TRUE
800 rs2659008 G A G A 0.055 -9.00E-04 0.45922 NA FALSE FALSE FALSE
GCST006061 0.0074 0.9067 537409 outcome TRUE reported
7849_3_QPCT_Glutaminyl_cyclase QPCT chr17 81243202 0.008326 3.951e-11
35141 TRUE reported textfile 2 1 TRUE
801 rs2948711 G A G A 0.0491 5e-04 0.46902 NA FALSE FALSE FALSE
GCST006061 0.0076 0.9427 537409 outcome TRUE reported
7849_3_QPCT_Glutaminyl_cyclase QPCT chr10 17734454 0.008293 3.2e-09 35355
TRUE reported textfile 2 1 TRUE
802 rs34562254 A G A G 0.1211 -0.0013 0.07695 NA FALSE FALSE FALSE
GCST006061 0.0109 0.902 537409 outcome TRUE reported
7849_3_QPCT_Glutaminyl_cyclase QPCT chr17 16939677 0.015517 5.97e-15
35357 TRUE reported textfile 2 1 TRUE
803 rs4384764 A G A G 0.2206 -0.0032 0.28728 NA FALSE FALSE FALSE
GCST006061 0.008 0.6916 537409 outcome TRUE reported
7849_3_QPCT_Glutaminyl_cyclase QPCT chr2 37363141 0.009194 3.192e-127
35362 TRUE reported textfile 2 1 TRUE
804 rs61747728 T C T C 0.1546 0.0236 0.04262 NA FALSE FALSE FALSE
GCST006061 0.0209 0.2605 537409 outcome TRUE reported
7849_3_QPCT_Glutaminyl_cyclase QPCT chr1 179557079 0.020396 3.459e-14
35358 TRUE reported textfile 2 1 TRUE
805 rs73169671 A C A C 0.0994 -0.0229 0.15184 NA FALSE FALSE FALSE
GCST006061 0.0119 0.05352 537409 outcome TRUE reported
7849_3_QPCT_Glutaminyl_cyclase QPCT chr7 151250603 0.011553 7.721e-18
35374 TRUE reported textfile 2 1 TRUE
806 rs74262625 T G T G 0.2136 0.0117 0.01889 NA FALSE FALSE FALSE
GCST006061 0.0356 0.7417 537409 outcome TRUE reported
7849_3_QPCT_Glutaminyl_cyclase QPCT chr2 36081193 0.03011 1.302e-12 35367
TRUE reported textfile 2 1 TRUE
807 rs76428106 C T C T 0.2191 -0.0446 0.01359 NA FALSE FALSE FALSE
GCST006061 0.0438 0.3085 537409 outcome TRUE reported
7849_3_QPCT_Glutaminyl_cyclase QPCT chr13 28029870 0.035979 1.132e-09
35363 TRUE reported textfile 2 1 TRUE
808 rs79685402 A G A G -0.1491 0.0065 0.03049 NA FALSE FALSE FALSE
GCST006061 0.0156 0.6788 537409 outcome TRUE reported
7849_3_QPCT_Glutaminyl_cyclase QPCT chr2 36617823 0.024406 1.001e-09
35362 TRUE reported textfile 2 1 TRUE
809 rs9901675 A G A G 0.0908 -0.0272 0.07646 NA FALSE FALSE FALSE
GCST006061 0.0161 0.0905 537409 outcome TRUE reported
7849_3_QPCT_Glutaminyl_cyclase QPCT chr17 7581494 0.015553 5.274e-09 35379
TRUE reported textfile 2 1 TRUE
810 rs10107630 T C T C -0.086 0.0063 0.40285 NA FALSE FALSE FALSE
GCST006061 0.0071 0.3806 537409 outcome TRUE reported 6997_32_RAB26_RAB26
RAB26 chr8 129591389 0.008333 5.678e-25 35372 TRUE reported
textfile 2 1 TRUE

811 rs10799536 G A G A 0.1041 -0.0116 0.1903 NA FALSE FALSE FALSE
GCST006061 0.0088 0.1851 537409 outcome TRUE reported 6997_32_RAB26_RAB26
RAB26 chr1 229525044 0.010376 1.092e-23 35361 TRUE reported
textfile 2 1 TRUE
812 rs117317284 T C T C 0.2044 0.0155 0.01706 NA FALSE FALSE FALSE
GCST006061 0.0327 0.6358 537409 outcome TRUE reported 6997_32_RAB26_RAB26
RAB26 chr12 101665112 0.032185 2.143e-10 35363 TRUE reported
textfile 2 1 TRUE
813 rs117405202 A G A G -0.332 0.0551 0.01005 NA FALSE FALSE FALSE
GCST006061 0.05 0.2699 537409 outcome TRUE reported 6997_32_RAB26_RAB26
RAB26 chr17 57782856 0.042085 3.053e-15 35367 TRUE reported
textfile 2 1 TRUE
814 rs138839548 T G T G -0.2542 0.0843 0.0139 NA FALSE FALSE FALSE
GCST006061 0.0531 0.1122 537409 outcome TRUE reported 6997_32_RAB26_RAB26
RAB26 chr17 64278919 0.035499 8.018e-13 35368 TRUE reported
textfile 2 1 TRUE
815 rs145433937 G A G A -0.6586 -0.1611 0.01131 NA FALSE FALSE FALSE
GCST006061 0.0682 0.01807 537409 outcome TRUE reported 6997_32_RAB26_RAB26
RAB26 chr17 59868859 0.039176 2.012e-63 35370 TRUE reported
textfile 2 1 TRUE
816 rs147414880 T C T C -0.2157 0.0294 0.0242 NA FALSE FALSE FALSE
GCST006061 0.0404 0.4672 537409 outcome TRUE reported 6997_32_RAB26_RAB26
RAB26 chr17 60173878 0.02668 6.229e-16 35370 TRUE reported textfile
2 1 TRUE
817 rs147850744 T C T C -0.3114 0.0025 0.02146 NA FALSE FALSE FALSE
GCST006061 0.0308 0.9342 537409 outcome TRUE reported 6997_32_RAB26_RAB26
RAB26 chr17 58297003 0.028306 3.776e-28 35369 TRUE reported
textfile 2 1 TRUE
818 rs1687481 T C T C 0.0624 -0.0026 0.32022 NA FALSE FALSE FALSE
GCST006061 0.0077 0.738 537409 outcome TRUE reported 6997_32_RAB26_RAB26
RAB26 chr3 126517237 0.008804 1.366e-12 35371 TRUE reported
textfile 2 1 TRUE
819 rs1707652 T C T C 0.0793 0.0081 0.3102 NA FALSE FALSE FALSE
GCST006061 0.0074 0.2701 537409 outcome TRUE reported 6997_32_RAB26_RAB26
RAB26 chr3 165761011 0.008827 2.622e-19 35339 TRUE reported
textfile 2 1 TRUE
820 rs2305637 T C T C 0.1238 5e-04 0.15762 NA FALSE FALSE FALSE
GCST006061 0.0097 0.961 537409 outcome TRUE reported 6997_32_RAB26_RAB26
RAB26 chr3 47004356 0.01113 9.702e-29 35343 TRUE reported textfile
2 1 TRUE
821 rs2606724 A G A G 0.0448 3e-04 0.49406 NA FALSE FALSE FALSE
GCST006061 0.0073 0.9683 537409 outcome TRUE reported 6997_32_RAB26_RAB26
RAB26 chr11 114087158 0.008202 4.709e-08 35364 TRUE reported
textfile 2 1 TRUE
822 rs28678897 A G A G 0.0949 0.024 0.10162 NA FALSE FALSE FALSE
GCST006061 0.0124 0.05344 537409 outcome TRUE reported 6997_32_RAB26_RAB26
RAB26 chr16 477485 0.013536 2.365e-12 35317 TRUE reported textfile
2 1 TRUE
823 rs35892171 A G A G -0.5581 -0.0027 0.02122 NA FALSE FALSE FALSE
GCST006061 0.0296 0.9269 537409 outcome TRUE reported 6997_32_RAB26_RAB26
RAB26 chr17 58194899 0.028885 3.556e-83 35367 TRUE reported
textfile 2 1 TRUE
824 rs4383561 C T C T 0.0579 -0.0051 0.22155 NA FALSE FALSE FALSE
GCST006061 0.0099 0.6079 537409 outcome TRUE reported 6997_32_RAB26_RAB26
RAB26 chr3 46278971 0.009943 5.777e-09 35345 TRUE reported
textfile 2 1 TRUE
825 rs444053 C T C T 0.063 -0.0082 0.25963 NA FALSE FALSE FALSE
GCST006061 0.0085 0.3344 537409 outcome TRUE reported 6997_32_RAB26_RAB26
RAB26 chr19 16141262 0.009311 1.319e-11 35360 TRUE reported
textfile 2 1 TRUE
826 rs6080761 A G A G 0.0717 -0.0037 0.45658 NA FALSE FALSE FALSE
GCST006061 0.0073 0.6166 537409 outcome TRUE reported 6997_32_RAB26_RAB26
RAB26 chr20 17648517 0.008172 1.729e-18 35374 TRUE reported
textfile 2 1 TRUE
827 rs62132278 T C T C -0.0617 -0.0145 0.27963 NA FALSE FALSE FALSE
GCST006061 0.01 0.1468 537409 outcome TRUE reported 6997_32_RAB26_RAB26

RAB26 chr19 836654 0.009197 1.968e-11 35311 TRUE reported textfile 2 1 TRUE
828 rs6544659 A C A C -0.0693 0.0019 0.47699 NA FALSE FALSE FALSE GCST006061 0.0072 0.7935 537409 outcome TRUE reported 6997_32_RAB26_RAB26 RAB26 chr2 43431284 0.00816 2.019e-17 35364 TRUE reported textfile 2 1 TRUE
829 rs6685931 C T C T 0.0449 0.0155 0.44505 NA FALSE FALSE FALSE GCST006061 0.0076 0.04182 537409 outcome TRUE reported 6997_32_RAB26_RAB26 RAB26 chr1 196898103 0.008219 4.686e-08 35338 TRUE reported textfile 2 1 TRUE
830 rs6775011 C T C T -0.0851 -9.00E-04 0.29896 NA FALSE FALSE FALSE GCST006061 0.0083 0.914 537409 outcome TRUE reported 6997_32_RAB26_RAB26 RAB26 chr3 128677640 0.008939 1.724e-21 35363 TRUE reported textfile 2 1 TRUE
831 rs72786786 A G A G 0.0672 0.0062 0.31073 NA FALSE FALSE FALSE GCST006061 0.0081 0.4475 537409 outcome TRUE reported 6997_32_RAB26_RAB26 RAB26 chr16 56951602 0.008839 2.896e-14 35369 TRUE reported textfile 2 1 TRUE
832 rs76428106 C T C T 0.3551 -0.0446 0.01359 NA FALSE FALSE FALSE GCST006061 0.0438 0.3085 537409 outcome TRUE reported 6997_32_RAB26_RAB26 RAB26 chr13 28029870 0.035468 1.353e-23 35363 TRUE reported textfile 2 1 TRUE
833 rs768053 C T C T 0.074 -0.008 0.11875 NA FALSE FALSE FALSE GCST006061 0.0118 0.5 537409 outcome TRUE reported 6997_32_RAB26_RAB26 RAB26 chr17 57707885 0.012784 7.105e-09 35367 TRUE reported textfile 2 1 TRUE
834 rs10107630 T C T C -0.0991 0.0063 0.40285 NA FALSE FALSE FALSE GCST006061 0.0071 0.3806 537409 outcome TRUE reported 2860_19_KPNA2_Karyopherin_a2 KPNA2 chr8 129591389 0.008262 3.787e-33 35372 TRUE reported textfile 2 1 TRUE
835 rs10799536 G A G A 0.0692 -0.0116 0.1903 NA FALSE FALSE FALSE GCST006061 0.0088 0.1851 537409 outcome TRUE reported 2860_19_KPNA2_Karyopherin_a2 KPNA2 chr1 229525044 0.01029 1.757e-11 35361 TRUE reported textfile 2 1 TRUE
836 rs117317284 T C T C 0.2327 0.0155 0.01706 NA FALSE FALSE FALSE GCST006061 0.0327 0.6358 537409 outcome TRUE reported 2860_19_KPNA2_Karyopherin_a2 KPNA2 chr12 101665112 0.031929 3.146e-13 35363 TRUE reported textfile 2 1 TRUE
837 rs117405202 A G A G -0.3691 0.0551 0.01005 NA FALSE FALSE FALSE GCST006061 0.05 0.2699 537409 outcome TRUE reported 2860_19_KPNA2_Karyopherin_a2 KPNA2 chr17 57782856 0.041954 1.395e-18 35367 TRUE reported textfile 2 1 TRUE
838 rs13100173 A G A G -0.0464 -0.017 0.46361 NA FALSE FALSE FALSE GCST006061 0.0073 0.01943 537409 outcome TRUE reported 2860_19_KPNA2_Karyopherin_a2 KPNA2 chr3 50295266 0.008173 1.368e-08 35342 TRUE reported textfile 2 1 TRUE
839 rs138839548 T G T G -0.2973 0.0843 0.0139 NA FALSE FALSE FALSE GCST006061 0.0531 0.1122 537409 outcome TRUE reported 2860_19_KPNA2_Karyopherin_a2 KPNA2 chr17 64278919 0.035385 4.396e-17 35368 TRUE reported textfile 2 1 TRUE
840 rs143977287 C A C A 0.1814 0.0367 0.0233 NA FALSE FALSE FALSE GCST006061 0.0275 0.1822 537409 outcome TRUE reported 2860_19_KPNA2_Karyopherin_a2 KPNA2 chr17 59227555 0.027714 5.933e-11 35369 TRUE reported textfile 2 1 TRUE
841 rs1443264 T C T C -0.0503 -0.003 0.43589 NA FALSE FALSE FALSE GCST006061 0.0072 0.6728 537409 outcome TRUE reported 2860_19_KPNA2_Karyopherin_a2 KPNA2 chr17 68836817 0.008373 1.884e-09 35371 TRUE reported textfile 2 1 TRUE
842 rs145433937 G A G A -0.6931 -0.1611 0.01131 NA FALSE FALSE FALSE GCST006061 0.0682 0.01807 537409 outcome TRUE reported 2860_19_KPNA2_Karyopherin_a2 KPNA2 chr17 59868859 0.039041 1.633e-70 35370 TRUE reported textfile 2 1 TRUE
843 rs147850744 T C T C -0.3719 0.0025 0.02146 NA FALSE FALSE FALSE GCST006061 0.0308 0.9342 537409 outcome TRUE reported 2860_19_KPNA2_Karyopherin_a2 KPNA2 chr17 58297003 0.028203 1.046e-39 35369 TRUE reported textfile 2 1 TRUE

844 rs1687481 T C T C 0.0729 -0.0026 0.32022 NA FALSE FALSE FALSE
GCST006061 0.0077 0.738 537409 outcome TRUE reported
2860_19_KPNA2_Karyopherin_a2 KPNA2 chr3 126517237 0.008727 6.653e-17
35371 TRUE reported textfile 2 1 TRUE
845 rs1864163 A G A G -0.0652 -0.0068 0.25315 NA FALSE FALSE FALSE
GCST006061 0.0083 0.418 537409 outcome TRUE reported
2860_19_KPNA2_Karyopherin_a2 KPNA2 chr16 56963321 0.009325 2.705e-12
35369 TRUE reported textfile 2 1 TRUE
846 rs2071408 A G A G -0.0536 -0.0093 0.38311 NA FALSE FALSE FALSE
GCST006061 0.0074 0.2061 537409 outcome TRUE reported
2860_19_KPNA2_Karyopherin_a2 KPNA2 chr14 103520741 0.008392 1.691e-10
35276 TRUE reported textfile 2 1 TRUE
847 rs2283255 G A G A -0.0457 -0.0091 0.42376 NA FALSE FALSE FALSE
GCST006061 0.0074 0.2163 537409 outcome TRUE reported
2860_19_KPNA2_Karyopherin_a2 KPNA2 chr11 17473504 0.008236 2.873e-08
35371 TRUE reported textfile 2 1 TRUE
848 rs2305637 T C T C 0.1473 5e-04 0.15762 NA FALSE FALSE FALSE
GCST006061 0.0097 0.961 537409 outcome TRUE reported
2860_19_KPNA2_Karyopherin_a2 KPNA2 chr3 47004356 0.011036 1.227e-40
35343 TRUE reported textfile 2 1 TRUE
849 rs34198449 A G A G -0.0913 -0.0011 0.29824 NA FALSE FALSE FALSE
GCST006061 0.0083 0.8965 537409 outcome TRUE reported
2860_19_KPNA2_Karyopherin_a2 KPNA2 chr3 128662427 0.008866 7.236e-25
35363 TRUE reported textfile 2 1 TRUE
850 rs35892171 A G A G -0.6175 -0.0027 0.02122 NA FALSE FALSE FALSE
GCST006061 0.0296 0.9269 537409 outcome TRUE reported
2860_19_KPNA2_Karyopherin_a2 KPNA2 chr17 58194899 0.028765 3.144e-102
35367 TRUE reported textfile 2 1 TRUE
851 rs3790316 G A G A 0.0795 -0.0033 0.4572 NA FALSE FALSE FALSE
GCST006061 0.0073 0.6524 537409 outcome TRUE reported
2860_19_KPNA2_Karyopherin_a2 KPNA2 chr20 17648794 0.008105 1.037e-22
35374 TRUE reported textfile 2 1 TRUE
852 rs6544659 A C A C -0.0694 0.0019 0.47699 NA FALSE FALSE FALSE
GCST006061 0.0072 0.7935 537409 outcome TRUE reported
2860_19_KPNA2_Karyopherin_a2 KPNA2 chr2 43431284 0.008101 1.061e-17
35364 TRUE reported textfile 2 1 TRUE
853 rs6573880 C T C T -0.0759 0.0082 0.1227 NA FALSE FALSE FALSE
GCST006061 0.0122 0.5042 537409 outcome TRUE reported
2860_19_KPNA2_Karyopherin_a2 KPNA2 chr14 24579647 0.012304 6.88e-10
35377 TRUE reported textfile 2 1 TRUE
854 rs6796 C T C T -0.0742 0.0015 0.23682 NA FALSE FALSE FALSE GCST006061
0.0081 0.857 537409 outcome TRUE reported 2860_19_KPNA2_Karyopherin_a2
KPNA2 chr7 6462736 0.009505 5.875e-15 35364 TRUE reported textfile
2 1 TRUE
855 rs7212738 C T C T -0.1937 0.0193 0.08212 NA FALSE FALSE FALSE
GCST006061 0.0173 0.2665 537409 outcome TRUE reported
2860_19_KPNA2_Karyopherin_a2 KPNA2 chr17 58769288 0.014878 9.493e-39
35370 TRUE reported textfile 2 1 TRUE
856 rs7500966 G A G A -0.0553 0.0094 0.47169 NA FALSE FALSE FALSE
GCST006061 0.0072 0.1916 537409 outcome TRUE reported
2860_19_KPNA2_Karyopherin_a2 KPNA2 chr16 480386 0.008166 1.272e-11 35316
TRUE reported textfile 2 1 TRUE
857 rs75380888 C T C T -0.1735 -0.0366 0.03635 NA FALSE FALSE FALSE
GCST006061 0.026 0.1594 537409 outcome TRUE reported
2860_19_KPNA2_Karyopherin_a2 KPNA2 chr17 61277200 0.022072 3.827e-15
35362 TRUE reported textfile 2 1 TRUE
858 rs76428106 C T C T 0.398 -0.0446 0.01359 NA FALSE FALSE FALSE
GCST006061 0.0438 0.3085 537409 outcome TRUE reported
2860_19_KPNA2_Karyopherin_a2 KPNA2 chr13 28029870 0.035184 1.144e-29
35363 TRUE reported textfile 2 1 TRUE
859 rs78169818 C T C T 0.1399 -0.0401 0.03205 NA FALSE FALSE FALSE
GCST006061 0.0324 0.2171 537409 outcome TRUE reported
2860_19_KPNA2_Karyopherin_a2 KPNA2 chr12 101739280 0.023841 4.412e-09
35364 TRUE reported textfile 2 1 TRUE
860 rs8102236 A G A G 0.0843 -0.0055 0.26364 NA FALSE FALSE FALSE
GCST006061 0.0083 0.5051 537409 outcome TRUE reported

2860_19_KPNA2_Karyopherin_a2 KPNA2 chr19 16139785 0.009195 4.8e-20 35363
TRUE reported textfile 2 1 TRUE
861 rs10107630 T C T C -0.0888 0.0063 0.40285 NA FALSE FALSE FALSE
GCST006061 0.0071 0.3806 537409 outcome TRUE reported
2580_83_MPO_Myeloperoxidase MPO chr8 129591389 0.008276 7.364e-27 35372
TRUE reported textfile 2 1 TRUE
862 rs11155297 T G T G 0.0595 7e-04 0.2561 NA FALSE FALSE FALSE
GCST006061 0.0085 0.931 537409 outcome TRUE reported
2580_83_MPO_Myeloperoxidase MPO chr6 143503967 0.009334 1.838e-10 35356
TRUE reported textfile 2 1 TRUE
863 rs117405202 A G A G -0.4146 0.0551 0.01005 NA FALSE FALSE FALSE
GCST006061 0.05 0.2699 537409 outcome TRUE reported
2580_83_MPO_Myeloperoxidase MPO chr17 57782856 0.042407 1.418e-22 35367
TRUE reported textfile 2 1 TRUE
864 rs117937075 A G A G 0.1573 -0.038 0.03295 NA FALSE FALSE FALSE
GCST006061 0.0369 0.3036 537409 outcome TRUE reported
2580_83_MPO_Myeloperoxidase MPO chr12 101834256 0.023449 1.971e-11 35364
TRUE reported textfile 2 1 TRUE
865 rs1263375 A C A C 0.0542 0.0209 0.22982 NA FALSE FALSE FALSE
GCST006061 0.0092 0.02329 537409 outcome TRUE reported
2580_83_MPO_Myeloperoxidase MPO chr14 93181663 0.009642 1.898e-08 35366
TRUE reported textfile 2 1 TRUE
866 rs12712881 C A C A -0.07 0.0025 0.47723 NA FALSE FALSE FALSE
GCST006061 0.0072 0.7277 537409 outcome TRUE reported
2580_83_MPO_Myeloperoxidase MPO chr2 43422641 0.008097 5.386e-18 35365
TRUE reported textfile 2 1 TRUE
867 rs13100173 A G A G -0.0495 -0.017 0.46361 NA FALSE FALSE FALSE
GCST006061 0.0073 0.01943 537409 outcome TRUE reported
2580_83_MPO_Myeloperoxidase MPO chr3 50295266 0.00818 1.435e-09 35342 TRUE
reported textfile 2 1 TRUE
868 rs139196635 A G A G 0.1983 0.0242 0.02061 NA FALSE FALSE FALSE
GCST006061 0.0312 0.4387 537409 outcome TRUE reported
2580_83_MPO_Myeloperoxidase MPO chr12 101863758 0.028985 7.839e-12 35364
TRUE reported textfile 2 1 TRUE
869 rs145433937 G A G A -0.7963 -0.1611 0.01131 NA FALSE FALSE FALSE
GCST006061 0.0682 0.01807 537409 outcome TRUE reported
2580_83_MPO_Myeloperoxidase MPO chr17 59868859 0.039425 1.02e-90 35370
TRUE reported textfile 2 1 TRUE
870 rs146512685 C T C T -0.3007 -0.0095 0.01424 NA FALSE FALSE FALSE
GCST006061 0.0314 0.7628 537409 outcome TRUE reported
2580_83_MPO_Myeloperoxidase MPO chr17 57710637 0.035946 5.989e-17 35368
TRUE reported textfile 2 1 TRUE
871 rs1687481 T C T C 0.0546 -0.0026 0.32022 NA FALSE FALSE FALSE
GCST006061 0.0077 0.738 537409 outcome TRUE reported
2580_83_MPO_Myeloperoxidase MPO chr3 126517237 0.008741 4.193e-10 35371
TRUE reported textfile 2 1 TRUE
872 rs190383363 T C T C -0.7756 0.0078 0.01537 NA FALSE FALSE FALSE
GCST006061 0.0374 0.8348 537409 outcome TRUE reported
2580_83_MPO_Myeloperoxidase MPO chr17 59235408 0.034384 1.145e-112 35369
TRUE reported textfile 2 1 TRUE
873 rs2071408 A G A G -0.0466 -0.0093 0.38311 NA FALSE FALSE FALSE
GCST006061 0.0074 0.2061 537409 outcome TRUE reported
2580_83_MPO_Myeloperoxidase MPO chr14 103520741 0.008388 2.764e-08 35276
TRUE reported textfile 2 1 TRUE
874 rs2698197 T C T C -0.0451 -0.0419 0.43054 NA FALSE FALSE FALSE
GCST006061 0.0072 4.858e-09 537409 outcome TRUE reported
2580_83_MPO_Myeloperoxidase MPO chr2 61155308 0.008222 4.133e-08 35353
TRUE reported textfile 2 1 TRUE
875 rs2974751 C A C A 0.0582 0.0152 0.45223 NA FALSE FALSE FALSE
GCST006061 0.0074 0.04127 537409 outcome TRUE reported
2580_83_MPO_Myeloperoxidase MPO chr19 12942220 0.008193 1.219e-12 35381
TRUE reported textfile 2 1 TRUE
876 rs34097845 T C T C -0.5179 -0.0021 0.0566 NA FALSE FALSE FALSE
GCST006061 0.0185 0.9114 537409 outcome TRUE reported
2580_83_MPO_Myeloperoxidase MPO chr17 58281068 0.018241 2.494e-177 35369
TRUE reported textfile 2 1 TRUE

877 rs34198449 A G A G -0.0741 -0.0011 0.29824 NA FALSE FALSE FALSE
GCST006061 0.0083 0.8965 537409 outcome TRUE reported
2580_83_MPO_Myeloperoxidase MPO chr3 128662427 0.008875 6.891e-17 35363
TRUE reported textfile 2 1 TRUE
878 rs3790316 G A G A 0.0736 -0.0033 0.4572 NA FALSE FALSE FALSE
GCST006061 0.0073 0.6524 537409 outcome TRUE reported
2580_83_MPO_Myeloperoxidase MPO chr20 17648794 0.008114 1.187e-19 35374
TRUE reported textfile 2 1 TRUE
879 rs55771110 A G A G 0.1226 0.0077 0.14938 NA FALSE FALSE FALSE
GCST006061 0.0102 0.4518 537409 outcome TRUE reported
2580_83_MPO_Myeloperoxidase MPO chr3 47305227 0.011294 1.877e-27 35343
TRUE reported textfile 2 1 TRUE
880 rs56757486 A G A G -0.051 -0.0166 0.28147 NA FALSE FALSE FALSE
GCST006061 0.0101 0.09933 537409 outcome TRUE reported
2580_83_MPO_Myeloperoxidase MPO chr19 836144 0.009136 2.373e-08 35311 TRUE
reported textfile 2 1 TRUE
881 rs62081796 C T C T 0.1485 0.0113 0.05492 NA FALSE FALSE FALSE
GCST006061 0.0189 0.5487 537409 outcome TRUE reported
2580_83_MPO_Myeloperoxidase MPO chr17 58020850 0.018364 6.149e-16 35366
TRUE reported textfile 2 1 TRUE
882 rs6962012 G T G T -0.0689 -0.0063 0.19628 NA FALSE FALSE FALSE
GCST006061 0.009 0.4868 537409 outcome TRUE reported
2580_83_MPO_Myeloperoxidase MPO chr7 6457870 0.010177 1.287e-11 35364 TRUE
reported textfile 2 1 TRUE
883 rs76428106 C T C T 0.3782 -0.0446 0.01359 NA FALSE FALSE FALSE
GCST006061 0.0438 0.3085 537409 outcome TRUE reported
2580_83_MPO_Myeloperoxidase MPO chr13 28029870 0.03524 7.182e-27 35363 TRUE
reported textfile 2 1 TRUE
884 rs77542162 G A G A 0.1544 0.0177 0.025 NA FALSE FALSE FALSE
GCST006061 0.0302 0.557 537409 outcome TRUE reported
2580_83_MPO_Myeloperoxidase MPO chr17 69085137 0.02637 4.769e-09 35371 TRUE
reported textfile 2 1 TRUE
885 rs9898547 T G T G -0.046 0.013 0.44771 NA FALSE FALSE FALSE
GCST006061 0.0071 0.06951 537409 outcome TRUE reported
2580_83_MPO_Myeloperoxidase MPO chr17 39979773 0.008419 4.667e-08 35379
TRUE reported textfile 2 1 TRUE
886 rs10107630 T C T C -0.0736 0.0063 0.40285 NA FALSE FALSE FALSE
GCST006061 0.0071 0.3806 537409 outcome TRUE reported
2966_65_CLEC11A_SCGF_beta CLEC11A chr8 129591389 0.008395 1.837e-18 35372
TRUE reported textfile 2 1 TRUE
887 rs11043 A G A G 0.079 0.0094 0.09755 NA FALSE FALSE FALSE GCST006061
0.0107 0.3784 537409 outcome TRUE reported 2966_65_CLEC11A_SCGF_beta CLEC11A
chr16 68229055 0.014 1.673e-08 35372 TRUE reported textfile 2 1
TRUE
888 rs11144926 T C T C -0.0601 -0.0023 0.18076 NA FALSE FALSE FALSE
GCST006061 0.0086 0.7927 537409 outcome TRUE reported
2966_65_CLEC11A_SCGF_beta CLEC11A chr9 76495162 0.010773 2.42e-08 35352
TRUE reported textfile 2 1 TRUE
889 rs112725054 A C A C 0.1728 -0.0408 0.03057 NA FALSE FALSE FALSE
GCST006061 0.0302 0.1768 537409 outcome TRUE reported
2966_65_CLEC11A_SCGF_beta CLEC11A chr12 103499150 0.024006 6.099e-13 35367
TRUE reported textfile 2 1 TRUE
890 rs116853700 A G A G -0.1431 -0.0084 0.04089 NA FALSE FALSE FALSE
GCST006061 0.0189 0.6569 537409 outcome TRUE reported
2966_65_CLEC11A_SCGF_beta CLEC11A chr17 57388934 0.020812 6.157e-12 35375
TRUE reported textfile 2 1 TRUE
891 rs116919905 C A C A 0.2059 0.0065 0.01551 NA FALSE FALSE FALSE
GCST006061 0.0342 0.8502 537409 outcome TRUE reported
2966_65_CLEC11A_SCGF_beta CLEC11A chr19 50626403 0.033584 8.734e-10 35373
TRUE reported textfile 2 1 TRUE
892 rs117143947 A G A G 0.1803 -0.0185 0.02207 NA FALSE FALSE FALSE
GCST006061 0.0293 0.5285 537409 outcome TRUE reported
2966_65_CLEC11A_SCGF_beta CLEC11A chr19 50300994 0.02885 4.116e-10 35373
TRUE reported textfile 2 1 TRUE
893 rs139675927 G A G A 0.2157 -0.0474 0.01255 NA FALSE FALSE FALSE
GCST006061 0.0585 0.4172 537409 outcome TRUE reported

2966_65_CLEC11A_SCGF_beta CLEC11A chr19 48009532 0.037352 7.703e-09 35366 TRUE reported textfile 2 1 TRUE
894 rs17075907 C T C T 0.0626 -0.0081 0.19967 NA FALSE FALSE FALSE GCST006061 0.0096 0.3969 537409 outcome TRUE reported 2966_65_CLEC11A_SCGF_beta CLEC11A chr13 51731328 0.010355 1.489e-09 35370 TRUE reported textfile 2 1 TRUE
895 rs17345153 G A G A -0.0498 -0.0193 0.40363 NA FALSE FALSE FALSE GCST006061 0.0075 0.009625 537409 outcome TRUE reported 2966_65_CLEC11A_SCGF_beta CLEC11A chr1 205210869 0.008412 3.224e-09 35375 TRUE reported textfile 2 1 TRUE
896 rs2305637 T C T C 0.1002 5e-04 0.15762 NA FALSE FALSE FALSE GCST006061 0.0097 0.961 537409 outcome TRUE reported 2966_65_CLEC11A_SCGF_beta CLEC11A chr3 47004356 0.011221 4.283e-19 35343 TRUE reported textfile 2 1 TRUE
897 rs2380606 C T C T 0.0454 -0.007 0.47568 NA FALSE FALSE FALSE GCST006061 0.0072 0.3283 537409 outcome TRUE reported 2966_65_CLEC11A_SCGF_beta CLEC11A chr8 69828661 0.008248 3.706e-08 35362 TRUE reported textfile 2 1 TRUE
898 rs41275788 G A G A 0.7117 0.0066 0.03164 NA FALSE FALSE FALSE GCST006061 0.0267 0.8057 537409 outcome TRUE reported 2966_65_CLEC11A_SCGF_beta CLEC11A chr19 50711843 0.02293 1e-200 35367 TRUE reported textfile 2 1 TRUE
899 rs62180989 T C T C 0.0529 -0.0075 0.34977 NA FALSE FALSE FALSE GCST006061 0.0078 0.3395 537409 outcome TRUE reported 2966_65_CLEC11A_SCGF_beta CLEC11A chr2 190406518 0.008641 9.227e-10 35377 TRUE reported textfile 2 1 TRUE
900 rs6573880 C T C T 0.0793 0.0082 0.1227 NA FALSE FALSE FALSE GCST006061 0.0122 0.5042 537409 outcome TRUE reported 2966_65_CLEC11A_SCGF_beta CLEC11A chr14 24579647 0.012494 2.194e-10 35377 TRUE reported textfile 2 1 TRUE
901 rs76239001 G T G T 0.144 0.0271 0.0342 NA FALSE FALSE FALSE GCST006061 0.0209 0.1957 537409 outcome TRUE reported 2966_65_CLEC11A_SCGF_beta CLEC11A chr12 102891505 0.022423 1.345e-10 35365 TRUE reported textfile 2 1 TRUE
902 rs76428106 C T C T 0.2234 -0.0446 0.01359 NA FALSE FALSE FALSE GCST006061 0.0438 0.3085 537409 outcome TRUE reported 2966_65_CLEC11A_SCGF_beta CLEC11A chr13 28029870 0.03577 4.227e-10 35363 TRUE reported textfile 2 1 TRUE
903 rs8006994 T C T C 0.0591 0.0168 0.32204 NA FALSE FALSE FALSE GCST006061 0.0079 0.03338 537409 outcome TRUE reported 2966_65_CLEC11A_SCGF_beta CLEC11A chr14 103374959 0.008837 2.271e-11 35277 TRUE reported textfile 2 1 TRUE
904 rs9970784 T C T C -0.0481 -0.0129 0.43157 NA FALSE FALSE FALSE GCST006061 0.0074 0.08036 537409 outcome TRUE reported 2966_65_CLEC11A_SCGF_beta CLEC11A chr1 196704672 0.008315 7.261e-09 35339 TRUE reported textfile 2 1 TRUE
905 rs10107630 T C T C -0.0519 0.0063 0.40285 NA FALSE FALSE FALSE GCST006061 0.0071 0.3806 537409 outcome TRUE reported 16915_153_SEMA4A_SEM4A SEMA4A chr8 129591389 0.008582 1.468e-09 35372 TRUE reported textfile 2 1 TRUE
906 rs10858304 C T C T 0.2784 0.0029 0.26107 NA FALSE FALSE FALSE GCST006061 0.008 0.7168 537409 outcome TRUE reported 16915_153_SEMA4A_SEM4A SEMA4A chr9 134966017 0.009524 7.993e-188 35329 TRUE reported textfile 2 1 TRUE
907 rs11103564 C T C T 0.0856 -1.00E-04 0.31136 NA FALSE FALSE FALSE GCST006061 0.008 0.9946 537409 outcome TRUE reported 16915_153_SEMA4A_SEM4A SEMA4A chr9 134888029 0.00914 7.573e-21 35333 TRUE reported textfile 2 1 TRUE
908 rs11775560 G A G A 0.0617 -0.0213 0.18623 NA FALSE FALSE FALSE GCST006061 0.009 0.01846 537409 outcome TRUE reported 16915_153_SEMA4A_SEM4A SEMA4A chr8 60747604 0.010759 9.753e-09 35360 TRUE reported textfile 2 1 TRUE
909 rs143501783 C T C T 0.2429 -0.0333 0.01116 NA FALSE FALSE FALSE GCST006061 0.0443 0.4525 537409 outcome TRUE reported 16915_153_SEMA4A_SEM4A SEMA4A chr17 7897001 0.038229 2.101e-10 35373 TRUE reported textfile 2 1 TRUE

910 rs148925417 A G A G 0.1994 -0.003 0.01629 NA FALSE FALSE FALSE
GCST006061 0.0328 0.928 537409 outcome TRUE reported 16915_153_SEMA4A_SEM4A
SEMA4A chr6 26462714 0.033776 3.557e-09 35376 TRUE reported
textfile 2 1 TRUE
911 rs3184504 C T C T -0.0606 0.0074 0.37862 NA FALSE FALSE FALSE
GCST006061 0.0075 0.3235 537409 outcome TRUE reported 16915_153_SEMA4A_SEM4A
SEMA4A chr12 111446804 0.008678 2.884e-12 35371 TRUE reported
textfile 2 1 TRUE
912 rs3821801 C T C T -0.0557 0.0039 0.3329 NA FALSE FALSE FALSE
GCST006061 0.0077 0.6103 537409 outcome TRUE reported 16915_153_SEMA4A_SEM4A
SEMA4A chr3 187219433 0.008908 4.032e-10 35370 TRUE reported
textfile 2 1 TRUE
913 rs4842221 G A G A 0.065 0.0153 0.20203 NA FALSE FALSE FALSE
GCST006061 0.0094 0.1044 537409 outcome TRUE reported 16915_153_SEMA4A_SEM4A
SEMA4A chr9 135111072 0.010511 6.259e-10 35324 TRUE reported
textfile 2 1 TRUE
914 rs55714927 T C T C 0.0722 1e-04 0.21166 NA FALSE FALSE FALSE
GCST006061 0.0098 0.9926 537409 outcome TRUE reported 16915_153_SEMA4A_SEM4A
SEMA4A chr17 7176997 0.010357 3.146e-12 35370 TRUE reported textfile
2 1 TRUE
915 rs55974215 A C A C -0.0638 0.0059 0.42574 NA FALSE FALSE FALSE
GCST006061 0.0074 0.4207 537409 outcome TRUE reported 16915_153_SEMA4A_SEM4A
SEMA4A chr17 73055132 0.008506 6.338e-14 35363 TRUE reported
textfile 2 1 TRUE
916 rs58560372 T C T C -0.0824 -0.0128 0.15484 NA FALSE FALSE FALSE
GCST006061 0.0102 0.2104 537409 outcome TRUE reported 16915_153_SEMA4A_SEM4A
SEMA4A chr19 38268112 0.011639 1.448e-12 35376 TRUE reported
textfile 2 1 TRUE
917 rs62019249 C T C T 0.0622 0.0024 0.3719 NA FALSE FALSE FALSE
GCST006061 0.0077 0.7545 537409 outcome TRUE reported 16915_153_SEMA4A_SEM4A
SEMA4A chr15 90205974 0.008727 1.021e-12 35372 TRUE reported
textfile 2 1 TRUE
918 rs76428106 C T C T 0.4009 -0.0446 0.01359 NA FALSE FALSE FALSE
GCST006061 0.0438 0.3085 537409 outcome TRUE reported 16915_153_SEMA4A_SEM4A
SEMA4A chr13 28029870 0.036507 4.69e-28 35363 TRUE reported
textfile 2 1 TRUE
919 rs7695 C T C T 0.1667 -0.0143 0.37641 NA FALSE FALSE FALSE GCST006061
0.0076 0.0578 537409 outcome TRUE reported 16915_153_SEMA4A_SEM4A SEMA4A
chr1 156177535 0.008701 8.287e-82 35361 TRUE reported textfile 2
1 TRUE
920 rs7899547 G T G T -0.0854 0.0054 0.33988 NA FALSE FALSE FALSE
GCST006061 0.0075 0.4742 537409 outcome TRUE reported 16915_153_SEMA4A_SEM4A
SEMA4A chr10 52777079 0.008916 9.917e-22 35361 TRUE reported
textfile 2 1 TRUE
921 rs905938 C T C T 0.0571 -0.0421 0.30351 NA FALSE FALSE FALSE
GCST006061 0.0084 4.751e-07 537409 outcome TRUE reported
16915_153_SEMA4A_SEM4A SEMA4A chr1 155018913 0.00926 6.989e-10 35365 TRUE
reported textfile 2 1 TRUE
922 rs9898547 T G T G -0.0558 0.013 0.44771 NA FALSE FALSE FALSE
GCST006061 0.0071 0.06951 537409 outcome TRUE reported 16915_153_SEMA4A_SEM4A
SEMA4A chr17 39979773 0.008477 4.626e-11 35379 TRUE reported
textfile 2 1 TRUE
923 rs1010881 G A G A 0.0689 0.0011 0.16209 NA FALSE FALSE FALSE
GCST006061 0.009 0.9032 537409 outcome TRUE reported 5628_21_SEMA3G_SEM3G
SEMA3G chr8 145039675 0.011403 1.519e-09 34537 TRUE reported
textfile 2 1 TRUE
924 rs10513546 A G A G 0.0609 -0.0087 0.30116 NA FALSE FALSE FALSE
GCST006061 0.0077 0.2621 537409 outcome TRUE reported 5628_21_SEMA3G_SEM3G
SEMA3G chr3 159833862 0.009057 1.77e-11 35338 TRUE reported
textfile 2 1 TRUE
925 rs10904974 A G A G 0.0596 0.0052 0.32105 NA FALSE FALSE FALSE
GCST006061 0.0077 0.5006 537409 outcome TRUE reported 5628_21_SEMA3G_SEM3G
SEMA3G chr10 17594736 0.008852 1.661e-11 35359 TRUE reported
textfile 2 1 TRUE
926 rs113245157 A G A G 0.3505 -0.019 0.01674 NA FALSE FALSE FALSE
GCST006061 0.0435 0.6624 537409 outcome TRUE reported 5628_21_SEMA3G_SEM3G

SEMA3G chr3 47091398 0.032349 2.347e-27 35342 TRUE reported
textfile 2 1 TRUE
927 rs115087321 A G A G -0.2595 -0.1124 0.01163 NA FALSE FALSE FALSE
GCST006061 0.0461 0.01476 537409 outcome TRUE reported 5628_21_SEMA3G_SEM3G
SEMA3G chr3 53607439 0.039326 4.147e-11 35350 TRUE reported
textfile 2 1 TRUE
928 rs115483359 T C T C 0.1759 0.0204 0.02305 NA FALSE FALSE FALSE
GCST006061 0.0644 0.7516 537409 outcome TRUE reported 5628_21_SEMA3G_SEM3G
SEMA3G chr3 52082358 0.027431 1.433e-10 35336 TRUE reported
textfile 2 1 TRUE
929 rs115990971 G A G A -0.3022 -0.0218 0.01409 NA FALSE FALSE FALSE
GCST006061 0.0332 0.5101 537409 outcome TRUE reported 5628_21_SEMA3G_SEM3G
SEMA3G chr3 53237177 0.035854 3.499e-17 35351 TRUE reported
textfile 2 1 TRUE
930 rs117241334 A C A C 0.1723 -0.095 0.01792 NA FALSE FALSE FALSE
GCST006061 0.0503 0.05877 537409 outcome TRUE reported 5628_21_SEMA3G_SEM3G
SEMA3G chr19 48633205 0.030913 2.495e-08 35376 TRUE reported
textfile 2 1 TRUE
931 rs12487573 T C T C -0.2343 0.0449 0.01533 NA FALSE FALSE FALSE
GCST006061 0.0429 0.2953 537409 outcome TRUE reported 5628_21_SEMA3G_SEM3G
SEMA3G chr3 46672980 0.034234 7.7e-12 35343 TRUE reported textfile
2 1 TRUE
932 rs12496329 T C T C -0.0522 0.0033 0.49856 NA FALSE FALSE FALSE
GCST006061 0.0072 0.6494 537409 outcome TRUE reported 5628_21_SEMA3G_SEM3G
SEMA3G chr3 169434875 0.00832 3.512e-10 35356 TRUE reported textfile
2 1 TRUE
933 rs13091025 C A C A -0.2814 -0.0187 0.11366 NA FALSE FALSE FALSE
GCST006061 0.0117 0.1094 537409 outcome TRUE reported 5628_21_SEMA3G_SEM3G
SEMA3G chr3 52433308 0.01294 7.4e-105 35350 TRUE reported textfile
2 1 TRUE
934 rs141686191 A G A G 0.1562 0.0068 0.02642 NA FALSE FALSE FALSE
GCST006061 0.0355 0.8484 537409 outcome TRUE reported 5628_21_SEMA3G_SEM3G
SEMA3G chr3 44072565 0.026364 3.126e-09 35345 TRUE reported
textfile 2 1 TRUE
935 rs143501783 C T C T 0.2586 -0.0333 0.01116 NA FALSE FALSE FALSE
GCST006061 0.0443 0.4525 537409 outcome TRUE reported 5628_21_SEMA3G_SEM3G
SEMA3G chr17 7897001 0.037489 5.27e-12 35373 TRUE reported textfile
2 1 TRUE
936 rs2008174 T C T C -0.1102 0.0184 0.23503 NA FALSE FALSE FALSE
GCST006061 0.0087 0.03407 537409 outcome TRUE reported 5628_21_SEMA3G_SEM3G
SEMA3G chr22 39464125 0.009699 6.494e-30 35364 TRUE reported
textfile 2 1 TRUE
937 rs2066985 T G T G -0.0882 0.0017 0.1001 NA FALSE FALSE FALSE
GCST006061 0.01 0.8649 537409 outcome TRUE reported 5628_21_SEMA3G_SEM3G
SEMA3G chr11 126381391 0.013961 2.658e-10 35376 TRUE reported
textfile 2 1 TRUE
938 rs333947 A G A G -0.0745 -0.0071 0.14937 NA FALSE FALSE FALSE
GCST006061 0.01 0.4808 537409 outcome TRUE reported 5628_21_SEMA3G_SEM3G
SEMA3G chr1 109928142 0.011655 1.635e-10 35349 TRUE reported
textfile 2 1 TRUE
939 rs55714927 T C T C 0.1134 1e-04 0.21166 NA FALSE FALSE FALSE
GCST006061 0.0098 0.9926 537409 outcome TRUE reported 5628_21_SEMA3G_SEM3G
SEMA3G chr17 7176997 0.010149 5.476e-29 35370 TRUE reported textfile
2 1 TRUE
940 rs8176743 T C T C 0.2497 0.017 0.06488 NA FALSE FALSE FALSE
GCST006061 0.0128 0.1834 537409 outcome TRUE reported 5628_21_SEMA3G_SEM3G
SEMA3G chr9 133256028 0.016764 3.533e-50 35370 TRUE reported
textfile 2 1 TRUE
941 rs9859898 G A G A 0.2071 0.0182 0.026 NA FALSE FALSE FALSE
GCST006061 0.0253 0.4709 537409 outcome TRUE reported 5628_21_SEMA3G_SEM3G
SEMA3G chr3 54348220 0.0264 4.34e-15 35346 TRUE reported textfile
2 1 TRUE
942 rs10109973 T C T C -0.2248 -0.0031 0.02808 NA FALSE FALSE FALSE
GCST006061 0.0272 0.9099 537409 outcome TRUE reported 16892_23_ENPP2_ENPP2
ENPP2 chr8 120053399 0.02489 1.689e-19 35360 TRUE reported textfile
2 1 TRUE

943 rs11780884 G A G A 0.0633 0.0152 0.16334 NA FALSE FALSE FALSE
GCST006061 0.009 0.09025 537409 outcome TRUE reported 16892_23_ENPP2_ENPP2
ENPP2 chr8 18388543 0.011261 1.899e-08 35377 TRUE reported
textfile 2 1 TRUE
944 rs147305264 T C T C -0.1868 0.0137 0.01854 NA FALSE FALSE FALSE
GCST006061 0.0368 0.71 537409 outcome TRUE reported 16892_23_ENPP2_ENPP2
ENPP2 chr8 119617719 0.031151 2.014e-09 35359 TRUE reported
textfile 2 1 TRUE
945 rs2264782 T C T C -0.0734 -0.009 0.32066 NA FALSE FALSE FALSE
GCST006061 0.0074 0.2214 537409 outcome TRUE reported 16892_23_ENPP2_ENPP2
ENPP2 chr12 120994800 0.008787 6.628e-17 35365 TRUE reported
textfile 2 1 TRUE
946 rs7829390 A G A G -0.1609 0 0.39446 NA FALSE FALSE FALSE GCST006061
0.0073 0.9975 537409 outcome TRUE reported 16892_23_ENPP2_ENPP2 ENPP2
chr8 119716654 0.008455 9.568e-81 35359 TRUE reported textfile 2
1 TRUE
947 rs10110738 T C T C 0.0654 7e-04 0.40115 NA FALSE FALSE FALSE
GCST006061 0.0072 0.9214 537409 outcome TRUE reported
2706_69_SERPINA7_Thyroxine_Binding_Globulin SERPINA7 chr8 115976022 0.008278
2.778e-15 35349 TRUE reported textfile 2 1 TRUE
948 rs1169288 C A C A 0.1 -0.0103 0.31006 NA FALSE FALSE FALSE GCST006061
0.0077 0.1791 537409 outcome TRUE reported
2706_69_SERPINA7_Thyroxine_Binding_Globulin SERPINA7 chr12 120978847 0.008789
5.43e-30 35365 TRUE reported textfile 2 1 TRUE
949 rs1800961 T C T C -0.117 -4.00E-04 0.04601 NA FALSE FALSE FALSE
GCST006061 0.0219 0.9854 537409 outcome TRUE reported
2706_69_SERPINA7_Thyroxine_Binding_Globulin SERPINA7 chr20 44413724 0.019551
2.174e-09 35371 TRUE reported textfile 2 1 TRUE
950 rs523118 G T G T -0.0608 -0.0288 0.23079 NA FALSE FALSE FALSE
GCST006061 0.0085 0.0006911 537409 outcome TRUE reported
2706_69_SERPINA7_Thyroxine_Binding_Globulin SERPINA7 chr3 136247046 0.009689
3.492e-10 35361 TRUE reported textfile 2 1 TRUE
951 rs804267 A G A G -0.0507 0.0253 0.31485 NA FALSE FALSE FALSE
GCST006061 0.0079 0.001307 537409 outcome TRUE reported
2706_69_SERPINA7_Thyroxine_Binding_Globulin SERPINA7 chr8 11771732 0.008763
7.21e-09 35377 TRUE reported textfile 2 1 TRUE
952 rs10117697 A G A G -0.0796 -0.0072 0.09035 NA FALSE FALSE FALSE
GCST006061 0.0128 0.5714 537409 outcome TRUE reported
19175_18_MARCKSL1_MARCKSL1 MARCKSL1 chr9 93398323 0.014308 2.647e-08
35360 TRUE reported textfile 2 1 TRUE
953 rs10437655 A G A G -0.0502 0.0098 0.37172 NA FALSE FALSE FALSE
GCST006061 0.0075 0.1881 537409 outcome TRUE reported
19175_18_MARCKSL1_MARCKSL1 MARCKSL1 chr11 47370397 0.008389 2.177e-09
35377 TRUE reported textfile 2 1 TRUE
954 rs1047891 A C A C -0.064 0.0155 0.26363 NA FALSE FALSE FALSE
GCST006061 0.0079 0.05042 537409 outcome TRUE reported
19175_18_MARCKSL1_MARCKSL1 MARCKSL1 chr2 210675783 0.009241 4.344e-12
35353 TRUE reported textfile 2 1 TRUE
955 rs12342201 A G A G -0.2137 -0.0063 0.49613 NA FALSE FALSE FALSE
GCST006061 0.0074 0.3926 537409 outcome TRUE reported
19175_18_MARCKSL1_MARCKSL1 MARCKSL1 chr9 93132682 0.008058 5.623e-155
35357 TRUE reported textfile 2 1 TRUE
956 rs2191225 G A G A -0.0672 -0.0031 0.18701 NA FALSE FALSE FALSE
GCST006061 0.009 0.731 537409 outcome TRUE reported
19175_18_MARCKSL1_MARCKSL1 MARCKSL1 chr19 51792687 0.010409 1.074e-10
35384 TRUE reported textfile 2 1 TRUE
957 rs2637678 C T C T 0.105 -0.002 0.37561 NA FALSE FALSE FALSE
GCST006061 0.0072 0.7832 537409 outcome TRUE reported
19175_18_MARCKSL1_MARCKSL1 MARCKSL1 chr6 116466215 0.008361 3.57e-36
35353 TRUE reported textfile 2 1 TRUE
958 rs6924874 C T C T -0.0688 -5.00E-04 0.17864 NA FALSE FALSE FALSE
GCST006061 0.0094 0.9584 537409 outcome TRUE reported
19175_18_MARCKSL1_MARCKSL1 MARCKSL1 chr6 113328679 0.010692 1.238e-10
35371 TRUE reported textfile 2 1 TRUE
959 rs80345890 T G T G -0.1768 -0.0049 0.0365 NA FALSE FALSE FALSE
GCST006061 0.0206 0.8137 537409 outcome TRUE reported

```
19175_18_MARCKSL1_MARCKSL1	MARCKSL1	chr9	93219096	0.02164	3.083e-16	35360	TRUE	reported	textfile	2	1	TRUE
960	rs10118718	A	G	A	G	0.0813	-0.0058	0.09976	NA	FALSE	FALSE	FALSE	GCST006061	0.0128	0.6503	537409	outcome	TRUE	reported	2475_1_KIT_SCF_sR	KIT	chr9	106676694	0.013841	4.253e-09	35369	TRUE	reported	textfile	2	1	TRUE
961	rs10444039	A	C	A	C	0.0765	-0.0083	0.10775	NA	FALSE	FALSE	FALSE	GCST006061	0.0109	0.4449	537409	outcome	TRUE	reported	2475_1_KIT_SCF_sR	KIT	chr10	117806027	0.013457	1.309e-08	35376	TRUE	reported	textfile	2	1	TRUE
962	rs10922098	T	C	T	C	-0.1834	-0.009	0.40011	NA	FALSE	FALSE	FALSE	GCST006061	0.0075	0.2275	537409	outcome	TRUE	reported	2475_1_KIT_SCF_sR	KIT	chr1	196695521	0.008482	1.128e-103	35338	TRUE	reported	textfile	2	1	TRUE
963	rs10962594	C	T	C	T	0.0924	-0.0157	0.07597	NA	FALSE	FALSE	FALSE	GCST006061	0.0106	0.1393	537409	outcome	TRUE	reported	2475_1_KIT_SCF_sR	KIT	chr9	16791745	0.015639	3.459e-09	35366	TRUE	reported	textfile	2	1	TRUE
964	rs12489516	T	C	T	C	0.1606	0.0223	0.03572	NA	FALSE	FALSE	FALSE	GCST006061	0.0175	0.2022	537409	outcome	TRUE	reported	2475_1_KIT_SCF_sR	KIT	chr3	148879825	0.022369	6.995e-13	35359	TRUE	reported	textfile	2	1	TRUE
965	rs13095431	T	C	T	C	-0.0778	0.0109	0.32492	NA	FALSE	FALSE	FALSE	GCST006061	0.0082	0.1815	537409	outcome	TRUE	reported	2475_1_KIT_SCF_sR	KIT	chr3	198078659	0.008966	4.042e-18	35187	TRUE	reported	textfile	2	1	TRUE
966	rs1571732	A	G	A	G	0.0716	0.0186	0.32044	NA	FALSE	FALSE	FALSE	GCST006061	0.0079	0.01911	537409	outcome	TRUE	reported	2475_1_KIT_SCF_sR	KIT	chr21	41145632	0.008901	8.69e-16	35364	TRUE	reported	textfile	2	1	TRUE
967	rs1707652	T	C	T	C	0.0814	0.0081	0.3102	NA	FALSE	FALSE	FALSE	GCST006061	0.0074	0.2701	537409	outcome	TRUE	reported	2475_1_KIT_SCF_sR	KIT	chr3	165761011	0.008973	1.176e-19	35339	TRUE	reported	textfile	2	1	TRUE
968	rs17758695	T	C	T	C	-0.1563	7e-04	0.03874	NA	FALSE	FALSE	FALSE	GCST006061	0.0295	0.9814	537409	outcome	TRUE	reported	2475_1_KIT_SCF_sR	KIT	chr18	63253621	0.022172	1.798e-12	35365	TRUE	reported	textfile	2	1	TRUE
969	rs363281	C	T	C	T	-0.0966	0.0103	0.08073	NA	FALSE	FALSE	FALSE	GCST006061	0.0234	0.6593	537409	outcome	TRUE	reported	2475_1_KIT_SCF_sR	KIT	chr10	117277634	0.015242	2.333e-10	35379	TRUE	reported	textfile	2	1	TRUE
970	rs56043070	A	G	A	G	0.127	0.0079	0.06676	NA	FALSE	FALSE	FALSE	GCST006061	0.015	0.5968	537409	outcome	TRUE	reported	2475_1_KIT_SCF_sR	KIT	chr1	247556467	0.016585	1.897e-14	35165	TRUE	reported	textfile	2	1	TRUE
971	rs61913605	T	C	T	C	0.0758	0.0022	0.18833	NA	FALSE	FALSE	FALSE	GCST006061	0.0092	0.8137	537409	outcome	TRUE	reported	2475_1_KIT_SCF_sR	KIT	chr12	12775141	0.010692	1.347e-12	35369	TRUE	reported	textfile	2	1	TRUE
972	rs62543565	A	C	A	C	-0.0934	0.0335	0.33125	NA	FALSE	FALSE	FALSE	GCST006061	0.0074	6.545e-06	537409	outcome	TRUE	reported	2475_1_KIT_SCF_sR	KIT	chr9	16901069	0.008791	2.304e-26	35364	TRUE	reported	textfile	2	1	TRUE
973	rs78744187	T	C	T	C	0.2301	0.017	0.09936	NA	FALSE	FALSE	FALSE	GCST006061	0.0145	0.2421	537409	outcome	TRUE	reported	2475_1_KIT_SCF_sR	KIT	chr19	33263642	0.013886	1.136e-61	35363	TRUE	reported	textfile	2	1	TRUE
974	rs80189144	C	T	C	T	0.0801	-0.0118	0.1137	NA	FALSE	FALSE	FALSE	GCST006061	0.0109	0.2785	537409	outcome	TRUE	reported	2475_1_KIT_SCF_sR	KIT	chr7	73525609	0.013152	1.126e-09	35354	TRUE	reported	textfile	2	1	TRUE
975	rs8051733	G	A	G	A	-0.0512	-0.0156	0.36602	NA	FALSE	FALSE	FALSE	GCST006061	0.0077	0.04393	537409	outcome	TRUE	reported	2475_1_KIT_SCF_sR	KIT	chr16	89957798	0.008633	3.02e-09	35324	TRUE	reported	textfile	2	1	TRUE
```

976 rs10119214 C A C A -0.0446 -0.0299 0.33815 NA FALSE FALSE FALSE GCST006061 0.008 0.0001913 537409 outcome TRUE reported 9282_12_CRISP2_CRIS2 CRISP2 chr9 106814990 0.00782 1.175e-08 35372 TRUE reported textfile 2 1 TRUE

977 rs10993994 G A G A -0.0521 3e-04 0.38221 NA FALSE FALSE FALSE GCST006061 0.0073 0.9655 537409 outcome TRUE reported 9282_12_CRISP2_CRIS2 CRISP2 chr10 46046326 0.007648 9.637e-12 35328 TRUE reported textfile 2 1 TRUE

978 rs112050018 A C A C 0.2701 0.0073 0.01235 NA FALSE FALSE FALSE GCST006061 0.0325 0.8209 537409 outcome TRUE reported 9282_12_CRISP2_CRIS2 CRISP2 chr6 47112023 0.03617 8.175e-14 35355 TRUE reported textfile 2 1 TRUE

979 rs113979133 T C T C -1.3499 0.0105 0.01103 NA FALSE FALSE FALSE GCST006061 0.0298 0.7241 537409 outcome TRUE reported 9282_12_CRISP2_CRIS2 CRISP2 chr6 49842853 0.036712 1e-200 35371 TRUE reported textfile 2 1 TRUE

980 rs114446310 G A G A -0.203 0.0025 0.01744 NA FALSE FALSE FALSE GCST006061 0.0523 0.9622 537409 outcome TRUE reported 9282_12_CRISP2_CRIS2 CRISP2 chr6 61498646 0.03125 8.244e-11 35349 TRUE reported textfile 2 1 TRUE

981 rs114502964 T C T C 0.1512 0.0158 0.02826 NA FALSE FALSE FALSE GCST006061 0.0318 0.6191 537409 outcome TRUE reported 9282_12_CRISP2_CRIS2 CRISP2 chr2 152504284 0.022973 4.649e-11 35345 TRUE reported textfile 2 1 TRUE

982 rs114986602 G A G A -0.194 0.0263 0.01679 NA FALSE FALSE FALSE GCST006061 0.0532 0.6207 537409 outcome TRUE reported 9282_12_CRISP2_CRIS2 CRISP2 chr6 48916522 0.031141 4.672e-10 35360 TRUE reported textfile 2 1 TRUE

983 rs13046908 T G T G -0.0488 0.0025 0.43417 NA FALSE FALSE FALSE GCST006061 0.0073 0.7355 537409 outcome TRUE reported 9282_12_CRISP2_CRIS2 CRISP2 chr21 41869980 0.007467 6.339e-11 35370 TRUE reported textfile 2 1 TRUE

984 rs16881244 A G A G -0.2059 0.0303 0.05858 NA FALSE FALSE FALSE GCST006061 0.0151 0.04429 537409 outcome TRUE reported 9282_12_CRISP2_CRIS2 CRISP2 chr6 51283730 0.017146 3.211e-33 35372 TRUE reported textfile 2 1 TRUE

985 rs17216646 G A G A 0.1361 0.0029 0.06237 NA FALSE FALSE FALSE GCST006061 0.0186 0.8775 537409 outcome TRUE reported 9282_12_CRISP2_CRIS2 CRISP2 chr6 46429358 0.016753 4.508e-16 35359 TRUE reported textfile 2 1 TRUE

986 rs1755785 T C T C -0.0444 0.0028 0.33909 NA FALSE FALSE FALSE GCST006061 0.0076 0.714 537409 outcome TRUE reported 9282_12_CRISP2_CRIS2 CRISP2 chr14 36232596 0.007826 1.401e-08 35358 TRUE reported textfile 2 1 TRUE

987 rs17659760 A G A G 0.3019 -0.002 0.06556 NA FALSE FALSE FALSE GCST006061 0.0167 0.9023 537409 outcome TRUE reported 9282_12_CRISP2_CRIS2 CRISP2 chr6 47995242 0.016373 6.42e-76 35362 TRUE reported textfile 2 1 TRUE

988 rs1779748 C T C T -0.0581 -0.0017 0.4598 NA FALSE FALSE FALSE GCST006061 0.0071 0.8076 537409 outcome TRUE reported 9282_12_CRISP2_CRIS2 CRISP2 chr6 63431804 0.008137 9.303e-13 35351 TRUE reported textfile 2 1 TRUE

989 rs2412640 G A G A 0.0555 -0.0075 0.4442 NA FALSE FALSE FALSE GCST006061 0.0072 0.2926 537409 outcome TRUE reported 9282_12_CRISP2_CRIS2 CRISP2 chr15 41831884 0.007459 1.002e-13 35364 TRUE reported textfile 2 1 TRUE

990 rs2424904 C T C T -0.0528 0.0199 0.40869 NA FALSE FALSE FALSE GCST006061 0.0075 0.008063 537409 outcome TRUE reported 9282_12_CRISP2_CRIS2 CRISP2 chr20 32764510 0.00753 2.345e-12 35364 TRUE reported textfile 2 1 TRUE

991 rs2709396 T C T C 0.1008 -0.0204 0.23917 NA FALSE FALSE FALSE GCST006061 0.0095 0.03225 537409 outcome TRUE reported 9282_12_CRISP2_CRIS2 CRISP2 chr2 207605724 0.008741 9.185e-31 35361 TRUE reported textfile 2 1 TRUE

992 rs2905421 C A C A 0.0526 -0.0113 0.3581 NA FALSE FALSE FALSE GCST006061 0.0076 0.1376 537409 outcome TRUE reported 9282_12_CRISP2_CRIS2

CRISP2 chr19 19347099 0.007728 9.987e-12 35353 TRUE reported
textfile 2 1 TRUE
993 rs3851028 G A G A -0.0751 0.0098 0.13785 NA FALSE FALSE FALSE
GCST006061 0.0104 0.3448 537409 outcome TRUE reported 9282_12_CRISP2_CRIS2
CRISP2 chr19 23254404 0.010744 2.744e-12 35361 TRUE reported
textfile 2 1 TRUE
994 rs59322 A C A C 0.0498 -0.0339 0.45411 NA FALSE FALSE FALSE GCST006061
0.0071 1.775e-06 537409 outcome TRUE reported 9282_12_CRISP2_CRIS2 CRISP2
chr6 87310803 0.008139 9.436e-10 35351 TRUE reported textfile 2
1 TRUE
995 rs62461227 G A G A 0.1167 8e-04 0.34058 NA FALSE FALSE FALSE
GCST006061 0.0077 0.9213 537409 outcome TRUE reported 9282_12_CRISP2_CRIS2
CRISP2 chr7 64236115 0.007796 1.158e-50 35344 TRUE reported
textfile 2 1 TRUE
996 rs6997943 C A C A -0.082 0.0043 0.27685 NA FALSE FALSE FALSE
GCST006061 0.0081 0.5979 537409 outcome TRUE reported 9282_12_CRISP2_CRIS2
CRISP2 chr8 119451368 0.008302 5.236e-23 35361 TRUE reported
textfile 2 1 TRUE
997 rs74987156 T C T C 0.2606 0.0121 0.06108 NA FALSE FALSE FALSE
GCST006061 0.0153 0.4303 537409 outcome TRUE reported 9282_12_CRISP2_CRIS2
CRISP2 chr6 49412097 0.016631 2.444e-55 35359 TRUE reported
textfile 2 1 TRUE
998 rs76237797 A G A G -0.4551 0.017 0.01513 NA FALSE FALSE FALSE
GCST006061 0.046 0.7117 537409 outcome TRUE reported 9282_12_CRISP2_CRIS2
CRISP2 chr6 47951317 0.032724 5.727e-44 35361 TRUE reported
textfile 2 1 TRUE
999 rs78192758 G A G A 0.1877 0.0139 0.02209 NA FALSE FALSE FALSE
GCST006061 0.0359 0.6985 537409 outcome TRUE reported 9282_12_CRISP2_CRIS2
CRISP2 chr6 46247906 0.027967 1.926e-11 35360 TRUE reported
textfile 2 1 TRUE
1000 rs9287979 G A G A -0.0768 0.0044 0.35799 NA FALSE FALSE FALSE
GCST006061 0.0073 0.5441 537409 outcome TRUE reported 9282_12_CRISP2_CRIS2
CRISP2 chr2 151499847 0.007754 4.001e-23 35345 TRUE reported
textfile 2 1 TRUE
1001 rs9351779 C T C T 0.0718 0.0064 0.11469 NA FALSE FALSE FALSE
GCST006061 0.0102 0.5296 537409 outcome TRUE reported 9282_12_CRISP2_CRIS2
CRISP2 chr6 70021686 0.012797 2.013e-08 35339 TRUE reported
textfile 2 1 TRUE
1002 rs1012143 G A G A 0.1247 0.003 0.30014 NA FALSE FALSE FALSE
GCST006061 0.0078 0.6972 537409 outcome TRUE reported 16613_3_CDH17_CAD17
CDH17 chr2 107045885 0.008731 2.842e-46 35355 TRUE reported
textfile 2 1 TRUE
1003 rs11120001 C T C T -0.0733 -0.0126 0.15212 NA FALSE FALSE FALSE
GCST006061 0.0101 0.2115 537409 outcome TRUE reported 16613_3_CDH17_CAD17
CDH17 chr1 212713971 0.011141 4.73e-11 35361 TRUE reported
textfile 2 1 TRUE
1004 rs117543080 A G A G 0.1087 -0.0252 0.05705 NA FALSE FALSE FALSE
GCST006061 0.0325 0.439 537409 outcome TRUE reported 16613_3_CDH17_CAD17
CDH17 chr19 49345940 0.018015 1.6e-09 35379 TRUE reported textfile
2 1 TRUE
1005 rs12791948 G A G A 0.0592 -0.0081 0.17954 NA FALSE FALSE FALSE
GCST006061 0.0095 0.3949 537409 outcome TRUE reported 16613_3_CDH17_CAD17
CDH17 chr11 126473759 0.010464 1.534e-08 35369 TRUE reported
textfile 2 1 TRUE
1006 rs145175118 C T C T 0.1357 0.0497 0.03677 NA FALSE FALSE FALSE
GCST006061 0.0405 0.2193 537409 outcome TRUE reported 16613_3_CDH17_CAD17
CDH17 chr8 91914841 0.021446 2.493e-10 35345 TRUE reported
textfile 2 1 TRUE
1007 rs190161436 G T G T 0.2191 -0.0447 0.01088 NA FALSE FALSE FALSE
GCST006061 0.0394 0.2567 537409 outcome TRUE reported 16613_3_CDH17_CAD17
CDH17 chr19 47858201 0.039512 2.938e-08 35374 TRUE reported
textfile 2 1 TRUE
1008 rs3115920 T C T C -0.15 0.0128 0.05921 NA FALSE FALSE FALSE
GCST006061 0.0169 0.448 537409 outcome TRUE reported 16613_3_CDH17_CAD17
CDH17 chr8 93940980 0.017266 3.708e-18 35351 TRUE reported
textfile 2 1 TRUE

1009 rs3124761 C T C T 0.3395 0.0014 0.09484 NA FALSE FALSE FALSE
GCST006061 0.0106 0.8981 537409 outcome TRUE reported 16613_3_CDH17_CAD17
CDH17 chr9 133474633 0.013846 9.229e-133 35369 TRUE reported
textfile 2 1 TRUE
1010 rs411482 T C T C -0.3198 -0.0057 0.45973 NA FALSE FALSE FALSE
GCST006061 0.008 0.4724 537409 outcome TRUE reported 16613_3_CDH17_CAD17
CDH17 chr19 48600190 0.005697 1e-200 35375 TRUE reported textfile
2 1 TRUE
1011 rs4556017 T C T C -0.1122 -0.022 0.11918 NA FALSE FALSE FALSE
GCST006061 0.0108 0.04182 537409 outcome TRUE reported 16613_3_CDH17_CAD17
CDH17 chr7 100989509 0.012283 6.55e-20 35368 TRUE reported
textfile 2 1 TRUE
1012 rs55714927 T C T C 0.0572 1e-04 0.21166 NA FALSE FALSE FALSE
GCST006061 0.0098 0.9926 537409 outcome TRUE reported 16613_3_CDH17_CAD17
CDH17 chr17 7176997 0.009804 5.397e-09 35370 TRUE reported textfile
2 1 TRUE
1013 rs55941736 G A G A 0.2297 0.0083 0.0238 NA FALSE FALSE FALSE
GCST006061 0.0327 0.8003 537409 outcome TRUE reported 16613_3_CDH17_CAD17
CDH17 chr19 48328629 0.026903 1.364e-17 35377 TRUE reported
textfile 2 1 TRUE
1014 rs590819 T C T C -0.2578 0.0089 0.04463 NA FALSE FALSE FALSE
GCST006061 0.0176 0.6123 537409 outcome TRUE reported 16613_3_CDH17_CAD17
CDH17 chr9 133338901 0.019784 8.205e-39 35371 TRUE reported
textfile 2 1 TRUE
1015 rs61743291 T C T C -0.1785 -0.0197 0.01794 NA FALSE FALSE FALSE
GCST006061 0.0302 0.5143 537409 outcome TRUE reported 16613_3_CDH17_CAD17
CDH17 chr19 47480979 0.030932 7.89e-09 35370 TRUE reported
textfile 2 1 TRUE
1016 rs62578578 A G A G 0.1826 -0.0469 0.03351 NA FALSE FALSE FALSE
GCST006061 0.0292 0.108 537409 outcome TRUE reported 16613_3_CDH17_CAD17
CDH17 chr9 132466382 0.022899 1.533e-15 35372 TRUE reported
textfile 2 1 TRUE
1017 rs7037570 T C T C 0.0677 -0.0086 0.19816 NA FALSE FALSE FALSE
GCST006061 0.0089 0.334 537409 outcome TRUE reported 16613_3_CDH17_CAD17
CDH17 chr9 132420954 0.010282 4.571e-11 35371 TRUE reported
textfile 2 1 TRUE
1018 rs708686 T C T C 0.1392 -4.00E-04 0.23081 NA FALSE FALSE FALSE
GCST006061 0.0081 0.9647 537409 outcome TRUE reported 16613_3_CDH17_CAD17
CDH17 chr19 5840608 0.0098 8.665e-46 35362 TRUE reported textfile 2
1 TRUE
1019 rs76321971 A G A G -0.1184 0.0052 0.09264 NA FALSE FALSE FALSE
GCST006061 0.0151 0.7297 537409 outcome TRUE reported 16613_3_CDH17_CAD17
CDH17 chr19 48276651 0.014268 1.054e-16 35375 TRUE reported
textfile 2 1 TRUE
1020 rs76324624 T C T C 0.1835 0.0364 0.0191 NA FALSE FALSE FALSE
GCST006061 0.036 0.3117 537409 outcome TRUE reported 16613_3_CDH17_CAD17
CDH17 chr9 133166068 0.030612 2.043e-09 35366 TRUE reported
textfile 2 1 TRUE
1021 rs7833351 A G A G -0.2677 0.0064 0.31181 NA FALSE FALSE FALSE
GCST006061 0.0075 0.3949 537409 outcome TRUE reported 16613_3_CDH17_CAD17
CDH17 chr8 94169950 0.00861 1e-200 35351 TRUE reported textfile 2
1 TRUE
1022 rs7839227 T C T C -0.0813 0 0.27942 NA FALSE FALSE FALSE GCST006061
0.0081 0.998 537409 outcome TRUE reported 16613_3_CDH17_CAD17 CDH17 chr8
93239985 0.009046 2.522e-19 35352 TRUE reported textfile 2 1 TRUE
1023 rs79775625 G A G A 0.1803 -0.0158 0.02083 NA FALSE FALSE FALSE
GCST006061 0.0346 0.6481 537409 outcome TRUE reported 16613_3_CDH17_CAD17
CDH17 chr19 46073042 0.028837 4.043e-10 35370 TRUE reported
textfile 2 1 TRUE
1024 rs80206894 G A G A 0.2836 -8.00E-04 0.04853 NA FALSE FALSE FALSE
GCST006061 0.0215 0.972 537409 outcome TRUE reported 16613_3_CDH17_CAD17
CDH17 chr19 48772968 0.019451 3.759e-48 35373 TRUE reported
textfile 2 1 TRUE
1025 rs9676447 T C T C 0.1165 -0.0079 0.05026 NA FALSE FALSE FALSE
GCST006061 0.02 0.6918 537409 outcome TRUE reported 16613_3_CDH17_CAD17
CDH17 chr19 48920990 0.019093 1.049e-09 35378 TRUE reported

textfile 2 1 TRUE
1026 rs9815073 A C A C -0.0544 0.0194 0.32976 NA FALSE FALSE FALSE GCST006061 0.0083 0.01863 537409 outcome TRUE reported 16613_3_CDH17_CAD17 CDH17 chr3 188397894 0.008455 1.241e-10 35373 TRUE reported textfile 2 1 TRUE
1027 rs10121987 G A G A 0.0782 0.0076 0.28723 NA FALSE FALSE FALSE GCST006061 0.0076 0.3168 537409 outcome TRUE reported 5596_75_CD5_CD5 CD5 chr9 33119820 0.008745 3.827e-19 35349 TRUE reported textfile 2 1 TRUE
1028 rs112655189 A C A C -0.1524 -0.008 0.02759 NA FALSE FALSE FALSE GCST006061 0.0339 0.8133 537409 outcome TRUE reported 5596_75_CD5_CD5 CD5 chr3 98225832 0.024792 7.889e-10 35329 TRUE reported textfile 2 1 TRUE
1029 rs114747602 G A G A 0.3066 0.0204 0.02243 NA FALSE FALSE FALSE GCST006061 0.036 0.57 537409 outcome TRUE reported 5596_75_CD5_CD5 CD5 chr3 100221204 0.028358 3.031e-27 35328 TRUE reported textfile 2 1 TRUE
1030 rs114978758 A C A C -0.2823 0.043 0.03083 NA FALSE FALSE FALSE GCST006061 0.0521 0.4093 537409 outcome TRUE reported 5596_75_CD5_CD5 CD5 chr3 100558926 0.023512 3.273e-33 35324 TRUE reported textfile 2 1 TRUE
1031 rs11571364 A G A G 0.198 -2.00E-04 0.04967 NA FALSE FALSE FALSE GCST006061 0.0193 0.9922 537409 outcome TRUE reported 5596_75_CD5_CD5 CD5 chr17 7008372 0.01811 8.017e-28 35374 TRUE reported textfile 2 1 TRUE
1032 rs11718075 C T C T 0.105 0.0084 0.23668 NA FALSE FALSE FALSE GCST006061 0.0089 0.3479 537409 outcome TRUE reported 5596_75_CD5_CD5 CD5 chr3 99439879 0.009762 5.546e-27 35331 TRUE reported textfile 2 1 TRUE
1033 rs13209376 T G T G 0.0726 -0.0321 0.1057 NA FALSE FALSE FALSE GCST006061 0.0131 0.01421 537409 outcome TRUE reported 5596_75_CD5_CD5 CD5 chr6 95622197 0.012861 1.652e-08 35343 TRUE reported textfile 2 1 TRUE
1034 rs150824684 A G A G -0.3022 -0.0731 0.01021 NA FALSE FALSE FALSE GCST006061 0.046 0.1122 537409 outcome TRUE reported 5596_75_CD5_CD5 CD5 chr19 51578388 0.039524 2.074e-14 35384 TRUE reported textfile 2 1 TRUE
1035 rs1866767 T C T C 0.0732 -0.003 0.13186 NA FALSE FALSE FALSE GCST006061 0.0111 0.7879 537409 outcome TRUE reported 5596_75_CD5_CD5 CD5 chr11 134404869 0.011775 5.075e-10 35150 TRUE reported textfile 2 1 TRUE
1036 rs2172259 G A G A 0.2232 0.0227 0.01566 NA FALSE FALSE FALSE GCST006061 0.0405 0.5761 537409 outcome TRUE reported 5596_75_CD5_CD5 CD5 chr3 98081991 0.03327 1.963e-11 35330 TRUE reported textfile 2 1 TRUE
1037 rs3747207 A G A G 0.0535 0.0153 0.22927 NA FALSE FALSE FALSE GCST006061 0.0085 0.07308 537409 outcome TRUE reported 5596_75_CD5_CD5 CD5 chr22 43928975 0.009414 1.324e-08 35372 TRUE reported textfile 2 1 TRUE
1038 rs3804623 C A C A -0.2544 -0.0031 0.49932 NA FALSE FALSE FALSE GCST006061 0.0074 0.678 537409 outcome TRUE reported 5596_75_CD5_CD5 CD5 chr3 98584088 0.008199 1e-200 35330 TRUE reported textfile 2 1 TRUE
1039 rs4841132 G A G A -0.0865 -0.019 0.07459 NA FALSE FALSE FALSE GCST006061 0.0129 0.1429 537409 outcome TRUE reported 5596_75_CD5_CD5 CD5 chr8 9326086 0.015043 8.92e-09 35385 TRUE reported textfile 2 1 TRUE
1040 rs4858926 A G A G -0.0656 0.0041 0.41962 NA FALSE FALSE FALSE GCST006061 0.0076 0.5944 537409 outcome TRUE reported 5596_75_CD5_CD5 CD5 chr3 87981395 0.008448 8.128e-15 35327 TRUE reported textfile 2 1 TRUE
1041 rs55714927 T C T C 0.1841 1e-04 0.21166 NA FALSE FALSE FALSE GCST006061 0.0098 0.9926 537409 outcome TRUE reported 5596_75_CD5_CD5 CD5 chr17 7176997 0.00979 6.857e-79 35370 TRUE reported textfile 2 1 TRUE
1042 rs62269703 A C A C -0.2952 -0.0044 0.04147 NA FALSE FALSE FALSE GCST006061 0.0198 0.8252 537409 outcome TRUE reported 5596_75_CD5_CD5 CD5 chr3 98152103 0.020882 2.262e-45 35331 TRUE reported textfile 2

1 TRUE
1043 rs635634 C T C T 0.2262 0.0285 0.12995 NA FALSE FALSE FALSE GCST006061 0.0089 0.001351 537409 outcome TRUE reported 5596_75_CD5_CD5 CD5 chr9 133279427 0.01176 1.914e-82 35371 TRUE reported textfile 2 1 TRUE
1044 rs73066161 A G A G -0.2576 0.0747 0.01392 NA FALSE FALSE FALSE GCST006061 0.0315 0.01787 537409 outcome TRUE reported 5596_75_CD5_CD5 CD5 chr19 52061867 0.033595 1.749e-14 35388 TRUE reported textfile 2 1 TRUE
1045 rs74890720 C T C T 0.5137 0.0153 0.06122 NA FALSE FALSE FALSE GCST006061 0.0173 0.3761 537409 outcome TRUE reported 5596_75_CD5_CD5 CD5 chr3 98750426 0.017244 5.153e-195 35331 TRUE reported textfile 2 1 TRUE
1046 rs9824576 T C T C -0.0794 -0.0035 0.18802 NA FALSE FALSE FALSE GCST006061 0.0093 0.708 537409 outcome TRUE reported 5596_75_CD5_CD5 CD5 chr3 81652026 0.010596 6.723e-14 35337 TRUE reported textfile 2 1 TRUE
1047 rs10121987 G A G A 0.073 0.0076 0.28723 NA FALSE FALSE FALSE GCST006061 0.0076 0.3168 537409 outcome TRUE reported 9451_20_UMOD_Uromodulin UMOD chr9 33119820 0.008991 4.697e-16 35349 TRUE reported textfile 2 1 TRUE
1048 rs10426 A G A G -0.0689 -0.0104 0.22953 NA FALSE FALSE FALSE GCST006061 0.0091 0.2514 537409 outcome TRUE reported 9451_20_UMOD_Uromodulin UMOD chr19 51014542 0.0099 3.412e-12 35367 TRUE reported textfile 2 1 TRUE
1049 rs1060461 G A G A -0.0568 -0.01 0.28169 NA FALSE FALSE FALSE GCST006061 0.0094 0.2888 537409 outcome TRUE reported 9451_20_UMOD_Uromodulin UMOD chr19 50775737 0.009291 9.748e-10 35362 TRUE reported textfile 2 1 TRUE
1050 rs10894808 T C T C 0.0699 0.0024 0.11933 NA FALSE FALSE FALSE GCST006061 0.0111 0.8297 537409 outcome TRUE reported 9451_20_UMOD_Uromodulin UMOD chr11 134386911 0.012558 2.602e-08 35150 TRUE reported textfile 2 1 TRUE
1051 rs114598716 T C T C 0.1035 0.0117 0.07001 NA FALSE FALSE FALSE GCST006061 0.0196 0.5507 537409 outcome TRUE reported 9451_20_UMOD_Uromodulin UMOD chr3 99603098 0.016634 4.902e-10 35332 TRUE reported textfile 2 1 TRUE
1052 rs117633773 A G A G 0.1464 0.0067 0.04197 NA FALSE FALSE FALSE GCST006061 0.0224 0.7662 537409 outcome TRUE reported 9451_20_UMOD_Uromodulin UMOD chr17 6953365 0.020194 4.18e-13 35374 TRUE reported textfile 2 1 TRUE
1053 rs143501783 C T C T 0.3807 -0.0333 0.01116 NA FALSE FALSE FALSE GCST006061 0.0443 0.4525 537409 outcome TRUE reported 9451_20_UMOD_Uromodulin UMOD chr17 7897001 0.037231 1.525e-24 35373 TRUE reported textfile 2 1 TRUE
1054 rs146900894 A G A G -0.2245 0.0329 0.02139 NA FALSE FALSE FALSE GCST006061 0.0418 0.4318 537409 outcome TRUE reported 9451_20_UMOD_Uromodulin UMOD chr3 94502925 0.029723 4.252e-14 35327 TRUE reported textfile 2 1 TRUE
1055 rs151078915 T C T C -0.1584 -0.0212 0.02589 NA FALSE FALSE FALSE GCST006061 0.0307 0.4898 537409 outcome TRUE reported 9451_20_UMOD_Uromodulin UMOD chr3 86766107 0.026658 2.816e-09 35331 TRUE reported textfile 2 1 TRUE
1056 rs2280806 G A G A 0.1078 0.0033 0.27116 NA FALSE FALSE FALSE GCST006061 0.0091 0.715 537409 outcome TRUE reported 9451_20_UMOD_Uromodulin UMOD chr19 51129600 0.009387 1.58e-30 35371 TRUE reported textfile 2 1 TRUE
1057 rs2691261 A G A G 0.065 -0.0065 0.21585 NA FALSE FALSE FALSE GCST006061 0.0088 0.4617 537409 outcome TRUE reported 9451_20_UMOD_Uromodulin UMOD chr19 51061055 0.010068 1.074e-10 35376 TRUE reported textfile 2 1 TRUE
1058 rs2691273 A G A G -0.2666 1e-04 0.4595 NA FALSE FALSE FALSE GCST006061 0.0075 0.9867 537409 outcome TRUE reported 9451_20_UMOD_Uromodulin UMOD chr19 51092879 0.008225 1e-200 35374 TRUE reported textfile 2 1 TRUE
1059 rs34616839 G A G A -0.0825 0.0072 0.26881 NA FALSE FALSE FALSE

GCST006061 0.0084 0.3881 537409 outcome TRUE reported 9451_20_UMOD_Uromodulin
UMOD chr19 51325796 0.009383 1.468e-18 35379 TRUE reported
textfile 2 1 TRUE
1060 rs3749260 A C A C 0.2945 0.0105 0.10261 NA FALSE FALSE FALSE
GCST006061 0.011 0.3367 537409 outcome TRUE reported 9451_20_UMOD_Uromodulin
UMOD chr3 98532018 0.01409 5.159e-97 35330 TRUE reported textfile
2 1 TRUE
1061 rs3804622 A G A G -0.3598 0.0013 0.42975 NA FALSE FALSE FALSE
GCST006061 0.0072 0.8575 537409 outcome TRUE reported 9451_20_UMOD_Uromodulin
UMOD chr3 98584338 0.00592 1e-200 35330 TRUE reported textfile 2
1 TRUE
1062 rs55714927 T C T C 0.1687 1e-04 0.21166 NA FALSE FALSE FALSE
GCST006061 0.0098 0.9926 537409 outcome TRUE reported 9451_20_UMOD_Uromodulin
UMOD chr17 7176997 0.010067 5.037e-63 35370 TRUE reported textfile
2 1 TRUE
1063 rs56162907 G A G A 0.1716 0.0151 0.02066 NA FALSE FALSE FALSE
GCST006061 0.0322 0.639 537409 outcome TRUE reported 9451_20_UMOD_Uromodulin
UMOD chr3 101897460 0.0294 5.324e-09 35331 TRUE reported textfile
2 1 TRUE
1064 rs635634 C T C T 0.2164 0.0285 0.12995 NA FALSE FALSE FALSE
GCST006061 0.0089 0.001351 537409 outcome TRUE reported
9451_20_UMOD_Uromodulin UMOD chr9 133279427 0.012096 1.406e-71 35371
TRUE reported textfile 2 1 TRUE
1065 rs6777831 A C A C -0.0651 -0.0033 0.19042 NA FALSE FALSE FALSE
GCST006061 0.009 0.712 537409 outcome TRUE reported 9451_20_UMOD_Uromodulin
UMOD chr3 81549380 0.010674 1.066e-09 35337 TRUE reported
textfile 2 1 TRUE
1066 rs73132003 G A G A -0.1202 0.0203 0.06575 NA FALSE FALSE FALSE
GCST006061 0.0187 0.276 537409 outcome TRUE reported 9451_20_UMOD_Uromodulin
UMOD chr3 87438467 0.017262 3.326e-12 35328 TRUE reported
textfile 2 1 TRUE
1067 rs77050848 G A G A -0.2361 -0.0059 0.03079 NA FALSE FALSE FALSE
GCST006061 0.0294 0.8417 537409 outcome TRUE reported 9451_20_UMOD_Uromodulin
UMOD chr3 100309356 0.024637 9.43e-22 35328 TRUE reported
textfile 2 1 TRUE
1068 rs78661192 T C T C 0.1564 0.0083 0.03426 NA FALSE FALSE FALSE
GCST006061 0.0505 0.8699 537409 outcome TRUE reported 9451_20_UMOD_Uromodulin
UMOD chr3 96014683 0.023354 2.127e-11 35326 TRUE reported
textfile 2 1 TRUE
1069 rs9987289 G A G A -0.088 -0.0155 0.07459 NA FALSE FALSE FALSE
GCST006061 0.0128 0.2254 537409 outcome TRUE reported 9451_20_UMOD_Uromodulin
UMOD chr8 9325848 0.015477 1.301e-08 35385 TRUE reported textfile
2 1 TRUE
1070 rs10123394 C T C T 0.0617 0.0072 0.13412 NA FALSE FALSE FALSE
GCST006061 0.0105 0.4923 537409 outcome TRUE reported 3290_50_CD109_CD109
CD109 chr9 133801438 0.011287 4.591e-08 35372 TRUE reported
textfile 2 1 TRUE
1071 rs10241439 T G T G -0.0749 0.0327 0.09048 NA FALSE FALSE FALSE
GCST006061 0.0121 0.006783 537409 outcome TRUE reported 3290_50_CD109_CD109
CD109 chr7 14807238 0.013163 1.268e-08 35363 TRUE reported
textfile 2 1 TRUE
1072 rs113501304 T G T G 0.197 -0.0801 0.02511 NA FALSE FALSE FALSE
GCST006061 0.0395 0.04252 537409 outcome TRUE reported 3290_50_CD109_CD109
CD109 chr6 75295536 0.025656 1.609e-14 35341 TRUE reported
textfile 2 1 TRUE
1073 rs117247182 C A C A -0.1248 -0.0245 0.03678 NA FALSE FALSE FALSE
GCST006061 0.0216 0.2569 537409 outcome TRUE reported 3290_50_CD109_CD109
CD109 chr6 86028658 0.021333 4.909e-09 35346 TRUE reported
textfile 2 1 TRUE
1074 rs138578493 T G T G 0.4807 -0.0309 0.02584 NA FALSE FALSE FALSE
GCST006061 0.0247 0.2102 537409 outcome TRUE reported 3290_50_CD109_CD109
CD109 chr6 74661203 0.025071 6.137e-82 35343 TRUE reported
textfile 2 1 TRUE
1075 rs139277266 A G A G -0.2606 -0.011 0.0274 NA FALSE FALSE FALSE
GCST006061 0.0297 0.712 537409 outcome TRUE reported 3290_50_CD109_CD109
CD109 chr6 73863449 0.024262 6.515e-27 35346 TRUE reported

textfile 2 1 TRUE
1076 rs140072581 G T G T -0.1617 -0.0592 0.03154 NA FALSE FALSE FALSE
GCST006061 0.0337 0.07834 537409 outcome TRUE reported 3290_50_CD109_CD109
CD109 chr6 71976741 0.022792 1.296e-12 35336 TRUE reported
textfile 2 1 TRUE
1077 rs140383761 C T C T -0.1946 -0.0241 0.02557 NA FALSE FALSE FALSE
GCST006061 0.0299 0.4199 537409 outcome TRUE reported 3290_50_CD109_CD109
CD109 chr6 84189984 0.025247 1.279e-14 35337 TRUE reported
textfile 2 1 TRUE
1078 rs141698681 T C T C 0.267 -0.0395 0.01789 NA FALSE FALSE FALSE
GCST006061 0.0321 0.2186 537409 outcome TRUE reported 3290_50_CD109_CD109
CD109 chr6 71140045 0.03011 7.474e-19 35332 TRUE reported textfile
2 1 TRUE
1079 rs141909149 A C A C -0.4728 0.0069 0.01289 NA FALSE FALSE FALSE
GCST006061 0.0474 0.8837 537409 outcome TRUE reported 3290_50_CD109_CD109
CD109 chr6 76509084 0.035465 1.519e-40 35343 TRUE reported
textfile 2 1 TRUE
1080 rs143249580 C T C T -0.2656 0.0033 0.0125 NA FALSE FALSE FALSE
GCST006061 0.0382 0.931 537409 outcome TRUE reported 3290_50_CD109_CD109
CD109 chr6 86599400 0.037423 1.273e-12 35346 TRUE reported
textfile 2 1 TRUE
1081 rs506317 T C T C -0.0472 -0.0159 0.43587 NA FALSE FALSE FALSE
GCST006061 0.0075 0.03311 537409 outcome TRUE reported 3290_50_CD109_CD109
CD109 chr1 196748023 0.007649 6.785e-10 35339 TRUE reported
textfile 2 1 TRUE
1082 rs55714927 T C T C 0.0643 1e-04 0.21166 NA FALSE FALSE FALSE
GCST006061 0.0098 0.9926 537409 outcome TRUE reported 3290_50_CD109_CD109
CD109 chr17 7176997 0.009347 6.033e-12 35370 TRUE reported textfile
2 1 TRUE
1083 rs61313163 G T G T 0.1361 -0.0026 0.042 NA FALSE FALSE FALSE
GCST006061 0.0186 0.8894 537409 outcome TRUE reported 3290_50_CD109_CD109
CD109 chr6 74852570 0.01974 5.402e-12 35343 TRUE reported textfile
2 1 TRUE
1084 rs62578578 A G A G 0.1508 -0.0469 0.03351 NA FALSE FALSE FALSE
GCST006061 0.0292 0.108 537409 outcome TRUE reported 3290_50_CD109_CD109
CD109 chr9 132466382 0.021487 2.247e-12 35372 TRUE reported
textfile 2 1 TRUE
1085 rs72961725 C A C A 0.1285 0.0091 0.03184 NA FALSE FALSE FALSE
GCST006061 0.0292 0.756 537409 outcome TRUE reported 3290_50_CD109_CD109
CD109 chr6 54693891 0.022628 1.356e-08 35356 TRUE reported
textfile 2 1 TRUE
1086 rs739468 G T G T 0.2162 0.0025 0.0968 NA FALSE FALSE FALSE
GCST006061 0.0104 0.8106 537409 outcome TRUE reported 3290_50_CD109_CD109
CD109 chr9 133461126 0.012933 9.863e-63 35369 TRUE reported
textfile 2 1 TRUE
1087 rs76081669 T C T C -0.2146 -0.0037 0.04658 NA FALSE FALSE FALSE
GCST006061 0.025 0.8815 537409 outcome TRUE reported 3290_50_CD109_CD109
CD109 chr6 73648334 0.018765 2.754e-30 35346 TRUE reported
textfile 2 1 TRUE
1088 rs76232494 G T G T -0.1453 -0.0035 0.02693 NA FALSE FALSE FALSE
GCST006061 0.0317 0.9131 537409 outcome TRUE reported 3290_50_CD109_CD109
CD109 chr6 75609773 0.023905 1.216e-09 35342 TRUE reported
textfile 2 1 TRUE
1089 rs7763514 T C T C -0.1366 0.0053 0.42251 NA FALSE FALSE FALSE
GCST006061 0.0074 0.4803 537409 outcome TRUE reported 3290_50_CD109_CD109
CD109 chr6 73168073 0.00803 6.754e-65 35338 TRUE reported textfile
2 1 TRUE
1090 rs79495888 A G A G 0.1173 0.0113 0.03851 NA FALSE FALSE FALSE
GCST006061 0.0274 0.6805 537409 outcome TRUE reported 3290_50_CD109_CD109
CD109 chr6 68585694 0.020854 1.858e-08 35338 TRUE reported
textfile 2 1 TRUE
1091 rs8176693 T C T C 0.4551 0.0144 0.06484 NA FALSE FALSE FALSE
GCST006061 0.0129 0.2638 537409 outcome TRUE reported 3290_50_CD109_CD109
CD109 chr9 133262254 0.015443 7.054e-191 35371 TRUE reported
textfile 2 1 TRUE
1092 rs10123570 T C T C 0.0459 -0.0099 0.48298 NA FALSE FALSE FALSE

GCST006061 0.0072 0.168 537409 outcome TRUE reported 3516_60_CXCL12_SDF_1
CXCL12 chr9 86741851 0.008224 2.389e-08 35356 TRUE reported
textfile 2 1 TRUE
1093 rs1023264 T C T C -0.1449 0.0112 0.25831 NA FALSE FALSE FALSE
GCST006061 0.0077 0.1465 537409 outcome TRUE reported 3516_60_CXCL12_SDF_1
CXCL12 chr10 44398308 0.009377 7.164e-54 35323 TRUE reported
textfile 2 1 TRUE
1094 rs10762477 G A G A 0.0792 -0.0243 0.15481 NA FALSE FALSE FALSE
GCST006061 0.0098 0.01289 537409 outcome TRUE reported 3516_60_CXCL12_SDF_1
CXCL12 chr10 71771312 0.011422 4.084e-12 35375 TRUE reported
textfile 2 1 TRUE
1095 rs10793518 G A G A 0.0666 0.0102 0.22326 NA FALSE FALSE FALSE
GCST006061 0.0091 0.2606 537409 outcome TRUE reported 3516_60_CXCL12_SDF_1
CXCL12 chr10 44015012 0.009874 1.529e-11 35323 TRUE reported
textfile 2 1 TRUE
1096 rs13015258 G T G T -0.0514 -0.0045 0.3882 NA FALSE FALSE FALSE
GCST006061 0.0075 0.5489 537409 outcome TRUE reported 3516_60_CXCL12_SDF_1
CXCL12 chr2 162074215 0.008477 1.335e-09 35341 TRUE reported
textfile 2 1 TRUE
1097 rs17138667 A G A G 0.0859 -0.0073 0.21392 NA FALSE FALSE FALSE
GCST006061 0.0089 0.4136 537409 outcome TRUE reported 3516_60_CXCL12_SDF_1
CXCL12 chr5 116015527 0.009994 8.302e-18 35351 TRUE reported
textfile 2 1 TRUE
1098 rs266085 T C T C 0.0612 -0.0031 0.32235 NA FALSE FALSE FALSE
GCST006061 0.0074 0.6799 537409 outcome TRUE reported 3516_60_CXCL12_SDF_1
CXCL12 chr10 44378805 0.008827 4.117e-12 35323 TRUE reported
textfile 2 1 TRUE
1099 rs3184504 C T C T -0.0498 0.0074 0.37862 NA FALSE FALSE FALSE
GCST006061 0.0075 0.3235 537409 outcome TRUE reported 3516_60_CXCL12_SDF_1
CXCL12 chr12 111446804 0.008487 4.408e-09 35371 TRUE reported
textfile 2 1 TRUE
1100 rs77542162 G A G A 0.3947 0.0177 0.025 NA FALSE FALSE FALSE
GCST006061 0.0302 0.557 537409 outcome TRUE reported 3516_60_CXCL12_SDF_1
CXCL12 chr17 69085137 0.025875 1.55e-52 35371 TRUE reported
textfile 2 1 TRUE
1101 rs10124390 A C A C 0.0401 -0.0023 0.49223 NA FALSE FALSE FALSE
GCST006061 0.0071 0.7465 537409 outcome TRUE reported 7628_40_CRELD1_CREL1
CRELD1 chr9 83935024 0.007139 1.944e-08 35363 TRUE reported
textfile 2 1 TRUE
1102 rs114286445 A G A G -0.7222 0.0501 0.01033 NA FALSE FALSE FALSE
GCST006061 0.0349 0.1515 537409 outcome TRUE reported 7628_40_CRELD1_CREL1
CRELD1 chr3 9944420 0.039323 2.462e-75 35371 TRUE reported textfile
2 1 TRUE
1103 rs1180047 G T G T 0.3229 -0.028 0.02247 NA FALSE FALSE FALSE
GCST006061 0.0453 0.537 537409 outcome TRUE reported 7628_40_CRELD1_CREL1
CRELD1 chr3 10247066 0.026453 2.866e-34 35372 TRUE reported
textfile 2 1 TRUE
1104 rs140614750 T C T C -0.7232 0.0147 0.01171 NA FALSE FALSE FALSE
GCST006061 0.0253 0.561 537409 outcome TRUE reported 7628_40_CRELD1_CREL1
CRELD1 chr3 10152105 0.037309 1.055e-83 35369 TRUE reported
textfile 2 1 TRUE
1105 rs142299532 A C A C -0.3325 -0.0057 0.03818 NA FALSE FALSE FALSE
GCST006061 0.0247 0.8176 537409 outcome TRUE reported 7628_40_CRELD1_CREL1
CRELD1 chr3 10193609 0.020496 3.487e-59 35371 TRUE reported
textfile 2 1 TRUE
1106 rs142444285 T C T C 0.3381 0.0609 0.01345 NA FALSE FALSE FALSE
GCST006061 0.0686 0.3745 537409 outcome TRUE reported 7628_40_CRELD1_CREL1
CRELD1 chr3 9815219 0.033469 5.423e-24 35370 TRUE reported textfile
2 1 TRUE
1107 rs144363827 A G A G 0.5604 -0.0133 0.02497 NA FALSE FALSE FALSE
GCST006061 0.034 0.6954 537409 outcome TRUE reported 7628_40_CRELD1_CREL1
CRELD1 chr3 10010880 0.025285 7.748e-109 35372 TRUE reported
textfile 2 1 TRUE
1108 rs17537486 G A G A 0.5561 -0.0122 0.03499 NA FALSE FALSE FALSE
GCST006061 0.0257 0.6347 537409 outcome TRUE reported 7628_40_CRELD1_CREL1
CRELD1 chr3 10115548 0.021297 2.652e-150 35373 TRUE reported

textfile 2 1 TRUE
1109 rs34684179 A G A G -0.6417 -0.0134 0.03482 NA FALSE FALSE FALSE GCST006061 0.0236 0.5707 537409 outcome TRUE reported 7628_40_CRELD1_CREL1 CRELD1 chr3 10031360 0.021534 3.922e-195 35372 TRUE reported textfile 2 1 TRUE
1110 rs57153389 A G A G -0.2633 -0.0198 0.04045 NA FALSE FALSE FALSE GCST006061 0.0188 0.2918 537409 outcome TRUE reported 7628_40_CRELD1_CREL1 CRELD1 chr3 9216231 0.020397 4.03e-38 35370 TRUE reported textfile 2 1 TRUE
1111 rs77602078 C A C A 0.2346 0.0188 0.01432 NA FALSE FALSE FALSE GCST006061 0.044 0.6685 537409 outcome TRUE reported 7628_40_CRELD1_CREL1 CRELD1 chr3 10434057 0.033535 2.639e-12 35372 TRUE reported textfile 2 1 TRUE
1112 rs10127790 T C T C -0.0582 -0.0021 0.29968 NA FALSE FALSE FALSE GCST006061 0.0081 0.796 537409 outcome TRUE reported 2811_27_ANGPT1_Angiopoietin_1 ANGPT1 chr1 109348511 0.008847 4.754e-11 35345 TRUE reported textfile 2 1 TRUE
1113 rs10822155 A C A C 0.0572 -0.0472 0.43479 NA FALSE FALSE FALSE GCST006061 0.0072 4.622e-11 537409 outcome TRUE reported 2811_27_ANGPT1_Angiopoietin_1 ANGPT1 chr10 63311455 0.008163 2.437e-12 35351 TRUE reported textfile 2 1 TRUE
1114 rs1522745 C A C A 0.0485 0.0038 0.36905 NA FALSE FALSE FALSE GCST006061 0.0076 0.6198 537409 outcome TRUE reported 2811_27_ANGPT1_Angiopoietin_1 ANGPT1 chr15 64895658 0.008407 7.981e-09 35362 TRUE reported textfile 2 1 TRUE
1115 rs61978204 T C T C 0.075 -0.0056 0.0977 NA FALSE FALSE FALSE GCST006061 0.0137 0.6837 537409 outcome TRUE reported 2811_27_ANGPT1_Angiopoietin_1 ANGPT1 chr14 70175820 0.013721 4.605e-08 35359 TRUE reported textfile 2 1 TRUE
1116 rs72686539 T C T C -0.0581 -0.0132 0.20121 NA FALSE FALSE FALSE GCST006061 0.0096 0.1686 537409 outcome TRUE reported 2811_27_ANGPT1_Angiopoietin_1 ANGPT1 chr8 106989654 0.010161 1.079e-08 35345 TRUE reported textfile 2 1 TRUE
1117 rs10127790 T C T C -0.0506 -0.0021 0.29968 NA FALSE FALSE FALSE GCST006061 0.0081 0.796 537409 outcome TRUE reported 15427_35_LOXL3_LOXL3 LOXL3 chr1 109348511 0.008746 7.238e-09 35345 TRUE reported textfile 2 1 TRUE
1118 rs10822155 A C A C 0.0663 -0.0472 0.43479 NA FALSE FALSE FALSE GCST006061 0.0072 4.622e-11 537409 outcome TRUE reported 15427_35_LOXL3_LOXL3 LOXL3 chr10 63311455 0.008083 2.355e-16 35351 TRUE reported textfile 2 1 TRUE
1119 rs115745987 A G A G -0.2272 0.0198 0.01907 NA FALSE FALSE FALSE GCST006061 0.0332 0.5506 537409 outcome TRUE reported 15427_35_LOXL3_LOXL3 LOXL3 chr13 97639918 0.03011 4.497e-14 35358 TRUE reported textfile 2 1 TRUE
1120 rs145897705 A G A G -0.42 -0.0169 0.01919 NA FALSE FALSE FALSE GCST006061 0.027 0.5322 537409 outcome TRUE reported 15427_35_LOXL3_LOXL3 LOXL3 chr13 95636436 0.029774 3.47e-45 35369 TRUE reported textfile 2 1 TRUE
1121 rs1678388 A G A G 0.1537 -0.0019 0.19822 NA FALSE FALSE FALSE GCST006061 0.0086 0.83 537409 outcome TRUE reported 15427_35_LOXL3_LOXL3 LOXL3 chr13 95201526 0.010264 1.071e-50 35372 TRUE reported textfile 2 1 TRUE
1122 rs17268589 A G A G -0.4008 0.0183 0.106 NA FALSE FALSE FALSE GCST006061 0.0132 0.1645 537409 outcome TRUE reported 15427_35_LOXL3_LOXL3 LOXL3 chr13 95783697 0.013166 1e-200 35370 TRUE reported textfile 2 1 TRUE
1123 rs2576976 C T C T -0.1407 -0.0177 0.1343 NA FALSE FALSE FALSE GCST006061 0.0112 0.1144 537409 outcome TRUE reported 15427_35_LOXL3_LOXL3 LOXL3 chr12 103916450 0.011798 8.721e-33 35361 TRUE reported textfile 2 1 TRUE
1124 rs5174 T C T C -0.0688 0.0087 0.43466 NA FALSE FALSE FALSE GCST006061 0.0075 0.2491 537409 outcome TRUE reported 15427_35_LOXL3_LOXL3 LOXL3 chr1 53247055 0.008128 2.567e-17 35374 TRUE reported textfile 2 1 TRUE
1125 rs61966959 A G A G 0.1821 -0.0064 0.02405 NA FALSE FALSE FALSE

GCST006061 0.0335 0.8487 537409 outcome TRUE reported 15427_35_LOXL3_LOXL3
LOXL3 chr13 96386801 0.026162 3.39e-12 35370 TRUE reported
textfile 2 1 TRUE
1126 rs6897795 A G A G -0.0768 0.0047 0.10004 NA FALSE FALSE FALSE
GCST006061 0.0104 0.6533 537409 outcome TRUE reported 15427_35_LOXL3_LOXL3
LOXL3 chr5 178179418 0.013304 7.8e-09 35363 TRUE reported textfile
2 1 TRUE
1127 rs76373179 G A G A -0.4323 -0.0211 0.03902 NA FALSE FALSE FALSE
GCST006061 0.0288 0.4643 537409 outcome TRUE reported 15427_35_LOXL3_LOXL3
LOXL3 chr13 96503748 0.021471 3.73e-90 35372 TRUE reported
textfile 2 1 TRUE
1128 rs10127790 T C T C -0.0579 -0.0021 0.29968 NA FALSE FALSE FALSE
GCST006061 0.0081 0.796 537409 outcome TRUE reported 6064_4_ERP44_TXNDC4
ERP44 chr1 109348511 0.008916 8.372e-11 35345 TRUE reported
textfile 2 1 TRUE
1129 rs10822155 A C A C 0.0663 -0.0472 0.43479 NA FALSE FALSE FALSE
GCST006061 0.0072 4.622e-11 537409 outcome TRUE reported 6064_4_ERP44_TXNDC4
ERP44 chr10 63311455 0.008223 7.434e-16 35351 TRUE reported
textfile 2 1 TRUE
1130 rs114587946 A G A G 0.1266 -0.0024 0.03551 NA FALSE FALSE FALSE
GCST006061 0.0312 0.9392 537409 outcome TRUE reported 6064_4_ERP44_TXNDC4
ERP44 chr2 32980739 0.02213 1.061e-08 35364 TRUE reported textfile
2 1 TRUE
1131 rs11896749 T C T C -0.0848 0.0062 0.11848 NA FALSE FALSE FALSE
GCST006061 0.0115 0.5897 537409 outcome TRUE reported 6064_4_ERP44_TXNDC4
ERP44 chr2 33072862 0.012578 1.561e-11 35363 TRUE reported
textfile 2 1 TRUE
1132 rs143997020 C T C T -0.1144 -0.0175 0.04239 NA FALSE FALSE FALSE
GCST006061 0.02 0.3819 537409 outcome TRUE reported 6064_4_ERP44_TXNDC4
ERP44 chr2 33331531 0.020351 1.894e-08 35363 TRUE reported
textfile 2 1 TRUE
1133 rs10127894 A G A G -0.0493 0.0102 0.31298 NA FALSE FALSE FALSE
GCST006061 0.0076 0.1775 537409 outcome TRUE reported 6168_11_TP53_p53_R175H
TP53 chr1 201975086 0.008769 1.889e-08 35357 TRUE reported
textfile 2 1 TRUE
1134 rs10922098 T C T C -0.183 -0.009 0.40011 NA FALSE FALSE FALSE
GCST006061 0.0075 0.2275 537409 outcome TRUE reported 6168_11_TP53_p53_R175H
TP53 chr1 196695521 0.008206 3.533e-110 35338 TRUE reported
textfile 2 1 TRUE
1135 rs117247004 A G A G -0.1821 -0.0574 0.02087 NA FALSE FALSE FALSE
GCST006061 0.0448 0.2 537409 outcome TRUE reported 6168_11_TP53_p53_R175H
TP53 chr20 39709522 0.028121 9.438e-11 35376 TRUE reported
textfile 2 1 TRUE
1136 rs11906988 G T G T -0.3209 -0.0389 0.01085 NA FALSE FALSE FALSE
GCST006061 0.0283 0.1697 537409 outcome TRUE reported 6168_11_TP53_p53_R175H
TP53 chr20 38383368 0.038316 5.516e-17 35372 TRUE reported
textfile 2 1 TRUE
1137 rs1707652 T C T C 0.1204 0.0081 0.3102 NA FALSE FALSE FALSE
GCST006061 0.0074 0.2701 537409 outcome TRUE reported 6168_11_TP53_p53_R175H
TP53 chr3 165761011 0.008691 1.211e-43 35339 TRUE reported
textfile 2 1 TRUE
1138 rs2232613 T C T C -0.3426 0.0151 0.0989 NA FALSE FALSE FALSE
GCST006061 0.0141 0.2834 537409 outcome TRUE reported 6168_11_TP53_p53_R175H
TP53 chr20 38369011 0.013231 7.844e-148 35375 TRUE reported
textfile 2 1 TRUE
1139 rs56317765 T C T C 0.0753 -0.0069 0.15401 NA FALSE FALSE FALSE
GCST006061 0.0104 0.5072 537409 outcome TRUE reported 6168_11_TP53_p53_R175H
TP53 chr20 37489853 0.011209 1.843e-11 35369 TRUE reported
textfile 2 1 TRUE
1140 rs10128996 A G A G 0.2886 -0.0026 0.41131 NA FALSE FALSE FALSE
GCST006061 0.0073 0.72 537409 outcome TRUE reported
9394_19_CPQ_Aminopeptidase CPQ chr12 101687720 0.008245 1e-200 35363 TRUE
reported textfile 2 1 TRUE
1141 rs11111203 G A G A 0.1153 -0.0234 0.15597 NA FALSE FALSE FALSE
GCST006061 0.01 0.02008 537409 outcome TRUE reported
9394_19_CPQ_Aminopeptidase CPQ chr12 102199966 0.011297 1.851e-24 35364

TRUE reported textfile 2 1 TRUE
1142 rs111581606 T C T C 0.1329 0.0019 0.04445 NA FALSE FALSE FALSE
GCST006061 0.0181 0.9143 537409 outcome TRUE reported
9394_19_CPQ_Aminopeptidase CPQ chr12 101368399 0.020062 3.485e-11 35363
TRUE reported textfile 2 1 TRUE
1143 rs111695502 C T C T -0.2793 0.0777 0.01389 NA FALSE FALSE FALSE
GCST006061 0.0532 0.1442 537409 outcome TRUE reported
9394_19_CPQ_Aminopeptidase CPQ chr8 93157356 0.035463 3.387e-15 35352
TRUE reported textfile 2 1 TRUE
1144 rs116876243 A G A G -0.3972 -0.0213 0.0111 NA FALSE FALSE FALSE
GCST006061 0.0466 0.6482 537409 outcome TRUE reported
9394_19_CPQ_Aminopeptidase CPQ chr8 101009357 0.039699 1.443e-23 35352
TRUE reported textfile 2 1 TRUE
1145 rs116908064 A G A G 0.2238 -0.008 0.02175 NA FALSE FALSE FALSE
GCST006061 0.0418 0.8479 537409 outcome TRUE reported
9394_19_CPQ_Aminopeptidase CPQ chr12 101919559 0.028181 1.997e-15 35364
TRUE reported textfile 2 1 TRUE
1146 rs13135092 G A G A -0.1626 -0.0083 0.02369 NA FALSE FALSE FALSE
GCST006061 0.0143 0.5607 537409 outcome TRUE reported
9394_19_CPQ_Aminopeptidase CPQ chr4 102276925 0.02693 1.562e-09 35343 TRUE
reported textfile 2 1 TRUE
1147 rs142439242 G A G A -1.1754 -0.0039 0.02776 NA FALSE FALSE FALSE
GCST006061 0.0395 0.9209 537409 outcome TRUE reported
9394_19_CPQ_Aminopeptidase CPQ chr8 96713932 0.018436 1e-200 35363 TRUE
reported textfile 2 1 TRUE
1148 rs143346386 T C T C -0.182 0.0384 0.01939 NA FALSE FALSE FALSE
GCST006061 0.0455 0.3983 537409 outcome TRUE reported
9394_19_CPQ_Aminopeptidase CPQ chr8 89418058 0.029506 6.901e-10 35346
TRUE reported textfile 2 1 TRUE
1149 rs145704203 T C T C -0.2824 0.0192 0.02017 NA FALSE FALSE FALSE
GCST006061 0.0312 0.5375 537409 outcome TRUE reported
9394_19_CPQ_Aminopeptidase CPQ chr8 97808404 0.030813 4.961e-20 35353
TRUE reported textfile 2 1 TRUE
1150 rs149181595 C A C A 0.1497 0.0348 0.03196 NA FALSE FALSE FALSE
GCST006061 0.0236 0.1412 537409 outcome TRUE reported
9394_19_CPQ_Aminopeptidase CPQ chr15 43393609 0.022738 4.587e-11 35363
TRUE reported textfile 2 1 TRUE
1151 rs149794999 T C T C 0.2414 -0.047 0.01101 NA FALSE FALSE FALSE
GCST006061 0.0525 0.3704 537409 outcome TRUE reported
9394_19_CPQ_Aminopeptidase CPQ chr8 100779483 0.039004 6.048e-10 35352
TRUE reported textfile 2 1 TRUE
1152 rs2272662 C T C T -0.0579 0.0109 0.35723 NA FALSE FALSE FALSE
GCST006061 0.0083 0.1873 537409 outcome TRUE reported
9394_19_CPQ_Aminopeptidase CPQ chr8 144414342 0.008801 4.749e-11 34529
TRUE reported textfile 2 1 TRUE
1153 rs3134374 G A G A 0.3188 0.0411 0.01322 NA FALSE FALSE FALSE
GCST006061 0.0362 0.2566 537409 outcome TRUE reported
9394_19_CPQ_Aminopeptidase CPQ chr8 100906631 0.037173 9.821e-18 35351
TRUE reported textfile 2 1 TRUE
1154 rs55671178 T C T C -0.3727 -0.0441 0.02134 NA FALSE FALSE FALSE
GCST006061 0.0296 0.1363 537409 outcome TRUE reported
9394_19_CPQ_Aminopeptidase CPQ chr8 96251611 0.029235 3.185e-37 35357
TRUE reported textfile 2 1 TRUE
1155 rs72680193 A C A C -0.5964 0.0032 0.03697 NA FALSE FALSE FALSE
GCST006061 0.0213 0.8814 537409 outcome TRUE reported
9394_19_CPQ_Aminopeptidase CPQ chr8 96645925 0.021933 8.134e-163 35363
TRUE reported textfile 2 1 TRUE
1156 rs72729610 G A G A -0.059 0.0247 0.18517 NA FALSE FALSE FALSE
GCST006061 0.0101 0.01451 537409 outcome TRUE reported
9394_19_CPQ_Aminopeptidase CPQ chr4 153269813 0.010412 1.455e-08 35376
TRUE reported textfile 2 1 TRUE
1157 rs75627662 T C T C -0.0557 0.0013 0.20205 NA FALSE FALSE FALSE
GCST006061 0.0092 0.8903 537409 outcome TRUE reported
9394_19_CPQ_Aminopeptidase CPQ chr19 44910319 0.01014 3.946e-08 35367 TRUE
reported textfile 2 1 TRUE
1158 rs79884132 T C T C 0.1901 0.0148 0.02359 NA FALSE FALSE FALSE

GCST006061 0.0242 0.54 537409 outcome TRUE reported
9394_19_CPQ_Aminopeptidase CPQ chr8 97352526 0.028397 2.167e-11 35360
TRUE reported textfile 2 1 TRUE

1159 rs10128996 A G A G 0.2619 -0.0026 0.41131 NA FALSE FALSE FALSE
GCST006061 0.0073 0.72 537409 outcome TRUE reported 9357_4_CREG1_CREG1
CREG1 chr12 101687720 0.008309 1e-200 35363 TRUE reported textfile
2 1 TRUE

1160 rs11111203 G A G A 0.1086 -0.0234 0.15597 NA FALSE FALSE FALSE
GCST006061 0.01 0.02008 537409 outcome TRUE reported 9357_4_CREG1_CREG1
CREG1 chr12 102199966 0.011353 1.118e-21 35364 TRUE reported
textfile 2 1 TRUE

1161 rs117483006 T C T C -0.3135 0.0115 0.01325 NA FALSE FALSE FALSE
GCST006061 0.0427 0.787 537409 outcome TRUE reported 9357_4_CREG1_CREG1
CREG1 chr12 99001366 0.037956 1.462e-16 35369 TRUE reported
textfile 2 1 TRUE

1162 rs1229429 T C T C -0.1007 0.0051 0.27857 NA FALSE FALSE FALSE
GCST006061 0.0079 0.5228 537409 outcome TRUE reported 9357_4_CREG1_CREG1
CREG1 chr1 167535829 0.009088 1.556e-28 35359 TRUE reported
textfile 2 1 TRUE

1163 rs12313323 T C T C 0.1211 0.0134 0.05983 NA FALSE FALSE FALSE
GCST006061 0.0175 0.4424 537409 outcome TRUE reported 9357_4_CREG1_CREG1
CREG1 chr12 102746329 0.017357 3.012e-12 35365 TRUE reported
textfile 2 1 TRUE

1164 rs34894639 T C T C -0.0777 0.0216 0.23112 NA FALSE FALSE FALSE
GCST006061 0.0087 0.01271 537409 outcome TRUE reported 9357_4_CREG1_CREG1
CREG1 chr3 136079816 0.009699 1.138e-15 35361 TRUE reported
textfile 2 1 TRUE

1165 rs429358 C T C T -0.0711 0.005 0.17407 NA FALSE FALSE FALSE
GCST006061 0.0103 0.6319 537409 outcome TRUE reported 9357_4_CREG1_CREG1
CREG1 chr19 44908684 0.010841 5.43e-11 35368 TRUE reported
textfile 2 1 TRUE

1166 rs56255430 C A C A 0.1486 -0.0034 0.0795 NA FALSE FALSE FALSE
GCST006061 0.0132 0.7997 537409 outcome TRUE reported 9357_4_CREG1_CREG1
CREG1 chr19 19367068 0.015143 9.878e-23 35353 TRUE reported
textfile 2 1 TRUE

1167 rs7513428 C T C T -0.2171 -0.007 0.15353 NA FALSE FALSE FALSE
GCST006061 0.0101 0.4879 537409 outcome TRUE reported 9357_4_CREG1_CREG1
CREG1 chr1 167546035 0.011292 2.247e-82 35360 TRUE reported
textfile 2 1 TRUE

1168 rs10130078 T C T C -0.0513 2e-04 0.29545 NA FALSE FALSE FALSE
GCST006061 0.0079 0.9811 537409 outcome TRUE reported
7918_114_AMY1A_Amylase__alpha_1A AMY1A chr14 36687246 0.008387 9.577e-10
35355 TRUE reported textfile 2 1 TRUE

1169 rs112063273 T G T G -0.188 -0.0068 0.01874 NA FALSE FALSE FALSE
GCST006061 0.0274 0.8035 537409 outcome TRUE reported
7918_114_AMY1A_Amylase__alpha_1A AMY1A chr1 105851494 0.03078 1.01e-09
35356 TRUE reported textfile 2 1 TRUE

1170 rs115456582 G A G A -0.2224 0.0436 0.01757 NA FALSE FALSE FALSE
GCST006061 0.0396 0.2714 537409 outcome TRUE reported
7918_114_AMY1A_Amylase__alpha_1A AMY1A chr1 101069907 0.032188 4.864e-12
35351 TRUE reported textfile 2 1 TRUE

1171 rs116274910 G A G A 0.1804 -0.0435 0.03018 NA FALSE FALSE FALSE
GCST006061 0.0261 0.09568 537409 outcome TRUE reported
7918_114_AMY1A_Amylase__alpha_1A AMY1A chr1 103214731 0.024948 4.797e-13
35348 TRUE reported textfile 2 1 TRUE

1172 rs12111617 A G A G 0.0839 -0.0076 0.0928 NA FALSE FALSE FALSE
GCST006061 0.0107 0.4766 537409 outcome TRUE reported
7918_114_AMY1A_Amylase__alpha_1A AMY1A chr7 26004119 0.013259 2.491e-10
35372 TRUE reported textfile 2 1 TRUE

1173 rs140026476 A G A G 0.5917 -0.066 0.0119 NA FALSE FALSE FALSE
GCST006061 0.0608 0.2773 537409 outcome TRUE reported
7918_114_AMY1A_Amylase__alpha_1A AMY1A chr1 103160755 0.037781 2.786e-55
35348 TRUE reported textfile 2 1 TRUE

1174 rs148587593 T G T G -0.2463 -4.00E-04 0.01347 NA FALSE FALSE FALSE
GCST006061 0.0363 0.9921 537409 outcome TRUE reported
7918_114_AMY1A_Amylase__alpha_1A AMY1A chr1 106230250 0.037854 7.684e-11

35352 TRUE reported textfile 2 1 TRUE
1175 rs2317401 T C T C -0.2213 0.0325 0.09255 NA FALSE FALSE FALSE
GCST006061 0.021 0.122 537409 outcome TRUE reported
7918_114_AMY1A_Amylase__alpha_1A AMY1A chr1 104511217 0.014647 1.407e-51
35354 TRUE reported textfile 2 1 TRUE
1176 rs34858945 A G A G 0.1098 -0.0127 0.12485 NA FALSE FALSE FALSE
GCST006061 0.0105 0.2275 537409 outcome TRUE reported
7918_114_AMY1A_Amylase__alpha_1A AMY1A chr12 11208431 0.011639 3.962e-21
35376 TRUE reported textfile 2 1 TRUE
1177 rs4434842 G T G T 0.5049 -0.0048 0.07385 NA FALSE FALSE FALSE
GCST006061 0.0149 0.7474 537409 outcome TRUE reported
7918_114_AMY1A_Amylase__alpha_1A AMY1A chr1 103949496 0.016271 1e-200
35351 TRUE reported textfile 2 1 TRUE
1178 rs601338 A G A G 0.0931 0.0056 0.39439 NA FALSE FALSE FALSE
GCST006061 0.0076 0.4559 537409 outcome TRUE reported
7918_114_AMY1A_Amylase__alpha_1A AMY1A chr19 48703417 0.007895 4.294e-32
35377 TRUE reported textfile 2 1 TRUE
1179 rs635634 C T C T 0.0748 0.0285 0.12995 NA FALSE FALSE FALSE
GCST006061 0.0089 0.001351 537409 outcome TRUE reported
7918_114_AMY1A_Amylase__alpha_1A AMY1A chr9 133279427 0.011448 6.412e-11
35371 TRUE reported textfile 2 1 TRUE
1180 rs6676813 C A C A -0.9393 -0.0197 0.04147 NA FALSE FALSE FALSE
GCST006061 0.0209 0.345 537409 outcome TRUE reported
7918_114_AMY1A_Amylase__alpha_1A AMY1A chr1 103512697 0.013853 1e-200
35349 TRUE reported textfile 2 1 TRUE
1181 rs74374280 A G A G 0.2584 0.0472 0.01261 NA FALSE FALSE FALSE
GCST006061 0.0427 0.2695 537409 outcome TRUE reported
7918_114_AMY1A_Amylase__alpha_1A AMY1A chr1 105450654 0.039098 3.869e-11
35355 TRUE reported textfile 2 1 TRUE
1182 rs7556372 G A G A 0.0851 0.0147 0.42515 NA FALSE FALSE FALSE
GCST006061 0.0071 0.03947 537409 outcome TRUE reported
7918_114_AMY1A_Amylase__alpha_1A AMY1A chr1 206959000 0.008586 3.719e-23
35373 TRUE reported textfile 2 1 TRUE
1183 rs77538122 C T C T -0.136 -0.0355 0.03169 NA FALSE FALSE FALSE
GCST006061 0.0243 0.1433 537409 outcome TRUE reported
7918_114_AMY1A_Amylase__alpha_1A AMY1A chr1 110459748 0.024172 1.841e-08
35350 TRUE reported textfile 2 1 TRUE
1184 rs77645215 T C T C 0.1378 0.0287 0.03567 NA FALSE FALSE FALSE
GCST006061 0.0236 0.2233 537409 outcome TRUE reported
7918_114_AMY1A_Amylase__alpha_1A AMY1A chr12 11513456 0.020187 8.731e-12
35376 TRUE reported textfile 2 1 TRUE
1185 rs78282816 A G A G -0.2499 -0.0335 0.02262 NA FALSE FALSE FALSE
GCST006061 0.0295 0.2559 537409 outcome TRUE reported
7918_114_AMY1A_Amylase__alpha_1A AMY1A chr1 95185561 0.02872 3.283e-18
35353 TRUE reported textfile 2 1 TRUE
1186 rs10143150 G A G A 0.085 0.0121 0.12536 NA FALSE FALSE FALSE
GCST006061 0.0114 0.2907 537409 outcome TRUE reported 9313_27_CBLN1_CBLN1
CBLN1 chr14 105113797 0.011817 6.331e-13 35113 TRUE reported
textfile 2 1 TRUE
1187 rs111546471 T C T C -0.1959 0.0636 0.0303 NA FALSE FALSE FALSE
GCST006061 0.042 0.1303 537409 outcome TRUE reported 9313_27_CBLN1_CBLN1
CBLN1 chr11 63770145 0.022654 5.258e-18 35362 TRUE reported
textfile 2 1 TRUE
1188 rs114398277 A G A G -0.4081 -0.0212 0.03315 NA FALSE FALSE FALSE
GCST006061 0.0231 0.3589 537409 outcome TRUE reported 9313_27_CBLN1_CBLN1
CBLN1 chr16 49461811 0.024627 1.121e-61 35363 TRUE reported
textfile 2 1 TRUE
1189 rs11644731 T C T C 0.2205 0.0088 0.03182 NA FALSE FALSE FALSE
GCST006061 0.017 0.6022 537409 outcome TRUE reported 9313_27_CBLN1_CBLN1
CBLN1 chr16 51160792 0.025448 4.521e-18 35368 TRUE reported
textfile 2 1 TRUE
1190 rs117462763 A C A C -0.3514 0.0038 0.07065 NA FALSE FALSE FALSE
GCST006061 0.0239 0.8744 537409 outcome TRUE reported 9313_27_CBLN1_CBLN1
CBLN1 chr16 31853896 0.01735 3.28e-91 35363 TRUE reported textfile
2 1 TRUE
1191 rs117959836 T C T C 0.2499 0.0088 0.0389 NA FALSE FALSE FALSE

GCST006061 0.0274 0.747 537409 outcome TRUE reported 9313_27_CBLN1_CBLN1
CBLN1 chr16 49454279 0.023295 7.546e-27 35364 TRUE reported
textfile 2 1 TRUE
1192 rs12443595 A C A C -0.4981 0.0346 0.01226 NA FALSE FALSE FALSE
GCST006061 0.0725 0.6335 537409 outcome TRUE reported 9313_27_CBLN1_CBLN1
CBLN1 chr16 35498753 0.039006 2.414e-37 35367 TRUE reported
textfile 2 1 TRUE
1193 rs149119631 G A G A -0.4575 -0.1485 0.01159 NA FALSE FALSE FALSE
GCST006061 0.0676 0.02815 537409 outcome TRUE reported 9313_27_CBLN1_CBLN1
CBLN1 chr16 30817906 0.041966 1.131e-27 35370 TRUE reported
textfile 2 1 TRUE
1194 rs1566044 C T C T 0.2596 -0.0191 0.02402 NA FALSE FALSE FALSE
GCST006061 0.0407 0.6395 537409 outcome TRUE reported 9313_27_CBLN1_CBLN1
CBLN1 chr16 51079435 0.029659 2.081e-18 35371 TRUE reported
textfile 2 1 TRUE
1195 rs193630 C T C T -0.3734 -0.0305 0.02218 NA FALSE FALSE FALSE
GCST006061 0.035 0.3838 537409 outcome TRUE reported 9313_27_CBLN1_CBLN1
CBLN1 chr16 28575994 0.030165 3.405e-35 35387 TRUE reported
textfile 2 1 TRUE
1196 rs215226 G A G A 0.0534 -0.0191 0.41752 NA FALSE FALSE FALSE
GCST006061 0.0073 0.008405 537409 outcome TRUE reported 9313_27_CBLN1_CBLN1
CBLN1 chr12 482134 0.007962 1.987e-11 35311 TRUE reported textfile
2 1 TRUE
1197 rs2413593 G A G A 0.1431 -0.009 0.40393 NA FALSE FALSE FALSE
GCST006061 0.0076 0.2341 537409 outcome TRUE reported 9313_27_CBLN1_CBLN1
CBLN1 chr22 39476122 0.007943 1.483e-72 35366 TRUE reported
textfile 2 1 TRUE
1198 rs2439377 A G A G 0.0447 3e-04 0.39666 NA FALSE FALSE FALSE
GCST006061 0.0075 0.9639 537409 outcome TRUE reported 9313_27_CBLN1_CBLN1
CBLN1 chr15 66654582 0.007991 2.22e-08 35366 TRUE reported
textfile 2 1 TRUE
1199 rs2647998 T C T C 0.2333 -0.0176 0.01498 NA FALSE FALSE FALSE
GCST006061 0.022 0.4227 537409 outcome TRUE reported 9313_27_CBLN1_CBLN1
CBLN1 chr16 51538879 0.036077 1.001e-10 35368 TRUE reported
textfile 2 1 TRUE
1200 rs27817 G A G A 0.5152 2e-04 0.02445 NA FALSE FALSE FALSE GCST006061
0.0283 0.9957 537409 outcome TRUE reported 9313_27_CBLN1_CBLN1 CBLN1 chr16
49422380 0.028831 2.034e-71 35364 TRUE reported textfile 2 1 TRUE
1201 rs2950790 A G A G -0.0762 -0.0086 0.26694 NA FALSE FALSE FALSE
GCST006061 0.0084 0.3028 537409 outcome TRUE reported 9313_27_CBLN1_CBLN1
CBLN1 chr2 145002154 0.00885 7.284e-18 35353 TRUE reported textfile
2 1 TRUE
1202 rs62029101 G A G A 0.2192 -0.0161 0.05247 NA FALSE FALSE FALSE
GCST006061 0.0216 0.4565 537409 outcome TRUE reported 9313_27_CBLN1_CBLN1
CBLN1 chr16 49379117 0.019742 1.21e-28 35365 TRUE reported
textfile 2 1 TRUE
1203 rs648997 T C T C 0.0453 0.0233 0.36916 NA FALSE FALSE FALSE
GCST006061 0.0085 0.006076 537409 outcome TRUE reported 9313_27_CBLN1_CBLN1
CBLN1 chr12 111538972 0.007851 7.94e-09 35371 TRUE reported
textfile 2 1 TRUE
1204 rs6504180 C T C T -0.055 0.0056 0.38013 NA FALSE FALSE FALSE
GCST006061 0.0074 0.4496 537409 outcome TRUE reported 9313_27_CBLN1_CBLN1
CBLN1 chr17 63735809 0.008071 9.446e-12 35368 TRUE reported
textfile 2 1 TRUE
1205 rs66619583 T C T C -0.0904 0.0155 0.09053 NA FALSE FALSE FALSE
GCST006061 0.0122 0.2036 537409 outcome TRUE reported 9313_27_CBLN1_CBLN1
CBLN1 chr11 126400946 0.013626 3.262e-11 35374 TRUE reported
textfile 2 1 TRUE
1206 rs72836800 C T C T 0.0832 0.0148 0.11727 NA FALSE FALSE FALSE
GCST006061 0.0115 0.1971 537409 outcome TRUE reported 9313_27_CBLN1_CBLN1
CBLN1 chr6 1617092 0.012093 5.994e-12 35320 TRUE reported textfile
2 1 TRUE
1207 rs7679276 G A G A 0.2334 0.0119 0.04606 NA FALSE FALSE FALSE
GCST006061 0.0217 0.5821 537409 outcome TRUE reported 9313_27_CBLN1_CBLN1
CBLN1 chr4 145939034 0.01846 1.211e-36 35359 TRUE reported textfile
2 1 TRUE

1208 rs79190220 C T C T -0.24 0.0093 0.02453 NA FALSE FALSE FALSE GCST006061 0.0322 0.7737 537409 outcome TRUE reported 9313_27_CBLN1_CBLN1 CBLN1 chr16 49828014 0.028198 1.723e-17 35368 TRUE reported textfile 2 1 TRUE
1209 rs7988061 T C T C 0.0575 -0.0215 0.46519 NA FALSE FALSE FALSE GCST006061 0.0072 0.002909 537409 outcome TRUE reported 9313_27_CBLN1_CBLN1 CBLN1 chr13 26470430 0.007851 2.409e-13 35355 TRUE reported textfile 2 1 TRUE
1210 rs9674555 A C A C -0.0554 0.0298 0.20503 NA FALSE FALSE FALSE GCST006061 0.0087 0.0006067 537409 outcome TRUE reported 9313_27_CBLN1_CBLN1 CBLN1 chr17 12699021 0.009638 9.012e-09 35361 TRUE reported textfile 2 1 TRUE
1211 rs115633223 T C T C 0.3512 -0.0217 0.01227 NA FALSE FALSE FALSE GCST006061 0.0525 0.6798 537409 outcome TRUE reported 5036_50_TNFAIP6_TSG_6 TNFAIP6 chr2 150920413 0.03594 1.487e-22 35347 TRUE reported textfile 2 1 TRUE
1212 rs1260326 C T C T 0.0541 -0.0069 0.34132 NA FALSE FALSE FALSE GCST006061 0.0073 0.3401 537409 outcome TRUE reported 5036_50_TNFAIP6_TSG_6 TNFAIP6 chr2 27508073 0.008576 2.824e-10 35373 TRUE reported textfile 2 1 TRUE
1213 rs139808240 A G A G -0.2145 -0.0298 0.01184 NA FALSE FALSE FALSE GCST006061 0.0445 0.503 537409 outcome TRUE reported 5036_50_TNFAIP6_TSG_6 TNFAIP6 chr2 140536405 0.036763 5.388e-09 35352 TRUE reported textfile 2 1 TRUE
1214 rs1475718 A G A G -0.0435 -0.0068 0.48366 NA FALSE FALSE FALSE GCST006061 0.0073 0.347 537409 outcome TRUE reported 5036_50_TNFAIP6_TSG_6 TNFAIP6 chr9 134226324 0.007756 2.044e-08 35368 TRUE reported textfile 2 1 TRUE
1215 rs16829593 G T G T 0.3084 0.02 0.263 NA FALSE FALSE FALSE GCST006061 0.0084 0.0179 537409 outcome TRUE reported 5036_50_TNFAIP6_TSG_6 TNFAIP6 chr2 151143377 0.009042 1e-200 35346 TRUE reported textfile 2 1 TRUE
1216 rs17270394 C A C A -0.167 0.0114 0.15447 NA FALSE FALSE FALSE GCST006061 0.0099 0.249 537409 outcome TRUE reported 5036_50_TNFAIP6_TSG_6 TNFAIP6 chr2 151987560 0.011174 1.665e-50 35343 TRUE reported textfile 2 1 TRUE
1217 rs189716947 T C T C -0.1437 -0.006 0.03149 NA FALSE FALSE FALSE GCST006061 0.022 0.7844 537409 outcome TRUE reported 5036_50_TNFAIP6_TSG_6 TNFAIP6 chr2 145440505 0.022768 2.764e-10 35352 TRUE reported textfile 2 1 TRUE
1218 rs2298318 G A G A 0.1228 -0.0021 0.04886 NA FALSE FALSE FALSE GCST006061 0.0174 0.9028 537409 outcome TRUE reported 5036_50_TNFAIP6_TSG_6 TNFAIP6 chr10 7732128 0.01805 1.023e-11 35363 TRUE reported textfile 2 1 TRUE
1219 rs28430325 T C T C -0.0947 0.0282 0.06314 NA FALSE FALSE FALSE GCST006061 0.0157 0.07284 537409 outcome TRUE reported 5036_50_TNFAIP6_TSG_6 TNFAIP6 chr22 36432957 0.015824 2.169e-09 35382 TRUE reported textfile 2 1 TRUE
1220 rs3774355 A G A G 0.0889 -0.0171 0.38301 NA FALSE FALSE FALSE GCST006061 0.0073 0.01922 537409 outcome TRUE reported 5036_50_TNFAIP6_TSG_6 TNFAIP6 chr3 52783762 0.007961 5.903e-29 35354 TRUE reported textfile 2 1 TRUE
1221 rs42122 A G A G 0.0733 -0.0113 0.16937 NA FALSE FALSE FALSE GCST006061 0.01 0.2559 537409 outcome TRUE reported 5036_50_TNFAIP6_TSG_6 TNFAIP6 chr7 73427493 0.010383 1.67e-12 35354 TRUE reported textfile 2 1 TRUE
1222 rs4263799 T C T C 0.0481 -0.0066 0.44282 NA FALSE FALSE FALSE GCST006061 0.0072 0.3562 537409 outcome TRUE reported 5036_50_TNFAIP6_TSG_6 TNFAIP6 chr8 71482756 0.007772 6.066e-10 35348 TRUE reported textfile 2 1 TRUE
1223 rs4604455 C T C T -0.0484 2e-04 0.33979 NA FALSE FALSE FALSE GCST006061 0.0077 0.9812 537409 outcome TRUE reported 5036_50_TNFAIP6_TSG_6 TNFAIP6 chr8 125505785 0.008136 2.696e-09 35367 TRUE reported textfile 2 1 TRUE
1224 rs4664356 G A G A -0.3352 -0.013 0.38862 NA FALSE FALSE FALSE GCST006061 0.0072 0.07265 537409 outcome TRUE reported 5036_50_TNFAIP6_TSG_6

TNFAIP6 chr2 151339420 0.006145 1e-200 35346 TRUE reported textfile
2 1 TRUE
1225 rs6768289 A G A G 0.2116 -0.0081 0.2666 NA FALSE FALSE FALSE
GCST006061 0.0081 0.318 537409 outcome TRUE reported 5036_50_TNFAIP6_TSG_6
TNFAIP6 chr3 126537373 0.008739 1.648e-129 35371 TRUE reported
textfile 2 1 TRUE
1226 rs9568476 C A C A 0.054 -0.0038 0.42444 NA FALSE FALSE FALSE
GCST006061 0.0071 0.596 537409 outcome TRUE reported 5036_50_TNFAIP6_TSG_6
TNFAIP6 chr13 50816706 0.007765 3.533e-12 35362 TRUE reported
textfile 2 1 TRUE
1227 rs9649546 C T C T -0.0821 -0.0037 0.0868 NA FALSE FALSE FALSE
GCST006061 0.013 0.7734 537409 outcome TRUE reported 5036_50_TNFAIP6_TSG_6
TNFAIP6 chr7 130891687 0.013787 2.604e-09 35356 TRUE reported
textfile 2 1 TRUE
1228 rs10135188 A G A G 0.0491 -0.0113 0.31763 NA FALSE FALSE FALSE
GCST006061 0.0082 0.1671 537409 outcome TRUE reported 5034_79_PRSS2_Trypsin_2
PRSS2 chr14 32004590 0.00895 4.111e-08 35353 TRUE reported textfile
2 1 TRUE
1229 rs1164032 G A G A -0.1039 0.0061 0.10893 NA FALSE FALSE FALSE
GCST006061 0.0184 0.7414 537409 outcome TRUE reported 5034_79_PRSS2_Trypsin_2
PRSS2 chr1 120072993 0.013477 1.263e-14 35358 TRUE reported
textfile 2 1 TRUE
1230 rs17032925 C T C T -0.1236 -9.00E-04 0.08221 NA FALSE FALSE FALSE
GCST006061 0.0134 0.9475 537409 outcome TRUE reported 5034_79_PRSS2_Trypsin_2
PRSS2 chr2 67039351 0.015201 4.252e-16 35359 TRUE reported
textfile 2 1 TRUE
1231 rs1799886 C T C T 0.2038 0.013 0.41013 NA FALSE FALSE FALSE
GCST006061 0.0076 0.08815 537409 outcome TRUE reported 5034_79_PRSS2_Trypsin_2
PRSS2 chr7 142800839 0.008582 1.192e-124 35374 TRUE reported
textfile 2 1 TRUE
1232 rs193098612 C T C T -0.2951 0.0346 0.01229 NA FALSE FALSE FALSE
GCST006061 0.0536 0.5177 537409 outcome TRUE reported 5034_79_PRSS2_Trypsin_2
PRSS2 chr7 139973968 0.03833 1.373e-14 35382 TRUE reported textfile
2 1 TRUE
1233 rs2277710 C T C T 0.1109 -0.0077 0.06862 NA FALSE FALSE FALSE
GCST006061 0.0134 0.5632 537409 outcome TRUE reported 5034_79_PRSS2_Trypsin_2
PRSS2 chr18 44792522 0.016552 2.083e-11 35349 TRUE reported
textfile 2 1 TRUE
1234 rs2638493 A G A G -0.0542 0.0073 0.31111 NA FALSE FALSE FALSE
GCST006061 0.008 0.3659 537409 outcome TRUE reported 5034_79_PRSS2_Trypsin_2
PRSS2 chr12 52882437 0.008978 1.567e-09 35373 TRUE reported
textfile 2 1 TRUE
1235 rs507695 T C T C -0.0643 0.0013 0.2959 NA FALSE FALSE FALSE
GCST006061 0.0079 0.8663 537409 outcome TRUE reported 5034_79_PRSS2_Trypsin_2
PRSS2 chr11 100776205 0.009097 1.568e-12 35356 TRUE reported
textfile 2 1 TRUE
1236 rs533406 G A G A -0.0735 -0.0177 0.41964 NA FALSE FALSE FALSE
GCST006061 0.0075 0.01777 537409 outcome TRUE reported 5034_79_PRSS2_Trypsin_2
PRSS2 chr16 88908452 0.008494 4.99e-18 35321 TRUE reported
textfile 2 1 TRUE
1237 rs62479377 C A C A 0.1093 0.0038 0.08062 NA FALSE FALSE FALSE
GCST006061 0.0198 0.8488 537409 outcome TRUE reported 5034_79_PRSS2_Trypsin_2
PRSS2 chr7 142109725 0.015541 2.022e-12 35369 TRUE reported
textfile 2 1 TRUE
1238 rs72802342 A C A C -0.3235 0.0024 0.06858 NA FALSE FALSE FALSE
GCST006061 0.0147 0.8687 537409 outcome TRUE reported 5034_79_PRSS2_Trypsin_2
PRSS2 chr16 75200974 0.01666 5.452e-84 35363 TRUE reported textfile
2 1 TRUE
1239 rs7383917 A C A C -0.1128 0.0057 0.13895 NA FALSE FALSE FALSE
GCST006061 0.011 0.6016 537409 outcome TRUE reported 5034_79_PRSS2_Trypsin_2
PRSS2 chr7 142619745 0.012262 3.619e-20 35370 TRUE reported
textfile 2 1 TRUE
1240 rs748208 C T C T -0.0528 -0.0201 0.35761 NA FALSE FALSE FALSE
GCST006061 0.0082 0.01395 537409 outcome TRUE reported 5034_79_PRSS2_Trypsin_2
PRSS2 chr8 18855003 0.008686 1.21e-09 35375 TRUE reported
textfile 2 1 TRUE

1241 rs8176743 T C T C 0.123 0.017 0.06488 NA FALSE FALSE FALSE
GCST006061 0.0128 0.1834 537409 outcome TRUE reported 5034_79_PRSS2_Trypsin_2
PRSS2 chr9 133256028 0.016982 4.389e-13 35370 TRUE reported
textfile 2 1 TRUE
1242 rs838133 G A G A 0.0615 -0.0061 0.49845 NA FALSE FALSE FALSE
GCST006061 0.0079 0.4418 537409 outcome TRUE reported 5034_79_PRSS2_Trypsin_2
PRSS2 chr19 48756272 0.008352 1.795e-13 35374 TRUE reported
textfile 2 1 TRUE
1243 rs9482772 C T C T 0.0643 0.0022 0.47592 NA FALSE FALSE FALSE
GCST006061 0.0071 0.7593 537409 outcome TRUE reported 5034_79_PRSS2_Trypsin_2
PRSS2 chr6 127125645 0.008356 1.419e-14 35355 TRUE reported
textfile 2 1 TRUE
1244 rs10135366 C T C T 0.0717 0.0142 0.26814 NA FALSE FALSE FALSE
GCST006061 0.0082 0.08443 537409 outcome TRUE reported 5644_60_RNASE4_RNAS4
RNASE4 chr14 21156038 0.009452 3.315e-14 35335 TRUE reported
textfile 2 1 TRUE
1245 rs11624594 G A G A -0.0782 -0.0025 0.26604 NA FALSE FALSE FALSE
GCST006061 0.0083 0.7632 537409 outcome TRUE reported 5644_60_RNASE4_RNAS4
RNASE4 chr14 20762013 0.009514 2.039e-16 35340 TRUE reported
textfile 2 1 TRUE
1246 rs12588573 T C T C -0.3626 -0.0019 0.21548 NA FALSE FALSE FALSE
GCST006061 0.0085 0.8238 537409 outcome TRUE reported 5644_60_RNASE4_RNAS4
RNASE4 chr14 20678425 0.010037 1e-200 35337 TRUE reported textfile
2 1 TRUE
1247 rs77924615 A G A G -0.0582 0.0136 0.19701 NA FALSE FALSE FALSE
GCST006061 0.0092 0.1375 537409 outcome TRUE reported 5644_60_RNASE4_RNAS4
RNASE4 chr16 20381010 0.010486 2.853e-08 35355 TRUE reported
textfile 2 1 TRUE
1248 rs10135681 C T C T 0.0554 0.0056 0.38847 NA FALSE FALSE FALSE
GCST006061 0.0074 0.4471 537409 outcome TRUE reported
15318_75_S100A10_Calpactin_I_light_chain S100A10 chr14 94541407 0.008248
1.862e-11 35371 TRUE reported textfile 2 1 TRUE
1249 rs10137923 A G A G 0.1625 -0.0132 0.24753 NA FALSE FALSE FALSE
GCST006061 0.0085 0.1203 537409 outcome TRUE reported
6551_94_SERPINA12_Vaspin SERPINA12 chr14 94556349 0.010096 2.765e-58
35372 TRUE reported textfile 2 1 TRUE
1250 rs10922098 T C T C -0.1274 -0.009 0.40011 NA FALSE FALSE FALSE
GCST006061 0.0075 0.2275 537409 outcome TRUE reported
6551_94_SERPINA12_Vaspin SERPINA12 chr1 196695521 0.008579 6.909e-50
35338 TRUE reported textfile 2 1 TRUE
1251 rs11160190 G A G A -0.3176 -5.00E-04 0.41721 NA FALSE FALSE FALSE
GCST006061 0.0073 0.9427 537409 outcome TRUE reported
6551_94_SERPINA12_Vaspin SERPINA12 chr14 94546047 0.008682 1e-200 35371
TRUE reported textfile 2 1 TRUE
1252 rs113958256 A G A G 0.6289 -0.0198 0.06189 NA FALSE FALSE FALSE
GCST006061 0.0147 0.1778 537409 outcome TRUE reported
6551_94_SERPINA12_Vaspin SERPINA12 chr14 94497408 0.017607 1e-200 35372
TRUE reported textfile 2 1 TRUE
1253 rs339969 A C A C -0.0595 -0.0083 0.33988 NA FALSE FALSE FALSE
GCST006061 0.0075 0.2675 537409 outcome TRUE reported
6551_94_SERPINA12_Vaspin SERPINA12 chr15 60591082 0.008851 1.788e-11
35370 TRUE reported textfile 2 1 TRUE
1254 rs55911632 G A G A -0.1763 0.0049 0.16181 NA FALSE FALSE FALSE
GCST006061 0.0096 0.6109 537409 outcome TRUE reported
6551_94_SERPINA12_Vaspin SERPINA12 chr14 94452451 0.011716 3.542e-51
35371 TRUE reported textfile 2 1 TRUE
1255 rs59233955 A G A G 0.0578 -0.0032 0.45073 NA FALSE FALSE FALSE
GCST006061 0.0074 0.6656 537409 outcome TRUE reported
6551_94_SERPINA12_Vaspin SERPINA12 chr14 94706204 0.008703 3.103e-11
35371 TRUE reported textfile 2 1 TRUE
1256 rs6445035 A G A G 0.0778 0.0154 0.1904 NA FALSE FALSE FALSE
GCST006061 0.0089 0.08256 537409 outcome TRUE reported
6551_94_SERPINA12_Vaspin SERPINA12 chr3 165762312 0.01069 3.39e-13 35339
TRUE reported textfile 2 1 TRUE
1257 rs704 A G A G -0.0642 -0.0155 0.49189 NA FALSE FALSE FALSE GCST006061
0.0072 0.03144 537409 outcome TRUE reported 6551_94_SERPINA12_Vaspin

SERPINA12 chr17 28367840 0.008413 2.33e-14 35360 TRUE reported
textfile 2 1 TRUE
1258 rs10138671 G A G A 0.192 -0.02 0.39722 NA FALSE FALSE FALSE
GCST006061 0.0073 0.006331 537409 outcome TRUE reported 4125_52_AGER_sRAGE
AGER chr14 65393374 0.008389 6.38e-116 35360 TRUE reported
textfile 2 1 TRUE
1259 rs1057239 A G A G -0.0469 0.0145 0.46634 NA FALSE FALSE FALSE
GCST006061 0.0073 0.0473 537409 outcome TRUE reported 4125_52_AGER_sRAGE
AGER chr1 175161068 0.008292 1.549e-08 35347 TRUE reported
textfile 2 1 TRUE
1260 rs10735335 A G A G -0.0535 -0.0022 0.34732 NA FALSE FALSE FALSE
GCST006061 0.0072 0.7596 537409 outcome TRUE reported 4125_52_AGER_sRAGE
AGER chr12 95869659 0.008664 6.629e-10 35364 TRUE reported
textfile 2 1 TRUE
1261 rs10824665 A G A G -0.0738 -0.0192 0.18965 NA FALSE FALSE FALSE
GCST006061 0.0091 0.03594 537409 outcome TRUE reported 4125_52_AGER_sRAGE
AGER chr10 78917233 0.010502 2.108e-12 35370 TRUE reported
textfile 2 1 TRUE
1262 rs111510768 A G A G -0.0688 -4.00E-04 0.16347 NA FALSE FALSE FALSE
GCST006061 0.01 0.9705 537409 outcome TRUE reported 4125_52_AGER_sRAGE
AGER chr8 101464878 0.011208 8.337e-10 35358 TRUE reported
textfile 2 1 TRUE
1263 rs11243421 C T C T -0.1876 0.0094 0.42171 NA FALSE FALSE FALSE
GCST006061 0.0072 0.1934 537409 outcome TRUE reported 4125_52_AGER_sRAGE
AGER chr9 131550651 0.00833 2.568e-112 35372 TRUE reported textfile
2 1 TRUE
1264 rs116592029 C T C T -0.106 0.0529 0.06491 NA FALSE FALSE FALSE
GCST006061 0.02 0.008106 537409 outcome TRUE reported 4125_52_AGER_sRAGE
AGER chr3 168987802 0.016921 3.742e-10 35355 TRUE reported
textfile 2 1 TRUE
1265 rs117048263 A C A C -0.2084 -0.017 0.02406 NA FALSE FALSE FALSE
GCST006061 0.0363 0.64 537409 outcome TRUE reported 4125_52_AGER_sRAGE
AGER chr3 169371794 0.027178 1.746e-14 35355 TRUE reported
textfile 2 1 TRUE
1266 rs12201133 A C A C 0.0967 -0.0093 0.27989 NA FALSE FALSE FALSE
GCST006061 0.0082 0.2544 537409 outcome TRUE reported 4125_52_AGER_sRAGE
AGER chr6 129969624 0.009345 4.283e-25 35365 TRUE reported
textfile 2 1 TRUE
1267 rs12461753 A G A G -0.0702 -2.00E-04 0.1638 NA FALSE FALSE FALSE
GCST006061 0.0097 0.9807 537409 outcome TRUE reported 4125_52_AGER_sRAGE
AGER chr19 37711614 0.011212 3.822e-10 35377 TRUE reported
textfile 2 1 TRUE
1268 rs12906898 A G A G 0.0533 -0.0136 0.31983 NA FALSE FALSE FALSE
GCST006061 0.0079 0.08257 537409 outcome TRUE reported 4125_52_AGER_sRAGE
AGER chr15 66754458 0.008908 2.182e-09 35364 TRUE reported
textfile 2 1 TRUE
1269 rs149438404 A G A G 0.1343 -0.0073 0.06057 NA FALSE FALSE FALSE
GCST006061 0.0201 0.7156 537409 outcome TRUE reported 4125_52_AGER_sRAGE
AGER chr7 74609727 0.017446 1.38e-14 35340 TRUE reported
textfile 2 1 TRUE
1270 rs1569419 C T C T -0.1348 0.0034 0.23113 NA FALSE FALSE FALSE
GCST006061 0.0087 0.6948 537409 outcome TRUE reported 4125_52_AGER_sRAGE
AGER chr1 3080038 0.009839 1e-42 35333 TRUE reported textfile 2
1 TRUE
1271 rs17762402 A G A G 0.0947 -0.0088 0.08419 NA FALSE FALSE FALSE
GCST006061 0.0151 0.5589 537409 outcome TRUE reported 4125_52_AGER_sRAGE
AGER chr11 61785729 0.014758 1.392e-10 35367 TRUE reported
textfile 2 1 TRUE
1272 rs2844456 C T C T -0.6535 -0.0245 0.04085 NA FALSE FALSE FALSE
GCST006061 0.0185 0.1851 537409 outcome TRUE reported 4125_52_AGER_sRAGE
AGER chr6 31896897 0.020853 1e-200 35441 TRUE reported textfile
2 1 TRUE
1273 rs2852746 G A G A -0.059 0.0099 0.3595 NA FALSE FALSE FALSE
GCST006061 0.0074 0.1819 537409 outcome TRUE reported 4125_52_AGER_sRAGE
AGER chr18 44649359 0.008569 5.773e-12 35346 TRUE reported
textfile 2 1 TRUE

1274 rs34562714 A C A C -0.0493 0.0197 0.37897 NA FALSE FALSE FALSE
GCST006061 0.0077 0.01009 537409 outcome TRUE reported 4125_52_AGER_sRAGE
AGER chr15 49183085 0.008517 7.102e-09 35359 TRUE reported
textfile 2 1 TRUE
1275 rs34979081 G T G T -0.0499 0.0089 0.3199 NA FALSE FALSE FALSE
GCST006061 0.0084 0.2915 537409 outcome TRUE reported 4125_52_AGER_sRAGE
AGER chr21 17618066 0.008863 1.801e-08 35355 TRUE reported
textfile 2 1 TRUE
1276 rs4685105 G A G A -0.0589 0.0104 0.40132 NA FALSE FALSE FALSE
GCST006061 0.0074 0.1584 537409 outcome TRUE reported 4125_52_AGER_sRAGE
AGER chr3 14278676 0.008421 2.66e-12 35369 TRUE reported
textfile 2 1 TRUE
1277 rs4975932 C T C T -0.054 -0.0167 0.2636 NA FALSE FALSE FALSE
GCST006061 0.0098 0.08945 537409 outcome TRUE reported 4125_52_AGER_sRAGE
AGER chr5 46359647 0.009354 7.79e-09 35362 TRUE reported
textfile 2 1 TRUE
1278 rs56049626 G T G T 0.1175 0.006 0.23736 NA FALSE FALSE FALSE
GCST006061 0.0084 0.4779 537409 outcome TRUE reported 4125_52_AGER_sRAGE
AGER chr13 73072892 0.009685 7.174e-34 35358 TRUE reported
textfile 2 1 TRUE
1279 rs6045291 T G T G 0.1041 -3.00E-04 0.1075 NA FALSE FALSE FALSE
GCST006061 0.0114 0.9764 537409 outcome TRUE reported 4125_52_AGER_sRAGE
AGER chr20 18248816 0.013309 5.213e-15 35379 TRUE reported
textfile 2 1 TRUE
1280 rs62055069 C A C A 0.1165 0.0594 0.03872 NA FALSE FALSE FALSE
GCST006061 0.0211 0.004963 537409 outcome TRUE reported 4125_52_AGER_sRAGE
AGER chr16 73043823 0.021219 4.014e-08 35370 TRUE reported
textfile 2 1 TRUE
1281 rs6745864 G T G T 0.0691 -0.0259 0.182 NA FALSE FALSE FALSE
GCST006061 0.0096 0.006995 537409 outcome TRUE reported 4125_52_AGER_sRAGE
AGER chr2 25611654 0.010695 1.039e-10 35369 TRUE reported
textfile 2 1 TRUE
1282 rs6897262 C T C T 0.0462 0.0094 0.48503 NA FALSE FALSE FALSE
GCST006061 0.0072 0.1933 537409 outcome TRUE reported 4125_52_AGER_sRAGE
AGER chr5 149263143 0.008215 1.868e-08 35361 TRUE reported
textfile 2 1 TRUE
1283 rs6961094 T C T C 0.0783 -0.0046 0.14314 NA FALSE FALSE FALSE
GCST006061 0.0112 0.679 537409 outcome TRUE reported 4125_52_AGER_sRAGE
AGER chr7 101903036 0.011711 2.294e-11 35367 TRUE reported
textfile 2 1 TRUE
1284 rs7212311 T C T C -0.0612 -0.0114 0.34807 NA FALSE FALSE FALSE
GCST006061 0.0078 0.1411 537409 outcome TRUE reported 4125_52_AGER_sRAGE
AGER chr17 75098312 0.008634 1.362e-12 35381 TRUE reported
textfile 2 1 TRUE
1285 rs7306778 T C T C -0.1627 -0.0134 0.19285 NA FALSE FALSE FALSE
GCST006061 0.0101 0.182 537409 outcome TRUE reported 4125_52_AGER_sRAGE
AGER chr12 508142 0.010445 1.037e-54 35313 TRUE reported textfile
2 1 TRUE
1286 rs73093253 A G A G 0.062 0.0267 0.30125 NA FALSE FALSE FALSE
GCST006061 0.0077 0.0005668 537409 outcome TRUE reported 4125_52_AGER_sRAGE
AGER chr4 24587441 0.009008 5.883e-12 35358 TRUE reported
textfile 2 1 TRUE
1287 rs7530075 A G A G 0.0902 -0.0023 0.21494 NA FALSE FALSE FALSE
GCST006061 0.008 0.7731 537409 outcome TRUE reported 4125_52_AGER_sRAGE
AGER chr1 3189498 0.010059 3.046e-19 35329 TRUE reported textfile
2 1 TRUE
1288 rs7616330 A C A C 0.0815 -0.0135 0.17817 NA FALSE FALSE FALSE
GCST006061 0.0093 0.1479 537409 outcome TRUE reported 4125_52_AGER_sRAGE
AGER chr3 71066600 0.010781 4.047e-14 35350 TRUE reported
textfile 2 1 TRUE
1289 rs7744366 A G A G 0.1256 0.0036 0.10944 NA FALSE FALSE FALSE
GCST006061 0.0101 0.7208 537409 outcome TRUE reported 4125_52_AGER_sRAGE
AGER chr6 6854256 0.013429 8.532e-21 35375 TRUE reported textfile
2 1 TRUE
1290 rs7868554 G A G A -0.0519 0.0157 0.29416 NA FALSE FALSE FALSE
GCST006061 0.0078 0.04376 537409 outcome TRUE reported 4125_52_AGER_sRAGE

AGER chr9 110155265 0.009069 1.047e-08 35373 TRUE reported
textfile 2 1 TRUE
1291 rs7920682 G A G A 0.0455 0.0138 0.47008 NA FALSE FALSE FALSE
GCST006061 0.0071 0.05392 537409 outcome TRUE reported 4125_52_AGER_sRAGE
AGER chr10 30028897 0.008265 3.689e-08 35355 TRUE reported
textfile 2 1 TRUE
1292 rs7962469 G A G A 0.0568 2e-04 0.35823 NA FALSE FALSE FALSE
GCST006061 0.0075 0.9761 537409 outcome TRUE reported 4125_52_AGER_sRAGE
AGER chr12 51954475 0.008602 4.026e-11 35384 TRUE reported
textfile 2 1 TRUE
1293 rs79730878 C T C T 0.1014 -0.0404 0.18684 NA FALSE FALSE FALSE
GCST006061 0.0094 1.641e-05 537409 outcome TRUE reported 4125_52_AGER_sRAGE
AGER chr17 45772049 0.010652 1.75e-21 35364 TRUE reported
textfile 2 1 TRUE
1294 rs116094872 G A G A 0.4272 0.0143 0.01676 NA FALSE FALSE FALSE
GCST006061 0.0329 0.6639 537409 outcome TRUE reported
13119_26_SERPINA10_protein_Z_inhibitor SERPINA10 chr14 94499381 0.031586
1.115e-41 35363 TRUE reported textfile 2 1 TRUE
1295 rs11621100 A G A G 0.2396 -0.0016 0.03299 NA FALSE FALSE FALSE
GCST006061 0.0284 0.9542 537409 outcome TRUE reported
13119_26_SERPINA10_protein_Z_inhibitor SERPINA10 chr14 93627843 0.02323
6.066e-25 35360 TRUE reported textfile 2 1 TRUE
1296 rs117955282 T C T C -0.1285 -0.0082 0.04616 NA FALSE FALSE FALSE
GCST006061 0.02 0.6807 537409 outcome TRUE reported
13119_26_SERPINA10_protein_Z_inhibitor SERPINA10 chr14 93996168 0.019658
6.292e-11 35361 TRUE reported textfile 2 1 TRUE
1297 rs12436634 C T C T -0.2151 0.0277 0.0222 NA FALSE FALSE FALSE
GCST006061 0.0213 0.1928 537409 outcome TRUE reported
13119_26_SERPINA10_protein_Z_inhibitor SERPINA10 chr14 95005209 0.028104
1.952e-14 35357 TRUE reported textfile 2 1 TRUE
1298 rs1260326 C T C T -0.0479 -0.0069 0.34132 NA FALSE FALSE FALSE
GCST006061 0.0073 0.3401 537409 outcome TRUE reported
13119_26_SERPINA10_protein_Z_inhibitor SERPINA10 chr2 27508073 0.008178
4.702e-09 35364 TRUE reported textfile 2 1 TRUE
1299 rs144634898 A G A G 0.3321 -0.0379 0.01827 NA FALSE FALSE FALSE
GCST006061 0.0332 0.2535 537409 outcome TRUE reported
13119_26_SERPINA10_protein_Z_inhibitor SERPINA10 chr14 94779298 0.030387
8.359e-28 35360 TRUE reported textfile 2 1 TRUE
1300 rs150331426 G A G A 0.7321 0.0212 0.03221 NA FALSE FALSE FALSE
GCST006061 0.0397 0.5929 537409 outcome TRUE reported
13119_26_SERPINA10_protein_Z_inhibitor SERPINA10 chr14 94297718 0.022856
1e-200 35363 TRUE reported textfile 2 1 TRUE
1301 rs2255531 A G A G 0.0503 -0.004 0.3496 NA FALSE FALSE FALSE
GCST006061 0.0074 0.5835 537409 outcome TRUE reported
13119_26_SERPINA10_protein_Z_inhibitor SERPINA10 chr12 120977112 0.008111
5.595e-10 35356 TRUE reported textfile 2 1 TRUE
1302 rs4150746 C T C T 0.0631 -0.0202 0.36098 NA FALSE FALSE FALSE
GCST006061 0.0074 0.006563 537409 outcome TRUE reported
13119_26_SERPINA10_protein_Z_inhibitor SERPINA10 chr13 113616906 0.008453
8.327e-14 34853 TRUE reported textfile 2 1 TRUE
1303 rs528218 A G A G -0.2988 0.0059 0.08472 NA FALSE FALSE FALSE
GCST006061 0.0138 0.6702 537409 outcome TRUE reported
13119_26_SERPINA10_protein_Z_inhibitor SERPINA10 chr13 113143032 0.014592
3.501e-93 34853 TRUE reported textfile 2 1 TRUE
1304 rs559054 C T C T 0.44 -0.0187 0.35764 NA FALSE FALSE FALSE
GCST006061 0.0078 0.01705 537409 outcome TRUE reported
13119_26_SERPINA10_protein_Z_inhibitor SERPINA10 chr13 113146308 0.005713
1e-200 34854 TRUE reported textfile 2 1 TRUE
1305 rs72661955 T C T C 0.119 -0.0121 0.36527 NA FALSE FALSE FALSE
GCST006061 0.0079 0.1238 537409 outcome TRUE reported
13119_26_SERPINA10_protein_Z_inhibitor SERPINA10 chr13 112981089 0.008437
3.6e-45 34887 TRUE reported textfile 2 1 TRUE
1306 rs746367 A C A C 0.0489 0.0044 0.39041 NA FALSE FALSE FALSE
GCST006061 0.0103 0.6684 537409 outcome TRUE reported
13119_26_SERPINA10_protein_Z_inhibitor SERPINA10 chr13 112624168 0.008352
4.781e-09 34935 TRUE reported textfile 2 1 TRUE

1307 rs76483535 A G A G -0.1266 0.0715 0.03805 NA FALSE FALSE FALSE
GCST006061 0.0312 0.02176 537409 outcome TRUE reported
13119_26_SERPINA10_protein_Z_inhibitor SERPINA10 chr14 94402826 0.021866
7.051e-09 35363 TRUE reported textfile 2 1 TRUE
1308 rs9604093 A G A G 0.0941 0.0021 0.48275 NA FALSE FALSE FALSE
GCST006061 0.0075 0.7753 537409 outcome TRUE reported
13119_26_SERPINA10_protein_Z_inhibitor SERPINA10 chr13 113373957 0.00815
7.738e-31 34850 TRUE reported textfile 2 1 TRUE
1309 rs10139374 C T C T 0.1762 -0.0019 0.22885 NA FALSE FALSE FALSE
GCST006061 0.0142 0.8949 537409 outcome TRUE reported
16305_10_CDH11_Cadherin_11 CDH11 chr14 105823495 0.010366 8.48e-65 34967
TRUE reported textfile 2 1 TRUE
1310 rs1155043 G A G A 0.05 0.0093 0.47254 NA FALSE FALSE FALSE
GCST006061 0.0071 0.1912 537409 outcome TRUE reported
16305_10_CDH11_Cadherin_11 CDH11 chr5 72843961 0.008311 1.79e-09 35354
TRUE reported textfile 2 1 TRUE
1311 rs117572554 T C T C -0.2588 0.0044 0.01385 NA FALSE FALSE FALSE
GCST006061 0.0301 0.8827 537409 outcome TRUE reported
16305_10_CDH11_Cadherin_11 CDH11 chr14 104964549 0.035897 5.615e-13 35115
TRUE reported textfile 2 1 TRUE
1312 rs12065184 C A C A -0.1067 -0.0195 0.06358 NA FALSE FALSE FALSE
GCST006061 0.016 0.2215 537409 outcome TRUE reported
16305_10_CDH11_Cadherin_11 CDH11 chr1 161308887 0.017207 5.618e-10 35355
TRUE reported textfile 2 1 TRUE
1313 rs12094497 A G A G -0.1062 0.0046 0.15082 NA FALSE FALSE FALSE
GCST006061 0.0122 0.7061 537409 outcome TRUE reported
16305_10_CDH11_Cadherin_11 CDH11 chr1 161216513 0.011758 1.683e-19 35364
TRUE reported textfile 2 1 TRUE
1314 rs12148488 T G T G 0.0493 0.0172 0.48657 NA FALSE FALSE FALSE
GCST006061 0.0072 0.01643 537409 outcome TRUE reported
16305_10_CDH11_Cadherin_11 CDH11 chr15 75090201 0.008288 2.713e-09 35361
TRUE reported textfile 2 1 TRUE
1315 rs12152337 C T C T -0.0631 0.0168 0.30077 NA FALSE FALSE FALSE
GCST006061 0.0077 0.02963 537409 outcome TRUE reported
16305_10_CDH11_Cadherin_11 CDH11 chr3 58379544 0.009129 4.789e-12 35358
TRUE reported textfile 2 1 TRUE
1316 rs140978623 T C T C 0.1378 0.0012 0.04153 NA FALSE FALSE FALSE
GCST006061 0.0211 0.9553 537409 outcome TRUE reported
16305_10_CDH11_Cadherin_11 CDH11 chr1 160894403 0.021171 7.565e-11 35365
TRUE reported textfile 2 1 TRUE
1317 rs2516960 G A G A -0.1454 0.0066 0.18594 NA FALSE FALSE FALSE
GCST006061 0.0162 0.6836 537409 outcome TRUE reported
16305_10_CDH11_Cadherin_11 CDH11 chr14 105903676 0.011221 2.13e-38 34966
TRUE reported textfile 2 1 TRUE
1318 rs3822960 T C T C -0.143 0.0231 0.33381 NA FALSE FALSE FALSE
GCST006061 0.0076 0.002263 537409 outcome TRUE reported
16305_10_CDH11_Cadherin_11 CDH11 chr6 73521136 0.008841 7.627e-59 35350
TRUE reported textfile 2 1 TRUE
1319 rs390288 A C A C 0.0472 -0.0077 0.48247 NA FALSE FALSE FALSE
GCST006061 0.0072 0.283 537409 outcome TRUE reported
16305_10_CDH11_Cadherin_11 CDH11 chr4 2790730 0.008358 1.633e-08 35281
TRUE reported textfile 2 1 TRUE
1320 rs56260553 T C T C -0.0836 0.0012 0.22676 NA FALSE FALSE FALSE
GCST006061 0.0094 0.8998 537409 outcome TRUE reported
16305_10_CDH11_Cadherin_11 CDH11 chr20 4135073 0.00993 3.803e-17 35372 TRUE
reported textfile 2 1 TRUE
1321 rs7154507 G T G T -0.0829 0.0165 0.10181 NA FALSE FALSE FALSE
GCST006061 0.0151 0.2737 537409 outcome TRUE reported
16305_10_CDH11_Cadherin_11 CDH11 chr14 102668767 0.013948 2.791e-09 35376
TRUE reported textfile 2 1 TRUE
1322 rs7532925 A G A G -0.1172 -0.0087 0.06647 NA FALSE FALSE FALSE
GCST006061 0.0128 0.495 537409 outcome TRUE reported
16305_10_CDH11_Cadherin_11 CDH11 chr1 161675165 0.017004 5.489e-12 35351
TRUE reported textfile 2 1 TRUE
1323 rs79697519 T C T C 0.3217 0.0217 0.01312 NA FALSE FALSE FALSE
GCST006061 0.0565 0.7011 537409 outcome TRUE reported

16305_10_CDH11_Cadherin_11 CDH11 chr14 105115677 0.036599 1.499e-18 35113 TRUE reported textfile 2 1 TRUE
1324 rs10140610 C T C T 0.0888 -0.0105 0.38751 NA FALSE FALSE FALSE GCST006061 0.0075 0.159 537409 outcome TRUE reported 2643_57_CDH3_P_Cadherin CDH3 chr14 94577881 0.008367 2.59e-26 35372 TRUE reported textfile 2 1 TRUE
1325 rs10143099 A G A G 0.0688 -0.0129 0.20609 NA FALSE FALSE FALSE GCST006061 0.0087 0.1371 537409 outcome TRUE reported 2643_57_CDH3_P_Cadherin CDH3 chr14 94600298 0.00999 5.706e-12 35372 TRUE reported textfile 2 1 TRUE
1326 rs11080055 C A C A 0.2477 -0.0182 0.45317 NA FALSE FALSE FALSE GCST006061 0.0072 0.01138 537409 outcome TRUE reported 2643_57_CDH3_P_Cadherin CDH3 chr17 28322698 0.008093 1e-200 35360 TRUE reported textfile 2 1 TRUE
1327 rs1260326 C T C T -0.0749 -0.0069 0.34132 NA FALSE FALSE FALSE GCST006061 0.0073 0.3401 537409 outcome TRUE reported 2643_57_CDH3_P_Cadherin CDH3 chr2 27508073 0.008594 2.901e-18 35373 TRUE reported textfile 2 1 TRUE
1328 rs1706435 G A G A 0.0641 0.0117 0.36981 NA FALSE FALSE FALSE GCST006061 0.0072 0.1047 537409 outcome TRUE reported 2643_57_CDH3_P_Cadherin CDH3 chr3 165743688 0.008394 2.24e-14 35339 TRUE reported textfile 2 1 TRUE
1329 rs2731672 C T C T 0.0538 -0.0025 0.25436 NA FALSE FALSE FALSE GCST006061 0.0083 0.7666 537409 outcome TRUE reported 2643_57_CDH3_P_Cadherin CDH3 chr5 177415473 0.009375 9.547e-09 35362 TRUE reported textfile 2 1 TRUE
1330 rs55870921 T C T C -0.0513 -0.0076 0.33388 NA FALSE FALSE FALSE GCST006061 0.0078 0.3291 537409 outcome TRUE reported 2643_57_CDH3_P_Cadherin CDH3 chr17 27461032 0.008599 2.438e-09 35359 TRUE reported textfile 2 1 TRUE
1331 rs10142728 A G A G -0.1297 -0.0127 0.05637 NA FALSE FALSE FALSE GCST006061 0.0125 0.3086 537409 outcome TRUE reported 2879_9_SERPINA3_a1_Antichymotrypsin SERPINA3 chr14 93998143 0.016823 1.262e-14 35370 TRUE reported textfile 2 1 TRUE
1332 rs61976127 G A G A -0.2405 -0.0073 0.188 NA FALSE FALSE FALSE GCST006061 0.0097 0.4535 537409 outcome TRUE reported 2879_9_SERPINA3_a1_Antichymotrypsin SERPINA3 chr14 94631329 0.009966 1.146e-128 35372 TRUE reported textfile 2 1 TRUE
1333 rs75498941 T C T C -0.1952 -0.0025 0.0338 NA FALSE FALSE FALSE GCST006061 0.0187 0.8928 537409 outcome TRUE reported 2879_9_SERPINA3_a1_Antichymotrypsin SERPINA3 chr14 94744337 0.021318 5.352e-20 35372 TRUE reported textfile 2 1 TRUE
1334 rs10144543 T C T C 0.0564 -0.0084 0.42633 NA FALSE FALSE FALSE GCST006061 0.0074 0.2608 537409 outcome TRUE reported 15452_5_NT5E_5__Nucleotidase NT5E chr14 103108377 0.008091 3.161e-12 35293 TRUE reported textfile 2 1 TRUE
1335 rs111795396 T G T G -0.3749 0.0144 0.02781 NA FALSE FALSE FALSE GCST006061 0.022 0.5129 537409 outcome TRUE reported 15452_5_NT5E_5__Nucleotidase NT5E chr6 86569158 0.024354 1.798e-53 35346 TRUE reported textfile 2 1 TRUE
1336 rs174529 C T C T 0.0537 0.011 0.40033 NA FALSE FALSE FALSE GCST006061 0.0074 0.1401 537409 outcome TRUE reported 15452_5_NT5E_5__Nucleotidase NT5E chr11 61776489 0.008165 4.799e-11 35363 TRUE reported textfile 2 1 TRUE
1337 rs2414577 C T C T -0.0472 -0.0167 0.41557 NA FALSE FALSE FALSE GCST006061 0.0076 0.02713 537409 outcome TRUE reported 15452_5_NT5E_5__Nucleotidase NT5E chr15 58388439 0.008125 6.282e-09 35364 TRUE reported textfile 2 1 TRUE
1338 rs35887873 C T C T 0.052 -0.0114 0.26311 NA FALSE FALSE FALSE GCST006061 0.0091 0.2089 537409 outcome TRUE reported 15452_5_NT5E_5__Nucleotidase NT5E chr11 126349501 0.009052 9.229e-09 35374 TRUE reported textfile 2 1 TRUE
1339 rs416745 C T C T -0.07 5e-04 0.43336 NA FALSE FALSE FALSE GCST006061 0.0074 0.9515 537409 outcome TRUE reported 15452_5_NT5E_5__Nucleotidase NT5E chr20 25458232 0.008145 8.379e-18 35365 TRUE reported textfile 2 1 TRUE

1340 rs4296866 T C T C -0.1227 -0.0048 0.48761 NA FALSE FALSE FALSE
GCST006061 0.0072 0.5068 537409 outcome TRUE reported
15452_5_NT5E_5__Nucleotidase NT5E chr6 85570019 0.008175 6.434e-51
35346 TRUE reported textfile 2 1 TRUE
1341 rs632350 T C T C -0.5319 0.0143 0.11795 NA FALSE FALSE FALSE
GCST006061 0.0113 0.2066 537409 outcome TRUE reported
15452_5_NT5E_5__Nucleotidase NT5E chr6 85377625 0.010138 1e-200 35343
TRUE reported textfile 2 1 TRUE
1342 rs77448558 C T C T -0.0981 0.0466 0.05384 NA FALSE FALSE FALSE
GCST006061 0.0189 0.01372 537409 outcome TRUE reported
15452_5_NT5E_5__Nucleotidase NT5E chr6 102401219 0.017844 3.846e-08
35351 TRUE reported textfile 2 1 TRUE
1343 rs77562907 A G A G -0.1759 0.0209 0.02586 NA FALSE FALSE FALSE
GCST006061 0.0673 0.7559 537409 outcome TRUE reported
15452_5_NT5E_5__Nucleotidase NT5E chr1 11881265 0.025445 4.745e-12
35390 TRUE reported textfile 2 1 TRUE
1344 rs77705726 C A C A 0.3147 -0.013 0.01573 NA FALSE FALSE FALSE
GCST006061 0.0332 0.6952 537409 outcome TRUE reported
15452_5_NT5E_5__Nucleotidase NT5E chr6 87389947 0.033394 4.344e-21
35351 TRUE reported textfile 2 1 TRUE
1345 rs9294301 G A G A 0.064 0.0011 0.32226 NA FALSE FALSE FALSE
GCST006061 0.0075 0.8839 537409 outcome TRUE reported
15452_5_NT5E_5__Nucleotidase NT5E chr6 84529807 0.008696 1.843e-13
35343 TRUE reported textfile 2 1 TRUE
1346 rs10148939 C A C A -0.4238 0.012 0.11292 NA FALSE FALSE FALSE
GCST006061 0.0109 0.2699 537409 outcome TRUE reported 16060_99_NID2_NID2
NID2 chr14 51986941 0.012695 1e-200 35369 TRUE reported textfile
2 1 TRUE
1347 rs11757455 A G A G 0.0886 0.0278 0.06865 NA FALSE FALSE FALSE
GCST006061 0.0157 0.07714 537409 outcome TRUE reported 16060_99_NID2_NID2
NID2 chr6 112201651 0.016096 3.702e-08 35370 TRUE reported
textfile 2 1 TRUE
1348 rs150394939 C T C T 0.1825 -0.0639 0.02574 NA FALSE FALSE FALSE
GCST006061 0.0425 0.1322 537409 outcome TRUE reported 16060_99_NID2_NID2
NID2 chr14 49460073 0.025643 1.104e-12 35361 TRUE reported
textfile 2 1 TRUE
1349 rs34598814 T C T C 0.1015 -0.0109 0.39007 NA FALSE FALSE FALSE
GCST006061 0.0081 0.178 537409 outcome TRUE reported 16060_99_NID2_NID2
NID2 chr14 51759112 0.008385 9.861e-34 35367 TRUE reported
textfile 2 1 TRUE
1350 rs7073753 T C T C 0.0661 -0.0472 0.4356 NA FALSE FALSE FALSE
GCST006061 0.0072 5.02e-11 537409 outcome TRUE reported 16060_99_NID2_NID2
NID2 chr10 63303060 0.008036 1.952e-16 35351 TRUE reported
textfile 2 1 TRUE
1351 rs754423 T C T C -0.2398 0.0034 0.18084 NA FALSE FALSE FALSE
GCST006061 0.0083 0.6814 537409 outcome TRUE reported 16060_99_NID2_NID2
NID2 chr14 52060469 0.010652 3.116e-112 35367 TRUE reported
textfile 2 1 TRUE
1352 rs10149025 G A G A -0.1086 9e-04 0.10333 NA FALSE FALSE FALSE
GCST006061 0.0111 0.9369 537409 outcome TRUE reported
4153_11_SERPINA3_alpha_1_antichymotrypsin_complex SERPINA3 chr14 94594699
0.013435 6.313e-16 35372 TRUE reported textfile 2 1 TRUE
1353 rs118137024 T C T C 0.2265 0.0185 0.01973 NA FALSE FALSE FALSE
GCST006061 0.0294 0.5276 537409 outcome TRUE reported
4153_11_SERPINA3_alpha_1_antichymotrypsin_complex SERPINA3 chr19 990657 0.02937
1.238e-14 35320 TRUE reported textfile 2 1 TRUE
1354 rs17753556 A C A C -0.3217 -0.008 0.18833 NA FALSE FALSE FALSE
GCST006061 0.0098 0.4154 537409 outcome TRUE reported
4153_11_SERPINA3_alpha_1_antichymotrypsin_complex SERPINA3 chr14 94629097
0.010487 1e-200 35371 TRUE reported textfile 2 1 TRUE
1355 rs56317088 G A G A -0.183 -0.0586 0.03193 NA FALSE FALSE FALSE
GCST006061 0.0318 0.06566 537409 outcome TRUE reported
4153_11_SERPINA3_alpha_1_antichymotrypsin_complex SERPINA3 chr14 94285014
0.023651 1.013e-14 35372 TRUE reported textfile 2 1 TRUE
1356 rs77262773 T C T C 0.1729 0.014 0.02263 NA FALSE FALSE FALSE
GCST006061 0.0283 0.6205 537409 outcome TRUE reported

4153_11_SERPINA3_alpha_1_antichymotrypsin_complex SERPINA3 chr17 69253570
0.027099 1.768e-10 35371 TRUE reported textfile 2 1 TRUE
1357 rs10149770 A G A G 0.0876 0.0012 0.0918 NA FALSE FALSE FALSE
GCST006061 0.0137 0.9302 537409 outcome TRUE reported 19372_7_MDGA2_MDGA2
MDGA2 chr14 35918522 0.014043 4.438e-10 35362 TRUE reported
textfile 2 1 TRUE
1358 rs112351914 A G A G 0.1872 0.0228 0.04204 NA FALSE FALSE FALSE
GCST006061 0.0267 0.3947 537409 outcome TRUE reported 19372_7_MDGA2_MDGA2
MDGA2 chr14 48805400 0.020382 4.132e-20 35361 TRUE reported
textfile 2 1 TRUE
1359 rs116174082 A G A G 1.0801 -0.011 0.02108 NA FALSE FALSE FALSE
GCST006061 0.03 0.7135 537409 outcome TRUE reported 19372_7_MDGA2_MDGA2
MDGA2 chr14 48391779 0.020399 1e-200 35361 TRUE reported textfile
2 1 TRUE
1360 rs12147146 C T C T 0.596 0.0234 0.01482 NA FALSE FALSE FALSE
GCST006061 0.0325 0.4715 537409 outcome TRUE reported 19372_7_MDGA2_MDGA2
MDGA2 chr14 44697112 0.033776 1.1e-69 35368 TRUE reported textfile
2 1 TRUE
1361 rs139953520 G A G A 0.5455 -0.0456 0.01655 NA FALSE FALSE FALSE
GCST006061 0.0492 0.3547 537409 outcome TRUE reported 19372_7_MDGA2_MDGA2
MDGA2 chr14 42722500 0.031984 3.173e-65 35358 TRUE reported
textfile 2 1 TRUE
1362 rs146048337 T C T C 0.1582 0.0292 0.02306 NA FALSE FALSE FALSE
GCST006061 0.0439 0.5061 537409 outcome TRUE reported 19372_7_MDGA2_MDGA2
MDGA2 chr14 68666849 0.027517 8.965e-09 35357 TRUE reported
textfile 2 1 TRUE
1363 rs147722382 T C T C -0.2174 0.0986 0.01006 NA FALSE FALSE FALSE
GCST006061 0.0474 0.03742 537409 outcome TRUE reported 19372_7_MDGA2_MDGA2
MDGA2 chr14 43985091 0.039342 3.279e-08 35366 TRUE reported
textfile 2 1 TRUE
1364 rs148244051 T G T G 0.1506 -0.0103 0.02993 NA FALSE FALSE FALSE
GCST006061 0.0246 0.6769 537409 outcome TRUE reported 19372_7_MDGA2_MDGA2
MDGA2 chr14 36650611 0.023549 1.603e-10 35359 TRUE reported
textfile 2 1 TRUE
1365 rs149817759 A G A G -0.369 0.0812 0.01447 NA FALSE FALSE FALSE
GCST006061 0.1511 0.5909 537409 outcome TRUE reported 19372_7_MDGA2_MDGA2
MDGA2 chr14 47198429 0.033482 3.037e-28 35362 TRUE reported
textfile 2 1 TRUE
1366 rs34231016 T C T C 0.0616 0.0224 0.13608 NA FALSE FALSE FALSE
GCST006061 0.0107 0.03724 537409 outcome TRUE reported 19372_7_MDGA2_MDGA2
MDGA2 chr2 233361281 0.011211 3.919e-08 35374 TRUE reported
textfile 2 1 TRUE
1367 rs34489253 G A G A 0.0874 0.0134 0.46966 NA FALSE FALSE FALSE
GCST006061 0.0071 0.05947 537409 outcome TRUE reported 19372_7_MDGA2_MDGA2
MDGA2 chr14 68802341 0.008128 5.766e-27 35357 TRUE reported
textfile 2 1 TRUE
1368 rs4665972 C T C T 0.0485 -0.0046 0.33903 NA FALSE FALSE FALSE
GCST006061 0.0075 0.5416 537409 outcome TRUE reported 19372_7_MDGA2_MDGA2
MDGA2 chr2 27375230 0.00816 2.787e-09 35374 TRUE reported textfile
2 1 TRUE
1369 rs4788706 G A G A -0.0425 -0.0079 0.46077 NA FALSE FALSE FALSE
GCST006061 0.0071 0.2656 537409 outcome TRUE reported 19372_7_MDGA2_MDGA2
MDGA2 chr16 73157810 0.007758 4.298e-08 35367 TRUE reported
textfile 2 1 TRUE
1370 rs4900811 T C T C -0.5405 0.0122 0.1206 NA FALSE FALSE FALSE
GCST006061 0.014 0.3831 537409 outcome TRUE reported 19372_7_MDGA2_MDGA2
MDGA2 chr14 47840365 0.009024 1e-200 35362 TRUE reported textfile
2 1 TRUE
1371 rs57844210 G A G A -0.1191 -0.0233 0.06082 NA FALSE FALSE FALSE
GCST006061 0.0122 0.05553 537409 outcome TRUE reported 19372_7_MDGA2_MDGA2
MDGA2 chr15 37011201 0.016173 1.785e-13 35375 TRUE reported
textfile 2 1 TRUE
1372 rs62621812 A G A G 0.1446 0.0125 0.02528 NA FALSE FALSE FALSE
GCST006061 0.0292 0.6692 537409 outcome TRUE reported 19372_7_MDGA2_MDGA2
MDGA2 chr7 127375029 0.024288 2.622e-09 35356 TRUE reported
textfile 2 1 TRUE

1373 rs75694823 C A C A 0.3634 0.1023 0.01751 NA FALSE FALSE FALSE GCST006061 0.0576 0.07607 537409 outcome TRUE reported 19372_7_MDGA2_MDGA2 MDGA2 chr14 50941270 0.030385 5.767e-33 35365 TRUE reported textfile 2 1 TRUE
1374 rs77544995 C T C T -0.2403 0.0012 0.03402 NA FALSE FALSE FALSE GCST006061 0.0218 0.9578 537409 outcome TRUE reported 19372_7_MDGA2_MDGA2 MDGA2 chr14 47416698 0.022097 1.523e-27 35362 TRUE reported textfile 2 1 TRUE
1375 rs79827742 A G A G 0.2189 -0.0016 0.03472 NA FALSE FALSE FALSE GCST006061 0.0203 0.9382 537409 outcome TRUE reported 19372_7_MDGA2_MDGA2 MDGA2 chr14 48028066 0.022201 6.201e-23 35362 TRUE reported textfile 2 1 TRUE
1376 rs849133 T C T C -0.043 0.0028 0.46599 NA FALSE FALSE FALSE GCST006061 0.0072 0.6981 537409 outcome TRUE reported 19372_7_MDGA2_MDGA2 MDGA2 chr7 28152661 0.00773 2.657e-08 35374 TRUE reported textfile 2 1 TRUE
1377 rs10151220 C T C T 0.0483 0.0122 0.37642 NA FALSE FALSE FALSE GCST006061 0.0075 0.1042 537409 outcome TRUE reported 11302_237_TNR_TENR TNR chr14 34246259 0.008346 7.151e-09 35358 TRUE reported textfile 2 1 TRUE
1378 rs10922098 T C T C -0.1577 -0.009 0.40011 NA FALSE FALSE FALSE GCST006061 0.0075 0.2275 537409 outcome TRUE reported 11302_237_TNR_TENR TNR chr1 196695521 0.008249 1.786e-81 35329 TRUE reported textfile 2 1 TRUE
1379 rs2235256 G A G A -0.1315 0.0089 0.4708 NA FALSE FALSE FALSE GCST006061 0.0073 0.2198 537409 outcome TRUE reported 11302_237_TNR_TENR TNR chr1 175599911 0.008083 1.667e-59 35340 TRUE reported textfile 2 1 TRUE
1380 rs2636882 C T C T 0.0513 -0.0021 0.37525 NA FALSE FALSE FALSE GCST006061 0.0076 0.7787 537409 outcome TRUE reported 11302_237_TNR_TENR TNR chr9 114334462 0.008278 5.744e-10 35352 TRUE reported textfile 2 1 TRUE
1381 rs35166255 A G A G 0.1279 0.0222 0.0367 NA FALSE FALSE FALSE GCST006061 0.0229 0.3329 537409 outcome TRUE reported 11302_237_TNR_TENR TNR chr11 126431861 0.020814 7.994e-10 35362 TRUE reported textfile 2 1 TRUE
1382 rs704 A G A G 0.1034 -0.0155 0.49189 NA FALSE FALSE FALSE GCST006061 0.0072 0.03144 537409 outcome TRUE reported 11302_237_TNR_TENR TNR chr17 28367840 0.008097 2.428e-37 35351 TRUE reported textfile 2 1 TRUE
1383 rs10156191 T C T C -0.579 -0.0013 0.26629 NA FALSE FALSE FALSE GCST006061 0.0083 0.8741 537409 outcome TRUE reported 15486_126_AOC1_ABP1 AOC1 chr7 150856517 0.006154 1e-200 35375 TRUE reported textfile 2 1 TRUE
1384 rs10237223 C T C T 0.0794 0.0038 0.30038 NA FALSE FALSE FALSE GCST006061 0.008 0.6364 537409 outcome TRUE reported 15486_126_AOC1_ABP1 AOC1 chr7 151953891 0.009428 3.711e-17 35378 TRUE reported textfile 2 1 TRUE
1385 rs10253089 T C T C -0.158 0.0264 0.04427 NA FALSE FALSE FALSE GCST006061 0.0191 0.1655 537409 outcome TRUE reported 15486_126_AOC1_ABP1 AOC1 chr7 149918952 0.020726 2.47e-14 35363 TRUE reported textfile 2 1 TRUE
1386 rs10952717 C T C T -0.057 -8.00E-04 0.26655 NA FALSE FALSE FALSE GCST006061 0.008 0.9207 537409 outcome TRUE reported 15486_126_AOC1_ABP1 AOC1 chr7 147940361 0.009834 6.79e-09 35374 TRUE reported textfile 2 1 TRUE
1387 rs1260326 C T C T 0.0452 -0.0069 0.34132 NA FALSE FALSE FALSE GCST006061 0.0073 0.3401 537409 outcome TRUE reported 15486_126_AOC1_ABP1 AOC1 chr2 27508073 0.007962 1.372e-08 35373 TRUE reported textfile 2 1 TRUE
1388 rs36036364 T C T C 0.0637 6e-04 0.4161 NA FALSE FALSE FALSE GCST006061 0.0081 0.9442 537409 outcome TRUE reported 15486_126_AOC1_ABP1 AOC1 chr7 152689842 0.008792 4.318e-13 35373 TRUE reported textfile 2 1 TRUE
1389 rs77855615 T C T C 0.9595 0.092 0.01139 NA FALSE FALSE FALSE GCST006061 0.0593 0.1206 537409 outcome TRUE reported 15486_126_AOC1_ABP1 AOC1 chr7 151205794 0.038882 1.876e-134 35375 TRUE reported

textfile 2 1 TRUE
1390 rs10157710 T C T C 0.1151 -0.007 0.19506 NA FALSE FALSE FALSE
GCST006061 0.0087 0.4225 537409 outcome TRUE reported
5491_12_SPOCK2_Testican_2 SPOCK2 chr1 47496019 0.010455 3.449e-28 35368
TRUE reported textfile 2 1 TRUE
1391 rs11722445 T C T C 0.0625 -0.0177 0.29372 NA FALSE FALSE FALSE
GCST006061 0.0081 0.02821 537409 outcome TRUE reported
5491_12_SPOCK2_Testican_2 SPOCK2 chr4 81287746 0.00908 5.846e-12 35350
TRUE reported textfile 2 1 TRUE
1392 rs1245548 A G A G 0.1888 -0.0065 0.45859 NA FALSE FALSE FALSE
GCST006061 0.0072 0.3639 537409 outcome TRUE reported
5491_12_SPOCK2_Testican_2 SPOCK2 chr10 72086126 0.008221 1.014e-116 35372
TRUE reported textfile 2 1 TRUE
1393 rs13210483 C T C T -0.0604 -0.0212 0.26541 NA FALSE FALSE FALSE
GCST006061 0.0082 0.01015 537409 outcome TRUE reported
5491_12_SPOCK2_Testican_2 SPOCK2 chr6 15099304 0.009335 9.763e-11 35384
TRUE reported textfile 2 1 TRUE
1394 rs138646309 C A C A -0.2238 -0.0089 0.01472 NA FALSE FALSE FALSE
GCST006061 0.0447 0.8419 537409 outcome TRUE reported
5491_12_SPOCK2_Testican_2 SPOCK2 chr10 77022364 0.033925 4.197e-11 35368
TRUE reported textfile 2 1 TRUE
1395 rs141343804 A C A C -0.1559 -0.0138 0.03568 NA FALSE FALSE FALSE
GCST006061 0.0257 0.5908 537409 outcome TRUE reported
5491_12_SPOCK2_Testican_2 SPOCK2 chr10 76431747 0.022723 6.847e-12 35366
TRUE reported textfile 2 1 TRUE
1396 rs143760180 A G A G -0.2174 0.0806 0.0108 NA FALSE FALSE FALSE
GCST006061 0.0455 0.07644 537409 outcome TRUE reported
5491_12_SPOCK2_Testican_2 SPOCK2 chr10 78933172 0.039199 2.921e-08 35370
TRUE reported textfile 2 1 TRUE
1397 rs192127052 A G A G -0.1839 0.0196 0.01905 NA FALSE FALSE FALSE
GCST006061 0.0215 0.3625 537409 outcome TRUE reported
5491_12_SPOCK2_Testican_2 SPOCK2 chr10 80543646 0.03029 1.268e-09 35368
TRUE reported textfile 2 1 TRUE
1398 rs72820511 A G A G -0.3899 -0.0353 0.01354 NA FALSE FALSE FALSE
GCST006061 0.0419 0.3998 537409 outcome TRUE reported
5491_12_SPOCK2_Testican_2 SPOCK2 chr10 74616541 0.036229 5.198e-27 35368
TRUE reported textfile 2 1 TRUE
1399 rs80279219 A G A G -0.1103 0.0203 0.04814 NA FALSE FALSE FALSE
GCST006061 0.0199 0.3072 537409 outcome TRUE reported
5491_12_SPOCK2_Testican_2 SPOCK2 chr12 112472415 0.019146 8.369e-09 35370
TRUE reported textfile 2 1 TRUE
1400 rs10159712 A G A G 0.1399 -0.0104 0.2838 NA FALSE FALSE FALSE
GCST006061 0.0079 0.1855 537409 outcome TRUE reported
4906_35_F5_Coagulation_Factor_V F5 chr10 19971440 0.008893 9.186e-56 35360
TRUE reported textfile 2 1 TRUE
1401 rs10437425 G T G T -0.0498 0.0072 0.27349 NA FALSE FALSE FALSE
GCST006061 0.0078 0.3515 537409 outcome TRUE reported
4906_35_F5_Coagulation_Factor_V F5 chr10 20078274 0.00896 2.729e-08 35363
TRUE reported textfile 2 1 TRUE
1402 rs1051317 T G T G 0.0597 -0.001 0.29891 NA FALSE FALSE FALSE
GCST006061 0.0081 0.9035 537409 outcome TRUE reported
4906_35_F5_Coagulation_Factor_V F5 chr2 46903009 0.008709 7.141e-12 35358
TRUE reported textfile 2 1 TRUE
1403 rs115977231 A G A G -0.2205 0.0568 0.01907 NA FALSE FALSE FALSE
GCST006061 0.0352 0.1065 537409 outcome TRUE reported
4906_35_F5_Coagulation_Factor_V F5 chr1 169097491 0.028848 2.113e-14 35361
TRUE reported textfile 2 1 TRUE
1404 rs149062332 C T C T 0.1557 0.0424 0.02045 NA FALSE FALSE FALSE
GCST006061 0.0435 0.33 537409 outcome TRUE reported
4906_35_F5_Coagulation_Factor_V F5 chr3 186623236 0.027511 1.518e-08 35367
TRUE reported textfile 2 1 TRUE
1405 rs2239853 C T C T -0.1213 0.0039 0.44202 NA FALSE FALSE FALSE
GCST006061 0.0073 0.5928 537409 outcome TRUE reported
4906_35_F5_Coagulation_Factor_V F5 chr1 169556528 0.008126 2.173e-50 35353
TRUE reported textfile 2 1 TRUE
1406 rs28360431 C T C T 0.1661 -0.0055 0.01834 NA FALSE FALSE FALSE

GCST006061 0.0337 0.8702 537409 outcome TRUE reported
4906_35_F5_Coagulation_Factor_V F5 chr1 171285127 0.030158 3.636e-08 35360
TRUE reported textfile 2 1 TRUE
1407 rs4654748 T C T C -0.0481 -0.0034 0.43409 NA FALSE FALSE FALSE
GCST006061 0.0073 0.6384 537409 outcome TRUE reported
4906_35_F5_Coagulation_Factor_V F5 chr1 21459575 0.008144 3.499e-09 35374
TRUE reported textfile 2 1 TRUE
1408 rs55731794 A G A G 0.0456 -0.002 0.44407 NA FALSE FALSE FALSE
GCST006061 0.0074 0.7846 537409 outcome TRUE reported
4906_35_F5_Coagulation_Factor_V F5 chr19 7765005 0.008038 1.401e-08 35372
TRUE reported textfile 2 1 TRUE
1409 rs61806985 T C T C -0.5 -0.0247 0.07704 NA FALSE FALSE FALSE
GCST006061 0.0227 0.2752 537409 outcome TRUE reported
4906_35_F5_Coagulation_Factor_V F5 chr1 169330603 0.014735 1e-200 35364
TRUE reported textfile 2 1 TRUE
1410 rs7894992 A G A G -0.0943 -0.0052 0.49304 NA FALSE FALSE FALSE
GCST006061 0.0071 0.4628 537409 outcome TRUE reported
4906_35_F5_Coagulation_Factor_V F5 chr10 19795284 0.007935 1.445e-32 35358
TRUE reported textfile 2 1 TRUE
1411 rs10159712 A G A G 0.127 -0.0104 0.2838 NA FALSE FALSE FALSE
GCST006061 0.0079 0.1855 537409 outcome TRUE reported 14689_3_SEC13_SEC13
SEC13 chr10 19971440 0.008952 1.114e-45 35360 TRUE reported
textfile 2 1 TRUE
1412 rs10437425 G T G T -0.0494 0.0072 0.27349 NA FALSE FALSE FALSE
GCST006061 0.0078 0.3515 537409 outcome TRUE reported 14689_3_SEC13_SEC13
SEC13 chr10 20078274 0.008997 4.002e-08 35363 TRUE reported
textfile 2 1 TRUE
1413 rs10799692 T C T C -0.0466 1e-04 0.48313 NA FALSE FALSE FALSE
GCST006061 0.0074 0.9895 537409 outcome TRUE reported 14689_3_SEC13_SEC13
SEC13 chr1 21449450 0.008064 7.535e-09 35374 TRUE reported
textfile 2 1 TRUE
1414 rs1265109 T G T G -0.0475 0.006 0.32126 NA FALSE FALSE FALSE
GCST006061 0.008 0.4516 537409 outcome TRUE reported 14689_3_SEC13_SEC13
SEC13 chr6 31151812 0.008592 3.234e-08 35439 TRUE reported
textfile 2 1 TRUE
1415 rs148108365 A G A G 0.1505 -0.038 0.02374 NA FALSE FALSE FALSE
GCST006061 0.0333 0.2545 537409 outcome TRUE reported 14689_3_SEC13_SEC13
SEC13 chr10 20241932 0.025882 6.067e-09 35375 TRUE reported
textfile 2 1 TRUE
1416 rs594793 T G T G -0.0463 0.0085 0.43083 NA FALSE FALSE FALSE
GCST006061 0.0076 0.2613 537409 outcome TRUE reported 14689_3_SEC13_SEC13
SEC13 chr19 7765060 0.008152 1.349e-08 35373 TRUE reported textfile
2 1 TRUE
1417 rs62140549 G A G A 0.0483 -3.00E-04 0.30898 NA FALSE FALSE FALSE
GCST006061 0.008 0.9672 537409 outcome TRUE reported 14689_3_SEC13_SEC13
SEC13 chr2 46914826 0.008705 2.879e-08 35358 TRUE reported
textfile 2 1 TRUE
1418 rs679899 A G A G 0.1343 -0.0016 0.48235 NA FALSE FALSE FALSE
GCST006061 0.0073 0.8278 537409 outcome TRUE reported 14689_3_SEC13_SEC13
SEC13 chr2 21028042 0.008057 2.195e-62 35373 TRUE reported
textfile 2 1 TRUE
1419 rs72702154 C T C T -0.1505 -0.0366 0.022 NA FALSE FALSE FALSE
GCST006061 0.0366 0.3177 537409 outcome TRUE reported 14689_3_SEC13_SEC13
SEC13 chr1 169360691 0.026552 1.443e-08 35364 TRUE reported
textfile 2 1 TRUE
1420 rs74717300 T G T G -0.0849 0.0033 0.08971 NA FALSE FALSE FALSE
GCST006061 0.0178 0.8533 537409 outcome TRUE reported 14689_3_SEC13_SEC13
SEC13 chr2 21313631 0.014036 1.46e-09 35372 TRUE reported
textfile 2 1 TRUE
1421 rs7535409 G A G A -0.1317 -7.00E-04 0.33792 NA FALSE FALSE FALSE
GCST006061 0.0075 0.9311 537409 outcome TRUE reported 14689_3_SEC13_SEC13
SEC13 chr1 169537003 0.008476 1.905e-54 35364 TRUE reported
textfile 2 1 TRUE
1422 rs10159712 A G A G 0.0553 -0.0104 0.2838 NA FALSE FALSE FALSE
GCST006061 0.0079 0.1855 537409 outcome TRUE reported 3592_4_CAMK1_CAMK1
CAMK1 chr10 19971440 0.008312 2.871e-11 35360 TRUE reported

textfile 2 1 TRUE
1423 rs113258252 A C A C 0.1796 -0.0683 0.02031 NA FALSE FALSE FALSE
GCST006061 0.0288 0.01763 537409 outcome TRUE reported 3592_4_CAMK1_CAMK1
CAMK1 chr1 171038101 0.027875 1.171e-10 35352 TRUE reported
textfile 2 1 TRUE
1424 rs12402584 T G T G 0.1337 0.0124 0.15162 NA FALSE FALSE FALSE
GCST006061 0.0102 0.2231 537409 outcome TRUE reported 3592_4_CAMK1_CAMK1
CAMK1 chr1 170826356 0.011187 6.365e-33 35359 TRUE reported
textfile 2 1 TRUE
1425 rs138517625 G A G A -0.2073 -0.0383 0.01825 NA FALSE FALSE FALSE
GCST006061 0.0467 0.4116 537409 outcome TRUE reported 3592_4_CAMK1_CAMK1
CAMK1 chr1 172712416 0.030202 6.708e-12 35352 TRUE reported
textfile 2 1 TRUE
1426 rs148302954 A G A G -0.3933 0.0245 0.01196 NA FALSE FALSE FALSE
GCST006061 0.0586 0.676 537409 outcome TRUE reported 3592_4_CAMK1_CAMK1
CAMK1 chr1 171252382 0.037751 2.048e-25 35352 TRUE reported
textfile 2 1 TRUE
1427 rs3908047 A G A G -0.0921 -0.0209 0.07139 NA FALSE FALSE FALSE
GCST006061 0.0148 0.1589 537409 outcome TRUE reported 3592_4_CAMK1_CAMK1
CAMK1 chr1 180915663 0.015495 2.782e-09 35355 TRUE reported
textfile 2 1 TRUE
1428 rs3917727 G A G A -0.1546 -0.0088 0.36908 NA FALSE FALSE FALSE
GCST006061 0.0078 0.2631 537409 outcome TRUE reported 3592_4_CAMK1_CAMK1
CAMK1 chr1 169612020 0.008249 2.254e-78 35363 TRUE reported
textfile 2 1 TRUE
1429 rs56168465 A C A C -0.1822 0.0073 0.0432 NA FALSE FALSE FALSE
GCST006061 0.0286 0.7997 537409 outcome TRUE reported 3592_4_CAMK1_CAMK1
CAMK1 chr1 168268135 0.0196 1.461e-20 35367 TRUE reported textfile
2 1 TRUE
1430 rs61751507 T C T C 0.1948 0.0047 0.04162 NA FALSE FALSE FALSE
GCST006061 0.018 0.794 537409 outcome TRUE reported 3592_4_CAMK1_CAMK1
CAMK1 chr10 100069757 0.018586 1.056e-25 35368 TRUE reported
textfile 2 1 TRUE
1431 rs61826722 T C T C -0.173 0.0236 0.02271 NA FALSE FALSE FALSE
GCST006061 0.0291 0.4183 537409 outcome TRUE reported 3592_4_CAMK1_CAMK1
CAMK1 chr1 173401891 0.027238 2.132e-10 35351 TRUE reported
textfile 2 1 TRUE
1432 rs71635640 G A G A -0.2358 0.0725 0.03774 NA FALSE FALSE FALSE
GCST006061 0.022 0.0009612 537409 outcome TRUE reported 3592_4_CAMK1_CAMK1
CAMK1 chr1 170019672 0.020648 3.33e-30 35362 TRUE reported
textfile 2 1 TRUE
1433 rs9332591 A G A G -0.6847 0.0016 0.13975 NA FALSE FALSE FALSE
GCST006061 0.0117 0.8906 537409 outcome TRUE reported 3592_4_CAMK1_CAMK1
CAMK1 chr1 169545552 0.008337 1e-200 35362 TRUE reported textfile
2 1 TRUE
1434 rs10159712 A G A G 0.1872 -0.0104 0.2838 NA FALSE FALSE FALSE
GCST006061 0.0079 0.1855 537409 outcome TRUE reported 9969_8_SLC22A16_S22AG
SLC22A16 chr10 19971440 0.00911 7.84e-94 35360 TRUE reported
textfile 2 1 TRUE
1435 rs10437425 G T G T -0.0629 0.0072 0.27349 NA FALSE FALSE FALSE
GCST006061 0.0078 0.3515 537409 outcome TRUE reported 9969_8_SLC22A16_S22AG
SLC22A16 chr10 20078274 0.009186 7.511e-12 35363 TRUE reported
textfile 2 1 TRUE
1436 rs11012153 A C A C -0.0935 0.017 0.0829 NA FALSE FALSE FALSE
GCST006061 0.0165 0.3026 537409 outcome TRUE reported 9969_8_SLC22A16_S22AG
SLC22A16 chr10 20565668 0.01448 1.068e-10 35369 TRUE reported
textfile 2 1 TRUE
1437 rs1591734 T C T C -0.1268 0.0198 0.1303 NA FALSE FALSE FALSE
GCST006061 0.0103 0.05506 537409 outcome TRUE reported 9969_8_SLC22A16_S22AG
SLC22A16 chr1 169302664 0.012344 9.434e-25 35364 TRUE reported
textfile 2 1 TRUE
1438 rs3820296 C T C T -0.0571 -4.00E-04 0.49913 NA FALSE FALSE FALSE
GCST006061 0.0073 0.9563 537409 outcome TRUE reported 9969_8_SLC22A16_S22AG
SLC22A16 chr1 21479954 0.008239 4.195e-12 35374 TRUE reported
textfile 2 1 TRUE
1439 rs594793 T G T G -0.0555 0.0085 0.43083 NA FALSE FALSE FALSE

GCST006061 0.0076 0.2613 537409 outcome TRUE reported 9969_8_SLC22A16_S22AG
SLC22A16 chr19 7765060 0.008285 2.099e-11 35373 TRUE reported
textfile 2 1 TRUE
1440 rs61808983 T C T C -0.407 -0.0103 0.07171 NA FALSE FALSE FALSE
GCST006061 0.0197 0.6008 537409 outcome TRUE reported 9969_8_SLC22A16_S22AG
SLC22A16 chr1 169505159 0.015731 1.382e-147 35363 TRUE reported
textfile 2 1 TRUE
1441 rs6544941 A G A G -0.071 0.0035 0.33068 NA FALSE FALSE FALSE
GCST006061 0.0077 0.6538 537409 outcome TRUE reported 9969_8_SLC22A16_S22AG
SLC22A16 chr2 46947977 0.008699 3.301e-16 35358 TRUE reported
textfile 2 1 TRUE
1442 rs6670678 G A G A -0.1716 0.0024 0.47186 NA FALSE FALSE FALSE
GCST006061 0.0073 0.7376 537409 outcome TRUE reported 9969_8_SLC22A16_S22AG
SLC22A16 chr1 169568440 0.008232 1.637e-96 35360 TRUE reported
textfile 2 1 TRUE
1443 rs7894992 A G A G -0.1054 -0.0052 0.49304 NA FALSE FALSE FALSE
GCST006061 0.0071 0.4628 537409 outcome TRUE reported 9969_8_SLC22A16_S22AG
SLC22A16 chr10 19795284 0.008147 2.778e-38 35358 TRUE reported
textfile 2 1 TRUE
1444 rs10159712 A G A G 0.1829 -0.0104 0.2838 NA FALSE FALSE FALSE
GCST006061 0.0079 0.1855 537409 outcome TRUE reported 8903_1_COX6C_COX6C
COX6C chr10 19971440 0.009056 1.056e-90 35360 TRUE reported
textfile 2 1 TRUE
1445 rs10437425 G T G T -0.068 0.0072 0.27349 NA FALSE FALSE FALSE
GCST006061 0.0078 0.3515 537409 outcome TRUE reported 8903_1_COX6C_COX6C
COX6C chr10 20078274 0.009132 9.601e-14 35363 TRUE reported
textfile 2 1 TRUE
1446 rs10764161 T C T C -0.0926 -0.0053 0.4988 NA FALSE FALSE FALSE
GCST006061 0.0071 0.4565 537409 outcome TRUE reported 8903_1_COX6C_COX6C
COX6C chr10 19797425 0.008098 2.796e-30 35358 TRUE reported
textfile 2 1 TRUE
1447 rs11012183 C T C T -0.0868 0.0152 0.07408 NA FALSE FALSE FALSE
GCST006061 0.0169 0.3705 537409 outcome TRUE reported 8903_1_COX6C_COX6C
COX6C chr10 20603824 0.01578 3.787e-08 35368 TRUE reported textfile
2 1 TRUE
1448 rs1106357 T C T C -0.0593 6e-04 0.40249 NA FALSE FALSE FALSE
GCST006061 0.0072 0.9314 537409 outcome TRUE reported 8903_1_COX6C_COX6C
COX6C chr1 21490592 0.008356 1.275e-12 35374 TRUE reported
textfile 2 1 TRUE
1449 rs1591734 T C T C -0.1453 0.0198 0.1303 NA FALSE FALSE FALSE
GCST006061 0.0103 0.05506 537409 outcome TRUE reported 8903_1_COX6C_COX6C
COX6C chr1 169302664 0.012231 1.509e-32 35364 TRUE reported
textfile 2 1 TRUE
1450 rs2239853 C T C T -0.1519 0.0039 0.44202 NA FALSE FALSE FALSE
GCST006061 0.0073 0.5928 537409 outcome TRUE reported 8903_1_COX6C_COX6C
COX6C chr1 169556528 0.008235 5.702e-76 35353 TRUE reported
textfile 2 1 TRUE
1451 rs594793 T G T G -0.0531 0.0085 0.43083 NA FALSE FALSE FALSE
GCST006061 0.0076 0.2613 537409 outcome TRUE reported 8903_1_COX6C_COX6C
COX6C chr19 7765060 0.008244 1.186e-10 35373 TRUE reported textfile
2 1 TRUE
1452 rs62140549 G A G A 0.0755 -3.00E-04 0.30898 NA FALSE FALSE FALSE
GCST006061 0.008 0.9672 537409 outcome TRUE reported 8903_1_COX6C_COX6C
COX6C chr2 46914826 0.00879 8.764e-18 35358 TRUE reported textfile
2 1 TRUE
1453 rs9332701 G A G A -0.4042 -0.0115 0.07189 NA FALSE FALSE FALSE
GCST006061 0.0196 0.5575 537409 outcome TRUE reported 8903_1_COX6C_COX6C
COX6C chr1 169515529 0.015595 4.095e-148 35364 TRUE reported
textfile 2 1 TRUE
1454 rs10159712 A G A G 0.0642 -0.0104 0.2838 NA FALSE FALSE FALSE
GCST006061 0.0079 0.1855 537409 outcome TRUE reported 6940_18_JPH1_JPH1
JPH1 chr10 19971440 0.008861 4.304e-13 35360 TRUE reported
textfile 2 1 TRUE
1455 rs10922098 T C T C -0.0722 -0.009 0.40011 NA FALSE FALSE FALSE
GCST006061 0.0075 0.2275 537409 outcome TRUE reported 6940_18_JPH1_JPH1
JPH1 chr1 196695521 0.008142 7.473e-19 35338 TRUE reported

textfile 2 1 TRUE
1456 rs145300538 A G A G -0.2224 -0.058 0.02178 NA FALSE FALSE FALSE
GCST006061 0.043 0.1771 537409 outcome TRUE reported 6940_18_JPH1_JPH1
JPH1 chr1 169284131 0.026336 3.048e-17 35364 TRUE reported
textfile 2 1 TRUE
1457 rs6127 T C T C -0.0595 0.004 0.47627 NA FALSE FALSE FALSE GCST006061
0.0074 0.5851 537409 outcome TRUE reported 6940_18_JPH1_JPH1 JPH1 chr1
169597075 0.007983 9.117e-14 35360 TRUE reported textfile 2 1 TRUE
1458 rs6544941 A G A G -0.0485 0.0035 0.33068 NA FALSE FALSE FALSE
GCST006061 0.0077 0.6538 537409 outcome TRUE reported 6940_18_JPH1_JPH1
JPH1 chr2 46947977 0.008466 1.01e-08 35358 TRUE reported
textfile 2 1 TRUE
1459 rs6672595 T C T C 0.1135 -0.0074 0.27909 NA FALSE FALSE FALSE
GCST006061 0.0085 0.3844 537409 outcome TRUE reported 6940_18_JPH1_JPH1
JPH1 chr1 169546298 0.008866 1.602e-37 35362 TRUE reported
textfile 2 1 TRUE
1460 rs10159712 A G A G 0.112 -0.0104 0.2838 NA FALSE FALSE FALSE
GCST006061 0.0079 0.1855 537409 outcome TRUE reported 3336_50_TFPI_TFPI
TFPI chr10 19971440 0.009191 3.716e-34 35360 TRUE reported
textfile 2 1 TRUE
1461 rs112875651 A G A G -0.0555 -0.0016 0.40253 NA FALSE FALSE FALSE
GCST006061 0.0076 0.8301 537409 outcome TRUE reported 3336_50_TFPI_TFPI
TFPI chr8 125494452 0.008472 5.723e-11 35367 TRUE reported
textfile 2 1 TRUE
1462 rs1209731 C T C T 0.2698 0.0293 0.01755 NA FALSE FALSE FALSE
GCST006061 0.0254 0.2489 537409 outcome TRUE reported 3336_50_TFPI_TFPI
TFPI chr1 169355555 0.031092 4.048e-18 35364 TRUE reported
textfile 2 1 TRUE
1463 rs6127 T C T C -0.1026 0.004 0.47627 NA FALSE FALSE FALSE GCST006061
0.0074 0.5851 537409 outcome TRUE reported 3336_50_TFPI_TFPI TFPI chr1
169597075 0.008321 6.211e-35 35360 TRUE reported textfile 2 1 TRUE
1464 rs6700487 C T C T 0.059 -0.0048 0.27952 NA FALSE FALSE FALSE
GCST006061 0.0078 0.5384 537409 outcome TRUE reported 3336_50_TFPI_TFPI
TFPI chr1 168580647 0.009299 2.228e-10 35364 TRUE reported
textfile 2 1 TRUE
1465 rs77262773 T C T C 0.1617 0.014 0.02263 NA FALSE FALSE FALSE
GCST006061 0.0283 0.6205 537409 outcome TRUE reported 3336_50_TFPI_TFPI
TFPI chr17 69253570 0.027386 3.536e-09 35371 TRUE reported
textfile 2 1 TRUE
1466 rs7894992 A G A G -0.0633 -0.0052 0.49304 NA FALSE FALSE FALSE
GCST006061 0.0071 0.4628 537409 outcome TRUE reported 3336_50_TFPI_TFPI
TFPI chr10 19795284 0.008211 1.264e-14 35358 TRUE reported
textfile 2 1 TRUE
1467 rs974793 T C T C 0.1869 -0.0065 0.3095 NA FALSE FALSE FALSE
GCST006061 0.008 0.4188 537409 outcome TRUE reported 3336_50_TFPI_TFPI
TFPI chr1 169509416 0.008941 4.962e-97 35363 TRUE reported
textfile 2 1 TRUE
1468 rs10159712 A G A G 0.1466 -0.0104 0.2838 NA FALSE FALSE FALSE
GCST006061 0.0079 0.1855 537409 outcome TRUE reported 8009_121_SURF1_SURF1
SURF1 chr10 19971440 0.009245 1.261e-56 35360 TRUE reported
textfile 2 1 TRUE
1469 rs10437425 G T G T -0.0558 0.0072 0.27349 NA FALSE FALSE FALSE
GCST006061 0.0078 0.3515 537409 outcome TRUE reported 8009_121_SURF1_SURF1
SURF1 chr10 20078274 0.009304 2.003e-09 35363 TRUE reported
textfile 2 1 TRUE
1470 rs11012153 A C A C -0.0895 0.017 0.0829 NA FALSE FALSE FALSE
GCST006061 0.0165 0.3026 537409 outcome TRUE reported 8009_121_SURF1_SURF1
SURF1 chr10 20565668 0.01465 1e-09 35369 TRUE reported textfile 2
1 TRUE
1471 rs11024589 C A C A -0.0899 0.0042 0.06935 NA FALSE FALSE FALSE
GCST006061 0.0122 0.7314 537409 outcome TRUE reported 8009_121_SURF1_SURF1
SURF1 chr11 18256876 0.016382 4.07e-08 35381 TRUE reported
textfile 2 1 TRUE
1472 rs117606766 T G T G 0.1267 0.0088 0.05509 NA FALSE FALSE FALSE
GCST006061 0.025 0.7235 537409 outcome TRUE reported 8009_121_SURF1_SURF1
SURF1 chr11 17726095 0.018186 3.238e-12 35370 TRUE reported

```
textfile	2	1	TRUE
1473	rs12053441	A	G	A	G	-0.0537	0.004	0.29903	NA	FALSE	FALSE	FALSE	GCST006061	0.0078	0.6064	537409	outcome	TRUE	reported	8009_121_SURF1_SURF1	SURF1	chr2	46961671	0.009102	3.636e-09	35357	TRUE	reported	textfile	2	1	TRUE
1474	rs1591734	T	C	T	C	-0.1245	0.0198	0.1303	NA	FALSE	FALSE	FALSE	GCST006061	0.0103	0.05506	537409	outcome	TRUE	reported	8009_121_SURF1_SURF1	SURF1	chr1	169302664	0.012473	1.838e-23	35364	TRUE	reported	textfile	2	1	TRUE
1475	rs2668202	G	A	G	A	-0.0802	-0.0076	0.30991	NA	FALSE	FALSE	FALSE	GCST006061	0.0074	0.3015	537409	outcome	TRUE	reported	8009_121_SURF1_SURF1	SURF1	chr3	165772680	0.008966	3.737e-19	35339	TRUE	reported	textfile	2	1	TRUE
1476	rs2956634	C	T	C	T	-0.7337	-0.0169	0.02769	NA	FALSE	FALSE	FALSE	GCST006061	0.0197	0.3905	537409	outcome	TRUE	reported	8009_121_SURF1_SURF1	SURF1	chr11	18226152	0.024778	1.088e-192	35383	TRUE	reported	textfile	2	1	TRUE
1477	rs548589	G	T	G	T	-0.0641	0.0035	0.36172	NA	FALSE	FALSE	FALSE	GCST006061	0.0076	0.6437	537409	outcome	TRUE	reported	8009_121_SURF1_SURF1	SURF1	chr11	17902313	0.008688	1.606e-13	35387	TRUE	reported	textfile	2	1	TRUE
1478	rs61808983	T	C	T	C	-0.3136	-0.0103	0.07171	NA	FALSE	FALSE	FALSE	GCST006061	0.0197	0.6008	537409	outcome	TRUE	reported	8009_121_SURF1_SURF1	SURF1	chr1	169505159	0.015954	5.123e-86	35363	TRUE	reported	textfile	2	1	TRUE
1479	rs6670678	G	A	G	A	-0.1402	0.0024	0.47186	NA	FALSE	FALSE	FALSE	GCST006061	0.0073	0.7376	537409	outcome	TRUE	reported	8009_121_SURF1_SURF1	SURF1	chr1	169568440	0.008336	1.803e-63	35360	TRUE	reported	textfile	2	1	TRUE
1480	rs71352239	T	C	T	C	0.1252	-0.0041	0.30263	NA	FALSE	FALSE	FALSE	GCST006061	0.0087	0.6372	537409	outcome	TRUE	reported	8009_121_SURF1_SURF1	SURF1	chr19	44926286	0.009059	1.928e-43	35368	TRUE	reported	textfile	2	1	TRUE
1481	rs7894992	A	G	A	G	-0.0807	-0.0052	0.49304	NA	FALSE	FALSE	FALSE	GCST006061	0.0071	0.4628	537409	outcome	TRUE	reported	8009_121_SURF1_SURF1	SURF1	chr10	19795284	0.00826	1.519e-22	35358	TRUE	reported	textfile	2	1	TRUE
1482	rs10159712	A	G	A	G	0.1935	-0.0104	0.2838	NA	FALSE	FALSE	FALSE	GCST006061	0.0079	0.1855	537409	outcome	TRUE	reported	9039_47_TOR1AIP1_TOIP1	TOR1AIP1	chr10	19971440	0.009095	1.893e-100	35360	TRUE	reported	textfile	2	1	TRUE
1483	rs10437425	G	T	G	T	-0.0702	0.0072	0.27349	NA	FALSE	FALSE	FALSE	GCST006061	0.0078	0.3515	537409	outcome	TRUE	reported	9039_47_TOR1AIP1_TOIP1	TOR1AIP1	chr10	20078274	0.009173	1.959e-14	35363	TRUE	reported	textfile	2	1	TRUE
1484	rs11012153	A	C	A	C	-0.0943	0.017	0.0829	NA	FALSE	FALSE	FALSE	GCST006061	0.0165	0.3026	537409	outcome	TRUE	reported	9039_47_TOR1AIP1_TOIP1	TOR1AIP1	chr10	20565668	0.014451	6.771e-11	35369	TRUE	reported	textfile	2	1	TRUE
1485	rs1591734	T	C	T	C	-0.1798	0.0198	0.1303	NA	FALSE	FALSE	FALSE	GCST006061	0.0103	0.05506	537409	outcome	TRUE	reported	9039_47_TOR1AIP1_TOIP1	TOR1AIP1	chr1	169302664	0.012307	2.453e-48	35364	TRUE	reported	textfile	2	1	TRUE
1486	rs2187955	A	G	A	G	-0.1711	0.0045	0.44224	NA	FALSE	FALSE	FALSE	GCST006061	0.0072	0.5306	537409	outcome	TRUE	reported	9039_47_TOR1AIP1_TOIP1	TOR1AIP1	chr1	169567412	0.008267	3.79e-95	35360	TRUE	reported	textfile	2	1	TRUE
1487	rs35814008	C	A	C	A	0.07	-0.013	0.11989	NA	FALSE	FALSE	FALSE	GCST006061	0.0105	0.2137	537409	outcome	TRUE	reported	9039_47_TOR1AIP1_TOIP1	TOR1AIP1	chr7	73641131	0.012606	2.807e-08	35353	TRUE	reported	textfile	2	1	TRUE
1488	rs41272457	A	G	A	G	-0.1783	-0.0446	0.03922	NA	FALSE	FALSE	FALSE	GCST006061	0.0309	0.1487	537409	outcome	TRUE	reported	9039_47_TOR1AIP1_TOIP1	TOR1AIP1	chr1	169542165	0.021332	6.362e-17	35364	TRUE	reported	textfile	2	1	TRUE
1489	rs4654748	T	C	T	C	-0.0612	-0.0034	0.43409	NA	FALSE	FALSE	FALSE
```

GCST006061 0.0073 0.6384 537409 outcome TRUE reported 9039_47_TOR1AIP1_TOIP1
TOR1AIP1 chr1 21459575 0.008324 1.943e-13 35374 TRUE reported
textfile 2 1 TRUE
1490 rs594793 T G T G -0.0542 0.0085 0.43083 NA FALSE FALSE FALSE
GCST006061 0.0076 0.2613 537409 outcome TRUE reported 9039_47_TOR1AIP1_TOIP1
TOR1AIP1 chr19 7765060 0.008281 5.934e-11 35373 TRUE reported
textfile 2 1 TRUE
1491 rs61808983 T C T C -0.3882 -0.0103 0.07171 NA FALSE FALSE FALSE
GCST006061 0.0197 0.6008 537409 outcome TRUE reported 9039_47_TOR1AIP1_TOIP1
TOR1AIP1 chr1 169505159 0.015727 1.591e-134 35363 TRUE reported
textfile 2 1 TRUE
1492 rs6544941 A G A G -0.0723 0.0035 0.33068 NA FALSE FALSE FALSE
GCST006061 0.0077 0.6538 537409 outcome TRUE reported 9039_47_TOR1AIP1_TOIP1
TOR1AIP1 chr2 46947977 0.008685 8.475e-17 35358 TRUE reported
textfile 2 1 TRUE
1493 rs704 A G A G 0.1352 -0.0155 0.49189 NA FALSE FALSE FALSE GCST006061
0.0072 0.03144 537409 outcome TRUE reported 9039_47_TOR1AIP1_TOIP1 TOR1AIP1
chr17 28367840 0.008175 1.946e-61 35360 TRUE reported textfile 2
1 TRUE
1494 rs7894992 A G A G -0.1124 -0.0052 0.49304 NA FALSE FALSE FALSE
GCST006061 0.0071 0.4628 537409 outcome TRUE reported 9039_47_TOR1AIP1_TOIP1
TOR1AIP1 chr10 19795284 0.008138 2.182e-43 35358 TRUE reported
textfile 2 1 TRUE
1495 rs10159712 A G A G 0.1008 -0.0104 0.2838 NA FALSE FALSE FALSE
GCST006061 0.0079 0.1855 537409 outcome TRUE reported 12671_35_SULT1B1_ST1B1
SULT1B1 chr10 19971440 0.008905 1.056e-29 35351 TRUE reported
textfile 2 1 TRUE
1496 rs114585353 G A G A -0.1011 0.0542 0.05784 NA FALSE FALSE FALSE
GCST006061 0.0333 0.1033 537409 outcome TRUE reported 12671_35_SULT1B1_ST1B1
SULT1B1 chr1 170349687 0.017048 3.023e-09 35352 TRUE reported
textfile 2 1 TRUE
1497 rs12127364 A C A C -0.2738 -0.006 0.3095 NA FALSE FALSE FALSE
GCST006061 0.0081 0.4569 537409 outcome TRUE reported 12671_35_SULT1B1_ST1B1
SULT1B1 chr1 169499904 0.008635 1e-200 35354 TRUE reported textfile
2 1 TRUE
1498 rs1265067 A G A G 0.057 6e-04 0.24493 NA FALSE FALSE FALSE
GCST006061 0.008 0.942 537409 outcome TRUE reported 12671_35_SULT1B1_ST1B1
SULT1B1 chr6 31148365 0.009373 1.194e-09 35430 TRUE reported
textfile 2 1 TRUE
1499 rs150192271 A G A G 0.1686 0.0297 0.03763 NA FALSE FALSE FALSE
GCST006061 0.0249 0.2336 537409 outcome TRUE reported 12671_35_SULT1B1_ST1B1
SULT1B1 chr1 169662795 0.0218 1.044e-14 35353 TRUE reported textfile
2 1 TRUE
1500 rs6029 T C T C 0.2474 0.008 0.16105 NA FALSE FALSE FALSE GCST006061
0.009 0.3713 537409 outcome TRUE reported 12671_35_SULT1B1_ST1B1 SULT1B1
chr1 169560735 0.011025 1.606e-111 35351 TRUE reported textfile 2
1 TRUE
1501 rs78334957 C T C T 0.0764 -0.002 0.13736 NA FALSE FALSE FALSE
GCST006061 0.0104 0.8478 537409 outcome TRUE reported 12671_35_SULT1B1_ST1B1
SULT1B1 chr10 19819940 0.011658 5.62e-11 35349 TRUE reported
textfile 2 1 TRUE
1502 rs10159712 A G A G 0.1283 -0.0104 0.2838 NA FALSE FALSE FALSE
GCST006061 0.0079 0.1855 537409 outcome TRUE reported 17750_8_RRM2_RIR2
RRM2 chr10 19971440 0.009014 5.678e-46 35360 TRUE reported
textfile 2 1 TRUE
1503 rs10764161 T C T C -0.0708 -0.0053 0.4988 NA FALSE FALSE FALSE
GCST006061 0.0071 0.4565 537409 outcome TRUE reported 17750_8_RRM2_RIR2
RRM2 chr10 19797425 0.008053 1.469e-18 35358 TRUE reported
textfile 2 1 TRUE
1504 rs10922098 T C T C -0.0936 -0.009 0.40011 NA FALSE FALSE FALSE
GCST006061 0.0075 0.2275 537409 outcome TRUE reported 17750_8_RRM2_RIR2
RRM2 chr1 196695521 0.008296 1.595e-29 35338 TRUE reported
textfile 2 1 TRUE
1505 rs3917751 A G A G -0.1058 5e-04 0.34669 NA FALSE FALSE FALSE
GCST006061 0.0077 0.9467 537409 outcome TRUE reported 17750_8_RRM2_RIR2
RRM2 chr1 169607330 0.008538 2.877e-35 35347 TRUE reported

textfile 2 1 TRUE
1506 rs72702154 C T C T -0.2182 -0.0366 0.022 NA FALSE FALSE FALSE
GCST006061 0.0366 0.3177 537409 outcome TRUE reported 17750_8_RRM2_RIR2
RRM2 chr1 169360691 0.02677 3.61e-16 35364 TRUE reported textfile
2 1 TRUE
1507 rs7535409 G A G A -0.1452 -7.00E-04 0.33792 NA FALSE FALSE FALSE
GCST006061 0.0075 0.9311 537409 outcome TRUE reported 17750_8_RRM2_RIR2
RRM2 chr1 169537003 0.008546 9.751e-65 35364 TRUE reported
textfile 2 1 TRUE
1508 rs10159712 A G A G 0.1018 -0.0104 0.2838 NA FALSE FALSE FALSE
GCST006061 0.0079 0.1855 537409 outcome TRUE reported 11430_49_DTX1_DTX1
DTX1 chr10 19971440 0.009014 1.422e-29 35351 TRUE reported
textfile 2 1 TRUE
1509 rs10922098 T C T C -0.1555 -0.009 0.40011 NA FALSE FALSE FALSE
GCST006061 0.0075 0.2275 537409 outcome TRUE reported 11430_49_DTX1_DTX1
DTX1 chr1 196695521 0.008275 8.726e-79 35329 TRUE reported
textfile 2 1 TRUE
1510 rs11569415 A G A G -0.0559 0.0011 0.24276 NA FALSE FALSE FALSE
GCST006061 0.0097 0.9129 537409 outcome TRUE reported 11430_49_DTX1_DTX1
DTX1 chr19 6716268 0.009414 2.886e-09 35363 TRUE reported textfile
2 1 TRUE
1511 rs1591734 T C T C -0.0877 0.0198 0.1303 NA FALSE FALSE FALSE
GCST006061 0.0103 0.05506 537409 outcome TRUE reported 11430_49_DTX1_DTX1
DTX1 chr1 169302664 0.012167 5.678e-13 35355 TRUE reported
textfile 2 1 TRUE
1512 rs2239853 C T C T -0.0943 0.0039 0.44202 NA FALSE FALSE FALSE
GCST006061 0.0073 0.5928 537409 outcome TRUE reported 11430_49_DTX1_DTX1
DTX1 chr1 169556528 0.008205 1.421e-30 35344 TRUE reported
textfile 2 1 TRUE
1513 rs2686395 C T C T -0.0752 -0.0077 0.3101 NA FALSE FALSE FALSE
GCST006061 0.0074 0.2965 537409 outcome TRUE reported 11430_49_DTX1_DTX1
DTX1 chr3 165767231 0.008741 7.79e-18 35330 TRUE reported
textfile 2 1 TRUE
1514 rs6544941 A G A G -0.0573 0.0035 0.33068 NA FALSE FALSE FALSE
GCST006061 0.0077 0.6538 537409 outcome TRUE reported 11430_49_DTX1_DTX1
DTX1 chr2 46947977 0.008607 2.786e-11 35349 TRUE reported
textfile 2 1 TRUE
1515 rs75519859 C T C T 0.0941 -0.0144 0.1016 NA FALSE FALSE FALSE
GCST006061 0.0141 0.3078 537409 outcome TRUE reported 11430_49_DTX1_DTX1
DTX1 chr10 19598843 0.013525 3.467e-12 35348 TRUE reported
textfile 2 1 TRUE
1516 rs9332701 G A G A -0.2329 -0.0115 0.07189 NA FALSE FALSE FALSE
GCST006061 0.0196 0.5575 537409 outcome TRUE reported 11430_49_DTX1_DTX1
DTX1 chr1 169515529 0.015579 1.576e-50 35355 TRUE reported
textfile 2 1 TRUE
1517 rs10159712 A G A G 0.2175 -0.0104 0.2838 NA FALSE FALSE FALSE
GCST006061 0.0079 0.1855 537409 outcome TRUE reported 7959_34_CDH7_CADH7
CDH7 chr10 19971440 0.009121 1.111e-125 35360 TRUE reported
textfile 2 1 TRUE
1518 rs10437425 G T G T -0.0773 0.0072 0.27349 NA FALSE FALSE FALSE
GCST006061 0.0078 0.3515 537409 outcome TRUE reported 7959_34_CDH7_CADH7
CDH7 chr10 20078274 0.009219 5.066e-17 35363 TRUE reported
textfile 2 1 TRUE
1519 rs11012153 A C A C -0.1032 0.017 0.0829 NA FALSE FALSE FALSE
GCST006061 0.0165 0.3026 537409 outcome TRUE reported 7959_34_CDH7_CADH7
CDH7 chr10 20565668 0.014513 1.155e-12 35369 TRUE reported
textfile 2 1 TRUE
1520 rs12132412 G A G A -0.0596 0.0055 0.34943 NA FALSE FALSE FALSE
GCST006061 0.0077 0.4794 537409 outcome TRUE reported 7959_34_CDH7_CADH7
CDH7 chr1 21493549 0.008703 7.497e-12 35374 TRUE reported
textfile 2 1 TRUE
1521 rs149062332 C T C T 0.1672 0.0424 0.02045 NA FALSE FALSE FALSE
GCST006061 0.0435 0.33 537409 outcome TRUE reported 7959_34_CDH7_CADH7
CDH7 chr3 186623236 0.028216 3.109e-09 35367 TRUE reported
textfile 2 1 TRUE
1522 rs1569631 T G T G 0.0752 0.0158 0.12741 NA FALSE FALSE FALSE

GCST006061 0.0106 0.1361 537409 outcome TRUE reported 7959_34_CDH7_CADH7
CDH7 chr1 169045460 0.01239 1.282e-09 35361 TRUE reported textfile
2 1 TRUE
1523 rs1591734 T C T C -0.1977 0.0198 0.1303 NA FALSE FALSE FALSE
GCST006061 0.0103 0.05506 537409 outcome TRUE reported 7959_34_CDH7_CADH7
CDH7 chr1 169302664 0.012357 1.29e-57 35364 TRUE reported
textfile 2 1 TRUE
1524 rs2239853 C T C T -0.1932 0.0039 0.44202 NA FALSE FALSE FALSE
GCST006061 0.0073 0.5928 537409 outcome TRUE reported 7959_34_CDH7_CADH7
CDH7 chr1 169556528 0.008306 1.145e-119 35353 TRUE reported
textfile 2 1 TRUE
1525 rs41272457 A G A G -0.2036 -0.0446 0.03922 NA FALSE FALSE FALSE
GCST006061 0.0309 0.1487 537409 outcome TRUE reported 7959_34_CDH7_CADH7
CDH7 chr1 169542165 0.021421 2.004e-21 35364 TRUE reported
textfile 2 1 TRUE
1526 rs594793 T G T G -0.0558 0.0085 0.43083 NA FALSE FALSE FALSE
GCST006061 0.0076 0.2613 537409 outcome TRUE reported 7959_34_CDH7_CADH7
CDH7 chr19 7765060 0.008298 1.766e-11 35373 TRUE reported textfile
2 1 TRUE
1527 rs61808983 T C T C -0.3871 -0.0103 0.07171 NA FALSE FALSE FALSE
GCST006061 0.0197 0.6008 537409 outcome TRUE reported 7959_34_CDH7_CADH7
CDH7 chr1 169505159 0.015793 1.116e-132 35363 TRUE reported
textfile 2 1 TRUE
1528 rs62140549 G A G A 0.0823 -3.00E-04 0.30898 NA FALSE FALSE FALSE
GCST006061 0.008 0.9672 537409 outcome TRUE reported 7959_34_CDH7_CADH7
CDH7 chr2 46914826 0.008858 1.53e-20 35358 TRUE reported
textfile 2 1 TRUE
1529 rs7894992 A G A G -0.1177 -0.0052 0.49304 NA FALSE FALSE FALSE
GCST006061 0.0071 0.4628 537409 outcome TRUE reported 7959_34_CDH7_CADH7
CDH7 chr10 19795284 0.008169 4.64e-47 35358 TRUE reported
textfile 2 1 TRUE
1530 rs10159712 A G A G 0.1441 -0.0104 0.2838 NA FALSE FALSE FALSE
GCST006061 0.0079 0.1855 537409 outcome TRUE reported 13236_25_WNT3A_WNT3A
WNT3A chr10 19971440 0.008973 4.855e-58 35351 TRUE reported
textfile 2 1 TRUE
1531 rs10437425 G T G T -0.0605 0.0072 0.27349 NA FALSE FALSE FALSE
GCST006061 0.0078 0.3515 537409 outcome TRUE reported 13236_25_WNT3A_WNT3A
WNT3A chr10 20078274 0.009034 2.133e-11 35354 TRUE reported
textfile 2 1 TRUE
1532 rs10764161 T C T C -0.0787 -0.0053 0.4988 NA FALSE FALSE FALSE
GCST006061 0.0071 0.4565 537409 outcome TRUE reported 13236_25_WNT3A_WNT3A
WNT3A chr10 19797425 0.008014 9.161e-23 35349 TRUE reported
textfile 2 1 TRUE
1533 rs11012153 A C A C -0.0858 0.017 0.0829 NA FALSE FALSE FALSE
GCST006061 0.0165 0.3026 537409 outcome TRUE reported 13236_25_WNT3A_WNT3A
WNT3A chr10 20565668 0.014227 1.631e-09 35360 TRUE reported
textfile 2 1 TRUE
1534 rs12132412 G A G A -0.0686 0.0055 0.34943 NA FALSE FALSE FALSE
GCST006061 0.0077 0.4794 537409 outcome TRUE reported 13236_25_WNT3A_WNT3A
WNT3A chr1 21493549 0.008511 7.639e-16 35365 TRUE reported
textfile 2 1 TRUE
1535 rs1591734 T C T C -0.1243 0.0198 0.1303 NA FALSE FALSE FALSE
GCST006061 0.0103 0.05506 537409 outcome TRUE reported 13236_25_WNT3A_WNT3A
WNT3A chr1 169302664 0.012094 8.85e-25 35355 TRUE reported
textfile 2 1 TRUE
1536 rs2117168 T C T C -0.0599 5e-04 0.30732 NA FALSE FALSE FALSE
GCST006061 0.008 0.9469 537409 outcome TRUE reported 13236_25_WNT3A_WNT3A
WNT3A chr2 46928080 0.008736 7.032e-12 35349 TRUE reported
textfile 2 1 TRUE
1537 rs2187955 A G A G -0.1304 0.0045 0.44224 NA FALSE FALSE FALSE
GCST006061 0.0072 0.5306 537409 outcome TRUE reported 13236_25_WNT3A_WNT3A
WNT3A chr1 169567412 0.008133 7.576e-58 35351 TRUE reported
textfile 2 1 TRUE
1538 rs429358 C T C T -0.0601 0.005 0.17407 NA FALSE FALSE FALSE
GCST006061 0.0103 0.6319 537409 outcome TRUE reported 13236_25_WNT3A_WNT3A
WNT3A chr19 44908684 0.010755 2.297e-08 35359 TRUE reported

textfile 2 1 TRUE
1539 rs61808983 T C T C -0.2851 -0.0103 0.07171 NA FALSE FALSE FALSE
GCST006061 0.0197 0.6008 537409 outcome TRUE reported 13236_25_WNT3A_WNT3A
WNT3A chr1 169505159 0.015486 1.088e-75 35354 TRUE reported
textfile 2 1 TRUE
1540 rs10159712 A G A G 0.1849 -0.0104 0.2838 NA FALSE FALSE FALSE
GCST006061 0.0079 0.1855 537409 outcome TRUE reported 8069_85_CD3E_CD3E
CD3E chr10 19971440 0.009069 2.179e-92 35360 TRUE reported
textfile 2 1 TRUE
1541 rs10437425 G T G T -0.0663 0.0072 0.27349 NA FALSE FALSE FALSE
GCST006061 0.0078 0.3515 537409 outcome TRUE reported 8069_85_CD3E_CD3E
CD3E chr10 20078274 0.009144 4.149e-13 35363 TRUE reported
textfile 2 1 TRUE
1542 rs1051317 T G T G 0.0781 -0.001 0.29891 NA FALSE FALSE FALSE
GCST006061 0.0081 0.9035 537409 outcome TRUE reported 8069_85_CD3E_CD3E
CD3E chr2 46903009 0.008895 1.629e-18 35358 TRUE reported
textfile 2 1 TRUE
1543 rs10764161 T C T C -0.1002 -0.0053 0.4988 NA FALSE FALSE FALSE
GCST006061 0.0071 0.4565 537409 outcome TRUE reported 8069_85_CD3E_CD3E
CD3E chr10 19797425 0.008114 4.908e-35 35358 TRUE reported
textfile 2 1 TRUE
1544 rs11012153 A C A C -0.0816 0.017 0.0829 NA FALSE FALSE FALSE
GCST006061 0.0165 0.3026 537409 outcome TRUE reported 8069_85_CD3E_CD3E
CD3E chr10 20565668 0.014414 1.503e-08 35369 TRUE reported
textfile 2 1 TRUE
1545 rs1591734 T C T C -0.1328 0.0198 0.1303 NA FALSE FALSE FALSE
GCST006061 0.0103 0.05506 537409 outcome TRUE reported 8069_85_CD3E_CD3E
CD3E chr1 169302664 0.012281 2.982e-27 35364 TRUE reported
textfile 2 1 TRUE
1546 rs3934504 A G A G 0.0804 -0.0043 0.08663 NA FALSE FALSE FALSE
GCST006061 0.0132 0.7424 537409 outcome TRUE reported 8069_85_CD3E_CD3E
CD3E chr2 241867325 0.014621 3.82e-08 35102 TRUE reported
textfile 2 1 TRUE
1547 rs4654748 T C T C -0.0557 -0.0034 0.43409 NA FALSE FALSE FALSE
GCST006061 0.0073 0.6384 537409 outcome TRUE reported 8069_85_CD3E_CD3E
CD3E chr1 21459575 0.008306 2.002e-11 35374 TRUE reported
textfile 2 1 TRUE
1548 rs594793 T G T G -0.0493 0.0085 0.43083 NA FALSE FALSE FALSE
GCST006061 0.0076 0.2613 537409 outcome TRUE reported 8069_85_CD3E_CD3E
CD3E chr19 7765060 0.008261 2.403e-09 35373 TRUE reported textfile
2 1 TRUE
1549 rs60642321 G A G A -0.0665 -8.00E-04 0.16758 NA FALSE FALSE FALSE
GCST006061 0.0092 0.9343 537409 outcome TRUE reported 8069_85_CD3E_CD3E
CD3E chr1 196853238 0.010992 1.45e-09 35338 TRUE reported
textfile 2 1 TRUE
1550 rs646441 A G A G -0.0635 -0.005 0.19338 NA FALSE FALSE FALSE
GCST006061 0.0101 0.6199 537409 outcome TRUE reported 8069_85_CD3E_CD3E
CD3E chr1 168693114 0.01046 1.272e-09 35362 TRUE reported textfile
2 1 TRUE
1551 rs80189144 C T C T 0.0757 -0.0118 0.1137 NA FALSE FALSE FALSE
GCST006061 0.0109 0.2785 537409 outcome TRUE reported 8069_85_CD3E_CD3E
CD3E chr7 73525609 0.012899 4.396e-09 35354 TRUE reported
textfile 2 1 TRUE
1552 rs9332701 G A G A -0.3777 -0.0115 0.07189 NA FALSE FALSE FALSE
GCST006061 0.0196 0.5575 537409 outcome TRUE reported 8069_85_CD3E_CD3E
CD3E chr1 169515529 0.015674 2.697e-128 35364 TRUE reported
textfile 2 1 TRUE
1553 rs10930409 A G A G 0.0906 -0.0026 0.06181 NA FALSE FALSE FALSE
GCST006061 0.017 0.8804 537409 outcome TRUE reported 16620_26_LY75_LY75
LY75 chr2 170243578 0.015658 7.205e-09 35362 TRUE reported
textfile 2 1 TRUE
1554 rs115200150 G A G A -1.0508 -0.0353 0.01719 NA FALSE FALSE FALSE
GCST006061 0.0275 0.2005 537409 outcome TRUE reported 16620_26_LY75_LY75
LY75 chr2 159737015 0.018296 1e-200 35350 TRUE reported textfile
2 1 TRUE
1555 rs116417234 T C T C 0.0936 0.0162 0.03776 NA FALSE FALSE FALSE

GCST006061 0.0196 0.4096 537409 outcome TRUE reported 16620_26_LY75_LY75
LY75 chr11 126454138 0.016447 1.263e-08 35369 TRUE reported
textfile 2 1 TRUE
1556 rs117633773 A G A G 0.0865 0.0067 0.04197 NA FALSE FALSE FALSE
GCST006061 0.0224 0.7662 537409 outcome TRUE reported 16620_26_LY75_LY75
LY75 chr17 6953365 0.015619 3.058e-08 35374 TRUE reported textfile
2 1 TRUE
1557 rs139721273 C T C T -0.5044 0.0592 0.01117 NA FALSE FALSE FALSE
GCST006061 0.0465 0.2027 537409 outcome TRUE reported 16620_26_LY75_LY75
LY75 chr2 159380107 0.03789 1.962e-40 35351 TRUE reported textfile
2 1 TRUE
1558 rs142029184 A G A G 0.1607 -0.0726 0.01311 NA FALSE FALSE FALSE
GCST006061 0.0428 0.09019 537409 outcome TRUE reported 16620_26_LY75_LY75
LY75 chr11 126401713 0.027856 7.977e-09 35374 TRUE reported
textfile 2 1 TRUE
1559 rs143501783 C T C T 0.2091 -0.0333 0.01116 NA FALSE FALSE FALSE
GCST006061 0.0443 0.4525 537409 outcome TRUE reported 16620_26_LY75_LY75
LY75 chr17 7897001 0.02882 4.008e-13 35373 TRUE reported textfile 2
1 TRUE
1560 rs150685660 C T C T -0.3507 0.0274 0.0136 NA FALSE FALSE FALSE
GCST006061 0.0362 0.4487 537409 outcome TRUE reported 16620_26_LY75_LY75
LY75 chr2 158192547 0.031679 1.744e-28 35342 TRUE reported
textfile 2 1 TRUE
1561 rs17705404 G A G A 0.1824 -0.0174 0.18487 NA FALSE FALSE FALSE
GCST006061 0.01 0.08109 537409 outcome TRUE reported 16620_26_LY75_LY75
LY75 chr2 161111891 0.009648 1.026e-79 35343 TRUE reported
textfile 2 1 TRUE
1562 rs1838381 C T C T 0.1036 -0.0086 0.07024 NA FALSE FALSE FALSE
GCST006061 0.0114 0.4527 537409 outcome TRUE reported 16620_26_LY75_LY75
LY75 chr2 145297281 0.01464 1.477e-12 35354 TRUE reported textfile
2 1 TRUE
1563 rs2298475 C T C T -0.0859 0.0304 0.0807 NA FALSE FALSE FALSE
GCST006061 0.0125 0.01516 537409 outcome TRUE reported 16620_26_LY75_LY75
LY75 chr11 126408308 0.011642 1.599e-13 35371 TRUE reported
textfile 2 1 TRUE
1564 rs2890714 A G A G 0.0569 0.012 0.1987 NA FALSE FALSE FALSE
GCST006061 0.0089 0.1811 537409 outcome TRUE reported 16620_26_LY75_LY75
LY75 chr2 143370043 0.009384 1.333e-09 35365 TRUE reported
textfile 2 1 TRUE
1565 rs2909447 T C T C 0.3573 -0.0162 0.01021 NA FALSE FALSE FALSE
GCST006061 0.0342 0.6359 537409 outcome TRUE reported 16620_26_LY75_LY75
LY75 chr2 162007965 0.036093 4.191e-23 35342 TRUE reported
textfile 2 1 TRUE
1566 rs62061425 G A G A 0.0763 0.001 0.21627 NA FALSE FALSE FALSE
GCST006061 0.0097 0.9151 537409 outcome TRUE reported 16620_26_LY75_LY75
LY75 chr17 7169772 0.007738 6.198e-23 35370 TRUE reported textfile
2 1 TRUE
1567 rs62176383 T C T C 0.0936 -0.0203 0.05549 NA FALSE FALSE FALSE
GCST006061 0.0163 0.2137 537409 outcome TRUE reported 16620_26_LY75_LY75
LY75 chr2 168293991 0.016165 7.026e-09 35352 TRUE reported
textfile 2 1 TRUE
1568 rs62194171 T G T G 0.1276 -0.0129 0.381 NA FALSE FALSE FALSE
GCST006061 0.0073 0.07883 537409 outcome TRUE reported 16620_26_LY75_LY75
LY75 chr2 161012157 0.0077 1.124e-61 35345 TRUE reported textfile
2 1 TRUE
1569 rs635634 C T C T 0.0696 0.0285 0.12995 NA FALSE FALSE FALSE
GCST006061 0.0089 0.001351 537409 outcome TRUE reported 16620_26_LY75_LY75
LY75 chr9 133279427 0.009415 1.445e-13 35371 TRUE reported
textfile 2 1 TRUE
1570 rs6730459 A G A G -0.0655 -0.0091 0.23927 NA FALSE FALSE FALSE
GCST006061 0.009 0.3113 537409 outcome TRUE reported 16620_26_LY75_LY75
LY75 chr2 170222127 0.008798 9.685e-14 35365 TRUE reported
textfile 2 1 TRUE
1571 rs681343 T C T C 0.0438 0.0054 0.3954 NA FALSE FALSE FALSE
GCST006061 0.0076 0.4742 537409 outcome TRUE reported 16620_26_LY75_LY75
LY75 chr19 48703205 0.006495 1.55e-11 35377 TRUE reported

textfile 2 1 TRUE
1572 rs7419274 T C T C 0.2322 -0.015 0.229 NA FALSE FALSE FALSE
GCST006061 0.0087 0.08654 537409 outcome TRUE reported 16620_26_LY75_LY75
LY75 chr2 160528780 0.00883 2.135e-152 35348 TRUE reported textfile
2 1 TRUE
1573 rs77080489 C A C A 0.1583 0.025 0.02073 NA FALSE FALSE FALSE
GCST006061 0.0345 0.4688 537409 outcome TRUE reported 16620_26_LY75_LY75
LY75 chr2 146536060 0.025632 6.583e-10 35348 TRUE reported
textfile 2 1 TRUE
1574 rs78110020 T C T C 0.0908 -0.016 0.05749 NA FALSE FALSE FALSE
GCST006061 0.0186 0.3915 537409 outcome TRUE reported 16620_26_LY75_LY75
LY75 chr17 6915030 0.013498 1.731e-11 35374 TRUE reported textfile
2 1 TRUE
1575 rs79443047 G T G T 0.0836 0.018 0.12333 NA FALSE FALSE FALSE
GCST006061 0.013 0.1651 537409 outcome TRUE reported 16620_26_LY75_LY75
LY75 chr2 171574611 0.011284 1.277e-13 35362 TRUE reported
textfile 2 1 TRUE
1576 rs843203 T C T C -0.0598 -0.0022 0.35611 NA FALSE FALSE FALSE
GCST006061 0.0075 0.765 537409 outcome TRUE reported 16620_26_LY75_LY75
LY75 chr2 155570301 0.00779 1.633e-14 35335 TRUE reported textfile
2 1 TRUE
1577 rs10165349 C T C T 0.0753 0.0223 0.4992 NA FALSE FALSE FALSE
GCST006061 0.0071 0.001788 537409 outcome TRUE reported 7258_5_PRG2_EMBP
PRG2 chr2 61373938 0.007913 1.794e-21 35353 TRUE reported
textfile 2 1 TRUE
1578 rs13256023 T C T C -0.0644 -0.0216 0.18528 NA FALSE FALSE FALSE
GCST006061 0.009 0.01682 537409 outcome TRUE reported 7258_5_PRG2_EMBP
PRG2 chr8 60735992 0.010179 2.503e-10 35360 TRUE reported
textfile 2 1 TRUE
1579 rs2493133 C T C T -0.062 -0.0093 0.4307 NA FALSE FALSE FALSE
GCST006061 0.0074 0.209 537409 outcome TRUE reported 7258_5_PRG2_EMBP
PRG2 chr1 230708527 0.008059 1.439e-14 35364 TRUE reported
textfile 2 1 TRUE
1580 rs2816618 A G A G 0.0484 0.0042 0.3085 NA FALSE FALSE FALSE
GCST006061 0.0081 0.6007 537409 outcome TRUE reported 7258_5_PRG2_EMBP
PRG2 chr14 105265490 0.008649 2.193e-08 35108 TRUE reported
textfile 2 1 TRUE
1581 rs4328821 G A G A -0.1077 0.0069 0.13521 NA FALSE FALSE FALSE
GCST006061 0.0107 0.5185 537409 outcome TRUE reported 7258_5_PRG2_EMBP
PRG2 chr3 128597592 0.011686 3.078e-20 35363 TRUE reported
textfile 2 1 TRUE
1582 rs10166836 T C T C -0.2114 -0.003 0.42444 NA FALSE FALSE FALSE
GCST006061 0.0072 0.6769 537409 outcome TRUE reported 2890_59_CCL28_CCL28
CCL28 chr2 206174592 0.008224 1.001e-145 35360 TRUE reported
textfile 2 1 TRUE
1583 rs10861891 A C A C -0.0625 0.0069 0.30578 NA FALSE FALSE FALSE
GCST006061 0.0078 0.3768 537409 outcome TRUE reported 2890_59_CCL28_CCL28
CCL28 chr12 108316546 0.008861 1.747e-12 35376 TRUE reported
textfile 2 1 TRUE
1584 rs10886430 G A G A -0.0918 0.0071 0.12424 NA FALSE FALSE FALSE
GCST006061 0.0118 0.5496 537409 outcome TRUE reported 2890_59_CCL28_CCL28
CCL28 chr10 119250744 0.012297 8.303e-14 35379 TRUE reported
textfile 2 1 TRUE
1585 rs10980560 A G A G 0.0468 -0.0151 0.39798 NA FALSE FALSE FALSE
GCST006061 0.0073 0.03937 537409 outcome TRUE reported 2890_59_CCL28_CCL28
CCL28 chr9 110759913 0.008369 2.24e-08 35370 TRUE reported
textfile 2 1 TRUE
1586 rs11534419 T C T C 0.0478 0.0154 0.39288 NA FALSE FALSE FALSE
GCST006061 0.0081 0.05784 537409 outcome TRUE reported 2890_59_CCL28_CCL28
CCL28 chr9 133523101 0.008448 1.531e-08 35360 TRUE reported
textfile 2 1 TRUE
1587 rs11701223 T C T C -0.1179 0.0123 0.08332 NA FALSE FALSE FALSE
GCST006061 0.0119 0.3045 537409 outcome TRUE reported 2890_59_CCL28_CCL28
CCL28 chr21 35007203 0.014806 1.682e-15 35378 TRUE reported
textfile 2 1 TRUE
1588 rs12829502 A G A G -0.0817 0.0015 0.39286 NA FALSE FALSE FALSE

GCST006061 0.0075 0.8392 537409 outcome TRUE reported 2890_59_CCL28_CCL28
CCL28 chr12 8004381 0.008431 3.315e-22 35377 TRUE reported textfile
2 1 TRUE
1589 rs139708128 A G A G -0.1878 0.029 0.01748 NA FALSE FALSE FALSE
GCST006061 0.0403 0.4708 537409 outcome TRUE reported 2890_59_CCL28_CCL28
CCL28 chr1 150272679 0.03157 2.703e-09 35360 TRUE reported textfile
2 1 TRUE
1590 rs17537823 T C T C -0.0974 -0.0192 0.0606 NA FALSE FALSE FALSE
GCST006061 0.0203 0.3431 537409 outcome TRUE reported 2890_59_CCL28_CCL28
CCL28 chr2 208010631 0.017236 1.597e-08 35356 TRUE reported
textfile 2 1 TRUE
1591 rs17712391 T C T C -0.0743 -0.002 0.12828 NA FALSE FALSE FALSE
GCST006061 0.0111 0.8565 537409 outcome TRUE reported 2890_59_CCL28_CCL28
CCL28 chr2 24022789 0.012402 2.089e-09 35369 TRUE reported
textfile 2 1 TRUE
1592 rs274555 T C T C 0.0526 -0.0056 0.4251 NA FALSE FALSE FALSE
GCST006061 0.0075 0.4548 537409 outcome TRUE reported 2890_59_CCL28_CCL28
CCL28 chr5 132387259 0.008269 2.004e-10 35351 TRUE reported
textfile 2 1 TRUE
1593 rs3792386 A G A G -0.0474 0.0089 0.35198 NA FALSE FALSE FALSE
GCST006061 0.0073 0.2182 537409 outcome TRUE reported 2890_59_CCL28_CCL28
CCL28 chr3 123134833 0.008586 3.374e-08 35367 TRUE reported
textfile 2 1 TRUE
1594 rs72956630 G A G A 0.1101 1e-04 0.06832 NA FALSE FALSE FALSE
GCST006061 0.0145 0.9947 537409 outcome TRUE reported 2890_59_CCL28_CCL28
CCL28 chr2 206572641 0.016015 6.214e-12 35360 TRUE reported
textfile 2 1 TRUE
1595 rs73000929 A G A G -0.2689 0.0131 0.04815 NA FALSE FALSE FALSE
GCST006061 0.0245 0.593 537409 outcome TRUE reported 2890_59_CCL28_CCL28
CCL28 chr11 114082900 0.018998 1.752e-45 35365 TRUE reported
textfile 2 1 TRUE
1596 rs7703662 G T G T -0.0551 -0.0104 0.47606 NA FALSE FALSE FALSE
GCST006061 0.0073 0.1547 537409 outcome TRUE reported 2890_59_CCL28_CCL28
CCL28 chr5 43420061 0.008212 1.955e-11 35361 TRUE reported
textfile 2 1 TRUE
1597 rs7758845 C A C A 0.0732 -0.0048 0.28301 NA FALSE FALSE FALSE
GCST006061 0.0078 0.5377 537409 outcome TRUE reported 2890_59_CCL28_CCL28
CCL28 chr6 135107399 0.009067 6.84e-16 35372 TRUE reported
textfile 2 1 TRUE
1598 rs10169065 G A G A 0.0603 0.0065 0.21645 NA FALSE FALSE FALSE
GCST006061 0.008 0.417 537409 outcome TRUE reported 10451_11_NUCB1_NUCB1
NUCB1 chr2 27555693 0.009829 8.506e-10 35361 TRUE reported
textfile 2 1 TRUE
1599 rs12462111 T C T C -0.0458 5e-04 0.48639 NA FALSE FALSE FALSE
GCST006061 0.0078 0.9499 537409 outcome TRUE reported 10451_11_NUCB1_NUCB1
NUCB1 chr19 48668049 0.008185 2.2e-08 35364 TRUE reported textfile
2 1 TRUE
1600 rs3826817 T C T C -0.145 0.0063 0.26776 NA FALSE FALSE FALSE
GCST006061 0.0083 0.4468 537409 outcome TRUE reported 10451_11_NUCB1_NUCB1
NUCB1 chr19 48898187 0.009147 1.356e-56 35367 TRUE reported
textfile 2 1 TRUE
1601 rs56199187 T C T C 0.0763 -0.0173 0.13349 NA FALSE FALSE FALSE
GCST006061 0.0124 0.1625 537409 outcome TRUE reported 10451_11_NUCB1_NUCB1
NUCB1 chr1 161551141 0.012016 2.153e-10 35339 TRUE reported
textfile 2 1 TRUE
1602 rs646776 T C T C 0.0837 -0.0142 0.20804 NA FALSE FALSE FALSE
GCST006061 0.0087 0.1014 537409 outcome TRUE reported 10451_11_NUCB1_NUCB1
NUCB1 chr1 109275908 0.010075 9.741e-17 35332 TRUE reported
textfile 2 1 TRUE
1603 rs860532 A C A C 0.0542 9e-04 0.24032 NA FALSE FALSE FALSE
GCST006061 0.0092 0.9209 537409 outcome TRUE reported 10451_11_NUCB1_NUCB1
NUCB1 chr12 103664436 0.009549 1.377e-08 35357 TRUE reported
textfile 2 1 TRUE
1604 rs10171729 T C T C 0.3234 0.0021 0.17982 NA FALSE FALSE FALSE
GCST006061 0.0093 0.825 537409 outcome TRUE reported 11440_58_SOCS3_SOCS_3
SOCS3 chr2 241069640 0.01047 1e-200 35157 TRUE reported textfile 2

1 TRUE
1605 rs12134934 T C T C -0.1381 -0.0343 0.05379 NA FALSE FALSE FALSE
GCST006061 0.0173 0.04781 537409 outcome TRUE reported 11440_58_SOCS3_SOCS_3
SOCS3 chr1 186312268 0.017309 1.48e-15 35349 TRUE reported
textfile 2 1 TRUE
1606 rs183812412 T C T C 0.1936 3e-04 0.03985 NA FALSE FALSE FALSE
GCST006061 0.0333 0.9923 537409 outcome TRUE reported 11440_58_SOCS3_SOCS_3
SOCS3 chr2 241602441 0.020629 6.308e-21 35108 TRUE reported
textfile 2 1 TRUE
1607 rs2097976 T C T C 0.1187 0.0122 0.0766 NA FALSE FALSE FALSE
GCST006061 0.0111 0.2695 537409 outcome TRUE reported 11440_58_SOCS3_SOCS_3
SOCS3 chr2 241184292 0.015093 3.697e-15 35107 TRUE reported
textfile 2 1 TRUE
1608 rs4666000 C T C T 0.0523 0.007 0.22594 NA FALSE FALSE FALSE
GCST006061 0.0078 0.3727 537409 outcome TRUE reported 11440_58_SOCS3_SOCS_3
SOCS3 chr2 27616502 0.009565 4.558e-08 35364 TRUE reported
textfile 2 1 TRUE
1609 rs4675969 A G A G 0.0776 0.006 0.19783 NA FALSE FALSE FALSE
GCST006061 0.0093 0.5211 537409 outcome TRUE reported 11440_58_SOCS3_SOCS_3
SOCS3 chr2 241278212 0.01024 3.499e-14 35114 TRUE reported textfile
2 1 TRUE
1610 rs7609518 T C T C -0.0795 0.001 0.22499 NA FALSE FALSE FALSE
GCST006061 0.0087 0.9114 537409 outcome TRUE reported 11440_58_SOCS3_SOCS_3
SOCS3 chr2 240357619 0.009746 3.433e-16 35263 TRUE reported
textfile 2 1 TRUE
1611 rs77418648 A G A G 0.0928 -0.0312 0.10556 NA FALSE FALSE FALSE
GCST006061 0.0119 0.008655 537409 outcome TRUE reported
11440_58_SOCS3_SOCS_3 SOCS3 chr2 240462987 0.013159 1.76e-12 35265
TRUE reported textfile 2 1 TRUE
1612 rs10171839 A G A G -0.2706 0.0032 0.49934 NA FALSE FALSE FALSE
GCST006061 0.0072 0.6591 537409 outcome TRUE reported 5716_49_CSAG1_CSAG1
CSAG1 chr2 218186591 0.007797 1e-200 35368 TRUE reported textfile
2 1 TRUE
1613 rs10922098 T C T C -0.0446 -0.009 0.40011 NA FALSE FALSE FALSE
GCST006061 0.0075 0.2275 537409 outcome TRUE reported 5716_49_CSAG1_CSAG1
CSAG1 chr1 196695521 0.008019 2.668e-08 35338 TRUE reported
textfile 2 1 TRUE
1614 rs139492403 T G T G 0.232 0.006 0.01052 NA FALSE FALSE FALSE
GCST006061 0.063 0.9238 537409 outcome TRUE reported 5716_49_CSAG1_CSAG1
CSAG1 chr2 217306872 0.039006 2.718e-09 35366 TRUE reported
textfile 2 1 TRUE
1615 rs146775047 G A G A 0.1264 0.0487 0.05263 NA FALSE FALSE FALSE
GCST006061 0.0245 0.04724 537409 outcome TRUE reported 5716_49_CSAG1_CSAG1
CSAG1 chr2 219196226 0.017957 1.935e-12 35367 TRUE reported
textfile 2 1 TRUE
1616 rs76705436 G T G T -0.1434 -0.0051 0.02306 NA FALSE FALSE FALSE
GCST006061 0.0374 0.8913 537409 outcome TRUE reported 5716_49_CSAG1_CSAG1
CSAG1 chr2 211911087 0.025992 3.445e-08 35356 TRUE reported
textfile 2 1 TRUE
1617 rs77939743 G A G A 0.1809 -0.0149 0.05758 NA FALSE FALSE FALSE
GCST006061 0.026 0.5673 537409 outcome TRUE reported 5716_49_CSAG1_CSAG1
CSAG1 chr2 217997136 0.017094 3.581e-26 35361 TRUE reported
textfile 2 1 TRUE
1618 rs10172650 A G A G -0.279 0.0016 0.30223 NA FALSE FALSE FALSE
GCST006061 0.0077 0.8327 537409 outcome TRUE reported 18275_5_CRIP1_CRIP1
CRIP1 chr2 20982585 0.009048 1e-200 35373 TRUE reported textfile
2 1 TRUE
1619 rs112709686 T G T G 0.1348 0.0353 0.03316 NA FALSE FALSE FALSE
GCST006061 0.0226 0.1195 537409 outcome TRUE reported 18275_5_CRIP1_CRIP1
CRIP1 chr2 23555070 0.023861 1.61e-08 35370 TRUE reported
textfile 2 1 TRUE
1620 rs112875651 A G A G -0.0654 -0.0016 0.40253 NA FALSE FALSE FALSE
GCST006061 0.0076 0.8301 537409 outcome TRUE reported 18275_5_CRIP1_CRIP1
CRIP1 chr8 125494452 0.008446 9.67e-15 35367 TRUE reported
textfile 2 1 TRUE
1621 rs115092560 A G A G -0.2385 -0.0403 0.01173 NA FALSE FALSE FALSE

GCST006061 0.0565 0.4756 537409 outcome TRUE reported 18275_5_CRIP1_CRIP1
CRIP1 chr2 21356272 0.038774 7.698e-10 35368 TRUE reported
textfile 2 1 TRUE
1622 rs11591147 T G T G -0.3144 0.0511 0.01208 NA FALSE FALSE FALSE
GCST006061 0.0356 0.1511 537409 outcome TRUE reported 18275_5_CRIP1_CRIP1
CRIP1 chr1 55039974 0.038947 6.89e-16 35363 TRUE reported
textfile 2 1 TRUE
1623 rs116609675 C T C T 0.2568 -0.0311 0.02575 NA FALSE FALSE FALSE
GCST006061 0.0255 0.2226 537409 outcome TRUE reported 18275_5_CRIP1_CRIP1
CRIP1 chr2 21256241 0.026607 4.834e-22 35373 TRUE reported
textfile 2 1 TRUE
1624 rs147711004 A G A G 0.0936 -0.0184 0.06423 NA FALSE FALSE FALSE
GCST006061 0.023 0.4248 537409 outcome TRUE reported 18275_5_CRIP1_CRIP1
CRIP1 chr19 44834661 0.01686 2.832e-08 35367 TRUE reported textfile
2 1 TRUE
1625 rs3764261 A C A C -0.0555 0.0132 0.32148 NA FALSE FALSE FALSE
GCST006061 0.0076 0.08361 537409 outcome TRUE reported 18275_5_CRIP1_CRIP1
CRIP1 chr16 56959412 0.008792 2.748e-10 35369 TRUE reported
textfile 2 1 TRUE
1626 rs639463 T C T C 0.0786 -0.0099 0.11502 NA FALSE FALSE FALSE
GCST006061 0.0147 0.4999 537409 outcome TRUE reported 18275_5_CRIP1_CRIP1
CRIP1 chr2 20516997 0.013146 2.247e-09 35369 TRUE reported
textfile 2 1 TRUE
1627 rs6511720 T G T G -0.1269 0.0068 0.08895 NA FALSE FALSE FALSE
GCST006061 0.0119 0.5645 537409 outcome TRUE reported 18275_5_CRIP1_CRIP1
CRIP1 chr19 11091630 0.014458 1.678e-18 35379 TRUE reported
textfile 2 1 TRUE
1628 rs660240 C T C T 0.0819 -0.0136 0.205 NA FALSE FALSE FALSE
GCST006061 0.0088 0.1214 537409 outcome TRUE reported 18275_5_CRIP1_CRIP1
CRIP1 chr1 109275216 0.010227 1.164e-15 35345 TRUE reported
textfile 2 1 TRUE
1629 rs6740545 A G A G -0.0537 0.0055 0.2547 NA FALSE FALSE FALSE
GCST006061 0.0084 0.514 537409 outcome TRUE reported 18275_5_CRIP1_CRIP1
CRIP1 chr2 43869000 0.009598 2.208e-08 35362 TRUE reported
textfile 2 1 TRUE
1630 rs684818 T C T C 0.0466 0.0038 0.44891 NA FALSE FALSE FALSE
GCST006061 0.0072 0.6 537409 outcome TRUE reported 18275_5_CRIP1_CRIP1 CRIP1
chr1 234719032 0.008287 1.877e-08 35355 TRUE reported textfile 2
1 TRUE
1631 rs7412 T C T C -0.2466 -0.0027 0.05941 NA FALSE FALSE FALSE GCST006061
0.0138 0.8459 537409 outcome TRUE reported 18275_5_CRIP1_CRIP1 CRIP1 chr19
44908822 0.017381 1.09e-45 35367 TRUE reported textfile 2 1 TRUE
1632 rs74869266 C A C A -0.0979 0.0124 0.07175 NA FALSE FALSE FALSE
GCST006061 0.0137 0.3655 537409 outcome TRUE reported 18275_5_CRIP1_CRIP1
CRIP1 chr8 20002686 0.015838 6.352e-10 35368 TRUE reported
textfile 2 1 TRUE
1633 rs754524 G T G T -0.2159 -0.004 0.29223 NA FALSE FALSE FALSE
GCST006061 0.0083 0.6297 537409 outcome TRUE reported 18275_5_CRIP1_CRIP1
CRIP1 chr2 21088669 0.009178 2.279e-122 35374 TRUE reported
textfile 2 1 TRUE
1634 rs10174098 A G A G -0.0502 0.0129 0.27925 NA FALSE FALSE FALSE
GCST006061 0.0076 0.09105 537409 outcome TRUE reported 11278_4_COL11A2_COL11A2
COL11A2 chr2 186628015 0.009085 3.281e-08 35354 TRUE reported
textfile 2 1 TRUE
1635 rs12975366 C T C T -0.0693 -0.0089 0.4155 NA FALSE FALSE FALSE
GCST006061 0.0081 0.2688 537409 outcome TRUE reported 11278_4_COL11A2_COL11A2
COL11A2 chr19 54255498 0.008283 5.91e-17 35361 TRUE reported
textfile 2 1 TRUE
1636 rs148658859 C A C A 0.1422 -0.078 0.03449 NA FALSE FALSE FALSE
GCST006061 0.0295 0.008151 537409 outcome TRUE reported
11278_4_COL11A2_COL11A2 COL11A2 chr6 36243339 0.022845 4.826e-10 35346
TRUE reported textfile 2 1 TRUE
1637 rs2450083 C T C T 0.0459 0.006 0.45529 NA FALSE FALSE FALSE
GCST006061 0.0072 0.404 537409 outcome TRUE reported 11278_4_COL11A2_COL11A2
COL11A2 chr8 119051303 0.008187 2.064e-08 35352 TRUE reported
textfile 2 1 TRUE

1638 rs271173 T C T C 0.0496 0.0165 0.45174 NA FALSE FALSE FALSE
GCST006061 0.0074 0.02567 537409 outcome TRUE reported 11278_4_COL11A2_COL11A2
COL11A2 chr6 132991083 0.008291 2.199e-09 35358 TRUE reported
textfile 2 1 TRUE
1639 rs61729512 A G A G 0.0703 3e-04 0.12561 NA FALSE FALSE FALSE
GCST006061 0.0121 0.9769 537409 outcome TRUE reported 11278_4_COL11A2_COL11A2
COL11A2 chr12 7485173 0.012405 1.452e-08 35379 TRUE reported textfile
2 1 TRUE
1640 rs8051661 G A G A 0.1043 -4.00E-04 0.10739 NA FALSE FALSE FALSE
GCST006061 0.0117 0.9726 537409 outcome TRUE reported 11278_4_COL11A2_COL11A2
COL11A2 chr16 88409532 0.013251 3.523e-15 35311 TRUE reported
textfile 2 1 TRUE
1641 rs9963868 C T C T -0.0585 -0.0035 0.34617 NA FALSE FALSE FALSE
GCST006061 0.0075 0.6451 537409 outcome TRUE reported 11278_4_COL11A2_COL11A2
COL11A2 chr18 48073063 0.008586 9.552e-12 35358 TRUE reported
textfile 2 1 TRUE
1642 rs10175354 T C T C 0.1568 -0.0016 0.06827 NA FALSE FALSE FALSE
GCST006061 0.0137 0.9061 537409 outcome TRUE reported
4430_44_COLEC11_Collectin_Kidney_1 COLEC11 chr2 2568138 0.017861 1.654e-18
35304 TRUE reported textfile 2 1 TRUE
1643 rs10183939 A G A G -0.6431 -0.004 0.1865 NA FALSE FALSE FALSE
GCST006061 0.0092 0.6622 537409 outcome TRUE reported
4430_44_COLEC11_Collectin_Kidney_1 COLEC11 chr2 3600300 0.007866 1e-200 35333
TRUE reported textfile 2 1 TRUE
1644 rs1031101 G A G A 0.2025 -0.0056 0.13742 NA FALSE FALSE FALSE
GCST006061 0.01 0.578 537409 outcome TRUE reported
4430_44_COLEC11_Collectin_Kidney_1 COLEC11 chr10 52773600 0.012338 1.54e-60
35361 TRUE reported textfile 2 1 TRUE
1645 rs10858300 T C T C -0.0947 0.0032 0.26445 NA FALSE FALSE FALSE
GCST006061 0.0079 0.688 537409 outcome TRUE reported
4430_44_COLEC11_Collectin_Kidney_1 COLEC11 chr9 134950654 0.009666 1.162e-22
35330 TRUE reported textfile 2 1 TRUE
1646 rs11123637 C T C T -0.2005 -0.0032 0.34378 NA FALSE FALSE FALSE
GCST006061 0.0077 0.6785 537409 outcome TRUE reported
4430_44_COLEC11_Collectin_Kidney_1 COLEC11 chr2 3606600 0.00948 2.799e-99 35333
TRUE reported textfile 2 1 TRUE
1647 rs111625037 A G A G 0.2295 -0.0141 0.03096 NA FALSE FALSE FALSE
GCST006061 0.0184 0.4443 537409 outcome TRUE reported
4430_44_COLEC11_Collectin_Kidney_1 COLEC11 chr2 3808453 0.02587 7.234e-19 35375
TRUE reported textfile 2 1 TRUE
1648 rs114986725 G A G A -0.1566 0.001 0.03175 NA FALSE FALSE FALSE
GCST006061 0.0351 0.9779 537409 outcome TRUE reported
4430_44_COLEC11_Collectin_Kidney_1 COLEC11 chr2 125750 0.025986 1.677e-09
35321 TRUE reported textfile 2 1 TRUE
1649 rs115785583 G T G T 0.3306 0.0107 0.02082 NA FALSE FALSE FALSE
GCST006061 0.0291 0.7138 537409 outcome TRUE reported
4430_44_COLEC11_Collectin_Kidney_1 COLEC11 chr2 2936036 0.032122 7.645e-25
35316 TRUE reported textfile 2 1 TRUE
1650 rs1486236 A C A C 0.0992 0.0029 0.34055 NA FALSE FALSE FALSE
GCST006061 0.0076 0.7053 537409 outcome TRUE reported
4430_44_COLEC11_Collectin_Kidney_1 COLEC11 chr2 179874723 0.009556 3.024e-25
35351 TRUE reported textfile 2 1 TRUE
1651 rs72682450 T G T G 0.5424 0.0371 0.0105 NA FALSE FALSE FALSE
GCST006061 0.052 0.4758 537409 outcome TRUE reported
4430_44_COLEC11_Collectin_Kidney_1 COLEC11 chr8 119129684 0.041995 3.669e-38
35360 TRUE reported textfile 2 1 TRUE
1652 rs72773101 A G A G -0.0921 0.0035 0.16929 NA FALSE FALSE FALSE
GCST006061 0.0095 0.7156 537409 outcome TRUE reported
4430_44_COLEC11_Collectin_Kidney_1 COLEC11 chr2 2671657 0.012084 2.51e-14
35307 TRUE reported textfile 2 1 TRUE
1653 rs73886131 A G A G -0.1415 -0.0017 0.06378 NA FALSE FALSE FALSE
GCST006061 0.0139 0.9031 537409 outcome TRUE reported
4430_44_COLEC11_Collectin_Kidney_1 COLEC11 chr3 187259995 0.017479 5.708e-16
35367 TRUE reported textfile 2 1 TRUE
1654 rs10176736 T G T G 0.0387 -0.0073 0.3074 NA FALSE FALSE FALSE
GCST006061 0.0079 0.3547 537409 outcome TRUE reported

8398_277_RARRES1_RAR_responsive_protein_TIG1 RARRES1 chr2 31242675 0.006812
1.337e-08 35369 TRUE reported textfile 2 1 TRUE
1655 rs10822155 A C A C 0.0349 -0.0472 0.43479 NA FALSE FALSE FALSE
GCST006061 0.0072 4.622e-11 537409 outcome TRUE reported
8398_277_RARRES1_RAR_responsive_protein_TIG1 RARRES1 chr10 63311455 0.006297
2.979e-08 35351 TRUE reported textfile 2 1 TRUE
1656 rs111756965 T C T C 0.1379 -0.0217 0.02564 NA FALSE FALSE FALSE
GCST006061 0.0273 0.4279 537409 outcome TRUE reported
8398_277_RARRES1_RAR_responsive_protein_TIG1 RARRES1 chr3 171477406 0.023578
4.955e-09 35362 TRUE reported textfile 2 1 TRUE
1657 rs114334418 C A C A 0.4657 -0.0173 0.06175 NA FALSE FALSE FALSE
GCST006061 0.0291 0.5509 537409 outcome TRUE reported
8398_277_RARRES1_RAR_responsive_protein_TIG1 RARRES1 chr3 155220614 0.01519
1e-200 35343 TRUE reported textfile 2 1 TRUE
1658 rs114694170 C T C T 0.0678 0.0053 0.08378 NA FALSE FALSE FALSE
GCST006061 0.0175 0.7624 537409 outcome TRUE reported
8398_277_RARRES1_RAR_responsive_protein_TIG1 RARRES1 chr5 88884379 0.011364
2.43e-09 35344 TRUE reported textfile 2 1 TRUE
1659 rs115106820 T C T C 0.2562 -0.0289 0.01524 NA FALSE FALSE FALSE
GCST006061 0.0574 0.6148 537409 outcome TRUE reported
8398_277_RARRES1_RAR_responsive_protein_TIG1 RARRES1 chr3 169322062 0.030463
4.089e-17 35354 TRUE reported textfile 2 1 TRUE
1660 rs12492447 G A G A 0.2823 -0.0319 0.02824 NA FALSE FALSE FALSE
GCST006061 0.0358 0.3723 537409 outcome TRUE reported
8398_277_RARRES1_RAR_responsive_protein_TIG1 RARRES1 chr3 167194810 0.022093
2.176e-37 35354 TRUE reported textfile 2 1 TRUE
1661 rs138017839 C T C T -0.1386 -0.0018 0.03169 NA FALSE FALSE FALSE
GCST006061 0.0274 0.9481 537409 outcome TRUE reported
8398_277_RARRES1_RAR_responsive_protein_TIG1 RARRES1 chr3 170007150 0.021167
5.836e-11 35361 TRUE reported textfile 2 1 TRUE
1662 rs139652760 A G A G 0.3996 -0.0586 0.01146 NA FALSE FALSE FALSE
GCST006061 0.0434 0.1773 537409 outcome TRUE reported
8398_277_RARRES1_RAR_responsive_protein_TIG1 RARRES1 chr3 167272123 0.035157
6.155e-30 35354 TRUE reported textfile 2 1 TRUE
1663 rs1434282 T C T C -0.0416 -0.0021 0.26524 NA FALSE FALSE FALSE
GCST006061 0.008 0.7916 537409 outcome TRUE reported
8398_277_RARRES1_RAR_responsive_protein_TIG1 RARRES1 chr1 199041592 0.007186
7.094e-09 35343 TRUE reported textfile 2 1 TRUE
1664 rs145138743 C T C T 0.1703 -0.0164 0.01999 NA FALSE FALSE FALSE
GCST006061 0.0404 0.6854 537409 outcome TRUE reported
8398_277_RARRES1_RAR_responsive_protein_TIG1 RARRES1 chr3 153627170 0.02674
1.906e-10 35353 TRUE reported textfile 2 1 TRUE
1665 rs146361965 T C T C 1.1454 0.0981 0.0109 NA FALSE FALSE FALSE
GCST006061 0.0361 0.006599 537409 outcome TRUE reported
8398_277_RARRES1_RAR_responsive_protein_TIG1 RARRES1 chr3 158355780 0.034538
1e-200 35339 TRUE reported textfile 2 1 TRUE
1666 rs1532207 C T C T 0.0561 0.0096 0.34674 NA FALSE FALSE FALSE
GCST006061 0.0075 0.2014 537409 outcome TRUE reported
8398_277_RARRES1_RAR_responsive_protein_TIG1 RARRES1 chr3 148658996 0.00773
3.94e-13 35356 TRUE reported textfile 2 1 TRUE
1667 rs1687481 T C T C 0.0594 -0.0026 0.32022 NA FALSE FALSE FALSE
GCST006061 0.0077 0.738 537409 outcome TRUE reported
8398_277_RARRES1_RAR_responsive_protein_TIG1 RARRES1 chr3 126517237 0.007929
6.827e-14 35371 TRUE reported textfile 2 1 TRUE
1668 rs2595260 C T C T -0.2021 0.009 0.26301 NA FALSE FALSE FALSE
GCST006061 0.0084 0.2826 537409 outcome TRUE reported
8398_277_RARRES1_RAR_responsive_protein_TIG1 RARRES1 chr3 159220149 0.00836
4.238e-129 35338 TRUE reported textfile 2 1 TRUE
1669 rs4759076 C T C T 0.0494 -0.0011 0.4756 NA FALSE FALSE FALSE
GCST006061 0.0073 0.8799 537409 outcome TRUE reported
8398_277_RARRES1_RAR_responsive_protein_TIG1 RARRES1 chr12 54336088 0.006272
3.383e-15 35372 TRUE reported textfile 2 1 TRUE
1670 rs62270393 A G A G 0.3272 0.0112 0.08517 NA FALSE FALSE FALSE
GCST006061 0.0134 0.4011 537409 outcome TRUE reported
8398_277_RARRES1_RAR_responsive_protein_TIG1 RARRES1 chr3 159810105 0.013262
2.173e-134 35339 TRUE reported textfile 2 1 TRUE

1671 rs62277159 G A G A 0.4714 -0.0159 0.09128 NA FALSE FALSE FALSE
GCST006061 0.0152 0.2962 537409 outcome TRUE reported
8398_277_RARRES1_RAR_responsive_protein_TIG1 RARRES1 chr3 156808293 0.012648
1e-200 35338 TRUE reported textfile 2 1 TRUE
1672 rs62278751 G A G A 0.3313 0.0225 0.13401 NA FALSE FALSE FALSE
GCST006061 0.0163 0.1683 537409 outcome TRUE reported
8398_277_RARRES1_RAR_responsive_protein_TIG1 RARRES1 chr3 157677061 0.010808
1e-200 35337 TRUE reported textfile 2 1 TRUE
1673 rs62278958 C T C T -0.4181 0.0154 0.01253 NA FALSE FALSE FALSE
GCST006061 0.0339 0.6485 537409 outcome TRUE reported
8398_277_RARRES1_RAR_responsive_protein_TIG1 RARRES1 chr3 157987629 0.03363
1.741e-35 35337 TRUE reported textfile 2 1 TRUE
1674 rs71314064 C T C T 0.5543 0.0221 0.02927 NA FALSE FALSE FALSE
GCST006061 0.0373 0.554 537409 outcome TRUE reported
8398_277_RARRES1_RAR_responsive_protein_TIG1 RARRES1 chr3 161780801 0.021655
1.671e-144 35339 TRUE reported textfile 2 1 TRUE
1675 rs80253876 A G A G 0.1745 -0.0121 0.01344 NA FALSE FALSE FALSE
GCST006061 0.027 0.6546 537409 outcome TRUE reported
8398_277_RARRES1_RAR_responsive_protein_TIG1 RARRES1 chr3 171017235 0.031927
4.614e-08 35366 TRUE reported textfile 2 1 TRUE
1676 rs9857086 C A C A 0.1029 -0.0138 0.04316 NA FALSE FALSE FALSE
GCST006061 0.019 0.4684 537409 outcome TRUE reported
8398_277_RARRES1_RAR_responsive_protein_TIG1 RARRES1 chr3 143700318 0.018135
1.393e-08 35365 TRUE reported textfile 2 1 TRUE
1677 rs10179340 G A G A -0.1107 -0.0049 0.13216 NA FALSE FALSE FALSE
GCST006061 0.0099 0.6182 537409 outcome TRUE reported 12457_10_ADI1_MTND
ADI1 chr2 3519553 0.011526 7.689e-22 35323 TRUE reported textfile
2 1 TRUE
1678 rs2297937 G T G T 0.0479 -0.0079 0.28957 NA FALSE FALSE FALSE
GCST006061 0.0076 0.303 537409 outcome TRUE reported 12457_10_ADI1_MTND
ADI1 chr10 97882636 0.008573 2.305e-08 35358 TRUE reported
textfile 2 1 TRUE
1679 rs704 A G A G -0.0752 -0.0155 0.49189 NA FALSE FALSE FALSE GCST006061
0.0072 0.03144 537409 outcome TRUE reported 12457_10_ADI1_MTND ADI1 chr17
28367840 0.007813 6.268e-22 35351 TRUE reported textfile 2 1 TRUE
1680 rs10179986 G A G A -0.0553 -0.0095 0.21482 NA FALSE FALSE FALSE
GCST006061 0.0079 0.2335 537409 outcome TRUE reported 15614_168_LILRA2_LIRA2
LILRA2 chr2 134106773 0.010093 4.274e-08 35391 TRUE reported
textfile 2 1 TRUE
1681 rs11574588 G A G A -0.3573 0.0074 0.05934 NA FALSE FALSE FALSE
GCST006061 0.0204 0.7173 537409 outcome TRUE reported 15614_168_LILRA2_LIRA2
LILRA2 chr19 54667590 0.017891 9.877e-89 35373 TRUE reported
textfile 2 1 TRUE
1682 rs12945886 G A G A 0.0627 0 0.24063 NA FALSE FALSE FALSE GCST006061
0.0094 0.9973 537409 outcome TRUE reported 15614_168_LILRA2_LIRA2 LILRA2
chr17 7169695 0.009676 9.164e-11 35370 TRUE reported textfile 2 1
TRUE
1683 rs2098953 C T C T -0.1808 0.0154 0.05751 NA FALSE FALSE FALSE
GCST006061 0.0165 0.3513 537409 outcome TRUE reported 15614_168_LILRA2_LIRA2
LILRA2 chr19 54634136 0.017847 4.034e-24 35377 TRUE reported
textfile 2 1 TRUE
1684 rs3184504 C T C T -0.0575 0.0074 0.37862 NA FALSE FALSE FALSE
GCST006061 0.0075 0.3235 537409 outcome TRUE reported 15614_168_LILRA2_LIRA2
LILRA2 chr12 111446804 0.0085 1.339e-11 35371 TRUE reported textfile
2 1 TRUE
1685 rs4233366 T C T C 0.0591 -0.0026 0.22917 NA FALSE FALSE FALSE
GCST006061 0.0079 0.7409 537409 outcome TRUE reported 15614_168_LILRA2_LIRA2
LILRA2 chr1 161189357 0.009811 1.705e-09 35364 TRUE reported
textfile 2 1 TRUE
1686 rs61997798 T C T C 0.104 -0.0032 0.05376 NA FALSE FALSE FALSE
GCST006061 0.0227 0.8884 537409 outcome TRUE reported 15614_168_LILRA2_LIRA2
LILRA2 chr14 106821535 0.018645 2.433e-08 34925 TRUE reported
textfile 2 1 TRUE
1687 rs74342641 A G A G -0.1899 -0.0562 0.01842 NA FALSE FALSE FALSE
GCST006061 0.031 0.06976 537409 outcome TRUE reported 15614_168_LILRA2_LIRA2
LILRA2 chr2 134217285 0.029848 1.988e-10 35391 TRUE reported

textfile 2 1 TRUE
1688 rs10183338 T G T G 0.1581 -0.0037 0.29264 NA FALSE FALSE FALSE
GCST006061 0.0077 0.6361 537409 outcome TRUE reported
2436_49_CXCL16_CXCL16__soluble CXCL16 chr2 110853239 0.009182 1.951e-66
35356 TRUE reported textfile 2 1 TRUE
1689 rs1250568 C T C T -0.0621 -0.0205 0.44577 NA FALSE FALSE FALSE
GCST006061 0.0072 0.004454 537409 outcome TRUE reported
2436_49_CXCL16_CXCL16__soluble CXCL16 chr10 79285523 0.008377 1.233e-13
35368 TRUE reported textfile 2 1 TRUE
1690 rs273507 C A C A 0.0554 -0.0181 0.28087 NA FALSE FALSE FALSE
GCST006061 0.0081 0.02459 537409 outcome TRUE reported
2436_49_CXCL16_CXCL16__soluble CXCL16 chr19 18111154 0.00925 2.107e-09 35353
TRUE reported textfile 2 1 TRUE
1691 rs35383686 A G A G -0.2 0.0219 0.23142 NA FALSE FALSE FALSE
GCST006061 0.009 0.0151 537409 outcome TRUE reported
2436_49_CXCL16_CXCL16__soluble CXCL16 chr17 4720161 0.009838 7.088e-92 35374
TRUE reported textfile 2 1 TRUE
1692 rs3819306 G A G A 0.0593 0.0173 0.36675 NA FALSE FALSE FALSE
GCST006061 0.0079 0.02892 537409 outcome TRUE reported
2436_49_CXCL16_CXCL16__soluble CXCL16 chr6 31354356 0.008663 7.62e-12
35440 TRUE reported textfile 2 1 TRUE
1693 rs6031305 G A G A -0.0582 0.0153 0.342 NA FALSE FALSE FALSE
GCST006061 0.008 0.05671 537409 outcome TRUE reported
2436_49_CXCL16_CXCL16__soluble CXCL16 chr20 44029443 0.008771 3.239e-11
35369 TRUE reported textfile 2 1 TRUE
1694 rs653178 T C T C 0.1012 0.0076 0.38124 NA FALSE FALSE FALSE
GCST006061 0.0075 0.3129 537409 outcome TRUE reported
2436_49_CXCL16_CXCL16__soluble CXCL16 chr12 111569952 0.008591 4.974e-32
35371 TRUE reported textfile 2 1 TRUE
1695 rs748113 C T C T -0.0851 0.0079 0.4447 NA FALSE FALSE FALSE
GCST006061 0.0079 0.3159 537409 outcome TRUE reported
2436_49_CXCL16_CXCL16__soluble CXCL16 chr10 71749034 0.008367 2.688e-24
35374 TRUE reported textfile 2 1 TRUE
1696 rs75819996 C T C T 0.0739 0 0.33581 NA FALSE FALSE FALSE GCST006061
0.0079 0.9997 537409 outcome TRUE reported 2436_49_CXCL16_CXCL16__soluble
CXCL16 chr19 16452216 0.008823 5.476e-17 35366 TRUE reported
textfile 2 1 TRUE
1697 rs77542162 G A G A 0.3236 0.0177 0.025 NA FALSE FALSE FALSE
GCST006061 0.0302 0.557 537409 outcome TRUE reported
2436_49_CXCL16_CXCL16__soluble CXCL16 chr17 69085137 0.026494 2.609e-34
35371 TRUE reported textfile 2 1 TRUE
1698 rs9712275 T C T C -0.0519 0.0233 0.47062 NA FALSE FALSE FALSE
GCST006061 0.0072 0.001142 537409 outcome TRUE reported
2436_49_CXCL16_CXCL16__soluble CXCL16 chr2 198042419 0.008381 5.912e-10
35367 TRUE reported textfile 2 1 TRUE
1699 rs9869609 G A G A -0.095 0.0042 0.41754 NA FALSE FALSE FALSE
GCST006061 0.0077 0.581 537409 outcome TRUE reported
2436_49_CXCL16_CXCL16__soluble CXCL16 chr3 46102959 0.008472 3.487e-29
35344 TRUE reported textfile 2 1 TRUE
1700 rs995298 T C T C 0.0634 -0.0064 0.34711 NA FALSE FALSE FALSE
GCST006061 0.0076 0.393 537409 outcome TRUE reported
2436_49_CXCL16_CXCL16__soluble CXCL16 chr17 5746623 0.008817 6.458e-13 35378
TRUE reported textfile 2 1 TRUE
1701 rs11902299 T C T C -0.1945 0.0133 0.02692 NA FALSE FALSE FALSE
GCST006061 0.0382 0.7266 537409 outcome TRUE reported 8954_30_MAP4K1_M4K1
MAP4K1 chr2 53275315 0.024595 2.61e-15 35347 TRUE reported
textfile 2 1 TRUE
1702 rs1329424 G T G T -0.1082 -0.0095 0.39735 NA FALSE FALSE FALSE
GCST006061 0.0075 0.2071 537409 outcome TRUE reported 8954_30_MAP4K1_M4K1
MAP4K1 chr1 196677046 0.008016 1.608e-41 35338 TRUE reported
textfile 2 1 TRUE
1703 rs1354034 C T C T 0.0559 0.0088 0.38119 NA FALSE FALSE FALSE
GCST006061 0.0073 0.2248 537409 outcome TRUE reported 8954_30_MAP4K1_M4K1
MAP4K1 chr3 56815721 0.00808 4.563e-12 35356 TRUE reported textfile
2 1 TRUE
1704 rs1978974 T C T C 0.0585 0.0016 0.38245 NA FALSE FALSE FALSE

GCST006061 0.0073 0.827 537409 outcome TRUE reported 8954_30_MAP4K1_M4K1
MAP4K1 chr2 55102993 0.008126 6.077e-13 35345 TRUE reported
textfile 2 1 TRUE
1705 rs2576707 A G A G 0.092 0.0066 0.46277 NA FALSE FALSE FALSE
GCST006061 0.0073 0.3694 537409 outcome TRUE reported 8954_30_MAP4K1_M4K1
MAP4K1 chr2 55324795 0.00797 7.972e-31 35345 TRUE reported textfile
2 1 TRUE
1706 rs78496188 G A G A -0.2914 0.0017 0.23093 NA FALSE FALSE FALSE
GCST006061 0.0094 0.8588 537409 outcome TRUE reported 8954_30_MAP4K1_M4K1
MAP4K1 chr2 54558356 0.009269 1e-200 35346 TRUE reported textfile
2 1 TRUE
1707 rs10184004 T C T C 0.0574 0.0016 0.47095 NA FALSE FALSE FALSE
GCST006061 0.0074 0.8293 537409 outcome TRUE reported 8484_24_LEP_Leptin LEP
chr2 164651879 0.008519 1.604e-11 35351 TRUE reported textfile 2
1 TRUE
1708 rs12154627 C T C T 0.0566 -0.0166 0.44092 NA FALSE FALSE FALSE
GCST006061 0.0073 0.02352 537409 outcome TRUE reported 8484_24_LEP_Leptin LEP
chr7 130738173 0.008475 2.409e-11 35352 TRUE reported textfile 2
1 TRUE
1709 rs55872725 T C T C 0.072 -0.024 0.4144 NA FALSE FALSE FALSE
GCST006061 0.0073 0.00103 537409 outcome TRUE reported 8484_24_LEP_Leptin LEP
chr16 53775211 0.008544 3.541e-17 35367 TRUE reported textfile 2
1 TRUE
1710 rs10184004 T C T C 0.0464 0.0016 0.47095 NA FALSE FALSE FALSE
GCST006061 0.0074 0.8293 537409 outcome TRUE reported 6462_12_TIMP4_TIMP_4
TIMP4 chr2 164651879 0.008371 2.979e-08 35351 TRUE reported
textfile 2 1 TRUE
1711 rs11708734 A G A G -0.1543 -0.0157 0.0686 NA FALSE FALSE FALSE
GCST006061 0.0194 0.4171 537409 outcome TRUE reported 6462_12_TIMP4_TIMP_4
TIMP4 chr3 12901652 0.01658 1.322e-20 35369 TRUE reported textfile
2 1 TRUE
1712 rs151094541 A G A G -0.1565 -0.0112 0.03228 NA FALSE FALSE FALSE
GCST006061 0.0205 0.5846 537409 outcome TRUE reported 6462_12_TIMP4_TIMP_4
TIMP4 chr3 11914636 0.023424 2.372e-11 35367 TRUE reported
textfile 2 1 TRUE
1713 rs2600261 T C T C 0.3241 0.0026 0.21552 NA FALSE FALSE FALSE
GCST006061 0.0086 0.761 537409 outcome TRUE reported 6462_12_TIMP4_TIMP_4
TIMP4 chr3 11987705 0.010065 1e-200 35367 TRUE reported textfile
2 1 TRUE
1714 rs434460 G A G A -0.0713 -0.0215 0.49926 NA FALSE FALSE FALSE
GCST006061 0.0072 0.00298 537409 outcome TRUE reported 6462_12_TIMP4_TIMP_4
TIMP4 chr3 12875708 0.008393 1.973e-17 35367 TRUE reported
textfile 2 1 TRUE
1715 rs56120972 T C T C -0.1088 -0.0076 0.08767 NA FALSE FALSE FALSE
GCST006061 0.0167 0.6481 537409 outcome TRUE reported 6462_12_TIMP4_TIMP_4
TIMP4 chr3 13055556 0.014882 2.655e-13 35368 TRUE reported
textfile 2 1 TRUE
1716 rs5994626 T G T G -0.0582 0.0175 0.4672 NA FALSE FALSE FALSE
GCST006061 0.0072 0.0151 537409 outcome TRUE reported 6462_12_TIMP4_TIMP_4
TIMP4 chr22 32772766 0.00826 1.837e-12 35375 TRUE reported textfile
2 1 TRUE
1717 rs7133378 A G A G 0.0622 0.0415 0.31512 NA FALSE FALSE FALSE
GCST006061 0.0077 7.765e-08 537409 outcome TRUE reported
6462_12_TIMP4_TIMP_4 TIMP4 chr12 123924955 0.008938 3.428e-12 35369
TRUE reported textfile 2 1 TRUE
1718 rs10779706 T G T G 0.0767 0.0117 0.11358 NA FALSE FALSE FALSE
GCST006061 0.0117 0.3157 537409 outcome TRUE reported 7161_25_H6PD_G6PE
H6PD chr1 8926623 0.013415 1.082e-08 35385 TRUE reported textfile
2 1 TRUE
1719 rs10843403 C T C T 0.1997 -0.0063 0.36436 NA FALSE FALSE FALSE
GCST006061 0.0073 0.384 537409 outcome TRUE reported 7161_25_H6PD_G6PE
H6PD chr12 29387376 0.00845 1.761e-123 35359 TRUE reported textfile
2 1 TRUE
1720 rs11585670 C A C A 0.086 0.0091 0.16005 NA FALSE FALSE FALSE
GCST006061 0.0138 0.5117 537409 outcome TRUE reported 7161_25_H6PD_G6PE
H6PD chr1 10120850 0.011791 3.02e-13 35391 TRUE reported

textfile 2 1 TRUE
1721 rs146361217 G T G T 0.3001 -0.0059 0.01475 NA FALSE FALSE FALSE
GCST006061 0.042 0.8884 537409 outcome TRUE reported 7161_25_H6PD_G6PE
H6PD chr3 186306215 0.033598 4.18e-19 35358 TRUE reported
textfile 2 1 TRUE
1722 rs1790861 C T C T 0.0494 -0.007 0.30469 NA FALSE FALSE FALSE
GCST006061 0.0082 0.3899 537409 outcome TRUE reported 7161_25_H6PD_G6PE
H6PD chr18 74278063 0.008902 2.869e-08 35378 TRUE reported
textfile 2 1 TRUE
1723 rs2239944 G A G A -0.0485 -0.0013 0.35327 NA FALSE FALSE FALSE
GCST006061 0.0075 0.8655 537409 outcome TRUE reported 7161_25_H6PD_G6PE
H6PD chr19 35541399 0.008573 1.535e-08 35370 TRUE reported
textfile 2 1 TRUE
1724 rs35166538 A G A G 0.2106 0.0048 0.03732 NA FALSE FALSE FALSE
GCST006061 0.0221 0.8288 537409 outcome TRUE reported 7161_25_H6PD_G6PE
H6PD chr3 185145854 0.022247 2.896e-21 35361 TRUE reported
textfile 2 1 TRUE
1725 rs35209896 A C A C -0.1253 0.0267 0.09263 NA FALSE FALSE FALSE
GCST006061 0.0355 0.4521 537409 outcome TRUE reported 7161_25_H6PD_G6PE
H6PD chr6 32519009 0.014249 1.448e-18 35439 TRUE reported
textfile 2 1 TRUE
1726 rs56255430 C A C A 0.2046 -0.0034 0.0795 NA FALSE FALSE FALSE
GCST006061 0.0132 0.7997 537409 outcome TRUE reported 7161_25_H6PD_G6PE
H6PD chr19 19367068 0.015204 2.816e-41 35353 TRUE reported
textfile 2 1 TRUE
1727 rs57853394 A G A G -0.1792 0.0011 0.08831 NA FALSE FALSE FALSE
GCST006061 0.0188 0.9551 537409 outcome TRUE reported 7161_25_H6PD_G6PE
H6PD chr1 9838135 0.015105 1.832e-32 35390 TRUE reported textfile
2 1 TRUE
1728 rs62140549 G A G A 0.0632 -3.00E-04 0.30898 NA FALSE FALSE FALSE
GCST006061 0.008 0.9672 537409 outcome TRUE reported 7161_25_H6PD_G6PE
H6PD chr2 46914826 0.00884 8.711e-13 35358 TRUE reported textfile
2 1 TRUE
1729 rs62642596 T C T C 0.2092 -0.0015 0.48587 NA FALSE FALSE FALSE
GCST006061 0.0073 0.84 537409 outcome TRUE reported 7161_25_H6PD_G6PE
H6PD chr1 9260328 0.008611 2.275e-130 35385 TRUE reported textfile
2 1 TRUE
1730 rs72643830 T C T C -0.1182 -0.0144 0.20733 NA FALSE FALSE FALSE
GCST006061 0.0089 0.1065 537409 outcome TRUE reported 7161_25_H6PD_G6PE
H6PD chr1 9338560 0.010638 1.113e-28 35385 TRUE reported textfile
2 1 TRUE
1731 rs73178183 A G A G 0.3552 -0.0064 0.02042 NA FALSE FALSE FALSE
GCST006061 0.042 0.8783 537409 outcome TRUE reported 7161_25_H6PD_G6PE
H6PD chr3 186172547 0.030073 3.411e-32 35362 TRUE reported
textfile 2 1 TRUE
1732 rs74315900 T C T C 0.2764 -0.0628 0.0104 NA FALSE FALSE FALSE
GCST006061 0.0574 0.2739 537409 outcome TRUE reported 7161_25_H6PD_G6PE
H6PD chr3 187794783 0.04182 3.864e-11 35370 TRUE reported textfile
2 1 TRUE
1733 rs76624319 G A G A 0.4414 0.0626 0.01805 NA FALSE FALSE FALSE
GCST006061 0.0463 0.1762 537409 outcome TRUE reported 7161_25_H6PD_G6PE
H6PD chr3 186019045 0.031453 9.68e-45 35357 TRUE reported
textfile 2 1 TRUE
1734 rs77188638 A G A G 0.3376 -0.0279 0.03969 NA FALSE FALSE FALSE
GCST006061 0.0211 0.1861 537409 outcome TRUE reported 7161_25_H6PD_G6PE
H6PD chr3 186693415 0.021323 1.85e-56 35368 TRUE reported
textfile 2 1 TRUE
1735 rs8176693 T C T C 0.1007 0.0144 0.06484 NA FALSE FALSE FALSE
GCST006061 0.0129 0.2638 537409 outcome TRUE reported 7161_25_H6PD_G6PE
H6PD chr9 133262254 0.016651 1.469e-09 35371 TRUE reported
textfile 2 1 TRUE
1736 rs8178824 T C T C 0.3585 -0.0854 0.01066 NA FALSE FALSE FALSE
GCST006061 0.023 0.0002035 537409 outcome TRUE reported 7161_25_H6PD_G6PE
H6PD chr17 66228657 0.039449 1.012e-19 35369 TRUE reported
textfile 2 1 TRUE
1737 rs995628 C T C T -0.0764 0.0137 0.22791 NA FALSE FALSE FALSE

GCST006061 0.0087 0.1151 537409 outcome TRUE reported 7161_25_H6PD_G6PE
H6PD chr12 29086572 0.009722 3.888e-15 35358 TRUE reported
textfile 2 1 TRUE
1738 rs10186962 G A G A 0.0852 9e-04 0.3622 NA FALSE FALSE FALSE
GCST006061 0.0074 0.9053 537409 outcome TRUE reported
14079_14_IL18R1_IL_18_Ra IL18R1 chr2 101375445 0.008413 4.211e-24 35368
TRUE reported textfile 2 1 TRUE
1739 rs114432729 T C T C 0.1445 0.0396 0.02937 NA FALSE FALSE FALSE
GCST006061 0.0251 0.114 537409 outcome TRUE reported
14079_14_IL18R1_IL_18_Ra IL18R1 chr2 88423553 0.023869 1.414e-09 35354
TRUE reported textfile 2 1 TRUE
1740 rs115232861 C T C T 0.3708 0.0396 0.02795 NA FALSE FALSE FALSE
GCST006061 0.0222 0.07386 537409 outcome TRUE reported
14079_14_IL18R1_IL_18_Ra IL18R1 chr2 101625575 0.024618 2.877e-51 35368
TRUE reported textfile 2 1 TRUE
1741 rs117278560 C T C T 0.075 0.0145 0.10819 NA FALSE FALSE FALSE
GCST006061 0.0122 0.2348 537409 outcome TRUE reported
14079_14_IL18R1_IL_18_Ra IL18R1 chr16 71565350 0.012605 2.681e-09 35376
TRUE reported textfile 2 1 TRUE
1742 rs13209376 T G T G 0.0703 -0.0321 0.1057 NA FALSE FALSE FALSE
GCST006061 0.0131 0.01421 537409 outcome TRUE reported
14079_14_IL18R1_IL_18_Ra IL18R1 chr6 95622197 0.012591 2.36e-08 35343
TRUE reported textfile 2 1 TRUE
1743 rs139899816 C T C T -0.1399 -0.0023 0.02779 NA FALSE FALSE FALSE
GCST006061 0.0303 0.9382 537409 outcome TRUE reported
14079_14_IL18R1_IL_18_Ra IL18R1 chr2 86250225 0.024952 2.062e-08 35344
TRUE reported textfile 2 1 TRUE
1744 rs141702610 C T C T 0.1914 0.0179 0.04482 NA FALSE FALSE FALSE
GCST006061 0.0233 0.4413 537409 outcome TRUE reported
14079_14_IL18R1_IL_18_Ra IL18R1 chr2 104101554 0.019484 8.912e-23 35361
TRUE reported textfile 2 1 TRUE
1745 rs55715763 G A G A -0.2005 -0.0085 0.05679 NA FALSE FALSE FALSE
GCST006061 0.0207 0.6824 537409 outcome TRUE reported
14079_14_IL18R1_IL_18_Ra IL18R1 chr2 101829431 0.017378 8.531e-31 35368
TRUE reported textfile 2 1 TRUE
1746 rs61418148 C T C T 0.0539 -0.0032 0.2305 NA FALSE FALSE FALSE
GCST006061 0.0083 0.6951 537409 outcome TRUE reported
14079_14_IL18R1_IL_18_Ra IL18R1 chr14 103100683 0.009255 5.749e-09 35296
TRUE reported textfile 2 1 TRUE
1747 rs78060698 A G A G 0.1289 0.0138 0.0339 NA FALSE FALSE FALSE
GCST006061 0.0185 0.4566 537409 outcome TRUE reported
14079_14_IL18R1_IL_18_Ra IL18R1 chr19 5832762 0.021206 1.214e-09 35364
TRUE reported textfile 2 1 TRUE
1748 rs80339564 C T C T 0.2547 -0.0017 0.03095 NA FALSE FALSE FALSE
GCST006061 0.0317 0.9579 537409 outcome TRUE reported
14079_14_IL18R1_IL_18_Ra IL18R1 chr2 102971459 0.023564 3.121e-27 35363
TRUE reported textfile 2 1 TRUE
1749 rs8178824 T C T C 0.2872 -0.0854 0.01066 NA FALSE FALSE FALSE
GCST006061 0.023 0.0002035 537409 outcome TRUE reported
14079_14_IL18R1_IL_18_Ra IL18R1 chr17 66228657 0.03748 1.82e-14 35369
TRUE reported textfile 2 1 TRUE
1750 rs117414814 A C A C -0.1587 0.0336 0.03741 NA FALSE FALSE FALSE
GCST006061 0.0217 0.1226 537409 outcome TRUE reported
5671_1_CTRB1_Chymotrypsin CTRB1 chr16 74809009 0.021399 1.205e-13 35366
TRUE reported textfile 2 1 TRUE
1751 rs149935681 A G A G 0.8354 -0.0099 0.02248 NA FALSE FALSE FALSE
GCST006061 0.025 0.6919 537409 outcome TRUE reported
5671_1_CTRB1_Chymotrypsin CTRB1 chr16 75206872 0.02674 1e-200 35363 TRUE
reported textfile 2 1 TRUE
1752 rs507695 T C T C -0.0567 0.0013 0.2959 NA FALSE FALSE FALSE
GCST006061 0.0079 0.8663 537409 outcome TRUE reported
5671_1_CTRB1_Chymotrypsin CTRB1 chr11 100776205 0.008815 1.26e-10 35356
TRUE reported textfile 2 1 TRUE
1753 rs533406 G A G A -0.059 -0.0177 0.41964 NA FALSE FALSE FALSE
GCST006061 0.0075 0.01777 537409 outcome TRUE reported
5671_1_CTRB1_Chymotrypsin CTRB1 chr16 88908452 0.008352 1.615e-12 35321

TRUE reported textfile 2 1 TRUE
1754 rs635634 C T C T 0.0771 0.0285 0.12995 NA FALSE FALSE FALSE
GCST006061 0.0089 0.001351 537409 outcome TRUE reported
5671_1_CTRB1_Chymotrypsin CTRB1 chr9 133279427 0.012017 1.4e-10 35371
TRUE reported textfile 2 1 TRUE
1755 rs6564267 G T G T 0.0815 -0.0093 0.38405 NA FALSE FALSE FALSE
GCST006061 0.0076 0.2184 537409 outcome TRUE reported
5671_1_CTRB1_Chymotrypsin CTRB1 chr16 75570027 0.00844 4.612e-22 35363
TRUE reported textfile 2 1 TRUE
1756 rs6686515 G A G A 0.083 0.0132 0.12331 NA FALSE FALSE FALSE
GCST006061 0.0118 0.2625 537409 outcome TRUE reported
5671_1_CTRB1_Chymotrypsin CTRB1 chr1 200090580 0.012367 1.931e-11 35355
TRUE reported textfile 2 1 TRUE
1757 rs6835370 A G A G -0.075 0.005 0.15553 NA FALSE FALSE FALSE
GCST006061 0.0102 0.6225 537409 outcome TRUE reported
5671_1_CTRB1_Chymotrypsin CTRB1 chr4 185652020 0.011142 1.682e-11 35383
TRUE reported textfile 2 1 TRUE
1758 rs6977665 G A G A 0.0449 -0.0062 0.42011 NA FALSE FALSE FALSE
GCST006061 0.0072 0.3924 537409 outcome TRUE reported
5671_1_CTRB1_Chymotrypsin CTRB1 chr7 117336776 0.00822 4.705e-08 35343
TRUE reported textfile 2 1 TRUE
1759 rs9482772 C T C T 0.0674 0.0022 0.47592 NA FALSE FALSE FALSE
GCST006061 0.0071 0.7593 537409 outcome TRUE reported
5671_1_CTRB1_Chymotrypsin CTRB1 chr6 127125645 0.008095 8.351e-17 35355
TRUE reported textfile 2 1 TRUE
1760 rs9928744 T C T C 0.1758 -0.0012 0.23269 NA FALSE FALSE FALSE
GCST006061 0.0091 0.8993 537409 outcome TRUE reported
5671_1_CTRB1_Chymotrypsin CTRB1 chr16 75343852 0.009629 1.806e-74 35362
TRUE reported textfile 2 1 TRUE
1761 rs10971925 A G A G -0.4457 -0.0219 0.04518 NA FALSE FALSE FALSE
GCST006061 0.0148 0.1389 537409 outcome TRUE reported 18864_7_PRSS3_TRY3
PRSS3 chr9 34113969 0.019688 1.81e-113 35349 TRUE reported
textfile 2 1 TRUE
1762 rs11622435 A G A G 0.1115 0.0239 0.04594 NA FALSE FALSE FALSE
GCST006061 0.0191 0.2112 537409 outcome TRUE reported 18864_7_PRSS3_TRY3
PRSS3 chr14 81151652 0.018899 3.642e-09 35353 TRUE reported
textfile 2 1 TRUE
1763 rs117173455 A G A G 0.2847 0.0369 0.0165 NA FALSE FALSE FALSE
GCST006061 0.0337 0.2726 537409 outcome TRUE reported 18864_7_PRSS3_TRY3
PRSS3 chr9 34535002 0.032366 1.416e-18 35350 TRUE reported
textfile 2 1 TRUE
1764 rs11759956 T C T C -0.0657 0.0288 0.38681 NA FALSE FALSE FALSE
GCST006061 0.0076 0.000139 537409 outcome TRUE reported 18864_7_PRSS3_TRY3
PRSS3 chr6 7108864 0.008162 8.31e-16 35373 TRUE reported textfile
2 1 TRUE
1765 rs12237401 T C T C 0.0853 0.0088 0.27531 NA FALSE FALSE FALSE
GCST006061 0.0079 0.2689 537409 outcome TRUE reported 18864_7_PRSS3_TRY3
PRSS3 chr9 33367090 0.009096 6.708e-21 35348 TRUE reported
textfile 2 1 TRUE
1766 rs12604466 T G T G 0.0959 -0.0118 0.06737 NA FALSE FALSE FALSE
GCST006061 0.014 0.4016 537409 outcome TRUE reported 18864_7_PRSS3_TRY3
PRSS3 chr18 44801214 0.015758 1.159e-09 35349 TRUE reported
textfile 2 1 TRUE
1767 rs12666392 C T C T -0.0533 -0.0113 0.23672 NA FALSE FALSE FALSE
GCST006061 0.0081 0.1657 537409 outcome TRUE reported 18864_7_PRSS3_TRY3
PRSS3 chr7 159101009 0.009502 2.032e-08 34911 TRUE reported
textfile 2 1 TRUE
1768 rs138062707 T G T G -0.3104 -0.0146 0.03477 NA FALSE FALSE FALSE
GCST006061 0.0264 0.5817 537409 outcome TRUE reported 18864_7_PRSS3_TRY3
PRSS3 chr9 33594544 0.022312 5.363e-44 35346 TRUE reported
textfile 2 1 TRUE
1769 rs157931 T C T C 0.0466 -0.013 0.33753 NA FALSE FALSE FALSE
GCST006061 0.0078 0.0977 537409 outcome TRUE reported 18864_7_PRSS3_TRY3
PRSS3 chr7 130897965 0.00834 2.3e-08 35355 TRUE reported textfile 2
1 TRUE
1770 rs1816308 C T C T 0.0749 6e-04 0.21752 NA FALSE FALSE FALSE

GCST006061 0.0085 0.9416 537409 outcome TRUE reported 18864_7_PRSS3_TRY3
PRSS3 chr3 169343407 0.009566 4.886e-15 35354 TRUE reported
textfile 2 1 TRUE

1771 rs183429905 C T C T -0.258 -0.1232 0.0109 NA FALSE FALSE FALSE
GCST006061 0.0554 0.02606 537409 outcome TRUE reported 18864_7_PRSS3_TRY3
PRSS3 chr9 29878857 0.03842 1.877e-11 35357 TRUE reported textfile
2 1 TRUE

1772 rs2583503 T C T C -0.0675 -0.0085 0.22945 NA FALSE FALSE FALSE
GCST006061 0.0085 0.3223 537409 outcome TRUE reported 18864_7_PRSS3_TRY3
PRSS3 chr8 97272509 0.009375 6.009e-13 35360 TRUE reported
textfile 2 1 TRUE

1773 rs2742676 A G A G -0.0883 0.0148 0.2375 NA FALSE FALSE FALSE
GCST006061 0.0085 0.08099 537409 outcome TRUE reported 18864_7_PRSS3_TRY3
PRSS3 chr1 3086752 0.009361 4.01e-21 35332 TRUE reported textfile
2 1 TRUE

1774 rs2743283 A G A G -0.049 -0.0042 0.48981 NA FALSE FALSE FALSE
GCST006061 0.0073 0.5637 537409 outcome TRUE reported 18864_7_PRSS3_TRY3
PRSS3 chr20 45302456 0.007873 4.848e-10 35369 TRUE reported
textfile 2 1 TRUE

1775 rs281377 T C T C -0.0526 -0.0091 0.3353 NA FALSE FALSE FALSE
GCST006061 0.0075 0.2214 537409 outcome TRUE reported 18864_7_PRSS3_TRY3
PRSS3 chr19 48703346 0.008521 6.696e-10 35377 TRUE reported
textfile 2 1 TRUE

1776 rs3771138 T C T C 0.0516 -0.0162 0.30733 NA FALSE FALSE FALSE
GCST006061 0.008 0.04142 537409 outcome TRUE reported 18864_7_PRSS3_TRY3
PRSS3 chr2 10404514 0.008613 2.09e-09 35368 TRUE reported
textfile 2 1 TRUE

1777 rs7068821 T G T G -0.0604 0.0042 0.24956 NA FALSE FALSE FALSE
GCST006061 0.0081 0.6068 537409 outcome TRUE reported 18864_7_PRSS3_TRY3
PRSS3 chr10 88291278 0.009083 2.933e-11 35356 TRUE reported
textfile 2 1 TRUE

1778 rs72709664 A C A C -0.5303 0.0197 0.05641 NA FALSE FALSE FALSE
GCST006061 0.0133 0.1376 537409 outcome TRUE reported 18864_7_PRSS3_TRY3
PRSS3 chr9 33623179 0.017383 1e-200 35348 TRUE reported textfile
2 1 TRUE

1779 rs9411464 T C T C 0.142 -0.0045 0.06111 NA FALSE FALSE FALSE
GCST006061 0.0131 0.7324 537409 outcome TRUE reported 18864_7_PRSS3_TRY3
PRSS3 chr9 133233778 0.017092 9.739e-17 35365 TRUE reported
textfile 2 1 TRUE

1780 rs9482770 C T C T 0.045 0.002 0.47412 NA FALSE FALSE FALSE
GCST006061 0.0071 0.7769 537409 outcome TRUE reported 18864_7_PRSS3_TRY3
PRSS3 chr6 127121947 0.00792 1.335e-08 35355 TRUE reported textfile
2 1 TRUE

1781 rs9652674 A G A G 0.0864 -0.0041 0.28175 NA FALSE FALSE FALSE
GCST006061 0.0081 0.6112 537409 outcome TRUE reported 18864_7_PRSS3_TRY3
PRSS3 chr16 75221575 0.008767 6.488e-23 35362 TRUE reported
textfile 2 1 TRUE

1782 rs10194022 T C T C 0.0803 -4.00E-04 0.15912 NA FALSE FALSE FALSE
GCST006061 0.0113 0.9751 537409 outcome TRUE reported 13973_62_TTL_TTL TTL
chr2 112479304 0.011121 5.179e-13 35366 TRUE reported textfile 2
1 TRUE

1783 rs1354034 C T C T 0.0675 0.0088 0.38119 NA FALSE FALSE FALSE
GCST006061 0.0073 0.2248 537409 outcome TRUE reported 13973_62_TTL_TTL TTL
chr3 56815721 0.008309 4.514e-16 35356 TRUE reported textfile 2
1 TRUE

1784 rs4980320 C T C T 0.0537 0.0041 0.46986 NA FALSE FALSE FALSE
GCST006061 0.0076 0.5858 537409 outcome TRUE reported 13973_62_TTL_TTL TTL
chr11 198986 0.008089 3.159e-11 35352 TRUE reported textfile 2 1
TRUE

1785 rs10196335 G T G T 0.0522 0.0033 0.4845 NA FALSE FALSE FALSE
GCST006061 0.0072 0.6423 537409 outcome TRUE reported 9409_11_TPSAB1_TRYB1
TPSAB1 chr2 144571821 0.008072 1.003e-10 35357 TRUE reported
textfile 2 1 TRUE

1786 rs114730474 T G T G -0.1565 0.0446 0.0232 NA FALSE FALSE FALSE
GCST006061 0.0314 0.1549 537409 outcome TRUE reported 9409_11_TPSAB1_TRYB1
TPSAB1 chr1 93482476 0.0267 4.592e-09 35353 TRUE reported textfile

2 1 TRUE
1787 rs115340020 A G A G -0.2863 0.0049 0.03502 NA FALSE FALSE FALSE
GCST006061 0.0282 0.8621 537409 outcome TRUE reported 9409_11_TPSAB1_TRYB1
TPSAB1 chr1 92300881 0.021516 2.12e-40 35352 TRUE reported
textfile 2 1 TRUE
1788 rs117492134 A G A G 0.145 0.0283 0.03467 NA FALSE FALSE FALSE
GCST006061 0.0237 0.232 537409 outcome TRUE reported 9409_11_TPSAB1_TRYB1
TPSAB1 chr16 1279752 0.024581 3.662e-09 35316 TRUE reported textfile
2 1 TRUE
1789 rs117712465 A G A G -0.4705 -0.0538 0.01287 NA FALSE FALSE FALSE
GCST006061 0.0427 0.2075 537409 outcome TRUE reported 9409_11_TPSAB1_TRYB1
TPSAB1 chr16 889679 0.040201 1.22e-31 35311 TRUE reported textfile
2 1 TRUE
1790 rs117717200 A G A G 0.3088 0.025 0.06116 NA FALSE FALSE FALSE
GCST006061 0.0218 0.2521 537409 outcome TRUE reported 9409_11_TPSAB1_TRYB1
TPSAB1 chr16 1140672 0.018502 1.541e-62 35310 TRUE reported textfile
2 1 TRUE
1791 rs118073880 T C T C -0.3228 -0.0733 0.01978 NA FALSE FALSE FALSE
GCST006061 0.0314 0.01964 537409 outcome TRUE reported 9409_11_TPSAB1_TRYB1
TPSAB1 chr16 1534248 0.032141 9.828e-24 35319 TRUE reported textfile
2 1 TRUE
1792 rs12695310 T G T G -0.0734 0.011 0.45472 NA FALSE FALSE FALSE
GCST006061 0.0074 0.1348 537409 outcome TRUE reported 9409_11_TPSAB1_TRYB1
TPSAB1 chr3 114855180 0.008139 1.91e-19 35366 TRUE reported
textfile 2 1 TRUE
1793 rs141196266 T C T C -0.4447 0.0104 0.03708 NA FALSE FALSE FALSE
GCST006061 0.0243 0.6687 537409 outcome TRUE reported 9409_11_TPSAB1_TRYB1
TPSAB1 chr16 1271903 0.023979 8.898e-77 35316 TRUE reported textfile
2 1 TRUE
1794 rs150298197 A G A G -0.3904 0.021 0.02134 NA FALSE FALSE FALSE
GCST006061 0.0457 0.6452 537409 outcome TRUE reported 9409_11_TPSAB1_TRYB1
TPSAB1 chr16 1257786 0.031291 1.004e-35 35316 TRUE reported textfile
2 1 TRUE
1795 rs16856110 G A G A -0.0749 -0.0325 0.27596 NA FALSE FALSE FALSE
GCST006061 0.0084 0.000113 537409 outcome TRUE reported
9409_11_TPSAB1_TRYB1 TPSAB1 chr1 205662639 0.009115 2.078e-16 35374
TRUE reported textfile 2 1 TRUE
1796 rs17758695 T C T C -0.1675 7e-04 0.03874 NA FALSE FALSE FALSE
GCST006061 0.0295 0.9814 537409 outcome TRUE reported 9409_11_TPSAB1_TRYB1
TPSAB1 chr18 63253621 0.021487 6.417e-15 35365 TRUE reported
textfile 2 1 TRUE
1797 rs184695141 T C T C -0.233 -0.0982 0.01889 NA FALSE FALSE FALSE
GCST006061 0.0591 0.0965 537409 outcome TRUE reported 9409_11_TPSAB1_TRYB1
TPSAB1 chr16 2080643 0.032739 1.103e-12 35319 TRUE reported textfile
2 1 TRUE
1798 rs188566928 T C T C -0.2637 0.0069 0.06179 NA FALSE FALSE FALSE
GCST006061 0.0264 0.7932 537409 outcome TRUE reported 9409_11_TPSAB1_TRYB1
TPSAB1 chr16 1143133 0.018635 1.852e-45 35309 TRUE reported textfile
2 1 TRUE
1799 rs2884425 T C T C -0.0518 0.0093 0.45982 NA FALSE FALSE FALSE
GCST006061 0.0073 0.1986 537409 outcome TRUE reported 9409_11_TPSAB1_TRYB1
TPSAB1 chr1 234558737 0.008113 1.715e-10 35361 TRUE reported
textfile 2 1 TRUE
1800 rs2961914 A C A C -0.0626 0.0116 0.17929 NA FALSE FALSE FALSE
GCST006061 0.0088 0.1898 537409 outcome TRUE reported 9409_11_TPSAB1_TRYB1
TPSAB1 chr5 160441951 0.010561 3.074e-09 35363 TRUE reported
textfile 2 1 TRUE
1801 rs35930348 T C T C 0.0606 -0.0098 0.34051 NA FALSE FALSE FALSE
GCST006061 0.0079 0.2179 537409 outcome TRUE reported 9409_11_TPSAB1_TRYB1
TPSAB1 chr19 51780063 0.008556 1.41e-12 35383 TRUE reported
textfile 2 1 TRUE
1802 rs56043070 A G A G 0.1732 0.0079 0.06676 NA FALSE FALSE FALSE
GCST006061 0.015 0.5968 537409 outcome TRUE reported 9409_11_TPSAB1_TRYB1
TPSAB1 chr1 247556467 0.016099 5.408e-27 35165 TRUE reported
textfile 2 1 TRUE
1803 rs56344893 A C A C 0.1113 -0.001 0.3699 NA FALSE FALSE FALSE

GCST006061 0.0079 0.9019 537409 outcome TRUE reported 9409_11_TPSAB1_TRYB1
TPSAB1 chr19 43778377 0.008369 2.336e-40 35367 TRUE reported
textfile 2 1 TRUE
1804 rs68082716 T C T C -0.091 -0.004 0.11022 NA FALSE FALSE FALSE
GCST006061 0.01 0.6905 537409 outcome TRUE reported 9409_11_TPSAB1_TRYB1
TPSAB1 chr4 159037663 0.01311 3.886e-12 35356 TRUE reported textfile
2 1 TRUE
1805 rs7127546 A G A G 0.0807 0.0013 0.41347 NA FALSE FALSE FALSE
GCST006061 0.0074 0.8616 537409 outcome TRUE reported 9409_11_TPSAB1_TRYB1
TPSAB1 chr11 61922259 0.008197 7.182e-23 35366 TRUE reported
textfile 2 1 TRUE
1806 rs716719 A C A C 0.0606 0.0013 0.27426 NA FALSE FALSE FALSE
GCST006061 0.0078 0.8714 537409 outcome TRUE reported 9409_11_TPSAB1_TRYB1
TPSAB1 chr7 50286121 0.009054 2.182e-11 35337 TRUE reported
textfile 2 1 TRUE
1807 rs72801130 T G T G 0.0583 -0.0033 0.29673 NA FALSE FALSE FALSE
GCST006061 0.0085 0.6953 537409 outcome TRUE reported 9409_11_TPSAB1_TRYB1
TPSAB1 chr16 85528774 0.009861 3.373e-09 35370 TRUE reported
textfile 2 1 TRUE
1808 rs73491534 C T C T -0.2079 0.0144 0.03437 NA FALSE FALSE FALSE
GCST006061 0.0292 0.6214 537409 outcome TRUE reported 9409_11_TPSAB1_TRYB1
TPSAB1 chr16 1064588 0.024476 1.994e-17 35315 TRUE reported textfile
2 1 TRUE
1809 rs78306371 C T C T -1.2315 0.0295 0.03282 NA FALSE FALSE FALSE
GCST006061 0.0264 0.2647 537409 outcome TRUE reported 9409_11_TPSAB1_TRYB1
TPSAB1 chr16 1213618 0.015486 1e-200 35316 TRUE reported textfile 2
1 TRUE
1810 rs78744187 T C T C 0.2459 0.017 0.09936 NA FALSE FALSE FALSE
GCST006061 0.0145 0.2421 537409 outcome TRUE reported 9409_11_TPSAB1_TRYB1
TPSAB1 chr19 33263642 0.013504 4.371e-74 35363 TRUE reported
textfile 2 1 TRUE
1811 rs10199432 T C T C -0.0754 0.0043 0.29524 NA FALSE FALSE FALSE
GCST006061 0.0079 0.5914 537409 outcome TRUE reported 18218_48_CNRIP1_CB032
CNRIP1 chr2 67822436 0.009044 7.633e-17 35359 TRUE reported
textfile 2 1 TRUE
1812 rs140590049 G A G A -0.4767 -0.0341 0.01092 NA FALSE FALSE FALSE
GCST006061 0.0332 0.3042 537409 outcome TRUE reported 18218_48_CNRIP1_CB032
CNRIP1 chr2 68349403 0.038898 1.58e-34 35362 TRUE reported
textfile 2 1 TRUE
1813 rs183550357 C T C T -0.2678 0.0114 0.01198 NA FALSE FALSE FALSE
GCST006061 0.0587 0.8454 537409 outcome TRUE reported 18218_48_CNRIP1_CB032
CNRIP1 chr2 69778584 0.037857 1.505e-12 35363 TRUE reported
textfile 2 1 TRUE
1814 rs184832464 T C T C -0.4231 -0.0446 0.03026 NA FALSE FALSE FALSE
GCST006061 0.0263 0.09014 537409 outcome TRUE reported 18218_48_CNRIP1_CB032
CNRIP1 chr2 68493846 0.023971 1.005e-69 35358 TRUE reported
textfile 2 1 TRUE
1815 rs4280488 T C T C -0.1571 0.017 0.03158 NA FALSE FALSE FALSE
GCST006061 0.0307 0.5784 537409 outcome TRUE reported 18218_48_CNRIP1_CB032
CNRIP1 chr2 68684772 0.023267 1.457e-11 35359 TRUE reported
textfile 2 1 TRUE
1816 rs6014993 G A G A 0.0449 0.0043 0.46061 NA FALSE FALSE FALSE
GCST006061 0.0072 0.5511 537409 outcome TRUE reported 18218_48_CNRIP1_CB032
CNRIP1 chr20 57416581 0.008106 3.046e-08 35389 TRUE reported
textfile 2 1 TRUE
1817 rs72830075 T C T C -0.1305 0.0355 0.03355 NA FALSE FALSE FALSE
GCST006061 0.0231 0.124 537409 outcome TRUE reported 18218_48_CNRIP1_CB032
CNRIP1 chr2 68093233 0.022904 1.214e-08 35359 TRUE reported
textfile 2 1 TRUE
1818 rs72835044 T G T G 0.2138 9e-04 0.03893 NA FALSE FALSE FALSE
GCST006061 0.0319 0.9778 537409 outcome TRUE reported 18218_48_CNRIP1_CB032
CNRIP1 chr2 68574958 0.021339 1.255e-23 35360 TRUE reported
textfile 2 1 TRUE
1819 rs7592976 G A G A -0.607 0.0026 0.07944 NA FALSE FALSE FALSE
GCST006061 0.0141 0.8536 537409 outcome TRUE reported 18218_48_CNRIP1_CB032
CNRIP1 chr2 68326669 0.01217 1e-200 35362 TRUE reported textfile 2

1 TRUE
1820 rs78857843 C T C T -0.1839 0.0181 0.01642 NA FALSE FALSE FALSE
GCST006061 0.0275 0.5114 537409 outcome TRUE reported 18218_48_CNRIP1_CB032
CNRIP1 chr2 68910908 0.032435 1.429e-08 35356 TRUE reported
textfile 2 1 TRUE
1821 rs9399136 C T C T -0.0735 -0.0032 0.26917 NA FALSE FALSE FALSE
GCST006061 0.0082 0.6956 537409 outcome TRUE reported 18218_48_CNRIP1_CB032
CNRIP1 chr6 135081201 0.009105 6.867e-16 35371 TRUE reported
textfile 2 1 TRUE
1822 rs1020839 T C T C -0.0893 0.001 0.16092 NA FALSE FALSE FALSE
GCST006061 0.0111 0.9288 537409 outcome TRUE reported
9484_75_DSG2_Desmoglein_2 DSG2 chr18 31409711 0.011092 8.199e-16 35350
TRUE reported textfile 2 1 TRUE
1823 rs11078597 C T C T -0.0548 0.0269 0.21836 NA FALSE FALSE FALSE
GCST006061 0.0093 0.003692 537409 outcome TRUE reported
9484_75_DSG2_Desmoglein_2 DSG2 chr17 1715069 0.009896 3.062e-08 35352
TRUE reported textfile 2 1 TRUE
1824 rs117225497 T G T G -0.2146 0.0701 0.01296 NA FALSE FALSE FALSE
GCST006061 0.0494 0.1555 537409 outcome TRUE reported
9484_75_DSG2_Desmoglein_2 DSG2 chr18 34596206 0.036255 3.235e-09 35337
TRUE reported textfile 2 1 TRUE
1825 rs1231281 A G A G -0.0473 0.006 0.44909 NA FALSE FALSE FALSE
GCST006061 0.0075 0.4251 537409 outcome TRUE reported
9484_75_DSG2_Desmoglein_2 DSG2 chr19 48735943 0.00825 9.846e-09 35377
TRUE reported textfile 2 1 TRUE
1826 rs147145691 T C T C -0.2669 -0.0676 0.02249 NA FALSE FALSE FALSE
GCST006061 0.0303 0.02585 537409 outcome TRUE reported
9484_75_DSG2_Desmoglein_2 DSG2 chr18 31526091 0.028244 3.4e-21 35348
TRUE reported textfile 2 1 TRUE
1827 rs148666188 C T C T -0.298 -0.0099 0.01098 NA FALSE FALSE FALSE
GCST006061 0.0364 0.7848 537409 outcome TRUE reported
9484_75_DSG2_Desmoglein_2 DSG2 chr18 30725028 0.038987 2.113e-14 35346
TRUE reported textfile 2 1 TRUE
1828 rs149181677 T C T C 0.1572 -0.0448 0.03967 NA FALSE FALSE FALSE
GCST006061 0.0194 0.02092 537409 outcome TRUE reported
9484_75_DSG2_Desmoglein_2 DSG2 chr9 133431409 0.021326 1.689e-13 35372
TRUE reported textfile 2 1 TRUE
1829 rs150816167 C T C T 0.1402 0.0293 0.04629 NA FALSE FALSE FALSE
GCST006061 0.0202 0.1468 537409 outcome TRUE reported
9484_75_DSG2_Desmoglein_2 DSG2 chr1 179602727 0.019401 4.959e-13 35358
TRUE reported textfile 2 1 TRUE
1830 rs537879 G T G T -0.1208 0.0065 0.46084 NA FALSE FALSE FALSE
GCST006061 0.0073 0.3746 537409 outcome TRUE reported
9484_75_DSG2_Desmoglein_2 DSG2 chr6 138092972 0.008187 2.847e-49 35362
TRUE reported textfile 2 1 TRUE
1831 rs62578578 A G A G 0.1761 -0.0469 0.03351 NA FALSE FALSE FALSE
GCST006061 0.0292 0.108 537409 outcome TRUE reported
9484_75_DSG2_Desmoglein_2 DSG2 chr9 132466382 0.023065 2.26e-14 35372
TRUE reported textfile 2 1 TRUE
1832 rs6905288 A G A G -0.0608 0.0119 0.45917 NA FALSE FALSE FALSE
GCST006061 0.0074 0.1081 537409 outcome TRUE reported
9484_75_DSG2_Desmoglein_2 DSG2 chr6 43791136 0.008237 1.568e-13 35359
TRUE reported textfile 2 1 TRUE
1833 rs708686 T C T C 0.0869 -4.00E-04 0.23081 NA FALSE FALSE FALSE
GCST006061 0.0081 0.9647 537409 outcome TRUE reported
9484_75_DSG2_Desmoglein_2 DSG2 chr19 5840608 0.009689 3.005e-19 35362
TRUE reported textfile 2 1 TRUE
1834 rs75426668 A G A G -0.1675 0.0205 0.02597 NA FALSE FALSE FALSE
GCST006061 0.0307 0.5052 537409 outcome TRUE reported
9484_75_DSG2_Desmoglein_2 DSG2 chr18 31797171 0.025716 7.348e-11 35350
TRUE reported textfile 2 1 TRUE
1835 rs9304098 T G T G -0.1905 0.0045 0.45421 NA FALSE FALSE FALSE
GCST006061 0.0072 0.5347 537409 outcome TRUE reported
9484_75_DSG2_Desmoglein_2 DSG2 chr18 31503667 0.008195 1.584e-119 35348
TRUE reported textfile 2 1 TRUE
1836 rs6445035 A G A G 0.0705 0.0154 0.1904 NA FALSE FALSE FALSE

```
     GCST006061  0.0089  0.08256 537409  outcome TRUE    reported    17774_38_SNRPD3_SMD3
     SNRPD3  chr3    165762312   0.010032    2.107e-12   35339   TRUE    reported
     textfile    2   1   TRUE
1837 rs10922098  T   C   T   C   -0.1953 -0.009  0.40011 NA  FALSE   FALSE   FALSE
     GCST006061  0.0075  0.2275  537409  outcome TRUE    reported    15430_165_OPTC_OPT
     OPTC    chr1    196695521   0.008122    9.436e-128  35338   TRUE    reported
     textfile    2   1   TRUE
1838 rs1707652   T   C   T   C   0.086   0.0081  0.3102  NA  FALSE   FALSE   FALSE
     GCST006061  0.0074  0.2701  537409  outcome TRUE    reported    15430_165_OPTC_OPT
     OPTC    chr3    165761011   0.008616    1.839e-23   35339   TRUE    reported
     textfile    2   1   TRUE
1839 rs35351085  T   C   T   C   0.1007  0.0061  0.04877 NA  FALSE   FALSE   FALSE
     GCST006061  0.022   0.7825  537409  outcome TRUE    reported    15430_165_OPTC_OPT
     OPTC    chr1    197992799   0.018457    4.87e-08    35343   TRUE    reported
     textfile    2   1   TRUE
1840 rs10922098  T   C   T   C   -0.1196 -0.009  0.40011 NA  FALSE   FALSE   FALSE
     GCST006061  0.0075  0.2275  537409  outcome TRUE    reported    8967_6_DUSP26_DUS26
     DUSP26  chr1    196695521   0.008179    1.985e-48   35338   TRUE    reported
     textfile    2   1   TRUE
1841 rs1707652   T   C   T   C   0.0982  0.0081  0.3102  NA  FALSE   FALSE   FALSE
     GCST006061  0.0074  0.2701  537409  outcome TRUE    reported    8967_6_DUSP26_DUS26
     DUSP26  chr3    165761011   0.008638    6.019e-30   35339   TRUE    reported
     textfile    2   1   TRUE
1842 rs71352239  T   C   T   C   0.064   -0.0041 0.30263 NA  FALSE   FALSE   FALSE
     GCST006061  0.0087  0.6372  537409  outcome TRUE    reported    8967_6_DUSP26_DUS26
     DUSP26  chr19   44926286    0.008754    2.657e-13   35368   TRUE    reported
     textfile    2   1   TRUE
1843 rs10922098  T   C   T   C   -0.1629 -0.009  0.40011 NA  FALSE   FALSE   FALSE
     GCST006061  0.0075  0.2275  537409  outcome TRUE    reported    9983_97_PRSS35_PRS35
     PRSS35  chr1    196695521   0.008066    1.059e-90   35338   TRUE    reported
     textfile    2   1   TRUE
1844 rs1707652   T   C   T   C   0.0689  0.0081  0.3102  NA  FALSE   FALSE   FALSE
     GCST006061  0.0074  0.2701  537409  outcome TRUE    reported    9983_97_PRSS35_PRS35
     PRSS35  chr3    165761011   0.00855 7.734e-16   35339   TRUE    reported    textfile
     2   1   TRUE
1845 rs1619994   G   A   G   A   0.0614  0.0168  0.2807  NA  FALSE   FALSE   FALSE
     GCST006061  0.0078  0.03057 537409  outcome TRUE    reported    17760_128_WDR5_WDR5
     WDR5    chr3    165744047   0.008758    2.366e-12   35339   TRUE    reported
     textfile    2   1   TRUE
1846 rs10922098  T   C   T   C   -0.1932 -0.009  0.40011 NA  FALSE   FALSE   FALSE
     GCST006061  0.0075  0.2275  537409  outcome TRUE    reported    8843_34_MYRF_MRF
     MYRF    chr1    196695521   0.008108    1.648e-125  35338   TRUE    reported
     textfile    2   1   TRUE
1847 rs11569415  A   G   A   G   -0.0616 0.0011  0.24276 NA  FALSE   FALSE   FALSE
     GCST006061  0.0097  0.9129  537409  outcome TRUE    reported    8843_34_MYRF_MRF
     MYRF    chr19   6716268 0.009251    2.766e-11   35372   TRUE    reported    textfile
     2   1   TRUE
1848 rs12403425  A   C   A   C   0.0493  -0.0064 0.2662  NA  FALSE   FALSE   FALSE
     GCST006061  0.009   0.4754  537409  outcome TRUE    reported    8843_34_MYRF_MRF
     MYRF    chr1    197845693   0.008999    4.294e-08   35337   TRUE    reported
     textfile    2   1   TRUE
1849 rs2686395   C   T   C   T   -0.0836 -0.0077 0.3101  NA  FALSE   FALSE   FALSE
     GCST006061  0.0074  0.2965  537409  outcome TRUE    reported    8843_34_MYRF_MRF
     MYRF    chr3    165767231   0.008598    2.404e-22   35339   TRUE    reported
     textfile    2   1   TRUE
1850 rs704   A   G   A   G   0.0441  -0.0155 0.49189 NA  FALSE   FALSE   FALSE   GCST006061
     0.0072  0.03144 537409  outcome TRUE    reported    8843_34_MYRF_MRF    MYRF    chr17
     28367840    0.007991    3.416e-08   35360   TRUE    reported    textfile    2   1   TRUE
1851 rs10922098  T   C   T   C   -0.1702 -0.009  0.40011 NA  FALSE   FALSE   FALSE
     GCST006061  0.0075  0.2275  537409  outcome TRUE    reported    17811_78_SULT1C2_ST1C2
     SULT1C2 chr1    196695521   0.008045    2.371e-99   35338   TRUE    reported
     textfile    2   1   TRUE
1852 rs1706435   G   A   G   A   0.0752  0.0117  0.36981 NA  FALSE   FALSE   FALSE
     GCST006061  0.0072  0.1047  537409  outcome TRUE    reported    17811_78_SULT1C2_ST1C2
     SULT1C2 chr3    165743688   0.008181    3.838e-20   35339   TRUE    reported
     textfile    2   1   TRUE
```

1853 rs111696008 A G A G -0.1578 -0.0259 0.02443 NA FALSE FALSE FALSE
GCST006061 0.0401 0.519 537409 outcome TRUE reported 12417_46_TPRKB_TPRKB
TPRKB chr2 216622986 0.026614 3.046e-09 35350 TRUE reported
textfile 2 1 TRUE
1854 rs583104 T G T G 0.0581 -0.0173 0.20819 NA FALSE FALSE FALSE
GCST006061 0.0087 0.04629 537409 outcome TRUE reported 12417_46_TPRKB_TPRKB
TPRKB chr1 109278685 0.009894 4.293e-09 35335 TRUE reported
textfile 2 1 TRUE
1855 rs6445035 A G A G 0.144 0.0154 0.1904 NA FALSE FALSE FALSE
GCST006061 0.0089 0.08256 537409 outcome TRUE reported 12417_46_TPRKB_TPRKB
TPRKB chr3 165762312 0.010158 1.296e-45 35330 TRUE reported
textfile 2 1 TRUE
1856 rs73169117 T C T C 0.0708 0.0051 0.11405 NA FALSE FALSE FALSE
GCST006061 0.0122 0.6755 537409 outcome TRUE reported 12417_46_TPRKB_TPRKB
TPRKB chr3 167217620 0.012677 2.341e-08 35345 TRUE reported
textfile 2 1 TRUE
1857 rs10922098 T C T C -0.1817 -0.009 0.40011 NA FALSE FALSE FALSE
GCST006061 0.0075 0.2275 537409 outcome TRUE reported 6528_95_EXTL2_EXTL2
EXTL2 chr1 196695521 0.008042 4.895e-113 35338 TRUE reported
textfile 2 1 TRUE
1858 rs28658508 G A G A 0.0559 0.0149 0.2026 NA FALSE FALSE FALSE
GCST006061 0.0089 0.09292 537409 outcome TRUE reported 6528_95_EXTL2_EXTL2
EXTL2 chr14 38746675 0.009807 1.2e-08 35364 TRUE reported textfile
2 1 TRUE
1859 rs704 A G A G 0.0789 -0.0155 0.49189 NA FALSE FALSE FALSE GCST006061
0.0072 0.03144 537409 outcome TRUE reported 6528_95_EXTL2_EXTL2 EXTL2 chr17
28367840 0.007922 2.294e-23 35360 TRUE reported textfile 2 1 TRUE
1860 rs10922098 T C T C -0.1084 -0.009 0.40011 NA FALSE FALSE FALSE
GCST006061 0.0075 0.2275 537409 outcome TRUE reported 12527_50_THRA_THA
THRA chr1 196695521 0.008084 5.317e-41 35329 TRUE reported
textfile 2 1 TRUE
1861 rs1706435 G A G A 0.0568 0.0117 0.36981 NA FALSE FALSE FALSE
GCST006061 0.0072 0.1047 537409 outcome TRUE reported 12527_50_THRA_THA
THRA chr3 165743688 0.008204 4.413e-12 35330 TRUE reported
textfile 2 1 TRUE
1862 rs704 A G A G 0.0543 -0.0155 0.49189 NA FALSE FALSE FALSE GCST006061
0.0072 0.03144 537409 outcome TRUE reported 12527_50_THRA_THA THRA chr17
28367840 0.00793 7.503e-12 35351 TRUE reported textfile 2 1 TRUE
1863 rs111696008 A G A G -0.1518 -0.0259 0.02443 NA FALSE FALSE FALSE
GCST006061 0.0401 0.519 537409 outcome TRUE reported 11160_56_RNF122_RN122
RNF122 chr2 216622986 0.026988 1.859e-08 35350 TRUE reported
textfile 2 1 TRUE
1864 rs1707652 T C T C 0.1234 0.0081 0.3102 NA FALSE FALSE FALSE
GCST006061 0.0074 0.2701 537409 outcome TRUE reported 11160_56_RNF122_RN122
RNF122 chr3 165761011 0.008742 3.061e-45 35330 TRUE reported
textfile 2 1 TRUE
1865 rs71352239 T C T C 0.08 -0.0041 0.30263 NA FALSE FALSE FALSE
GCST006061 0.0087 0.6372 537409 outcome TRUE reported 11160_56_RNF122_RN122
RNF122 chr19 44926286 0.008858 1.697e-19 35359 TRUE reported
textfile 2 1 TRUE
1866 rs1707652 T C T C 0.0958 0.0081 0.3102 NA FALSE FALSE FALSE
GCST006061 0.0074 0.2701 537409 outcome TRUE reported 7934_11_NDRG4_NDRG4
NDRG4 chr3 165761011 0.008554 4.123e-29 35339 TRUE reported
textfile 2 1 TRUE
1867 rs35351085 T C T C 0.1026 0.0061 0.04877 NA FALSE FALSE FALSE
GCST006061 0.022 0.7825 537409 outcome TRUE reported 7934_11_NDRG4_NDRG4
NDRG4 chr1 197992799 0.018307 2.091e-08 35343 TRUE reported
textfile 2 1 TRUE
1868 rs10922098 T C T C -0.2055 -0.009 0.40011 NA FALSE FALSE FALSE
GCST006061 0.0075 0.2275 537409 outcome TRUE reported 3472_40_BIRC5_Survivin
BIRC5 chr1 196695521 0.008102 6.039e-142 35338 TRUE reported
textfile 2 1 TRUE
1869 rs11569415 A G A G -0.0613 0.0011 0.24276 NA FALSE FALSE FALSE
GCST006061 0.0097 0.9129 537409 outcome TRUE reported 3472_40_BIRC5_Survivin
BIRC5 chr19 6716268 0.009255 3.501e-11 35372 TRUE reported textfile
2 1 TRUE

1870 rs1451915 A C A C 0.0646 -0.0107 0.13641 NA FALSE FALSE FALSE
GCST006061 0.0124 0.3895 537409 outcome TRUE reported 3472_40_BIRC5_Survivin
BIRC5 chr1 196058002 0.011568 2.345e-08 35335 TRUE reported
textfile 2 1 TRUE
1871 rs2686395 C T C T -0.0865 -0.0077 0.3101 NA FALSE FALSE FALSE
GCST006061 0.0074 0.2965 537409 outcome TRUE reported 3472_40_BIRC5_Survivin
BIRC5 chr3 165767231 0.008602 8.671e-24 35339 TRUE reported
textfile 2 1 TRUE
1872 rs1047286 A G A G -0.0592 -0.0013 0.24066 NA FALSE FALSE FALSE
GCST006061 0.0098 0.8921 537409 outcome TRUE reported 17822_57_RERG_RERG
RERG chr19 6713251 0.009306 2e-10 35372 TRUE reported textfile 2
1 TRUE
1873 rs10922098 T C T C -0.1671 -0.009 0.40011 NA FALSE FALSE FALSE
GCST006061 0.0075 0.2275 537409 outcome TRUE reported 17822_57_RERG_RERG
RERG chr1 196695521 0.008099 1.441e-94 35338 TRUE reported
textfile 2 1 TRUE
1874 rs1707652 T C T C 0.0767 0.0081 0.3102 NA FALSE FALSE FALSE
GCST006061 0.0074 0.2701 537409 outcome TRUE reported 17822_57_RERG_RERG
RERG chr3 165761011 0.008581 3.959e-19 35339 TRUE reported
textfile 2 1 TRUE
1875 rs10922098 T C T C -0.1996 -0.009 0.40011 NA FALSE FALSE FALSE
GCST006061 0.0075 0.2275 537409 outcome TRUE reported 10356_21_JUN_c_Jun JUN
chr1 196695521 0.008116 1.457e-133 35326 TRUE reported textfile 2
1 TRUE
1876 rs11569415 A G A G -0.066 0.0011 0.24276 NA FALSE FALSE FALSE
GCST006061 0.0097 0.9129 537409 outcome TRUE reported 10356_21_JUN_c_Jun JUN
chr19 6716268 0.009269 1.074e-12 35360 TRUE reported textfile 2 1
TRUE
1877 rs1707652 T C T C 0.0949 0.0081 0.3102 NA FALSE FALSE FALSE
GCST006061 0.0074 0.2701 537409 outcome TRUE reported 10356_21_JUN_c_Jun JUN
chr3 165761011 0.008608 2.926e-28 35327 TRUE reported textfile 2
1 TRUE
1878 rs10922098 T C T C -0.1774 -0.009 0.40011 NA FALSE FALSE FALSE
GCST006061 0.0075 0.2275 537409 outcome TRUE reported 17435_43_ETFA_ETFA
ETFA chr1 196695521 0.008129 1.406e-105 35338 TRUE reported
textfile 2 1 TRUE
1879 rs115181106 C T C T 0.2995 0.0055 0.01565 NA FALSE FALSE FALSE
GCST006061 0.0402 0.8903 537409 outcome TRUE reported 17435_43_ETFA_ETFA
ETFA chr4 73687208 0.03211 1.086e-20 35347 TRUE reported textfile
2 1 TRUE
1880 rs11569415 A G A G -0.0585 0.0011 0.24276 NA FALSE FALSE FALSE
GCST006061 0.0097 0.9129 537409 outcome TRUE reported 17435_43_ETFA_ETFA
ETFA chr19 6716268 0.009269 2.766e-10 35372 TRUE reported textfile
2 1 TRUE
1881 rs13107461 A G A G 0.2163 0.0472 0.01666 NA FALSE FALSE FALSE
GCST006061 0.0461 0.3058 537409 outcome TRUE reported 17435_43_ETFA_ETFA
ETFA chr4 68112851 0.031871 1.147e-11 35350 TRUE reported
textfile 2 1 TRUE
1882 rs13117588 T C T C 0.1929 0.0204 0.02482 NA FALSE FALSE FALSE
GCST006061 0.0265 0.4403 537409 outcome TRUE reported 17435_43_ETFA_ETFA
ETFA chr4 74508313 0.02559 4.77e-14 35350 TRUE reported textfile
2 1 TRUE
1883 rs1707652 T C T C 0.1156 0.0081 0.3102 NA FALSE FALSE FALSE
GCST006061 0.0074 0.2701 537409 outcome TRUE reported 17435_43_ETFA_ETFA
ETFA chr3 165761011 0.008604 3.738e-41 35339 TRUE reported
textfile 2 1 TRUE
1884 rs704 A G A G 0.1284 -0.0155 0.49189 NA FALSE FALSE FALSE GCST006061
0.0072 0.03144 537409 outcome TRUE reported 17435_43_ETFA_ETFA ETFA chr17
28367840 0.007983 3.242e-58 35360 TRUE reported textfile 2 1 TRUE
1885 rs78185702 T C T C 0.0867 -0.0197 0.11241 NA FALSE FALSE FALSE
GCST006061 0.0125 0.1153 537409 outcome TRUE reported 17435_43_ETFA_ETFA
ETFA chr15 76317894 0.012656 7.351e-12 35361 TRUE reported
textfile 2 1 TRUE
1886 rs10784960 C T C T 0.2079 -0.0123 0.331 NA FALSE FALSE FALSE
GCST006061 0.0079 0.1202 537409 outcome TRUE reported 8888_33_SMIM9_CX068
SMIM9 chr12 72275646 0.008533 4.113e-131 35362 TRUE reported

textfile 2 1 TRUE
1887 rs111696008 A G A G -0.1519 -0.0259 0.02443 NA FALSE FALSE FALSE
GCST006061 0.0401 0.519 537409 outcome TRUE reported 8888_33_SMIM9_CX068
SMIM9 chr2 216622986 0.026891 1.617e-08 35359 TRUE reported
textfile 2 1 TRUE
1888 rs6445035 A G A G 0.106 0.0154 0.1904 NA FALSE FALSE FALSE
GCST006061 0.0089 0.08256 537409 outcome TRUE reported 8888_33_SMIM9_CX068
SMIM9 chr3 165762312 0.010274 5.882e-25 35339 TRUE reported
textfile 2 1 TRUE
1889 rs10922098 T C T C -0.1541 -0.009 0.40011 NA FALSE FALSE FALSE
GCST006061 0.0075 0.2275 537409 outcome TRUE reported 18943_4_PTGR2_PTGR2
PTGR2 chr1 196695521 0.008138 5.78e-80 35338 TRUE reported
textfile 2 1 TRUE
1890 rs143243910 T C T C -0.1605 -0.0025 0.02114 NA FALSE FALSE FALSE
GCST006061 0.0233 0.9153 537409 outcome TRUE reported 18943_4_PTGR2_PTGR2
PTGR2 chr3 53520536 0.027261 3.922e-09 35350 TRUE reported
textfile 2 1 TRUE
1891 rs1707652 T C T C 0.0742 0.0081 0.3102 NA FALSE FALSE FALSE
GCST006061 0.0074 0.2701 537409 outcome TRUE reported 18943_4_PTGR2_PTGR2
PTGR2 chr3 165761011 0.008615 7.134e-18 35339 TRUE reported
textfile 2 1 TRUE
1892 rs77444140 A G A G 0.1499 0.0241 0.02383 NA FALSE FALSE FALSE
GCST006061 0.0329 0.4631 537409 outcome TRUE reported 18943_4_PTGR2_PTGR2
PTGR2 chr5 41171293 0.025941 7.541e-09 35357 TRUE reported
textfile 2 1 TRUE
1893 rs11064498 G A G A -0.0667 -8.00E-04 0.1306 NA FALSE FALSE FALSE
GCST006061 0.0102 0.939 537409 outcome TRUE reported 14663_44_RNF8_RNF8
RNF8 chr12 7064203 0.012108 3.615e-08 35390 TRUE reported textfile
2 1 TRUE
1894 rs1706435 G A G A 0.0762 0.0117 0.36981 NA FALSE FALSE FALSE
GCST006061 0.0072 0.1047 537409 outcome TRUE reported 14663_44_RNF8_RNF8
RNF8 chr3 165743688 0.008302 4.35e-20 35339 TRUE reported
textfile 2 1 TRUE
1895 rs2057681 G A G A 0.1162 -0.0136 0.28541 NA FALSE FALSE FALSE
GCST006061 0.0078 0.07965 537409 outcome TRUE reported 14663_44_RNF8_RNF8
RNF8 chr7 95308945 0.008921 8.691e-39 35349 TRUE reported
textfile 2 1 TRUE
1896 rs704 A G A G 0.0505 -0.0155 0.49189 NA FALSE FALSE FALSE GCST006061
0.0072 0.03144 537409 outcome TRUE reported 14663_44_RNF8_RNF8 RNF8 chr17
28367840 0.008049 3.523e-10 35360 TRUE reported textfile 2 1 TRUE
1897 rs1706435 G A G A 0.0469 0.0117 0.36981 NA FALSE FALSE FALSE
GCST006061 0.0072 0.1047 537409 outcome TRUE reported 13422_66_ENOX2_ENOX2
ENOX2 chr3 165743688 0.008201 1.072e-08 35333 TRUE reported
textfile 2 1 TRUE
1898 rs4784323 G A G A 0.0455 -0.0177 0.35334 NA FALSE FALSE FALSE
GCST006061 0.0081 0.02904 537409 outcome TRUE reported 13422_66_ENOX2_ENOX2
ENOX2 chr16 53763653 0.00829 4.052e-08 35360 TRUE reported textfile
2 1 TRUE
1899 rs1354034 C T C T 0.0779 0.0088 0.38119 NA FALSE FALSE FALSE
GCST006061 0.0073 0.2248 537409 outcome TRUE reported 16599_38_GPN1_GPN1
GPN1 chr3 56815721 0.008267 4.378e-21 35356 TRUE reported
textfile 2 1 TRUE
1900 rs2297380 T C T C 0.0593 -0.0053 0.42613 NA FALSE FALSE FALSE
GCST006061 0.0072 0.4559 537409 outcome TRUE reported 16599_38_GPN1_GPN1
GPN1 chr15 41527124 0.008117 2.752e-13 35363 TRUE reported
textfile 2 1 TRUE
1901 rs3170660 C T C T 0.1992 0.0108 0.41395 NA FALSE FALSE FALSE
GCST006061 0.0077 0.1572 537409 outcome TRUE reported 16599_38_GPN1_GPN1
GPN1 chr1 26884230 0.008114 4.192e-133 35370 TRUE reported
textfile 2 1 TRUE
1902 rs36004172 C A C A 0.0774 3e-04 0.13469 NA FALSE FALSE FALSE
GCST006061 0.012 0.9816 537409 outcome TRUE reported 16599_38_GPN1_GPN1
GPN1 chr1 26606265 0.011871 7.034e-11 35371 TRUE reported
textfile 2 1 TRUE
1903 rs3856240 C T C T 0.0471 -0.0011 0.48849 NA FALSE FALSE FALSE
GCST006061 0.0071 0.8713 537409 outcome TRUE reported 16599_38_GPN1_GPN1

GPN1 chr1 25566034 0.008063 5.184e-09 35368 TRUE reported
textfile 2 1 TRUE

1904 rs4980320 C T C T 0.0464 0.0041 0.46986 NA FALSE FALSE FALSE
GCST006061 0.0076 0.5858 537409 outcome TRUE reported 16599_38_GPN1_GPN1
GPN1 chr11 198986 0.008066 8.806e-09 35352 TRUE reported textfile
2 1 TRUE

1905 rs111696008 A G A G -0.1541 -0.0259 0.02443 NA FALSE FALSE FALSE
GCST006061 0.0401 0.519 537409 outcome TRUE reported 12730_3_NDC80_KNTC2
NDC80 chr2 216622986 0.026695 7.81e-09 35350 TRUE reported
textfile 2 1 TRUE

1906 rs1707652 T C T C 0.126 0.0081 0.3102 NA FALSE FALSE FALSE
GCST006061 0.0074 0.2701 537409 outcome TRUE reported 12730_3_NDC80_KNTC2
NDC80 chr3 165761011 0.00865 4.587e-48 35330 TRUE reported textfile
2 1 TRUE

1907 rs8106813 G A G A 0.0463 -0.0037 0.46477 NA FALSE FALSE FALSE
GCST006061 0.0086 0.672 537409 outcome TRUE reported 12730_3_NDC80_KNTC2
NDC80 chr19 44928401 0.008095 1.067e-08 35359 TRUE reported
textfile 2 1 TRUE

1908 rs1047286 A G A G -0.0629 -0.0013 0.24066 NA FALSE FALSE FALSE
GCST006061 0.0098 0.8921 537409 outcome TRUE reported 5024_67_RB1_Rb RB1
chr19 6713251 0.009281 1.227e-11 35372 TRUE reported textfile 2 1
TRUE

1909 rs10922098 T C T C -0.1763 -0.009 0.40011 NA FALSE FALSE FALSE
GCST006061 0.0075 0.2275 537409 outcome TRUE reported 5024_67_RB1_Rb RB1
chr1 196695521 0.008086 2.136e-105 35338 TRUE reported textfile 2
1 TRUE

1910 rs1707652 T C T C 0.0866 0.0081 0.3102 NA FALSE FALSE FALSE
GCST006061 0.0074 0.2701 537409 outcome TRUE reported 5024_67_RB1_Rb RB1
chr3 165761011 0.008572 5.377e-24 35339 TRUE reported textfile 2
1 TRUE

1911 rs10922098 T C T C -0.1446 -0.009 0.40011 NA FALSE FALSE FALSE
GCST006061 0.0075 0.2275 537409 outcome TRUE reported 7991_54_IQCF1_IQCF1
IQCF1 chr1 196695521 0.008051 4.024e-72 35338 TRUE reported
textfile 2 1 TRUE

1912 rs1355538 G A G A -0.0672 -0.0076 0.39847 NA FALSE FALSE FALSE
GCST006061 0.0072 0.2923 537409 outcome TRUE reported 7991_54_IQCF1_IQCF1
IQCF1 chr3 165787389 0.008058 7.478e-17 35338 TRUE reported
textfile 2 1 TRUE

1913 rs1697422 C T C T -0.0493 0.0053 0.48923 NA FALSE FALSE FALSE
GCST006061 0.0072 0.4547 537409 outcome TRUE reported 7991_54_IQCF1_IQCF1
IQCF1 chr1 21495404 0.00794 5.336e-10 35374 TRUE reported textfile
2 1 TRUE

1914 rs12146727 A G A G -0.0781 -0.0037 0.11875 NA FALSE FALSE FALSE
GCST006061 0.0105 0.7269 537409 outcome TRUE reported 6369_82_DEAF1_DEAF1
DEAF1 chr12 7063032 0.012716 8.167e-10 35390 TRUE reported textfile
2 1 TRUE

1915 rs1706435 G A G A 0.0803 0.0117 0.36981 NA FALSE FALSE FALSE
GCST006061 0.0072 0.1047 537409 outcome TRUE reported 6369_82_DEAF1_DEAF1
DEAF1 chr3 165743688 0.008352 6.909e-22 35339 TRUE reported
textfile 2 1 TRUE

1916 rs66489920 C T C T -0.055 0.0189 0.25071 NA FALSE FALSE FALSE
GCST006061 0.0078 0.01607 537409 outcome TRUE reported 6369_82_DEAF1_DEAF1
DEAF1 chr8 105580311 0.009338 3.857e-09 35362 TRUE reported
textfile 2 1 TRUE

1917 rs10922098 T C T C -0.1036 -0.009 0.40011 NA FALSE FALSE FALSE
GCST006061 0.0075 0.2275 537409 outcome TRUE reported 13430_50_SLC14A1_UT1
SLC14A1 chr1 196695521 0.008057 7.776e-38 35332 TRUE reported
textfile 2 1 TRUE

1918 rs10922098 T C T C -0.0721 -0.009 0.40011 NA FALSE FALSE FALSE
GCST006061 0.0075 0.2275 537409 outcome TRUE reported 13628_58_GRB14_GRB14
GRB14 chr1 196695521 0.008265 2.7e-18 35338 TRUE reported textfile
2 1 TRUE

1919 rs1707652 T C T C 0.092 0.0081 0.3102 NA FALSE FALSE FALSE
GCST006061 0.0074 0.2701 537409 outcome TRUE reported 13628_58_GRB14_GRB14
GRB14 chr3 165761011 0.00872 5.032e-26 35339 TRUE reported textfile
2 1 TRUE

1920 rs704 A G A G 0.0499 -0.0155 0.49189 NA FALSE FALSE FALSE GCST006061 0.0072 0.03144 537409 outcome TRUE reported 13628_58_GRB14_GRB14 GRB14 chr17 28367840 0.00811 7.622e-10 35360 TRUE reported textfile 2 1 TRUE

1921 rs71352239 T C T C 0.0593 -0.0041 0.30263 NA FALSE FALSE FALSE GCST006061 0.0087 0.6372 537409 outcome TRUE reported 13628_58_GRB14_GRB14 GRB14 chr19 44926286 0.008838 1.955e-11 35368 TRUE reported textfile 2 1 TRUE

1922 rs75327431 C T C T -0.0982 0.0083 0.05347 NA FALSE FALSE FALSE GCST006061 0.0222 0.7083 537409 outcome TRUE reported 13628_58_GRB14_GRB14 GRB14 chr12 42802980 0.017743 3.119e-08 35361 TRUE reported textfile 2 1 TRUE

1923 rs10922098 T C T C -0.1024 -0.009 0.40011 NA FALSE FALSE FALSE GCST006061 0.0075 0.2275 537409 outcome TRUE reported 9114_84_UMODL1_UROL1 UMODL1 chr1 196695521 0.00809 1.013e-36 35338 TRUE reported textfile 2 1 TRUE

1924 rs429358 C T C T -0.0763 0.005 0.17407 NA FALSE FALSE FALSE GCST006061 0.0103 0.6319 537409 outcome TRUE reported 14593_152_FAM210A_F210A FAM210A chr19 44908684 0.010712 1.056e-12 35368 TRUE reported textfile 2 1 TRUE

1925 rs704 A G A G 0.0969 -0.0155 0.49189 NA FALSE FALSE FALSE GCST006061 0.0072 0.03144 537409 outcome TRUE reported 14593_152_FAM210A_F210A FAM210A chr17 28367840 0.008043 2.009e-33 35360 TRUE reported textfile 2 1 TRUE

1926 rs872914 A G A G -0.049 0.0019 0.28935 NA FALSE FALSE FALSE GCST006061 0.0079 0.8076 537409 outcome TRUE reported 14593_152_FAM210A_F210A FAM210A chr4 73852384 0.008838 2.955e-08 35348 TRUE reported textfile 2 1 TRUE

1927 rs10822186 G A G A 0.0494 -0.0493 0.4663 NA FALSE FALSE FALSE GCST006061 0.0071 2.964e-12 537409 outcome TRUE reported 13652_2_TOM1L1_TM1L1 TOM1L1 chr10 63590623 0.0081 1.069e-09 35351 TRUE reported textfile 2 1 TRUE

1928 rs1355538 G A G A -0.0617 -0.0076 0.39847 NA FALSE FALSE FALSE GCST006061 0.0072 0.2923 537409 outcome TRUE reported 13652_2_TOM1L1_TM1L1 TOM1L1 chr3 165787389 0.008233 6.654e-14 35338 TRUE reported textfile 2 1 TRUE

1929 rs1926447 G A G A -0.0857 -0.0032 0.24322 NA FALSE FALSE FALSE GCST006061 0.0079 0.6866 537409 outcome TRUE reported 13652_2_TOM1L1_TM1L1 TOM1L1 chr13 46055809 0.009458 1.29e-19 35361 TRUE reported textfile 2 1 TRUE

1930 rs438811 T C T C -0.1164 -5.00E-04 0.23201 NA FALSE FALSE FALSE GCST006061 0.0088 0.9556 537409 outcome TRUE reported 13652_2_TOM1L1_TM1L1 TOM1L1 chr19 44913484 0.009645 1.56e-33 35366 TRUE reported textfile 2 1 TRUE

1931 rs111987450 A G A G 0.2391 -0.028 0.03492 NA FALSE FALSE FALSE GCST006061 0.0299 0.3489 537409 outcome TRUE reported 14294_61_MBD1_MBD1 MBD1 chr17 29118687 0.022755 7.986e-26 35361 TRUE reported textfile 2 1 TRUE

1932 rs12952423 C T C T 0.2948 -0.0737 0.01861 NA FALSE FALSE FALSE GCST006061 0.0626 0.2389 537409 outcome TRUE reported 14294_61_MBD1_MBD1 MBD1 chr17 27896901 0.030912 1.475e-21 35359 TRUE reported textfile 2 1 TRUE

1933 rs1355538 G A G A -0.0539 -0.0076 0.39847 NA FALSE FALSE FALSE GCST006061 0.0072 0.2923 537409 outcome TRUE reported 14294_61_MBD1_MBD1 MBD1 chr3 165787389 0.008052 2.172e-11 35338 TRUE reported textfile 2 1 TRUE

1934 rs1697422 C T C T -0.0528 0.0053 0.48923 NA FALSE FALSE FALSE GCST006061 0.0072 0.4547 537409 outcome TRUE reported 14294_61_MBD1_MBD1 MBD1 chr1 21495404 0.00794 2.939e-11 35374 TRUE reported textfile 2 1 TRUE

1935 rs184713097 A G A G 0.2208 -0.0241 0.02829 NA FALSE FALSE FALSE GCST006061 0.0287 0.4006 537409 outcome TRUE reported 14294_61_MBD1_MBD1 MBD1 chr17 30323750 0.025236 2.143e-18 35362 TRUE reported textfile 2 1 TRUE

1936 rs2945405 T C T C -0.103 0.0104 0.41098 NA FALSE FALSE FALSE GCST006061 0.0072 0.1511 537409 outcome TRUE reported 14294_61_MBD1_MBD1

MBD1 chr17 27581113 0.008517 1.153e-33 35359 TRUE reported
textfile 2 1 TRUE
1937 rs3764866 G A G A 0.1646 0.002 0.45167 NA FALSE FALSE FALSE
GCST006061 0.0075 0.7853 537409 outcome TRUE reported 14294_61_MBD1_MBD1
MBD1 chr17 28473186 0.008389 1.012e-85 35361 TRUE reported
textfile 2 1 TRUE
1938 rs4792597 G A G A -0.0806 -0.0123 0.09197 NA FALSE FALSE FALSE
GCST006061 0.012 0.3036 537409 outcome TRUE reported 14294_61_MBD1_MBD1
MBD1 chr17 15405219 0.01463 3.605e-08 35351 TRUE reported textfile
2 1 TRUE
1939 rs62058594 A G A G 0.2692 0.033 0.01245 NA FALSE FALSE FALSE
GCST006061 0.0392 0.4003 537409 outcome TRUE reported 14294_61_MBD1_MBD1
MBD1 chr17 27350440 0.039931 1.567e-11 35361 TRUE reported
textfile 2 1 TRUE
1940 rs8080168 G T G T 0.0926 0.0078 0.27295 NA FALSE FALSE FALSE
GCST006061 0.0084 0.3525 537409 outcome TRUE reported 14294_61_MBD1_MBD1
MBD1 chr17 18352252 0.009384 5.732e-23 35359 TRUE reported
textfile 2 1 TRUE
1941 rs887469 C T C T 0.1173 0.0043 0.12274 NA FALSE FALSE FALSE
GCST006061 0.0132 0.7458 537409 outcome TRUE reported 14294_61_MBD1_MBD1
MBD1 chr17 30365556 0.012704 2.628e-20 35362 TRUE reported
textfile 2 1 TRUE
1942 rs9902833 G A G A -0.276 -0.013 0.38604 NA FALSE FALSE FALSE
GCST006061 0.0074 0.07835 537409 outcome TRUE reported 14294_61_MBD1_MBD1
MBD1 chr17 28071104 0.008516 1e-200 35360 TRUE reported textfile
2 1 TRUE
1943 rs10922098 T C T C -0.1485 -0.009 0.40011 NA FALSE FALSE FALSE
GCST006061 0.0075 0.2275 537409 outcome TRUE reported 12827_37_RGS3_RGS3
RGS3 chr1 196695521 0.008088 2.675e-75 35329 TRUE reported
textfile 2 1 TRUE
1944 rs6445035 A G A G 0.0899 0.0154 0.1904 NA FALSE FALSE FALSE
GCST006061 0.0089 0.08256 537409 outcome TRUE reported 12827_37_RGS3_RGS3
RGS3 chr3 165762312 0.010094 5.274e-19 35330 TRUE reported
textfile 2 1 TRUE
1945 rs10922098 T C T C -0.1455 -0.009 0.40011 NA FALSE FALSE FALSE
GCST006061 0.0075 0.2275 537409 outcome TRUE reported 8853_2_CLEC4A_CLC4A
CLEC4A chr1 196695521 0.008127 1.102e-71 35338 TRUE reported
textfile 2 1 TRUE
1946 rs1707652 T C T C 0.083 0.0081 0.3102 NA FALSE FALSE FALSE
GCST006061 0.0074 0.2701 537409 outcome TRUE reported 8853_2_CLEC4A_CLC4A
CLEC4A chr3 165761011 0.008599 4.793e-22 35339 TRUE reported
textfile 2 1 TRUE
1947 rs2232710 C T C T 0.1616 -0.0163 0.0257 NA FALSE FALSE FALSE
GCST006061 0.042 0.6983 537409 outcome TRUE reported 8853_2_CLEC4A_CLC4A
CLEC4A chr14 94284149 0.024865 8.076e-11 35372 TRUE reported
textfile 2 1 TRUE
1948 rs704 A G A G 0.0519 -0.0155 0.49189 NA FALSE FALSE FALSE GCST006061
0.0072 0.03144 537409 outcome TRUE reported 8853_2_CLEC4A_CLC4A CLEC4A chr17
28367840 0.007999 8.687e-11 35360 TRUE reported textfile 2 1 TRUE
1949 rs11920905 A G A G -0.0541 -0.0047 0.28369 NA FALSE FALSE FALSE
GCST006061 0.008 0.5529 537409 outcome TRUE reported 12632_14_NAT1_ARY1
NAT1 chr3 126548264 0.008841 9.416e-10 35362 TRUE reported
textfile 2 1 TRUE
1950 rs12257143 C T C T -0.2866 -0.009 0.03461 NA FALSE FALSE FALSE
GCST006061 0.0174 0.6034 537409 outcome TRUE reported 12632_14_NAT1_ARY1
NAT1 chr10 7732765 0.021959 6.214e-39 35354 TRUE reported textfile
2 1 TRUE
1951 rs143827255 A G A G -0.1214 -0.0035 0.03945 NA FALSE FALSE FALSE
GCST006061 0.0249 0.8879 537409 outcome TRUE reported 12632_14_NAT1_ARY1
NAT1 chr3 50081211 0.020421 2.765e-09 35335 TRUE reported
textfile 2 1 TRUE
1952 rs2878628 G A G A -0.2469 -0.0117 0.42337 NA FALSE FALSE FALSE
GCST006061 0.0071 0.1016 537409 outcome TRUE reported 12632_14_NAT1_ARY1
NAT1 chr3 52550699 0.00796 1e-200 35343 TRUE reported textfile 2
1 TRUE
1953 rs10208580 G T G T 0.0926 0.0142 0.32373 NA FALSE FALSE FALSE

GCST006061 0.008 0.07643 537409 outcome TRUE reported 15449_33_TIMD4_TIM_4
TIMD4 chr2 202625718 0.008956 4.656e-25 35365 TRUE reported
textfile 2 1 TRUE
1954 rs10456852 T C T C 0.0883 0.0316 0.14474 NA FALSE FALSE FALSE
GCST006061 0.0116 0.00659 537409 outcome TRUE reported 15449_33_TIMD4_TIM_4
TIMD4 chr6 105908171 0.01192 1.282e-13 35368 TRUE reported textfile
2 1 TRUE
1955 rs10778118 T C T C -0.0536 -0.0091 0.24408 NA FALSE FALSE FALSE
GCST006061 0.0087 0.2948 537409 outcome TRUE reported 15449_33_TIMD4_TIM_4
TIMD4 chr12 101430728 0.009756 3.931e-08 35356 TRUE reported
textfile 2 1 TRUE
1956 rs10843390 T C T C 0.0817 -0.0203 0.29947 NA FALSE FALSE FALSE
GCST006061 0.0078 0.008999 537409 outcome TRUE reported
15449_33_TIMD4_TIM_4 TIMD4 chr12 29344058 0.009139 3.912e-19 35365
TRUE reported textfile 2 1 TRUE
1957 rs10849819 T C T C -0.0521 0.0073 0.29672 NA FALSE FALSE FALSE
GCST006061 0.0076 0.3344 537409 outcome TRUE reported 15449_33_TIMD4_TIM_4
TIMD4 chr12 120935886 0.00925 1.774e-08 35367 TRUE reported textfile
2 1 TRUE
1958 rs11230633 C T C T 0.0499 0.0071 0.3893 NA FALSE FALSE FALSE
GCST006061 0.0082 0.3865 537409 outcome TRUE reported 15449_33_TIMD4_TIM_4
TIMD4 chr11 61197989 0.008633 7.466e-09 35363 TRUE reported
textfile 2 1 TRUE
1959 rs116678101 T C T C 0.1227 -0.0235 0.1066 NA FALSE FALSE FALSE
GCST006061 0.0103 0.02274 537409 outcome TRUE reported 15449_33_TIMD4_TIM_4
TIMD4 chr20 35545279 0.013591 1.743e-19 35366 TRUE reported
textfile 2 1 TRUE
1960 rs12513886 A G A G 0.2543 0.0029 0.02658 NA FALSE FALSE FALSE
GCST006061 0.022 0.895 537409 outcome TRUE reported 15449_33_TIMD4_TIM_4
TIMD4 chr5 157067476 0.025937 1.078e-22 35351 TRUE reported
textfile 2 1 TRUE
1961 rs1377416 T C T C -0.0911 0.0088 0.36584 NA FALSE FALSE FALSE
GCST006061 0.0075 0.2358 537409 outcome TRUE reported 15449_33_TIMD4_TIM_4
TIMD4 chr11 47395195 0.008784 3.354e-25 35376 TRUE reported
textfile 2 1 TRUE
1962 rs143501783 C T C T 0.3086 -0.0333 0.01116 NA FALSE FALSE FALSE
GCST006061 0.0443 0.4525 537409 outcome TRUE reported 15449_33_TIMD4_TIM_4
TIMD4 chr17 7897001 0.038235 6.969e-16 35373 TRUE reported textfile
2 1 TRUE
1963 rs1864163 A G A G -0.0577 -0.0068 0.25315 NA FALSE FALSE FALSE
GCST006061 0.0083 0.418 537409 outcome TRUE reported 15449_33_TIMD4_TIM_4
TIMD4 chr16 56963321 0.009641 2.162e-09 35369 TRUE reported
textfile 2 1 TRUE
1964 rs2068888 A G A G 0.0515 0.0184 0.42949 NA FALSE FALSE FALSE
GCST006061 0.0071 0.009729 537409 outcome TRUE reported
15449_33_TIMD4_TIM_4 TIMD4 chr10 93079885 0.008432 1.009e-09 35374
TRUE reported textfile 2 1 TRUE
1965 rs2282718 A G A G 0.0467 0.0051 0.41077 NA FALSE FALSE FALSE
GCST006061 0.0075 0.5023 537409 outcome TRUE reported 15449_33_TIMD4_TIM_4
TIMD4 chr1 24914565 0.008546 4.638e-08 35371 TRUE reported
textfile 2 1 TRUE
1966 rs27289 T G T G -0.0585 0.0154 0.45961 NA FALSE FALSE FALSE GCST006061
0.0072 0.0309 537409 outcome TRUE reported 15449_33_TIMD4_TIM_4 TIMD4
chr5 97010574 0.008472 5.026e-12 35348 TRUE reported textfile 2
1 TRUE
1967 rs2737245 T G T G 0.0531 0.023 0.26133 NA FALSE FALSE FALSE
GCST006061 0.008 0.004078 537409 outcome TRUE reported
15449_33_TIMD4_TIM_4 TIMD4 chr8 115646356 0.009507 2.333e-08 35349
TRUE reported textfile 2 1 TRUE
1968 rs28638168 A G A G 0.0854 0.0156 0.12918 NA FALSE FALSE FALSE
GCST006061 0.0113 0.1699 537409 outcome TRUE reported 15449_33_TIMD4_TIM_4
TIMD4 chr5 157231931 0.012636 1.395e-11 35350 TRUE reported
textfile 2 1 TRUE
1969 rs34318854 T C T C 0.0505 0.0013 0.41174 NA FALSE FALSE FALSE
GCST006061 0.0073 0.8532 537409 outcome TRUE reported 15449_33_TIMD4_TIM_4
TIMD4 chr16 85881600 0.008509 2.939e-09 35376 TRUE reported

textfile 2 1 TRUE
1970 rs35068491 C T C T 0.057 0.0094 0.21885 NA FALSE FALSE FALSE
GCST006061 0.0095 0.3261 537409 outcome TRUE reported 15449_33_TIMD4_TIM_4
TIMD4 chr21 34866010 0.010088 1.601e-08 35374 TRUE reported
textfile 2 1 TRUE
1971 rs4704826 A C A C -0.2416 0.0109 0.35793 NA FALSE FALSE FALSE
GCST006061 0.0075 0.1446 537409 outcome TRUE reported 15449_33_TIMD4_TIM_4
TIMD4 chr5 156965071 0.008727 1.039e-168 35351 TRUE reported
textfile 2 1 TRUE
1972 rs56206139 C A C A 0.0876 0.0297 0.07854 NA FALSE FALSE FALSE
GCST006061 0.015 0.04848 537409 outcome TRUE reported 15449_33_TIMD4_TIM_4
TIMD4 chr20 40480065 0.015602 1.969e-08 35373 TRUE reported
textfile 2 1 TRUE
1973 rs59870886 T C T C 0.0753 -0.0113 0.16744 NA FALSE FALSE FALSE
GCST006061 0.0115 0.3267 537409 outcome TRUE reported 15449_33_TIMD4_TIM_4
TIMD4 chr2 231377634 0.011308 2.755e-11 35367 TRUE reported
textfile 2 1 TRUE
1974 rs601338 A G A G 0.0532 0.0056 0.39439 NA FALSE FALSE FALSE
GCST006061 0.0076 0.4559 537409 outcome TRUE reported 15449_33_TIMD4_TIM_4
TIMD4 chr19 48703417 0.008615 6.604e-10 35377 TRUE reported
textfile 2 1 TRUE
1975 rs6555971 C T C T 0.0832 -0.0182 0.35519 NA FALSE FALSE FALSE
GCST006061 0.0077 0.01811 537409 outcome TRUE reported 15449_33_TIMD4_TIM_4
TIMD4 chr5 157364668 0.008826 4.222e-21 35354 TRUE reported
textfile 2 1 TRUE
1976 rs66817580 T G T G 0.134 0.0024 0.14508 NA FALSE FALSE FALSE
GCST006061 0.0102 0.814 537409 outcome TRUE reported 15449_33_TIMD4_TIM_4
TIMD4 chr21 41317710 0.01189 1.852e-29 35368 TRUE reported textfile
2 1 TRUE
1977 rs6810489 G T G T 0.0555 0.0023 0.41542 NA FALSE FALSE FALSE
GCST006061 0.0075 0.7592 537409 outcome TRUE reported 15449_33_TIMD4_TIM_4
TIMD4 chr4 98956294 0.008559 8.918e-11 35343 TRUE reported
textfile 2 1 TRUE
1978 rs7214944 A C A C -0.0494 0.0025 0.41565 NA FALSE FALSE FALSE
GCST006061 0.0073 0.7262 537409 outcome TRUE reported 15449_33_TIMD4_TIM_4
TIMD4 chr17 76613078 0.008503 6.266e-09 35380 TRUE reported
textfile 2 1 TRUE
1979 rs73026960 G A G A 0.1032 -0.004 0.09399 NA FALSE FALSE FALSE
GCST006061 0.012 0.7369 537409 outcome TRUE reported 15449_33_TIMD4_TIM_4
TIMD4 chr11 126165516 0.014699 2.208e-12 35367 TRUE reported
textfile 2 1 TRUE
1980 rs7626629 C T C T -0.0535 0.0067 0.42856 NA FALSE FALSE FALSE
GCST006061 0.0075 0.3709 537409 outcome TRUE reported 15449_33_TIMD4_TIM_4
TIMD4 chr3 69795789 0.008474 2.732e-10 35359 TRUE reported
textfile 2 1 TRUE
1981 rs77262773 T C T C 0.2101 0.014 0.02263 NA FALSE FALSE FALSE
GCST006061 0.0283 0.6205 537409 outcome TRUE reported 15449_33_TIMD4_TIM_4
TIMD4 chr17 69253570 0.027908 5.135e-14 35371 TRUE reported
textfile 2 1 TRUE
1982 rs780094 C T C T 0.052 -0.0076 0.33917 NA FALSE FALSE FALSE
GCST006061 0.0073 0.2951 537409 outcome TRUE reported 15449_33_TIMD4_TIM_4
TIMD4 chr2 27518370 0.008901 5.16e-09 35373 TRUE reported
textfile 2 1 TRUE
1983 rs894491 G A G A 0.051 0.0234 0.31758 NA FALSE FALSE FALSE
GCST006061 0.008 0.003551 537409 outcome TRUE reported
15449_33_TIMD4_TIM_4 TIMD4 chr15 65333881 0.009 1.456e-08 35361 TRUE
reported textfile 2 1 TRUE
1984 rs10208580 G T G T 0.0531 0.0142 0.32373 NA FALSE FALSE FALSE
GCST006061 0.008 0.07643 537409 outcome TRUE reported 5028_59_CD163_sCD163
CD163 chr2 202625718 0.008925 2.693e-09 35365 TRUE reported
textfile 2 1 TRUE
1985 rs10838705 A G A G 0.0573 0.0084 0.35566 NA FALSE FALSE FALSE
GCST006061 0.0074 0.2547 537409 outcome TRUE reported 5028_59_CD163_sCD163
CD163 chr11 47403380 0.008706 4.656e-11 35376 TRUE reported
textfile 2 1 TRUE
1986 rs11054859 A G A G -0.1402 -0.0016 0.10581 NA FALSE FALSE FALSE

GCST006061 0.0108 0.8811 537409 outcome TRUE reported 5028_59_CD163_sCD163
CD163 chr12 7617180 0.01367 1.109e-24 35389 TRUE reported textfile 2
1 TRUE
1987 rs11153164 A G A G -0.0587 -0.0063 0.30333 NA FALSE FALSE FALSE
GCST006061 0.0076 0.4061 537409 outcome TRUE reported 5028_59_CD163_sCD163
CD163 chr6 109275106 0.009049 8.773e-11 35380 TRUE reported
textfile 2 1 TRUE
1988 rs1139490 T C T C -0.0726 0.003 0.29555 NA FALSE FALSE FALSE
GCST006061 0.008 0.7022 537409 outcome TRUE reported 5028_59_CD163_sCD163
CD163 chr4 99123990 0.009097 1.453e-15 35346 TRUE reported
textfile 2 1 TRUE
1989 rs114475253 A G A G 0.2259 0.0314 0.0114 NA FALSE FALSE FALSE
GCST006061 0.0301 0.297 537409 outcome TRUE reported 5028_59_CD163_sCD163
CD163 chr2 134177065 0.040686 2.82e-08 35391 TRUE reported
textfile 2 1 TRUE
1990 rs11571364 A G A G 0.2027 -2.00E-04 0.04967 NA FALSE FALSE FALSE
GCST006061 0.0193 0.9922 537409 outcome TRUE reported 5028_59_CD163_sCD163
CD163 chr17 7008372 0.019015 1.565e-26 35374 TRUE reported textfile
2 1 TRUE
1991 rs11820880 C T C T 0.0752 -0.0027 0.16454 NA FALSE FALSE FALSE
GCST006061 0.0103 0.794 537409 outcome TRUE reported 5028_59_CD163_sCD163
CD163 chr11 134398559 0.011201 1.898e-11 35150 TRUE reported
textfile 2 1 TRUE
1992 rs1226013 G T G T -0.0456 0.0067 0.48318 NA FALSE FALSE FALSE
GCST006061 0.0075 0.3673 537409 outcome TRUE reported 5028_59_CD163_sCD163
CD163 chr6 10539056 0.008344 4.622e-08 35373 TRUE reported
textfile 2 1 TRUE
1993 rs13203302 T C T C 0.1024 -0.0338 0.10316 NA FALSE FALSE FALSE
GCST006061 0.013 0.009309 537409 outcome TRUE reported
5028_59_CD163_sCD163 CD163 chr6 95559822 0.013636 5.943e-14 35343
TRUE reported textfile 2 1 TRUE
1994 rs1444087 C T C T -0.0497 0.0023 0.47008 NA FALSE FALSE FALSE
GCST006061 0.0074 0.7504 537409 outcome TRUE reported 5028_59_CD163_sCD163
CD163 chr4 145451146 0.008316 2.282e-09 35361 TRUE reported
textfile 2 1 TRUE
1995 rs1571262 G T G T -0.0552 0.0179 0.23055 NA FALSE FALSE FALSE
GCST006061 0.0083 0.03146 537409 outcome TRUE reported 5028_59_CD163_sCD163
CD163 chr9 110125975 0.009906 2.514e-08 35370 TRUE reported
textfile 2 1 TRUE
1996 rs192992318 T G T G -0.2066 -0.0332 0.01802 NA FALSE FALSE FALSE
GCST006061 0.0386 0.3891 537409 outcome TRUE reported 5028_59_CD163_sCD163
CD163 chr2 137367017 0.031376 4.557e-11 35393 TRUE reported
textfile 2 1 TRUE
1997 rs2099684 G A G A 0.0528 -0.0015 0.30891 NA FALSE FALSE FALSE
GCST006061 0.0077 0.8444 537409 outcome TRUE reported 5028_59_CD163_sCD163
CD163 chr1 161530340 0.008997 4.401e-09 35351 TRUE reported
textfile 2 1 TRUE
1998 rs3184504 C T C T -0.0664 0.0074 0.37862 NA FALSE FALSE FALSE
GCST006061 0.0075 0.3235 537409 outcome TRUE reported 5028_59_CD163_sCD163
CD163 chr12 111446804 0.008569 9.285e-15 35371 TRUE reported
textfile 2 1 TRUE
1999 rs3747207 A G A G 0.0602 0.0153 0.22927 NA FALSE FALSE FALSE
GCST006061 0.0085 0.07308 537409 outcome TRUE reported 5028_59_CD163_sCD163
CD163 chr22 43928975 0.009865 1.043e-09 35372 TRUE reported
textfile 2 1 TRUE
2000 rs4788460 T C T C 0.0876 -5.00E-04 0.26356 NA FALSE FALSE FALSE
GCST006061 0.008 0.9514 537409 outcome TRUE reported 5028_59_CD163_sCD163
CD163 chr16 72120610 0.009398 1.155e-20 35375 TRUE reported
textfile 2 1 TRUE
2001 rs55714927 T C T C 0.2609 1e-04 0.21166 NA FALSE FALSE FALSE
GCST006061 0.0098 0.9926 537409 outcome TRUE reported 5028_59_CD163_sCD163
CD163 chr17 7176997 0.010242 3.944e-143 35370 TRUE reported textfile
2 1 TRUE
2002 rs55924807 A G A G -0.1221 -0.0099 0.05005 NA FALSE FALSE FALSE
GCST006061 0.0234 0.6732 537409 outcome TRUE reported 5028_59_CD163_sCD163
CD163 chr2 134198007 0.019341 2.735e-10 35391 TRUE reported

textfile 2 1 TRUE
2003 rs62165726 A C A C -0.6505 -0.0263 0.03053 NA FALSE FALSE FALSE
GCST006061 0.0212 0.2141 537409 outcome TRUE reported 5028_59_CD163_sCD163
CD163 chr2 134208991 0.023913 5.951e-163 35391 TRUE reported
textfile 2 1 TRUE
2004 rs681343 T C T C 0.0812 0.0054 0.3954 NA FALSE FALSE FALSE
GCST006061 0.0076 0.4742 537409 outcome TRUE reported 5028_59_CD163_sCD163
CD163 chr19 48703205 0.008524 1.632e-21 35377 TRUE reported
textfile 2 1 TRUE
2005 rs7037849 T C T C 0.0631 -1.00E-04 0.3756 NA FALSE FALSE FALSE
GCST006061 0.0074 0.9858 537409 outcome TRUE reported 5028_59_CD163_sCD163
CD163 chr9 137079368 0.008584 1.965e-13 35262 TRUE reported
textfile 2 1 TRUE
2006 rs73021423 C T C T -0.208 0.0053 0.05901 NA FALSE FALSE FALSE
GCST006061 0.0143 0.7111 537409 outcome TRUE reported 5028_59_CD163_sCD163
CD163 chr11 126383746 0.01766 5.048e-32 35375 TRUE reported textfile
2 1 TRUE
2007 rs73600961 G A G A -0.0668 -0.0039 0.16867 NA FALSE FALSE FALSE
GCST006061 0.0111 0.7275 537409 outcome TRUE reported 5028_59_CD163_sCD163
CD163 chr11 134732947 0.01074 4.986e-10 35143 TRUE reported textfile
2 1 TRUE
2008 rs79744308 A G A G -0.1226 0.014 0.03495 NA FALSE FALSE FALSE
GCST006061 0.0185 0.4498 537409 outcome TRUE reported 5028_59_CD163_sCD163
CD163 chr19 5827754 0.022324 3.98e-08 35366 TRUE reported textfile
2 1 TRUE
2009 rs9677256 G A G A -0.1626 -0.0064 0.08003 NA FALSE FALSE FALSE
GCST006061 0.0115 0.5775 537409 outcome TRUE reported 5028_59_CD163_sCD163
CD163 chr2 134274292 0.015524 1.133e-25 35392 TRUE reported
textfile 2 1 TRUE
2010 rs9860747 C T C T 0.1065 -0.0121 0.14574 NA FALSE FALSE FALSE
GCST006061 0.01 0.2233 537409 outcome TRUE reported 5028_59_CD163_sCD163
CD163 chr3 186869628 0.012 6.992e-19 35364 TRUE reported textfile
2 1 TRUE
2011 rs1047286 A G A G -0.0653 -0.0013 0.24066 NA FALSE FALSE FALSE
GCST006061 0.0098 0.8921 537409 outcome TRUE reported
15570_99_CR2_Complement_receptor_type_2 CR2 chr19 6713251 0.009871 3.708e-11
35372 TRUE reported textfile 2 1 TRUE
2012 rs10849448 G A G A 0.0513 0.0056 0.28422 NA FALSE FALSE FALSE
GCST006061 0.009 0.532 537409 outcome TRUE reported
15570_99_CR2_Complement_receptor_type_2 CR2 chr12 6384185 0.009371 4.388e-08
35380 TRUE reported textfile 2 1 TRUE
2013 rs111727450 T C T C -0.1649 -0.0245 0.02449 NA FALSE FALSE FALSE
GCST006061 0.0285 0.3913 537409 outcome TRUE reported
15570_99_CR2_Complement_receptor_type_2 CR2 chr13 108263046 0.026704 6.612e-10
35356 TRUE reported textfile 2 1 TRUE
2014 rs11241688 G T G T -0.0743 0.0161 0.23093 NA FALSE FALSE FALSE
GCST006061 0.0086 0.06168 537409 outcome TRUE reported
15570_99_CR2_Complement_receptor_type_2 CR2 chr5 123342854 0.010076 1.657e-13
35351 TRUE reported textfile 2 1 TRUE
2015 rs116852327 T G T G 0.2591 -0.0213 0.01186 NA FALSE FALSE FALSE
GCST006061 0.0385 0.5806 537409 outcome TRUE reported
15570_99_CR2_Complement_receptor_type_2 CR2 chr17 7721481 0.036557 1.364e-12
35377 TRUE reported textfile 2 1 TRUE
2016 rs12535578 A G A G 0.0741 0.002 0.26162 NA FALSE FALSE FALSE
GCST006061 0.0082 0.8051 537409 outcome TRUE reported
15570_99_CR2_Complement_receptor_type_2 CR2 chr7 55086893 0.009643 1.539e-14
35337 TRUE reported textfile 2 1 TRUE
2017 rs12950377 C T C T -0.0976 0.0091 0.1524 NA FALSE FALSE FALSE
GCST006061 0.0106 0.3893 537409 outcome TRUE reported
15570_99_CR2_Complement_receptor_type_2 CR2 chr17 83053776 0.011952 3.193e-16
35022 TRUE reported textfile 2 1 TRUE
2018 rs1528151 G T G T 0.098 0.0059 0.2366 NA FALSE FALSE FALSE
GCST006061 0.0085 0.4866 537409 outcome TRUE reported
15570_99_CR2_Complement_receptor_type_2 CR2 chr7 16067549 0.009901 4.268e-23
35368 TRUE reported textfile 2 1 TRUE
2019 rs1660367 C T C T 0.0938 0.0235 0.20448 NA FALSE FALSE FALSE

GCST006061 0.0091 0.009955 537409 outcome TRUE reported
15570_99_CR2_Complement_receptor_type_2 CR2 chr1 214233596 0.01053 5.202e-19
35359 TRUE reported textfile 2 1 TRUE
2020 rs187099314 T C T C 0.1191 0.0287 0.06588 NA FALSE FALSE FALSE
GCST006061 0.016 0.07216 537409 outcome TRUE reported
15570_99_CR2_Complement_receptor_type_2 CR2 chr9 133255354 0.017006 2.498e-12
35370 TRUE reported textfile 2 1 TRUE
2021 rs2058269 C T C T 0.057 -0.001 0.42611 NA FALSE FALSE FALSE
GCST006061 0.0072 0.8843 537409 outcome TRUE reported
15570_99_CR2_Complement_receptor_type_2 CR2 chr7 16193629 0.008548 2.591e-11
35368 TRUE reported textfile 2 1 TRUE
2022 rs2273215 A G A G 0.0691 -0.0042 0.46881 NA FALSE FALSE FALSE
GCST006061 0.0073 0.5691 537409 outcome TRUE reported
15570_99_CR2_Complement_receptor_type_2 CR2 chr6 170276994 0.00855 6.384e-16
34944 TRUE reported textfile 2 1 TRUE
2023 rs3813948 C T C T -0.1177 -0.015 0.0696 NA FALSE FALSE FALSE
GCST006061 0.0129 0.2459 537409 outcome TRUE reported
15570_99_CR2_Complement_receptor_type_2 CR2 chr1 207096513 0.016661 1.614e-12
35369 TRUE reported textfile 2 1 TRUE
2024 rs41556717 T C T C 0.1903 -0.0483 0.01931 NA FALSE FALSE FALSE
GCST006061 0.0325 0.1371 537409 outcome TRUE reported
15570_99_CR2_Complement_receptor_type_2 CR2 chr17 7496000 0.029662 1.403e-10
35374 TRUE reported textfile 2 1 TRUE
2025 rs56115403 A G A G 0.092 0.0012 0.21597 NA FALSE FALSE FALSE
GCST006061 0.01 0.901 537409 outcome TRUE reported
15570_99_CR2_Complement_receptor_type_2 CR2 chr17 7160579 0.010348 6.056e-19
35371 TRUE reported textfile 2 1 TRUE
2026 rs61240730 C T C T -0.2527 -0.0016 0.14193 NA FALSE FALSE FALSE
GCST006061 0.0119 0.8911 537409 outcome TRUE reported
15570_99_CR2_Complement_receptor_type_2 CR2 chr1 207450585 0.01227 2.998e-94
35377 TRUE reported textfile 2 1 TRUE
2027 rs67838388 C T C T 0.0805 -0.0085 0.1597 NA FALSE FALSE FALSE
GCST006061 0.0095 0.3705 537409 outcome TRUE reported
15570_99_CR2_Complement_receptor_type_2 CR2 chr4 177049701 0.011537 2.998e-12
35356 TRUE reported textfile 2 1 TRUE
2028 rs7196849 G A G A -0.0475 0.0059 0.39749 NA FALSE FALSE FALSE
GCST006061 0.0073 0.417 537409 outcome TRUE reported
15570_99_CR2_Complement_receptor_type_2 CR2 chr16 86000204 0.008586 3.16e-08
35378 TRUE reported textfile 2 1 TRUE
2029 rs76324903 A G A G 0.1259 0.0277 0.0381 NA FALSE FALSE FALSE
GCST006061 0.0262 0.289 537409 outcome TRUE reported
15570_99_CR2_Complement_receptor_type_2 CR2 chr9 20489771 0.022032 1.1e-08
35359 TRUE reported textfile 2 1 TRUE
2030 rs76368427 C T C T 0.0905 0.027 0.09353 NA FALSE FALSE FALSE
GCST006061 0.0145 0.06232 537409 outcome TRUE reported
15570_99_CR2_Complement_receptor_type_2 CR2 chr12 94429267 0.014428 3.557e-10
35366 TRUE reported textfile 2 1 TRUE
2031 rs76428106 C T C T 0.205 -0.0446 0.01359 NA FALSE FALSE FALSE
GCST006061 0.0438 0.3085 537409 outcome TRUE reported
15570_99_CR2_Complement_receptor_type_2 CR2 chr13 28029870 0.036727 2.382e-08
35363 TRUE reported textfile 2 1 TRUE
2032 rs77328788 T C T C -0.069 -0.0115 0.12995 NA FALSE FALSE FALSE
GCST006061 0.0116 0.3227 537409 outcome TRUE reported
15570_99_CR2_Complement_receptor_type_2 CR2 chr9 36994015 0.01248 3.22e-08
35353 TRUE reported textfile 2 1 TRUE
2033 rs10956485 C T C T -0.089 -0.0038 0.25402 NA FALSE FALSE FALSE
GCST006061 0.0079 0.6333 537409 outcome TRUE reported 5728_60_FCRL1_FCRL1
FCRL1 chr8 129608339 0.009327 1.405e-21 35372 TRUE reported
textfile 2 1 TRUE
2034 rs12784975 C T C T -0.0728 -0.0062 0.16605 NA FALSE FALSE FALSE
GCST006061 0.0094 0.5063 537409 outcome TRUE reported 5728_60_FCRL1_FCRL1
FCRL1 chr10 96620380 0.010978 3.32e-11 35368 TRUE reported
textfile 2 1 TRUE
2035 rs13104150 A G A G -0.0533 -0.0054 0.42427 NA FALSE FALSE FALSE
GCST006061 0.0072 0.4466 537409 outcome TRUE reported 5728_60_FCRL1_FCRL1
FCRL1 chr4 25876874 0.008254 1.063e-10 35357 TRUE reported

textfile 2 1 TRUE
2036 rs160045 A G A G 0.0635 0.0183 0.2828 NA FALSE FALSE FALSE
GCST006061 0.0079 0.01983 537409 outcome TRUE reported 5728_60_FCRL1_FCRL1
FCRL1 chr5 88845125 0.009052 2.299e-12 35345 TRUE reported
textfile 2 1 TRUE
2037 rs2867429 A G A G 0.0583 -0.0157 0.17798 NA FALSE FALSE FALSE
GCST006061 0.0087 0.07155 537409 outcome TRUE reported 5728_60_FCRL1_FCRL1
FCRL1 chr15 81611197 0.010653 4.438e-08 35365 TRUE reported
textfile 2 1 TRUE
2038 rs41264463 A C A C 0.1404 0.0055 0.05404 NA FALSE FALSE FALSE
GCST006061 0.0135 0.6816 537409 outcome TRUE reported 5728_60_FCRL1_FCRL1
FCRL1 chr1 159140582 0.018459 2.826e-14 35358 TRUE reported
textfile 2 1 TRUE
2039 rs4494728 T C T C -0.0506 0.0133 0.42807 NA FALSE FALSE FALSE
GCST006061 0.0072 0.06589 537409 outcome TRUE reported 5728_60_FCRL1_FCRL1
FCRL1 chr2 65362379 0.008247 8.492e-10 35353 TRUE reported
textfile 2 1 TRUE
2040 rs4948492 T C T C -0.1157 0.0088 0.27999 NA FALSE FALSE FALSE
GCST006061 0.0075 0.242 537409 outcome TRUE reported 5728_60_FCRL1_FCRL1
FCRL1 chr10 61959980 0.008978 5.362e-38 35351 TRUE reported
textfile 2 1 TRUE
2041 rs55803162 T C T C 0.086 0.0069 0.24959 NA FALSE FALSE FALSE
GCST006061 0.0081 0.395 537409 outcome TRUE reported 5728_60_FCRL1_FCRL1
FCRL1 chr19 33260733 0.00943 7.511e-20 35364 TRUE reported textfile
2 1 TRUE
2042 rs58889466 C T C T -0.1639 -0.0344 0.05582 NA FALSE FALSE FALSE
GCST006061 0.0139 0.01342 537409 outcome TRUE reported 5728_60_FCRL1_FCRL1
FCRL1 chr5 158826675 0.01763 1.451e-20 35369 TRUE reported textfile
2 1 TRUE
2043 rs7266148 T G T G 0.0576 0.0063 0.21937 NA FALSE FALSE FALSE
GCST006061 0.009 0.4828 537409 outcome TRUE reported 5728_60_FCRL1_FCRL1
FCRL1 chr20 31843267 0.009915 6.277e-09 35367 TRUE reported
textfile 2 1 TRUE
2044 rs73217470 G A G A -0.101 -0.015 0.06028 NA FALSE FALSE FALSE
GCST006061 0.0197 0.4472 537409 outcome TRUE reported 5728_60_FCRL1_FCRL1
FCRL1 chr13 71939282 0.017273 5.001e-09 35356 TRUE reported
textfile 2 1 TRUE
2045 rs76428106 C T C T 0.2619 -0.0446 0.01359 NA FALSE FALSE FALSE
GCST006061 0.0438 0.3085 537409 outcome TRUE reported 5728_60_FCRL1_FCRL1
FCRL1 chr13 28029870 0.035306 1.189e-13 35363 TRUE reported
textfile 2 1 TRUE
2046 rs76529516 G A G A 0.1277 0.0421 0.03763 NA FALSE FALSE FALSE
GCST006061 0.026 0.1054 537409 outcome TRUE reported 5728_60_FCRL1_FCRL1
FCRL1 chr2 60356235 0.021168 1.612e-09 35346 TRUE reported
textfile 2 1 TRUE
2047 rs77262773 T C T C 0.1553 0.014 0.02263 NA FALSE FALSE FALSE
GCST006061 0.0283 0.6205 537409 outcome TRUE reported 5728_60_FCRL1_FCRL1
FCRL1 chr17 69253570 0.026994 8.756e-09 35371 TRUE reported
textfile 2 1 TRUE
2048 rs907091 T C T C 0.0737 -0.028 0.44536 NA FALSE FALSE FALSE
GCST006061 0.0071 8.357e-05 537409 outcome TRUE reported 5728_60_FCRL1_FCRL1
FCRL1 chr17 39765489 0.008218 3.004e-19 35379 TRUE reported
textfile 2 1 TRUE
2049 rs9939427 A G A G 0.0715 0.0031 0.22677 NA FALSE FALSE FALSE
GCST006061 0.0088 0.723 537409 outcome TRUE reported 5728_60_FCRL1_FCRL1
FCRL1 chr16 85982485 0.009776 2.599e-13 35378 TRUE reported
textfile 2 1 TRUE
2050 rs10212397 A G A G -0.2611 -4.00E-04 0.17565 NA FALSE FALSE FALSE
GCST006061 0.0098 0.9666 537409 outcome TRUE reported 14271_23_RAB6B_RAB6B
RAB6B chr3 133888296 0.010569 9.444e-135 35358 TRUE reported
textfile 2 1 TRUE
2051 rs10984373 T C T C 0.0491 -0.0056 0.35333 NA FALSE FALSE FALSE
GCST006061 0.0075 0.456 537409 outcome TRUE reported 14271_23_RAB6B_RAB6B
RAB6B chr9 97921471 0.00836 4.272e-09 35345 TRUE reported textfile
2 1 TRUE
2052 rs1354034 C T C T 0.0686 0.0088 0.38119 NA FALSE FALSE FALSE

GCST006061 0.0073 0.2248 537409 outcome TRUE reported 14271_23_RAB6B_RAB6B
RAB6B chr3 56815721 0.008273 1.111e-16 35356 TRUE reported
textfile 2 1 TRUE
2053 rs10213052 C T C T 0.0515 0.0033 0.26945 NA FALSE FALSE FALSE
GCST006061 0.008 0.6829 537409 outcome TRUE reported 11142_11_ANGPTL1_ANGL1
ANGPTL1 chr4 174897101 0.009088 1.455e-08 35358 TRUE reported
textfile 2 1 TRUE
2054 rs12130811 A G A G -0.0921 0.0076 0.12063 NA FALSE FALSE FALSE
GCST006061 0.0108 0.4806 537409 outcome TRUE reported 11142_11_ANGPTL1_ANGL1
ANGPTL1 chr1 177546126 0.012577 2.43e-13 35354 TRUE reported
textfile 2 1 TRUE
2055 rs140477729 T G T G 0.1785 -0.018 0.01607 NA FALSE FALSE FALSE
GCST006061 0.0361 0.619 537409 outcome TRUE reported 11142_11_ANGPTL1_ANGL1
ANGPTL1 chr1 179134848 0.032565 4.22e-08 35349 TRUE reported
textfile 2 1 TRUE
2056 rs333947 A G A G -0.0647 -0.0071 0.14937 NA FALSE FALSE FALSE
GCST006061 0.01 0.4808 537409 outcome TRUE reported 11142_11_ANGPTL1_ANGL1
ANGPTL1 chr1 109928142 0.011561 2.188e-08 35340 TRUE reported
textfile 2 1 TRUE
2057 rs4457535 G A G A -0.2531 0.0123 0.37199 NA FALSE FALSE FALSE
GCST006061 0.0073 0.09044 537409 outcome TRUE reported 11142_11_ANGPTL1_ANGL1
ANGPTL1 chr1 178528051 0.008364 1e-200 35348 TRUE reported textfile
2 1 TRUE
2058 rs4681671 T G T G -0.0449 -0.0325 0.49918 NA FALSE FALSE FALSE
GCST006061 0.0072 6.058e-06 537409 outcome TRUE reported
11142_11_ANGPTL1_ANGL1 ANGPTL1 chr3 58242901 0.008084 2.793e-08 35348
TRUE reported textfile 2 1 TRUE
2059 rs6678237 G A G A -0.1008 -0.0158 0.06933 NA FALSE FALSE FALSE
GCST006061 0.0136 0.2424 537409 outcome TRUE reported 11142_11_ANGPTL1_ANGL1
ANGPTL1 chr1 168639966 0.016267 5.771e-10 35353 TRUE reported
textfile 2 1 TRUE
2060 rs78811502 C T C T 0.2238 -0.0247 0.02787 NA FALSE FALSE FALSE
GCST006061 0.0242 0.3065 537409 outcome TRUE reported 11142_11_ANGPTL1_ANGL1
ANGPTL1 chr1 180072659 0.024913 2.626e-19 35349 TRUE reported
textfile 2 1 TRUE
2061 rs10225292 C T C T -0.0928 0.0082 0.34802 NA FALSE FALSE FALSE
GCST006061 0.0073 0.2619 537409 outcome TRUE reported
2743_5_SHH_Sonic_Hedgehog SHH chr7 156380544 0.008731 2.183e-26 35337
TRUE reported textfile 2 1 TRUE
2062 rs114006326 T G T G -0.2846 -0.046 0.01107 NA FALSE FALSE FALSE
GCST006061 0.0539 0.3936 537409 outcome TRUE reported
2743_5_SHH_Sonic_Hedgehog SHH chr2 219330488 0.040305 1.652e-12 35369
TRUE reported textfile 2 1 TRUE
2063 rs1864163 A G A G -0.0618 -0.0068 0.25315 NA FALSE FALSE FALSE
GCST006061 0.0083 0.418 537409 outcome TRUE reported
2743_5_SHH_Sonic_Hedgehog SHH chr16 56963321 0.00958 1.112e-10 35369 TRUE
reported textfile 2 1 TRUE
2064 rs9333592 T C T C -0.123 -0.0121 0.05384 NA FALSE FALSE FALSE
GCST006061 0.0155 0.4328 537409 outcome TRUE reported
2743_5_SHH_Sonic_Hedgehog SHH chr7 155812377 0.018499 2.953e-11 35340
TRUE reported textfile 2 1 TRUE
2065 rs102275 C T C T -0.0627 0.011 0.39406 NA FALSE FALSE FALSE
GCST006061 0.0074 0.1363 537409 outcome TRUE reported 5843_60_EHMT2_NG36
EHMT2 chr11 61790331 0.00862 3.494e-13 35367 TRUE reported textfile
2 1 TRUE
2066 rs111696008 A G A G -0.2285 -0.0259 0.02443 NA FALSE FALSE FALSE
GCST006061 0.0401 0.519 537409 outcome TRUE reported 5843_60_EHMT2_NG36
EHMT2 chr2 216622986 0.028056 3.807e-16 35359 TRUE reported
textfile 2 1 TRUE
2067 rs12045422 T G T G -0.0587 -0.006 0.33302 NA FALSE FALSE FALSE
GCST006061 0.0076 0.4302 537409 outcome TRUE reported 5843_60_EHMT2_NG36
EHMT2 chr1 201975384 0.008939 5.14e-11 35355 TRUE reported
textfile 2 1 TRUE
2068 rs1601935 T G T G -0.0835 0.0106 0.3473 NA FALSE FALSE FALSE
GCST006061 0.0076 0.1648 537409 outcome TRUE reported 5843_60_EHMT2_NG36
EHMT2 chr15 58379566 0.008854 4.056e-21 35364 TRUE reported

textfile 2 1 TRUE
2069 rs1800588 T C T C 0.0993 0.0034 0.19095 NA FALSE FALSE FALSE
GCST006061 0.0084 0.6863 537409 outcome TRUE reported 5843_60_EHMT2_NG36
EHMT2 chr15 58431476 0.0107 1.695e-20 35365 TRUE reported textfile
2 1 TRUE
2070 rs3764261 A C A C 0.1251 0.0132 0.32148 NA FALSE FALSE FALSE
GCST006061 0.0076 0.08361 537409 outcome TRUE reported 5843_60_EHMT2_NG36
EHMT2 chr16 56959412 0.009006 7.157e-44 35369 TRUE reported
textfile 2 1 TRUE
2071 rs4128744 T C T C 0.0801 0.0229 0.10386 NA FALSE FALSE FALSE
GCST006061 0.0107 0.03258 537409 outcome TRUE reported 5843_60_EHMT2_NG36
EHMT2 chr8 20062144 0.013974 9.916e-09 35369 TRUE reported
textfile 2 1 TRUE
2072 rs55881728 G A G A -0.0569 -0.0072 0.41277 NA FALSE FALSE FALSE
GCST006061 0.0074 0.3268 537409 outcome TRUE reported 5843_60_EHMT2_NG36
EHMT2 chr2 203441633 0.008621 4.106e-11 35365 TRUE reported
textfile 2 1 TRUE
2073 rs6073971 T C T C -0.0691 -0.0096 0.21924 NA FALSE FALSE FALSE
GCST006061 0.0097 0.32 537409 outcome TRUE reported 5843_60_EHMT2_NG36
EHMT2 chr20 45961331 0.010182 1.15e-11 35370 TRUE reported
textfile 2 1 TRUE
2074 rs73169103 T C T C 0.1365 0.0178 0.03563 NA FALSE FALSE FALSE
GCST006061 0.0249 0.4756 537409 outcome TRUE reported 5843_60_EHMT2_NG36
EHMT2 chr3 167190675 0.02281 2.175e-09 35354 TRUE reported textfile
2 1 TRUE
2075 rs7674623 T C T C 0.114 -6.00E-04 0.21323 NA FALSE FALSE FALSE
GCST006061 0.0092 0.9469 537409 outcome TRUE reported 5843_60_EHMT2_NG36
EHMT2 chr4 79873527 0.010338 2.828e-28 35355 TRUE reported
textfile 2 1 TRUE
2076 rs780093 C T C T 0.06 -0.0074 0.34157 NA FALSE FALSE FALSE
GCST006061 0.0073 0.3116 537409 outcome TRUE reported 5843_60_EHMT2_NG36
EHMT2 chr2 27519736 0.008911 1.663e-11 35373 TRUE reported
textfile 2 1 TRUE
2077 rs8106813 G A G A -0.053 -0.0037 0.46477 NA FALSE FALSE FALSE
GCST006061 0.0086 0.672 537409 outcome TRUE reported 5843_60_EHMT2_NG36
EHMT2 chr19 44928401 0.008508 4.688e-10 35368 TRUE reported
textfile 2 1 TRUE
2078 rs102275 C T C T -0.0788 0.011 0.39406 NA FALSE FALSE FALSE
GCST006061 0.0074 0.1363 537409 outcome TRUE reported 7223_60_S100A13_S100A13
S100A13 chr11 61790331 0.008616 5.9e-20 35367 TRUE reported textfile
2 1 TRUE
2079 rs11668861 T G T G 0.3922 0.0017 0.46793 NA FALSE FALSE FALSE
GCST006061 0.0073 0.8169 537409 outcome TRUE reported 7223_60_S100A13_S100A13
S100A13 chr19 44877713 0.006151 1e-200 35366 TRUE reported textfile
2 1 TRUE
2080 rs149857247 T C T C -0.4183 0.0325 0.01619 NA FALSE FALSE FALSE
GCST006061 0.0323 0.3151 537409 outcome TRUE reported 7223_60_S100A13_S100A13
S100A13 chr19 46373607 0.039466 3.014e-26 35367 TRUE reported
textfile 2 1 TRUE
2081 rs2075649 G A G A 0.4976 -0.0104 0.40218 NA FALSE FALSE FALSE
GCST006061 0.0074 0.1623 537409 outcome TRUE reported 7223_60_S100A13_S100A13
S100A13 chr19 44892073 0.006149 1e-200 35366 TRUE reported textfile
2 1 TRUE
2082 rs2722757 G A G A 0.1117 -0.0114 0.39365 NA FALSE FALSE FALSE
GCST006061 0.0077 0.1364 537409 outcome TRUE reported 7223_60_S100A13_S100A13
S100A13 chr19 44385498 0.00997 3.932e-29 35369 TRUE reported textfile
2 1 TRUE
2083 rs525028 A G A G 0.0568 0.0036 0.29251 NA FALSE FALSE FALSE
GCST006061 0.0078 0.6444 537409 outcome TRUE reported 7223_60_S100A13_S100A13
S100A13 chr11 116834800 0.009253 8.322e-10 35377 TRUE reported
textfile 2 1 TRUE
2084 rs55923289 C T C T -0.4014 -0.0094 0.17219 NA FALSE FALSE FALSE
GCST006061 0.0115 0.4142 537409 outcome TRUE reported 7223_60_S100A13_S100A13
S100A13 chr19 44737327 0.012735 1e-200 35369 TRUE reported textfile
2 1 TRUE
2085 rs6509222 C T C T -0.0885 -0.0101 0.41704 NA FALSE FALSE FALSE

GCST006061 0.0074 0.1733 537409 outcome TRUE reported 7223_60_S100A13_S100A13
S100A13 chr19 45569743 0.009867 2.989e-19 35372 TRUE reported
textfile 2 1 TRUE
2086 rs7819706 G A G A 0.1104 0.0163 0.0959 NA FALSE FALSE FALSE
GCST006061 0.0108 0.132 537409 outcome TRUE reported 7223_60_S100A13_S100A13
S100A13 chr8 19986904 0.01422 8.249e-15 35368 TRUE reported textfile
2 1 TRUE
2087 rs8099992 G A G A 0.0543 0.0083 0.47155 NA FALSE FALSE FALSE
GCST006061 0.0075 0.2672 537409 outcome TRUE reported 7223_60_S100A13_S100A13
S100A13 chr19 46273233 0.009764 2.68e-08 35366 TRUE reported
textfile 2 1 TRUE
2088 rs10229613 C T C T 0.0512 -0.0084 0.36781 NA FALSE FALSE FALSE
GCST006061 0.0074 0.2579 537409 outcome TRUE reported 14618_26_VOPP1_ECOP
VOPP1 chr7 55467171 0.008639 3.093e-09 35340 TRUE reported
textfile 2 1 TRUE
2089 rs10751648 C T C T -0.0529 -0.0053 0.47184 NA FALSE FALSE FALSE
GCST006061 0.0086 0.5379 537409 outcome TRUE reported 14618_26_VOPP1_ECOP
VOPP1 chr11 307244 0.008366 2.567e-10 35338 TRUE reported textfile
2 1 TRUE
2090 rs10922098 T C T C -0.0839 -0.009 0.40011 NA FALSE FALSE FALSE
GCST006061 0.0075 0.2275 537409 outcome TRUE reported 14618_26_VOPP1_ECOP
VOPP1 chr1 196695521 0.008462 3.572e-23 35338 TRUE reported
textfile 2 1 TRUE
2091 rs111696008 A G A G -0.166 -0.0259 0.02443 NA FALSE FALSE FALSE
GCST006061 0.0401 0.519 537409 outcome TRUE reported 14618_26_VOPP1_ECOP
VOPP1 chr2 216622986 0.027511 1.6e-09 35359 TRUE reported textfile
2 1 TRUE
2092 rs117042408 A G A G -0.2917 0.0052 0.01999 NA FALSE FALSE FALSE
GCST006061 0.0243 0.8313 537409 outcome TRUE reported 14618_26_VOPP1_ECOP
VOPP1 chr7 55555698 0.029687 8.725e-23 35339 TRUE reported
textfile 2 1 TRUE
2093 rs12117692 T C T C -0.0602 0.0036 0.17728 NA FALSE FALSE FALSE
GCST006061 0.0094 0.701 537409 outcome TRUE reported 14618_26_VOPP1_ECOP
VOPP1 chr1 30754408 0.010957 3.927e-08 35364 TRUE reported
textfile 2 1 TRUE
2094 rs1260326 C T C T 0.0492 -0.0069 0.34132 NA FALSE FALSE FALSE
GCST006061 0.0073 0.3401 537409 outcome TRUE reported 14618_26_VOPP1_ECOP
VOPP1 chr2 27508073 0.008768 2.006e-08 35373 TRUE reported
textfile 2 1 TRUE
2095 rs1373064 T C T C 0.0761 -0.0016 0.36005 NA FALSE FALSE FALSE
GCST006061 0.0074 0.8265 537409 outcome TRUE reported 14618_26_VOPP1_ECOP
VOPP1 chr11 60400727 0.008659 1.509e-18 35374 TRUE reported
textfile 2 1 TRUE
2096 rs1800588 T C T C 0.0826 0.0034 0.19095 NA FALSE FALSE FALSE
GCST006061 0.0084 0.6863 537409 outcome TRUE reported 14618_26_VOPP1_ECOP
VOPP1 chr15 58431476 0.010503 3.711e-15 35365 TRUE reported
textfile 2 1 TRUE
2097 rs2066714 C T C T 0.0848 -0.0189 0.10312 NA FALSE FALSE FALSE
GCST006061 0.0099 0.05666 537409 outcome TRUE reported 14618_26_VOPP1_ECOP
VOPP1 chr9 104824472 0.013587 4.335e-10 35364 TRUE reported
textfile 2 1 TRUE
2098 rs2304262 C T C T -0.1497 0.0294 0.13449 NA FALSE FALSE FALSE
GCST006061 0.0108 0.006308 537409 outcome TRUE reported 14618_26_VOPP1_ECOP
VOPP1 chr19 1036553 0.012199 1.288e-34 35321 TRUE reported textfile
2 1 TRUE
2099 rs2686395 C T C T -0.0975 -0.0077 0.3101 NA FALSE FALSE FALSE
GCST006061 0.0074 0.2965 537409 outcome TRUE reported 14618_26_VOPP1_ECOP
VOPP1 chr3 165767231 0.008922 8.507e-28 35339 TRUE reported
textfile 2 1 TRUE
2100 rs2740488 C A C A -0.063 -0.0159 0.25976 NA FALSE FALSE FALSE
GCST006061 0.0081 0.04886 537409 outcome TRUE reported 14618_26_VOPP1_ECOP
VOPP1 chr9 104899461 0.009425 2.322e-11 35363 TRUE reported
textfile 2 1 TRUE
2101 rs34216592 A G A G 0.0715 0.0105 0.13279 NA FALSE FALSE FALSE
GCST006061 0.0127 0.4071 537409 outcome TRUE reported 14618_26_VOPP1_ECOP
VOPP1 chr17 68781934 0.012171 4.24e-09 35370 TRUE reported

textfile 2 1 TRUE
2102 rs3764261 A C A C 0.1449 0.0132 0.32148 NA FALSE FALSE FALSE
GCST006061 0.0076 0.08361 537409 outcome TRUE reported 14618_26_VOPP1_ECOP
VOPP1 chr16 56959412 0.008831 1.692e-60 35369 TRUE reported
textfile 2 1 TRUE
2103 rs3917538 A G A G 0.0578 -0.0137 0.21967 NA FALSE FALSE FALSE
GCST006061 0.0082 0.09535 537409 outcome TRUE reported 14618_26_VOPP1_ECOP
VOPP1 chr7 95308581 0.009993 7.29e-09 35349 TRUE reported
textfile 2 1 TRUE
2104 rs4810479 T C T C 0.0903 0.0129 0.26936 NA FALSE FALSE FALSE
GCST006061 0.0081 0.1093 537409 outcome TRUE reported 14618_26_VOPP1_ECOP
VOPP1 chr20 45916409 0.009298 2.691e-22 35370 TRUE reported
textfile 2 1 TRUE
2105 rs705379 A G A G -0.089 -8.00E-04 0.38289 NA FALSE FALSE FALSE
GCST006061 0.0072 0.9068 537409 outcome TRUE reported 14618_26_VOPP1_ECOP
VOPP1 chr7 95324583 0.008544 2.094e-25 35349 TRUE reported
textfile 2 1 TRUE
2106 rs8106813 G A G A -0.069 -0.0037 0.46477 NA FALSE FALSE FALSE
GCST006061 0.0086 0.672 537409 outcome TRUE reported 14618_26_VOPP1_ECOP
VOPP1 chr19 44928401 0.008366 1.615e-16 35368 TRUE reported
textfile 2 1 TRUE
2107 rs10231308 C T C T 0.1249 -0.0186 0.23473 NA FALSE FALSE FALSE
GCST006061 0.0082 0.02308 537409 outcome TRUE reported 11103_24_HSPB1_HSP_27
HSPB1 chr7 76376379 0.009638 2.101e-38 35340 TRUE reported
textfile 2 1 TRUE
2108 rs113088829 C T C T -0.1281 0.0065 0.05337 NA FALSE FALSE FALSE
GCST006061 0.0202 0.7474 537409 outcome TRUE reported 11103_24_HSPB1_HSP_27
HSPB1 chr7 75667928 0.017664 4.105e-13 35340 TRUE reported
textfile 2 1 TRUE
2109 rs117296956 A G A G -0.2958 0.0408 0.02756 NA FALSE FALSE FALSE
GCST006061 0.0297 0.1694 537409 outcome TRUE reported 11103_24_HSPB1_HSP_27
HSPB1 chr7 77220299 0.024674 4.086e-33 35342 TRUE reported
textfile 2 1 TRUE
2110 rs13240755 A G A G 0.2916 -0.0011 0.34276 NA FALSE FALSE FALSE
GCST006061 0.0075 0.8824 537409 outcome TRUE reported 11103_24_HSPB1_HSP_27
HSPB1 chr7 75976791 0.008488 1e-200 35341 TRUE reported textfile
2 1 TRUE
2111 rs34179550 A G A G 0.0766 -0.0501 0.11116 NA FALSE FALSE FALSE
GCST006061 0.0127 7.809e-05 537409 outcome TRUE reported
11103_24_HSPB1_HSP_27 HSPB1 chr7 75615065 0.013176 6.11e-09 35339
TRUE reported textfile 2 1 TRUE
2112 rs6460050 A C A C 0.0508 0.0019 0.32954 NA FALSE FALSE FALSE
GCST006061 0.008 0.8071 537409 outcome TRUE reported 11103_24_HSPB1_HSP_27
HSPB1 chr7 73661400 0.008661 4.485e-09 35346 TRUE reported
textfile 2 1 TRUE
2113 rs6926219 A G A G -0.0824 0.0192 0.46006 NA FALSE FALSE FALSE
GCST006061 0.0071 0.006928 537409 outcome TRUE reported
11103_24_HSPB1_HSP_27 HSPB1 chr6 122399661 0.008059 1.532e-24 35347
TRUE reported textfile 2 1 TRUE
2114 rs7926172 A G A G 0.0596 -0.0023 0.23069 NA FALSE FALSE FALSE
GCST006061 0.0093 0.8091 537409 outcome TRUE reported 11103_24_HSPB1_HSP_27
HSPB1 chr11 225180 0.009533 4.053e-10 35343 TRUE reported textfile
2 1 TRUE
2115 rs10235272 G A G A 0.0568 0.0078 0.20746 NA FALSE FALSE FALSE
GCST006061 0.0091 0.3885 537409 outcome TRUE reported
12975_11_KRT20_Keratin_20 KRT20 chr7 1243961 0.009904 9.76e-09 35316
TRUE reported textfile 2 1 TRUE
2116 rs10922098 T C T C -0.0966 -0.009 0.40011 NA FALSE FALSE FALSE
GCST006061 0.0075 0.2275 537409 outcome TRUE reported
12975_11_KRT20_Keratin_20 KRT20 chr1 196695521 0.008133 1.543e-32 35329
TRUE reported textfile 2 1 TRUE
2117 rs11650915 A G A G -0.0457 -0.0088 0.4129 NA FALSE FALSE FALSE
GCST006061 0.0075 0.2396 537409 outcome TRUE reported
12975_11_KRT20_Keratin_20 KRT20 chr17 40867144 0.00807 1.487e-08 35372
TRUE reported textfile 2 1 TRUE
2118 rs1706435 G A G A 0.046 0.0117 0.36981 NA FALSE FALSE FALSE

GCST006061 0.0072 0.1047 537409 outcome TRUE reported
12975_11_KRT20_Keratin_20 KRT20 chr3 165743688 0.008236 2.338e-08 35330
TRUE reported textfile 2 1 TRUE
2119 rs4796667 C T C T -0.0586 -6.00E-04 0.3592 NA FALSE FALSE FALSE
GCST006061 0.0076 0.9327 537409 outcome TRUE reported
12975_11_KRT20_Keratin_20 KRT20 chr17 41522464 0.008303 1.691e-12 35371
TRUE reported textfile 2 1 TRUE
2120 rs632111 G A G A -0.0534 0.0049 0.37262 NA FALSE FALSE FALSE
GCST006061 0.0075 0.5123 537409 outcome TRUE reported
12975_11_KRT20_Keratin_20 KRT20 chr19 48705721 0.008277 1.109e-10 35368
TRUE reported textfile 2 1 TRUE
2121 rs1023875 G A G A -0.1932 -0.0016 0.02562 NA FALSE FALSE FALSE
GCST006061 0.0271 0.9533 537409 outcome TRUE reported 9906_21_SPOCK3_TICN3
SPOCK3 chr4 166817793 0.025942 9.511e-14 35364 TRUE reported
textfile 2 1 TRUE
2122 rs116614155 A G A G -0.354 -0.0013 0.0196 NA FALSE FALSE FALSE
GCST006061 0.0374 0.972 537409 outcome TRUE reported 9906_21_SPOCK3_TICN3
SPOCK3 chr4 167641203 0.028804 1.024e-34 35362 TRUE reported
textfile 2 1 TRUE
2123 rs11668974 A G A G 0.1228 -0.0118 0.04573 NA FALSE FALSE FALSE
GCST006061 0.025 0.6379 537409 outcome TRUE reported 9906_21_SPOCK3_TICN3
SPOCK3 chr19 44156638 0.018981 9.833e-11 35370 TRUE reported
textfile 2 1 TRUE
2124 rs13143783 T C T C -0.404 0.0032 0.08876 NA FALSE FALSE FALSE
GCST006061 0.013 0.8076 537409 outcome TRUE reported 9906_21_SPOCK3_TICN3
SPOCK3 chr4 167209986 0.014183 1.778e-178 35363 TRUE reported
textfile 2 1 TRUE
2125 rs138620872 G A G A 0.2799 -0.0106 0.01929 NA FALSE FALSE FALSE
GCST006061 0.0388 0.7847 537409 outcome TRUE reported 9906_21_SPOCK3_TICN3
SPOCK3 chr16 72672741 0.029125 7.239e-22 35372 TRUE reported
textfile 2 1 TRUE
2126 rs1434282 T C T C 0.0608 -0.0021 0.26524 NA FALSE FALSE FALSE
GCST006061 0.008 0.7916 537409 outcome TRUE reported 9906_21_SPOCK3_TICN3
SPOCK3 chr1 199041592 0.009109 2.476e-11 35343 TRUE reported
textfile 2 1 TRUE
2127 rs17504449 T C T C 0.2635 0.0219 0.0298 NA FALSE FALSE FALSE
GCST006061 0.0223 0.3281 537409 outcome TRUE reported 9906_21_SPOCK3_TICN3
SPOCK3 chr4 165724362 0.024004 4.923e-28 35365 TRUE reported
textfile 2 1 TRUE
2128 rs17599599 A G A G 0.9313 0.0047 0.0324 NA FALSE FALSE FALSE
GCST006061 0.0264 0.8603 537409 outcome TRUE reported 9906_21_SPOCK3_TICN3
SPOCK3 chr4 167040587 0.017419 1e-200 35363 TRUE reported textfile
2 1 TRUE
2129 rs181329862 A C A C 0.2677 0.0031 0.01031 NA FALSE FALSE FALSE
GCST006061 0.0595 0.9585 537409 outcome TRUE reported 9906_21_SPOCK3_TICN3
SPOCK3 chr4 162031690 0.039764 1.671e-11 35349 TRUE reported
textfile 2 1 TRUE
2130 rs192358371 G A G A 0.7037 0.0259 0.01166 NA FALSE FALSE FALSE
GCST006061 0.0545 0.6349 537409 outcome TRUE reported 9906_21_SPOCK3_TICN3
SPOCK3 chr4 167944432 0.037077 2.518e-80 35359 TRUE reported
textfile 2 1 TRUE
2131 rs2556099 T C T C -0.0478 -0.0018 0.49007 NA FALSE FALSE FALSE
GCST006061 0.0071 0.806 537409 outcome TRUE reported 9906_21_SPOCK3_TICN3
SPOCK3 chr2 159830857 0.007969 1.996e-09 35348 TRUE reported
textfile 2 1 TRUE
2132 rs34924625 G A G A 0.1776 0.0035 0.01967 NA FALSE FALSE FALSE
GCST006061 0.0378 0.9265 537409 outcome TRUE reported 9906_21_SPOCK3_TICN3
SPOCK3 chr16 69432734 0.028536 4.852e-10 35374 TRUE reported
textfile 2 1 TRUE
2133 rs67647638 A G A G -0.0823 -0.0055 0.07903 NA FALSE FALSE FALSE
GCST006061 0.0112 0.6253 537409 outcome TRUE reported 9906_21_SPOCK3_TICN3
SPOCK3 chr7 149731157 0.014889 3.249e-08 35372 TRUE reported
textfile 2 1 TRUE
2134 rs6834264 G A G A 0.2222 0.0197 0.04441 NA FALSE FALSE FALSE
GCST006061 0.0187 0.2928 537409 outcome TRUE reported 9906_21_SPOCK3_TICN3
SPOCK3 chr4 166165565 0.019712 1.792e-29 35363 TRUE reported

textfile 2 1 TRUE
2135 rs6902010 G T G T -0.0489 -0.0095 0.48116 NA FALSE FALSE FALSE GCST006061 0.0074 0.1971 537409 outcome TRUE reported 9906_21_SPOCK3_TICN3 SPOCK3 chr6 107125336 0.008013 1.046e-09 35371 TRUE reported textfile 2 1 TRUE
2136 rs7030887 T C T C -0.0504 -0.0038 0.35329 NA FALSE FALSE FALSE GCST006061 0.0075 0.612 537409 outcome TRUE reported 9906_21_SPOCK3_TICN3 SPOCK3 chr9 97926229 0.00828 1.149e-09 35345 TRUE reported textfile 2 1 TRUE
2137 rs704 A G A G -0.0771 -0.0155 0.49189 NA FALSE FALSE FALSE GCST006061 0.0072 0.03144 537409 outcome TRUE reported 9906_21_SPOCK3_TICN3 SPOCK3 chr17 28367840 0.00795 3.086e-22 35360 TRUE reported textfile 2 1 TRUE
2138 rs75897620 A G A G 0.3922 -0.0206 0.0443 NA FALSE FALSE FALSE GCST006061 0.0178 0.2491 537409 outcome TRUE reported 9906_21_SPOCK3_TICN3 SPOCK3 chr4 166735672 0.019733 6.592e-88 35364 TRUE reported textfile 2 1 TRUE
2139 rs77904897 C A C A -0.2083 -0.0483 0.01591 NA FALSE FALSE FALSE GCST006061 0.0328 0.1405 537409 outcome TRUE reported 9906_21_SPOCK3_TICN3 SPOCK3 chr4 167187687 0.032449 1.369e-10 35363 TRUE reported textfile 2 1 TRUE
2140 rs10243942 T C T C -0.0716 0.0013 0.45164 NA FALSE FALSE FALSE GCST006061 0.0075 0.8601 537409 outcome TRUE reported 5078_82_EPHB6_EphB6 EPHB6 chr7 142426465 0.008308 6.819e-18 35368 TRUE reported textfile 2 1 TRUE
2141 rs116887927 G A G A 0.2209 -0.0436 0.02132 NA FALSE FALSE FALSE GCST006061 0.0376 0.2459 537409 outcome TRUE reported 5078_82_EPHB6_EphB6 EPHB6 chr7 142701560 0.029022 2.711e-14 35373 TRUE reported textfile 2 1 TRUE
2142 rs13108218 G A G A 0.0504 -0.01 0.36204 NA FALSE FALSE FALSE GCST006061 0.0074 0.1774 537409 outcome TRUE reported 5078_82_EPHB6_EphB6 EPHB6 chr4 3442204 0.008479 2.774e-09 35280 TRUE reported textfile 2 1 TRUE
2143 rs145312664 A G A G 0.1841 -0.0433 0.02056 NA FALSE FALSE FALSE GCST006061 0.0353 0.2205 537409 outcome TRUE reported 5078_82_EPHB6_EphB6 EPHB6 chr7 140648359 0.028517 1.076e-10 35374 TRUE reported textfile 2 1 TRUE
2144 rs61747728 T C T C 0.1867 0.0236 0.04262 NA FALSE FALSE FALSE GCST006061 0.0209 0.2605 537409 outcome TRUE reported 5078_82_EPHB6_EphB6 EPHB6 chr1 179557079 0.020114 1.659e-20 35358 TRUE reported textfile 2 1 TRUE
2145 rs77924615 A G A G -0.0798 0.0136 0.19701 NA FALSE FALSE FALSE GCST006061 0.0092 0.1375 537409 outcome TRUE reported 5078_82_EPHB6_EphB6 EPHB6 chr16 20381010 0.010342 1.198e-14 35355 TRUE reported textfile 2 1 TRUE
2146 rs8177107 G A G A -0.2683 0.0141 0.17522 NA FALSE FALSE FALSE GCST006061 0.0098 0.1519 537409 outcome TRUE reported 5078_82_EPHB6_EphB6 EPHB6 chr7 142856383 0.010845 4.059e-135 35374 TRUE reported textfile 2 1 TRUE
2147 rs10250602 C T C T -0.2351 -0.0092 0.32919 NA FALSE FALSE FALSE GCST006061 0.0075 0.2164 537409 outcome TRUE reported 8289_8_GPNMB_GPNMB GPNMB chr7 23264082 0.008794 1.96e-157 35371 TRUE reported textfile 2 1 TRUE
2148 rs12203592 T C T C -0.0768 -0.0061 0.11862 NA FALSE FALSE FALSE GCST006061 0.0109 0.5753 537409 outcome TRUE reported 8289_8_GPNMB_GPNMB GPNMB chr6 396321 0.012738 1.647e-09 35317 TRUE reported textfile 2 1 TRUE
2149 rs12350739 A G A G -0.0481 0.0308 0.39544 NA FALSE FALSE FALSE GCST006061 0.0076 5.192e-05 537409 outcome TRUE reported 8289_8_GPNMB_GPNMB GPNMB chr9 16885019 0.008352 8.469e-09 35364 TRUE reported textfile 2 1 TRUE
2150 rs140966178 A G A G -0.3481 0.0052 0.01332 NA FALSE FALSE FALSE GCST006061 0.0386 0.8934 537409 outcome TRUE reported 8289_8_GPNMB_GPNMB GPNMB chr7 22500268 0.036696 2.401e-21 35370 TRUE reported textfile 2 1 TRUE
2151 rs2737212 T C T C -0.0557 8e-04 0.43699 NA FALSE FALSE FALSE

GCST006061 0.0073 0.9129 537409 outcome TRUE reported 8289_8_GPNMB_GPNMB
GPNMB chr8 115608987 0.008317 2.123e-11 35347 TRUE reported
textfile 2 1 TRUE
2152 rs56344867 C T C T 0.0772 -0.005 0.35465 NA FALSE FALSE FALSE
GCST006061 0.008 0.5298 537409 outcome TRUE reported 8289_8_GPNMB_GPNMB
GPNMB chr11 126361219 0.008615 3.223e-19 35374 TRUE reported
textfile 2 1 TRUE
2153 rs702814 T C T C -0.0644 0.0027 0.4774 NA FALSE FALSE FALSE
GCST006061 0.0074 0.7116 537409 outcome TRUE reported 8289_8_GPNMB_GPNMB
GPNMB chr7 28133113 0.008339 1.14e-14 35374 TRUE reported
textfile 2 1 TRUE
2154 rs75988976 A G A G -0.1012 -0.021 0.0902 NA FALSE FALSE FALSE
GCST006061 0.0117 0.07395 537409 outcome TRUE reported 8289_8_GPNMB_GPNMB
GPNMB chr7 23893439 0.014558 3.61e-12 35370 TRUE reported
textfile 2 1 TRUE
2155 rs77262773 T C T C 0.152 0.014 0.02263 NA FALSE FALSE FALSE
GCST006061 0.0283 0.6205 537409 outcome TRUE reported 8289_8_GPNMB_GPNMB
GPNMB chr17 69253570 0.027357 2.757e-08 35371 TRUE reported
textfile 2 1 TRUE
2156 rs10253736 T C T C 0.0507 -0.0035 0.26815 NA FALSE FALSE FALSE
GCST006061 0.0082 0.6665 537409 outcome TRUE reported 2609_59_CST3_Cystatin_C
CST3 chr7 151718170 0.009278 4.642e-08 35372 TRUE reported
textfile 2 1 TRUE
2157 rs73102389 A C A C 0.1148 -0.0021 0.15734 NA FALSE FALSE FALSE
GCST006061 0.0097 0.8268 537409 outcome TRUE reported 2609_59_CST3_Cystatin_C
CST3 chr20 23655054 0.011335 4.151e-24 35363 TRUE reported
textfile 2 1 TRUE
2158 rs77924615 A G A G -0.07 0.0136 0.19701 NA FALSE FALSE FALSE
GCST006061 0.0092 0.1375 537409 outcome TRUE reported 2609_59_CST3_Cystatin_C
CST3 chr16 20381010 0.010434 1.959e-11 35355 TRUE reported
textfile 2 1 TRUE
2159 rs9992101 A G A G 0.0485 0.0224 0.39832 NA FALSE FALSE FALSE
GCST006061 0.0075 0.00273 537409 outcome TRUE reported 2609_59_CST3_Cystatin_C
CST3 chr4 76439278 0.008406 7.932e-09 35356 TRUE reported
textfile 2 1 TRUE
2160 rs10253736 T C T C 0.0528 -0.0035 0.26815 NA FALSE FALSE FALSE
GCST006061 0.0082 0.6665 537409 outcome TRUE reported 8974_172_COL15A1_COFA1
COL15A1 chr7 151718170 0.009364 1.712e-08 35372 TRUE reported
textfile 2 1 TRUE
2161 rs1031755 C A C A -0.0583 -0.0073 0.22276 NA FALSE FALSE FALSE
GCST006061 0.0085 0.39 537409 outcome TRUE reported 8974_172_COL15A1_COFA1
COL15A1 chr15 53659238 0.009955 4.736e-09 35362 TRUE reported
textfile 2 1 TRUE
2162 rs1360098 G A G A 0.058 0.0015 0.46244 NA FALSE FALSE FALSE
GCST006061 0.0071 0.8329 537409 outcome TRUE reported 8974_172_COL15A1_COFA1
COL15A1 chr9 101113822 0.0084 5.033e-12 35342 TRUE reported textfile
2 1 TRUE
2163 rs16918163 G A G A 0.2481 -0.0243 0.09172 NA FALSE FALSE FALSE
GCST006061 0.0106 0.02215 537409 outcome TRUE reported 8974_172_COL15A1_COFA1
COL15A1 chr9 99051270 0.014367 8.142e-67 35343 TRUE reported
textfile 2 1 TRUE
2164 rs56997979 A G A G 0.0662 -0.0058 0.17075 NA FALSE FALSE FALSE
GCST006061 0.0098 0.5514 537409 outcome TRUE reported 8974_172_COL15A1_COFA1
COL15A1 chr9 98978115 0.011187 3.27e-09 35343 TRUE reported
textfile 2 1 TRUE
2165 rs77924615 A G A G -0.0831 0.0136 0.19701 NA FALSE FALSE FALSE
GCST006061 0.0092 0.1375 537409 outcome TRUE reported 8974_172_COL15A1_COFA1
COL15A1 chr16 20381010 0.010548 3.327e-15 35355 TRUE reported
textfile 2 1 TRUE
2166 rs9992101 A G A G 0.0509 0.0224 0.39832 NA FALSE FALSE FALSE
GCST006061 0.0075 0.00273 537409 outcome TRUE reported 8974_172_COL15A1_COFA1
COL15A1 chr4 76439278 0.008498 2.101e-09 35356 TRUE reported
textfile 2 1 TRUE
2167 rs10254101 T C T C 0.052 -0.0046 0.26853 NA FALSE FALSE FALSE
GCST006061 0.0082 0.5755 537409 outcome TRUE reported 10702_1_COL28A1_COSA1
COL28A1 chr7 151718450 0.009193 1.546e-08 35360 TRUE reported

textfile 2 1 TRUE
2168 rs12126578 C A C A -0.0449 0.0092 0.48174 NA FALSE FALSE FALSE
GCST006061 0.0076 0.2261 537409 outcome TRUE reported 10702_1_COL28A1_COSA1
COL28A1 chr1 106947932 0.008231 4.9e-08 35337 TRUE reported textfile
2 1 TRUE
2169 rs3088026 T C T C -0.0945 -0.0111 0.09272 NA FALSE FALSE FALSE
GCST006061 0.0131 0.3954 537409 outcome TRUE reported 10702_1_COL28A1_COSA1
COL28A1 chr21 46129699 0.01416 2.492e-11 34723 TRUE reported textfile
2 1 TRUE
2170 rs611462 A G A G -0.1357 0.0525 0.02813 NA FALSE FALSE FALSE
GCST006061 0.021 0.01235 537409 outcome TRUE reported 10702_1_COL28A1_COSA1
COL28A1 chr21 44821531 0.024663 3.752e-08 35248 TRUE reported
textfile 2 1 TRUE
2171 rs10254413 C T C T -0.0708 -0.0178 0.16682 NA FALSE FALSE FALSE
GCST006061 0.012 0.1358 537409 outcome TRUE reported 2571_12_IGFBP3_IGFBP_3
IGFBP3 chr7 46315964 0.011056 1.515e-10 35347 TRUE reported
textfile 2 1 TRUE
2172 rs11042543 G A G A 0.0488 -0.0057 0.28947 NA FALSE FALSE FALSE
GCST006061 0.0082 0.4913 537409 outcome TRUE reported 2571_12_IGFBP3_IGFBP_3
IGFBP3 chr11 9918271 0.008931 4.66e-08 35372 TRUE reported textfile
2 1 TRUE
2173 rs113339946 T C T C -0.1261 -0.0249 0.06432 NA FALSE FALSE FALSE
GCST006061 0.0153 0.1034 537409 outcome TRUE reported 2571_12_IGFBP3_IGFBP_3
IGFBP3 chr16 1428318 0.016862 7.525e-14 35317 TRUE reported textfile
2 1 TRUE
2174 rs117651825 A G A G -0.1113 -0.0211 0.05971 NA FALSE FALSE FALSE
GCST006061 0.0249 0.3974 537409 outcome TRUE reported 2571_12_IGFBP3_IGFBP_3
IGFBP3 chr16 2256612 0.016862 4.099e-11 35318 TRUE reported textfile
2 1 TRUE
2175 rs12540611 C A C A -0.1312 -0.0125 0.04024 NA FALSE FALSE FALSE
GCST006061 0.0194 0.519 537409 outcome TRUE reported 2571_12_IGFBP3_IGFBP_3
IGFBP3 chr7 43345424 0.020411 1.295e-10 35354 TRUE reported
textfile 2 1 TRUE
2176 rs139482112 C T C T -0.4745 0.0833 0.01509 NA FALSE FALSE FALSE
GCST006061 0.044 0.05833 537409 outcome TRUE reported 2571_12_IGFBP3_IGFBP_3
IGFBP3 chr16 2023892 0.033686 4.625e-45 35319 TRUE reported textfile
2 1 TRUE
2177 rs143008659 T G T G -0.2136 0.0125 0.02269 NA FALSE FALSE FALSE
GCST006061 0.0368 0.7341 537409 outcome TRUE reported 2571_12_IGFBP3_IGFBP_3
IGFBP3 chr16 1792939 0.028178 3.443e-14 35321 TRUE reported textfile
2 1 TRUE
2178 rs144318789 T C T C -0.1089 -0.0558 0.08643 NA FALSE FALSE FALSE
GCST006061 0.0261 0.03274 537409 outcome TRUE reported 2571_12_IGFBP3_IGFBP_3
IGFBP3 chr7 46142286 0.014725 1.408e-13 35348 TRUE reported
textfile 2 1 TRUE
2179 rs145188037 A G A G 1.0281 0.0188 0.0176 NA FALSE FALSE FALSE
GCST006061 0.0387 0.6272 537409 outcome TRUE reported 2571_12_IGFBP3_IGFBP_3
IGFBP3 chr7 45914866 0.031093 1e-200 35347 TRUE reported textfile
2 1 TRUE
2180 rs145410566 A G A G -0.125 0.0102 0.03287 NA FALSE FALSE FALSE
GCST006061 0.0254 0.6871 537409 outcome TRUE reported 2571_12_IGFBP3_IGFBP_3
IGFBP3 chr16 3722303 0.022575 3.075e-08 35353 TRUE reported textfile
2 1 TRUE
2181 rs147400256 A C A C -0.2842 0.0183 0.0192 NA FALSE FALSE FALSE
GCST006061 0.0327 0.5755 537409 outcome TRUE reported 2571_12_IGFBP3_IGFBP_3
IGFBP3 chr16 1892181 0.028785 5.432e-23 35321 TRUE reported textfile
2 1 TRUE
2182 rs4234797 A G A G 0.0683 0.0023 0.44184 NA FALSE FALSE FALSE
GCST006061 0.0073 0.7558 537409 outcome TRUE reported 2571_12_IGFBP3_IGFBP_3
IGFBP3 chr4 7218180 0.008194 7.702e-17 35379 TRUE reported textfile
2 1 TRUE
2183 rs55932586 T C T C -0.1035 -0.017 0.33605 NA FALSE FALSE FALSE
GCST006061 0.0079 0.03144 537409 outcome TRUE reported 2571_12_IGFBP3_IGFBP_3
IGFBP3 chr7 45792878 0.008701 1.261e-32 35347 TRUE reported
textfile 2 1 TRUE
2184 rs77542162 G A G A 0.152 0.0177 0.025 NA FALSE FALSE FALSE

GCST006061 0.0302 0.557 537409 outcome TRUE reported 2571_12_IGFBP3_IGFBP_3 IGFBP3 chr17 69085137 0.025634 3.036e-09 35371 TRUE reported textfile 2 1 TRUE
2185 rs77589580 G T G T -0.3835 -0.0921 0.0116 NA FALSE FALSE FALSE GCST006061 0.0345 0.007576 537409 outcome TRUE reported 2571_12_IGFBP3_IGFBP_3 IGFBP3 chr16 3700087 0.0379 4.568e-24 35350 TRUE reported textfile 2 1 TRUE
2186 rs10777719 C T C T 0.0795 -0.0142 0.29946 NA FALSE FALSE FALSE GCST006061 0.0077 0.06494 537409 outcome TRUE reported 3327_27_NTN4_NET4 NTN4 chr12 95687308 0.009135 3.228e-18 35362 TRUE reported textfile 2 1 TRUE
2187 rs11612228 T C T C -0.0574 -0.0162 0.3782 NA FALSE FALSE FALSE GCST006061 0.0079 0.04029 537409 outcome TRUE reported 3327_27_NTN4_NET4 NTN4 chr12 467818 0.008613 2.654e-11 35311 TRUE reported textfile 2 1 TRUE
2188 rs17288108 G A G A -0.2526 1e-04 0.14241 NA FALSE FALSE FALSE GCST006061 0.0099 0.9914 537409 outcome TRUE reported 3327_27_NTN4_NET4 NTN4 chr12 95738117 0.011818 2.349e-101 35364 TRUE reported textfile 2 1 TRUE
2189 rs1800127 T C T C 0.1493 0.0335 0.02781 NA FALSE FALSE FALSE GCST006061 0.0283 0.237 537409 outcome TRUE reported 3327_27_NTN4_NET4 NTN4 chr12 57145299 0.02574 6.618e-09 35365 TRUE reported textfile 2 1 TRUE
2190 rs2660888 G A G A 0.0605 -0.0032 0.48797 NA FALSE FALSE FALSE GCST006061 0.0071 0.651 537409 outcome TRUE reported 3327_27_NTN4_NET4 NTN4 chr12 96057909 0.008369 4.86e-13 35361 TRUE reported textfile 2 1 TRUE
2191 rs6121571 G T G T 0.0884 0.0078 0.26947 NA FALSE FALSE FALSE GCST006061 0.0081 0.3363 537409 outcome TRUE reported 3327_27_NTN4_NET4 NTN4 chr20 62402267 0.009431 7.044e-21 35335 TRUE reported textfile 2 1 TRUE
2192 rs71352239 T C T C 0.0528 -0.0041 0.30263 NA FALSE FALSE FALSE GCST006061 0.0087 0.6372 537409 outcome TRUE reported 3327_27_NTN4_NET4 NTN4 chr19 44926286 0.009082 6.114e-09 35368 TRUE reported textfile 2 1 TRUE
2193 rs77542162 G A G A 0.2091 0.0177 0.025 NA FALSE FALSE FALSE GCST006061 0.0302 0.557 537409 outcome TRUE reported 3327_27_NTN4_NET4 NTN4 chr17 69085137 0.026223 1.538e-15 35371 TRUE reported textfile 2 1 TRUE
2194 rs1025647 C T C T -0.0557 0.0018 0.17559 NA FALSE FALSE FALSE GCST006061 0.0097 0.8529 537409 outcome TRUE reported 7145_1_ITIH3_ITIH3 ITIH3 chr1 216980584 0.010136 3.905e-08 35355 TRUE reported textfile 2 1 TRUE
2195 rs1070073 G T G T 0.0504 0.0043 0.32569 NA FALSE FALSE FALSE GCST006061 0.0078 0.5805 537409 outcome TRUE reported 7145_1_ITIH3_ITIH3 ITIH3 chr12 103606541 0.008135 5.806e-10 35366 TRUE reported textfile 2 1 TRUE
2196 rs10922098 T C T C -0.0431 -0.009 0.40011 NA FALSE FALSE FALSE GCST006061 0.0075 0.2275 537409 outcome TRUE reported 7145_1_ITIH3_ITIH3 ITIH3 chr1 196695521 0.007822 3.584e-08 35338 TRUE reported textfile 2 1 TRUE
2197 rs111989250 A G A G -0.3358 0.011 0.02177 NA FALSE FALSE FALSE GCST006061 0.0416 0.7907 537409 outcome TRUE reported 7145_1_ITIH3_ITIH3 ITIH3 chr3 54733249 0.026939 1.158e-35 35349 TRUE reported textfile 2 1 TRUE
2198 rs112875651 A G A G 0.0606 -0.0016 0.40253 NA FALSE FALSE FALSE GCST006061 0.0076 0.8301 537409 outcome TRUE reported 7145_1_ITIH3_ITIH3 ITIH3 chr8 125494452 0.007861 1.273e-14 35367 TRUE reported textfile 2 1 TRUE
2199 rs142044921 T C T C 0.1352 0.0043 0.06921 NA FALSE FALSE FALSE GCST006061 0.02 0.8298 537409 outcome TRUE reported 7145_1_ITIH3_ITIH3 ITIH3 chr3 49134297 0.01578 1.055e-17 35344 TRUE reported textfile 2 1 TRUE
2200 rs142131136 T C T C -0.1893 -0.0232 0.02221 NA FALSE FALSE FALSE GCST006061 0.0436 0.5948 537409 outcome TRUE reported 7145_1_ITIH3_ITIH3 ITIH3 chr3 42431036 0.027265 3.837e-12 35351 TRUE reported

textfile 2 1 TRUE
2201 rs1495741 A G A G 0.063 0.015 0.18772 NA FALSE FALSE FALSE
GCST006061 0.0083 0.06968 537409 outcome TRUE reported 7145_1_ITIH3_ITIH3
ITIH3 chr8 18415371 0.009897 1.949e-10 35377 TRUE reported
textfile 2 1 TRUE
2202 rs35142429 A G A G -0.2674 -0.0118 0.02173 NA FALSE FALSE FALSE
GCST006061 0.0342 0.7299 537409 outcome TRUE reported 7145_1_ITIH3_ITIH3
ITIH3 chr3 44959134 0.027559 2.931e-22 35347 TRUE reported
textfile 2 1 TRUE
2203 rs4981022 A G A G 0.0622 -5.00E-04 0.3206 NA FALSE FALSE FALSE
GCST006061 0.0078 0.9508 537409 outcome TRUE reported 7145_1_ITIH3_ITIH3
ITIH3 chr12 103756096 0.008189 3.055e-14 35361 TRUE reported
textfile 2 1 TRUE
2204 rs62246138 A G A G -0.2353 -0.0159 0.03736 NA FALSE FALSE FALSE
GCST006061 0.0219 0.4682 537409 outcome TRUE reported 7145_1_ITIH3_ITIH3
ITIH3 chr3 46412462 0.021166 1.04e-28 35342 TRUE reported
textfile 2 1 TRUE
2205 rs6802320 C T C T 0.2885 0.0043 0.37345 NA FALSE FALSE FALSE
GCST006061 0.0075 0.5634 537409 outcome TRUE reported 7145_1_ITIH3_ITIH3
ITIH3 chr3 52207819 0.00815 1e-200 35347 TRUE reported textfile 2
1 TRUE
2206 rs74320783 A G A G -1.2672 0.0332 0.01686 NA FALSE FALSE FALSE
GCST006061 0.0425 0.4353 537409 outcome TRUE reported 7145_1_ITIH3_ITIH3
ITIH3 chr3 52802517 0.023293 1e-200 35354 TRUE reported textfile
2 1 TRUE
2207 rs9841474 A G A G -0.0545 -0.0145 0.2332 NA FALSE FALSE FALSE
GCST006061 0.0092 0.1136 537409 outcome TRUE reported 7145_1_ITIH3_ITIH3
ITIH3 chr3 55172180 0.00952 1.035e-08 35352 TRUE reported textfile
2 1 TRUE
2208 rs1026566 A G A G 0.1322 0.0058 0.46037 NA FALSE FALSE FALSE
GCST006061 0.0072 0.42 537409 outcome TRUE reported 13109_82_NEGR1_NEGR1
NEGR1 chr1 72243359 0.008358 2.365e-56 35343 TRUE reported
textfile 2 1 TRUE
2209 rs10922098 T C T C -0.096 -0.009 0.40011 NA FALSE FALSE FALSE
GCST006061 0.0075 0.2275 537409 outcome TRUE reported 13109_82_NEGR1_NEGR1
NEGR1 chr1 196695521 0.008551 3.026e-29 35329 TRUE reported
textfile 2 1 TRUE
2210 rs12280645 G A G A 0.0591 -0.0059 0.33798 NA FALSE FALSE FALSE
GCST006061 0.0079 0.4507 537409 outcome TRUE reported 13109_82_NEGR1_NEGR1
NEGR1 chr11 126393990 0.008815 2.016e-11 35365 TRUE reported
textfile 2 1 TRUE
2211 rs17145750 T C T C 0.0644 -0.0143 0.15023 NA FALSE FALSE FALSE
GCST006061 0.01 0.1504 537409 outcome TRUE reported 13109_82_NEGR1_NEGR1
NEGR1 chr7 73612048 0.011683 3.543e-08 35345 TRUE reported
textfile 2 1 TRUE
2212 rs2686395 C T C T -0.0688 -0.0077 0.3101 NA FALSE FALSE FALSE
GCST006061 0.0074 0.2965 537409 outcome TRUE reported 13109_82_NEGR1_NEGR1
NEGR1 chr3 165767231 0.009013 2.28e-14 35330 TRUE reported
textfile 2 1 TRUE
2213 rs72704449 C T C T 0.1328 0.013 0.04582 NA FALSE FALSE FALSE
GCST006061 0.0199 0.5128 537409 outcome TRUE reported 13109_82_NEGR1_NEGR1
NEGR1 chr1 179504138 0.019944 2.763e-11 35349 TRUE reported
textfile 2 1 TRUE
2214 rs77924615 A G A G -0.0607 0.0136 0.19701 NA FALSE FALSE FALSE
GCST006061 0.0092 0.1375 537409 outcome TRUE reported 13109_82_NEGR1_NEGR1
NEGR1 chr16 20381010 0.010591 9.96e-09 35346 TRUE reported
textfile 2 1 TRUE
2215 rs780094 C T C T 0.0611 -0.0076 0.33917 NA FALSE FALSE FALSE
GCST006061 0.0073 0.2951 537409 outcome TRUE reported 13109_82_NEGR1_NEGR1
NEGR1 chr2 27518370 0.00885 5.049e-12 35364 TRUE reported textfile
2 1 TRUE
2216 rs10268683 G A G A 0.0485 0.0027 0.43523 NA FALSE FALSE FALSE
GCST006061 0.0073 0.7131 537409 outcome TRUE reported
15566_10_CNN1_Calponin_1 CNN1 chr7 5511307 0.008468 1.02e-08 35373
TRUE reported textfile 2 1 TRUE
2217 rs1047891 A C A C 0.093 0.0155 0.26363 NA FALSE FALSE FALSE

GCST006061 0.0079 0.05042 537409 outcome TRUE reported
15566_10_CNN1_Calponin_1 CNN1 chr2 210675783 0.00949 1.131e-22 35353
TRUE reported textfile 2 1 TRUE
2218 rs10922098 T C T C -0.0612 -0.009 0.40011 NA FALSE FALSE FALSE
GCST006061 0.0075 0.2275 537409 outcome TRUE reported
15566_10_CNN1_Calponin_1 CNN1 chr1 196695521 0.008519 6.758e-13 35338
TRUE reported textfile 2 1 TRUE
2219 rs114694170 C T C T 0.1324 0.0053 0.08378 NA FALSE FALSE FALSE
GCST006061 0.0175 0.7624 537409 outcome TRUE reported
15566_10_CNN1_Calponin_1 CNN1 chr5 88884379 0.015113 1.937e-18 35344
TRUE reported textfile 2 1 TRUE
2220 rs11553699 G A G A -0.1326 -0.0209 0.14277 NA FALSE FALSE FALSE
GCST006061 0.012 0.08291 537409 outcome TRUE reported
15566_10_CNN1_Calponin_1 CNN1 chr12 121779004 0.011911 8.7e-29 35366
TRUE reported textfile 2 1 TRUE
2221 rs12445547 T G T G 0.0581 -0.0218 0.27767 NA FALSE FALSE FALSE
GCST006061 0.0077 0.004718 537409 outcome TRUE reported
15566_10_CNN1_Calponin_1 CNN1 chr16 88452161 0.009349 5.136e-10 35320
TRUE reported textfile 2 1 TRUE
2222 rs12767683 A C A C -0.1091 0.0038 0.26443 NA FALSE FALSE FALSE
GCST006061 0.0079 0.6284 537409 outcome TRUE reported
15566_10_CNN1_Calponin_1 CNN1 chr10 102556824 0.009491 1.401e-30 35370
TRUE reported textfile 2 1 TRUE
2223 rs1769758 T G T G 0.0756 -0.031 0.45567 NA FALSE FALSE FALSE
GCST006061 0.0082 0.0001497 537409 outcome TRUE reported
15566_10_CNN1_Calponin_1 CNN1 chr10 79139212 0.008369 1.669e-19 35363
TRUE reported textfile 2 1 TRUE
2224 rs342296 A G A G -0.1182 -0.0017 0.45412 NA FALSE FALSE FALSE
GCST006061 0.0073 0.8216 537409 outcome TRUE reported
15566_10_CNN1_Calponin_1 CNN1 chr7 106732457 0.008322 8.64e-46 35357
TRUE reported textfile 2 1 TRUE
2225 rs4693545 G T G T -0.0897 0.0286 0.15993 NA FALSE FALSE FALSE
GCST006061 0.0093 0.002024 537409 outcome TRUE reported
15566_10_CNN1_Calponin_1 CNN1 chr4 83034489 0.011338 2.551e-15 35355
TRUE reported textfile 2 1 TRUE
2226 rs4783187 C T C T 0.0699 -0.0124 0.12255 NA FALSE FALSE FALSE
GCST006061 0.0108 0.2492 537409 outcome TRUE reported
15566_10_CNN1_Calponin_1 CNN1 chr16 85382232 0.012693 3.655e-08 35368
TRUE reported textfile 2 1 TRUE
2227 rs5750957 T C T C -0.0575 -0.01 0.40972 NA FALSE FALSE FALSE
GCST006061 0.0074 0.1793 537409 outcome TRUE reported
15566_10_CNN1_Calponin_1 CNN1 chr22 40539289 0.008425 8.804e-12 35370
TRUE reported textfile 2 1 TRUE
2228 rs62191340 A G A G -0.0959 0.0194 0.4806 NA FALSE FALSE FALSE
GCST006061 0.008 0.01565 537409 outcome TRUE reported
15566_10_CNN1_Calponin_1 CNN1 chr2 241706051 0.008381 2.556e-30 35115
TRUE reported textfile 2 1 TRUE
2229 rs680478 T C T C -0.0587 -0.0127 0.19849 NA FALSE FALSE FALSE
GCST006061 0.0087 0.1464 537409 outcome TRUE reported
15566_10_CNN1_Calponin_1 CNN1 chr18 9609959 0.010446 1.918e-08 35369
TRUE reported textfile 2 1 TRUE
2230 rs72716646 A G A G 0.1177 8e-04 0.04336 NA FALSE FALSE FALSE
GCST006061 0.0176 0.9629 537409 outcome TRUE reported
15566_10_CNN1_Calponin_1 CNN1 chr14 55078104 0.020622 1.146e-08 35359
TRUE reported textfile 2 1 TRUE
2231 rs73000929 A G A G 0.1622 0.0131 0.04815 NA FALSE FALSE FALSE
GCST006061 0.0245 0.593 537409 outcome TRUE reported
15566_10_CNN1_Calponin_1 CNN1 chr11 114082900 0.019432 7.011e-17 35365
TRUE reported textfile 2 1 TRUE
2232 rs10270210 C T C T 0.056 0.0042 0.41284 NA FALSE FALSE FALSE
GCST006061 0.0074 0.5686 537409 outcome TRUE reported 8080_24_MSMP_PSMP
MSMP chr7 18880587 0.008443 3.301e-11 35376 TRUE reported
textfile 2 1 TRUE
2233 rs10758322 T C T C 0.0624 -0.0082 0.41831 NA FALSE FALSE FALSE
GCST006061 0.0073 0.263 537409 outcome TRUE reported 8080_24_MSMP_PSMP
MSMP chr9 35776193 0.008408 1.156e-13 35350 TRUE reported

textfile 2 1 TRUE
2234 rs1598307 T C T C 0.1307 -0.0021 0.25092 NA FALSE FALSE FALSE
GCST006061 0.0085 0.8004 537409 outcome TRUE reported 8080_24_MSMP_PSMP
MSMP chr9 3837797 0.00954 1.004e-42 35372 TRUE reported textfile 2
1 TRUE
2235 rs2130499 T G T G -0.0536 -0.008 0.41395 NA FALSE FALSE FALSE
GCST006061 0.0074 0.2809 537409 outcome TRUE reported 8080_24_MSMP_PSMP
MSMP chr4 144467682 0.008445 2.2e-10 35360 TRUE reported textfile
2 1 TRUE
2236 rs34125642 A G A G -0.0508 -0.0172 0.38045 NA FALSE FALSE FALSE
GCST006061 0.0078 0.02839 537409 outcome TRUE reported 8080_24_MSMP_PSMP
MSMP chr18 49075002 0.008538 2.679e-09 35366 TRUE reported
textfile 2 1 TRUE
2237 rs4740734 A G A G -0.0903 0.017 0.11033 NA FALSE FALSE FALSE
GCST006061 0.011 0.1232 537409 outcome TRUE reported 8080_24_MSMP_PSMP
MSMP chr9 3761548 0.013327 1.24e-11 35372 TRUE reported textfile
2 1 TRUE
2238 rs515380 G A G A -0.1184 -0.0102 0.37736 NA FALSE FALSE FALSE
GCST006061 0.0072 0.1553 537409 outcome TRUE reported 8080_24_MSMP_PSMP
MSMP chr9 4036449 0.008561 1.676e-43 35373 TRUE reported textfile
2 1 TRUE
2239 rs59345518 G A G A 0.0487 0.0117 0.40493 NA FALSE FALSE FALSE
GCST006061 0.0075 0.1206 537409 outcome TRUE reported 8080_24_MSMP_PSMP
MSMP chr19 47821016 0.008443 8.006e-09 35373 TRUE reported
textfile 2 1 TRUE
2240 rs6547787 G T G T -0.0695 -0.0096 0.30628 NA FALSE FALSE FALSE
GCST006061 0.0075 0.2017 537409 outcome TRUE reported 8080_24_MSMP_PSMP
MSMP chr2 88630218 0.009028 1.375e-14 35354 TRUE reported
textfile 2 1 TRUE
2241 rs72979233 G A G A 0.1453 0.0045 0.25638 NA FALSE FALSE FALSE
GCST006061 0.0084 0.5944 537409 outcome TRUE reported 8080_24_MSMP_PSMP
MSMP chr11 74644478 0.009511 1.094e-52 35370 TRUE reported
textfile 2 1 TRUE
2242 rs78862061 G A G A 0.076 0.019 0.19016 NA FALSE FALSE FALSE
GCST006061 0.0107 0.07653 537409 outcome TRUE reported 8080_24_MSMP_PSMP
MSMP chr3 186009040 0.010504 4.64e-13 35356 TRUE reported
textfile 2 1 TRUE
2243 rs9316871 G A G A 0.0797 0.0076 0.19195 NA FALSE FALSE FALSE
GCST006061 0.0084 0.3633 537409 outcome TRUE reported 8080_24_MSMP_PSMP
MSMP chr13 22287782 0.010582 5.02e-14 35354 TRUE reported
textfile 2 1 TRUE
2244 rs10272589 A G A G 0.1225 -0.0076 0.12378 NA FALSE FALSE FALSE
GCST006061 0.0101 0.4474 537409 outcome TRUE reported 9337_43_TAC1_TKN1
TAC1 chr7 97267327 0.012393 4.842e-23 35354 TRUE reported
textfile 2 1 TRUE
2245 rs1108109 C T C T 0.0495 0.0078 0.36525 NA FALSE FALSE FALSE
GCST006061 0.008 0.3285 537409 outcome TRUE reported 9337_43_TAC1_TKN1
TAC1 chr12 124371786 0.008518 6.214e-09 35364 TRUE reported
textfile 2 1 TRUE
2246 rs117081694 A G A G -0.1299 -0.0291 0.03132 NA FALSE FALSE FALSE
GCST006061 0.0236 0.217 537409 outcome TRUE reported 9337_43_TAC1_TKN1
TAC1 chr10 101097541 0.023698 4.216e-08 35366 TRUE reported
textfile 2 1 TRUE
2247 rs17168679 C T C T 0.3595 -0.0099 0.0139 NA FALSE FALSE FALSE
GCST006061 0.0229 0.6656 537409 outcome TRUE reported 9337_43_TAC1_TKN1
TAC1 chr7 97479442 0.034744 4.315e-25 35354 TRUE reported
textfile 2 1 TRUE
2248 rs17431071 G A G A -0.1205 0.0442 0.04078 NA FALSE FALSE FALSE
GCST006061 0.0278 0.1113 537409 outcome TRUE reported 9337_43_TAC1_TKN1
TAC1 chr7 121297272 0.020602 4.951e-09 35357 TRUE reported
textfile 2 1 TRUE
2249 rs3184504 C T C T -0.0706 0.0074 0.37862 NA FALSE FALSE FALSE
GCST006061 0.0075 0.3235 537409 outcome TRUE reported 9337_43_TAC1_TKN1
TAC1 chr12 111446804 0.008426 5.342e-17 35371 TRUE reported
textfile 2 1 TRUE
2250 rs3779470 A G A G -0.1996 0.0042 0.18313 NA FALSE FALSE FALSE

GCST006061 0.0093 0.6513 537409 outcome TRUE reported 9337_43_TAC1_TKN1
TAC1 chr7 97736619 0.010516 2.51e-80 35366 TRUE reported
textfile 2 1 TRUE
2251 rs4432538 A G A G 0.0752 0.0193 0.4912 NA FALSE FALSE FALSE
GCST006061 0.0072 0.007157 537409 outcome TRUE reported 9337_43_TAC1_TKN1
TAC1 chr20 8626746 0.00816 3.085e-20 35369 TRUE reported textfile 2
1 TRUE
2252 rs10272702 C T C T -0.1532 0.0024 0.08611 NA FALSE FALSE FALSE
GCST006061 0.0129 0.8532 537409 outcome TRUE reported 3431_54_EPHA1_EphA1
EPHA1 chr7 143261090 0.016815 8.164e-20 35371 TRUE reported
textfile 2 1 TRUE
2253 rs1181753 T C T C -0.0894 -0.0139 0.17327 NA FALSE FALSE FALSE
GCST006061 0.0091 0.1274 537409 outcome TRUE reported 3431_54_EPHA1_EphA1
EPHA1 chr7 141442332 0.012332 4.186e-13 35376 TRUE reported
textfile 2 1 TRUE
2254 rs146670385 C T C T -0.4286 -0.0425 0.01351 NA FALSE FALSE FALSE
GCST006061 0.0439 0.3329 537409 outcome TRUE reported 3431_54_EPHA1_EphA1
EPHA1 chr7 144748577 0.038765 2.045e-28 35375 TRUE reported
textfile 2 1 TRUE
2255 rs17286117 A C A C -0.1579 0.0013 0.18657 NA FALSE FALSE FALSE
GCST006061 0.0105 0.902 537409 outcome TRUE reported 3431_54_EPHA1_EphA1
EPHA1 chr7 144608426 0.011879 2.577e-40 35371 TRUE reported
textfile 2 1 TRUE
2256 rs2158751 T C T C 0.1789 0.0338 0.04129 NA FALSE FALSE FALSE
GCST006061 0.0181 0.06202 537409 outcome TRUE reported 3431_54_EPHA1_EphA1
EPHA1 chr7 145196970 0.023914 7.382e-14 35375 TRUE reported
textfile 2 1 TRUE
2257 rs73154244 A G A G -0.2075 0.0068 0.29348 NA FALSE FALSE FALSE
GCST006061 0.0079 0.3878 537409 outcome TRUE reported 3431_54_EPHA1_EphA1
EPHA1 chr7 143492801 0.010197 4.758e-92 35373 TRUE reported
textfile 2 1 TRUE
2258 rs10276126 A G A G -0.1216 -0.0097 0.14675 NA FALSE FALSE FALSE
GCST006061 0.0099 0.3265 537409 outcome TRUE reported 9267_2_CPA4_CBPA4
CPA4 chr7 131621451 0.011835 9.144e-25 35352 TRUE reported
textfile 2 1 TRUE
2259 rs1033063 T C T C -0.2309 -0.0054 0.01073 NA FALSE FALSE FALSE
GCST006061 0.051 0.9162 537409 outcome TRUE reported 9267_2_CPA4_CBPA4
CPA4 chr7 118687914 0.040152 8.888e-09 35349 TRUE reported
textfile 2 1 TRUE
2260 rs111576843 T C T C -0.2858 -0.0288 0.02684 NA FALSE FALSE FALSE
GCST006061 0.0226 0.2032 537409 outcome TRUE reported 9267_2_CPA4_CBPA4
CPA4 chr7 131050254 0.026319 1.809e-27 35355 TRUE reported
textfile 2 1 TRUE
2261 rs117925228 A C A C -0.2474 -0.0868 0.01155 NA FALSE FALSE FALSE
GCST006061 0.0706 0.2187 537409 outcome TRUE reported 9267_2_CPA4_CBPA4
CPA4 chr7 119053467 0.039395 3.386e-10 35352 TRUE reported
textfile 2 1 TRUE
2262 rs138970916 G A G A -0.1597 -0.0096 0.04524 NA FALSE FALSE FALSE
GCST006061 0.028 0.7316 537409 outcome TRUE reported 9267_2_CPA4_CBPA4
CPA4 chr7 124804560 0.019675 4.784e-16 35355 TRUE reported
textfile 2 1 TRUE
2263 rs145375923 A G A G -0.241 -0.0078 0.02166 NA FALSE FALSE FALSE
GCST006061 0.0338 0.8188 537409 outcome TRUE reported 9267_2_CPA4_CBPA4
CPA4 chr7 134337033 0.028373 1.998e-17 35356 TRUE reported
textfile 2 1 TRUE
2264 rs146499629 T C T C -0.2106 0.035 0.0219 NA FALSE FALSE FALSE
GCST006061 0.0405 0.3874 537409 outcome TRUE reported 9267_2_CPA4_CBPA4
CPA4 chr7 117251912 0.028608 1.817e-13 35341 TRUE reported
textfile 2 1 TRUE
2265 rs17583841 A G A G -0.1852 -0.028 0.01906 NA FALSE FALSE FALSE
GCST006061 0.0394 0.4781 537409 outcome TRUE reported 9267_2_CPA4_CBPA4
CPA4 chr7 114932526 0.029459 3.241e-10 35351 TRUE reported
textfile 2 1 TRUE
2266 rs192805484 A C A C 0.3548 0.0028 0.04181 NA FALSE FALSE FALSE
GCST006061 0.0346 0.9346 537409 outcome TRUE reported 9267_2_CPA4_CBPA4
CPA4 chr7 129628104 0.021014 5.869e-64 35352 TRUE reported

textfile 2 1 TRUE
2267 rs74632709 C T C T -0.1277 -0.0031 0.04825 NA FALSE FALSE FALSE
GCST006061 0.0192 0.8703 537409 outcome TRUE reported 9267_2_CPA4_CBPA4
CPA4 chr7 118131739 0.019552 6.522e-11 35346 TRUE reported
textfile 2 1 TRUE
2268 rs76284662 T C T C 0.1508 0.0068 0.0487 NA FALSE FALSE FALSE
GCST006061 0.0222 0.7587 537409 outcome TRUE reported 9267_2_CPA4_CBPA4
CPA4 chr7 128288722 0.019543 1.198e-14 35352 TRUE reported
textfile 2 1 TRUE
2269 rs78283599 G A G A -0.8486 0.0011 0.08054 NA FALSE FALSE FALSE
GCST006061 0.0157 0.9464 537409 outcome TRUE reported 9267_2_CPA4_CBPA4
CPA4 chr7 130293484 0.008554 1e-200 35354 TRUE reported textfile
2 1 TRUE
2270 rs78924983 T C T C -0.0892 0.0065 0.05372 NA FALSE FALSE FALSE
GCST006061 0.0193 0.7372 537409 outcome TRUE reported 9267_2_CPA4_CBPA4
CPA4 chr1 8144536 0.014018 1.975e-10 35384 TRUE reported textfile
2 1 TRUE
2271 rs9641869 G A G A 0.3797 0.0158 0.12836 NA FALSE FALSE FALSE
GCST006061 0.0095 0.09641 537409 outcome TRUE reported 9267_2_CPA4_CBPA4
CPA4 chr7 130276190 0.012427 1e-200 35357 TRUE reported textfile
2 1 TRUE
2272 rs10281146 G A G A 0.0739 0.0033 0.34684 NA FALSE FALSE FALSE
GCST006061 0.008 0.6741 537409 outcome TRUE reported 11360_39_RRM1_RRM1
RRM1 chr7 32995730 0.00844 2.029e-18 35333 TRUE reported textfile
2 1 TRUE
2273 rs11031142 T C T C -0.055 0.0134 0.43826 NA FALSE FALSE FALSE
GCST006061 0.0071 0.05864 537409 outcome TRUE reported 11360_39_RRM1_RRM1
RRM1 chr11 4153145 0.008113 1.205e-11 35366 TRUE reported textfile
2 1 TRUE
2274 rs11666771 A G A G -0.061 -0.0022 0.1666 NA FALSE FALSE FALSE
GCST006061 0.0105 0.8348 537409 outcome TRUE reported 11360_39_RRM1_RRM1
RRM1 chr19 4351659 0.01086 1.946e-08 35345 TRUE reported textfile 2
1 TRUE
2275 rs11900746 T G T G 0.1223 -0.001 0.06719 NA FALSE FALSE FALSE
GCST006061 0.0142 0.9434 537409 outcome TRUE reported 11360_39_RRM1_RRM1
RRM1 chr2 68448519 0.01609 2.941e-14 35352 TRUE reported textfile
2 1 TRUE
2276 rs17692518 A G A G 0.2511 0.0197 0.10813 NA FALSE FALSE FALSE
GCST006061 0.0151 0.1919 537409 outcome TRUE reported 11360_39_RRM1_RRM1
RRM1 chr22 32466867 0.013377 1.319e-78 35360 TRUE reported
textfile 2 1 TRUE
2277 rs182172833 T C T C -0.1644 -0.0146 0.02694 NA FALSE FALSE FALSE
GCST006061 0.0302 0.6286 537409 outcome TRUE reported 11360_39_RRM1_RRM1
RRM1 chr22 32227638 0.025178 6.595e-11 35358 TRUE reported
textfile 2 1 TRUE
2278 rs230508 G A G A 0.1306 0.021 0.34327 NA FALSE FALSE FALSE
GCST006061 0.0075 0.005168 537409 outcome TRUE reported 11360_39_RRM1_RRM1
RRM1 chr4 102558122 0.008404 1.876e-54 35334 TRUE reported
textfile 2 1 TRUE
2279 rs2425031 C T C T -0.0709 -0.0204 0.13576 NA FALSE FALSE FALSE
GCST006061 0.0104 0.05023 537409 outcome TRUE reported 11360_39_RRM1_RRM1
RRM1 chr20 35267481 0.01182 1.992e-09 35356 TRUE reported textfile
2 1 TRUE
2280 rs3811444 T C T C 0.081 0.0103 0.34232 NA FALSE FALSE FALSE
GCST006061 0.0077 0.1812 537409 outcome TRUE reported 11360_39_RRM1_RRM1
RRM1 chr1 247876149 0.008545 2.565e-21 35147 TRUE reported
textfile 2 1 TRUE
2281 rs58141407 T C T C -0.0746 0.0094 0.13145 NA FALSE FALSE FALSE
GCST006061 0.0099 0.3441 537409 outcome TRUE reported 11360_39_RRM1_RRM1
RRM1 chr8 21934261 0.011878 3.369e-10 35367 TRUE reported
textfile 2 1 TRUE
2282 rs6014993 G A G A 0.0531 0.0043 0.46061 NA FALSE FALSE FALSE
GCST006061 0.0072 0.5511 537409 outcome TRUE reported 11360_39_RRM1_RRM1
RRM1 chr20 57416581 0.008091 5.276e-11 35380 TRUE reported
textfile 2 1 TRUE
2283 rs6017001 G A G A -0.1974 0.0026 0.16968 NA FALSE FALSE FALSE

GCST006061 0.0099 0.7926 537409 outcome TRUE reported 11360_39_RRM1_RRM1
RRM1 chr20 37015543 0.010731 1.431e-75 35360 TRUE reported
textfile 2 1 TRUE
2284 rs68149176 T C T C -0.0598 -0.0011 0.32314 NA FALSE FALSE FALSE
GCST006061 0.0086 0.8944 537409 outcome TRUE reported 11360_39_RRM1_RRM1
RRM1 chr16 87852884 0.008602 3.602e-12 35320 TRUE reported
textfile 2 1 TRUE
2285 rs73660574 A G A G -0.1304 0.0036 0.06789 NA FALSE FALSE FALSE
GCST006061 0.0213 0.8677 537409 outcome TRUE reported 11360_39_RRM1_RRM1
RRM1 chr9 132985025 0.01609 5.306e-16 35357 TRUE reported textfile
2 1 TRUE
2286 rs74459970 G A G A -0.2111 -0.0791 0.01567 NA FALSE FALSE FALSE
GCST006061 0.0371 0.03293 537409 outcome TRUE reported 11360_39_RRM1_RRM1
RRM1 chr2 68436234 0.033938 4.965e-10 35352 TRUE reported
textfile 2 1 TRUE
2287 rs74717647 T C T C 0.1189 -4.00E-04 0.04503 NA FALSE FALSE FALSE
GCST006061 0.0215 0.9837 537409 outcome TRUE reported 11360_39_RRM1_RRM1
RRM1 chr17 29019782 0.019192 5.812e-10 35351 TRUE reported
textfile 2 1 TRUE
2288 rs76966048 C T C T 0.1481 0.007 0.02868 NA FALSE FALSE FALSE
GCST006061 0.0235 0.7649 537409 outcome TRUE reported 11360_39_RRM1_RRM1
RRM1 chr22 34035140 0.0253 4.808e-09 35356 TRUE reported textfile
2 1 TRUE
2289 rs78038115 A G A G 0.1539 -0.0219 0.04768 NA FALSE FALSE FALSE
GCST006061 0.0322 0.4965 537409 outcome TRUE reported 11360_39_RRM1_RRM1
RRM1 chr22 32727047 0.019719 5.967e-15 35365 TRUE reported
textfile 2 1 TRUE
2290 rs8887 C T C T 0.0677 0.0035 0.39121 NA FALSE FALSE FALSE GCST006061
0.0075 0.6388 537409 outcome TRUE reported 11360_39_RRM1_RRM1 RRM1 chr19
4502189 0.008238 2.061e-16 35346 TRUE reported textfile 2 1 TRUE
2291 rs9400888 T C T C -0.0602 0.0054 0.45583 NA FALSE FALSE FALSE
GCST006061 0.0072 0.4569 537409 outcome TRUE reported 11360_39_RRM1_RRM1
RRM1 chr6 116101371 0.008095 1.034e-13 35344 TRUE reported
textfile 2 1 TRUE
2292 rs9609519 A G A G -0.0699 -0.0039 0.14221 NA FALSE FALSE FALSE
GCST006061 0.0098 0.6945 537409 outcome TRUE reported 11360_39_RRM1_RRM1
RRM1 chr22 32381582 0.011675 2.135e-09 35358 TRUE reported
textfile 2 1 TRUE
2293 rs1028373 T C T C -0.2275 -0.0412 0.05302 NA FALSE FALSE FALSE
GCST006061 0.0213 0.0528 537409 outcome TRUE reported 5631_83_MLN_MOTI MLN
chr20 16941367 0.017668 6.102e-38 35378 TRUE reported textfile 2
1 TRUE
2294 rs1041309 C T C T 0.1148 0.0175 0.14705 NA FALSE FALSE FALSE
GCST006061 0.0096 0.06914 537409 outcome TRUE reported 5631_83_MLN_MOTI MLN
chr1 205492435 0.01138 6.265e-24 35373 TRUE reported textfile 2 1
TRUE
2295 rs10906953 T C T C -0.0677 -0.0022 0.4379 NA FALSE FALSE FALSE
GCST006061 0.0074 0.7628 537409 outcome TRUE reported 5631_83_MLN_MOTI MLN
chr10 46331115 0.00819 1.383e-16 35326 TRUE reported textfile 2 1
TRUE
2296 rs10994838 T C T C -0.0465 0.0044 0.38616 NA FALSE FALSE FALSE
GCST006061 0.0074 0.5538 537409 outcome TRUE reported 5631_83_MLN_MOTI MLN
chr10 50882740 0.008227 1.587e-08 35354 TRUE reported textfile 2
1 TRUE
2297 rs116491682 A G A G -0.1637 -0.0345 0.0311 NA FALSE FALSE FALSE
GCST006061 0.0398 0.3861 537409 outcome TRUE reported 5631_83_MLN_MOTI MLN
chr6 21280589 0.024355 1.8e-11 35369 TRUE reported textfile 2 1
TRUE
2298 rs13209147 A G A G 0.298 0.0054 0.18842 NA FALSE FALSE FALSE
GCST006061 0.0092 0.5601 537409 outcome TRUE reported 5631_83_MLN_MOTI MLN
chr6 33824722 0.010623 3.759e-173 35357 TRUE reported textfile 2
1 TRUE
2299 rs150325021 A G A G -0.0823 0.0373 0.07613 NA FALSE FALSE FALSE
GCST006061 0.0191 0.0502 537409 outcome TRUE reported 5631_83_MLN_MOTI MLN
chr16 1445455 0.015019 4.258e-08 35317 TRUE reported textfile 2 1
TRUE

2300 rs16862260 A G A G -0.2369 0.0056 0.24312 NA FALSE FALSE FALSE
GCST006061 0.0089 0.5298 537409 outcome TRUE reported 5631_83_MLN_MOTI MLN
chr2 221205237 0.009407 6.12e-140 35374 TRUE reported textfile 2
1 TRUE
2301 rs17358295 G T G T -0.0812 0.002 0.09391 NA FALSE FALSE FALSE
GCST006061 0.012 0.8648 537409 outcome TRUE reported 5631_83_MLN_MOTI MLN
chr11 34632224 0.013693 3.028e-09 35377 TRUE reported textfile 2
1 TRUE
2302 rs2206734 T C T C 0.1839 0.0065 0.14103 NA FALSE FALSE FALSE
GCST006061 0.009 0.4697 537409 outcome TRUE reported 5631_83_MLN_MOTI MLN
chr6 20694653 0.011917 1.008e-53 35381 TRUE reported textfile 2
1 TRUE
2303 rs2296329 G A G A -0.3803 0.0067 0.18569 NA FALSE FALSE FALSE
GCST006061 0.009 0.4571 537409 outcome TRUE reported 5631_83_MLN_MOTI MLN
chr6 33682966 0.010655 1e-200 35360 TRUE reported textfile 2 1
TRUE
2304 rs2366040 G A G A 0.0446 -0.0059 0.42508 NA FALSE FALSE FALSE
GCST006061 0.0072 0.4119 537409 outcome TRUE reported 5631_83_MLN_MOTI MLN
chr10 125831860 0.008172 4.817e-08 35375 TRUE reported textfile 2
1 TRUE
2305 rs2810491 C T C T -0.0583 0.0115 0.26324 NA FALSE FALSE FALSE
GCST006061 0.008 0.1503 537409 outcome TRUE reported 5631_83_MLN_MOTI MLN
chr9 134058209 0.009166 2.007e-10 35366 TRUE reported textfile 2
1 TRUE
2306 rs323031 A G A G 0.2139 0.0207 0.0113 NA FALSE FALSE FALSE
GCST006061 0.0523 0.692 537409 outcome TRUE reported 5631_83_MLN_MOTI MLN
chr16 2306823 0.037724 1.427e-08 35318 TRUE reported textfile 2 1
TRUE
2307 rs4536103 G A G A -0.057 0.0025 0.25812 NA FALSE FALSE FALSE
GCST006061 0.0076 0.7399 537409 outcome TRUE reported 5631_83_MLN_MOTI MLN
chr10 69572448 0.009196 5.711e-10 35363 TRUE reported textfile 2
1 TRUE
2308 rs492602 G A G A -0.086 0.0055 0.39439 NA FALSE FALSE FALSE
GCST006061 0.0076 0.4635 537409 outcome TRUE reported 5631_83_MLN_MOTI MLN
chr19 48703160 0.008244 1.786e-25 35377 TRUE reported textfile 2
1 TRUE
2309 rs56092644 T G T G 0.0825 -0.0083 0.23637 NA FALSE FALSE FALSE
GCST006061 0.0095 0.3849 537409 outcome TRUE reported 5631_83_MLN_MOTI MLN
chr5 96439105 0.009358 1.186e-18 35348 TRUE reported textfile 2
1 TRUE
2310 rs679332 G A G A 0.1112 0.0245 0.13635 NA FALSE FALSE FALSE
GCST006061 0.009 0.00649 537409 outcome TRUE reported 5631_83_MLN_MOTI MLN
chr6 116935593 0.012201 7.961e-20 35353 TRUE reported textfile 2
1 TRUE
2311 rs73256185 T C T C 0.086 -0.0103 0.10266 NA FALSE FALSE FALSE
GCST006061 0.0135 0.4461 537409 outcome TRUE reported 5631_83_MLN_MOTI MLN
chr20 17221738 0.013359 1.214e-10 35378 TRUE reported textfile 2
1 TRUE
2312 rs73408232 T C T C 0.209 -5.00E-04 0.09578 NA FALSE FALSE FALSE
GCST006061 0.0115 0.9652 537409 outcome TRUE reported 5631_83_MLN_MOTI MLN
chr6 33658265 0.014107 1.171e-49 35365 TRUE reported textfile 2
1 TRUE
2313 rs74395814 C T C T 0.0614 0.0191 0.16103 NA FALSE FALSE FALSE
GCST006061 0.0111 0.08504 537409 outcome TRUE reported 5631_83_MLN_MOTI MLN
chr15 61976712 0.010905 1.795e-08 35376 TRUE reported textfile 2
1 TRUE
2314 rs7516442 T C T C 0.0538 -0.0074 0.3939 NA FALSE FALSE FALSE
GCST006061 0.0073 0.3164 537409 outcome TRUE reported 5631_83_MLN_MOTI MLN
chr1 54122437 0.008264 7.501e-11 35365 TRUE reported textfile 2
1 TRUE
2315 rs815233 A C A C 0.0581 -0.0088 0.19376 NA FALSE FALSE FALSE
GCST006061 0.0092 0.3419 537409 outcome TRUE reported 5631_83_MLN_MOTI MLN
chr4 167141851 0.010148 1.032e-08 35363 TRUE reported textfile 2
1 TRUE
2316 rs826404 C T C T -0.0637 0.0088 0.4317 NA FALSE FALSE FALSE
GCST006061 0.0072 0.2189 537409 outcome TRUE reported 5631_83_MLN_MOTI MLN

chr1 117622100 0.008129 4.651e-15 35364 TRUE reported textfile 2
1 TRUE
2317 rs9380392 T C T C 0.092 -0.0078 0.41859 NA FALSE FALSE FALSE
GCST006061 0.0073 0.2888 537409 outcome TRUE reported 5631_83_MLN_MOTI MLN
chr6 33960658 0.008471 1.772e-27 35350 TRUE reported textfile 2
1 TRUE
2318 rs9808155 T G T G 0.074 0.0061 0.23104 NA FALSE FALSE FALSE
GCST006061 0.0088 0.4892 537409 outcome TRUE reported 5631_83_MLN_MOTI MLN
chr2 221063132 0.009631 1.551e-14 35375 TRUE reported textfile 2
1 TRUE
2319 rs1028883 G T G T -0.0583 -0.005 0.47011 NA FALSE FALSE FALSE
GCST006061 0.0072 0.4886 537409 outcome TRUE reported 4498_62_NCAM1_NCAM_120
NCAM1 chr13 73534450 0.008436 4.813e-12 35358 TRUE reported
textfile 2 1 TRUE
2320 rs11074065 A G A G 0.0616 0.0015 0.44086 NA FALSE FALSE FALSE
GCST006061 0.0072 0.8388 537409 outcome TRUE reported 4498_62_NCAM1_NCAM_120
NCAM1 chr15 92401605 0.008444 2.983e-13 35374 TRUE reported
textfile 2 1 TRUE
2321 rs11130306 G A G A -0.0522 0.0055 0.45979 NA FALSE FALSE FALSE
GCST006061 0.0071 0.4384 537409 outcome TRUE reported 4498_62_NCAM1_NCAM_120
NCAM1 chr3 52507237 0.008456 6.707e-10 35350 TRUE reported
textfile 2 1 TRUE
2322 rs11214489 T C T C -0.2693 -5.00E-04 0.18757 NA FALSE FALSE FALSE
GCST006061 0.0088 0.9579 537409 outcome TRUE reported 4498_62_NCAM1_NCAM_120
NCAM1 chr11 113105212 0.010819 8.989e-137 35364 TRUE reported
textfile 2 1 TRUE
2323 rs117896937 T C T C 0.1719 0.0787 0.03066 NA FALSE FALSE FALSE
GCST006061 0.0292 0.007042 537409 outcome TRUE reported
4498_62_NCAM1_NCAM_120 NCAM1 chr17 41697767 0.024919 5.261e-12 35385
TRUE reported textfile 2 1 TRUE
2324 rs1260326 C T C T 0.069 -0.0069 0.34132 NA FALSE FALSE FALSE
GCST006061 0.0073 0.3401 537409 outcome TRUE reported 4498_62_NCAM1_NCAM_120
NCAM1 chr2 27508073 0.008911 9.681e-15 35373 TRUE reported
textfile 2 1 TRUE
2325 rs142597063 C A C A 0.2015 -0.1172 0.01398 NA FALSE FALSE FALSE
GCST006061 0.0512 0.02202 537409 outcome TRUE reported 4498_62_NCAM1_NCAM_120
NCAM1 chr11 108719313 0.035899 1.989e-08 35355 TRUE reported
textfile 2 1 TRUE
2326 rs143966156 T C T C 0.27 -0.0416 0.02144 NA FALSE FALSE FALSE
GCST006061 0.0425 0.3273 537409 outcome TRUE reported 4498_62_NCAM1_NCAM_120
NCAM1 chr17 41490386 0.02913 1.883e-20 35386 TRUE reported textfile
2 1 TRUE
2327 rs145887694 G T G T 0.1787 -0.0117 0.01977 NA FALSE FALSE FALSE
GCST006061 0.0305 0.7001 537409 outcome TRUE reported 4498_62_NCAM1_NCAM_120
NCAM1 chr3 53630094 0.03074 6.127e-09 35350 TRUE reported textfile
2 1 TRUE
2328 rs148404036 T C T C 0.2248 -0.053 0.03047 NA FALSE FALSE FALSE
GCST006061 0.0426 0.2139 537409 outcome TRUE reported 4498_62_NCAM1_NCAM_120
NCAM1 chr17 38536272 0.024843 1.443e-19 35375 TRUE reported
textfile 2 1 TRUE
2329 rs2288158 G T G T 0.327 -0.0075 0.13012 NA FALSE FALSE FALSE
GCST006061 0.0101 0.4538 537409 outcome TRUE reported 4498_62_NCAM1_NCAM_120
NCAM1 chr11 113262954 0.012566 2.689e-149 35365 TRUE reported
textfile 2 1 TRUE
2330 rs333947 A G A G -0.0987 -0.0071 0.14937 NA FALSE FALSE FALSE
GCST006061 0.01 0.4808 537409 outcome TRUE reported 4498_62_NCAM1_NCAM_120
NCAM1 chr1 109928142 0.011884 9.933e-17 35349 TRUE reported
textfile 2 1 TRUE
2331 rs35996069 C T C T 0.2077 -0.0283 0.02222 NA FALSE FALSE FALSE
GCST006061 0.0286 0.3221 537409 outcome TRUE reported 4498_62_NCAM1_NCAM_120
NCAM1 chr17 37842173 0.028608 3.863e-13 35373 TRUE reported
textfile 2 1 TRUE
2332 rs41455646 T C T C -0.1596 -0.0498 0.03148 NA FALSE FALSE FALSE
GCST006061 0.0305 0.1029 537409 outcome TRUE reported 4498_62_NCAM1_NCAM_120
NCAM1 chr12 111308794 0.023634 1.449e-11 35370 TRUE reported
textfile 2 1 TRUE

2333 rs41463547 C T C T 0.0752 0.0068 0.10489 NA FALSE FALSE FALSE
GCST006061 0.0125 0.5847 537409 outcome TRUE reported 4498_62_NCAM1_NCAM_120
NCAM1 chr16 79520098 0.013767 4.7e-08 35362 TRUE reported textfile
2 1 TRUE
2334 rs61902388 T C T C 0.1438 2e-04 0.45148 NA FALSE FALSE FALSE
GCST006061 0.0075 0.9812 537409 outcome TRUE reported 4498_62_NCAM1_NCAM_120
NCAM1 chr11 112771352 0.00852 6.435e-64 35362 TRUE reported textfile
2 1 TRUE
2335 rs78091364 T C T C -0.1309 -4.00E-04 0.04163 NA FALSE FALSE FALSE
GCST006061 0.0258 0.9864 537409 outcome TRUE reported 4498_62_NCAM1_NCAM_120
NCAM1 chr12 110800175 0.021614 1.394e-09 35371 TRUE reported
textfile 2 1 TRUE
2336 rs79953286 G A G A -0.1079 -0.0245 0.05714 NA FALSE FALSE FALSE
GCST006061 0.0165 0.1362 537409 outcome TRUE reported 4498_62_NCAM1_NCAM_120
NCAM1 chr3 132507256 0.018309 3.786e-09 35359 TRUE reported
textfile 2 1 TRUE
2337 rs9425316 T C T C 0.0518 0.0042 0.46888 NA FALSE FALSE FALSE
GCST006061 0.0072 0.5574 537409 outcome TRUE reported 4498_62_NCAM1_NCAM_120
NCAM1 chr1 172431720 0.008425 7.826e-10 35350 TRUE reported
textfile 2 1 TRUE
2338 rs11130306 G A G A -0.074 0.0055 0.45979 NA FALSE FALSE FALSE
GCST006061 0.0071 0.4384 537409 outcome TRUE reported 2278_61_TIMP2_TIMP_2
TIMP2 chr3 52507237 0.00832 5.883e-19 35350 TRUE reported textfile
2 1 TRUE
2339 rs1260326 C T C T 0.0675 -0.0069 0.34132 NA FALSE FALSE FALSE
GCST006061 0.0073 0.3401 537409 outcome TRUE reported 2278_61_TIMP2_TIMP_2
TIMP2 chr2 27508073 0.008756 1.264e-14 35373 TRUE reported
textfile 2 1 TRUE
2340 rs243841 T G T G -0.0796 0.0033 0.37114 NA FALSE FALSE FALSE
GCST006061 0.0074 0.6546 537409 outcome TRUE reported 2278_61_TIMP2_TIMP_2
TIMP2 chr16 55494092 0.008549 1.269e-20 35374 TRUE reported
textfile 2 1 TRUE
2341 rs75609019 G A G A -0.0764 -0.0178 0.12079 NA FALSE FALSE FALSE
GCST006061 0.0146 0.2226 537409 outcome TRUE reported 2278_61_TIMP2_TIMP_2
TIMP2 chr16 55682247 0.012719 1.891e-09 35369 TRUE reported
textfile 2 1 TRUE
2342 rs77542162 G A G A 0.1542 0.0177 0.025 NA FALSE FALSE FALSE
GCST006061 0.0302 0.557 537409 outcome TRUE reported 2278_61_TIMP2_TIMP_2
TIMP2 chr17 69085137 0.026071 3.325e-09 35371 TRUE reported
textfile 2 1 TRUE
2343 rs1031707 C T C T 0.3022 0.0024 0.27769 NA FALSE FALSE FALSE
GCST006061 0.0083 0.7695 537409 outcome TRUE reported 18819_21_PPIC_PPIC
PPIC chr5 122714265 0.009034 1e-200 35354 TRUE reported textfile
2 1 TRUE
2344 rs113638297 A G A G 0.1524 -0.0011 0.0401 NA FALSE FALSE FALSE
GCST006061 0.0195 0.9544 537409 outcome TRUE reported 18819_21_PPIC_PPIC
PPIC chr5 123324883 0.020995 3.903e-13 35350 TRUE reported
textfile 2 1 TRUE
2345 rs139578440 T C T C -0.1501 -0.0246 0.04615 NA FALSE FALSE FALSE
GCST006061 0.0335 0.4627 537409 outcome TRUE reported 18819_21_PPIC_PPIC
PPIC chr5 118233056 0.019512 1.441e-14 35362 TRUE reported
textfile 2 1 TRUE
2346 rs141771168 A G A G -0.4083 0.0059 0.01312 NA FALSE FALSE FALSE
GCST006061 0.0399 0.8828 537409 outcome TRUE reported 18819_21_PPIC_PPIC
PPIC chr5 122535082 0.036512 4.956e-29 35354 TRUE reported
textfile 2 1 TRUE
2347 rs146895994 T C T C -0.1967 -0.0886 0.01713 NA FALSE FALSE FALSE
GCST006061 0.0449 0.04849 537409 outcome TRUE reported 18819_21_PPIC_PPIC
PPIC chr5 121227325 0.031779 6.033e-10 35356 TRUE reported
textfile 2 1 TRUE
2348 rs1801261 A G A G 0.1356 -0.0153 0.07643 NA FALSE FALSE FALSE
GCST006061 0.0177 0.3865 537409 outcome TRUE reported 18819_21_PPIC_PPIC
PPIC chr11 17415318 0.014798 5.03e-20 35373 TRUE reported
textfile 2 1 TRUE
2349 rs464531 A C A C 0.2323 -0.0117 0.02614 NA FALSE FALSE FALSE
GCST006061 0.0228 0.6078 537409 outcome TRUE reported 18819_21_PPIC_PPIC

PPIC chr5 124429212 0.02576 1.919e-19 35353 TRUE reported textfile
2 1 TRUE
2350 rs491567 C A C A -0.0508 -0.005 0.23278 NA FALSE FALSE FALSE
GCST006061 0.0083 0.5437 537409 outcome TRUE reported 18819_21_PPIC_PPIC
PPIC chr15 53654396 0.009261 4.132e-08 35362 TRUE reported
textfile 2 1 TRUE
2351 rs57792329 C T C T -0.4119 0.0033 0.01448 NA FALSE FALSE FALSE
GCST006061 0.0293 0.9098 537409 outcome TRUE reported 18819_21_PPIC_PPIC
PPIC chr5 124589522 0.033702 2.377e-34 35353 TRUE reported
textfile 2 1 TRUE
2352 rs61372352 T C T C 0.2732 2e-04 0.02049 NA FALSE FALSE FALSE
GCST006061 0.043 0.9972 537409 outcome TRUE reported 18819_21_PPIC_PPIC
PPIC chr5 123268019 0.028795 2.359e-21 35352 TRUE reported
textfile 2 1 TRUE
2353 rs77924615 A G A G -0.0597 0.0136 0.19701 NA FALSE FALSE FALSE
GCST006061 0.0092 0.1375 537409 outcome TRUE reported 18819_21_PPIC_PPIC
PPIC chr16 20381010 0.009952 1.987e-09 35355 TRUE reported
textfile 2 1 TRUE
2354 rs80089406 C T C T -0.415 0.0227 0.01592 NA FALSE FALSE FALSE
GCST006061 0.0303 0.4542 537409 outcome TRUE reported 18819_21_PPIC_PPIC
PPIC chr5 121946448 0.033182 6.844e-36 35354 TRUE reported
textfile 2 1 TRUE
2355 rs1033415 G A G A 0.046 -0.0128 0.41548 NA FALSE FALSE FALSE
GCST006061 0.008 0.1094 537409 outcome TRUE reported 8459_10_BMP6_BMP_6
BMP6 chr22 39492769 0.008286 2.829e-08 35368 TRUE reported
textfile 2 1 TRUE
2356 rs10793092 A G A G 0.0709 0.0057 0.45727 NA FALSE FALSE FALSE
GCST006061 0.0072 0.4273 537409 outcome TRUE reported 8459_10_BMP6_BMP_6
BMP6 chr11 74567662 0.008192 4.916e-18 35369 TRUE reported
textfile 2 1 TRUE
2357 rs10861032 C T C T 0.0731 0.0049 0.15546 NA FALSE FALSE FALSE
GCST006061 0.0096 0.609 537409 outcome TRUE reported 8459_10_BMP6_BMP_6
BMP6 chr12 103518728 0.011316 1.048e-10 35367 TRUE reported
textfile 2 1 TRUE
2358 rs113747960 A G A G -0.0731 -0.0034 0.12384 NA FALSE FALSE FALSE
GCST006061 0.0107 0.7473 537409 outcome TRUE reported 8459_10_BMP6_BMP_6
BMP6 chr12 7074644 0.012578 6.186e-09 35389 TRUE reported textfile
2 1 TRUE
2359 rs12751502 T C T C -0.0823 -0.0247 0.22251 NA FALSE FALSE FALSE
GCST006061 0.0085 0.003782 537409 outcome TRUE reported 8459_10_BMP6_BMP_6
BMP6 chr1 19354038 0.009804 4.678e-17 35379 TRUE reported
textfile 2 1 TRUE
2360 rs143501783 C T C T 0.2558 -0.0333 0.01116 NA FALSE FALSE FALSE
GCST006061 0.0443 0.4525 537409 outcome TRUE reported 8459_10_BMP6_BMP_6
BMP6 chr17 7897001 0.037037 4.963e-12 35373 TRUE reported textfile
2 1 TRUE
2361 rs55714927 T C T C 0.0967 1e-04 0.21166 NA FALSE FALSE FALSE
GCST006061 0.0098 0.9926 537409 outcome TRUE reported 8459_10_BMP6_BMP_6
BMP6 chr17 7176997 0.010026 5.175e-22 35370 TRUE reported textfile
2 1 TRUE
2362 rs61389091 T C T C -0.3823 0.029 0.05062 NA FALSE FALSE FALSE
GCST006061 0.019 0.127 537409 outcome TRUE reported 8459_10_BMP6_BMP_6
BMP6 chr11 74716876 0.018414 9.669e-96 35367 TRUE reported
textfile 2 1 TRUE
2363 rs635634 C T C T 0.089 0.0285 0.12995 NA FALSE FALSE FALSE
GCST006061 0.0089 0.001351 537409 outcome TRUE reported 8459_10_BMP6_BMP_6
BMP6 chr9 133279427 0.012113 2.017e-13 35371 TRUE reported
textfile 2 1 TRUE
2364 rs1033415 G A G A 0.0577 -0.0128 0.41548 NA FALSE FALSE FALSE
GCST006061 0.008 0.1094 537409 outcome TRUE reported 6620_82_LINGO1_LIGO1
LINGO1 chr22 39492769 0.008308 3.78e-12 35368 TRUE reported
textfile 2 1 TRUE
2365 rs10762694 A C A C 0.0801 0.0013 0.20953 NA FALSE FALSE FALSE
GCST006061 0.0083 0.8733 537409 outcome TRUE reported 6620_82_LINGO1_LIGO1
LINGO1 chr10 76127195 0.010106 2.267e-15 35367 TRUE reported
textfile 2 1 TRUE

2366 rs10922098 T C T C -0.1275 -0.009 0.40011 NA FALSE FALSE FALSE GCST006061 0.0075 0.2275 537409 outcome TRUE reported 6620_82_LINGO1_LIGO1 LINGO1 chr1 196695521 0.008342 9.829e-53 35338 TRUE reported textfile 2 1 TRUE

2367 rs11153801 A C A C 0.0581 2e-04 0.19772 NA FALSE FALSE FALSE GCST006061 0.0088 0.9856 537409 outcome TRUE reported 6620_82_LINGO1_LIGO1 LINGO1 chr6 119262615 0.010248 1.431e-08 35354 TRUE reported textfile 2 1 TRUE

2368 rs1467206 T C T C 0.0604 -0.0229 0.27093 NA FALSE FALSE FALSE GCST006061 0.0082 0.005511 537409 outcome TRUE reported 6620_82_LINGO1_LIGO1 LINGO1 chr4 81220740 0.009231 6.013e-11 35354 TRUE reported textfile 2 1 TRUE

2369 rs1706435 G A G A 0.0684 0.0117 0.36981 NA FALSE FALSE FALSE GCST006061 0.0072 0.1047 537409 outcome TRUE reported 6620_82_LINGO1_LIGO1 LINGO1 chr3 165743688 0.008452 5.835e-16 35339 TRUE reported textfile 2 1 TRUE

2370 rs17423152 C T C T 0.0868 -0.0272 0.07262 NA FALSE FALSE FALSE GCST006061 0.0139 0.04993 537409 outcome TRUE reported 6620_82_LINGO1_LIGO1 LINGO1 chr1 87428680 0.01574 3.498e-08 35340 TRUE reported textfile 2 1 TRUE

2371 rs215226 G A G A 0.0534 -0.0191 0.41752 NA FALSE FALSE FALSE GCST006061 0.0073 0.008405 537409 outcome TRUE reported 6620_82_LINGO1_LIGO1 LINGO1 chr12 482134 0.008326 1.424e-10 35311 TRUE reported textfile 2 1 TRUE

2372 rs62009067 C A C A 0.0864 0.0088 0.30299 NA FALSE FALSE FALSE GCST006061 0.0077 0.2548 537409 outcome TRUE reported 6620_82_LINGO1_LIGO1 LINGO1 chr15 77520745 0.008862 1.86e-22 35362 TRUE reported textfile 2 1 TRUE

2373 rs1033779 C A C A -0.0538 -9.00E-04 0.28645 NA FALSE FALSE FALSE GCST006061 0.0082 0.9154 537409 outcome TRUE reported 18397_5_AKR1C4_AK1C4 AKR1C4 chr10 4121087 0.008782 8.993e-10 35373 TRUE reported textfile 2 1 TRUE

2374 rs10904475 C T C T 0.0929 0.0121 0.39788 NA FALSE FALSE FALSE GCST006061 0.0078 0.118 537409 outcome TRUE reported 18397_5_AKR1C4_AK1C4 AKR1C4 chr10 5351766 0.008123 2.754e-30 35365 TRUE reported textfile 2 1 TRUE

2375 rs1476029 T C T C -0.0681 -0.0048 0.14341 NA FALSE FALSE FALSE GCST006061 0.0104 0.6443 537409 outcome TRUE reported 18397_5_AKR1C4_AK1C4 AKR1C4 chr22 36121884 0.011054 7.232e-10 35372 TRUE reported textfile 2 1 TRUE

2376 rs2954038 A C A C -0.0505 0.0046 0.31787 NA FALSE FALSE FALSE GCST006061 0.0078 0.5552 537409 outcome TRUE reported 18397_5_AKR1C4_AK1C4 AKR1C4 chr8 125495147 0.00829 1.116e-09 35367 TRUE reported textfile 2 1 TRUE

2377 rs3747207 A G A G 0.074 0.0153 0.22927 NA FALSE FALSE FALSE GCST006061 0.0085 0.07308 537409 outcome TRUE reported 18397_5_AKR1C4_AK1C4 AKR1C4 chr22 43928975 0.009193 8.319e-16 35372 TRUE reported textfile 2 1 TRUE

2378 rs4746822 T C T C 0.0604 0.0082 0.27426 NA FALSE FALSE FALSE GCST006061 0.0077 0.287 537409 outcome TRUE reported 18397_5_AKR1C4_AK1C4 AKR1C4 chr10 69223185 0.008891 1.094e-11 35362 TRUE reported textfile 2 1 TRUE

2379 rs4747987 A G A G -0.2231 0.0238 0.04108 NA FALSE FALSE FALSE GCST006061 0.0215 0.2677 537409 outcome TRUE reported 18397_5_AKR1C4_AK1C4 AKR1C4 chr10 5664589 0.020071 1.053e-28 35378 TRUE reported textfile 2 1 TRUE

2380 rs72779812 A G A G -0.2379 -0.0051 0.01669 NA FALSE FALSE FALSE GCST006061 0.0223 0.8181 537409 outcome TRUE reported 18397_5_AKR1C4_AK1C4 AKR1C4 chr10 5383468 0.030751 1.024e-14 35372 TRUE reported textfile 2 1 TRUE

2381 rs75056746 G A G A -0.2174 0.0323 0.03968 NA FALSE FALSE FALSE GCST006061 0.028 0.2484 537409 outcome TRUE reported 18397_5_AKR1C4_AK1C4 AKR1C4 chr10 4540637 0.020323 1.049e-26 35371 TRUE reported textfile 2 1 TRUE

2382 rs1034826 C T C T -0.0679 -0.0142 0.22809 NA FALSE FALSE FALSE GCST006061 0.0078 0.07094 537409 outcome TRUE reported 5109_24_NRCAM_Nr_CAM

NRCAM chr7 108443160 0.009752 3.345e-12 35365 TRUE reported
textfile 2 1 TRUE
2383 rs11465428 A C A C -0.1045 0.0059 0.07459 NA FALSE FALSE FALSE
GCST006061 0.0155 0.7028 537409 outcome TRUE reported 5109_24_NRCAM_Nr_CAM
NRCAM chr19 7739786 0.015818 3.939e-11 35369 TRUE reported textfile
2 1 TRUE
2384 rs635634 C T C T 0.1415 0.0285 0.12995 NA FALSE FALSE FALSE
GCST006061 0.0089 0.001351 537409 outcome TRUE reported
5109_24_NRCAM_Nr_CAM NRCAM chr9 133279427 0.01222 5.259e-31 35371 TRUE
reported textfile 2 1 TRUE
2385 rs1035002 T C T C 0.165 -0.0075 0.02407 NA FALSE FALSE FALSE
GCST006061 0.0174 0.6679 537409 outcome TRUE reported
2602_2_ANGPT2_Angiopoietin_2 ANGPT2 chr3 52454186 0.02778 2.86e-09 35350
TRUE reported textfile 2 1 TRUE
2386 rs10935480 T C T C -0.0501 0.0038 0.43728 NA FALSE FALSE FALSE
GCST006061 0.0072 0.5965 537409 outcome TRUE reported
2602_2_ANGPT2_Angiopoietin_2 ANGPT2 chr3 98713142 0.008464 3.23e-09
35330 TRUE reported textfile 2 1 TRUE
2387 rs142029184 A G A G 0.2395 -0.0726 0.01311 NA FALSE FALSE FALSE
GCST006061 0.0428 0.09019 537409 outcome TRUE reported
2602_2_ANGPT2_Angiopoietin_2 ANGPT2 chr11 126401713 0.037067 1.038e-10
35374 TRUE reported textfile 2 1 TRUE
2388 rs143501783 C T C T 0.2528 -0.0333 0.01116 NA FALSE FALSE FALSE
GCST006061 0.0443 0.4525 537409 outcome TRUE reported
2602_2_ANGPT2_Angiopoietin_2 ANGPT2 chr17 7897001 0.038247 3.853e-11 35373
TRUE reported textfile 2 1 TRUE
2389 rs187099314 T C T C 0.2217 0.0287 0.06588 NA FALSE FALSE FALSE
GCST006061 0.016 0.07216 537409 outcome TRUE reported
2602_2_ANGPT2_Angiopoietin_2 ANGPT2 chr9 133255354 0.016892 2.391e-39
35370 TRUE reported textfile 2 1 TRUE
2390 rs1968586 T C T C -0.1308 -0.0109 0.3488 NA FALSE FALSE FALSE
GCST006061 0.0079 0.168 537409 outcome TRUE reported
2602_2_ANGPT2_Angiopoietin_2 ANGPT2 chr8 6421990 0.00886 2.536e-49 35377
TRUE reported textfile 2 1 TRUE
2391 rs35257264 T C T C 0.2019 0.0195 0.03294 NA FALSE FALSE FALSE
GCST006061 0.0269 0.4693 537409 outcome TRUE reported
2602_2_ANGPT2_Angiopoietin_2 ANGPT2 chr11 126426921 0.022945 1.376e-18
35371 TRUE reported textfile 2 1 TRUE
2392 rs56115403 A G A G 0.0839 0.0012 0.21597 NA FALSE FALSE FALSE
GCST006061 0.01 0.901 537409 outcome TRUE reported
2602_2_ANGPT2_Angiopoietin_2 ANGPT2 chr17 7160579 0.010283 3.38e-16 35371
TRUE reported textfile 2 1 TRUE
2393 rs61776478 T C T C 0.1625 0.125 0.02717 NA FALSE FALSE FALSE
GCST006061 0.0989 0.2064 537409 outcome TRUE reported
2602_2_ANGPT2_Angiopoietin_2 ANGPT2 chr1 11923034 0.025841 3.208e-10
35389 TRUE reported textfile 2 1 TRUE
2394 rs62576042 T C T C 0.0615 -0.0019 0.19144 NA FALSE FALSE FALSE
GCST006061 0.0109 0.8608 537409 outcome TRUE reported
2602_2_ANGPT2_Angiopoietin_2 ANGPT2 chr9 133513605 0.010737 1.017e-08
35360 TRUE reported textfile 2 1 TRUE
2395 rs6601299 C T C T -0.0914 -0.0123 0.08373 NA FALSE FALSE FALSE
GCST006061 0.0123 0.3154 537409 outcome TRUE reported
2602_2_ANGPT2_Angiopoietin_2 ANGPT2 chr8 9327181 0.015127 1.522e-09 35385
TRUE reported textfile 2 1 TRUE
2396 rs1035029 A G A G 0.1156 0.0197 0.37924 NA FALSE FALSE FALSE
GCST006061 0.0073 0.007006 537409 outcome TRUE reported 2381_52_C5_C5 C5
chr9 120980540 0.008523 6.598e-42 35376 TRUE reported textfile 2
1 TRUE
2397 rs10801551 A G A G 0.0535 -0.003 0.2428 NA FALSE FALSE FALSE
GCST006061 0.0085 0.7222 537409 outcome TRUE reported 2381_52_C5_C5 C5
chr1 196610909 0.009652 2.977e-08 35338 TRUE reported textfile 2
1 TRUE
2398 rs17408255 A G A G 0.1041 0.0034 0.06189 NA FALSE FALSE FALSE
GCST006061 0.0196 0.8636 537409 outcome TRUE reported 2381_52_C5_C5 C5
chr12 24103060 0.017355 1.993e-09 35360 TRUE reported textfile 2
1 TRUE

2399 rs780094 C T C T -0.066 -0.0076 0.33917 NA FALSE FALSE FALSE
GCST006061 0.0073 0.2951 537409 outcome TRUE reported 2381_52_C5_C5 C5
chr2 27518370 0.008775 5.427e-14 35373 TRUE reported textfile 2
1 TRUE
2400 rs9775994 T C T C -0.0735 0.0156 0.15623 NA FALSE FALSE FALSE
GCST006061 0.0094 0.09636 537409 outcome TRUE reported 2381_52_C5_C5 C5
chr9 121555175 0.011449 1.366e-10 35373 TRUE reported textfile 2
1 TRUE
2401 rs1035376 A G A G 0.2207 0.026 0.15924 NA FALSE FALSE FALSE
GCST006061 0.0114 0.02248 537409 outcome TRUE reported 12563_2_TNFAIP8_TFIP8
TNFAIP8 chr5 119312555 0.010836 3.325e-92 35353 TRUE reported
textfile 2 1 TRUE
2402 rs12234017 G A G A -0.0652 -0.0098 0.23636 NA FALSE FALSE FALSE
GCST006061 0.0084 0.2431 537409 outcome TRUE reported 12563_2_TNFAIP8_TFIP8
TNFAIP8 chr5 119376139 0.009241 1.724e-12 35353 TRUE reported
textfile 2 1 TRUE
2403 rs1036491 C A C A -0.1517 -0.0046 0.17052 NA FALSE FALSE FALSE
GCST006061 0.0087 0.5985 537409 outcome TRUE reported 5688_65_CBLN4_CBLN4
CBLN4 chr20 55980132 0.011082 1.183e-42 35388 TRUE reported
textfile 2 1 TRUE
2404 rs1277294 T C T C -0.0848 0.0038 0.08453 NA FALSE FALSE FALSE
GCST006061 0.0161 0.8111 537409 outcome TRUE reported 5688_65_CBLN4_CBLN4
CBLN4 chr4 57063075 0.014992 1.545e-08 35358 TRUE reported
textfile 2 1 TRUE
2405 rs35887873 C T C T 0.085 -0.0114 0.26311 NA FALSE FALSE FALSE
GCST006061 0.0091 0.2089 537409 outcome TRUE reported 5688_65_CBLN4_CBLN4
CBLN4 chr11 126349501 0.009271 4.79e-20 35374 TRUE reported
textfile 2 1 TRUE
2406 rs601279 A C A C -0.0511 -0.0136 0.42207 NA FALSE FALSE FALSE
GCST006061 0.0074 0.06479 537409 outcome TRUE reported 5688_65_CBLN4_CBLN4
CBLN4 chr20 55605367 0.008385 1.098e-09 35389 TRUE reported
textfile 2 1 TRUE
2407 rs6024417 G A G A -0.1539 0.0096 0.29741 NA FALSE FALSE FALSE
GCST006061 0.0081 0.236 537409 outcome TRUE reported 5688_65_CBLN4_CBLN4
CBLN4 chr20 55794632 0.009032 4.156e-65 35388 TRUE reported
textfile 2 1 TRUE
2408 rs7351418 T C T C 0.1538 0.0165 0.34464 NA FALSE FALSE FALSE
GCST006061 0.008 0.03894 537409 outcome TRUE reported 5688_65_CBLN4_CBLN4
CBLN4 chr20 55721241 0.008695 5.192e-70 35390 TRUE reported
textfile 2 1 TRUE
2409 rs7684253 T C T C -0.0986 0.0214 0.44824 NA FALSE FALSE FALSE
GCST006061 0.0072 0.002914 537409 outcome TRUE reported 5688_65_CBLN4_CBLN4
CBLN4 chr4 56861145 0.008265 8.26e-33 35358 TRUE reported
textfile 2 1 TRUE
2410 rs78604342 T C T C -0.1666 -0.0199 0.04871 NA FALSE FALSE FALSE
GCST006061 0.0219 0.3647 537409 outcome TRUE reported 5688_65_CBLN4_CBLN4
CBLN4 chr20 55439565 0.019484 1.225e-17 35380 TRUE reported
textfile 2 1 TRUE
2411 rs1037590 T G T G 0.1182 -6.00E-04 0.15136 NA FALSE FALSE FALSE
GCST006061 0.009 0.9475 537409 outcome TRUE reported 9758_17_RPS4X_RS4X
RPS4X chr17 35575616 0.011126 2.302e-26 35371 TRUE reported
textfile 2 1 TRUE
2412 rs10876550 A G A G 0.0507 -1.00E-04 0.43699 NA FALSE FALSE FALSE
GCST006061 0.0073 0.991 537409 outcome TRUE reported 9758_17_RPS4X_RS4X
RPS4X chr12 54318524 0.008077 3.454e-10 35374 TRUE reported
textfile 2 1 TRUE
2413 rs1354034 C T C T 0.1725 0.0088 0.38119 NA FALSE FALSE FALSE
GCST006061 0.0073 0.2248 537409 outcome TRUE reported 9758_17_RPS4X_RS4X
RPS4X chr3 56815721 0.008197 2.636e-98 35356 TRUE reported
textfile 2 1 TRUE
2414 rs1037590 T G T G 0.1047 -6.00E-04 0.15136 NA FALSE FALSE FALSE
GCST006061 0.009 0.9475 537409 outcome TRUE reported 3864_5_RPS7_RS7 RPS7
chr17 35575616 0.011099 3.979e-21 35371 TRUE reported textfile 2
1 TRUE
2415 rs10922098 T C T C -0.0752 -0.009 0.40011 NA FALSE FALSE FALSE
GCST006061 0.0075 0.2275 537409 outcome TRUE reported 3864_5_RPS7_RS7 RPS7

chr1 196695521 0.008151 2.822e-20 35338 TRUE reported textfile 2
1 TRUE
2416 rs1354034 C T C T 0.1553 0.0088 0.38119 NA FALSE FALSE FALSE
GCST006061 0.0073 0.2248 537409 outcome TRUE reported 3864_5_RPS7_RS7 RPS7
chr3 56815721 0.008186 2.959e-80 35356 TRUE reported textfile 2
1 TRUE
2417 rs1037590 T G T G 0.1079 -6.00E-04 0.15136 NA FALSE FALSE FALSE
GCST006061 0.009 0.9475 537409 outcome TRUE reported 19166_15_RPS19_RS19
RPS19 chr17 35575616 0.01115 3.765e-22 35371 TRUE reported textfile
2 1 TRUE
2418 rs11553699 G A G A 0.0673 -0.0209 0.14277 NA FALSE FALSE FALSE
GCST006061 0.012 0.08291 537409 outcome TRUE reported 19166_15_RPS19_RS19
RPS19 chr12 121779004 0.011462 4.316e-09 35366 TRUE reported
textfile 2 1 TRUE
2419 rs1354034 C T C T 0.1687 0.0088 0.38119 NA FALSE FALSE FALSE
GCST006061 0.0073 0.2248 537409 outcome TRUE reported 19166_15_RPS19_RS19
RPS19 chr3 56815721 0.008218 1.209e-93 35356 TRUE reported
textfile 2 1 TRUE
2420 rs203687 T C T C -0.0529 -0.0146 0.4315 NA FALSE FALSE FALSE
GCST006061 0.0075 0.05005 537409 outcome TRUE reported 19166_15_RPS19_RS19
RPS19 chr1 196705140 0.008084 6.003e-11 35339 TRUE reported
textfile 2 1 TRUE
2421 rs72865316 A G A G 0.0789 -2.00E-04 0.24101 NA FALSE FALSE FALSE
GCST006061 0.0092 0.9865 537409 outcome TRUE reported 19166_15_RPS19_RS19
RPS19 chr11 247028 0.009363 3.553e-17 35349 TRUE reported textfile
2 1 TRUE
2422 rs1037590 T G T G 0.0763 -6.00E-04 0.15136 NA FALSE FALSE FALSE
GCST006061 0.009 0.9475 537409 outcome TRUE reported 6377_54_LYZL2_LYZL2
LYZL2 chr17 35575616 0.011018 4.366e-12 35371 TRUE reported
textfile 2 1 TRUE
2423 rs10922098 T C T C -0.0467 -0.009 0.40011 NA FALSE FALSE FALSE
GCST006061 0.0075 0.2275 537409 outcome TRUE reported 6377_54_LYZL2_LYZL2
LYZL2 chr1 196695521 0.008084 7.601e-09 35338 TRUE reported
textfile 2 1 TRUE
2424 rs114647468 A G A G 0.1949 -0.1272 0.01647 NA FALSE FALSE FALSE
GCST006061 0.0501 0.01106 537409 outcome TRUE reported 6377_54_LYZL2_LYZL2
LYZL2 chr3 127171400 0.030327 1.304e-10 35367 TRUE reported
textfile 2 1 TRUE
2425 rs1354034 C T C T 0.1211 0.0088 0.38119 NA FALSE FALSE FALSE
GCST006061 0.0073 0.2248 537409 outcome TRUE reported 6377_54_LYZL2_LYZL2
LYZL2 chr3 56815721 0.008127 3.255e-50 35356 TRUE reported
textfile 2 1 TRUE
2426 rs4980320 C T C T 0.0634 0.0041 0.46986 NA FALSE FALSE FALSE
GCST006061 0.0076 0.5858 537409 outcome TRUE reported 6377_54_LYZL2_LYZL2
LYZL2 chr11 198986 0.007941 1.414e-15 35352 TRUE reported textfile
2 1 TRUE
2427 rs1037590 T G T G 0.0923 -6.00E-04 0.15136 NA FALSE FALSE FALSE
GCST006061 0.009 0.9475 537409 outcome TRUE reported 19195_85_RPS5_RS5
RPS5 chr17 35575616 0.011085 8.333e-17 35371 TRUE reported
textfile 2 1 TRUE
2428 rs11606393 A G A G 0.0727 -0.0024 0.2405 NA FALSE FALSE FALSE
GCST006061 0.009 0.7938 537409 outcome TRUE reported 19195_85_RPS5_RS5
RPS5 chr11 208235 0.009317 6.044e-15 35350 TRUE reported textfile
2 1 TRUE
2429 rs1354034 C T C T 0.1359 0.0088 0.38119 NA FALSE FALSE FALSE
GCST006061 0.0073 0.2248 537409 outcome TRUE reported 19195_85_RPS5_RS5
RPS5 chr3 56815721 0.008179 5.357e-62 35356 TRUE reported
textfile 2 1 TRUE
2430 rs203687 T C T C -0.0528 -0.0146 0.4315 NA FALSE FALSE FALSE
GCST006061 0.0075 0.05005 537409 outcome TRUE reported 19195_85_RPS5_RS5
RPS5 chr1 196705140 0.008037 5.037e-11 35339 TRUE reported
textfile 2 1 TRUE
2431 rs1037590 T G T G 0.1666 -6.00E-04 0.15136 NA FALSE FALSE FALSE
GCST006061 0.009 0.9475 537409 outcome TRUE reported 6372_7_YBX2_YBOX2
YBX2 chr17 35575616 0.011237 9.935e-50 35371 TRUE reported
textfile 2 1 TRUE

2432 rs11082513 T C T C -0.0447 0.0048 0.48528 NA FALSE FALSE FALSE
GCST006061 0.0075 0.5225 537409 outcome TRUE reported 6372_7_YBX2_YBOX2
YBX2 chr18 46167187 0.008137 3.944e-08 35350 TRUE reported
textfile 2 1 TRUE
2433 rs1354034 C T C T 0.1799 0.0088 0.38119 NA FALSE FALSE FALSE
GCST006061 0.0073 0.2248 537409 outcome TRUE reported 6372_7_YBX2_YBOX2
YBX2 chr3 56815721 0.008291 2.125e-104 35356 TRUE reported
textfile 2 1 TRUE
2434 rs4820091 G T G T 0.0624 -0.0166 0.19742 NA FALSE FALSE FALSE
GCST006061 0.0088 0.06081 537409 outcome TRUE reported 6372_7_YBX2_YBOX2
YBX2 chr22 21585900 0.01019 9.134e-10 35367 TRUE reported textfile
2 1 TRUE
2435 rs72865316 A G A G 0.0935 -2.00E-04 0.24101 NA FALSE FALSE FALSE
GCST006061 0.0092 0.9865 537409 outcome TRUE reported 6372_7_YBX2_YBOX2
YBX2 chr11 247028 0.009455 4.649e-23 35349 TRUE reported textfile
2 1 TRUE
2436 rs9894813 T C T C 0.0724 0.0029 0.21357 NA FALSE FALSE FALSE
GCST006061 0.0089 0.7467 537409 outcome TRUE reported 6372_7_YBX2_YBOX2
YBX2 chr17 35546829 0.009871 2.222e-13 35369 TRUE reported
textfile 2 1 TRUE
2437 rs1037590 T G T G 0.07 -6.00E-04 0.15136 NA FALSE FALSE FALSE
GCST006061 0.009 0.9475 537409 outcome TRUE reported
17781_191_MAP1LC3A_MLP3A MAP1LC3A chr17 35575616 0.011429 9.087e-10
35371 TRUE reported textfile 2 1 TRUE
2438 rs10878645 C T C T -0.0488 0.0019 0.49581 NA FALSE FALSE FALSE
GCST006061 0.0071 0.7883 537409 outcome TRUE reported
17781_191_MAP1LC3A_MLP3A MAP1LC3A chr12 67711948 0.008226 2.987e-09
35363 TRUE reported textfile 2 1 TRUE
2439 rs11071720 C T C T 0.0492 0.0059 0.309 NA FALSE FALSE FALSE
GCST006061 0.0077 0.447 537409 outcome TRUE reported
17781_191_MAP1LC3A_MLP3A MAP1LC3A chr15 63049797 0.008862 2.823e-08
35364 TRUE reported textfile 2 1 TRUE
2440 rs11171911 T C T C -0.0481 0.0322 0.45134 NA FALSE FALSE FALSE
GCST006061 0.0075 1.841e-05 537409 outcome TRUE reported
17781_191_MAP1LC3A_MLP3A MAP1LC3A chr12 56623731 0.008295 6.685e-09
35369 TRUE reported textfile 2 1 TRUE
2441 rs114694170 C T C T 0.0855 0.0053 0.08378 NA FALSE FALSE FALSE
GCST006061 0.0175 0.7624 537409 outcome TRUE reported
17781_191_MAP1LC3A_MLP3A MAP1LC3A chr5 88884379 0.014902 9.612e-09
35344 TRUE reported textfile 2 1 TRUE
2442 rs11553699 G A G A -0.0662 -0.0209 0.14277 NA FALSE FALSE FALSE
GCST006061 0.012 0.08291 537409 outcome TRUE reported
17781_191_MAP1LC3A_MLP3A MAP1LC3A chr12 121779004 0.011734 1.684e-08
35366 TRUE reported textfile 2 1 TRUE
2443 rs12767683 A C A C -0.062 0.0038 0.26443 NA FALSE FALSE FALSE
GCST006061 0.0079 0.6284 537409 outcome TRUE reported
17781_191_MAP1LC3A_MLP3A MAP1LC3A chr10 102556824 0.009353 3.376e-11
35370 TRUE reported textfile 2 1 TRUE
2444 rs1354034 C T C T 0.219 0.0088 0.38119 NA FALSE FALSE FALSE
GCST006061 0.0073 0.2248 537409 outcome TRUE reported
17781_191_MAP1LC3A_MLP3A MAP1LC3A chr3 56815721 0.008404 1.084e-149
35356 TRUE reported textfile 2 1 TRUE
2445 rs185009887 A G A G -0.1729 -0.0595 0.02519 NA FALSE FALSE FALSE
GCST006061 0.0496 0.2302 537409 outcome TRUE reported
17781_191_MAP1LC3A_MLP3A MAP1LC3A chr17 5848332 0.026614 8.214e-11 35374
TRUE reported textfile 2 1 TRUE
2446 rs3112401 C T C T -0.0637 0.0132 0.37671 NA FALSE FALSE FALSE
GCST006061 0.0074 0.07569 537409 outcome TRUE reported
17781_191_MAP1LC3A_MLP3A MAP1LC3A chr5 72107446 0.008516 7.446e-14
35356 TRUE reported textfile 2 1 TRUE
2447 rs35340377 A G A G -0.0544 0.0075 0.25094 NA FALSE FALSE FALSE
GCST006061 0.0082 0.3585 537409 outcome TRUE reported
17781_191_MAP1LC3A_MLP3A MAP1LC3A chr1 247874908 0.009555 1.244e-08
35156 TRUE reported textfile 2 1 TRUE
2448 rs56225072 T C T C -0.0607 -0.0065 0.24159 NA FALSE FALSE FALSE
GCST006061 0.0089 0.4679 537409 outcome TRUE reported

17781_191_MAP1LC3A_MLP3A MAP1LC3A chr8 52800687 0.009576 2.319e-10
35356 TRUE reported textfile 2 1 TRUE
2449 rs6120675 C T C T 0.0496 0.0151 0.48415 NA FALSE FALSE FALSE
GCST006061 0.0072 0.03615 537409 outcome TRUE reported
17781_191_MAP1LC3A_MLP3A MAP1LC3A chr20 34551066 0.008163 1.228e-09
35366 TRUE reported textfile 2 1 TRUE
2450 rs740290 C T C T 0.0472 3e-04 0.38302 NA FALSE FALSE FALSE
GCST006061 0.0073 0.9697 537409 outcome TRUE reported
17781_191_MAP1LC3A_MLP3A MAP1LC3A chr7 106577756 0.008441 2.246e-08
35360 TRUE reported textfile 2 1 TRUE
2451 rs7648740 A G A G 0.0547 -0.0027 0.27068 NA FALSE FALSE FALSE
GCST006061 0.0085 0.7478 537409 outcome TRUE reported
17781_191_MAP1LC3A_MLP3A MAP1LC3A chr3 57973167 0.009316 4.314e-09
35359 TRUE reported textfile 2 1 TRUE
2452 rs7926172 A G A G 0.0744 -0.0023 0.23069 NA FALSE FALSE FALSE
GCST006061 0.0093 0.8091 537409 outcome TRUE reported
17781_191_MAP1LC3A_MLP3A MAP1LC3A chr11 225180 0.009722 1.965e-14 35352
TRUE reported textfile 2 1 TRUE
2453 rs826404 C T C T 0.0515 0.0088 0.4317 NA FALSE FALSE FALSE
GCST006061 0.0072 0.2189 537409 outcome TRUE reported
17781_191_MAP1LC3A_MLP3A MAP1LC3A chr1 117622100 0.008274 4.838e-10
35364 TRUE reported textfile 2 1 TRUE
2454 rs1037590 T G T G 0.067 -6.00E-04 0.15136 NA FALSE FALSE FALSE
GCST006061 0.009 0.9475 537409 outcome TRUE reported 13930_3_APOBEC3G_ABC3G
APOBEC3G chr17 35575616 0.011482 5.378e-09 35371 TRUE reported
textfile 2 1 TRUE
2455 rs10876550 A G A G 0.0559 -1.00E-04 0.43699 NA FALSE FALSE FALSE
GCST006061 0.0073 0.991 537409 outcome TRUE reported 13930_3_APOBEC3G_ABC3G
APOBEC3G chr12 54318524 0.00834 2.046e-11 35374 TRUE reported
textfile 2 1 TRUE
2456 rs1354034 C T C T 0.0729 0.0088 0.38119 NA FALSE FALSE FALSE
GCST006061 0.0073 0.2248 537409 outcome TRUE reported 13930_3_APOBEC3G_ABC3G
APOBEC3G chr3 56815721 0.008487 8.74e-18 35356 TRUE reported
textfile 2 1 TRUE
2457 rs4980320 C T C T 0.0597 0.0041 0.46986 NA FALSE FALSE FALSE
GCST006061 0.0076 0.5858 537409 outcome TRUE reported 13930_3_APOBEC3G_ABC3G
APOBEC3G chr11 198986 0.008273 5.337e-13 35352 TRUE reported
textfile 2 1 TRUE
2458 rs6519166 A C A C -0.119 0.0014 0.36272 NA FALSE FALSE FALSE
GCST006061 0.0078 0.8553 537409 outcome TRUE reported 13930_3_APOBEC3G_ABC3G
APOBEC3G chr22 39076172 0.008596 1.395e-43 35364 TRUE reported
textfile 2 1 TRUE
2459 rs1039405 G A G A -0.1555 -0.0127 0.45344 NA FALSE FALSE FALSE
GCST006061 0.0075 0.08904 537409 outcome TRUE reported
2742_68_SIGLEC7_Siglec_7 SIGLEC7 chr19 51122980 0.008658 3.927e-72 35371
TRUE reported textfile 2 1 TRUE
2460 rs12462100 T C T C -0.0566 0.0099 0.26807 NA FALSE FALSE FALSE
GCST006061 0.0084 0.2344 537409 outcome TRUE reported
2742_68_SIGLEC7_Siglec_7 SIGLEC7 chr19 51322795 0.009725 5.887e-09 35379
TRUE reported textfile 2 1 TRUE
2461 rs143501783 C T C T 0.2666 -0.0333 0.01116 NA FALSE FALSE FALSE
GCST006061 0.0443 0.4525 537409 outcome TRUE reported
2742_68_SIGLEC7_Siglec_7 SIGLEC7 chr17 7897001 0.038931 7.486e-12 35373
TRUE reported textfile 2 1 TRUE
2462 rs2160725 C A C A -0.0628 -0.0072 0.48448 NA FALSE FALSE FALSE
GCST006061 0.0071 0.3111 537409 outcome TRUE reported
2742_68_SIGLEC7_Siglec_7 SIGLEC7 chr17 68399585 0.008639 3.607e-13 35368
TRUE reported textfile 2 1 TRUE
2463 rs34211178 A G A G -0.0535 -0.0027 0.42846 NA FALSE FALSE FALSE
GCST006061 0.0072 0.7123 537409 outcome TRUE reported
2742_68_SIGLEC7_Siglec_7 SIGLEC7 chr3 98664718 0.008608 5.135e-10 35330
TRUE reported textfile 2 1 TRUE
2464 rs55714927 T C T C 0.0933 1e-04 0.21166 NA FALSE FALSE FALSE
GCST006061 0.0098 0.9926 537409 outcome TRUE reported
2742_68_SIGLEC7_Siglec_7 SIGLEC7 chr17 7176997 0.010549 9.215e-19 35370
TRUE reported textfile 2 1 TRUE

2465 rs62165726 A C A C -0.1754 -0.0263 0.03053 NA FALSE FALSE FALSE
GCST006061 0.0212 0.2141 537409 outcome TRUE reported
2742_68_SIGLEC7_Siglec_7 SIGLEC7 chr2 134208991 0.024709 1.261e-12 35391
TRUE reported textfile 2 1 TRUE
2466 rs1039405 G A G A -0.0504 -0.0127 0.45344 NA FALSE FALSE FALSE
GCST006061 0.0075 0.08904 537409 outcome TRUE reported
11152_46_KLK13_kallikrein_13 KLK13 chr19 51122980 0.009028 2.367e-08
35362 TRUE reported textfile 2 1 TRUE
2467 rs116960573 T G T G -0.2765 -0.0395 0.05439 NA FALSE FALSE FALSE
GCST006061 0.0203 0.05168 537409 outcome TRUE reported
11152_46_KLK13_kallikrein_13 KLK13 chr19 51181877 0.019857 4.497e-44
35365 TRUE reported textfile 2 1 TRUE
2468 rs12712266 C T C T 0.052 0.0063 0.40029 NA FALSE FALSE FALSE
GCST006061 0.0073 0.3833 537409 outcome TRUE reported
11152_46_KLK13_kallikrein_13 KLK13 chr2 106933402 0.008963 6.56e-09
35349 TRUE reported textfile 2 1 TRUE
2469 rs17855739 T C T C 0.1899 0.0098 0.03389 NA FALSE FALSE FALSE
GCST006061 0.0173 0.5715 537409 outcome TRUE reported
11152_46_KLK13_kallikrein_13 KLK13 chr19 5831829 0.024425 7.564e-15 35357
TRUE reported textfile 2 1 TRUE
2470 rs2479016 G A G A -0.0572 -0.0048 0.33808 NA FALSE FALSE FALSE
GCST006061 0.0074 0.5144 537409 outcome TRUE reported
11152_46_KLK13_kallikrein_13 KLK13 chr6 2245111 0.00928 7.091e-10 35326
TRUE reported textfile 2 1 TRUE
2471 rs2548459 C T C T -0.0862 0.0052 0.37265 NA FALSE FALSE FALSE
GCST006061 0.0076 0.4961 537409 outcome TRUE reported
11152_46_KLK13_kallikrein_13 KLK13 chr19 48706082 0.009292 1.75e-20
35368 TRUE reported textfile 2 1 TRUE
2472 rs55714927 T C T C 0.0798 1e-04 0.21166 NA FALSE FALSE FALSE
GCST006061 0.0098 0.9926 537409 outcome TRUE reported
11152_46_KLK13_kallikrein_13 KLK13 chr17 7176997 0.010804 1.515e-13 35361
TRUE reported textfile 2 1 TRUE
2473 rs103947 C T C T -0.0905 -0.0083 0.38773 NA FALSE FALSE FALSE
GCST006061 0.0073 0.257 537409 outcome TRUE reported
13700_10_ANXA2_annexin_II ANXA2 chr15 60619685 0.008581 5.246e-26 35370
TRUE reported textfile 2 1 TRUE
2474 rs114694170 C T C T -0.0976 0.0053 0.08378 NA FALSE FALSE FALSE
GCST006061 0.0175 0.7624 537409 outcome TRUE reported
13700_10_ANXA2_annexin_II ANXA2 chr5 88884379 0.014515 1.769e-11 35344
TRUE reported textfile 2 1 TRUE
2475 rs16977653 G A G A 0.0831 0.0255 0.08203 NA FALSE FALSE FALSE
GCST006061 0.0141 0.06972 537409 outcome TRUE reported
13700_10_ANXA2_annexin_II ANXA2 chr15 57740535 0.015104 3.759e-08 35361
TRUE reported textfile 2 1 TRUE
2476 rs3743268 A G A G -0.33 -0.0017 0.36954 NA FALSE FALSE FALSE
GCST006061 0.0084 0.8392 537409 outcome TRUE reported
13700_10_ANXA2_annexin_II ANXA2 chr15 60385961 0.006643 1e-200 35366
TRUE reported textfile 2 1 TRUE
2477 rs72759453 T C T C 0.2108 -0.0025 0.01281 NA FALSE FALSE FALSE
GCST006061 0.0328 0.9382 537409 outcome TRUE reported
13700_10_ANXA2_annexin_II ANXA2 chr9 132974013 0.036007 4.785e-09 35368
TRUE reported textfile 2 1 TRUE
2478 rs77538017 T C T C 0.2731 0.0036 0.05162 NA FALSE FALSE FALSE
GCST006061 0.0155 0.8192 537409 outcome TRUE reported
13700_10_ANXA2_annexin_II ANXA2 chr15 59440475 0.018982 6.227e-47 35371
TRUE reported textfile 2 1 TRUE
2479 rs79120050 T C T C -0.2051 -0.017 0.14398 NA FALSE FALSE FALSE
GCST006061 0.0113 0.1319 537409 outcome TRUE reported
13700_10_ANXA2_annexin_II ANXA2 chr15 60438051 0.01195 4.997e-66 35368
TRUE reported textfile 2 1 TRUE
2480 rs10399785 C T C T -0.1014 -0.0084 0.49741 NA FALSE FALSE FALSE
GCST006061 0.0072 0.2478 537409 outcome TRUE reported 18343_10_DECR2_DECR2
DECR2 chr1 94412245 0.008171 2.301e-35 35353 TRUE reported
textfile 2 1 TRUE
2481 rs10822155 A C A C 0.0564 -0.0472 0.43479 NA FALSE FALSE FALSE
GCST006061 0.0072 4.622e-11 537409 outcome TRUE reported

18343_10_DECR2_DECR2 DECR2 chr10 63311455 0.008267 8.972e-12 35351
TRUE reported textfile 2 1 TRUE
2482 rs1354034 C T C T 0.0592 0.0088 0.38119 NA FALSE FALSE FALSE
GCST006061 0.0073 0.2248 537409 outcome TRUE reported 18343_10_DECR2_DECR2
DECR2 chr3 56815721 0.008446 2.393e-12 35356 TRUE reported
textfile 2 1 TRUE
2483 rs436075 C T C T 0.285 -0.0104 0.49548 NA FALSE FALSE FALSE
GCST006061 0.0077 0.1765 537409 outcome TRUE reported 18343_10_DECR2_DECR2
DECR2 chr16 391710 0.008271 1e-200 35318 TRUE reported textfile 2
1 TRUE
2484 rs74770429 C T C T -0.1548 0.0364 0.04468 NA FALSE FALSE FALSE
GCST006061 0.0335 0.2774 537409 outcome TRUE reported 18343_10_DECR2_DECR2
DECR2 chr22 40814137 0.019975 9.219e-15 35371 TRUE reported
textfile 2 1 TRUE
2485 rs9788833 C T C T -0.135 0.0011 0.31095 NA FALSE FALSE FALSE
GCST006061 0.0075 0.8796 537409 outcome TRUE reported 18343_10_DECR2_DECR2
DECR2 chr16 242773 0.009041 2.041e-50 35314 TRUE reported textfile
2 1 TRUE
2486 rs10399785 C T C T -0.0682 -0.0084 0.49741 NA FALSE FALSE FALSE
GCST006061 0.0072 0.2478 537409 outcome TRUE reported 17400_71_ACOT8_ACOT8
ACOT8 chr1 94412245 0.007851 3.714e-18 35353 TRUE reported
textfile 2 1 TRUE
2487 rs10771511 C T C T -0.0501 -0.0064 0.44238 NA FALSE FALSE FALSE
GCST006061 0.0071 0.3677 537409 outcome TRUE reported 17400_71_ACOT8_ACOT8
ACOT8 chr12 29284284 0.007946 2.878e-10 35365 TRUE reported
textfile 2 1 TRUE
2488 rs10922098 T C T C -0.0504 -0.009 0.40011 NA FALSE FALSE FALSE
GCST006061 0.0075 0.2275 537409 outcome TRUE reported 17400_71_ACOT8_ACOT8
ACOT8 chr1 196695521 0.008049 3.8e-10 35338 TRUE reported textfile
2 1 TRUE
2489 rs11704359 A G A G -0.0822 0.0035 0.13862 NA FALSE FALSE FALSE
GCST006061 0.0109 0.7489 537409 outcome TRUE reported 17400_71_ACOT8_ACOT8
ACOT8 chr22 22325537 0.011464 7.481e-13 35353 TRUE reported
textfile 2 1 TRUE
2490 rs12897483 T G T G -0.0509 0.0072 0.25403 NA FALSE FALSE FALSE
GCST006061 0.0138 0.6038 537409 outcome TRUE reported 17400_71_ACOT8_ACOT8
ACOT8 chr14 106048187 0.009335 4.961e-08 34966 TRUE reported
textfile 2 1 TRUE
2491 rs704 A G A G 0.0508 -0.0155 0.49189 NA FALSE FALSE FALSE GCST006061
0.0072 0.03144 537409 outcome TRUE reported 17400_71_ACOT8_ACOT8 ACOT8
chr17 28367840 0.007907 1.318e-10 35360 TRUE reported textfile 2
1 TRUE
2492 rs7161336 T C T C 0.112 0.0071 0.25928 NA FALSE FALSE FALSE
GCST006061 0.0104 0.494 537409 outcome TRUE reported 17400_71_ACOT8_ACOT8
ACOT8 chr14 106540204 0.009249 9.35e-34 34966 TRUE reported
textfile 2 1 TRUE
2493 rs74770429 C T C T -0.1739 0.0364 0.04468 NA FALSE FALSE FALSE
GCST006061 0.0335 0.2774 537409 outcome TRUE reported 17400_71_ACOT8_ACOT8
ACOT8 chr22 40814137 0.019197 1.321e-19 35371 TRUE reported
textfile 2 1 TRUE
2494 rs10401234 A G A G 0.2913 -0.0049 0.41289 NA FALSE FALSE FALSE
GCST006061 0.0076 0.5242 537409 outcome TRUE reported
16792_4_SIGLEC5_Siglec_5 SIGLEC5 chr19 51548505 0.009435 1e-200 35384
TRUE reported textfile 2 1 TRUE
2495 rs115494840 G T G T -0.5063 0.0018 0.0964 NA FALSE FALSE FALSE
GCST006061 0.0135 0.8957 537409 outcome TRUE reported
16792_4_SIGLEC5_Siglec_5 SIGLEC5 chr19 51477049 0.015636 1e-200 35382
TRUE reported textfile 2 1 TRUE
2496 rs12610918 C A C A -0.1682 -3.00E-04 0.43511 NA FALSE FALSE FALSE
GCST006061 0.0075 0.9664 537409 outcome TRUE reported
16792_4_SIGLEC5_Siglec_5 SIGLEC5 chr19 51752736 0.009424 3.008e-71 35381
TRUE reported textfile 2 1 TRUE
2497 rs2708751 G A G A -0.08 0.0051 0.445 NA FALSE FALSE FALSE
GCST006061 0.0075 0.4973 537409 outcome TRUE reported
16792_4_SIGLEC5_Siglec_5 SIGLEC5 chr19 53446227 0.009443 2.417e-17 35378
TRUE reported textfile 2 1 TRUE

2498 rs35692207 G A G A 0.3105 -0.0079 0.46757 NA FALSE FALSE FALSE GCST006061 0.0074 0.2836 537409 outcome TRUE reported 16792_4_SIGLEC5_Siglec_5 SIGLEC5 chr19 51582593 0.00929 1e-200 35384 TRUE reported textfile 2 1 TRUE

2499 rs3829649 A G A G -0.2824 -0.0069 0.47561 NA FALSE FALSE FALSE GCST006061 0.0078 0.378 537409 outcome TRUE reported 16792_4_SIGLEC5_Siglec_5 SIGLEC5 chr19 51713211 0.009271 1e-200 35377 TRUE reported textfile 2 1 TRUE

2500 rs4802782 A G A G 0.0665 -0.0033 0.4441 NA FALSE FALSE FALSE GCST006061 0.0075 0.6624 537409 outcome TRUE reported 16792_4_SIGLEC5_Siglec_5 SIGLEC5 chr19 51331497 0.009444 1.9e-12 35379 TRUE reported textfile 2 1 TRUE

2501 rs653178 T C T C -0.048 0.0076 0.38124 NA FALSE FALSE FALSE GCST006061 0.0075 0.3129 537409 outcome TRUE reported 16792_4_SIGLEC5_Siglec_5 SIGLEC5 chr12 111569952 0.007763 6.29e-10 35371 TRUE reported textfile 2 1 TRUE

2502 rs10401969 C T C T 0.1031 0.0067 0.07816 NA FALSE FALSE FALSE GCST006061 0.0132 0.611 537409 outcome TRUE reported 4549_78_FUT5_FUT5 FUT5 chr19 19296909 0.01682 8.814e-10 35353 TRUE reported textfile 2 1 TRUE

2503 rs10402728 C T C T -0.0984 -0.014 0.33382 NA FALSE FALSE FALSE GCST006061 0.0082 0.08858 537409 outcome TRUE reported 4549_78_FUT5_FUT5 FUT5 chr19 5833611 0.009472 2.81e-25 35365 TRUE reported textfile 2 1 TRUE

2504 rs10771887 T C T C -0.0628 0.003 0.42154 NA FALSE FALSE FALSE GCST006061 0.0073 0.6806 537409 outcome TRUE reported 4549_78_FUT5_FUT5 FUT5 chr12 31892850 0.008643 3.712e-13 35356 TRUE reported textfile 2 1 TRUE

2505 rs117032075 T C T C -0.8943 -0.0036 0.02648 NA FALSE FALSE FALSE GCST006061 0.0319 0.9115 537409 outcome TRUE reported 4549_78_FUT5_FUT5 FUT5 chr19 5808717 0.027067 1e-200 35360 TRUE reported textfile 2 1 TRUE

2506 rs11878862 G A G A -0.5911 0.0075 0.09987 NA FALSE FALSE FALSE GCST006061 0.0107 0.4825 537409 outcome TRUE reported 4549_78_FUT5_FUT5 FUT5 chr19 5817207 0.010692 1e-200 35363 TRUE reported textfile 2 1 TRUE

2507 rs144449404 T C T C -0.2747 0.0186 0.02852 NA FALSE FALSE FALSE GCST006061 0.0376 0.6207 537409 outcome TRUE reported 4549_78_FUT5_FUT5 FUT5 chr19 5158608 0.027009 2.681e-24 35362 TRUE reported textfile 2 1 TRUE

2508 rs145405806 A G A G 0.5566 -0.1074 0.01781 NA FALSE FALSE FALSE GCST006061 0.0462 0.01993 537409 outcome TRUE reported 4549_78_FUT5_FUT5 FUT5 chr19 5744815 0.034131 8.685e-60 35363 TRUE reported textfile 2 1 TRUE

2509 rs147632697 T C T C -0.1571 0.0687 0.04655 NA FALSE FALSE FALSE GCST006061 0.0282 0.01486 537409 outcome TRUE reported 4549_78_FUT5_FUT5 FUT5 chr19 7221613 0.021073 8.989e-14 35374 TRUE reported textfile 2 1 TRUE

2510 rs1800759 G T G T -0.0511 -0.0048 0.37859 NA FALSE FALSE FALSE GCST006061 0.0074 0.5184 537409 outcome TRUE reported 4549_78_FUT5_FUT5 FUT5 chr4 99144358 0.008805 6.497e-09 35346 TRUE reported textfile 2 1 TRUE

2511 rs1800961 T C T C -0.1232 -4.00E-04 0.04601 NA FALSE FALSE FALSE GCST006061 0.0219 0.9854 537409 outcome TRUE reported 4549_78_FUT5_FUT5 FUT5 chr20 44413724 0.020495 1.842e-09 35371 TRUE reported textfile 2 1 TRUE

2512 rs2393775 A G A G 0.1148 0.0121 0.35369 NA FALSE FALSE FALSE GCST006061 0.0072 0.09297 537409 outcome TRUE reported 4549_78_FUT5_FUT5 FUT5 chr12 120986771 0.0089 4.595e-38 35365 TRUE reported textfile 2 1 TRUE

2513 rs4845623 G A G A -0.0535 0.0412 0.43935 NA FALSE FALSE FALSE GCST006061 0.0072 1.001e-08 537409 outcome TRUE reported 4549_78_FUT5_FUT5 FUT5 chr1 154443301 0.008636 5.817e-10 35364 TRUE reported textfile 2 1 TRUE

2514 rs504963 A G A G -0.0727 0.0056 0.37239 NA FALSE FALSE FALSE GCST006061 0.0075 0.4571 537409 outcome TRUE reported 4549_78_FUT5_FUT5

FUT5 chr19 48705608 0.009313 5.884e-15 35377 TRUE reported
textfile 2 1 TRUE
2515 rs55780081 A G A G -0.1063 -0.0142 0.05852 NA FALSE FALSE FALSE
GCST006061 0.0184 0.4412 537409 outcome TRUE reported 4549_78_FUT5_FUT5
FUT5 chr13 40959359 0.018192 5.115e-09 35359 TRUE reported
textfile 2 1 TRUE
2516 rs73920020 C T C T -0.2055 0.0155 0.07384 NA FALSE FALSE FALSE
GCST006061 0.0141 0.269 537409 outcome TRUE reported 4549_78_FUT5_FUT5
FUT5 chr19 5583503 0.017237 9.064e-33 35366 TRUE reported textfile
2 1 TRUE
2517 rs9738365 A C A C 0.0974 -0.0022 0.23481 NA FALSE FALSE FALSE
GCST006061 0.0081 0.7804 537409 outcome TRUE reported 4549_78_FUT5_FUT5
FUT5 chr12 31844701 0.010102 5.334e-22 35355 TRUE reported
textfile 2 1 TRUE
2518 rs11665816 A G A G 0.1624 -0.016 0.33944 NA FALSE FALSE FALSE
GCST006061 0.0083 0.05262 537409 outcome TRUE reported 9275_2_CTSL_Cathepsin_L
CTSL chr19 52173508 0.0096 3.41e-64 35382 TRUE reported textfile
2 1 TRUE
2519 rs2218886 G T G T -0.2906 -0.0167 0.28422 NA FALSE FALSE FALSE
GCST006061 0.008 0.03768 537409 outcome TRUE reported 9275_2_CTSL_Cathepsin_L
CTSL chr19 51674810 0.009983 2.787e-186 35381 TRUE reported
textfile 2 1 TRUE
2520 rs7258020 T C T C 0.1236 0.0061 0.38742 NA FALSE FALSE FALSE
GCST006061 0.0077 0.4302 537409 outcome TRUE reported 9275_2_CTSL_Cathepsin_L
CTSL chr19 51376905 0.009348 6.576e-40 35376 TRUE reported
textfile 2 1 TRUE
2521 rs75818312 A G A G -0.473 0.0018 0.09445 NA FALSE FALSE FALSE
GCST006061 0.0135 0.8917 537409 outcome TRUE reported 9275_2_CTSL_Cathepsin_L
CTSL chr19 51478711 0.015474 1e-200 35382 TRUE reported textfile
2 1 TRUE
2522 rs12459949 G A G A 0.0653 -0.0144 0.16343 NA FALSE FALSE FALSE
GCST006061 0.0105 0.1735 537409 outcome TRUE reported 3232_28_ACP5_TrATPase
ACP5 chr19 10004515 0.011459 1.208e-08 35371 TRUE reported
textfile 2 1 TRUE
2523 rs139196635 A G A G 0.2797 0.0242 0.02061 NA FALSE FALSE FALSE
GCST006061 0.0312 0.4387 537409 outcome TRUE reported 3232_28_ACP5_TrATPase
ACP5 chr12 101863758 0.029946 9.617e-21 35364 TRUE reported
textfile 2 1 TRUE
2524 rs2073333 T C T C 0.0669 0.0103 0.20754 NA FALSE FALSE FALSE
GCST006061 0.0081 0.2059 537409 outcome TRUE reported 3232_28_ACP5_TrATPase
ACP5 chr14 94378225 0.010366 1.093e-10 35371 TRUE reported
textfile 2 1 TRUE
2525 rs34007487 T C T C 0.07 0.0193 0.23469 NA FALSE FALSE FALSE
GCST006061 0.0092 0.03525 537409 outcome TRUE reported 3232_28_ACP5_TrATPase
ACP5 chr8 104956595 0.009922 1.725e-12 35360 TRUE reported
textfile 2 1 TRUE
2526 rs3780495 C A C A -0.0673 0.0077 0.29174 NA FALSE FALSE FALSE
GCST006061 0.0076 0.3116 537409 outcome TRUE reported 3232_28_ACP5_TrATPase
ACP5 chr9 33125944 0.009188 2.392e-13 35349 TRUE reported
textfile 2 1 TRUE
2527 rs4919420 G A G A 0.0552 -0.0011 0.37537 NA FALSE FALSE FALSE
GCST006061 0.0074 0.8788 537409 outcome TRUE reported 3232_28_ACP5_TrATPase
ACP5 chr10 100046495 0.008656 1.809e-10 35368 TRUE reported
textfile 2 1 TRUE
2528 rs5851 G A G A -0.0843 -0.0081 0.45555 NA FALSE FALSE FALSE GCST006061
0.0073 0.2681 537409 outcome TRUE reported 3232_28_ACP5_TrATPase ACP5
chr19 11721468 0.008449 1.917e-23 35382 TRUE reported textfile 2
1 TRUE
2529 rs76513382 G A G A -0.1494 -0.0444 0.03065 NA FALSE FALSE FALSE
GCST006061 0.0229 0.05294 537409 outcome TRUE reported 3232_28_ACP5_TrATPase
ACP5 chr19 12879688 0.025074 2.548e-09 35381 TRUE reported
textfile 2 1 TRUE
2530 rs77095127 A G A G 0.0986 6e-04 0.07404 NA FALSE FALSE FALSE
GCST006061 0.0144 0.9661 537409 outcome TRUE reported 3232_28_ACP5_TrATPase
ACP5 chr16 79524968 0.01612 9.559e-10 35363 TRUE reported textfile
2 1 TRUE

2531 rs7910087 C T C T 0.0532 -0.0112 0.39936 NA FALSE FALSE FALSE
GCST006061 0.0072 0.1178 537409 outcome TRUE reported 3232_28_ACP5_TrATPase
ACP5 chr10 75449387 0.008585 5.748e-10 35361 TRUE reported
textfile 2 1 TRUE
2532 rs10403676 T G T G -0.0527 0.0133 0.29644 NA FALSE FALSE FALSE
GCST006061 0.008 0.09747 537409 outcome TRUE reported 8097_77_LIPN_LIPN
LIPN chr19 16308554 0.008375 3.121e-10 35363 TRUE reported
textfile 2 1 TRUE
2533 rs1238449 T C T C -0.1586 0.0112 0.46965 NA FALSE FALSE FALSE
GCST006061 0.0071 0.1155 537409 outcome TRUE reported 8097_77_LIPN_LIPN
LIPN chr10 88827092 0.008227 8.262e-83 35357 TRUE reported
textfile 2 1 TRUE
2534 rs17109158 T C T C 0.2876 0.0251 0.02165 NA FALSE FALSE FALSE
GCST006061 0.0272 0.3559 537409 outcome TRUE reported 8097_77_LIPN_LIPN
LIPN chr10 88119009 0.028415 4.442e-24 35356 TRUE reported
textfile 2 1 TRUE
2535 rs186488150 T C T C 0.3693 -0.0286 0.02051 NA FALSE FALSE FALSE
GCST006061 0.063 0.6497 537409 outcome TRUE reported 8097_77_LIPN_LIPN
LIPN chr10 87197547 0.028519 2.377e-38 35356 TRUE reported
textfile 2 1 TRUE
2536 rs2576155 T C T C -0.318 -0.0018 0.29115 NA FALSE FALSE FALSE
GCST006061 0.0083 0.8301 537409 outcome TRUE reported 8097_77_LIPN_LIPN
LIPN chr10 88579836 0.008998 1e-200 35357 TRUE reported textfile
2 1 TRUE
2537 rs265492 A C A C -0.1328 -0.0047 0.03731 NA FALSE FALSE FALSE
GCST006061 0.0308 0.8788 537409 outcome TRUE reported 8097_77_LIPN_LIPN
LIPN chr10 81356642 0.022218 2.271e-09 35373 TRUE reported
textfile 2 1 TRUE
2538 rs28475163 A G A G 0.0509 -0.0082 0.23936 NA FALSE FALSE FALSE
GCST006061 0.0088 0.3494 537409 outcome TRUE reported 8097_77_LIPN_LIPN
LIPN chr11 327143 0.008934 1.216e-08 35342 TRUE reported textfile
2 1 TRUE
2539 rs3746638 A G A G 0.0419 -0.0072 0.45232 NA FALSE FALSE FALSE
GCST006061 0.0072 0.3145 537409 outcome TRUE reported 8097_77_LIPN_LIPN
LIPN chr20 3694686 0.007635 4.066e-08 35373 TRUE reported textfile
2 1 TRUE
2540 rs6065904 A G A G 0.05 -0.013 0.23915 NA FALSE FALSE FALSE
GCST006061 0.0085 0.1241 537409 outcome TRUE reported 8097_77_LIPN_LIPN
LIPN chr20 45906012 0.008901 1.937e-08 35369 TRUE reported
textfile 2 1 TRUE
2541 rs7095217 T C T C 0.4812 -0.0018 0.01941 NA FALSE FALSE FALSE
GCST006061 0.0236 0.94 537409 outcome TRUE reported 8097_77_LIPN_LIPN
LIPN chr10 89820054 0.029839 1.664e-58 35359 TRUE reported
textfile 2 1 TRUE
2542 rs78866702 A G A G 0.1585 -0.022 0.08646 NA FALSE FALSE FALSE
GCST006061 0.0172 0.2012 537409 outcome TRUE reported 8097_77_LIPN_LIPN
LIPN chr10 90288091 0.014728 5.211e-27 35360 TRUE reported
textfile 2 1 TRUE
2543 rs7918518 T C T C 0.0834 0.011 0.24946 NA FALSE FALSE FALSE
GCST006061 0.0084 0.191 537409 outcome TRUE reported 8097_77_LIPN_LIPN
LIPN chr10 86101625 0.009519 1.932e-18 35364 TRUE reported
textfile 2 1 TRUE
2544 rs9970784 T C T C -0.0525 -0.0129 0.43157 NA FALSE FALSE FALSE
GCST006061 0.0074 0.08036 537409 outcome TRUE reported 8097_77_LIPN_LIPN
LIPN chr1 196704672 0.007667 7.511e-12 35339 TRUE reported
textfile 2 1 TRUE
2545 rs1040402 A G A G -0.1851 0.0184 0.19092 NA FALSE FALSE FALSE
GCST006061 0.0094 0.05028 537409 outcome TRUE reported 6470_19_FBLN1_fibulin_1
FBLN1 chr22 45451084 0.010605 3.227e-68 35370 TRUE reported
textfile 2 1 TRUE
2546 rs11130306 G A G A -0.0677 0.0055 0.45979 NA FALSE FALSE FALSE
GCST006061 0.0071 0.4384 537409 outcome TRUE reported 6470_19_FBLN1_fibulin_1
FBLN1 chr3 52507237 0.008418 8.839e-16 35350 TRUE reported
textfile 2 1 TRUE
2547 rs115578531 G A G A 0.2024 0.0546 0.01424 NA FALSE FALSE FALSE
GCST006061 0.051 0.2849 537409 outcome TRUE reported 6470_19_FBLN1_fibulin_1

FBLN1 chr3 51398350 0.034987 7.249e-09 35342 TRUE reported
textfile 2 1 TRUE
2548 rs1260326 C T C T 0.0643 -0.0069 0.34132 NA FALSE FALSE FALSE
GCST006061 0.0073 0.3401 537409 outcome TRUE reported 6470_19_FBLN1_fibulin_1
FBLN1 chr2 27508073 0.008845 3.608e-13 35373 TRUE reported
textfile 2 1 TRUE
2549 rs136743 C A C A 0.0689 0.0071 0.44531 NA FALSE FALSE FALSE
GCST006061 0.0074 0.3374 537409 outcome TRUE reported 6470_19_FBLN1_fibulin_1
FBLN1 chr22 45532866 0.008469 4.105e-16 35369 TRUE reported
textfile 2 1 TRUE
2550 rs141359357 A G A G 0.1551 -0.022 0.03367 NA FALSE FALSE FALSE
GCST006061 0.0238 0.355 537409 outcome TRUE reported 6470_19_FBLN1_fibulin_1
FBLN1 chr3 53161104 0.023476 3.929e-11 35343 TRUE reported
textfile 2 1 TRUE
2551 rs145887694 G T G T 0.2124 -0.0117 0.01977 NA FALSE FALSE FALSE
GCST006061 0.0305 0.7001 537409 outcome TRUE reported 6470_19_FBLN1_fibulin_1
FBLN1 chr3 53630094 0.030602 3.9e-12 35350 TRUE reported textfile
2 1 TRUE
2552 rs183784507 T C T C 0.2026 0.0114 0.01631 NA FALSE FALSE FALSE
GCST006061 0.0355 0.7481 537409 outcome TRUE reported 6470_19_FBLN1_fibulin_1
FBLN1 chr3 45379552 0.032251 3.345e-10 35346 TRUE reported
textfile 2 1 TRUE
2553 rs333947 A G A G -0.0672 -0.0071 0.14937 NA FALSE FALSE FALSE
GCST006061 0.01 0.4808 537409 outcome TRUE reported 6470_19_FBLN1_fibulin_1
FBLN1 chr1 109928142 0.011811 1.275e-08 35349 TRUE reported
textfile 2 1 TRUE
2554 rs56344867 C T C T 0.055 -0.005 0.35465 NA FALSE FALSE FALSE
GCST006061 0.008 0.5298 537409 outcome TRUE reported 6470_19_FBLN1_fibulin_1
FBLN1 chr11 126361219 0.008744 3.168e-10 35374 TRUE reported
textfile 2 1 TRUE
2555 rs71313011 G A G A -0.1942 0.051 0.02264 NA FALSE FALSE FALSE
GCST006061 0.0434 0.2402 537409 outcome TRUE reported 6470_19_FBLN1_fibulin_1
FBLN1 chr22 45478207 0.027878 3.257e-12 35371 TRUE reported
textfile 2 1 TRUE
2556 rs73215978 C T C T -0.103 -0.0152 0.05534 NA FALSE FALSE FALSE
GCST006061 0.0168 0.365 537409 outcome TRUE reported 6470_19_FBLN1_fibulin_1
FBLN1 chr3 132391608 0.018683 3.529e-08 35356 TRUE reported
textfile 2 1 TRUE
2557 rs74814272 G A G A 0.207 0.0247 0.01224 NA FALSE FALSE FALSE
GCST006061 0.0362 0.4957 537409 outcome TRUE reported 6470_19_FBLN1_fibulin_1
FBLN1 chr3 41080294 0.037322 2.918e-08 35353 TRUE reported
textfile 2 1 TRUE
2558 rs10405357 C T C T -0.0461 -0.0077 0.44277 NA FALSE FALSE FALSE
GCST006061 0.0078 0.3285 537409 outcome TRUE reported
3600_2_CHIT1_Chitotriosidase_1 CHIT1 chr19 54255803 0.00836 3.503e-08 35371
TRUE reported textfile 2 1 TRUE
2559 rs10793756 G A G A -0.2152 0.0034 0.03287 NA FALSE FALSE FALSE
GCST006061 0.0174 0.8444 537409 outcome TRUE reported
3600_2_CHIT1_Chitotriosidase_1 CHIT1 chr1 203658706 0.025938 1.071e-16
35367 TRUE reported textfile 2 1 TRUE
2560 rs114066783 A G A G -0.3749 -0.0053 0.03476 NA FALSE FALSE FALSE
GCST006061 0.0233 0.8211 537409 outcome TRUE reported
3600_2_CHIT1_Chitotriosidase_1 CHIT1 chr1 203515747 0.02593 2.224e-47 35364
TRUE reported textfile 2 1 TRUE
2561 rs115402627 A G A G 0.286 0.0339 0.02883 NA FALSE FALSE FALSE
GCST006061 0.0315 0.2811 537409 outcome TRUE reported
3600_2_CHIT1_Chitotriosidase_1 CHIT1 chr1 203073657 0.027903 1.184e-24
35362 TRUE reported textfile 2 1 TRUE
2562 rs116201986 T C T C -0.3447 0.0054 0.0255 NA FALSE FALSE FALSE
GCST006061 0.0275 0.8459 537409 outcome TRUE reported
3600_2_CHIT1_Chitotriosidase_1 CHIT1 chr1 201665027 0.029492 1.47e-31
35353 TRUE reported textfile 2 1 TRUE
2563 rs143841225 T C T C 0.1676 0.0177 0.02362 NA FALSE FALSE FALSE
GCST006061 0.031 0.5667 537409 outcome TRUE reported
3600_2_CHIT1_Chitotriosidase_1 CHIT1 chr1 196637400 0.030061 2.471e-08
35338 TRUE reported textfile 2 1 TRUE

2564 rs148541330 G A G A 1.0241 -0.0613 0.01215 NA FALSE FALSE FALSE
GCST006061 0.042 0.144 537409 outcome TRUE reported
3600_2_CHIT1_Chitotriosidase_1 CHIT1 chr1 203240346 0.043117 1.062e-124
35362 TRUE reported textfile 2 1 TRUE
2565 rs149820561 T C T C -0.3472 0.0566 0.01714 NA FALSE FALSE FALSE
GCST006061 0.0538 0.2924 537409 outcome TRUE reported
3600_2_CHIT1_Chitotriosidase_1 CHIT1 chr1 202311286 0.035586 1.729e-22
35361 TRUE reported textfile 2 1 TRUE
2566 rs190770651 A G A G 0.2686 0.045 0.0112 NA FALSE FALSE FALSE
GCST006061 0.0703 0.5219 537409 outcome TRUE reported
3600_2_CHIT1_Chitotriosidase_1 CHIT1 chr1 198444488 0.044874 2.156e-09
35344 TRUE reported textfile 2 1 TRUE
2567 rs3766562 T C T C -0.7095 -0.0233 0.05194 NA FALSE FALSE FALSE
GCST006061 0.0149 0.1185 537409 outcome TRUE reported
3600_2_CHIT1_Chitotriosidase_1 CHIT1 chr1 203142755 0.020546 1e-200 35363
TRUE reported textfile 2 1 TRUE
2568 rs3862944 C T C T 0.3568 0.0039 0.16415 NA FALSE FALSE FALSE
GCST006061 0.0095 0.6849 537409 outcome TRUE reported
3600_2_CHIT1_Chitotriosidase_1 CHIT1 chr1 203214885 0.012545 6.198e-178
35365 TRUE reported textfile 2 1 TRUE
2569 rs79991585 A G A G 0.1608 -0.0158 0.0375 NA FALSE FALSE FALSE
GCST006061 0.0215 0.4631 537409 outcome TRUE reported
3600_2_CHIT1_Chitotriosidase_1 CHIT1 chr1 202129368 0.024739 8.038e-11
35356 TRUE reported textfile 2 1 TRUE
2570 rs10405357 C T C T -0.0824 -0.0077 0.44277 NA FALSE FALSE FALSE
GCST006061 0.0078 0.3285 537409 outcome TRUE reported 18841_1_SERPINB13_SPB13
SERPINB13 chr19 54255803 0.008255 1.826e-23 35371 TRUE reported
textfile 2 1 TRUE
2571 rs118015107 T C T C -0.2808 0.011 0.01387 NA FALSE FALSE FALSE
GCST006061 0.034 0.7452 537409 outcome TRUE reported 18841_1_SERPINB13_SPB13
SERPINB13 chr18 63534907 0.035122 1.296e-15 35368 TRUE reported
textfile 2 1 TRUE
2572 rs1260326 C T C T 0.0542 -0.0069 0.34132 NA FALSE FALSE FALSE
GCST006061 0.0073 0.3401 537409 outcome TRUE reported 18841_1_SERPINB13_SPB13
SERPINB13 chr2 27508073 0.008672 4.114e-10 35373 TRUE reported
textfile 2 1 TRUE
2573 rs17756365 C T C T -0.1481 0.0087 0.02353 NA FALSE FALSE FALSE
GCST006061 0.0257 0.7356 537409 outcome TRUE reported 18841_1_SERPINB13_SPB13
SERPINB13 chr18 63151579 0.02664 2.71e-08 35366 TRUE reported
textfile 2 1 TRUE
2574 rs492602 G A G A 0.1348 0.0055 0.39439 NA FALSE FALSE FALSE
GCST006061 0.0076 0.4635 537409 outcome TRUE reported 18841_1_SERPINB13_SPB13
SERPINB13 chr19 48703160 0.008382 3.418e-58 35377 TRUE reported
textfile 2 1 TRUE
2575 rs72704449 C T C T 0.1099 0.013 0.04582 NA FALSE FALSE FALSE
GCST006061 0.0199 0.5128 537409 outcome TRUE reported 18841_1_SERPINB13_SPB13
SERPINB13 chr1 179504138 0.019487 1.704e-08 35358 TRUE reported
textfile 2 1 TRUE
2576 rs77157727 A G A G -0.4352 -0.0356 0.04297 NA FALSE FALSE FALSE
GCST006061 0.0234 0.1283 537409 outcome TRUE reported 18841_1_SERPINB13_SPB13
SERPINB13 chr18 63586727 0.019515 3.602e-110 35368 TRUE reported
textfile 2 1 TRUE
2577 rs77924615 A G A G -0.0568 0.0136 0.19701 NA FALSE FALSE FALSE
GCST006061 0.0092 0.1375 537409 outcome TRUE reported 18841_1_SERPINB13_SPB13
SERPINB13 chr16 20381010 0.010351 4.075e-08 35355 TRUE reported
textfile 2 1 TRUE
2578 rs10405535 G A G A 0.0482 0.0026 0.3249 NA FALSE FALSE FALSE
GCST006061 0.0083 0.7567 537409 outcome TRUE reported 18897_31_HDAC2_HDAC2
HDAC2 chr19 32581179 0.008698 3.002e-08 35367 TRUE reported
textfile 2 1 TRUE
2579 rs114694170 C T C T 0.0806 0.0053 0.08378 NA FALSE FALSE FALSE
GCST006061 0.0175 0.7624 537409 outcome TRUE reported 18897_31_HDAC2_HDAC2
HDAC2 chr5 88884379 0.014678 3.991e-08 35344 TRUE reported
textfile 2 1 TRUE
2580 rs1354034 C T C T 0.117 0.0088 0.38119 NA FALSE FALSE FALSE
GCST006061 0.0073 0.2248 537409 outcome TRUE reported 18897_31_HDAC2_HDAC2

HDAC2 chr3 56815721 0.008308 4.872e-45 35356 TRUE reported
textfile 2 1 TRUE

2581 rs4980320 C T C T 0.0637 0.0041 0.46986 NA FALSE FALSE FALSE
GCST006061 0.0076 0.5858 537409 outcome TRUE reported 18897_31_HDAC2_HDAC2
HDAC2 chr11 198986 0.008108 3.938e-15 35352 TRUE reported textfile
2 1 TRUE

2582 rs10405535 G A G A 0.074 0.0026 0.3249 NA FALSE FALSE FALSE
GCST006061 0.0083 0.7567 537409 outcome TRUE reported 4271_75_PFDN5_PFD5
PFDN5 chr19 32581179 0.008524 3.899e-18 35367 TRUE reported
textfile 2 1 TRUE

2583 rs1329424 G T G T -0.1236 -0.0095 0.39735 NA FALSE FALSE FALSE
GCST006061 0.0075 0.2071 537409 outcome TRUE reported 4271_75_PFDN5_PFD5
PFDN5 chr1 196677046 0.008095 1.231e-52 35338 TRUE reported
textfile 2 1 TRUE

2584 rs1706435 G A G A 0.0601 0.0117 0.36981 NA FALSE FALSE FALSE
GCST006061 0.0072 0.1047 537409 outcome TRUE reported 4271_75_PFDN5_PFD5
PFDN5 chr3 165743688 0.0082 2.308e-13 35339 TRUE reported textfile
2 1 TRUE

2585 rs10405535 G A G A 0.0769 0.0026 0.3249 NA FALSE FALSE FALSE
GCST006061 0.0083 0.7567 537409 outcome TRUE reported 17383_4_STARD5_STAR5
STARD5 chr19 32581179 0.008675 7.68e-19 35367 TRUE reported
textfile 2 1 TRUE

2586 rs12910527 C T C T -0.2557 0.0285 0.01017 NA FALSE FALSE FALSE
GCST006061 0.0346 0.4111 537409 outcome TRUE reported 17383_4_STARD5_STAR5
STARD5 chr15 80728206 0.038923 5.052e-11 35359 TRUE reported
textfile 2 1 TRUE

2587 rs1354034 C T C T 0.0488 0.0088 0.38119 NA FALSE FALSE FALSE
GCST006061 0.0073 0.2248 537409 outcome TRUE reported 17383_4_STARD5_STAR5
STARD5 chr3 56815721 0.008304 4.183e-09 35356 TRUE reported
textfile 2 1 TRUE

2588 rs4392019 T C T C 0.7511 -0.0091 0.01382 NA FALSE FALSE FALSE
GCST006061 0.0194 0.6392 537409 outcome TRUE reported 17383_4_STARD5_STAR5
STARD5 chr15 81324218 0.034117 2.037e-107 35357 TRUE reported
textfile 2 1 TRUE

2589 rs10405535 G A G A 0.0929 0.0026 0.3249 NA FALSE FALSE FALSE
GCST006061 0.0083 0.7567 537409 outcome TRUE reported 3170_6_METAP2_AMPM2
METAP2 chr19 32581179 0.008807 5.139e-26 35367 TRUE reported
textfile 2 1 TRUE

2590 rs1354034 C T C T 0.0906 0.0088 0.38119 NA FALSE FALSE FALSE
GCST006061 0.0073 0.2248 537409 outcome TRUE reported 3170_6_METAP2_AMPM2
METAP2 chr3 56815721 0.008432 6.284e-27 35356 TRUE reported
textfile 2 1 TRUE

2591 rs4541868 A C A C -0.0629 0.0211 0.21915 NA FALSE FALSE FALSE
GCST006061 0.0082 0.01029 537409 outcome TRUE reported 3170_6_METAP2_AMPM2
METAP2 chr8 105578477 0.009921 2.295e-10 35364 TRUE reported
textfile 2 1 TRUE

2592 rs4980320 C T C T 0.0566 0.0041 0.46986 NA FALSE FALSE FALSE
GCST006061 0.0076 0.5858 537409 outcome TRUE reported 3170_6_METAP2_AMPM2
METAP2 chr11 198986 0.008224 5.898e-12 35352 TRUE reported textfile
2 1 TRUE

2593 rs78909033 A G A G 0.086 -0.0167 0.16064 NA FALSE FALSE FALSE
GCST006061 0.0117 0.1526 537409 outcome TRUE reported 3170_6_METAP2_AMPM2
METAP2 chr2 240571486 0.011177 1.424e-14 35270 TRUE reported
textfile 2 1 TRUE

2594 rs10406069 A G A G -0.0722 0.0096 0.22596 NA FALSE FALSE FALSE
GCST006061 0.0092 0.2971 537409 outcome TRUE reported 8074_32_TMEM70_TMM70
TMEM70 chr19 35345627 0.009549 3.998e-14 35369 TRUE reported
textfile 2 1 TRUE

2595 rs16864367 A G A G -0.0692 -0.0137 0.29477 NA FALSE FALSE FALSE
GCST006061 0.008 0.08625 537409 outcome TRUE reported 8074_32_TMEM70_TMM70
TMEM70 chr3 152516377 0.008756 2.717e-15 35348 TRUE reported
textfile 2 1 TRUE

2596 rs2317231 T G T G 0.0598 -0.008 0.41244 NA FALSE FALSE FALSE
GCST006061 0.0072 0.2679 537409 outcome TRUE reported 8074_32_TMEM70_TMM70
TMEM70 chr1 157716547 0.008115 1.718e-13 35365 TRUE reported
textfile 2 1 TRUE

2597 rs4707609 C T C T 0.0645 -0.0044 0.34794 NA FALSE FALSE FALSE
GCST006061 0.0076 0.5671 537409 outcome TRUE reported 8074_32_TMEM70_TMM70
TMEM70 chr6 90236760 0.008463 2.502e-14 35344 TRUE reported
textfile 2 1 TRUE
2598 rs10407046 C T C T -0.0591 0.0058 0.24405 NA FALSE FALSE FALSE
GCST006061 0.0083 0.4842 537409 outcome TRUE reported 9335_28_PSG9_PSG9
PSG9 chr19 43412402 0.009039 6.213e-11 35361 TRUE reported
textfile 2 1 TRUE
2599 rs117199551 A G A G 0.1367 -0.0214 0.04404 NA FALSE FALSE FALSE
GCST006061 0.0219 0.3281 537409 outcome TRUE reported 9335_28_PSG9_PSG9
PSG9 chr19 41956501 0.018935 5.221e-13 35370 TRUE reported
textfile 2 1 TRUE
2600 rs117875851 T G T G 0.1068 -0.0244 0.06271 NA FALSE FALSE FALSE
GCST006061 0.0217 0.2611 537409 outcome TRUE reported 9335_28_PSG9_PSG9
PSG9 chr19 41765662 0.016355 6.568e-11 35370 TRUE reported
textfile 2 1 TRUE
2601 rs80142449 A G A G 0.2102 0.0017 0.01448 NA FALSE FALSE FALSE
GCST006061 0.0417 0.9666 537409 outcome TRUE reported 9335_28_PSG9_PSG9
PSG9 chr19 41207084 0.031248 1.735e-11 35371 TRUE reported
textfile 2 1 TRUE
2602 rs117778189 C A C A -0.1902 0.0341 0.02372 NA FALSE FALSE FALSE
GCST006061 0.0276 0.217 537409 outcome TRUE reported
2834_54_KLK8_kallikrein_8 KLK8 chr19 49145774 0.028357 1.982e-11 35377
TRUE reported textfile 2 1 TRUE
2603 rs143815637 T C T C -0.2101 0.0201 0.01421 NA FALSE FALSE FALSE
GCST006061 0.1317 0.8786 537409 outcome TRUE reported
2834_54_KLK8_kallikrein_8 KLK8 chr19 50834532 0.036338 7.387e-09 35371
TRUE reported textfile 2 1 TRUE
2604 rs145673783 T C T C 0.1795 -0.0617 0.02799 NA FALSE FALSE FALSE
GCST006061 0.0296 0.03711 537409 outcome TRUE reported
2834_54_KLK8_kallikrein_8 KLK8 chr19 51178226 0.025345 1.418e-12 35374
TRUE reported textfile 2 1 TRUE
2605 rs2739412 C A C A 0.0487 0.0211 0.38502 NA FALSE FALSE FALSE
GCST006061 0.0076 0.005603 537409 outcome TRUE reported
2834_54_KLK8_kallikrein_8 KLK8 chr19 50952558 0.008662 1.885e-08 35360
TRUE reported textfile 2 1 TRUE
2606 rs45533637 C T C T 0.2293 -0.0483 0.01743 NA FALSE FALSE FALSE
GCST006061 0.0352 0.1698 537409 outcome TRUE reported
2834_54_KLK8_kallikrein_8 KLK8 chr19 50793881 0.031852 6.068e-13 35363
TRUE reported textfile 2 1 TRUE
2607 rs6445035 A G A G 0.0623 0.0154 0.1904 NA FALSE FALSE FALSE
GCST006061 0.0089 0.08256 537409 outcome TRUE reported
2834_54_KLK8_kallikrein_8 KLK8 chr3 165762312 0.01062 4.46e-09 35339
TRUE reported textfile 2 1 TRUE
2608 rs74705037 A G A G 0.8629 0.0189 0.02708 NA FALSE FALSE FALSE
GCST006061 0.0245 0.4403 537409 outcome TRUE reported
2834_54_KLK8_kallikrein_8 KLK8 chr19 51001552 0.025492 1e-200 35371
TRUE reported textfile 2 1 TRUE
2609 rs10922098 T C T C -0.1476 -0.009 0.40011 NA FALSE FALSE FALSE
GCST006061 0.0075 0.2275 537409 outcome TRUE reported 12587_65_ARL2_ARL2
ARL2 chr1 196695521 0.008612 7.652e-66 35329 TRUE reported
textfile 2 1 TRUE
2610 rs111696008 A G A G -0.2047 -0.0259 0.02443 NA FALSE FALSE FALSE
GCST006061 0.0401 0.519 537409 outcome TRUE reported 12587_65_ARL2_ARL2
ARL2 chr2 216622986 0.028081 3.108e-13 35350 TRUE reported
textfile 2 1 TRUE
2611 rs113280663 T C T C -0.0962 -0.0107 0.1573 NA FALSE FALSE FALSE
GCST006061 0.01 0.2833 537409 outcome TRUE reported 12587_65_ARL2_ARL2
ARL2 chr19 45894401 0.012028 1.266e-15 35362 TRUE reported
textfile 2 1 TRUE
2612 rs144202860 A G A G -0.2707 -0.0629 0.01274 NA FALSE FALSE FALSE
GCST006061 0.0538 0.2423 537409 outcome TRUE reported 12587_65_ARL2_ARL2
ARL2 chr2 216625740 0.037585 5.916e-13 35350 TRUE reported
textfile 2 1 TRUE
2613 rs174536 C A C A -0.0572 0.0085 0.39086 NA FALSE FALSE FALSE
GCST006061 0.0075 0.256 537409 outcome TRUE reported 12587_65_ARL2_ARL2

ARL2 chr11 61784455 0.008639 3.567e-11 35358 TRUE reported
textfile 2 1 TRUE
2614 rs2686395 C T C T -0.1653 -0.0077 0.3101 NA FALSE FALSE FALSE
GCST006061 0.0074 0.2965 537409 outcome TRUE reported 12587_65_ARL2_ARL2
ARL2 chr3 165767231 0.009087 6.092e-74 35330 TRUE reported
textfile 2 1 TRUE
2615 rs406315 A G A G 0.1628 -0.0026 0.36876 NA FALSE FALSE FALSE
GCST006061 0.0078 0.7394 537409 outcome TRUE reported 12587_65_ARL2_ARL2
ARL2 chr19 44880859 0.009012 6.091e-73 35358 TRUE reported
textfile 2 1 TRUE
2616 rs55923289 C T C T -0.2016 -0.0094 0.17219 NA FALSE FALSE FALSE
GCST006061 0.0115 0.4142 537409 outcome TRUE reported 12587_65_ARL2_ARL2
ARL2 chr19 44737327 0.011469 3.605e-69 35360 TRUE reported
textfile 2 1 TRUE
2617 rs73169117 T C T C 0.0782 0.0051 0.11405 NA FALSE FALSE FALSE
GCST006061 0.0122 0.6755 537409 outcome TRUE reported 12587_65_ARL2_ARL2
ARL2 chr3 167217620 0.013387 5.171e-09 35345 TRUE reported
textfile 2 1 TRUE
2618 rs78264306 G T G T -0.2149 -0.0789 0.01635 NA FALSE FALSE FALSE
GCST006061 0.0458 0.08521 537409 outcome TRUE reported 12587_65_ARL2_ARL2
ARL2 chr19 43873053 0.033649 1.698e-10 35363 TRUE reported
textfile 2 1 TRUE
2619 rs111420738 G A G A -0.1833 0.0045 0.09006 NA FALSE FALSE FALSE
GCST006061 0.0137 0.7434 537409 outcome TRUE reported 12501_10_TBCA_TBCA
TBCA chr19 45482274 0.016282 2.119e-29 35362 TRUE reported
textfile 2 1 TRUE
2620 rs11668861 T G T G 0.3058 0.0017 0.46793 NA FALSE FALSE FALSE
GCST006061 0.0073 0.8169 537409 outcome TRUE reported 12501_10_TBCA_TBCA
TBCA chr19 44877713 0.009236 1e-200 35357 TRUE reported textfile
2 1 TRUE
2621 rs117884013 T C T C -0.4501 -0.0391 0.0101 NA FALSE FALSE FALSE
GCST006061 0.0481 0.4161 537409 outcome TRUE reported 12501_10_TBCA_TBCA
TBCA chr19 46538512 0.048931 3.624e-20 35360 TRUE reported
textfile 2 1 TRUE
2622 rs12978968 T C T C -0.073 -0.0058 0.29051 NA FALSE FALSE FALSE
GCST006061 0.0087 0.507 537409 outcome TRUE reported 12501_10_TBCA_TBCA
TBCA chr19 43340025 0.010294 1.327e-12 35351 TRUE reported
textfile 2 1 TRUE
2623 rs174536 C A C A -0.0759 0.0085 0.39086 NA FALSE FALSE FALSE
GCST006061 0.0075 0.256 537409 outcome TRUE reported 12501_10_TBCA_TBCA
TBCA chr11 61784455 0.008739 3.796e-18 35358 TRUE reported
textfile 2 1 TRUE
2624 rs2075649 G A G A 0.3966 -0.0104 0.40218 NA FALSE FALSE FALSE
GCST006061 0.0074 0.1623 537409 outcome TRUE reported 12501_10_TBCA_TBCA
TBCA chr19 44892073 0.006504 1e-200 35357 TRUE reported textfile
2 1 TRUE
2625 rs2722757 G A G A 0.0971 -0.0114 0.39365 NA FALSE FALSE FALSE
GCST006061 0.0077 0.1364 537409 outcome TRUE reported 12501_10_TBCA_TBCA
TBCA chr19 44385498 0.009542 2.525e-24 35360 TRUE reported
textfile 2 1 TRUE
2626 rs55923289 C T C T -0.3724 -0.0094 0.17219 NA FALSE FALSE FALSE
GCST006061 0.0115 0.4142 537409 outcome TRUE reported 12501_10_TBCA_TBCA
TBCA chr19 44737327 0.012199 1e-200 35360 TRUE reported textfile
2 1 TRUE
2627 rs60819212 A G A G -0.0729 0.0214 0.15772 NA FALSE FALSE FALSE
GCST006061 0.0112 0.05669 537409 outcome TRUE reported 12501_10_TBCA_TBCA
TBCA chr19 47823499 0.012716 9.88e-09 35364 TRUE reported
textfile 2 1 TRUE
2628 rs10408682 T C T C -0.0833 0.0091 0.12699 NA FALSE FALSE FALSE
GCST006061 0.0108 0.399 537409 outcome TRUE reported 2683_1_C3_iC3b C3
chr19 6699319 0.012249 1.044e-11 35372 TRUE reported textfile 2 1
TRUE
2629 rs12975366 C T C T 0.0478 -0.0089 0.4155 NA FALSE FALSE FALSE
GCST006061 0.0081 0.2688 537409 outcome TRUE reported 2683_1_C3_iC3b C3
chr19 54255498 0.00829 8.121e-09 35370 TRUE reported textfile 2 1
TRUE

2630 rs10409425 T C T C -0.0748 0.0036 0.10219 NA FALSE FALSE FALSE GCST006061 0.012 0.7647 537409 outcome TRUE reported 3438_10_FSTL3_FSTL3 FSTL3 chr19 614865 0.013646 4.221e-08 35312 TRUE reported textfile 2 1 TRUE
2631 rs56014328 G A G A -0.2394 0.0965 0.01744 NA FALSE FALSE FALSE GCST006061 0.073 0.1865 537409 outcome TRUE reported 3438_10_FSTL3_FSTL3 FSTL3 chr19 571044 0.031551 3.257e-14 35305 TRUE reported textfile 2 1 TRUE
2632 rs7250835 C T C T 0.0965 0.0048 0.16751 NA FALSE FALSE FALSE GCST006061 0.0106 0.6491 537409 outcome TRUE reported 3438_10_FSTL3_FSTL3 FSTL3 chr19 670234 0.01109 3.281e-18 35313 TRUE reported textfile 2 1 TRUE
2633 rs72704449 C T C T 0.1221 0.013 0.04582 NA FALSE FALSE FALSE GCST006061 0.0199 0.5128 537409 outcome TRUE reported 3438_10_FSTL3_FSTL3 FSTL3 chr1 179504138 0.019681 5.509e-10 35358 TRUE reported textfile 2 1 TRUE
2634 rs77924615 A G A G -0.0661 0.0136 0.19701 NA FALSE FALSE FALSE GCST006061 0.0092 0.1375 537409 outcome TRUE reported 3438_10_FSTL3_FSTL3 FSTL3 chr16 20381010 0.010464 2.665e-10 35355 TRUE reported textfile 2 1 TRUE
2635 rs116855556 T C T C -0.1289 -0.0277 0.07042 NA FALSE FALSE FALSE GCST006061 0.0233 0.2343 537409 outcome TRUE reported 13671_40_ELANE_Elastase ELANE chr9 79608742 0.016758 1.45e-14 35352 TRUE reported textfile 2 1 TRUE
2636 rs12903325 G T G T -0.0735 0.017 0.25867 NA FALSE FALSE FALSE GCST006061 0.0083 0.04074 537409 outcome TRUE reported 13671_40_ELANE_Elastase ELANE chr15 50061080 0.009667 2.881e-14 35359 TRUE reported textfile 2 1 TRUE
2637 rs13382133 T C T C 0.0507 -0.0125 0.47336 NA FALSE FALSE FALSE GCST006061 0.0075 0.09609 537409 outcome TRUE reported 13671_40_ELANE_Elastase ELANE chr19 17748127 0.008678 5.147e-09 35353 TRUE reported textfile 2 1 TRUE
2638 rs141548740 G A G A 0.128 -0.0124 0.05881 NA FALSE FALSE FALSE GCST006061 0.0369 0.7377 537409 outcome TRUE reported 13671_40_ELANE_Elastase ELANE chr19 1335789 0.018551 5.206e-12 35315 TRUE reported textfile 2 1 TRUE
2639 rs145870849 A G A G 0.1828 0.022 0.02223 NA FALSE FALSE FALSE GCST006061 0.0313 0.4831 537409 outcome TRUE reported 13671_40_ELANE_Elastase ELANE chr17 58248410 0.028713 1.935e-10 35369 TRUE reported textfile 2 1 TRUE
2640 rs186642113 A G A G 0.2097 0.0313 0.01741 NA FALSE FALSE FALSE GCST006061 0.033 0.3427 537409 outcome TRUE reported 13671_40_ELANE_Elastase ELANE chr19 1050344 0.034033 7.201e-10 35322 TRUE reported textfile 2 1 TRUE
2641 rs2380606 C T C T 0.0573 -0.007 0.47568 NA FALSE FALSE FALSE GCST006061 0.0072 0.3283 537409 outcome TRUE reported 13671_40_ELANE_Elastase ELANE chr8 69828661 0.008551 2.068e-11 35362 TRUE reported textfile 2 1 TRUE
2642 rs351970 C T C T -0.1295 -0.0043 0.40789 NA FALSE FALSE FALSE GCST006061 0.0085 0.6118 537409 outcome TRUE reported 13671_40_ELANE_Elastase ELANE chr19 792199 0.008794 4.35e-49 35308 TRUE reported textfile 2 1 TRUE
2643 rs6605578 C T C T -0.0603 0.003 0.38175 NA FALSE FALSE FALSE GCST006061 0.0075 0.6901 537409 outcome TRUE reported 13671_40_ELANE_Elastase ELANE chr8 7023441 0.008799 7.212e-12 35392 TRUE reported textfile 2 1 TRUE
2644 rs7502514 G A G A -0.0681 0.0189 0.37292 NA FALSE FALSE FALSE GCST006061 0.0073 0.009352 537409 outcome TRUE reported 13671_40_ELANE_Elastase ELANE chr17 40032591 0.008864 1.553e-14 35378 TRUE reported textfile 2 1 TRUE
2645 rs76428106 C T C T 0.2055 -0.0446 0.01359 NA FALSE FALSE FALSE GCST006061 0.0438 0.3085 537409 outcome TRUE reported 13671_40_ELANE_Elastase ELANE chr13 28029870 0.03714 3.145e-08 35363 TRUE reported textfile 2 1 TRUE
2646 rs10410923 A G A G 0.0715 -0.0211 0.12079 NA FALSE FALSE FALSE GCST006061 0.0111 0.05667 537409 outcome TRUE reported 8376_25_LHB_LSHB LHB

chr19 49576504 0.012163 4.137e-09 35375 TRUE reported textfile 2
1 TRUE
2647 rs11669977 G A G A -0.1024 -0.0015 0.1931 NA FALSE FALSE FALSE
GCST006061 0.0096 0.8732 537409 outcome TRUE reported 8376_25_LHB_LSHB LHB
chr19 49060867 0.0101 3.731e-24 35377 TRUE reported textfile 2 1
TRUE
2648 rs7409311 A G A G -0.4324 -0.0061 0.1142 NA FALSE FALSE FALSE
GCST006061 0.0144 0.6716 537409 outcome TRUE reported 8376_25_LHB_LSHB LHB
chr19 48989565 0.012407 1e-200 35377 TRUE reported textfile 2 1
TRUE
2649 rs10411016 T G T G -0.0755 -0.0067 0.45455 NA FALSE FALSE FALSE
GCST006061 0.008 0.4027 537409 outcome TRUE reported 3029_52_CD209_DC_SIGN
CD209 chr19 7680157 0.008083 9.612e-21 35371 TRUE reported textfile
2 1 TRUE
2650 rs10441802 T C T C -0.0796 -0.0088 0.11703 NA FALSE FALSE FALSE
GCST006061 0.0139 0.5288 537409 outcome TRUE reported 3029_52_CD209_DC_SIGN
CD209 chr9 131384575 0.01353 4.021e-09 35371 TRUE reported textfile
2 1 TRUE
2651 rs10821524 C A C A 0.0633 0.0053 0.42012 NA FALSE FALSE FALSE
GCST006061 0.0073 0.4653 537409 outcome TRUE reported 3029_52_CD209_DC_SIGN
CD209 chr9 133789431 0.008831 7.627e-13 35370 TRUE reported
textfile 2 1 TRUE
2652 rs11244084 T C T C 0.565 -0.0172 0.05861 NA FALSE FALSE FALSE
GCST006061 0.0154 0.2645 537409 outcome TRUE reported 3029_52_CD209_DC_SIGN
CD209 chr9 133324174 0.018157 1e-200 35371 TRUE reported textfile
2 1 TRUE
2653 rs12378537 T C T C 0.7227 0.0068 0.07663 NA FALSE FALSE FALSE
GCST006061 0.0125 0.5833 537409 outcome TRUE reported 3029_52_CD209_DC_SIGN
CD209 chr9 133190139 0.0111 1e-200 35368 TRUE reported textfile 2
1 TRUE
2654 rs139562936 T G T G 0.2364 -0.0301 0.0211 NA FALSE FALSE FALSE
GCST006061 0.034 0.3765 537409 outcome TRUE reported 3029_52_CD209_DC_SIGN
CD209 chr9 134215558 0.030317 6.309e-15 35372 TRUE reported
textfile 2 1 TRUE
2655 rs143037947 T C T C -0.2554 -0.0118 0.03125 NA FALSE FALSE FALSE
GCST006061 0.0337 0.7268 537409 outcome TRUE reported 3029_52_CD209_DC_SIGN
CD209 chr9 133356665 0.024933 1.268e-24 35371 TRUE reported
textfile 2 1 TRUE
2656 rs2986616 A G A G 0.0738 0.0017 0.43949 NA FALSE FALSE FALSE
GCST006061 0.0078 0.8247 537409 outcome TRUE reported 3029_52_CD209_DC_SIGN
CD209 chr9 131343511 0.008753 3.405e-17 35372 TRUE reported
textfile 2 1 TRUE
2657 rs3094377 C T C T -0.4841 -0.005 0.02787 NA FALSE FALSE FALSE
GCST006061 0.0202 0.8033 537409 outcome TRUE reported 3029_52_CD209_DC_SIGN
CD209 chr9 133446998 0.026528 2.112e-74 35372 TRUE reported
textfile 2 1 TRUE
2658 rs34840745 T C T C -0.055 -0.0061 0.25189 NA FALSE FALSE FALSE
GCST006061 0.0082 0.4563 537409 outcome TRUE reported 3029_52_CD209_DC_SIGN
CD209 chr19 7183786 0.009194 2.203e-09 35375 TRUE reported textfile
2 1 TRUE
2659 rs36093130 G T G T 0.2441 0.0119 0.01612 NA FALSE FALSE FALSE
GCST006061 0.0352 0.7356 537409 outcome TRUE reported 3029_52_CD209_DC_SIGN
CD209 chr9 134041864 0.034736 2.107e-12 35367 TRUE reported
textfile 2 1 TRUE
2660 rs55806425 G A G A 0.0887 -0.0108 0.1649 NA FALSE FALSE FALSE
GCST006061 0.0092 0.2433 537409 outcome TRUE reported 3029_52_CD209_DC_SIGN
CD209 chr19 7655982 0.010943 5.237e-16 35372 TRUE reported textfile
2 1 TRUE
2661 rs62578600 T C T C 0.1739 -0.014 0.03036 NA FALSE FALSE FALSE
GCST006061 0.0315 0.6573 537409 outcome TRUE reported 3029_52_CD209_DC_SIGN
CD209 chr9 132540263 0.025689 1.292e-11 35372 TRUE reported
textfile 2 1 TRUE
2662 rs629301 T G T G 0.1141 -0.0141 0.20782 NA FALSE FALSE FALSE
GCST006061 0.0087 0.1045 537409 outcome TRUE reported 3029_52_CD209_DC_SIGN
CD209 chr1 109275684 0.009735 9.963e-32 35344 TRUE reported
textfile 2 1 TRUE

2663 rs8193007 T C T C 0.1136 -0.0104 0.10164 NA FALSE FALSE FALSE
GCST006061 0.011 0.3421 537409 outcome TRUE reported 3029_52_CD209_DC_SIGN
CD209 chr9 132996286 0.014354 2.484e-15 35366 TRUE reported
textfile 2 1 TRUE
2664 rs10411119 C T C T 0.3251 -0.0032 0.32437 NA FALSE FALSE FALSE
GCST006061 0.0097 0.7433 537409 outcome TRUE reported
13720_95_PRTN3_Proteinase_3 PRTN3 chr19 834154 0.009259 1e-200 35311 TRUE
reported textfile 2 1 TRUE
2665 rs1060442 G A G A -0.1456 0.0063 0.35341 NA FALSE FALSE FALSE
GCST006061 0.008 0.4301 537409 outcome TRUE reported
13720_95_PRTN3_Proteinase_3 PRTN3 chr19 885818 0.009209 2.632e-56 35319
TRUE reported textfile 2 1 TRUE
2666 rs116184613 C T C T 0.112 0.0188 0.1152 NA FALSE FALSE FALSE
GCST006061 0.0145 0.1955 537409 outcome TRUE reported
13720_95_PRTN3_Proteinase_3 PRTN3 chr19 557979 0.013904 7.93e-16 35305
TRUE reported textfile 2 1 TRUE
2667 rs12453341 T C T C -0.065 0.0272 0.37505 NA FALSE FALSE FALSE
GCST006061 0.0074 0.0002422 537409 outcome TRUE reported
13720_95_PRTN3_Proteinase_3 PRTN3 chr17 40024880 0.00894 3.569e-13 35379
TRUE reported textfile 2 1 TRUE
2668 rs13277237 A G A G -0.0781 0.0084 0.40349 NA FALSE FALSE FALSE
GCST006061 0.0071 0.2393 537409 outcome TRUE reported
13720_95_PRTN3_Proteinase_3 PRTN3 chr8 129592317 0.008791 6.425e-19 35372
TRUE reported textfile 2 1 TRUE
2669 rs186905046 T G T G 0.2351 0.012 0.01934 NA FALSE FALSE FALSE
GCST006061 0.045 0.789 537409 outcome TRUE reported
13720_95_PRTN3_Proteinase_3 PRTN3 chr17 59075506 0.031579 9.703e-14 35370
TRUE reported textfile 2 1 TRUE
2670 rs2071408 A G A G -0.0508 -0.0093 0.38311 NA FALSE FALSE FALSE
GCST006061 0.0074 0.2061 537409 outcome TRUE reported
13720_95_PRTN3_Proteinase_3 PRTN3 chr14 103520741 0.008915 1.211e-08 35276
TRUE reported textfile 2 1 TRUE
2671 rs2236947 A C A C -0.054 -0.0143 0.46371 NA FALSE FALSE FALSE
GCST006061 0.0072 0.04619 537409 outcome TRUE reported
13720_95_PRTN3_Proteinase_3 PRTN3 chr3 50334001 0.008677 4.874e-10 35343
TRUE reported textfile 2 1 TRUE
2672 rs2380606 C T C T 0.0505 -0.007 0.47568 NA FALSE FALSE FALSE
GCST006061 0.0072 0.3283 537409 outcome TRUE reported
13720_95_PRTN3_Proteinase_3 PRTN3 chr8 69828661 0.008638 5.02e-09 35362
TRUE reported textfile 2 1 TRUE
2673 rs28678897 A G A G 0.1376 0.024 0.10162 NA FALSE FALSE FALSE
GCST006061 0.0124 0.05344 537409 outcome TRUE reported
13720_95_PRTN3_Proteinase_3 PRTN3 chr16 477485 0.01427 5.282e-22 35317 TRUE
reported textfile 2 1 TRUE
2674 rs28730837 A G A G 0.2496 -0.0026 0.0242 NA FALSE FALSE FALSE
GCST006061 0.0357 0.9427 537409 outcome TRUE reported
13720_95_PRTN3_Proteinase_3 PRTN3 chr17 58278036 0.028369 1.39e-18 35369
TRUE reported textfile 2 1 TRUE
2675 rs34354538 C T C T 0.0837 -0.0164 0.18827 NA FALSE FALSE FALSE
GCST006061 0.0089 0.06463 537409 outcome TRUE reported
13720_95_PRTN3_Proteinase_3 PRTN3 chr1 76072161 0.010991 2.628e-14 35354
TRUE reported textfile 2 1 TRUE
2676 rs4807455 A G A G 0.0887 0.0018 0.27637 NA FALSE FALSE FALSE
GCST006061 0.0087 0.8364 537409 outcome TRUE reported
13720_95_PRTN3_Proteinase_3 PRTN3 chr19 1033560 0.009878 2.725e-19 35320
TRUE reported textfile 2 1 TRUE
2677 rs55771110 A G A G 0.0759 0.0077 0.14938 NA FALSE FALSE FALSE
GCST006061 0.0102 0.4518 537409 outcome TRUE reported
13720_95_PRTN3_Proteinase_3 PRTN3 chr3 47305227 0.011993 2.472e-10 35343
TRUE reported textfile 2 1 TRUE
2678 rs59653410 G A G A -0.1362 0.0113 0.23818 NA FALSE FALSE FALSE
GCST006061 0.0097 0.2463 537409 outcome TRUE reported
13720_95_PRTN3_Proteinase_3 PRTN3 chr19 787096 0.010389 2.908e-39 35308
TRUE reported textfile 2 1 TRUE
2679 rs62128522 A G A G 0.0541 -0.0041 0.47543 NA FALSE FALSE FALSE
GCST006061 0.0083 0.6231 537409 outcome TRUE reported

13720_95_PRTN3_Proteinase_3 PRTN3 chr19 1542400 0.008817 8.48e-10 35318
TRUE reported textfile 2 1 TRUE
2680 rs6775011 C T C T -0.0651 -9.00E-04 0.29896 NA FALSE FALSE FALSE
GCST006061 0.0083 0.914 537409 outcome TRUE reported
13720_95_PRTN3_Proteinase_3 PRTN3 chr3 128677640 0.009416 4.711e-12 35363
TRUE reported textfile 2 1 TRUE
2681 rs74899816 T C T C 0.2785 0.0219 0.03221 NA FALSE FALSE FALSE
GCST006061 0.0215 0.3091 537409 outcome TRUE reported
13720_95_PRTN3_Proteinase_3 PRTN3 chr6 31358435 0.024549 7.892e-30 35440
TRUE reported textfile 2 1 TRUE
2682 rs76428106 C T C T 0.262 -0.0446 0.01359 NA FALSE FALSE FALSE
GCST006061 0.0438 0.3085 537409 outcome TRUE reported
13720_95_PRTN3_Proteinase_3 PRTN3 chr13 28029870 0.037444 2.614e-12 35363
TRUE reported textfile 2 1 TRUE
2683 rs79408316 T C T C 0.0919 -0.0224 0.07123 NA FALSE FALSE FALSE
GCST006061 0.0145 0.1219 537409 outcome TRUE reported
13720_95_PRTN3_Proteinase_3 PRTN3 chr3 186902765 0.016852 4.943e-08 35367
TRUE reported textfile 2 1 TRUE
2684 rs8178414 T C T C 0.382 0.0359 0.01741 NA FALSE FALSE FALSE
GCST006061 0.0374 0.3376 537409 outcome TRUE reported
13720_95_PRTN3_Proteinase_3 PRTN3 chr17 58268002 0.032566 8.943e-32 35369
TRUE reported textfile 2 1 TRUE
2685 rs915125 T C T C 0.0766 0.0056 0.30374 NA FALSE FALSE FALSE
GCST006061 0.0079 0.4797 537409 outcome TRUE reported
13720_95_PRTN3_Proteinase_3 PRTN3 chr6 81753659 0.009463 5.73e-16 35331
TRUE reported textfile 2 1 TRUE
2686 rs10411127 C T C T 0.1496 -2.00E-04 0.07796 NA FALSE FALSE FALSE
GCST006061 0.0168 0.9913 537409 outcome TRUE reported 11293_14_LRRN1_LRRN1
LRRN1 chr19 43099301 0.017397 8.037e-18 35355 TRUE reported
textfile 2 1 TRUE
2687 rs10922098 T C T C -0.0771 -0.009 0.40011 NA FALSE FALSE FALSE
GCST006061 0.0075 0.2275 537409 outcome TRUE reported 11293_14_LRRN1_LRRN1
LRRN1 chr1 196695521 0.008807 2.056e-18 35329 TRUE reported
textfile 2 1 TRUE
2688 rs147442832 A G A G 0.2651 0.0381 0.02855 NA FALSE FALSE FALSE
GCST006061 0.0373 0.3073 537409 outcome TRUE reported 11293_14_LRRN1_LRRN1
LRRN1 chr19 45465204 0.028037 3.215e-21 35363 TRUE reported
textfile 2 1 TRUE
2689 rs405509 G T G T -0.2356 -0.0104 0.49396 NA FALSE FALSE FALSE
GCST006061 0.0073 0.1508 537409 outcome TRUE reported 11293_14_LRRN1_LRRN1
LRRN1 chr19 44905579 0.009193 7.155e-145 35358 TRUE reported
textfile 2 1 TRUE
2690 rs55923289 C T C T 0.3732 -0.0094 0.17219 NA FALSE FALSE FALSE
GCST006061 0.0115 0.4142 537409 outcome TRUE reported 11293_14_LRRN1_LRRN1
LRRN1 chr19 44737327 0.012107 1e-200 35360 TRUE reported textfile
2 1 TRUE
2691 rs6859 G A G A -0.2815 -9.00E-04 0.42659 NA FALSE FALSE FALSE
GCST006061 0.0073 0.907 537409 outcome TRUE reported 11293_14_LRRN1_LRRN1
LRRN1 chr19 44878777 0.009267 1e-200 35358 TRUE reported textfile
2 1 TRUE
2692 rs10411716 C T C T -0.3777 -0.0434 0.03235 NA FALSE FALSE FALSE
GCST006061 0.0285 0.1274 537409 outcome TRUE reported 6457_50_PCDHGA1_PCDG1
PCDHGA1 chr19 43285762 0.022677 2.737e-62 35365 TRUE reported
textfile 2 1 TRUE
2693 rs111984974 C A C A -0.6117 0.0645 0.02771 NA FALSE FALSE FALSE
GCST006061 0.0576 0.2627 537409 outcome TRUE reported 6457_50_PCDHGA1_PCDG1
PCDHGA1 chr19 43090727 0.023713 9.973e-147 35364 TRUE reported
textfile 2 1 TRUE
2694 rs112052262 C T C T 0.3773 -0.0177 0.19915 NA FALSE FALSE FALSE
GCST006061 0.0382 0.6428 537409 outcome TRUE reported 6457_50_PCDHGA1_PCDG1
PCDHGA1 chr19 42919450 0.010473 1e-200 35363 TRUE reported textfile
2 1 TRUE
2695 rs11668974 A G A G 0.2659 -0.0118 0.04573 NA FALSE FALSE FALSE
GCST006061 0.025 0.6379 537409 outcome TRUE reported 6457_50_PCDHGA1_PCDG1
PCDHGA1 chr19 44156638 0.018807 2.206e-45 35370 TRUE reported
textfile 2 1 TRUE

2696 rs117090502 T C T C 0.2954 -0.01 0.03716 NA FALSE FALSE FALSE
GCST006061 0.0236 0.6729 537409 outcome TRUE reported 6457_50_PCDHGA1_PCDG1
PCDHGA1 chr19 41663006 0.021072 1.201e-44 35381 TRUE reported
textfile 2 1 TRUE
2697 rs117216419 G A G A -0.1659 0.0103 0.02024 NA FALSE FALSE FALSE
GCST006061 0.0357 0.7739 537409 outcome TRUE reported 6457_50_PCDHGA1_PCDG1
PCDHGA1 chr19 41194441 0.027047 8.578e-10 35369 TRUE reported
textfile 2 1 TRUE
2698 rs117453984 G A G A -0.6807 -0.019 0.04585 NA FALSE FALSE FALSE
GCST006061 0.04 0.6344 537409 outcome TRUE reported 6457_50_PCDHGA1_PCDG1
PCDHGA1 chr19 42706995 0.019061 1e-200 35367 TRUE reported textfile
2 1 TRUE
2699 rs140064353 G T G T 0.4492 0.0142 0.03004 NA FALSE FALSE FALSE
GCST006061 0.0271 0.6003 537409 outcome TRUE reported 6457_50_PCDHGA1_PCDG1
PCDHGA1 chr19 43167528 0.022921 1.623e-85 35365 TRUE reported
textfile 2 1 TRUE
2700 rs144494943 C T C T 0.1512 -9.00E-04 0.02232 NA FALSE FALSE FALSE
GCST006061 0.0405 0.9826 537409 outcome TRUE reported 6457_50_PCDHGA1_PCDG1
PCDHGA1 chr19 43074297 0.026948 2.014e-08 35365 TRUE reported
textfile 2 1 TRUE
2701 rs17656836 A G A G -0.3165 -0.0415 0.03418 NA FALSE FALSE FALSE
GCST006061 0.0259 0.1088 537409 outcome TRUE reported 6457_50_PCDHGA1_PCDG1
PCDHGA1 chr19 44052138 0.021737 5.035e-48 35371 TRUE reported
textfile 2 1 TRUE
2702 rs2354283 A C A C 0.5845 0.0106 0.07299 NA FALSE FALSE FALSE
GCST006061 0.017 0.5332 537409 outcome TRUE reported 6457_50_PCDHGA1_PCDG1
PCDHGA1 chr19 43129377 0.008974 1e-200 35365 TRUE reported textfile
2 1 TRUE
2703 rs34408465 T C T C 0.698 0.0116 0.02095 NA FALSE FALSE FALSE
GCST006061 0.0376 0.7585 537409 outcome TRUE reported 6457_50_PCDHGA1_PCDG1
PCDHGA1 chr19 43063301 0.026865 8.019e-149 35365 TRUE reported
textfile 2 1 TRUE
2704 rs76081751 G A G A 0.1412 0.0345 0.03348 NA FALSE FALSE FALSE
GCST006061 0.0357 0.334 537409 outcome TRUE reported 6457_50_PCDHGA1_PCDG1
PCDHGA1 chr19 36938124 0.022008 1.4e-10 35377 TRUE reported textfile
2 1 TRUE
2705 rs10411862 T C T C 0.113 0.0108 0.26222 NA FALSE FALSE FALSE
GCST006061 0.0085 0.2044 537409 outcome TRUE reported 9118_7_PSD2_PSD2
PSD2 chr19 45316838 0.00969 2.003e-31 35372 TRUE reported textfile
2 1 TRUE
2706 rs111696008 A G A G -0.2714 -0.0259 0.02443 NA FALSE FALSE FALSE
GCST006061 0.0401 0.519 537409 outcome TRUE reported 9118_7_PSD2_PSD2
PSD2 chr2 216622986 0.027609 8.358e-23 35359 TRUE reported
textfile 2 1 TRUE
2707 rs112450640 A G A G 0.302 0.031 0.0157 NA FALSE FALSE FALSE
GCST006061 0.0317 0.3289 537409 outcome TRUE reported 9118_7_PSD2_PSD2
PSD2 chr19 44793107 0.034356 1.492e-18 35367 TRUE reported
textfile 2 1 TRUE
2708 rs113708952 C A C A 0.0876 -0.0045 0.18053 NA FALSE FALSE FALSE
GCST006061 0.0102 0.6587 537409 outcome TRUE reported 9118_7_PSD2_PSD2
PSD2 chr19 45091745 0.011196 5.097e-15 35371 TRUE reported
textfile 2 1 TRUE
2709 rs12026141 T C T C -0.0665 0.0094 0.31246 NA FALSE FALSE FALSE
GCST006061 0.0076 0.2137 537409 outcome TRUE reported 9118_7_PSD2_PSD2
PSD2 chr1 201980465 0.009025 1.724e-13 35357 TRUE reported
textfile 2 1 TRUE
2710 rs144202860 A G A G -0.2509 -0.0629 0.01274 NA FALSE FALSE FALSE
GCST006061 0.0538 0.2423 537409 outcome TRUE reported 9118_7_PSD2_PSD2
PSD2 chr2 216625740 0.036997 1.189e-11 35359 TRUE reported
textfile 2 1 TRUE
2711 rs2068888 A G A G -0.0469 0.0184 0.42949 NA FALSE FALSE FALSE
GCST006061 0.0071 0.009729 537409 outcome TRUE reported 9118_7_PSD2_PSD2
PSD2 chr10 93079885 0.008335 1.837e-08 35374 TRUE reported
textfile 2 1 TRUE
2712 rs2871768 T C T C -0.0662 -0.0233 0.19572 NA FALSE FALSE FALSE
GCST006061 0.0102 0.02306 537409 outcome TRUE reported 9118_7_PSD2_PSD2

PSD2 chr19 46484601 0.010757 7.554e-10 35369 TRUE reported
textfile 2 1 TRUE
2713 rs62182810 A G A G -0.0566 -0.006 0.4375 NA FALSE FALSE FALSE
GCST006061 0.0073 0.4103 537409 outcome TRUE reported 9118_7_PSD2_PSD2
PSD2 chr2 203522759 0.008413 1.724e-11 35365 TRUE reported
textfile 2 1 TRUE
2714 rs62290472 A G A G 0.0893 0.011 0.08266 NA FALSE FALSE FALSE
GCST006061 0.0106 0.3013 537409 outcome TRUE reported 9118_7_PSD2_PSD2
PSD2 chr3 162193597 0.015281 5.1e-09 35340 TRUE reported textfile
2 1 TRUE
2715 rs673548 A G A G -0.0691 -0.009 0.23304 NA FALSE FALSE FALSE
GCST006061 0.0085 0.2884 537409 outcome TRUE reported 9118_7_PSD2_PSD2
PSD2 chr2 21014672 0.009879 2.655e-12 35373 TRUE reported
textfile 2 1 TRUE
2716 rs687339 T C T C 0.0903 -0.0273 0.22733 NA FALSE FALSE FALSE
GCST006061 0.0085 0.001387 537409 outcome TRUE reported 9118_7_PSD2_PSD2
PSD2 chr3 136213517 0.010024 2.092e-19 35361 TRUE reported
textfile 2 1 TRUE
2717 rs6874202 C T C T 0.0899 0.0107 0.35789 NA FALSE FALSE FALSE
GCST006061 0.0075 0.1501 537409 outcome TRUE reported 9118_7_PSD2_PSD2
PSD2 chr5 156964617 0.008626 1.967e-25 35351 TRUE reported
textfile 2 1 TRUE
2718 rs73045691 A G A G -0.4245 0.0102 0.26022 NA FALSE FALSE FALSE
GCST006061 0.0083 0.2192 537409 outcome TRUE reported 9118_7_PSD2_PSD2
PSD2 chr19 44937272 0.007015 1e-200 35368 TRUE reported textfile
2 1 TRUE
2719 rs73169103 T C T C 0.1951 0.0178 0.03563 NA FALSE FALSE FALSE
GCST006061 0.0249 0.4756 537409 outcome TRUE reported 9118_7_PSD2_PSD2
PSD2 chr3 167190675 0.022526 4.669e-18 35354 TRUE reported
textfile 2 1 TRUE
2720 rs76315187 A C A C 0.2501 0.0164 0.01665 NA FALSE FALSE FALSE
GCST006061 0.0459 0.7211 537409 outcome TRUE reported 9118_7_PSD2_PSD2
PSD2 chr3 165254582 0.03264 1.825e-14 35340 TRUE reported textfile
2 1 TRUE
2721 rs780093 C T C T -0.0506 -0.0074 0.34157 NA FALSE FALSE FALSE
GCST006061 0.0073 0.3116 537409 outcome TRUE reported 9118_7_PSD2_PSD2
PSD2 chr2 27519736 0.008777 8.149e-09 35373 TRUE reported
textfile 2 1 TRUE
2722 rs78986976 A G A G 0.2136 -0.0233 0.06075 NA FALSE FALSE FALSE
GCST006061 0.0178 0.1899 537409 outcome TRUE reported 9118_7_PSD2_PSD2
PSD2 chr19 44826181 0.017959 1.272e-32 35367 TRUE reported
textfile 2 1 TRUE
2723 rs1041316 A G A G 0.0729 -0.0125 0.24956 NA FALSE FALSE FALSE
GCST006061 0.008 0.1207 537409 outcome TRUE reported 2837_3_MET_Met MET
chr14 56633141 0.009626 3.638e-14 35358 TRUE reported textfile 2
1 TRUE
2724 rs10435378 A G A G 0.1309 0.0168 0.44018 NA FALSE FALSE FALSE
GCST006061 0.0071 0.01844 537409 outcome TRUE reported 2837_3_MET_Met MET
chr7 116785439 0.008365 3.41e-55 35343 TRUE reported textfile 2
1 TRUE
2725 rs11045856 G T G T 0.0796 -0.017 0.22646 NA FALSE FALSE FALSE
GCST006061 0.0088 0.05224 537409 outcome TRUE reported 2837_3_MET_Met MET
chr12 21197755 0.009942 1.18e-15 35423 TRUE reported textfile 2
1 TRUE
2726 rs11603123 A G A G 0.1839 0.0206 0.03671 NA FALSE FALSE FALSE
GCST006061 0.023 0.3702 537409 outcome TRUE reported 2837_3_MET_Met MET
chr11 126435600 0.021361 7.353e-18 35371 TRUE reported textfile 2
1 TRUE
2727 rs117210485 A G A G 0.1952 0.033 0.0198 NA FALSE FALSE FALSE
GCST006061 0.0265 0.2129 537409 outcome TRUE reported 2837_3_MET_Met MET
chr9 133523026 0.029983 7.492e-11 35360 TRUE reported textfile 2
1 TRUE
2728 rs13226546 C A C A 0.0715 0.0045 0.47752 NA FALSE FALSE FALSE
GCST006061 0.0073 0.5367 537409 outcome TRUE reported 2837_3_MET_Met MET
chr7 129099895 0.008308 7.531e-18 35351 TRUE reported textfile 2
1 TRUE

2729 rs138121767 T C T C 0.1538 0.0112 0.03875 NA FALSE FALSE FALSE
GCST006061 0.0263 0.6694 537409 outcome TRUE reported 2837_3_MET_Met MET
chr7 117510258 0.02152 8.874e-13 35343 TRUE reported textfile 2 1
TRUE
2730 rs139974673 C T C T -0.1547 0.0351 0.02734 NA FALSE FALSE FALSE
GCST006061 0.0241 0.1443 537409 outcome TRUE reported 2837_3_MET_Met MET
chr15 43735687 0.02534 1.028e-09 35362 TRUE reported textfile 2 1
TRUE
2731 rs143779007 C T C T -0.153 -0.009 0.02951 NA FALSE FALSE FALSE
GCST006061 0.0606 0.8821 537409 outcome TRUE reported 2837_3_MET_Met MET
chr7 117230881 0.024898 7.991e-10 35339 TRUE reported textfile 2
1 TRUE
2732 rs145179853 T C T C -0.2067 -0.0399 0.01376 NA FALSE FALSE FALSE
GCST006061 0.036 0.2678 537409 outcome TRUE reported 2837_3_MET_Met MET
chr7 117166049 0.035196 4.283e-09 35339 TRUE reported textfile 2
1 TRUE
2733 rs1604038 T C T C -0.0685 -0.0183 0.25937 NA FALSE FALSE FALSE
GCST006061 0.0078 0.01886 537409 outcome TRUE reported 2837_3_MET_Met MET
chr3 170991404 0.009461 4.49e-13 35366 TRUE reported textfile 2
1 TRUE
2734 rs174562 G A G A 0.0588 0.0106 0.38814 NA FALSE FALSE FALSE
GCST006061 0.0075 0.1572 537409 outcome TRUE reported 2837_3_MET_Met MET
chr11 61817672 0.008518 5.1e-12 35367 TRUE reported textfile 2 1
TRUE
2735 rs3827211 G A G A -0.0664 -0.009 0.42239 NA FALSE FALSE FALSE
GCST006061 0.0073 0.2203 537409 outcome TRUE reported 2837_3_MET_Met MET
chr21 41247327 0.008414 2.981e-15 35367 TRUE reported textfile 2
1 TRUE
2736 rs55714927 T C T C 0.0573 1e-04 0.21166 NA FALSE FALSE FALSE
GCST006061 0.0098 0.9926 537409 outcome TRUE reported 2837_3_MET_Met MET
chr17 7176997 0.010236 2.171e-08 35370 TRUE reported textfile 2 1
TRUE
2737 rs635634 C T C T 0.395 0.0285 0.12995 NA FALSE FALSE FALSE
GCST006061 0.0089 0.001351 537409 outcome TRUE reported 2837_3_MET_Met MET
chr9 133279427 0.012281 1e-200 35371 TRUE reported textfile 2 1
TRUE
2738 rs72784938 C A C A 0.1215 -0.0374 0.09601 NA FALSE FALSE FALSE
GCST006061 0.0186 0.04381 537409 outcome TRUE reported 2837_3_MET_Met MET
chr16 22080746 0.014032 4.777e-18 35367 TRUE reported textfile 2
1 TRUE
2739 rs77542162 G A G A 0.3272 0.0177 0.025 NA FALSE FALSE FALSE
GCST006061 0.0302 0.557 537409 outcome TRUE reported 2837_3_MET_Met MET
chr17 69085137 0.026162 6.848e-36 35371 TRUE reported textfile 2
1 TRUE
2740 rs10413815 T C T C -0.1507 -0.0024 0.22131 NA FALSE FALSE FALSE
GCST006061 0.0092 0.7976 537409 outcome TRUE reported 6458_6_RPN1_RPN1
RPN1 chr19 51727779 0.010764 1.548e-44 35381 TRUE reported
textfile 2 1 TRUE
2741 rs12978451 A C A C 0.0641 -0.0076 0.2962 NA FALSE FALSE FALSE
GCST006061 0.0077 0.3259 537409 outcome TRUE reported 6458_6_RPN1_RPN1
RPN1 chr19 53519489 0.009817 6.599e-11 35379 TRUE reported
textfile 2 1 TRUE
2742 rs1551556 A G A G 0.2878 -0.0117 0.32549 NA FALSE FALSE FALSE
GCST006061 0.0074 0.1126 537409 outcome TRUE reported 6458_6_RPN1_RPN1
RPN1 chr19 51502415 0.009424 1e-200 35383 TRUE reported textfile
2 1 TRUE
2743 rs2864138 A G A G -0.1537 0.0095 0.40978 NA FALSE FALSE FALSE
GCST006061 0.0074 0.1981 537409 outcome TRUE reported 6458_6_RPN1_RPN1
RPN1 chr19 51597882 0.009095 4.546e-64 35382 TRUE reported
textfile 2 1 TRUE
2744 rs10413980 T C T C -0.089 0.0095 0.36719 NA FALSE FALSE FALSE
GCST006061 0.0077 0.2202 537409 outcome TRUE reported 18882_7_CLSTN2_CSTN2
CLSTN2 chr19 53092077 0.008409 3.558e-26 35380 TRUE reported
textfile 2 1 TRUE
2745 rs113351462 G A G A 0.1816 -0.0448 0.01911 NA FALSE FALSE FALSE
GCST006061 0.0315 0.1548 537409 outcome TRUE reported 18882_7_CLSTN2_CSTN2

CLSTN2 chr3 140598445 0.030956 4.456e-09 35367 TRUE reported
textfile 2 1 TRUE
2746 rs12484809 T C T C 0.0611 0.0206 0.1694 NA FALSE FALSE FALSE
GCST006061 0.0094 0.0278 537409 outcome TRUE reported 18882_7_CLSTN2_CSTN2
CLSTN2 chr22 43929751 0.01081 1.585e-08 35372 TRUE reported textfile
2 1 TRUE
2747 rs148302872 T G T G 0.3175 0.0038 0.01161 NA FALSE FALSE FALSE
GCST006061 0.055 0.9442 537409 outcome TRUE reported 18882_7_CLSTN2_CSTN2
CLSTN2 chr3 141133729 0.037956 6.011e-17 35363 TRUE reported
textfile 2 1 TRUE
2748 rs2071386 A G A G -0.1292 -0.0094 0.05458 NA FALSE FALSE FALSE
GCST006061 0.0134 0.4827 537409 outcome TRUE reported 18882_7_CLSTN2_CSTN2
CLSTN2 chr3 139539666 0.017907 5.395e-13 35364 TRUE reported
textfile 2 1 TRUE
2749 rs2220837 C A C A 0.1068 -0.0019 0.28849 NA FALSE FALSE FALSE
GCST006061 0.0078 0.8088 537409 outcome TRUE reported 18882_7_CLSTN2_CSTN2
CLSTN2 chr3 140644507 0.009073 5.512e-32 35367 TRUE reported
textfile 2 1 TRUE
2750 rs35220837 A G A G 0.4115 -0.0019 0.07582 NA FALSE FALSE FALSE
GCST006061 0.0176 0.9142 537409 outcome TRUE reported 18882_7_CLSTN2_CSTN2
CLSTN2 chr3 139904479 0.015247 1.955e-160 35363 TRUE reported
textfile 2 1 TRUE
2751 rs4073898 G T G T 0.1276 -0.0047 0.28449 NA FALSE FALSE FALSE
GCST006061 0.0081 0.5626 537409 outcome TRUE reported 18882_7_CLSTN2_CSTN2
CLSTN2 chr3 139740454 0.00914 2.711e-44 35364 TRUE reported textfile
2 1 TRUE
2752 rs693293 T C T C -0.1154 0.0126 0.0973 NA FALSE FALSE FALSE
GCST006061 0.0129 0.3273 537409 outcome TRUE reported 18882_7_CLSTN2_CSTN2
CLSTN2 chr3 138690334 0.013933 1.206e-16 35362 TRUE reported
textfile 2 1 TRUE
2753 rs7569795 G A G A 0.0495 0.0056 0.28556 NA FALSE FALSE FALSE
GCST006061 0.008 0.4887 537409 outcome TRUE reported 18882_7_CLSTN2_CSTN2
CLSTN2 chr2 229263222 0.008974 3.475e-08 35359 TRUE reported
textfile 2 1 TRUE
2754 rs76824284 G A G A -0.1466 0.0149 0.0288 NA FALSE FALSE FALSE
GCST006061 0.0285 0.5998 537409 outcome TRUE reported 18882_7_CLSTN2_CSTN2
CLSTN2 chr3 140464444 0.024388 1.843e-09 35370 TRUE reported
textfile 2 1 TRUE
2755 rs77542162 G A G A 0.2227 0.0177 0.025 NA FALSE FALSE FALSE
GCST006061 0.0302 0.557 537409 outcome TRUE reported 18882_7_CLSTN2_CSTN2
CLSTN2 chr17 69085137 0.025488 2.388e-18 35371 TRUE reported
textfile 2 1 TRUE
2756 rs10417163 G T G T 0.0482 -0.005 0.43536 NA FALSE FALSE FALSE
GCST006061 0.0079 0.5286 537409 outcome TRUE reported 3339_33_THBS2_TSP2
THBS2 chr19 52800452 0.008198 4.113e-09 35376 TRUE reported
textfile 2 1 TRUE
2757 rs12664015 A G A G -0.3363 -0.0046 0.14517 NA FALSE FALSE FALSE
GCST006061 0.0111 0.6781 537409 outcome TRUE reported 3339_33_THBS2_TSP2
THBS2 chr6 169222563 0.011837 1.523e-177 35019 TRUE reported
textfile 2 1 TRUE
2758 rs139841116 T C T C 0.3313 -0.0679 0.02658 NA FALSE FALSE FALSE
GCST006061 0.0391 0.08225 537409 outcome TRUE reported 3339_33_THBS2_TSP2
THBS2 chr6 169822789 0.025907 1.913e-37 34955 TRUE reported
textfile 2 1 TRUE
2759 rs148506905 C T C T 0.1964 -0.0506 0.04498 NA FALSE FALSE FALSE
GCST006061 0.0276 0.0664 537409 outcome TRUE reported 3339_33_THBS2_TSP2
THBS2 chr6 169312002 0.020245 2.978e-22 34981 TRUE reported
textfile 2 1 TRUE
2760 rs148674511 G A G A 0.2433 0.0222 0.0206 NA FALSE FALSE FALSE
GCST006061 0.0389 0.568 537409 outcome TRUE reported 3339_33_THBS2_TSP2
THBS2 chr6 168578075 0.029277 9.559e-17 35327 TRUE reported
textfile 2 1 TRUE
2761 rs186322434 T C T C 0.3577 0.0148 0.01606 NA FALSE FALSE FALSE
GCST006061 0.0556 0.7896 537409 outcome TRUE reported 3339_33_THBS2_TSP2
THBS2 chr6 168868392 0.033558 1.582e-26 35285 TRUE reported
textfile 2 1 TRUE

2762 rs6456172 C T C T 0.0838 0.0041 0.17515 NA FALSE FALSE FALSE
GCST006061 0.0107 0.6998 537409 outcome TRUE reported 3339_33_THBS2_TSP2
THBS2 chr6 170157248 0.011038 3.152e-14 34944 TRUE reported
textfile 2 1 TRUE
2763 rs71574910 C T C T 0.1743 -0.0209 0.03538 NA FALSE FALSE FALSE
GCST006061 0.0174 0.2293 537409 outcome TRUE reported 3339_33_THBS2_TSP2
THBS2 chr6 168839203 0.022298 5.415e-15 35285 TRUE reported
textfile 2 1 TRUE
2764 rs73034020 T C T C 0.4493 1e-04 0.11214 NA FALSE FALSE FALSE
GCST006061 0.0138 0.9936 537409 outcome TRUE reported 3339_33_THBS2_TSP2
THBS2 chr6 169075585 0.013039 1e-200 35039 TRUE reported textfile
2 1 TRUE
2765 rs10417239 A G A G 0.113 0.0045 0.12757 NA FALSE FALSE FALSE
GCST006061 0.0101 0.6577 537409 outcome TRUE reported 5108_72_NOTCH3_Notch_3
NOTCH3 chr19 15183913 0.012723 6.585e-19 35375 TRUE reported
textfile 2 1 TRUE
2766 rs1260326 C T C T 0.0541 -0.0069 0.34132 NA FALSE FALSE FALSE
GCST006061 0.0073 0.3401 537409 outcome TRUE reported 5108_72_NOTCH3_Notch_3
NOTCH3 chr2 27508073 0.008927 1.358e-09 35373 TRUE reported
textfile 2 1 TRUE
2767 rs150844304 C A C A -0.1465 0.0439 0.0276 NA FALSE FALSE FALSE
GCST006061 0.0244 0.07203 537409 outcome TRUE reported 5108_72_NOTCH3_Notch_3
NOTCH3 chr15 43434427 0.025664 1.14e-08 35363 TRUE reported
textfile 2 1 TRUE
2768 rs186511240 T C T C 0.1382 -0.0051 0.0437 NA FALSE FALSE FALSE
GCST006061 0.0218 0.8147 537409 outcome TRUE reported 5108_72_NOTCH3_Notch_3
NOTCH3 chr12 56703338 0.020433 1.345e-11 35369 TRUE reported
textfile 2 1 TRUE
2769 rs2237582 G A G A 0.0981 -0.0128 0.28548 NA FALSE FALSE FALSE
GCST006061 0.0078 0.0999 537409 outcome TRUE reported 5108_72_NOTCH3_Notch_3
NOTCH3 chr7 95304888 0.009374 1.25e-25 35349 TRUE reported
textfile 2 1 TRUE
2770 rs2279373 T C T C 0.1187 0.0065 0.09103 NA FALSE FALSE FALSE
GCST006061 0.0124 0.6001 537409 outcome TRUE reported 5108_72_NOTCH3_Notch_3
NOTCH3 chr12 56994414 0.014616 4.612e-16 35369 TRUE reported
textfile 2 1 TRUE
2771 rs55714927 T C T C 0.059 1e-04 0.21166 NA FALSE FALSE FALSE
GCST006061 0.0098 0.9926 537409 outcome TRUE reported 5108_72_NOTCH3_Notch_3
NOTCH3 chr17 7176997 0.010386 1.343e-08 35370 TRUE reported textfile
2 1 TRUE
2772 rs635634 C T C T 0.0876 0.0285 0.12995 NA FALSE FALSE FALSE
GCST006061 0.0089 0.001351 537409 outcome TRUE reported
5108_72_NOTCH3_Notch_3 NOTCH3 chr9 133279427 0.012542 2.856e-12 35371
TRUE reported textfile 2 1 TRUE
2773 rs1275922 G A G A 0.0517 -0.0045 0.25722 NA FALSE FALSE FALSE
GCST006061 0.0081 0.5763 537409 outcome TRUE reported 5124_69_ICAM5_sICAM_5
ICAM5 chr2 26710019 0.00927 2.442e-08 35369 TRUE reported textfile
2 1 TRUE
2774 rs140787039 A G A G 0.2733 0.021 0.01789 NA FALSE FALSE FALSE
GCST006061 0.0362 0.5619 537409 outcome TRUE reported 5124_69_ICAM5_sICAM_5
ICAM5 chr19 10136342 0.032749 7.102e-17 35375 TRUE reported
textfile 2 1 TRUE
2775 rs2116877 T C T C -0.0733 -0.0046 0.12186 NA FALSE FALSE FALSE
GCST006061 0.0118 0.6966 537409 outcome TRUE reported 5124_69_ICAM5_sICAM_5
ICAM5 chr19 11247764 0.013339 3.907e-08 35381 TRUE reported
textfile 2 1 TRUE
2776 rs3093035 A G A G -0.6241 -0.0418 0.05887 NA FALSE FALSE FALSE
GCST006061 0.0175 0.01673 537409 outcome TRUE reported 5124_69_ICAM5_sICAM_5
ICAM5 chr19 10272297 0.018321 1e-200 35376 TRUE reported textfile
2 1 TRUE
2777 rs635634 C T C T 0.3992 0.0285 0.12995 NA FALSE FALSE FALSE
GCST006061 0.0089 0.001351 537409 outcome TRUE reported
5124_69_ICAM5_sICAM_5 ICAM5 chr9 133279427 0.011946 1e-200 35371 TRUE
reported textfile 2 1 TRUE
2778 rs77542162 G A G A 0.5088 0.0177 0.025 NA FALSE FALSE FALSE
GCST006061 0.0302 0.557 537409 outcome TRUE reported 5124_69_ICAM5_sICAM_5

ICAM5 chr17 69085137 0.02538 2.128e-89 35371 TRUE reported textfile
2 1 TRUE
2779 rs8101473 T C T C 0.5588 0.0039 0.15399 NA FALSE FALSE FALSE
GCST006061 0.0093 0.6749 537409 outcome TRUE reported 5124_69_ICAM5_sICAM_5
ICAM5 chr19 10312662 0.008258 1e-200 35377 TRUE reported textfile
2 1 TRUE
2780 rs9916644 T G T G 0.2195 -0.0029 0.05234 NA FALSE FALSE FALSE
GCST006061 0.0168 0.8617 537409 outcome TRUE reported 5124_69_ICAM5_sICAM_5
ICAM5 chr17 69106726 0.018135 1.009e-33 35371 TRUE reported
textfile 2 1 TRUE
2781 rs10418846 C T C T 0.4903 -0.0162 0.09522 NA FALSE FALSE FALSE
GCST006061 0.0142 0.2546 537409 outcome TRUE reported 9314_9_PSG5_PSG5
PSG5 chr19 42815321 0.013951 1e-200 35366 TRUE reported textfile
2 1 TRUE
2782 rs1041968 A G A G 0.0448 0.001 0.47345 NA FALSE FALSE FALSE
GCST006061 0.0075 0.8901 537409 outcome TRUE reported 9314_9_PSG5_PSG5
PSG5 chr2 21009932 0.008029 2.409e-08 35373 TRUE reported
textfile 2 1 TRUE
2783 rs10922098 T C T C -0.0747 -0.009 0.40011 NA FALSE FALSE FALSE
GCST006061 0.0075 0.2275 537409 outcome TRUE reported 9314_9_PSG5_PSG5
PSG5 chr1 196695521 0.008174 6.333e-20 35338 TRUE reported
textfile 2 1 TRUE
2784 rs111696008 A G A G -0.2059 -0.0259 0.02443 NA FALSE FALSE FALSE
GCST006061 0.0401 0.519 537409 outcome TRUE reported 9314_9_PSG5_PSG5
PSG5 chr2 216622986 0.026593 9.735e-15 35359 TRUE reported
textfile 2 1 TRUE
2785 rs113631324 G A G A 0.1557 -0.0459 0.13996 NA FALSE FALSE FALSE
GCST006061 0.0118 0.000106 537409 outcome TRUE reported 9314_9_PSG5_PSG5
PSG5 chr19 42193005 0.011867 2.53e-39 35370 TRUE reported
textfile 2 1 TRUE
2786 rs12026141 T C T C -0.0505 0.0094 0.31246 NA FALSE FALSE FALSE
GCST006061 0.0076 0.2137 537409 outcome TRUE reported 9314_9_PSG5_PSG5
PSG5 chr1 201980465 0.008687 6.13e-09 35357 TRUE reported
textfile 2 1 TRUE
2787 rs143650967 A G A G 0.1867 -0.0505 0.01506 NA FALSE FALSE FALSE
GCST006061 0.0359 0.1596 537409 outcome TRUE reported 9314_9_PSG5_PSG5
PSG5 chr19 40892186 0.034101 4.376e-08 35375 TRUE reported
textfile 2 1 TRUE
2788 rs144202860 A G A G -0.2896 -0.0629 0.01274 NA FALSE FALSE FALSE
GCST006061 0.0538 0.2423 537409 outcome TRUE reported 9314_9_PSG5_PSG5
PSG5 chr2 216625740 0.035617 4.263e-16 35359 TRUE reported
textfile 2 1 TRUE
2789 rs150252870 T C T C 0.281 0.078 0.02409 NA FALSE FALSE FALSE
GCST006061 0.039 0.04576 537409 outcome TRUE reported 9314_9_PSG5_PSG5
PSG5 chr19 42543577 0.027179 4.703e-25 35369 TRUE reported
textfile 2 1 TRUE
2790 rs157582 T C T C -0.2859 7e-04 0.21951 NA FALSE FALSE FALSE
GCST006061 0.0087 0.9326 537409 outcome TRUE reported 9314_9_PSG5_PSG5
PSG5 chr19 44892962 0.009977 1.311e-180 35368 TRUE reported
textfile 2 1 TRUE
2791 rs2965160 G A G A 0.0579 0.0054 0.38049 NA FALSE FALSE FALSE
GCST006061 0.0075 0.4741 537409 outcome TRUE reported 9314_9_PSG5_PSG5
PSG5 chr19 44692865 0.008533 1.158e-11 35370 TRUE reported
textfile 2 1 TRUE
2792 rs3810408 T G T G 0.2107 0.0116 0.41004 NA FALSE FALSE FALSE
GCST006061 0.0082 0.1563 537409 outcome TRUE reported 9314_9_PSG5_PSG5
PSG5 chr19 43083965 0.008342 9.644e-141 35363 TRUE reported
textfile 2 1 TRUE
2793 rs62183912 A G A G -0.0553 -0.0055 0.43705 NA FALSE FALSE FALSE
GCST006061 0.0072 0.4469 537409 outcome TRUE reported 9314_9_PSG5_PSG5
PSG5 chr2 203302651 0.008105 8.944e-12 35364 TRUE reported
textfile 2 1 TRUE
2794 rs62290472 A G A G 0.1 0.011 0.08266 NA FALSE FALSE FALSE GCST006061
0.0106 0.3013 537409 outcome TRUE reported 9314_9_PSG5_PSG5 PSG5 chr3
162193597 0.014681 9.658e-12 35340 TRUE reported textfile 2 1 TRUE
2795 rs73169103 T C T C 0.1673 0.0178 0.03563 NA FALSE FALSE FALSE

GCST006061 0.0249 0.4756 537409 outcome TRUE reported 9314_9_PSG5_PSG5
PSG5 chr3 167190675 0.021642 1.072e-14 35354 TRUE reported
textfile 2 1 TRUE
2796 rs10419330 A G A G 0.0761 0.0081 0.13679 NA FALSE FALSE FALSE
GCST006061 0.0106 0.4441 537409 outcome TRUE reported 5601_2_PGLYRP2_PGRP_L
PGLYRP2 chr19 15083845 0.011981 2.128e-10 35374 TRUE reported
textfile 2 1 TRUE
2797 rs11078597 C T C T 0.0601 0.0269 0.21836 NA FALSE FALSE FALSE
GCST006061 0.0093 0.003692 537409 outcome TRUE reported
5601_2_PGLYRP2_PGRP_L PGLYRP2 chr17 1715069 0.009886 1.209e-09 35352 TRUE
reported textfile 2 1 TRUE
2798 rs1122713 G A G A 0.0498 -0.0161 0.42186 NA FALSE FALSE FALSE
GCST006061 0.0072 0.0252 537409 outcome TRUE reported 5601_2_PGLYRP2_PGRP_L
PGLYRP2 chr19 14917370 0.008296 1.939e-09 35378 TRUE reported
textfile 2 1 TRUE
2799 rs12609794 A G A G 0.0558 0.0153 0.28119 NA FALSE FALSE FALSE
GCST006061 0.0081 0.0592 537409 outcome TRUE reported 5601_2_PGLYRP2_PGRP_L
PGLYRP2 chr19 35064681 0.009132 9.958e-10 35369 TRUE reported
textfile 2 1 TRUE
2800 rs1273532 T C T C -0.0994 0.009 0.22534 NA FALSE FALSE FALSE
GCST006061 0.0095 0.3466 537409 outcome TRUE reported 5601_2_PGLYRP2_PGRP_L
PGLYRP2 chr19 15539451 0.009705 1.277e-24 35378 TRUE reported
textfile 2 1 TRUE
2801 rs59950280 A G A G 0.065 0.0034 0.31944 NA FALSE FALSE FALSE
GCST006061 0.0077 0.6578 537409 outcome TRUE reported 5601_2_PGLYRP2_PGRP_L
PGLYRP2 chr4 3450618 0.008772 1.263e-13 35280 TRUE reported textfile
2 1 TRUE
2802 rs61747527 C T C T 0.1921 0.031 0.0206 NA FALSE FALSE FALSE
GCST006061 0.0255 0.2238 537409 outcome TRUE reported 5601_2_PGLYRP2_PGRP_L
PGLYRP2 chr19 14891963 0.028638 1.975e-11 35379 TRUE reported
textfile 2 1 TRUE
2803 rs1042034 T C T C 0.0683 0.0089 0.23471 NA FALSE FALSE FALSE
GCST006061 0.0085 0.2969 537409 outcome TRUE reported 6461_54_APOC3_Apo_C_III
APOC3 chr2 21002409 0.009683 1.746e-12 35373 TRUE reported
textfile 2 1 TRUE
2804 rs10889353 C A C A -0.0806 -0.0244 0.34024 NA FALSE FALSE FALSE
GCST006061 0.0076 0.001249 537409 outcome TRUE reported
6461_54_APOC3_Apo_C_III APOC3 chr1 62652525 0.008606 7.542e-21 35358
TRUE reported textfile 2 1 TRUE
2805 rs111696008 A G A G -0.1639 -0.0259 0.02443 NA FALSE FALSE FALSE
GCST006061 0.0401 0.519 537409 outcome TRUE reported 6461_54_APOC3_Apo_C_III
APOC3 chr2 216622986 0.027154 1.581e-09 35359 TRUE reported
textfile 2 1 TRUE
2806 rs1260326 C T C T -0.1208 -0.0069 0.34132 NA FALSE FALSE FALSE
GCST006061 0.0073 0.3401 537409 outcome TRUE reported 6461_54_APOC3_Apo_C_III
APOC3 chr2 27508073 0.008641 2.063e-44 35373 TRUE reported
textfile 2 1 TRUE
2807 rs139076649 C T C T -0.1481 0.0518 0.02935 NA FALSE FALSE FALSE
GCST006061 0.0306 0.09061 537409 outcome TRUE reported 6461_54_APOC3_Apo_C_III
APOC3 chr11 118950068 0.024567 1.657e-09 35378 TRUE reported
textfile 2 1 TRUE
2808 rs141469619 G A G A 0.2635 -0.0376 0.01201 NA FALSE FALSE FALSE
GCST006061 0.0531 0.4786 537409 outcome TRUE reported 6461_54_APOC3_Apo_C_III
APOC3 chr11 116843577 0.036467 4.985e-13 35377 TRUE reported
textfile 2 1 TRUE
2809 rs144009388 C T C T -0.1794 -0.0558 0.01813 NA FALSE FALSE FALSE
GCST006061 0.0414 0.1782 537409 outcome TRUE reported 6461_54_APOC3_Apo_C_III
APOC3 chr1 63752896 0.03103 7.404e-09 35356 TRUE reported textfile
2 1 TRUE
2810 rs148077724 G A G A 0.1588 -0.0095 0.02611 NA FALSE FALSE FALSE
GCST006061 0.0214 0.6574 537409 outcome TRUE reported 6461_54_APOC3_Apo_C_III
APOC3 chr3 165952844 0.025387 3.972e-10 35340 TRUE reported
textfile 2 1 TRUE
2811 rs261332 G A G A -0.0651 -0.0047 0.18171 NA FALSE FALSE FALSE
GCST006061 0.0087 0.587 537409 outcome TRUE reported 6461_54_APOC3_Apo_C_III
APOC3 chr15 58435126 0.010538 6.497e-10 35366 TRUE reported

textfile 2 1 TRUE
2812 rs2740488 C A C A -0.0539 -0.0159 0.25976 NA FALSE FALSE FALSE
GCST006061 0.0081 0.04886 537409 outcome TRUE reported 6461_54_APOC3_Apo_C_III
APOC3 chr9 104899461 0.009284 6.405e-09 35363 TRUE reported
textfile 2 1 TRUE
2813 rs2954021 G A G A -0.0521 -0.0016 0.45781 NA FALSE FALSE FALSE
GCST006061 0.0071 0.8249 537409 outcome TRUE reported 6461_54_APOC3_Apo_C_III
APOC3 chr8 125469835 0.008211 2.221e-10 35368 TRUE reported
textfile 2 1 TRUE
2814 rs34895872 G A G A 0.0585 0.0069 0.26584 NA FALSE FALSE FALSE
GCST006061 0.0085 0.4126 537409 outcome TRUE reported 6461_54_APOC3_Apo_C_III
APOC3 chr11 48000226 0.009222 2.245e-10 35369 TRUE reported
textfile 2 1 TRUE
2815 rs4704826 A C A C 0.0759 0.0109 0.35793 NA FALSE FALSE FALSE
GCST006061 0.0075 0.1446 537409 outcome TRUE reported 6461_54_APOC3_Apo_C_III
APOC3 chr5 156965071 0.008486 3.761e-19 35351 TRUE reported
textfile 2 1 TRUE
2816 rs4921915 A G A G -0.0623 0.0167 0.18763 NA FALSE FALSE FALSE
GCST006061 0.0083 0.04427 537409 outcome TRUE reported 6461_54_APOC3_Apo_C_III
APOC3 chr8 18414956 0.01054 3.399e-09 35377 TRUE reported textfile
2 1 TRUE
2817 rs56228609 T C T C 0.0905 0.0116 0.31582 NA FALSE FALSE FALSE
GCST006061 0.0078 0.1378 537409 outcome TRUE reported 6461_54_APOC3_Apo_C_III
APOC3 chr16 56953853 0.008743 4.125e-25 35369 TRUE reported
textfile 2 1 TRUE
2818 rs6029143 T C T C -0.0845 0.0275 0.07715 NA FALSE FALSE FALSE
GCST006061 0.015 0.06765 537409 outcome TRUE reported 6461_54_APOC3_Apo_C_III
APOC3 chr20 40490022 0.015295 3.299e-08 35373 TRUE reported
textfile 2 1 TRUE
2819 rs61010704 G A G A -0.076 -0.0079 0.30256 NA FALSE FALSE FALSE
GCST006061 0.0081 0.3262 537409 outcome TRUE reported 6461_54_APOC3_Apo_C_III
APOC3 chr7 73605645 0.008847 8.692e-18 35354 TRUE reported
textfile 2 1 TRUE
2820 rs6445035 A G A G 0.1916 0.0154 0.1904 NA FALSE FALSE FALSE
GCST006061 0.0089 0.08256 537409 outcome TRUE reported 6461_54_APOC3_Apo_C_III
APOC3 chr3 165762312 0.010347 1.477e-76 35339 TRUE reported
textfile 2 1 TRUE
2821 rs73169103 T C T C 0.1486 0.0178 0.03563 NA FALSE FALSE FALSE
GCST006061 0.0249 0.4756 537409 outcome TRUE reported 6461_54_APOC3_Apo_C_III
APOC3 chr3 167190675 0.022031 1.529e-11 35354 TRUE reported
textfile 2 1 TRUE
2822 rs9267542 T C T C 0.0587 0.0017 0.22412 NA FALSE FALSE FALSE
GCST006061 0.009 0.8453 537409 outcome TRUE reported 6461_54_APOC3_Apo_C_III
APOC3 chr6 31700272 0.009756 1.779e-09 35439 TRUE reported
textfile 2 1 TRUE
2823 rs10420758 A G A G 0.14 -0.052 0.03178 NA FALSE FALSE FALSE
GCST006061 0.0249 0.037 537409 outcome TRUE reported 8760_10_DCUN1D5_DCNL5
DCUN1D5 chr19 46337314 0.024316 8.531e-09 35367 TRUE reported
textfile 2 1 TRUE
2824 rs117310449 T C T C -0.3607 -0.025 0.01434 NA FALSE FALSE FALSE
GCST006061 0.0435 0.5653 537409 outcome TRUE reported 8760_10_DCUN1D5_DCNL5
DCUN1D5 chr19 44890259 0.034534 1.55e-25 35368 TRUE reported
textfile 2 1 TRUE
2825 rs1707652 T C T C 0.0623 0.0081 0.3102 NA FALSE FALSE FALSE
GCST006061 0.0074 0.2701 537409 outcome TRUE reported 8760_10_DCUN1D5_DCNL5
DCUN1D5 chr3 165761011 0.008998 4.391e-12 35339 TRUE reported
textfile 2 1 TRUE
2826 rs190529845 A G A G -0.241 0.0484 0.01137 NA FALSE FALSE FALSE
GCST006061 0.0445 0.2759 537409 outcome TRUE reported 8760_10_DCUN1D5_DCNL5
DCUN1D5 chr19 46134118 0.039988 1.672e-09 35370 TRUE reported
textfile 2 1 TRUE
2827 rs2414577 C T C T 0.0479 -0.0167 0.41557 NA FALSE FALSE FALSE
GCST006061 0.0076 0.02713 537409 outcome TRUE reported 8760_10_DCUN1D5_DCNL5
DCUN1D5 chr15 58388439 0.008446 1.416e-08 35364 TRUE reported
textfile 2 1 TRUE
2828 rs76856627 G A G A -0.2141 0.0135 0.0343 NA FALSE FALSE FALSE

GCST006061 0.0266 0.6107 537409 outcome TRUE reported 8760_10_DCUN1D5_DCNL5
DCUN1D5 chr19 45040497 0.023737 1.887e-19 35368 TRUE reported
textfile 2 1 TRUE
2829 rs780093 C T C T -0.071 -0.0074 0.34157 NA FALSE FALSE FALSE
GCST006061 0.0073 0.3116 537409 outcome TRUE reported 8760_10_DCUN1D5_DCNL5
DCUN1D5 chr2 27519736 0.00881 7.669e-16 35373 TRUE reported textfile
2 1 TRUE
2830 rs8106813 G A G A 0.284 -0.0037 0.46477 NA FALSE FALSE FALSE
GCST006061 0.0086 0.672 537409 outcome TRUE reported 8760_10_DCUN1D5_DCNL5
DCUN1D5 chr19 44928401 0.008426 1e-200 35368 TRUE reported textfile
2 1 TRUE
2831 rs1042303 C T C T 0.0618 0.0046 0.41761 NA FALSE FALSE FALSE
GCST006061 0.0072 0.5275 537409 outcome TRUE reported 11348_132_P4HA2_P4HA2
P4HA2 chr6 24437230 0.008205 5.007e-14 35370 TRUE reported
textfile 2 1 TRUE
2832 rs12146727 A G A G -0.1771 -0.0037 0.11875 NA FALSE FALSE FALSE
GCST006061 0.0105 0.7269 537409 outcome TRUE reported 11348_132_P4HA2_P4HA2
P4HA2 chr12 7063032 0.012732 5.479e-44 35381 TRUE reported textfile
2 1 TRUE
2833 rs1355538 G A G A -0.0496 -0.0076 0.39847 NA FALSE FALSE FALSE
GCST006061 0.0072 0.2923 537409 outcome TRUE reported 11348_132_P4HA2_P4HA2
P4HA2 chr3 165787389 0.00827 2.006e-09 35329 TRUE reported textfile
2 1 TRUE
2834 rs1042303 C T C T 0.0752 0.0046 0.41761 NA FALSE FALSE FALSE
GCST006061 0.0072 0.5275 537409 outcome TRUE reported 14685_17_AKT2_PKB_beta
AKT2 chr6 24437230 0.008236 6.782e-20 35379 TRUE reported
textfile 2 1 TRUE
2835 rs146385031 C T C T 0.1555 -0.0764 0.02222 NA FALSE FALSE FALSE
GCST006061 0.0263 0.003618 537409 outcome TRUE reported
14685_17_AKT2_PKB_beta AKT2 chr6 22417343 0.028232 3.629e-08 35364
TRUE reported textfile 2 1 TRUE
2836 rs438811 T C T C -0.1198 -5.00E-04 0.23201 NA FALSE FALSE FALSE
GCST006061 0.0088 0.9556 537409 outcome TRUE reported 14685_17_AKT2_PKB_beta
AKT2 chr19 44913484 0.009674 3.198e-35 35366 TRUE reported
textfile 2 1 TRUE
2837 rs6445035 A G A G 0.1048 0.0154 0.1904 NA FALSE FALSE FALSE
GCST006061 0.0089 0.08256 537409 outcome TRUE reported 14685_17_AKT2_PKB_beta
AKT2 chr3 165762312 0.010285 2.204e-24 35339 TRUE reported
textfile 2 1 TRUE
2838 rs927985 A G A G -0.0982 0.0263 0.11724 NA FALSE FALSE FALSE
GCST006061 0.0104 0.01157 537409 outcome TRUE reported 14685_17_AKT2_PKB_beta
AKT2 chr6 25412583 0.012722 1.174e-14 35370 TRUE reported
textfile 2 1 TRUE
2839 rs1042445 T C T C -0.0521 8e-04 0.23639 NA FALSE FALSE FALSE
GCST006061 0.0083 0.9214 537409 outcome TRUE reported
4811_33_ITIH4_ITI_heavy_chain_H4 ITIH4 chr3 186677647 0.009496 4.099e-08
35368 TRUE reported textfile 2 1 TRUE
2840 rs12478841 G A G A 0.0579 0.0049 0.21569 NA FALSE FALSE FALSE
GCST006061 0.008 0.5413 537409 outcome TRUE reported
4811_33_ITIH4_ITI_heavy_chain_H4 ITIH4 chr2 27588855 0.009751 2.884e-09
35373 TRUE reported textfile 2 1 TRUE
2841 rs6807194 G A G A -0.0566 0.0072 0.32642 NA FALSE FALSE FALSE
GCST006061 0.0079 0.3579 537409 outcome TRUE reported
4811_33_ITIH4_ITI_heavy_chain_H4 ITIH4 chr3 50198940 0.008616 5.047e-11
35343 TRUE reported textfile 2 1 TRUE
2842 rs77347777 T C T C -0.1433 -0.0043 0.08722 NA FALSE FALSE FALSE
GCST006061 0.0121 0.7229 537409 outcome TRUE reported
4811_33_ITIH4_ITI_heavy_chain_H4 ITIH4 chr3 52814191 0.014533 6.199e-23
35351 TRUE reported textfile 2 1 TRUE
2843 rs1042445 T C T C 0.0762 8e-04 0.23639 NA FALSE FALSE FALSE
GCST006061 0.0083 0.9214 537409 outcome TRUE reported 12820_1_GRAP_GRAP
GRAP chr3 186677647 0.00942 5.991e-16 35359 TRUE reported textfile
2 1 TRUE
2844 rs10922098 T C T C -0.091 -0.009 0.40011 NA FALSE FALSE FALSE
GCST006061 0.0075 0.2275 537409 outcome TRUE reported 12820_1_GRAP_GRAP
GRAP chr1 196695521 0.008217 1.674e-28 35329 TRUE reported

textfile 2 1 TRUE
2845 rs1354034 C T C T 0.0497 0.0088 0.38119 NA FALSE FALSE FALSE
GCST006061 0.0073 0.2248 537409 outcome TRUE reported 12820_1_GRAP_GRAP
GRAP chr3 56815721 0.00828 1.946e-09 35347 TRUE reported textfile
2 1 TRUE
2846 rs1355538 G A G A -0.0487 -0.0076 0.39847 NA FALSE FALSE FALSE
GCST006061 0.0072 0.2923 537409 outcome TRUE reported 12820_1_GRAP_GRAP
GRAP chr3 165787389 0.008198 2.84e-09 35329 TRUE reported
textfile 2 1 TRUE
2847 rs148328786 G A G A 0.1582 0.0085 0.04808 NA FALSE FALSE FALSE
GCST006061 0.0409 0.8356 537409 outcome TRUE reported 12820_1_GRAP_GRAP
GRAP chr22 39946989 0.018583 1.693e-17 35357 TRUE reported
textfile 2 1 TRUE
2848 rs1042445 T C T C 0.1117 8e-04 0.23639 NA FALSE FALSE FALSE
GCST006061 0.0083 0.9214 537409 outcome TRUE reported 10615_18_MPZ_MYP0 MPZ
chr3 186677647 0.009703 1.142e-30 35356 TRUE reported textfile 2
1 TRUE
2849 rs114459700 C T C T 0.1701 0.0097 0.0506 NA FALSE FALSE FALSE
GCST006061 0.0271 0.7206 537409 outcome TRUE reported 10615_18_MPZ_MYP0 MPZ
chr1 231372993 0.019119 5.743e-19 35352 TRUE reported textfile 2
1 TRUE
2850 rs117801489 C T C T 0.4798 -0.0141 0.04343 NA FALSE FALSE FALSE
GCST006061 0.034 0.6772 537409 outcome TRUE reported 10615_18_MPZ_MYP0 MPZ
chr12 104015054 0.020222 1.926e-124 35349 TRUE reported textfile 2
1 TRUE
2851 rs12581309 A C A C 0.1184 -2.00E-04 0.11297 NA FALSE FALSE FALSE
GCST006061 0.0119 0.9833 537409 outcome TRUE reported 10615_18_MPZ_MYP0 MPZ
chr12 104452194 0.013019 9.519e-20 35348 TRUE reported textfile 2
1 TRUE
2852 rs13269700 G A G A 0.0513 -0.0074 0.34845 NA FALSE FALSE FALSE
GCST006061 0.0077 0.3338 537409 outcome TRUE reported 10615_18_MPZ_MYP0 MPZ
chr8 22713095 0.008729 4.18e-09 35359 TRUE reported textfile 2
1 TRUE
2853 rs139349382 A G A G 0.1587 -0.0202 0.02806 NA FALSE FALSE FALSE
GCST006061 0.027 0.454 537409 outcome TRUE reported 10615_18_MPZ_MYP0 MPZ
chr12 100232130 0.025543 5.193e-10 35353 TRUE reported textfile 2
1 TRUE
2854 rs2802788 C T C T -0.0834 -0.0068 0.18856 NA FALSE FALSE FALSE
GCST006061 0.0091 0.4554 537409 outcome TRUE reported 10615_18_MPZ_MYP0 MPZ
chr1 203459557 0.010706 6.707e-15 35356 TRUE reported textfile 2
1 TRUE
2855 rs3217964 C T C T -0.1115 -0.006 0.25916 NA FALSE FALSE FALSE
GCST006061 0.0082 0.4595 537409 outcome TRUE reported 10615_18_MPZ_MYP0 MPZ
chr7 44118226 0.00958 2.605e-31 35342 TRUE reported textfile 2 1
TRUE
2856 rs4724390 G T G T 0.0593 -1.00E-04 0.20971 NA FALSE FALSE FALSE
GCST006061 0.0081 0.9865 537409 outcome TRUE reported 10615_18_MPZ_MYP0 MPZ
chr7 45330132 0.010134 4.875e-09 35335 TRUE reported textfile 2
1 TRUE
2857 rs6541311 C T C T 0.1892 2e-04 0.15446 NA FALSE FALSE FALSE
GCST006061 0.0098 0.9844 537409 outcome TRUE reported 10615_18_MPZ_MYP0 MPZ
chr1 230288384 0.01159 6.57e-60 35348 TRUE reported textfile 2 1
TRUE
2858 rs6746339 T C T C -0.1889 0.047 0.0161 NA FALSE FALSE FALSE
GCST006061 0.0225 0.03676 537409 outcome TRUE reported 10615_18_MPZ_MYP0 MPZ
chr2 162230915 0.034041 2.869e-08 35330 TRUE reported textfile 2
1 TRUE
2859 rs6759217 T C T C 0.0472 0.0112 0.48142 NA FALSE FALSE FALSE
GCST006061 0.0072 0.118 537409 outcome TRUE reported 10615_18_MPZ_MYP0 MPZ
chr2 72027967 0.008341 1.525e-08 35339 TRUE reported textfile 2
1 TRUE
2860 rs7529009 G A G A 0.2333 -0.0066 0.43366 NA FALSE FALSE FALSE
GCST006061 0.0072 0.3587 537409 outcome TRUE reported 10615_18_MPZ_MYP0 MPZ
chr1 230096333 0.008431 1.558e-168 35344 TRUE reported textfile 2
1 TRUE
2861 rs78931415 A G A G -0.1346 0.0247 0.03536 NA FALSE FALSE FALSE

GCST006061 0.0175 0.1597 537409 outcome TRUE reported 10615_18_MPZ_MYP0 MPZ chr1 230572373 0.022488 2.16e-09 35351 TRUE reported textfile 2 1 TRUE
2862 rs79242340 C T C T -0.1936 0.0633 0.0173 NA FALSE FALSE FALSE GCST006061 0.0336 0.05928 537409 outcome TRUE reported 10615_18_MPZ_MYP0 MPZ chr1 230358909 0.033244 5.757e-09 35348 TRUE reported textfile 2 1 TRUE
2863 rs8035692 C T C T 0.0507 7e-04 0.40946 NA FALSE FALSE FALSE GCST006061 0.0073 0.9288 537409 outcome TRUE reported 10615_18_MPZ_MYP0 MPZ chr15 69117654 0.008429 1.797e-09 35364 TRUE reported textfile 2 1 TRUE
2864 rs1042445 T C T C 0.0879 8e-04 0.23639 NA FALSE FALSE FALSE GCST006061 0.0083 0.9214 537409 outcome TRUE reported 12386_11_RNPEP_AMPB RNPEP chr3 186677647 0.009572 4.189e-20 35359 TRUE reported textfile 2 1 TRUE
2865 rs10920287 T G T G 0.2825 0.0139 0.35077 NA FALSE FALSE FALSE GCST006061 0.0078 0.07729 537409 outcome TRUE reported 12386_11_RNPEP_AMPB RNPEP chr1 201973882 0.008632 1e-200 35346 TRUE reported textfile 2 1 TRUE
2866 rs112451455 A G A G 0.1462 0.0216 0.03854 NA FALSE FALSE FALSE GCST006061 0.0179 0.2284 537409 outcome TRUE reported 12386_11_RNPEP_AMPB RNPEP chr1 202132437 0.021625 1.373e-11 35349 TRUE reported textfile 2 1 TRUE
2867 rs112482827 C T C T 0.1736 -0.0153 0.0214 NA FALSE FALSE FALSE GCST006061 0.0258 0.5536 537409 outcome TRUE reported 12386_11_RNPEP_AMPB RNPEP chr1 202111519 0.029254 2.953e-09 35349 TRUE reported textfile 2 1 TRUE
2868 rs144457182 T C T C -0.2631 0.0662 0.03832 NA FALSE FALSE FALSE GCST006061 0.0225 0.003274 537409 outcome TRUE reported 12386_11_RNPEP_AMPB RNPEP chr1 203163875 0.021689 7.27e-34 35357 TRUE reported textfile 2 1 TRUE
2869 rs2678208 G T G T -0.2003 -0.0156 0.14123 NA FALSE FALSE FALSE GCST006061 0.0136 0.2496 537409 outcome TRUE reported 12386_11_RNPEP_AMPB RNPEP chr1 201853976 0.011957 5.449e-63 35347 TRUE reported textfile 2 1 TRUE
2870 rs1042445 T C T C 0.0848 8e-04 0.23639 NA FALSE FALSE FALSE GCST006061 0.0083 0.9214 537409 outcome TRUE reported 8400_74_GRP_Gastrin_releasing_peptide GRP chr3 186677647 0.009278 6.279e-20 35368 TRUE reported textfile 2 1 TRUE
2871 rs10922098 T C T C -0.1238 -0.009 0.40011 NA FALSE FALSE FALSE GCST006061 0.0075 0.2275 537409 outcome TRUE reported 8400_74_GRP_Gastrin_releasing_peptide GRP chr1 196695521 0.00808 5.397e-53 35338 TRUE reported textfile 2 1 TRUE
2872 rs1706435 G A G A 0.048 0.0117 0.36981 NA FALSE FALSE FALSE GCST006061 0.0072 0.1047 537409 outcome TRUE reported 8400_74_GRP_Gastrin_releasing_peptide GRP chr3 165743688 0.008199 4.789e-09 35339 TRUE reported textfile 2 1 TRUE
2873 rs1042445 T C T C 0.0631 8e-04 0.23639 NA FALSE FALSE FALSE GCST006061 0.0083 0.9214 537409 outcome TRUE reported 10924_258_CYB5D2_NEUFC CYB5D2 chr3 186677647 0.009261 9.508e-12 35357 TRUE reported textfile 2 1 TRUE
2874 rs10922098 T C T C -0.0507 -0.009 0.40011 NA FALSE FALSE FALSE GCST006061 0.0075 0.2275 537409 outcome TRUE reported 10924_258_CYB5D2_NEUFC CYB5D2 chr1 196695521 0.008069 3.309e-10 35327 TRUE reported textfile 2 1 TRUE
2875 rs149208504 C T C T -0.6911 0.0389 0.02263 NA FALSE FALSE FALSE GCST006061 0.0359 0.2782 537409 outcome TRUE reported 10924_258_CYB5D2_NEUFC CYB5D2 chr17 4011662 0.025794 3.92e-158 35358 TRUE reported textfile 2 1 TRUE
2876 rs17176065 T C T C 0.3578 0.0521 0.0447 NA FALSE FALSE FALSE GCST006061 0.0227 0.02176 537409 outcome TRUE reported 10924_258_CYB5D2_NEUFC CYB5D2 chr17 4058560 0.018882 4.497e-80 35361 TRUE reported textfile 2 1 TRUE
2877 rs181444731 A G A G -0.1765 0.0056 0.01669 NA FALSE FALSE FALSE GCST006061 0.0515 0.9132 537409 outcome TRUE reported 10924_258_CYB5D2_NEUFC CYB5D2 chr17 4387026 0.030387 6.305e-09 35359 TRUE reported textfile

2 1 TRUE
2878 rs2854001 A G A G 0.1288 0.0014 0.20994 NA FALSE FALSE FALSE
GCST006061 0.0095 0.8845 537409 outcome TRUE reported 10924_258_CYB5D2_NEUFC
CYB5D2 chr6 31355235 0.009671 1.804e-40 35429 TRUE reported
textfile 2 1 TRUE
2879 rs7224464 T C T C -0.0531 -0.0045 0.29 NA FALSE FALSE FALSE
GCST006061 0.0083 0.5846 537409 outcome TRUE reported 10924_258_CYB5D2_NEUFC
CYB5D2 chr17 4449244 0.00876 1.348e-09 35362 TRUE reported textfile 2
1 TRUE
2880 rs10426931 T C T C 0.0472 -0.0038 0.42536 NA FALSE FALSE FALSE
GCST006061 0.0084 0.6509 537409 outcome TRUE reported 5638_23_COLGALT1_GT251
COLGALT1 chr19 17587742 0.008264 1.121e-08 35348 TRUE reported
textfile 2 1 TRUE
2881 rs13225119 A G A G 0.0466 -0.0136 0.45479 NA FALSE FALSE FALSE
GCST006061 0.0073 0.06162 537409 outcome TRUE reported 5638_23_COLGALT1_GT251
COLGALT1 chr7 6579021 0.008226 1.468e-08 35363 TRUE reported
textfile 2 1 TRUE
2882 rs144577567 A G A G 0.1908 0.0717 0.01579 NA FALSE FALSE FALSE
GCST006061 0.0403 0.07558 537409 outcome TRUE reported 5638_23_COLGALT1_GT251
COLGALT1 chr19 16194091 0.033479 1.205e-08 35362 TRUE reported
textfile 2 1 TRUE
2883 rs2169387 G A G A -0.1091 -0.0154 0.0785 NA FALSE FALSE FALSE
GCST006061 0.0125 0.215 537409 outcome TRUE reported 5638_23_COLGALT1_GT251
COLGALT1 chr8 9323885 0.015174 6.491e-13 35385 TRUE reported
textfile 2 1 TRUE
2884 rs35745856 C T C T -0.2289 0.0624 0.01039 NA FALSE FALSE FALSE
GCST006061 0.0583 0.2846 537409 outcome TRUE reported 5638_23_COLGALT1_GT251
COLGALT1 chr19 18444394 0.040395 1.457e-08 35352 TRUE reported
textfile 2 1 TRUE
2885 rs3752617 T G T G -0.1134 0.0059 0.06955 NA FALSE FALSE FALSE
GCST006061 0.0148 0.6885 537409 outcome TRUE reported 5638_23_COLGALT1_GT251
COLGALT1 chr7 127580863 0.016357 4.125e-12 35355 TRUE reported
textfile 2 1 TRUE
2886 rs4340676 G A G A -0.1085 0.0337 0.17993 NA FALSE FALSE FALSE
GCST006061 0.0097 0.0005006 537409 outcome TRUE reported
5638_23_COLGALT1_GT251 COLGALT1 chr3 57596361 0.010645 2.13e-24 35359
TRUE reported textfile 2 1 TRUE
2887 rs4808666 T C T C -0.1275 -0.0031 0.44648 NA FALSE FALSE FALSE
GCST006061 0.0076 0.6863 537409 outcome TRUE reported 5638_23_COLGALT1_GT251
COLGALT1 chr19 17557324 0.008253 7.6e-54 35344 TRUE reported
textfile 2 1 TRUE
2888 rs4847398 T C T C -0.0531 0.0034 0.39715 NA FALSE FALSE FALSE
GCST006061 0.0073 0.6416 537409 outcome TRUE reported 5638_23_COLGALT1_GT251
COLGALT1 chr1 93112845 0.008362 2.152e-10 35353 TRUE reported
textfile 2 1 TRUE
2889 rs6796 C T C T 0.226 0.0015 0.23682 NA FALSE FALSE FALSE GCST006061
0.0081 0.857 537409 outcome TRUE reported 5638_23_COLGALT1_GT251 COLGALT1
chr7 6462736 0.009553 1.009e-123 35364 TRUE reported textfile 2 1
TRUE
2890 rs140614696 T C T C 0.2068 0.0109 0.05432 NA FALSE FALSE FALSE
GCST006061 0.0176 0.5332 537409 outcome TRUE reported 4342_10_ICAM1_sICAM_1
ICAM1 chr19 13736467 0.020606 1.059e-23 35375 TRUE reported
textfile 2 1 TRUE
2891 rs35186095 T C T C -0.6455 0.0345 0.16987 NA FALSE FALSE FALSE
GCST006061 0.0099 0.0004894 537409 outcome TRUE reported
4342_10_ICAM1_sICAM_1 ICAM1 chr19 10307575 0.00709 1e-200 35377 TRUE
reported textfile 2 1 TRUE
2892 rs62118265 G A G A -0.1318 0.002 0.22043 NA FALSE FALSE FALSE
GCST006061 0.0103 0.8431 537409 outcome TRUE reported 4342_10_ICAM1_sICAM_1
ICAM1 chr19 8907234 0.0113 1.944e-31 35377 TRUE reported textfile 2
1 TRUE
2893 rs635634 C T C T 0.2294 0.0285 0.12995 NA FALSE FALSE FALSE
GCST006061 0.0089 0.001351 537409 outcome TRUE reported
4342_10_ICAM1_sICAM_1 ICAM1 chr9 133279427 0.010565 1.529e-104 35371
TRUE reported textfile 2 1 TRUE
2894 rs6603148 T G T G -0.1201 -0.0064 0.0743 NA FALSE FALSE FALSE

GCST006061 0.013 0.6227 537409 outcome TRUE reported 4342_10_ICAM1_sICAM_1
ICAM1 chr19 8135875 0.018232 4.479e-11 35376 TRUE reported textfile
2 1 TRUE
2895 rs75410819 T C T C -0.2801 -0.0088 0.04583 NA FALSE FALSE FALSE
GCST006061 0.0218 0.6859 537409 outcome TRUE reported 4342_10_ICAM1_sICAM_1
ICAM1 chr19 12683044 0.022685 5.031e-35 35380 TRUE reported
textfile 2 1 TRUE
2896 rs78374606 A G A G 0.3365 -0.0061 0.02099 NA FALSE FALSE FALSE
GCST006061 0.0392 0.8774 537409 outcome TRUE reported 4342_10_ICAM1_sICAM_1
ICAM1 chr19 10108401 0.032236 1.654e-25 35373 TRUE reported
textfile 2 1 TRUE
2897 rs1042704 A G A G -0.1298 0.01 0.18511 NA FALSE FALSE FALSE
GCST006061 0.0094 0.2878 537409 outcome TRUE reported 9005_16_PLXNA1_PLXA1
PLXNA1 chr14 22843385 0.010515 5.228e-35 35373 TRUE reported
textfile 2 1 TRUE
2898 rs1260326 C T C T 0.0603 -0.0069 0.34132 NA FALSE FALSE FALSE
GCST006061 0.0073 0.3401 537409 outcome TRUE reported 9005_16_PLXNA1_PLXA1
PLXNA1 chr2 27508073 0.0087 4.168e-12 35373 TRUE reported textfile
2 1 TRUE
2899 rs12695485 C T C T -0.08 -0.0117 0.12975 NA FALSE FALSE FALSE
GCST006061 0.0107 0.2717 537409 outcome TRUE reported 9005_16_PLXNA1_PLXA1
PLXNA1 chr3 126392261 0.012273 7.103e-11 35371 TRUE reported
textfile 2 1 TRUE
2900 rs4679138 A G A G 0.2543 0.0027 0.30365 NA FALSE FALSE FALSE
GCST006061 0.0076 0.7183 537409 outcome TRUE reported 9005_16_PLXNA1_PLXA1
PLXNA1 chr3 126992227 0.008833 2.948e-182 35369 TRUE reported
textfile 2 1 TRUE
2901 rs55714927 T C T C 0.0709 1e-04 0.21166 NA FALSE FALSE FALSE
GCST006061 0.0098 0.9926 537409 outcome TRUE reported 9005_16_PLXNA1_PLXA1
PLXNA1 chr17 7176997 0.010105 2.273e-12 35370 TRUE reported textfile
2 1 TRUE
2902 rs56344867 C T C T 0.071 -0.005 0.35465 NA FALSE FALSE FALSE
GCST006061 0.008 0.5298 537409 outcome TRUE reported 9005_16_PLXNA1_PLXA1
PLXNA1 chr11 126361219 0.008585 1.338e-16 35374 TRUE reported
textfile 2 1 TRUE
2903 rs79118580 T C T C -0.2176 -0.0394 0.01292 NA FALSE FALSE FALSE
GCST006061 0.0269 0.1429 537409 outcome TRUE reported 9005_16_PLXNA1_PLXA1
PLXNA1 chr3 127110472 0.037139 4.655e-09 35367 TRUE reported
textfile 2 1 TRUE
2904 rs10427049 G A G A -0.0916 -0.0132 0.08773 NA FALSE FALSE FALSE
GCST006061 0.0119 0.2702 537409 outcome TRUE reported 3195_50_GNLY_Granulysin
GNLY chr19 8679639 0.014312 1.55e-10 35370 TRUE reported textfile
2 1 TRUE
2905 rs10732976 A G A G -0.059 -0.0017 0.27908 NA FALSE FALSE FALSE
GCST006061 0.0077 0.8292 537409 outcome TRUE reported 3195_50_GNLY_Granulysin
GNLY chr1 185439929 0.008988 5.235e-11 35356 TRUE reported
textfile 2 1 TRUE
2906 rs10780319 C A C A -0.0483 -0.0161 0.33607 NA FALSE FALSE FALSE
GCST006061 0.0075 0.03282 537409 outcome TRUE reported 3195_50_GNLY_Granulysin
GNLY chr9 79662302 0.008478 1.217e-08 35351 TRUE reported
textfile 2 1 TRUE
2907 rs1130866 A G A G -0.1457 0.0014 0.47918 NA FALSE FALSE FALSE
GCST006061 0.0073 0.8478 537409 outcome TRUE reported 3195_50_GNLY_Granulysin
GNLY chr2 85666618 0.008675 2.598e-63 35344 TRUE reported
textfile 2 1 TRUE
2908 rs113974174 T C T C 0.2014 -0.0016 0.05467 NA FALSE FALSE FALSE
GCST006061 0.0178 0.9269 537409 outcome TRUE reported 3195_50_GNLY_Granulysin
GNLY chr2 95391021 0.019143 6.925e-26 35361 TRUE reported
textfile 2 1 TRUE
2909 rs11993233 G A G A 0.0537 0.0085 0.40657 NA FALSE FALSE FALSE
GCST006061 0.0074 0.2489 537409 outcome TRUE reported 3195_50_GNLY_Granulysin
GNLY chr8 143928115 0.008262 8.036e-11 34630 TRUE reported
textfile 2 1 TRUE
2910 rs13017718 T C T C -0.1254 -4.00E-04 0.04737 NA FALSE FALSE FALSE
GCST006061 0.0181 0.9828 537409 outcome TRUE reported 3195_50_GNLY_Granulysin
GNLY chr2 100937574 0.020432 8.39e-10 35361 TRUE reported

textfile 2 1 TRUE
2911 rs13028852 A C A C 0.1742 -0.0456 0.06534 NA FALSE FALSE FALSE
GCST006061 0.019 0.0165 537409 outcome TRUE reported 3195_50_GNLY_Granulysin
GNLY chr2 86686390 0.017364 1.098e-23 35344 TRUE reported
textfile 2 1 TRUE
2912 rs183053322 T C T C -0.3256 -0.0158 0.01327 NA FALSE FALSE FALSE
GCST006061 0.049 0.7476 537409 outcome TRUE reported 3195_50_GNLY_Granulysin
GNLY chr1 24749568 0.034305 2.282e-21 35371 TRUE reported
textfile 2 1 TRUE
2913 rs2497304 T C T C 0.0562 0.0054 0.49358 NA FALSE FALSE FALSE
GCST006061 0.0074 0.4657 537409 outcome TRUE reported 3195_50_GNLY_Granulysin
GNLY chr10 92732959 0.008078 3.477e-12 35373 TRUE reported
textfile 2 1 TRUE
2914 rs27295 T C T C -0.0812 0.0157 0.45961 NA FALSE FALSE FALSE GCST006061
0.0072 0.02864 537409 outcome TRUE reported 3195_50_GNLY_Granulysin GNLY
chr5 97022983 0.008037 5.311e-24 35344 TRUE reported textfile 2
1 TRUE
2915 rs3024798 T G T G -0.3173 0.005 0.3051 NA FALSE FALSE FALSE
GCST006061 0.0077 0.5152 537409 outcome TRUE reported 3195_50_GNLY_Granulysin
GNLY chr2 85667185 0.009264 1e-200 35344 TRUE reported textfile
2 1 TRUE
2916 rs3732477 T C T C -0.0566 -0.0092 0.22988 NA FALSE FALSE FALSE
GCST006061 0.0078 0.2421 537409 outcome TRUE reported 3195_50_GNLY_Granulysin
GNLY chr3 194341790 0.00961 3.874e-09 35299 TRUE reported textfile
2 1 TRUE
2917 rs4141634 G A G A 0.0489 -0.0053 0.39916 NA FALSE FALSE FALSE
GCST006061 0.0076 0.4889 537409 outcome TRUE reported 3195_50_GNLY_Granulysin
GNLY chr2 102060608 0.008888 3.759e-08 35368 TRUE reported
textfile 2 1 TRUE
2918 rs56054119 C T C T -0.1163 -0.0059 0.33102 NA FALSE FALSE FALSE
GCST006061 0.0078 0.452 537409 outcome TRUE reported 3195_50_GNLY_Granulysin
GNLY chr2 85808010 0.009216 1.649e-36 35341 TRUE reported
textfile 2 1 TRUE
2919 rs607268 C T C T 0.0634 0.0088 0.33888 NA FALSE FALSE FALSE
GCST006061 0.0075 0.2411 537409 outcome TRUE reported 3195_50_GNLY_Granulysin
GNLY chr1 56913999 0.008465 6.887e-14 35369 TRUE reported
textfile 2 1 TRUE
2920 rs61751507 T C T C -0.2806 0.0047 0.04162 NA FALSE FALSE FALSE
GCST006061 0.018 0.794 537409 outcome TRUE reported 3195_50_GNLY_Granulysin
GNLY chr10 100069757 0.020023 1.284e-44 35368 TRUE reported
textfile 2 1 TRUE
2921 rs6543485 C T C T -0.1893 0.0027 0.03858 NA FALSE FALSE FALSE
GCST006061 0.0247 0.9127 537409 outcome TRUE reported 3195_50_GNLY_Granulysin
GNLY chr2 98305192 0.02234 2.376e-17 35360 TRUE reported textfile
2 1 TRUE
2922 rs7040705 A G A G 0.0746 -7.00E-04 0.43804 NA FALSE FALSE FALSE
GCST006061 0.0074 0.9216 537409 outcome TRUE reported 3195_50_GNLY_Granulysin
GNLY chr9 136941910 0.008105 3.425e-20 35258 TRUE reported
textfile 2 1 TRUE
2923 rs75124059 G T G T 0.1176 -0.013 0.10435 NA FALSE FALSE FALSE
GCST006061 0.0111 0.24 537409 outcome TRUE reported 3195_50_GNLY_Granulysin
GNLY chr2 84728005 0.014251 1.558e-16 35346 TRUE reported
textfile 2 1 TRUE
2924 rs7595080 A G A G 0.1358 0.0086 0.37715 NA FALSE FALSE FALSE
GCST006061 0.0074 0.2471 537409 outcome TRUE reported 3195_50_GNLY_Granulysin
GNLY chr2 85447487 0.008953 5.696e-52 35343 TRUE reported
textfile 2 1 TRUE
2925 rs7620997 T C T C 0.0594 -0.011 0.34162 NA FALSE FALSE FALSE
GCST006061 0.0073 0.1347 537409 outcome TRUE reported 3195_50_GNLY_Granulysin
GNLY chr3 47172632 0.008481 2.488e-12 35343 TRUE reported
textfile 2 1 TRUE
2926 rs79507667 G T G T -0.2714 -0.0017 0.01451 NA FALSE FALSE FALSE
GCST006061 0.0294 0.9537 537409 outcome TRUE reported 3195_50_GNLY_Granulysin
GNLY chr6 33251387 0.034477 3.49e-15 35422 TRUE reported
textfile 2 1 TRUE
2927 rs1043160 G A G A 0.0779 -0.0069 0.34817 NA FALSE FALSE FALSE

GCST006061 0.0073 0.3486 537409 outcome TRUE reported 3329_14_PGLYRP1_PGRP_S
PGLYRP1 chr2 219173034 0.008614 1.516e-19 35369 TRUE reported
textfile 2 1 TRUE
2928 rs11667174 A G A G -0.0887 -0.0156 0.1081 NA FALSE FALSE FALSE
GCST006061 0.0133 0.2399 537409 outcome TRUE reported 3329_14_PGLYRP1_PGRP_S
PGLYRP1 chr19 46231636 0.013112 1.335e-11 35368 TRUE reported
textfile 2 1 TRUE
2929 rs11881751 A G A G -0.0567 -0.003 0.21088 NA FALSE FALSE FALSE
GCST006061 0.009 0.7347 537409 outcome TRUE reported 3329_14_PGLYRP1_PGRP_S
PGLYRP1 chr19 4997409 0.010038 1.619e-08 35362 TRUE reported textfile
2 1 TRUE
2930 rs13346756 C T C T 0.1084 0.0201 0.04809 NA FALSE FALSE FALSE
GCST006061 0.0229 0.3822 537409 outcome TRUE reported 3329_14_PGLYRP1_PGRP_S
PGLYRP1 chr19 46523625 0.019088 1.355e-08 35369 TRUE reported
textfile 2 1 TRUE
2931 rs2072563 A G A G -0.1912 0.0034 0.31858 NA FALSE FALSE FALSE
GCST006061 0.0078 0.6642 537409 outcome TRUE reported 3329_14_PGLYRP1_PGRP_S
PGLYRP1 chr19 46023390 0.008708 7.363e-107 35369 TRUE reported
textfile 2 1 TRUE
2932 rs10432210 T C T C 0.0986 0.003 0.1773 NA FALSE FALSE FALSE
GCST006061 0.0085 0.7258 537409 outcome TRUE reported 17454_15_EGFL6_EGFL6
EGFL6 chr18 25281830 0.0109 1.48e-19 35367 TRUE reported textfile
2 1 TRUE
2933 rs11700925 T C T C -0.0526 0.0035 0.26886 NA FALSE FALSE FALSE
GCST006061 0.0087 0.6901 537409 outcome TRUE reported 17454_15_EGFL6_EGFL6
EGFL6 chr21 34865359 0.009335 1.752e-08 35374 TRUE reported
textfile 2 1 TRUE
2934 rs12022510 G A G A 0.0964 -0.0025 0.07399 NA FALSE FALSE FALSE
GCST006061 0.0108 0.8137 537409 outcome TRUE reported 17454_15_EGFL6_EGFL6
EGFL6 chr1 54379015 0.015719 8.636e-10 35363 TRUE reported
textfile 2 1 TRUE
2935 rs2032903 C T C T 0.1603 0.0031 0.38302 NA FALSE FALSE FALSE
GCST006061 0.0075 0.6793 537409 outcome TRUE reported 17454_15_EGFL6_EGFL6
EGFL6 chr19 14684737 0.008534 1.032e-78 35379 TRUE reported
textfile 2 1 TRUE
2936 rs3780420 C T C T 0.0663 -0.0063 0.35496 NA FALSE FALSE FALSE
GCST006061 0.0075 0.4019 537409 outcome TRUE reported 17454_15_EGFL6_EGFL6
EGFL6 chr9 97914884 0.008664 1.98e-14 35345 TRUE reported
textfile 2 1 TRUE
2937 rs7280028 C T C T 0.0603 -0.0068 0.20408 NA FALSE FALSE FALSE
GCST006061 0.0096 0.4787 537409 outcome TRUE reported 17454_15_EGFL6_EGFL6
EGFL6 chr21 35044543 0.010283 4.512e-09 35376 TRUE reported
textfile 2 1 TRUE
2938 rs1043314 G A G A -0.0628 0.001 0.463 NA FALSE FALSE FALSE
GCST006061 0.0073 0.8865 537409 outcome TRUE reported 5252_33_PDE11A_PDE11
PDE11A chr11 119045232 0.007873 1.506e-15 35380 TRUE reported
textfile 2 1 TRUE
2939 rs2588512 G A G A -0.0544 -0.003 0.19505 NA FALSE FALSE FALSE
GCST006061 0.0088 0.7359 537409 outcome TRUE reported 5252_33_PDE11A_PDE11
PDE11A chr2 54964946 0.009916 4.112e-08 35346 TRUE reported
textfile 2 1 TRUE
2940 rs75327431 C T C T -0.0963 0.0083 0.05347 NA FALSE FALSE FALSE
GCST006061 0.0222 0.7083 537409 outcome TRUE reported 5252_33_PDE11A_PDE11
PDE11A chr12 42802980 0.017215 2.219e-08 35361 TRUE reported
textfile 2 1 TRUE
2941 rs10435096 C T C T 0.0831 -0.0092 0.08674 NA FALSE FALSE FALSE
GCST006061 0.0112 0.4105 537409 outcome TRUE reported 4324_33_CST2_CYTT
CST2 chr7 26060150 0.01437 7.342e-09 35372 TRUE reported textfile
2 1 TRUE
2942 rs113166859 A G A G 0.2126 0.0216 0.01481 NA FALSE FALSE FALSE
GCST006061 0.0306 0.4816 537409 outcome TRUE reported 4324_33_CST2_CYTT
CST2 chr20 24878141 0.035412 1.93e-09 35366 TRUE reported
textfile 2 1 TRUE
2943 rs141264356 A G A G -0.1808 -0.0241 0.01881 NA FALSE FALSE FALSE
GCST006061 0.0432 0.5774 537409 outcome TRUE reported 4324_33_CST2_CYTT
CST2 chr20 22709866 0.032325 2.23e-08 35370 TRUE reported

textfile 2 1 TRUE
2944 rs4246778 A G A G -0.0707 0.0024 0.40474 NA FALSE FALSE FALSE
GCST006061 0.0072 0.7352 537409 outcome TRUE reported 4324_33_CST2_CYTT
CST2 chr5 135183655 0.008281 1.373e-17 35359 TRUE reported
textfile 2 1 TRUE
2945 rs55733944 A C A C 0.0468 -0.0062 0.47383 NA FALSE FALSE FALSE
GCST006061 0.0076 0.4083 537409 outcome TRUE reported 4324_33_CST2_CYTT
CST2 chr11 34486879 0.00817 1.015e-08 35376 TRUE reported textfile
2 1 TRUE
2946 rs6049023 C A C A -0.4573 3e-04 0.09412 NA FALSE FALSE FALSE
GCST006061 0.0117 0.9787 537409 outcome TRUE reported 4324_33_CST2_CYTT
CST2 chr20 23721306 0.014446 1e-200 35363 TRUE reported textfile
2 1 TRUE
2947 rs6132718 T C T C -0.1051 -0.0092 0.06057 NA FALSE FALSE FALSE
GCST006061 0.0153 0.5481 537409 outcome TRUE reported 4324_33_CST2_CYTT
CST2 chr20 24245717 0.017854 3.944e-09 35366 TRUE reported
textfile 2 1 TRUE
2948 rs6137875 A G A G -0.1988 0.0043 0.12534 NA FALSE FALSE FALSE
GCST006061 0.0098 0.6633 537409 outcome TRUE reported 4324_33_CST2_CYTT
CST2 chr20 23244537 0.012816 2.907e-54 35366 TRUE reported
textfile 2 1 TRUE
2949 rs62208869 T C T C -0.3603 -0.0147 0.14808 NA FALSE FALSE FALSE
GCST006061 0.0096 0.1241 537409 outcome TRUE reported 4324_33_CST2_CYTT
CST2 chr20 23632342 0.011807 1e-200 35365 TRUE reported textfile
2 1 TRUE
2950 rs62213165 G A G A -0.1238 0.0197 0.073 NA FALSE FALSE FALSE
GCST006061 0.0203 0.3334 537409 outcome TRUE reported 4324_33_CST2_CYTT
CST2 chr20 36010404 0.016096 1.458e-14 35365 TRUE reported
textfile 2 1 TRUE
2951 rs6427943 A G A G -0.0947 0.0238 0.4103 NA FALSE FALSE FALSE
GCST006061 0.0073 0.001097 537409 outcome TRUE reported 4324_33_CST2_CYTT
CST2 chr1 202217817 0.008265 2.14e-30 35361 TRUE reported
textfile 2 1 TRUE
2952 rs67020211 G T G T -0.092 1e-04 0.4901 NA FALSE FALSE FALSE
GCST006061 0.0073 0.9882 537409 outcome TRUE reported 4324_33_CST2_CYTT
CST2 chr15 63347445 0.008096 6.305e-30 35362 TRUE reported
textfile 2 1 TRUE
2953 rs681343 T C T C 0.0732 0.0054 0.3954 NA FALSE FALSE FALSE
GCST006061 0.0076 0.4742 537409 outcome TRUE reported 4324_33_CST2_CYTT
CST2 chr19 48703205 0.008323 1.436e-18 35377 TRUE reported
textfile 2 1 TRUE
2954 rs73125764 A G A G -0.151 -0.0128 0.06822 NA FALSE FALSE FALSE
GCST006061 0.0217 0.557 537409 outcome TRUE reported 4324_33_CST2_CYTT
CST2 chr20 22092986 0.017027 7.426e-19 35373 TRUE reported
textfile 2 1 TRUE
2955 rs77709598 G A G A 0.1308 -0.0071 0.06676 NA FALSE FALSE FALSE
GCST006061 0.0196 0.7178 537409 outcome TRUE reported 4324_33_CST2_CYTT
CST2 chr20 23021281 0.016836 7.916e-15 35368 TRUE reported
textfile 2 1 TRUE
2956 rs836124 G T G T 0.0607 -0.0027 0.42792 NA FALSE FALSE FALSE
GCST006061 0.0072 0.708 537409 outcome TRUE reported 4324_33_CST2_CYTT
CST2 chr11 34540700 0.008234 1.687e-13 35378 TRUE reported
textfile 2 1 TRUE
2957 rs848097 C T C T 0.0602 -0.0121 0.39216 NA FALSE FALSE FALSE
GCST006061 0.0072 0.09641 537409 outcome TRUE reported 4324_33_CST2_CYTT
CST2 chr14 36792277 0.008333 5.04e-13 35360 TRUE reported
textfile 2 1 TRUE
2958 rs10437383 A G A G 0.1451 -0.0067 0.34214 NA FALSE FALSE FALSE
GCST006061 0.0078 0.3961 537409 outcome TRUE reported 3000_66_MBL2_MBL
MBL2 chr10 52164008 0.008699 1.82e-62 35359 TRUE reported
textfile 2 1 TRUE
2959 rs10823734 G A G A 0.0742 -0.0028 0.38484 NA FALSE FALSE FALSE
GCST006061 0.0077 0.7096 537409 outcome TRUE reported 3000_66_MBL2_MBL
MBL2 chr10 51860626 0.008476 2.064e-18 35359 TRUE reported
textfile 2 1 TRUE
2960 rs11002760 G A G A -0.5566 -0.0363 0.03271 NA FALSE FALSE FALSE

GCST006061 0.0235 0.1227 537409 outcome TRUE reported 3000_66_MBL2_MBL
MBL2 chr10 52617801 0.023489 3.924e-124 35357 TRUE reported
textfile 2 1 TRUE
2961 rs117573752 T G T G 0.2176 3e-04 0.05425 NA FALSE FALSE FALSE
GCST006061 0.0232 0.9892 537409 outcome TRUE reported 3000_66_MBL2_MBL
MBL2 chr10 55348008 0.018002 1.23e-33 35358 TRUE reported
textfile 2 1 TRUE
2962 rs117917923 T C T C -0.2555 -0.031 0.01906 NA FALSE FALSE FALSE
GCST006061 0.025 0.2135 537409 outcome TRUE reported 3000_66_MBL2_MBL
MBL2 chr10 54862132 0.02982 1.053e-17 35356 TRUE reported textfile
2 1 TRUE
2963 rs117944916 T C T C -0.1445 0.0731 0.02727 NA FALSE FALSE FALSE
GCST006061 0.0544 0.1789 537409 outcome TRUE reported 3000_66_MBL2_MBL
MBL2 chr10 36528941 0.0247 4.913e-09 35340 TRUE reported textfile
2 1 TRUE
2964 rs1260326 C T C T 0.0688 -0.0069 0.34132 NA FALSE FALSE FALSE
GCST006061 0.0073 0.3401 537409 outcome TRUE reported 3000_66_MBL2_MBL
MBL2 chr2 27508073 0.007393 1.333e-20 35373 TRUE reported
textfile 2 1 TRUE
2965 rs150524062 A G A G 0.149 -6.00E-04 0.07904 NA FALSE FALSE FALSE
GCST006061 0.019 0.9744 537409 outcome TRUE reported 3000_66_MBL2_MBL
MBL2 chr10 52987008 0.015347 2.768e-22 35352 TRUE reported
textfile 2 1 TRUE
2966 rs1916565 A G A G -0.1876 -0.0099 0.09808 NA FALSE FALSE FALSE
GCST006061 0.0109 0.3627 537409 outcome TRUE reported 3000_66_MBL2_MBL
MBL2 chr10 49677255 0.013737 1.85e-42 35353 TRUE reported
textfile 2 1 TRUE
2967 rs2073925 A G A G 0.1086 -0.0107 0.05706 NA FALSE FALSE FALSE
GCST006061 0.0141 0.4457 537409 outcome TRUE reported 3000_66_MBL2_MBL
MBL2 chr9 133161872 0.015445 2.045e-12 35368 TRUE reported
textfile 2 1 TRUE
2968 rs2261695 T C T C 0.1226 0.0053 0.17544 NA FALSE FALSE FALSE
GCST006061 0.0095 0.5767 537409 outcome TRUE reported 3000_66_MBL2_MBL
MBL2 chr1 11026382 0.009138 4.814e-41 35388 TRUE reported
textfile 2 1 TRUE
2969 rs2578062 C T C T -0.0697 0.0162 0.17209 NA FALSE FALSE FALSE
GCST006061 0.0111 0.1452 537409 outcome TRUE reported 3000_66_MBL2_MBL
MBL2 chr10 63972695 0.010873 1.45e-10 35350 TRUE reported
textfile 2 1 TRUE
2970 rs34529023 C T C T -0.2677 0.0024 0.03011 NA FALSE FALSE FALSE
GCST006061 0.0213 0.9089 537409 outcome TRUE reported 3000_66_MBL2_MBL
MBL2 chr10 53812313 0.024043 8.531e-29 35356 TRUE reported
textfile 2 1 TRUE
2971 rs71503158 A G A G 0.1214 -0.017 0.02658 NA FALSE FALSE FALSE
GCST006061 0.0382 0.6575 537409 outcome TRUE reported 3000_66_MBL2_MBL
MBL2 chr9 132811227 0.021779 2.486e-08 35373 TRUE reported
textfile 2 1 TRUE
2972 rs7475006 C T C T -0.2644 -0.004 0.38843 NA FALSE FALSE FALSE
GCST006061 0.0073 0.5813 537409 outcome TRUE reported 3000_66_MBL2_MBL
MBL2 chr10 52798850 0.00834 1e-200 35361 TRUE reported textfile 2
1 TRUE
2973 rs10437425 G T G T -0.0619 0.0072 0.27349 NA FALSE FALSE FALSE
GCST006061 0.0078 0.3515 537409 outcome TRUE reported
18925_24_PSMA5_Proteasome_subunit_alpha_type_5 PSMA5 chr10 20078274 0.009113
1.102e-11 35363 TRUE reported textfile 2 1 TRUE
2974 rs10764161 T C T C -0.0818 -0.0053 0.4988 NA FALSE FALSE FALSE
GCST006061 0.0071 0.4565 537409 outcome TRUE reported
18925_24_PSMA5_Proteasome_subunit_alpha_type_5 PSMA5 chr10 19797425 0.008091
4.965e-24 35358 TRUE reported textfile 2 1 TRUE
2975 rs112616165 A G A G -0.0934 -0.0844 0.06729 NA FALSE FALSE FALSE
GCST006061 0.0177 1.764e-06 537409 outcome TRUE reported
18925_24_PSMA5_Proteasome_subunit_alpha_type_5 PSMA5 chr1 170878414 0.016432
1.315e-08 35359 TRUE reported textfile 2 1 TRUE
2976 rs12132412 G A G A -0.0649 0.0055 0.34943 NA FALSE FALSE FALSE
GCST006061 0.0077 0.4794 537409 outcome TRUE reported
18925_24_PSMA5_Proteasome_subunit_alpha_type_5 PSMA5 chr1 21493549 0.008603

4.564e-14 35374 TRUE reported textfile 2 1 TRUE
2977 rs1591734 T C T C -0.1478 0.0198 0.1303 NA FALSE FALSE FALSE
GCST006061 0.0103 0.05506 537409 outcome TRUE reported
18925_24_PSMA5_Proteasome_subunit_alpha_type_5 PSMA5 chr1 169302664 0.012232
1.293e-33 35364 TRUE reported textfile 2 1 TRUE
2978 rs594793 T G T G -0.0455 0.0085 0.43083 NA FALSE FALSE FALSE
GCST006061 0.0076 0.2613 537409 outcome TRUE reported
18925_24_PSMA5_Proteasome_subunit_alpha_type_5 PSMA5 chr19 7765060 0.008236
3.299e-08 35373 TRUE reported textfile 2 1 TRUE
2979 rs6544941 A G A G -0.0642 0.0035 0.33068 NA FALSE FALSE FALSE
GCST006061 0.0077 0.6538 537409 outcome TRUE reported
18925_24_PSMA5_Proteasome_subunit_alpha_type_5 PSMA5 chr2 46947977 0.008644
1.109e-13 35358 TRUE reported textfile 2 1 TRUE
2980 rs6670678 G A G A -0.1622 0.0024 0.47186 NA FALSE FALSE FALSE
GCST006061 0.0073 0.7376 537409 outcome TRUE reported
18925_24_PSMA5_Proteasome_subunit_alpha_type_5 PSMA5 chr1 169568440 0.008163
7.351e-88 35360 TRUE reported textfile 2 1 TRUE
2981 rs7070003 T C T C 0.1723 -0.0121 0.27929 NA FALSE FALSE FALSE
GCST006061 0.0079 0.1277 537409 outcome TRUE reported
18925_24_PSMA5_Proteasome_subunit_alpha_type_5 PSMA5 chr10 19983733 0.009096
5.012e-80 35360 TRUE reported textfile 2 1 TRUE
2982 rs9332701 G A G A -0.3935 -0.0115 0.07189 NA FALSE FALSE FALSE
GCST006061 0.0196 0.5575 537409 outcome TRUE reported
18925_24_PSMA5_Proteasome_subunit_alpha_type_5 PSMA5 chr1 169515529 0.015598
1.982e-140 35364 TRUE reported textfile 2 1 TRUE
2983 rs10437655 A G A G -0.0524 0.0098 0.37172 NA FALSE FALSE FALSE
GCST006061 0.0075 0.1881 537409 outcome TRUE reported 7980_72_B3GNT2_B3GN2
B3GNT2 chr11 47370397 0.008321 3.025e-10 35377 TRUE reported
textfile 2 1 TRUE
2984 rs11083616 A G A G -0.2532 0.0102 0.37363 NA FALSE FALSE FALSE
GCST006061 0.0073 0.1623 537409 outcome TRUE reported 7980_72_B3GNT2_B3GN2
B3GNT2 chr19 41359738 0.008368 1e-200 35372 TRUE reported textfile
2 1 TRUE
2985 rs11244083 A G A G 0.0694 -0.0039 0.24597 NA FALSE FALSE FALSE
GCST006061 0.0085 0.6435 537409 outcome TRUE reported 7980_72_B3GNT2_B3GN2
B3GNT2 chr9 133324112 0.009352 1.164e-13 35371 TRUE reported
textfile 2 1 TRUE
2986 rs281377 T C T C 0.1243 -0.0091 0.3353 NA FALSE FALSE FALSE
GCST006061 0.0075 0.2214 537409 outcome TRUE reported 7980_72_B3GNT2_B3GN2
B3GNT2 chr19 48703346 0.008781 1.709e-45 35377 TRUE reported
textfile 2 1 TRUE
2987 rs34012279 T G T G -0.1149 -0.0057 0.24621 NA FALSE FALSE FALSE
GCST006061 0.0087 0.5126 537409 outcome TRUE reported 7980_72_B3GNT2_B3GN2
B3GNT2 chr2 62103461 0.009377 1.607e-34 35353 TRUE reported
textfile 2 1 TRUE
2988 rs4073090 G A G A 0.1595 -7.00E-04 0.44746 NA FALSE FALSE FALSE
GCST006061 0.0072 0.922 537409 outcome TRUE reported 7980_72_B3GNT2_B3GN2
B3GNT2 chr2 62336639 0.008093 1.848e-86 35352 TRUE reported
textfile 2 1 TRUE
2989 rs4803517 G A G A 0.0955 0.0155 0.13446 NA FALSE FALSE FALSE
GCST006061 0.0116 0.1838 537409 outcome TRUE reported 7980_72_B3GNT2_B3GN2
B3GNT2 chr19 41972201 0.011946 1.301e-15 35370 TRUE reported
textfile 2 1 TRUE
2990 rs55997514 A G A G 0.0734 -0.0056 0.36307 NA FALSE FALSE FALSE
GCST006061 0.0078 0.4693 537409 outcome TRUE reported 7980_72_B3GNT2_B3GN2
B3GNT2 chr19 41725497 0.008411 2.633e-18 35375 TRUE reported
textfile 2 1 TRUE
2991 rs635634 C T C T -0.2534 0.0285 0.12995 NA FALSE FALSE FALSE
GCST006061 0.0089 0.001351 537409 outcome TRUE reported
7980_72_B3GNT2_B3GN2 B3GNT2 chr9 133279427 0.011948 7.841e-100 35371
TRUE reported textfile 2 1 TRUE
2992 rs9862378 T G T G -0.0465 -0.0332 0.49422 NA FALSE FALSE FALSE
GCST006061 0.0072 4.254e-06 537409 outcome TRUE reported
7980_72_B3GNT2_B3GN2 B3GNT2 chr3 58338667 0.008092 9.101e-09 35357
TRUE reported textfile 2 1 TRUE
2993 rs10437655 A G A G -0.0496 0.0098 0.37172 NA FALSE FALSE FALSE

GCST006061 0.0075 0.1881 537409 outcome TRUE reported 13748_4_CCL8_MCP_2
CCL8 chr11 47370397 0.007955 4.526e-10 35377 TRUE reported
textfile 2 1 TRUE
2994 rs10512462 G A G A -0.563 0.0034 0.07829 NA FALSE FALSE FALSE
GCST006061 0.0125 0.7877 537409 outcome TRUE reported 13748_4_CCL8_MCP_2
CCL8 chr17 34172040 0.015534 1e-200 35372 TRUE reported textfile
2 1 TRUE
2995 rs10853162 G A G A -0.3335 0.0144 0.32396 NA FALSE FALSE FALSE
GCST006061 0.0077 0.05992 537409 outcome TRUE reported 13748_4_CCL8_MCP_2
CCL8 chr17 34303211 0.00629 1e-200 35373 TRUE reported textfile 2
1 TRUE
2996 rs12075 A G A G -0.0844 5e-04 0.42429 NA FALSE FALSE FALSE GCST006061
0.0074 0.9502 537409 outcome TRUE reported 13748_4_CCL8_MCP_2 CCL8 chr1
159205564 0.007794 2.503e-27 35358 TRUE reported textfile 2 1 TRUE
2997 rs12451648 A G A G -0.2181 -0.0109 0.02255 NA FALSE FALSE FALSE
GCST006061 0.0213 0.6105 537409 outcome TRUE reported 13748_4_CCL8_MCP_2
CCL8 chr17 34646904 0.027865 4.999e-15 35364 TRUE reported
textfile 2 1 TRUE
2998 rs12943905 T C T C -0.1288 -0.0314 0.03491 NA FALSE FALSE FALSE
GCST006061 0.0239 0.1886 537409 outcome TRUE reported 13748_4_CCL8_MCP_2
CCL8 chr17 33476934 0.022643 1.284e-08 35374 TRUE reported
textfile 2 1 TRUE
2999 rs145626091 T C T C -0.1794 -0.0303 0.03126 NA FALSE FALSE FALSE
GCST006061 0.0243 0.2122 537409 outcome TRUE reported 13748_4_CCL8_MCP_2
CCL8 chr17 36789225 0.024385 1.883e-13 35380 TRUE reported
textfile 2 1 TRUE
3000 rs149062779 A G A G -0.16 -0.0063 0.02221 NA FALSE FALSE FALSE
GCST006061 0.0379 0.8673 537409 outcome TRUE reported 13748_4_CCL8_MCP_2
CCL8 chr17 21017228 0.028603 2.222e-08 35363 TRUE reported
textfile 2 1 TRUE
3001 rs2215230 A G A G -0.1666 -0.0066 0.25834 NA FALSE FALSE FALSE
GCST006061 0.0081 0.4203 537409 outcome TRUE reported 13748_4_CCL8_MCP_2
CCL8 chr17 34509093 0.009543 3.04e-68 35366 TRUE reported
textfile 2 1 TRUE
3002 rs3135980 C T C T -0.1243 0.0044 0.22264 NA FALSE FALSE FALSE
GCST006061 0.009 0.6301 537409 outcome TRUE reported 13748_4_CCL8_MCP_2
CCL8 chr17 34990777 0.010113 1.015e-34 35371 TRUE reported
textfile 2 1 TRUE
3003 rs319778 G A G A 0.0753 0.0021 0.38334 NA FALSE FALSE FALSE
GCST006061 0.0076 0.7845 537409 outcome TRUE reported 13748_4_CCL8_MCP_2
CCL8 chr17 33170507 0.008593 1.91e-18 35374 TRUE reported
textfile 2 1 TRUE
3004 rs73988096 T C T C -0.2321 -0.014 0.01227 NA FALSE FALSE FALSE
GCST006061 0.0268 0.6011 537409 outcome TRUE reported 13748_4_CCL8_MCP_2
CCL8 chr17 31540249 0.03746 5.795e-10 35362 TRUE reported textfile
2 1 TRUE
3005 rs79051484 T C T C -0.3433 -0.0131 0.0184 NA FALSE FALSE FALSE
GCST006061 0.0251 0.6011 537409 outcome TRUE reported 13748_4_CCL8_MCP_2
CCL8 chr17 35435201 0.031473 1.058e-27 35367 TRUE reported
textfile 2 1 TRUE
3006 rs1043784 C T C T -0.1949 -0.0102 0.10918 NA FALSE FALSE FALSE
GCST006061 0.0112 0.3642 537409 outcome TRUE reported 11212_7_TXNDC5_TXND5
TXNDC5 chr6 7881698 0.013075 3.001e-50 35364 TRUE reported textfile
2 1 TRUE
3007 rs112483799 G A G A 0.223 -0.0163 0.02332 NA FALSE FALSE FALSE
GCST006061 0.0265 0.5389 537409 outcome TRUE reported 11212_7_TXNDC5_TXND5
TXNDC5 chr6 8941812 0.027034 1.601e-16 35366 TRUE reported textfile
2 1 TRUE
3008 rs13209436 T C T C 0.2466 -0.0425 0.0146 NA FALSE FALSE FALSE
GCST006061 0.0423 0.3143 537409 outcome TRUE reported 11212_7_TXNDC5_TXND5
TXNDC5 chr6 8133259 0.033498 1.816e-13 35365 TRUE reported textfile
2 1 TRUE
3009 rs56238200 A G A G 0.1296 0.0083 0.35087 NA FALSE FALSE FALSE
GCST006061 0.008 0.2985 537409 outcome TRUE reported 11212_7_TXNDC5_TXND5
TXNDC5 chr17 82398802 0.008598 2.453e-51 35009 TRUE reported
textfile 2 1 TRUE

3010 rs6796 C T C T 0.0557 0.0015 0.23682 NA FALSE FALSE FALSE GCST006061 0.0081 0.857 537409 outcome TRUE reported 11212_7_TXNDC5_TXND5 TXNDC5 chr7 6462736 0.009576 6.013e-09 35355 TRUE reported textfile 2 1 TRUE
3011 rs10437954 A G A G -0.1808 0.0107 0.11558 NA FALSE FALSE FALSE GCST006061 0.0124 0.3889 537409 outcome TRUE reported 13519_112_ARHGEF25_ARHGP ARHGEF25 chr12 57610139 0.012343 1.394e-48 35360 TRUE reported textfile 2 1 TRUE
3012 rs10922098 T C T C -0.0925 -0.009 0.40011 NA FALSE FALSE FALSE GCST006061 0.0075 0.2275 537409 outcome TRUE reported 13519_112_ARHGEF25_ARHGP ARHGEF25 chr1 196695521 0.008065 1.888e-30 35332 TRUE reported textfile 2 1 TRUE
3013 rs1706435 G A G A 0.052 0.0117 0.36981 NA FALSE FALSE FALSE GCST006061 0.0072 0.1047 537409 outcome TRUE reported 13519_112_ARHGEF25_ARHGP ARHGEF25 chr3 165743688 0.008176 2.019e-10 35333 TRUE reported textfile 2 1 TRUE
3014 rs4237845 T C T C -0.0469 0.0023 0.49536 NA FALSE FALSE FALSE GCST006061 0.0073 0.7562 537409 outcome TRUE reported 13519_112_ARHGEF25_ARHGP ARHGEF25 chr12 57908653 0.007905 2.972e-09 35360 TRUE reported textfile 2 1 TRUE
3015 rs1043836 T C T C 0.1773 0.0084 0.37038 NA FALSE FALSE FALSE GCST006061 0.0074 0.2561 537409 outcome TRUE reported 19482_11_HDHD3_HDHD3 HDHD3 chr9 113373918 0.008364 9.843e-100 35362 TRUE reported textfile 2 1 TRUE
3016 rs1355538 G A G A -0.0597 -0.0076 0.39847 NA FALSE FALSE FALSE GCST006061 0.0072 0.2923 537409 outcome TRUE reported 19482_11_HDHD3_HDHD3 HDHD3 chr3 165787389 0.008189 3.091e-13 35338 TRUE reported textfile 2 1 TRUE
3017 rs203688 A G A G -0.0783 -0.012 0.39961 NA FALSE FALSE FALSE GCST006061 0.0075 0.1106 537409 outcome TRUE reported 19482_11_HDHD3_HDHD3 HDHD3 chr1 196703255 0.00819 1.166e-21 35339 TRUE reported textfile 2 1 TRUE
3018 rs1043897 T G T G -0.0809 0.0069 0.43439 NA FALSE FALSE FALSE GCST006061 0.0073 0.3487 537409 outcome TRUE reported 5242_37_MAP2K4_MP2K4 MAP2K4 chr1 230280653 0.008171 4.104e-23 35360 TRUE reported textfile 2 1 TRUE
3019 rs111611525 C T C T 0.2614 -0.0465 0.01532 NA FALSE FALSE FALSE GCST006061 0.0305 0.1271 537409 outcome TRUE reported 5242_37_MAP2K4_MP2K4 MAP2K4 chr3 184576552 0.036104 4.478e-13 35357 TRUE reported textfile 2 1 TRUE
3020 rs1348638 A G A G 0.0571 -0.0059 0.29339 NA FALSE FALSE FALSE GCST006061 0.0084 0.4798 537409 outcome TRUE reported 5242_37_MAP2K4_MP2K4 MAP2K4 chr3 187971204 0.009602 2.738e-09 35371 TRUE reported textfile 2 1 TRUE
3021 rs144052318 A G A G 0.2144 -0.0219 0.01279 NA FALSE FALSE FALSE GCST006061 0.0327 0.5038 537409 outcome TRUE reported 5242_37_MAP2K4_MP2K4 MAP2K4 chr1 229567230 0.035233 1.163e-09 35361 TRUE reported textfile 2 1 TRUE
3022 rs1574648 C T C T 0.2435 -9.00E-04 0.02426 NA FALSE FALSE FALSE GCST006061 0.0372 0.9797 537409 outcome TRUE reported 5242_37_MAP2K4_MP2K4 MAP2K4 chr3 187458809 0.028279 7.269e-18 35367 TRUE reported textfile 2 1 TRUE
3023 rs2889825 G A G A -0.1413 -0.0174 0.1238 NA FALSE FALSE FALSE GCST006061 0.0126 0.1683 537409 outcome TRUE reported 5242_37_MAP2K4_MP2K4 MAP2K4 chr3 186875332 0.013212 1.075e-26 35362 TRUE reported textfile 2 1 TRUE
3024 rs4631704 T C T C -0.2777 -0.0038 0.39231 NA FALSE FALSE FALSE GCST006061 0.0075 0.6161 537409 outcome TRUE reported 5242_37_MAP2K4_MP2K4 MAP2K4 chr1 230157783 0.008159 1e-200 35358 TRUE reported textfile 2 1 TRUE
3025 rs5030073 G A G A -0.235 -0.0019 0.43758 NA FALSE FALSE FALSE GCST006061 0.0072 0.7954 537409 outcome TRUE reported 5242_37_MAP2K4_MP2K4 MAP2K4 chr3 186737999 0.008792 2.206e-157 35367 TRUE reported textfile 2 1 TRUE
3026 rs66965282 G A G A 0.9445 -0.0046 0.11299 NA FALSE FALSE FALSE GCST006061 0.0102 0.6533 537409 outcome TRUE reported 5242_37_MAP2K4_MP2K4

MAP2K4 chr3 186640979 0.008874 1e-200 35367 TRUE reported textfile
2 1 TRUE
3027 rs75129524 A G A G -0.1402 0.0094 0.03197 NA FALSE FALSE FALSE
GCST006061 0.0374 0.8014 537409 outcome TRUE reported 5242_37_MAP2K4_MP2K4
MAP2K4 chr3 183698592 0.024545 1.117e-08 35358 TRUE reported
textfile 2 1 TRUE
3028 rs8179931 C T C T 0.2544 0.0122 0.14982 NA FALSE FALSE FALSE
GCST006061 0.0111 0.2733 537409 outcome TRUE reported 5242_37_MAP2K4_MP2K4
MAP2K4 chr3 186598181 0.012191 1.037e-96 35367 TRUE reported
textfile 2 1 TRUE
3029 rs907866 A G A G 0.0794 -0.0029 0.49419 NA FALSE FALSE FALSE
GCST006061 0.0072 0.6861 537409 outcome TRUE reported 5242_37_MAP2K4_MP2K4
MAP2K4 chr2 20171619 0.007981 2.558e-23 35372 TRUE reported
textfile 2 1 TRUE
3030 rs1043897 T G T G 0.0592 0.0069 0.43439 NA FALSE FALSE FALSE
GCST006061 0.0073 0.3487 537409 outcome TRUE reported 5542_22_NRP1_NRP1
NRP1 chr1 230280653 0.008307 1.03e-12 35360 TRUE reported
textfile 2 1 TRUE
3031 rs10864726 T C T C 0.1728 -0.0051 0.39025 NA FALSE FALSE FALSE
GCST006061 0.0074 0.4873 537409 outcome TRUE reported 5542_22_NRP1_NRP1
NRP1 chr1 230160406 0.008388 2.64e-94 35358 TRUE reported
textfile 2 1 TRUE
3032 rs1260326 C T C T 0.0717 -0.0069 0.34132 NA FALSE FALSE FALSE
GCST006061 0.0073 0.3401 537409 outcome TRUE reported 5542_22_NRP1_NRP1
NRP1 chr2 27508073 0.008672 1.36e-16 35373 TRUE reported
textfile 2 1 TRUE
3033 rs142268798 C T C T 0.1615 0.0019 0.03947 NA FALSE FALSE FALSE
GCST006061 0.0214 0.9277 537409 outcome TRUE reported 5542_22_NRP1_NRP1
NRP1 chr10 32920901 0.021043 1.659e-14 35357 TRUE reported
textfile 2 1 TRUE
3034 rs143501783 C T C T 0.2447 -0.0333 0.01116 NA FALSE FALSE FALSE
GCST006061 0.0443 0.4525 537409 outcome TRUE reported 5542_22_NRP1_NRP1
NRP1 chr17 7897001 0.037222 4.894e-11 35373 TRUE reported textfile
2 1 TRUE
3035 rs146107972 G A G A 0.1501 -0.0521 0.02634 NA FALSE FALSE FALSE
GCST006061 0.0345 0.1304 537409 outcome TRUE reported 5542_22_NRP1_NRP1
NRP1 chr10 31157228 0.025631 4.739e-09 35356 TRUE reported
textfile 2 1 TRUE
3036 rs148255421 A G A G 0.2424 -0.0037 0.01372 NA FALSE FALSE FALSE
GCST006061 0.033 0.9119 537409 outcome TRUE reported 5542_22_NRP1_NRP1
NRP1 chr10 44055149 0.035632 1.025e-11 35323 TRUE reported
textfile 2 1 TRUE
3037 rs187621637 T C T C -0.1649 -0.0011 0.02394 NA FALSE FALSE FALSE
GCST006061 0.0385 0.9767 537409 outcome TRUE reported 5542_22_NRP1_NRP1
NRP1 chr10 31362314 0.026972 9.734e-10 35355 TRUE reported
textfile 2 1 TRUE
3038 rs189444333 G A G A 0.2793 -0.0089 0.01646 NA FALSE FALSE FALSE
GCST006061 0.0321 0.7818 537409 outcome TRUE reported 5542_22_NRP1_NRP1
NRP1 chr10 33486023 0.032489 8.195e-18 35354 TRUE reported
textfile 2 1 TRUE
3039 rs2383984 A G A G -0.3068 0.0052 0.23984 NA FALSE FALSE FALSE
GCST006061 0.008 0.5195 537409 outcome TRUE reported 5542_22_NRP1_NRP1
NRP1 chr10 33187213 0.009533 1e-200 35359 TRUE reported textfile
2 1 TRUE
3040 rs3006692 T C T C -0.0527 -0.0135 0.38963 NA FALSE FALSE FALSE
GCST006061 0.0073 0.06514 537409 outcome TRUE reported 5542_22_NRP1_NRP1
NRP1 chr10 32180307 0.008464 4.769e-10 35356 TRUE reported
textfile 2 1 TRUE
3041 rs41556717 T C T C 0.1611 -0.0483 0.01931 NA FALSE FALSE FALSE
GCST006061 0.0325 0.1371 537409 outcome TRUE reported 5542_22_NRP1_NRP1
NRP1 chr17 7496000 0.028647 1.869e-08 35374 TRUE reported textfile
2 1 TRUE
3042 rs7555577 C A C A -0.0893 0.0233 0.33298 NA FALSE FALSE FALSE
GCST006061 0.0076 0.002023 537409 outcome TRUE reported 5542_22_NRP1_NRP1
NRP1 chr1 62568692 0.008697 9.875e-25 35358 TRUE reported
textfile 2 1 TRUE

3043 rs77542162 G A G A 0.1797 0.0177 0.025 NA FALSE FALSE FALSE
GCST006061 0.0302 0.557 537409 outcome TRUE reported 5542_22_NRP1_NRP1
NRP1 chr17 69085137 0.025807 3.324e-12 35371 TRUE reported
textfile 2 1 TRUE
3044 rs10439301 C T C T -0.0541 -0.0109 0.22825 NA FALSE FALSE FALSE
GCST006061 0.0082 0.1859 537409 outcome TRUE reported
15324_58_FTL_Ferritin_light_chain FTL chr2 180395899 0.009775 3.119e-08
35354 TRUE reported textfile 2 1 TRUE
3045 rs115740542 C T C T 0.1043 -0.039 0.06666 NA FALSE FALSE FALSE
GCST006061 0.0158 0.0139 537409 outcome TRUE reported
15324_58_FTL_Ferritin_light_chain FTL chr6 26123274 0.016516 2.702e-10
35376 TRUE reported textfile 2 1 TRUE
3046 rs12693541 T C T C -0.0776 -0.0128 0.11028 NA FALSE FALSE FALSE
GCST006061 0.0106 0.2285 537409 outcome TRUE reported
15324_58_FTL_Ferritin_light_chain FTL chr2 189553964 0.013137 3.485e-09
35367 TRUE reported textfile 2 1 TRUE
3047 rs199138 G A G A 0.1213 -0.0054 0.08139 NA FALSE FALSE FALSE
GCST006061 0.0139 0.6955 537409 outcome TRUE reported
15324_58_FTL_Ferritin_light_chain FTL chr15 45095352 0.01486 3.271e-16 35362
TRUE reported textfile 2 1 TRUE
3048 rs8263 T C T C -0.0506 -0.0196 0.4894 NA FALSE FALSE FALSE GCST006061
0.0072 0.006238 537409 outcome TRUE reported
15324_58_FTL_Ferritin_light_chain FTL chr17 58345753 0.008207 7.033e-10
35368 TRUE reported textfile 2 1 TRUE
3049 rs12403568 T G T G -0.0447 0.0056 0.47071 NA FALSE FALSE FALSE
GCST006061 0.0071 0.4334 537409 outcome TRUE reported 13993_20_EPB41L1_E41L1
EPB41L1 chr1 29131266 0.007902 1.542e-08 35370 TRUE reported
textfile 2 1 TRUE
3050 rs204074 C T C T -0.0707 -0.0196 0.11346 NA FALSE FALSE FALSE
GCST006061 0.013 0.1321 537409 outcome TRUE reported 13993_20_EPB41L1_E41L1
EPB41L1 chr1 28865051 0.012463 1.405e-08 35368 TRUE reported
textfile 2 1 TRUE
3051 rs3896594 G T G T -0.0444 0.0107 0.38501 NA FALSE FALSE FALSE
GCST006061 0.0074 0.148 537409 outcome TRUE reported 13993_20_EPB41L1_E41L1
EPB41L1 chr2 8622284 0.008104 4.275e-08 35355 TRUE reported textfile
2 1 TRUE
3052 rs12403568 T G T G -0.0515 0.0056 0.47071 NA FALSE FALSE FALSE
GCST006061 0.0071 0.4334 537409 outcome TRUE reported 4706_17_EPB41_41
EPB41 chr1 29131266 0.007955 9.555e-11 35370 TRUE reported
textfile 2 1 TRUE
3053 rs204074 C T C T -0.098 -0.0196 0.11346 NA FALSE FALSE FALSE
GCST006061 0.013 0.1321 537409 outcome TRUE reported 4706_17_EPB41_41
EPB41 chr1 28865051 0.012543 5.566e-15 35368 TRUE reported
textfile 2 1 TRUE
3054 rs10447396 A G A G 0.1156 0.0173 0.24445 NA FALSE FALSE FALSE
GCST006061 0.0082 0.0349 537409 outcome TRUE reported 5006_71_MAPK13_MK13
MAPK13 chr6 36145821 0.009245 7.041e-36 35356 TRUE reported
textfile 2 1 TRUE
3055 rs203688 A G A G -0.1052 -0.012 0.39961 NA FALSE FALSE FALSE
GCST006061 0.0075 0.1106 537409 outcome TRUE reported 5006_71_MAPK13_MK13
MAPK13 chr1 196703255 0.008123 2.333e-38 35339 TRUE reported
textfile 2 1 TRUE
3056 rs10454088 A G A G -0.2126 -0.0107 0.0732 NA FALSE FALSE FALSE
GCST006061 0.0146 0.464 537409 outcome TRUE reported 12817_1_GEM_GEM GEM
chr17 28423862 0.016058 5.206e-40 35351 TRUE reported textfile 2
1 TRUE
3057 rs12478841 G A G A 0.0567 0.0049 0.21569 NA FALSE FALSE FALSE
GCST006061 0.008 0.5413 537409 outcome TRUE reported 12817_1_GEM_GEM GEM
chr2 27588855 0.009548 2.88e-09 35364 TRUE reported textfile 2
1 TRUE
3058 rs140390732 A C A C -0.3185 0.0526 0.01316 NA FALSE FALSE FALSE
GCST006061 0.0456 0.2487 537409 outcome TRUE reported 12817_1_GEM_GEM GEM
chr17 27292324 0.036535 2.838e-18 35352 TRUE reported textfile 2
1 TRUE
3059 rs140870715 A G A G 0.2614 0.0178 0.01034 NA FALSE FALSE FALSE
GCST006061 0.0429 0.6791 537409 outcome TRUE reported 12817_1_GEM_GEM GEM

chr11 6217450 0.03953 3.775e-11 35370 TRUE reported textfile 2 1 TRUE

3060 rs140915766 T C T C -0.2516 -0.0337 0.01498 NA FALSE FALSE FALSE GCST006061 0.052 0.5169 537409 outcome TRUE reported 12817_1_GEM_GEM GEM chr17 32465402 0.034099 1.6e-13 35356 TRUE reported textfile 2 1 TRUE

3061 rs147092838 C T C T 0.1832 -0.01 0.01969 NA FALSE FALSE FALSE GCST006061 0.0363 0.7816 537409 outcome TRUE reported 12817_1_GEM_GEM GEM chr11 5449749 0.028641 1.591e-10 35370 TRUE reported textfile 2 1 TRUE

3062 rs147804221 T C T C -0.1548 -0.0204 0.02403 NA FALSE FALSE FALSE GCST006061 0.0321 0.5263 537409 outcome TRUE reported 12817_1_GEM_GEM GEM chr17 15132253 0.026921 8.916e-09 35346 TRUE reported textfile 2 1 TRUE

3063 rs182655273 A G A G 0.312 -0.0186 0.01498 NA FALSE FALSE FALSE GCST006061 0.0311 0.5488 537409 outcome TRUE reported 12817_1_GEM_GEM GEM chr17 27613426 0.032458 7.079e-22 35350 TRUE reported textfile 2 1 TRUE

3064 rs28999383 A G A G 0.2373 -0.0064 0.01233 NA FALSE FALSE FALSE GCST006061 0.0233 0.7841 537409 outcome TRUE reported 12817_1_GEM_GEM GEM chr17 27777803 0.03735 2.106e-10 35349 TRUE reported textfile 2 1 TRUE

3065 rs76608048 C T C T -0.1882 -0.0422 0.02479 NA FALSE FALSE FALSE GCST006061 0.0429 0.3251 537409 outcome TRUE reported 12817_1_GEM_GEM GEM chr17 31485186 0.027241 4.892e-12 35353 TRUE reported textfile 2 1 TRUE

3066 rs9899944 G A G A -0.1576 -0.0122 0.06039 NA FALSE FALSE FALSE GCST006061 0.0142 0.388 537409 outcome TRUE reported 12817_1_GEM_GEM GEM chr17 16585276 0.017498 2.126e-19 35346 TRUE reported textfile 2 1 TRUE

3067 rs9902833 G A G A 0.2813 -0.013 0.38604 NA FALSE FALSE FALSE GCST006061 0.0074 0.07835 537409 outcome TRUE reported 12817_1_GEM_GEM GEM chr17 28071104 0.008475 1e-200 35351 TRUE reported textfile 2 1 TRUE

3068 rs1045553 A G A G -0.4102 -0.0032 0.22794 NA FALSE FALSE FALSE GCST006061 0.0086 0.7153 537409 outcome TRUE reported 6364_7_TAPBPL_TPSNR TAPBPL chr12 6638129 0.005513 1e-200 35387 TRUE reported textfile 2 1 TRUE

3069 rs10849454 C T C T -0.2997 0.0027 0.33901 NA FALSE FALSE FALSE GCST006061 0.0084 0.7496 537409 outcome TRUE reported 6364_7_TAPBPL_TPSNR TAPBPL chr12 6425488 0.008932 1e-200 35381 TRUE reported textfile 2 1 TRUE

3070 rs11063208 T C T C -0.1937 0.0124 0.04082 NA FALSE FALSE FALSE GCST006061 0.0241 0.6076 537409 outcome TRUE reported 6364_7_TAPBPL_TPSNR TAPBPL chr12 4499159 0.021874 8.339e-19 35381 TRUE reported textfile 2 1 TRUE

3071 rs116455037 A G A G -0.0943 0.0244 0.03718 NA FALSE FALSE FALSE GCST006061 0.0246 0.3206 537409 outcome TRUE reported 6364_7_TAPBPL_TPSNR TAPBPL chr6 25764433 0.015904 3.045e-09 35376 TRUE reported textfile 2 1 TRUE

3072 rs117244485 C T C T -0.4774 -0.067 0.02438 NA FALSE FALSE FALSE GCST006061 0.0419 0.1098 537409 outcome TRUE reported 6364_7_TAPBPL_TPSNR TAPBPL chr12 6686246 0.026953 3.368e-70 35388 TRUE reported textfile 2 1 TRUE

3073 rs117443665 A G A G -0.1271 0.0327 0.03543 NA FALSE FALSE FALSE GCST006061 0.0422 0.4387 537409 outcome TRUE reported 6364_7_TAPBPL_TPSNR TAPBPL chr12 5686769 0.023136 3.938e-08 35383 TRUE reported textfile 2 1 TRUE

3074 rs12370543 G T G T 0.1159 -0.0176 0.1015 NA FALSE FALSE FALSE GCST006061 0.0113 0.1188 537409 outcome TRUE reported 6364_7_TAPBPL_TPSNR TAPBPL chr12 7107564 0.014285 4.93e-16 35391 TRUE reported textfile 2 1 TRUE

3075 rs146840578 C A C A 0.2101 0.0247 0.02258 NA FALSE FALSE FALSE GCST006061 0.0481 0.6081 537409 outcome TRUE reported 6364_7_TAPBPL_TPSNR TAPBPL chr12 7670828 0.027658 3.049e-14 35388 TRUE reported textfile 2 1 TRUE

3076 rs182944391 A G A G 0.3456 -0.0436 0.02592 NA FALSE FALSE FALSE

GCST006061 0.0366 0.2343 537409 outcome TRUE reported 6364_7_TAPBPL_TPSNR TAPBPL chr12 6098561 0.027224 6.318e-37 35381 TRUE reported textfile 2 1 TRUE
3077 rs504963 A G A G -0.0345 0.0056 0.37239 NA FALSE FALSE FALSE GCST006061 0.0075 0.4571 537409 outcome TRUE reported 6364_7_TAPBPL_TPSNR TAPBPL chr19 48705608 0.006117 1.701e-08 35377 TRUE reported textfile 2 1 TRUE
3078 rs56983450 A G A G 0.7165 -0.0027 0.03437 NA FALSE FALSE FALSE GCST006061 0.022 0.9013 537409 outcome TRUE reported 6364_7_TAPBPL_TPSNR TAPBPL chr12 6382463 0.023536 1e-200 35375 TRUE reported textfile 2 1 TRUE
3079 rs6488340 A G A G 0.2294 -0.0066 0.09274 NA FALSE FALSE FALSE GCST006061 0.0117 0.5752 537409 outcome TRUE reported 6364_7_TAPBPL_TPSNR TAPBPL chr12 7483197 0.014709 7.772e-55 35388 TRUE reported textfile 2 1 TRUE
3080 rs10457139 G A G A -0.1368 -0.0089 0.08932 NA FALSE FALSE FALSE GCST006061 0.0137 0.5162 537409 outcome TRUE reported 15304_1_REG3A_PAP1 REG3A chr6 105953789 0.01467 1.111e-20 35369 TRUE reported textfile 2 1 TRUE
3081 rs10853981 A G A G 0.0491 -0.0184 0.37039 NA FALSE FALSE FALSE GCST006061 0.0079 0.02035 537409 outcome TRUE reported 15304_1_REG3A_PAP1 REG3A chr19 4965053 0.008669 1.48e-08 35360 TRUE reported textfile 2 1 TRUE
3082 rs112787300 T C T C 0.1884 0.0226 0.06733 NA FALSE FALSE FALSE GCST006061 0.0144 0.1174 537409 outcome TRUE reported 15304_1_REG3A_PAP1 REG3A chr2 79213567 0.017199 6.344e-28 35353 TRUE reported textfile 2 1 TRUE
3083 rs12368026 C A C A 0.06 -0.0022 0.39093 NA FALSE FALSE FALSE GCST006061 0.0078 0.7775 537409 outcome TRUE reported 15304_1_REG3A_PAP1 REG3A chr12 31956517 0.008613 3.248e-12 35358 TRUE reported textfile 2 1 TRUE
3084 rs13215052 A G A G 0.1189 0.0115 0.11188 NA FALSE FALSE FALSE GCST006061 0.013 0.3766 537409 outcome TRUE reported 15304_1_REG3A_PAP1 REG3A chr6 41714097 0.013452 9.677e-19 35366 TRUE reported textfile 2 1 TRUE
3085 rs13253974 A G A G -0.0643 0.0016 0.33974 NA FALSE FALSE FALSE GCST006061 0.0076 0.8331 537409 outcome TRUE reported 15304_1_REG3A_PAP1 REG3A chr8 23520397 0.00886 3.955e-13 35369 TRUE reported textfile 2 1 TRUE
3086 rs17338413 T C T C -0.3323 -0.0045 0.06125 NA FALSE FALSE FALSE GCST006061 0.0147 0.7608 537409 outcome TRUE reported 15304_1_REG3A_PAP1 REG3A chr15 75182595 0.017192 3.061e-83 35359 TRUE reported textfile 2 1 TRUE
3087 rs17616434 C T C T 0.0528 -0.0064 0.30802 NA FALSE FALSE FALSE GCST006061 0.0084 0.4467 537409 outcome TRUE reported 15304_1_REG3A_PAP1 REG3A chr4 38811255 0.009104 6.646e-09 35365 TRUE reported textfile 2 1 TRUE
3088 rs191783020 C A C A -0.3935 -0.0665 0.01119 NA FALSE FALSE FALSE GCST006061 0.0634 0.2946 537409 outcome TRUE reported 15304_1_REG3A_PAP1 REG3A chr2 79247096 0.040205 1.275e-22 35354 TRUE reported textfile 2 1 TRUE
3089 rs3217810 T C T C -0.0683 -0.0105 0.13867 NA FALSE FALSE FALSE GCST006061 0.0123 0.3945 537409 outcome TRUE reported 15304_1_REG3A_PAP1 REG3A chr12 4279105 0.012099 1.65e-08 35376 TRUE reported textfile 2 1 TRUE
3090 rs4459759 A G A G 0.4073 0.0101 0.04659 NA FALSE FALSE FALSE GCST006061 0.0166 0.5417 537409 outcome TRUE reported 15304_1_REG3A_PAP1 REG3A chr2 79170240 0.019734 1.223e-94 35353 TRUE reported textfile 2 1 TRUE
3091 rs673854 A G A G -0.0525 -0.0028 0.4614 NA FALSE FALSE FALSE GCST006061 0.0071 0.6969 537409 outcome TRUE reported 15304_1_REG3A_PAP1 REG3A chr8 108179609 0.008415 4.41e-10 35343 TRUE reported textfile 2 1 TRUE
3092 rs72913277 G A G A -0.2895 -0.0338 0.04288 NA FALSE FALSE FALSE GCST006061 0.0186 0.0681 537409 outcome TRUE reported 15304_1_REG3A_PAP1 REG3A chr2 79140251 0.021209 2.018e-42 35350 TRUE reported

textfile 2 1 TRUE
3093 rs8133137 G A G A -0.0555 -2.00E-04 0.38462 NA FALSE FALSE FALSE
GCST006061 0.0076 0.9762 537409 outcome TRUE reported 15304_1_REG3A_PAP1
REG3A chr21 38918579 0.008596 1.071e-10 35376 TRUE reported
textfile 2 1 TRUE
3094 rs11885419 G A G A 0.0718 6e-04 0.14876 NA FALSE FALSE FALSE
GCST006061 0.0102 0.9497 537409 outcome TRUE reported 3858_5_ACP1_PPAC
ACP1 chr2 1888158 0.011424 3.28e-10 35304 TRUE reported textfile
2 1 TRUE
3095 rs11895897 T G T G -0.1433 -0.0151 0.29118 NA FALSE FALSE FALSE
GCST006061 0.008 0.05745 537409 outcome TRUE reported 3858_5_ACP1_PPAC
ACP1 chr2 962386 0.008874 1.175e-58 35304 TRUE reported textfile
2 1 TRUE
3096 rs17042314 C T C T -0.1939 0.006 0.13301 NA FALSE FALSE FALSE
GCST006061 0.0114 0.5987 537409 outcome TRUE reported 3858_5_ACP1_PPAC
ACP1 chr2 661188 0.011932 2.205e-59 35302 TRUE reported textfile
2 1 TRUE
3097 rs2685226 G A G A -0.1174 -0.0025 0.19491 NA FALSE FALSE FALSE
GCST006061 0.0093 0.7886 537409 outcome TRUE reported 3858_5_ACP1_PPAC
ACP1 chr2 561786 0.010182 9.239e-31 35304 TRUE reported textfile
2 1 TRUE
3098 rs7576574 A G A G -0.1248 -0.0178 0.06587 NA FALSE FALSE FALSE
GCST006061 0.014 0.2052 537409 outcome TRUE reported 3858_5_ACP1_PPAC
ACP1 chr2 1210553 0.016184 1.245e-14 35304 TRUE reported textfile
2 1 TRUE
3099 rs79857466 C A C A 0.1928 -0.0087 0.05601 NA FALSE FALSE FALSE
GCST006061 0.0184 0.6374 537409 outcome TRUE reported 3858_5_ACP1_PPAC
ACP1 chr2 1489136 0.017451 2.247e-28 35305 TRUE reported textfile
2 1 TRUE
3100 rs9677202 A G A G 0.0822 0.0093 0.4119 NA FALSE FALSE FALSE
GCST006061 0.0074 0.2075 537409 outcome TRUE reported 3858_5_ACP1_PPAC
ACP1 chr2 1346435 0.008201 1.205e-23 35305 TRUE reported textfile
2 1 TRUE
3101 rs1354034 C T C T 0.1313 0.0088 0.38119 NA FALSE FALSE FALSE
GCST006061 0.0073 0.2248 537409 outcome TRUE reported 5095_21_KIR2DL4_KI2L4
KIR2DL4 chr3 56815721 0.008391 3.452e-55 35356 TRUE reported
textfile 2 1 TRUE
3102 rs203687 T C T C -0.0609 -0.0146 0.4315 NA FALSE FALSE FALSE
GCST006061 0.0075 0.05005 537409 outcome TRUE reported 5095_21_KIR2DL4_KI2L4
KIR2DL4 chr1 196705140 0.008241 1.465e-13 35339 TRUE reported
textfile 2 1 TRUE
3103 rs2298429 G A G A 0.0595 -0.0179 0.19776 NA FALSE FALSE FALSE
GCST006061 0.0088 0.04327 537409 outcome TRUE reported 5095_21_KIR2DL4_KI2L4
KIR2DL4 chr22 21628971 0.010316 8.025e-09 35367 TRUE reported
textfile 2 1 TRUE
3104 rs11053902 G T G T 0.1114 -0.0143 0.1035 NA FALSE FALSE FALSE
GCST006061 0.0129 0.2664 537409 outcome TRUE reported 9751_72_YBX1_NSEP1
YBX1 chr12 10692619 0.013219 3.533e-17 35391 TRUE reported
textfile 2 1 TRUE
3105 rs11082513 T C T C -0.049 0.0048 0.48528 NA FALSE FALSE FALSE
GCST006061 0.0075 0.5225 537409 outcome TRUE reported 9751_72_YBX1_NSEP1
YBX1 chr18 46167187 0.008119 1.588e-09 35350 TRUE reported
textfile 2 1 TRUE
3106 rs1354034 C T C T 0.158 0.0088 0.38119 NA FALSE FALSE FALSE
GCST006061 0.0073 0.2248 537409 outcome TRUE reported 9751_72_YBX1_NSEP1
YBX1 chr3 56815721 0.008281 3.709e-81 35356 TRUE reported
textfile 2 1 TRUE
3107 rs4820091 G T G T 0.0811 -0.0166 0.19742 NA FALSE FALSE FALSE
GCST006061 0.0088 0.06081 537409 outcome TRUE reported 9751_72_YBX1_NSEP1
YBX1 chr22 21585900 0.010157 1.412e-15 35367 TRUE reported
textfile 2 1 TRUE
3108 rs10467359 G A G A -0.3185 -0.0208 0.04031 NA FALSE FALSE FALSE
GCST006061 0.0138 0.1317 537409 outcome TRUE reported 15384_15_KL_KLOTHO KL
chr13 32955126 0.020808 6.897e-53 35364 TRUE reported textfile 2
1 TRUE
3109 rs10752124 C T C T -0.0688 6e-04 0.46214 NA FALSE FALSE FALSE

GCST006061 0.0072 0.9321 537409 outcome TRUE reported 15384_15_KL_KLOTHO KL chr10 8004957 0.008418 3.013e-16 35361 TRUE reported textfile 2 1 TRUE

3110 rs11605349 C T C T 0.1363 0.0065 0.08574 NA FALSE FALSE FALSE GCST006061 0.0124 0.6026 537409 outcome TRUE reported 15384_15_KL_KLOTHO KL chr11 126435860 0.014662 1.453e-20 35371 TRUE reported textfile 2 1 TRUE

3111 rs1169311 T C T C -0.0651 -0.009 0.32019 NA FALSE FALSE FALSE GCST006061 0.0074 0.2193 537409 outcome TRUE reported 15384_15_KL_KLOTHO KL chr12 121002928 0.00903 5.621e-13 35365 TRUE reported textfile 2 1 TRUE

3112 rs117241334 A C A C 0.2333 -0.095 0.01792 NA FALSE FALSE FALSE GCST006061 0.0503 0.05877 537409 outcome TRUE reported 15384_15_KL_KLOTHO KL chr19 48633205 0.031381 1.05e-13 35376 TRUE reported textfile 2 1 TRUE

3113 rs117633773 A G A G 0.1209 0.0067 0.04197 NA FALSE FALSE FALSE GCST006061 0.0224 0.7662 537409 outcome TRUE reported 15384_15_KL_KLOTHO KL chr17 6953365 0.020653 4.802e-09 35374 TRUE reported textfile 2 1 TRUE

3114 rs117877056 A C A C 0.1885 0.0106 0.02225 NA FALSE FALSE FALSE GCST006061 0.0381 0.7806 537409 outcome TRUE reported 15384_15_KL_KLOTHO KL chr19 48480239 0.028096 1.957e-11 35375 TRUE reported textfile 2 1 TRUE

3115 rs143501783 C T C T 0.2709 -0.0333 0.01116 NA FALSE FALSE FALSE GCST006061 0.0443 0.4525 537409 outcome TRUE reported 15384_15_KL_KLOTHO KL chr17 7897001 0.038094 1.149e-12 35373 TRUE reported textfile 2 1 TRUE

3116 rs162000 A C A C -0.0484 0.0064 0.43225 NA FALSE FALSE FALSE GCST006061 0.0075 0.3995 537409 outcome TRUE reported 15384_15_KL_KLOTHO KL chr18 26872471 0.008467 1.088e-08 35368 TRUE reported textfile 2 1 TRUE

3117 rs17855739 T C T C 0.1392 0.0098 0.03389 NA FALSE FALSE FALSE GCST006061 0.0173 0.5715 537409 outcome TRUE reported 15384_15_KL_KLOTHO KL chr19 5831829 0.022852 1.12e-09 35366 TRUE reported textfile 2 1 TRUE

3118 rs2823253 A C A C -0.0495 -6.00E-04 0.3457 NA FALSE FALSE FALSE GCST006061 0.0079 0.9406 537409 outcome TRUE reported 15384_15_KL_KLOTHO KL chr21 15408817 0.008861 2.32e-08 35280 TRUE reported textfile 2 1 TRUE

3119 rs2974937 T C T C 0.0531 0.0172 0.41796 NA FALSE FALSE FALSE GCST006061 0.0072 0.01741 537409 outcome TRUE reported 15384_15_KL_KLOTHO KL chr1 155199058 0.008521 4.614e-10 35360 TRUE reported textfile 2 1 TRUE

3120 rs36078145 A G A G 0.1887 0.001 0.0673 NA FALSE FALSE FALSE GCST006061 0.0157 0.9511 537409 outcome TRUE reported 15384_15_KL_KLOTHO KL chr12 561429 0.016845 3.975e-29 35309 TRUE reported textfile 2 1 TRUE

3121 rs4846806 T C T C -0.068 -0.0097 0.35612 NA FALSE FALSE FALSE GCST006061 0.0073 0.1851 537409 outcome TRUE reported 15384_15_KL_KLOTHO KL chr1 217033747 0.008746 7.551e-15 35356 TRUE reported textfile 2 1 TRUE

3122 rs55714927 T C T C 0.1123 1e-04 0.21166 NA FALSE FALSE FALSE GCST006061 0.0098 0.9926 537409 outcome TRUE reported 15384_15_KL_KLOTHO KL chr17 7176997 0.010318 1.377e-27 35370 TRUE reported textfile 2 1 TRUE

3123 rs66497154 C T C T -0.0608 -0.0037 0.26277 NA FALSE FALSE FALSE GCST006061 0.0083 0.6522 537409 outcome TRUE reported 15384_15_KL_KLOTHO KL chr7 151705816 0.009477 1.403e-10 35372 TRUE reported textfile 2 1 TRUE

3124 rs7298766 G A G A 0.2302 0.0051 0.27291 NA FALSE FALSE FALSE GCST006061 0.0078 0.5143 537409 outcome TRUE reported 15384_15_KL_KLOTHO KL chr12 552490 0.009481 3.266e-130 35311 TRUE reported textfile 2 1 TRUE

3125 rs739468 G T G T 0.1861 0.0025 0.0968 NA FALSE FALSE FALSE GCST006061 0.0104 0.8106 537409 outcome TRUE reported 15384_15_KL_KLOTHO KL chr9 133461126 0.014248 5.462e-39 35369 TRUE reported textfile 2

1 TRUE
3126 rs79122344 T C T C 0.1966 0.0106 0.01559 NA FALSE FALSE FALSE
GCST006061 0.0244 0.664 537409 outcome TRUE reported 15384_15_KL_KLOTHO KL
chr12 1503757 0.034119 8.307e-09 35315 TRUE reported textfile 2 1
TRUE
3127 rs79282194 G A G A -0.1086 -0.0197 0.05101 NA FALSE FALSE FALSE
GCST006061 0.0212 0.3513 537409 outcome TRUE reported 15384_15_KL_KLOTHO KL
chr13 33913961 0.019309 1.862e-08 35359 TRUE reported textfile 2
1 TRUE
3128 rs7984515 G A G A 0.086 -0.0095 0.21951 NA FALSE FALSE FALSE
GCST006061 0.0091 0.2952 537409 outcome TRUE reported 15384_15_KL_KLOTHO KL
chr13 32992027 0.01017 2.757e-17 35363 TRUE reported textfile 2 1
TRUE
3129 rs9536282 T C T C 0.1411 -0.0115 0.17926 NA FALSE FALSE FALSE
GCST006061 0.01 0.2496 537409 outcome TRUE reported 15384_15_KL_KLOTHO KL
chr13 33043525 0.010944 4.968e-38 35363 TRUE reported textfile 2
1 TRUE
3130 rs9961915 T C T C 0.2282 -0.0074 0.32989 NA FALSE FALSE FALSE
GCST006061 0.0075 0.3231 537409 outcome TRUE reported 15384_15_KL_KLOTHO KL
chr18 27107360 0.008867 4.709e-146 35370 TRUE reported textfile 2
1 TRUE
3131 rs10468017 T C T C -0.0671 -0.0147 0.30659 NA FALSE FALSE FALSE
GCST006061 0.0079 0.06249 537409 outcome TRUE reported 9061_3_ARHGEF10_ARHGA
ARHGEF10 chr15 58386313 0.008673 1.021e-14 35364 TRUE reported
textfile 2 1 TRUE
3132 rs6083 G A G A 0.0708 -6.00E-04 0.40059 NA FALSE FALSE FALSE
GCST006061 0.0077 0.9388 537409 outcome TRUE reported 9061_3_ARHGEF10_ARHGA
ARHGEF10 chr15 58545811 0.008159 4.03e-18 35367 TRUE reported
textfile 2 1 TRUE
3133 rs10469327 T C T C -0.236 -0.0151 0.07136 NA FALSE FALSE FALSE
GCST006061 0.0187 0.4209 537409 outcome TRUE reported 2946_52_CFD_Factor_D
CFD chr19 858376 0.016532 3.124e-46 35315 TRUE reported textfile 2
1 TRUE
3134 rs10922098 T C T C -0.1921 -0.009 0.40011 NA FALSE FALSE FALSE
GCST006061 0.0075 0.2275 537409 outcome TRUE reported 2946_52_CFD_Factor_D
CFD chr1 196695521 0.008516 1.104e-112 35338 TRUE reported textfile
2 1 TRUE
3135 rs116940656 T G T G -0.1236 0.0137 0.04498 NA FALSE FALSE FALSE
GCST006061 0.0324 0.6733 537409 outcome TRUE reported 2946_52_CFD_Factor_D
CFD chr19 1368489 0.020406 1.386e-09 35319 TRUE reported textfile 2
1 TRUE
3136 rs117778730 G T G T 0.1353 -0.0165 0.05015 NA FALSE FALSE FALSE
GCST006061 0.0449 0.7132 537409 outcome TRUE reported 2946_52_CFD_Factor_D
CFD chr19 1098280 0.019432 3.34e-12 35319 TRUE reported textfile 2
1 TRUE
3137 rs1357134 G A G A -0.0709 0.0061 0.43695 NA FALSE FALSE FALSE
GCST006061 0.0073 0.4052 537409 outcome TRUE reported 2946_52_CFD_Factor_D
CFD chr3 187261980 0.00848 6.22e-17 35368 TRUE reported textfile 2
1 TRUE
3138 rs147416397 C T C T -0.1648 0.0543 0.03043 NA FALSE FALSE FALSE
GCST006061 0.0459 0.2366 537409 outcome TRUE reported 2946_52_CFD_Factor_D
CFD chr19 897601 0.024668 2.379e-11 35318 TRUE reported textfile 2
1 TRUE
3139 rs1486236 A C A C 0.0998 0.0029 0.34055 NA FALSE FALSE FALSE
GCST006061 0.0076 0.7053 537409 outcome TRUE reported 2946_52_CFD_Factor_D
CFD chr2 179874723 0.008816 1.048e-29 35351 TRUE reported textfile
2 1 TRUE
3140 rs1707652 T C T C 0.0765 0.0081 0.3102 NA FALSE FALSE FALSE
GCST006061 0.0074 0.2701 537409 outcome TRUE reported 2946_52_CFD_Factor_D
CFD chr3 165761011 0.009021 2.243e-17 35339 TRUE reported textfile
2 1 TRUE
3141 rs6542680 T C T C 0.0679 -0.0012 0.18362 NA FALSE FALSE FALSE
GCST006061 0.0091 0.8935 537409 outcome TRUE reported 2946_52_CFD_Factor_D
CFD chr2 3592552 0.01083 3.617e-10 35332 TRUE reported textfile 2 1
TRUE
3142 rs704 A G A G -0.0671 -0.0155 0.49189 NA FALSE FALSE FALSE GCST006061

0.0072 0.03144 537409 outcome TRUE reported 2946_52_CFD_Factor_D CFD chr17 28367840 0.008376 1.142e-15 35360 TRUE reported textfile 2 1 TRUE
3143 rs74520045 T C T C 0.1317 -0.0263 0.06667 NA FALSE FALSE FALSE GCST006061 0.0232 0.2566 537409 outcome TRUE reported 2946_52_CFD_Factor_D CFD chr19 925677 0.017 9.399e-15 35318 TRUE reported textfile 2 1 TRUE
3144 rs10471928 C T C T 0.1435 0.0023 0.43667 NA FALSE FALSE FALSE GCST006061 0.0073 0.7497 537409 outcome TRUE reported 9545_156_GZMK_Granzyme_K GZMK chr5 55002903 0.008142 1.598e-69 35362 TRUE reported textfile 2 1 TRUE
3145 rs11567694 G A G A -0.049 0.0014 0.31984 NA FALSE FALSE FALSE GCST006061 0.008 0.8653 537409 outcome TRUE reported 9545_156_GZMK_Granzyme_K GZMK chr5 35857602 0.008685 1.682e-08 35381 TRUE reported textfile 2 1 TRUE
3146 rs12054477 C T C T 0.0705 0.0089 0.1248 NA FALSE FALSE FALSE GCST006061 0.0095 0.3465 537409 outcome TRUE reported 9545_156_GZMK_Granzyme_K GZMK chr3 28483541 0.012291 9.689e-09 35345 TRUE reported textfile 2 1 TRUE
3147 rs2110690 G A G A 0.0445 -0.0063 0.45578 NA FALSE FALSE FALSE GCST006061 0.0071 0.377 537409 outcome TRUE reported 9545_156_GZMK_Granzyme_K GZMK chr2 201320409 0.008132 4.441e-08 35365 TRUE reported textfile 2 1 TRUE
3148 rs3181348 A G A G -0.0562 -0.0165 0.44477 NA FALSE FALSE FALSE GCST006061 0.0072 0.02195 537409 outcome TRUE reported 9545_156_GZMK_Granzyme_K GZMK chr9 114931904 0.008154 5.49e-12 35362 TRUE reported textfile 2 1 TRUE
3149 rs3797235 C T C T -0.0582 -0.0052 0.18751 NA FALSE FALSE FALSE GCST006061 0.0089 0.5635 537409 outcome TRUE reported 9545_156_GZMK_Granzyme_K GZMK chr5 54252130 0.010389 2.121e-08 35366 TRUE reported textfile 2 1 TRUE
3150 rs6693121 A C A C -0.0643 0.0017 0.43231 NA FALSE FALSE FALSE GCST006061 0.0073 0.8191 537409 outcome TRUE reported 9545_156_GZMK_Granzyme_K GZMK chr1 101279077 0.008162 3.328e-15 35350 TRUE reported textfile 2 1 TRUE
3151 rs10472392 A G A G 0.0485 0.0038 0.34484 NA FALSE FALSE FALSE GCST006061 0.0075 0.6099 537409 outcome TRUE reported 18917_53_AMY2A_Pancreatic_alpha_amylase AMY2A chr5 44290472 0.008352 6.363e-09 35361 TRUE reported textfile 2 1 TRUE
3152 rs114118243 A G A G 0.1765 -0.0237 0.02129 NA FALSE FALSE FALSE GCST006061 0.0303 0.4347 537409 outcome TRUE reported 18917_53_AMY2A_Pancreatic_alpha_amylase AMY2A chr1 107590695 0.029151 1.407e-09 35351 TRUE reported textfile 2 1 TRUE
3153 rs114500706 G A G A -0.6143 -0.0297 0.07265 NA FALSE FALSE FALSE GCST006061 0.0188 0.114 537409 outcome TRUE reported 18917_53_AMY2A_Pancreatic_alpha_amylase AMY2A chr1 103849546 0.011576 1e-200 35349 TRUE reported textfile 2 1 TRUE
3154 rs115614347 G A G A 0.1871 -0.0395 0.01644 NA FALSE FALSE FALSE GCST006061 0.0462 0.3924 537409 outcome TRUE reported 18917_53_AMY2A_Pancreatic_alpha_amylase AMY2A chr1 95697962 0.033239 1.813e-08 35356 TRUE reported textfile 2 1 TRUE
3155 rs11578575 T C T C -0.221 -0.0143 0.02307 NA FALSE FALSE FALSE GCST006061 0.0395 0.7164 537409 outcome TRUE reported 18917_53_AMY2A_Pancreatic_alpha_amylase AMY2A chr1 102023783 0.027703 1.495e-15 35344 TRUE reported textfile 2 1 TRUE
3156 rs116131946 A C A C -0.3766 0.0483 0.01151 NA FALSE FALSE FALSE GCST006061 0.0405 0.2322 537409 outcome TRUE reported 18917_53_AMY2A_Pancreatic_alpha_amylase AMY2A chr1 105330223 0.040337 9.964e-21 35352 TRUE reported textfile 2 1 TRUE
3157 rs138026918 G A G A -0.5394 0.005 0.02273 NA FALSE FALSE FALSE GCST006061 0.038 0.8948 537409 outcome TRUE reported 18917_53_AMY2A_Pancreatic_alpha_amylase AMY2A chr1 105562652 0.02837 1.338e-80 35349 TRUE reported textfile 2 1 TRUE
3158 rs28365941 C T C T -0.0857 -0.0107 0.10449 NA FALSE FALSE FALSE GCST006061 0.0114 0.3499 537409 outcome TRUE reported 18917_53_AMY2A_Pancreatic_alpha_amylase AMY2A chr16 283138 0.013191 8.197e-11 35313 TRUE reported textfile 2 1 TRUE

3159 rs4847148 A C A C -0.5642 0.001 0.01533 NA FALSE FALSE FALSE
GCST006061 0.0386 0.9788 537409 outcome TRUE reported
18917_53_AMY2A_Pancreatic_alpha_amylase AMY2A chr1 103907224 0.033797
1.457e-62 35349 TRUE reported textfile 2 1 TRUE
3160 rs533406 G A G A -0.0819 -0.0177 0.41964 NA FALSE FALSE FALSE
GCST006061 0.0075 0.01777 537409 outcome TRUE reported
18917_53_AMY2A_Pancreatic_alpha_amylase AMY2A chr16 88908452 0.008111
5.676e-24 35321 TRUE reported textfile 2 1 TRUE
3161 rs635634 C T C T 0.1986 0.0285 0.12995 NA FALSE FALSE FALSE
GCST006061 0.0089 0.001351 537409 outcome TRUE reported
18917_53_AMY2A_Pancreatic_alpha_amylase AMY2A chr9 133279427 0.011855
5.405e-63 35371 TRUE reported textfile 2 1 TRUE
3162 rs67824503 C T C T -0.0692 0.0058 0.24087 NA FALSE FALSE FALSE
GCST006061 0.0088 0.5145 537409 outcome TRUE reported
18917_53_AMY2A_Pancreatic_alpha_amylase AMY2A chr8 128523018 0.009289
9.372e-14 35366 TRUE reported textfile 2 1 TRUE
3163 rs75235839 T C T C 0.1853 0.0175 0.01822 NA FALSE FALSE FALSE
GCST006061 0.0303 0.564 537409 outcome TRUE reported
18917_53_AMY2A_Pancreatic_alpha_amylase AMY2A chr1 104862493 0.031327
3.319e-09 35348 TRUE reported textfile 2 1 TRUE
3164 rs76527254 A C A C 0.182 -0.0801 0.037 NA FALSE FALSE FALSE
GCST006061 0.0322 0.01291 537409 outcome TRUE reported
18917_53_AMY2A_Pancreatic_alpha_amylase AMY2A chr1 101712735 0.022438
5.012e-16 35344 TRUE reported textfile 2 1 TRUE
3165 rs76836317 T C T C -0.2303 -0.0168 0.02037 NA FALSE FALSE FALSE
GCST006061 0.037 0.6501 537409 outcome TRUE reported
18917_53_AMY2A_Pancreatic_alpha_amylase AMY2A chr1 104568569 0.030202
2.434e-14 35354 TRUE reported textfile 2 1 TRUE
3166 rs8051363 G A G A 0.0599 -0.005 0.26103 NA FALSE FALSE FALSE
GCST006061 0.0083 0.5493 537409 outcome TRUE reported
18917_53_AMY2A_Pancreatic_alpha_amylase AMY2A chr16 75221319 0.00909 4.413e-11
35362 TRUE reported textfile 2 1 TRUE
3167 rs9482770 C T C T 0.0564 0.002 0.47412 NA FALSE FALSE FALSE
GCST006061 0.0071 0.7769 537409 outcome TRUE reported
18917_53_AMY2A_Pancreatic_alpha_amylase AMY2A chr6 127121947 0.008007
1.865e-12 35355 TRUE reported textfile 2 1 TRUE
3168 rs10472392 A G A G 0.0647 0.0038 0.34484 NA FALSE FALSE FALSE
GCST006061 0.0075 0.6099 537409 outcome TRUE reported
15556_49_AMY2B_Alpha_amylase_2B AMY2B chr5 44290472 0.008322 7.571e-15
35361 TRUE reported textfile 2 1 TRUE
3169 rs114118243 A G A G 0.212 -0.0237 0.02129 NA FALSE FALSE FALSE
GCST006061 0.0303 0.4347 537409 outcome TRUE reported
15556_49_AMY2B_Alpha_amylase_2B AMY2B chr1 107590695 0.029103 3.231e-13
35351 TRUE reported textfile 2 1 TRUE
3170 rs114279212 C A C A 0.1938 0.002 0.02596 NA FALSE FALSE FALSE
GCST006061 0.0326 0.9507 537409 outcome TRUE reported
15556_49_AMY2B_Alpha_amylase_2B AMY2B chr1 105266751 0.026512 2.675e-13
35352 TRUE reported textfile 2 1 TRUE
3171 rs114500706 G A G A -0.487 -0.0297 0.07265 NA FALSE FALSE FALSE
GCST006061 0.0188 0.114 537409 outcome TRUE reported
15556_49_AMY2B_Alpha_amylase_2B AMY2B chr1 103849546 0.01608 1e-200 35349
TRUE reported textfile 2 1 TRUE
3172 rs11578575 T C T C -0.2949 -0.0143 0.02307 NA FALSE FALSE FALSE
GCST006061 0.0395 0.7164 537409 outcome TRUE reported
15556_49_AMY2B_Alpha_amylase_2B AMY2B chr1 102023783 0.027653 1.495e-26
35344 TRUE reported textfile 2 1 TRUE
3173 rs138026918 G A G A -0.4032 0.005 0.02273 NA FALSE FALSE FALSE
GCST006061 0.038 0.8948 537409 outcome TRUE reported
15556_49_AMY2B_Alpha_amylase_2B AMY2B chr1 105562652 0.028389 8.802e-46
35349 TRUE reported textfile 2 1 TRUE
3174 rs1713235 T C T C 0.0548 0.0071 0.4341 NA FALSE FALSE FALSE
GCST006061 0.0073 0.3361 537409 outcome TRUE reported
15556_49_AMY2B_Alpha_amylase_2B AMY2B chr1 206888427 0.008481 1.037e-10
35370 TRUE reported textfile 2 1 TRUE
3175 rs34858945 A G A G 0.078 -0.0127 0.12485 NA FALSE FALSE FALSE
GCST006061 0.0105 0.2275 537409 outcome TRUE reported

15556_49_AMY2B_Alpha_amylase_2B AMY2B chr12 11208431 0.011992 7.804e-11 35376 TRUE reported textfile 2 1 TRUE
3176 rs4847148 A C A C -0.4514 0.001 0.01533 NA FALSE FALSE FALSE GCST006061 0.0386 0.9788 537409 outcome TRUE reported 15556_49_AMY2B_Alpha_amylase_2B AMY2B chr1 103907224 0.033791 1.058e-40 35349 TRUE reported textfile 2 1 TRUE
3177 rs533406 G A G A -0.0595 -0.0177 0.41964 NA FALSE FALSE FALSE GCST006061 0.0075 0.01777 537409 outcome TRUE reported 15556_49_AMY2B_Alpha_amylase_2B AMY2B chr16 88908452 0.008066 1.624e-13 35321 TRUE reported textfile 2 1 TRUE
3178 rs635634 C T C T 0.1679 0.0285 0.12995 NA FALSE FALSE FALSE GCST006061 0.0089 0.001351 537409 outcome TRUE reported 15556_49_AMY2B_Alpha_amylase_2B AMY2B chr9 133279427 0.011808 6.919e-46 35371 TRUE reported textfile 2 1 TRUE
3179 rs6678423 C T C T 0.0883 -0.0026 0.17578 NA FALSE FALSE FALSE GCST006061 0.0101 0.7992 537409 outcome TRUE reported 15556_49_AMY2B_Alpha_amylase_2B AMY2B chr1 106079579 0.011004 1.023e-15 35353 TRUE reported textfile 2 1 TRUE
3180 rs75782947 C T C T -0.197 3e-04 0.05105 NA FALSE FALSE FALSE GCST006061 0.0218 0.9875 537409 outcome TRUE reported 15556_49_AMY2B_Alpha_amylase_2B AMY2B chr1 102619351 0.019066 5.032e-25 35349 TRUE reported textfile 2 1 TRUE
3181 rs76527254 A C A C 0.2193 -0.0801 0.037 NA FALSE FALSE FALSE GCST006061 0.0322 0.01291 537409 outcome TRUE reported 15556_49_AMY2B_Alpha_amylase_2B AMY2B chr1 101712735 0.022408 1.286e-22 35344 TRUE reported textfile 2 1 TRUE
3182 rs76836317 T C T C -0.2149 -0.0168 0.02037 NA FALSE FALSE FALSE GCST006061 0.037 0.6501 537409 outcome TRUE reported 15556_49_AMY2B_Alpha_amylase_2B AMY2B chr1 104568569 0.03017 1.056e-12 35354 TRUE reported textfile 2 1 TRUE
3183 rs1047286 A G A G -0.0613 -0.0013 0.24066 NA FALSE FALSE FALSE GCST006061 0.0098 0.8921 537409 outcome TRUE reported 10677_9_NDUFB4_NDUB4 NDUFB4 chr19 6713251 0.009393 6.76e-11 35360 TRUE reported textfile 2 1 TRUE
3184 rs10801660 A G A G 0.0868 -0.0119 0.11574 NA FALSE FALSE FALSE GCST006061 0.0108 0.2735 537409 outcome TRUE reported 10677_9_NDUFB4_NDUB4 NDUFB4 chr1 198272225 0.012591 5.431e-12 35333 TRUE reported textfile 2 1 TRUE
3185 rs1160610 A C A C -0.0513 -0.0033 0.44388 NA FALSE FALSE FALSE GCST006061 0.0072 0.6396 537409 outcome TRUE reported 10677_9_NDUFB4_NDUB4 NDUFB4 chr17 33048645 0.008137 2.897e-10 35360 TRUE reported textfile 2 1 TRUE
3186 rs1329424 G T G T -0.2984 -0.0095 0.39735 NA FALSE FALSE FALSE GCST006061 0.0075 0.2071 537409 outcome TRUE reported 10677_9_NDUFB4_NDUB4 NDUFB4 chr1 196677046 0.00816 1e-200 35326 TRUE reported textfile 2 1 TRUE
3187 rs1707652 T C T C 0.1232 0.0081 0.3102 NA FALSE FALSE FALSE GCST006061 0.0074 0.2701 537409 outcome TRUE reported 10677_9_NDUFB4_NDUB4 NDUFB4 chr3 165761011 0.008667 7.449e-46 35327 TRUE reported textfile 2 1 TRUE
3188 rs72748420 T C T C 0.1059 0.0095 0.07417 NA FALSE FALSE FALSE GCST006061 0.0134 0.4784 537409 outcome TRUE reported 10677_9_NDUFB4_NDUB4 NDUFB4 chr1 196141224 0.015367 5.517e-12 35325 TRUE reported textfile 2 1 TRUE
3189 rs73169117 T C T C 0.0713 0.0051 0.11405 NA FALSE FALSE FALSE GCST006061 0.0122 0.6755 537409 outcome TRUE reported 10677_9_NDUFB4_NDUB4 NDUFB4 chr3 167217620 0.012746 2.219e-08 35342 TRUE reported textfile 2 1 TRUE
3190 rs9282801 A C A C 0.0667 -0.0102 0.35559 NA FALSE FALSE FALSE GCST006061 0.0076 0.1783 537409 outcome TRUE reported 10677_9_NDUFB4_NDUB4 NDUFB4 chr17 27769447 0.008505 4.417e-15 35346 TRUE reported textfile 2 1 TRUE
3191 rs1047286 A G A G -0.0623 -0.0013 0.24066 NA FALSE FALSE FALSE GCST006061 0.0098 0.8921 537409 outcome TRUE reported 16854_17_LSM4_LSM4 LSM4 chr19 6713251 0.00939 3.255e-11 35372 TRUE reported textfile 2 1 TRUE

3192 rs114009540 A G A G -0.1215 0.0322 0.03606 NA FALSE FALSE FALSE GCST006061 0.0299 0.2809 537409 outcome TRUE reported 16854_17_LSM4_LSM4 LSM4 chr1 196158413 0.021802 2.506e-08 35337 TRUE reported textfile 2 1 TRUE
3193 rs1329424 G T G T -0.2507 -0.0095 0.39735 NA FALSE FALSE FALSE GCST006061 0.0075 0.2071 537409 outcome TRUE reported 16854_17_LSM4_LSM4 LSM4 chr1 196677046 0.008161 1e-200 35338 TRUE reported textfile 2 1 TRUE
3194 rs1707652 T C T C 0.1146 0.0081 0.3102 NA FALSE FALSE FALSE GCST006061 0.0074 0.2701 537409 outcome TRUE reported 16854_17_LSM4_LSM4 LSM4 chr3 165761011 0.008655 5.081e-40 35339 TRUE reported textfile 2 1 TRUE
3195 rs704 A G A G 0.0528 -0.0155 0.49189 NA FALSE FALSE FALSE GCST006061 0.0072 0.03144 537409 outcome TRUE reported 16854_17_LSM4_LSM4 LSM4 chr17 28367840 0.008049 5.39e-11 35360 TRUE reported textfile 2 1 TRUE
3196 rs72748420 T C T C 0.085 0.0095 0.07417 NA FALSE FALSE FALSE GCST006061 0.0134 0.4784 537409 outcome TRUE reported 16854_17_LSM4_LSM4 LSM4 chr1 196141224 0.015306 2.8e-08 35337 TRUE reported textfile 2 1 TRUE
3197 rs1047286 A G A G -0.0571 -0.0013 0.24066 NA FALSE FALSE FALSE GCST006061 0.0098 0.8921 537409 outcome TRUE reported 13525_17_KCNG4_KCNG4 KCNG4 chr19 6713251 0.009325 9.159e-10 35366 TRUE reported textfile 2 1 TRUE
3198 rs10922098 T C T C -0.207 -0.009 0.40011 NA FALSE FALSE FALSE GCST006061 0.0075 0.2275 537409 outcome TRUE reported 13525_17_KCNG4_KCNG4 KCNG4 chr1 196695521 0.008111 1.136e-143 35332 TRUE reported textfile 2 1 TRUE
3199 rs1707652 T C T C 0.0917 0.0081 0.3102 NA FALSE FALSE FALSE GCST006061 0.0074 0.2701 537409 outcome TRUE reported 13525_17_KCNG4_KCNG4 KCNG4 chr3 165761011 0.008606 1.645e-26 35333 TRUE reported textfile 2 1 TRUE
3200 rs1047286 A G A G -0.0613 -0.0013 0.24066 NA FALSE FALSE FALSE GCST006061 0.0098 0.8921 537409 outcome TRUE reported 4556_10_IL34_IL_34 IL34 chr19 6713251 0.009419 7.612e-11 35372 TRUE reported textfile 2 1 TRUE
3201 rs10801660 A G A G 0.0806 -0.0119 0.11574 NA FALSE FALSE FALSE GCST006061 0.0108 0.2735 537409 outcome TRUE reported 4556_10_IL34_IL_34 IL34 chr1 198272225 0.0126 1.586e-10 35345 TRUE reported textfile 2 1 TRUE
3202 rs1329424 G T G T -0.3025 -0.0095 0.39735 NA FALSE FALSE FALSE GCST006061 0.0075 0.2071 537409 outcome TRUE reported 4556_10_IL34_IL_34 IL34 chr1 196677046 0.008156 1e-200 35338 TRUE reported textfile 2 1 TRUE
3203 rs1707652 T C T C 0.1177 0.0081 0.3102 NA FALSE FALSE FALSE GCST006061 0.0074 0.2701 537409 outcome TRUE reported 4556_10_IL34_IL_34 IL34 chr3 165761011 0.00868 6.987e-42 35339 TRUE reported textfile 2 1 TRUE
3204 rs3747843 G A G A -0.0445 -0.0037 0.47828 NA FALSE FALSE FALSE GCST006061 0.0071 0.6026 537409 outcome TRUE reported 4556_10_IL34_IL_34 IL34 chr9 121152028 0.008065 3.429e-08 35373 TRUE reported textfile 2 1 TRUE
3205 rs72748420 T C T C 0.0981 0.0095 0.07417 NA FALSE FALSE FALSE GCST006061 0.0134 0.4784 537409 outcome TRUE reported 4556_10_IL34_IL_34 IL34 chr1 196141224 0.01538 1.791e-10 35337 TRUE reported textfile 2 1 TRUE
3206 rs1047286 A G A G -0.0534 -0.0013 0.24066 NA FALSE FALSE FALSE GCST006061 0.0098 0.8921 537409 outcome TRUE reported 16852_10_PDZK1_NHRF3 PDZK1 chr19 6713251 0.009305 9.546e-09 35372 TRUE reported textfile 2 1 TRUE
3207 rs10922098 T C T C -0.157 -0.009 0.40011 NA FALSE FALSE FALSE GCST006061 0.0075 0.2275 537409 outcome TRUE reported 16852_10_PDZK1_NHRF3 PDZK1 chr1 196695521 0.008109 1.62e-83 35338 TRUE reported textfile 2 1 TRUE
3208 rs1707652 T C T C 0.075 0.0081 0.3102 NA FALSE FALSE FALSE GCST006061 0.0074 0.2701 537409 outcome TRUE reported 16852_10_PDZK1_NHRF3 PDZK1 chr3 165761011 0.008592 2.563e-18 35339 TRUE reported

textfile 2 1 TRUE
3209 rs1047286 A G A G -0.0654 -0.0013 0.24066 NA FALSE FALSE FALSE
GCST006061 0.0098 0.8921 537409 outcome TRUE reported 6468_37_TAC4_TKN4
TAC4 chr19 6713251 0.009332 2.413e-12 35372 TRUE reported textfile
2 1 TRUE
3210 rs10922098 T C T C -0.1901 -0.009 0.40011 NA FALSE FALSE FALSE
GCST006061 0.0075 0.2275 537409 outcome TRUE reported 6468_37_TAC4_TKN4
TAC4 chr1 196695521 0.008126 4.86e-121 35338 TRUE reported
textfile 2 1 TRUE
3211 rs1451915 A C A C 0.0646 -0.0107 0.13641 NA FALSE FALSE FALSE
GCST006061 0.0124 0.3895 537409 outcome TRUE reported 6468_37_TAC4_TKN4
TAC4 chr1 196058002 0.01159 2.49e-08 35335 TRUE reported textfile
2 1 TRUE
3212 rs2686395 C T C T -0.105 -0.0077 0.3101 NA FALSE FALSE FALSE
GCST006061 0.0074 0.2965 537409 outcome TRUE reported 6468_37_TAC4_TKN4
TAC4 chr3 165767231 0.008617 3.721e-34 35339 TRUE reported
textfile 2 1 TRUE
3213 rs1047286 A G A G -0.0646 -0.0013 0.24066 NA FALSE FALSE FALSE
GCST006061 0.0098 0.8921 537409 outcome TRUE reported 19374_72_MTCP1_MTCP1
MTCP1 chr19 6713251 0.009389 5.979e-12 35372 TRUE reported textfile
2 1 TRUE
3214 rs10922098 T C T C -0.1665 -0.009 0.40011 NA FALSE FALSE FALSE
GCST006061 0.0075 0.2275 537409 outcome TRUE reported 19374_72_MTCP1_MTCP1
MTCP1 chr1 196695521 0.008172 2.786e-92 35338 TRUE reported
textfile 2 1 TRUE
3215 rs1706420 C T C T 0.0778 0.0122 0.36627 NA FALSE FALSE FALSE
GCST006061 0.0072 0.0907 537409 outcome TRUE reported 19374_72_MTCP1_MTCP1
MTCP1 chr3 165732681 0.008325 9.15e-21 35339 TRUE reported
textfile 2 1 TRUE
3216 rs35209896 A C A C 0.0774 0.0267 0.09263 NA FALSE FALSE FALSE
GCST006061 0.0355 0.4521 537409 outcome TRUE reported 19374_72_MTCP1_MTCP1
MTCP1 chr6 32519009 0.013744 1.784e-08 35439 TRUE reported
textfile 2 1 TRUE
3217 rs1047286 A G A G -0.0523 -0.0013 0.24066 NA FALSE FALSE FALSE
GCST006061 0.0098 0.8921 537409 outcome TRUE reported 13604_27_NEURL1_NEUL1
NEURL1 chr19 6713251 0.009342 2.167e-08 35366 TRUE reported textfile
2 1 TRUE
3218 rs10922098 T C T C -0.1915 -0.009 0.40011 NA FALSE FALSE FALSE
GCST006061 0.0075 0.2275 537409 outcome TRUE reported 13604_27_NEURL1_NEUL1
NEURL1 chr1 196695521 0.008138 1.876e-122 35332 TRUE reported
textfile 2 1 TRUE
3219 rs1707652 T C T C 0.1054 0.0081 0.3102 NA FALSE FALSE FALSE
GCST006061 0.0074 0.2701 537409 outcome TRUE reported 13604_27_NEURL1_NEUL1
NEURL1 chr3 165761011 0.008624 2.382e-34 35333 TRUE reported
textfile 2 1 TRUE
3220 rs1047286 A G A G -0.0671 -0.0013 0.24066 NA FALSE FALSE FALSE
GCST006061 0.0098 0.8921 537409 outcome TRUE reported 7752_31_CLEC4D_CLC4D
CLEC4D chr19 6713251 0.009367 7.885e-13 35372 TRUE reported textfile
2 1 TRUE
3221 rs10922098 T C T C -0.1944 -0.009 0.40011 NA FALSE FALSE FALSE
GCST006061 0.0075 0.2275 537409 outcome TRUE reported 7752_31_CLEC4D_CLC4D
CLEC4D chr1 196695521 0.008147 7.715e-126 35338 TRUE reported
textfile 2 1 TRUE
3222 rs1707652 T C T C 0.1018 0.0081 0.3102 NA FALSE FALSE FALSE
GCST006061 0.0074 0.2701 537409 outcome TRUE reported 7752_31_CLEC4D_CLC4D
CLEC4D chr3 165761011 0.008638 4.649e-32 35339 TRUE reported
textfile 2 1 TRUE
3223 rs35351085 T C T C 0.1021 0.0061 0.04877 NA FALSE FALSE FALSE
GCST006061 0.022 0.7825 537409 outcome TRUE reported 7752_31_CLEC4D_CLC4D
CLEC4D chr1 197992799 0.018513 3.487e-08 35343 TRUE reported
textfile 2 1 TRUE
3224 rs1047286 A G A G 0.0727 -0.0013 0.24066 NA FALSE FALSE FALSE
GCST006061 0.0098 0.8921 537409 outcome TRUE reported 15698_6_TXLNA_TXLNA
TXLNA chr19 6713251 0.00944 1.344e-14 35372 TRUE reported textfile 2
1 TRUE
3225 rs10876550 A G A G 0.0467 -1.00E-04 0.43699 NA FALSE FALSE FALSE

GCST006061 0.0073 0.991 537409 outcome TRUE reported 15698_6_TXLNA_TXLNA
TXLNA chr12 54318524 0.008164 1.063e-08 35374 TRUE reported
textfile 2 1 TRUE
3226 rs10922098 T C T C -0.046 -0.009 0.40011 NA FALSE FALSE FALSE
GCST006061 0.0075 0.2275 537409 outcome TRUE reported 15698_6_TXLNA_TXLNA
TXLNA chr1 196695521 0.008253 2.494e-08 35338 TRUE reported
textfile 2 1 TRUE
3227 rs1354034 C T C T 0.0698 0.0088 0.38119 NA FALSE FALSE FALSE
GCST006061 0.0073 0.2248 537409 outcome TRUE reported 15698_6_TXLNA_TXLNA
TXLNA chr3 56815721 0.008313 4.614e-17 35356 TRUE reported
textfile 2 1 TRUE
3228 rs1355538 G A G A -0.0464 -0.0076 0.39847 NA FALSE FALSE FALSE
GCST006061 0.0072 0.2923 537409 outcome TRUE reported 15698_6_TXLNA_TXLNA
TXLNA chr3 165787389 0.008234 1.75e-08 35338 TRUE reported
textfile 2 1 TRUE
3229 rs4980320 C T C T 0.0496 0.0041 0.46986 NA FALSE FALSE FALSE
GCST006061 0.0076 0.5858 537409 outcome TRUE reported 15698_6_TXLNA_TXLNA
TXLNA chr11 198986 0.008096 8.98e-10 35352 TRUE reported textfile
2 1 TRUE
3230 rs1047286 A G A G -0.0566 -0.0013 0.24066 NA FALSE FALSE FALSE
GCST006061 0.0098 0.8921 537409 outcome TRUE reported 18306_1_PALM_PALM
PALM chr19 6713251 0.00938 1.598e-09 35372 TRUE reported textfile 2
1 TRUE
3231 rs10922098 T C T C -0.2053 -0.009 0.40011 NA FALSE FALSE FALSE
GCST006061 0.0075 0.2275 537409 outcome TRUE reported 18306_1_PALM_PALM
PALM chr1 196695521 0.008157 9.179e-140 35338 TRUE reported
textfile 2 1 TRUE
3232 rs1555405 A G A G -0.0479 -0.0047 0.30381 NA FALSE FALSE FALSE
GCST006061 0.0081 0.5592 537409 outcome TRUE reported 18306_1_PALM_PALM
PALM chr14 100710432 0.008772 4.749e-08 35380 TRUE reported
textfile 2 1 TRUE
3233 rs2686395 C T C T -0.0964 -0.0077 0.3101 NA FALSE FALSE FALSE
GCST006061 0.0074 0.2965 537409 outcome TRUE reported 18306_1_PALM_PALM
PALM chr3 165767231 0.008656 8.366e-29 35339 TRUE reported
textfile 2 1 TRUE
3234 rs1047286 A G A G -0.0607 -0.0013 0.24066 NA FALSE FALSE FALSE
GCST006061 0.0098 0.8921 537409 outcome TRUE reported 13007_66_LCN10_LCN10
LCN10 chr19 6713251 0.009389 1.013e-10 35363 TRUE reported textfile
2 1 TRUE
3235 rs111696008 A G A G -0.1649 -0.0259 0.02443 NA FALSE FALSE FALSE
GCST006061 0.0401 0.519 537409 outcome TRUE reported 13007_66_LCN10_LCN10
LCN10 chr2 216622986 0.026713 6.699e-10 35350 TRUE reported
textfile 2 1 TRUE
3236 rs11208686 A G A G -0.0569 -0.0159 0.20129 NA FALSE FALSE FALSE
GCST006061 0.009 0.07749 537409 outcome TRUE reported 13007_66_LCN10_LCN10
LCN10 chr1 65604919 0.010062 1.557e-08 35338 TRUE reported
textfile 2 1 TRUE
3237 rs1329424 G T G T -0.2833 -0.0095 0.39735 NA FALSE FALSE FALSE
GCST006061 0.0075 0.2071 537409 outcome TRUE reported 13007_66_LCN10_LCN10
LCN10 chr1 196677046 0.00815 1e-200 35329 TRUE reported textfile 2
1 TRUE
3238 rs1707652 T C T C 0.1372 0.0081 0.3102 NA FALSE FALSE FALSE
GCST006061 0.0074 0.2701 537409 outcome TRUE reported 13007_66_LCN10_LCN10
LCN10 chr3 165761011 0.008653 1.277e-56 35330 TRUE reported
textfile 2 1 TRUE
3239 rs7513100 C T C T 0.0822 0 0.14871 NA FALSE FALSE FALSE GCST006061
0.0098 0.9981 537409 outcome TRUE reported 13007_66_LCN10_LCN10 LCN10
chr1 198065935 0.011302 3.515e-13 35334 TRUE reported textfile 2
1 TRUE
3240 rs1047286 A G A G -0.0674 -0.0013 0.24066 NA FALSE FALSE FALSE
GCST006061 0.0098 0.8921 537409 outcome TRUE reported
8767_44_ECE1_Endothelin_converting_enzyme_1 ECE1 chr19 6713251 0.009355
5.817e-13 35372 TRUE reported textfile 2 1 TRUE
3241 rs10922098 T C T C -0.2305 -0.009 0.40011 NA FALSE FALSE FALSE
GCST006061 0.0075 0.2275 537409 outcome TRUE reported
8767_44_ECE1_Endothelin_converting_enzyme_1 ECE1 chr1 196695521 0.008128

6.457e-177 35338 TRUE reported textfile 2 1 TRUE
3242 rs1707652 T C T C 0.1183 0.0081 0.3102 NA FALSE FALSE FALSE
GCST006061 0.0074 0.2701 537409 outcome TRUE reported
8767_44_ECE1_Endothelin_converting_enzyme_1 ECE1 chr3 165761011 0.008631
9.233e-43 35339 TRUE reported textfile 2 1 TRUE
3243 rs704 A G A G 0.0532 -0.0155 0.49189 NA FALSE FALSE FALSE GCST006061
0.0072 0.03144 537409 outcome TRUE reported
8767_44_ECE1_Endothelin_converting_enzyme_1 ECE1 chr17 28367840 0.008023
3.33e-11 35360 TRUE reported textfile 2 1 TRUE
3244 rs72748420 T C T C 0.084 0.0095 0.07417 NA FALSE FALSE FALSE
GCST006061 0.0134 0.4784 537409 outcome TRUE reported
8767_44_ECE1_Endothelin_converting_enzyme_1 ECE1 chr1 196141224 0.015237
3.532e-08 35337 TRUE reported textfile 2 1 TRUE
3245 rs1047286 A G A G -0.0671 -0.0013 0.24066 NA FALSE FALSE FALSE
GCST006061 0.0098 0.8921 537409 outcome TRUE reported 19356_20_TOM1L2_TM1L2
TOM1L2 chr19 6713251 0.009411 1.005e-12 35372 TRUE reported textfile
2 1 TRUE
3246 rs10801660 A G A G 0.0763 -0.0119 0.11574 NA FALSE FALSE FALSE
GCST006061 0.0108 0.2735 537409 outcome TRUE reported 19356_20_TOM1L2_TM1L2
TOM1L2 chr1 198272225 0.012574 1.294e-09 35345 TRUE reported
textfile 2 1 TRUE
3247 rs111696008 A G A G -0.1635 -0.0259 0.02443 NA FALSE FALSE FALSE
GCST006061 0.0401 0.519 537409 outcome TRUE reported 19356_20_TOM1L2_TM1L2
TOM1L2 chr2 216622986 0.026733 9.591e-10 35359 TRUE reported
textfile 2 1 TRUE
3248 rs1329424 G T G T -0.2789 -0.0095 0.39735 NA FALSE FALSE FALSE
GCST006061 0.0075 0.2071 537409 outcome TRUE reported 19356_20_TOM1L2_TM1L2
TOM1L2 chr1 196677046 0.008161 1e-200 35338 TRUE reported textfile
2 1 TRUE
3249 rs1707652 T C T C 0.1361 0.0081 0.3102 NA FALSE FALSE FALSE
GCST006061 0.0074 0.2701 537409 outcome TRUE reported 19356_20_TOM1L2_TM1L2
TOM1L2 chr3 165761011 0.008669 1.514e-55 35339 TRUE reported
textfile 2 1 TRUE
3250 rs704 A G A G 0.0778 -0.0155 0.49189 NA FALSE FALSE FALSE GCST006061
0.0072 0.03144 537409 outcome TRUE reported 19356_20_TOM1L2_TM1L2 TOM1L2
chr17 28367840 0.008053 4.43e-22 35360 TRUE reported textfile 2
1 TRUE
3251 rs72748420 T C T C 0.0915 0.0095 0.07417 NA FALSE FALSE FALSE
GCST006061 0.0134 0.4784 537409 outcome TRUE reported 19356_20_TOM1L2_TM1L2
TOM1L2 chr1 196141224 0.015355 2.538e-09 35337 TRUE reported
textfile 2 1 TRUE
3252 rs73169117 T C T C 0.0718 0.0051 0.11405 NA FALSE FALSE FALSE
GCST006061 0.0122 0.6755 537409 outcome TRUE reported 19356_20_TOM1L2_TM1L2
TOM1L2 chr3 167217620 0.01276 1.834e-08 35354 TRUE reported textfile
2 1 TRUE
3253 rs1047286 A G A G -0.0566 -0.0013 0.24066 NA FALSE FALSE FALSE
GCST006061 0.0098 0.8921 537409 outcome TRUE reported 10847_1_SIGLEC15_SIG15
SIGLEC15 chr19 6713251 0.009311 1.213e-09 35360 TRUE reported
textfile 2 1 TRUE
3254 rs10922098 T C T C -0.1836 -0.009 0.40011 NA FALSE FALSE FALSE
GCST006061 0.0075 0.2275 537409 outcome TRUE reported 10847_1_SIGLEC15_SIG15
SIGLEC15 chr1 196695521 0.00811 1.847e-113 35326 TRUE reported
textfile 2 1 TRUE
3255 rs1707652 T C T C 0.0963 0.0081 0.3102 NA FALSE FALSE FALSE
GCST006061 0.0074 0.2701 537409 outcome TRUE reported 10847_1_SIGLEC15_SIG15
SIGLEC15 chr3 165761011 0.008598 4.069e-29 35327 TRUE reported
textfile 2 1 TRUE
3256 rs35209896 A C A C 0.09 0.0267 0.09263 NA FALSE FALSE FALSE
GCST006061 0.0355 0.4521 537409 outcome TRUE reported 10847_1_SIGLEC15_SIG15
SIGLEC15 chr6 32519009 0.013637 4.115e-11 35427 TRUE reported
textfile 2 1 TRUE
3257 rs1047286 A G A G -0.0574 -0.0013 0.24066 NA FALSE FALSE FALSE
GCST006061 0.0098 0.8921 537409 outcome TRUE reported 11327_56_CLK2_CLK2
CLK2 chr19 6713251 0.009397 1.006e-09 35363 TRUE reported textfile
2 1 TRUE
3258 rs10801660 A G A G 0.0779 -0.0119 0.11574 NA FALSE FALSE FALSE

GCST006061 0.0108 0.2735 537409 outcome TRUE reported 11327_56_CLK2_CLK2
CLK2 chr1 198272225 0.012567 5.686e-10 35336 TRUE reported
textfile 2 1 TRUE
3259 rs111696008 A G A G -0.1485 -0.0259 0.02443 NA FALSE FALSE FALSE
GCST006061 0.0401 0.519 537409 outcome TRUE reported 11327_56_CLK2_CLK2
CLK2 chr2 216622986 0.026744 2.814e-08 35350 TRUE reported
textfile 2 1 TRUE
3260 rs1329424 G T G T -0.2761 -0.0095 0.39735 NA FALSE FALSE FALSE
GCST006061 0.0075 0.2071 537409 outcome TRUE reported 11327_56_CLK2_CLK2
CLK2 chr1 196677046 0.008164 1e-200 35329 TRUE reported textfile
2 1 TRUE
3261 rs1707652 T C T C 0.1235 0.0081 0.3102 NA FALSE FALSE FALSE
GCST006061 0.0074 0.2701 537409 outcome TRUE reported 11327_56_CLK2_CLK2
CLK2 chr3 165761011 0.008666 4.371e-46 35330 TRUE reported
textfile 2 1 TRUE
3262 rs704 A G A G 0.183 -0.0155 0.49189 NA FALSE FALSE FALSE GCST006061
0.0072 0.03144 537409 outcome TRUE reported 11327_56_CLK2_CLK2 CLK2 chr17
28367840 0.008023 3.712e-115 35351 TRUE reported textfile 2 1 TRUE
3263 rs72748420 T C T C 0.0934 0.0095 0.07417 NA FALSE FALSE FALSE
GCST006061 0.0134 0.4784 537409 outcome TRUE reported 11327_56_CLK2_CLK2
CLK2 chr1 196141224 0.015349 1.164e-09 35328 TRUE reported
textfile 2 1 TRUE
3264 rs1047286 A G A G -0.0666 -0.0013 0.24066 NA FALSE FALSE FALSE
GCST006061 0.0098 0.8921 537409 outcome TRUE reported 10449_31_SHISA2_TMM46
SHISA2 chr19 6713251 0.009407 1.445e-12 35360 TRUE reported textfile
2 1 TRUE
3265 rs1329424 G T G T -0.2584 -0.0095 0.39735 NA FALSE FALSE FALSE
GCST006061 0.0075 0.2071 537409 outcome TRUE reported 10449_31_SHISA2_TMM46
SHISA2 chr1 196677046 0.008174 1e-200 35326 TRUE reported textfile
2 1 TRUE
3266 rs2686395 C T C T -0.1198 -0.0077 0.3101 NA FALSE FALSE FALSE
GCST006061 0.0074 0.2965 537409 outcome TRUE reported 10449_31_SHISA2_TMM46
SHISA2 chr3 165767231 0.008675 2.24e-43 35327 TRUE reported
textfile 2 1 TRUE
3267 rs72748420 T C T C 0.0881 0.0095 0.07417 NA FALSE FALSE FALSE
GCST006061 0.0134 0.4784 537409 outcome TRUE reported 10449_31_SHISA2_TMM46
SHISA2 chr1 196141224 0.015347 9.438e-09 35325 TRUE reported
textfile 2 1 TRUE
3268 rs1047286 A G A G -0.0556 -0.0013 0.24066 NA FALSE FALSE FALSE
GCST006061 0.0098 0.8921 537409 outcome TRUE reported 7880_9_PSMA1_PSA1
PSMA1 chr19 6713251 0.009317 2.411e-09 35372 TRUE reported textfile
2 1 TRUE
3269 rs10922098 T C T C -0.2172 -0.009 0.40011 NA FALSE FALSE FALSE
GCST006061 0.0075 0.2275 537409 outcome TRUE reported 7880_9_PSMA1_PSA1
PSMA1 chr1 196695521 0.008104 3.177e-158 35338 TRUE reported
textfile 2 1 TRUE
3270 rs111696008 A G A G -0.1482 -0.0259 0.02443 NA FALSE FALSE FALSE
GCST006061 0.0401 0.519 537409 outcome TRUE reported 7880_9_PSMA1_PSA1
PSMA1 chr2 216622986 0.026517 2.286e-08 35359 TRUE reported
textfile 2 1 TRUE
3271 rs1555405 A G A G -0.0499 -0.0047 0.30381 NA FALSE FALSE FALSE
GCST006061 0.0081 0.5592 537409 outcome TRUE reported 7880_9_PSMA1_PSA1
PSMA1 chr14 100710432 0.008718 1.043e-08 35380 TRUE reported
textfile 2 1 TRUE
3272 rs1707652 T C T C 0.1011 0.0081 0.3102 NA FALSE FALSE FALSE
GCST006061 0.0074 0.2701 537409 outcome TRUE reported 7880_9_PSMA1_PSA1
PSMA1 chr3 165761011 0.008601 6.675e-32 35339 TRUE reported
textfile 2 1 TRUE
3273 rs704 A G A G 0.0829 -0.0155 0.49189 NA FALSE FALSE FALSE GCST006061
0.0072 0.03144 537409 outcome TRUE reported 7880_9_PSMA1_PSA1 PSMA1 chr17
28367840 0.007984 2.97e-25 35360 TRUE reported textfile 2 1 TRUE
3274 rs9276578 G A G A 0.0541 0.0195 0.21102 NA FALSE FALSE FALSE
GCST006061 0.01 0.05075 537409 outcome TRUE reported 7880_9_PSMA1_PSA1
PSMA1 chr6 32758497 0.009835 3.778e-08 35438 TRUE reported
textfile 2 1 TRUE
3275 rs1047286 A G A G -0.0575 -0.0013 0.24066 NA FALSE FALSE FALSE

GCST006061 0.0098 0.8921 537409 outcome TRUE reported 13540_1_GPR26_GPR26
GPR26 chr19 6713251 0.009362 8.169e-10 35366 TRUE reported textfile
2 1 TRUE
3276 rs10922098 T C T C -0.1863 -0.009 0.40011 NA FALSE FALSE FALSE
GCST006061 0.0075 0.2275 537409 outcome TRUE reported 13540_1_GPR26_GPR26
GPR26 chr1 196695521 0.008155 1.681e-115 35332 TRUE reported
textfile 2 1 TRUE
3277 rs1707652 T C T C 0.0915 0.0081 0.3102 NA FALSE FALSE FALSE
GCST006061 0.0074 0.2701 537409 outcome TRUE reported 13540_1_GPR26_GPR26
GPR26 chr3 165761011 0.008638 3.233e-26 35333 TRUE reported
textfile 2 1 TRUE
3278 rs1047286 A G A G -0.0742 -0.0013 0.24066 NA FALSE FALSE FALSE
GCST006061 0.0098 0.8921 537409 outcome TRUE reported 7911_29_SCN4B_SCN4B
SCN4B chr19 6713251 0.009296 1.436e-15 35372 TRUE reported textfile
2 1 TRUE
3279 rs10922098 T C T C -0.2044 -0.009 0.40011 NA FALSE FALSE FALSE
GCST006061 0.0075 0.2275 537409 outcome TRUE reported 7911_29_SCN4B_SCN4B
SCN4B chr1 196695521 0.008081 3.723e-141 35338 TRUE reported
textfile 2 1 TRUE
3280 rs35351085 T C T C 0.105 0.0061 0.04877 NA FALSE FALSE FALSE
GCST006061 0.022 0.7825 537409 outcome TRUE reported 7911_29_SCN4B_SCN4B
SCN4B chr1 197992799 0.018383 1.118e-08 35343 TRUE reported
textfile 2 1 TRUE
3281 rs1047286 A G A G -0.0611 -0.0013 0.24066 NA FALSE FALSE FALSE
GCST006061 0.0098 0.8921 537409 outcome TRUE reported 8990_42_ATP1B3_AT1B3
ATP1B3 chr19 6713251 0.009343 6.168e-11 35372 TRUE reported textfile
2 1 TRUE
3282 rs10922098 T C T C -0.2294 -0.009 0.40011 NA FALSE FALSE FALSE
GCST006061 0.0075 0.2275 537409 outcome TRUE reported 8990_42_ATP1B3_AT1B3
ATP1B3 chr1 196695521 0.00811 5.241e-176 35338 TRUE reported textfile
2 1 TRUE
3283 rs2686395 C T C T -0.1097 -0.0077 0.3101 NA FALSE FALSE FALSE
GCST006061 0.0074 0.2965 537409 outcome TRUE reported 8990_42_ATP1B3_AT1B3
ATP1B3 chr3 165767231 0.008617 3.993e-37 35339 TRUE reported
textfile 2 1 TRUE
3284 rs35351085 T C T C 0.1156 0.0061 0.04877 NA FALSE FALSE FALSE
GCST006061 0.022 0.7825 537409 outcome TRUE reported 8990_42_ATP1B3_AT1B3
ATP1B3 chr1 197992799 0.018477 3.941e-10 35343 TRUE reported
textfile 2 1 TRUE
3285 rs1047286 A G A G -0.0622 -0.0013 0.24066 NA FALSE FALSE FALSE
GCST006061 0.0098 0.8921 537409 outcome TRUE reported 9773_15_RNF149_RN149
RNF149 chr19 6713251 0.009365 3.103e-11 35372 TRUE reported textfile
2 1 TRUE
3286 rs10922098 T C T C -0.2055 -0.009 0.40011 NA FALSE FALSE FALSE
GCST006061 0.0075 0.2275 537409 outcome TRUE reported 9773_15_RNF149_RN149
RNF149 chr1 196695521 0.008149 2.581e-140 35338 TRUE reported
textfile 2 1 TRUE
3287 rs111696008 A G A G -0.1577 -0.0259 0.02443 NA FALSE FALSE FALSE
GCST006061 0.0401 0.519 537409 outcome TRUE reported 9773_15_RNF149_RN149
RNF149 chr2 216622986 0.026653 3.284e-09 35359 TRUE reported
textfile 2 1 TRUE
3288 rs1707652 T C T C 0.1069 0.0081 0.3102 NA FALSE FALSE FALSE
GCST006061 0.0074 0.2701 537409 outcome TRUE reported 9773_15_RNF149_RN149
RNF149 chr3 165761011 0.00864 3.702e-35 35339 TRUE reported textfile
2 1 TRUE
3289 rs35351085 T C T C 0.108 0.0061 0.04877 NA FALSE FALSE FALSE
GCST006061 0.022 0.7825 537409 outcome TRUE reported 9773_15_RNF149_RN149
RNF149 chr1 197992799 0.01854 5.699e-09 35343 TRUE reported textfile
2 1 TRUE
3290 rs1047286 A G A G -0.068 -0.0013 0.24066 NA FALSE FALSE FALSE
GCST006061 0.0098 0.8921 537409 outcome TRUE reported 14314_6_PPIL2_PPIL2
PPIL2 chr19 6713251 0.009369 3.922e-13 35372 TRUE reported textfile
2 1 TRUE
3291 rs10922098 T C T C -0.2378 -0.009 0.40011 NA FALSE FALSE FALSE
GCST006061 0.0075 0.2275 537409 outcome TRUE reported 14314_6_PPIL2_PPIL2
PPIL2 chr1 196695521 0.008134 6.842e-188 35338 TRUE reported

textfile 2 1 TRUE
3292 rs10922341 C T C T 0.047 0.0082 0.35011 NA FALSE FALSE FALSE
GCST006061 0.0076 0.2799 537409 outcome TRUE reported 14314_6_PPIL2_PPIL2
PPIL2 chr1 197973943 0.008437 2.541e-08 35343 TRUE reported
textfile 2 1 TRUE
3293 rs1451915 A C A C 0.0733 -0.0107 0.13641 NA FALSE FALSE FALSE
GCST006061 0.0124 0.3895 537409 outcome TRUE reported 14314_6_PPIL2_PPIL2
PPIL2 chr1 196058002 0.011636 2.989e-10 35335 TRUE reported
textfile 2 1 TRUE
3294 rs1707652 T C T C 0.1167 0.0081 0.3102 NA FALSE FALSE FALSE
GCST006061 0.0074 0.2701 537409 outcome TRUE reported 14314_6_PPIL2_PPIL2
PPIL2 chr3 165761011 0.008638 1.378e-41 35339 TRUE reported
textfile 2 1 TRUE
3295 rs1047286 A G A G -0.0765 -0.0013 0.24066 NA FALSE FALSE FALSE
GCST006061 0.0098 0.8921 537409 outcome TRUE reported 3418_12_CAMK1D_CAMK1D
CAMK1D chr19 6713251 0.009386 3.627e-16 35372 TRUE reported textfile
2 1 TRUE
3296 rs10922098 T C T C -0.2252 -0.009 0.40011 NA FALSE FALSE FALSE
GCST006061 0.0075 0.2275 537409 outcome TRUE reported 3418_12_CAMK1D_CAMK1D
CAMK1D chr1 196695521 0.008155 7.444e-168 35338 TRUE reported
textfile 2 1 TRUE
3297 rs1451915 A C A C 0.0674 -0.0107 0.13641 NA FALSE FALSE FALSE
GCST006061 0.0124 0.3895 537409 outcome TRUE reported 3418_12_CAMK1D_CAMK1D
CAMK1D chr1 196058002 0.011662 7.485e-09 35335 TRUE reported
textfile 2 1 TRUE
3298 rs2686395 C T C T -0.0819 -0.0077 0.3101 NA FALSE FALSE FALSE
GCST006061 0.0074 0.2965 537409 outcome TRUE reported 3418_12_CAMK1D_CAMK1D
CAMK1D chr3 165767231 0.008671 3.552e-21 35339 TRUE reported
textfile 2 1 TRUE
3299 rs704 A G A G 0.0588 -0.0155 0.49189 NA FALSE FALSE FALSE GCST006061
0.0072 0.03144 537409 outcome TRUE reported 3418_12_CAMK1D_CAMK1D CAMK1D
chr17 28367840 0.008054 2.861e-13 35360 TRUE reported textfile 2
1 TRUE
3300 rs1047286 A G A G -0.0521 -0.0013 0.24066 NA FALSE FALSE FALSE
GCST006061 0.0098 0.8921 537409 outcome TRUE reported 13681_173_CSNK2A2_CSK22
CSNK2A2 chr19 6713251 0.009304 2.145e-08 35372 TRUE reported textfile
2 1 TRUE
3301 rs1329424 G T G T -0.1852 -0.0095 0.39735 NA FALSE FALSE FALSE
GCST006061 0.0075 0.2071 537409 outcome TRUE reported 13681_173_CSNK2A2_CSK22
CSNK2A2 chr1 196677046 0.008108 1.77e-115 35338 TRUE reported
textfile 2 1 TRUE
3302 rs1707652 T C T C 0.089 0.0081 0.3102 NA FALSE FALSE FALSE
GCST006061 0.0074 0.2701 537409 outcome TRUE reported 13681_173_CSNK2A2_CSK22
CSNK2A2 chr3 165761011 0.008589 3.696e-25 35339 TRUE reported
textfile 2 1 TRUE
3303 rs1047286 A G A G -0.0699 -0.0013 0.24066 NA FALSE FALSE FALSE
GCST006061 0.0098 0.8921 537409 outcome TRUE reported 7242_14_VSTM4_CJ072
VSTM4 chr19 6713251 0.009395 1.008e-13 35372 TRUE reported textfile
2 1 TRUE
3304 rs10922098 T C T C -0.223 -0.009 0.40011 NA FALSE FALSE FALSE
GCST006061 0.0075 0.2275 537409 outcome TRUE reported 7242_14_VSTM4_CJ072
VSTM4 chr1 196695521 0.008167 3.763e-164 35338 TRUE reported
textfile 2 1 TRUE
3305 rs111696008 A G A G -0.1507 -0.0259 0.02443 NA FALSE FALSE FALSE
GCST006061 0.0401 0.519 537409 outcome TRUE reported 7242_14_VSTM4_CJ072
VSTM4 chr2 216622986 0.026737 1.737e-08 35359 TRUE reported
textfile 2 1 TRUE
3306 rs2686395 C T C T -0.1074 -0.0077 0.3101 NA FALSE FALSE FALSE
GCST006061 0.0074 0.2965 537409 outcome TRUE reported 7242_14_VSTM4_CJ072
VSTM4 chr3 165767231 0.008671 3.085e-35 35339 TRUE reported
textfile 2 1 TRUE
3307 rs1047286 A G A G -0.0524 -0.0013 0.24066 NA FALSE FALSE FALSE
GCST006061 0.0098 0.8921 537409 outcome TRUE reported 8071_114_PCDHB7_PCDB7
PCDHB7 chr19 6713251 0.009199 1.225e-08 35372 TRUE reported textfile
2 1 TRUE
3308 rs10922098 T C T C -0.1438 -0.009 0.40011 NA FALSE FALSE FALSE

GCST006061 0.0075 0.2275 537409 outcome TRUE reported 8071_114_PCDHB7_PCDB7
PCDHB7 chr1 196695521 0.008023 7.798e-72 35338 TRUE reported
textfile 2 1 TRUE
3309 rs1047286 A G A G -0.0563 -0.0013 0.24066 NA FALSE FALSE FALSE
GCST006061 0.0098 0.8921 537409 outcome TRUE reported 6405_74_PSG2_PSG2
PSG2 chr19 6713251 0.009398 2.092e-09 35372 TRUE reported textfile
2 1 TRUE
3310 rs10922098 T C T C -0.1493 -0.009 0.40011 NA FALSE FALSE FALSE
GCST006061 0.0075 0.2275 537409 outcome TRUE reported 6405_74_PSG2_PSG2
PSG2 chr1 196695521 0.008195 3.671e-74 35338 TRUE reported
textfile 2 1 TRUE
3311 rs1047286 A G A G -0.0601 -0.0013 0.24066 NA FALSE FALSE FALSE
GCST006061 0.0098 0.8921 537409 outcome TRUE reported 17772_7_UBD_UBD UBD
chr19 6713251 0.009327 1.168e-10 35372 TRUE reported textfile 2 1
TRUE
3312 rs10922098 T C T C -0.1948 -0.009 0.40011 NA FALSE FALSE FALSE
GCST006061 0.0075 0.2275 537409 outcome TRUE reported 17772_7_UBD_UBD UBD
chr1 196695521 0.008109 1.607e-127 35338 TRUE reported textfile 2
1 TRUE
3313 rs2686395 C T C T -0.0966 -0.0077 0.3101 NA FALSE FALSE FALSE
GCST006061 0.0074 0.2965 537409 outcome TRUE reported 17772_7_UBD_UBD UBD
chr3 165767231 0.008599 2.787e-29 35339 TRUE reported textfile 2
1 TRUE
3314 rs1047286 A G A G -0.0527 -0.0013 0.24066 NA FALSE FALSE FALSE
GCST006061 0.0098 0.8921 537409 outcome TRUE reported 11167_6_MTMR1_MTMR1
MTMR1 chr19 6713251 0.009348 1.727e-08 35363 TRUE reported textfile
2 1 TRUE
3315 rs10922098 T C T C -0.1949 -0.009 0.40011 NA FALSE FALSE FALSE
GCST006061 0.0075 0.2275 537409 outcome TRUE reported 11167_6_MTMR1_MTMR1
MTMR1 chr1 196695521 0.008127 4.375e-127 35329 TRUE reported
textfile 2 1 TRUE
3316 rs12478841 G A G A 0.0559 0.0049 0.21569 NA FALSE FALSE FALSE
GCST006061 0.008 0.5413 537409 outcome TRUE reported 11167_6_MTMR1_MTMR1
MTMR1 chr2 27588855 0.009634 6.544e-09 35364 TRUE reported
textfile 2 1 TRUE
3317 rs144704969 T C T C 0.1068 -0.0238 0.04536 NA FALSE FALSE FALSE
GCST006061 0.0154 0.1235 537409 outcome TRUE reported 11167_6_MTMR1_MTMR1
MTMR1 chr1 196158214 0.019084 2.188e-08 35328 TRUE reported
textfile 2 1 TRUE
3318 rs1707652 T C T C 0.0945 0.0081 0.3102 NA FALSE FALSE FALSE
GCST006061 0.0074 0.2701 537409 outcome TRUE reported 11167_6_MTMR1_MTMR1
MTMR1 chr3 165761011 0.008623 6.036e-28 35330 TRUE reported
textfile 2 1 TRUE
3319 rs1047286 A G A G -0.0606 -0.0013 0.24066 NA FALSE FALSE FALSE
GCST006061 0.0098 0.8921 537409 outcome TRUE reported 12455_48_MYNN_MYNN
MYNN chr19 6713251 0.009315 7.745e-11 35363 TRUE reported textfile
2 1 TRUE
3320 rs704 A G A G 0.0581 -0.0155 0.49189 NA FALSE FALSE FALSE GCST006061
0.0072 0.03144 537409 outcome TRUE reported 12455_48_MYNN_MYNN MYNN chr17
28367840 0.007976 3.232e-13 35351 TRUE reported textfile 2 1 TRUE
3321 rs1047286 A G A G -0.0894 -0.0013 0.24066 NA FALSE FALSE FALSE
GCST006061 0.0098 0.8921 537409 outcome TRUE reported 4436_1_ENTPD3_ENTP3
ENTPD3 chr19 6713251 0.009393 1.775e-21 35372 TRUE reported textfile
2 1 TRUE
3322 rs10922098 T C T C -0.2371 -0.009 0.40011 NA FALSE FALSE FALSE
GCST006061 0.0075 0.2275 537409 outcome TRUE reported 4436_1_ENTPD3_ENTP3
ENTPD3 chr1 196695521 0.008157 9.716e-186 35338 TRUE reported
textfile 2 1 TRUE
3323 rs2686395 C T C T -0.0935 -0.0077 0.3101 NA FALSE FALSE FALSE
GCST006061 0.0074 0.2965 537409 outcome TRUE reported 4436_1_ENTPD3_ENTP3
ENTPD3 chr3 165767231 0.008673 4.265e-27 35339 TRUE reported
textfile 2 1 TRUE
3324 rs7513100 C T C T 0.0686 0 0.14871 NA FALSE FALSE FALSE GCST006061
0.0098 0.9981 537409 outcome TRUE reported 4436_1_ENTPD3_ENTP3 ENTPD3 chr1
198065935 0.011281 1.194e-09 35343 TRUE reported textfile 2 1 TRUE
3325 rs1047286 A G A G -0.0565 -0.0013 0.24066 NA FALSE FALSE FALSE

GCST006061 0.0098 0.8921 537409 outcome TRUE reported
4423_77_BCL2L1_BCL2_like_1_protein BCL2L1 chr19 6713251 0.009414 1.95e-09
35372 TRUE reported textfile 2 1 TRUE
3326 rs10922098 T C T C -0.2111 -0.009 0.40011 NA FALSE FALSE FALSE
GCST006061 0.0075 0.2275 537409 outcome TRUE reported
4423_77_BCL2L1_BCL2_like_1_protein BCL2L1 chr1 196695521 0.008184 1.04e-146
35338 TRUE reported textfile 2 1 TRUE
3327 rs1707652 T C T C 0.1047 0.0081 0.3102 NA FALSE FALSE FALSE
GCST006061 0.0074 0.2701 537409 outcome TRUE reported
4423_77_BCL2L1_BCL2_like_1_protein BCL2L1 chr3 165761011 0.008673 1.495e-33
35339 TRUE reported textfile 2 1 TRUE
3328 rs72748420 T C T C 0.0844 0.0095 0.07417 NA FALSE FALSE FALSE
GCST006061 0.0134 0.4784 537409 outcome TRUE reported
4423_77_BCL2L1_BCL2_like_1_protein BCL2L1 chr1 196141224 0.015316 3.575e-08
35337 TRUE reported textfile 2 1 TRUE
3329 rs1047286 A G A G -0.0592 -0.0013 0.24066 NA FALSE FALSE FALSE
GCST006061 0.0098 0.8921 537409 outcome TRUE reported 6621_10_RLN2_REL2
RLN2 chr19 6713251 0.009317 2.098e-10 35372 TRUE reported textfile
2 1 TRUE
3330 rs10922098 T C T C -0.1602 -0.009 0.40011 NA FALSE FALSE FALSE
GCST006061 0.0075 0.2275 537409 outcome TRUE reported 6621_10_RLN2_REL2
RLN2 chr1 196695521 0.008113 8.795e-87 35338 TRUE reported
textfile 2 1 TRUE
3331 rs6445035 A G A G 0.1349 0.0154 0.1904 NA FALSE FALSE FALSE
GCST006061 0.0089 0.08256 537409 outcome TRUE reported 6621_10_RLN2_REL2
RLN2 chr3 165762312 0.010112 1.335e-40 35339 TRUE reported
textfile 2 1 TRUE
3332 rs1047286 A G A G -0.0557 -0.0013 0.24066 NA FALSE FALSE FALSE
GCST006061 0.0098 0.8921 537409 outcome TRUE reported
7879_12_CYP3A4_Cytochrome_P450_3A4 CYP3A4 chr19 6713251 0.009319 2.27e-09
35372 TRUE reported textfile 2 1 TRUE
3333 rs10922098 T C T C -0.1964 -0.009 0.40011 NA FALSE FALSE FALSE
GCST006061 0.0075 0.2275 537409 outcome TRUE reported
7879_12_CYP3A4_Cytochrome_P450_3A4 CYP3A4 chr1 196695521 0.008099 6.755e-130
35338 TRUE reported textfile 2 1 TRUE
3334 rs111696008 A G A G -0.1448 -0.0259 0.02443 NA FALSE FALSE FALSE
GCST006061 0.0401 0.519 537409 outcome TRUE reported
7879_12_CYP3A4_Cytochrome_P450_3A4 CYP3A4 chr2 216622986 0.026486 4.575e-08
35359 TRUE reported textfile 2 1 TRUE
3335 rs115041442 T C T C -0.1423 -0.0276 0.02598 NA FALSE FALSE FALSE
GCST006061 0.0475 0.5604 537409 outcome TRUE reported
7879_12_CYP3A4_Cytochrome_P450_3A4 CYP3A4 chr1 152992214 0.025433 2.204e-08
35362 TRUE reported textfile 2 1 TRUE
3336 rs1451915 A C A C 0.0665 -0.0107 0.13641 NA FALSE FALSE FALSE
GCST006061 0.0124 0.3895 537409 outcome TRUE reported
7879_12_CYP3A4_Cytochrome_P450_3A4 CYP3A4 chr1 196058002 0.011547 8.461e-09
35335 TRUE reported textfile 2 1 TRUE
3337 rs2686395 C T C T -0.0874 -0.0077 0.3101 NA FALSE FALSE FALSE
GCST006061 0.0074 0.2965 537409 outcome TRUE reported
7879_12_CYP3A4_Cytochrome_P450_3A4 CYP3A4 chr3 165767231 0.008594 2.716e-24
35339 TRUE reported textfile 2 1 TRUE
3338 rs1047286 A G A G -0.0672 -0.0013 0.24066 NA FALSE FALSE FALSE
GCST006061 0.0098 0.8921 537409 outcome TRUE reported 6210_100_IFNA5_IFNA5
IFNA5 chr19 6713251 0.009362 7.066e-13 35372 TRUE reported textfile
2 1 TRUE
3339 rs10922098 T C T C -0.2015 -0.009 0.40011 NA FALSE FALSE FALSE
GCST006061 0.0075 0.2275 537409 outcome TRUE reported 6210_100_IFNA5_IFNA5
IFNA5 chr1 196695521 0.008139 2.573e-135 35338 TRUE reported
textfile 2 1 TRUE
3340 rs2686395 C T C T -0.0968 -0.0077 0.3101 NA FALSE FALSE FALSE
GCST006061 0.0074 0.2965 537409 outcome TRUE reported 6210_100_IFNA5_IFNA5
IFNA5 chr3 165767231 0.008639 3.875e-29 35339 TRUE reported
textfile 2 1 TRUE
3341 rs1047286 A G A G -0.0696 -0.0013 0.24066 NA FALSE FALSE FALSE
GCST006061 0.0098 0.8921 537409 outcome TRUE reported 16748_1_BMP3_BMP_3
BMP3 chr19 6713251 0.009397 1.297e-13 35372 TRUE reported textfile

2 1 TRUE
3342 rs10922098 T C T C -0.1868 -0.009 0.40011 NA FALSE FALSE FALSE
GCST006061 0.0075 0.2275 537409 outcome TRUE reported 16748_1_BMP3_BMP_3
BMP3 chr1 196695521 0.008175 1.445e-115 35338 TRUE reported
textfile 2 1 TRUE
3343 rs6445035 A G A G 0.1081 0.0154 0.1904 NA FALSE FALSE FALSE
GCST006061 0.0089 0.08256 537409 outcome TRUE reported 16748_1_BMP3_BMP_3
BMP3 chr3 165762312 0.010205 3.201e-26 35339 TRUE reported
textfile 2 1 TRUE
3344 rs1047286 A G A G -0.0672 -0.0013 0.24066 NA FALSE FALSE FALSE
GCST006061 0.0098 0.8921 537409 outcome TRUE reported 19316_2_HSCB_HSC20
HSCB chr19 6713251 0.009422 9.856e-13 35372 TRUE reported textfile
2 1 TRUE
3345 rs111696008 A G A G -0.1575 -0.0259 0.02443 NA FALSE FALSE FALSE
GCST006061 0.0401 0.519 537409 outcome TRUE reported 19316_2_HSCB_HSC20
HSCB chr2 216622986 0.026777 4.055e-09 35359 TRUE reported
textfile 2 1 TRUE
3346 rs114009540 A G A G -0.1299 0.0322 0.03606 NA FALSE FALSE FALSE
GCST006061 0.0299 0.2809 537409 outcome TRUE reported 19316_2_HSCB_HSC20
HSCB chr1 196158413 0.021889 2.948e-09 35337 TRUE reported
textfile 2 1 TRUE
3347 rs1329424 G T G T -0.2708 -0.0095 0.39735 NA FALSE FALSE FALSE
GCST006061 0.0075 0.2071 537409 outcome TRUE reported 19316_2_HSCB_HSC20
HSCB chr1 196677046 0.008174 1e-200 35338 TRUE reported textfile
2 1 TRUE
3348 rs2686395 C T C T -0.1355 -0.0077 0.3101 NA FALSE FALSE FALSE
GCST006061 0.0074 0.2965 537409 outcome TRUE reported 19316_2_HSCB_HSC20
HSCB chr3 165767231 0.008683 6.753e-55 35339 TRUE reported
textfile 2 1 TRUE
3349 rs704 A G A G 0.0475 -0.0155 0.49189 NA FALSE FALSE FALSE GCST006061
0.0072 0.03144 537409 outcome TRUE reported 19316_2_HSCB_HSC20 HSCB chr17
28367840 0.008068 3.914e-09 35360 TRUE reported textfile 2 1 TRUE
3350 rs72748420 T C T C 0.0935 0.0095 0.07417 NA FALSE FALSE FALSE
GCST006061 0.0134 0.4784 537409 outcome TRUE reported 19316_2_HSCB_HSC20
HSCB chr1 196141224 0.015365 1.163e-09 35337 TRUE reported
textfile 2 1 TRUE
3351 rs1047286 A G A G -0.0638 -0.0013 0.24066 NA FALSE FALSE FALSE
GCST006061 0.0098 0.8921 537409 outcome TRUE reported 18927_14_RAET1G_RET1G
RAET1G chr19 6713251 0.009349 8.828e-12 35372 TRUE reported textfile
2 1 TRUE
3352 rs10922098 T C T C -0.2159 -0.009 0.40011 NA FALSE FALSE FALSE
GCST006061 0.0075 0.2275 537409 outcome TRUE reported 18927_14_RAET1G_RET1G
RAET1G chr1 196695521 0.008127 1.689e-155 35338 TRUE reported
textfile 2 1 TRUE
3353 rs4955716 C T C T 0.0478 0.003 0.32947 NA FALSE FALSE FALSE
GCST006061 0.0076 0.6983 537409 outcome TRUE reported 18927_14_RAET1G_RET1G
RAET1G chr3 167239055 0.008511 1.954e-08 35354 TRUE reported
textfile 2 1 TRUE
3354 rs704 A G A G 0.124 -0.0155 0.49189 NA FALSE FALSE FALSE GCST006061
0.0072 0.03144 537409 outcome TRUE reported 18927_14_RAET1G_RET1G RAET1G
chr17 28367840 0.007999 3.336e-54 35360 TRUE reported textfile 2
1 TRUE
3355 rs1047286 A G A G -0.0612 -0.0013 0.24066 NA FALSE FALSE FALSE
GCST006061 0.0098 0.8921 537409 outcome TRUE reported 5008_51_SOD2_Mn_SOD
SOD2 chr19 6713251 0.009566 1.577e-10 35372 TRUE reported textfile
2 1 TRUE
3356 rs10922098 T C T C -0.1662 -0.009 0.40011 NA FALSE FALSE FALSE
GCST006061 0.0075 0.2275 537409 outcome TRUE reported 5008_51_SOD2_Mn_SOD
SOD2 chr1 196695521 0.008338 2.074e-88 35338 TRUE reported
textfile 2 1 TRUE
3357 rs112305384 A G A G 0.1204 -0.0379 0.0367 NA FALSE FALSE FALSE
GCST006061 0.0259 0.1438 537409 outcome TRUE reported 5008_51_SOD2_Mn_SOD
SOD2 chr5 178125157 0.021774 3.211e-08 35364 TRUE reported
textfile 2 1 TRUE
3358 rs12975366 C T C T 0.0483 -0.0089 0.4155 NA FALSE FALSE FALSE
GCST006061 0.0081 0.2688 537409 outcome TRUE reported 5008_51_SOD2_Mn_SOD

SOD2 chr19 54255498 0.008312 6.213e-09 35370 TRUE reported
textfile 2 1 TRUE

3359 rs1707652 T C T C 0.0817 0.0081 0.3102 NA FALSE FALSE FALSE
GCST006061 0.0074 0.2701 537409 outcome TRUE reported 5008_51_SOD2_Mn_SOD
SOD2 chr3 165761011 0.00882 1.992e-20 35339 TRUE reported textfile
2 1 TRUE

3360 rs2913230 T C T C -0.1208 -0.0067 0.08206 NA FALSE FALSE FALSE
GCST006061 0.0162 0.6808 537409 outcome TRUE reported 5008_51_SOD2_Mn_SOD
SOD2 chr5 175624547 0.015071 1.099e-15 35366 TRUE reported
textfile 2 1 TRUE

3361 rs4976704 C T C T -0.1081 -0.0102 0.17782 NA FALSE FALSE FALSE
GCST006061 0.0107 0.3402 537409 outcome TRUE reported 5008_51_SOD2_Mn_SOD
SOD2 chr5 177615619 0.010887 3.101e-23 35363 TRUE reported
textfile 2 1 TRUE

3362 rs1047286 A G A G -0.0608 -0.0013 0.24066 NA FALSE FALSE FALSE
GCST006061 0.0098 0.8921 537409 outcome TRUE reported 6915_2_CD164L2_C16L2
CD164L2 chr19 6713251 0.009325 7.028e-11 35372 TRUE reported textfile
2 1 TRUE

3363 rs10922098 T C T C -0.1878 -0.009 0.40011 NA FALSE FALSE FALSE
GCST006061 0.0075 0.2275 537409 outcome TRUE reported 6915_2_CD164L2_C16L2
CD164L2 chr1 196695521 0.008106 9.28e-119 35338 TRUE reported
textfile 2 1 TRUE

3364 rs1451915 A C A C 0.0666 -0.0107 0.13641 NA FALSE FALSE FALSE
GCST006061 0.0124 0.3895 537409 outcome TRUE reported 6915_2_CD164L2_C16L2
CD164L2 chr1 196058002 0.011561 8.381e-09 35335 TRUE reported
textfile 2 1 TRUE

3365 rs1707652 T C T C 0.0821 0.0081 0.3102 NA FALSE FALSE FALSE
GCST006061 0.0074 0.2701 537409 outcome TRUE reported 6915_2_CD164L2_C16L2
CD164L2 chr3 165761011 0.0086 1.342e-21 35339 TRUE reported textfile
2 1 TRUE

3366 rs1047286 A G A G -0.0621 -0.0013 0.24066 NA FALSE FALSE FALSE
GCST006061 0.0098 0.8921 537409 outcome TRUE reported 13932_45_ARHGEF7_COOL_1
ARHGEF7 chr19 6713251 0.009357 3.211e-11 35372 TRUE reported textfile
2 1 TRUE

3367 rs10922098 T C T C -0.214 -0.009 0.40011 NA FALSE FALSE FALSE
GCST006061 0.0075 0.2275 537409 outcome TRUE reported 13932_45_ARHGEF7_COOL_1
ARHGEF7 chr1 196695521 0.008127 8.195e-153 35338 TRUE reported
textfile 2 1 TRUE

3368 rs1451915 A C A C 0.0705 -0.0107 0.13641 NA FALSE FALSE FALSE
GCST006061 0.0124 0.3895 537409 outcome TRUE reported 13932_45_ARHGEF7_COOL_1
ARHGEF7 chr1 196058002 0.011608 1.254e-09 35335 TRUE reported
textfile 2 1 TRUE

3369 rs2686395 C T C T -0.113 -0.0077 0.3101 NA FALSE FALSE FALSE
GCST006061 0.0074 0.2965 537409 outcome TRUE reported 13932_45_ARHGEF7_COOL_1
ARHGEF7 chr3 165767231 0.008622 3.033e-39 35339 TRUE reported
textfile 2 1 TRUE

3370 rs1047286 A G A G -0.0592 -0.0013 0.24066 NA FALSE FALSE FALSE
GCST006061 0.0098 0.8921 537409 outcome TRUE reported 9059_14_RAPGEF5_RPGF5
RAPGEF5 chr19 6713251 0.009361 2.543e-10 35372 TRUE reported textfile
2 1 TRUE

3371 rs10922098 T C T C -0.2324 -0.009 0.40011 NA FALSE FALSE FALSE
GCST006061 0.0075 0.2275 537409 outcome TRUE reported 9059_14_RAPGEF5_RPGF5
RAPGEF5 chr1 196695521 0.008126 6.936e-180 35338 TRUE reported
textfile 2 1 TRUE

3372 rs114009540 A G A G -0.1198 0.0322 0.03606 NA FALSE FALSE FALSE
GCST006061 0.0299 0.2809 537409 outcome TRUE reported 9059_14_RAPGEF5_RPGF5
RAPGEF5 chr1 196158413 0.021715 3.449e-08 35337 TRUE reported
textfile 2 1 TRUE

3373 rs1707652 T C T C 0.1014 0.0081 0.3102 NA FALSE FALSE FALSE
GCST006061 0.0074 0.2701 537409 outcome TRUE reported 9059_14_RAPGEF5_RPGF5
RAPGEF5 chr3 165761011 0.008638 8.062e-32 35339 TRUE reported
textfile 2 1 TRUE

3374 rs1047286 A G A G -0.0686 -0.0013 0.24066 NA FALSE FALSE FALSE
GCST006061 0.0098 0.8921 537409 outcome TRUE reported 13650_11_KCNIP1_KCIP1
KCNIP1 chr19 6713251 0.009381 2.622e-13 35372 TRUE reported textfile
2 1 TRUE

3375 rs12366520 A C A C -0.0849 -0.0053 0.12386 NA FALSE FALSE FALSE
GCST006061 0.0106 0.6159 537409 outcome TRUE reported 13650_11_KCNIP1_KCIP1
KCNIP1 chr12 7073858 0.012383 7.076e-12 35389 TRUE reported textfile
2 1 TRUE
3376 rs2686395 C T C T -0.1175 -0.0077 0.3101 NA FALSE FALSE FALSE
GCST006061 0.0074 0.2965 537409 outcome TRUE reported 13650_11_KCNIP1_KCIP1
KCNIP1 chr3 165767231 0.008655 5.526e-42 35339 TRUE reported
textfile 2 1 TRUE
3377 rs704 A G A G 0.0595 -0.0155 0.49189 NA FALSE FALSE FALSE GCST006061
0.0072 0.03144 537409 outcome TRUE reported 13650_11_KCNIP1_KCIP1 KCNIP1
chr17 28367840 0.008044 1.391e-13 35360 TRUE reported textfile 2
1 TRUE
3378 rs75215871 A C A C -0.1369 -0.0094 0.02774 NA FALSE FALSE FALSE
GCST006061 0.0396 0.8133 537409 outcome TRUE reported 13650_11_KCNIP1_KCIP1
KCNIP1 chr1 150090689 0.024733 3.112e-08 35356 TRUE reported
textfile 2 1 TRUE
3379 rs1047286 A G A G -0.0727 -0.0013 0.24066 NA FALSE FALSE FALSE
GCST006061 0.0098 0.8921 537409 outcome TRUE reported 14043_12_EEA1_EEA1
EEA1 chr19 6713251 0.009381 9.203e-15 35372 TRUE reported textfile
2 1 TRUE
3380 rs10922098 T C T C -0.2133 -0.009 0.40011 NA FALSE FALSE FALSE
GCST006061 0.0075 0.2275 537409 outcome TRUE reported 14043_12_EEA1_EEA1
EEA1 chr1 196695521 0.008153 6.886e-151 35338 TRUE reported
textfile 2 1 TRUE
3381 rs1451915 A C A C 0.068 -0.0107 0.13641 NA FALSE FALSE FALSE
GCST006061 0.0124 0.3895 537409 outcome TRUE reported 14043_12_EEA1_EEA1
EEA1 chr1 196058002 0.011639 5.143e-09 35335 TRUE reported
textfile 2 1 TRUE
3382 rs1707652 T C T C 0.1013 0.0081 0.3102 NA FALSE FALSE FALSE
GCST006061 0.0074 0.2701 537409 outcome TRUE reported 14043_12_EEA1_EEA1
EEA1 chr3 165761011 0.00865 1.111e-31 35339 TRUE reported textfile
2 1 TRUE
3383 rs1047286 A G A G -0.0506 -0.0013 0.24066 NA FALSE FALSE FALSE
GCST006061 0.0098 0.8921 537409 outcome TRUE reported 9856_22_DHPS_DHYS
DHPS chr19 6713251 0.008925 1.432e-08 35372 TRUE reported textfile
2 1 TRUE
3384 rs10922098 T C T C -0.098 -0.009 0.40011 NA FALSE FALSE FALSE
GCST006061 0.0075 0.2275 537409 outcome TRUE reported 9856_22_DHPS_DHYS
DHPS chr1 196695521 0.007788 2.63e-36 35338 TRUE reported
textfile 2 1 TRUE
3385 rs1047286 A G A G -0.0703 -0.0013 0.24066 NA FALSE FALSE FALSE
GCST006061 0.0098 0.8921 537409 outcome TRUE reported 13506_10_DUS2_DUS2L
DUS2 chr19 6713251 0.009288 3.757e-14 35366 TRUE reported textfile
2 1 TRUE
3386 rs10922098 T C T C -0.2015 -0.009 0.40011 NA FALSE FALSE FALSE
GCST006061 0.0075 0.2275 537409 outcome TRUE reported 13506_10_DUS2_DUS2L
DUS2 chr1 196695521 0.008081 3.147e-137 35332 TRUE reported
textfile 2 1 TRUE
3387 rs2686395 C T C T -0.0852 -0.0077 0.3101 NA FALSE FALSE FALSE
GCST006061 0.0074 0.2965 537409 outcome TRUE reported 13506_10_DUS2_DUS2L
DUS2 chr3 165767231 0.008572 2.819e-23 35333 TRUE reported
textfile 2 1 TRUE
3388 rs1047286 A G A G -0.0553 -0.0013 0.24066 NA FALSE FALSE FALSE
GCST006061 0.0098 0.8921 537409 outcome TRUE reported 7846_44_PSG11_PSG11
PSG11 chr19 6713251 0.009274 2.48e-09 35372 TRUE reported textfile
2 1 TRUE
3389 rs10922098 T C T C -0.1706 -0.009 0.40011 NA FALSE FALSE FALSE
GCST006061 0.0075 0.2275 537409 outcome TRUE reported 7846_44_PSG11_PSG11
PSG11 chr1 196695521 0.008067 2.865e-99 35338 TRUE reported
textfile 2 1 TRUE
3390 rs2686395 C T C T -0.0894 -0.0077 0.3101 NA FALSE FALSE FALSE
GCST006061 0.0074 0.2965 537409 outcome TRUE reported 7846_44_PSG11_PSG11
PSG11 chr3 165767231 0.008554 1.446e-25 35339 TRUE reported
textfile 2 1 TRUE
3391 rs1047286 A G A G -0.0773 -0.0013 0.24066 NA FALSE FALSE FALSE
GCST006061 0.0098 0.8921 537409 outcome TRUE reported 3873_51_TPO_TMA TPO

chr19 6713251 0.009335 1.229e-16 35372 TRUE reported textfile 2 1
TRUE

3392 rs10922098 T C T C -0.2084 -0.009 0.40011 NA FALSE FALSE FALSE
GCST006061 0.0075 0.2275 537409 outcome TRUE reported 3873_51_TPO_TMA TPO
chr1 196695521 0.008121 3.198e-145 35338 TRUE reported textfile 2
1 TRUE

3393 rs1451915 A C A C 0.0652 -0.0107 0.13641 NA FALSE FALSE FALSE
GCST006061 0.0124 0.3895 537409 outcome TRUE reported 3873_51_TPO_TMA TPO
chr1 196058002 0.011603 1.918e-08 35335 TRUE reported textfile 2
1 TRUE

3394 rs2686395 C T C T -0.0906 -0.0077 0.3101 NA FALSE FALSE FALSE
GCST006061 0.0074 0.2965 537409 outcome TRUE reported 3873_51_TPO_TMA TPO
chr3 165767231 0.008624 8.093e-26 35339 TRUE reported textfile 2
1 TRUE

3395 rs35209896 A C A C 0.0931 0.0267 0.09263 NA FALSE FALSE FALSE
GCST006061 0.0355 0.4521 537409 outcome TRUE reported 3873_51_TPO_TMA TPO
chr6 32519009 0.013669 9.706e-12 35439 TRUE reported textfile 2
1 TRUE

3396 rs1047286 A G A G -0.0637 -0.0013 0.24066 NA FALSE FALSE FALSE
GCST006061 0.0098 0.8921 537409 outcome TRUE reported 14024_196_EDA_EDA EDA
chr19 6713251 0.009352 9.687e-12 35372 TRUE reported textfile 2 1
TRUE

3397 rs1451915 A C A C 0.0708 -0.0107 0.13641 NA FALSE FALSE FALSE
GCST006061 0.0124 0.3895 537409 outcome TRUE reported 14024_196_EDA_EDA EDA
chr1 196058002 0.0116 1.039e-09 35335 TRUE reported textfile 2 1
TRUE

3398 rs1707652 T C T C 0.0894 0.0081 0.3102 NA FALSE FALSE FALSE
GCST006061 0.0074 0.2701 537409 outcome TRUE reported 14024_196_EDA_EDA EDA
chr3 165761011 0.008622 3.437e-25 35339 TRUE reported textfile 2
1 TRUE

3399 rs1047286 A G A G -0.0558 -0.0013 0.24066 NA FALSE FALSE FALSE
GCST006061 0.0098 0.8921 537409 outcome TRUE reported 13055_53_PSD_PSD1 PSD
chr19 6713251 0.009501 4.274e-09 35363 TRUE reported textfile 2 1
TRUE

3400 rs10922098 T C T C -0.2453 -0.009 0.40011 NA FALSE FALSE FALSE
GCST006061 0.0075 0.2275 537409 outcome TRUE reported 13055_53_PSD_PSD1 PSD
chr1 196695521 0.008244 1.473e-194 35329 TRUE reported textfile 2
1 TRUE

3401 rs111696008 A G A G -0.1708 -0.0259 0.02443 NA FALSE FALSE FALSE
GCST006061 0.0401 0.519 537409 outcome TRUE reported 13055_53_PSD_PSD1 PSD
chr2 216622986 0.027005 2.537e-10 35350 TRUE reported textfile 2
1 TRUE

3402 rs1451915 A C A C 0.069 -0.0107 0.13641 NA FALSE FALSE FALSE
GCST006061 0.0124 0.3895 537409 outcome TRUE reported 13055_53_PSD_PSD1 PSD
chr1 196058002 0.011811 5.15e-09 35326 TRUE reported textfile 2
1 TRUE

3403 rs1707652 T C T C 0.1144 0.0081 0.3102 NA FALSE FALSE FALSE
GCST006061 0.0074 0.2701 537409 outcome TRUE reported 13055_53_PSD_PSD1 PSD
chr3 165761011 0.008753 4.866e-39 35330 TRUE reported textfile 2
1 TRUE

3404 rs2073333 T C T C 0.1798 0.0103 0.20754 NA FALSE FALSE FALSE
GCST006061 0.0081 0.2059 537409 outcome TRUE reported 13055_53_PSD_PSD1 PSD
chr14 94378225 0.010024 5.991e-72 35362 TRUE reported textfile 2
1 TRUE

3405 rs429358 C T C T 0.1533 0.005 0.17407 NA FALSE FALSE FALSE
GCST006061 0.0103 0.6319 537409 outcome TRUE reported 13055_53_PSD_PSD1 PSD
chr19 44908684 0.01081 1.205e-45 35359 TRUE reported textfile 2 1
TRUE

3406 rs704 A G A G -0.0811 -0.0155 0.49189 NA FALSE FALSE FALSE GCST006061
0.0072 0.03144 537409 outcome TRUE reported 13055_53_PSD_PSD1 PSD chr17
28367840 0.008135 2.084e-23 35351 TRUE reported textfile 2 1 TRUE

3407 rs1047286 A G A G -0.0664 -0.0013 0.24066 NA FALSE FALSE FALSE
GCST006061 0.0098 0.8921 537409 outcome TRUE reported 3837_6_ZAP70_ZAP70
ZAP70 chr19 6713251 0.009341 1.175e-12 35372 TRUE reported textfile
2 1 TRUE

3408 rs10922098 T C T C -0.2267 -0.009 0.40011 NA FALSE FALSE FALSE

GCST006061 0.0075 0.2275 537409 outcome TRUE reported 3837_6_ZAP70_ZAP70
ZAP70 chr1 196695521 0.008105 3.562e-172 35338 TRUE reported
textfile 2 1 TRUE
3409 rs6445035 A G A G 0.1233 0.0154 0.1904 NA FALSE FALSE FALSE
GCST006061 0.0089 0.08256 537409 outcome TRUE reported 3837_6_ZAP70_ZAP70
ZAP70 chr3 165762312 0.010147 5.631e-34 35339 TRUE reported
textfile 2 1 TRUE
3410 rs1047286 A G A G -0.0583 -0.0013 0.24066 NA FALSE FALSE FALSE
GCST006061 0.0098 0.8921 537409 outcome TRUE reported 18399_1_AKAP7_AKA7A
AKAP7 chr19 6713251 0.009353 4.575e-10 35372 TRUE reported textfile
2 1 TRUE
3411 rs10922098 T C T C -0.2155 -0.009 0.40011 NA FALSE FALSE FALSE
GCST006061 0.0075 0.2275 537409 outcome TRUE reported 18399_1_AKAP7_AKA7A
AKAP7 chr1 196695521 0.008127 6.045e-155 35338 TRUE reported
textfile 2 1 TRUE
3412 rs1451915 A C A C 0.0692 -0.0107 0.13641 NA FALSE FALSE FALSE
GCST006061 0.0124 0.3895 537409 outcome TRUE reported 18399_1_AKAP7_AKA7A
AKAP7 chr1 196058002 0.011613 2.54e-09 35335 TRUE reported
textfile 2 1 TRUE
3413 rs1707652 T C T C 0.1082 0.0081 0.3102 NA FALSE FALSE FALSE
GCST006061 0.0074 0.2701 537409 outcome TRUE reported 18399_1_AKAP7_AKA7A
AKAP7 chr3 165761011 0.008622 4.046e-36 35339 TRUE reported
textfile 2 1 TRUE
3414 rs7513100 C T C T 0.0616 0 0.14871 NA FALSE FALSE FALSE GCST006061
0.0098 0.9981 537409 outcome TRUE reported 18399_1_AKAP7_AKA7A AKAP7 chr1
198065935 0.011228 4.109e-08 35343 TRUE reported textfile 2 1 TRUE
3415 rs1047286 A G A G -0.0565 -0.0013 0.24066 NA FALSE FALSE FALSE
GCST006061 0.0098 0.8921 537409 outcome TRUE reported 6969_14_WFDC5_WFDC5
WFDC5 chr19 6713251 0.009287 1.174e-09 35372 TRUE reported textfile
2 1 TRUE
3416 rs10922098 T C T C -0.1744 -0.009 0.40011 NA FALSE FALSE FALSE
GCST006061 0.0075 0.2275 537409 outcome TRUE reported 6969_14_WFDC5_WFDC5
WFDC5 chr1 196695521 0.008088 4.123e-103 35338 TRUE reported
textfile 2 1 TRUE
3417 rs1707652 T C T C 0.0833 0.0081 0.3102 NA FALSE FALSE FALSE
GCST006061 0.0074 0.2701 537409 outcome TRUE reported 6969_14_WFDC5_WFDC5
WFDC5 chr3 165761011 0.008572 2.54e-22 35339 TRUE reported
textfile 2 1 TRUE
3418 rs1047286 A G A G -0.0643 -0.0013 0.24066 NA FALSE FALSE FALSE
GCST006061 0.0098 0.8921 537409 outcome TRUE reported 8664_36_PKDCC_PKDCC
PKDCC chr19 6713251 0.009429 9.128e-12 35372 TRUE reported textfile
2 1 TRUE
3419 rs10922098 T C T C -0.2139 -0.009 0.40011 NA FALSE FALSE FALSE
GCST006061 0.0075 0.2275 537409 outcome TRUE reported 8664_36_PKDCC_PKDCC
PKDCC chr1 196695521 0.008201 5.576e-150 35338 TRUE reported
textfile 2 1 TRUE
3420 rs114009540 A G A G -0.1264 0.0322 0.03606 NA FALSE FALSE FALSE
GCST006061 0.0299 0.2809 537409 outcome TRUE reported 8664_36_PKDCC_PKDCC
PKDCC chr1 196158413 0.021867 7.448e-09 35337 TRUE reported
textfile 2 1 TRUE
3421 rs11902166 A C A C 0.0802 0.0068 0.24839 NA FALSE FALSE FALSE
GCST006061 0.0089 0.4444 537409 outcome TRUE reported 8664_36_PKDCC_PKDCC
PKDCC chr2 42055689 0.009357 1.028e-17 35362 TRUE reported
textfile 2 1 TRUE
3422 rs12478841 G A G A 0.0532 0.0049 0.21569 NA FALSE FALSE FALSE
GCST006061 0.008 0.5413 537409 outcome TRUE reported 8664_36_PKDCC_PKDCC
PKDCC chr2 27588855 0.009726 4.498e-08 35373 TRUE reported
textfile 2 1 TRUE
3423 rs1707652 T C T C 0.1018 0.0081 0.3102 NA FALSE FALSE FALSE
GCST006061 0.0074 0.2701 537409 outcome TRUE reported 8664_36_PKDCC_PKDCC
PKDCC chr3 165761011 0.008703 1.327e-31 35339 TRUE reported
textfile 2 1 TRUE
3424 rs78947001 A G A G 0.1027 0.0094 0.06051 NA FALSE FALSE FALSE
GCST006061 0.0176 0.5915 537409 outcome TRUE reported 8664_36_PKDCC_PKDCC
PKDCC chr2 42069912 0.017166 2.195e-09 35362 TRUE reported
textfile 2 1 TRUE

3425 rs1047286 A G A G -0.0742 -0.0013 0.24066 NA FALSE FALSE FALSE GCST006061 0.0098 0.8921 537409 outcome TRUE reported 2770_51_CCL1_I_309 CCL1 chr19 6713251 0.009485 5.16e-15 35372 TRUE reported textfile 2 1 TRUE

3426 rs10801660 A G A G 0.0771 -0.0119 0.11574 NA FALSE FALSE FALSE GCST006061 0.0108 0.2735 537409 outcome TRUE reported 2770_51_CCL1_I_309 CCL1 chr1 198272225 0.012685 1.216e-09 35345 TRUE reported textfile 2 1 TRUE

3427 rs111696008 A G A G -0.1487 -0.0259 0.02443 NA FALSE FALSE FALSE GCST006061 0.0401 0.519 537409 outcome TRUE reported 2770_51_CCL1_I_309 CCL1 chr2 216622986 0.026985 3.58e-08 35359 TRUE reported textfile 2 1 TRUE

3428 rs114558472 T C T C -0.0774 0.0072 0.09165 NA FALSE FALSE FALSE GCST006061 0.0139 0.6063 537409 outcome TRUE reported 2770_51_CCL1_I_309 CCL1 chr1 196140161 0.014066 3.742e-08 35337 TRUE reported textfile 2 1 TRUE

3429 rs1329424 G T G T -0.2767 -0.0095 0.39735 NA FALSE FALSE FALSE GCST006061 0.0075 0.2071 537409 outcome TRUE reported 2770_51_CCL1_I_309 CCL1 chr1 196677046 0.008236 1e-200 35338 TRUE reported textfile 2 1 TRUE

3430 rs13469 T C T C -0.0459 -0.0177 0.45244 NA FALSE FALSE FALSE GCST006061 0.0071 0.01333 537409 outcome TRUE reported 2770_51_CCL1_I_309 CCL1 chr17 28349109 0.008154 1.81e-08 35360 TRUE reported textfile 2 1 TRUE

3431 rs1707652 T C T C 0.1213 0.0081 0.3102 NA FALSE FALSE FALSE GCST006061 0.0074 0.2701 537409 outcome TRUE reported 2770_51_CCL1_I_309 CCL1 chr3 165761011 0.008746 9.733e-44 35339 TRUE reported textfile 2 1 TRUE

3432 rs687628 A G A G 0.0492 0.0132 0.29696 NA FALSE FALSE FALSE GCST006061 0.0078 0.09099 537409 outcome TRUE reported 2770_51_CCL1_I_309 CCL1 chr1 56911906 0.008889 3.111e-08 35369 TRUE reported textfile 2 1 TRUE

3433 rs72748420 T C T C 0.0979 0.0095 0.07417 NA FALSE FALSE FALSE GCST006061 0.0134 0.4784 537409 outcome TRUE reported 2770_51_CCL1_I_309 CCL1 chr1 196141224 0.015479 2.537e-10 35337 TRUE reported textfile 2 1 TRUE

3434 rs1047286 A G A G -0.0652 -0.0013 0.24066 NA FALSE FALSE FALSE GCST006061 0.0098 0.8921 537409 outcome TRUE reported 16802_31_OCIAD1_OCAD1 OCIAD1 chr19 6713251 0.009443 5.036e-12 35372 TRUE reported textfile 2 1 TRUE

3435 rs10922098 T C T C -0.2331 -0.009 0.40011 NA FALSE FALSE FALSE GCST006061 0.0075 0.2275 537409 outcome TRUE reported 16802_31_OCIAD1_OCAD1 OCIAD1 chr1 196695521 0.0082 9.744e-178 35338 TRUE reported textfile 2 1 TRUE

3436 rs111696008 A G A G -0.1656 -0.0259 0.02443 NA FALSE FALSE FALSE GCST006061 0.0401 0.519 537409 outcome TRUE reported 16802_31_OCIAD1_OCAD1 OCIAD1 chr2 216622986 0.026843 6.865e-10 35359 TRUE reported textfile 2 1 TRUE

3437 rs114009540 A G A G -0.1203 0.0322 0.03606 NA FALSE FALSE FALSE GCST006061 0.0299 0.2809 537409 outcome TRUE reported 16802_31_OCIAD1_OCAD1 OCIAD1 chr1 196158413 0.021907 3.986e-08 35337 TRUE reported textfile 2 1 TRUE

3438 rs2686395 C T C T -0.1179 -0.0077 0.3101 NA FALSE FALSE FALSE GCST006061 0.0074 0.2965 537409 outcome TRUE reported 16802_31_OCIAD1_OCAD1 OCIAD1 chr3 165767231 0.008703 8.285e-42 35339 TRUE reported textfile 2 1 TRUE

3439 rs704 A G A G 0.1056 -0.0155 0.49189 NA FALSE FALSE FALSE GCST006061 0.0072 0.03144 537409 outcome TRUE reported 16802_31_OCIAD1_OCAD1 OCIAD1 chr17 28367840 0.008083 5.23e-39 35360 TRUE reported textfile 2 1 TRUE

3440 rs1047286 A G A G -0.0617 -0.0013 0.24066 NA FALSE FALSE FALSE GCST006061 0.0098 0.8921 537409 outcome TRUE reported 14645_253_GSTA4_GSTA4 GSTA4 chr19 6713251 0.009438 6.27e-11 35372 TRUE reported textfile 2 1 TRUE

3441 rs111696008 A G A G -0.1563 -0.0259 0.02443 NA FALSE FALSE FALSE GCST006061 0.0401 0.519 537409 outcome TRUE reported 14645_253_GSTA4_GSTA4 GSTA4 chr2 216622986 0.026822 5.635e-09 35359 TRUE reported

```
     textfile    2   1   TRUE
3442 rs114009540 A   G   A   G   -0.1217 0.0322  0.03606 NA  FALSE   FALSE   FALSE
     GCST006061  0.0299  0.2809  537409  outcome TRUE    reported    14645_253_GSTA4_GSTA4
     GSTA4   chr1    196158413   0.021916    2.806e-08   35337   TRUE    reported
     textfile    2   1   TRUE
3443 rs12629202  A   C   A   C   0.0481  0.0052  0.31116 NA  FALSE   FALSE   FALSE
     GCST006061  0.0081  0.5213  537409  outcome TRUE    reported    14645_253_GSTA4_GSTA4
     GSTA4   chr3    167083477   0.008736    3.673e-08   35354   TRUE    reported
     textfile    2   1   TRUE
3444 rs1329424   G   T   G   T   -0.2568 -0.0095 0.39735 NA  FALSE   FALSE   FALSE
     GCST006061  0.0075  0.2071  537409  outcome TRUE    reported    14645_253_GSTA4_GSTA4
     GSTA4   chr1    196677046   0.008198    1e-200  35338   TRUE    reported    textfile
     2   1   TRUE
3445 rs1707652   T   C   T   C   0.124   0.0081  0.3102  NA  FALSE   FALSE   FALSE
     GCST006061  0.0074  0.2701  537409  outcome TRUE    reported    14645_253_GSTA4_GSTA4
     GSTA4   chr3    165761011   0.008703    4.629e-46   35339   TRUE    reported
     textfile    2   1   TRUE
3446 rs72748420  T   C   T   C   0.0859  0.0095  0.07417 NA  FALSE   FALSE   FALSE
     GCST006061  0.0134  0.4784  537409  outcome TRUE    reported    14645_253_GSTA4_GSTA4
     GSTA4   chr1    196141224   0.015379    2.328e-08   35337   TRUE    reported
     textfile    2   1   TRUE
3447 rs1047286   A   G   A   G   -0.0513 -0.0013 0.24066 NA  FALSE   FALSE   FALSE
     GCST006061  0.0098  0.8921  537409  outcome TRUE    reported    7751_121_SFRP2_SARP_1
     SFRP2   chr19   6713251 0.00936 4.232e-08   35372   TRUE    reported    textfile    2
     1   TRUE
3448 rs10922098  T   C   T   C   -0.2306 -0.009  0.40011 NA  FALSE   FALSE   FALSE
     GCST006061  0.0075  0.2275  537409  outcome TRUE    reported    7751_121_SFRP2_SARP_1
     SFRP2   chr1    196695521   0.008125    3.288e-177  35338   TRUE    reported
     textfile    2   1   TRUE
3449 rs1451915   A   C   A   C   0.0722  -0.0107 0.13641 NA  FALSE   FALSE   FALSE
     GCST006061  0.0124  0.3895  537409  outcome TRUE    reported    7751_121_SFRP2_SARP_1
     SFRP2   chr1    196058002   0.011628    5.33e-10    35335   TRUE    reported
     textfile    2   1   TRUE
3450 rs1707652   T   C   T   C   0.1015  0.0081  0.3102  NA  FALSE   FALSE   FALSE
     GCST006061  0.0074  0.2701  537409  outcome TRUE    reported    7751_121_SFRP2_SARP_1
     SFRP2   chr3    165761011   0.008636    6.852e-32   35339   TRUE    reported
     textfile    2   1   TRUE
3451 rs704   A   G   A   G   0.0608  -0.0155 0.49189 NA  FALSE   FALSE   FALSE   GCST006061
     0.0072  0.03144 537409  outcome TRUE    reported    7751_121_SFRP2_SARP_1   SFRP2
     chr17   28367840    0.008022    3.484e-14   35360   TRUE    reported    textfile    2
     1   TRUE
3452 rs1047286   A   G   A   G   -0.0734 -0.0013 0.24066 NA  FALSE   FALSE   FALSE
     GCST006061  0.0098  0.8921  537409  outcome TRUE    reported
     2783_18_CCL3L1_LD78_beta    CCL3L1  chr19   6713251 0.009427    6.927e-15   35372
     TRUE    reported    textfile    2   1   TRUE
3453 rs10922098  T   C   T   C   -0.2476 -0.009  0.40011 NA  FALSE   FALSE   FALSE
     GCST006061  0.0075  0.2275  537409  outcome TRUE    reported
     2783_18_CCL3L1_LD78_beta    CCL3L1  chr1    196695521   0.008188    1e-200  35338
     TRUE    reported    textfile    2   1   TRUE
3454 rs2686395   C   T   C   T   -0.1075 -0.0077 0.3101  NA  FALSE   FALSE   FALSE
     GCST006061  0.0074  0.2965  537409  outcome TRUE    reported
     2783_18_CCL3L1_LD78_beta    CCL3L1  chr3    165767231   0.0087  4.482e-35   35339
     TRUE    reported    textfile    2   1   TRUE
3455 rs72748420  T   C   T   C   0.0858  0.0095  0.07417 NA  FALSE   FALSE   FALSE
     GCST006061  0.0134  0.4784  537409  outcome TRUE    reported
     2783_18_CCL3L1_LD78_beta    CCL3L1  chr1    196141224   0.015369    2.371e-08   35337
     TRUE    reported    textfile    2   1   TRUE
3456 rs854469    G   A   G   A   0.0859  0.0059  0.11349 NA  FALSE   FALSE   FALSE
     GCST006061  0.0104  0.5713  537409  outcome TRUE    reported
     2783_18_CCL3L1_LD78_beta    CCL3L1  chr17   36062001    0.012717    1.432e-11   35370
     TRUE    reported    textfile    2   1   TRUE
3457 rs1047286   A   G   A   G   -0.0635 -0.0013 0.24066 NA  FALSE   FALSE   FALSE
     GCST006061  0.0098  0.8921  537409  outcome TRUE    reported    17516_7_RAB3A_Rab3A
     RAB3A   chr19   6713251 0.009282    7.849e-12   35372   TRUE    reported    textfile
     2   1   TRUE
3458 rs10922098  T   C   T   C   -0.2017 -0.009  0.40011 NA  FALSE   FALSE   FALSE
```

GCST006061 0.0075 0.2275 537409 outcome TRUE reported 17516_7_RAB3A_Rab3A
RAB3A chr1 196695521 0.008063 4.192e-138 35338 TRUE reported
textfile 2 1 TRUE
3459 rs1707652 T C T C 0.0769 0.0081 0.3102 NA FALSE FALSE FALSE
GCST006061 0.0074 0.2701 537409 outcome TRUE reported 17516_7_RAB3A_Rab3A
RAB3A chr3 165761011 0.008562 2.659e-19 35339 TRUE reported
textfile 2 1 TRUE
3460 rs1047286 A G A G -0.0646 -0.0013 0.24066 NA FALSE FALSE FALSE
GCST006061 0.0098 0.8921 537409 outcome TRUE reported
2636_10_LTBR_Lymphotoxin_b_R LTBR chr19 6713251 0.009461 8.624e-12 35372
TRUE reported textfile 2 1 TRUE
3461 rs10801660 A G A G 0.0793 -0.0119 0.11574 NA FALSE FALSE FALSE
GCST006061 0.0108 0.2735 537409 outcome TRUE reported
2636_10_LTBR_Lymphotoxin_b_R LTBR chr1 198272225 0.01266 3.754e-10 35345
TRUE reported textfile 2 1 TRUE
3462 rs1329424 G T G T -0.2993 -0.0095 0.39735 NA FALSE FALSE FALSE
GCST006061 0.0075 0.2071 537409 outcome TRUE reported
2636_10_LTBR_Lymphotoxin_b_R LTBR chr1 196677046 0.008202 1e-200 35338
TRUE reported textfile 2 1 TRUE
3463 rs1707652 T C T C 0.124 0.0081 0.3102 NA FALSE FALSE FALSE
GCST006061 0.0074 0.2701 537409 outcome TRUE reported
2636_10_LTBR_Lymphotoxin_b_R LTBR chr3 165761011 0.008725 7.724e-46
35339 TRUE reported textfile 2 1 TRUE
3464 rs2161196 C T C T -0.0796 -0.0073 0.08129 NA FALSE FALSE FALSE
GCST006061 0.0124 0.5591 537409 outcome TRUE reported
2636_10_LTBR_Lymphotoxin_b_R LTBR chr5 115304767 0.014561 4.585e-08
35350 TRUE reported textfile 2 1 TRUE
3465 rs72748420 T C T C 0.105 0.0095 0.07417 NA FALSE FALSE FALSE
GCST006061 0.0134 0.4784 537409 outcome TRUE reported
2636_10_LTBR_Lymphotoxin_b_R LTBR chr1 196141224 0.01545 1.074e-11 35337
TRUE reported textfile 2 1 TRUE
3466 rs1047286 A G A G -0.073 -0.0013 0.24066 NA FALSE FALSE FALSE
GCST006061 0.0098 0.8921 537409 outcome TRUE reported 14245_195_SNX7_SNX7
SNX7 chr19 6713251 0.009405 8.378e-15 35372 TRUE reported textfile
2 1 TRUE
3467 rs10922098 T C T C -0.2241 -0.009 0.40011 NA FALSE FALSE FALSE
GCST006061 0.0075 0.2275 537409 outcome TRUE reported 14245_195_SNX7_SNX7
SNX7 chr1 196695521 0.00817 1.256e-165 35338 TRUE reported textfile
2 1 TRUE
3468 rs114009540 A G A G -0.1195 0.0322 0.03606 NA FALSE FALSE FALSE
GCST006061 0.0299 0.2809 537409 outcome TRUE reported 14245_195_SNX7_SNX7
SNX7 chr1 196158413 0.021812 4.284e-08 35337 TRUE reported
textfile 2 1 TRUE
3469 rs1707652 T C T C 0.111 0.0081 0.3102 NA FALSE FALSE FALSE
GCST006061 0.0074 0.2701 537409 outcome TRUE reported 14245_195_SNX7_SNX7
SNX7 chr3 165761011 0.008677 1.812e-37 35339 TRUE reported
textfile 2 1 TRUE
3470 rs1047286 A G A G -0.0576 -0.0013 0.24066 NA FALSE FALSE FALSE
GCST006061 0.0098 0.8921 537409 outcome TRUE reported 13131_5_HK1_HXK1 HK1
chr19 6713251 0.009316 6.297e-10 35363 TRUE reported textfile 2 1
TRUE
3471 rs10922098 T C T C -0.2437 -0.009 0.40011 NA FALSE FALSE FALSE
GCST006061 0.0075 0.2275 537409 outcome TRUE reported 13131_5_HK1_HXK1 HK1
chr1 196695521 0.008085 1.353e-199 35329 TRUE reported textfile 2
1 TRUE
3472 rs144704969 T C T C 0.1051 -0.0238 0.04536 NA FALSE FALSE FALSE
GCST006061 0.0154 0.1235 537409 outcome TRUE reported 13131_5_HK1_HXK1 HK1
chr1 196158214 0.019057 3.486e-08 35328 TRUE reported textfile 2
1 TRUE
3473 rs2686395 C T C T -0.1055 -0.0077 0.3101 NA FALSE FALSE FALSE
GCST006061 0.0074 0.2965 537409 outcome TRUE reported 13131_5_HK1_HXK1 HK1
chr3 165767231 0.008589 1.108e-34 35330 TRUE reported textfile 2
1 TRUE
3474 rs1047286 A G A G -0.0661 -0.0013 0.24066 NA FALSE FALSE FALSE
GCST006061 0.0098 0.8921 537409 outcome TRUE reported 18194_18_FABP9_FABP9
FABP9 chr19 6713251 0.009385 1.879e-12 35372 TRUE reported textfile

2 1 TRUE
3475 rs114009540 A G A G -0.1337 0.0322 0.03606 NA FALSE FALSE FALSE
GCST006061 0.0299 0.2809 537409 outcome TRUE reported 18194_18_FABP9_FABP9
FABP9 chr1 196158413 0.021823 8.978e-10 35337 TRUE reported
textfile 2 1 TRUE
3476 rs1329424 G T G T -0.2621 -0.0095 0.39735 NA FALSE FALSE FALSE
GCST006061 0.0075 0.2071 537409 outcome TRUE reported 18194_18_FABP9_FABP9
FABP9 chr1 196677046 0.008155 1e-200 35338 TRUE reported textfile
2 1 TRUE
3477 rs2686395 C T C T -0.127 -0.0077 0.3101 NA FALSE FALSE FALSE
GCST006061 0.0074 0.2965 537409 outcome TRUE reported 18194_18_FABP9_FABP9
FABP9 chr3 165767231 0.008654 9.352e-49 35339 TRUE reported
textfile 2 1 TRUE
3478 rs3926704 A G A G 0.0498 7e-04 0.32404 NA FALSE FALSE FALSE
GCST006061 0.0078 0.9316 537409 outcome TRUE reported 18194_18_FABP9_FABP9
FABP9 chr3 167076696 0.008588 6.693e-09 35354 TRUE reported
textfile 2 1 TRUE
3479 rs72748420 T C T C 0.0942 0.0095 0.07417 NA FALSE FALSE FALSE
GCST006061 0.0134 0.4784 537409 outcome TRUE reported 18194_18_FABP9_FABP9
FABP9 chr1 196141224 0.015316 7.737e-10 35337 TRUE reported
textfile 2 1 TRUE
3480 rs1047286 A G A G -0.0564 -0.0013 0.24066 NA FALSE FALSE FALSE
GCST006061 0.0098 0.8921 537409 outcome TRUE reported 7081_2_BTN3A1_BT3A1
BTN3A1 chr19 6713251 0.009232 9.998e-10 35372 TRUE reported textfile
2 1 TRUE
3481 rs10922098 T C T C -0.1381 -0.009 0.40011 NA FALSE FALSE FALSE
GCST006061 0.0075 0.2275 537409 outcome TRUE reported 7081_2_BTN3A1_BT3A1
BTN3A1 chr1 196695521 0.00805 5.686e-66 35338 TRUE reported textfile
2 1 TRUE
3482 rs117841447 T C T C -0.0893 -0.012 0.06694 NA FALSE FALSE FALSE
GCST006061 0.016 0.4536 537409 outcome TRUE reported 7081_2_BTN3A1_BT3A1
BTN3A1 chr12 24415521 0.015998 2.38e-08 35361 TRUE reported
textfile 2 1 TRUE
3483 rs2686395 C T C T -0.0595 -0.0077 0.3101 NA FALSE FALSE FALSE
GCST006061 0.0074 0.2965 537409 outcome TRUE reported 7081_2_BTN3A1_BT3A1
BTN3A1 chr3 165767231 0.008522 2.911e-12 35339 TRUE reported
textfile 2 1 TRUE
3484 rs1047286 A G A G -0.0572 -0.0013 0.24066 NA FALSE FALSE FALSE
GCST006061 0.0098 0.8921 537409 outcome TRUE reported 11118_107_RAB17_RAB17
RAB17 chr19 6713251 0.009402 1.174e-09 35363 TRUE reported textfile
2 1 TRUE
3485 rs10801660 A G A G 0.0796 -0.0119 0.11574 NA FALSE FALSE FALSE
GCST006061 0.0108 0.2735 537409 outcome TRUE reported 11118_107_RAB17_RAB17
RAB17 chr1 198272225 0.01258 2.492e-10 35336 TRUE reported textfile
2 1 TRUE
3486 rs111696008 A G A G -0.1533 -0.0259 0.02443 NA FALSE FALSE FALSE
GCST006061 0.0401 0.519 537409 outcome TRUE reported 11118_107_RAB17_RAB17
RAB17 chr2 216622986 0.026747 9.951e-09 35350 TRUE reported
textfile 2 1 TRUE
3487 rs1329424 G T G T -0.2918 -0.0095 0.39735 NA FALSE FALSE FALSE
GCST006061 0.0075 0.2071 537409 outcome TRUE reported 11118_107_RAB17_RAB17
RAB17 chr1 196677046 0.008156 1e-200 35329 TRUE reported textfile
2 1 TRUE
3488 rs1707652 T C T C 0.1323 0.0081 0.3102 NA FALSE FALSE FALSE
GCST006061 0.0074 0.2701 537409 outcome TRUE reported 11118_107_RAB17_RAB17
RAB17 chr3 165761011 0.008668 1.331e-52 35330 TRUE reported
textfile 2 1 TRUE
3489 rs72748420 T C T C 0.1015 0.0095 0.07417 NA FALSE FALSE FALSE
GCST006061 0.0134 0.4784 537409 outcome TRUE reported 11118_107_RAB17_RAB17
RAB17 chr1 196141224 0.015352 3.805e-11 35328 TRUE reported
textfile 2 1 TRUE
3490 rs1047286 A G A G -0.0578 -0.0013 0.24066 NA FALSE FALSE FALSE
GCST006061 0.0098 0.8921 537409 outcome TRUE reported
3450_4_KLK6_Kallikrein_6 KLK6 chr19 6713251 0.009418 8.414e-10 35372
TRUE reported textfile 2 1 TRUE
3491 rs10801660 A G A G 0.082 -0.0119 0.11574 NA FALSE FALSE FALSE

GCST006061 0.0108 0.2735 537409 outcome TRUE reported
3450_4_KLK6_Kallikrein_6 KLK6 chr1 198272225 0.012585 7.231e-11 35345
TRUE reported textfile 2 1 TRUE
3492 rs111696008 A G A G -0.1488 -0.0259 0.02443 NA FALSE FALSE FALSE
GCST006061 0.0401 0.519 537409 outcome TRUE reported
3450_4_KLK6_Kallikrein_6 KLK6 chr2 216622986 0.026763 2.699e-08 35359
TRUE reported textfile 2 1 TRUE
3493 rs114558472 T C T C -0.0805 0.0072 0.09165 NA FALSE FALSE FALSE
GCST006061 0.0139 0.6063 537409 outcome TRUE reported
3450_4_KLK6_Kallikrein_6 KLK6 chr1 196140161 0.013966 8.222e-09 35337
TRUE reported textfile 2 1 TRUE
3494 rs1329424 G T G T -0.295 -0.0095 0.39735 NA FALSE FALSE FALSE
GCST006061 0.0075 0.2071 537409 outcome TRUE reported
3450_4_KLK6_Kallikrein_6 KLK6 chr1 196677046 0.008158 1e-200 35338
TRUE reported textfile 2 1 TRUE
3495 rs1707652 T C T C 0.1356 0.0081 0.3102 NA FALSE FALSE FALSE
GCST006061 0.0074 0.2701 537409 outcome TRUE reported
3450_4_KLK6_Kallikrein_6 KLK6 chr3 165761011 0.008675 4.479e-55 35339
TRUE reported textfile 2 1 TRUE
3496 rs72748420 T C T C 0.1028 0.0095 0.07417 NA FALSE FALSE FALSE
GCST006061 0.0134 0.4784 537409 outcome TRUE reported
3450_4_KLK6_Kallikrein_6 KLK6 chr1 196141224 0.015371 2.267e-11 35337
TRUE reported textfile 2 1 TRUE
3497 rs73169117 T C T C 0.0713 0.0051 0.11405 NA FALSE FALSE FALSE
GCST006061 0.0122 0.6755 537409 outcome TRUE reported
3450_4_KLK6_Kallikrein_6 KLK6 chr3 167217620 0.012762 2.312e-08 35354
TRUE reported textfile 2 1 TRUE
3498 rs1047286 A G A G -0.0538 -0.0013 0.24066 NA FALSE FALSE FALSE
GCST006061 0.0098 0.8921 537409 outcome TRUE reported 14331_262_DYNLT1_DYLT1
DYNLT1 chr19 6713251 0.009333 8.182e-09 35372 TRUE reported textfile
2 1 TRUE
3499 rs10922098 T C T C -0.1523 -0.009 0.40011 NA FALSE FALSE FALSE
GCST006061 0.0075 0.2275 537409 outcome TRUE reported 14331_262_DYNLT1_DYLT1
DYNLT1 chr1 196695521 0.008125 2.114e-78 35338 TRUE reported
textfile 2 1 TRUE
3500 rs1355538 G A G A -0.0657 -0.0076 0.39847 NA FALSE FALSE FALSE
GCST006061 0.0072 0.2923 537409 outcome TRUE reported 14331_262_DYNLT1_DYLT1
DYNLT1 chr3 165787389 0.008134 6.633e-16 35338 TRUE reported
textfile 2 1 TRUE
3501 rs61923082 C A C A -0.0908 0.0038 0.0616 NA FALSE FALSE FALSE
GCST006061 0.0164 0.8176 537409 outcome TRUE reported 14331_262_DYNLT1_DYLT1
DYNLT1 chr12 46179199 0.016557 4.156e-08 35361 TRUE reported
textfile 2 1 TRUE
3502 rs1047286 A G A G -0.0586 -0.0013 0.24066 NA FALSE FALSE FALSE
GCST006061 0.0098 0.8921 537409 outcome TRUE reported 18182_24_PCK1_PCKGC
PCK1 chr19 6713251 0.009373 4.049e-10 35372 TRUE reported textfile
2 1 TRUE
3503 rs1329424 G T G T -0.2616 -0.0095 0.39735 NA FALSE FALSE FALSE
GCST006061 0.0075 0.2071 537409 outcome TRUE reported 18182_24_PCK1_PCKGC
PCK1 chr1 196677046 0.008135 1e-200 35338 TRUE reported textfile
2 1 TRUE
3504 rs2686395 C T C T -0.1306 -0.0077 0.3101 NA FALSE FALSE FALSE
GCST006061 0.0074 0.2965 537409 outcome TRUE reported 18182_24_PCK1_PCKGC
PCK1 chr3 165767231 0.008635 1.106e-51 35339 TRUE reported
textfile 2 1 TRUE
3505 rs7513100 C T C T 0.0781 0 0.14871 NA FALSE FALSE FALSE GCST006061
0.0098 0.9981 537409 outcome TRUE reported 18182_24_PCK1_PCKGC PCK1 chr1
198065935 0.011267 4.154e-12 35343 TRUE reported textfile 2 1 TRUE
3506 rs1047286 A G A G -0.0586 -0.0013 0.24066 NA FALSE FALSE FALSE
GCST006061 0.0098 0.8921 537409 outcome TRUE reported 13653_335_MACROD1_MACD1
MACROD1 chr19 6713251 0.009328 3.333e-10 35372 TRUE reported textfile
2 1 TRUE
3507 rs10922098 T C T C -0.2145 -0.009 0.40011 NA FALSE FALSE FALSE
GCST006061 0.0075 0.2275 537409 outcome TRUE reported 13653_335_MACROD1_MACD1
MACROD1 chr1 196695521 0.00811 3.657e-154 35338 TRUE reported textfile
2 1 TRUE

3508 rs1707652 T C T C 0.1209 0.0081 0.3102 NA FALSE FALSE FALSE GCST006061 0.0074 0.2701 537409 outcome TRUE reported 13653_335_MACROD1_MACD1 MACROD1 chr3 165761011 0.008602 7.271e-45 35339 TRUE reported textfile 2 1 TRUE

3509 rs704 A G A G 0.0587 -0.0155 0.49189 NA FALSE FALSE FALSE GCST006061 0.0072 0.03144 537409 outcome TRUE reported 13653_335_MACROD1_MACD1 MACROD1 chr17 28367840 0.007999 2.165e-13 35360 TRUE reported textfile 2 1 TRUE

3510 rs1047286 A G A G -0.0589 -0.0013 0.24066 NA FALSE FALSE FALSE GCST006061 0.0098 0.8921 537409 outcome TRUE reported 13966_30_MYOM3_MYOM3 MYOM3 chr19 6713251 0.009435 4.298e-10 35372 TRUE reported textfile 2 1 TRUE

3511 rs1329424 G T G T -0.2266 -0.0095 0.39735 NA FALSE FALSE FALSE GCST006061 0.0075 0.2071 537409 outcome TRUE reported 13966_30_MYOM3_MYOM3 MYOM3 chr1 196677046 0.008213 1.465e-167 35338 TRUE reported textfile 2 1 TRUE

3512 rs144704969 T C T C 0.1086 -0.0238 0.04536 NA FALSE FALSE FALSE GCST006061 0.0154 0.1235 537409 outcome TRUE reported 13966_30_MYOM3_MYOM3 MYOM3 chr1 196158214 0.019296 1.822e-08 35337 TRUE reported textfile 2 1 TRUE

3513 rs2686395 C T C T -0.1064 -0.0077 0.3101 NA FALSE FALSE FALSE GCST006061 0.0074 0.2965 537409 outcome TRUE reported 13966_30_MYOM3_MYOM3 MYOM3 chr3 165767231 0.008708 2.487e-34 35339 TRUE reported textfile 2 1 TRUE

3514 rs72748420 T C T C 0.089 0.0095 0.07417 NA FALSE FALSE FALSE GCST006061 0.0134 0.4784 537409 outcome TRUE reported 13966_30_MYOM3_MYOM3 MYOM3 chr1 196141224 0.015365 6.941e-09 35337 TRUE reported textfile 2 1 TRUE

3515 rs1047286 A G A G -0.0602 -0.0013 0.24066 NA FALSE FALSE FALSE GCST006061 0.0098 0.8921 537409 outcome TRUE reported 13665_35_PPP2R3A_PP2A__subunit_B PPP2R3A chr19 6713251 0.0093 9.622e-11 35372 TRUE reported textfile 2 1 TRUE

3516 rs114009540 A G A G -0.1382 0.0322 0.03606 NA FALSE FALSE FALSE GCST006061 0.0299 0.2809 537409 outcome TRUE reported 13665_35_PPP2R3A_PP2A__subunit_B PPP2R3A chr1 196158413 0.021613 1.613e-10 35337 TRUE reported textfile 2 1 TRUE

3517 rs1329424 G T G T -0.2478 -0.0095 0.39735 NA FALSE FALSE FALSE GCST006061 0.0075 0.2071 537409 outcome TRUE reported 13665_35_PPP2R3A_PP2A__subunit_B PPP2R3A chr1 196677046 0.008088 1e-200 35338 TRUE reported textfile 2 1 TRUE

3518 rs190548044 C T C T -0.1765 -0.0653 0.01609 NA FALSE FALSE FALSE GCST006061 0.0416 0.1161 537409 outcome TRUE reported 13665_35_PPP2R3A_PP2A__subunit_B PPP2R3A chr1 197304559 0.031828 2.932e-08 35338 TRUE reported textfile 2 1 TRUE

3519 rs35351085 T C T C 0.1172 0.0061 0.04877 NA FALSE FALSE FALSE GCST006061 0.022 0.7825 537409 outcome TRUE reported 13665_35_PPP2R3A_PP2A__subunit_B PPP2R3A chr1 197992799 0.018443 2.088e-10 35343 TRUE reported textfile 2 1 TRUE

3520 rs1047286 A G A G -0.0622 -0.0013 0.24066 NA FALSE FALSE FALSE GCST006061 0.0098 0.8921 537409 outcome TRUE reported 19213_1_SPINK4_ISK4 SPINK4 chr19 6713251 0.009358 3.004e-11 35372 TRUE reported textfile 2 1 TRUE

3521 rs10758227 T C T C -0.0535 0.0087 0.31512 NA FALSE FALSE FALSE GCST006061 0.0074 0.2433 537409 outcome TRUE reported 19213_1_SPINK4_ISK4 SPINK4 chr9 33762137 0.008662 6.55e-10 35350 TRUE reported textfile 2 1 TRUE

3522 rs10922098 T C T C -0.2208 -0.009 0.40011 NA FALSE FALSE FALSE GCST006061 0.0075 0.2275 537409 outcome TRUE reported 19213_1_SPINK4_ISK4 SPINK4 chr1 196695521 0.008141 5.232e-162 35338 TRUE reported textfile 2 1 TRUE

3523 rs144704969 T C T C 0.1044 -0.0238 0.04536 NA FALSE FALSE FALSE GCST006061 0.0154 0.1235 537409 outcome TRUE reported 19213_1_SPINK4_ISK4 SPINK4 chr1 196158214 0.019143 4.93e-08 35337 TRUE reported textfile 2 1 TRUE

3524 rs1630171 G A G A 0.2788 -0.001 0.15268 NA FALSE FALSE FALSE GCST006061 0.0097 0.9177 537409 outcome TRUE reported 19213_1_SPINK4_ISK4

SPINK4 chr9 33234041 0.011113 6.624e-139 35348 TRUE reported textfile 2 1 TRUE

3525 rs1707652 T C T C 0.0937 0.0081 0.3102 NA FALSE FALSE FALSE GCST006061 0.0074 0.2701 537409 outcome TRUE reported 19213_1_SPINK4_ISK4 SPINK4 chr3 165761011 0.008639 2.072e-27 35339 TRUE reported textfile 2 1 TRUE

3526 rs1047286 A G A G -0.0587 -0.0013 0.24066 NA FALSE FALSE FALSE GCST006061 0.0098 0.8921 537409 outcome TRUE reported 7018_10_TSR3_TSR3 TSR3 chr19 6713251 0.009353 3.48e-10 35372 TRUE reported textfile 2 1 TRUE

3527 rs10922098 T C T C -0.1907 -0.009 0.40011 NA FALSE FALSE FALSE GCST006061 0.0075 0.2275 537409 outcome TRUE reported 7018_10_TSR3_TSR3 TSR3 chr1 196695521 0.008137 1.83e-121 35338 TRUE reported textfile 2 1 TRUE

3528 rs1451915 A C A C 0.0639 -0.0107 0.13641 NA FALSE FALSE FALSE GCST006061 0.0124 0.3895 537409 outcome TRUE reported 7018_10_TSR3_TSR3 TSR3 chr1 196058002 0.011598 3.595e-08 35335 TRUE reported textfile 2 1 TRUE

3529 rs1707652 T C T C 0.1041 0.0081 0.3102 NA FALSE FALSE FALSE GCST006061 0.0074 0.2701 537409 outcome TRUE reported 7018_10_TSR3_TSR3 TSR3 chr3 165761011 0.008629 1.638e-33 35339 TRUE reported textfile 2 1 TRUE

3530 rs1047286 A G A G -0.0598 -0.0013 0.24066 NA FALSE FALSE FALSE GCST006061 0.0098 0.8921 537409 outcome TRUE reported 9082_25_SEL1L2_SE1L2 SEL1L2 chr19 6713251 0.009393 1.934e-10 35372 TRUE reported textfile 2 1 TRUE

3531 rs10801660 A G A G 0.0695 -0.0119 0.11574 NA FALSE FALSE FALSE GCST006061 0.0108 0.2735 537409 outcome TRUE reported 9082_25_SEL1L2_SE1L2 SEL1L2 chr1 198272225 0.012532 2.926e-08 35345 TRUE reported textfile 2 1 TRUE

3532 rs111696008 A G A G -0.1456 -0.0259 0.02443 NA FALSE FALSE FALSE GCST006061 0.0401 0.519 537409 outcome TRUE reported 9082_25_SEL1L2_SE1L2 SEL1L2 chr2 216622986 0.02668 4.833e-08 35359 TRUE reported textfile 2 1 TRUE

3533 rs1329424 G T G T -0.2537 -0.0095 0.39735 NA FALSE FALSE FALSE GCST006061 0.0075 0.2071 537409 outcome TRUE reported 9082_25_SEL1L2_SE1L2 SEL1L2 chr1 196677046 0.008154 1e-200 35338 TRUE reported textfile 2 1 TRUE

3534 rs1707652 T C T C 0.129 0.0081 0.3102 NA FALSE FALSE FALSE GCST006061 0.0074 0.2701 537409 outcome TRUE reported 9082_25_SEL1L2_SE1L2 SEL1L2 chr3 165761011 0.008653 2.931e-50 35339 TRUE reported textfile 2 1 TRUE

3535 rs704 A G A G 0.0693 -0.0155 0.49189 NA FALSE FALSE FALSE GCST006061 0.0072 0.03144 537409 outcome TRUE reported 9082_25_SEL1L2_SE1L2 SEL1L2 chr17 28367840 0.008036 6.487e-18 35360 TRUE reported textfile 2 1 TRUE

3536 rs73169117 T C T C 0.0753 0.0051 0.11405 NA FALSE FALSE FALSE GCST006061 0.0122 0.6755 537409 outcome TRUE reported 9082_25_SEL1L2_SE1L2 SEL1L2 chr3 167217620 0.012731 3.329e-09 35354 TRUE reported textfile 2 1 TRUE

3537 rs1047286 A G A G -0.0748 -0.0013 0.24066 NA FALSE FALSE FALSE GCST006061 0.0098 0.8921 537409 outcome TRUE reported 12480_9_OTUD5_OTUD5 OTUD5 chr19 6713251 0.009394 1.684e-15 35363 TRUE reported textfile 2 1 TRUE

3538 rs10922098 T C T C -0.229 -0.009 0.40011 NA FALSE FALSE FALSE GCST006061 0.0075 0.2275 537409 outcome TRUE reported 12480_9_OTUD5_OTUD5 OTUD5 chr1 196695521 0.008164 4.183e-173 35329 TRUE reported textfile 2 1 TRUE

3539 rs1451915 A C A C 0.0718 -0.0107 0.13641 NA FALSE FALSE FALSE GCST006061 0.0124 0.3895 537409 outcome TRUE reported 12480_9_OTUD5_OTUD5 OTUD5 chr1 196058002 0.011672 7.678e-10 35326 TRUE reported textfile 2 1 TRUE

3540 rs1707652 T C T C 0.1074 0.0081 0.3102 NA FALSE FALSE FALSE GCST006061 0.0074 0.2701 537409 outcome TRUE reported 12480_9_OTUD5_OTUD5 OTUD5 chr3 165761011 0.008664 2.75e-35 35330 TRUE reported textfile 2 1 TRUE

3541 rs1047286 A G A G -0.0602 -0.0013 0.24066 NA FALSE FALSE FALSE GCST006061 0.0098 0.8921 537409 outcome TRUE reported 9954_2_NPIPB3_NPPL3 NPIPB3 chr19 6713251 0.00942 1.653e-10 35372 TRUE reported textfile 2 1 TRUE

3542 rs10922098 T C T C -0.2318 -0.009 0.40011 NA FALSE FALSE FALSE GCST006061 0.0075 0.2275 537409 outcome TRUE reported 9954_2_NPIPB3_NPPL3 NPIPB3 chr1 196695521 0.008173 5.785e-177 35338 TRUE reported textfile 2 1 TRUE

3543 rs111696008 A G A G -0.1543 -0.0259 0.02443 NA FALSE FALSE FALSE GCST006061 0.0401 0.519 537409 outcome TRUE reported 9954_2_NPIPB3_NPPL3 NPIPB3 chr2 216622986 0.026769 8.208e-09 35359 TRUE reported textfile 2 1 TRUE

3544 rs1451915 A C A C 0.0712 -0.0107 0.13641 NA FALSE FALSE FALSE GCST006061 0.0124 0.3895 537409 outcome TRUE reported 9954_2_NPIPB3_NPPL3 NPIPB3 chr1 196058002 0.011688 1.116e-09 35335 TRUE reported textfile 2 1 TRUE

3545 rs2686395 C T C T -0.1148 -0.0077 0.3101 NA FALSE FALSE FALSE GCST006061 0.0074 0.2965 537409 outcome TRUE reported 9954_2_NPIPB3_NPPL3 NPIPB3 chr3 165767231 0.008679 6.131e-40 35339 TRUE reported textfile 2 1 TRUE

3546 rs704 A G A G 0.0794 -0.0155 0.49189 NA FALSE FALSE FALSE GCST006061 0.0072 0.03144 537409 outcome TRUE reported 9954_2_NPIPB3_NPPL3 NPIPB3 chr17 28367840 0.008062 6.973e-23 35360 TRUE reported textfile 2 1 TRUE

3547 rs7513100 C T C T 0.0643 0 0.14871 NA FALSE FALSE FALSE GCST006061 0.0098 0.9981 537409 outcome TRUE reported 9954_2_NPIPB3_NPPL3 NPIPB3 chr1 198065935 0.0113 1.27e-08 35343 TRUE reported textfile 2 1 TRUE

3548 rs1047286 A G A G -0.0514 -0.0013 0.24066 NA FALSE FALSE FALSE GCST006061 0.0098 0.8921 537409 outcome TRUE reported 11439_88_RHPN2_RHPN2 RHPN2 chr19 6713251 0.009336 3.679e-08 35363 TRUE reported textfile 2 1 TRUE

3549 rs10922098 T C T C -0.1985 -0.009 0.40011 NA FALSE FALSE FALSE GCST006061 0.0075 0.2275 537409 outcome TRUE reported 11439_88_RHPN2_RHPN2 RHPN2 chr1 196695521 0.008109 2.426e-132 35329 TRUE reported textfile 2 1 TRUE

3550 rs1451915 A C A C 0.0651 -0.0107 0.13641 NA FALSE FALSE FALSE GCST006061 0.0124 0.3895 537409 outcome TRUE reported 11439_88_RHPN2_RHPN2 RHPN2 chr1 196058002 0.011573 1.855e-08 35326 TRUE reported textfile 2 1 TRUE

3551 rs1707652 T C T C 0.1008 0.0081 0.3102 NA FALSE FALSE FALSE GCST006061 0.0074 0.2701 537409 outcome TRUE reported 11439_88_RHPN2_RHPN2 RHPN2 chr3 165761011 0.008601 1.011e-31 35330 TRUE reported textfile 2 1 TRUE

3552 rs704 A G A G 0.0488 -0.0155 0.49189 NA FALSE FALSE FALSE GCST006061 0.0072 0.03144 537409 outcome TRUE reported 11439_88_RHPN2_RHPN2 RHPN2 chr17 28367840 0.007997 1.047e-09 35351 TRUE reported textfile 2 1 TRUE

3553 rs1047286 A G A G -0.0645 -0.0013 0.24066 NA FALSE FALSE FALSE GCST006061 0.0098 0.8921 537409 outcome TRUE reported 19596_18_BRF1_BRF_1 BRF1 chr19 6713251 0.009321 4.517e-12 35372 TRUE reported textfile 2 1 TRUE

3554 rs10922098 T C T C -0.1837 -0.009 0.40011 NA FALSE FALSE FALSE GCST006061 0.0075 0.2275 537409 outcome TRUE reported 19596_18_BRF1_BRF_1 BRF1 chr1 196695521 0.008117 2.14e-113 35338 TRUE reported textfile 2 1 TRUE

3555 rs111696008 A G A G -0.1732 -0.0259 0.02443 NA FALSE FALSE FALSE GCST006061 0.0401 0.519 537409 outcome TRUE reported 19596_18_BRF1_BRF_1 BRF1 chr2 216622986 0.026505 6.377e-11 35359 TRUE reported textfile 2 1 TRUE

3556 rs11906988 G T G T -0.2426 -0.0389 0.01085 NA FALSE FALSE FALSE GCST006061 0.0283 0.1697 537409 outcome TRUE reported 19596_18_BRF1_BRF_1 BRF1 chr20 38383368 0.037862 1.48e-10 35372 TRUE reported textfile 2 1 TRUE

3557 rs2145943 G A G A -0.0477 -0.0035 0.41676 NA FALSE FALSE FALSE GCST006061 0.0073 0.6331 537409 outcome TRUE reported 19596_18_BRF1_BRF_1 BRF1 chr20 39934603 0.008053 3.154e-09 35376 TRUE reported textfile 2 1 TRUE

3558 rs2232613 T C T C -0.2689 0.0151 0.0989 NA FALSE FALSE FALSE
GCST006061 0.0141 0.2834 537409 outcome TRUE reported 19596_18_BRF1_BRF_1
BRF1 chr20 38369011 0.013114 1.93e-93 35375 TRUE reported
textfile 2 1 TRUE
3559 rs6445035 A G A G 0.1524 0.0154 0.1904 NA FALSE FALSE FALSE
GCST006061 0.0089 0.08256 537409 outcome TRUE reported 19596_18_BRF1_BRF_1
BRF1 chr3 165762312 0.010123 3.236e-51 35339 TRUE reported
textfile 2 1 TRUE
3560 rs73169117 T C T C 0.0697 0.0051 0.11405 NA FALSE FALSE FALSE
GCST006061 0.0122 0.6755 537409 outcome TRUE reported 19596_18_BRF1_BRF_1
BRF1 chr3 167217620 0.012656 3.643e-08 35354 TRUE reported
textfile 2 1 TRUE
3561 rs1047286 A G A G -0.0585 -0.0013 0.24066 NA FALSE FALSE FALSE
GCST006061 0.0098 0.8921 537409 outcome TRUE reported 9043_7_TFIP11_TFP11
TFIP11 chr19 6713251 0.009308 3.287e-10 35372 TRUE reported textfile
2 1 TRUE
3562 rs10922098 T C T C -0.2024 -0.009 0.40011 NA FALSE FALSE FALSE
GCST006061 0.0075 0.2275 537409 outcome TRUE reported 9043_7_TFIP11_TFP11
TFIP11 chr1 196695521 0.008096 6.196e-138 35338 TRUE reported
textfile 2 1 TRUE
3563 rs1707652 T C T C 0.0978 0.0081 0.3102 NA FALSE FALSE FALSE
GCST006061 0.0074 0.2701 537409 outcome TRUE reported 9043_7_TFIP11_TFP11
TFIP11 chr3 165761011 0.008591 5.061e-30 35339 TRUE reported
textfile 2 1 TRUE
3564 rs1047286 A G A G -0.0645 -0.0013 0.24066 NA FALSE FALSE FALSE
GCST006061 0.0098 0.8921 537409 outcome TRUE reported
8424_269_CTNNB1_b_Catenin CTNNB1 chr19 6713251 0.009386 6.336e-12 35372
TRUE reported textfile 2 1 TRUE
3565 rs114009540 A G A G -0.142 0.0322 0.03606 NA FALSE FALSE FALSE
GCST006061 0.0299 0.2809 537409 outcome TRUE reported
8424_269_CTNNB1_b_Catenin CTNNB1 chr1 196158413 0.021795 7.253e-11 35337
TRUE reported textfile 2 1 TRUE
3566 rs1329424 G T G T -0.2569 -0.0095 0.39735 NA FALSE FALSE FALSE
GCST006061 0.0075 0.2071 537409 outcome TRUE reported
8424_269_CTNNB1_b_Catenin CTNNB1 chr1 196677046 0.008153 1e-200 35338
TRUE reported textfile 2 1 TRUE
3567 rs2686395 C T C T -0.1243 -0.0077 0.3101 NA FALSE FALSE FALSE
GCST006061 0.0074 0.2965 537409 outcome TRUE reported
8424_269_CTNNB1_b_Catenin CTNNB1 chr3 165767231 0.008651 8.108e-47 35339
TRUE reported textfile 2 1 TRUE
3568 rs73169117 T C T C 0.0695 0.0051 0.11405 NA FALSE FALSE FALSE
GCST006061 0.0122 0.6755 537409 outcome TRUE reported
8424_269_CTNNB1_b_Catenin CTNNB1 chr3 167217620 0.012729 4.766e-08 35354
TRUE reported textfile 2 1 TRUE
3569 rs1047286 A G A G -0.1135 -0.0013 0.24066 NA FALSE FALSE FALSE
GCST006061 0.0098 0.8921 537409 outcome TRUE reported 9254_18_FCER1A_FCERA
FCER1A chr19 6713251 0.009417 1.89e-33 35372 TRUE reported textfile
2 1 TRUE
3570 rs10922098 T C T C -0.228 -0.009 0.40011 NA FALSE FALSE FALSE
GCST006061 0.0075 0.2275 537409 outcome TRUE reported 9254_18_FCER1A_FCERA
FCER1A chr1 196695521 0.008186 1.052e-170 35338 TRUE reported
textfile 2 1 TRUE
3571 rs114009540 A G A G -0.1278 0.0322 0.03606 NA FALSE FALSE FALSE
GCST006061 0.0299 0.2809 537409 outcome TRUE reported 9254_18_FCER1A_FCERA
FCER1A chr1 196158413 0.021855 4.988e-09 35337 TRUE reported
textfile 2 1 TRUE
3572 rs1707652 T C T C 0.0824 0.0081 0.3102 NA FALSE FALSE FALSE
GCST006061 0.0074 0.2701 537409 outcome TRUE reported 9254_18_FCER1A_FCERA
FCER1A chr3 165761011 0.008697 2.683e-21 35339 TRUE reported
textfile 2 1 TRUE
3573 rs687628 A G A G 0.0574 0.0132 0.29696 NA FALSE FALSE FALSE
GCST006061 0.0078 0.09099 537409 outcome TRUE reported 9254_18_FCER1A_FCERA
FCER1A chr1 56911906 0.008811 7.288e-11 35369 TRUE reported
textfile 2 1 TRUE
3574 rs1047286 A G A G -0.0797 -0.0013 0.24066 NA FALSE FALSE FALSE
GCST006061 0.0098 0.8921 537409 outcome TRUE reported 11666_72_RGS8_RGS8

RGS8 chr19 6713251 0.009433 2.934e-17 35363 TRUE reported textfile
2 1 TRUE
3575 rs10922098 T C T C -0.2478 -0.009 0.40011 NA FALSE FALSE FALSE
GCST006061 0.0075 0.2275 537409 outcome TRUE reported 11666_72_RGS8_RGS8
RGS8 chr1 196695521 0.008191 1e-200 35329 TRUE reported textfile
2 1 TRUE
3576 rs111696008 A G A G -0.1508 -0.0259 0.02443 NA FALSE FALSE FALSE
GCST006061 0.0401 0.519 537409 outcome TRUE reported 11666_72_RGS8_RGS8
RGS8 chr2 216622986 0.026835 1.915e-08 35350 TRUE reported
textfile 2 1 TRUE
3577 rs114009540 A G A G -0.1289 0.0322 0.03606 NA FALSE FALSE FALSE
GCST006061 0.0299 0.2809 537409 outcome TRUE reported 11666_72_RGS8_RGS8
RGS8 chr1 196158413 0.02192 4.093e-09 35328 TRUE reported textfile
2 1 TRUE
3578 rs1707652 T C T C 0.1211 0.0081 0.3102 NA FALSE FALSE FALSE
GCST006061 0.0074 0.2701 537409 outcome TRUE reported 11666_72_RGS8_RGS8
RGS8 chr3 165761011 0.0087 4.8e-44 35330 TRUE reported textfile 2
1 TRUE
3579 rs72748420 T C T C 0.0851 0.0095 0.07417 NA FALSE FALSE FALSE
GCST006061 0.0134 0.4784 537409 outcome TRUE reported 11666_72_RGS8_RGS8
RGS8 chr1 196141224 0.015376 3.12e-08 35328 TRUE reported
textfile 2 1 TRUE
3580 rs1047286 A G A G -0.0639 -0.0013 0.24066 NA FALSE FALSE FALSE
GCST006061 0.0098 0.8921 537409 outcome TRUE reported 11281_6_GRB7_GRB7
GRB7 chr19 6713251 0.009351 8.276e-12 35363 TRUE reported textfile
2 1 TRUE
3581 rs10922098 T C T C -0.2326 -0.009 0.40011 NA FALSE FALSE FALSE
GCST006061 0.0075 0.2275 537409 outcome TRUE reported 11281_6_GRB7_GRB7
GRB7 chr1 196695521 0.008117 1.394e-180 35329 TRUE reported
textfile 2 1 TRUE
3582 rs111696008 A G A G -0.1449 -0.0259 0.02443 NA FALSE FALSE FALSE
GCST006061 0.0401 0.519 537409 outcome TRUE reported 11281_6_GRB7_GRB7
GRB7 chr2 216622986 0.026572 4.949e-08 35350 TRUE reported
textfile 2 1 TRUE
3583 rs144704969 T C T C 0.1057 -0.0238 0.04536 NA FALSE FALSE FALSE
GCST006061 0.0154 0.1235 537409 outcome TRUE reported 11281_6_GRB7_GRB7
GRB7 chr1 196158214 0.019103 3.144e-08 35328 TRUE reported
textfile 2 1 TRUE
3584 rs1707652 T C T C 0.1085 0.0081 0.3102 NA FALSE FALSE FALSE
GCST006061 0.0074 0.2701 537409 outcome TRUE reported 11281_6_GRB7_GRB7
GRB7 chr3 165761011 0.008619 2.457e-36 35330 TRUE reported
textfile 2 1 TRUE
3585 rs704 A G A G 0.0825 -0.0155 0.49189 NA FALSE FALSE FALSE GCST006061
0.0072 0.03144 537409 outcome TRUE reported 11281_6_GRB7_GRB7 GRB7 chr17
28367840 0.008005 6.574e-25 35351 TRUE reported textfile 2 1 TRUE
3586 rs1047286 A G A G -0.0535 -0.0013 0.24066 NA FALSE FALSE FALSE
GCST006061 0.0098 0.8921 537409 outcome TRUE reported 12463_7_KLHL13_KLH13
KLHL13 chr19 6713251 0.009284 8.274e-09 35363 TRUE reported textfile
2 1 TRUE
3587 rs10922098 T C T C -0.1819 -0.009 0.40011 NA FALSE FALSE FALSE
GCST006061 0.0075 0.2275 537409 outcome TRUE reported 12463_7_KLHL13_KLH13
KLHL13 chr1 196695521 0.008084 3.94e-112 35329 TRUE reported
textfile 2 1 TRUE
3588 rs1707652 T C T C 0.1022 0.0081 0.3102 NA FALSE FALSE FALSE
GCST006061 0.0074 0.2701 537409 outcome TRUE reported 12463_7_KLHL13_KLH13
KLHL13 chr3 165761011 0.008563 7.827e-33 35330 TRUE reported
textfile 2 1 TRUE
3589 rs704 A G A G 0.0794 -0.0155 0.49189 NA FALSE FALSE FALSE GCST006061
0.0072 0.03144 537409 outcome TRUE reported 12463_7_KLHL13_KLH13 KLHL13
chr17 28367840 0.007958 1.922e-23 35351 TRUE reported textfile 2
1 TRUE
3590 rs1047286 A G A G -0.056 -0.0013 0.24066 NA FALSE FALSE FALSE
GCST006061 0.0098 0.8921 537409 outcome TRUE reported 8017_23_FOXRED1_FXRD1
FOXRED1 chr19 6713251 0.009263 1.488e-09 35372 TRUE reported textfile
2 1 TRUE
3591 rs10922098 T C T C -0.158 -0.009 0.40011 NA FALSE FALSE FALSE

GCST006061 0.0075 0.2275 537409 outcome TRUE reported 8017_23_FOXRED1_FXRD1
FOXRED1 chr1 196695521 0.008066 1.956e-85 35338 TRUE reported
textfile 2 1 TRUE
3592 rs2686395 C T C T -0.0916 -0.0077 0.3101 NA FALSE FALSE FALSE
GCST006061 0.0074 0.2965 537409 outcome TRUE reported 8017_23_FOXRED1_FXRD1
FOXRED1 chr3 165767231 0.008543 7.967e-27 35339 TRUE reported
textfile 2 1 TRUE
3593 rs34698754 G T G T 0.0554 -7.00E-04 0.1897 NA FALSE FALSE FALSE
GCST006061 0.0092 0.9393 537409 outcome TRUE reported 8017_23_FOXRED1_FXRD1
FOXRED1 chr14 49933544 0.010091 4.016e-08 35359 TRUE reported
textfile 2 1 TRUE
3594 rs1047286 A G A G -0.0517 -0.0013 0.24066 NA FALSE FALSE FALSE
GCST006061 0.0098 0.8921 537409 outcome TRUE reported
19208_8_ACVR2A_Activin_RIIA ACVR2A chr19 6713251 0.009335 3.057e-08 35372
TRUE reported textfile 2 1 TRUE
3595 rs10922098 T C T C -0.2092 -0.009 0.40011 NA FALSE FALSE FALSE
GCST006061 0.0075 0.2275 537409 outcome TRUE reported
19208_8_ACVR2A_Activin_RIIA ACVR2A chr1 196695521 0.008112 1.167e-146 35338
TRUE reported textfile 2 1 TRUE
3596 rs111696008 A G A G -0.1575 -0.0259 0.02443 NA FALSE FALSE FALSE
GCST006061 0.0401 0.519 537409 outcome TRUE reported
19208_8_ACVR2A_Activin_RIIA ACVR2A chr2 216622986 0.026533 2.921e-09 35359
TRUE reported textfile 2 1 TRUE
3597 rs1047286 A G A G -0.0696 -0.0013 0.24066 NA FALSE FALSE FALSE
GCST006061 0.0098 0.8921 537409 outcome TRUE reported 18938_3_VLDLR_VLDLR
VLDLR chr19 6713251 0.009373 1.12e-13 35372 TRUE reported textfile
2 1 TRUE
3598 rs111696008 A G A G -0.1494 -0.0259 0.02443 NA FALSE FALSE FALSE
GCST006061 0.0401 0.519 537409 outcome TRUE reported 18938_3_VLDLR_VLDLR
VLDLR chr2 216622986 0.026666 2.111e-08 35359 TRUE reported
textfile 2 1 TRUE
3599 rs114009540 A G A G -0.122 0.0322 0.03606 NA FALSE FALSE FALSE
GCST006061 0.0299 0.2809 537409 outcome TRUE reported 18938_3_VLDLR_VLDLR
VLDLR chr1 196158413 0.021786 2.144e-08 35337 TRUE reported
textfile 2 1 TRUE
3600 rs1329424 G T G T -0.2535 -0.0095 0.39735 NA FALSE FALSE FALSE
GCST006061 0.0075 0.2071 537409 outcome TRUE reported 18938_3_VLDLR_VLDLR
VLDLR chr1 196677046 0.008148 1e-200 35338 TRUE reported textfile
2 1 TRUE
3601 rs1707652 T C T C 0.118 0.0081 0.3102 NA FALSE FALSE FALSE
GCST006061 0.0074 0.2701 537409 outcome TRUE reported 18938_3_VLDLR_VLDLR
VLDLR chr3 165761011 0.008649 2.235e-42 35339 TRUE reported
textfile 2 1 TRUE
3602 rs72748420 T C T C 0.0848 0.0095 0.07417 NA FALSE FALSE FALSE
GCST006061 0.0134 0.4784 537409 outcome TRUE reported 18938_3_VLDLR_VLDLR
VLDLR chr1 196141224 0.015301 2.988e-08 35337 TRUE reported
textfile 2 1 TRUE
3603 rs1047286 A G A G -0.0547 -0.0013 0.24066 NA FALSE FALSE FALSE
GCST006061 0.0098 0.8921 537409 outcome TRUE reported 9128_34_FLRT3_FLRT3
FLRT3 chr19 6713251 0.009279 3.752e-09 35372 TRUE reported textfile
2 1 TRUE
3604 rs10922098 T C T C -0.1417 -0.009 0.40011 NA FALSE FALSE FALSE
GCST006061 0.0075 0.2275 537409 outcome TRUE reported 9128_34_FLRT3_FLRT3
FLRT3 chr1 196695521 0.008096 1.378e-68 35338 TRUE reported
textfile 2 1 TRUE
3605 rs1706435 G A G A 0.0719 0.0117 0.36981 NA FALSE FALSE FALSE
GCST006061 0.0072 0.1047 537409 outcome TRUE reported 9128_34_FLRT3_FLRT3
FLRT3 chr3 165743688 0.008219 2.173e-18 35339 TRUE reported
textfile 2 1 TRUE
3606 rs6542680 T C T C -0.066 -0.0012 0.18362 NA FALSE FALSE FALSE
GCST006061 0.0091 0.8935 537409 outcome TRUE reported 9128_34_FLRT3_FLRT3
FLRT3 chr2 3592552 0.010285 1.389e-10 35332 TRUE reported textfile
2 1 TRUE
3607 rs1047286 A G A G -0.0735 -0.0013 0.24066 NA FALSE FALSE FALSE
GCST006061 0.0098 0.8921 537409 outcome TRUE reported 8681_93_PGRMC2_PGRC2
PGRMC2 chr19 6713251 0.009322 3.156e-15 35372 TRUE reported textfile

2 1 TRUE
3608 rs114009540 A G A G -0.1196 0.0322 0.03606 NA FALSE FALSE FALSE
GCST006061 0.0299 0.2809 537409 outcome TRUE reported 8681_93_PGRMC2_PGRC2
PGRMC2 chr1 196158413 0.021573 2.959e-08 35337 TRUE reported
textfile 2 1 TRUE
3609 rs1707652 T C T C 0.0865 0.0081 0.3102 NA FALSE FALSE FALSE
GCST006061 0.0074 0.2701 537409 outcome TRUE reported 8681_93_PGRMC2_PGRC2
PGRMC2 chr3 165761011 0.008601 8.556e-24 35339 TRUE reported
textfile 2 1 TRUE
3610 rs1047286 A G A G -0.0583 -0.0013 0.24066 NA FALSE FALSE FALSE
GCST006061 0.0098 0.8921 537409 outcome TRUE reported 3387_1_PAK3_PAK3
PAK3 chr19 6713251 0.009397 5.5e-10 35372 TRUE reported textfile 2
1 TRUE
3611 rs111696008 A G A G -0.147 -0.0259 0.02443 NA FALSE FALSE FALSE
GCST006061 0.0401 0.519 537409 outcome TRUE reported 3387_1_PAK3_PAK3
PAK3 chr2 216622986 0.02669 3.634e-08 35359 TRUE reported textfile
2 1 TRUE
3612 rs1329424 G T G T -0.2766 -0.0095 0.39735 NA FALSE FALSE FALSE
GCST006061 0.0075 0.2071 537409 outcome TRUE reported 3387_1_PAK3_PAK3
PAK3 chr1 196677046 0.008144 1e-200 35338 TRUE reported textfile
2 1 TRUE
3613 rs1707652 T C T C 0.1148 0.0081 0.3102 NA FALSE FALSE FALSE
GCST006061 0.0074 0.2701 537409 outcome TRUE reported 3387_1_PAK3_PAK3
PAK3 chr3 165761011 0.008655 3.737e-40 35339 TRUE reported
textfile 2 1 TRUE
3614 rs7513100 C T C T 0.0817 0 0.14871 NA FALSE FALSE FALSE GCST006061
0.0098 0.9981 537409 outcome TRUE reported 3387_1_PAK3_PAK3 PAK3 chr1
198065935 0.011289 4.591e-13 35343 TRUE reported textfile 2 1 TRUE
3615 rs1047286 A G A G -0.0584 -0.0013 0.24066 NA FALSE FALSE FALSE
GCST006061 0.0098 0.8921 537409 outcome TRUE reported 15675_3_CEBPB_CEBPB
CEBPB chr19 6713251 0.009298 3.368e-10 35372 TRUE reported textfile
2 1 TRUE
3616 rs10801660 A G A G 0.0775 -0.0119 0.11574 NA FALSE FALSE FALSE
GCST006061 0.0108 0.2735 537409 outcome TRUE reported 15675_3_CEBPB_CEBPB
CEBPB chr1 198272225 0.012423 4.414e-10 35345 TRUE reported
textfile 2 1 TRUE
3617 rs111696008 A G A G -0.1558 -0.0259 0.02443 NA FALSE FALSE FALSE
GCST006061 0.0401 0.519 537409 outcome TRUE reported 15675_3_CEBPB_CEBPB
CEBPB chr2 216622986 0.02644 3.802e-09 35359 TRUE reported textfile
2 1 TRUE
3618 rs1329424 G T G T -0.2756 -0.0095 0.39735 NA FALSE FALSE FALSE
GCST006061 0.0075 0.2071 537409 outcome TRUE reported 15675_3_CEBPB_CEBPB
CEBPB chr1 196677046 0.008065 1e-200 35338 TRUE reported textfile
2 1 TRUE
3619 rs2686395 C T C T -0.1378 -0.0077 0.3101 NA FALSE FALSE FALSE
GCST006061 0.0074 0.2965 537409 outcome TRUE reported 15675_3_CEBPB_CEBPB
CEBPB chr3 165767231 0.008574 3.987e-58 35339 TRUE reported
textfile 2 1 TRUE
3620 rs72748420 T C T C 0.0924 0.0095 0.07417 NA FALSE FALSE FALSE
GCST006061 0.0134 0.4784 537409 outcome TRUE reported 15675_3_CEBPB_CEBPB
CEBPB chr1 196141224 0.015174 1.133e-09 35337 TRUE reported
textfile 2 1 TRUE
3621 rs1047286 A G A G -0.0696 -0.0013 0.24066 NA FALSE FALSE FALSE
GCST006061 0.0098 0.8921 537409 outcome TRUE reported 13994_1_FBL_FBRL FBL
chr19 6713251 0.009279 6.34e-14 35372 TRUE reported textfile 2 1
TRUE
3622 rs10922098 T C T C -0.2097 -0.009 0.40011 NA FALSE FALSE FALSE
GCST006061 0.0075 0.2275 537409 outcome TRUE reported 13994_1_FBL_FBRL FBL
chr1 196695521 0.008068 6.332e-149 35338 TRUE reported textfile 2
1 TRUE
3623 rs2686395 C T C T -0.0991 -0.0077 0.3101 NA FALSE FALSE FALSE
GCST006061 0.0074 0.2965 537409 outcome TRUE reported 13994_1_FBL_FBRL FBL
chr3 165767231 0.008567 6.011e-31 35339 TRUE reported textfile 2
1 TRUE
3624 rs1047286 A G A G -0.0521 -0.0013 0.24066 NA FALSE FALSE FALSE
GCST006061 0.0098 0.8921 537409 outcome TRUE reported 18208_3_CCNB2_CCNB2

CCNB2 chr19 6713251 0.009399 2.966e-08 35372 TRUE reported textfile
2 1 TRUE
3625 rs10801660 A G A G 0.069 -0.0119 0.11574 NA FALSE FALSE FALSE
GCST006061 0.0108 0.2735 537409 outcome TRUE reported 18208_3_CCNB2_CCNB2
CCNB2 chr1 198272225 0.012557 3.905e-08 35345 TRUE reported
textfile 2 1 TRUE
3626 rs111696008 A G A G -0.1559 -0.0259 0.02443 NA FALSE FALSE FALSE
GCST006061 0.0401 0.519 537409 outcome TRUE reported 18208_3_CCNB2_CCNB2
CCNB2 chr2 216622986 0.026734 5.495e-09 35359 TRUE reported
textfile 2 1 TRUE
3627 rs12629202 A C A C 0.0484 0.0052 0.31116 NA FALSE FALSE FALSE
GCST006061 0.0081 0.5213 537409 outcome TRUE reported 18208_3_CCNB2_CCNB2
CCNB2 chr3 167083477 0.008687 2.521e-08 35354 TRUE reported
textfile 2 1 TRUE
3628 rs1329424 G T G T -0.2586 -0.0095 0.39735 NA FALSE FALSE FALSE
GCST006061 0.0075 0.2071 537409 outcome TRUE reported 18208_3_CCNB2_CCNB2
CCNB2 chr1 196677046 0.008164 1e-200 35338 TRUE reported textfile
2 1 TRUE
3629 rs2686395 C T C T -0.1383 -0.0077 0.3101 NA FALSE FALSE FALSE
GCST006061 0.0074 0.2965 537409 outcome TRUE reported 18208_3_CCNB2_CCNB2
CCNB2 chr3 165767231 0.008661 2.132e-57 35339 TRUE reported
textfile 2 1 TRUE
3630 rs72748420 T C T C 0.0902 0.0095 0.07417 NA FALSE FALSE FALSE
GCST006061 0.0134 0.4784 537409 outcome TRUE reported 18208_3_CCNB2_CCNB2
CCNB2 chr1 196141224 0.015333 4.033e-09 35337 TRUE reported
textfile 2 1 TRUE
3631 rs1047286 A G A G -0.0676 -0.0013 0.24066 NA FALSE FALSE FALSE
GCST006061 0.0098 0.8921 537409 outcome TRUE reported
7867_154_ST6GALNAC1_SIA7A ST6GALNAC1 chr19 6713251 0.009308 3.789e-13 35372
TRUE reported textfile 2 1 TRUE
3632 rs10922098 T C T C -0.1782 -0.009 0.40011 NA FALSE FALSE FALSE
GCST006061 0.0075 0.2275 537409 outcome TRUE reported
7867_154_ST6GALNAC1_SIA7A ST6GALNAC1 chr1 196695521 0.008099 2.781e-107
35338 TRUE reported textfile 2 1 TRUE
3633 rs1707652 T C T C 0.0861 0.0081 0.3102 NA FALSE FALSE FALSE
GCST006061 0.0074 0.2701 537409 outcome TRUE reported
7867_154_ST6GALNAC1_SIA7A ST6GALNAC1 chr3 165761011 0.008591 1.212e-23
35339 TRUE reported textfile 2 1 TRUE
3634 rs1047286 A G A G -0.0543 -0.0013 0.24066 NA FALSE FALSE FALSE
GCST006061 0.0098 0.8921 537409 outcome TRUE reported 9457_3_CAV2_CAV2
CAV2 chr19 6713251 0.009238 4.148e-09 35372 TRUE reported textfile
2 1 TRUE
3635 rs10922098 T C T C -0.1755 -0.009 0.40011 NA FALSE FALSE FALSE
GCST006061 0.0075 0.2275 537409 outcome TRUE reported 9457_3_CAV2_CAV2
CAV2 chr1 196695521 0.008033 8.336e-106 35338 TRUE reported
textfile 2 1 TRUE
3636 rs1555405 A G A G -0.0483 -0.0047 0.30381 NA FALSE FALSE FALSE
GCST006061 0.0081 0.5592 537409 outcome TRUE reported 9457_3_CAV2_CAV2
CAV2 chr14 100710432 0.008619 2.094e-08 35380 TRUE reported
textfile 2 1 TRUE
3637 rs1707652 T C T C 0.0878 0.0081 0.3102 NA FALSE FALSE FALSE
GCST006061 0.0074 0.2701 537409 outcome TRUE reported 9457_3_CAV2_CAV2
CAV2 chr3 165761011 0.008516 6.364e-25 35339 TRUE reported
textfile 2 1 TRUE
3638 rs1047286 A G A G -0.0764 -0.0013 0.24066 NA FALSE FALSE FALSE
GCST006061 0.0098 0.8921 537409 outcome TRUE reported
7823_22_ST6GALNAC2_SIA7B ST6GALNAC2 chr19 6713251 0.009396 4.25e-16 35372
TRUE reported textfile 2 1 TRUE
3639 rs114009540 A G A G -0.1397 0.0322 0.03606 NA FALSE FALSE FALSE
GCST006061 0.0299 0.2809 537409 outcome TRUE reported
7823_22_ST6GALNAC2_SIA7B ST6GALNAC2 chr1 196158413 0.021842 1.597e-10
35337 TRUE reported textfile 2 1 TRUE
3640 rs1329424 G T G T -0.2762 -0.0095 0.39735 NA FALSE FALSE FALSE
GCST006061 0.0075 0.2071 537409 outcome TRUE reported
7823_22_ST6GALNAC2_SIA7B ST6GALNAC2 chr1 196677046 0.008154 1e-200 35338
TRUE reported textfile 2 1 TRUE

3641 rs1707652 T C T C 0.1231 0.0081 0.3102 NA FALSE FALSE FALSE
GCST006061 0.0074 0.2701 537409 outcome TRUE reported
7823_22_ST6GALNAC2_SIA7B ST6GALNAC2 chr3 165761011 0.008659 7.185e-46
35339 TRUE reported textfile 2 1 TRUE
3642 rs72748420 T C T C 0.0931 0.0095 0.07417 NA FALSE FALSE FALSE
GCST006061 0.0134 0.4784 537409 outcome TRUE reported
7823_22_ST6GALNAC2_SIA7B ST6GALNAC2 chr1 196141224 0.015336 1.275e-09
35337 TRUE reported textfile 2 1 TRUE
3643 rs1047286 A G A G -0.0593 -0.0013 0.24066 NA FALSE FALSE FALSE
GCST006061 0.0098 0.8921 537409 outcome TRUE reported
3192_3_CNDP2_Glutamate_carboxypeptidase CNDP2 chr19 6713251 0.009383 2.612e-10
35372 TRUE reported textfile 2 1 TRUE
3644 rs10922098 T C T C -0.2126 -0.009 0.40011 NA FALSE FALSE FALSE
GCST006061 0.0075 0.2275 537409 outcome TRUE reported
3192_3_CNDP2_Glutamate_carboxypeptidase CNDP2 chr1 196695521 0.008153
6.878e-150 35338 TRUE reported textfile 2 1 TRUE
3645 rs1707652 T C T C 0.0904 0.0081 0.3102 NA FALSE FALSE FALSE
GCST006061 0.0074 0.2701 537409 outcome TRUE reported
3192_3_CNDP2_Glutamate_carboxypeptidase CNDP2 chr3 165761011 0.008652
1.48e-25 35339 TRUE reported textfile 2 1 TRUE
3646 rs35209896 A C A C 0.0895 0.0267 0.09263 NA FALSE FALSE FALSE
GCST006061 0.0355 0.4521 537409 outcome TRUE reported
3192_3_CNDP2_Glutamate_carboxypeptidase CNDP2 chr6 32519009 0.013738
7.279e-11 35439 TRUE reported textfile 2 1 TRUE
3647 rs1047286 A G A G -0.064 -0.0013 0.24066 NA FALSE FALSE FALSE
GCST006061 0.0098 0.8921 537409 outcome TRUE reported 10675_223_TMX2_TMX2
TMX2 chr19 6713251 0.009279 5.289e-12 35360 TRUE reported textfile
2 1 TRUE
3648 rs10922098 T C T C -0.2112 -0.009 0.40011 NA FALSE FALSE FALSE
GCST006061 0.0075 0.2275 537409 outcome TRUE reported 10675_223_TMX2_TMX2
TMX2 chr1 196695521 0.008061 2.688e-151 35326 TRUE reported
textfile 2 1 TRUE
3649 rs114009540 A G A G -0.1279 0.0322 0.03606 NA FALSE FALSE FALSE
GCST006061 0.0299 0.2809 537409 outcome TRUE reported 10675_223_TMX2_TMX2
TMX2 chr1 196158413 0.021506 2.727e-09 35325 TRUE reported
textfile 2 1 TRUE
3650 rs2686395 C T C T -0.1094 -0.0077 0.3101 NA FALSE FALSE FALSE
GCST006061 0.0074 0.2965 537409 outcome TRUE reported 10675_223_TMX2_TMX2
TMX2 chr3 165767231 0.008549 1.709e-37 35327 TRUE reported
textfile 2 1 TRUE
3651 rs1047286 A G A G -0.0565 -0.0013 0.24066 NA FALSE FALSE FALSE
GCST006061 0.0098 0.8921 537409 outcome TRUE reported 12451_62_BACH1_BACH1
BACH1 chr19 6713251 0.009391 1.785e-09 35363 TRUE reported textfile
2 1 TRUE
3652 rs10922098 T C T C -0.2189 -0.009 0.40011 NA FALSE FALSE FALSE
GCST006061 0.0075 0.2275 537409 outcome TRUE reported 12451_62_BACH1_BACH1
BACH1 chr1 196695521 0.008165 2.406e-158 35329 TRUE reported
textfile 2 1 TRUE
3653 rs1707652 T C T C 0.105 0.0081 0.3102 NA FALSE FALSE FALSE
GCST006061 0.0074 0.2701 537409 outcome TRUE reported 12451_62_BACH1_BACH1
BACH1 chr3 165761011 0.00866 7.849e-34 35330 TRUE reported textfile
2 1 TRUE
3654 rs704 A G A G 0.0709 -0.0155 0.49189 NA FALSE FALSE FALSE GCST006061
0.0072 0.03144 537409 outcome TRUE reported 12451_62_BACH1_BACH1 BACH1
chr17 28367840 0.008051 1.287e-18 35351 TRUE reported textfile 2
1 TRUE
3655 rs72748418 A G A G 0.0847 0.0089 0.07164 NA FALSE FALSE FALSE
GCST006061 0.0129 0.4914 537409 outcome TRUE reported 12451_62_BACH1_BACH1
BACH1 chr1 196122755 0.015524 4.864e-08 35328 TRUE reported
textfile 2 1 TRUE
3656 rs1047286 A G A G -0.0535 -0.0013 0.24066 NA FALSE FALSE FALSE
GCST006061 0.0098 0.8921 537409 outcome TRUE reported
10561_5_PGLYRP3_PGRP_I_alpha PGLYRP3 chr19 6713251 0.009317 9.355e-09 35360
TRUE reported textfile 2 1 TRUE
3657 rs10922098 T C T C -0.1593 -0.009 0.40011 NA FALSE FALSE FALSE
GCST006061 0.0075 0.2275 537409 outcome TRUE reported

10561_5_PGLYRP3_PGRP_I_alpha PGLYRP3 chr1 196695521 0.008121 1.126e-85
35326 TRUE reported textfile 2 1 TRUE
3658 rs12478841 G A G A 0.0562 0.0049 0.21569 NA FALSE FALSE FALSE
GCST006061 0.008 0.5413 537409 outcome TRUE reported
10561_5_PGLYRP3_PGRP_I_alpha PGLYRP3 chr2 27588855 0.009611 4.985e-09
35361 TRUE reported textfile 2 1 TRUE
3659 rs141738509 C A C A -0.1795 0.0503 0.02076 NA FALSE FALSE FALSE
GCST006061 0.0361 0.1633 537409 outcome TRUE reported
10561_5_PGLYRP3_PGRP_I_alpha PGLYRP3 chr1 153340595 0.027834 1.126e-10
35351 TRUE reported textfile 2 1 TRUE
3660 rs1047286 A G A G -0.0683 -0.0013 0.24066 NA FALSE FALSE FALSE
GCST006061 0.0098 0.8921 537409 outcome TRUE reported 19247_1_UBE2D1_UB2D1
UBE2D1 chr19 6713251 0.009363 3.001e-13 35372 TRUE reported textfile
2 1 TRUE
3661 rs10922098 T C T C -0.2395 -0.009 0.40011 NA FALSE FALSE FALSE
GCST006061 0.0075 0.2275 537409 outcome TRUE reported 19247_1_UBE2D1_UB2D1
UBE2D1 chr1 196695521 0.008124 5.023e-191 35338 TRUE reported
textfile 2 1 TRUE
3662 rs1451915 A C A C 0.0721 -0.0107 0.13641 NA FALSE FALSE FALSE
GCST006061 0.0124 0.3895 537409 outcome TRUE reported 19247_1_UBE2D1_UB2D1
UBE2D1 chr1 196058002 0.011626 5.591e-10 35335 TRUE reported
textfile 2 1 TRUE
3663 rs1707652 T C T C 0.103 0.0081 0.3102 NA FALSE FALSE FALSE
GCST006061 0.0074 0.2701 537409 outcome TRUE reported 19247_1_UBE2D1_UB2D1
UBE2D1 chr3 165761011 0.008632 7.975e-33 35339 TRUE reported
textfile 2 1 TRUE
3664 rs7513100 C T C T 0.0711 0 0.14871 NA FALSE FALSE FALSE GCST006061
0.0098 0.9981 537409 outcome TRUE reported 19247_1_UBE2D1_UB2D1 UBE2D1
chr1 198065935 0.011246 2.574e-10 35343 TRUE reported textfile 2
1 TRUE
3665 rs1047286 A G A G -0.061 -0.0013 0.24066 NA FALSE FALSE FALSE
GCST006061 0.0098 0.8921 537409 outcome TRUE reported 7888_58_COA3_CCD56
COA3 chr19 6713251 0.009365 7.339e-11 35372 TRUE reported textfile
2 1 TRUE
3666 rs10922098 T C T C -0.2114 -0.009 0.40011 NA FALSE FALSE FALSE
GCST006061 0.0075 0.2275 537409 outcome TRUE reported 7888_58_COA3_CCD56
COA3 chr1 196695521 0.008133 5.764e-149 35338 TRUE reported
textfile 2 1 TRUE
3667 rs2686395 C T C T -0.0917 -0.0077 0.3101 NA FALSE FALSE FALSE
GCST006061 0.0074 0.2965 537409 outcome TRUE reported 7888_58_COA3_CCD56
COA3 chr3 165767231 0.008632 2.313e-26 35339 TRUE reported
textfile 2 1 TRUE
3668 rs1047286 A G A G -0.0683 -0.0013 0.24066 NA FALSE FALSE FALSE
GCST006061 0.0098 0.8921 537409 outcome TRUE reported 18282_1_GNG11_GBG11
GNG11 chr19 6713251 0.009302 2.093e-13 35372 TRUE reported textfile
2 1 TRUE
3669 rs10922098 T C T C -0.2232 -0.009 0.40011 NA FALSE FALSE FALSE
GCST006061 0.0075 0.2275 537409 outcome TRUE reported 18282_1_GNG11_GBG11
GNG11 chr1 196695521 0.008086 1.019e-167 35338 TRUE reported
textfile 2 1 TRUE
3670 rs2686395 C T C T -0.1113 -0.0077 0.3101 NA FALSE FALSE FALSE
GCST006061 0.0074 0.2965 537409 outcome TRUE reported 18282_1_GNG11_GBG11
GNG11 chr3 165767231 0.008583 1.857e-38 35339 TRUE reported
textfile 2 1 TRUE
3671 rs7513100 C T C T 0.0677 0 0.14871 NA FALSE FALSE FALSE GCST006061
0.0098 0.9981 537409 outcome TRUE reported 18282_1_GNG11_GBG11 GNG11 chr1
198065935 0.011177 1.384e-09 35343 TRUE reported textfile 2 1 TRUE
3672 rs1047286 A G A G -0.0569 -0.0013 0.24066 NA FALSE FALSE FALSE
GCST006061 0.0098 0.8921 537409 outcome TRUE reported 13507_51_TRADD_TRADD
TRADD chr19 6713251 0.009429 1.591e-09 35366 TRUE reported textfile
2 1 TRUE
3673 rs10922098 T C T C -0.1822 -0.009 0.40011 NA FALSE FALSE FALSE
GCST006061 0.0075 0.2275 537409 outcome TRUE reported 13507_51_TRADD_TRADD
TRADD chr1 196695521 0.008208 3.571e-109 35332 TRUE reported
textfile 2 1 TRUE
3674 rs111696008 A G A G -0.1617 -0.0259 0.02443 NA FALSE FALSE FALSE

GCST006061 0.0401 0.519 537409 outcome TRUE reported 13507_51_TRADD_TRADD
TRADD chr2 216622986 0.026814 1.635e-09 35353 TRUE reported
textfile 2 1 TRUE
3675 rs1451915 A C A C 0.0654 -0.0107 0.13641 NA FALSE FALSE FALSE
GCST006061 0.0124 0.3895 537409 outcome TRUE reported 13507_51_TRADD_TRADD
TRADD chr1 196058002 0.011693 2.229e-08 35329 TRUE reported
textfile 2 1 TRUE
3676 rs1707652 T C T C 0.1276 0.0081 0.3102 NA FALSE FALSE FALSE
GCST006061 0.0074 0.2701 537409 outcome TRUE reported 13507_51_TRADD_TRADD
TRADD chr3 165761011 0.008683 6.927e-49 35333 TRUE reported
textfile 2 1 TRUE
3677 rs704 A G A G 0.0601 -0.0155 0.49189 NA FALSE FALSE FALSE GCST006061
0.0072 0.03144 537409 outcome TRUE reported 13507_51_TRADD_TRADD TRADD
chr17 28367840 0.008076 9.911e-14 35354 TRUE reported textfile 2
1 TRUE
3678 rs71352239 T C T C 0.0583 -0.0041 0.30263 NA FALSE FALSE FALSE
GCST006061 0.0087 0.6372 537409 outcome TRUE reported 13507_51_TRADD_TRADD
TRADD chr19 44926286 0.008803 3.517e-11 35362 TRUE reported
textfile 2 1 TRUE
3679 rs1047286 A G A G -0.0523 -0.0013 0.24066 NA FALSE FALSE FALSE
GCST006061 0.0098 0.8921 537409 outcome TRUE reported
18158_45_CASP8_Caspase_8 CASP8 chr19 6713251 0.009326 2.049e-08 35372
TRUE reported textfile 2 1 TRUE
3680 rs1706435 G A G A 0.0692 0.0117 0.36981 NA FALSE FALSE FALSE
GCST006061 0.0072 0.1047 537409 outcome TRUE reported
18158_45_CASP8_Caspase_8 CASP8 chr3 165743688 0.008251 5.009e-17 35339
TRUE reported textfile 2 1 TRUE
3681 rs1047286 A G A G -0.0735 -0.0013 0.24066 NA FALSE FALSE FALSE
GCST006061 0.0098 0.8921 537409 outcome TRUE reported 2723_9_IL37_IL_1F7
IL37 chr19 6713251 0.009384 4.787e-15 35372 TRUE reported textfile
2 1 TRUE
3682 rs1329424 G T G T -0.2692 -0.0095 0.39735 NA FALSE FALSE FALSE
GCST006061 0.0075 0.2071 537409 outcome TRUE reported 2723_9_IL37_IL_1F7
IL37 chr1 196677046 0.008152 1e-200 35338 TRUE reported textfile
2 1 TRUE
3683 rs1707652 T C T C 0.1115 0.0081 0.3102 NA FALSE FALSE FALSE
GCST006061 0.0074 0.2701 537409 outcome TRUE reported 2723_9_IL37_IL_1F7
IL37 chr3 165761011 0.008657 5.812e-38 35339 TRUE reported
textfile 2 1 TRUE
3684 rs72748420 T C T C 0.0925 0.0095 0.07417 NA FALSE FALSE FALSE
GCST006061 0.0134 0.4784 537409 outcome TRUE reported 2723_9_IL37_IL_1F7
IL37 chr1 196141224 0.015308 1.516e-09 35337 TRUE reported
textfile 2 1 TRUE
3685 rs1047286 A G A G -0.0554 -0.0013 0.24066 NA FALSE FALSE FALSE
GCST006061 0.0098 0.8921 537409 outcome TRUE reported 6473_55_BPIFA1_PLUNC
BPIFA1 chr19 6713251 0.009353 3.153e-09 35372 TRUE reported textfile
2 1 TRUE
3686 rs10922098 T C T C -0.1656 -0.009 0.40011 NA FALSE FALSE FALSE
GCST006061 0.0075 0.2275 537409 outcome TRUE reported 6473_55_BPIFA1_PLUNC
BPIFA1 chr1 196695521 0.008147 7.505e-92 35338 TRUE reported
textfile 2 1 TRUE
3687 rs2686395 C T C T -0.0858 -0.0077 0.3101 NA FALSE FALSE FALSE
GCST006061 0.0074 0.2965 537409 outcome TRUE reported 6473_55_BPIFA1_PLUNC
BPIFA1 chr3 165767231 0.008633 2.814e-23 35339 TRUE reported
textfile 2 1 TRUE
3688 rs1047286 A G A G -0.0793 -0.0013 0.24066 NA FALSE FALSE FALSE
GCST006061 0.0098 0.8921 537409 outcome TRUE reported
4472_5_TPM2_Tropomyosin_2 TPM2 chr19 6713251 0.009435 4.295e-17 35372
TRUE reported textfile 2 1 TRUE
3689 rs10801660 A G A G 0.0808 -0.0119 0.11574 NA FALSE FALSE FALSE
GCST006061 0.0108 0.2735 537409 outcome TRUE reported
4472_5_TPM2_Tropomyosin_2 TPM2 chr1 198272225 0.012618 1.518e-10 35345
TRUE reported textfile 2 1 TRUE
3690 rs1329424 G T G T -0.2945 -0.0095 0.39735 NA FALSE FALSE FALSE
GCST006061 0.0075 0.2071 537409 outcome TRUE reported
4472_5_TPM2_Tropomyosin_2 TPM2 chr1 196677046 0.008179 1e-200 35338

TRUE reported textfile 2 1 TRUE
3691 rs1707652 T C T C 0.1251 0.0081 0.3102 NA FALSE FALSE FALSE
GCST006061 0.0074 0.2701 537409 outcome TRUE reported
4472_5_TPM2_Tropomyosin_2 TPM2 chr3 165761011 0.008692 5.813e-47 35339
TRUE reported textfile 2 1 TRUE
3692 rs72748420 T C T C 0.1043 0.0095 0.07417 NA FALSE FALSE FALSE
GCST006061 0.0134 0.4784 537409 outcome TRUE reported
4472_5_TPM2_Tropomyosin_2 TPM2 chr1 196141224 0.015413 1.316e-11 35337
TRUE reported textfile 2 1 TRUE
3693 rs1047286 A G A G -0.0568 -0.0013 0.24066 NA FALSE FALSE FALSE
GCST006061 0.0098 0.8921 537409 outcome TRUE reported
16587_1_CSN2_Beta_casein CSN2 chr19 6713251 0.009162 5.652e-10 35372
TRUE reported textfile 2 1 TRUE
3694 rs10922098 T C T C -0.1753 -0.009 0.40011 NA FALSE FALSE FALSE
GCST006061 0.0075 0.2275 537409 outcome TRUE reported
16587_1_CSN2_Beta_casein CSN2 chr1 196695521 0.007963 2.091e-107 35338
TRUE reported textfile 2 1 TRUE
3695 rs114009540 A G A G -0.133 0.0322 0.03606 NA FALSE FALSE FALSE
GCST006061 0.0299 0.2809 537409 outcome TRUE reported
16587_1_CSN2_Beta_casein CSN2 chr1 196158413 0.02118 3.398e-10 35337
TRUE reported textfile 2 1 TRUE
3696 rs2686395 C T C T -0.0738 -0.0077 0.3101 NA FALSE FALSE FALSE
GCST006061 0.0074 0.2965 537409 outcome TRUE reported
16587_1_CSN2_Beta_casein CSN2 chr3 165767231 0.00845 2.455e-18 35339
TRUE reported textfile 2 1 TRUE
3697 rs1047286 A G A G -0.0793 -0.0013 0.24066 NA FALSE FALSE FALSE
GCST006061 0.0098 0.8921 537409 outcome TRUE reported 3182_38_ENTPD1_CD39
ENTPD1 chr19 6713251 0.009425 3.969e-17 35372 TRUE reported textfile
2 1 TRUE
3698 rs10922098 T C T C -0.2253 -0.009 0.40011 NA FALSE FALSE FALSE
GCST006061 0.0075 0.2275 537409 outcome TRUE reported 3182_38_ENTPD1_CD39
ENTPD1 chr1 196695521 0.008191 1.463e-166 35338 TRUE reported
textfile 2 1 TRUE
3699 rs2686395 C T C T -0.089 -0.0077 0.3101 NA FALSE FALSE FALSE
GCST006061 0.0074 0.2965 537409 outcome TRUE reported 3182_38_ENTPD1_CD39
ENTPD1 chr3 165767231 0.008701 1.468e-24 35339 TRUE reported
textfile 2 1 TRUE
3700 rs1047286 A G A G -0.0632 -0.0013 0.24066 NA FALSE FALSE FALSE
GCST006061 0.0098 0.8921 537409 outcome TRUE reported 12761_12_APBB2_APBB2
APBB2 chr19 6713251 0.009295 1.053e-11 35363 TRUE reported textfile
2 1 TRUE
3701 rs11606706 T G T G 0.0596 -0.0054 0.24209 NA FALSE FALSE FALSE
GCST006061 0.0082 0.5083 537409 outcome TRUE reported 12761_12_APBB2_APBB2
APBB2 chr11 57616102 0.00933 1.684e-10 35355 TRUE reported textfile
2 1 TRUE
3702 rs12146727 A G A G -0.2722 -0.0037 0.11875 NA FALSE FALSE FALSE
GCST006061 0.0105 0.7269 537409 outcome TRUE reported 12761_12_APBB2_APBB2
APBB2 chr12 7063032 0.012455 6.898e-106 35381 TRUE reported textfile
2 1 TRUE
3703 rs1047286 A G A G -0.0508 -0.0013 0.24066 NA FALSE FALSE FALSE
GCST006061 0.0098 0.8921 537409 outcome TRUE reported 11570_94_BCAP29_BAP29
BCAP29 chr19 6713251 0.009264 4.173e-08 35363 TRUE reported textfile
2 1 TRUE
3704 rs10922098 T C T C -0.158 -0.009 0.40011 NA FALSE FALSE FALSE
GCST006061 0.0075 0.2275 537409 outcome TRUE reported 11570_94_BCAP29_BAP29
BCAP29 chr1 196695521 0.008061 1.522e-85 35329 TRUE reported
textfile 2 1 TRUE
3705 rs1707652 T C T C 0.0677 0.0081 0.3102 NA FALSE FALSE FALSE
GCST006061 0.0074 0.2701 537409 outcome TRUE reported 11570_94_BCAP29_BAP29
BCAP29 chr3 165761011 0.008544 2.31e-15 35330 TRUE reported
textfile 2 1 TRUE
3706 rs704 A G A G 0.0616 -0.0155 0.49189 NA FALSE FALSE FALSE GCST006061
0.0072 0.03144 537409 outcome TRUE reported 11570_94_BCAP29_BAP29 BCAP29
chr17 28367840 0.007936 8.381e-15 35351 TRUE reported textfile 2
1 TRUE
3707 rs1047286 A G A G -0.056 -0.0013 0.24066 NA FALSE FALSE FALSE

GCST006061 0.0098 0.8921 537409 outcome TRUE reported 13068_139_CCS_CCS CCS chr19 6713251 0.009344 2.055e-09 35363 TRUE reported textfile 2 1 TRUE

3708 rs10922098 T C T C -0.1849 -0.009 0.40011 NA FALSE FALSE FALSE GCST006061 0.0075 0.2275 537409 outcome TRUE reported 13068_139_CCS_CCS CCS chr1 196695521 0.008128 1.483e-114 35329 TRUE reported textfile 2 1 TRUE

3709 rs2686395 C T C T -0.0891 -0.0077 0.3101 NA FALSE FALSE FALSE GCST006061 0.0074 0.2965 537409 outcome TRUE reported 13068_139_CCS_CCS CCS chr3 165767231 0.008612 4.382e-25 35330 TRUE reported textfile 2 1 TRUE

3710 rs498045 G A G A 0.1131 0.0027 0.44838 NA FALSE FALSE FALSE GCST006061 0.0071 0.7059 537409 outcome TRUE reported 13068_139_CCS_CCS CCS chr11 66595526 0.008053 8.306e-45 35354 TRUE reported textfile 2 1 TRUE

3711 rs1047286 A G A G -0.0748 -0.0013 0.24066 NA FALSE FALSE FALSE GCST006061 0.0098 0.8921 537409 outcome TRUE reported 14060_67_NCF4_NCF4 NCF4 chr19 6713251 0.009312 9.558e-16 35372 TRUE reported textfile 2 1 TRUE

3712 rs10922098 T C T C -0.1522 -0.009 0.40011 NA FALSE FALSE FALSE GCST006061 0.0075 0.2275 537409 outcome TRUE reported 14060_67_NCF4_NCF4 NCF4 chr1 196695521 0.008106 1.205e-78 35338 TRUE reported textfile 2 1 TRUE

3713 rs12478841 G A G A 0.0539 0.0049 0.21569 NA FALSE FALSE FALSE GCST006061 0.008 0.5413 537409 outcome TRUE reported 14060_67_NCF4_NCF4 NCF4 chr2 27588855 0.009588 1.895e-08 35373 TRUE reported textfile 2 1 TRUE

3714 rs35209896 A C A C 0.0887 0.0267 0.09263 NA FALSE FALSE FALSE GCST006061 0.0355 0.4521 537409 outcome TRUE reported 14060_67_NCF4_NCF4 NCF4 chr6 32519009 0.013613 7.22e-11 35439 TRUE reported textfile 2 1 TRUE

3715 rs1047286 A G A G -0.0712 -0.0013 0.24066 NA FALSE FALSE FALSE GCST006061 0.0098 0.8921 537409 outcome TRUE reported 14216_35_DDX46_DDX46 DDX46 chr19 6713251 0.009339 2.46e-14 35372 TRUE reported textfile 2 1 TRUE

3716 rs10922098 T C T C -0.2428 -0.009 0.40011 NA FALSE FALSE FALSE GCST006061 0.0075 0.2275 537409 outcome TRUE reported 14216_35_DDX46_DDX46 DDX46 chr1 196695521 0.008103 3.012e-197 35338 TRUE reported textfile 2 1 TRUE

3717 rs11064498 G A G A -0.0716 -8.00E-04 0.1306 NA FALSE FALSE FALSE GCST006061 0.0102 0.939 537409 outcome TRUE reported 14216_35_DDX46_DDX46 DDX46 chr12 7064203 0.012035 2.695e-09 35390 TRUE reported textfile 2 1 TRUE

3718 rs114009540 A G A G -0.1184 0.0322 0.03606 NA FALSE FALSE FALSE GCST006061 0.0299 0.2809 537409 outcome TRUE reported 14216_35_DDX46_DDX46 DDX46 chr1 196158413 0.021675 4.697e-08 35337 TRUE reported textfile 2 1 TRUE

3719 rs1707652 T C T C 0.1192 0.0081 0.3102 NA FALSE FALSE FALSE GCST006061 0.0074 0.2701 537409 outcome TRUE reported 14216_35_DDX46_DDX46 DDX46 chr3 165761011 0.008614 1.503e-43 35339 TRUE reported textfile 2 1 TRUE

3720 rs704 A G A G 0.1123 -0.0155 0.49189 NA FALSE FALSE FALSE GCST006061 0.0072 0.03144 537409 outcome TRUE reported 14216_35_DDX46_DDX46 DDX46 chr17 28367840 0.007995 8.075e-45 35360 TRUE reported textfile 2 1 TRUE

3721 rs73169117 T C T C 0.07 0.0051 0.11405 NA FALSE FALSE FALSE GCST006061 0.0122 0.6755 537409 outcome TRUE reported 14216_35_DDX46_DDX46 DDX46 chr3 167217620 0.012658 3.202e-08 35354 TRUE reported textfile 2 1 TRUE

3722 rs1047286 A G A G -0.0557 -0.0013 0.24066 NA FALSE FALSE FALSE GCST006061 0.0098 0.8921 537409 outcome TRUE reported 12716_3_ZNF175_ZN175 ZNF175 chr19 6713251 0.009349 2.553e-09 35363 TRUE reported textfile 2 1 TRUE

3723 rs10922098 T C T C -0.1753 -0.009 0.40011 NA FALSE FALSE FALSE GCST006061 0.0075 0.2275 537409 outcome TRUE reported 12716_3_ZNF175_ZN175 ZNF175 chr1 196695521 0.00814 7.156e-103 35329 TRUE reported textfile

2 1 TRUE
3724 rs1707652 T C T C 0.0779 0.0081 0.3102 NA FALSE FALSE FALSE GCST006061 0.0074 0.2701 537409 outcome TRUE reported 12716_3_ZNF175_ZN175 ZNF175 chr3 165761011 0.008618 1.583e-19 35330 TRUE reported textfile 2 1 TRUE
3725 rs1047286 A G A G -0.0532 -0.0013 0.24066 NA FALSE FALSE FALSE GCST006061 0.0098 0.8921 537409 outcome TRUE reported 18328_36_PYCR2_P5CR2 PYCR2 chr19 6713251 0.009389 1.46e-08 35372 TRUE reported textfile 2 1 TRUE
3726 rs10922098 T C T C -0.2163 -0.009 0.40011 NA FALSE FALSE FALSE GCST006061 0.0075 0.2275 537409 outcome TRUE reported 18328_36_PYCR2_P5CR2 PYCR2 chr1 196695521 0.008151 3.76e-155 35338 TRUE reported textfile 2 1 TRUE
3727 rs111696008 A G A G -0.1592 -0.0259 0.02443 NA FALSE FALSE FALSE GCST006061 0.0401 0.519 537409 outcome TRUE reported 18328_36_PYCR2_P5CR2 PYCR2 chr2 216622986 0.026668 2.376e-09 35359 TRUE reported textfile 2 1 TRUE
3728 rs1707652 T C T C 0.1312 0.0081 0.3102 NA FALSE FALSE FALSE GCST006061 0.0074 0.2701 537409 outcome TRUE reported 18328_36_PYCR2_P5CR2 PYCR2 chr3 165761011 0.008645 5.102e-52 35339 TRUE reported textfile 2 1 TRUE
3729 rs35209896 A C A C 0.0971 0.0267 0.09263 NA FALSE FALSE FALSE GCST006061 0.0355 0.4521 537409 outcome TRUE reported 18328_36_PYCR2_P5CR2 PYCR2 chr6 32519009 0.013726 1.506e-12 35439 TRUE reported textfile 2 1 TRUE
3730 rs35351085 T C T C 0.1029 0.0061 0.04877 NA FALSE FALSE FALSE GCST006061 0.022 0.7825 537409 outcome TRUE reported 18328_36_PYCR2_P5CR2 PYCR2 chr1 197992799 0.018542 2.862e-08 35343 TRUE reported textfile 2 1 TRUE
3731 rs1047286 A G A G -0.052 -0.0013 0.24066 NA FALSE FALSE FALSE GCST006061 0.0098 0.8921 537409 outcome TRUE reported 10344_334_IL10RA_IL_10_Ra IL10RA chr19 6713251 0.009322 2.432e-08 35360 TRUE reported textfile 2 1 TRUE
3732 rs10733084 C T C T -0.0663 0.006 0.1337 NA FALSE FALSE FALSE GCST006061 0.0097 0.5319 537409 outcome TRUE reported 10344_334_IL10RA_IL_10_Ra IL10RA chr1 196181819 0.011727 1.571e-08 35325 TRUE reported textfile 2 1 TRUE
3733 rs10801660 A G A G 0.0745 -0.0119 0.11574 NA FALSE FALSE FALSE GCST006061 0.0108 0.2735 537409 outcome TRUE reported 10344_334_IL10RA_IL_10_Ra IL10RA chr1 198272225 0.012448 2.168e-09 35333 TRUE reported textfile 2 1 TRUE
3734 rs111696008 A G A G -0.1579 -0.0259 0.02443 NA FALSE FALSE FALSE GCST006061 0.0401 0.519 537409 outcome TRUE reported 10344_334_IL10RA_IL_10_Ra IL10RA chr2 216622986 0.026499 2.544e-09 35347 TRUE reported textfile 2 1 TRUE
3735 rs1329424 G T G T -0.2691 -0.0095 0.39735 NA FALSE FALSE FALSE GCST006061 0.0075 0.2071 537409 outcome TRUE reported 10344_334_IL10RA_IL_10_Ra IL10RA chr1 196677046 0.008091 1e-200 35326 TRUE reported textfile 2 1 TRUE
3736 rs1707652 T C T C 0.1283 0.0081 0.3102 NA FALSE FALSE FALSE GCST006061 0.0074 0.2701 537409 outcome TRUE reported 10344_334_IL10RA_IL_10_Ra IL10RA chr3 165761011 0.008597 2.326e-50 35327 TRUE reported textfile 2 1 TRUE
3737 rs704 A G A G 0.1033 -0.0155 0.49189 NA FALSE FALSE FALSE GCST006061 0.0072 0.03144 537409 outcome TRUE reported 10344_334_IL10RA_IL_10_Ra IL10RA chr17 28367840 0.007976 2.327e-38 35348 TRUE reported textfile 2 1 TRUE
3738 rs1047286 A G A G -0.0574 -0.0013 0.24066 NA FALSE FALSE FALSE GCST006061 0.0098 0.8921 537409 outcome TRUE reported 9310_2_CD8B_CD8B CD8B chr19 6713251 0.009324 7.461e-10 35372 TRUE reported textfile 2 1 TRUE
3739 rs10922098 T C T C -0.2301 -0.009 0.40011 NA FALSE FALSE FALSE GCST006061 0.0075 0.2275 537409 outcome TRUE reported 9310_2_CD8B_CD8B CD8B chr1 196695521 0.008092 7.361e-178 35338 TRUE reported textfile 2 1 TRUE
3740 rs114009540 A G A G -0.119 0.0322 0.03606 NA FALSE FALSE FALSE

GCST006061 0.0299 0.2809 537409 outcome TRUE reported 9310_2_CD8B_CD8B
CD8B chr1 196158413 0.021623 3.725e-08 35337 TRUE reported
textfile 2 1 TRUE
3741 rs1707652 T C T C 0.111 0.0081 0.3102 NA FALSE FALSE FALSE
GCST006061 0.0074 0.2701 537409 outcome TRUE reported 9310_2_CD8B_CD8B
CD8B chr3 165761011 0.008591 3.418e-38 35339 TRUE reported
textfile 2 1 TRUE
3742 rs1047286 A G A G -0.0521 -0.0013 0.24066 NA FALSE FALSE FALSE
GCST006061 0.0098 0.8921 537409 outcome TRUE reported 14226_120_BHMT2_BHMT2
BHMT2 chr19 6713251 0.009306 2.161e-08 35372 TRUE reported textfile
2 1 TRUE
3743 rs10922098 T C T C -0.1779 -0.009 0.40011 NA FALSE FALSE FALSE
GCST006061 0.0075 0.2275 537409 outcome TRUE reported 14226_120_BHMT2_BHMT2
BHMT2 chr1 196695521 0.008099 6.131e-107 35338 TRUE reported
textfile 2 1 TRUE
3744 rs1706435 G A G A 0.0884 0.0117 0.36981 NA FALSE FALSE FALSE
GCST006061 0.0072 0.1047 537409 outcome TRUE reported 14226_120_BHMT2_BHMT2
BHMT2 chr3 165743688 0.008232 6.734e-27 35339 TRUE reported
textfile 2 1 TRUE
3745 rs1047286 A G A G -0.0721 -0.0013 0.24066 NA FALSE FALSE FALSE
GCST006061 0.0098 0.8921 537409 outcome TRUE reported 4540_11_CBX5_CBX5
CBX5 chr19 6713251 0.009342 1.182e-14 35372 TRUE reported textfile
2 1 TRUE
3746 rs10922098 T C T C -0.2334 -0.009 0.40011 NA FALSE FALSE FALSE
GCST006061 0.0075 0.2275 537409 outcome TRUE reported 4540_11_CBX5_CBX5
CBX5 chr1 196695521 0.008106 2.673e-182 35338 TRUE reported
textfile 2 1 TRUE
3747 rs1451915 A C A C 0.0718 -0.0107 0.13641 NA FALSE FALSE FALSE
GCST006061 0.0124 0.3895 537409 outcome TRUE reported 4540_11_CBX5_CBX5
CBX5 chr1 196058002 0.011606 6.154e-10 35335 TRUE reported
textfile 2 1 TRUE
3748 rs1707652 T C T C 0.1128 0.0081 0.3102 NA FALSE FALSE FALSE
GCST006061 0.0074 0.2701 537409 outcome TRUE reported 4540_11_CBX5_CBX5
CBX5 chr3 165761011 0.008614 3.498e-39 35339 TRUE reported
textfile 2 1 TRUE
3749 rs7513100 C T C T 0.0648 0 0.14871 NA FALSE FALSE FALSE GCST006061
0.0098 0.9981 537409 outcome TRUE reported 4540_11_CBX5_CBX5 CBX5 chr1
198065935 0.01121 7.439e-09 35343 TRUE reported textfile 2 1 TRUE
3750 rs1047286 A G A G -0.0535 -0.0013 0.24066 NA FALSE FALSE FALSE
GCST006061 0.0098 0.8921 537409 outcome TRUE reported 13011_20_RPS10_RS10
RPS10 chr19 6713251 0.009434 1.419e-08 35363 TRUE reported textfile
2 1 TRUE
3751 rs10801660 A G A G 0.085 -0.0119 0.11574 NA FALSE FALSE FALSE
GCST006061 0.0108 0.2735 537409 outcome TRUE reported 13011_20_RPS10_RS10
RPS10 chr1 198272225 0.01263 1.694e-11 35336 TRUE reported textfile
2 1 TRUE
3752 rs111696008 A G A G -0.1575 -0.0259 0.02443 NA FALSE FALSE FALSE
GCST006061 0.0401 0.519 537409 outcome TRUE reported 13011_20_RPS10_RS10
RPS10 chr2 216622986 0.026847 4.446e-09 35350 TRUE reported
textfile 2 1 TRUE
3753 rs1329424 G T G T -0.2926 -0.0095 0.39735 NA FALSE FALSE FALSE
GCST006061 0.0075 0.2071 537409 outcome TRUE reported 13011_20_RPS10_RS10
RPS10 chr1 196677046 0.008187 1e-200 35329 TRUE reported textfile
2 1 TRUE
3754 rs1707652 T C T C 0.1385 0.0081 0.3102 NA FALSE FALSE FALSE
GCST006061 0.0074 0.2701 537409 outcome TRUE reported 13011_20_RPS10_RS10
RPS10 chr3 165761011 0.0087 4.7e-57 35330 TRUE reported textfile 2
1 TRUE
3755 rs72748420 T C T C 0.1043 0.0095 0.07417 NA FALSE FALSE FALSE
GCST006061 0.0134 0.4784 537409 outcome TRUE reported 13011_20_RPS10_RS10
RPS10 chr1 196141224 0.015421 1.347e-11 35328 TRUE reported
textfile 2 1 TRUE
3756 rs1047286 A G A G -0.0721 -0.0013 0.24066 NA FALSE FALSE FALSE
GCST006061 0.0098 0.8921 537409 outcome TRUE reported 11380_84_RBM24_RBM24
RBM24 chr19 6713251 0.009325 1.061e-14 35363 TRUE reported textfile
2 1 TRUE

3757 rs10922098 T C T C -0.2331 -0.009 0.40011 NA FALSE FALSE FALSE GCST006061 0.0075 0.2275 537409 outcome TRUE reported 11380_84_RBM24_RBM24 RBM24 chr1 196695521 0.008092 1.721e-182 35329 TRUE reported textfile 2 1 TRUE

3758 rs114009540 A G A G -0.127 0.0322 0.03606 NA FALSE FALSE FALSE GCST006061 0.0299 0.2809 537409 outcome TRUE reported 11380_84_RBM24_RBM24 RBM24 chr1 196158413 0.021616 4.219e-09 35328 TRUE reported textfile 2 1 TRUE

3759 rs2686395 C T C T -0.0993 -0.0077 0.3101 NA FALSE FALSE FALSE GCST006061 0.0074 0.2965 537409 outcome TRUE reported 11380_84_RBM24_RBM24 RBM24 chr3 165767231 0.008596 7.186e-31 35330 TRUE reported textfile 2 1 TRUE

3760 rs1047286 A G A G -0.07 -0.0013 0.24066 NA FALSE FALSE FALSE GCST006061 0.0098 0.8921 537409 outcome TRUE reported 14229_5_MOCS3_MOCS3 MOCS3 chr19 6713251 0.009361 7.542e-14 35372 TRUE reported textfile 2 1 TRUE

3761 rs111696008 A G A G -0.1503 -0.0259 0.02443 NA FALSE FALSE FALSE GCST006061 0.0401 0.519 537409 outcome TRUE reported 14229_5_MOCS3_MOCS3 MOCS3 chr2 216622986 0.026626 1.653e-08 35359 TRUE reported textfile 2 1 TRUE

3762 rs114009540 A G A G -0.1229 0.0322 0.03606 NA FALSE FALSE FALSE GCST006061 0.0299 0.2809 537409 outcome TRUE reported 14229_5_MOCS3_MOCS3 MOCS3 chr1 196158413 0.02174 1.576e-08 35337 TRUE reported textfile 2 1 TRUE

3763 rs12629202 A C A C 0.0479 0.0052 0.31116 NA FALSE FALSE FALSE GCST006061 0.0081 0.5213 537409 outcome TRUE reported 14229_5_MOCS3_MOCS3 MOCS3 chr3 167083477 0.008657 3.151e-08 35354 TRUE reported textfile 2 1 TRUE

3764 rs1329424 G T G T -0.2527 -0.0095 0.39735 NA FALSE FALSE FALSE GCST006061 0.0075 0.2071 537409 outcome TRUE reported 14229_5_MOCS3_MOCS3 MOCS3 chr1 196677046 0.008136 1e-200 35338 TRUE reported textfile 2 1 TRUE

3765 rs2686395 C T C T -0.1246 -0.0077 0.3101 NA FALSE FALSE FALSE GCST006061 0.0074 0.2965 537409 outcome TRUE reported 14229_5_MOCS3_MOCS3 MOCS3 chr3 165767231 0.008634 3.252e-47 35339 TRUE reported textfile 2 1 TRUE

3766 rs35351085 T C T C 0.1249 0.0061 0.04877 NA FALSE FALSE FALSE GCST006061 0.022 0.7825 537409 outcome TRUE reported 14229_5_MOCS3_MOCS3 MOCS3 chr1 197992799 0.018545 1.638e-11 35343 TRUE reported textfile 2 1 TRUE

3767 rs1047286 A G A G -0.0608 -0.0013 0.24066 NA FALSE FALSE FALSE GCST006061 0.0098 0.8921 537409 outcome TRUE reported 11682_7_TXNIP_TXNIP TXNIP chr19 6713251 0.009348 7.818e-11 35363 TRUE reported textfile 2 1 TRUE

3768 rs10922098 T C T C -0.2233 -0.009 0.40011 NA FALSE FALSE FALSE GCST006061 0.0075 0.2275 537409 outcome TRUE reported 11682_7_TXNIP_TXNIP TXNIP chr1 196695521 0.008123 2.259e-166 35329 TRUE reported textfile 2 1 TRUE

3769 rs114009540 A G A G -0.1321 0.0322 0.03606 NA FALSE FALSE FALSE GCST006061 0.0299 0.2809 537409 outcome TRUE reported 11682_7_TXNIP_TXNIP TXNIP chr1 196158413 0.021687 1.12e-09 35328 TRUE reported textfile 2 1 TRUE

3770 rs1707652 T C T C 0.0977 0.0081 0.3102 NA FALSE FALSE FALSE GCST006061 0.0074 0.2701 537409 outcome TRUE reported 11682_7_TXNIP_TXNIP TXNIP chr3 165761011 0.008627 9.819e-30 35330 TRUE reported textfile 2 1 TRUE

3771 rs1047286 A G A G -0.069 -0.0013 0.24066 NA FALSE FALSE FALSE GCST006061 0.0098 0.8921 537409 outcome TRUE reported 6992_67_HBD_HBD HBD chr19 6713251 0.009366 1.748e-13 35372 TRUE reported textfile 2 1 TRUE

3772 rs10922098 T C T C -0.2292 -0.009 0.40011 NA FALSE FALSE FALSE GCST006061 0.0075 0.2275 537409 outcome TRUE reported 6992_67_HBD_HBD HBD chr1 196695521 0.008134 1.065e-174 35338 TRUE reported textfile 2 1 TRUE

3773 rs114009540 A G A G -0.1196 0.0322 0.03606 NA FALSE FALSE FALSE GCST006061 0.0299 0.2809 537409 outcome TRUE reported 6992_67_HBD_HBD HBD

chr1 196158413 0.021733 3.728e-08 35337 TRUE reported textfile 2
1 TRUE
3774 rs2686395 C T C T -0.1039 -0.0077 0.3101 NA FALSE FALSE FALSE
GCST006061 0.0074 0.2965 537409 outcome TRUE reported 6992_67_HBD_HBD HBD
chr3 165767231 0.008637 2.468e-33 35339 TRUE reported textfile 2
1 TRUE
3775 rs704 A G A G 0.0626 -0.0155 0.49189 NA FALSE FALSE FALSE GCST006061
0.0072 0.03144 537409 outcome TRUE reported 6992_67_HBD_HBD HBD chr17
28367840 0.008022 6.024e-15 35360 TRUE reported textfile 2 1 TRUE
3776 rs1047286 A G A G -0.0542 -0.0013 0.24066 NA FALSE FALSE FALSE
GCST006061 0.0098 0.8921 537409 outcome TRUE reported
14121_24_TNFRSF10D_TRAIL_R4 TNFRSF10D chr19 6713251 0.009302 5.643e-09 35372
TRUE reported textfile 2 1 TRUE
3777 rs10922098 T C T C -0.1292 -0.009 0.40011 NA FALSE FALSE FALSE
GCST006061 0.0075 0.2275 537409 outcome TRUE reported
14121_24_TNFRSF10D_TRAIL_R4 TNFRSF10D chr1 196695521 0.008112 4.166e-57
35338 TRUE reported textfile 2 1 TRUE
3778 rs1706435 G A G A 0.0636 0.0117 0.36981 NA FALSE FALSE FALSE
GCST006061 0.0072 0.1047 537409 outcome TRUE reported
14121_24_TNFRSF10D_TRAIL_R4 TNFRSF10D chr3 165743688 0.00823 1.092e-14 35339
TRUE reported textfile 2 1 TRUE
3779 rs7638018 G A G A 0.0752 -0.0016 0.27033 NA FALSE FALSE FALSE
GCST006061 0.0075 0.8261 537409 outcome TRUE reported
14121_24_TNFRSF10D_TRAIL_R4 TNFRSF10D chr3 133776617 0.008967 5.02e-17
35359 TRUE reported textfile 2 1 TRUE
3780 rs1047286 A G A G -0.069 -0.0013 0.24066 NA FALSE FALSE FALSE
GCST006061 0.0098 0.8921 537409 outcome TRUE reported 15358_28_SCG5_7B2
SCG5 chr19 6713251 0.009466 3.113e-13 35372 TRUE reported textfile
2 1 TRUE
3781 rs10801660 A G A G 0.0835 -0.0119 0.11574 NA FALSE FALSE FALSE
GCST006061 0.0108 0.2735 537409 outcome TRUE reported 15358_28_SCG5_7B2
SCG5 chr1 198272225 0.012661 4.252e-11 35345 TRUE reported
textfile 2 1 TRUE
3782 rs111696008 A G A G -0.1536 -0.0259 0.02443 NA FALSE FALSE FALSE
GCST006061 0.0401 0.519 537409 outcome TRUE reported 15358_28_SCG5_7B2
SCG5 chr2 216622986 0.026912 1.146e-08 35359 TRUE reported
textfile 2 1 TRUE
3783 rs1329424 G T G T -0.3043 -0.0095 0.39735 NA FALSE FALSE FALSE
GCST006061 0.0075 0.2071 537409 outcome TRUE reported 15358_28_SCG5_7B2
SCG5 chr1 196677046 0.008204 1e-200 35338 TRUE reported textfile
2 1 TRUE
3784 rs1707652 T C T C 0.1262 0.0081 0.3102 NA FALSE FALSE FALSE
GCST006061 0.0074 0.2701 537409 outcome TRUE reported 15358_28_SCG5_7B2
SCG5 chr3 165761011 0.008725 2.016e-47 35339 TRUE reported
textfile 2 1 TRUE
3785 rs72748420 T C T C 0.1065 0.0095 0.07417 NA FALSE FALSE FALSE
GCST006061 0.0134 0.4784 537409 outcome TRUE reported 15358_28_SCG5_7B2
SCG5 chr1 196141224 0.015467 5.758e-12 35337 TRUE reported
textfile 2 1 TRUE
3786 rs1047286 A G A G -0.0555 -0.0013 0.24066 NA FALSE FALSE FALSE
GCST006061 0.0098 0.8921 537409 outcome TRUE reported 7864_3_SIGLEC8_SIGL8
SIGLEC8 chr19 6713251 0.009295 2.358e-09 35372 TRUE reported textfile
2 1 TRUE
3787 rs572515 G A G A -0.1776 -0.0113 0.3973 NA FALSE FALSE FALSE
GCST006061 0.0075 0.1355 537409 outcome TRUE reported 7864_3_SIGLEC8_SIGL8
SIGLEC8 chr1 196677131 0.008097 1.255e-106 35338 TRUE reported
textfile 2 1 TRUE
3788 rs697357 G A G A 0.0709 0.0156 0.38884 NA FALSE FALSE FALSE
GCST006061 0.0072 0.03066 537409 outcome TRUE reported 7864_3_SIGLEC8_SIGL8
SIGLEC8 chr3 165718484 0.008136 2.937e-18 35339 TRUE reported
textfile 2 1 TRUE
3789 rs704 A G A G 0.0481 -0.0155 0.49189 NA FALSE FALSE FALSE GCST006061
0.0072 0.03144 537409 outcome TRUE reported 7864_3_SIGLEC8_SIGL8 SIGLEC8
chr17 28367840 0.007968 1.577e-09 35360 TRUE reported textfile 2
1 TRUE
3790 rs1047286 A G A G -0.0632 -0.0013 0.24066 NA FALSE FALSE FALSE

GCST006061 0.0098 0.8921 537409 outcome TRUE reported 7910_41_LRFN1_LRFN1
LRFN1 chr19 6713251 0.009412 1.887e-11 35372 TRUE reported textfile
2 1 TRUE
3791 rs10922098 T C T C -0.213 -0.009 0.40011 NA FALSE FALSE FALSE
GCST006061 0.0075 0.2275 537409 outcome TRUE reported 7910_41_LRFN1_LRFN1
LRFN1 chr1 196695521 0.00818 1.843e-149 35338 TRUE reported textfile
2 1 TRUE
3792 rs111696008 A G A G -0.1496 -0.0259 0.02443 NA FALSE FALSE FALSE
GCST006061 0.0401 0.519 537409 outcome TRUE reported 7910_41_LRFN1_LRFN1
LRFN1 chr2 216622986 0.026755 2.251e-08 35359 TRUE reported
textfile 2 1 TRUE
3793 rs1451915 A C A C 0.0726 -0.0107 0.13641 NA FALSE FALSE FALSE
GCST006061 0.0124 0.3895 537409 outcome TRUE reported 7910_41_LRFN1_LRFN1
LRFN1 chr1 196058002 0.011677 5.051e-10 35335 TRUE reported
textfile 2 1 TRUE
3794 rs2686395 C T C T -0.0967 -0.0077 0.3101 NA FALSE FALSE FALSE
GCST006061 0.0074 0.2965 537409 outcome TRUE reported 7910_41_LRFN1_LRFN1
LRFN1 chr3 165767231 0.008676 7.57e-29 35339 TRUE reported
textfile 2 1 TRUE
3795 rs1047286 A G A G -0.0732 -0.0013 0.24066 NA FALSE FALSE FALSE
GCST006061 0.0098 0.8921 537409 outcome TRUE reported 9081_39_SLC30A3_ZNT3
SLC30A3 chr19 6713251 0.009442 9.027e-15 35372 TRUE reported textfile
2 1 TRUE
3796 rs10922098 T C T C -0.2231 -0.009 0.40011 NA FALSE FALSE FALSE
GCST006061 0.0075 0.2275 537409 outcome TRUE reported 9081_39_SLC30A3_ZNT3
SLC30A3 chr1 196695521 0.008201 5.579e-163 35338 TRUE reported
textfile 2 1 TRUE
3797 rs111696008 A G A G -0.1522 -0.0259 0.02443 NA FALSE FALSE FALSE
GCST006061 0.0401 0.519 537409 outcome TRUE reported 9081_39_SLC30A3_ZNT3
SLC30A3 chr2 216622986 0.026847 1.435e-08 35359 TRUE reported
textfile 2 1 TRUE
3798 rs1707652 T C T C 0.1067 0.0081 0.3102 NA FALSE FALSE FALSE
GCST006061 0.0074 0.2701 537409 outcome TRUE reported 9081_39_SLC30A3_ZNT3
SLC30A3 chr3 165761011 0.008705 1.527e-34 35339 TRUE reported
textfile 2 1 TRUE
3799 rs35351085 T C T C 0.1063 0.0061 0.04877 NA FALSE FALSE FALSE
GCST006061 0.022 0.7825 537409 outcome TRUE reported 9081_39_SLC30A3_ZNT3
SLC30A3 chr1 197992799 0.018672 1.249e-08 35343 TRUE reported
textfile 2 1 TRUE
3800 rs1047286 A G A G -0.0574 -0.0013 0.24066 NA FALSE FALSE FALSE
GCST006061 0.0098 0.8921 537409 outcome TRUE reported 6932_42_ITGA5_ITA5
ITGA5 chr19 6713251 0.009424 1.124e-09 35372 TRUE reported textfile
2 1 TRUE
3801 rs10922098 T C T C -0.2005 -0.009 0.40011 NA FALSE FALSE FALSE
GCST006061 0.0075 0.2275 537409 outcome TRUE reported 6932_42_ITGA5_ITA5
ITGA5 chr1 196695521 0.008198 4.284e-132 35338 TRUE reported
textfile 2 1 TRUE
3802 rs1707652 T C T C 0.0974 0.0081 0.3102 NA FALSE FALSE FALSE
GCST006061 0.0074 0.2701 537409 outcome TRUE reported 6932_42_ITGA5_ITA5
ITGA5 chr3 165761011 0.008693 3.876e-29 35339 TRUE reported
textfile 2 1 TRUE
3803 rs1047286 A G A G -0.0602 -0.0013 0.24066 NA FALSE FALSE FALSE
GCST006061 0.0098 0.8921 537409 outcome TRUE reported 10817_26_TOMM20L_TO20L
TOMM20L chr19 6713251 0.00937 1.323e-10 35360 TRUE reported textfile 2
1 TRUE
3804 rs10801660 A G A G 0.0756 -0.0119 0.11574 NA FALSE FALSE FALSE
GCST006061 0.0108 0.2735 537409 outcome TRUE reported 10817_26_TOMM20L_TO20L
TOMM20L chr1 198272225 0.012505 1.49e-09 35333 TRUE reported
textfile 2 1 TRUE
3805 rs1329424 G T G T -0.2631 -0.0095 0.39735 NA FALSE FALSE FALSE
GCST006061 0.0075 0.2071 537409 outcome TRUE reported 10817_26_TOMM20L_TO20L
TOMM20L chr1 196677046 0.008137 1e-200 35326 TRUE reported textfile
2 1 TRUE
3806 rs2686395 C T C T -0.1214 -0.0077 0.3101 NA FALSE FALSE FALSE
GCST006061 0.0074 0.2965 537409 outcome TRUE reported 10817_26_TOMM20L_TO20L
TOMM20L chr3 165767231 0.008636 6.916e-45 35327 TRUE reported

```
textfile	2	1	TRUE
3807	rs72748420	T	C	T	C	0.0851	0.0095	0.07417	NA	FALSE	FALSE	FALSE
GCST006061	0.0134	0.4784	537409	outcome	TRUE	reported	10817_26_TOMM20L_TO20L
TOMM20L	chr1	196141224	0.015288	2.597e-08	35325	TRUE	reported
textfile	2	1	TRUE
3808	rs1047286	A	G	A	G	-0.063	-0.0013	0.24066	NA	FALSE	FALSE	FALSE
GCST006061	0.0098	0.8921	537409	outcome	TRUE	reported
3199_54_KLK12_kallikrein_12	KLK12	chr19	6713251	0.009467	2.844e-11	35372
TRUE	reported	textfile	2	1	TRUE
3809	rs10801660	A	G	A	G	0.077	-0.0119	0.11574	NA	FALSE	FALSE	FALSE
GCST006061	0.0108	0.2735	537409	outcome	TRUE	reported
3199_54_KLK12_kallikrein_12	KLK12	chr1	198272225	0.012646	1.138e-09	35345
TRUE	reported	textfile	2	1	TRUE
3810	rs1329424	G	T	G	T	-0.2926	-0.0095	0.39735	NA	FALSE	FALSE	FALSE
GCST006061	0.0075	0.2071	537409	outcome	TRUE	reported
3199_54_KLK12_kallikrein_12	KLK12	chr1	196677046	0.008203	1e-200	35338
TRUE	reported	textfile	2	1	TRUE
3811	rs1707652	T	C	T	C	0.1316	0.0081	0.3102	NA	FALSE	FALSE	FALSE
GCST006061	0.0074	0.2701	537409	outcome	TRUE	reported
3199_54_KLK12_kallikrein_12	KLK12	chr3	165761011	0.008715	1.589e-51	35339
TRUE	reported	textfile	2	1	TRUE
3812	rs2161196	C	T	C	T	-0.08	-0.0073	0.08129	NA	FALSE	FALSE	FALSE
GCST006061	0.0124	0.5591	537409	outcome	TRUE	reported
3199_54_KLK12_kallikrein_12	KLK12	chr5	115304767	0.014556	3.885e-08	35350
TRUE	reported	textfile	2	1	TRUE
3813	rs3745540	G	A	G	A	-0.1384	-0.0036	0.41851	NA	FALSE	FALSE	FALSE
GCST006061	0.0076	0.6316	537409	outcome	TRUE	reported
3199_54_KLK12_kallikrein_12	KLK12	chr19	51031874	0.008253	4.12e-63	35365
TRUE	reported	textfile	2	1	TRUE
3814	rs3747843	G	A	G	A	-0.0443	-0.0037	0.47828	NA	FALSE	FALSE	FALSE
GCST006061	0.0071	0.6026	537409	outcome	TRUE	reported
3199_54_KLK12_kallikrein_12	KLK12	chr9	121152028	0.008108	4.656e-08	35373
TRUE	reported	textfile	2	1	TRUE
3815	rs61821527	T	G	T	G	-0.0495	-0.006	0.33477	NA	FALSE	FALSE	FALSE
GCST006061	0.0076	0.4251	537409	outcome	TRUE	reported
3199_54_KLK12_kallikrein_12	KLK12	chr1	201978878	0.008606	8.815e-09	35355
TRUE	reported	textfile	2	1	TRUE
3816	rs72748420	T	C	T	C	0.0975	0.0095	0.07417	NA	FALSE	FALSE	FALSE
GCST006061	0.0134	0.4784	537409	outcome	TRUE	reported
3199_54_KLK12_kallikrein_12	KLK12	chr1	196141224	0.015439	2.696e-10	35337
TRUE	reported	textfile	2	1	TRUE
3817	rs1047286	A	G	A	G	-0.0668	-0.0013	0.24066	NA	FALSE	FALSE	FALSE
GCST006061	0.0098	0.8921	537409	outcome	TRUE	reported	9022_49_DEFB132_DB132
DEFB132	chr19	6713251	0.009417	1.304e-12	35372	TRUE	reported	textfile
2	1	TRUE
3818	rs10801660	A	G	A	G	0.0804	-0.0119	0.11574	NA	FALSE	FALSE	FALSE
GCST006061	0.0108	0.2735	537409	outcome	TRUE	reported	9022_49_DEFB132_DB132
DEFB132	chr1	198272225	0.012596	1.738e-10	35345	TRUE	reported
textfile	2	1	TRUE
3819	rs111696008	A	G	A	G	-0.1519	-0.0259	0.02443	NA	FALSE	FALSE	FALSE
GCST006061	0.0401	0.519	537409	outcome	TRUE	reported	9022_49_DEFB132_DB132
DEFB132	chr2	216622986	0.026778	1.406e-08	35359	TRUE	reported
textfile	2	1	TRUE
3820	rs1329424	G	T	G	T	-0.2898	-0.0095	0.39735	NA	FALSE	FALSE	FALSE
GCST006061	0.0075	0.2071	537409	outcome	TRUE	reported	9022_49_DEFB132_DB132
DEFB132	chr1	196677046	0.008169	1e-200	35338	TRUE	reported	textfile
2	1	TRUE
3821	rs1707652	T	C	T	C	0.1252	0.0081	0.3102	NA	FALSE	FALSE	FALSE
GCST006061	0.0074	0.2701	537409	outcome	TRUE	reported	9022_49_DEFB132_DB132
DEFB132	chr3	165761011	0.008688	4.416e-47	35339	TRUE	reported
textfile	2	1	TRUE
3822	rs704	A	G	A	G	0.051	-0.0155	0.49189	NA	FALSE	FALSE	FALSE	GCST006061
0.0072	0.03144	537409	outcome	TRUE	reported	9022_49_DEFB132_DB132	DEFB132
chr17	28367840	0.008079	2.741e-10	35360	TRUE	reported	textfile	2
1	TRUE
3823	rs72748420	T	C	T	C	0.1012	0.0095	0.07417	NA	FALSE	FALSE	FALSE
```

GCST006061 0.0134 0.4784 537409 outcome TRUE reported 9022_49_DEFB132_DB132
DEFB132 chr1 196141224 0.015385 4.767e-11 35337 TRUE reported
textfile 2 1 TRUE
3824 rs1047286 A G A G -0.0535 -0.0013 0.24066 NA FALSE FALSE FALSE
GCST006061 0.0098 0.8921 537409 outcome TRUE reported 7850_1_COX4I2_COX42
COX4I2 chr19 6713251 0.009379 1.167e-08 35372 TRUE reported textfile
2 1 TRUE
3825 rs111696008 A G A G -0.1598 -0.0259 0.02443 NA FALSE FALSE FALSE
GCST006061 0.0401 0.519 537409 outcome TRUE reported 7850_1_COX4I2_COX42
COX4I2 chr2 216622986 0.026649 2.016e-09 35359 TRUE reported
textfile 2 1 TRUE
3826 rs114009540 A G A G -0.1297 0.0322 0.03606 NA FALSE FALSE FALSE
GCST006061 0.0299 0.2809 537409 outcome TRUE reported 7850_1_COX4I2_COX42
COX4I2 chr1 196158413 0.021779 2.598e-09 35337 TRUE reported
textfile 2 1 TRUE
3827 rs1329424 G T G T -0.2698 -0.0095 0.39735 NA FALSE FALSE FALSE
GCST006061 0.0075 0.2071 537409 outcome TRUE reported 7850_1_COX4I2_COX42
COX4I2 chr1 196677046 0.008135 1e-200 35338 TRUE reported textfile
2 1 TRUE
3828 rs1707652 T C T C 0.1235 0.0081 0.3102 NA FALSE FALSE FALSE
GCST006061 0.0074 0.2701 537409 outcome TRUE reported 7850_1_COX4I2_COX42
COX4I2 chr3 165761011 0.008639 2.308e-46 35339 TRUE reported
textfile 2 1 TRUE
3829 rs72748420 T C T C 0.0888 0.0095 0.07417 NA FALSE FALSE FALSE
GCST006061 0.0134 0.4784 537409 outcome TRUE reported 7850_1_COX4I2_COX42
COX4I2 chr1 196141224 0.015292 6.362e-09 35337 TRUE reported
textfile 2 1 TRUE
3830 rs1047286 A G A G -0.0619 -0.0013 0.24066 NA FALSE FALSE FALSE
GCST006061 0.0098 0.8921 537409 outcome TRUE reported 14633_26_TMEM185A_T185A
TMEM185A chr19 6713251 0.009384 4.208e-11 35372 TRUE reported
textfile 2 1 TRUE
3831 rs10922098 T C T C -0.2323 -0.009 0.40011 NA FALSE FALSE FALSE
GCST006061 0.0075 0.2275 537409 outcome TRUE reported 14633_26_TMEM185A_T185A
TMEM185A chr1 196695521 0.008145 6.419e-179 35338 TRUE reported
textfile 2 1 TRUE
3832 rs12629202 A C A C 0.048 0.0052 0.31116 NA FALSE FALSE FALSE
GCST006061 0.0081 0.5213 537409 outcome TRUE reported 14633_26_TMEM185A_T185A
TMEM185A chr3 167083477 0.008674 3.132e-08 35354 TRUE reported
textfile 2 1 TRUE
3833 rs1451915 A C A C 0.0694 -0.0107 0.13641 NA FALSE FALSE FALSE
GCST006061 0.0124 0.3895 537409 outcome TRUE reported 14633_26_TMEM185A_T185A
TMEM185A chr1 196058002 0.011646 2.538e-09 35335 TRUE reported
textfile 2 1 TRUE
3834 rs1707652 T C T C 0.1176 0.0081 0.3102 NA FALSE FALSE FALSE
GCST006061 0.0074 0.2701 537409 outcome TRUE reported 14633_26_TMEM185A_T185A
TMEM185A chr3 165761011 0.008646 3.899e-42 35339 TRUE reported
textfile 2 1 TRUE
3835 rs704 A G A G 0.0946 -0.0155 0.49189 NA FALSE FALSE FALSE GCST006061
0.0072 0.03144 537409 outcome TRUE reported 14633_26_TMEM185A_T185A TMEM185A
chr17 28367840 0.008029 4.814e-32 35360 TRUE reported textfile 2
1 TRUE
3836 rs7513100 C T C T 0.0675 0 0.14871 NA FALSE FALSE FALSE GCST006061
0.0098 0.9981 537409 outcome TRUE reported 14633_26_TMEM185A_T185A TMEM185A
chr1 198065935 0.011268 2.092e-09 35343 TRUE reported textfile 2
1 TRUE
3837 rs1047286 A G A G -0.0731 -0.0013 0.24066 NA FALSE FALSE FALSE
GCST006061 0.0098 0.8921 537409 outcome TRUE reported 2640_3_MST1R_MSP_R
MST1R chr19 6713251 0.009366 5.967e-15 35372 TRUE reported textfile
2 1 TRUE
3838 rs10922098 T C T C -0.2443 -0.009 0.40011 NA FALSE FALSE FALSE
GCST006061 0.0075 0.2275 537409 outcome TRUE reported 2640_3_MST1R_MSP_R
MST1R chr1 196695521 0.008127 1.684e-198 35338 TRUE reported
textfile 2 1 TRUE
3839 rs114009540 A G A G -0.1234 0.0322 0.03606 NA FALSE FALSE FALSE
GCST006061 0.0299 0.2809 537409 outcome TRUE reported 2640_3_MST1R_MSP_R
MST1R chr1 196158413 0.021741 1.38e-08 35337 TRUE reported

textfile 2 1 TRUE
3840 rs1707652 T C T C 0.099 0.0081 0.3102 NA FALSE FALSE FALSE
GCST006061 0.0074 0.2701 537409 outcome TRUE reported 2640_3_MST1R_MSP_R
MST1R chr3 165761011 0.008646 2.331e-30 35339 TRUE reported
textfile 2 1 TRUE
3841 rs1047286 A G A G -0.0524 -0.0013 0.24066 NA FALSE FALSE FALSE
GCST006061 0.0098 0.8921 537409 outcome TRUE reported 10814_7_CD1A_CD1A
CD1A chr19 6713251 0.009325 1.919e-08 35360 TRUE reported textfile
2 1 TRUE
3842 rs10922098 T C T C -0.2251 -0.009 0.40011 NA FALSE FALSE FALSE
GCST006061 0.0075 0.2275 537409 outcome TRUE reported 10814_7_CD1A_CD1A
CD1A chr1 196695521 0.008098 4.8e-170 35326 TRUE reported
textfile 2 1 TRUE
3843 rs111696008 A G A G -0.1487 -0.0259 0.02443 NA FALSE FALSE FALSE
GCST006061 0.0401 0.519 537409 outcome TRUE reported 10814_7_CD1A_CD1A
CD1A chr2 216622986 0.026512 2.037e-08 35347 TRUE reported
textfile 2 1 TRUE
3844 rs114009540 A G A G -0.1209 0.0322 0.03606 NA FALSE FALSE FALSE
GCST006061 0.0299 0.2809 537409 outcome TRUE reported 10814_7_CD1A_CD1A
CD1A chr1 196158413 0.021621 2.248e-08 35325 TRUE reported
textfile 2 1 TRUE
3845 rs1707652 T C T C 0.1218 0.0081 0.3102 NA FALSE FALSE FALSE
GCST006061 0.0074 0.2701 537409 outcome TRUE reported 10814_7_CD1A_CD1A
CD1A chr3 165761011 0.008593 1.307e-45 35327 TRUE reported
textfile 2 1 TRUE
3846 rs1047286 A G A G -0.053 -0.0013 0.24066 NA FALSE FALSE FALSE
GCST006061 0.0098 0.8921 537409 outcome TRUE reported 10582_36_POMK_SG196
POMK chr19 6713251 0.009273 1.093e-08 35360 TRUE reported textfile
2 1 TRUE
3847 rs10922098 T C T C -0.1699 -0.009 0.40011 NA FALSE FALSE FALSE
GCST006061 0.0075 0.2275 537409 outcome TRUE reported 10582_36_POMK_SG196
POMK chr1 196695521 0.008066 1.745e-98 35326 TRUE reported
textfile 2 1 TRUE
3848 rs2686395 C T C T -0.0861 -0.0077 0.3101 NA FALSE FALSE FALSE
GCST006061 0.0074 0.2965 537409 outcome TRUE reported 10582_36_POMK_SG196
POMK chr3 165767231 0.008551 7.534e-24 35327 TRUE reported
textfile 2 1 TRUE
3849 rs1047286 A G A G -0.0589 -0.0013 0.24066 NA FALSE FALSE FALSE
GCST006061 0.0098 0.8921 537409 outcome TRUE reported 2871_73_RAD51_RAD51
RAD51 chr19 6713251 0.009358 3.091e-10 35372 TRUE reported textfile
2 1 TRUE
3850 rs10801660 A G A G 0.0798 -0.0119 0.11574 NA FALSE FALSE FALSE
GCST006061 0.0108 0.2735 537409 outcome TRUE reported 2871_73_RAD51_RAD51
RAD51 chr1 198272225 0.012512 1.793e-10 35345 TRUE reported
textfile 2 1 TRUE
3851 rs111696008 A G A G -0.1565 -0.0259 0.02443 NA FALSE FALSE FALSE
GCST006061 0.0401 0.519 537409 outcome TRUE reported 2871_73_RAD51_RAD51
RAD51 chr2 216622986 0.026592 3.976e-09 35359 TRUE reported
textfile 2 1 TRUE
3852 rs114558472 T C T C -0.0819 0.0072 0.09165 NA FALSE FALSE FALSE
GCST006061 0.0139 0.6063 537409 outcome TRUE reported 2871_73_RAD51_RAD51
RAD51 chr1 196140161 0.013883 3.646e-09 35337 TRUE reported
textfile 2 1 TRUE
3853 rs1329424 G T G T -0.2987 -0.0095 0.39735 NA FALSE FALSE FALSE
GCST006061 0.0075 0.2071 537409 outcome TRUE reported 2871_73_RAD51_RAD51
RAD51 chr1 196677046 0.008105 1e-200 35338 TRUE reported textfile
2 1 TRUE
3854 rs1707652 T C T C 0.1346 0.0081 0.3102 NA FALSE FALSE FALSE
GCST006061 0.0074 0.2701 537409 outcome TRUE reported 2871_73_RAD51_RAD51
RAD51 chr3 165761011 0.008623 6.242e-55 35339 TRUE reported
textfile 2 1 TRUE
3855 rs3747843 G A G A -0.0448 -0.0037 0.47828 NA FALSE FALSE FALSE
GCST006061 0.0071 0.6026 537409 outcome TRUE reported 2871_73_RAD51_RAD51
RAD51 chr9 121152028 0.008022 2.343e-08 35373 TRUE reported
textfile 2 1 TRUE
3856 rs72748420 T C T C 0.0983 0.0095 0.07417 NA FALSE FALSE FALSE

GCST006061 0.0134 0.4784 537409 outcome TRUE reported 2871_73_RAD51_RAD51
RAD51 chr1 196141224 0.015282 1.255e-10 35337 TRUE reported
textfile 2 1 TRUE
3857 rs73169117 T C T C 0.0702 0.0051 0.11405 NA FALSE FALSE FALSE
GCST006061 0.0122 0.6755 537409 outcome TRUE reported 2871_73_RAD51_RAD51
RAD51 chr3 167217620 0.012681 3.097e-08 35354 TRUE reported
textfile 2 1 TRUE
3858 rs9572914 A G A G -0.0491 -0.0036 0.28557 NA FALSE FALSE FALSE
GCST006061 0.0078 0.6454 537409 outcome TRUE reported 2871_73_RAD51_RAD51
RAD51 chr13 31265137 0.008833 2.719e-08 35365 TRUE reported
textfile 2 1 TRUE
3859 rs1047286 A G A G -0.0569 -0.0013 0.24066 NA FALSE FALSE FALSE
GCST006061 0.0098 0.8921 537409 outcome TRUE reported 13599_15_RABEPK_RABEK
RABEPK chr19 6713251 0.009327 1.055e-09 35366 TRUE reported textfile
2 1 TRUE
3860 rs10922098 T C T C -0.2211 -0.009 0.40011 NA FALSE FALSE FALSE
GCST006061 0.0075 0.2275 537409 outcome TRUE reported 13599_15_RABEPK_RABEK
RABEPK chr1 196695521 0.00811 1.133e-163 35332 TRUE reported textfile
2 1 TRUE
3861 rs114009540 A G A G -0.1193 0.0322 0.03606 NA FALSE FALSE FALSE
GCST006061 0.0299 0.2809 537409 outcome TRUE reported 13599_15_RABEPK_RABEK
RABEPK chr1 196158413 0.021644 3.551e-08 35331 TRUE reported
textfile 2 1 TRUE
3862 rs12146727 A G A G -0.1154 -0.0037 0.11875 NA FALSE FALSE FALSE
GCST006061 0.0105 0.7269 537409 outcome TRUE reported 13599_15_RABEPK_RABEK
RABEPK chr12 7063032 0.012561 4.046e-20 35384 TRUE reported textfile
2 1 TRUE
3863 rs1707652 T C T C 0.1087 0.0081 0.3102 NA FALSE FALSE FALSE
GCST006061 0.0074 0.2701 537409 outcome TRUE reported 13599_15_RABEPK_RABEK
RABEPK chr3 165761011 0.008606 1.425e-36 35333 TRUE reported
textfile 2 1 TRUE
3864 rs35351085 T C T C 0.1127 0.0061 0.04877 NA FALSE FALSE FALSE
GCST006061 0.022 0.7825 537409 outcome TRUE reported 13599_15_RABEPK_RABEK
RABEPK chr1 197992799 0.018471 1.052e-09 35337 TRUE reported
textfile 2 1 TRUE
3865 rs704 A G A G 0.1072 -0.0155 0.49189 NA FALSE FALSE FALSE GCST006061
0.0072 0.03144 537409 outcome TRUE reported 13599_15_RABEPK_RABEK RABEPK
chr17 28367840 0.007987 4.473e-41 35354 TRUE reported textfile 2
1 TRUE
3866 rs1047286 A G A G -0.0838 -0.0013 0.24066 NA FALSE FALSE FALSE
GCST006061 0.0098 0.8921 537409 outcome TRUE reported 2693_20_OSM_OSM OSM
chr19 6713251 0.009423 5.946e-19 35372 TRUE reported textfile 2 1
TRUE
3867 rs114009540 A G A G -0.1351 0.0322 0.03606 NA FALSE FALSE FALSE
GCST006061 0.0299 0.2809 537409 outcome TRUE reported 2693_20_OSM_OSM OSM
chr1 196158413 0.021912 7.02e-10 35337 TRUE reported textfile 2
1 TRUE
3868 rs1329424 G T G T -0.2721 -0.0095 0.39735 NA FALSE FALSE FALSE
GCST006061 0.0075 0.2071 537409 outcome TRUE reported 2693_20_OSM_OSM OSM
chr1 196677046 0.008185 1e-200 35338 TRUE reported textfile 2 1
TRUE
3869 rs1707652 T C T C 0.1086 0.0081 0.3102 NA FALSE FALSE FALSE
GCST006061 0.0074 0.2701 537409 outcome TRUE reported 2693_20_OSM_OSM OSM
chr3 165761011 0.008693 8.191e-36 35339 TRUE reported textfile 2
1 TRUE
3870 rs72748420 T C T C 0.0935 0.0095 0.07417 NA FALSE FALSE FALSE
GCST006061 0.0134 0.4784 537409 outcome TRUE reported 2693_20_OSM_OSM OSM
chr1 196141224 0.015384 1.219e-09 35337 TRUE reported textfile 2
1 TRUE
3871 rs1047286 A G A G -0.0595 -0.0013 0.24066 NA FALSE FALSE FALSE
GCST006061 0.0098 0.8921 537409 outcome TRUE reported 7818_101_GBP6_GBP6
GBP6 chr19 6713251 0.009419 2.662e-10 35372 TRUE reported textfile
2 1 TRUE
3872 rs114009540 A G A G -0.1237 0.0322 0.03606 NA FALSE FALSE FALSE
GCST006061 0.0299 0.2809 537409 outcome TRUE reported 7818_101_GBP6_GBP6
GBP6 chr1 196158413 0.021884 1.582e-08 35337 TRUE reported

textfile 2 1 TRUE
3873 rs1329424 G T G T -0.2524 -0.0095 0.39735 NA FALSE FALSE FALSE
GCST006061 0.0075 0.2071 537409 outcome TRUE reported 7818_101_GBP6_GBP6
GBP6 chr1 196677046 0.008187 1e-200 35338 TRUE reported textfile
2 1 TRUE
3874 rs1707652 T C T C 0.1088 0.0081 0.3102 NA FALSE FALSE FALSE
GCST006061 0.0074 0.2701 537409 outcome TRUE reported 7818_101_GBP6_GBP6
GBP6 chr3 165761011 0.00869 5.803e-36 35339 TRUE reported textfile
2 1 TRUE
3875 rs35351085 T C T C 0.1203 0.0061 0.04877 NA FALSE FALSE FALSE
GCST006061 0.022 0.7825 537409 outcome TRUE reported 7818_101_GBP6_GBP6
GBP6 chr1 197992799 0.018653 1.124e-10 35343 TRUE reported
textfile 2 1 TRUE
3876 rs704 A G A G 0.0798 -0.0155 0.49189 NA FALSE FALSE FALSE GCST006061
0.0072 0.03144 537409 outcome TRUE reported 7818_101_GBP6_GBP6 GBP6 chr17
28367840 0.008071 4.714e-23 35360 TRUE reported textfile 2 1 TRUE
3877 rs1047286 A G A G -0.0721 -0.0013 0.24066 NA FALSE FALSE FALSE
GCST006061 0.0098 0.8921 537409 outcome TRUE reported 6961_14_IGFL3_IGFL3
IGFL3 chr19 6713251 0.009385 1.558e-14 35372 TRUE reported textfile
2 1 TRUE
3878 rs111696008 A G A G -0.1571 -0.0259 0.02443 NA FALSE FALSE FALSE
GCST006061 0.0401 0.519 537409 outcome TRUE reported 6961_14_IGFL3_IGFL3
IGFL3 chr2 216622986 0.02667 3.85e-09 35359 TRUE reported textfile
2 1 TRUE
3879 rs114009540 A G A G -0.1343 0.0322 0.03606 NA FALSE FALSE FALSE
GCST006061 0.0299 0.2809 537409 outcome TRUE reported 6961_14_IGFL3_IGFL3
IGFL3 chr1 196158413 0.0218 7.249e-10 35337 TRUE reported textfile
2 1 TRUE
3880 rs1329424 G T G T -0.2743 -0.0095 0.39735 NA FALSE FALSE FALSE
GCST006061 0.0075 0.2071 537409 outcome TRUE reported 6961_14_IGFL3_IGFL3
IGFL3 chr1 196677046 0.008144 1e-200 35338 TRUE reported textfile
2 1 TRUE
3881 rs2686395 C T C T -0.1308 -0.0077 0.3101 NA FALSE FALSE FALSE
GCST006061 0.0074 0.2965 537409 outcome TRUE reported 6961_14_IGFL3_IGFL3
IGFL3 chr3 165767231 0.008651 1.208e-51 35339 TRUE reported
textfile 2 1 TRUE
3882 rs704 A G A G 0.0624 -0.0155 0.49189 NA FALSE FALSE FALSE GCST006061
0.0072 0.03144 537409 outcome TRUE reported 6961_14_IGFL3_IGFL3 IGFL3 chr17
28367840 0.008042 8.515e-15 35360 TRUE reported textfile 2 1 TRUE
3883 rs72748420 T C T C 0.0915 0.0095 0.07417 NA FALSE FALSE FALSE
GCST006061 0.0134 0.4784 537409 outcome TRUE reported 6961_14_IGFL3_IGFL3
IGFL3 chr1 196141224 0.015309 2.272e-09 35337 TRUE reported
textfile 2 1 TRUE
3884 rs1047286 A G A G -0.0637 -0.0013 0.24066 NA FALSE FALSE FALSE
GCST006061 0.0098 0.8921 537409 outcome TRUE reported 15419_15_MMP20_MMP20
MMP20 chr19 6713251 0.009293 7.138e-12 35372 TRUE reported textfile
2 1 TRUE
3885 rs10922098 T C T C -0.1897 -0.009 0.40011 NA FALSE FALSE FALSE
GCST006061 0.0075 0.2275 537409 outcome TRUE reported 15419_15_MMP20_MMP20
MMP20 chr1 196695521 0.008083 8.353e-122 35338 TRUE reported
textfile 2 1 TRUE
3886 rs1707652 T C T C 0.0914 0.0081 0.3102 NA FALSE FALSE FALSE
GCST006061 0.0074 0.2701 537409 outcome TRUE reported 15419_15_MMP20_MMP20
MMP20 chr3 165761011 0.008573 1.548e-26 35339 TRUE reported
textfile 2 1 TRUE
3887 rs1047286 A G A G -0.0548 -0.0013 0.24066 NA FALSE FALSE FALSE
GCST006061 0.0098 0.8921 537409 outcome TRUE reported 9962_1_CDHR5_MUCDL
CDHR5 chr19 6713251 0.009362 4.822e-09 35372 TRUE reported textfile
2 1 TRUE
3888 rs12360820 C A C A 0.0702 -0.0151 0.17775 NA FALSE FALSE FALSE
GCST006061 0.0093 0.106 537409 outcome TRUE reported 9962_1_CDHR5_MUCDL
CDHR5 chr11 620927 0.010525 2.566e-11 35357 TRUE reported textfile
2 1 TRUE
3889 rs1707652 T C T C 0.0947 0.0081 0.3102 NA FALSE FALSE FALSE
GCST006061 0.0074 0.2701 537409 outcome TRUE reported 9962_1_CDHR5_MUCDL
CDHR5 chr3 165761011 0.00863 5.159e-28 35339 TRUE reported textfile

2 1 TRUE
3890 rs71352239 T C T C 0.0512 -0.0041 0.30263 NA FALSE FALSE FALSE
GCST006061 0.0087 0.6372 537409 outcome TRUE reported 9962_1_CDHR5_MUCDL
CDHR5 chr19 44926286 0.008748 4.827e-09 35368 TRUE reported
textfile 2 1 TRUE
3891 rs1047286 A G A G -0.0538 -0.0013 0.24066 NA FALSE FALSE FALSE
GCST006061 0.0098 0.8921 537409 outcome TRUE reported 11396_39_DNAI1_DNAI1
DNAI1 chr19 6713251 0.009357 8.954e-09 35363 TRUE reported textfile
2 1 TRUE
3892 rs10922098 T C T C -0.1667 -0.009 0.40011 NA FALSE FALSE FALSE
GCST006061 0.0075 0.2275 537409 outcome TRUE reported 11396_39_DNAI1_DNAI1
DNAI1 chr1 196695521 0.008148 4.989e-93 35329 TRUE reported
textfile 2 1 TRUE
3893 rs1707652 T C T C 0.0826 0.0081 0.3102 NA FALSE FALSE FALSE
GCST006061 0.0074 0.2701 537409 outcome TRUE reported 11396_39_DNAI1_DNAI1
DNAI1 chr3 165761011 0.00863 1.052e-21 35330 TRUE reported textfile
2 1 TRUE
3894 rs1047286 A G A G -0.0528 -0.0013 0.24066 NA FALSE FALSE FALSE
GCST006061 0.0098 0.8921 537409 outcome TRUE reported 12925_105_AXIN2_AXIN2
AXIN2 chr19 6713251 0.009297 1.353e-08 35364 TRUE reported textfile
2 1 TRUE
3895 rs10922098 T C T C -0.2259 -0.009 0.40011 NA FALSE FALSE FALSE
GCST006061 0.0075 0.2275 537409 outcome TRUE reported 12925_105_AXIN2_AXIN2
AXIN2 chr1 196695521 0.008079 4.682e-172 35330 TRUE reported
textfile 2 1 TRUE
3896 rs114009540 A G A G -0.125 0.0322 0.03606 NA FALSE FALSE FALSE
GCST006061 0.0299 0.2809 537409 outcome TRUE reported 12925_105_AXIN2_AXIN2
AXIN2 chr1 196158413 0.02158 6.944e-09 35329 TRUE reported textfile
2 1 TRUE
3897 rs1707652 T C T C 0.0995 0.0081 0.3102 NA FALSE FALSE FALSE
GCST006061 0.0074 0.2701 537409 outcome TRUE reported 12925_105_AXIN2_AXIN2
AXIN2 chr3 165761011 0.008581 4.346e-31 35331 TRUE reported
textfile 2 1 TRUE
3898 rs1047286 A G A G -0.052 -0.0013 0.24066 NA FALSE FALSE FALSE
GCST006061 0.0098 0.8921 537409 outcome TRUE reported 12713_365_RGS19_RGS19
RGS19 chr19 6713251 0.009316 2.384e-08 35363 TRUE reported textfile
2 1 TRUE
3899 rs10733084 C T C T -0.0658 0.006 0.1337 NA FALSE FALSE FALSE
GCST006061 0.0097 0.5319 537409 outcome TRUE reported 12713_365_RGS19_RGS19
RGS19 chr1 196181819 0.011666 1.697e-08 35328 TRUE reported
textfile 2 1 TRUE
3900 rs10922098 T C T C -0.1984 -0.009 0.40011 NA FALSE FALSE FALSE
GCST006061 0.0075 0.2275 537409 outcome TRUE reported 12713_365_RGS19_RGS19
RGS19 chr1 196695521 0.008097 1.35e-132 35329 TRUE reported
textfile 2 1 TRUE
3901 rs1707652 T C T C 0.1048 0.0081 0.3102 NA FALSE FALSE FALSE
GCST006061 0.0074 0.2701 537409 outcome TRUE reported 12713_365_RGS19_RGS19
RGS19 chr3 165761011 0.008586 2.881e-34 35330 TRUE reported
textfile 2 1 TRUE
3902 rs1916675 G A G A 0.0484 -0.0013 0.30309 NA FALSE FALSE FALSE
GCST006061 0.0075 0.8595 537409 outcome TRUE reported 12713_365_RGS19_RGS19
RGS19 chr9 104484123 0.008659 2.273e-08 35354 TRUE reported
textfile 2 1 TRUE
3903 rs2384656 G A G A 0.0565 0.0053 0.21641 NA FALSE FALSE FALSE
GCST006061 0.0079 0.5066 537409 outcome TRUE reported 12713_365_RGS19_RGS19
RGS19 chr2 27609188 0.009597 3.928e-09 35364 TRUE reported
textfile 2 1 TRUE
3904 rs704 A G A G 0.0443 -0.0155 0.49189 NA FALSE FALSE FALSE GCST006061
0.0072 0.03144 537409 outcome TRUE reported 12713_365_RGS19_RGS19 RGS19
chr17 28367840 0.00798 2.829e-08 35351 TRUE reported textfile 2 1
TRUE
3905 rs1047286 A G A G -0.0583 -0.0013 0.24066 NA FALSE FALSE FALSE
GCST006061 0.0098 0.8921 537409 outcome TRUE reported 12420_10_GPD1L_GPD1L
GPD1L chr19 6713251 0.009397 5.507e-10 35363 TRUE reported textfile
2 1 TRUE
3906 rs10801660 A G A G 0.0802 -0.0119 0.11574 NA FALSE FALSE FALSE

GCST006061 0.0108 0.2735 537409 outcome TRUE reported 12420_10_GPD1L_GPD1L
GPD1L chr1 198272225 0.012578 1.815e-10 35336 TRUE reported
textfile 2 1 TRUE
3907 rs111696008 A G A G -0.1696 -0.0259 0.02443 NA FALSE FALSE FALSE
GCST006061 0.0401 0.519 537409 outcome TRUE reported 12420_10_GPD1L_GPD1L
GPD1L chr2 216622986 0.026724 2.205e-10 35350 TRUE reported
textfile 2 1 TRUE
3908 rs1329424 G T G T -0.2983 -0.0095 0.39735 NA FALSE FALSE FALSE
GCST006061 0.0075 0.2071 537409 outcome TRUE reported 12420_10_GPD1L_GPD1L
GPD1L chr1 196677046 0.008151 1e-200 35329 TRUE reported textfile
2 1 TRUE
3909 rs1707652 T C T C 0.1435 0.0081 0.3102 NA FALSE FALSE FALSE
GCST006061 0.0074 0.2701 537409 outcome TRUE reported 12420_10_GPD1L_GPD1L
GPD1L chr3 165761011 0.008657 1.021e-61 35330 TRUE reported
textfile 2 1 TRUE
3910 rs61821527 T G T G -0.0467 -0.006 0.33477 NA FALSE FALSE FALSE
GCST006061 0.0076 0.4251 537409 outcome TRUE reported 12420_10_GPD1L_GPD1L
GPD1L chr1 201978878 0.008564 4.958e-08 35346 TRUE reported
textfile 2 1 TRUE
3911 rs704 A G A G 0.0954 -0.0155 0.49189 NA FALSE FALSE FALSE GCST006061
0.0072 0.03144 537409 outcome TRUE reported 12420_10_GPD1L_GPD1L GPD1L
chr17 28367840 0.008044 1.915e-32 35351 TRUE reported textfile 2
1 TRUE
3912 rs72748420 T C T C 0.106 0.0095 0.07417 NA FALSE FALSE FALSE
GCST006061 0.0134 0.4784 537409 outcome TRUE reported 12420_10_GPD1L_GPD1L
GPD1L chr1 196141224 0.015354 5.07e-12 35328 TRUE reported
textfile 2 1 TRUE
3913 rs73169117 T C T C 0.074 0.0051 0.11405 NA FALSE FALSE FALSE
GCST006061 0.0122 0.6755 537409 outcome TRUE reported 12420_10_GPD1L_GPD1L
GPD1L chr3 167217620 0.012749 6.461e-09 35345 TRUE reported
textfile 2 1 TRUE
3914 rs1047286 A G A G -0.0656 -0.0013 0.24066 NA FALSE FALSE FALSE
GCST006061 0.0098 0.8921 537409 outcome TRUE reported 17154_2_PTGES3_TEBP
PTGES3 chr19 6713251 0.009394 2.887e-12 35372 TRUE reported textfile
2 1 TRUE
3915 rs10801660 A G A G 0.075 -0.0119 0.11574 NA FALSE FALSE FALSE
GCST006061 0.0108 0.2735 537409 outcome TRUE reported 17154_2_PTGES3_TEBP
PTGES3 chr1 198272225 0.012556 2.328e-09 35345 TRUE reported
textfile 2 1 TRUE
3916 rs111696008 A G A G -0.1518 -0.0259 0.02443 NA FALSE FALSE FALSE
GCST006061 0.0401 0.519 537409 outcome TRUE reported 17154_2_PTGES3_TEBP
PTGES3 chr2 216622986 0.02671 1.321e-08 35359 TRUE reported textfile
2 1 TRUE
3917 rs1329424 G T G T -0.2814 -0.0095 0.39735 NA FALSE FALSE FALSE
GCST006061 0.0075 0.2071 537409 outcome TRUE reported 17154_2_PTGES3_TEBP
PTGES3 chr1 196677046 0.00815 1e-200 35338 TRUE reported textfile 2
1 TRUE
3918 rs1707652 T C T C 0.1208 0.0081 0.3102 NA FALSE FALSE FALSE
GCST006061 0.0074 0.2701 537409 outcome TRUE reported 17154_2_PTGES3_TEBP
PTGES3 chr3 165761011 0.00866 3.211e-44 35339 TRUE reported textfile
2 1 TRUE
3919 rs704 A G A G 0.067 -0.0155 0.49189 NA FALSE FALSE FALSE GCST006061
0.0072 0.03144 537409 outcome TRUE reported 17154_2_PTGES3_TEBP PTGES3 chr17
28367840 0.008054 8.844e-17 35360 TRUE reported textfile 2 1 TRUE
3920 rs72748420 T C T C 0.0954 0.0095 0.07417 NA FALSE FALSE FALSE
GCST006061 0.0134 0.4784 537409 outcome TRUE reported 17154_2_PTGES3_TEBP
PTGES3 chr1 196141224 0.015338 4.974e-10 35337 TRUE reported
textfile 2 1 TRUE
3921 rs1047286 A G A G -0.0693 -0.0013 0.24066 NA FALSE FALSE FALSE
GCST006061 0.0098 0.8921 537409 outcome TRUE reported 8389_8_LRRC74A_CN16B
LRRC74A chr19 6713251 0.009348 1.235e-13 35372 TRUE reported textfile
2 1 TRUE
3922 rs10922098 T C T C -0.2191 -0.009 0.40011 NA FALSE FALSE FALSE
GCST006061 0.0075 0.2275 537409 outcome TRUE reported 8389_8_LRRC74A_CN16B
LRRC74A chr1 196695521 0.008126 4.038e-160 35338 TRUE reported
textfile 2 1 TRUE

3923 rs2686395 C T C T -0.1067 -0.0077 0.3101 NA FALSE FALSE FALSE GCST006061 0.0074 0.2965 537409 outcome TRUE reported 8389_8_LRRC74A_CN16B LRRC74A chr3 165767231 0.008625 3.765e-35 35339 TRUE reported textfile 2 1 TRUE
3924 rs1047286 A G A G -0.0607 -0.0013 0.24066 NA FALSE FALSE FALSE GCST006061 0.0098 0.8921 537409 outcome TRUE reported 3471_49_STK16_STK16 STK16 chr19 6713251 0.009411 1.117e-10 35372 TRUE reported textfile 2 1 TRUE
3925 rs10922098 T C T C -0.2452 -0.009 0.40011 NA FALSE FALSE FALSE GCST006061 0.0075 0.2275 537409 outcome TRUE reported 3471_49_STK16_STK16 STK16 chr1 196695521 0.00816 2.301e-198 35338 TRUE reported textfile 2 1 TRUE
3926 rs1707652 T C T C 0.1083 0.0081 0.3102 NA FALSE FALSE FALSE GCST006061 0.0074 0.2701 537409 outcome TRUE reported 3471_49_STK16_STK16 STK16 chr3 165761011 0.008676 9.24e-36 35339 TRUE reported textfile 2 1 TRUE
3927 rs704 A G A G 0.0532 -0.0155 0.49189 NA FALSE FALSE FALSE GCST006061 0.0072 0.03144 537409 outcome TRUE reported 3471_49_STK16_STK16 STK16 chr17 28367840 0.008065 4.208e-11 35360 TRUE reported textfile 2 1 TRUE
3928 rs7513100 C T C T 0.0655 0 0.14871 NA FALSE FALSE FALSE GCST006061 0.0098 0.9981 537409 outcome TRUE reported 3471_49_STK16_STK16 STK16 chr1 198065935 0.011299 6.751e-09 35343 TRUE reported textfile 2 1 TRUE
3929 rs1047286 A G A G -0.0699 -0.0013 0.24066 NA FALSE FALSE FALSE GCST006061 0.0098 0.8921 537409 outcome TRUE reported 17200_50_KIR2DL1_KI2L1 KIR2DL1 chr19 6713251 0.009295 5.458e-14 35372 TRUE reported textfile 2 1 TRUE
3930 rs10922098 T C T C -0.1976 -0.009 0.40011 NA FALSE FALSE FALSE GCST006061 0.0075 0.2275 537409 outcome TRUE reported 17200_50_KIR2DL1_KI2L1 KIR2DL1 chr1 196695521 0.008087 7.377e-132 35338 TRUE reported textfile 2 1 TRUE
3931 rs111696008 A G A G -0.1483 -0.0259 0.02443 NA FALSE FALSE FALSE GCST006061 0.0401 0.519 537409 outcome TRUE reported 17200_50_KIR2DL1_KI2L1 KIR2DL1 chr2 216622986 0.026451 2.064e-08 35359 TRUE reported textfile 2 1 TRUE
3932 rs1451915 A C A C 0.0636 -0.0107 0.13641 NA FALSE FALSE FALSE GCST006061 0.0124 0.3895 537409 outcome TRUE reported 17200_50_KIR2DL1_KI2L1 KIR2DL1 chr1 196058002 0.011549 3.652e-08 35335 TRUE reported textfile 2 1 TRUE
3933 rs1707652 T C T C 0.0954 0.0081 0.3102 NA FALSE FALSE FALSE GCST006061 0.0074 0.2701 537409 outcome TRUE reported 17200_50_KIR2DL1_KI2L1 KIR2DL1 chr3 165761011 0.008582 1.039e-28 35339 TRUE reported textfile 2 1 TRUE
3934 rs1047286 A G A G -0.0587 -0.0013 0.24066 NA FALSE FALSE FALSE GCST006061 0.0098 0.8921 537409 outcome TRUE reported 13645_14_DHX38_PRP16 DHX38 chr19 6713251 0.009331 3.16e-10 35372 TRUE reported textfile 2 1 TRUE
3935 rs10922098 T C T C -0.1962 -0.009 0.40011 NA FALSE FALSE FALSE GCST006061 0.0075 0.2275 537409 outcome TRUE reported 13645_14_DHX38_PRP16 DHX38 chr1 196695521 0.008118 4.795e-129 35338 TRUE reported textfile 2 1 TRUE
3936 rs1707652 T C T C 0.0831 0.0081 0.3102 NA FALSE FALSE FALSE GCST006061 0.0074 0.2701 537409 outcome TRUE reported 13645_14_DHX38_PRP16 DHX38 chr3 165761011 0.008613 4.984e-22 35339 TRUE reported textfile 2 1 TRUE
3937 rs17095637 G A G A 0.2402 -0.082 0.01054 NA FALSE FALSE FALSE GCST006061 0.058 0.1574 537409 outcome TRUE reported 13645_14_DHX38_PRP16 DHX38 chr14 106028744 0.041663 8.15e-09 34966 TRUE reported textfile 2 1 TRUE
3938 rs1047286 A G A G -0.0694 -0.0013 0.24066 NA FALSE FALSE FALSE GCST006061 0.0098 0.8921 537409 outcome TRUE reported 13539_131_KCNN1_KCNN1 KCNN1 chr19 6713251 0.009315 9.297e-14 35366 TRUE reported textfile 2 1 TRUE
3939 rs10922098 T C T C -0.2047 -0.009 0.40011 NA FALSE FALSE FALSE GCST006061 0.0075 0.2275 537409 outcome TRUE reported 13539_131_KCNN1_KCNN1 KCNN1 chr1 196695521 0.008101 7.04e-141 35332 TRUE reported textfile 2 1 TRUE

3940 rs1707652 T C T C 0.0961 0.0081 0.3102 NA FALSE FALSE FALSE
GCST006061 0.0074 0.2701 537409 outcome TRUE reported 13539_131_KCNN1_KCNN1
KCNN1 chr3 165761011 0.008594 5.002e-29 35333 TRUE reported
textfile 2 1 TRUE
3941 rs1047286 A G A G -0.0551 -0.0013 0.24066 NA FALSE FALSE FALSE
GCST006061 0.0098 0.8921 537409 outcome TRUE reported 14692_3_ZNF276_ZN276
ZNF276 chr19 6713251 0.009426 5.054e-09 35372 TRUE reported textfile
2 1 TRUE
3942 rs10801660 A G A G 0.0802 -0.0119 0.11574 NA FALSE FALSE FALSE
GCST006061 0.0108 0.2735 537409 outcome TRUE reported 14692_3_ZNF276_ZN276
ZNF276 chr1 198272225 0.012612 2.033e-10 35345 TRUE reported
textfile 2 1 TRUE
3943 rs111696008 A G A G -0.1532 -0.0259 0.02443 NA FALSE FALSE FALSE
GCST006061 0.0401 0.519 537409 outcome TRUE reported 14692_3_ZNF276_ZN276
ZNF276 chr2 216622986 0.026809 1.1e-08 35359 TRUE reported textfile
2 1 TRUE
3944 rs1329424 G T G T -0.302 -0.0095 0.39735 NA FALSE FALSE FALSE
GCST006061 0.0075 0.2071 537409 outcome TRUE reported 14692_3_ZNF276_ZN276
ZNF276 chr1 196677046 0.008172 1e-200 35338 TRUE reported textfile
2 1 TRUE
3945 rs1707652 T C T C 0.1317 0.0081 0.3102 NA FALSE FALSE FALSE
GCST006061 0.0074 0.2701 537409 outcome TRUE reported 14692_3_ZNF276_ZN276
ZNF276 chr3 165761011 0.00869 7.001e-52 35339 TRUE reported textfile
2 1 TRUE
3946 rs72748420 T C T C 0.1025 0.0095 0.07417 NA FALSE FALSE FALSE
GCST006061 0.0134 0.4784 537409 outcome TRUE reported 14692_3_ZNF276_ZN276
ZNF276 chr1 196141224 0.015414 2.933e-11 35337 TRUE reported
textfile 2 1 TRUE
3947 rs1047286 A G A G -0.076 -0.0013 0.24066 NA FALSE FALSE FALSE
GCST006061 0.0098 0.8921 537409 outcome TRUE reported 18866_8_PPP3R2_CANB2
PPP3R2 chr19 6713251 0.009427 7.497e-16 35372 TRUE reported textfile
2 1 TRUE
3948 rs111696008 A G A G -0.1589 -0.0259 0.02443 NA FALSE FALSE FALSE
GCST006061 0.0401 0.519 537409 outcome TRUE reported 18866_8_PPP3R2_CANB2
PPP3R2 chr2 216622986 0.026825 3.152e-09 35359 TRUE reported
textfile 2 1 TRUE
3949 rs114009540 A G A G -0.1305 0.0322 0.03606 NA FALSE FALSE FALSE
GCST006061 0.0299 0.2809 537409 outcome TRUE reported 18866_8_PPP3R2_CANB2
PPP3R2 chr1 196158413 0.021905 2.56e-09 35337 TRUE reported
textfile 2 1 TRUE
3950 rs1329424 G T G T -0.2501 -0.0095 0.39735 NA FALSE FALSE FALSE
GCST006061 0.0075 0.2071 537409 outcome TRUE reported 18866_8_PPP3R2_CANB2
PPP3R2 chr1 196677046 0.0082 1e-200 35338 TRUE reported textfile 2
1 TRUE
3951 rs1707652 T C T C 0.1294 0.0081 0.3102 NA FALSE FALSE FALSE
GCST006061 0.0074 0.2701 537409 outcome TRUE reported 18866_8_PPP3R2_CANB2
PPP3R2 chr3 165761011 0.0087 4.92e-50 35339 TRUE reported textfile
2 1 TRUE
3952 rs704 A G A G 0.0957 -0.0155 0.49189 NA FALSE FALSE FALSE GCST006061
0.0072 0.03144 537409 outcome TRUE reported 18866_8_PPP3R2_CANB2 PPP3R2
chr17 28367840 0.008074 2.095e-32 35360 TRUE reported textfile 2
1 TRUE
3953 rs72748420 T C T C 0.0874 0.0095 0.07417 NA FALSE FALSE FALSE
GCST006061 0.0134 0.4784 537409 outcome TRUE reported 18866_8_PPP3R2_CANB2
PPP3R2 chr1 196141224 0.015373 1.305e-08 35337 TRUE reported
textfile 2 1 TRUE
3954 rs1047286 A G A G -0.0684 -0.0013 0.24066 NA FALSE FALSE FALSE
GCST006061 0.0098 0.8921 537409 outcome TRUE reported
2976_58_DSG1_Desmoglein_1 DSG1 chr19 6713251 0.00944 4.301e-13 35372 TRUE
reported textfile 2 1 TRUE
3955 rs10801660 A G A G 0.0745 -0.0119 0.11574 NA FALSE FALSE FALSE
GCST006061 0.0108 0.2735 537409 outcome TRUE reported
2976_58_DSG1_Desmoglein_1 DSG1 chr1 198272225 0.012614 3.498e-09 35345
TRUE reported textfile 2 1 TRUE
3956 rs1329424 G T G T -0.2795 -0.0095 0.39735 NA FALSE FALSE FALSE
GCST006061 0.0075 0.2071 537409 outcome TRUE reported

2976_58_DSG1_Desmoglein_1 DSG1 chr1 196677046 0.008194 1e-200 35338 TRUE reported textfile 2 1 TRUE
3957 rs2686395 C T C T -0.1161 -0.0077 0.3101 NA FALSE FALSE FALSE GCST006061 0.0074 0.2965 537409 outcome TRUE reported 2976_58_DSG1_Desmoglein_1 DSG1 chr3 165767231 0.00871 1.546e-40 35339 TRUE reported textfile 2 1 TRUE
3958 rs72748420 T C T C 0.1015 0.0095 0.07417 NA FALSE FALSE FALSE GCST006061 0.0134 0.4784 537409 outcome TRUE reported 2976_58_DSG1_Desmoglein_1 DSG1 chr1 196141224 0.015406 4.443e-11 35337 TRUE reported textfile 2 1 TRUE
3959 rs1047286 A G A G -0.0942 -0.0013 0.24066 NA FALSE FALSE FALSE GCST006061 0.0098 0.8921 537409 outcome TRUE reported 16081_38_AKR1B10_Aldose_reductase_like AKR1B10 chr19 6713251 0.009441 1.899e-23 35372 TRUE reported textfile 2 1 TRUE
3960 rs10922098 T C T C -0.2442 -0.009 0.40011 NA FALSE FALSE FALSE GCST006061 0.0075 0.2275 537409 outcome TRUE reported 16081_38_AKR1B10_Aldose_reductase_like AKR1B10 chr1 196695521 0.008202 8.314e-195 35338 TRUE reported textfile 2 1 TRUE
3961 rs111696008 A G A G -0.1503 -0.0259 0.02443 NA FALSE FALSE FALSE GCST006061 0.0401 0.519 537409 outcome TRUE reported 16081_38_AKR1B10_Aldose_reductase_like AKR1B10 chr2 216622986 0.026845 2.159e-08 35359 TRUE reported textfile 2 1 TRUE
3962 rs114056970 A G A G -0.0909 0.0056 0.09198 NA FALSE FALSE FALSE GCST006061 0.0139 0.6856 537409 outcome TRUE reported 16081_38_AKR1B10_Aldose_reductase_like AKR1B10 chr1 196136527 0.01398 7.906e-11 35337 TRUE reported textfile 2 1 TRUE
3963 rs1707652 T C T C 0.1069 0.0081 0.3102 NA FALSE FALSE FALSE GCST006061 0.0074 0.2701 537409 outcome TRUE reported 16081_38_AKR1B10_Aldose_reductase_like AKR1B10 chr3 165761011 0.008711 1.294e-34 35339 TRUE reported textfile 2 1 TRUE
3964 rs704 A G A G 0.1425 -0.0155 0.49189 NA FALSE FALSE FALSE GCST006061 0.0072 0.03144 537409 outcome TRUE reported 16081_38_AKR1B10_Aldose_reductase_like AKR1B10 chr17 28367840 0.008074 1.03e-69 35360 TRUE reported textfile 2 1 TRUE
3965 rs72748418 A G A G 0.09 0.0089 0.07164 NA FALSE FALSE FALSE GCST006061 0.0129 0.4914 537409 outcome TRUE reported 16081_38_AKR1B10_Aldose_reductase_like AKR1B10 chr1 196122755 0.015623 8.368e-09 35337 TRUE reported textfile 2 1 TRUE
3966 rs7513100 C T C T 0.0695 0 0.14871 NA FALSE FALSE FALSE GCST006061 0.0098 0.9981 537409 outcome TRUE reported 16081_38_AKR1B10_Aldose_reductase_like AKR1B10 chr1 198065935 0.011357 9.378e-10 35343 TRUE reported textfile 2 1 TRUE
3967 rs1047286 A G A G -0.0515 -0.0013 0.24066 NA FALSE FALSE FALSE GCST006061 0.0098 0.8921 537409 outcome TRUE reported 18302_204_ZWILCH_ZWILC ZWILCH chr19 6713251 0.009395 4.214e-08 35372 TRUE reported textfile 2 1 TRUE
3968 rs10801660 A G A G 0.0703 -0.0119 0.11574 NA FALSE FALSE FALSE GCST006061 0.0108 0.2735 537409 outcome TRUE reported 18302_204_ZWILCH_ZWILC ZWILCH chr1 198272225 0.012537 2.052e-08 35345 TRUE reported textfile 2 1 TRUE
3969 rs111696008 A G A G -0.1503 -0.0259 0.02443 NA FALSE FALSE FALSE GCST006061 0.0401 0.519 537409 outcome TRUE reported 18302_204_ZWILCH_ZWILC ZWILCH chr2 216622986 0.0267 1.811e-08 35359 TRUE reported textfile 2 1 TRUE
3970 rs1329424 G T G T -0.2698 -0.0095 0.39735 NA FALSE FALSE FALSE GCST006061 0.0075 0.2071 537409 outcome TRUE reported 18302_204_ZWILCH_ZWILC ZWILCH chr1 196677046 0.008149 1e-200 35338 TRUE reported textfile 2 1 TRUE
3971 rs2686395 C T C T -0.1248 -0.0077 0.3101 NA FALSE FALSE FALSE GCST006061 0.0074 0.2965 537409 outcome TRUE reported 18302_204_ZWILCH_ZWILC ZWILCH chr3 165767231 0.008652 3.636e-47 35339 TRUE reported textfile 2 1 TRUE
3972 rs72748420 T C T C 0.0939 0.0095 0.07417 NA FALSE FALSE FALSE GCST006061 0.0134 0.4784 537409 outcome TRUE reported 18302_204_ZWILCH_ZWILC ZWILCH chr1 196141224 0.015305 8.498e-10 35337 TRUE reported textfile 2 1 TRUE

3973 rs1047286 A G A G -0.0787 -0.0013 0.24066 NA FALSE FALSE FALSE
GCST006061 0.0098 0.8921 537409 outcome TRUE reported 12933_17_ENOX1_ENOX1
ENOX1 chr19 6713251 0.009389 5.185e-17 35363 TRUE reported textfile
2 1 TRUE
3974 rs10922098 T C T C -0.235 -0.009 0.40011 NA FALSE FALSE FALSE
GCST006061 0.0075 0.2275 537409 outcome TRUE reported 12933_17_ENOX1_ENOX1
ENOX1 chr1 196695521 0.008159 2.022e-182 35329 TRUE reported
textfile 2 1 TRUE
3975 rs111696008 A G A G -0.1658 -0.0259 0.02443 NA FALSE FALSE FALSE
GCST006061 0.0401 0.519 537409 outcome TRUE reported 12933_17_ENOX1_ENOX1
ENOX1 chr2 216622986 0.026722 5.481e-10 35350 TRUE reported
textfile 2 1 TRUE
3976 rs114009540 A G A G -0.1218 0.0322 0.03606 NA FALSE FALSE FALSE
GCST006061 0.0299 0.2809 537409 outcome TRUE reported 12933_17_ENOX1_ENOX1
ENOX1 chr1 196158413 0.021798 2.302e-08 35328 TRUE reported
textfile 2 1 TRUE
3977 rs2686395 C T C T -0.1237 -0.0077 0.3101 NA FALSE FALSE FALSE
GCST006061 0.0074 0.2965 537409 outcome TRUE reported 12933_17_ENOX1_ENOX1
ENOX1 chr3 165767231 0.008661 2.809e-46 35330 TRUE reported
textfile 2 1 TRUE
3978 rs3917538 A G A G 0.0561 -0.0137 0.21967 NA FALSE FALSE FALSE
GCST006061 0.0082 0.09535 537409 outcome TRUE reported 12933_17_ENOX1_ENOX1
ENOX1 chr7 95308581 0.009712 7.624e-09 35340 TRUE reported
textfile 2 1 TRUE
3979 rs72748420 T C T C 0.0885 0.0095 0.07417 NA FALSE FALSE FALSE
GCST006061 0.0134 0.4784 537409 outcome TRUE reported 12933_17_ENOX1_ENOX1
ENOX1 chr1 196141224 0.015307 7.401e-09 35328 TRUE reported
textfile 2 1 TRUE
3980 rs1047286 A G A G -0.0565 -0.0013 0.24066 NA FALSE FALSE FALSE
GCST006061 0.0098 0.8921 537409 outcome TRUE reported
7763_25_CDH11_Cadherin_11 CDH11 chr19 6713251 0.009373 1.662e-09 35372
TRUE reported textfile 2 1 TRUE
3981 rs10922098 T C T C -0.221 -0.009 0.40011 NA FALSE FALSE FALSE
GCST006061 0.0075 0.2275 537409 outcome TRUE reported
7763_25_CDH11_Cadherin_11 CDH11 chr1 196695521 0.008147 4.609e-162 35338
TRUE reported textfile 2 1 TRUE
3982 rs12478841 G A G A 0.0568 0.0049 0.21569 NA FALSE FALSE FALSE
GCST006061 0.008 0.5413 537409 outcome TRUE reported
7763_25_CDH11_Cadherin_11 CDH11 chr2 27588855 0.009671 4.278e-09 35373
TRUE reported textfile 2 1 TRUE
3983 rs1451915 A C A C 0.0647 -0.0107 0.13641 NA FALSE FALSE FALSE
GCST006061 0.0124 0.3895 537409 outcome TRUE reported
7763_25_CDH11_Cadherin_11 CDH11 chr1 196058002 0.011636 2.692e-08 35335
TRUE reported textfile 2 1 TRUE
3984 rs1707652 T C T C 0.0934 0.0081 0.3102 NA FALSE FALSE FALSE
GCST006061 0.0074 0.2701 537409 outcome TRUE reported
7763_25_CDH11_Cadherin_11 CDH11 chr3 165761011 0.008652 3.616e-27 35339
TRUE reported textfile 2 1 TRUE
3985 rs704 A G A G 0.0446 -0.0155 0.49189 NA FALSE FALSE FALSE GCST006061
0.0072 0.03144 537409 outcome TRUE reported 7763_25_CDH11_Cadherin_11 CDH11
chr17 28367840 0.008031 2.8e-08 35360 TRUE reported textfile 2 1
TRUE
3986 rs1047286 A G A G -0.0632 -0.0013 0.24066 NA FALSE FALSE FALSE
GCST006061 0.0098 0.8921 537409 outcome TRUE reported 6283_60_MCEMP1_MCEM1
MCEMP1 chr19 6713251 0.009357 1.436e-11 35372 TRUE reported textfile
2 1 TRUE
3987 rs10922098 T C T C -0.2378 -0.009 0.40011 NA FALSE FALSE FALSE
GCST006061 0.0075 0.2275 537409 outcome TRUE reported 6283_60_MCEMP1_MCEM1
MCEMP1 chr1 196695521 0.008118 1.335e-188 35338 TRUE reported
textfile 2 1 TRUE
3988 rs1354034 C T C T 0.0506 0.0088 0.38119 NA FALSE FALSE FALSE
GCST006061 0.0073 0.2248 537409 outcome TRUE reported 6283_60_MCEMP1_MCEM1
MCEMP1 chr3 56815721 0.008236 8.058e-10 35356 TRUE reported
textfile 2 1 TRUE
3989 rs1707652 T C T C 0.1133 0.0081 0.3102 NA FALSE FALSE FALSE
GCST006061 0.0074 0.2701 537409 outcome TRUE reported 6283_60_MCEMP1_MCEM1

MCEMP1 chr3 165761011 0.008622 1.93e-39 35339 TRUE reported
textfile 2 1 TRUE
3990 rs35351085 T C T C 0.1133 0.0061 0.04877 NA FALSE FALSE FALSE
GCST006061 0.022 0.7825 537409 outcome TRUE reported 6283_60_MCEMP1_MCEM1
MCEMP1 chr1 197992799 0.01851 9.304e-10 35343 TRUE reported textfile
2 1 TRUE
3991 rs1047286 A G A G -0.0699 -0.0013 0.24066 NA FALSE FALSE FALSE
GCST006061 0.0098 0.8921 537409 outcome TRUE reported 5988_49_TNFSF14_LIGHT
TNFSF14 chr19 6713251 0.009388 9.629e-14 35372 TRUE reported textfile
2 1 TRUE
3992 rs10922098 T C T C -0.1849 -0.009 0.40011 NA FALSE FALSE FALSE
GCST006061 0.0075 0.2275 537409 outcome TRUE reported 5988_49_TNFSF14_LIGHT
TNFSF14 chr1 196695521 0.008171 2.281e-113 35338 TRUE reported
textfile 2 1 TRUE
3993 rs1707652 T C T C 0.095 0.0081 0.3102 NA FALSE FALSE FALSE
GCST006061 0.0074 0.2701 537409 outcome TRUE reported 5988_49_TNFSF14_LIGHT
TNFSF14 chr3 165761011 0.008659 5.267e-28 35339 TRUE reported
textfile 2 1 TRUE
3994 rs28645249 C T C T 0.0573 0.019 0.26162 NA FALSE FALSE FALSE
GCST006061 0.0115 0.09815 537409 outcome TRUE reported 5988_49_TNFSF14_LIGHT
TNFSF14 chr6 32557458 0.008819 8.179e-11 35438 TRUE reported
textfile 2 1 TRUE
3995 rs1047286 A G A G -0.0599 -0.0013 0.24066 NA FALSE FALSE FALSE
GCST006061 0.0098 0.8921 537409 outcome TRUE reported 16755_195_GDF3_GDF_3
GDF3 chr19 6713251 0.009308 1.233e-10 35372 TRUE reported textfile
2 1 TRUE
3996 rs10922098 T C T C -0.1672 -0.009 0.40011 NA FALSE FALSE FALSE
GCST006061 0.0075 0.2275 537409 outcome TRUE reported 16755_195_GDF3_GDF_3
GDF3 chr1 196695521 0.008113 2.298e-94 35338 TRUE reported
textfile 2 1 TRUE
3997 rs1707652 T C T C 0.0846 0.0081 0.3102 NA FALSE FALSE FALSE
GCST006061 0.0074 0.2701 537409 outcome TRUE reported 16755_195_GDF3_GDF_3
GDF3 chr3 165761011 0.0086 7.754e-23 35339 TRUE reported textfile
2 1 TRUE
3998 rs1047286 A G A G -0.0538 -0.0013 0.24066 NA FALSE FALSE FALSE
GCST006061 0.0098 0.8921 537409 outcome TRUE reported 13053_6_SLC6A14_S6A14
SLC6A14 chr19 6713251 0.00934 8.393e-09 35363 TRUE reported textfile 2
1 TRUE
3999 rs12478841 G A G A 0.0636 0.0049 0.21569 NA FALSE FALSE FALSE
GCST006061 0.008 0.5413 537409 outcome TRUE reported 13053_6_SLC6A14_S6A14
SLC6A14 chr2 27588855 0.009644 4.265e-11 35364 TRUE reported
textfile 2 1 TRUE
4000 rs704 A G A G -0.1106 -0.0155 0.49189 NA FALSE FALSE FALSE GCST006061
0.0072 0.03144 537409 outcome TRUE reported 13053_6_SLC6A14_S6A14 SLC6A14
chr17 28367840 0.007999 1.748e-43 35351 TRUE reported textfile 2
1 TRUE
4001 rs7610437 G A G A 0.077 -0.0236 0.16111 NA FALSE FALSE FALSE
GCST006061 0.0091 0.009827 537409 outcome TRUE reported
13053_6_SLC6A14_S6A14 SLC6A14 chr3 186416495 0.010926 1.822e-12 35356
TRUE reported textfile 2 1 TRUE
4002 rs9856702 G A G A 0.2499 -0.0084 0.13966 NA FALSE FALSE FALSE
GCST006061 0.0109 0.4432 537409 outcome TRUE reported 13053_6_SLC6A14_S6A14
SLC6A14 chr3 186672039 0.011431 6.1e-106 35359 TRUE reported
textfile 2 1 TRUE
4003 rs1047286 A G A G -0.0579 -0.0013 0.24066 NA FALSE FALSE FALSE
GCST006061 0.0098 0.8921 537409 outcome TRUE reported 3399_31_STAB2_STAB2
STAB2 chr19 6713251 0.009298 4.754e-10 35372 TRUE reported textfile
2 1 TRUE
4004 rs1329424 G T G T -0.2551 -0.0095 0.39735 NA FALSE FALSE FALSE
GCST006061 0.0075 0.2071 537409 outcome TRUE reported 3399_31_STAB2_STAB2
STAB2 chr1 196677046 0.008069 1e-200 35338 TRUE reported textfile
2 1 TRUE
4005 rs144704969 T C T C 0.1096 -0.0238 0.04536 NA FALSE FALSE FALSE
GCST006061 0.0154 0.1235 537409 outcome TRUE reported 3399_31_STAB2_STAB2
STAB2 chr1 196158214 0.019014 8.208e-09 35337 TRUE reported
textfile 2 1 TRUE

4006 rs2686395 C T C T -0.1131 -0.0077 0.3101 NA FALSE FALSE FALSE
GCST006061 0.0074 0.2965 537409 outcome TRUE reported 3399_31_STAB2_STAB2
STAB2 chr3 165767231 0.008573 9.707e-40 35339 TRUE reported
textfile 2 1 TRUE
4007 rs1047286 A G A G -0.0655 -0.0013 0.24066 NA FALSE FALSE FALSE
GCST006061 0.0098 0.8921 537409 outcome TRUE reported 16807_35_TNNT3_TNNT3
TNNT3 chr19 6713251 0.009376 2.832e-12 35372 TRUE reported textfile
2 1 TRUE
4008 rs10922098 T C T C -0.2199 -0.009 0.40011 NA FALSE FALSE FALSE
GCST006061 0.0075 0.2275 537409 outcome TRUE reported 16807_35_TNNT3_TNNT3
TNNT3 chr1 196695521 0.008147 1.835e-160 35338 TRUE reported
textfile 2 1 TRUE
4009 rs1707652 T C T C 0.1102 0.0081 0.3102 NA FALSE FALSE FALSE
GCST006061 0.0074 0.2701 537409 outcome TRUE reported 16807_35_TNNT3_TNNT3
TNNT3 chr3 165761011 0.008648 3.386e-37 35339 TRUE reported
textfile 2 1 TRUE
4010 rs61679841 T C T C 0.0539 -0.0199 0.21787 NA FALSE FALSE FALSE
GCST006061 0.0087 0.0228 537409 outcome TRUE reported 16807_35_TNNT3_TNNT3
TNNT3 chr15 60176779 0.00975 3.239e-08 35369 TRUE reported textfile
2 1 TRUE
4011 rs1047286 A G A G -0.0517 -0.0013 0.24066 NA FALSE FALSE FALSE
GCST006061 0.0098 0.8921 537409 outcome TRUE reported 11383_41_KRT7_Keratin_7
KRT7 chr19 6713251 0.009349 3.207e-08 35363 TRUE reported textfile
2 1 TRUE
4012 rs10922098 T C T C -0.1874 -0.009 0.40011 NA FALSE FALSE FALSE
GCST006061 0.0075 0.2275 537409 outcome TRUE reported 11383_41_KRT7_Keratin_7
KRT7 chr1 196695521 0.008136 2.219e-117 35329 TRUE reported
textfile 2 1 TRUE
4013 rs112189508 C T C T 0.1698 -0.0306 0.05907 NA FALSE FALSE FALSE
GCST006061 0.0175 0.08086 537409 outcome TRUE reported 11383_41_KRT7_Keratin_7
KRT7 chr11 6262548 0.017085 2.822e-23 35365 TRUE reported textfile
2 1 TRUE
4014 rs117627469 A G A G 0.2833 -0.0504 0.01774 NA FALSE FALSE FALSE
GCST006061 0.0491 0.3048 537409 outcome TRUE reported 11383_41_KRT7_Keratin_7
KRT7 chr11 8178769 0.030074 4.503e-21 35365 TRUE reported textfile
2 1 TRUE
4015 rs16914574 C T C T 0.3409 -0.0098 0.04057 NA FALSE FALSE FALSE
GCST006061 0.0233 0.6742 537409 outcome TRUE reported 11383_41_KRT7_Keratin_7
KRT7 chr11 6511260 0.020443 1.973e-62 35371 TRUE reported textfile
2 1 TRUE
4016 rs2686395 C T C T -0.1037 -0.0077 0.3101 NA FALSE FALSE FALSE
GCST006061 0.0074 0.2965 537409 outcome TRUE reported 11383_41_KRT7_Keratin_7
KRT7 chr3 165767231 0.008629 2.858e-33 35330 TRUE reported
textfile 2 1 TRUE
4017 rs4977155 C T C T -0.0869 -0.0069 0.16663 NA FALSE FALSE FALSE
GCST006061 0.0127 0.5868 537409 outcome TRUE reported 11383_41_KRT7_Keratin_7
KRT7 chr14 106473508 0.010988 2.603e-15 34957 TRUE reported
textfile 2 1 TRUE
4018 rs1047286 A G A G -0.0554 -0.0013 0.24066 NA FALSE FALSE FALSE
GCST006061 0.0098 0.8921 537409 outcome TRUE reported 13926_1_RMI1_RMI1
RMI1 chr19 6713251 0.009351 3.128e-09 35372 TRUE reported textfile
2 1 TRUE
4019 rs1707652 T C T C 0.0874 0.0081 0.3102 NA FALSE FALSE FALSE
GCST006061 0.0074 0.2701 537409 outcome TRUE reported 13926_1_RMI1_RMI1
RMI1 chr3 165761011 0.008617 3.567e-24 35339 TRUE reported
textfile 2 1 TRUE
4020 rs35209896 A C A C 0.0886 0.0267 0.09263 NA FALSE FALSE FALSE
GCST006061 0.0355 0.4521 537409 outcome TRUE reported 13926_1_RMI1_RMI1
RMI1 chr6 32519009 0.013672 9.139e-11 35439 TRUE reported
textfile 2 1 TRUE
4021 rs1047286 A G A G -0.0538 -0.0013 0.24066 NA FALSE FALSE FALSE
GCST006061 0.0098 0.8921 537409 outcome TRUE reported 14120_2_RNF43_RNF43
RNF43 chr19 6713251 0.009368 9.301e-09 35372 TRUE reported textfile
2 1 TRUE
4022 rs10922098 T C T C -0.1658 -0.009 0.40011 NA FALSE FALSE FALSE
GCST006061 0.0075 0.2275 537409 outcome TRUE reported 14120_2_RNF43_RNF43

RNF43 chr1 196695521 0.008154 6.428e-92 35338 TRUE reported textfile 2 1 TRUE
4023 rs1707652 T C T C 0.1013 0.0081 0.3102 NA FALSE FALSE FALSE GCST006061 0.0074 0.2701 537409 outcome TRUE reported 14120_2_RNF43_RNF43 RNF43 chr3 165761011 0.008638 9.181e-32 35339 TRUE reported textfile 2 1 TRUE
4024 rs241771 C T C T -0.053 0.0101 0.45473 NA FALSE FALSE FALSE GCST006061 0.0071 0.1567 537409 outcome TRUE reported 14120_2_RNF43_RNF43 RNF43 chr17 28265920 0.008067 5.042e-11 35359 TRUE reported textfile 2 1 TRUE
4025 rs1047286 A G A G -0.06 -0.0013 0.24066 NA FALSE FALSE FALSE GCST006061 0.0098 0.8921 537409 outcome TRUE reported 16585_16_B3GNT4_B3GN4 B3GNT4 chr19 6713251 0.009129 4.957e-11 35372 TRUE reported textfile 2 1 TRUE
4026 rs10922098 T C T C -0.1433 -0.009 0.40011 NA FALSE FALSE FALSE GCST006061 0.0075 0.2275 537409 outcome TRUE reported 16585_16_B3GNT4_B3GN4 B3GNT4 chr1 196695521 0.007956 1.562e-72 35338 TRUE reported textfile 2 1 TRUE
4027 rs1706435 G A G A 0.0591 0.0117 0.36981 NA FALSE FALSE FALSE GCST006061 0.0072 0.1047 537409 outcome TRUE reported 16585_16_B3GNT4_B3GN4 B3GNT4 chr3 165743688 0.008083 2.645e-13 35339 TRUE reported textfile 2 1 TRUE
4028 rs704 A G A G 0.0515 -0.0155 0.49189 NA FALSE FALSE FALSE GCST006061 0.0072 0.03144 537409 outcome TRUE reported 16585_16_B3GNT4_B3GN4 B3GNT4 chr17 28367840 0.007825 4.648e-11 35360 TRUE reported textfile 2 1 TRUE
4029 rs1047286 A G A G -0.0725 -0.0013 0.24066 NA FALSE FALSE FALSE GCST006061 0.0098 0.8921 537409 outcome TRUE reported 12939_1_POLR1C_RPAC1 POLR1C chr19 6713251 0.009417 1.369e-14 35363 TRUE reported textfile 2 1 TRUE
4030 rs10801660 A G A G 0.0742 -0.0119 0.11574 NA FALSE FALSE FALSE GCST006061 0.0108 0.2735 537409 outcome TRUE reported 12939_1_POLR1C_RPAC1 POLR1C chr1 198272225 0.012581 3.688e-09 35336 TRUE reported textfile 2 1 TRUE
4031 rs111696008 A G A G -0.1761 -0.0259 0.02443 NA FALSE FALSE FALSE GCST006061 0.0401 0.519 537409 outcome TRUE reported 12939_1_POLR1C_RPAC1 POLR1C chr2 216622986 0.026799 4.992e-11 35350 TRUE reported textfile 2 1 TRUE
4032 rs1329424 G T G T -0.2595 -0.0095 0.39735 NA FALSE FALSE FALSE GCST006061 0.0075 0.2071 537409 outcome TRUE reported 12939_1_POLR1C_RPAC1 POLR1C chr1 196677046 0.008187 1e-200 35329 TRUE reported textfile 2 1 TRUE
4033 rs1707652 T C T C 0.1272 0.0081 0.3102 NA FALSE FALSE FALSE GCST006061 0.0074 0.2701 537409 outcome TRUE reported 12939_1_POLR1C_RPAC1 POLR1C chr3 165761011 0.008689 1.56e-48 35330 TRUE reported textfile 2 1 TRUE
4034 rs72748420 T C T C 0.0849 0.0095 0.07417 NA FALSE FALSE FALSE GCST006061 0.0134 0.4784 537409 outcome TRUE reported 12939_1_POLR1C_RPAC1 POLR1C chr1 196141224 0.015373 3.338e-08 35328 TRUE reported textfile 2 1 TRUE
4035 rs1047286 A G A G -0.0646 -0.0013 0.24066 NA FALSE FALSE FALSE GCST006061 0.0098 0.8921 537409 outcome TRUE reported 2443_10_FGF8_FGF_8B FGF8 chr19 6713251 0.009362 5.204e-12 35372 TRUE reported textfile 2 1 TRUE
4036 rs10922098 T C T C -0.2001 -0.009 0.40011 NA FALSE FALSE FALSE GCST006061 0.0075 0.2275 537409 outcome TRUE reported 2443_10_FGF8_FGF_8B FGF8 chr1 196695521 0.008142 2.237e-133 35338 TRUE reported textfile 2 1 TRUE
4037 rs2686395 C T C T -0.0831 -0.0077 0.3101 NA FALSE FALSE FALSE GCST006061 0.0074 0.2965 537409 outcome TRUE reported 2443_10_FGF8_FGF_8B FGF8 chr3 165767231 0.008641 6.764e-22 35339 TRUE reported textfile 2 1 TRUE
4038 rs1047286 A G A G -0.0617 -0.0013 0.24066 NA FALSE FALSE FALSE GCST006061 0.0098 0.8921 537409 outcome TRUE reported 19303_64_PET117_PT117 PET117 chr19 6713251 0.009387 4.941e-11 35372 TRUE reported textfile 2 1 TRUE

4039 rs10922098 T C T C -0.2012 -0.009 0.40011 NA FALSE FALSE FALSE
GCST006061 0.0075 0.2275 537409 outcome TRUE reported 19303_64_PET117_PT117
PET117 chr1 196695521 0.008158 2.622e-134 35338 TRUE reported
textfile 2 1 TRUE
4040 rs1707652 T C T C 0.103 0.0081 0.3102 NA FALSE FALSE FALSE
GCST006061 0.0074 0.2701 537409 outcome TRUE reported 19303_64_PET117_PT117
PET117 chr3 165761011 0.008654 1.147e-32 35339 TRUE reported
textfile 2 1 TRUE
4041 rs1047286 A G A G -0.063 -0.0013 0.24066 NA FALSE FALSE FALSE
GCST006061 0.0098 0.8921 537409 outcome TRUE reported 11456_2_MAGEB10_MAGBA
MAGEB10 chr19 6713251 0.00932 1.38e-11 35363 TRUE reported textfile 2
1 TRUE
4042 rs10922098 T C T C -0.1683 -0.009 0.40011 NA FALSE FALSE FALSE
GCST006061 0.0075 0.2275 537409 outcome TRUE reported 11456_2_MAGEB10_MAGBA
MAGEB10 chr1 196695521 0.008124 2.415e-95 35329 TRUE reported
textfile 2 1 TRUE
4043 rs2686395 C T C T -0.0806 -0.0077 0.3101 NA FALSE FALSE FALSE
GCST006061 0.0074 0.2965 537409 outcome TRUE reported 11456_2_MAGEB10_MAGBA
MAGEB10 chr3 165767231 0.008606 7.587e-21 35330 TRUE reported
textfile 2 1 TRUE
4044 rs1047286 A G A G -0.0548 -0.0013 0.24066 NA FALSE FALSE FALSE
GCST006061 0.0098 0.8921 537409 outcome TRUE reported 8776_10_ERLIN1_ERLN1
ERLIN1 chr19 6713251 0.009279 3.505e-09 35372 TRUE reported textfile
2 1 TRUE
4045 rs10922098 T C T C -0.171 -0.009 0.40011 NA FALSE FALSE FALSE
GCST006061 0.0075 0.2275 537409 outcome TRUE reported 8776_10_ERLIN1_ERLN1
ERLIN1 chr1 196695521 0.008084 2.548e-99 35338 TRUE reported
textfile 2 1 TRUE
4046 rs12478841 G A G A 0.0538 0.0049 0.21569 NA FALSE FALSE FALSE
GCST006061 0.008 0.5413 537409 outcome TRUE reported 8776_10_ERLIN1_ERLN1
ERLIN1 chr2 27588855 0.009577 1.938e-08 35373 TRUE reported
textfile 2 1 TRUE
4047 rs704 A G A G 0.0764 -0.0155 0.49189 NA FALSE FALSE FALSE GCST006061
0.0072 0.03144 537409 outcome TRUE reported 8776_10_ERLIN1_ERLN1 ERLIN1
chr17 28367840 0.007945 6.856e-22 35360 TRUE reported textfile 2
1 TRUE
4048 rs1047286 A G A G -0.0527 -0.0013 0.24066 NA FALSE FALSE FALSE
GCST006061 0.0098 0.8921 537409 outcome TRUE reported 13105_7_SNAP25_SNP25
SNAP25 chr19 6713251 0.00938 1.925e-08 35363 TRUE reported textfile 2
1 TRUE
4049 rs10922098 T C T C -0.1185 -0.009 0.40011 NA FALSE FALSE FALSE
GCST006061 0.0075 0.2275 537409 outcome TRUE reported 13105_7_SNAP25_SNP25
SNAP25 chr1 196695521 0.00819 1.913e-47 35329 TRUE reported textfile
2 1 TRUE
4050 rs111397271 A G A G 0.2076 -0.0178 0.0137 NA FALSE FALSE FALSE
GCST006061 0.0374 0.6336 537409 outcome TRUE reported 13105_7_SNAP25_SNP25
SNAP25 chr14 94611890 0.032843 2.599e-10 35363 TRUE reported
textfile 2 1 TRUE
4051 rs11629238 A G A G 0.0619 -0.0035 0.18088 NA FALSE FALSE FALSE
GCST006061 0.0091 0.6994 537409 outcome TRUE reported 13105_7_SNAP25_SNP25
SNAP25 chr14 96134240 0.010572 4.769e-09 35369 TRUE reported
textfile 2 1 TRUE
4052 rs1243160 A G A G -0.096 3e-04 0.17627 NA FALSE FALSE FALSE
GCST006061 0.0098 0.9755 537409 outcome TRUE reported 13105_7_SNAP25_SNP25
SNAP25 chr14 94388540 0.010692 2.739e-19 35363 TRUE reported
textfile 2 1 TRUE
4053 rs12437339 T C T C 0.1122 0.0276 0.04897 NA FALSE FALSE FALSE
GCST006061 0.0215 0.1994 537409 outcome TRUE reported 13105_7_SNAP25_SNP25
SNAP25 chr14 94683813 0.018926 3.058e-09 35361 TRUE reported
textfile 2 1 TRUE
4054 rs1706435 G A G A 0.068 0.0117 0.36981 NA FALSE FALSE FALSE
GCST006061 0.0072 0.1047 537409 outcome TRUE reported 13105_7_SNAP25_SNP25
SNAP25 chr3 165743688 0.008304 2.63e-16 35330 TRUE reported
textfile 2 1 TRUE
4055 rs1047286 A G A G -0.1549 -0.0013 0.24066 NA FALSE FALSE FALSE
GCST006061 0.0098 0.8921 537409 outcome TRUE reported 6896_3_C1orf162_CA162

C1orf162 chr19 6713251 0.009574 6.994e-59 35372 TRUE reported
textfile 2 1 TRUE
4056 rs10922098 T C T C -0.1682 -0.009 0.40011 NA FALSE FALSE FALSE
GCST006061 0.0075 0.2275 537409 outcome TRUE reported 6896_3_C1orf162_CA162
C1orf162 chr1 196695521 0.008356 4.094e-90 35338 TRUE reported
textfile 2 1 TRUE
4057 rs116347702 C A C A -0.204 0.0026 0.03344 NA FALSE FALSE FALSE
GCST006061 0.0243 0.9149 537409 outcome TRUE reported 6896_3_C1orf162_CA162
C1orf162 chr3 53024443 0.022946 6.089e-19 35349 TRUE reported
textfile 2 1 TRUE
4058 rs116749582 T C T C -0.2415 -0.0044 0.01257 NA FALSE FALSE FALSE
GCST006061 0.0567 0.9381 537409 outcome TRUE reported 6896_3_C1orf162_CA162
C1orf162 chr3 55895022 0.037862 1.79e-10 35343 TRUE reported
textfile 2 1 TRUE
4059 rs1260326 C T C T -0.0715 -0.0069 0.34132 NA FALSE FALSE FALSE
GCST006061 0.0073 0.3401 537409 outcome TRUE reported 6896_3_C1orf162_CA162
C1orf162 chr2 27508073 0.00869 1.904e-16 35373 TRUE reported
textfile 2 1 TRUE
4060 rs35209896 A C A C 0.0851 0.0267 0.09263 NA FALSE FALSE FALSE
GCST006061 0.0355 0.4521 537409 outcome TRUE reported 6896_3_C1orf162_CA162
C1orf162 chr6 32519009 0.014045 1.369e-09 35439 TRUE reported
textfile 2 1 TRUE
4061 rs704 A G A G -0.07 -0.0155 0.49189 NA FALSE FALSE FALSE GCST006061
0.0072 0.03144 537409 outcome TRUE reported 6896_3_C1orf162_CA162 C1orf162
chr17 28367840 0.008214 1.564e-17 35360 TRUE reported textfile 2
1 TRUE
4062 rs72840032 T C T C -0.3228 0.0125 0.04145 NA FALSE FALSE FALSE
GCST006061 0.0184 0.4965 537409 outcome TRUE reported 6896_3_C1orf162_CA162
C1orf162 chr10 100130207 0.020421 2.758e-56 35368 TRUE reported
textfile 2 1 TRUE
4063 rs8192297 C T C T -0.09 0.0057 0.09766 NA FALSE FALSE FALSE
GCST006061 0.0109 0.5973 537409 outcome TRUE reported 6896_3_C1orf162_CA162
C1orf162 chr15 89801121 0.01395 1.107e-10 35354 TRUE reported
textfile 2 1 TRUE
4064 rs1047286 A G A G -0.0768 -0.0013 0.24066 NA FALSE FALSE FALSE
GCST006061 0.0098 0.8921 537409 outcome TRUE reported 6587_6_LRFN5_LRFN5
LRFN5 chr19 6713251 0.009344 2.056e-16 35372 TRUE reported textfile
2 1 TRUE
4065 rs10922098 T C T C -0.2233 -0.009 0.40011 NA FALSE FALSE FALSE
GCST006061 0.0075 0.2275 537409 outcome TRUE reported 6587_6_LRFN5_LRFN5
LRFN5 chr1 196695521 0.008118 1.412e-166 35338 TRUE reported
textfile 2 1 TRUE
4066 rs1451915 A C A C 0.0721 -0.0107 0.13641 NA FALSE FALSE FALSE
GCST006061 0.0124 0.3895 537409 outcome TRUE reported 6587_6_LRFN5_LRFN5
LRFN5 chr1 196058002 0.011599 5.098e-10 35335 TRUE reported
textfile 2 1 TRUE
4067 rs1707652 T C T C 0.1052 0.0081 0.3102 NA FALSE FALSE FALSE
GCST006061 0.0074 0.2701 537409 outcome TRUE reported 6587_6_LRFN5_LRFN5
LRFN5 chr3 165761011 0.008617 2.811e-34 35339 TRUE reported
textfile 2 1 TRUE
4068 rs7513100 C T C T 0.0636 0 0.14871 NA FALSE FALSE FALSE GCST006061
0.0098 0.9981 537409 outcome TRUE reported 6587_6_LRFN5_LRFN5 LRFN5 chr1
198065935 0.011222 1.449e-08 35343 TRUE reported textfile 2 1 TRUE
4069 rs1047286 A G A G -0.0679 -0.0013 0.24066 NA FALSE FALSE FALSE
GCST006061 0.0098 0.8921 537409 outcome TRUE reported 12030_82_DES_Desmin DES
chr19 6713251 0.009419 5.65e-13 35363 TRUE reported textfile 2 1
TRUE
4070 rs10801660 A G A G 0.0715 -0.0119 0.11574 NA FALSE FALSE FALSE
GCST006061 0.0108 0.2735 537409 outcome TRUE reported 12030_82_DES_Desmin DES
chr1 198272225 0.01256 1.251e-08 35336 TRUE reported textfile 2 1
TRUE
4071 rs10922098 T C T C -0.2384 -0.009 0.40011 NA FALSE FALSE FALSE
GCST006061 0.0075 0.2275 537409 outcome TRUE reported 12030_82_DES_Desmin DES
chr1 196695521 0.008178 8.017e-187 35329 TRUE reported textfile 2
1 TRUE
4072 rs111696008 A G A G -0.1639 -0.0259 0.02443 NA FALSE FALSE FALSE

GCST006061 0.0401 0.519 537409 outcome TRUE reported 12030_82_DES_Desmin DES
chr2 216622986 0.026787 9.442e-10 35350 TRUE reported textfile 2
1 TRUE
4073 rs2686395 C T C T -0.1128 -0.0077 0.3101 NA FALSE FALSE FALSE
GCST006061 0.0074 0.2965 537409 outcome TRUE reported 12030_82_DES_Desmin DES
chr3 165767231 0.008692 1.637e-38 35330 TRUE reported textfile 2
1 TRUE
4074 rs1047286 A G A G -0.0537 -0.0013 0.24066 NA FALSE FALSE FALSE
GCST006061 0.0098 0.8921 537409 outcome TRUE reported 9003_99_MARCO_MARCO
MARCO chr19 6713251 0.009264 6.759e-09 35372 TRUE reported textfile
2 1 TRUE
4075 rs10922098 T C T C -0.1531 -0.009 0.40011 NA FALSE FALSE FALSE
GCST006061 0.0075 0.2275 537409 outcome TRUE reported 9003_99_MARCO_MARCO
MARCO chr1 196695521 0.008071 3.1e-80 35338 TRUE reported textfile
2 1 TRUE
4076 rs11714342 A G A G 0.1725 0.0048 0.01815 NA FALSE FALSE FALSE
GCST006061 0.0325 0.8836 537409 outcome TRUE reported 9003_99_MARCO_MARCO
MARCO chr3 58431175 0.029489 4.928e-09 35358 TRUE reported
textfile 2 1 TRUE
4077 rs1355538 G A G A -0.0646 -0.0076 0.39847 NA FALSE FALSE FALSE
GCST006061 0.0072 0.2923 537409 outcome TRUE reported 9003_99_MARCO_MARCO
MARCO chr3 165787389 0.008091 1.415e-15 35338 TRUE reported
textfile 2 1 TRUE
4078 rs140678946 A G A G 0.3417 0.0043 0.01375 NA FALSE FALSE FALSE
GCST006061 0.0329 0.8967 537409 outcome TRUE reported 9003_99_MARCO_MARCO
MARCO chr3 54542330 0.034815 9.721e-23 35346 TRUE reported
textfile 2 1 TRUE
4079 rs142275326 C A C A 0.3209 -0.001 0.01661 NA FALSE FALSE FALSE
GCST006061 0.028 0.9719 537409 outcome TRUE reported 9003_99_MARCO_MARCO
MARCO chr3 53945107 0.030664 1.249e-25 35346 TRUE reported
textfile 2 1 TRUE
4080 rs41338844 A G A G 0.165 -0.0574 0.02407 NA FALSE FALSE FALSE
GCST006061 0.0321 0.07341 537409 outcome TRUE reported 9003_99_MARCO_MARCO
MARCO chr3 46231460 0.025527 1.021e-10 35344 TRUE reported
textfile 2 1 TRUE
4081 rs704 A G A G 0.0824 -0.0155 0.49189 NA FALSE FALSE FALSE GCST006061
0.0072 0.03144 537409 outcome TRUE reported 9003_99_MARCO_MARCO MARCO chr17
28367840 0.007932 2.795e-25 35360 TRUE reported textfile 2 1 TRUE
4082 rs1047286 A G A G -0.0745 -0.0013 0.24066 NA FALSE FALSE FALSE
GCST006061 0.0098 0.8921 537409 outcome TRUE reported 11967_23_CRABP1_RABP1
CRABP1 chr19 6713251 0.009431 2.81e-15 35363 TRUE reported textfile
2 1 TRUE
4083 rs10922098 T C T C -0.2201 -0.009 0.40011 NA FALSE FALSE FALSE
GCST006061 0.0075 0.2275 537409 outcome TRUE reported 11967_23_CRABP1_RABP1
CRABP1 chr1 196695521 0.008195 6.785e-159 35329 TRUE reported
textfile 2 1 TRUE
4084 rs111696008 A G A G -0.1578 -0.0259 0.02443 NA FALSE FALSE FALSE
GCST006061 0.0401 0.519 537409 outcome TRUE reported 11967_23_CRABP1_RABP1
CRABP1 chr2 216622986 0.026808 3.949e-09 35350 TRUE reported
textfile 2 1 TRUE
4085 rs1707652 T C T C 0.1149 0.0081 0.3102 NA FALSE FALSE FALSE
GCST006061 0.0074 0.2701 537409 outcome TRUE reported 11967_23_CRABP1_RABP1
CRABP1 chr3 165761011 0.008692 6.879e-40 35330 TRUE reported
textfile 2 1 TRUE
4086 rs704 A G A G 0.05 -0.0155 0.49189 NA FALSE FALSE FALSE GCST006061
0.0072 0.03144 537409 outcome TRUE reported 11967_23_CRABP1_RABP1 CRABP1
chr17 28367840 0.008084 6.197e-10 35351 TRUE reported textfile 2
1 TRUE
4087 rs1047286 A G A G -0.054 -0.0013 0.24066 NA FALSE FALSE FALSE
GCST006061 0.0098 0.8921 537409 outcome TRUE reported 12473_48_KCTD5_KCTD5
KCTD5 chr19 6713251 0.009264 5.569e-09 35363 TRUE reported textfile
2 1 TRUE
4088 rs10922098 T C T C -0.1555 -0.009 0.40011 NA FALSE FALSE FALSE
GCST006061 0.0075 0.2275 537409 outcome TRUE reported 12473_48_KCTD5_KCTD5
KCTD5 chr1 196695521 0.008076 1.29e-82 35329 TRUE reported
textfile 2 1 TRUE

4089 rs12478841 G A G A 0.0533 0.0049 0.21569 NA FALSE FALSE FALSE GCST006061 0.008 0.5413 537409 outcome TRUE reported 12473_48_KCTD5_KCTD5 KCTD5 chr2 27588855 0.009557 2.449e-08 35364 TRUE reported textfile 2 1 TRUE

4090 rs2686395 C T C T -0.076 -0.0077 0.3101 NA FALSE FALSE FALSE GCST006061 0.0074 0.2965 537409 outcome TRUE reported 12473_48_KCTD5_KCTD5 KCTD5 chr3 165767231 0.008548 6.073e-19 35330 TRUE reported textfile 2 1 TRUE

4091 rs1047286 A G A G -0.0585 -0.0013 0.24066 NA FALSE FALSE FALSE GCST006061 0.0098 0.8921 537409 outcome TRUE reported 4150_75_PLG_Plasmin PLG chr19 6713251 0.00937 4.292e-10 35372 TRUE reported textfile 2 1 TRUE

4092 rs10922098 T C T C -0.2083 -0.009 0.40011 NA FALSE FALSE FALSE GCST006061 0.0075 0.2275 537409 outcome TRUE reported 4150_75_PLG_Plasmin PLG chr1 196695521 0.008139 1.825e-144 35338 TRUE reported textfile 2 1 TRUE

4093 rs1707652 T C T C 0.0775 0.0081 0.3102 NA FALSE FALSE FALSE GCST006061 0.0074 0.2701 537409 outcome TRUE reported 4150_75_PLG_Plasmin PLG chr3 165761011 0.008641 3.008e-19 35339 TRUE reported textfile 2 1 TRUE

4094 rs1047286 A G A G -0.0591 -0.0013 0.24066 NA FALSE FALSE FALSE GCST006061 0.0098 0.8921 537409 outcome TRUE reported 12891_1_MICALL2_MILK2 MICALL2 chr19 6713251 0.009367 2.805e-10 35363 TRUE reported textfile 2 1 TRUE

4095 rs10922098 T C T C -0.1928 -0.009 0.40011 NA FALSE FALSE FALSE GCST006061 0.0075 0.2275 537409 outcome TRUE reported 12891_1_MICALL2_MILK2 MICALL2 chr1 196695521 0.008144 6.552e-124 35329 TRUE reported textfile 2 1 TRUE

4096 rs12478841 G A G A 0.0542 0.0049 0.21569 NA FALSE FALSE FALSE GCST006061 0.008 0.5413 537409 outcome TRUE reported 12891_1_MICALL2_MILK2 MICALL2 chr2 27588855 0.009652 1.958e-08 35364 TRUE reported textfile 2 1 TRUE

4097 rs1451915 A C A C 0.0637 -0.0107 0.13641 NA FALSE FALSE FALSE GCST006061 0.0124 0.3895 537409 outcome TRUE reported 12891_1_MICALL2_MILK2 MICALL2 chr1 196058002 0.011613 4.126e-08 35326 TRUE reported textfile 2 1 TRUE

4098 rs1707652 T C T C 0.1018 0.0081 0.3102 NA FALSE FALSE FALSE GCST006061 0.0074 0.2701 537409 outcome TRUE reported 12891_1_MICALL2_MILK2 MICALL2 chr3 165761011 0.008631 4.145e-32 35330 TRUE reported textfile 2 1 TRUE

4099 rs704 A G A G 0.1284 -0.0155 0.49189 NA FALSE FALSE FALSE GCST006061 0.0072 0.03144 537409 outcome TRUE reported 12891_1_MICALL2_MILK2 MICALL2 chr17 28367840 0.008004 6.452e-58 35351 TRUE reported textfile 2 1 TRUE

4100 rs1047286 A G A G -0.0546 -0.0013 0.24066 NA FALSE FALSE FALSE GCST006061 0.0098 0.8921 537409 outcome TRUE reported 2631_50_IL10RB_IL_10_Rb IL10RB chr19 6713251 0.009396 6.204e-09 35372 TRUE reported textfile 2 1 TRUE

4101 rs10922098 T C T C -0.1958 -0.009 0.40011 NA FALSE FALSE FALSE GCST006061 0.0075 0.2275 537409 outcome TRUE reported 2631_50_IL10RB_IL_10_Rb IL10RB chr1 196695521 0.008171 6.625e-127 35338 TRUE reported textfile 2 1 TRUE

4102 rs2686395 C T C T -0.0809 -0.0077 0.3101 NA FALSE FALSE FALSE GCST006061 0.0074 0.2965 537409 outcome TRUE reported 2631_50_IL10RB_IL_10_Rb IL10RB chr3 165767231 0.008674 1.09e-20 35339 TRUE reported textfile 2 1 TRUE

4103 rs35209896 A C A C 0.0816 0.0267 0.09263 NA FALSE FALSE FALSE GCST006061 0.0355 0.4521 537409 outcome TRUE reported 2631_50_IL10RB_IL_10_Rb IL10RB chr6 32519009 0.013752 2.959e-09 35439 TRUE reported textfile 2 1 TRUE

4104 rs4455239 C T C T 0.0705 0.0081 0.43466 NA FALSE FALSE FALSE GCST006061 0.0074 0.2746 537409 outcome TRUE reported 2631_50_IL10RB_IL_10_Rb IL10RB chr21 33281729 0.008117 3.766e-18 35376 TRUE reported textfile 2 1 TRUE

4105 rs1047891 A C A C -0.062 0.0155 0.26363 NA FALSE FALSE FALSE GCST006061 0.0079 0.05042 537409 outcome TRUE reported 4913_78_CCL16_HCC_4 CCL16 chr2 210675783 0.009507 6.948e-11 35353 TRUE reported

textfile 2 1 TRUE
4106 rs113132408 T C T C -1.3266 -0.0191 0.01368 NA FALSE FALSE FALSE
GCST006061 0.0588 0.7458 537409 outcome TRUE reported 4913_78_CCL16_HCC_4
CCL16 chr17 35959906 0.037454 1e-200 35366 TRUE reported textfile
2 1 TRUE
4107 rs148663776 T C T C -0.1832 -0.065 0.02373 NA FALSE FALSE FALSE
GCST006061 0.0461 0.1583 537409 outcome TRUE reported 4913_78_CCL16_HCC_4
CCL16 chr17 41334614 0.028798 1.998e-10 35385 TRUE reported
textfile 2 1 TRUE
4108 rs2268078 A G A G 0.0536 0.0097 0.35799 NA FALSE FALSE FALSE
GCST006061 0.0076 0.2042 537409 outcome TRUE reported 4913_78_CCL16_HCC_4
CCL16 chr20 34008898 0.0086 4.583e-10 35367 TRUE reported textfile
2 1 TRUE
4109 rs4251784 A G A G -0.4369 0.0162 0.04233 NA FALSE FALSE FALSE
GCST006061 0.0321 0.6139 537409 outcome TRUE reported 4913_78_CCL16_HCC_4
CCL16 chr17 35844448 0.021482 5.962e-92 35370 TRUE reported
textfile 2 1 TRUE
4110 rs4682863 G A G A -0.0522 0.0165 0.39195 NA FALSE FALSE FALSE
GCST006061 0.0072 0.02287 537409 outcome TRUE reported 4913_78_CCL16_HCC_4
CCL16 chr3 42836219 0.008551 1.03e-09 35353 TRUE reported
textfile 2 1 TRUE
4111 rs6505427 C T C T 0.173 -0.0119 0.06362 NA FALSE FALSE FALSE
GCST006061 0.0134 0.3722 537409 outcome TRUE reported 4913_78_CCL16_HCC_4
CCL16 chr17 34697182 0.017849 3.243e-22 35371 TRUE reported
textfile 2 1 TRUE
4112 rs72829262 A G A G -0.2407 0.0232 0.04454 NA FALSE FALSE FALSE
GCST006061 0.0195 0.2335 537409 outcome TRUE reported 4913_78_CCL16_HCC_4
CCL16 chr17 34505152 0.021296 1.277e-29 35365 TRUE reported
textfile 2 1 TRUE
4113 rs1047891 A C A C -0.0599 0.0155 0.26363 NA FALSE FALSE FALSE
GCST006061 0.0079 0.05042 537409 outcome TRUE reported
5658_64_F13B_coagulation_factor_XIII_B F13B chr2 210675783 0.009131
5.384e-11 35353 TRUE reported textfile 2 1 TRUE
4114 rs111430107 T C T C 0.2215 -0.0572 0.01971 NA FALSE FALSE FALSE
GCST006061 0.0409 0.1621 537409 outcome TRUE reported
5658_64_F13B_coagulation_factor_XIII_B F13B chr1 196109945 0.028847
1.612e-14 35337 TRUE reported textfile 2 1 TRUE
4115 rs12590165 A G A G -0.0609 -0.0103 0.15971 NA FALSE FALSE FALSE
GCST006061 0.0107 0.3317 537409 outcome TRUE reported
5658_64_F13B_coagulation_factor_XIII_B F13B chr14 68846905 0.010923
2.472e-08 35357 TRUE reported textfile 2 1 TRUE
4116 rs1412636 A G A G -0.3923 2e-04 0.13677 NA FALSE FALSE FALSE
GCST006061 0.0094 0.9863 537409 outcome TRUE reported
5658_64_F13B_coagulation_factor_XIII_B F13B chr1 196982329 0.011738 1e-200
35338 TRUE reported textfile 2 1 TRUE
4117 rs4220 A G A G 0.0658 0.0061 0.15374 NA FALSE FALSE FALSE GCST006061
0.0093 0.5115 537409 outcome TRUE reported
5658_64_F13B_coagulation_factor_XIII_B F13B chr4 154570607 0.011226
4.587e-09 35381 TRUE reported textfile 2 1 TRUE
4118 rs5982 A G A G 0.0584 -0.0102 0.18579 NA FALSE FALSE FALSE GCST006061
0.0089 0.2519 537409 outcome TRUE reported
5658_64_F13B_coagulation_factor_XIII_B F13B chr6 6174633 0.010337 1.607e-08
35387 TRUE reported textfile 2 1 TRUE
4119 rs73079476 C A C A -0.0658 0.02 0.16415 NA FALSE FALSE FALSE
GCST006061 0.0098 0.04092 537409 outcome TRUE reported
5658_64_F13B_coagulation_factor_XIII_B F13B chr12 21190899 0.010749
9.263e-10 35423 TRUE reported textfile 2 1 TRUE
4120 rs75109228 A C A C -0.1388 -0.0108 0.10086 NA FALSE FALSE FALSE
GCST006061 0.014 0.4405 537409 outcome TRUE reported
5658_64_F13B_coagulation_factor_XIII_B F13B chr1 196151633 0.013357
2.717e-25 35337 TRUE reported textfile 2 1 TRUE
4121 rs7532568 T G T G -0.0678 0.003 0.39918 NA FALSE FALSE FALSE
GCST006061 0.0075 0.6891 537409 outcome TRUE reported
5658_64_F13B_coagulation_factor_XIII_B F13B chr1 195957479 0.008322
3.724e-16 35338 TRUE reported textfile 2 1 TRUE
4122 rs79844463 G T G T 0.1287 -0.0063 0.04817 NA FALSE FALSE FALSE

GCST006061 0.0256 0.806 537409 outcome TRUE reported
5658_64_F13B_coagulation_factor_XIII_B F13B chr1 197337274 0.019266
2.384e-11 35336 TRUE reported textfile 2 1 TRUE
4123 rs1047891 A C A C 0.0775 0.0155 0.26363 NA FALSE FALSE FALSE
GCST006061 0.0079 0.05042 537409 outcome TRUE reported 3617_80_HGFAC_HGFA
HGFAC chr2 210675783 0.00851 8.436e-20 35353 TRUE reported textfile
2 1 TRUE
4124 rs114185131 T C T C -0.6478 0.1401 0.01511 NA FALSE FALSE FALSE
GCST006061 0.1955 0.4737 537409 outcome TRUE reported 3617_80_HGFAC_HGFA
HGFAC chr4 3724127 0.03638 6.291e-71 35279 TRUE reported textfile 2
1 TRUE
4125 rs142962885 A G A G -0.7093 0.0191 0.01317 NA FALSE FALSE FALSE
GCST006061 0.0372 0.6082 537409 outcome TRUE reported 3617_80_HGFAC_HGFA
HGFAC chr4 2044719 0.039367 1.422e-72 35277 TRUE reported textfile
2 1 TRUE
4126 rs147126736 T C T C -0.4626 -0.0444 0.01777 NA FALSE FALSE FALSE
GCST006061 0.032 0.1648 537409 outcome TRUE reported 3617_80_HGFAC_HGFA
HGFAC chr4 2153616 0.033619 4.437e-43 35278 TRUE reported textfile
2 1 TRUE
4127 rs3775067 A G A G -0.2775 0.0114 0.39186 NA FALSE FALSE FALSE
GCST006061 0.0073 0.1195 537409 outcome TRUE reported 3617_80_HGFAC_HGFA
HGFAC chr4 2886895 0.008919 1e-200 35283 TRUE reported textfile 2
1 TRUE
4128 rs4841133 G A G A 0.0924 -0.0155 0.0746 NA FALSE FALSE FALSE
GCST006061 0.0128 0.2254 537409 outcome TRUE reported 3617_80_HGFAC_HGFA
HGFAC chr8 9326154 0.014189 7.412e-11 35385 TRUE reported textfile
2 1 TRUE
4129 rs75176598 A G A G -0.3251 -0.039 0.01387 NA FALSE FALSE FALSE
GCST006061 0.0401 0.3303 537409 outcome TRUE reported 3617_80_HGFAC_HGFA
HGFAC chr4 1555594 0.037129 2.023e-18 35273 TRUE reported textfile
2 1 TRUE
4130 rs1047891 A C A C -0.0581 0.0155 0.26363 NA FALSE FALSE FALSE
GCST006061 0.0079 0.05042 537409 outcome TRUE reported 15562_24_GUSB_BGLR
GUSB chr2 210675783 0.009286 3.937e-10 35353 TRUE reported
textfile 2 1 TRUE
4131 rs10778141 A G A G -0.11 2e-04 0.18198 NA FALSE FALSE FALSE
GCST006061 0.0092 0.982 537409 outcome TRUE reported 15562_24_GUSB_BGLR
GUSB chr12 101699457 0.010703 8.909e-25 35364 TRUE reported
textfile 2 1 TRUE
4132 rs10949891 A G A G -0.0681 0.0066 0.23887 NA FALSE FALSE FALSE
GCST006061 0.0083 0.4227 537409 outcome TRUE reported 15562_24_GUSB_BGLR
GUSB chr7 64329528 0.009549 9.911e-13 35344 TRUE reported
textfile 2 1 TRUE
4133 rs111849297 T G T G -0.0603 -0.0015 0.32627 NA FALSE FALSE FALSE
GCST006061 0.0081 0.8513 537409 outcome TRUE reported 15562_24_GUSB_BGLR
GUSB chr12 101551411 0.008801 7.317e-12 35363 TRUE reported
textfile 2 1 TRUE
4134 rs116587722 C T C T 0.1819 0.0084 0.05421 NA FALSE FALSE FALSE
GCST006061 0.0173 0.6281 537409 outcome TRUE reported 15562_24_GUSB_BGLR
GUSB chr4 118869362 0.018142 1.168e-23 35351 TRUE reported
textfile 2 1 TRUE
4135 rs142443403 T C T C -0.3998 0.045 0.01163 NA FALSE FALSE FALSE
GCST006061 0.0671 0.502 537409 outcome TRUE reported 15562_24_GUSB_BGLR
GUSB chr7 57757974 0.038424 2.353e-25 35339 TRUE reported
textfile 2 1 TRUE
4136 rs145851867 A G A G -0.305 -0.0485 0.03705 NA FALSE FALSE FALSE
GCST006061 0.0305 0.1117 537409 outcome TRUE reported 15562_24_GUSB_BGLR
GUSB chr7 65837235 0.021791 1.636e-44 35358 TRUE reported
textfile 2 1 TRUE
4137 rs147233090 T C T C 0.2611 0.0375 0.02693 NA FALSE FALSE FALSE
GCST006061 0.0254 0.1396 537409 outcome TRUE reported 15562_24_GUSB_BGLR
GUSB chr15 43735849 0.02502 1.707e-25 35362 TRUE reported textfile
2 1 TRUE
4138 rs174574 C A C A -0.0681 -0.0115 0.39343 NA FALSE FALSE FALSE
GCST006061 0.0074 0.122 537409 outcome TRUE reported 15562_24_GUSB_BGLR
GUSB chr11 61832870 0.008317 2.661e-16 35367 TRUE reported

textfile 2 1 TRUE
4139 rs17532301 A G A G 0.0902 -0.016 0.06761 NA FALSE FALSE FALSE
GCST006061 0.0149 0.282 537409 outcome TRUE reported 15562_24_GUSB_BGLR
GUSB chr13 41034911 0.016204 2.601e-08 35358 TRUE reported
textfile 2 1 TRUE
4140 rs1788606 T C T C 0.0891 -4.00E-04 0.35125 NA FALSE FALSE FALSE
GCST006061 0.0078 0.9577 537409 outcome TRUE reported 15562_24_GUSB_BGLR
GUSB chr18 74297397 0.008559 2.225e-25 35378 TRUE reported
textfile 2 1 TRUE
4141 rs1800450 T C T C 0.1006 -0.0064 0.13736 NA FALSE FALSE FALSE
GCST006061 0.01 0.5249 537409 outcome TRUE reported 15562_24_GUSB_BGLR
GUSB chr10 52771475 0.011767 1.239e-17 35361 TRUE reported
textfile 2 1 TRUE
4142 rs190670827 T G T G 0.1028 0.0138 0.05919 NA FALSE FALSE FALSE
GCST006061 0.0334 0.6799 537409 outcome TRUE reported 15562_24_GUSB_BGLR
GUSB chr7 67013189 0.017458 3.901e-09 35357 TRUE reported
textfile 2 1 TRUE
4143 rs2900660 A C A C 0.0648 0.0163 0.45429 NA FALSE FALSE FALSE
GCST006061 0.0074 0.02751 537409 outcome TRUE reported 15562_24_GUSB_BGLR
GUSB chr19 12912045 0.008219 3.171e-15 35381 TRUE reported
textfile 2 1 TRUE
4144 rs34356500 T G T G 0.1393 0.0256 0.4642 NA FALSE FALSE FALSE
GCST006061 0.0072 0.0004039 537409 outcome TRUE reported 15562_24_GUSB_BGLR
GUSB chr7 66771620 0.008204 1.175e-64 35358 TRUE reported
textfile 2 1 TRUE
4145 rs4420638 G A G A -0.0569 0.0079 0.19982 NA FALSE FALSE FALSE
GCST006061 0.0096 0.411 537409 outcome TRUE reported 15562_24_GUSB_BGLR
GUSB chr19 44919689 0.01028 3.109e-08 35367 TRUE reported textfile
2 1 TRUE
4146 rs4574742 G A G A 0.0566 0.0056 0.3137 NA FALSE FALSE FALSE
GCST006061 0.0077 0.4686 537409 outcome TRUE reported 15562_24_GUSB_BGLR
GUSB chr7 25955443 0.008814 1.347e-10 35372 TRUE reported
textfile 2 1 TRUE
4147 rs4764822 T C T C -0.2767 0.0045 0.2787 NA FALSE FALSE FALSE
GCST006061 0.0078 0.564 537409 outcome TRUE reported 15562_24_GUSB_BGLR
GUSB chr12 101822895 0.009138 1e-200 35364 TRUE reported textfile
2 1 TRUE
4148 rs56272941 C T C T -0.1051 -0.0107 0.0513 NA FALSE FALSE FALSE
GCST006061 0.0261 0.6813 537409 outcome TRUE reported 15562_24_GUSB_BGLR
GUSB chr12 102702636 0.018618 1.65e-08 35363 TRUE reported
textfile 2 1 TRUE
4149 rs62440901 T C T C -0.0807 0.0026 0.15439 NA FALSE FALSE FALSE
GCST006061 0.0106 0.8028 537409 outcome TRUE reported 15562_24_GUSB_BGLR
GUSB chr6 160148036 0.011127 4.087e-13 35389 TRUE reported
textfile 2 1 TRUE
4150 rs62455836 C T C T -0.1618 -0.0156 0.02346 NA FALSE FALSE FALSE
GCST006061 0.0231 0.5001 537409 outcome TRUE reported 15562_24_GUSB_BGLR
GUSB chr7 70600206 0.02691 1.825e-09 35359 TRUE reported textfile
2 1 TRUE
4151 rs76476993 A G A G -0.3068 -0.0328 0.01091 NA FALSE FALSE FALSE
GCST006061 0.0368 0.3734 537409 outcome TRUE reported 15562_24_GUSB_BGLR
GUSB chr7 55548382 0.042224 3.703e-13 35339 TRUE reported
textfile 2 1 TRUE
4152 rs7970695 A G A G 0.0522 0.0118 0.35262 NA FALSE FALSE FALSE
GCST006061 0.0072 0.1015 537409 outcome TRUE reported 15562_24_GUSB_BGLR
GUSB chr12 120985573 0.008587 1.209e-09 35365 TRUE reported
textfile 2 1 TRUE
4153 rs1047891 A C A C 0.0824 0.0155 0.26363 NA FALSE FALSE FALSE
GCST006061 0.0079 0.05042 537409 outcome TRUE reported
3799_11_CA3_Carbonic_anhydrase_III CA3 chr2 210675783 0.009217 3.876e-19
35353 TRUE reported textfile 2 1 TRUE
4154 rs11085825 T C T C -0.0595 -0.0083 0.4072 NA FALSE FALSE FALSE
GCST006061 0.0076 0.2715 537409 outcome TRUE reported
3799_11_CA3_Carbonic_anhydrase_III CA3 chr19 12896644 0.008294 7.288e-13
35381 TRUE reported textfile 2 1 TRUE
4155 rs11097789 C T C T 0.0499 -0.0243 0.35148 NA FALSE FALSE FALSE

GCST006061 0.0075 0.001154 537409 outcome TRUE reported
3799_11_CA3_Carbonic_anhydrase_III CA3 chr4 102581605 0.008445 3.452e-09
35344 TRUE reported textfile 2 1 TRUE
4156 rs112126364 A G A G 0.2665 0.0105 0.02406 NA FALSE FALSE FALSE
GCST006061 0.0265 0.6911 537409 outcome TRUE reported
3799_11_CA3_Carbonic_anhydrase_III CA3 chr8 85551362 0.026386 5.53e-24
35349 TRUE reported textfile 2 1 TRUE
4157 rs1387065 C T C T -0.1582 -0.0342 0.02267 NA FALSE FALSE FALSE
GCST006061 0.0268 0.2023 537409 outcome TRUE reported
3799_11_CA3_Carbonic_anhydrase_III CA3 chr8 84722571 0.0275 8.785e-09 35348
TRUE reported textfile 2 1 TRUE
4158 rs1405212 C T C T -0.0487 -0.0095 0.39069 NA FALSE FALSE FALSE
GCST006061 0.0074 0.2031 537409 outcome TRUE reported
3799_11_CA3_Carbonic_anhydrase_III CA3 chr6 117169501 0.008254 3.638e-09
35353 TRUE reported textfile 2 1 TRUE
4159 rs192681919 G A G A 0.3744 0.0424 0.0109 NA FALSE FALSE FALSE
GCST006061 0.0721 0.5569 537409 outcome TRUE reported
3799_11_CA3_Carbonic_anhydrase_III CA3 chr8 84486359 0.039192 1.26e-21
35348 TRUE reported textfile 2 1 TRUE
4160 rs3811444 T C T C 0.1058 0.0103 0.34232 NA FALSE FALSE FALSE
GCST006061 0.0077 0.1812 537409 outcome TRUE reported
3799_11_CA3_Carbonic_anhydrase_III CA3 chr1 247876149 0.008609 1.039e-34
35156 TRUE reported textfile 2 1 TRUE
4161 rs56387760 G A G A 0.1574 0.006 0.0284 NA FALSE FALSE FALSE
GCST006061 0.0254 0.8128 537409 outcome TRUE reported
3799_11_CA3_Carbonic_anhydrase_III CA3 chr17 29480398 0.023776 3.592e-11
35361 TRUE reported textfile 2 1 TRUE
4162 rs72749982 A G A G -0.2307 -0.0019 0.02666 NA FALSE FALSE FALSE
GCST006061 0.0312 0.9517 537409 outcome TRUE reported
3799_11_CA3_Carbonic_anhydrase_III CA3 chr1 234643320 0.025442 1.216e-19
35361 TRUE reported textfile 2 1 TRUE
4163 rs1047891 A C A C -0.0883 0.0155 0.26363 NA FALSE FALSE FALSE
GCST006061 0.0079 0.05042 537409 outcome TRUE reported
18313_4_ASRGL1_Asparaginase_like_protein_1 ASRGL1 chr2 210675783 0.009121
3.637e-22 35353 TRUE reported textfile 2 1 TRUE
4164 rs12275541 A G A G -0.2112 -0.0068 0.31592 NA FALSE FALSE FALSE
GCST006061 0.0078 0.3882 537409 outcome TRUE reported
18313_4_ASRGL1_Asparaginase_like_protein_1 ASRGL1 chr11 62322339 0.008638
5.143e-132 35364 TRUE reported textfile 2 1 TRUE
4165 rs1354034 C T C T 0.0655 0.0088 0.38119 NA FALSE FALSE FALSE
GCST006061 0.0073 0.2248 537409 outcome TRUE reported
18313_4_ASRGL1_Asparaginase_like_protein_1 ASRGL1 chr3 56815721 0.008235
1.815e-15 35356 TRUE reported textfile 2 1 TRUE
4166 rs572515 G A G A -0.056 -0.0113 0.3973 NA FALSE FALSE FALSE
GCST006061 0.0075 0.1355 537409 outcome TRUE reported
18313_4_ASRGL1_Asparaginase_like_protein_1 ASRGL1 chr1 196677131 0.008194
8.257e-12 35338 TRUE reported textfile 2 1 TRUE
4167 rs7073753 T C T C 0.0546 -0.0472 0.4356 NA FALSE FALSE FALSE
GCST006061 0.0072 5.02e-11 537409 outcome TRUE reported
18313_4_ASRGL1_Asparaginase_like_protein_1 ASRGL1 chr10 63303060 0.008066
1.293e-11 35351 TRUE reported textfile 2 1 TRUE
4168 rs1047891 A C A C -0.0508 0.0155 0.26363 NA FALSE FALSE FALSE
GCST006061 0.0079 0.05042 537409 outcome TRUE reported 19622_7_INHBA_Activin_A
INHBA chr2 210675783 0.009226 3.666e-08 35353 TRUE reported
textfile 2 1 TRUE
4169 rs12614487 T C T C -0.0884 -0.0059 0.08168 NA FALSE FALSE FALSE
GCST006061 0.0126 0.6409 537409 outcome TRUE reported 19622_7_INHBA_Activin_A
INHBA chr2 157578057 0.014869 2.761e-09 35334 TRUE reported
textfile 2 1 TRUE
4170 rs3741414 T C T C -0.0702 -0.0052 0.31233 NA FALSE FALSE FALSE
GCST006061 0.0087 0.548 537409 outcome TRUE reported 19622_7_INHBA_Activin_A
INHBA chr12 57450266 0.008684 6.292e-16 35367 TRUE reported
textfile 2 1 TRUE
4171 rs7311982 C T C T 0.065 -0.0049 0.30107 NA FALSE FALSE FALSE
GCST006061 0.0078 0.5309 537409 outcome TRUE reported 19622_7_INHBA_Activin_A
INHBA chr12 9162261 0.00886 2.195e-13 35387 TRUE reported textfile 2

1 TRUE
4172 rs1047891 A C A C -0.0618 0.0155 0.26363 NA FALSE FALSE FALSE
GCST006061 0.0079 0.05042 537409 outcome TRUE reported 12630_8_ARFIP2_ARFP2
ARFIP2 chr2 210675783 0.00928 2.753e-11 35344 TRUE reported textfile
2 1 TRUE
4173 rs112963922 T C T C -0.2128 0.0542 0.01119 NA FALSE FALSE FALSE
GCST006061 0.0399 0.1741 537409 outcome TRUE reported 12630_8_ARFIP2_ARFP2
ARFIP2 chr14 94411531 0.038237 2.617e-08 35362 TRUE reported
textfile 2 1 TRUE
4174 rs116009877 A G A G -0.1134 -0.0393 0.06469 NA FALSE FALSE FALSE
GCST006061 0.0161 0.01479 537409 outcome TRUE reported 12630_8_ARFIP2_ARFP2
ARFIP2 chr6 25715429 0.016472 5.807e-12 35366 TRUE reported
textfile 2 1 TRUE
4175 rs2275355 T C T C 0.0501 1e-04 0.28576 NA FALSE FALSE FALSE
GCST006061 0.0081 0.9933 537409 outcome TRUE reported 12630_8_ARFIP2_ARFP2
ARFIP2 chr1 23397690 0.009073 3.352e-08 35363 TRUE reported
textfile 2 1 TRUE
4176 rs3219110 C T C T -0.0694 0.0067 0.45417 NA FALSE FALSE FALSE
GCST006061 0.0074 0.3612 537409 outcome TRUE reported 12630_8_ARFIP2_ARFP2
ARFIP2 chr1 226370177 0.008188 2.338e-17 35348 TRUE reported
textfile 2 1 TRUE
4177 rs62294359 A G A G 0.0533 0.0164 0.24788 NA FALSE FALSE FALSE
GCST006061 0.0081 0.04287 537409 outcome TRUE reported 12630_8_ARFIP2_ARFP2
ARFIP2 chr3 165744429 0.00945 1.698e-08 35330 TRUE reported textfile
2 1 TRUE
4178 rs72649156 G T G T -0.2453 -0.0462 0.01575 NA FALSE FALSE FALSE
GCST006061 0.0289 0.1094 537409 outcome TRUE reported 12630_8_ARFIP2_ARFP2
ARFIP2 chr4 73623958 0.032567 4.99e-14 35341 TRUE reported
textfile 2 1 TRUE
4179 rs73224404 G A G A -0.0626 -0.0188 0.22123 NA FALSE FALSE FALSE
GCST006061 0.0091 0.03969 537409 outcome TRUE reported 12630_8_ARFIP2_ARFP2
ARFIP2 chr8 23225988 0.009738 1.291e-10 35359 TRUE reported
textfile 2 1 TRUE
4180 rs77542162 G A G A 0.2081 0.0177 0.025 NA FALSE FALSE FALSE
GCST006061 0.0302 0.557 537409 outcome TRUE reported 12630_8_ARFIP2_ARFP2
ARFIP2 chr17 69085137 0.025683 5.38e-16 35362 TRUE reported
textfile 2 1 TRUE
4181 rs10479093 A G A G -0.056 0.0026 0.38972 NA FALSE FALSE FALSE
GCST006061 0.0073 0.7247 537409 outcome TRUE reported 6060_2_PIP_PIP PIP
chr5 135172869 0.008594 7.203e-11 35360 TRUE reported textfile 2
1 TRUE
4182 rs10876635 G A G A 0.0596 -4.00E-04 0.48965 NA FALSE FALSE FALSE
GCST006061 0.0072 0.9562 537409 outcome TRUE reported 6060_2_PIP_PIP PIP
chr12 54852845 0.008393 1.234e-12 35375 TRUE reported textfile 2
1 TRUE
4183 rs137860941 A G A G 0.1082 0.02 0.07389 NA FALSE FALSE FALSE
GCST006061 0.0224 0.3734 537409 outcome TRUE reported 6060_2_PIP_PIP PIP
chr7 143721834 0.016578 6.718e-11 35371 TRUE reported textfile 2
1 TRUE
4184 rs148389263 T C T C -0.1865 0.0463 0.01987 NA FALSE FALSE FALSE
GCST006061 0.0294 0.1152 537409 outcome TRUE reported 6060_2_PIP_PIP PIP
chr7 144801360 0.030451 9.092e-10 35374 TRUE reported textfile 2
1 TRUE
4185 rs150747152 T C T C -0.3392 -0.0021 0.01168 NA FALSE FALSE FALSE
GCST006061 0.0432 0.9613 537409 outcome TRUE reported 6060_2_PIP_PIP PIP
chr7 141359833 0.0397 1.295e-17 35375 TRUE reported textfile 2 1
TRUE
4186 rs17169742 G A G A -0.3507 -0.0722 0.01606 NA FALSE FALSE FALSE
GCST006061 0.0337 0.03221 537409 outcome TRUE reported 6060_2_PIP_PIP PIP
chr7 145530267 0.033915 4.625e-25 35373 TRUE reported textfile 2
1 TRUE
4187 rs186410432 C A C A 0.1789 -0.0134 0.01998 NA FALSE FALSE FALSE
GCST006061 0.0417 0.7487 537409 outcome TRUE reported 6060_2_PIP_PIP PIP
chr7 140372654 0.030903 7.08e-09 35375 TRUE reported textfile 2
1 TRUE
4188 rs2227411 G A G A 0.0875 0.0072 0.15372 NA FALSE FALSE FALSE

GCST006061 0.0093 0.444 537409 outcome TRUE reported 6060_2_PIP_PIP PIP
chr4 154567933 0.0117 7.495e-14 35381 TRUE reported textfile 2 1
TRUE

4189 rs6678914 A G A G 0.0473 0.0239 0.41141 NA FALSE FALSE FALSE
GCST006061 0.0073 0.001025 537409 outcome TRUE reported 6060_2_PIP_PIP PIP
chr1 202218048 0.008519 2.817e-08 35361 TRUE reported textfile 2
1 TRUE

4190 rs681343 T C T C 0.1524 0.0054 0.3954 NA FALSE FALSE FALSE
GCST006061 0.0076 0.4742 537409 outcome TRUE reported 6060_2_PIP_PIP PIP
chr19 48703205 0.008568 8.877e-71 35377 TRUE reported textfile 2
1 TRUE

4191 rs74920406 T C T C -0.1224 0.0118 0.04848 NA FALSE FALSE FALSE
GCST006061 0.0197 0.5477 537409 outcome TRUE reported 6060_2_PIP_PIP PIP
chr1 109161903 0.019545 3.791e-10 35343 TRUE reported textfile 2
1 TRUE

4192 rs8177171 G A G A -0.5384 0.0267 0.02152 NA FALSE FALSE FALSE
GCST006061 0.0272 0.3265 537409 outcome TRUE reported 6060_2_PIP_PIP PIP
chr7 142859983 0.02915 3.611e-76 35374 TRUE reported textfile 2 1
TRUE

4193 rs11670056 T C T C -0.1153 -0.0027 0.05756 NA FALSE FALSE FALSE
GCST006061 0.0151 0.8572 537409 outcome TRUE reported
2836_68_LCN2_Lipocalin_2 LCN2 chr19 18479133 0.017681 6.974e-11 35352
TRUE reported textfile 2 1 TRUE

4194 rs13277237 A G A G -0.0529 0.0084 0.40349 NA FALSE FALSE FALSE
GCST006061 0.0071 0.2393 537409 outcome TRUE reported
2836_68_LCN2_Lipocalin_2 LCN2 chr8 129592317 0.008454 3.911e-10 35372
TRUE reported textfile 2 1 TRUE

4195 rs1555405 A G A G -0.0503 -0.0047 0.30381 NA FALSE FALSE FALSE
GCST006061 0.0081 0.5592 537409 outcome TRUE reported
2836_68_LCN2_Lipocalin_2 LCN2 chr14 100710432 0.009035 2.59e-08 35380
TRUE reported textfile 2 1 TRUE

4196 rs1680789 T G T G 0.0478 0.0013 0.42964 NA FALSE FALSE FALSE
GCST006061 0.0072 0.8541 537409 outcome TRUE reported
2836_68_LCN2_Lipocalin_2 LCN2 chr3 128906947 0.008364 1.096e-08 35363
TRUE reported textfile 2 1 TRUE

4197 rs2712431 A C A C 0.0629 0.0015 0.37659 NA FALSE FALSE FALSE
GCST006061 0.0078 0.8421 537409 outcome TRUE reported
2836_68_LCN2_Lipocalin_2 LCN2 chr3 128598047 0.008501 1.375e-13 35363
TRUE reported textfile 2 1 TRUE

4198 rs3014874 A G A G 0.0587 -0.0044 0.25791 NA FALSE FALSE FALSE
GCST006061 0.0084 0.5994 537409 outcome TRUE reported
2836_68_LCN2_Lipocalin_2 LCN2 chr1 153365467 0.009532 7.348e-10 35360
TRUE reported textfile 2 1 TRUE

4199 rs35795243 A C A C -0.058 0.0117 0.23178 NA FALSE FALSE FALSE
GCST006061 0.0088 0.1853 537409 outcome TRUE reported
2836_68_LCN2_Lipocalin_2 LCN2 chr19 12487433 0.009803 3.286e-09 35379
TRUE reported textfile 2 1 TRUE

4200 rs3814526 G A G A -0.1541 -0.012 0.03943 NA FALSE FALSE FALSE
GCST006061 0.0146 0.4108 537409 outcome TRUE reported
2836_68_LCN2_Lipocalin_2 LCN2 chr9 128148408 0.02132 4.899e-13 35358
TRUE reported textfile 2 1 TRUE

4201 rs607138 G A G A -0.0487 -0.0107 0.47296 NA FALSE FALSE FALSE
GCST006061 0.0073 0.1438 537409 outcome TRUE reported
2836_68_LCN2_Lipocalin_2 LCN2 chr6 16749336 0.008308 4.571e-09 35385
TRUE reported textfile 2 1 TRUE

4202 rs6503533 T C T C -0.1058 0.0194 0.36413 NA FALSE FALSE FALSE
GCST006061 0.0073 0.008033 537409 outcome TRUE reported
2836_68_LCN2_Lipocalin_2 LCN2 chr17 40028327 0.008607 9.975e-35 35379
TRUE reported textfile 2 1 TRUE

4203 rs6870370 C T C T 0.0662 -0.0078 0.3332 NA FALSE FALSE FALSE
GCST006061 0.0077 0.3112 537409 outcome TRUE reported
2836_68_LCN2_Lipocalin_2 LCN2 chr5 95915188 0.008779 4.687e-14 35348
TRUE reported textfile 2 1 TRUE

4204 rs74172675 C T C T -0.0528 0.0041 0.30662 NA FALSE FALSE FALSE
GCST006061 0.0082 0.6184 537409 outcome TRUE reported
2836_68_LCN2_Lipocalin_2 LCN2 chr19 4962959 0.008968 3.918e-09 35360

TRUE reported textfile 2 1 TRUE
4205 rs77924615 A G A G -0.0578 0.0136 0.19701 NA FALSE FALSE FALSE
GCST006061 0.0092 0.1375 537409 outcome TRUE reported
2836_68_LCN2_Lipocalin_2 LCN2 chr16 20381010 0.010484 3.525e-08 35355
TRUE reported textfile 2 1 TRUE
4206 rs10489705 A G A G -0.2109 0.0715 0.01287 NA FALSE FALSE FALSE
GCST006061 0.0288 0.01311 537409 outcome TRUE reported 15509_2_NAGLU_NAG
NAGLU chr1 185624307 0.035142 1.956e-09 35358 TRUE reported
textfile 2 1 TRUE
4207 rs10797949 G A G A -0.0473 -0.007 0.44574 NA FALSE FALSE FALSE
GCST006061 0.0074 0.3465 537409 outcome TRUE reported 15509_2_NAGLU_NAG
NAGLU chr1 184243993 0.007697 7.999e-10 35360 TRUE reported
textfile 2 1 TRUE
4208 rs116394272 A G A G 0.3376 0.1037 0.02162 NA FALSE FALSE FALSE
GCST006061 0.0388 0.007564 537409 outcome TRUE reported 15509_2_NAGLU_NAG
NAGLU chr1 199284878 0.026675 1.033e-36 35346 TRUE reported
textfile 2 1 TRUE
4209 rs12083166 G A G A 0.1779 -0.0361 0.01601 NA FALSE FALSE FALSE
GCST006061 0.0401 0.3677 537409 outcome TRUE reported 15509_2_NAGLU_NAG
NAGLU chr1 202157555 0.030479 5.323e-09 35351 TRUE reported
textfile 2 1 TRUE
4210 rs12565263 A G A G -0.1996 0.0175 0.01246 NA FALSE FALSE FALSE
GCST006061 0.0194 0.3695 537409 outcome TRUE reported 15509_2_NAGLU_NAG
NAGLU chr1 200513346 0.034627 8.198e-09 35355 TRUE reported
textfile 2 1 TRUE
4211 rs137905573 G A G A 0.384 0.0472 0.01524 NA FALSE FALSE FALSE
GCST006061 0.0427 0.2689 537409 outcome TRUE reported 15509_2_NAGLU_NAG
NAGLU chr1 199276405 0.030517 2.609e-36 35346 TRUE reported
textfile 2 1 TRUE
4212 rs139322023 T C T C 0.1527 -0.0168 0.03259 NA FALSE FALSE FALSE
GCST006061 0.0252 0.5046 537409 outcome TRUE reported 15509_2_NAGLU_NAG
NAGLU chr17 48119431 0.020008 2.312e-14 35364 TRUE reported
textfile 2 1 TRUE
4213 rs139325057 A G A G -0.1734 -0.0079 0.021 NA FALSE FALSE FALSE
GCST006061 0.0453 0.8622 537409 outcome TRUE reported 15509_2_NAGLU_NAG
NAGLU chr17 42752122 0.025074 4.658e-12 35372 TRUE reported
textfile 2 1 TRUE
4214 rs140629687 G A G A -0.3388 -0.0102 0.01131 NA FALSE FALSE FALSE
GCST006061 0.0425 0.8099 537409 outcome TRUE reported 15509_2_NAGLU_NAG
NAGLU chr1 195401104 0.036457 1.498e-20 35340 TRUE reported
textfile 2 1 TRUE
4215 rs146409195 A G A G -0.0971 0.0275 0.06218 NA FALSE FALSE FALSE
GCST006061 0.0181 0.1285 537409 outcome TRUE reported 15509_2_NAGLU_NAG
NAGLU chr1 184842622 0.015867 9.381e-10 35360 TRUE reported
textfile 2 1 TRUE
4216 rs147319422 C A C A -0.3491 0.0613 0.01619 NA FALSE FALSE FALSE
GCST006061 0.0411 0.1359 537409 outcome TRUE reported 15509_2_NAGLU_NAG
NAGLU chr1 195503116 0.030358 1.325e-30 35339 TRUE reported
textfile 2 1 TRUE
4217 rs147677460 A G A G 0.3845 -0.0045 0.02249 NA FALSE FALSE FALSE
GCST006061 0.0305 0.8821 537409 outcome TRUE reported 15509_2_NAGLU_NAG
NAGLU chr17 43450493 0.023272 2.547e-61 35373 TRUE reported
textfile 2 1 TRUE
4218 rs148215732 C T C T 0.9546 -0.0287 0.01901 NA FALSE FALSE FALSE
GCST006061 0.0339 0.3964 537409 outcome TRUE reported 15509_2_NAGLU_NAG
NAGLU chr17 42703243 0.015949 1e-200 35373 TRUE reported textfile
2 1 TRUE
4219 rs150161290 T C T C 0.1689 0.0607 0.02297 NA FALSE FALSE FALSE
GCST006061 0.0537 0.2587 537409 outcome TRUE reported 15509_2_NAGLU_NAG
NAGLU chr1 185996591 0.024973 1.35e-11 35358 TRUE reported
textfile 2 1 TRUE
4220 rs16838346 A G A G 0.3613 0.0269 0.03339 NA FALSE FALSE FALSE
GCST006061 0.0242 0.2669 537409 outcome TRUE reported 15509_2_NAGLU_NAG
NAGLU chr1 195412835 0.021438 9.878e-64 35339 TRUE reported
textfile 2 1 TRUE
4221 rs191076788 T G T G -0.6897 -0.0119 0.01687 NA FALSE FALSE FALSE

GCST006061 0.0282 0.6719 537409 outcome TRUE reported 15509_2_NAGLU_NAG
NAGLU chr1 196946276 0.029706 3.055e-119 35338 TRUE reported
textfile 2 1 TRUE
4222 rs35449482 C A C A -0.7582 -0.0328 0.03212 NA FALSE FALSE FALSE
GCST006061 0.0288 0.2549 537409 outcome TRUE reported 15509_2_NAGLU_NAG
NAGLU chr1 196683150 0.021544 1e-200 35338 TRUE reported textfile
2 1 TRUE
4223 rs4597119 A G A G 0.0466 -9.00E-04 0.2178 NA FALSE FALSE FALSE
GCST006061 0.0086 0.9167 537409 outcome TRUE reported 15509_2_NAGLU_NAG
NAGLU chr12 101687672 0.008104 8.897e-09 35363 TRUE reported
textfile 2 1 TRUE
4224 rs4651228 G A G A -0.1679 0.0117 0.01804 NA FALSE FALSE FALSE
GCST006061 0.0475 0.8049 537409 outcome TRUE reported 15509_2_NAGLU_NAG
NAGLU chr1 184636095 0.029438 1.174e-08 35359 TRUE reported
textfile 2 1 TRUE
4225 rs56097721 T C T C 0.5522 -0.0212 0.01612 NA FALSE FALSE FALSE
GCST006061 0.0376 0.5738 537409 outcome TRUE reported 15509_2_NAGLU_NAG
NAGLU chr17 43145553 0.029007 8.415e-81 35374 TRUE reported
textfile 2 1 TRUE
4226 rs58947745 A G A G 1.1366 0.0182 0.01005 NA FALSE FALSE FALSE
GCST006061 0.0267 0.4949 537409 outcome TRUE reported 15509_2_NAGLU_NAG
NAGLU chr17 42305129 0.035823 1e-200 35383 TRUE reported textfile
2 1 TRUE
4227 rs62076601 T C T C 0.1714 0.0061 0.04256 NA FALSE FALSE FALSE
GCST006061 0.0187 0.7447 537409 outcome TRUE reported 15509_2_NAGLU_NAG
NAGLU chr17 39067156 0.017709 3.721e-22 35380 TRUE reported
textfile 2 1 TRUE
4228 rs74939104 A C A C 0.1491 -0.0155 0.08143 NA FALSE FALSE FALSE
GCST006061 0.0169 0.3591 537409 outcome TRUE reported 15509_2_NAGLU_NAG
NAGLU chr17 40774966 0.012955 1.189e-30 35380 TRUE reported
textfile 2 1 TRUE
4229 rs7537588 G A G A 0.7905 2e-04 0.05794 NA FALSE FALSE FALSE
GCST006061 0.0173 0.9922 537409 outcome TRUE reported 15509_2_NAGLU_NAG
NAGLU chr1 196993852 0.010262 1e-200 35338 TRUE reported textfile
2 1 TRUE
4230 rs76505423 G T G T 0.3077 0.0224 0.03066 NA FALSE FALSE FALSE
GCST006061 0.0296 0.4496 537409 outcome TRUE reported 15509_2_NAGLU_NAG
NAGLU chr1 197425817 0.021946 1.167e-44 35341 TRUE reported
textfile 2 1 TRUE
4231 rs77386381 C T C T 0.4048 0.0288 0.02537 NA FALSE FALSE FALSE
GCST006061 0.0215 0.1809 537409 outcome TRUE reported 15509_2_NAGLU_NAG
NAGLU chr17 42876827 0.021922 3.906e-76 35369 TRUE reported
textfile 2 1 TRUE
4232 rs78379279 C T C T 0.7712 -0.0014 0.03026 NA FALSE FALSE FALSE
GCST006061 0.0324 0.9667 537409 outcome TRUE reported 15509_2_NAGLU_NAG
NAGLU chr1 196246440 0.022149 1e-200 35337 TRUE reported textfile
2 1 TRUE
4233 rs12916255 A G A G 0.0665 -0.002 0.20556 NA FALSE FALSE FALSE
GCST006061 0.0087 0.8161 537409 outcome TRUE reported 5029_3_FAP_SEPR FAP
chr15 50897510 0.010146 5.601e-11 35364 TRUE reported textfile 2
1 TRUE
4234 rs188235382 A G A G 0.2226 0.051 0.01672 NA FALSE FALSE FALSE
GCST006061 0.0532 0.3376 537409 outcome TRUE reported 5029_3_FAP_SEPR FAP
chr2 161893728 0.032062 3.845e-12 35342 TRUE reported textfile 2
1 TRUE
4235 rs2668202 G A G A -0.0591 -0.0076 0.30991 NA FALSE FALSE FALSE
GCST006061 0.0074 0.3015 537409 outcome TRUE reported 5029_3_FAP_SEPR FAP
chr3 165772680 0.008779 1.678e-11 35339 TRUE reported textfile 2
1 TRUE
4236 rs506317 T C T C -0.0466 -0.0159 0.43587 NA FALSE FALSE FALSE
GCST006061 0.0075 0.03311 537409 outcome TRUE reported 5029_3_FAP_SEPR FAP
chr1 196748023 0.008199 1.318e-08 35339 TRUE reported textfile 2
1 TRUE
4237 rs62018183 G T G T -0.0994 0.0028 0.22103 NA FALSE FALSE FALSE
GCST006061 0.0091 0.7585 537409 outcome TRUE reported 5029_3_FAP_SEPR FAP
chr15 51009035 0.009847 5.817e-24 35364 TRUE reported textfile 2

1 TRUE
4238 rs76713183 C A C A -0.2099 -0.0061 0.03043 NA FALSE FALSE FALSE
GCST006061 0.0322 0.8496 537409 outcome TRUE reported 5029_3_FAP_SEPR FAP
chr2 162197789 0.024252 4.924e-18 35341 TRUE reported textfile 2
1 TRUE
4239 rs78722278 G A G A 0.2808 0.0701 0.0296 NA FALSE FALSE FALSE
GCST006061 0.0536 0.1909 537409 outcome TRUE reported 5029_3_FAP_SEPR FAP
chr2 162219117 0.024186 3.661e-31 35341 TRUE reported textfile 2
1 TRUE
4240 rs79900435 T C T C -0.1587 0.006 0.03107 NA FALSE FALSE FALSE
GCST006061 0.0278 0.8289 537409 outcome TRUE reported 5029_3_FAP_SEPR FAP
chr2 162404944 0.023721 2.229e-11 35342 TRUE reported textfile 2
1 TRUE
4241 rs10490468 G A G A -0.0709 -0.0142 0.30878 NA FALSE FALSE FALSE
GCST006061 0.0077 0.06595 537409 outcome TRUE reported 4258_15_PA2G4_PA2G4
PA2G4 chr2 53737369 0.008714 4.069e-16 35346 TRUE reported
textfile 2 1 TRUE
4242 rs10913644 A G A G -0.0802 -0.0022 0.2594 NA FALSE FALSE FALSE
GCST006061 0.0079 0.7825 537409 outcome TRUE reported 4258_15_PA2G4_PA2G4
PA2G4 chr1 171777813 0.009134 1.63e-18 35359 TRUE reported
textfile 2 1 TRUE
4243 rs1354034 C T C T 0.0689 0.0088 0.38119 NA FALSE FALSE FALSE
GCST006061 0.0073 0.2248 537409 outcome TRUE reported 4258_15_PA2G4_PA2G4
PA2G4 chr3 56815721 0.008286 9.131e-17 35356 TRUE reported
textfile 2 1 TRUE
4244 rs35979828 T C T C -0.0911 -0.0044 0.08648 NA FALSE FALSE FALSE
GCST006061 0.0156 0.7763 537409 outcome TRUE reported 4258_15_PA2G4_PA2G4
PA2G4 chr12 54292096 0.01453 3.62e-10 35375 TRUE reported textfile
2 1 TRUE
4245 rs4980320 C T C T 0.0483 0.0041 0.46986 NA FALSE FALSE FALSE
GCST006061 0.0076 0.5858 537409 outcome TRUE reported 4258_15_PA2G4_PA2G4
PA2G4 chr11 198986 0.008083 2.293e-09 35352 TRUE reported textfile
2 1 TRUE
4246 rs78909033 A G A G 0.1027 -0.0167 0.16064 NA FALSE FALSE FALSE
GCST006061 0.0117 0.1526 537409 outcome TRUE reported 4258_15_PA2G4_PA2G4
PA2G4 chr2 240571486 0.010978 8.322e-21 35270 TRUE reported
textfile 2 1 TRUE
4247 rs9494145 C T C T 0.0554 -0.0073 0.24334 NA FALSE FALSE FALSE
GCST006061 0.0083 0.3799 537409 outcome TRUE reported 4258_15_PA2G4_PA2G4
PA2G4 chr6 135111414 0.009393 3.676e-09 35372 TRUE reported
textfile 2 1 TRUE
4248 rs10490842 A C A C 0.1718 0.0158 0.02639 NA FALSE FALSE FALSE
GCST006061 0.0276 0.5673 537409 outcome TRUE reported 8958_51_CHL1_CHL1
CHL1 chr3 409982 0.02603 4.108e-11 35228 TRUE reported textfile 2
1 TRUE
4249 rs11129766 A G A G 0.4362 -0.0287 0.01316 NA FALSE FALSE FALSE
GCST006061 0.0274 0.2954 537409 outcome TRUE reported 8958_51_CHL1_CHL1
CHL1 chr3 393460 0.035978 7.869e-34 35229 TRUE reported textfile
2 1 TRUE
4250 rs12945886 G A G A 0.0719 0 0.24063 NA FALSE FALSE FALSE GCST006061
0.0094 0.9973 537409 outcome TRUE reported 8958_51_CHL1_CHL1 CHL1 chr17
7169695 0.009603 7.018e-14 35370 TRUE reported textfile 2 1 TRUE
4251 rs13077895 G A G A -0.2291 -0.0119 0.35544 NA FALSE FALSE FALSE
GCST006061 0.0075 0.1154 537409 outcome TRUE reported 8958_51_CHL1_CHL1
CHL1 chr3 51395 0.008612 6.138e-156 35349 TRUE reported textfile
2 1 TRUE
4252 rs17016909 C A C A 0.2345 -0.0089 0.01563 NA FALSE FALSE FALSE
GCST006061 0.0351 0.7996 537409 outcome TRUE reported 8958_51_CHL1_CHL1
CHL1 chr3 2578658 0.03389 4.534e-12 35283 TRUE reported textfile 2
1 TRUE
4253 rs2057417 G A G A -0.0492 0.0038 0.31795 NA FALSE FALSE FALSE
GCST006061 0.0078 0.6283 537409 outcome TRUE reported 8958_51_CHL1_CHL1
CHL1 chr13 49484731 0.008811 2.349e-08 35364 TRUE reported
textfile 2 1 TRUE
4254 rs2066985 T G T G -0.1371 0.0017 0.1001 NA FALSE FALSE FALSE
GCST006061 0.01 0.8649 537409 outcome TRUE reported 8958_51_CHL1_CHL1

CHL1 chr11 126381391 0.013867 4.74e-23 35376 TRUE reported
textfile 2 1 TRUE
4255 rs2454673 A G A G -0.1116 0.0081 0.49561 NA FALSE FALSE FALSE
GCST006061 0.0072 0.2653 537409 outcome TRUE reported 8958_51_CHL1_CHL1
CHL1 chr3 98902275 0.008275 1.867e-41 35331 TRUE reported
textfile 2 1 TRUE
4256 rs6442794 A G A G 0.0907 -0.0042 0.44395 NA FALSE FALSE FALSE
GCST006061 0.0073 0.5603 537409 outcome TRUE reported 8958_51_CHL1_CHL1
CHL1 chr3 362547 0.008345 1.616e-27 35226 TRUE reported textfile
2 1 TRUE
4257 rs7253490 A C A C -0.0517 -0.0077 0.32349 NA FALSE FALSE FALSE
GCST006061 0.0078 0.321 537409 outcome TRUE reported 8958_51_CHL1_CHL1
CHL1 chr19 22110904 0.008757 3.55e-09 35357 TRUE reported
textfile 2 1 TRUE
4258 rs10491534 C T C T 0.1252 -0.0041 0.09634 NA FALSE FALSE FALSE
GCST006061 0.0119 0.7272 537409 outcome TRUE reported 9796_4_CEL_CEL CEL
chr9 132894301 0.014539 7.226e-18 35373 TRUE reported textfile 2
1 TRUE
4259 rs113529953 T C T C 0.6875 0.0043 0.04005 NA FALSE FALSE FALSE
GCST006061 0.0213 0.842 537409 outcome TRUE reported 9796_4_CEL_CEL CEL
chr9 133028852 0.021307 1e-200 35372 TRUE reported textfile 2 1
TRUE
4260 rs117119759 A G A G 0.2993 -0.0106 0.03561 NA FALSE FALSE FALSE
GCST006061 0.0283 0.7084 537409 outcome TRUE reported 9796_4_CEL_CEL CEL
chr9 133345313 0.023337 1.189e-37 35371 TRUE reported textfile 2
1 TRUE
4261 rs148690761 G A G A 0.3483 -0.0846 0.0302 NA FALSE FALSE FALSE
GCST006061 0.0536 0.1142 537409 outcome TRUE reported 9796_4_CEL_CEL CEL
chr9 132553968 0.024909 1.977e-44 35372 TRUE reported textfile 2
1 TRUE
4262 rs3780420 C T C T 0.059 -0.0063 0.35496 NA FALSE FALSE FALSE
GCST006061 0.0075 0.4019 537409 outcome TRUE reported 9796_4_CEL_CEL CEL
chr9 97914884 0.008915 3.631e-11 35345 TRUE reported textfile 2
1 TRUE
4263 rs72767846 A G A G 0.2453 0.0214 0.01632 NA FALSE FALSE FALSE
GCST006061 0.0356 0.5476 537409 outcome TRUE reported 9796_4_CEL_CEL CEL
chr9 132572921 0.033962 5.097e-13 35372 TRUE reported textfile 2
1 TRUE
4264 rs79100721 A G A G 0.2476 -0.0022 0.03702 NA FALSE FALSE FALSE
GCST006061 0.0207 0.9147 537409 outcome TRUE reported 9796_4_CEL_CEL CEL
chr9 131534429 0.023006 5.18e-27 35372 TRUE reported textfile 2
1 TRUE
4265 rs886136 A G A G -0.0627 0.002 0.23636 NA FALSE FALSE FALSE
GCST006061 0.009 0.8244 537409 outcome TRUE reported 9796_4_CEL_CEL CEL
chr19 35173904 0.009714 1.086e-10 35366 TRUE reported textfile 2
1 TRUE
4266 rs1354034 C T C T 0.0578 0.0088 0.38119 NA FALSE FALSE FALSE
GCST006061 0.0073 0.2248 537409 outcome TRUE reported 13934_3_MCF2L_MCF2L
MCF2L chr3 56815721 0.008257 2.559e-12 35356 TRUE reported
textfile 2 1 TRUE
4267 rs1355538 G A G A -0.0525 -0.0076 0.39847 NA FALSE FALSE FALSE
GCST006061 0.0072 0.2923 537409 outcome TRUE reported 13934_3_MCF2L_MCF2L
MCF2L chr3 165787389 0.008186 1.421e-10 35338 TRUE reported
textfile 2 1 TRUE
4268 rs1963140 G A G A -0.0823 0.0156 0.07973 NA FALSE FALSE FALSE
GCST006061 0.0122 0.2023 537409 outcome TRUE reported 13934_3_MCF2L_MCF2L
MCF2L chr22 27289583 0.015075 4.781e-08 35372 TRUE reported
textfile 2 1 TRUE
4269 rs3011487 C T C T 0.2089 -0.0035 0.48488 NA FALSE FALSE FALSE
GCST006061 0.0074 0.6302 537409 outcome TRUE reported 13934_3_MCF2L_MCF2L
MCF2L chr13 112924107 0.008039 6.985e-149 34900 TRUE reported
textfile 2 1 TRUE
4270 rs1049296 T C T C -0.0601 -0.0158 0.16759 NA FALSE FALSE FALSE
GCST006061 0.0095 0.09597 537409 outcome TRUE reported 19568_17_IL15_IL_15
IL15 chr3 133775510 0.010633 1.585e-08 35359 TRUE reported
textfile 2 1 TRUE

4271 rs1354034 C T C T 0.0543 0.0088 0.38119 NA FALSE FALSE FALSE
GCST006061 0.0073 0.2248 537409 outcome TRUE reported 19568_17_IL15_IL_15
IL15 chr3 56815721 0.008215 3.847e-11 35356 TRUE reported
textfile 2 1 TRUE
4272 rs10494247 T C T C -0.0448 0.0259 0.44774 NA FALSE FALSE FALSE
GCST006061 0.0071 0.0002685 537409 outcome TRUE reported
3181_50_CTSS_Cathepsin_S CTSS chr1 147641588 0.008165 4.09e-08 35358
TRUE reported textfile 2 1 TRUE
4273 rs10896415 A G A G -0.0798 0.0011 0.37201 NA FALSE FALSE FALSE
GCST006061 0.0078 0.8924 537409 outcome TRUE reported
3181_50_CTSS_Cathepsin_S CTSS chr11 69071866 0.008217 2.677e-22 35366
TRUE reported textfile 2 1 TRUE
4274 rs12139264 A G A G -0.079 -0.0153 0.46408 NA FALSE FALSE FALSE
GCST006061 0.0072 0.03413 537409 outcome TRUE reported
3181_50_CTSS_Cathepsin_S CTSS chr1 151723428 0.008081 1.43e-22 35365
TRUE reported textfile 2 1 TRUE
4275 rs12291142 T C T C -0.0547 0.011 0.23989 NA FALSE FALSE FALSE
GCST006061 0.008 0.1668 537409 outcome TRUE reported
3181_50_CTSS_Cathepsin_S CTSS chr11 68966752 0.009279 3.753e-09 35366
TRUE reported textfile 2 1 TRUE
4276 rs12602829 G A G A -0.0838 0.0169 0.14444 NA FALSE FALSE FALSE
GCST006061 0.01 0.09062 537409 outcome TRUE reported
3181_50_CTSS_Cathepsin_S CTSS chr17 5300901 0.011422 2.189e-13 35379
TRUE reported textfile 2 1 TRUE
4277 rs1332327 T C T C 0.0613 -0.0107 0.32583 NA FALSE FALSE FALSE
GCST006061 0.008 0.1814 537409 outcome TRUE reported
3181_50_CTSS_Cathepsin_S CTSS chr10 89251924 0.008516 6.095e-13 35353
TRUE reported textfile 2 1 TRUE
4278 rs139020547 T C T C -0.1704 -0.0344 0.01939 NA FALSE FALSE FALSE
GCST006061 0.031 0.2674 537409 outcome TRUE reported
3181_50_CTSS_Cathepsin_S CTSS chr1 153262027 0.028689 2.857e-09 35361
TRUE reported textfile 2 1 TRUE
4279 rs4883201 G A G A 0.1703 -0.0182 0.12502 NA FALSE FALSE FALSE
GCST006061 0.0114 0.1094 537409 outcome TRUE reported
3181_50_CTSS_Cathepsin_S CTSS chr12 8929985 0.012037 1.914e-45 35387
TRUE reported textfile 2 1 TRUE
4280 rs4919333 G A G A -0.0886 -0.0109 0.07787 NA FALSE FALSE FALSE
GCST006061 0.0118 0.3563 537409 outcome TRUE reported
3181_50_CTSS_Cathepsin_S CTSS chr10 99490912 0.014886 2.651e-09 35368
TRUE reported textfile 2 1 TRUE
4281 rs6655975 G A G A -0.1864 -0.0032 0.39688 NA FALSE FALSE FALSE
GCST006061 0.0074 0.6675 537409 outcome TRUE reported
3181_50_CTSS_Cathepsin_S CTSS chr1 150569652 0.008239 2.442e-113 35357
TRUE reported textfile 2 1 TRUE
4282 rs6727677 G A G A 0.06 0.0087 0.20211 NA FALSE FALSE FALSE
GCST006061 0.0089 0.3237 537409 outcome TRUE reported
3181_50_CTSS_Cathepsin_S CTSS chr2 105318709 0.009953 1.659e-09 35363
TRUE reported textfile 2 1 TRUE
4283 rs67873985 C T C T 0.0482 0.0034 0.32619 NA FALSE FALSE FALSE
GCST006061 0.0076 0.6597 537409 outcome TRUE reported
3181_50_CTSS_Cathepsin_S CTSS chr17 82611964 0.008511 1.483e-08 35024
TRUE reported textfile 2 1 TRUE
4284 rs72692873 A G A G -0.5187 -0.0175 0.08211 NA FALSE FALSE FALSE
GCST006061 0.0151 0.2456 537409 outcome TRUE reported
3181_50_CTSS_Cathepsin_S CTSS chr1 150050850 0.014799 1e-200 35356
TRUE reported textfile 2 1 TRUE
4285 rs9457811 T C T C 0.0902 -0.0095 0.15183 NA FALSE FALSE FALSE
GCST006061 0.0102 0.3539 537409 outcome TRUE reported
3181_50_CTSS_Cathepsin_S CTSS chr6 160022829 0.011165 6.529e-16 35390
TRUE reported textfile 2 1 TRUE
4286 rs10494275 A G A G -0.2261 -0.0374 0.08129 NA FALSE FALSE FALSE
GCST006061 0.0141 0.008064 537409 outcome TRUE reported 17738_7_CRNN_CRNN
CRNN chr1 152419091 0.013958 5.126e-59 35363 TRUE reported
textfile 2 1 TRUE
4287 rs704 A G A G 0.0502 -0.0155 0.49189 NA FALSE FALSE FALSE GCST006061
0.0072 0.03144 537409 outcome TRUE reported 17738_7_CRNN_CRNN CRNN chr17

28367840 0.007714 7.645e-11 35360 TRUE reported textfile 2 1 TRUE
4288 rs10494305 T C T C 0.0865 6e-04 0.08671 NA FALSE FALSE FALSE
GCST006061 0.0143 0.9643 537409 outcome TRUE reported 11696_7_CRABP2_RABP2
CRABP2 chr1 156430268 0.014891 6.294e-09 35351 TRUE reported
textfile 2 1 TRUE
4289 rs4457570 G A G A 0.2855 -0.002 0.42511 NA FALSE FALSE FALSE
GCST006061 0.0077 0.7928 537409 outcome TRUE reported 11696_7_CRABP2_RABP2
CRABP2 chr1 156703170 0.008313 1e-200 35347 TRUE reported textfile
2 1 TRUE
4290 rs4795734 A G A G -0.0552 0.012 0.23628 NA FALSE FALSE FALSE
GCST006061 0.0084 0.1516 537409 outcome TRUE reported 11696_7_CRABP2_RABP2
CRABP2 chr17 33030407 0.009723 1.371e-08 35363 TRUE reported
textfile 2 1 TRUE
4291 rs6687516 A G A G 0.0545 0.0045 0.35758 NA FALSE FALSE FALSE
GCST006061 0.0074 0.5436 537409 outcome TRUE reported 11696_7_CRABP2_RABP2
CRABP2 chr1 157367728 0.008689 3.55e-10 35357 TRUE reported
textfile 2 1 TRUE
4292 rs10495440 T C T C 0.08 0.0129 0.19979 NA FALSE FALSE FALSE
GCST006061 0.0088 0.1411 537409 outcome TRUE reported 16785_45_DEFA5_HD_5
DEFA5 chr1 239112279 0.010442 1.835e-14 35371 TRUE reported
textfile 2 1 TRUE
4293 rs10799448 A G A G 0.0622 -0.0012 0.4786 NA FALSE FALSE FALSE
GCST006061 0.0071 0.8706 537409 outcome TRUE reported 16785_45_DEFA5_HD_5
DEFA5 chr1 227780406 0.008456 1.897e-13 35360 TRUE reported
textfile 2 1 TRUE
4294 rs1131853 C T C T 0.0618 0.0125 0.18841 NA FALSE FALSE FALSE
GCST006061 0.0089 0.1591 537409 outcome TRUE reported 16785_45_DEFA5_HD_5
DEFA5 chr4 1003307 0.010824 1.134e-08 35273 TRUE reported textfile
2 1 TRUE
4295 rs11603094 T C T C -0.0703 -0.0126 0.33769 NA FALSE FALSE FALSE
GCST006061 0.008 0.1162 537409 outcome TRUE reported 16785_45_DEFA5_HD_5
DEFA5 chr11 111436971 0.008906 2.94e-15 35361 TRUE reported
textfile 2 1 TRUE
4296 rs12419620 G T G T 0.0735 -0.0236 0.16221 NA FALSE FALSE FALSE
GCST006061 0.0095 0.01279 537409 outcome TRUE reported 16785_45_DEFA5_HD_5
DEFA5 chr11 2211323 0.01145 1.368e-10 35361 TRUE reported textfile 2
1 TRUE
4297 rs12599863 G A G A 0.0515 -0.0017 0.32864 NA FALSE FALSE FALSE
GCST006061 0.0079 0.8297 537409 outcome TRUE reported 16785_45_DEFA5_HD_5
DEFA5 chr16 75560034 0.00895 8.698e-09 35363 TRUE reported textfile
2 1 TRUE
4298 rs13029745 A G A G 0.0592 -0.0062 0.27729 NA FALSE FALSE FALSE
GCST006061 0.0081 0.4426 537409 outcome TRUE reported 16785_45_DEFA5_HD_5
DEFA5 chr2 190810487 0.009335 2.276e-10 35375 TRUE reported
textfile 2 1 TRUE
4299 rs16837903 A G A G 0.0694 -0.0037 0.14838 NA FALSE FALSE FALSE
GCST006061 0.0106 0.7264 537409 outcome TRUE reported 16785_45_DEFA5_HD_5
DEFA5 chr1 150579566 0.011911 5.664e-09 35358 TRUE reported
textfile 2 1 TRUE
4300 rs188963550 A C A C -0.2611 -0.0435 0.01121 NA FALSE FALSE FALSE
GCST006061 0.0395 0.2708 537409 outcome TRUE reported 16785_45_DEFA5_HD_5
DEFA5 chr8 7092535 0.041234 2.417e-10 35379 TRUE reported textfile
2 1 TRUE
4301 rs2522545 C T C T 0.05 -4.00E-04 0.40594 NA FALSE FALSE FALSE
GCST006061 0.0073 0.9615 537409 outcome TRUE reported 16785_45_DEFA5_HD_5
DEFA5 chr4 105457127 0.008574 5.492e-09 35353 TRUE reported
textfile 2 1 TRUE
4302 rs28702953 A C A C 0.0838 -0.0028 0.21853 NA FALSE FALSE FALSE
GCST006061 0.009 0.7541 537409 outcome TRUE reported 16785_45_DEFA5_HD_5
DEFA5 chr9 83178036 0.010187 1.938e-16 35366 TRUE reported
textfile 2 1 TRUE
4303 rs2881260 A G A G -0.0563 0.0053 0.21727 NA FALSE FALSE FALSE
GCST006061 0.0089 0.5512 537409 outcome TRUE reported 16785_45_DEFA5_HD_5
DEFA5 chr2 46118839 0.010243 3.876e-08 35359 TRUE reported
textfile 2 1 TRUE
4304 rs4800364 A G A G 0.1008 0.0021 0.12905 NA FALSE FALSE FALSE

GCST006061 0.011 0.8474 537409 outcome TRUE reported 16785_45_DEFA5_HD_5
DEFA5 chr18 22099180 0.012632 1.466e-15 35348 TRUE reported
textfile 2 1 TRUE
4305 rs485073 G A G A -0.1106 0.0044 0.37007 NA FALSE FALSE FALSE
GCST006061 0.0076 0.5645 537409 outcome TRUE reported 16785_45_DEFA5_HD_5
DEFA5 chr19 48703998 0.008705 5.542e-37 35377 TRUE reported
textfile 2 1 TRUE
4306 rs610891 G A G A -0.0531 -0.0025 0.46 NA FALSE FALSE FALSE
GCST006061 0.0071 0.7279 537409 outcome TRUE reported 16785_45_DEFA5_HD_5
DEFA5 chr8 108148774 0.008409 2.713e-10 35343 TRUE reported
textfile 2 1 TRUE
4307 rs635634 C T C T -0.1173 0.0285 0.12995 NA FALSE FALSE FALSE
GCST006061 0.0089 0.001351 537409 outcome TRUE reported 16785_45_DEFA5_HD_5
DEFA5 chr9 133279427 0.012474 5.276e-21 35371 TRUE reported
textfile 2 1 TRUE
4308 rs7207133 G A G A 0.0664 -0.0014 0.1609 NA FALSE FALSE FALSE
GCST006061 0.0085 0.871 537409 outcome TRUE reported 16785_45_DEFA5_HD_5
DEFA5 chr17 71912428 0.011371 5.235e-09 35371 TRUE reported
textfile 2 1 TRUE
4309 rs72856733 C T C T -0.0721 -2.00E-04 0.39278 NA FALSE FALSE FALSE
GCST006061 0.0079 0.9835 537409 outcome TRUE reported 16785_45_DEFA5_HD_5
DEFA5 chr17 65576442 0.008654 8.005e-17 35362 TRUE reported
textfile 2 1 TRUE
4310 rs7693632 C T C T -0.0806 -0.0151 0.1459 NA FALSE FALSE FALSE
GCST006061 0.0099 0.1271 537409 outcome TRUE reported 16785_45_DEFA5_HD_5
DEFA5 chr4 93852851 0.011961 1.602e-11 35337 TRUE reported
textfile 2 1 TRUE
4311 rs80158569 A G A G 0.0959 -0.0326 0.11267 NA FALSE FALSE FALSE
GCST006061 0.0151 0.03096 537409 outcome TRUE reported 16785_45_DEFA5_HD_5
DEFA5 chr14 99316600 0.013156 3.117e-13 35379 TRUE reported
textfile 2 1 TRUE
4312 rs1049564 A G A G -0.1732 -0.002 0.16518 NA FALSE FALSE FALSE
GCST006061 0.0091 0.8223 537409 outcome TRUE reported 15435_4_PNP_PNP PNP
chr14 20472447 0.011018 1.117e-55 35333 TRUE reported textfile 2
1 TRUE
4313 rs1760928 G A G A -0.0815 0.0078 0.23685 NA FALSE FALSE FALSE
GCST006061 0.0089 0.383 537409 outcome TRUE reported 15435_4_PNP_PNP PNP
chr14 20487536 0.009651 3.052e-17 35326 TRUE reported textfile 2
1 TRUE
4314 rs4541868 A C A C -0.0594 0.0211 0.21915 NA FALSE FALSE FALSE
GCST006061 0.0082 0.01029 537409 outcome TRUE reported 15435_4_PNP_PNP PNP
chr8 105578477 0.009854 1.658e-09 35364 TRUE reported textfile 2
1 TRUE
4315 rs1049709 C T C T -0.0868 -0.0146 0.10605 NA FALSE FALSE FALSE
GCST006061 0.0107 0.1708 537409 outcome TRUE reported 5735_54_C1GALT1C1_C1GLC
C1GALT1C1 chr6 31269077 0.013587 1.677e-10 35439 TRUE reported
textfile 2 1 TRUE
4316 rs1638216 A G A G 0.0481 -6.00E-04 0.45494 NA FALSE FALSE FALSE
GCST006061 0.0071 0.9316 537409 outcome TRUE reported 5735_54_C1GALT1C1_C1GLC
C1GALT1C1 chr7 7276470 0.008342 8.118e-09 35360 TRUE reported
textfile 2 1 TRUE
4317 rs601338 A G A G -0.0745 0.0056 0.39439 NA FALSE FALSE FALSE
GCST006061 0.0076 0.4559 537409 outcome TRUE reported 5735_54_C1GALT1C1_C1GLC
C1GALT1C1 chr19 48703417 0.008511 2.068e-18 35377 TRUE reported
textfile 2 1 TRUE
4318 rs635634 C T C T -0.3875 0.0285 0.12995 NA FALSE FALSE FALSE
GCST006061 0.0089 0.001351 537409 outcome TRUE reported
5735_54_C1GALT1C1_C1GLC C1GALT1C1 chr9 133279427 0.012251 1e-200 35371
TRUE reported textfile 2 1 TRUE
4319 rs9639031 C T C T 0.1665 -0.0056 0.39888 NA FALSE FALSE FALSE
GCST006061 0.0072 0.4365 537409 outcome TRUE reported 5735_54_C1GALT1C1_C1GLC
C1GALT1C1 chr7 7233524 0.008441 1.337e-86 35362 TRUE reported
textfile 2 1 TRUE
4320 rs10497223 A G A G -0.1745 -0.0217 0.02165 NA FALSE FALSE FALSE
GCST006061 0.0211 0.3023 537409 outcome TRUE reported 9278_9_EPYC_Epiphycan
EPYC chr2 161975013 0.028459 8.701e-10 35342 TRUE reported

textfile 2 1 TRUE
4321 rs10900074 A G A G 0.0636 -0.0015 0.48143 NA FALSE FALSE FALSE
GCST006061 0.0071 0.83 537409 outcome TRUE reported 9278_9_EPYC_Epiphycan
EPYC chr10 44575864 0.008262 1.388e-14 35322 TRUE reported
textfile 2 1 TRUE
4322 rs12773941 T C T C -0.0572 0.0067 0.19939 NA FALSE FALSE FALSE
GCST006061 0.009 0.4565 537409 outcome TRUE reported 9278_9_EPYC_Epiphycan
EPYC chr10 71802781 0.010327 3.048e-08 35375 TRUE reported
textfile 2 1 TRUE
4323 rs13015258 G T G T -0.0821 -0.0045 0.3882 NA FALSE FALSE FALSE
GCST006061 0.0075 0.5489 537409 outcome TRUE reported 9278_9_EPYC_Epiphycan
EPYC chr2 162074215 0.008493 4.167e-22 35341 TRUE reported
textfile 2 1 TRUE
4324 rs148242830 C T C T -0.1699 0.0619 0.02232 NA FALSE FALSE FALSE
GCST006061 0.0522 0.2364 537409 outcome TRUE reported 9278_9_EPYC_Epiphycan
EPYC chr2 160416685 0.028969 4.494e-09 35348 TRUE reported
textfile 2 1 TRUE
4325 rs2648312 G A G A 0.0466 -0.031 0.42662 NA FALSE FALSE FALSE
GCST006061 0.0073 2.371e-05 537409 outcome TRUE reported
9278_9_EPYC_Epiphycan EPYC chr3 12505648 0.008287 1.874e-08 35368
TRUE reported textfile 2 1 TRUE
4326 rs2802492 G A G A -0.0655 -0.0076 0.33737 NA FALSE FALSE FALSE
GCST006061 0.0074 0.3072 537409 outcome TRUE reported 9278_9_EPYC_Epiphycan
EPYC chr10 44056794 0.008705 5.286e-14 35323 TRUE reported
textfile 2 1 TRUE
4327 rs66478236 T C T C 0.0522 0.0027 0.26119 NA FALSE FALSE FALSE
GCST006061 0.0084 0.7505 537409 outcome TRUE reported 9278_9_EPYC_Epiphycan
EPYC chr4 174792076 0.009393 2.736e-08 35367 TRUE reported
textfile 2 1 TRUE
4328 rs7412 T C T C 0.0967 -0.0027 0.05941 NA FALSE FALSE FALSE GCST006061
0.0138 0.8459 537409 outcome TRUE reported 9278_9_EPYC_Epiphycan EPYC
chr19 44908822 0.01741 2.786e-08 35367 TRUE reported textfile 2 1
TRUE
4329 rs75232675 C T C T -0.1484 0.0547 0.02928 NA FALSE FALSE FALSE
GCST006061 0.0376 0.1459 537409 outcome TRUE reported 9278_9_EPYC_Epiphycan
EPYC chr2 162336608 0.024914 2.577e-09 35342 TRUE reported
textfile 2 1 TRUE
4330 rs77542162 G A G A 0.5805 0.0177 0.025 NA FALSE FALSE FALSE
GCST006061 0.0302 0.557 537409 outcome TRUE reported 9278_9_EPYC_Epiphycan
EPYC chr17 69085137 0.025835 8.187e-112 35371 TRUE reported
textfile 2 1 TRUE
4331 rs1049739 G A G A -0.0528 0.0112 0.43681 NA FALSE FALSE FALSE
GCST006061 0.0074 0.1281 537409 outcome TRUE reported 6572_10_LRRTM4_LRRT4
LRRTM4 chr19 45475117 0.008078 6.312e-11 35371 TRUE reported
textfile 2 1 TRUE
4332 rs1355538 G A G A -0.0636 -0.0076 0.39847 NA FALSE FALSE FALSE
GCST006061 0.0072 0.2923 537409 outcome TRUE reported 6572_10_LRRTM4_LRRT4
LRRTM4 chr3 165787389 0.008081 3.543e-15 35338 TRUE reported
textfile 2 1 TRUE
4333 rs62119261 C A C A 0.114 -0.0201 0.04025 NA FALSE FALSE FALSE
GCST006061 0.0198 0.3106 537409 outcome TRUE reported 6572_10_LRRTM4_LRRT4
LRRTM4 chr19 44618732 0.020618 3.216e-08 35371 TRUE reported
textfile 2 1 TRUE
4334 rs73045691 A G A G 0.3595 0.0102 0.26022 NA FALSE FALSE FALSE
GCST006061 0.0083 0.2192 537409 outcome TRUE reported 6572_10_LRRTM4_LRRT4
LRRTM4 chr19 44937272 0.007847 1e-200 35368 TRUE reported textfile
2 1 TRUE
4335 rs77660932 C T C T -0.0759 -0.0044 0.18053 NA FALSE FALSE FALSE
GCST006061 0.0102 0.6647 537409 outcome TRUE reported 6572_10_LRRTM4_LRRT4
LRRTM4 chr19 45091584 0.010495 4.761e-13 35371 TRUE reported
textfile 2 1 TRUE
4336 rs1049739 G A G A -0.0537 0.0112 0.43681 NA FALSE FALSE FALSE
GCST006061 0.0074 0.1281 537409 outcome TRUE reported 14021_81_MED4_MED4
MED4 chr19 45475117 0.008114 3.636e-11 35371 TRUE reported
textfile 2 1 TRUE
4337 rs6445035 A G A G 0.1141 0.0154 0.1904 NA FALSE FALSE FALSE

GCST006061 0.0089 0.08256 537409 outcome TRUE reported 14021_81_MED4_MED4 MED4 chr3 165762312 0.010135 2.106e-29 35339 TRUE reported textfile 2 1 TRUE

4338 rs73045691 A G A G 0.2856 0.0102 0.26022 NA FALSE FALSE FALSE GCST006061 0.0083 0.2192 537409 outcome TRUE reported 14021_81_MED4_MED4 MED4 chr19 44937272 0.009082 1e-200 35368 TRUE reported textfile 2 1 TRUE

4339 rs1049739 G A G A -0.1469 0.0112 0.43681 NA FALSE FALSE FALSE GCST006061 0.0074 0.1281 537409 outcome TRUE reported 16760_2_IL26_IL_26 IL26 chr19 45475117 0.008601 2.095e-65 35371 TRUE reported textfile 2 1 TRUE

4340 rs11134475 G A G A 0.0475 0.0109 0.35718 NA FALSE FALSE FALSE GCST006061 0.0075 0.1444 537409 outcome TRUE reported 16760_2_IL26_IL_26 IL26 chr5 156972939 0.00714 2.879e-11 35351 TRUE reported textfile 2 1 TRUE

4341 rs111696008 A G A G -0.137 -0.0259 0.02443 NA FALSE FALSE FALSE GCST006061 0.0401 0.519 537409 outcome TRUE reported 16760_2_IL26_IL_26 IL26 chr2 216622986 0.022816 1.919e-09 35359 TRUE reported textfile 2 1 TRUE

4342 rs148077724 G A G A 0.1618 -0.0095 0.02611 NA FALSE FALSE FALSE GCST006061 0.0214 0.6574 537409 outcome TRUE reported 16760_2_IL26_IL_26 IL26 chr3 165952844 0.021415 4.171e-14 35340 TRUE reported textfile 2 1 TRUE

4343 rs182320411 A G A G -0.278 -0.0817 0.01498 NA FALSE FALSE FALSE GCST006061 0.0532 0.124 537409 outcome TRUE reported 16760_2_IL26_IL_26 IL26 chr19 45317999 0.035439 4.346e-15 35372 TRUE reported textfile 2 1 TRUE

4344 rs2571068 T G T G -0.0884 -0.0049 0.42085 NA FALSE FALSE FALSE GCST006061 0.0075 0.5104 537409 outcome TRUE reported 16760_2_IL26_IL_26 IL26 chr19 44361300 0.008658 1.794e-24 35369 TRUE reported textfile 2 1 TRUE

4345 rs394353 G A G A -0.0796 0.0078 0.43076 NA FALSE FALSE FALSE GCST006061 0.0073 0.29 537409 outcome TRUE reported 16760_2_IL26_IL_26 IL26 chr19 44864216 0.008554 1.337e-20 35367 TRUE reported textfile 2 1 TRUE

4346 rs483465 G A G A 0.0442 -0.0204 0.24663 NA FALSE FALSE FALSE GCST006061 0.0083 0.01415 537409 outcome TRUE reported 16760_2_IL26_IL_26 IL26 chr3 136329135 0.008031 3.721e-08 35361 TRUE reported textfile 2 1 TRUE

4347 rs62119281 T G T G 0.2513 -0.0221 0.04304 NA FALSE FALSE FALSE GCST006061 0.0168 0.1897 537409 outcome TRUE reported 16760_2_IL26_IL_26 IL26 chr19 44640467 0.021045 7.25e-33 35372 TRUE reported textfile 2 1 TRUE

4348 rs704 A G A G 0.0455 -0.0155 0.49189 NA FALSE FALSE FALSE GCST006061 0.0072 0.03144 537409 outcome TRUE reported 16760_2_IL26_IL_26 IL26 chr17 28367840 0.006859 3.27e-11 35360 TRUE reported textfile 2 1 TRUE

4349 rs73045691 A G A G 1.0534 0.0102 0.26022 NA FALSE FALSE FALSE GCST006061 0.0083 0.2192 537409 outcome TRUE reported 16760_2_IL26_IL_26 IL26 chr19 44937272 0.005008 1e-200 35368 TRUE reported textfile 2 1 TRUE

4350 rs73169103 T C T C 0.1032 0.0178 0.03563 NA FALSE FALSE FALSE GCST006061 0.0249 0.4756 537409 outcome TRUE reported 16760_2_IL26_IL_26 IL26 chr3 167190675 0.018583 2.802e-08 35354 TRUE reported textfile 2 1 TRUE

4351 rs77660932 C T C T -0.2727 -0.0044 0.18053 NA FALSE FALSE FALSE GCST006061 0.0102 0.6647 537409 outcome TRUE reported 16760_2_IL26_IL_26 IL26 chr19 45091584 0.011122 9.483e-133 35371 TRUE reported textfile 2 1 TRUE

4352 rs78980904 C T C T -0.0448 0.0012 0.26023 NA FALSE FALSE FALSE GCST006061 0.0083 0.8846 537409 outcome TRUE reported 16760_2_IL26_IL_26 IL26 chr2 20921704 0.007847 1.137e-08 35370 TRUE reported textfile 2 1 TRUE

4353 rs8101216 C T C T 0.1052 -0.0029 0.10889 NA FALSE FALSE FALSE GCST006061 0.0117 0.8061 537409 outcome TRUE reported 16760_2_IL26_IL_26 IL26 chr19 46312040 0.013729 1.82e-14 35367 TRUE reported textfile 2 1 TRUE

4354 rs1049739 G A G A -0.1495 0.0112 0.43681 NA FALSE FALSE FALSE
GCST006061 0.0074 0.1281 537409 outcome TRUE reported 8971_9_NRXN1_NRX1A
NRXN1 chr19 45475117 0.008742 1.452e-65 35371 TRUE reported
textfile 2 1 TRUE
4355 rs111266377 T G T G 0.1222 0.037 0.02883 NA FALSE FALSE FALSE
GCST006061 0.0258 0.1519 537409 outcome TRUE reported 8971_9_NRXN1_NRX1A
NRXN1 chr3 167239870 0.019713 5.684e-10 35354 TRUE reported
textfile 2 1 TRUE
4356 rs111696008 A G A G -0.14 -0.0259 0.02443 NA FALSE FALSE FALSE
GCST006061 0.0401 0.519 537409 outcome TRUE reported 8971_9_NRXN1_NRX1A
NRXN1 chr2 216622986 0.021896 1.616e-10 35359 TRUE reported
textfile 2 1 TRUE
4357 rs117629091 A G A G -0.1996 -0.0115 0.03431 NA FALSE FALSE FALSE
GCST006061 0.025 0.645 537409 outcome TRUE reported 8971_9_NRXN1_NRX1A
NRXN1 chr19 46773949 0.02378 4.717e-17 35371 TRUE reported textfile
2 1 TRUE
4358 rs147033786 C A C A 0.1885 0.0651 0.01101 NA FALSE FALSE FALSE
GCST006061 0.05 0.1932 537409 outcome TRUE reported 8971_9_NRXN1_NRX1A
NRXN1 chr3 168506251 0.032243 5.026e-09 35354 TRUE reported
textfile 2 1 TRUE
4359 rs148077724 G A G A 0.1711 -0.0095 0.02611 NA FALSE FALSE FALSE
GCST006061 0.0214 0.6574 537409 outcome TRUE reported 8971_9_NRXN1_NRX1A
NRXN1 chr3 165952844 0.020563 8.735e-17 35340 TRUE reported
textfile 2 1 TRUE
4360 rs2678379 G A G A 0.0537 0.0099 0.23322 NA FALSE FALSE FALSE
GCST006061 0.0085 0.2464 537409 outcome TRUE reported 8971_9_NRXN1_NRX1A
NRXN1 chr2 21003688 0.007825 6.742e-12 35373 TRUE reported
textfile 2 1 TRUE
4361 rs394353 G A G A -0.0848 0.0078 0.43076 NA FALSE FALSE FALSE
GCST006061 0.0073 0.29 537409 outcome TRUE reported 8971_9_NRXN1_NRX1A
NRXN1 chr19 44864216 0.008692 1.738e-22 35367 TRUE reported
textfile 2 1 TRUE
4362 rs4704826 A C A C 0.0523 0.0109 0.35793 NA FALSE FALSE FALSE
GCST006061 0.0075 0.1446 537409 outcome TRUE reported 8971_9_NRXN1_NRX1A
NRXN1 chr5 156965071 0.006845 2.155e-14 35351 TRUE reported
textfile 2 1 TRUE
4363 rs62087820 T G T G 0.0478 0.0031 0.21738 NA FALSE FALSE FALSE
GCST006061 0.0088 0.7278 537409 outcome TRUE reported 8971_9_NRXN1_NRX1A
NRXN1 chr17 68902274 0.00805 2.885e-09 35372 TRUE reported textfile
2 1 TRUE
4364 rs62119281 T G T G 0.267 -0.0221 0.04304 NA FALSE FALSE FALSE
GCST006061 0.0168 0.1897 537409 outcome TRUE reported 8971_9_NRXN1_NRX1A
NRXN1 chr19 44640467 0.021387 9.088e-36 35372 TRUE reported
textfile 2 1 TRUE
4365 rs73045691 A G A G 1.1219 0.0102 0.26022 NA FALSE FALSE FALSE
GCST006061 0.0083 0.2192 537409 outcome TRUE reported 8971_9_NRXN1_NRX1A
NRXN1 chr19 44937272 0.004661 1e-200 35368 TRUE reported textfile
2 1 TRUE
4366 rs73051460 T G T G -0.1103 -0.0256 0.06429 NA FALSE FALSE FALSE
GCST006061 0.019 0.178 537409 outcome TRUE reported 8971_9_NRXN1_NRX1A
NRXN1 chr19 42064923 0.017778 5.498e-10 35370 TRUE reported
textfile 2 1 TRUE
4367 rs77660932 C T C T -0.2911 -0.0044 0.18053 NA FALSE FALSE FALSE
GCST006061 0.0102 0.6647 537409 outcome TRUE reported 8971_9_NRXN1_NRX1A
NRXN1 chr19 45091584 0.011297 2.043e-146 35371 TRUE reported
textfile 2 1 TRUE
4368 rs8101216 C T C T 0.1132 -0.0029 0.10889 NA FALSE FALSE FALSE
GCST006061 0.0117 0.8061 537409 outcome TRUE reported 8971_9_NRXN1_NRX1A
NRXN1 chr19 46312040 0.01395 4.878e-16 35367 TRUE reported textfile
2 1 TRUE
4369 rs1049739 G A G A -0.1456 0.0112 0.43681 NA FALSE FALSE FALSE
GCST006061 0.0074 0.1281 537409 outcome TRUE reported 12384_92_COPS7B_CSN7B
COPS7B chr19 45475117 0.008508 1.173e-65 35362 TRUE reported
textfile 2 1 TRUE
4370 rs111696008 A G A G -0.1935 -0.0259 0.02443 NA FALSE FALSE FALSE
GCST006061 0.0401 0.519 537409 outcome TRUE reported 12384_92_COPS7B_CSN7B

COPS7B chr2 216622986 0.023306 1.02e-16 35350 TRUE reported
textfile 2 1 TRUE
4371 rs144202860 A G A G -0.1947 -0.0629 0.01274 NA FALSE FALSE FALSE
GCST006061 0.0538 0.2423 537409 outcome TRUE reported 12384_92_COPS7B_CSN7B
COPS7B chr2 216625740 0.031226 4.515e-10 35350 TRUE reported
textfile 2 1 TRUE
4372 rs148077724 G A G A 0.1775 -0.0095 0.02611 NA FALSE FALSE FALSE
GCST006061 0.0214 0.6574 537409 outcome TRUE reported 12384_92_COPS7B_CSN7B
COPS7B chr3 165952844 0.021889 5.095e-16 35331 TRUE reported
textfile 2 1 TRUE
4373 rs182320411 A G A G -0.2487 -0.0817 0.01498 NA FALSE FALSE FALSE
GCST006061 0.0532 0.124 537409 outcome TRUE reported 12384_92_COPS7B_CSN7B
COPS7B chr19 45317999 0.035058 1.303e-12 35363 TRUE reported
textfile 2 1 TRUE
4374 rs2571068 T G T G -0.0772 -0.0049 0.42085 NA FALSE FALSE FALSE
GCST006061 0.0075 0.5104 537409 outcome TRUE reported 12384_92_COPS7B_CSN7B
COPS7B chr19 44361300 0.008564 1.985e-19 35360 TRUE reported
textfile 2 1 TRUE
4375 rs394353 G A G A -0.0714 0.0078 0.43076 NA FALSE FALSE FALSE
GCST006061 0.0073 0.29 537409 outcome TRUE reported 12384_92_COPS7B_CSN7B
COPS7B chr19 44864216 0.008457 3.1e-17 35358 TRUE reported textfile
2 1 TRUE
4376 rs4704826 A C A C 0.0461 0.0109 0.35793 NA FALSE FALSE FALSE
GCST006061 0.0075 0.1446 537409 outcome TRUE reported 12384_92_COPS7B_CSN7B
COPS7B chr5 156965071 0.007268 2.256e-10 35342 TRUE reported
textfile 2 1 TRUE
4377 rs62119281 T G T G 0.2204 -0.0221 0.04304 NA FALSE FALSE FALSE
GCST006061 0.0168 0.1897 537409 outcome TRUE reported 12384_92_COPS7B_CSN7B
COPS7B chr19 44640467 0.020831 3.674e-26 35363 TRUE reported
textfile 2 1 TRUE
4378 rs62290472 A G A G 0.0728 0.011 0.08266 NA FALSE FALSE FALSE
GCST006061 0.0106 0.3013 537409 outcome TRUE reported 12384_92_COPS7B_CSN7B
COPS7B chr3 162193597 0.012887 1.613e-08 35331 TRUE reported
textfile 2 1 TRUE
4379 rs73045691 A G A G 0.9955 0.0102 0.26022 NA FALSE FALSE FALSE
GCST006061 0.0083 0.2192 537409 outcome TRUE reported 12384_92_COPS7B_CSN7B
COPS7B chr19 44937272 0.005177 1e-200 35359 TRUE reported textfile
2 1 TRUE
4380 rs73169103 T C T C 0.1307 0.0178 0.03563 NA FALSE FALSE FALSE
GCST006061 0.0249 0.4756 537409 outcome TRUE reported 12384_92_COPS7B_CSN7B
COPS7B chr3 167190675 0.018985 5.807e-12 35345 TRUE reported
textfile 2 1 TRUE
4381 rs77660932 C T C T -0.2708 -0.0044 0.18053 NA FALSE FALSE FALSE
GCST006061 0.0102 0.6647 537409 outcome TRUE reported 12384_92_COPS7B_CSN7B
COPS7B chr19 45091584 0.011004 9.788e-134 35362 TRUE reported
textfile 2 1 TRUE
4382 rs78980904 C T C T -0.0482 0.0012 0.26023 NA FALSE FALSE FALSE
GCST006061 0.0083 0.8846 537409 outcome TRUE reported 12384_92_COPS7B_CSN7B
COPS7B chr2 20921704 0.00803 1.941e-09 35361 TRUE reported textfile
2 1 TRUE
4383 rs887829 T C T C 0.0451 0.0019 0.31955 NA FALSE FALSE FALSE
GCST006061 0.0077 0.8072 537409 outcome TRUE reported 12384_92_COPS7B_CSN7B
COPS7B chr2 233759924 0.007539 2.205e-09 35363 TRUE reported
textfile 2 1 TRUE
4384 rs114264570 A G A G -0.3918 -0.0052 0.01344 NA FALSE FALSE FALSE
GCST006061 0.0579 0.9282 537409 outcome TRUE reported
19154_41_SERPINE2_Protease_nexin_I SERPINE2 chr2 225116399 0.037636
2.227e-25 35367 TRUE reported textfile 2 1 TRUE
4385 rs4631576 T C T C 0.0691 -0.0052 0.35116 NA FALSE FALSE FALSE
GCST006061 0.0075 0.4837 537409 outcome TRUE reported
19154_41_SERPINE2_Protease_nexin_I SERPINE2 chr9 97942928 0.008535
5.676e-16 35345 TRUE reported textfile 2 1 TRUE
4386 rs4674836 A G A G 0.3356 -0.0025 0.34557 NA FALSE FALSE FALSE
GCST006061 0.0074 0.7339 537409 outcome TRUE reported
19154_41_SERPINE2_Protease_nexin_I SERPINE2 chr2 223990794 0.006534 1e-200
35366 TRUE reported textfile 2 1 TRUE

4387 rs6715495 A G A G 0.087 -0.0027 0.20316 NA FALSE FALSE FALSE
GCST006061 0.0095 0.7784 537409 outcome TRUE reported
19154_41_SERPINE2_Protease_nexin_I SERPINE2 chr2 223367385 0.010636
2.835e-16 35366 TRUE reported textfile 2 1 TRUE
4388 rs7172696 G A G A -0.1202 0.0115 0.34251 NA FALSE FALSE FALSE
GCST006061 0.0077 0.1371 537409 outcome TRUE reported
19154_41_SERPINE2_Protease_nexin_I SERPINE2 chr15 101451106 0.008572
1.139e-44 35308 TRUE reported textfile 2 1 TRUE
4389 rs7561846 G A G A -0.067 -0.0097 0.2764 NA FALSE FALSE FALSE
GCST006061 0.01 0.3305 537409 outcome TRUE reported
19154_41_SERPINE2_Protease_nexin_I SERPINE2 chr2 221996715 0.009613
3.178e-12 35364 TRUE reported textfile 2 1 TRUE
4390 rs10499638 C A C A -0.0505 -0.0118 0.25731 NA FALSE FALSE FALSE
GCST006061 0.0086 0.1692 537409 outcome TRUE reported 5364_7_SET_SET SET
chr7 46127333 0.009234 4.534e-08 35347 TRUE reported textfile 2
1 TRUE
4391 rs2667684 G A G A -0.0445 -0.0093 0.44815 NA FALSE FALSE FALSE
GCST006061 0.0074 0.2091 537409 outcome TRUE reported 5364_7_SET_SET SET
chr16 1600945 0.008063 3.405e-08 35319 TRUE reported textfile 2 1
TRUE
4392 rs10499842 T C T C 0.3399 -0.0037 0.18383 NA FALSE FALSE FALSE
GCST006061 0.0091 0.6807 537409 outcome TRUE reported 14066_49_MAGI2_MAGI2
MAGI2 chr7 79341720 0.01014 1e-200 35348 TRUE reported textfile 2
1 TRUE
4393 rs111348213 A G A G 0.1772 -0.0037 0.02319 NA FALSE FALSE FALSE
GCST006061 0.0218 0.8649 537409 outcome TRUE reported 14066_49_MAGI2_MAGI2
MAGI2 chr7 80110190 0.026109 1.146e-11 35347 TRUE reported
textfile 2 1 TRUE
4394 rs117656916 C T C T 0.3605 -0.0296 0.0162 NA FALSE FALSE FALSE
GCST006061 0.0415 0.4753 537409 outcome TRUE reported 14066_49_MAGI2_MAGI2
MAGI2 chr7 79644503 0.031579 3.492e-30 35347 TRUE reported
textfile 2 1 TRUE
4395 rs140903980 T C T C 0.4712 0.0469 0.01243 NA FALSE FALSE FALSE
GCST006061 0.0449 0.296 537409 outcome TRUE reported 14066_49_MAGI2_MAGI2
MAGI2 chr7 81964906 0.036396 2.457e-38 35349 TRUE reported
textfile 2 1 TRUE
4396 rs147184500 T C T C 0.3837 -0.0491 0.01134 NA FALSE FALSE FALSE
GCST006061 0.0678 0.4685 537409 outcome TRUE reported 14066_49_MAGI2_MAGI2
MAGI2 chr7 81029213 0.038028 6.116e-24 35346 TRUE reported
textfile 2 1 TRUE
4397 rs2667740 C T C T -0.2548 0.0016 0.03167 NA FALSE FALSE FALSE
GCST006061 0.0219 0.941 537409 outcome TRUE reported 14066_49_MAGI2_MAGI2
MAGI2 chr7 80251543 0.023194 4.477e-28 35348 TRUE reported
textfile 2 1 TRUE
4398 rs62460682 G T G T 0.1761 0.0217 0.02192 NA FALSE FALSE FALSE
GCST006061 0.0344 0.5273 537409 outcome TRUE reported 14066_49_MAGI2_MAGI2
MAGI2 chr7 80704029 0.02743 1.363e-10 35342 TRUE reported textfile
2 1 TRUE
4399 rs77494173 C T C T 0.2621 -0.0175 0.02215 NA FALSE FALSE FALSE
GCST006061 0.0254 0.4901 537409 outcome TRUE reported 14066_49_MAGI2_MAGI2
MAGI2 chr7 82174854 0.027516 1.646e-21 35351 TRUE reported
textfile 2 1 TRUE
4400 rs78438715 G A G A 0.644 0.0388 0.01406 NA FALSE FALSE FALSE
GCST006061 0.0498 0.4362 537409 outcome TRUE reported 14066_49_MAGI2_MAGI2
MAGI2 chr7 79820586 0.033565 4.782e-82 35347 TRUE reported
textfile 2 1 TRUE
4401 rs10500661 C T C T 0.0544 -0.0016 0.22999 NA FALSE FALSE FALSE
GCST006061 0.0089 0.8585 537409 outcome TRUE reported 11102_22_REG4_REG4
REG4 chr11 6252514 0.009844 3.276e-08 35369 TRUE reported textfile
2 1 TRUE
4402 rs12125319 C T C T 0.1599 -7.00E-04 0.02414 NA FALSE FALSE FALSE
GCST006061 0.0304 0.9806 537409 outcome TRUE reported 11102_22_REG4_REG4
REG4 chr1 119108785 0.027012 3.228e-09 35359 TRUE reported
textfile 2 1 TRUE
4403 rs3217810 T C T C 0.0823 -0.0105 0.13867 NA FALSE FALSE FALSE
GCST006061 0.0123 0.3945 537409 outcome TRUE reported 11102_22_REG4_REG4

REG4 chr12 4279105 0.011913 4.908e-12 35367 TRUE reported textfile
2 1 TRUE
4404 rs72697218 T C T C -0.2246 -1.00E-04 0.02416 NA FALSE FALSE FALSE
GCST006061 0.0244 0.9966 537409 outcome TRUE reported 11102_22_REG4_REG4
REG4 chr1 119853675 0.02673 4.37e-17 35352 TRUE reported textfile
2 1 TRUE
4405 rs731541 C A C A 0.0513 0.0018 0.3283 NA FALSE FALSE FALSE
GCST006061 0.0082 0.8281 537409 outcome TRUE reported 11102_22_REG4_REG4
REG4 chr17 9895845 0.00886 7.026e-09 35363 TRUE reported textfile 2
1 TRUE
4406 rs7480143 T C T C 0.0953 -0.0224 0.11386 NA FALSE FALSE FALSE
GCST006061 0.0108 0.03888 537409 outcome TRUE reported 11102_22_REG4_REG4
REG4 chr11 2203793 0.0131 3.47e-13 35352 TRUE reported textfile 2
1 TRUE
4407 rs11041116 A G A G -0.0682 -0.0142 0.31114 NA FALSE FALSE FALSE
GCST006061 0.0078 0.06706 537409 outcome TRUE reported 19143_38_CYB5R2_NB5R2
CYB5R2 chr11 6962867 0.008997 3.44e-14 35380 TRUE reported textfile
2 1 TRUE
4408 rs12787991 G T G T 0.1697 0.0448 0.0233 NA FALSE FALSE FALSE
GCST006061 0.0374 0.2311 537409 outcome TRUE reported 19143_38_CYB5R2_NB5R2
CYB5R2 chr11 8002455 0.02766 8.51e-10 35374 TRUE reported textfile 2
1 TRUE
4409 rs7122082 A G A G -0.1345 0.0011 0.45782 NA FALSE FALSE FALSE
GCST006061 0.0073 0.8852 537409 outcome TRUE reported 19143_38_CYB5R2_NB5R2
CYB5R2 chr11 7556593 0.008407 1.298e-57 35375 TRUE reported textfile
2 1 TRUE
4410 rs75667685 A G A G 0.1178 -0.0057 0.0402 NA FALSE FALSE FALSE
GCST006061 0.0174 0.742 537409 outcome TRUE reported 19143_38_CYB5R2_NB5R2
CYB5R2 chr11 7688502 0.021007 2.052e-08 35374 TRUE reported textfile
2 1 TRUE
4411 rs10501089 T C T C 0.1625 -0.0077 0.07064 NA FALSE FALSE FALSE
GCST006061 0.0161 0.6293 537409 outcome TRUE reported 14047_78_BDNF_BDNF
BDNF chr11 27767312 0.016105 6.102e-24 35360 TRUE reported
textfile 2 1 TRUE
4412 rs1071960 C T C T -0.0518 0.0045 0.29144 NA FALSE FALSE FALSE
GCST006061 0.0081 0.5763 537409 outcome TRUE reported 14047_78_BDNF_BDNF
BDNF chr22 42953626 0.009041 1.008e-08 35374 TRUE reported
textfile 2 1 TRUE
4413 rs10822155 A C A C 0.0865 -0.0472 0.43479 NA FALSE FALSE FALSE
GCST006061 0.0072 4.622e-11 537409 outcome TRUE reported 14047_78_BDNF_BDNF
BDNF chr10 63311455 0.008281 1.536e-25 35351 TRUE reported
textfile 2 1 TRUE
4414 rs12468189 G A G A 0.0483 -0.0052 0.40103 NA FALSE FALSE FALSE
GCST006061 0.0074 0.4799 537409 outcome TRUE reported 14047_78_BDNF_BDNF
BDNF chr2 31255954 0.008427 9.948e-09 35369 TRUE reported
textfile 2 1 TRUE
4415 rs1354034 C T C T -0.0521 0.0088 0.38119 NA FALSE FALSE FALSE
GCST006061 0.0073 0.2248 537409 outcome TRUE reported 14047_78_BDNF_BDNF
BDNF chr3 56815721 0.008467 7.589e-10 35356 TRUE reported
textfile 2 1 TRUE
4416 rs5008180 G A G A 0.0475 -0.0079 0.34313 NA FALSE FALSE FALSE
GCST006061 0.0078 0.3123 537409 outcome TRUE reported 14047_78_BDNF_BDNF
BDNF chr6 163443299 0.008692 4.629e-08 35372 TRUE reported
textfile 2 1 TRUE
4417 rs775824 C T C T 0.0704 0.0102 0.16338 NA FALSE FALSE FALSE
GCST006061 0.0101 0.3143 537409 outcome TRUE reported 14047_78_BDNF_BDNF
BDNF chr19 55050202 0.011092 2.196e-10 35381 TRUE reported
textfile 2 1 TRUE
4418 rs111696008 A G A G 0.518 -0.0259 0.02443 NA FALSE FALSE FALSE
GCST006061 0.0401 0.519 537409 outcome TRUE reported
15514_26_BCHE_Pseudocholinesterase BCHE chr2 216622986 0.026392 9.04e-86
35359 TRUE reported textfile 2 1 TRUE
4419 rs114893996 C T C T -0.1557 -0.0323 0.021 NA FALSE FALSE FALSE
GCST006061 0.0295 0.2728 537409 outcome TRUE reported
15514_26_BCHE_Pseudocholinesterase BCHE chr3 169673288 0.02829 3.719e-08
35359 TRUE reported textfile 2 1 TRUE

4420 rs11924273 T G T G -0.0704 0.0096 0.3688 NA FALSE FALSE FALSE
GCST006061 0.0072 0.1806 537409 outcome TRUE reported
15514_26_BCHE_Pseudocholinesterase BCHE chr3 166322984 0.008405 5.491e-17
35354 TRUE reported textfile 2 1 TRUE
4421 rs11968875 T C T C 0.1445 1e-04 0.04765 NA FALSE FALSE FALSE
GCST006061 0.0176 0.9954 537409 outcome TRUE reported
15514_26_BCHE_Pseudocholinesterase BCHE chr6 105402761 0.018762 1.344e-14
35353 TRUE reported textfile 2 1 TRUE
4422 rs13244614 A C A C -0.0617 -0.0088 0.30553 NA FALSE FALSE FALSE
GCST006061 0.0081 0.2729 537409 outcome TRUE reported
15514_26_BCHE_Pseudocholinesterase BCHE chr7 73559524 0.008557 5.587e-13
35354 TRUE reported textfile 2 1 TRUE
4423 rs144202860 A G A G 0.5204 -0.0629 0.01274 NA FALSE FALSE FALSE
GCST006061 0.0538 0.2423 537409 outcome TRUE reported
15514_26_BCHE_Pseudocholinesterase BCHE chr2 216625740 0.035413 6.927e-49
35359 TRUE reported textfile 2 1 TRUE
4424 rs147033786 C A C A -0.338 0.0651 0.01101 NA FALSE FALSE FALSE
GCST006061 0.05 0.1932 537409 outcome TRUE reported
15514_26_BCHE_Pseudocholinesterase BCHE chr3 168506251 0.039539 1.247e-17
35354 TRUE reported textfile 2 1 TRUE
4425 rs174564 G A G A 0.0489 0.0107 0.39357 NA FALSE FALSE FALSE
GCST006061 0.0075 0.1516 537409 outcome TRUE reported
15514_26_BCHE_Pseudocholinesterase BCHE chr11 61820833 0.008072 1.377e-09
35367 TRUE reported textfile 2 1 TRUE
4426 rs3814365 C T C T 0.1234 -0.0075 0.42924 NA FALSE FALSE FALSE
GCST006061 0.0073 0.3034 537409 outcome TRUE reported
15514_26_BCHE_Pseudocholinesterase BCHE chr2 203440370 0.008062 6.875e-53
35365 TRUE reported textfile 2 1 TRUE
4427 rs4240624 A G A G 0.111 -0.016 0.07464 NA FALSE FALSE FALSE
GCST006061 0.0128 0.2119 537409 outcome TRUE reported
15514_26_BCHE_Pseudocholinesterase BCHE chr8 9326721 0.015017 1.451e-13
35385 TRUE reported textfile 2 1 TRUE
4428 rs6435932 C T C T -0.0477 0.002 0.37142 NA FALSE FALSE FALSE
GCST006061 0.0072 0.7781 537409 outcome TRUE reported
15514_26_BCHE_Pseudocholinesterase BCHE chr2 216041307 0.008313 9.576e-09
35358 TRUE reported textfile 2 1 TRUE
4429 rs687339 T C T C -0.0611 -0.0273 0.22733 NA FALSE FALSE FALSE
GCST006061 0.0085 0.001387 537409 outcome TRUE reported
15514_26_BCHE_Pseudocholinesterase BCHE chr3 136213517 0.009744 3.6e-10
35361 TRUE reported textfile 2 1 TRUE
4430 rs77230198 A C A C -0.3255 0.0828 0.02175 NA FALSE FALSE FALSE
GCST006061 0.0283 0.003378 537409 outcome TRUE reported
15514_26_BCHE_Pseudocholinesterase BCHE chr3 166581568 0.028187 7.577e-31
35354 TRUE reported textfile 2 1 TRUE
4431 rs780093 C T C T -0.0517 -0.0074 0.34157 NA FALSE FALSE FALSE
GCST006061 0.0073 0.3116 537409 outcome TRUE reported
15514_26_BCHE_Pseudocholinesterase BCHE chr2 27519736 0.00843 8.624e-10
35373 TRUE reported textfile 2 1 TRUE
4432 rs887829 T C T C -0.0722 0.0019 0.31955 NA FALSE FALSE FALSE
GCST006061 0.0077 0.8072 537409 outcome TRUE reported
15514_26_BCHE_Pseudocholinesterase BCHE chr2 233759924 0.008568 3.546e-17
35372 TRUE reported textfile 2 1 TRUE
4433 rs1105880 G A G A 0.055 0.0045 0.33722 NA FALSE FALSE FALSE
GCST006061 0.0075 0.5471 537409 outcome TRUE reported 18380_78_ALB_Albumin
ALB chr2 233693319 0.008468 8.287e-11 35374 TRUE reported textfile
2 1 TRUE
4434 rs111696008 A G A G -0.345 -0.0259 0.02443 NA FALSE FALSE FALSE
GCST006061 0.0401 0.519 537409 outcome TRUE reported 18380_78_ALB_Albumin
ALB chr2 216622986 0.026501 9.645e-39 35359 TRUE reported textfile
2 1 TRUE
4435 rs12026141 T C T C -0.0773 0.0094 0.31246 NA FALSE FALSE FALSE
GCST006061 0.0076 0.2137 537409 outcome TRUE reported 18380_78_ALB_Albumin
ALB chr1 201980465 0.008652 4.102e-19 35357 TRUE reported textfile
2 1 TRUE
4436 rs13095777 A G A G -0.0665 -0.008 0.4371 NA FALSE FALSE FALSE
GCST006061 0.0071 0.2622 537409 outcome TRUE reported 18380_78_ALB_Albumin

ALB chr3 167270520 0.008114 2.482e-16 35354 TRUE reported textfile
2 1 TRUE
4437 rs144202860 A G A G -0.3665 -0.0629 0.01274 NA FALSE FALSE FALSE
GCST006061 0.0538 0.2423 537409 outcome TRUE reported 18380_78_ALB_Albumin
ALB chr2 216625740 0.035515 5.744e-25 35359 TRUE reported textfile
2 1 TRUE
4438 rs147033786 C A C A 0.3807 0.0651 0.01101 NA FALSE FALSE FALSE
GCST006061 0.05 0.1932 537409 outcome TRUE reported 18380_78_ALB_Albumin
ALB chr3 168506251 0.03924 2.964e-22 35354 TRUE reported textfile 2
1 TRUE
4439 rs7603427 T C T C -0.0807 -0.0055 0.45017 NA FALSE FALSE FALSE
GCST006061 0.0073 0.4447 537409 outcome TRUE reported 18380_78_ALB_Albumin
ALB chr2 203452830 0.008051 1.2e-23 35365 TRUE reported textfile 2
1 TRUE
4440 rs79330559 T C T C 0.2526 0.0142 0.01152 NA FALSE FALSE FALSE
GCST006061 0.0409 0.7273 537409 outcome TRUE reported 18380_78_ALB_Albumin
ALB chr3 165020966 0.037702 2.086e-11 35343 TRUE reported textfile
2 1 TRUE
4441 rs9872765 A G A G -0.0733 0.0113 0.15934 NA FALSE FALSE FALSE
GCST006061 0.0112 0.3148 537409 outcome TRUE reported 18380_78_ALB_Albumin
ALB chr3 163516410 0.010992 2.583e-11 35341 TRUE reported textfile
2 1 TRUE
4442 rs1050529 T C T C -0.058 -0.017 0.28239 NA FALSE FALSE FALSE
GCST006061 0.0108 0.1131 537409 outcome TRUE reported 5636_10_MFAP4_MFAP4
MFAP4 chr6 31356838 0.009242 3.481e-10 35440 TRUE reported
textfile 2 1 TRUE
4443 rs114208897 A G A G 0.2184 -0.0278 0.01211 NA FALSE FALSE FALSE
GCST006061 0.0439 0.5264 537409 outcome TRUE reported 5636_10_MFAP4_MFAP4
MFAP4 chr5 179511505 0.039494 3.204e-08 35338 TRUE reported
textfile 2 1 TRUE
4444 rs11658402 T G T G -0.1754 0.0099 0.02227 NA FALSE FALSE FALSE
GCST006061 0.0263 0.707 537409 outcome TRUE reported 5636_10_MFAP4_MFAP4
MFAP4 chr17 18943726 0.028353 6.16e-10 35357 TRUE reported
textfile 2 1 TRUE
4445 rs117731563 C T C T -0.1695 -0.0042 0.03117 NA FALSE FALSE FALSE
GCST006061 0.0285 0.883 537409 outcome TRUE reported 5636_10_MFAP4_MFAP4
MFAP4 chr17 20848092 0.024469 4.296e-12 35355 TRUE reported
textfile 2 1 TRUE
4446 rs12757307 A G A G 0.0889 0.0053 0.09474 NA FALSE FALSE FALSE
GCST006061 0.0124 0.668 537409 outcome TRUE reported 5636_10_MFAP4_MFAP4
MFAP4 chr1 92934792 0.014277 4.76e-10 35353 TRUE reported
textfile 2 1 TRUE
4447 rs141359357 A G A G 0.2002 -0.022 0.03367 NA FALSE FALSE FALSE
GCST006061 0.0238 0.355 537409 outcome TRUE reported 5636_10_MFAP4_MFAP4
MFAP4 chr3 53161104 0.023456 1.402e-17 35343 TRUE reported
textfile 2 1 TRUE
4448 rs144628398 G T G T -0.154 -0.0535 0.02418 NA FALSE FALSE FALSE
GCST006061 0.038 0.1585 537409 outcome TRUE reported 5636_10_MFAP4_MFAP4
MFAP4 chr17 19022840 0.027606 2.426e-08 35359 TRUE reported
textfile 2 1 TRUE
4449 rs144881474 A G A G 0.3811 0.0141 0.01938 NA FALSE FALSE FALSE
GCST006061 0.0375 0.706 537409 outcome TRUE reported 5636_10_MFAP4_MFAP4
MFAP4 chr3 50468499 0.030017 6.219e-37 35341 TRUE reported
textfile 2 1 TRUE
4450 rs186189054 T C T C 0.3425 0.0157 0.01482 NA FALSE FALSE FALSE
GCST006061 0.0561 0.7794 537409 outcome TRUE reported 5636_10_MFAP4_MFAP4
MFAP4 chr3 52075762 0.034193 1.287e-23 35336 TRUE reported
textfile 2 1 TRUE
4451 rs333947 A G A G -0.0716 -0.0071 0.14937 NA FALSE FALSE FALSE
GCST006061 0.01 0.4808 537409 outcome TRUE reported 5636_10_MFAP4_MFAP4
MFAP4 chr1 109928142 0.011787 1.244e-09 35349 TRUE reported
textfile 2 1 TRUE
4452 rs34958 G A G A 0.0534 0.0093 0.46278 NA FALSE FALSE FALSE GCST006061
0.0071 0.1947 537409 outcome TRUE reported 5636_10_MFAP4_MFAP4 MFAP4 chr5
72908272 0.00831 1.31e-10 35354 TRUE reported textfile 2 1 TRUE
4453 rs373724912 T C T C -0.1248 -0.0281 0.03727 NA FALSE FALSE FALSE

GCST006061 0.0445 0.5276 537409 outcome TRUE reported 5636_10_MFAP4_MFAP4
MFAP4 chr17 21613157 0.02229 2.158e-08 35355 TRUE reported textfile
2 1 TRUE
4454 rs635634 C T C T 0.1066 0.0285 0.12995 NA FALSE FALSE FALSE
GCST006061 0.0089 0.001351 537409 outcome TRUE reported 5636_10_MFAP4_MFAP4
MFAP4 chr9 133279427 0.012375 7.058e-18 35371 TRUE reported
textfile 2 1 TRUE
4455 rs74580987 G A G A 0.1145 0.0194 0.05343 NA FALSE FALSE FALSE
GCST006061 0.0173 0.261 537409 outcome TRUE reported 5636_10_MFAP4_MFAP4
MFAP4 chr3 53487594 0.018758 1.035e-09 35351 TRUE reported
textfile 2 1 TRUE
4456 rs74814272 G A G A 0.249 0.0247 0.01224 NA FALSE FALSE FALSE
GCST006061 0.0362 0.4957 537409 outcome TRUE reported 5636_10_MFAP4_MFAP4
MFAP4 chr3 41080294 0.037303 2.47e-11 35353 TRUE reported
textfile 2 1 TRUE
4457 rs930948 C T C T 0.2676 -0.0158 0.01878 NA FALSE FALSE FALSE
GCST006061 0.0308 0.6072 537409 outcome TRUE reported 5636_10_MFAP4_MFAP4
MFAP4 chr3 53643625 0.031381 1.496e-17 35350 TRUE reported
textfile 2 1 TRUE
4458 rs1050541 G T G T -0.1178 6e-04 0.41141 NA FALSE FALSE FALSE
GCST006061 0.0073 0.9394 537409 outcome TRUE reported 7218_87_ATP1B2_AT1B2
ATP1B2 chr17 7657517 0.008638 2.4e-42 35378 TRUE reported textfile 2
1 TRUE
4459 rs105290 C T C T 0.0645 -0.0122 0.44268 NA FALSE FALSE FALSE
GCST006061 0.0073 0.09304 537409 outcome TRUE reported 7218_87_ATP1B2_AT1B2
ATP1B2 chr11 17270521 0.008436 2.078e-14 35370 TRUE reported
textfile 2 1 TRUE
4460 rs2413593 G A G A 0.0846 -0.009 0.40393 NA FALSE FALSE FALSE
GCST006061 0.0076 0.2341 537409 outcome TRUE reported 7218_87_ATP1B2_AT1B2
ATP1B2 chr22 39476122 0.008516 2.971e-23 35366 TRUE reported
textfile 2 1 TRUE
4461 rs34873228 T C T C -0.1556 0.0264 0.08142 NA FALSE FALSE FALSE
GCST006061 0.0113 0.01907 537409 outcome TRUE reported 7218_87_ATP1B2_AT1B2
ATP1B2 chr17 7715419 0.015614 2.157e-23 35377 TRUE reported textfile
2 1 TRUE
4462 rs3759752 T C T C -0.0517 0.0112 0.28276 NA FALSE FALSE FALSE
GCST006061 0.0075 0.138 537409 outcome TRUE reported 7218_87_ATP1B2_AT1B2
ATP1B2 chr14 73947911 0.009267 2.418e-08 35351 TRUE reported
textfile 2 1 TRUE
4463 rs480330 T C T C -0.0526 -0.0242 0.29837 NA FALSE FALSE FALSE
GCST006061 0.0076 0.001381 537409 outcome TRUE reported
7218_87_ATP1B2_AT1B2 ATP1B2 chr3 136310145 0.009148 8.926e-09 35361
TRUE reported textfile 2 1 TRUE
4464 rs61776478 T C T C -0.1568 0.125 0.02717 NA FALSE FALSE FALSE
GCST006061 0.0989 0.2064 537409 outcome TRUE reported 7218_87_ATP1B2_AT1B2
ATP1B2 chr1 11923034 0.025712 1.072e-09 35389 TRUE reported
textfile 2 1 TRUE
4465 rs635634 C T C T 0.195 0.0285 0.12995 NA FALSE FALSE FALSE
GCST006061 0.0089 0.001351 537409 outcome TRUE reported
7218_87_ATP1B2_AT1B2 ATP1B2 chr9 133279427 0.012428 1.745e-55 35371
TRUE reported textfile 2 1 TRUE
4466 rs726679 A G A G -0.0498 -0.0101 0.43031 NA FALSE FALSE FALSE
GCST006061 0.0072 0.1643 537409 outcome TRUE reported 7218_87_ATP1B2_AT1B2
ATP1B2 chr17 8908361 0.00855 5.718e-09 35372 TRUE reported textfile 2
1 TRUE
4467 rs858519 C T C T -0.2678 0.0231 0.47003 NA FALSE FALSE FALSE
GCST006061 0.0072 0.001349 537409 outcome TRUE reported
7218_87_ATP1B2_AT1B2 ATP1B2 chr17 7628647 0.008396 1e-200 35378 TRUE
reported textfile 2 1 TRUE
4468 rs1050628 G A G A -0.2264 0.0647 0.06789 NA FALSE FALSE FALSE
GCST006061 0.0662 0.3284 537409 outcome TRUE reported 7244_16_IGFLR1_TM149
IGFLR1 chr6 31356375 0.016472 5.509e-43 35440 TRUE reported
textfile 2 1 TRUE
4469 rs118146634 A G A G -0.2815 0.0643 0.03017 NA FALSE FALSE FALSE
GCST006061 0.0354 0.06914 537409 outcome TRUE reported 7244_16_IGFLR1_TM149
IGFLR1 chr19 36236310 0.024052 1.217e-31 35377 TRUE reported

textfile 2 1 TRUE
4470 rs12459073 A G A G -0.342 0.0072 0.17816 NA FALSE FALSE FALSE
GCST006061 0.01 0.4706 537409 outcome TRUE reported 7244_16_IGFLR1_TM149
IGFLR1 chr19 35657886 0.010745 1e-200 35376 TRUE reported textfile
2 1 TRUE
4471 rs144347438 T C T C -0.2167 0.0287 0.01815 NA FALSE FALSE FALSE
GCST006061 0.0615 0.6409 537409 outcome TRUE reported 7244_16_IGFLR1_TM149
IGFLR1 chr19 36174580 0.031443 5.509e-12 35370 TRUE reported
textfile 2 1 TRUE
4472 rs145086956 A G A G 0.1595 0.0068 0.11274 NA FALSE FALSE FALSE
GCST006061 0.0113 0.5485 537409 outcome TRUE reported 7244_16_IGFLR1_TM149
IGFLR1 chr19 35971169 0.013009 1.477e-34 35374 TRUE reported
textfile 2 1 TRUE
4473 rs75572774 T C T C -0.1296 -0.0179 0.04203 NA FALSE FALSE FALSE
GCST006061 0.0222 0.4206 537409 outcome TRUE reported 7244_16_IGFLR1_TM149
IGFLR1 chr19 37310048 0.020694 3.781e-10 35376 TRUE reported
textfile 2 1 TRUE
4474 rs1050628 G A G A -0.0899 0.0647 0.06789 NA FALSE FALSE FALSE
GCST006061 0.0662 0.3284 537409 outcome TRUE reported 6538_90_KIAA2013_K2013
KIAA2013 chr6 31356375 0.015765 1.181e-08 35440 TRUE reported
textfile 2 1 TRUE
4475 rs1375663 C A C A 0.0646 0.0049 0.34831 NA FALSE FALSE FALSE
GCST006061 0.0081 0.5435 537409 outcome TRUE reported 6538_90_KIAA2013_K2013
KIAA2013 chr13 48923858 0.008347 1.003e-14 35363 TRUE reported
textfile 2 1 TRUE
4476 rs17281886 G A G A 0.085 -0.0064 0.14987 NA FALSE FALSE FALSE
GCST006061 0.0098 0.515 537409 outcome TRUE reported 6538_90_KIAA2013_K2013
KIAA2013 chr13 46707616 0.011164 2.657e-14 35364 TRUE reported
textfile 2 1 TRUE
4477 rs34289921 C T C T -0.1199 0.0041 0.27648 NA FALSE FALSE FALSE
GCST006061 0.0085 0.6286 537409 outcome TRUE reported 6538_90_KIAA2013_K2013
KIAA2013 chr13 46114124 0.008861 1.024e-41 35358 TRUE reported
textfile 2 1 TRUE
4478 rs7328313 C T C T -0.0628 0.0171 0.38045 NA FALSE FALSE FALSE
GCST006061 0.0075 0.02257 537409 outcome TRUE reported 6538_90_KIAA2013_K2013
KIAA2013 chr13 47864864 0.00818 1.629e-14 35366 TRUE reported
textfile 2 1 TRUE
4479 rs7987805 C T C T 0.177 -0.0162 0.07825 NA FALSE FALSE FALSE
GCST006061 0.0124 0.1894 537409 outcome TRUE reported 6538_90_KIAA2013_K2013
KIAA2013 chr13 45839643 0.01499 3.568e-32 35359 TRUE reported
textfile 2 1 TRUE
4480 rs7995412 A C A C 0.2825 0.0032 0.28884 NA FALSE FALSE FALSE
GCST006061 0.0081 0.6898 537409 outcome TRUE reported 6538_90_KIAA2013_K2013
KIAA2013 chr13 45925929 0.008704 1e-200 35357 TRUE reported
textfile 2 1 TRUE
4481 rs9567566 T C T C 0.0622 0.0019 0.34814 NA FALSE FALSE FALSE
GCST006061 0.0077 0.8013 537409 outcome TRUE reported 6538_90_KIAA2013_K2013
KIAA2013 chr13 45723751 0.008377 1.129e-13 35357 TRUE reported
textfile 2 1 TRUE
4482 rs111494201 A G A G -0.1411 -0.0167 0.05268 NA FALSE FALSE FALSE
GCST006061 0.0315 0.5957 537409 outcome TRUE reported 11177_16_KRT5_K2C5
KRT5 chr17 42135981 0.018015 4.79e-15 35374 TRUE reported
textfile 2 1 TRUE
4483 rs117484558 A G A G -0.2842 -0.0321 0.0311 NA FALSE FALSE FALSE
GCST006061 0.0556 0.5641 537409 outcome TRUE reported 11177_16_KRT5_K2C5
KRT5 chr17 41586669 0.022787 1.061e-35 35375 TRUE reported
textfile 2 1 TRUE
4484 rs118132312 G A G A -0.1272 0.0266 0.0434 NA FALSE FALSE FALSE
GCST006061 0.0316 0.3994 537409 outcome TRUE reported 11177_16_KRT5_K2C5
KRT5 chr17 39694762 0.019638 9.344e-11 35370 TRUE reported
textfile 2 1 TRUE
4485 rs58539907 C T C T -0.179 0.033 0.02867 NA FALSE FALSE FALSE
GCST006061 0.0368 0.3695 537409 outcome TRUE reported 11177_16_KRT5_K2C5
KRT5 chr17 40101687 0.024186 1.351e-13 35365 TRUE reported
textfile 2 1 TRUE
4486 rs7240884 G A G A 0.0754 0.0024 0.43472 NA FALSE FALSE FALSE

GCST006061 0.0073 0.7478 537409 outcome TRUE reported 11177_16_KRT5_K2C5
KRT5 chr18 59901461 0.008084 1.092e-20 35355 TRUE reported
textfile 2 1 TRUE
4487 rs10506328 C A C A 0.0688 0.0071 0.35611 NA FALSE FALSE FALSE
GCST006061 0.0078 0.3608 537409 outcome TRUE reported 13955_33_DAPK1_DAPK1
DAPK1 chr12 54293448 0.008416 2.954e-16 35373 TRUE reported
textfile 2 1 TRUE
4488 rs113252264 A C A C -0.1607 -0.0215 0.02042 NA FALSE FALSE FALSE
GCST006061 0.031 0.4869 537409 outcome TRUE reported 13955_33_DAPK1_DAPK1
DAPK1 chr15 65291524 0.028739 2.248e-08 35361 TRUE reported
textfile 2 1 TRUE
4489 rs1354034 C T C T 0.0597 0.0088 0.38119 NA FALSE FALSE FALSE
GCST006061 0.0073 0.2248 537409 outcome TRUE reported 13955_33_DAPK1_DAPK1
DAPK1 chr3 56815721 0.008259 4.895e-13 35356 TRUE reported
textfile 2 1 TRUE
4490 rs34772505 T C T C 0.2291 -0.0072 0.41744 NA FALSE FALSE FALSE
GCST006061 0.0075 0.3383 537409 outcome TRUE reported 13955_33_DAPK1_DAPK1
DAPK1 chr15 63984573 0.008119 3.519e-175 35363 TRUE reported
textfile 2 1 TRUE
4491 rs7113761 A G A G -0.0528 -0.0146 0.24918 NA FALSE FALSE FALSE
GCST006061 0.0087 0.09269 537409 outcome TRUE reported 13955_33_DAPK1_DAPK1
DAPK1 chr11 65159251 0.009289 1.317e-08 35363 TRUE reported
textfile 2 1 TRUE
4492 rs79227106 C A C A -0.1722 0.0068 0.03657 NA FALSE FALSE FALSE
GCST006061 0.0235 0.7737 537409 outcome TRUE reported 13955_33_DAPK1_DAPK1
DAPK1 chr15 64104226 0.021195 4.495e-16 35361 TRUE reported
textfile 2 1 TRUE
4493 rs10506806 T C T C -0.0492 -0.006 0.37291 NA FALSE FALSE FALSE
GCST006061 0.0078 0.4437 537409 outcome TRUE reported 9565_6_PAWR_PAWR
PAWR chr12 79061202 0.008386 4.432e-09 35360 TRUE reported
textfile 2 1 TRUE
4494 rs114694170 C T C T 0.1235 0.0053 0.08378 NA FALSE FALSE FALSE
GCST006061 0.0175 0.7624 537409 outcome TRUE reported 9565_6_PAWR_PAWR
PAWR chr5 88884379 0.014781 6.541e-17 35344 TRUE reported
textfile 2 1 TRUE
4495 rs4759076 C T C T 0.0692 -0.0011 0.4756 NA FALSE FALSE FALSE
GCST006061 0.0073 0.8799 537409 outcome TRUE reported 9565_6_PAWR_PAWR
PAWR chr12 54336088 0.008166 2.368e-17 35372 TRUE reported
textfile 2 1 TRUE
4496 rs10509186 T C T C 0.0574 -0.0522 0.48106 NA FALSE FALSE FALSE
GCST006061 0.0071 1.598e-13 537409 outcome TRUE reported 3413_50_BCL2A1_BFL1
BCL2A1 chr10 63447258 0.007998 7.126e-13 35351 TRUE reported
textfile 2 1 TRUE
4497 rs111696008 A G A G -0.1505 -0.0259 0.02443 NA FALSE FALSE FALSE
GCST006061 0.0401 0.519 537409 outcome TRUE reported 3413_50_BCL2A1_BFL1
BCL2A1 chr2 216622986 0.026568 1.472e-08 35359 TRUE reported
textfile 2 1 TRUE
4498 rs1355538 G A G A -0.0966 -0.0076 0.39847 NA FALSE FALSE FALSE
GCST006061 0.0072 0.2923 537409 outcome TRUE reported 3413_50_BCL2A1_BFL1
BCL2A1 chr3 165787389 0.008146 1.933e-32 35338 TRUE reported
textfile 2 1 TRUE
4499 rs10509186 T C T C 0.0673 -0.0522 0.48106 NA FALSE FALSE FALSE
GCST006061 0.0071 1.598e-13 537409 outcome TRUE reported 4435_66_ENPP7_ENPP7
ENPP7 chr10 63447258 0.007971 3.08e-17 35351 TRUE reported
textfile 2 1 TRUE
4500 rs113286331 T C T C 0.52 -0.01 0.05825 NA FALSE FALSE FALSE
GCST006061 0.0178 0.5755 537409 outcome TRUE reported 4435_66_ENPP7_ENPP7
ENPP7 chr17 79733484 0.018947 8.109e-166 35375 TRUE reported
textfile 2 1 TRUE
4501 rs147002317 T C T C 0.6571 -0.0714 0.01171 NA FALSE FALSE FALSE
GCST006061 0.0572 0.2118 537409 outcome TRUE reported 4435_66_ENPP7_ENPP7
ENPP7 chr17 79737452 0.039569 6.275e-62 35375 TRUE reported
textfile 2 1 TRUE
4502 rs6130613 T C T C 0.0464 -0.0112 0.49427 NA FALSE FALSE FALSE
GCST006061 0.0074 0.1319 537409 outcome TRUE reported 4435_66_ENPP7_ENPP7
ENPP7 chr20 44425801 0.007985 6.214e-09 35369 TRUE reported

textfile 2 1 TRUE
4503 rs7217011 A C A C 0.0905 0.0075 0.42717 NA FALSE FALSE FALSE
GCST006061 0.0071 0.2939 537409 outcome TRUE reported 4435_66_ENPP7_ENPP7
ENPP7 chr17 80686125 0.00906 1.695e-23 35350 TRUE reported textfile
2 1 TRUE
4504 rs74000618 C T C T 0.1898 -0.0195 0.02511 NA FALSE FALSE FALSE
GCST006061 0.0269 0.4674 537409 outcome TRUE reported 4435_66_ENPP7_ENPP7
ENPP7 chr17 80211194 0.028504 2.761e-11 35385 TRUE reported
textfile 2 1 TRUE
4505 rs80071684 C A C A 0.481 -0.008 0.02607 NA FALSE FALSE FALSE
GCST006061 0.0241 0.7394 537409 outcome TRUE reported 4435_66_ENPP7_ENPP7
ENPP7 chr17 79549910 0.028201 3.134e-65 35366 TRUE reported
textfile 2 1 TRUE
4506 rs10509186 T C T C 0.0568 -0.0522 0.48106 NA FALSE FALSE FALSE
GCST006061 0.0071 1.598e-13 537409 outcome TRUE reported 9910_9_MTAP_MTAP
MTAP chr10 63447258 0.008123 2.694e-12 35351 TRUE reported
textfile 2 1 TRUE
4507 rs13217620 T C T C -0.095 0.0056 0.06733 NA FALSE FALSE FALSE
GCST006061 0.0134 0.6768 537409 outcome TRUE reported 9910_9_MTAP_MTAP
MTAP chr6 27685341 0.016141 3.961e-09 35442 TRUE reported
textfile 2 1 TRUE
4508 rs138711481 G A G A -0.2233 -0.0646 0.0369 NA FALSE FALSE FALSE
GCST006061 0.0276 0.01941 537409 outcome TRUE reported 9910_9_MTAP_MTAP
MTAP chr6 24441857 0.022075 4.726e-24 35380 TRUE reported
textfile 2 1 TRUE
4509 rs2760180 A C A C -0.4498 0.005 0.02277 NA FALSE FALSE FALSE
GCST006061 0.0254 0.8445 537409 outcome TRUE reported 9910_9_MTAP_MTAP
MTAP chr6 24549312 0.027552 6.481e-60 35378 TRUE reported
textfile 2 1 TRUE
4510 rs62269916 A G A G 0.1253 -0.0234 0.03471 NA FALSE FALSE FALSE
GCST006061 0.0238 0.3259 537409 outcome TRUE reported 9910_9_MTAP_MTAP
MTAP chr3 74348841 0.022263 1.821e-08 35343 TRUE reported
textfile 2 1 TRUE
4511 rs75327431 C T C T -0.1018 0.0083 0.05347 NA FALSE FALSE FALSE
GCST006061 0.0222 0.7083 537409 outcome TRUE reported 9910_9_MTAP_MTAP
MTAP chr12 42802980 0.017789 1.048e-08 35361 TRUE reported
textfile 2 1 TRUE
4512 rs79391862 C A C A -0.0974 0.0467 0.06536 NA FALSE FALSE FALSE
GCST006061 0.0414 0.2586 537409 outcome TRUE reported 9910_9_MTAP_MTAP
MTAP chr15 53447229 0.016381 2.751e-09 35362 TRUE reported
textfile 2 1 TRUE
4513 rs13153461 A G A G 0.0894 -0.0019 0.18663 NA FALSE FALSE FALSE
GCST006061 0.008 0.8087 537409 outcome TRUE reported 13642_90_IFIT3_CIG49
IFIT3 chr5 139472784 0.010056 6.118e-19 35356 TRUE reported
textfile 2 1 TRUE
4514 rs4639017 C T C T -0.0442 -0.0061 0.39283 NA FALSE FALSE FALSE
GCST006061 0.0073 0.4022 537409 outcome TRUE reported 13642_90_IFIT3_CIG49
IFIT3 chr3 165833296 0.00804 3.846e-08 35339 TRUE reported textfile
2 1 TRUE
4515 rs10512472 C T C T 0.0842 -0.0014 0.1512 NA FALSE FALSE FALSE
GCST006061 0.009 0.8777 537409 outcome TRUE reported 10390_21_ZNRF3_ZNRF3
ZNRF3 chr17 35557785 0.011188 5.234e-14 35359 TRUE reported
textfile 2 1 TRUE
4516 rs114315741 C T C T 0.2294 -0.1356 0.01304 NA FALSE FALSE FALSE
GCST006061 0.0493 0.005969 537409 outcome TRUE reported
10390_21_ZNRF3_ZNRF3 ZNRF3 chr3 127279529 0.03465 3.581e-11 35355 TRUE
reported textfile 2 1 TRUE
4517 rs1354034 C T C T 0.1451 0.0088 0.38119 NA FALSE FALSE FALSE
GCST006061 0.0073 0.2248 537409 outcome TRUE reported 10390_21_ZNRF3_ZNRF3
ZNRF3 chr3 56815721 0.008252 3.35e-69 35344 TRUE reported
textfile 2 1 TRUE
4518 rs1619994 G A G A 0.0781 0.0168 0.2807 NA FALSE FALSE FALSE
GCST006061 0.0078 0.03057 537409 outcome TRUE reported 10390_21_ZNRF3_ZNRF3
ZNRF3 chr3 165744047 0.008963 2.956e-18 35327 TRUE reported
textfile 2 1 TRUE
4519 rs56312618 A G A G 0.0772 -0.0015 0.24153 NA FALSE FALSE FALSE

GCST006061 0.0089 0.8694 537409 outcome TRUE reported 10390_21_ZNRF3_ZNRF3
ZNRF3 chr11 237209 0.009387 1.972e-16 35340 TRUE reported textfile
2 1 TRUE
4520 rs10513546 A G A G 0.0498 -0.0087 0.30116 NA FALSE FALSE FALSE
GCST006061 0.0077 0.2621 537409 outcome TRUE reported 16315_105_FLT1_VEGF_sR1
FLT1 chr3 159833862 0.008868 1.956e-08 35338 TRUE reported
textfile 2 1 TRUE
4521 rs10922098 T C T C -0.0672 -0.009 0.40011 NA FALSE FALSE FALSE
GCST006061 0.0075 0.2275 537409 outcome TRUE reported 16315_105_FLT1_VEGF_sR1
FLT1 chr1 196695521 0.008286 5.071e-16 35338 TRUE reported
textfile 2 1 TRUE
4522 rs6921438 A G A G 0.1976 0.008 0.47889 NA FALSE FALSE FALSE
GCST006061 0.0072 0.2632 537409 outcome TRUE reported 16315_105_FLT1_VEGF_sR1
FLT1 chr6 43957870 0.008089 8.535e-132 35362 TRUE reported
textfile 2 1 TRUE
4523 rs10513807 A G A G -0.0493 -0.0034 0.49845 NA FALSE FALSE FALSE
GCST006061 0.0072 0.6324 537409 outcome TRUE reported 17331_138_KREMEN1_KREM1
KREMEN1 chr3 186938324 0.008395 4.283e-09 35368 TRUE reported
textfile 2 1 TRUE
4524 rs117210485 A G A G 0.2031 0.033 0.0198 NA FALSE FALSE FALSE
GCST006061 0.0265 0.2129 537409 outcome TRUE reported 17331_138_KREMEN1_KREM1
KREMEN1 chr9 133523026 0.030077 1.452e-11 35360 TRUE reported
textfile 2 1 TRUE
4525 rs138013760 T C T C -0.3366 -0.0382 0.01593 NA FALSE FALSE FALSE
GCST006061 0.0342 0.265 537409 outcome TRUE reported 17331_138_KREMEN1_KREM1
KREMEN1 chr22 31393529 0.032995 1.949e-24 35365 TRUE reported
textfile 2 1 TRUE
4526 rs143501783 C T C T 0.2354 -0.0333 0.01116 NA FALSE FALSE FALSE
GCST006061 0.0443 0.4525 537409 outcome TRUE reported 17331_138_KREMEN1_KREM1
KREMEN1 chr17 7897001 0.038164 6.913e-10 35373 TRUE reported textfile
2 1 TRUE
4527 rs147756739 T C T C -0.2051 -0.0193 0.02081 NA FALSE FALSE FALSE
GCST006061 0.0335 0.5644 537409 outcome TRUE reported 17331_138_KREMEN1_KREM1
KREMEN1 chr22 31894587 0.029479 3.465e-12 35366 TRUE reported
textfile 2 1 TRUE
4528 rs1929423 A G A G -0.1405 -0.0057 0.0386 NA FALSE FALSE FALSE
GCST006061 0.0205 0.782 537409 outcome TRUE reported 17331_138_KREMEN1_KREM1
KREMEN1 chr10 18156755 0.021799 1.154e-10 35358 TRUE reported
textfile 2 1 TRUE
4529 rs2205771 A G A G 0.2258 -0.0058 0.17462 NA FALSE FALSE FALSE
GCST006061 0.0095 0.5406 537409 outcome TRUE reported 17331_138_KREMEN1_KREM1
KREMEN1 chr22 29098605 0.011143 2.679e-91 35377 TRUE reported
textfile 2 1 TRUE
4530 rs333947 A G A G -0.0813 -0.0071 0.14937 NA FALSE FALSE FALSE
GCST006061 0.01 0.4808 537409 outcome TRUE reported 17331_138_KREMEN1_KREM1
KREMEN1 chr1 109928142 0.011866 7.293e-12 35349 TRUE reported
textfile 2 1 TRUE
4531 rs34931250 T C T C -0.1097 0.0028 0.08188 NA FALSE FALSE FALSE
GCST006061 0.0174 0.8738 537409 outcome TRUE reported 17331_138_KREMEN1_KREM1
KREMEN1 chr17 68883786 0.015383 9.937e-13 35372 TRUE reported
textfile 2 1 TRUE
4532 rs4704044 T C T C 0.0506 0.0099 0.46368 NA FALSE FALSE FALSE
GCST006061 0.0071 0.1621 537409 outcome TRUE reported 17331_138_KREMEN1_KREM1
KREMEN1 chr5 72884820 0.008392 1.641e-09 35354 TRUE reported
textfile 2 1 TRUE
4533 rs56344867 C T C T 0.051 -0.005 0.35465 NA FALSE FALSE FALSE
GCST006061 0.008 0.5298 537409 outcome TRUE reported 17331_138_KREMEN1_KREM1
KREMEN1 chr11 126361219 0.008772 6.093e-09 35374 TRUE reported
textfile 2 1 TRUE
4534 rs5762962 A G A G -0.1661 -0.02 0.02878 NA FALSE FALSE FALSE
GCST006061 0.02 0.3163 537409 outcome TRUE reported 17331_138_KREMEN1_KREM1
KREMEN1 chr22 29056293 0.025041 3.286e-11 35383 TRUE reported
textfile 2 1 TRUE
4535 rs635634 C T C T 0.1197 0.0285 0.12995 NA FALSE FALSE FALSE
GCST006061 0.0089 0.001351 537409 outcome TRUE reported
17331_138_KREMEN1_KREM1 KREMEN1 chr9 133279427 0.012476 8.456e-22 35371

TRUE reported textfile 2 1 TRUE
4536 rs6695321 G A G A -0.0502 -0.0121 0.40304 NA FALSE FALSE FALSE
GCST006061 0.0074 0.1028 537409 outcome TRUE reported 17331_138_KREMEN1_KREM1
KREMEN1 chr1 196706731 0.008525 3.893e-09 35339 TRUE reported
textfile 2 1 TRUE
4537 rs723183 A G A G -0.3989 0.0419 0.01244 NA FALSE FALSE FALSE
GCST006061 0.0253 0.09839 537409 outcome TRUE reported 17331_138_KREMEN1_KREM1
KREMEN1 chr22 27566757 0.037894 6.496e-26 35375 TRUE reported
textfile 2 1 TRUE
4538 rs7716761 T C T C 0.0562 -0.0205 0.42505 NA FALSE FALSE FALSE
GCST006061 0.0072 0.004602 537409 outcome TRUE reported
17331_138_KREMEN1_KREM1 KREMEN1 chr5 109788070 0.008509 3.991e-11 35351
TRUE reported textfile 2 1 TRUE
4539 rs77542162 G A G A 0.1643 0.0177 0.025 NA FALSE FALSE FALSE
GCST006061 0.0302 0.557 537409 outcome TRUE reported 17331_138_KREMEN1_KREM1
KREMEN1 chr17 69085137 0.026459 5.311e-10 35371 TRUE reported
textfile 2 1 TRUE
4540 rs876556 T C T C 0.0505 0.0015 0.31807 NA FALSE FALSE FALSE
GCST006061 0.0076 0.8465 537409 outcome TRUE reported 17331_138_KREMEN1_KREM1
KREMEN1 chr12 89491665 0.00904 2.32e-08 35357 TRUE reported textfile
2 1 TRUE
4541 rs9862378 T G T G -0.0543 -0.0332 0.49422 NA FALSE FALSE FALSE
GCST006061 0.0072 4.254e-06 537409 outcome TRUE reported
17331_138_KREMEN1_KREM1 KREMEN1 chr3 58338667 0.008427 1.166e-10 35357
TRUE reported textfile 2 1 TRUE
4542 rs10935478 T C T C -0.0625 4e-04 0.4385 NA FALSE FALSE FALSE
GCST006061 0.0072 0.9573 537409 outcome TRUE reported 5092_51_JAG1_JAG1
JAG1 chr3 98709311 0.008105 1.241e-14 35330 TRUE reported
textfile 2 1 TRUE
4543 rs41307428 T C T C 0.29 0.0291 0.01987 NA FALSE FALSE FALSE
GCST006061 0.023 0.206 537409 outcome TRUE reported 5092_51_JAG1_JAG1
JAG1 chr9 133471682 0.028795 7.409e-24 35369 TRUE reported
textfile 2 1 TRUE
4544 rs55714927 T C T C 0.0795 1e-04 0.21166 NA FALSE FALSE FALSE
GCST006061 0.0098 0.9926 537409 outcome TRUE reported 5092_51_JAG1_JAG1
JAG1 chr17 7176997 0.009944 1.299e-15 35370 TRUE reported textfile
2 1 TRUE
4545 rs635634 C T C T 0.2073 0.0285 0.12995 NA FALSE FALSE FALSE
GCST006061 0.0089 0.001351 537409 outcome TRUE reported 5092_51_JAG1_JAG1
JAG1 chr9 133279427 0.011994 6.207e-67 35371 TRUE reported
textfile 2 1 TRUE
4546 rs77542162 G A G A 0.184 0.0177 0.025 NA FALSE FALSE FALSE
GCST006061 0.0302 0.557 537409 outcome TRUE reported 5092_51_JAG1_JAG1
JAG1 chr17 69085137 0.025465 4.992e-13 35371 TRUE reported
textfile 2 1 TRUE
4547 rs10922098 T C T C -0.076 -0.009 0.40011 NA FALSE FALSE FALSE
GCST006061 0.0075 0.2275 537409 outcome TRUE reported 13101_60_SOST_SOST
SOST chr1 196695521 0.008341 8.103e-20 35329 TRUE reported
textfile 2 1 TRUE
4548 rs10955898 T C T C -0.0658 -0.0141 0.13491 NA FALSE FALSE FALSE
GCST006061 0.0106 0.1825 537409 outcome TRUE reported 13101_60_SOST_SOST
SOST chr8 118781344 0.011915 3.341e-08 35351 TRUE reported
textfile 2 1 TRUE
4549 rs1485307 C T C T -0.0871 -0.0019 0.4666 NA FALSE FALSE FALSE
GCST006061 0.0071 0.7851 537409 outcome TRUE reported 13101_60_SOST_SOST
SOST chr8 118995156 0.00817 1.554e-26 35353 TRUE reported textfile
2 1 TRUE
4550 rs17138656 A G A G 0.1071 6e-04 0.12785 NA FALSE FALSE FALSE
GCST006061 0.0107 0.9575 537409 outcome TRUE reported 13101_60_SOST_SOST
SOST chr5 116013119 0.012136 1.092e-18 35342 TRUE reported
textfile 2 1 TRUE
4551 rs244730 A G A G 0.0547 -0.0204 0.30061 NA FALSE FALSE FALSE
GCST006061 0.0088 0.02061 537409 outcome TRUE reported 13101_60_SOST_SOST
SOST chr5 177112211 0.008927 8.943e-10 35358 TRUE reported
textfile 2 1 TRUE
4552 rs2669572 T C T C 0.0447 0.0076 0.46488 NA FALSE FALSE FALSE

GCST006061 0.0072 0.2918 537409 outcome TRUE reported 13101_60_SOST_SOST
SOST chr3 165660856 0.008189 4.795e-08 35328 TRUE reported
textfile 2 1 TRUE
4553 rs7294354 G T G T 0.0619 0.0186 0.43907 NA FALSE FALSE FALSE
GCST006061 0.0072 0.01012 537409 outcome TRUE reported 13101_60_SOST_SOST
SOST chr12 479778 0.008227 5.301e-14 35302 TRUE reported textfile
2 1 TRUE
4554 rs10516428 G T G T -0.054 -8.00E-04 0.23193 NA FALSE FALSE FALSE
GCST006061 0.0082 0.9239 537409 outcome TRUE reported
14106_46_RAP1GDS1_GDP_GTP_exchange_protein RAP1GDS1 chr4 98316288 0.009444
1.08e-08 35336 TRUE reported textfile 2 1 TRUE
4555 rs1354034 C T C T 0.0531 0.0088 0.38119 NA FALSE FALSE FALSE
GCST006061 0.0073 0.2248 537409 outcome TRUE reported
14106_46_RAP1GDS1_GDP_GTP_exchange_protein RAP1GDS1 chr3 56815721 0.00827
1.357e-10 35356 TRUE reported textfile 2 1 TRUE
4556 rs4980320 C T C T 0.0452 0.0041 0.46986 NA FALSE FALSE FALSE
GCST006061 0.0076 0.5858 537409 outcome TRUE reported
14106_46_RAP1GDS1_GDP_GTP_exchange_protein RAP1GDS1 chr11 198986 0.008062
2.067e-08 35352 TRUE reported textfile 2 1 TRUE
4557 rs705016 T C T C 0.0506 0.0182 0.30763 NA FALSE FALSE FALSE
GCST006061 0.0075 0.01538 537409 outcome TRUE reported
14106_46_RAP1GDS1_GDP_GTP_exchange_protein RAP1GDS1 chr3 165725994 0.008724
6.622e-09 35339 TRUE reported textfile 2 1 TRUE
4558 rs150214082 T C T C 0.264 0.046 0.01244 NA FALSE FALSE FALSE
GCST006061 0.036 0.2012 537409 outcome TRUE reported 18330_7_PGM2_PGM2
PGM2 chr4 40399136 0.036426 4.241e-13 35359 TRUE reported
textfile 2 1 TRUE
4559 rs16995688 G A G A 0.2584 0.0024 0.01295 NA FALSE FALSE FALSE
GCST006061 0.0339 0.9426 537409 outcome TRUE reported 18330_7_PGM2_PGM2
PGM2 chr4 40258578 0.034406 5.896e-14 35362 TRUE reported
textfile 2 1 TRUE
4560 rs1995963 T C T C 0.1388 -1.00E-04 0.02978 NA FALSE FALSE FALSE
GCST006061 0.0195 0.9948 537409 outcome TRUE reported 18330_7_PGM2_PGM2
PGM2 chr4 38276283 0.023585 3.976e-09 35363 TRUE reported
textfile 2 1 TRUE
4561 rs28434334 C T C T 0.3609 -0.0159 0.02164 NA FALSE FALSE FALSE
GCST006061 0.0217 0.466 537409 outcome TRUE reported 18330_7_PGM2_PGM2
PGM2 chr4 37798001 0.028017 5.727e-38 35363 TRUE reported
textfile 2 1 TRUE
4562 rs77566893 A G A G 0.1427 -0.0483 0.02598 NA FALSE FALSE FALSE
GCST006061 0.0269 0.0724 537409 outcome TRUE reported 18330_7_PGM2_PGM2
PGM2 chr4 37898324 0.024835 9.14e-09 35362 TRUE reported
textfile 2 1 TRUE
4563 rs77864400 A G A G -0.0913 -0.0017 0.06796 NA FALSE FALSE FALSE
GCST006061 0.0308 0.957 537409 outcome TRUE reported 18330_7_PGM2_PGM2
PGM2 chr4 37827794 0.015773 7.112e-09 35364 TRUE reported
textfile 2 1 TRUE
4564 rs10836312 C T C T -0.2147 0.0052 0.42652 NA FALSE FALSE FALSE
GCST006061 0.0072 0.4667 537409 outcome TRUE reported
6252_62_SCGB3A1_Secretoglobin_family_3A_member_1 SCGB3A1 chr11 34788896
0.008178 6.565e-152 35378 TRUE reported textfile 2 1 TRUE
4565 rs11032734 C T C T 0.0561 0.0018 0.30321 NA FALSE FALSE FALSE
GCST006061 0.0081 0.8292 537409 outcome TRUE reported
6252_62_SCGB3A1_Secretoglobin_family_3A_member_1 SCGB3A1 chr11 34513355
0.008821 2.023e-10 35377 TRUE reported textfile 2 1 TRUE
4566 rs11741572 G A G A -0.0451 -0.0145 0.48056 NA FALSE FALSE FALSE
GCST006061 0.0073 0.046 537409 outcome TRUE reported
6252_62_SCGB3A1_Secretoglobin_family_3A_member_1 SCGB3A1 chr5 52073151
0.008116 2.749e-08 35363 TRUE reported textfile 2 1 TRUE
4567 rs1420533 A G A G -0.0501 -0.0068 0.46073 NA FALSE FALSE FALSE
GCST006061 0.0072 0.3436 537409 outcome TRUE reported
6252_62_SCGB3A1_Secretoglobin_family_3A_member_1 SCGB3A1 chr16 52529714
0.008077 5.551e-10 35363 TRUE reported textfile 2 1 TRUE
4568 rs1515496 C T C T -0.0788 -0.0023 0.43249 NA FALSE FALSE FALSE
GCST006061 0.0076 0.7648 537409 outcome TRUE reported
6252_62_SCGB3A1_Secretoglobin_family_3A_member_1 SCGB3A1 chr3 189790777

0.008156 4.394e-22 35380 TRUE reported textfile 2 1 TRUE
4569 rs17257455 G A G A 0.0546 -0.0063 0.27325 NA FALSE FALSE FALSE
GCST006061 0.0077 0.4162 537409 outcome TRUE reported
6252_62_SCGB3A1_Secretoglobin_family_3A_member_1 SCGB3A1 chr1 117347907
0.009032 1.492e-09 35364 TRUE reported textfile 2 1 TRUE
4570 rs17678708 T C T C -0.0643 -0.0233 0.27005 NA FALSE FALSE FALSE
GCST006061 0.008 0.003665 537409 outcome TRUE reported
6252_62_SCGB3A1_Secretoglobin_family_3A_member_1 SCGB3A1 chr5 57292983
0.009133 1.915e-12 35366 TRUE reported textfile 2 1 TRUE
4571 rs186618572 G A G A 0.1098 -0.0244 0.04217 NA FALSE FALSE FALSE
GCST006061 0.0285 0.3931 537409 outcome TRUE reported
6252_62_SCGB3A1_Secretoglobin_family_3A_member_1 SCGB3A1 chr11 63681535
0.020021 4.154e-08 35362 TRUE reported textfile 2 1 TRUE
4572 rs1877593 T C T C -0.0564 -0.0035 0.3237 NA FALSE FALSE FALSE
GCST006061 0.0075 0.6382 537409 outcome TRUE reported
6252_62_SCGB3A1_Secretoglobin_family_3A_member_1 SCGB3A1 chr3 157820199
0.008593 5.267e-11 35337 TRUE reported textfile 2 1 TRUE
4573 rs2238133 C A C A -0.075 -0.0013 0.41356 NA FALSE FALSE FALSE
GCST006061 0.0076 0.8664 537409 outcome TRUE reported
6252_62_SCGB3A1_Secretoglobin_family_3A_member_1 SCGB3A1 chr12 11878531
0.008161 3.919e-20 35373 TRUE reported textfile 2 1 TRUE
4574 rs2431320 T C T C -0.1331 -0.008 0.35445 NA FALSE FALSE FALSE
GCST006061 0.0074 0.2853 537409 outcome TRUE reported
6252_62_SCGB3A1_Secretoglobin_family_3A_member_1 SCGB3A1 chr5 102782681
0.008419 2.684e-56 35343 TRUE reported textfile 2 1 TRUE
4575 rs307803 C T C T 0.1204 -0.0081 0.38443 NA FALSE FALSE FALSE
GCST006061 0.0075 0.2787 537409 outcome TRUE reported
6252_62_SCGB3A1_Secretoglobin_family_3A_member_1 SCGB3A1 chr5 180592395
0.008317 1.708e-47 35330 TRUE reported textfile 2 1 TRUE
4576 rs34249863 A G A G -0.0848 0.0049 0.42599 NA FALSE FALSE FALSE
GCST006061 0.0073 0.5075 537409 outcome TRUE reported
6252_62_SCGB3A1_Secretoglobin_family_3A_member_1 SCGB3A1 chr7 98053596
0.008138 2.008e-25 35368 TRUE reported textfile 2 1 TRUE
4577 rs3741240 A G A G 0.1861 -0.0048 0.34036 NA FALSE FALSE FALSE
GCST006061 0.0075 0.5223 537409 outcome TRUE reported
6252_62_SCGB3A1_Secretoglobin_family_3A_member_1 SCGB3A1 chr11 62419070
0.008554 6.231e-105 35362 TRUE reported textfile 2 1 TRUE
4578 rs382266 C T C T 0.1824 -0.0043 0.075 NA FALSE FALSE FALSE
GCST006061 0.0135 0.7517 537409 outcome TRUE reported
6252_62_SCGB3A1_Secretoglobin_family_3A_member_1 SCGB3A1 chr5 180620412
0.015423 2.856e-32 35332 TRUE reported textfile 2 1 TRUE
4579 rs4671448 T C T C 0.0834 0.0035 0.40041 NA FALSE FALSE FALSE
GCST006061 0.0074 0.635 537409 outcome TRUE reported
6252_62_SCGB3A1_Secretoglobin_family_3A_member_1 SCGB3A1 chr2 62754663
0.008249 4.997e-24 35349 TRUE reported textfile 2 1 TRUE
4580 rs4970836 A G A G 0.0712 -0.0169 0.2075 NA FALSE FALSE FALSE
GCST006061 0.0087 0.05153 537409 outcome TRUE reported
6252_62_SCGB3A1_Secretoglobin_family_3A_member_1 SCGB3A1 chr1 109279175
0.009974 9.416e-13 35344 TRUE reported textfile 2 1 TRUE
4581 rs56228609 T C T C 0.0698 0.0116 0.31582 NA FALSE FALSE FALSE
GCST006061 0.0078 0.1378 537409 outcome TRUE reported
6252_62_SCGB3A1_Secretoglobin_family_3A_member_1 SCGB3A1 chr16 56953853
0.008634 6.25e-16 35369 TRUE reported textfile 2 1 TRUE
4582 rs61703905 T C T C 0.1201 -0.0015 0.11012 NA FALSE FALSE FALSE
GCST006061 0.012 0.8983 537409 outcome TRUE reported
6252_62_SCGB3A1_Secretoglobin_family_3A_member_1 SCGB3A1 chr19 45867043
0.012876 1.082e-20 35369 TRUE reported textfile 2 1 TRUE
4583 rs704735 T C T C 0.1468 -0.0076 0.27921 NA FALSE FALSE FALSE
GCST006061 0.0081 0.3439 537409 outcome TRUE reported
6252_62_SCGB3A1_Secretoglobin_family_3A_member_1 SCGB3A1 chr11 34674224
0.009067 5.864e-59 35378 TRUE reported textfile 2 1 TRUE
4584 rs7122541 T C T C 0.0757 -0.0203 0.13457 NA FALSE FALSE FALSE
GCST006061 0.0127 0.1099 537409 outcome TRUE reported
6252_62_SCGB3A1_Secretoglobin_family_3A_member_1 SCGB3A1 chr11 62557404 0.01181
1.459e-10 35362 TRUE reported textfile 2 1 TRUE
4585 rs712333 C T C T 0.0671 -0.0171 0.16762 NA FALSE FALSE FALSE

GCST006061 0.0092 0.06395 537409 outcome TRUE reported
6252_62_SCGB3A1_Secretoglobin_family_3A_member_1 SCGB3A1 chr14 36797605
0.010907 7.65e-10 35360 TRUE reported textfile 2 1 TRUE
4586 rs771997 A G A G 0.0556 0.0076 0.32796 NA FALSE FALSE FALSE
GCST006061 0.0076 0.316 537409 outcome TRUE reported
6252_62_SCGB3A1_Secretoglobin_family_3A_member_1 SCGB3A1 chr12 51964384
0.008568 8.63e-11 35383 TRUE reported textfile 2 1 TRUE
4587 rs7728174 A G A G 0.0922 -0.0114 0.06692 NA FALSE FALSE FALSE
GCST006061 0.0156 0.466 537409 outcome TRUE reported
6252_62_SCGB3A1_Secretoglobin_family_3A_member_1 SCGB3A1 chr5 180357979
0.016284 1.495e-08 35330 TRUE reported textfile 2 1 TRUE
4588 rs77519145 C T C T -0.137 0.0396 0.05417 NA FALSE FALSE FALSE
GCST006061 0.0175 0.02355 537409 outcome TRUE reported
6252_62_SCGB3A1_Secretoglobin_family_3A_member_1 SCGB3A1 chr18 22542379
0.017562 6.147e-15 35349 TRUE reported textfile 2 1 TRUE
4589 rs10521222 T C T C 0.0946 -0.0262 0.06103 NA FALSE FALSE FALSE
GCST006061 0.0187 0.1608 537409 outcome TRUE reported
14133_93_IL1R2_IL_1_sRII IL1R2 chr16 51124799 0.017039 2.824e-08 35370
TRUE reported textfile 2 1 TRUE
4590 rs111645402 G A G A -0.2456 0.0135 0.0175 NA FALSE FALSE FALSE
GCST006061 0.0317 0.6702 537409 outcome TRUE reported
14133_93_IL1R2_IL_1_sRII IL1R2 chr2 95185101 0.031421 5.434e-15 35364
TRUE reported textfile 2 1 TRUE
4591 rs1169288 C A C A 0.0588 -0.0103 0.31006 NA FALSE FALSE FALSE
GCST006061 0.0077 0.1791 537409 outcome TRUE reported
14133_93_IL1R2_IL_1_sRII IL1R2 chr12 120978847 0.008764 1.96e-11 35365
TRUE reported textfile 2 1 TRUE
4592 rs141094656 C T C T -0.1887 0.018 0.02759 NA FALSE FALSE FALSE
GCST006061 0.0394 0.6484 537409 outcome TRUE reported
14133_93_IL1R2_IL_1_sRII IL1R2 chr1 92287779 0.024638 1.876e-14 35352
TRUE reported textfile 2 1 TRUE
4593 rs143501783 C T C T 0.2268 -0.0333 0.01116 NA FALSE FALSE FALSE
GCST006061 0.0443 0.4525 537409 outcome TRUE reported
14133_93_IL1R2_IL_1_sRII IL1R2 chr17 7897001 0.036816 7.259e-10 35373
TRUE reported textfile 2 1 TRUE
4594 rs17030637 C T C T 0.1725 -0.0224 0.0265 NA FALSE FALSE FALSE
GCST006061 0.0463 0.6277 537409 outcome TRUE reported
14133_93_IL1R2_IL_1_sRII IL1R2 chr2 105174301 0.025839 2.457e-11 35365
TRUE reported textfile 2 1 TRUE
4595 rs1870140 G A G A 0.0684 0.0203 0.14008 NA FALSE FALSE FALSE
GCST006061 0.0102 0.04694 537409 outcome TRUE reported
14133_93_IL1R2_IL_1_sRII IL1R2 chr10 80486993 0.011664 4.508e-09 35370
TRUE reported textfile 2 1 TRUE
4596 rs1892548 C T C T 0.0541 0.0016 0.34568 NA FALSE FALSE FALSE
GCST006061 0.0076 0.831 537409 outcome TRUE reported
14133_93_IL1R2_IL_1_sRII IL1R2 chr13 40428504 0.008578 2.851e-10 35361
TRUE reported textfile 2 1 TRUE
4597 rs469802 G A G A -0.056 -0.0043 0.22108 NA FALSE FALSE FALSE
GCST006061 0.009 0.6307 537409 outcome TRUE reported
14133_93_IL1R2_IL_1_sRII IL1R2 chr1 91080269 0.009778 1.023e-08 35342
TRUE reported textfile 2 1 TRUE
4598 rs55714927 T C T C 0.0799 1e-04 0.21166 NA FALSE FALSE FALSE
GCST006061 0.0098 0.9926 537409 outcome TRUE reported
14133_93_IL1R2_IL_1_sRII IL1R2 chr17 7176997 0.009977 1.165e-15 35370
TRUE reported textfile 2 1 TRUE
4599 rs7120907 T C T C 0.0538 0.006 0.39507 NA FALSE FALSE FALSE
GCST006061 0.0072 0.4091 537409 outcome TRUE reported
14133_93_IL1R2_IL_1_sRII IL1R2 chr11 94593814 0.008258 7.269e-11 35358
TRUE reported textfile 2 1 TRUE
4600 rs77542162 G A G A 0.1916 0.0177 0.025 NA FALSE FALSE FALSE
GCST006061 0.0302 0.557 537409 outcome TRUE reported
14133_93_IL1R2_IL_1_sRII IL1R2 chr17 69085137 0.025522 6.034e-14 35371
TRUE reported textfile 2 1 TRUE
4601 rs7779671 G A G A 0.0577 0.0028 0.48596 NA FALSE FALSE FALSE
GCST006061 0.0071 0.6937 537409 outcome TRUE reported
14133_93_IL1R2_IL_1_sRII IL1R2 chr7 129102167 0.008055 7.88e-13 35351

TRUE reported textfile 2 1 TRUE
4602 rs78423067 A G A G -0.6643 -0.0213 0.03461 NA FALSE FALSE FALSE
GCST006061 0.0402 0.5965 537409 outcome TRUE reported
14133_93_IL1R2_IL_1_sRII IL1R2 chr2 101991888 0.022453 2.277e-192 35368
TRUE reported textfile 2 1 TRUE
4603 rs79204775 C T C T -0.1356 0.0056 0.06765 NA FALSE FALSE FALSE
GCST006061 0.0164 0.7333 537409 outcome TRUE reported
14133_93_IL1R2_IL_1_sRII IL1R2 chr2 104251541 0.016126 4.142e-17 35360
TRUE reported textfile 2 1 TRUE
4604 rs1052454 A C A C 0.053 -0.0131 0.38703 NA FALSE FALSE FALSE
GCST006061 0.0076 0.08636 537409 outcome TRUE reported
6367_66_FMOD_fibromodulin FMOD chr12 563185 0.008514 4.814e-10 35312
TRUE reported textfile 2 1 TRUE
4605 rs11128309 C A C A 0.0484 -0.0101 0.49016 NA FALSE FALSE FALSE
GCST006061 0.0072 0.1589 537409 outcome TRUE reported
6367_66_FMOD_fibromodulin FMOD chr3 73096907 0.008257 4.584e-09 35350
TRUE reported textfile 2 1 TRUE
4606 rs117801489 C T C T -0.4171 -0.0141 0.04343 NA FALSE FALSE FALSE
GCST006061 0.034 0.6772 537409 outcome TRUE reported
6367_66_FMOD_fibromodulin FMOD chr12 104015054 0.020099 1.163e-95 35361
TRUE reported textfile 2 1 TRUE
4607 rs12581309 A C A C -0.088 -2.00E-04 0.11297 NA FALSE FALSE FALSE
GCST006061 0.0119 0.9833 537409 outcome TRUE reported
6367_66_FMOD_fibromodulin FMOD chr12 104452194 0.012925 9.863e-12 35360
TRUE reported textfile 2 1 TRUE
4608 rs139343579 T C T C -0.1498 0.0247 0.0299 NA FALSE FALSE FALSE
GCST006061 0.0293 0.3983 537409 outcome TRUE reported
6367_66_FMOD_fibromodulin FMOD chr1 203567529 0.024057 4.757e-10 35362
TRUE reported textfile 2 1 TRUE
4609 rs139774444 T C T C -0.1241 0.0743 0.03706 NA FALSE FALSE FALSE
GCST006061 0.0583 0.2025 537409 outcome TRUE reported
6367_66_FMOD_fibromodulin FMOD chr1 202148970 0.022158 2.136e-08 35358
TRUE reported textfile 2 1 TRUE
4610 rs16851364 A G A G -0.2396 0.0012 0.07712 NA FALSE FALSE FALSE
GCST006061 0.0106 0.9091 537409 outcome TRUE reported
6367_66_FMOD_fibromodulin FMOD chr1 203349016 0.015397 1.326e-54 35368
TRUE reported textfile 2 1 TRUE
4611 rs36136188 T C T C -0.2528 0.0759 0.01051 NA FALSE FALSE FALSE
GCST006061 0.0405 0.06065 537409 outcome TRUE reported
6367_66_FMOD_fibromodulin FMOD chr12 103296052 0.040932 6.573e-10 35366
TRUE reported textfile 2 1 TRUE
4612 rs56035601 T C T C -0.1003 -0.0216 0.10416 NA FALSE FALSE FALSE
GCST006061 0.0125 0.08368 537409 outcome TRUE reported
6367_66_FMOD_fibromodulin FMOD chr1 203227714 0.01359 1.58e-13 35362
TRUE reported textfile 2 1 TRUE
4613 rs78242118 C T C T -0.1647 0.0346 0.02666 NA FALSE FALSE FALSE
GCST006061 0.0226 0.1257 537409 outcome TRUE reported
6367_66_FMOD_fibromodulin FMOD chr1 205423145 0.026407 4.459e-10 35370
TRUE reported textfile 2 1 TRUE
4614 rs9033 G A G A 0.0537 -0.0109 0.40506 NA FALSE FALSE FALSE GCST006061
0.0073 0.1323 537409 outcome TRUE reported 6367_66_FMOD_fibromodulin FMOD
chr16 67148096 0.008352 1.277e-10 35371 TRUE reported textfile 2
1 TRUE
4615 rs1053733 A G A G 0.0461 -7.00E-04 0.40068 NA FALSE FALSE FALSE
GCST006061 0.0072 0.9217 537409 outcome TRUE reported 5196_7_NMT1_NMT1
NMT1 chr17 45105660 0.008173 1.693e-08 35368 TRUE reported
textfile 2 1 TRUE
4616 rs1354034 C T C T 0.0772 0.0088 0.38119 NA FALSE FALSE FALSE
GCST006061 0.0073 0.2248 537409 outcome TRUE reported 5196_7_NMT1_NMT1
NMT1 chr3 56815721 0.008267 9.832e-21 35356 TRUE reported
textfile 2 1 TRUE
4617 rs35340377 A G A G -0.0554 0.0075 0.25094 NA FALSE FALSE FALSE
GCST006061 0.0082 0.3585 537409 outcome TRUE reported 5196_7_NMT1_NMT1
NMT1 chr1 247874908 0.009353 3.156e-09 35156 TRUE reported
textfile 2 1 TRUE
4618 rs1055167 A G A G -0.0501 -0.0063 0.42075 NA FALSE FALSE FALSE

GCST006061 0.0073 0.3872 537409 outcome TRUE reported 16015_19_GPT_ALT GPT chr8 144485003 0.008242 1.21e-09 34530 TRUE reported textfile 2 1 TRUE
4619 rs10922098 T C T C -0.2415 -0.009 0.40011 NA FALSE FALSE FALSE GCST006061 0.0075 0.2275 537409 outcome TRUE reported 16015_19_GPT_ALT GPT chr1 196695521 0.008146 3.682e-193 35338 TRUE reported textfile 2 1 TRUE
4620 rs1451915 A C A C 0.0756 -0.0107 0.13641 NA FALSE FALSE FALSE GCST006061 0.0124 0.3895 537409 outcome TRUE reported 16015_19_GPT_ALT GPT chr1 196058002 0.011658 8.897e-11 35335 TRUE reported textfile 2 1 TRUE
4621 rs2686395 C T C T -0.1067 -0.0077 0.3101 NA FALSE FALSE FALSE GCST006061 0.0074 0.2965 537409 outcome TRUE reported 16015_19_GPT_ALT GPT chr3 165767231 0.008649 5.73e-35 35339 TRUE reported textfile 2 1 TRUE
4622 rs704 A G A G -0.0542 -0.0155 0.49189 NA FALSE FALSE FALSE GCST006061 0.0072 0.03144 537409 outcome TRUE reported 16015_19_GPT_ALT GPT chr17 28367840 0.008038 1.551e-11 35360 TRUE reported textfile 2 1 TRUE
4623 rs7513100 C T C T 0.0669 0 0.14871 NA FALSE FALSE FALSE GCST006061 0.0098 0.9981 537409 outcome TRUE reported 16015_19_GPT_ALT GPT chr1 198065935 0.011274 2.954e-09 35343 TRUE reported textfile 2 1 TRUE
4624 rs1056429 A G A G 0.0611 0.0021 0.14978 NA FALSE FALSE FALSE GCST006061 0.0118 0.8564 537409 outcome TRUE reported 13470_43_PTH1R_PTH1R PTH1R chr6 31354106 0.011058 3.291e-08 35434 TRUE reported textfile 2 1 TRUE
4625 rs10922098 T C T C -0.1162 -0.009 0.40011 NA FALSE FALSE FALSE GCST006061 0.0075 0.2275 537409 outcome TRUE reported 13470_43_PTH1R_PTH1R PTH1R chr1 196695521 0.008098 1.064e-46 35332 TRUE reported textfile 2 1 TRUE
4626 rs1706435 G A G A 0.0606 0.0117 0.36981 NA FALSE FALSE FALSE GCST006061 0.0072 0.1047 537409 outcome TRUE reported 13470_43_PTH1R_PTH1R PTH1R chr3 165743688 0.008207 1.539e-13 35333 TRUE reported textfile 2 1 TRUE
4627 rs6542680 T C T C -0.0566 -0.0012 0.18362 NA FALSE FALSE FALSE GCST006061 0.0091 0.8935 537409 outcome TRUE reported 13470_43_PTH1R_PTH1R PTH1R chr2 3592552 0.010281 3.684e-08 35326 TRUE reported textfile 2 1 TRUE
4628 rs1056429 A G A G 0.0611 0.0021 0.14978 NA FALSE FALSE FALSE GCST006061 0.0118 0.8564 537409 outcome TRUE reported 11254_13_PAPLN_PPN PAPLN chr6 31354106 0.011025 2.993e-08 35431 TRUE reported textfile 2 1 TRUE
4629 rs10922098 T C T C -0.0948 -0.009 0.40011 NA FALSE FALSE FALSE GCST006061 0.0075 0.2275 537409 outcome TRUE reported 11254_13_PAPLN_PPN PAPLN chr1 196695521 0.008073 7.734e-32 35329 TRUE reported textfile 2 1 TRUE
4630 rs1355538 G A G A -0.0575 -0.0076 0.39847 NA FALSE FALSE FALSE GCST006061 0.0072 0.2923 537409 outcome TRUE reported 11254_13_PAPLN_PPN PAPLN chr3 165787389 0.008066 1.016e-12 35329 TRUE reported textfile 2 1 TRUE
4631 rs6542680 T C T C -0.0572 -0.0012 0.18362 NA FALSE FALSE FALSE GCST006061 0.0091 0.8935 537409 outcome TRUE reported 11254_13_PAPLN_PPN PAPLN chr2 3592552 0.010243 2.349e-08 35323 TRUE reported textfile 2 1 TRUE
4632 rs1056429 A G A G 0.0616 0.0021 0.14978 NA FALSE FALSE FALSE GCST006061 0.0118 0.8564 537409 outcome TRUE reported 7189_55_CHST3_CHST3 CHST3 chr6 31354106 0.011161 3.408e-08 35440 TRUE reported textfile 2 1 TRUE
4633 rs6445035 A G A G 0.0786 0.0154 0.1904 NA FALSE FALSE FALSE GCST006061 0.0089 0.08256 537409 outcome TRUE reported 7189_55_CHST3_CHST3 CHST3 chr3 165762312 0.010178 1.141e-14 35339 TRUE reported textfile 2 1 TRUE
4634 rs1056522 A G A G 0.132 -0.001 0.31069 NA FALSE FALSE FALSE GCST006061 0.0077 0.8922 537409 outcome TRUE reported 7955_195_ITIH1_ITI_heavy_chain_H1 ITIH1 chr3 126542502 0.00855 9.051e-54 35371 TRUE reported textfile 2 1 TRUE
4635 rs112727502 A G A G -0.1737 -2.00E-04 0.05405 NA FALSE FALSE FALSE

GCST006061 0.015 0.9906 537409 outcome TRUE reported
7955_195_ITIH1_ITI_heavy_chain_H1 ITIH1 chr10 7673597 0.017236 6.95e-24
35357 TRUE reported textfile 2 1 TRUE
4636 rs114419941 T C T C 0.1286 0.0108 0.04625 NA FALSE FALSE FALSE
GCST006061 0.0223 0.628 537409 outcome TRUE reported
7955_195_ITIH1_ITI_heavy_chain_H1 ITIH1 chr10 8021363 0.018185 1.53e-12
35359 TRUE reported textfile 2 1 TRUE
4637 rs1260326 C T C T -0.052 -0.0069 0.34132 NA FALSE FALSE FALSE
GCST006061 0.0073 0.3401 537409 outcome TRUE reported
7955_195_ITIH1_ITI_heavy_chain_H1 ITIH1 chr2 27508073 0.00817 1.957e-10
35373 TRUE reported textfile 2 1 TRUE
4638 rs187630010 T C T C 0.1688 0.005 0.02231 NA FALSE FALSE FALSE
GCST006061 0.0406 0.9017 537409 outcome TRUE reported
7955_195_ITIH1_ITI_heavy_chain_H1 ITIH1 chr3 47484471 0.026893 3.456e-10
35342 TRUE reported textfile 2 1 TRUE
4639 rs2286276 T C T C -0.0526 -0.0099 0.30229 NA FALSE FALSE FALSE
GCST006061 0.008 0.2152 537409 outcome TRUE reported
7955_195_ITIH1_ITI_heavy_chain_H1 ITIH1 chr7 73573024 0.008359 3.127e-10
35354 TRUE reported textfile 2 1 TRUE
4640 rs35703936 T C T C -0.2657 0.0022 0.02299 NA FALSE FALSE FALSE
GCST006061 0.023 0.9223 537409 outcome TRUE reported
7955_195_ITIH1_ITI_heavy_chain_H1 ITIH1 chr3 48344067 0.026121 2.643e-24
35344 TRUE reported textfile 2 1 TRUE
4641 rs57031794 A G A G 0.1421 0.018 0.02532 NA FALSE FALSE FALSE
GCST006061 0.0209 0.3887 537409 outcome TRUE reported
7955_195_ITIH1_ITI_heavy_chain_H1 ITIH1 chr10 7079140 0.023992 3.163e-09
35354 TRUE reported textfile 2 1 TRUE
4642 rs62251870 A G A G -0.118 -0.0079 0.2568 NA FALSE FALSE FALSE
GCST006061 0.0085 0.3532 537409 outcome TRUE reported
7955_195_ITIH1_ITI_heavy_chain_H1 ITIH1 chr3 53556451 0.009048 7.154e-39
35350 TRUE reported textfile 2 1 TRUE
4643 rs77819425 A G A G -0.2087 0.0313 0.01881 NA FALSE FALSE FALSE
GCST006061 0.0369 0.3961 537409 outcome TRUE reported
7955_195_ITIH1_ITI_heavy_chain_H1 ITIH1 chr3 46687701 0.029065 6.947e-13
35343 TRUE reported textfile 2 1 TRUE
4644 rs77938199 G A G A 0.3325 -0.0182 0.06588 NA FALSE FALSE FALSE
GCST006061 0.0152 0.2317 537409 outcome TRUE reported
7955_195_ITIH1_ITI_heavy_chain_H1 ITIH1 chr10 7700504 0.015609 1.099e-100
35361 TRUE reported textfile 2 1 TRUE
4645 rs7970695 A G A G 0.05 0.0118 0.35262 NA FALSE FALSE FALSE
GCST006061 0.0072 0.1015 537409 outcome TRUE reported
7955_195_ITIH1_ITI_heavy_chain_H1 ITIH1 chr12 120985573 0.008065 5.666e-10
35365 TRUE reported textfile 2 1 TRUE
4646 rs1056522 A G A G 0.0703 -0.001 0.31069 NA FALSE FALSE FALSE
GCST006061 0.0077 0.8922 537409 outcome TRUE reported
9326_33_ITIH2_ITI_heavy_chain_H2 ITIH2 chr3 126542502 0.00878 1.173e-15
35371 TRUE reported textfile 2 1 TRUE
4647 rs10795548 T C T C 0.0997 0.0055 0.17157 NA FALSE FALSE FALSE
GCST006061 0.0099 0.5747 537409 outcome TRUE reported
9326_33_ITIH2_ITI_heavy_chain_H2 ITIH2 chr10 7506147 0.01109 2.468e-19 35354
TRUE reported textfile 2 1 TRUE
4648 rs10905252 A G A G 0.108 -0.0017 0.41073 NA FALSE FALSE FALSE
GCST006061 0.0073 0.8179 537409 outcome TRUE reported
9326_33_ITIH2_ITI_heavy_chain_H2 ITIH2 chr10 7922254 0.008483 3.972e-37
35363 TRUE reported textfile 2 1 TRUE
4649 rs112725054 A C A C 0.1416 -0.0408 0.03057 NA FALSE FALSE FALSE
GCST006061 0.0302 0.1768 537409 outcome TRUE reported
9326_33_ITIH2_ITI_heavy_chain_H2 ITIH2 chr12 103499150 0.023629 2.066e-09
35367 TRUE reported textfile 2 1 TRUE
4650 rs12413578 T C T C 0.0891 0.0014 0.10019 NA FALSE FALSE FALSE
GCST006061 0.0126 0.9128 537409 outcome TRUE reported
9326_33_ITIH2_ITI_heavy_chain_H2 ITIH2 chr10 9007290 0.013932 1.6e-10 35359
TRUE reported textfile 2 1 TRUE
4651 rs190284 A G A G -0.0664 0.0053 0.45529 NA FALSE FALSE FALSE
GCST006061 0.0072 0.461 537409 outcome TRUE reported
9326_33_ITIH2_ITI_heavy_chain_H2 ITIH2 chr5 129894231 0.00814 3.439e-16

35352 TRUE reported textfile 2 1 TRUE
4652 rs2787154 G A G A 0.0829 6e-04 0.35863 NA FALSE FALSE FALSE
GCST006061 0.0076 0.9404 537409 outcome TRUE reported
9326_33_ITIH2_ITI_heavy_chain_H2 ITIH2 chr10 8368291 0.008719 1.949e-21
35355 TRUE reported textfile 2 1 TRUE
4653 rs7342066 G A G A -0.1746 0.0013 0.27647 NA FALSE FALSE FALSE
GCST006061 0.0078 0.8675 537409 outcome TRUE reported
9326_33_ITIH2_ITI_heavy_chain_H2 ITIH2 chr10 7753948 0.00932 2.657e-78 35363
TRUE reported textfile 2 1 TRUE
4654 rs78189039 A G A G 0.4762 -3.00E-04 0.08228 NA FALSE FALSE FALSE
GCST006061 0.0137 0.9804 537409 outcome TRUE reported
9326_33_ITIH2_ITI_heavy_chain_H2 ITIH2 chr10 7685924 0.015174 1e-200 35358
TRUE reported textfile 2 1 TRUE
4655 rs831428 T C T C 0.0501 -0.0037 0.30451 NA FALSE FALSE FALSE
GCST006061 0.0084 0.661 537409 outcome TRUE reported
9326_33_ITIH2_ITI_heavy_chain_H2 ITIH2 chr12 103667747 0.008787 1.186e-08
35364 TRUE reported textfile 2 1 TRUE
4656 rs1056692 G A G A -0.0493 -0.0127 0.3169 NA FALSE FALSE FALSE
GCST006061 0.0079 0.109 537409 outcome TRUE reported 11140_56_COL1A1_CO1A1
COL1A1 chr12 53293603 0.008925 3.318e-08 35365 TRUE reported
textfile 2 1 TRUE
4657 rs10955915 G A G A 0.0543 -9.00E-04 0.48 NA FALSE FALSE FALSE
GCST006061 0.0072 0.9056 537409 outcome TRUE reported 11140_56_COL1A1_CO1A1
COL1A1 chr8 118951308 0.008327 6.995e-11 35352 TRUE reported
textfile 2 1 TRUE
4658 rs116585561 C A C A -0.328 0.0057 0.03327 NA FALSE FALSE FALSE
GCST006061 0.025 0.8199 537409 outcome TRUE reported 11140_56_COL1A1_CO1A1
COL1A1 chr17 50180556 0.023063 6.715e-46 35355 TRUE reported
textfile 2 1 TRUE
4659 rs11726170 T C T C 0.0729 -0.0236 0.13062 NA FALSE FALSE FALSE
GCST006061 0.0124 0.05613 537409 outcome TRUE reported 11140_56_COL1A1_CO1A1
COL1A1 chr4 38391654 0.012273 2.85e-09 35354 TRUE reported
textfile 2 1 TRUE
4660 rs118081843 C A C A -0.3212 -0.0296 0.01174 NA FALSE FALSE FALSE
GCST006061 0.033 0.3699 537409 outcome TRUE reported 11140_56_COL1A1_CO1A1
COL1A1 chr17 50335804 0.040959 4.435e-15 35356 TRUE reported
textfile 2 1 TRUE
4661 rs138103546 G A G A -0.237 0.0735 0.01993 NA FALSE FALSE FALSE
GCST006061 0.035 0.03604 537409 outcome TRUE reported 11140_56_COL1A1_CO1A1
COL1A1 chr17 49894179 0.030488 7.625e-15 35356 TRUE reported
textfile 2 1 TRUE
4662 rs139072136 A C A C -0.2568 0.0142 0.01199 NA FALSE FALSE FALSE
GCST006061 0.0355 0.6884 537409 outcome TRUE reported 11140_56_COL1A1_CO1A1
COL1A1 chr17 44298644 0.037584 8.329e-12 35360 TRUE reported
textfile 2 1 TRUE
4663 rs145495890 T C T C -0.1938 0.0011 0.03969 NA FALSE FALSE FALSE
GCST006061 0.0207 0.9582 537409 outcome TRUE reported 11140_56_COL1A1_CO1A1
COL1A1 chr17 62499184 0.021627 3.215e-19 35359 TRUE reported
textfile 2 1 TRUE
4664 rs146385050 A C A C 0.248 0.0166 0.20263 NA FALSE FALSE FALSE
GCST006061 0.0106 0.1184 537409 outcome TRUE reported 11140_56_COL1A1_CO1A1
COL1A1 chr17 62559897 0.010457 2.437e-124 35359 TRUE reported
textfile 2 1 TRUE
4665 rs149631508 C T C T 0.2184 -0.0682 0.01743 NA FALSE FALSE FALSE
GCST006061 0.0477 0.1526 537409 outcome TRUE reported 11140_56_COL1A1_CO1A1
COL1A1 chr17 62782860 0.032284 1.334e-11 35359 TRUE reported
textfile 2 1 TRUE
4666 rs2663343 A G A G 0.0601 -0.0172 0.31972 NA FALSE FALSE FALSE
GCST006061 0.0085 0.04342 537409 outcome TRUE reported 11140_56_COL1A1_CO1A1
COL1A1 chr17 1023500 0.009033 2.868e-11 35337 TRUE reported textfile
2 1 TRUE
4667 rs6679677 A C A C -0.0745 -0.0086 0.13318 NA FALSE FALSE FALSE
GCST006061 0.0124 0.487 537409 outcome TRUE reported 11140_56_COL1A1_CO1A1
COL1A1 chr1 113761186 0.012308 1.423e-09 35341 TRUE reported
textfile 2 1 TRUE
4668 rs74485878 T C T C -0.2494 0.0512 0.01629 NA FALSE FALSE FALSE

GCST006061 0.0437 0.2414 537409 outcome TRUE reported 11140_56_COL1A1_CO1A1
COL1A1 chr17 51855685 0.033849 1.732e-13 35353 TRUE reported
textfile 2 1 TRUE
4669 rs9594738 T C T C 0.1011 -3.00E-04 0.4356 NA FALSE FALSE FALSE
GCST006061 0.0073 0.9701 537409 outcome TRUE reported 11140_56_COL1A1_CO1A1
COL1A1 chr13 42378009 0.008404 2.478e-33 35352 TRUE reported
textfile 2 1 TRUE
4670 rs174619 G A G A -0.081 -0.0053 0.47168 NA FALSE FALSE FALSE
GCST006061 0.0073 0.4677 537409 outcome TRUE reported 7127_3_APOA2_Apo_A_II
APOA2 chr11 61862194 0.008264 1.105e-22 35367 TRUE reported
textfile 2 1 TRUE
4671 rs185241069 T C T C -0.0952 -0.0097 0.06276 NA FALSE FALSE FALSE
GCST006061 0.0198 0.6249 537409 outcome TRUE reported 7127_3_APOA2_Apo_A_II
APOA2 chr11 63417740 0.017333 3.965e-08 35364 TRUE reported
textfile 2 1 TRUE
4672 rs2509962 C T C T -0.2983 4e-04 0.44185 NA FALSE FALSE FALSE
GCST006061 0.0076 0.9596 537409 outcome TRUE reported 7127_3_APOA2_Apo_A_II
APOA2 chr11 62542315 0.008243 1e-200 35361 TRUE reported textfile
2 1 TRUE
4673 rs61732279 C T C T -0.0733 0.0116 0.23427 NA FALSE FALSE FALSE
GCST006061 0.0087 0.1862 537409 outcome TRUE reported 7127_3_APOA2_Apo_A_II
APOA2 chr2 70985275 0.009694 3.985e-14 35351 TRUE reported
textfile 2 1 TRUE
4674 rs9630213 T C T C -0.161 0.0167 0.02871 NA FALSE FALSE FALSE
GCST006061 0.0199 0.4011 537409 outcome TRUE reported 7127_3_APOA2_Apo_A_II
APOA2 chr11 60819015 0.02511 1.438e-10 35370 TRUE reported textfile
2 1 TRUE
4675 rs1059367 T C T C -0.1255 0.0195 0.07965 NA FALSE FALSE FALSE
GCST006061 0.013 0.1334 537409 outcome TRUE reported
8894_80_HNRNPAB_hnRNP_A_B HNRNPAB chr5 178209198 0.014933 4.318e-17 35360
TRUE reported textfile 2 1 TRUE
4676 rs1354034 C T C T 0.092 0.0088 0.38119 NA FALSE FALSE FALSE
GCST006061 0.0073 0.2248 537409 outcome TRUE reported
8894_80_HNRNPAB_hnRNP_A_B HNRNPAB chr3 56815721 0.00822 4.469e-29 35356
TRUE reported textfile 2 1 TRUE
4677 rs1580881 C T C T -0.0462 -0.008 0.4513 NA FALSE FALSE FALSE
GCST006061 0.0071 0.2609 537409 outcome TRUE reported
8894_80_HNRNPAB_hnRNP_A_B HNRNPAB chr9 83027397 0.008036 8.982e-09 35366
TRUE reported textfile 2 1 TRUE
4678 rs2856354 G A G A -0.1775 0.0036 0.01869 NA FALSE FALSE FALSE
GCST006061 0.0179 0.839 537409 outcome TRUE reported
8894_80_HNRNPAB_hnRNP_A_B HNRNPAB chr5 178978728 0.029099 1.062e-09 35343
TRUE reported textfile 2 1 TRUE
4679 rs148025035 C T C T 0.2107 -0.0105 0.02326 NA FALSE FALSE FALSE
GCST006061 0.0489 0.83 537409 outcome TRUE reported 4874_3_ANG_Angiogenin
ANG chr14 20487214 0.027591 2.231e-14 35326 TRUE reported textfile
2 1 TRUE
4680 rs150844304 C A C A -0.1723 0.0439 0.0276 NA FALSE FALSE FALSE
GCST006061 0.0244 0.07203 537409 outcome TRUE reported 4874_3_ANG_Angiogenin
ANG chr15 43434427 0.024669 2.859e-12 35363 TRUE reported textfile
2 1 TRUE
4681 rs645040 T G T G 0.0698 -0.0287 0.22729 NA FALSE FALSE FALSE
GCST006061 0.0085 0.0007847 537409 outcome TRUE reported
4874_3_ANG_Angiogenin ANG chr3 136207780 0.009715 6.742e-13 35361 TRUE
reported textfile 2 1 TRUE
4682 rs71410230 A G A G -0.221 -0.0163 0.02429 NA FALSE FALSE FALSE
GCST006061 0.0206 0.4296 537409 outcome TRUE reported 4874_3_ANG_Angiogenin
ANG chr14 20375572 0.027882 2.258e-15 35339 TRUE reported textfile
2 1 TRUE
4683 rs72659937 T G T G -0.3058 0.0013 0.11138 NA FALSE FALSE FALSE
GCST006061 0.0152 0.9318 537409 outcome TRUE reported 4874_3_ANG_Angiogenin
ANG chr14 20642526 0.013374 1.013e-115 35330 TRUE reported textfile
2 1 TRUE
4684 rs1063178 T C T C -0.1535 0.023 0.29705 NA FALSE FALSE FALSE
GCST006061 0.0074 0.002015 537409 outcome TRUE reported
15583_18_FCRLB_FCRLB FCRLB chr1 161751917 0.008804 4.525e-68 35361

TRUE reported textfile 2 1 TRUE
4685 rs113810201 G A G A 0.0813 -0.0255 0.14955 NA FALSE FALSE FALSE
GCST006061 0.0117 0.02922 537409 outcome TRUE reported 15583_18_FCRLB_FCRLB
FCRLB chr7 151229168 0.011219 4.268e-13 35374 TRUE reported
textfile 2 1 TRUE
4686 rs12121649 A G A G 0.1323 -0.0062 0.20853 NA FALSE FALSE FALSE
GCST006061 0.01 0.5316 537409 outcome TRUE reported 15583_18_FCRLB_FCRLB
FCRLB chr1 160958481 0.009963 3.077e-40 35363 TRUE reported
textfile 2 1 TRUE
4687 rs1801274 G A G A -0.0866 0.0165 0.4405 NA FALSE FALSE FALSE
GCST006061 0.0072 0.02201 537409 outcome TRUE reported 15583_18_FCRLB_FCRLB
FCRLB chr1 161509955 0.008171 3.016e-26 35351 TRUE reported
textfile 2 1 TRUE
4688 rs1954172 C T C T 0.4512 -0.0204 0.13687 NA FALSE FALSE FALSE
GCST006061 0.0104 0.04927 537409 outcome TRUE reported 15583_18_FCRLB_FCRLB
FCRLB chr1 161707945 0.01016 1e-200 35357 TRUE reported textfile 2
1 TRUE
4689 rs73021687 C A C A 0.2542 -0.0148 0.01696 NA FALSE FALSE FALSE
GCST006061 0.0272 0.5869 537409 outcome TRUE reported 15583_18_FCRLB_FCRLB
FCRLB chr1 160044847 0.030729 1.315e-16 35358 TRUE reported
textfile 2 1 TRUE
4690 rs10655 C T C T 0.2264 -0.0752 0.0167 NA FALSE FALSE FALSE GCST006061
0.0428 0.07857 537409 outcome TRUE reported 6472_40_PMEL_GP100 PMEL chr12
53543084 0.03234 2.549e-12 35374 TRUE reported textfile 2 1 TRUE
4691 rs11076664 G A G A 0.2008 0.0038 0.24398 NA FALSE FALSE FALSE
GCST006061 0.0097 0.6978 537409 outcome TRUE reported 6472_40_PMEL_GP100
PMEL chr16 90056154 0.009611 6.373e-97 35316 TRUE reported
textfile 2 1 TRUE
4692 rs1126809 A G A G 0.0619 -0.0039 0.30155 NA FALSE FALSE FALSE
GCST006061 0.0083 0.6394 537409 outcome TRUE reported 6472_40_PMEL_GP100
PMEL chr11 89284793 0.008969 5.148e-12 35345 TRUE reported
textfile 2 1 TRUE
4693 rs11668064 G A G A 0.0522 0.019 0.25319 NA FALSE FALSE FALSE
GCST006061 0.0081 0.01855 537409 outcome TRUE reported 6472_40_PMEL_GP100
PMEL chr19 1169067 0.009456 3.389e-08 35313 TRUE reported textfile
2 1 TRUE
4694 rs117204628 T C T C -0.234 0.0125 0.03029 NA FALSE FALSE FALSE
GCST006061 0.027 0.6429 537409 outcome TRUE reported 6472_40_PMEL_GP100
PMEL chr16 89966047 0.024294 5.865e-22 35324 TRUE reported
textfile 2 1 TRUE
4695 rs1204371 C T C T 0.096 -0.0163 0.25088 NA FALSE FALSE FALSE
GCST006061 0.0081 0.04302 537409 outcome TRUE reported 6472_40_PMEL_GP100
PMEL chr7 105779823 0.009469 3.74e-24 35355 TRUE reported
textfile 2 1 TRUE
4696 rs1205312 G A G A 0.1353 0.0023 0.08571 NA FALSE FALSE FALSE
GCST006061 0.0148 0.876 537409 outcome TRUE reported 6472_40_PMEL_GP100
PMEL chr20 34261610 0.014755 4.737e-20 35366 TRUE reported
textfile 2 1 TRUE
4697 rs12350739 A G A G -0.1334 0.0308 0.39544 NA FALSE FALSE FALSE
GCST006061 0.0076 5.192e-05 537409 outcome TRUE reported 6472_40_PMEL_GP100
PMEL chr9 16885019 0.008304 4.461e-58 35364 TRUE reported
textfile 2 1 TRUE
4698 rs12447797 T C T C -0.1121 0.0116 0.07006 NA FALSE FALSE FALSE
GCST006061 0.0157 0.4617 537409 outcome TRUE reported 6472_40_PMEL_GP100
PMEL chr16 88924548 0.015944 2.05e-12 35320 TRUE reported
textfile 2 1 TRUE
4699 rs140367586 G T G T -0.2086 0.0171 0.02536 NA FALSE FALSE FALSE
GCST006061 0.0324 0.597 537409 outcome TRUE reported 6472_40_PMEL_GP100
PMEL chr7 100168166 0.026333 2.346e-15 35368 TRUE reported
textfile 2 1 TRUE
4700 rs183671 G T G T 0.289 -0.0391 0.01167 NA FALSE FALSE FALSE
GCST006061 0.0194 0.0442 537409 outcome TRUE reported 6472_40_PMEL_GP100
PMEL chr5 33964105 0.03874 8.647e-14 35379 TRUE reported textfile
2 1 TRUE
4701 rs2069398 A G A G 0.416 -0.0258 0.09396 NA FALSE FALSE FALSE
GCST006061 0.0136 0.05712 537409 outcome TRUE reported 6472_40_PMEL_GP100

PMEL chr12 55967092 0.014119 8.192e-191 35366 TRUE reported textfile 2 1 TRUE

4702 rs2120470 G T G T -0.0999 0.0028 0.35797 NA FALSE FALSE FALSE GCST006061 0.0074 0.7036 537409 outcome TRUE reported 6472_40_PMEL_GP100 PMEL chr8 71951203 0.008604 3.648e-31 35348 TRUE reported textfile 2 1 TRUE

4703 rs2550671 G T G T 0.064 0.0038 0.15422 NA FALSE FALSE FALSE GCST006061 0.0099 0.7015 537409 outcome TRUE reported 6472_40_PMEL_GP100 PMEL chr12 55636308 0.01131 1.524e-08 35381 TRUE reported textfile 2 1 TRUE

4704 rs34255600 C A C A -0.0511 0.0088 0.43402 NA FALSE FALSE FALSE GCST006061 0.0075 0.2407 537409 outcome TRUE reported 6472_40_PMEL_GP100 PMEL chr3 69851369 0.008262 6.223e-10 35360 TRUE reported textfile 2 1 TRUE

4705 rs3829241 A G A G 0.046 0.0086 0.43474 NA FALSE FALSE FALSE GCST006061 0.0073 0.2407 537409 outcome TRUE reported 6472_40_PMEL_GP100 PMEL chr11 69087895 0.00824 2.367e-08 35366 TRUE reported textfile 2 1 TRUE

4706 rs6873160 G A G A 0.0549 -0.0141 0.42147 NA FALSE FALSE FALSE GCST006061 0.0074 0.05507 537409 outcome TRUE reported 6472_40_PMEL_GP100 PMEL chr5 109775131 0.008353 4.947e-11 35351 TRUE reported textfile 2 1 TRUE

4707 rs849134 G A G A -0.0578 0.0016 0.46615 NA FALSE FALSE FALSE GCST006061 0.0072 0.8218 537409 outcome TRUE reported 6472_40_PMEL_GP100 PMEL chr7 28156603 0.008248 2.423e-12 35374 TRUE reported textfile 2 1 TRUE

4708 rs9297881 A G A G 0.0503 -0.0078 0.29397 NA FALSE FALSE FALSE GCST006061 0.0077 0.3102 537409 outcome TRUE reported 6472_40_PMEL_GP100 PMEL chr8 134274746 0.00901 2.371e-08 35372 TRUE reported textfile 2 1 TRUE

4709 rs1071960 C T C T -0.0499 0.0045 0.29144 NA FALSE FALSE FALSE GCST006061 0.0081 0.5763 537409 outcome TRUE reported 10627_87_APLP2_APLP2 APLP2 chr22 42953626 0.008958 2.545e-08 35362 TRUE reported textfile 2 1 TRUE

4710 rs10822155 A C A C 0.0596 -0.0472 0.43479 NA FALSE FALSE FALSE GCST006061 0.0072 4.622e-11 537409 outcome TRUE reported 10627_87_APLP2_APLP2 APLP2 chr10 63311455 0.008207 3.814e-13 35339 TRUE reported textfile 2 1 TRUE

4711 rs73583419 A C A C 0.2786 6e-04 0.06312 NA FALSE FALSE FALSE GCST006061 0.0148 0.9657 537409 outcome TRUE reported 10627_87_APLP2_APLP2 APLP2 chr11 130069495 0.016588 2.64e-63 35359 TRUE reported textfile 2 1 TRUE

4712 rs741736 T C T C 0.0548 -0.004 0.26382 NA FALSE FALSE FALSE GCST006061 0.0088 0.6529 537409 outcome TRUE reported 10627_87_APLP2_APLP2 APLP2 chr11 256978 0.009228 2.873e-09 35340 TRUE reported textfile 2 1 TRUE

4713 rs775824 C T C T 0.061 0.0102 0.16338 NA FALSE FALSE FALSE GCST006061 0.0101 0.3143 537409 outcome TRUE reported 10627_87_APLP2_APLP2 APLP2 chr19 55050202 0.010984 2.796e-08 35369 TRUE reported textfile 2 1 TRUE

4714 rs1071960 C T C T -0.0514 0.0045 0.29144 NA FALSE FALSE FALSE GCST006061 0.0081 0.5763 537409 outcome TRUE reported 2697_7_PF4_PF_4 PF4 chr22 42953626 0.008976 1.025e-08 35374 TRUE reported textfile 2 1 TRUE

4715 rs10822155 A C A C 0.1044 -0.0472 0.43479 NA FALSE FALSE FALSE GCST006061 0.0072 4.622e-11 537409 outcome TRUE reported 2697_7_PF4_PF_4 PF4 chr10 63311455 0.008214 5.239e-37 35351 TRUE reported textfile 2 1 TRUE

4716 rs11574452 A C A C -0.2194 0.0147 0.02727 NA FALSE FALSE FALSE GCST006061 0.0173 0.3972 537409 outcome TRUE reported 2697_7_PF4_PF_4 PF4 chr4 73980944 0.025682 1.307e-17 35351 TRUE reported textfile 2 1 TRUE

4717 rs1398015 T C T C -0.1518 0.0026 0.05914 NA FALSE FALSE FALSE GCST006061 0.0215 0.9043 537409 outcome TRUE reported 2697_7_PF4_PF_4 PF4 chr4 73891519 0.017459 3.478e-18 35349 TRUE reported textfile 2 1 TRUE

4718 rs1071960 C T C T -0.0489 0.0045 0.29144 NA FALSE FALSE FALSE GCST006061 0.0081 0.5763 537409 outcome TRUE reported 3171_57_APP_amyloid_precursor_protein APP chr22 42953626 0.008969 4.974e-08 35374 TRUE reported textfile 2 1 TRUE

4719 rs10822155 A C A C 0.0759 -0.0472 0.43479 NA FALSE FALSE FALSE GCST006061 0.0072 4.622e-11 537409 outcome TRUE reported 3171_57_APP_amyloid_precursor_protein APP chr10 63311455 0.008212 2.394e-20 35351 TRUE reported textfile 2 1 TRUE

4720 rs2229498 A G A G 0.1643 0.0047 0.16514 NA FALSE FALSE FALSE GCST006061 0.0092 0.6113 537409 outcome TRUE reported 3171_57_APP_amyloid_precursor_protein APP chr10 69097096 0.011013 2.511e-50 35362 TRUE reported textfile 2 1 TRUE

4721 rs439826 G T G T -0.0779 0.0138 0.31709 NA FALSE FALSE FALSE GCST006061 0.0075 0.06656 537409 outcome TRUE reported 3171_57_APP_amyloid_precursor_protein APP chr21 26156328 0.008786 7.566e-19 35366 TRUE reported textfile 2 1 TRUE

4722 rs1071960 C T C T -0.0498 0.0045 0.29144 NA FALSE FALSE FALSE GCST006061 0.0081 0.5763 537409 outcome TRUE reported 9835_16_ALDH1A3_AL1A3 ALDH1A3 chr22 42953626 0.00911 4.582e-08 35374 TRUE reported textfile 2 1 TRUE

4723 rs368465 C T C T -0.0583 -0.012 0.39961 NA FALSE FALSE FALSE GCST006061 0.0075 0.1119 537409 outcome TRUE reported 9835_16_ALDH1A3_AL1A3 ALDH1A3 chr1 196702851 0.008456 5.403e-12 35339 TRUE reported textfile 2 1 TRUE

4724 rs610077 A G A G 0.0534 -0.0059 0.30363 NA FALSE FALSE FALSE GCST006061 0.0081 0.4637 537409 outcome TRUE reported 9835_16_ALDH1A3_AL1A3 ALDH1A3 chr2 31235324 0.009036 3.426e-09 35367 TRUE reported textfile 2 1 TRUE

4725 rs7080386 A C A C 0.104 -0.0468 0.43502 NA FALSE FALSE FALSE GCST006061 0.0072 7.735e-11 537409 outcome TRUE reported 9835_16_ALDH1A3_AL1A3 ALDH1A3 chr10 63288546 0.008328 8.61e-36 35351 TRUE reported textfile 2 1 TRUE

4726 rs1071960 C T C T -0.05 0.0045 0.29144 NA FALSE FALSE FALSE GCST006061 0.0081 0.5763 537409 outcome TRUE reported 17140_57_PDGFD_PDGFD PDGFD chr22 42953626 0.008988 2.651e-08 35374 TRUE reported textfile 2 1 TRUE

4727 rs11225768 A G A G -0.1788 -0.0188 0.07153 NA FALSE FALSE FALSE GCST006061 0.0141 0.1811 537409 outcome TRUE reported 17140_57_PDGFD_PDGFD PDGFD chr11 103402370 0.01603 6.847e-29 35364 TRUE reported textfile 2 1 TRUE

4728 rs11226172 G A G A -0.3447 0.0024 0.23021 NA FALSE FALSE FALSE GCST006061 0.009 0.794 537409 outcome TRUE reported 17140_57_PDGFD_PDGFD PDGFD chr11 104114013 0.009646 1e-200 35365 TRUE reported textfile 2 1 TRUE

4729 rs12223045 T C T C 0.1521 -0.0081 0.11514 NA FALSE FALSE FALSE GCST006061 0.0105 0.436 537409 outcome TRUE reported 17140_57_PDGFD_PDGFD PDGFD chr11 103861349 0.013023 1.622e-31 35364 TRUE reported textfile 2 1 TRUE

4730 rs1475695 A G A G -0.0513 -0.0036 0.35373 NA FALSE FALSE FALSE GCST006061 0.0075 0.6311 537409 outcome TRUE reported 17140_57_PDGFD_PDGFD PDGFD chr9 97928886 0.008518 1.716e-09 35345 TRUE reported textfile 2 1 TRUE

4731 rs1542541 A G A G -0.0622 -0.001 0.17451 NA FALSE FALSE FALSE GCST006061 0.0095 0.9185 537409 outcome TRUE reported 17140_57_PDGFD_PDGFD PDGFD chr16 8932543 0.010808 8.653e-09 35378 TRUE reported textfile 2 1 TRUE

4732 rs1926231 C T C T 0.0469 -0.0014 0.4388 NA FALSE FALSE FALSE GCST006061 0.0074 0.8497 537409 outcome TRUE reported 17140_57_PDGFD_PDGFD PDGFD chr1 198634149 0.008256 1.342e-08 35343 TRUE reported textfile 2 1 TRUE

4733 rs2515082 C T C T 0.1622 0.009 0.27961 NA FALSE FALSE FALSE GCST006061 0.0087 0.3005 537409 outcome TRUE reported 17140_57_PDGFD_PDGFD PDGFD chr11 104097896 0.009187 9.368e-70 35365 TRUE reported textfile 2 1 TRUE

4734 rs561267 G A G A -0.0945 0.0185 0.10387 NA FALSE FALSE FALSE GCST006061 0.0114 0.1035 537409 outcome TRUE reported 17140_57_PDGFD_PDGFD

PDGFD chr11 100835049 0.013561 3.198e-12 35357 TRUE reported textfile 2 1 TRUE
4735 rs1071960 C T C T -0.053 0.0045 0.29144 NA FALSE FALSE FALSE GCST006061 0.0081 0.5763 537409 outcome TRUE reported 3474_19_THBS1_Thrombospondin_1 THBS1 chr22 42953626 0.009012 4.081e-09 35374 TRUE reported textfile 2 1 TRUE
4736 rs114694170 C T C T 0.0915 0.0053 0.08378 NA FALSE FALSE FALSE GCST006061 0.0175 0.7624 537409 outcome TRUE reported 3474_19_THBS1_Thrombospondin_1 THBS1 chr5 88884379 0.01486 7.398e-10 35344 TRUE reported textfile 2 1 TRUE
4737 rs35340377 A G A G -0.0544 0.0075 0.25094 NA FALSE FALSE FALSE GCST006061 0.0082 0.3585 537409 outcome TRUE reported 3474_19_THBS1_Thrombospondin_1 THBS1 chr1 247874908 0.009531 1.146e-08 35156 TRUE reported textfile 2 1 TRUE
4738 rs7080386 A C A C 0.0893 -0.0468 0.43502 NA FALSE FALSE FALSE GCST006061 0.0072 7.735e-11 537409 outcome TRUE reported 3474_19_THBS1_Thrombospondin_1 THBS1 chr10 63288546 0.008241 2.331e-27 35351 TRUE reported textfile 2 1 TRUE
4739 rs10733084 C T C T -0.0646 0.006 0.1337 NA FALSE FALSE FALSE GCST006061 0.0097 0.5319 537409 outcome TRUE reported 7104_71_CABP7_CABP7 CABP7 chr1 196181819 0.011719 3.544e-08 35337 TRUE reported textfile 2 1 TRUE
4740 rs10922098 T C T C -0.1386 -0.009 0.40011 NA FALSE FALSE FALSE GCST006061 0.0075 0.2275 537409 outcome TRUE reported 7104_71_CABP7_CABP7 CABP7 chr1 196695521 0.008156 9.242e-65 35338 TRUE reported textfile 2 1 TRUE
4741 rs1706435 G A G A 0.0674 0.0117 0.36981 NA FALSE FALSE FALSE GCST006061 0.0072 0.1047 537409 outcome TRUE reported 7104_71_CABP7_CABP7 CABP7 chr3 165743688 0.00828 3.937e-16 35339 TRUE reported textfile 2 1 TRUE
4742 rs483082 T G T G -0.0648 2e-04 0.23213 NA FALSE FALSE FALSE GCST006061 0.0088 0.9858 537409 outcome TRUE reported 7104_71_CABP7_CABP7 CABP7 chr19 44912921 0.009572 1.288e-11 35366 TRUE reported textfile 2 1 TRUE
4743 rs704 A G A G 0.1162 -0.0155 0.49189 NA FALSE FALSE FALSE GCST006061 0.0072 0.03144 537409 outcome TRUE reported 7104_71_CABP7_CABP7 CABP7 chr17 28367840 0.008007 1.021e-47 35360 TRUE reported textfile 2 1 TRUE
4744 rs872914 A G A G -0.0718 0.0019 0.28935 NA FALSE FALSE FALSE GCST006061 0.0079 0.8076 537409 outcome TRUE reported 7104_71_CABP7_CABP7 CABP7 chr4 73852384 0.008793 3.206e-16 35348 TRUE reported textfile 2 1 TRUE
4745 rs10733084 C T C T -0.0726 0.006 0.1337 NA FALSE FALSE FALSE GCST006061 0.0097 0.5319 537409 outcome TRUE reported 16309_30_DKKL1_Soggy_1 DKKL1 chr1 196181819 0.011682 5.148e-10 35337 TRUE reported textfile 2 1 TRUE
4746 rs111696008 A G A G -0.1539 -0.0259 0.02443 NA FALSE FALSE FALSE GCST006061 0.0401 0.519 537409 outcome TRUE reported 16309_30_DKKL1_Soggy_1 DKKL1 chr2 216622986 0.02644 5.86e-09 35359 TRUE reported textfile 2 1 TRUE
4747 rs1329424 G T G T -0.2501 -0.0095 0.39735 NA FALSE FALSE FALSE GCST006061 0.0075 0.2071 537409 outcome TRUE reported 16309_30_DKKL1_Soggy_1 DKKL1 chr1 196677046 0.008073 1e-200 35338 TRUE reported textfile 2 1 TRUE
4748 rs1555405 A G A G -0.0484 -0.0047 0.30381 NA FALSE FALSE FALSE GCST006061 0.0081 0.5592 537409 outcome TRUE reported 16309_30_DKKL1_Soggy_1 DKKL1 chr14 100710432 0.008697 2.624e-08 35380 TRUE reported textfile 2 1 TRUE
4749 rs2686395 C T C T -0.139 -0.0077 0.3101 NA FALSE FALSE FALSE GCST006061 0.0074 0.2965 537409 outcome TRUE reported 16309_30_DKKL1_Soggy_1 DKKL1 chr3 165767231 0.008566 3.273e-59 35339 TRUE reported textfile 2 1 TRUE
4750 rs606725 G A G A 0.0601 -0.0229 0.24228 NA FALSE FALSE FALSE GCST006061 0.0083 0.00588 537409 outcome TRUE reported 16309_30_DKKL1_Soggy_1 DKKL1 chr3 136267245 0.009344 1.258e-10 35361 TRUE reported textfile 2 1 TRUE
4751 rs704 A G A G 0.0573 -0.0155 0.49189 NA FALSE FALSE FALSE GCST006061

0.0072 0.03144 537409 outcome TRUE reported 16309_30_DKKL1_Soggy_1 DKKL1
chr17 28367840 0.007965 6.292e-13 35360 TRUE reported textfile 2
1 TRUE
4752 rs10733084 C T C T -0.0668 0.006 0.1337 NA FALSE FALSE FALSE
GCST006061 0.0097 0.5319 537409 outcome TRUE reported 3437_80_FLT3_Flt_3
FLT3 chr1 196181819 0.011792 1.471e-08 35337 TRUE reported
textfile 2 1 TRUE
4753 rs10922098 T C T C -0.2246 -0.009 0.40011 NA FALSE FALSE FALSE
GCST006061 0.0075 0.2275 537409 outcome TRUE reported 3437_80_FLT3_Flt_3
FLT3 chr1 196695521 0.008166 1.501e-166 35338 TRUE reported
textfile 2 1 TRUE
4754 rs1707652 T C T C 0.0895 0.0081 0.3102 NA FALSE FALSE FALSE
GCST006061 0.0074 0.2701 537409 outcome TRUE reported 3437_80_FLT3_Flt_3
FLT3 chr3 165761011 0.008673 5.729e-25 35339 TRUE reported
textfile 2 1 TRUE
4755 rs10733084 C T C T -0.0637 0.006 0.1337 NA FALSE FALSE FALSE
GCST006061 0.0097 0.5319 537409 outcome TRUE reported 19587_12_KPNA1_IMA5
KPNA1 chr1 196181819 0.011679 4.92e-08 35337 TRUE reported
textfile 2 1 TRUE
4756 rs11227136 A G A G -0.0536 -0.0129 0.25007 NA FALSE FALSE FALSE
GCST006061 0.0087 0.1386 537409 outcome TRUE reported 19587_12_KPNA1_IMA5
KPNA1 chr11 65164493 0.009219 6.097e-09 35363 TRUE reported
textfile 2 1 TRUE
4757 rs12403425 A C A C 0.0525 -0.0064 0.2662 NA FALSE FALSE FALSE
GCST006061 0.009 0.4754 537409 outcome TRUE reported 19587_12_KPNA1_IMA5
KPNA1 chr1 197845693 0.009003 5.5e-09 35337 TRUE reported textfile
2 1 TRUE
4758 rs1355538 G A G A -0.1062 -0.0076 0.39847 NA FALSE FALSE FALSE
GCST006061 0.0072 0.2923 537409 outcome TRUE reported 19587_12_KPNA1_IMA5
KPNA1 chr3 165787389 0.008119 4.241e-39 35338 TRUE reported
textfile 2 1 TRUE
4759 rs72952553 A G A G -0.0483 -0.0042 0.31481 NA FALSE FALSE FALSE
GCST006061 0.0085 0.6159 537409 outcome TRUE reported 19587_12_KPNA1_IMA5
KPNA1 chr2 216616793 0.008607 2.004e-08 35359 TRUE reported
textfile 2 1 TRUE
4760 rs73169117 T C T C 0.0742 0.0051 0.11405 NA FALSE FALSE FALSE
GCST006061 0.0122 0.6755 537409 outcome TRUE reported 19587_12_KPNA1_IMA5
KPNA1 chr3 167217620 0.01263 4.227e-09 35354 TRUE reported textfile
2 1 TRUE
4761 rs10734190 C T C T 0.186 0.0152 0.20096 NA FALSE FALSE FALSE
GCST006061 0.0105 0.1462 537409 outcome TRUE reported 3607_71_DKK3_DKK3
DKK3 chr11 11980040 0.010474 1.481e-70 35375 TRUE reported
textfile 2 1 TRUE
4762 rs11022114 A G A G 0.332 0.0094 0.33332 NA FALSE FALSE FALSE
GCST006061 0.008 0.2425 537409 outcome TRUE reported 3607_71_DKK3_DKK3
DKK3 chr11 12017327 0.006955 1e-200 35377 TRUE reported textfile
2 1 TRUE
4763 rs139804748 G A G A -0.1384 0.0278 0.03052 NA FALSE FALSE FALSE
GCST006061 0.037 0.4525 537409 outcome TRUE reported 3607_71_DKK3_DKK3
DKK3 chr11 13658756 0.024593 1.828e-08 35370 TRUE reported
textfile 2 1 TRUE
4764 rs1404421 T C T C -0.062 0.004 0.4813 NA FALSE FALSE FALSE
GCST006061 0.0072 0.5745 537409 outcome TRUE reported 3607_71_DKK3_DKK3
DKK3 chr7 17777807 0.008297 7.887e-14 35366 TRUE reported
textfile 2 1 TRUE
4765 rs17384316 T C T C -0.1328 0.0121 0.03225 NA FALSE FALSE FALSE
GCST006061 0.0199 0.5414 537409 outcome TRUE reported 3607_71_DKK3_DKK3
DKK3 chr11 12127248 0.023942 2.912e-08 35376 TRUE reported
textfile 2 1 TRUE
4766 rs17548461 G A G A 0.1134 0.0062 0.05445 NA FALSE FALSE FALSE
GCST006061 0.0182 0.7346 537409 outcome TRUE reported 3607_71_DKK3_DKK3
DKK3 chr12 103014498 0.018488 8.587e-10 35364 TRUE reported
textfile 2 1 TRUE
4767 rs188547348 G A G A 0.2306 0.0654 0.02666 NA FALSE FALSE FALSE
GCST006061 0.0352 0.06349 537409 outcome TRUE reported 3607_71_DKK3_DKK3
DKK3 chr12 104682338 0.026145 1.143e-18 35353 TRUE reported

textfile 2 1 TRUE
4768 rs4981022 A G A G 0.1152 -5.00E-04 0.3206 NA FALSE FALSE FALSE
GCST006061 0.0078 0.9508 537409 outcome TRUE reported 3607_71_DKK3_DKK3
DKK3 chr12 103756096 0.008915 3.361e-38 35361 TRUE reported
textfile 2 1 TRUE
4769 rs635634 C T C T 0.1044 0.0285 0.12995 NA FALSE FALSE FALSE
GCST006061 0.0089 0.001351 537409 outcome TRUE reported 3607_71_DKK3_DKK3
DKK3 chr9 133279427 0.012349 2.81e-17 35371 TRUE reported
textfile 2 1 TRUE
4770 rs78528738 A G A G 0.1749 0.005 0.03277 NA FALSE FALSE FALSE
GCST006061 0.0244 0.8367 537409 outcome TRUE reported 3607_71_DKK3_DKK3
DKK3 chr11 11681859 0.023664 1.459e-13 35376 TRUE reported
textfile 2 1 TRUE
4771 rs79435523 G T G T 0.1279 0.0134 0.04681 NA FALSE FALSE FALSE
GCST006061 0.0174 0.441 537409 outcome TRUE reported 3607_71_DKK3_DKK3
DKK3 chr12 102948871 0.019403 4.342e-11 35363 TRUE reported
textfile 2 1 TRUE
4772 rs10734844 G A G A -0.1367 0.0043 0.03595 NA FALSE FALSE FALSE
GCST006061 0.0175 0.8046 537409 outcome TRUE reported 12338_27_EGFLAM_EGFLA
EGFLAM chr12 7482442 0.022826 2.113e-09 35379 TRUE reported textfile
2 1 TRUE
4773 rs10838529 A G A G 0.0572 -0.0015 0.30862 NA FALSE FALSE FALSE
GCST006061 0.0083 0.8566 537409 outcome TRUE reported 12338_27_EGFLAM_EGFLA
EGFLAM chr11 45909951 0.009252 6.304e-10 35367 TRUE reported
textfile 2 1 TRUE
4774 rs12975366 C T C T 0.2587 -0.0089 0.4155 NA FALSE FALSE FALSE
GCST006061 0.0081 0.2688 537409 outcome TRUE reported 12338_27_EGFLAM_EGFLA
EGFLAM chr19 54255498 0.008587 2.065e-199 35361 TRUE reported
textfile 2 1 TRUE
4775 rs148393709 T C T C -0.1687 -0.1441 0.0193 NA FALSE FALSE FALSE
GCST006061 0.0677 0.03345 537409 outcome TRUE reported 12338_27_EGFLAM_EGFLA
EGFLAM chr1 202101770 0.030774 4.207e-08 35349 TRUE reported
textfile 2 1 TRUE
4776 rs17329885 C T C T 0.0685 -0.0145 0.14511 NA FALSE FALSE FALSE
GCST006061 0.0102 0.154 537409 outcome TRUE reported 12338_27_EGFLAM_EGFLA
EGFLAM chr12 21175631 0.012182 1.876e-08 35414 TRUE reported
textfile 2 1 TRUE
4777 rs2427312 T C T C 0.0616 0.0089 0.17377 NA FALSE FALSE FALSE
GCST006061 0.0095 0.3514 537409 outcome TRUE reported 12338_27_EGFLAM_EGFLA
EGFLAM chr20 62395535 0.0113 4.994e-08 35328 TRUE reported textfile
2 1 TRUE
4778 rs2589775 G A G A -0.1793 0.0103 0.18933 NA FALSE FALSE FALSE
GCST006061 0.0087 0.2369 537409 outcome TRUE reported 12338_27_EGFLAM_EGFLA
EGFLAM chr5 38381304 0.010953 3.101e-60 35364 TRUE reported
textfile 2 1 TRUE
4779 rs2740594 G A G A 0.0718 -0.0115 0.2579 NA FALSE FALSE FALSE
GCST006061 0.0086 0.1805 537409 outcome TRUE reported 12338_27_EGFLAM_EGFLA
EGFLAM chr8 11849665 0.009766 1.954e-13 35368 TRUE reported
textfile 2 1 TRUE
4780 rs620982 C T C T -0.075 0.0071 0.4641 NA FALSE FALSE FALSE
GCST006061 0.0071 0.318 537409 outcome TRUE reported 12338_27_EGFLAM_EGFLA
EGFLAM chr18 32223308 0.008526 1.409e-18 35341 TRUE reported
textfile 2 1 TRUE
4781 rs780094 C T C T 0.0496 -0.0076 0.33917 NA FALSE FALSE FALSE
GCST006061 0.0073 0.2951 537409 outcome TRUE reported 12338_27_EGFLAM_EGFLA
EGFLAM chr2 27518370 0.009009 3.685e-08 35364 TRUE reported
textfile 2 1 TRUE
4782 rs78804971 C T C T 0.1196 0.0114 0.06552 NA FALSE FALSE FALSE
GCST006061 0.0144 0.4298 537409 outcome TRUE reported 12338_27_EGFLAM_EGFLA
EGFLAM chr3 49784336 0.017107 2.726e-12 35335 TRUE reported
textfile 2 1 TRUE
4783 rs10737300 C T C T 0.0636 0.0069 0.17809 NA FALSE FALSE FALSE
GCST006061 0.0088 0.4332 537409 outcome TRUE reported 7157_22_PCDHA4_PCDA4
PCDHA4 chr1 173416047 0.011186 1.303e-08 35351 TRUE reported
textfile 2 1 TRUE
4784 rs10863663 G A G A 0.0526 -0.0047 0.29804 NA FALSE FALSE FALSE

GCST006061 0.008 0.561 537409 outcome TRUE reported 7157_22_PCDHA4_PCDA4
PCDHA4 chr1 221821183 0.009335 1.753e-08 35357 TRUE reported
textfile 2 1 TRUE
4785 rs11039197 G A G A 0.0695 -0.0217 0.15153 NA FALSE FALSE FALSE
GCST006061 0.0128 0.09075 537409 outcome TRUE reported 7157_22_PCDHA4_PCDA4
PCDHA4 chr11 47356848 0.011775 3.583e-09 35377 TRUE reported
textfile 2 1 TRUE
4786 rs1110701 G A G A -0.0606 0.0065 0.2602 NA FALSE FALSE FALSE
GCST006061 0.008 0.4178 537409 outcome TRUE reported 7157_22_PCDHA4_PCDA4
PCDHA4 chr7 50410929 0.009779 5.753e-10 35338 TRUE reported
textfile 2 1 TRUE
4787 rs112763553 A G A G -0.0915 0.0048 0.14835 NA FALSE FALSE FALSE
GCST006061 0.0199 0.8104 537409 outcome TRUE reported 7157_22_PCDHA4_PCDA4
PCDHA4 chr14 105709338 0.012439 1.899e-13 35099 TRUE reported
textfile 2 1 TRUE
4788 rs12989427 A G A G 0.0707 0.0273 0.16728 NA FALSE FALSE FALSE
GCST006061 0.0104 0.008464 537409 outcome TRUE reported
7157_22_PCDHA4_PCDA4 PCDHA4 chr2 60910371 0.011572 9.988e-10 35354
TRUE reported textfile 2 1 TRUE
4789 rs139199587 A G A G -0.1288 -0.0076 0.0708 NA FALSE FALSE FALSE
GCST006061 0.0184 0.6796 537409 outcome TRUE reported 7157_22_PCDHA4_PCDA4
PCDHA4 chr6 263937 0.017098 4.952e-14 35309 TRUE reported textfile
2 1 TRUE
4790 rs17578693 C T C T 0.0768 0.0085 0.11123 NA FALSE FALSE FALSE
GCST006061 0.0131 0.5155 537409 outcome TRUE reported 7157_22_PCDHA4_PCDA4
PCDHA4 chr1 234566161 0.013724 2.195e-08 35361 TRUE reported
textfile 2 1 TRUE
4791 rs1953209 G A G A -0.0648 -0.0116 0.30207 NA FALSE FALSE FALSE
GCST006061 0.0075 0.1244 537409 outcome TRUE reported 7157_22_PCDHA4_PCDA4
PCDHA4 chr14 29856487 0.00935 4.187e-12 35361 TRUE reported textfile
2 1 TRUE
4792 rs2295616 A G A G -0.0548 0.0018 0.33112 NA FALSE FALSE FALSE
GCST006061 0.0078 0.8148 537409 outcome TRUE reported 7157_22_PCDHA4_PCDA4
PCDHA4 chr1 160740367 0.009093 1.675e-09 35361 TRUE reported
textfile 2 1 TRUE
4793 rs247559 A C A C -0.0561 0.0053 0.34544 NA FALSE FALSE FALSE
GCST006061 0.0073 0.471 537409 outcome TRUE reported 7157_22_PCDHA4_PCDA4
PCDHA4 chr5 89415254 0.008997 4.507e-10 35342 TRUE reported
textfile 2 1 TRUE
4794 rs2511737 C A C A -0.0938 -0.0263 0.27692 NA FALSE FALSE FALSE
GCST006061 0.0084 0.001647 537409 outcome TRUE reported
7157_22_PCDHA4_PCDA4 PCDHA4 chr8 102568299 0.009601 1.523e-22 35360
TRUE reported textfile 2 1 TRUE
4795 rs3807306 T G T G 0.0472 -0.0051 0.49316 NA FALSE FALSE FALSE
GCST006061 0.0072 0.4743 537409 outcome TRUE reported 7157_22_PCDHA4_PCDA4
PCDHA4 chr7 128940626 0.008556 3.453e-08 35351 TRUE reported
textfile 2 1 TRUE
4796 rs3810560 C T C T -0.0653 -0.0033 0.43172 NA FALSE FALSE FALSE
GCST006061 0.0075 0.661 537409 outcome TRUE reported 7157_22_PCDHA4_PCDA4
PCDHA4 chr20 3227173 0.00866 4.671e-14 35378 TRUE reported textfile 2
1 TRUE
4797 rs614004 A G A G 0.0646 -0.0109 0.46989 NA FALSE FALSE FALSE
GCST006061 0.0071 0.121 537409 outcome TRUE reported 7157_22_PCDHA4_PCDA4
PCDHA4 chr3 32413589 0.008638 7.528e-14 35350 TRUE reported
textfile 2 1 TRUE
4798 rs6679677 A C A C -0.0966 -0.0086 0.13318 NA FALSE FALSE FALSE
GCST006061 0.0124 0.487 537409 outcome TRUE reported 7157_22_PCDHA4_PCDA4
PCDHA4 chr1 113761186 0.012707 2.919e-14 35350 TRUE reported
textfile 2 1 TRUE
4799 rs6845263 C T C T -0.0471 7e-04 0.47781 NA FALSE FALSE FALSE
GCST006061 0.0074 0.9269 537409 outcome TRUE reported 7157_22_PCDHA4_PCDA4
PCDHA4 chr4 101355525 0.008623 4.708e-08 35340 TRUE reported
textfile 2 1 TRUE
4800 rs6891054 G A G A -0.0677 -0.0176 0.25565 NA FALSE FALSE FALSE
GCST006061 0.0081 0.03003 537409 outcome TRUE reported 7157_22_PCDHA4_PCDA4
PCDHA4 chr5 125005010 0.009855 6.428e-12 35354 TRUE reported

textfile 2 1 TRUE
4801 rs735665 A G A G 0.0876 0.0019 0.16987 NA FALSE FALSE FALSE
GCST006061 0.0094 0.8439 537409 outcome TRUE reported 7157_22_PCDHA4_PCDA4
PCDHA4 chr11 123490689 0.011537 3.129e-14 35361 TRUE reported
textfile 2 1 TRUE
4802 rs7500719 A G A G 0.0679 -0.0148 0.28475 NA FALSE FALSE FALSE
GCST006061 0.0081 0.06712 537409 outcome TRUE reported 7157_22_PCDHA4_PCDA4
PCDHA4 chr16 30829556 0.009459 7.037e-13 35369 TRUE reported
textfile 2 1 TRUE
4803 rs79395339 C T C T -0.1646 -0.0409 0.02357 NA FALSE FALSE FALSE
GCST006061 0.0273 0.1349 537409 outcome TRUE reported 7157_22_PCDHA4_PCDA4
PCDHA4 chr20 3755220 0.028563 8.281e-09 35375 TRUE reported textfile
2 1 TRUE
4804 rs10739407 T C T C -0.111 1e-04 0.2237 NA FALSE FALSE FALSE
GCST006061 0.0086 0.9916 537409 outcome TRUE reported 6107_3_CELA1_ELA1
CELA1 chr9 114335475 0.009839 1.623e-29 35361 TRUE reported
textfile 2 1 TRUE
4805 rs112463699 G T G T -0.4121 0.025 0.0204 NA FALSE FALSE FALSE
GCST006061 0.0461 0.5871 537409 outcome TRUE reported 6107_3_CELA1_ELA1
CELA1 chr9 115680591 0.029462 1.86e-44 35367 TRUE reported
textfile 2 1 TRUE
4806 rs117157109 A C A C -0.4856 0.0017 0.08515 NA FALSE FALSE FALSE
GCST006061 0.0149 0.9107 537409 outcome TRUE reported 6107_3_CELA1_ELA1
CELA1 chr9 114968171 0.014706 1e-200 35362 TRUE reported textfile
2 1 TRUE
4807 rs12288924 A G A G 0.0616 -0.0092 0.22569 NA FALSE FALSE FALSE
GCST006061 0.0092 0.3186 537409 outcome TRUE reported 6107_3_CELA1_ELA1
CELA1 chr11 126416115 0.009093 1.248e-11 35370 TRUE reported
textfile 2 1 TRUE
4808 rs1871808 C T C T 0.0861 -0.0157 0.1475 NA FALSE FALSE FALSE
GCST006061 0.0096 0.1019 537409 outcome TRUE reported 6107_3_CELA1_ELA1
CELA1 chr8 26985635 0.010874 2.412e-15 35368 TRUE reported
textfile 2 1 TRUE
4809 rs4732746 A G A G 0.0872 -0.0147 0.23937 NA FALSE FALSE FALSE
GCST006061 0.0085 0.08379 537409 outcome TRUE reported 6107_3_CELA1_ELA1
CELA1 chr8 27772288 0.009022 4.247e-22 35369 TRUE reported
textfile 2 1 TRUE
4810 rs55714927 T C T C 0.0562 1e-04 0.21166 NA FALSE FALSE FALSE
GCST006061 0.0098 0.9926 537409 outcome TRUE reported 6107_3_CELA1_ELA1
CELA1 chr17 7176997 0.009372 2.012e-09 35370 TRUE reported textfile
2 1 TRUE
4811 rs56289258 T C T C -0.0783 -0.0052 0.08168 NA FALSE FALSE FALSE
GCST006061 0.0144 0.7168 537409 outcome TRUE reported 6107_3_CELA1_ELA1
CELA1 chr8 28663331 0.014208 3.566e-08 35367 TRUE reported
textfile 2 1 TRUE
4812 rs58090448 G A G A -0.1339 -0.0191 0.22069 NA FALSE FALSE FALSE
GCST006061 0.0083 0.02161 537409 outcome TRUE reported 6107_3_CELA1_ELA1
CELA1 chr9 114644886 0.009968 3.873e-41 35363 TRUE reported
textfile 2 1 TRUE
4813 rs7044769 T C T C 0.1384 -0.004 0.04944 NA FALSE FALSE FALSE
GCST006061 0.018 0.8247 537409 outcome TRUE reported 6107_3_CELA1_ELA1
CELA1 chr9 117186179 0.018896 2.402e-13 35368 TRUE reported
textfile 2 1 TRUE
4814 rs7045062 G A G A -0.2132 -0.0494 0.02773 NA FALSE FALSE FALSE
GCST006061 0.0287 0.085 537409 outcome TRUE reported 6107_3_CELA1_ELA1
CELA1 chr9 114229104 0.025576 7.687e-17 35363 TRUE reported
textfile 2 1 TRUE
4815 rs760715 C T C T 0.0762 0.0035 0.26206 NA FALSE FALSE FALSE
GCST006061 0.0092 0.7055 537409 outcome TRUE reported 6107_3_CELA1_ELA1
CELA1 chr22 39466338 0.00863 1.047e-18 35366 TRUE reported textfile
2 1 TRUE
4816 rs9300169 G A G A -0.0526 0.0092 0.26387 NA FALSE FALSE FALSE
GCST006061 0.0079 0.2436 537409 outcome TRUE reported 6107_3_CELA1_ELA1
CELA1 chr12 26273738 0.008618 1.038e-09 35359 TRUE reported
textfile 2 1 TRUE
4817 rs10739734 G T G T -0.0914 -0.0067 0.40136 NA FALSE FALSE FALSE

GCST006061 0.0073 0.3584 537409 outcome TRUE reported 18222_34_SH3GLB2_SHLB2
SH3GLB2 chr9 128765397 0.008357 7.62e-28 35360 TRUE reported
textfile 2 1 TRUE
4818 rs10858851 T C T C 0.0984 0.0082 0.25242 NA FALSE FALSE FALSE
GCST006061 0.0088 0.3488 537409 outcome TRUE reported 7948_129_GLTPD2_GLTD2
GLTPD2 chr12 89437093 0.009619 1.465e-24 35357 TRUE reported
textfile 2 1 TRUE
4819 rs1169288 C A C A 0.0513 -0.0103 0.31006 NA FALSE FALSE FALSE
GCST006061 0.0077 0.1791 537409 outcome TRUE reported 7948_129_GLTPD2_GLTD2
GLTPD2 chr12 120978847 0.009085 1.637e-08 35365 TRUE reported
textfile 2 1 TRUE
4820 rs1260326 C T C T -0.0645 -0.0069 0.34132 NA FALSE FALSE FALSE
GCST006061 0.0073 0.3401 537409 outcome TRUE reported 7948_129_GLTPD2_GLTD2
GLTPD2 chr2 27508073 0.008879 3.759e-13 35373 TRUE reported
textfile 2 1 TRUE
4821 rs147233090 T C T C 0.2199 0.0375 0.02693 NA FALSE FALSE FALSE
GCST006061 0.0254 0.1396 537409 outcome TRUE reported 7948_129_GLTPD2_GLTD2
GLTPD2 chr15 43735849 0.02578 1.466e-17 35362 TRUE reported textfile
2 1 TRUE
4822 rs149062332 C T C T 0.4602 0.0424 0.02045 NA FALSE FALSE FALSE
GCST006061 0.0435 0.33 537409 outcome TRUE reported 7948_129_GLTPD2_GLTD2
GLTPD2 chr3 186623236 0.028765 1.302e-57 35367 TRUE reported
textfile 2 1 TRUE
4823 rs1800961 T C T C -0.1131 -4.00E-04 0.04601 NA FALSE FALSE FALSE
GCST006061 0.0219 0.9854 537409 outcome TRUE reported 7948_129_GLTPD2_GLTD2
GLTPD2 chr20 44413724 0.020181 2.091e-08 35371 TRUE reported
textfile 2 1 TRUE
4824 rs2304969 T G T G -0.217 0.0028 0.14714 NA FALSE FALSE FALSE
GCST006061 0.0099 0.7783 537409 outcome TRUE reported 7948_129_GLTPD2_GLTD2
GLTPD2 chr17 4789264 0.011846 5.972e-75 35379 TRUE reported textfile
2 1 TRUE
4825 rs339969 A C A C 0.053 -0.0083 0.33988 NA FALSE FALSE FALSE
GCST006061 0.0075 0.2675 537409 outcome TRUE reported 7948_129_GLTPD2_GLTD2
GLTPD2 chr15 60591082 0.008834 1.978e-09 35370 TRUE reported
textfile 2 1 TRUE
4826 rs35491981 C T C T -0.0628 -0.0061 0.2541 NA FALSE FALSE FALSE
GCST006061 0.0084 0.4624 537409 outcome TRUE reported 7948_129_GLTPD2_GLTD2
GLTPD2 chr4 99148183 0.009644 7.421e-11 35345 TRUE reported
textfile 2 1 TRUE
4827 rs4523990 G A G A -0.0743 0.0034 0.24624 NA FALSE FALSE FALSE
GCST006061 0.0088 0.6969 537409 outcome TRUE reported 7948_129_GLTPD2_GLTD2
GLTPD2 chr17 4725146 0.009796 3.322e-14 35373 TRUE reported textfile
2 1 TRUE
4828 rs62440901 T C T C -0.0669 0.0026 0.15439 NA FALSE FALSE FALSE
GCST006061 0.0106 0.8028 537409 outcome TRUE reported 7948_129_GLTPD2_GLTD2
GLTPD2 chr6 160148036 0.011457 5.24e-09 35389 TRUE reported
textfile 2 1 TRUE
4829 rs77232416 A G A G 0.0974 -0.011 0.06298 NA FALSE FALSE FALSE
GCST006061 0.02 0.5822 537409 outcome TRUE reported 7948_129_GLTPD2_GLTD2
GLTPD2 chr11 59860784 0.017315 1.854e-08 35365 TRUE reported
textfile 2 1 TRUE
4830 rs8078118 A C A C 0.1937 -0.0066 0.35579 NA FALSE FALSE FALSE
GCST006061 0.0072 0.3583 537409 outcome TRUE reported 7948_129_GLTPD2_GLTD2
GLTPD2 chr17 4786743 0.008716 2.05e-109 35378 TRUE reported textfile
2 1 TRUE
4831 rs10740134 C T C T 0.0658 -0.0533 0.48017 NA FALSE FALSE FALSE
GCST006061 0.0071 5.362e-14 537409 outcome TRUE reported
9002_36_SERPINA11_SPA11 SERPINA11 chr10 63555673 0.008209 1.097e-15 35351
TRUE reported textfile 2 1 TRUE
4832 rs12468558 A G A G 0.0582 0.0079 0.28325 NA FALSE FALSE FALSE
GCST006061 0.008 0.3266 537409 outcome TRUE reported 9002_36_SERPINA11_SPA11
SERPINA11 chr2 229268990 0.009107 1.654e-10 35359 TRUE reported
textfile 2 1 TRUE
4833 rs144507412 T C T C -0.1651 0.0024 0.0216 NA FALSE FALSE FALSE
GCST006061 0.0375 0.9493 537409 outcome TRUE reported 9002_36_SERPINA11_SPA11
SERPINA11 chr14 95419604 0.028652 8.298e-09 35376 TRUE reported

textfile 2 1 TRUE
4834 rs149062332 C T C T -0.2493 0.0424 0.02045 NA FALSE FALSE FALSE
GCST006061 0.0435 0.33 537409 outcome TRUE reported 9002_36_SERPINA11_SPA11
SERPINA11 chr3 186623236 0.028177 8.941e-19 35367 TRUE reported
textfile 2 1 TRUE
4835 rs34717890 T C T C -0.117 -0.0448 0.08864 NA FALSE FALSE FALSE
GCST006061 0.0116 0.0001135 537409 outcome TRUE reported
9002_36_SERPINA11_SPA11 SERPINA11 chr19 45897185 0.014512 7.471e-16 35371
TRUE reported textfile 2 1 TRUE
4836 rs4484649 A C A C 0.0592 -0.0063 0.36672 NA FALSE FALSE FALSE
GCST006061 0.0074 0.3923 537409 outcome TRUE reported 9002_36_SERPINA11_SPA11
SERPINA11 chr8 10713981 0.008474 2.827e-12 35383 TRUE reported
textfile 2 1 TRUE
4837 rs4665972 C T C T -0.0676 -0.0046 0.33903 NA FALSE FALSE FALSE
GCST006061 0.0075 0.5416 537409 outcome TRUE reported 9002_36_SERPINA11_SPA11
SERPINA11 chr2 27375230 0.008676 6.623e-15 35374 TRUE reported
textfile 2 1 TRUE
4838 rs59233955 A G A G 0.0548 -0.0032 0.45073 NA FALSE FALSE FALSE
GCST006061 0.0074 0.6656 537409 outcome TRUE reported 9002_36_SERPINA11_SPA11
SERPINA11 chr14 94706204 0.008291 3.85e-11 35371 TRUE reported
textfile 2 1 TRUE
4839 rs7156347 T C T C 0.0721 -0.0218 0.24249 NA FALSE FALSE FALSE
GCST006061 0.0092 0.01749 537409 outcome TRUE reported 9002_36_SERPINA11_SPA11
SERPINA11 chr14 94416800 0.009683 9.603e-14 35372 TRUE reported
textfile 2 1 TRUE
4840 rs72631635 T G T G -0.2965 0.0052 0.16187 NA FALSE FALSE FALSE
GCST006061 0.0096 0.5899 537409 outcome TRUE reported 9002_36_SERPINA11_SPA11
SERPINA11 chr14 94455724 0.011076 7.358e-158 35372 TRUE reported
textfile 2 1 TRUE
4841 rs77403344 T C T C 0.1054 0.0102 0.08704 NA FALSE FALSE FALSE
GCST006061 0.0159 0.5224 537409 outcome TRUE reported 9002_36_SERPINA11_SPA11
SERPINA11 chr14 94672718 0.01465 6.272e-13 35372 TRUE reported
textfile 2 1 TRUE
4842 rs9671989 G A G A -0.0935 -4.00E-04 0.41734 NA FALSE FALSE FALSE
GCST006061 0.0073 0.9516 537409 outcome TRUE reported 9002_36_SERPINA11_SPA11
SERPINA11 chr14 94547244 0.008398 8.576e-29 35371 TRUE reported
textfile 2 1 TRUE
4843 rs10745330 T C T C 0.0998 -0.0013 0.4894 NA FALSE FALSE FALSE
GCST006061 0.0071 0.8578 537409 outcome TRUE reported 17764_108_RHOC_RHOC
RHOC chr1 112540817 0.008177 2.922e-34 35343 TRUE reported
textfile 2 1 TRUE
4844 rs11082304 T G T G -0.0498 -0.0179 0.48036 NA FALSE FALSE FALSE
GCST006061 0.0071 0.01203 537409 outcome TRUE reported 17764_108_RHOC_RHOC
RHOC chr18 23141009 0.008201 1.26e-09 35351 TRUE reported
textfile 2 1 TRUE
4845 rs112595944 A G A G -0.0905 -0.0346 0.06824 NA FALSE FALSE FALSE
GCST006061 0.016 0.03019 537409 outcome TRUE reported 17764_108_RHOC_RHOC
RHOC chr19 18314948 0.016343 3.066e-08 35350 TRUE reported
textfile 2 1 TRUE
4846 rs1354034 C T C T 0.0706 0.0088 0.38119 NA FALSE FALSE FALSE
GCST006061 0.0073 0.2248 537409 outcome TRUE reported 17764_108_RHOC_RHOC
RHOC chr3 56815721 0.008395 4.111e-17 35356 TRUE reported
textfile 2 1 TRUE
4847 rs3811444 T C T C -0.0546 0.0103 0.34232 NA FALSE FALSE FALSE
GCST006061 0.0077 0.1812 537409 outcome TRUE reported 17764_108_RHOC_RHOC
RHOC chr1 247876149 0.008689 3.308e-10 35156 TRUE reported
textfile 2 1 TRUE
4848 rs73165061 A G A G 0.1203 0.015 0.1903 NA FALSE FALSE FALSE
GCST006061 0.0089 0.09083 537409 outcome TRUE reported 17764_108_RHOC_RHOC
RHOC chr3 165764157 0.010362 3.687e-31 35339 TRUE reported
textfile 2 1 TRUE
4849 rs7412 T C T C 0.0944 -0.0027 0.05941 NA FALSE FALSE FALSE GCST006061
0.0138 0.8459 537409 outcome TRUE reported 17764_108_RHOC_RHOC RHOC chr19
44908822 0.01726 4.522e-08 35367 TRUE reported textfile 2 1 TRUE
4850 rs10745925 C T C T -0.097 0.0051 0.27828 NA FALSE FALSE FALSE
GCST006061 0.0078 0.5103 537409 outcome TRUE reported 9385_4_GAA_GAA GAA

chr12 101825121 0.009228 7.652e-26 35364 TRUE reported textfile 2
1 TRUE
4851 rs145961846 A G A G 0.2588 -0.0202 0.01877 NA FALSE FALSE FALSE
GCST006061 0.0334 0.5455 537409 outcome TRUE reported 9385_4_GAA_GAA GAA
chr17 80007145 0.030403 1.704e-17 35378 TRUE reported textfile 2
1 TRUE
4852 rs2304850 T C T C -0.3344 0.0035 0.22453 NA FALSE FALSE FALSE
GCST006061 0.0085 0.6838 537409 outcome TRUE reported 9385_4_GAA_GAA GAA
chr17 80101429 0.009847 1e-200 35383 TRUE reported textfile 2 1
TRUE
4853 rs2672896 A G A G -0.0736 -0.0057 0.19427 NA FALSE FALSE FALSE
GCST006061 0.0088 0.5162 537409 outcome TRUE reported 9385_4_GAA_GAA GAA
chr17 80851003 0.010581 3.504e-12 35196 TRUE reported textfile 2
1 TRUE
4854 rs56255430 C A C A 0.0928 -0.0034 0.0795 NA FALSE FALSE FALSE
GCST006061 0.0132 0.7997 537409 outcome TRUE reported 9385_4_GAA_GAA GAA
chr19 19367068 0.015272 1.23e-09 35353 TRUE reported textfile 2
1 TRUE
4855 rs7211532 G A G A -0.055 -0.0026 0.45993 NA FALSE FALSE FALSE
GCST006061 0.0074 0.7289 537409 outcome TRUE reported 9385_4_GAA_GAA GAA
chr17 79957692 0.008401 5.875e-11 35376 TRUE reported textfile 2
1 TRUE
4856 rs77319086 A C A C -0.1546 -0.0047 0.0487 NA FALSE FALSE FALSE
GCST006061 0.0142 0.7417 537409 outcome TRUE reported 9385_4_GAA_GAA GAA
chr17 80137118 0.019505 2.258e-15 35386 TRUE reported textfile 2
1 TRUE
4857 rs8071514 A C A C 0.0783 -0.0047 0.36089 NA FALSE FALSE FALSE
GCST006061 0.0072 0.517 537409 outcome TRUE reported 9385_4_GAA_GAA GAA
chr17 80990283 0.008743 3.367e-19 35161 TRUE reported textfile 2
1 TRUE
4858 rs10746399 C T C T -0.2398 0.0045 0.45255 NA FALSE FALSE FALSE
GCST006061 0.0074 0.5445 537409 outcome TRUE reported 17682_1_CD46_CD46
CD46 chr1 207770727 0.008173 3.182e-189 35370 TRUE reported
textfile 2 1 TRUE
4859 rs60467252 A G A G 0.0839 -0.0055 0.12495 NA FALSE FALSE FALSE
GCST006061 0.0107 0.6066 537409 outcome TRUE reported 17682_1_CD46_CD46
CD46 chr1 207651037 0.012446 1.574e-11 35370 TRUE reported
textfile 2 1 TRUE
4860 rs72704449 C T C T 0.1774 0.013 0.04582 NA FALSE FALSE FALSE
GCST006061 0.0199 0.5128 537409 outcome TRUE reported 17682_1_CD46_CD46
CD46 chr1 179504138 0.019601 1.425e-19 35358 TRUE reported
textfile 2 1 TRUE
4861 rs77924615 A G A G -0.0635 0.0136 0.19701 NA FALSE FALSE FALSE
GCST006061 0.0092 0.1375 537409 outcome TRUE reported 17682_1_CD46_CD46
CD46 chr16 20381010 0.010374 9.307e-10 35355 TRUE reported
textfile 2 1 TRUE
4862 rs972570 T C T C 0.0906 0.0034 0.47747 NA FALSE FALSE FALSE
GCST006061 0.0072 0.6375 537409 outcome TRUE reported 17682_1_CD46_CD46
CD46 chr10 69512262 0.008181 1.672e-28 35365 TRUE reported
textfile 2 1 TRUE
4863 rs10746529 A G A G -0.3123 0.043 0.03502 NA FALSE FALSE FALSE
GCST006061 0.0183 0.01903 537409 outcome TRUE reported 19272_9_PEF1_PEF1
PEF1 chr2 21259078 0.023104 1.239e-41 35373 TRUE reported
textfile 2 1 TRUE
4864 rs111696008 A G A G -0.319 -0.0259 0.02443 NA FALSE FALSE FALSE
GCST006061 0.0401 0.519 537409 outcome TRUE reported 19272_9_PEF1_PEF1
PEF1 chr2 216622986 0.02786 2.346e-30 35359 TRUE reported textfile
2 1 TRUE
4865 rs113840968 G A G A -0.3641 -0.0199 0.01515 NA FALSE FALSE FALSE
GCST006061 0.0421 0.6363 537409 outcome TRUE reported 19272_9_PEF1_PEF1
PEF1 chr2 23321683 0.034276 2.336e-26 35370 TRUE reported
textfile 2 1 TRUE
4866 rs114833464 T C T C 0.1412 -0.0017 0.02948 NA FALSE FALSE FALSE
GCST006061 0.0252 0.9475 537409 outcome TRUE reported 19272_9_PEF1_PEF1
PEF1 chr3 164194396 0.024531 8.617e-09 35342 TRUE reported
textfile 2 1 TRUE

4867 rs116498710 T C T C -0.7936 -0.0134 0.01295 NA FALSE FALSE FALSE
GCST006061 0.0274 0.6245 537409 outcome TRUE reported 19272_9_PEF1_PEF1
PEF1 chr2 21052184 0.036192 1.426e-106 35373 TRUE reported
textfile 2 1 TRUE
4868 rs12026141 T C T C -0.0683 0.0094 0.31246 NA FALSE FALSE FALSE
GCST006061 0.0076 0.2137 537409 outcome TRUE reported 19272_9_PEF1_PEF1
PEF1 chr1 201980465 0.008886 1.515e-14 35357 TRUE reported
textfile 2 1 TRUE
4869 rs144202860 A G A G -0.3219 -0.0629 0.01274 NA FALSE FALSE FALSE
GCST006061 0.0538 0.2423 537409 outcome TRUE reported 19272_9_PEF1_PEF1
PEF1 chr2 216625740 0.037336 6.597e-18 35359 TRUE reported
textfile 2 1 TRUE
4870 rs145242450 C T C T 0.1611 0.0204 0.02099 NA FALSE FALSE FALSE
GCST006061 0.0323 0.5269 537409 outcome TRUE reported 19272_9_PEF1_PEF1
PEF1 chr3 167952807 0.028958 2.648e-08 35354 TRUE reported
textfile 2 1 TRUE
4871 rs147033786 C A C A 0.3531 0.0651 0.01101 NA FALSE FALSE FALSE
GCST006061 0.05 0.1932 537409 outcome TRUE reported 19272_9_PEF1_PEF1
PEF1 chr3 168506251 0.040282 1.856e-18 35354 TRUE reported
textfile 2 1 TRUE
4872 rs147711004 A G A G 0.1234 -0.0184 0.06423 NA FALSE FALSE FALSE
GCST006061 0.023 0.4248 537409 outcome TRUE reported 19272_9_PEF1_PEF1
PEF1 chr19 44834661 0.01673 1.631e-13 35367 TRUE reported textfile
2 1 TRUE
4873 rs1532085 G A G A 0.0955 0.0148 0.41111 NA FALSE FALSE FALSE
GCST006061 0.0073 0.04221 537409 outcome TRUE reported 19272_9_PEF1_PEF1
PEF1 chr15 58391167 0.008283 9.331e-31 35364 TRUE reported
textfile 2 1 TRUE
4874 rs2070895 A G A G -0.1005 0.0021 0.19622 NA FALSE FALSE FALSE
GCST006061 0.0084 0.7984 537409 outcome TRUE reported 19272_9_PEF1_PEF1
PEF1 chr15 58431740 0.010254 1.111e-22 35366 TRUE reported
textfile 2 1 TRUE
4875 rs247617 A C A C -0.0625 0.0125 0.32148 NA FALSE FALSE FALSE
GCST006061 0.0077 0.1049 537409 outcome TRUE reported 19272_9_PEF1_PEF1
PEF1 chr16 56956804 0.008724 7.844e-13 35369 TRUE reported
textfile 2 1 TRUE
4876 rs55915628 C T C T -0.0528 -0.0059 0.43037 NA FALSE FALSE FALSE
GCST006061 0.0074 0.4248 537409 outcome TRUE reported 19272_9_PEF1_PEF1
PEF1 chr2 203441729 0.008511 5.521e-10 35365 TRUE reported
textfile 2 1 TRUE
4877 rs583104 T G T G 0.1181 -0.0173 0.20819 NA FALSE FALSE FALSE
GCST006061 0.0087 0.04629 537409 outcome TRUE reported 19272_9_PEF1_PEF1
PEF1 chr1 109278685 0.0101 1.375e-31 35344 TRUE reported textfile
2 1 TRUE
4878 rs61754186 A G A G -0.215 -0.1093 0.01903 NA FALSE FALSE FALSE
GCST006061 0.0646 0.09076 537409 outcome TRUE reported 19272_9_PEF1_PEF1
PEF1 chr2 24293710 0.030602 2.131e-12 35369 TRUE reported
textfile 2 1 TRUE
4879 rs696516 T G T G 0.0573 -0.0165 0.27351 NA FALSE FALSE FALSE
GCST006061 0.0082 0.04314 537409 outcome TRUE reported 19272_9_PEF1_PEF1
PEF1 chr3 136348936 0.009323 7.953e-10 35361 TRUE reported
textfile 2 1 TRUE
4880 rs72784378 T C T C -0.3007 0.0089 0.02458 NA FALSE FALSE FALSE
GCST006061 0.0233 0.7022 537409 outcome TRUE reported 19272_9_PEF1_PEF1
PEF1 chr2 20798530 0.026871 4.548e-29 35370 TRUE reported
textfile 2 1 TRUE
4881 rs7412 T C T C -0.3394 -0.0027 0.05941 NA FALSE FALSE FALSE GCST006061
0.0138 0.8459 537409 outcome TRUE reported 19272_9_PEF1_PEF1 PEF1 chr19
44908822 0.017216 1.639e-86 35367 TRUE reported textfile 2 1 TRUE
4882 rs76693471 T C T C -0.2492 -0.0205 0.03559 NA FALSE FALSE FALSE
GCST006061 0.0316 0.5159 537409 outcome TRUE reported 19272_9_PEF1_PEF1
PEF1 chr2 22911945 0.022645 3.636e-28 35369 TRUE reported
textfile 2 1 TRUE
4883 rs77230198 A C A C 0.2303 0.0828 0.02175 NA FALSE FALSE FALSE
GCST006061 0.0283 0.003378 537409 outcome TRUE reported 19272_9_PEF1_PEF1
PEF1 chr3 166581568 0.028743 1.124e-15 35354 TRUE reported

textfile 2 1 TRUE
4884 rs78980904 C T C T 0.3683 0.0012 0.26023 NA FALSE FALSE FALSE
GCST006061 0.0083 0.8846 537409 outcome TRUE reported 19272_9_PEF1_PEF1
PEF1 chr2 20921704 0.006936 1e-200 35370 TRUE reported textfile
2 1 TRUE
4885 rs10747050 A G A G 0.2808 0.0298 0.07975 NA FALSE FALSE FALSE
GCST006061 0.0175 0.08879 537409 outcome TRUE reported 8346_9_DPP7_DPP2
DPP7 chr9 137161424 0.015309 3.838e-75 35254 TRUE reported
textfile 2 1 TRUE
4886 rs1242103 C T C T 0.0599 0.0109 0.32703 NA FALSE FALSE FALSE
GCST006061 0.008 0.1714 537409 outcome TRUE reported 8346_9_DPP7_DPP2
DPP7 chr14 92734784 0.008643 4.191e-12 35362 TRUE reported
textfile 2 1 TRUE
4887 rs146403924 A G A G -0.412 0.0154 0.01356 NA FALSE FALSE FALSE
GCST006061 0.0655 0.8137 537409 outcome TRUE reported 8346_9_DPP7_DPP2
DPP7 chr9 135720421 0.036931 6.696e-29 35296 TRUE reported
textfile 2 1 TRUE
4888 rs147058434 A G A G -0.2437 0.0726 0.03066 NA FALSE FALSE FALSE
GCST006061 0.0247 0.003254 537409 outcome TRUE reported 8346_9_DPP7_DPP2
DPP7 chr9 137125435 0.024037 3.727e-24 35259 TRUE reported
textfile 2 1 TRUE
4889 rs2811789 G A G A 0.0887 0.0132 0.46978 NA FALSE FALSE FALSE
GCST006061 0.0074 0.075 537409 outcome TRUE reported 8346_9_DPP7_DPP2
DPP7 chr9 136799504 0.008331 1.81e-26 35256 TRUE reported
textfile 2 1 TRUE
4890 rs72763283 A G A G -0.095 -0.0026 0.073 NA FALSE FALSE FALSE
GCST006061 0.0175 0.88 537409 outcome TRUE reported 8346_9_DPP7_DPP2
DPP7 chr9 137182012 0.016017 3.004e-09 35258 TRUE reported
textfile 2 1 TRUE
4891 rs77298262 A G A G 0.2432 -0.0245 0.01198 NA FALSE FALSE FALSE
GCST006061 0.0381 0.5209 537409 outcome TRUE reported 8346_9_DPP7_DPP2
DPP7 chr9 137171670 0.037811 1.259e-10 35257 TRUE reported
textfile 2 1 TRUE
4892 rs10750096 A C A C 0.3 -0.002 0.06851 NA FALSE FALSE FALSE GCST006061
0.0128 0.8791 537409 outcome TRUE reported 9511_61_NXPH2_NXPH2 NXPH2 chr11
116786072 0.015872 1.115e-79 35376 TRUE reported textfile 2 1 TRUE
4893 rs111696008 A G A G -0.3528 -0.0259 0.02443 NA FALSE FALSE FALSE
GCST006061 0.0401 0.519 537409 outcome TRUE reported 9511_61_NXPH2_NXPH2
NXPH2 chr2 216622986 0.026828 1.688e-39 35359 TRUE reported
textfile 2 1 TRUE
4894 rs11668738 G T G T -0.0721 -0.0073 0.13121 NA FALSE FALSE FALSE
GCST006061 0.0098 0.4584 537409 outcome TRUE reported 9511_61_NXPH2_NXPH2
NXPH2 chr19 44844304 0.011962 1.665e-09 35367 TRUE reported
textfile 2 1 TRUE
4895 rs1291921 G A G A 0.0543 -0.0157 0.2763 NA FALSE FALSE FALSE
GCST006061 0.0081 0.05326 537409 outcome TRUE reported 9511_61_NXPH2_NXPH2
NXPH2 chr3 136317384 0.009126 2.685e-09 35361 TRUE reported
textfile 2 1 TRUE
4896 rs144202860 A G A G -0.338 -0.0629 0.01274 NA FALSE FALSE FALSE
GCST006061 0.0538 0.2423 537409 outcome TRUE reported 9511_61_NXPH2_NXPH2
NXPH2 chr2 216625740 0.035956 5.441e-21 35359 TRUE reported
textfile 2 1 TRUE
4897 rs148077724 G A G A 0.1784 -0.0095 0.02611 NA FALSE FALSE FALSE
GCST006061 0.0214 0.6574 537409 outcome TRUE reported 9511_61_NXPH2_NXPH2
NXPH2 chr3 165952844 0.025272 1.676e-12 35340 TRUE reported
textfile 2 1 TRUE
4898 rs150456509 G A G A -0.1358 0.0104 0.03345 NA FALSE FALSE FALSE
GCST006061 0.0347 0.7634 537409 outcome TRUE reported 9511_61_NXPH2_NXPH2
NXPH2 chr3 167573467 0.022684 2.142e-09 35354 TRUE reported
textfile 2 1 TRUE
4899 rs2740488 C A C A -0.0592 -0.0159 0.25976 NA FALSE FALSE FALSE
GCST006061 0.0081 0.04886 537409 outcome TRUE reported 9511_61_NXPH2_NXPH2
NXPH2 chr9 104899461 0.009187 1.165e-10 35363 TRUE reported
textfile 2 1 TRUE
4900 rs35173225 T C T C 0.0798 -0.0095 0.11621 NA FALSE FALSE FALSE
GCST006061 0.0111 0.3889 537409 outcome TRUE reported 9511_61_NXPH2_NXPH2

NXPH2 chr7 73586691 0.012563 2.129e-10 35354 TRUE reported
textfile 2 1 TRUE
4901 rs3816117 C T C T 0.0903 0.0137 0.49421 NA FALSE FALSE FALSE
GCST006061 0.0071 0.05455 537409 outcome TRUE reported 9511_61_NXPH2_NXPH2
NXPH2 chr16 56962246 0.008078 5.203e-29 35369 TRUE reported
textfile 2 1 TRUE
4902 rs429358 C T C T -0.1005 0.005 0.17407 NA FALSE FALSE FALSE
GCST006061 0.0103 0.6319 537409 outcome TRUE reported 9511_61_NXPH2_NXPH2
NXPH2 chr19 44908684 0.010764 9.925e-21 35368 TRUE reported
textfile 2 1 TRUE
4903 rs4950806 C T C T -0.0646 0.0068 0.36177 NA FALSE FALSE FALSE
GCST006061 0.0072 0.3457 537409 outcome TRUE reported 9511_61_NXPH2_NXPH2
NXPH2 chr1 201983446 0.008454 2.149e-14 35357 TRUE reported
textfile 2 1 TRUE
4904 rs55881728 G A G A -0.0789 -0.0072 0.41277 NA FALSE FALSE FALSE
GCST006061 0.0074 0.3268 537409 outcome TRUE reported 9511_61_NXPH2_NXPH2
NXPH2 chr2 203441633 0.00825 1.133e-21 35365 TRUE reported textfile
2 1 TRUE
4905 rs73169103 T C T C 0.1881 0.0178 0.03563 NA FALSE FALSE FALSE
GCST006061 0.0249 0.4756 537409 outcome TRUE reported 9511_61_NXPH2_NXPH2
NXPH2 chr3 167190675 0.021916 9.27e-18 35354 TRUE reported
textfile 2 1 TRUE
4906 rs887829 T C T C 0.0619 0.0019 0.31955 NA FALSE FALSE FALSE
GCST006061 0.0077 0.8072 537409 outcome TRUE reported 9511_61_NXPH2_NXPH2
NXPH2 chr2 233759924 0.008703 1.139e-12 35372 TRUE reported
textfile 2 1 TRUE
4907 rs10751648 C T C T -0.0506 -0.0053 0.47184 NA FALSE FALSE FALSE
GCST006061 0.0086 0.5379 537409 outcome TRUE reported 8687_26_TMEM106B_T106B
TMEM106B chr11 307244 0.007963 2.093e-10 35338 TRUE reported
textfile 2 1 TRUE
4908 rs10922098 T C T C -0.0744 -0.009 0.40011 NA FALSE FALSE FALSE
GCST006061 0.0075 0.2275 537409 outcome TRUE reported 8687_26_TMEM106B_T106B
TMEM106B chr1 196695521 0.008053 2.5e-20 35338 TRUE reported
textfile 2 1 TRUE
4909 rs10751648 C T C T -0.0567 -0.0053 0.47184 NA FALSE FALSE FALSE
GCST006061 0.0086 0.5379 537409 outcome TRUE reported
17806_6_SDCBP_Syntenin_1 SDCBP chr11 307244 0.008176 4.076e-12 35338
TRUE reported textfile 2 1 TRUE
4910 rs10914357 T C T C -0.0582 -0.0062 0.21025 NA FALSE FALSE FALSE
GCST006061 0.0094 0.5085 537409 outcome TRUE reported
17806_6_SDCBP_Syntenin_1 SDCBP chr1 31336881 0.009947 4.878e-09 35374
TRUE reported textfile 2 1 TRUE
4911 rs11557154 T C T C 0.12 0.0043 0.13413 NA FALSE FALSE FALSE
GCST006061 0.0102 0.6725 537409 outcome TRUE reported
17806_6_SDCBP_Syntenin_1 SDCBP chr9 34107507 0.011878 5.392e-24 35349
TRUE reported textfile 2 1 TRUE
4912 rs13043452 A G A G -0.0819 -0.0021 0.35271 NA FALSE FALSE FALSE
GCST006061 0.0075 0.7745 537409 outcome TRUE reported
17806_6_SDCBP_Syntenin_1 SDCBP chr20 3725467 0.008494 5.31e-22 35376
TRUE reported textfile 2 1 TRUE
4913 rs1354034 C T C T 0.0549 0.0088 0.38119 NA FALSE FALSE FALSE
GCST006061 0.0073 0.2248 537409 outcome TRUE reported
17806_6_SDCBP_Syntenin_1 SDCBP chr3 56815721 0.008335 4.497e-11 35356
TRUE reported textfile 2 1 TRUE
4914 rs755240 G T G T 0.1854 0.0357 0.02262 NA FALSE FALSE FALSE
GCST006061 0.0218 0.1021 537409 outcome TRUE reported
17806_6_SDCBP_Syntenin_1 SDCBP chr20 3691763 0.027168 8.849e-12 35374
TRUE reported textfile 2 1 TRUE
4915 rs10752113 T C T C 0.3157 0.0038 0.23779 NA FALSE FALSE FALSE
GCST006061 0.0084 0.6494 537409 outcome TRUE reported 8233_2_ITIH5_ITIH5
ITIH5 chr10 7606227 0.010255 1e-200 35351 TRUE reported textfile 2
1 TRUE
4916 rs10789510 G A G A -0.0867 -0.0074 0.19276 NA FALSE FALSE FALSE
GCST006061 0.0088 0.3997 537409 outcome TRUE reported 8233_2_ITIH5_ITIH5
ITIH5 chr1 47484460 0.010907 1.88e-15 35368 TRUE reported
textfile 2 1 TRUE

4917 rs118063690 G A G A -0.3403 -0.0466 0.01034 NA FALSE FALSE FALSE GCST006061 0.0504 0.3551 537409 outcome TRUE reported 8233_2_ITIH5_ITIH5 ITIH5 chr10 7707528 0.043797 7.847e-15 35360 TRUE reported textfile 2 1 TRUE

4918 rs12573122 G A G A 0.0803 -0.018 0.17825 NA FALSE FALSE FALSE GCST006061 0.0101 0.07515 537409 outcome TRUE reported 8233_2_ITIH5_ITIH5 ITIH5 chr10 6696351 0.011557 3.696e-12 35354 TRUE reported textfile 2 1 TRUE

4919 rs191234395 C T C T -0.3893 -0.0241 0.01617 NA FALSE FALSE FALSE GCST006061 0.0443 0.5853 537409 outcome TRUE reported 8233_2_ITIH5_ITIH5 ITIH5 chr10 7113567 0.034186 4.809e-30 35354 TRUE reported textfile 2 1 TRUE

4920 rs2413595 C T C T 0.0519 -0.0037 0.45877 NA FALSE FALSE FALSE GCST006061 0.0075 0.6281 537409 outcome TRUE reported 8233_2_ITIH5_ITIH5 ITIH5 chr22 39534192 0.008577 1.438e-09 35368 TRUE reported textfile 2 1 TRUE

4921 rs28616296 C A C A 0.0721 -0.0012 0.35882 NA FALSE FALSE FALSE GCST006061 0.0076 0.8727 537409 outcome TRUE reported 8233_2_ITIH5_ITIH5 ITIH5 chr2 128362258 0.008874 4.463e-16 35362 TRUE reported textfile 2 1 TRUE

4922 rs61830291 C A C A 0.0877 -0.0236 0.10488 NA FALSE FALSE FALSE GCST006061 0.0132 0.07315 537409 outcome TRUE reported 8233_2_ITIH5_ITIH5 ITIH5 chr1 220827800 0.013965 3.388e-10 35353 TRUE reported textfile 2 1 TRUE

4923 rs74124181 A G A G -0.2824 -0.0118 0.01192 NA FALSE FALSE FALSE GCST006061 0.0324 0.7153 537409 outcome TRUE reported 8233_2_ITIH5_ITIH5 ITIH5 chr10 8462155 0.040229 2.222e-12 35356 TRUE reported textfile 2 1 TRUE

4924 rs760715 C T C T 0.1041 0.0035 0.26206 NA FALSE FALSE FALSE GCST006061 0.0092 0.7055 537409 outcome TRUE reported 8233_2_ITIH5_ITIH5 ITIH5 chr22 39466338 0.009659 4.401e-27 35366 TRUE reported textfile 2 1 TRUE

4925 rs79051267 G A G A 0.1435 -0.0225 0.05379 NA FALSE FALSE FALSE GCST006061 0.0242 0.3511 537409 outcome TRUE reported 8233_2_ITIH5_ITIH5 ITIH5 chr10 7698129 0.01986 4.984e-13 35361 TRUE reported textfile 2 1 TRUE

4926 rs10753508 C T C T 0.0516 -0.0145 0.25798 NA FALSE FALSE FALSE GCST006061 0.0083 0.08122 537409 outcome TRUE reported 11530_37_HMBS_HEM3 HMBS chr1 19066539 0.009145 1.675e-08 35371 TRUE reported textfile 2 1 TRUE

4927 rs115740542 C T C T 0.0971 -0.039 0.06666 NA FALSE FALSE FALSE GCST006061 0.0158 0.0139 537409 outcome TRUE reported 11530_37_HMBS_HEM3 HMBS chr6 26123274 0.016085 1.575e-09 35367 TRUE reported textfile 2 1 TRUE

4928 rs2072860 A G A G 0.0575 -0.0029 0.4257 NA FALSE FALSE FALSE GCST006061 0.0072 0.6893 537409 outcome TRUE reported 11530_37_HMBS_HEM3 HMBS chr22 37074564 0.008164 1.879e-12 35364 TRUE reported textfile 2 1 TRUE

4929 rs34164109 T C T C 0.0587 -0.0022 0.27022 NA FALSE FALSE FALSE GCST006061 0.008 0.7805 537409 outcome TRUE reported 11530_37_HMBS_HEM3 HMBS chr6 135100038 0.009054 8.963e-11 35362 TRUE reported textfile 2 1 TRUE

4930 rs45510602 T C T C -0.0668 -0.0058 0.19079 NA FALSE FALSE FALSE GCST006061 0.0097 0.5509 537409 outcome TRUE reported 11530_37_HMBS_HEM3 HMBS chr11 119046875 0.01023 6.581e-11 35371 TRUE reported textfile 2 1 TRUE

4931 rs10754199 G A G A -0.0841 -0.0096 0.39961 NA FALSE FALSE FALSE GCST006061 0.0075 0.1987 537409 outcome TRUE reported 9779_63_HERPUD2_HERP2 HERPUD2 chr1 196701709 0.008019 9.86e-26 35339 TRUE reported textfile 2 1 TRUE

4932 rs2929937 G A G A -0.0445 -0.0033 0.4557 NA FALSE FALSE FALSE GCST006061 0.0076 0.6579 537409 outcome TRUE reported 9779_63_HERPUD2_HERP2 HERPUD2 chr8 133206297 0.007846 1.417e-08 35375 TRUE reported textfile 2 1 TRUE

4933 rs73161871 G A G A 0.1085 -5.00E-04 0.04552 NA FALSE FALSE FALSE GCST006061 0.0193 0.9779 537409 outcome TRUE reported 9779_63_HERPUD2_HERP2

HERPUD2 chr7 152020681 0.018945 1.022e-08 35374 TRUE reported
textfile 2 1 TRUE
4934 rs10755215 T C T C 0.3618 -0.0152 0.43891 NA FALSE FALSE FALSE
GCST006061 0.0074 0.04129 537409 outcome TRUE reported 4535_50_BST1_BST1
BST1 chr4 15531414 0.004836 1e-200 35361 TRUE reported textfile
2 1 TRUE
4935 rs116210165 G A G A 0.1681 0.0083 0.01924 NA FALSE FALSE FALSE
GCST006061 0.0487 0.8652 537409 outcome TRUE reported 4535_50_BST1_BST1
BST1 chr4 11716717 0.028943 6.324e-09 35360 TRUE reported
textfile 2 1 TRUE
4936 rs116852327 T G T G 0.1604 -0.0213 0.01186 NA FALSE FALSE FALSE
GCST006061 0.0385 0.5806 537409 outcome TRUE reported 4535_50_BST1_BST1
BST1 chr17 7721481 0.028338 1.511e-08 35377 TRUE reported textfile
2 1 TRUE
4937 rs12280645 G A G A 0.0819 -0.0059 0.33798 NA FALSE FALSE FALSE
GCST006061 0.0079 0.4507 537409 outcome TRUE reported 4535_50_BST1_BST1
BST1 chr11 126393990 0.006927 2.96e-32 35374 TRUE reported
textfile 2 1 TRUE
4938 rs142983596 A G A G -0.4921 -0.0215 0.01447 NA FALSE FALSE FALSE
GCST006061 0.0491 0.6613 537409 outcome TRUE reported 4535_50_BST1_BST1
BST1 chr4 16787397 0.03394 1.228e-47 35357 TRUE reported textfile
2 1 TRUE
4939 rs143786529 G A G A -0.3804 -0.0351 0.01356 NA FALSE FALSE FALSE
GCST006061 0.0501 0.4838 537409 outcome TRUE reported 4535_50_BST1_BST1
BST1 chr4 17009182 0.034887 1.106e-27 35358 TRUE reported
textfile 2 1 TRUE
4940 rs144572272 C T C T -0.9948 0.0368 0.02353 NA FALSE FALSE FALSE
GCST006061 0.0515 0.4754 537409 outcome TRUE reported 4535_50_BST1_BST1
BST1 chr4 15157963 0.01559 1e-200 35365 TRUE reported textfile 2
1 TRUE
4941 rs16890557 G T G T -0.2317 -0.0023 0.03951 NA FALSE FALSE FALSE
GCST006061 0.017 0.8918 537409 outcome TRUE reported 4535_50_BST1_BST1
BST1 chr4 14462411 0.020552 1.771e-29 35366 TRUE reported
textfile 2 1 TRUE
4942 rs4760 G A G A -0.1736 -0.0025 0.12052 NA FALSE FALSE FALSE GCST006061
0.0109 0.8165 537409 outcome TRUE reported 4535_50_BST1_BST1 BST1 chr19
43648948 0.01005 7.511e-67 35369 TRUE reported textfile 2 1 TRUE
4943 rs55714927 T C T C 0.0553 1e-04 0.21166 NA FALSE FALSE FALSE
GCST006061 0.0098 0.9926 537409 outcome TRUE reported 4535_50_BST1_BST1
BST1 chr17 7176997 0.00809 8.154e-12 35370 TRUE reported textfile 2
1 TRUE
4944 rs55768074 T C T C 0.1322 0.0025 0.47252 NA FALSE FALSE FALSE
GCST006061 0.0073 0.7344 537409 outcome TRUE reported 4535_50_BST1_BST1
BST1 chr4 15985026 0.008028 6.326e-61 35361 TRUE reported
textfile 2 1 TRUE
4945 rs73801515 T C T C -0.3892 -0.0037 0.05024 NA FALSE FALSE FALSE
GCST006061 0.0184 0.8391 537409 outcome TRUE reported 4535_50_BST1_BST1
BST1 chr4 15877548 0.018554 1.062e-97 35362 TRUE reported
textfile 2 1 TRUE
4946 rs80094095 G A G A 0.2797 -0.009 0.03966 NA FALSE FALSE FALSE
GCST006061 0.0236 0.704 537409 outcome TRUE reported 4535_50_BST1_BST1
BST1 chr4 15209204 0.020636 7.472e-42 35365 TRUE reported
textfile 2 1 TRUE
4947 rs10758659 G A G A 0.0572 0.0023 0.45931 NA FALSE FALSE FALSE
GCST006061 0.0073 0.7531 537409 outcome TRUE reported 4990_87_GP1BA_GP1BA
GP1BA chr9 4858019 0.008242 3.929e-12 35377 TRUE reported textfile
2 1 TRUE
4948 rs10876913 A G A G -0.0562 -0.0441 0.31332 NA FALSE FALSE FALSE
GCST006061 0.0075 4.331e-09 537409 outcome TRUE reported 4990_87_GP1BA_GP1BA
GP1BA chr12 56650886 0.008916 2.911e-10 35369 TRUE reported
textfile 2 1 TRUE
4949 rs114694170 C T C T -0.0973 0.0053 0.08378 NA FALSE FALSE FALSE
GCST006061 0.0175 0.7624 537409 outcome TRUE reported 4990_87_GP1BA_GP1BA
GP1BA chr5 88884379 0.01487 6.018e-11 35344 TRUE reported textfile
2 1 TRUE
4950 rs117107082 T C T C -0.1759 0.0012 0.02592 NA FALSE FALSE FALSE

GCST006061 0.0343 0.9717 537409 outcome TRUE reported 4990_87_GP1BA_GP1BA
GP1BA chr17 4841942 0.025927 1.165e-11 35379 TRUE reported textfile
2 1 TRUE
4951 rs117240565 A G A G -0.1331 0.0153 0.04322 NA FALSE FALSE FALSE
GCST006061 0.0228 0.5006 537409 outcome TRUE reported 4990_87_GP1BA_GP1BA
GP1BA chr17 5788807 0.020389 6.658e-11 35377 TRUE reported textfile
2 1 TRUE
4952 rs12005199 A G A G 0.1019 -0.0059 0.34389 NA FALSE FALSE FALSE
GCST006061 0.0082 0.4694 537409 outcome TRUE reported 4990_87_GP1BA_GP1BA
GP1BA chr9 4763491 0.008607 2.456e-32 35374 TRUE reported textfile
2 1 TRUE
4953 rs1434282 T C T C 0.0747 -0.0021 0.26524 NA FALSE FALSE FALSE
GCST006061 0.008 0.7916 537409 outcome TRUE reported 4990_87_GP1BA_GP1BA
GP1BA chr1 199041592 0.009399 1.896e-15 35343 TRUE reported
textfile 2 1 TRUE
4954 rs1555405 A G A G -0.0732 -0.0047 0.30381 NA FALSE FALSE FALSE
GCST006061 0.0081 0.5592 537409 outcome TRUE reported 4990_87_GP1BA_GP1BA
GP1BA chr14 100710432 0.008944 2.747e-16 35380 TRUE reported
textfile 2 1 TRUE
4955 rs17834140 T C T C -0.1139 0.0031 0.04648 NA FALSE FALSE FALSE
GCST006061 0.0181 0.8631 537409 outcome TRUE reported 4990_87_GP1BA_GP1BA
GP1BA chr17 57388410 0.019578 5.964e-09 35375 TRUE reported
textfile 2 1 TRUE
4956 rs238241 G A G A 0.1685 0.0354 0.0578 NA FALSE FALSE FALSE
GCST006061 0.0273 0.1957 537409 outcome TRUE reported 4990_87_GP1BA_GP1BA
GP1BA chr17 4947777 0.017671 1.49e-21 35381 TRUE reported textfile
2 1 TRUE
4957 rs3184504 C T C T -0.1399 0.0074 0.37862 NA FALSE FALSE FALSE
GCST006061 0.0075 0.3235 537409 outcome TRUE reported 4990_87_GP1BA_GP1BA
GP1BA chr12 111446804 0.008454 1.644e-61 35371 TRUE reported
textfile 2 1 TRUE
4958 rs56407378 T G T G -0.0551 -0.0084 0.3953 NA FALSE FALSE FALSE
GCST006061 0.0073 0.2493 537409 outcome TRUE reported 4990_87_GP1BA_GP1BA
GP1BA chr3 143085132 0.008385 4.99e-11 35365 TRUE reported
textfile 2 1 TRUE
4959 rs62175229 T C T C -0.0581 4e-04 0.44909 NA FALSE FALSE FALSE
GCST006061 0.0072 0.9505 537409 outcome TRUE reported 4990_87_GP1BA_GP1BA
GP1BA chr2 159821048 0.008246 1.844e-12 35348 TRUE reported
textfile 2 1 TRUE
4960 rs72835078 T G T G 0.2536 0.023 0.08167 NA FALSE FALSE FALSE
GCST006061 0.0128 0.07198 537409 outcome TRUE reported 4990_87_GP1BA_GP1BA
GP1BA chr17 4923297 0.014843 1.906e-65 35379 TRUE reported textfile
2 1 TRUE
4961 rs78565404 T C T C 0.1258 -0.0236 0.06105 NA FALSE FALSE FALSE
GCST006061 0.0176 0.1794 537409 outcome TRUE reported 4990_87_GP1BA_GP1BA
GP1BA chr3 184372454 0.016996 1.344e-13 35358 TRUE reported
textfile 2 1 TRUE
4962 rs10759806 G A G A 0.1889 -0.0052 0.45331 NA FALSE FALSE FALSE
GCST006061 0.0072 0.4674 537409 outcome TRUE reported 4155_3_TNC_Tenascin TNC
chr9 115580424 0.008354 3.316e-113 35367 TRUE reported textfile 2
1 TRUE
4963 rs10817700 G A G A -0.3159 0.0016 0.31621 NA FALSE FALSE FALSE
GCST006061 0.0076 0.8357 537409 outcome TRUE reported 4155_3_TNC_Tenascin TNC
chr9 115013524 0.00888 1e-200 35362 TRUE reported textfile 2 1 TRUE
4964 rs10983509 G A G A 0.1477 -0.0205 0.11152 NA FALSE FALSE FALSE
GCST006061 0.0113 0.06987 537409 outcome TRUE reported 4155_3_TNC_Tenascin TNC
chr9 117074663 0.013315 1.363e-28 35369 TRUE reported textfile 2
1 TRUE
4965 rs138529507 C T C T 0.1625 -0.0161 0.0454 NA FALSE FALSE FALSE
GCST006061 0.0199 0.4167 537409 outcome TRUE reported 4155_3_TNC_Tenascin TNC
chr8 27636849 0.018382 9.555e-19 35368 TRUE reported textfile 2
1 TRUE
4966 rs2722830 T G T G 0.0857 0.0028 0.24541 NA FALSE FALSE FALSE
GCST006061 0.0084 0.7415 537409 outcome TRUE reported 4155_3_TNC_Tenascin TNC
chr9 117981819 0.009771 1.767e-18 35370 TRUE reported textfile 2
1 TRUE

4967 rs36118932 T C T C 0.1386 -0.0021 0.22461 NA FALSE FALSE FALSE
GCST006061 0.0088 0.8129 537409 outcome TRUE reported 4155_3_TNC_Tenascin TNC
chr9 114854087 0.010079 4.995e-43 35362 TRUE reported textfile 2
1 TRUE
4968 rs74742617 T C T C 0.1419 9e-04 0.10707 NA FALSE FALSE FALSE
GCST006061 0.0137 0.9467 537409 outcome TRUE reported 4155_3_TNC_Tenascin TNC
chr9 114107678 0.013793 7.968e-25 35363 TRUE reported textfile 2
1 TRUE
4969 rs760715 C T C T 0.0736 0.0035 0.26206 NA FALSE FALSE FALSE
GCST006061 0.0092 0.7055 537409 outcome TRUE reported 4155_3_TNC_Tenascin TNC
chr22 39466338 0.008534 6.424e-18 35366 TRUE reported textfile 2
1 TRUE
4970 rs79098169 T C T C 0.1609 -0.0368 0.04091 NA FALSE FALSE FALSE
GCST006061 0.0338 0.2772 537409 outcome TRUE reported 4155_3_TNC_Tenascin TNC
chr9 111231279 0.021201 3.217e-14 35370 TRUE reported textfile 2
1 TRUE
4971 rs9668337 A G A G -0.051 0.0092 0.26322 NA FALSE FALSE FALSE
GCST006061 0.0079 0.2411 537409 outcome TRUE reported 4155_3_TNC_Tenascin TNC
chr12 26273405 0.008516 2.112e-09 35359 TRUE reported textfile 2
1 TRUE
4972 rs10761339 A G A G 0.0551 -0.0072 0.47971 NA FALSE FALSE FALSE
GCST006061 0.0072 0.3198 537409 outcome TRUE reported 9867_23_FBP2_F16P2
FBP2 chr9 94566031 0.008009 6.002e-12 35349 TRUE reported
textfile 2 1 TRUE
4973 rs10922098 T C T C -0.1606 -0.009 0.40011 NA FALSE FALSE FALSE
GCST006061 0.0075 0.2275 537409 outcome TRUE reported 9867_23_FBP2_F16P2
FBP2 chr1 196695521 0.008133 8.439e-87 35338 TRUE reported
textfile 2 1 TRUE
4974 rs2686395 C T C T -0.0778 -0.0077 0.3101 NA FALSE FALSE FALSE
GCST006061 0.0074 0.2965 537409 outcome TRUE reported 9867_23_FBP2_F16P2
FBP2 chr3 165767231 0.008623 1.842e-19 35339 TRUE reported
textfile 2 1 TRUE
4975 rs10761727 T C T C -0.0569 -0.0525 0.47803 NA FALSE FALSE FALSE
GCST006061 0.0071 1.359e-13 537409 outcome TRUE reported
10565_19_SLITRK3_SLIK3 SLITRK3 chr10 63235733 0.008181 3.523e-12 35339
TRUE reported textfile 2 1 TRUE
4976 rs114464925 T G T G 0.1608 -0.0076 0.03638 NA FALSE FALSE FALSE
GCST006061 0.0217 0.7284 537409 outcome TRUE reported 10565_19_SLITRK3_SLIK3
SLITRK3 chr3 166349458 0.022172 4.095e-13 35342 TRUE reported
textfile 2 1 TRUE
4977 rs148190310 A G A G 0.1273 -0.0417 0.03692 NA FALSE FALSE FALSE
GCST006061 0.0296 0.1596 537409 outcome TRUE reported 10565_19_SLITRK3_SLIK3
SLITRK3 chr7 148867046 0.022086 8.221e-09 35362 TRUE reported
textfile 2 1 TRUE
4978 rs2036291 G A G A 0.0456 -0.0027 0.44401 NA FALSE FALSE FALSE
GCST006061 0.0071 0.7047 537409 outcome TRUE reported 10565_19_SLITRK3_SLIK3
SLITRK3 chr3 152349004 0.008326 4.328e-08 35337 TRUE reported
textfile 2 1 TRUE
4979 rs2111557 T C T C 0.0525 0.0054 0.48945 NA FALSE FALSE FALSE
GCST006061 0.0071 0.4475 537409 outcome TRUE reported 10565_19_SLITRK3_SLIK3
SLITRK3 chr3 169607833 0.008297 2.491e-10 35345 TRUE reported
textfile 2 1 TRUE
4980 rs2259820 T C T C -0.0645 -0.0095 0.2883 NA FALSE FALSE FALSE
GCST006061 0.0076 0.2142 537409 outcome TRUE reported 10565_19_SLITRK3_SLIK3
SLITRK3 chr12 120997539 0.009002 7.765e-13 35353 TRUE reported
textfile 2 1 TRUE
4981 rs273492 A G A G -0.0547 -0.0131 0.24761 NA FALSE FALSE FALSE
GCST006061 0.0083 0.1152 537409 outcome TRUE reported 10565_19_SLITRK3_SLIK3
SLITRK3 chr19 18127072 0.009491 8.246e-09 35342 TRUE reported
textfile 2 1 TRUE
4982 rs36170987 T G T G -0.0686 0 0.46293 NA FALSE FALSE FALSE GCST006061
0.0092 0.9966 537409 outcome TRUE reported 10565_19_SLITRK3_SLIK3 SLITRK3
chr7 259884 0.008187 5.327e-17 35310 TRUE reported textfile 2 1
TRUE
4983 rs41278228 A G A G 0.0838 9e-04 0.20241 NA FALSE FALSE FALSE
GCST006061 0.0101 0.9283 537409 outcome TRUE reported 10565_19_SLITRK3_SLIK3

SLITRK3 chr20 63963478 0.010276 3.493e-16 34853 TRUE reported textfile 2 1 TRUE

4984 rs56292801 A G A G 0.0682 -0.0277 0.25442 NA FALSE FALSE FALSE GCST006061 0.0083 0.0008887 537409 outcome TRUE reported 10565_19_SLITRK3_SLIK3 SLITRK3 chr16 88468933 0.009478 6.234e-13 35308 TRUE reported textfile 2 1 TRUE

4985 rs57526361 A G A G -0.0501 -0.0122 0.36365 NA FALSE FALSE FALSE GCST006061 0.0077 0.112 537409 outcome TRUE reported 10565_19_SLITRK3_SLIK3 SLITRK3 chr16 30635175 0.00848 3.466e-09 35358 TRUE reported textfile 2 1 TRUE

4986 rs722740 T C T C -0.0486 -1.00E-04 0.39677 NA FALSE FALSE FALSE GCST006061 0.0072 0.9879 537409 outcome TRUE reported 10565_19_SLITRK3_SLIK3 SLITRK3 chr8 115949755 0.008345 5.747e-09 35337 TRUE reported textfile 2 1 TRUE

4987 rs74397112 T C T C 0.0621 0.0284 0.16588 NA FALSE FALSE FALSE GCST006061 0.0109 0.009008 537409 outcome TRUE reported 10565_19_SLITRK3_SLIK3 SLITRK3 chr12 132517525 0.011039 1.849e-08 34856 TRUE reported textfile 2 1 TRUE

4988 rs7540799 T C T C 0.0631 0.0158 0.15738 NA FALSE FALSE FALSE GCST006061 0.0108 0.1439 537409 outcome TRUE reported 10565_19_SLITRK3_SLIK3 SLITRK3 chr1 151825217 0.011187 1.694e-08 35349 TRUE reported textfile 2 1 TRUE

4989 rs79598313 T C T C 0.2346 0.0118 0.0134 NA FALSE FALSE FALSE GCST006061 0.0264 0.6549 537409 outcome TRUE reported 10565_19_SLITRK3_SLIK3 SLITRK3 chr1 26958422 0.034729 1.426e-11 35358 TRUE reported textfile 2 1 TRUE

4990 rs80336735 C A C A -0.1232 0.0272 0.09881 NA FALSE FALSE FALSE GCST006061 0.0145 0.06043 537409 outcome TRUE reported 10565_19_SLITRK3_SLIK3 SLITRK3 chr3 164927647 0.013889 7.278e-19 35330 TRUE reported textfile 2 1 TRUE

4991 rs9462852 T C T C -0.1313 0.0377 0.05885 NA FALSE FALSE FALSE GCST006061 0.0169 0.02581 537409 outcome TRUE reported 10565_19_SLITRK3_SLIK3 SLITRK3 chr6 42939589 0.017678 1.109e-13 35355 TRUE reported textfile 2 1 TRUE

4992 rs10761737 C T C T -0.0543 -0.0474 0.43543 NA FALSE FALSE FALSE GCST006061 0.0072 4.478e-11 537409 outcome TRUE reported 3077_66_F10_Coagulation_Factor_Xa F10 chr10 63292445 0.00834 7.459e-11 35351 TRUE reported textfile 2 1 TRUE

4993 rs11793434 G T G T 0.0719 -0.0031 0.16253 NA FALSE FALSE FALSE GCST006061 0.0104 0.7683 537409 outcome TRUE reported 3077_66_F10_Coagulation_Factor_Xa F10 chr9 114316413 0.011273 1.792e-10 35362 TRUE reported textfile 2 1 TRUE

4994 rs1260326 C T C T -0.0655 -0.0069 0.34132 NA FALSE FALSE FALSE GCST006061 0.0073 0.3401 537409 outcome TRUE reported 3077_66_F10_Coagulation_Factor_Xa F10 chr2 27508073 0.008775 8.371e-14 35373 TRUE reported textfile 2 1 TRUE

4995 rs1623718 T C T C 0.0692 -0.0113 0.46457 NA FALSE FALSE FALSE GCST006061 0.0072 0.117 537409 outcome TRUE reported 3077_66_F10_Coagulation_Factor_Xa F10 chr9 114264788 0.008336 1.027e-16 35363 TRUE reported textfile 2 1 TRUE

4996 rs211833 G A G A 0.0452 -6.00E-04 0.47677 NA FALSE FALSE FALSE GCST006061 0.0071 0.9289 537409 outcome TRUE reported 3077_66_F10_Coagulation_Factor_Xa F10 chr20 39850639 0.008281 4.809e-08 35376 TRUE reported textfile 2 1 TRUE

4997 rs2306956 A G A G 0.0809 -0.0031 0.16423 NA FALSE FALSE FALSE GCST006061 0.0088 0.7234 537409 outcome TRUE reported 3077_66_F10_Coagulation_Factor_Xa F10 chr9 114228297 0.011237 6.053e-13 35362 TRUE reported textfile 2 1 TRUE

4998 rs2954021 G A G A -0.0547 -0.0016 0.45781 NA FALSE FALSE FALSE GCST006061 0.0071 0.8249 537409 outcome TRUE reported 3077_66_F10_Coagulation_Factor_Xa F10 chr8 125469835 0.008342 5.494e-11 35368 TRUE reported textfile 2 1 TRUE

4999 rs8002283 G A G A -0.0511 -0.0048 0.49401 NA FALSE FALSE FALSE GCST006061 0.0074 0.5139 537409 outcome TRUE reported 3077_66_F10_Coagulation_Factor_Xa F10 chr13 112999265 0.008418 1.274e-09 34894 TRUE reported textfile 2 1 TRUE

5000 rs1740073 C T C T -0.0582 0.0163 0.38053 NA FALSE FALSE FALSE GCST006061 0.0081 0.04527 537409 outcome TRUE reported 2597_8_VEGFA_VEGF VEGFA chr6 43979661 0.008561 1.057e-11 35364 TRUE reported textfile 2 1 TRUE
5001 rs7692 A C A C -0.0564 -0.0148 0.27906 NA FALSE FALSE FALSE GCST006061 0.0077 0.05366 537409 outcome TRUE reported 2597_8_VEGFA_VEGF VEGFA chr6 43336269 0.009198 8.679e-10 35367 TRUE reported textfile 2 1 TRUE
5002 rs9472159 A C A C -0.2773 0.0076 0.49356 NA FALSE FALSE FALSE GCST006061 0.0073 0.2972 537409 outcome TRUE reported 2597_8_VEGFA_VEGF VEGFA chr6 43951958 0.008171 1e-200 35363 TRUE reported textfile 2 1 TRUE
5003 rs10888208 G T G T 0.4584 0.0632 0.01397 NA FALSE FALSE FALSE GCST006061 0.0409 0.1227 537409 outcome TRUE reported 3535_84_DKK1_DKK1 DKK1 chr10 47096668 0.03542 2.615e-38 35333 TRUE reported textfile 2 1 TRUE
5004 rs12468189 G A G A 0.0465 -0.0052 0.40103 NA FALSE FALSE FALSE GCST006061 0.0074 0.4799 537409 outcome TRUE reported 3535_84_DKK1_DKK1 DKK1 chr2 31255954 0.008516 4.753e-08 35369 TRUE reported textfile 2 1 TRUE
5005 rs141099634 C T C T 0.6876 -0.1632 0.0118 NA FALSE FALSE FALSE GCST006061 0.1635 0.3183 537409 outcome TRUE reported 3535_84_DKK1_DKK1 DKK1 chr10 50347786 0.038287 4.059e-72 35353 TRUE reported textfile 2 1 TRUE
5006 rs145591269 G A G A 0.1879 0.0025 0.01489 NA FALSE FALSE FALSE GCST006061 0.0441 0.9541 537409 outcome TRUE reported 3535_84_DKK1_DKK1 DKK1 chr10 61660551 0.034032 3.367e-08 35354 TRUE reported textfile 2 1 TRUE
5007 rs150513777 G A G A 0.4508 -0.0025 0.0321 NA FALSE FALSE FALSE GCST006061 0.0255 0.9229 537409 outcome TRUE reported 3535_84_DKK1_DKK1 DKK1 chr10 52474587 0.0234 1.054e-82 35355 TRUE reported textfile 2 1 TRUE
5008 rs34416903 C T C T 0.0523 -0.001 0.30061 NA FALSE FALSE FALSE GCST006061 0.0084 0.9015 537409 outcome TRUE reported 3535_84_DKK1_DKK1 DKK1 chr16 88492837 0.00901 6.45e-09 35318 TRUE reported textfile 2 1 TRUE
5009 rs4648825 C T C T 0.0587 0.0069 0.19892 NA FALSE FALSE FALSE GCST006061 0.0099 0.4849 537409 outcome TRUE reported 3535_84_DKK1_DKK1 DKK1 chr1 2304900 0.010456 1.975e-08 35328 TRUE reported textfile 2 1 TRUE
5010 rs61851099 G T G T 0.2249 -0.004 0.01831 NA FALSE FALSE FALSE GCST006061 0.032 0.9004 537409 outcome TRUE reported 3535_84_DKK1_DKK1 DKK1 chr10 55341083 0.03116 5.292e-13 35358 TRUE reported textfile 2 1 TRUE
5011 rs7101062 G A G A -0.2753 -0.036 0.03623 NA FALSE FALSE FALSE GCST006061 0.0364 0.3234 537409 outcome TRUE reported 3535_84_DKK1_DKK1 DKK1 chr10 51837325 0.022108 1.353e-35 35358 TRUE reported textfile 2 1 TRUE
5012 rs75580312 A G A G 0.7202 0.0425 0.01464 NA FALSE FALSE FALSE GCST006061 0.0478 0.3745 537409 outcome TRUE reported 3535_84_DKK1_DKK1 DKK1 chr10 51875766 0.033293 8.971e-104 35362 TRUE reported textfile 2 1 TRUE
5013 rs11053499 C A C A -0.1281 -0.0034 0.06278 NA FALSE FALSE FALSE GCST006061 0.0147 0.8143 537409 outcome TRUE reported 4332_6_CLEC1B_CLC1B CLEC1B chr12 9895466 0.016944 4.024e-14 35385 TRUE reported textfile 2 1 TRUE
5014 rs114694170 C T C T -0.0903 0.0053 0.08378 NA FALSE FALSE FALSE GCST006061 0.0175 0.7624 537409 outcome TRUE reported 4332_6_CLEC1B_CLC1B CLEC1B chr5 88884379 0.014769 9.719e-10 35344 TRUE reported textfile 2 1 TRUE
5015 rs3792386 A G A G -0.047 0.0089 0.35198 NA FALSE FALSE FALSE GCST006061 0.0073 0.2182 537409 outcome TRUE reported 4332_6_CLEC1B_CLC1B CLEC1B chr3 123134833 0.008542 3.754e-08 35367 TRUE reported textfile 2 1 TRUE
5016 rs659928 A G A G 0.1722 -8.00E-04 0.22486 NA FALSE FALSE FALSE GCST006061 0.0082 0.9221 537409 outcome TRUE reported 4332_6_CLEC1B_CLC1B CLEC1B chr12 9996104 0.009737 5.522e-70 35387 TRUE reported textfile

2 1 TRUE
5017 rs10922098 T C T C -0.0557 -0.009 0.40011 NA FALSE FALSE FALSE
GCST006061 0.0075 0.2275 537409 outcome TRUE reported 16618_7_CD69_CD69
CD69 chr1 196695521 0.008139 7.728e-12 35338 TRUE reported
textfile 2 1 TRUE
5018 rs1354034 C T C T 0.1006 0.0088 0.38119 NA FALSE FALSE FALSE
GCST006061 0.0073 0.2248 537409 outcome TRUE reported 10447_18_PABPC3_PABP3
PABPC3 chr3 56815721 0.008305 8.989e-34 35344 TRUE reported
textfile 2 1 TRUE
5019 rs4980320 C T C T 0.0578 0.0041 0.46986 NA FALSE FALSE FALSE
GCST006061 0.0076 0.5858 537409 outcome TRUE reported 10447_18_PABPC3_PABP3
PABPC3 chr11 198986 0.008105 9.966e-13 35340 TRUE reported textfile
2 1 TRUE
5020 rs12313283 A G A G 0.0893 0.0092 0.07132 NA FALSE FALSE FALSE
GCST006061 0.0186 0.6186 537409 outcome TRUE reported 10339_48_ENO2_NSE
ENO2 chr12 8363333 0.016034 2.554e-08 35368 TRUE reported textfile
2 1 TRUE
5021 rs2239165 C T C T 0.1562 -0.0132 0.28884 NA FALSE FALSE FALSE
GCST006061 0.0078 0.09017 537409 outcome TRUE reported 10339_48_ENO2_NSE
ENO2 chr12 6914702 0.009027 4.468e-67 35378 TRUE reported textfile
2 1 TRUE
5022 rs4734879 G A G A -0.148 0.0194 0.21866 NA FALSE FALSE FALSE
GCST006061 0.0079 0.01412 537409 outcome TRUE reported 10339_48_ENO2_NSE
ENO2 chr8 105570896 0.009929 2.999e-50 35353 TRUE reported
textfile 2 1 TRUE
5023 rs6580981 A G A G -0.0512 4e-04 0.47567 NA FALSE FALSE FALSE
GCST006061 0.0072 0.9564 537409 outcome TRUE reported 10339_48_ENO2_NSE
ENO2 chr12 54329244 0.008224 4.793e-10 35360 TRUE reported
textfile 2 1 TRUE
5024 rs1354034 C T C T 0.1787 0.0088 0.38119 NA FALSE FALSE FALSE
GCST006061 0.0073 0.2248 537409 outcome TRUE reported 11608_5_MAP1LC3B_MLP3B
MAP1LC3B chr3 56815721 0.008418 5.287e-100 35347 TRUE reported
textfile 2 1 TRUE
5025 rs16975847 A G A G -0.1274 0.0196 0.12656 NA FALSE FALSE FALSE
GCST006061 0.0129 0.1288 537409 outcome TRUE reported 11608_5_MAP1LC3B_MLP3B
MAP1LC3B chr16 75555692 0.012359 6.473e-25 35354 TRUE reported
textfile 2 1 TRUE
5026 rs185009887 A G A G -0.1544 -0.0595 0.02519 NA FALSE FALSE FALSE
GCST006061 0.0496 0.2302 537409 outcome TRUE reported 11608_5_MAP1LC3B_MLP3B
MAP1LC3B chr17 5848332 0.026641 6.806e-09 35365 TRUE reported
textfile 2 1 TRUE
5027 rs3811444 T C T C -0.0608 0.0103 0.34232 NA FALSE FALSE FALSE
GCST006061 0.0077 0.1812 537409 outcome TRUE reported 11608_5_MAP1LC3B_MLP3B
MAP1LC3B chr1 247876149 0.008731 3.317e-12 35147 TRUE reported
textfile 2 1 TRUE
5028 rs55665228 T C T C 0.0612 0.0198 0.16169 NA FALSE FALSE FALSE
GCST006061 0.01 0.04717 537409 outcome TRUE reported 11608_5_MAP1LC3B_MLP3B
MAP1LC3B chr9 96472047 0.011199 4.631e-08 35336 TRUE reported
textfile 2 1 TRUE
5029 rs7926172 A G A G 0.0737 -0.0023 0.23069 NA FALSE FALSE FALSE
GCST006061 0.0093 0.8091 537409 outcome TRUE reported 11608_5_MAP1LC3B_MLP3B
MAP1LC3B chr11 225180 0.009735 3.712e-14 35343 TRUE reported
textfile 2 1 TRUE
5030 rs10761785 T G T G 0.0566 -0.0598 0.44261 NA FALSE FALSE FALSE
GCST006061 0.0085 1.801e-12 537409 outcome TRUE reported 9263_57_SIAE_SIAE
SIAE chr10 63559006 0.008214 5.541e-12 35351 TRUE reported
textfile 2 1 TRUE
5031 rs10812628 A G A G -0.0824 0.0026 0.2427 NA FALSE FALSE FALSE
GCST006061 0.0084 0.7586 537409 outcome TRUE reported 9263_57_SIAE_SIAE
SIAE chr9 27631392 0.009499 4.14e-18 35358 TRUE reported
textfile 2 1 TRUE
5032 rs12191772 A G A G 0.0801 -0.0041 0.16169 NA FALSE FALSE FALSE
GCST006061 0.0092 0.6566 537409 outcome TRUE reported 9263_57_SIAE_SIAE
SIAE chr6 136938587 0.011069 4.612e-13 35364 TRUE reported
textfile 2 1 TRUE
5033 rs1271816 T C T C 0.0637 0.0119 0.32958 NA FALSE FALSE FALSE

GCST006061 0.0079 0.1319 537409 outcome TRUE reported 9263_57_SIAE_SIAE
SIAE chr14 92730774 0.008637 1.642e-13 35361 TRUE reported
textfile 2 1 TRUE
5034 rs2808466 C T C T 0.0978 -0.0081 0.11154 NA FALSE FALSE FALSE
GCST006061 0.0104 0.4395 537409 outcome TRUE reported 9263_57_SIAE_SIAE
SIAE chr1 207100406 0.012993 5.177e-14 35369 TRUE reported
textfile 2 1 TRUE
5035 rs302644 T G T G 0.0485 -0.0121 0.29783 NA FALSE FALSE FALSE
GCST006061 0.0077 0.117 537409 outcome TRUE reported 9263_57_SIAE_SIAE
SIAE chr11 88180303 0.008891 4.894e-08 35351 TRUE reported
textfile 2 1 TRUE
5036 rs34533614 A G A G -0.0859 0.0013 0.14678 NA FALSE FALSE FALSE
GCST006061 0.0107 0.9016 537409 outcome TRUE reported 9263_57_SIAE_SIAE
SIAE chr10 98429485 0.011577 1.17e-13 35368 TRUE reported
textfile 2 1 TRUE
5037 rs61269268 C A C A 0.057 -0.002 0.20448 NA FALSE FALSE FALSE
GCST006061 0.0098 0.835 537409 outcome TRUE reported 9263_57_SIAE_SIAE
SIAE chr14 91775008 0.010117 1.759e-08 35360 TRUE reported
textfile 2 1 TRUE
5038 rs8100156 C A C A -0.0642 -0.0113 0.16666 NA FALSE FALSE FALSE
GCST006061 0.0106 0.2827 537409 outcome TRUE reported 9263_57_SIAE_SIAE
SIAE chr19 2005703 0.011014 5.574e-09 35316 TRUE reported textfile
2 1 TRUE
5039 rs112963922 T C T C -0.9147 0.0542 0.01119 NA FALSE FALSE FALSE
GCST006061 0.0399 0.1741 537409 outcome TRUE reported
3580_25_SERPINA1_a1_Antitrypsin SERPINA1 chr14 94411531 0.039496 1.172e-118
35371 TRUE reported textfile 2 1 TRUE
5040 rs117562583 G A G A 0.183 -0.0087 0.03103 NA FALSE FALSE FALSE
GCST006061 0.0268 0.7445 537409 outcome TRUE reported
3580_25_SERPINA1_a1_Antitrypsin SERPINA1 chr14 94186080 0.024623 1.069e-13
35372 TRUE reported textfile 2 1 TRUE
5041 rs1260326 C T C T 0.0653 -0.0069 0.34132 NA FALSE FALSE FALSE
GCST006061 0.0073 0.3401 537409 outcome TRUE reported
3580_25_SERPINA1_a1_Antitrypsin SERPINA1 chr2 27508073 0.008638 4.049e-14
35373 TRUE reported textfile 2 1 TRUE
5042 rs148597299 C T C T 0.2293 0.0315 0.01958 NA FALSE FALSE FALSE
GCST006061 0.0292 0.2815 537409 outcome TRUE reported
3580_25_SERPINA1_a1_Antitrypsin SERPINA1 chr14 94134775 0.030774 9.256e-14
35372 TRUE reported textfile 2 1 TRUE
5043 rs17091144 T C T C 0.3189 0.0123 0.02967 NA FALSE FALSE FALSE
GCST006061 0.0175 0.4816 537409 outcome TRUE reported
3580_25_SERPINA1_a1_Antitrypsin SERPINA1 chr14 94597380 0.023965 2.107e-40
35372 TRUE reported textfile 2 1 TRUE
5044 rs17825092 G A G A 0.108 0.0127 0.07767 NA FALSE FALSE FALSE
GCST006061 0.0147 0.3876 537409 outcome TRUE reported
3580_25_SERPINA1_a1_Antitrypsin SERPINA1 chr14 94424021 0.01576 7.246e-12
35370 TRUE reported textfile 2 1 TRUE
5045 rs2402227 G A G A -0.2132 0.0251 0.02072 NA FALSE FALSE FALSE
GCST006061 0.0294 0.3937 537409 outcome TRUE reported
3580_25_SERPINA1_a1_Antitrypsin SERPINA1 chr14 92973564 0.029778 8.083e-13
35366 TRUE reported textfile 2 1 TRUE
5046 rs660240 C T C T 0.1567 -0.0136 0.205 NA FALSE FALSE FALSE
GCST006061 0.0088 0.1214 537409 outcome TRUE reported
3580_25_SERPINA1_a1_Antitrypsin SERPINA1 chr1 109275216 0.010122 4.651e-54
35345 TRUE reported textfile 2 1 TRUE
5047 rs704 A G A G 0.0722 -0.0155 0.49189 NA FALSE FALSE FALSE GCST006061
0.0072 0.03144 537409 outcome TRUE reported 3580_25_SERPINA1_a1_Antitrypsin
SERPINA1 chr17 28367840 0.008167 9.508e-19 35360 TRUE reported
textfile 2 1 TRUE
5048 rs7160243 A G A G 0.2748 0.0168 0.01675 NA FALSE FALSE FALSE
GCST006061 0.0323 0.6029 537409 outcome TRUE reported
3580_25_SERPINA1_a1_Antitrypsin SERPINA1 chr14 94504220 0.032737 4.693e-17
35372 TRUE reported textfile 2 1 TRUE
5049 rs72702354 G A G A -0.8024 0.0128 0.02988 NA FALSE FALSE FALSE
GCST006061 0.0216 0.5537 537409 outcome TRUE reported
3580_25_SERPINA1_a1_Antitrypsin SERPINA1 chr14 94176870 0.024505 1e-200

35372 TRUE reported textfile 2 1 TRUE

5050 rs10762487 T C T C 0.067 -0.0083 0.41214 NA FALSE FALSE FALSE GCST006061 0.0075 0.2685 537409 outcome TRUE reported 6304_8_C1QTNF1_C1QT1 C1QTNF1 chr10 71887908 0.008359 1.102e-15 35374 TRUE reported textfile 2 1 TRUE

5051 rs114003685 T C T C -0.3322 -0.0112 0.0233 NA FALSE FALSE FALSE GCST006061 0.0293 0.7027 537409 outcome TRUE reported 6304_8_C1QTNF1_C1QT1 C1QTNF1 chr1 109580328 0.026963 7.001e-35 35346 TRUE reported textfile 2 1 TRUE

5052 rs12023150 T C T C 0.0703 -0.0109 0.2844 NA FALSE FALSE FALSE GCST006061 0.0083 0.1861 537409 outcome TRUE reported 6304_8_C1QTNF1_C1QT1 C1QTNF1 chr1 109282969 0.009208 2.258e-14 35344 TRUE reported textfile 2 1 TRUE

5053 rs140183833 A G A G -0.1864 -0.0363 0.01872 NA FALSE FALSE FALSE GCST006061 0.0307 0.2369 537409 outcome TRUE reported 6304_8_C1QTNF1_C1QT1 C1QTNF1 chr17 47710194 0.030123 6.092e-10 35364 TRUE reported textfile 2 1 TRUE

5054 rs2289702 T C T C 0.0965 -0.0406 0.10564 NA FALSE FALSE FALSE GCST006061 0.0124 0.001103 537409 outcome TRUE reported 6304_8_C1QTNF1_C1QT1 C1QTNF1 chr15 78944951 0.013386 5.625e-13 35358 TRUE reported textfile 2 1 TRUE

5055 rs5848 T C T C -0.1666 0.0191 0.25559 NA FALSE FALSE FALSE GCST006061 0.0081 0.01926 537409 outcome TRUE reported 6304_8_C1QTNF1_C1QT1 C1QTNF1 chr17 44352876 0.009366 8.698e-71 35370 TRUE reported textfile 2 1 TRUE

5056 rs7210348 C T C T 0.1743 0.0519 0.0212 NA FALSE FALSE FALSE GCST006061 0.0199 0.009034 537409 outcome TRUE reported 6304_8_C1QTNF1_C1QT1 C1QTNF1 chr17 42045639 0.028563 1.046e-09 35387 TRUE reported textfile 2 1 TRUE

5057 rs72802342 A C A C -0.1171 0.0024 0.06858 NA FALSE FALSE FALSE GCST006061 0.0147 0.8687 537409 outcome TRUE reported 6304_8_C1QTNF1_C1QT1 C1QTNF1 chr16 75200974 0.016419 9.88e-13 35363 TRUE reported textfile 2 1 TRUE

5058 rs7538475 T C T C -0.1023 -0.0017 0.11233 NA FALSE FALSE FALSE GCST006061 0.0107 0.8756 537409 outcome TRUE reported 6304_8_C1QTNF1_C1QT1 C1QTNF1 chr1 109149257 0.013107 5.961e-15 35342 TRUE reported textfile 2 1 TRUE

5059 rs8176693 T C T C 0.0975 0.0144 0.06484 NA FALSE FALSE FALSE GCST006061 0.0129 0.2638 537409 outcome TRUE reported 6304_8_C1QTNF1_C1QT1 C1QTNF1 chr9 133262254 0.01672 5.5e-09 35371 TRUE reported textfile 2 1 TRUE

5060 rs943616 C T C T -0.0542 0.0156 0.23142 NA FALSE FALSE FALSE GCST006061 0.0083 0.0591 537409 outcome TRUE reported 6304_8_C1QTNF1_C1QT1 C1QTNF1 chr9 110131570 0.00977 2.899e-08 35370 TRUE reported textfile 2 1 TRUE

5061 rs10762487 T C T C 0.0978 -0.0083 0.41214 NA FALSE FALSE FALSE GCST006061 0.0075 0.2685 537409 outcome TRUE reported 4992_49_GRN_GRN GRN chr10 71887908 0.008373 1.611e-31 35374 TRUE reported textfile 2 1 TRUE

5062 rs11102684 A G A G 0.0681 -0.0112 0.29109 NA FALSE FALSE FALSE GCST006061 0.008 0.1618 537409 outcome TRUE reported 4992_49_GRN_GRN GRN chr1 108951277 0.009247 1.781e-13 35346 TRUE reported textfile 2 1 TRUE

5063 rs13240039 G A G A 0.0621 0.0129 0.16625 NA FALSE FALSE FALSE GCST006061 0.0107 0.2276 537409 outcome TRUE reported 4992_49_GRN_GRN GRN chr7 130374641 0.010907 1.245e-08 35357 TRUE reported textfile 2 1 TRUE

5064 rs140183833 A G A G -0.2227 -0.0363 0.01872 NA FALSE FALSE FALSE GCST006061 0.0307 0.2369 537409 outcome TRUE reported 4992_49_GRN_GRN GRN chr17 47710194 0.030225 1.732e-13 35364 TRUE reported textfile 2 1 TRUE

5065 rs145803034 T G T G -0.2101 -0.0167 0.01575 NA FALSE FALSE FALSE GCST006061 0.0713 0.8142 537409 outcome TRUE reported 4992_49_GRN_GRN GRN chr1 112266021 0.034395 1.006e-09 35341 TRUE reported textfile 2 1 TRUE

5066 rs187542824 T G T G -0.3394 0.0575 0.01477 NA FALSE FALSE FALSE

GCST006061 0.047 0.2216 537409 outcome TRUE reported 4992_49_GRN_GRN GRN
chr1 109086421 0.035349 7.889e-22 35342 TRUE reported textfile 2
1 TRUE
5067 rs35358959 A G A G -0.5003 -0.0228 0.07682 NA FALSE FALSE FALSE
GCST006061 0.0135 0.09084 537409 outcome TRUE reported 4992_49_GRN_GRN GRN
chr1 109281628 0.01559 1e-200 35344 TRUE reported textfile 2 1 TRUE
5068 rs35645512 T C T C -0.1561 -0.0207 0.02791 NA FALSE FALSE FALSE
GCST006061 0.0255 0.4175 537409 outcome TRUE reported 4992_49_GRN_GRN GRN
chr1 111763740 0.025899 1.668e-09 35343 TRUE reported textfile 2
1 TRUE
5069 rs4397962 C T C T 0.0547 0.001 0.22059 NA FALSE FALSE FALSE
GCST006061 0.0084 0.9021 537409 outcome TRUE reported 4992_49_GRN_GRN GRN
chr13 28112947 0.009899 3.277e-08 35359 TRUE reported textfile 2
1 TRUE
5070 rs5848 T C T C -0.2177 0.0191 0.25559 NA FALSE FALSE FALSE GCST006061
0.0081 0.01926 537409 outcome TRUE reported 4992_49_GRN_GRN GRN chr17
44352876 0.00937 2.052e-119 35370 TRUE reported textfile 2 1 TRUE
5071 rs7210348 C T C T 0.2164 0.0519 0.0212 NA FALSE FALSE FALSE
GCST006061 0.0199 0.009034 537409 outcome TRUE reported 4992_49_GRN_GRN GRN
chr17 42045639 0.028655 4.293e-14 35387 TRUE reported textfile 2
1 TRUE
5072 rs72687286 T C T C -0.1225 0.0097 0.03809 NA FALSE FALSE FALSE
GCST006061 0.0175 0.5814 537409 outcome TRUE reported 4992_49_GRN_GRN GRN
chr1 109962371 0.021936 2.345e-08 35349 TRUE reported textfile 2
1 TRUE
5073 rs72802342 A C A C -0.1711 0.0024 0.06858 NA FALSE FALSE FALSE
GCST006061 0.0147 0.8687 537409 outcome TRUE reported 4992_49_GRN_GRN GRN
chr16 75200974 0.016397 1.719e-25 35363 TRUE reported textfile 2
1 TRUE
5074 rs8176743 T C T C 0.1223 0.017 0.06488 NA FALSE FALSE FALSE
GCST006061 0.0128 0.1834 537409 outcome TRUE reported 4992_49_GRN_GRN GRN
chr9 133256028 0.016713 2.522e-13 35370 TRUE reported textfile 2
1 TRUE
5075 rs10766205 A G A G 0.2492 0.0083 0.2753 NA FALSE FALSE FALSE
GCST006061 0.008 0.2962 537409 outcome TRUE reported 17170_15_CALCB_CALCB
CALCB chr11 15070774 0.008738 6.579e-179 35369 TRUE reported
textfile 2 1 TRUE
5076 rs10832218 C T C T -0.0502 -0.0033 0.39808 NA FALSE FALSE FALSE
GCST006061 0.0092 0.7209 537409 outcome TRUE reported 17170_15_CALCB_CALCB
CALCB chr11 14159628 0.008074 5.056e-10 35370 TRUE reported
textfile 2 1 TRUE
5077 rs10922098 T C T C -0.0682 -0.009 0.40011 NA FALSE FALSE FALSE
GCST006061 0.0075 0.2275 537409 outcome TRUE reported 17170_15_CALCB_CALCB
CALCB chr1 196695521 0.008064 2.732e-17 35338 TRUE reported
textfile 2 1 TRUE
5078 rs10766303 C T C T -0.0464 -1.00E-04 0.48745 NA FALSE FALSE FALSE
GCST006061 0.0071 0.9926 537409 outcome TRUE reported 2851_63_C5_C5a C5
chr11 16217830 0.008018 7.174e-09 35369 TRUE reported textfile 2
1 TRUE
5079 rs10819449 G A G A 0.0843 0.0197 0.1044 NA FALSE FALSE FALSE
GCST006061 0.0116 0.09087 537409 outcome TRUE reported 2851_63_C5_C5a C5
chr9 128819825 0.013147 1.434e-10 35362 TRUE reported textfile 2
1 TRUE
5080 rs17220750 A G A G 0.186 -0.0347 0.10741 NA FALSE FALSE FALSE
GCST006061 0.0127 0.006304 537409 outcome TRUE reported 2851_63_C5_C5a C5
chr9 121025721 0.012762 4.105e-48 35376 TRUE reported textfile 2
1 TRUE
5081 rs8178824 T C T C 0.3399 -0.0854 0.01066 NA FALSE FALSE FALSE
GCST006061 0.023 0.0002035 537409 outcome TRUE reported 2851_63_C5_C5a C5
chr17 66228657 0.03859 1.274e-18 35369 TRUE reported textfile 2 1
TRUE
5082 rs10766492 A C A C 0.1341 -0.0115 0.11235 NA FALSE FALSE FALSE
GCST006061 0.0121 0.339 537409 outcome TRUE reported 15516_12_SAA4_SAA_4
SAA4 chr11 18692916 0.015302 1.89e-18 35379 TRUE reported
textfile 2 1 TRUE
5083 rs117147437 A G A G 1.1372 0.0213 0.03847 NA FALSE FALSE FALSE

GCST006061	0.0249	0.3922	537409	outcome	TRUE	reported	15516_12_SAA4_SAA_4	SAA4	chr11	17943896	0.017098	1e-200	35387	TRUE	reported	textfile	2	1	TRUE
5084	rs12418493	C	T	C	T	0.3133	-0.0256	0.03471	NA	FALSE	FALSE	FALSE	GCST006061	0.0212	0.2277	537409	outcome	TRUE	reported	15516_12_SAA4_SAA_4	SAA4	chr11	16952634	0.026259	8.165e-33	35368	TRUE	reported	textfile	2	1	TRUE
5085	rs12720922	A	G	A	G	-0.0965	-0.0123	0.19419	NA	FALSE	FALSE	FALSE	GCST006061	0.0093	0.186	537409	outcome	TRUE	reported	15516_12_SAA4_SAA_4	SAA4	chr16	56966973	0.01117	5.662e-18	35369	TRUE	reported	textfile	2	1	TRUE
5086	rs1356979	C	T	C	T	-0.3806	-0.0085	0.39372	NA	FALSE	FALSE	FALSE	GCST006061	0.008	0.2865	537409	outcome	TRUE	reported	15516_12_SAA4_SAA_4	SAA4	chr11	18197693	0.006799	1e-200	35383	TRUE	reported	textfile	2	1	TRUE
5087	rs1648233	A	C	A	C	-0.2834	-0.015	0.05831	NA	FALSE	FALSE	FALSE	GCST006061	0.0151	0.3188	537409	outcome	TRUE	reported	15516_12_SAA4_SAA_4	SAA4	chr11	19210963	0.020568	3.435e-43	35383	TRUE	reported	textfile	2	1	TRUE
5088	rs2702652	T	G	T	G	0.0822	0.0126	0.25058	NA	FALSE	FALSE	FALSE	GCST006061	0.0079	0.1135	537409	outcome	TRUE	reported	15516_12_SAA4_SAA_4	SAA4	chr11	19444928	0.011125	1.481e-13	35384	TRUE	reported	textfile	2	1	TRUE
5089	rs58810621	T	G	T	G	0.0738	-0.0012	0.16169	NA	FALSE	FALSE	FALSE	GCST006061	0.01	0.9011	537409	outcome	TRUE	reported	15516_12_SAA4_SAA_4	SAA4	chr11	19906548	0.013056	1.582e-08	35383	TRUE	reported	textfile	2	1	TRUE
5090	rs73438377	T	C	T	C	-0.1752	-0.0042	0.03731	NA	FALSE	FALSE	FALSE	GCST006061	0.0175	0.8078	537409	outcome	TRUE	reported	15516_12_SAA4_SAA_4	SAA4	chr11	19162627	0.025552	7.056e-12	35382	TRUE	reported	textfile	2	1	TRUE
5091	rs10768174	A	G	A	G	-0.1802	-0.0123	0.40499	NA	FALSE	FALSE	FALSE	GCST006061	0.0072	0.08861	537409	outcome	TRUE	reported	7921_65_FJX1_FJX1	FJX1	chr11	36057706	0.008352	3.124e-103	35368	TRUE	reported	textfile	2	1	TRUE
5092	rs11065979	T	C	T	C	0.0699	-0.0099	0.34967	NA	FALSE	FALSE	FALSE	GCST006061	0.0076	0.1912	537409	outcome	TRUE	reported	7921_65_FJX1_FJX1	FJX1	chr12	111621753	0.008615	4.913e-16	35371	TRUE	reported	textfile	2	1	TRUE
5093	rs11110316	G	A	G	A	0.0542	1e-04	0.24109	NA	FALSE	FALSE	FALSE	GCST006061	0.0083	0.9913	537409	outcome	TRUE	reported	7921_65_FJX1_FJX1	FJX1	chr12	100249032	0.009626	1.799e-08	35365	TRUE	reported	textfile	2	1	TRUE
5094	rs12574547	G	A	G	A	0.1114	0.0043	0.32423	NA	FALSE	FALSE	FALSE	GCST006061	0.0076	0.569	537409	outcome	TRUE	reported	7921_65_FJX1_FJX1	FJX1	chr11	36151539	0.008808	1.145e-36	35372	TRUE	reported	textfile	2	1	TRUE
5095	rs12576371	G	A	G	A	0.1794	0.015	0.03224	NA	FALSE	FALSE	FALSE	GCST006061	0.0157	0.3365	537409	outcome	TRUE	reported	7921_65_FJX1_FJX1	FJX1	chr11	36162895	0.023362	1.6e-14	35370	TRUE	reported	textfile	2	1	TRUE
5096	rs140624338	C	T	C	T	0.2426	0.0021	0.01047	NA	FALSE	FALSE	FALSE	GCST006061	0.0539	0.9695	537409	outcome	TRUE	reported	7921_65_FJX1_FJX1	FJX1	chr12	101963654	0.040497	2.091e-09	35362	TRUE	reported	textfile	2	1	TRUE
5097	rs143875230	A	G	A	G	0.1509	0.0139	0.02944	NA	FALSE	FALSE	FALSE	GCST006061	0.027	0.6057	537409	outcome	TRUE	reported	7921_65_FJX1_FJX1	FJX1	chr15	42986528	0.024406	6.296e-10	35363	TRUE	reported	textfile	2	1	TRUE
5098	rs474415	A	G	A	G	0.107	0.0071	0.37824	NA	FALSE	FALSE	FALSE	GCST006061	0.0078	0.3652	537409	outcome	TRUE	reported	7921_65_FJX1_FJX1	FJX1	chr11	35621175	0.008485	1.865e-36	35371	TRUE	reported	textfile	2	1	TRUE
5099	rs629301	T	G	T	G	0.2696	-0.0141	0.20782	NA	FALSE	FALSE	FALSE	GCST006061	0.0087	0.1045	537409	outcome	TRUE	reported	7921_65_FJX1_FJX1	FJX1	chr1	109275684	0.010092	3.243e-157	35344	TRUE	reported

textfile 2 1 TRUE
5100 rs643785 T G T G 0.0626 0.0092 0.19715 NA FALSE FALSE FALSE
GCST006061 0.0086 0.2798 537409 outcome TRUE reported 7921_65_FJX1_FJX1
FJX1 chr1 109191603 0.010345 1.436e-09 35343 TRUE reported
textfile 2 1 TRUE
5101 rs10769106 T C T C -0.0671 -0.0047 0.2594 NA FALSE FALSE FALSE
GCST006061 0.0081 0.5671 537409 outcome TRUE reported 19614_8_TCN1_Holo_TC_I
TCN1 chr11 45025165 0.009586 2.569e-12 35373 TRUE reported
textfile 2 1 TRUE
5102 rs11229862 G A G A -0.133 0.0247 0.05201 NA FALSE FALSE FALSE
GCST006061 0.0194 0.2023 537409 outcome TRUE reported 19614_8_TCN1_Holo_TC_I
TCN1 chr11 59165059 0.018765 1.365e-12 35369 TRUE reported
textfile 2 1 TRUE
5103 rs1131603 C T C T -0.1857 0.0439 0.05267 NA FALSE FALSE FALSE
GCST006061 0.0181 0.01534 537409 outcome TRUE reported 19614_8_TCN1_Holo_TC_I
TCN1 chr22 30622988 0.018244 2.472e-24 35365 TRUE reported
textfile 2 1 TRUE
5104 rs113453659 T C T C -0.1359 -0.0209 0.08208 NA FALSE FALSE FALSE
GCST006061 0.0189 0.2704 537409 outcome TRUE reported 19614_8_TCN1_Holo_TC_I
TCN1 chr11 47920125 0.015262 5.378e-19 35374 TRUE reported
textfile 2 1 TRUE
5105 rs12799025 A G A G -0.1208 -0.0221 0.04615 NA FALSE FALSE FALSE
GCST006061 0.0191 0.2472 537409 outcome TRUE reported 19614_8_TCN1_Holo_TC_I
TCN1 chr11 57960400 0.020164 2.087e-09 35366 TRUE reported
textfile 2 1 TRUE
5106 rs139128886 C T C T -0.2993 0.1032 0.01223 NA FALSE FALSE FALSE
GCST006061 0.0455 0.02336 537409 outcome TRUE reported 19614_8_TCN1_Holo_TC_I
TCN1 chr11 48555288 0.038661 9.81e-15 35364 TRUE reported
textfile 2 1 TRUE
5107 rs147981159 A G A G -0.2152 0.0169 0.03953 NA FALSE FALSE FALSE
GCST006061 0.0223 0.4498 537409 outcome TRUE reported 19614_8_TCN1_Holo_TC_I
TCN1 chr11 62056826 0.021823 6.146e-23 35365 TRUE reported
textfile 2 1 TRUE
5108 rs16938342 T C T C -0.1446 0.0022 0.04551 NA FALSE FALSE FALSE
GCST006061 0.019 0.909 537409 outcome TRUE reported 19614_8_TCN1_Holo_TC_I
TCN1 chr11 45684744 0.020623 2.356e-12 35376 TRUE reported
textfile 2 1 TRUE
5109 rs1801222 G A G A -0.0499 2e-04 0.41836 NA FALSE FALSE FALSE
GCST006061 0.0075 0.9745 537409 outcome TRUE reported 19614_8_TCN1_Holo_TC_I
TCN1 chr10 17114152 0.008332 2.109e-09 35363 TRUE reported
textfile 2 1 TRUE
5110 rs1870028 T C T C 0.0848 0.0076 0.10976 NA FALSE FALSE FALSE
GCST006061 0.0134 0.5688 537409 outcome TRUE reported 19614_8_TCN1_Holo_TC_I
TCN1 chr7 50218717 0.013206 1.352e-10 35337 TRUE reported
textfile 2 1 TRUE
5111 rs34324219 A C A C -0.4887 0.0192 0.10898 NA FALSE FALSE FALSE
GCST006061 0.0124 0.1214 537409 outcome TRUE reported 19614_8_TCN1_Holo_TC_I
TCN1 chr11 59855905 0.013236 1e-200 35365 TRUE reported textfile
2 1 TRUE
5112 rs3826331 C T C T -0.0985 0.0108 0.37917 NA FALSE FALSE FALSE
GCST006061 0.0072 0.1345 537409 outcome TRUE reported 19614_8_TCN1_Holo_TC_I
TCN1 chr17 39994239 0.008485 3.743e-31 35379 TRUE reported
textfile 2 1 TRUE
5113 rs61885288 C T C T -0.1991 0.0155 0.02326 NA FALSE FALSE FALSE
GCST006061 0.0285 0.5861 537409 outcome TRUE reported 19614_8_TCN1_Holo_TC_I
TCN1 chr11 49274786 0.027827 8.366e-13 35363 TRUE reported
textfile 2 1 TRUE
5114 rs681343 T C T C 0.0928 0.0054 0.3954 NA FALSE FALSE FALSE
GCST006061 0.0076 0.4742 537409 outcome TRUE reported 19614_8_TCN1_Holo_TC_I
TCN1 chr19 48703205 0.00844 4.004e-28 35377 TRUE reported textfile
2 1 TRUE
5115 rs72916973 T C T C -0.1091 0.0182 0.09389 NA FALSE FALSE FALSE
GCST006061 0.0126 0.1474 537409 outcome TRUE reported 19614_8_TCN1_Holo_TC_I
TCN1 chr11 60895880 0.014621 8.545e-14 35370 TRUE reported
textfile 2 1 TRUE
5116 rs80089870 A G A G -0.2714 0.0396 0.01559 NA FALSE FALSE FALSE

GCST006061 0.0339 0.2426 537409 outcome TRUE reported 19614_8_TCN1_Holo_TC_I
TCN1 chr11 61591453 0.033913 1.217e-15 35362 TRUE reported
textfile 2 1 TRUE
5117 rs10769124 A G A G -0.0527 -0.007 0.3097 NA FALSE FALSE FALSE
GCST006061 0.0082 0.3931 537409 outcome TRUE reported 4923_79_AMH_MIS AMH
chr11 5610982 0.008931 3.624e-09 35378 TRUE reported textfile 2 1
TRUE
5118 rs112189508 C T C T 0.2533 -0.0306 0.05907 NA FALSE FALSE FALSE
GCST006061 0.0175 0.08086 537409 outcome TRUE reported 4923_79_AMH_MIS AMH
chr11 6262548 0.017594 5.422e-47 35374 TRUE reported textfile 2 1
TRUE
5119 rs117627469 A G A G 0.3811 -0.0504 0.01774 NA FALSE FALSE FALSE
GCST006061 0.0491 0.3048 537409 outcome TRUE reported 4923_79_AMH_MIS AMH
chr11 8178769 0.031 9.789e-35 35374 TRUE reported textfile 2 1 TRUE
5120 rs1619994 G A G A 0.0594 0.0168 0.2807 NA FALSE FALSE FALSE
GCST006061 0.0078 0.03057 537409 outcome TRUE reported 4923_79_AMH_MIS AMH
chr3 165744047 0.00913 7.725e-11 35339 TRUE reported textfile 2 1
TRUE
5121 rs16914574 C T C T 0.4427 -0.0098 0.04057 NA FALSE FALSE FALSE
GCST006061 0.0233 0.6742 537409 outcome TRUE reported 4923_79_AMH_MIS AMH
chr11 6511260 0.02104 2.75e-98 35380 TRUE reported textfile 2 1 TRUE
5122 rs56385952 G A G A -0.0635 -0.006 0.44609 NA FALSE FALSE FALSE
GCST006061 0.0071 0.3968 537409 outcome TRUE reported 4923_79_AMH_MIS AMH
chr2 159707684 0.00824 1.291e-14 35350 TRUE reported textfile 2 1
TRUE
5123 rs10769263 C A C A -0.0613 0.0086 0.36683 NA FALSE FALSE FALSE
GCST006061 0.0075 0.2513 537409 outcome TRUE reported 3206_4_LYVE1_LYVE1
LYVE1 chr11 47395632 0.008779 2.905e-12 35376 TRUE reported
textfile 2 1 TRUE
5124 rs11216906 A G A G -0.0646 0.0064 0.15822 NA FALSE FALSE FALSE
GCST006061 0.0101 0.5248 537409 outcome TRUE reported 3206_4_LYVE1_LYVE1
LYVE1 chr11 118573229 0.011657 2.991e-08 35380 TRUE reported
textfile 2 1 TRUE
5125 rs117633773 A G A G 0.1522 0.0067 0.04197 NA FALSE FALSE FALSE
GCST006061 0.0224 0.7662 537409 outcome TRUE reported 3206_4_LYVE1_LYVE1
LYVE1 chr17 6953365 0.020677 1.826e-13 35374 TRUE reported textfile
2 1 TRUE
5126 rs12289747 G A G A -0.1629 -0.0101 0.04072 NA FALSE FALSE FALSE
GCST006061 0.026 0.6968 537409 outcome TRUE reported 3206_4_LYVE1_LYVE1
LYVE1 chr11 10585458 0.021281 1.94e-14 35377 TRUE reported
textfile 2 1 TRUE
5127 rs12978875 A G A G 0.0504 0.0024 0.34064 NA FALSE FALSE FALSE
GCST006061 0.0084 0.7719 537409 outcome TRUE reported 3206_4_LYVE1_LYVE1
LYVE1 chr19 3386575 0.008791 9.841e-09 35340 TRUE reported textfile
2 1 TRUE
5128 rs143501783 C T C T 0.3588 -0.0333 0.01116 NA FALSE FALSE FALSE
GCST006061 0.0443 0.4525 537409 outcome TRUE reported 3206_4_LYVE1_LYVE1
LYVE1 chr17 7897001 0.038122 4.869e-21 35373 TRUE reported textfile
2 1 TRUE
5129 rs1453869 C T C T -0.0491 6e-04 0.42574 NA FALSE FALSE FALSE
GCST006061 0.0073 0.9359 537409 outcome TRUE reported 3206_4_LYVE1_LYVE1
LYVE1 chr4 98970487 0.008446 6.11e-09 35342 TRUE reported
textfile 2 1 TRUE
5130 rs1955512 A G A G 0.0894 0.0104 0.4254 NA FALSE FALSE FALSE
GCST006061 0.0074 0.1586 537409 outcome TRUE reported 3206_4_LYVE1_LYVE1
LYVE1 chr14 32706616 0.008414 2.284e-26 35356 TRUE reported
textfile 2 1 TRUE
5131 rs2394668 A G A G -0.0627 0.015 0.36113 NA FALSE FALSE FALSE
GCST006061 0.008 0.05862 537409 outcome TRUE reported 3206_4_LYVE1_LYVE1
LYVE1 chr10 70184195 0.008717 6.339e-13 35372 TRUE reported
textfile 2 1 TRUE
5132 rs333947 A G A G 0.0654 -0.0071 0.14937 NA FALSE FALSE FALSE
GCST006061 0.01 0.4808 537409 outcome TRUE reported 3206_4_LYVE1_LYVE1
LYVE1 chr1 109928142 0.011827 3.211e-08 35349 TRUE reported
textfile 2 1 TRUE
5133 rs35257264 T C T C 0.1743 0.0195 0.03294 NA FALSE FALSE FALSE

GCST006061 0.0269 0.4693 537409 outcome TRUE reported 3206_4_LYVE1_LYVE1
LYVE1 chr11 126426921 0.023047 3.946e-14 35371 TRUE reported
textfile 2 1 TRUE
5134 rs4910063 A G A G -0.071 0.0051 0.2254 NA FALSE FALSE FALSE
GCST006061 0.0081 0.5303 537409 outcome TRUE reported 3206_4_LYVE1_LYVE1
LYVE1 chr11 9783777 0.010132 2.423e-12 35373 TRUE reported textfile
2 1 TRUE
5135 rs56157435 T C T C 0.057 0.0116 0.34927 NA FALSE FALSE FALSE
GCST006061 0.0074 0.1169 537409 outcome TRUE reported 3206_4_LYVE1_LYVE1
LYVE1 chr9 34111105 0.008768 7.977e-11 35349 TRUE reported
textfile 2 1 TRUE
5136 rs700801 A G A G -0.08 0.0074 0.21125 NA FALSE FALSE FALSE
GCST006061 0.0085 0.383 537409 outcome TRUE reported 3206_4_LYVE1_LYVE1
LYVE1 chr9 76619731 0.010321 9.113e-15 35351 TRUE reported
textfile 2 1 TRUE
5137 rs72861085 G A G A -0.2527 -0.0197 0.01971 NA FALSE FALSE FALSE
GCST006061 0.0342 0.5639 537409 outcome TRUE reported 3206_4_LYVE1_LYVE1
LYVE1 chr11 10581034 0.030469 1.098e-16 35377 TRUE reported
textfile 2 1 TRUE
5138 rs75071241 A G A G -0.1882 0.0049 0.05897 NA FALSE FALSE FALSE
GCST006061 0.0145 0.7368 537409 outcome TRUE reported 3206_4_LYVE1_LYVE1
LYVE1 chr11 126362291 0.017948 1.002e-25 35375 TRUE reported
textfile 2 1 TRUE
5139 rs7601767 A G A G -0.0556 0.01 0.31396 NA FALSE FALSE FALSE
GCST006061 0.0076 0.1911 537409 outcome TRUE reported 3206_4_LYVE1_LYVE1
LYVE1 chr2 48050351 0.009079 9.109e-10 35362 TRUE reported
textfile 2 1 TRUE
5140 rs76318574 C T C T 0.3353 0.0218 0.02434 NA FALSE FALSE FALSE
GCST006061 0.0248 0.3797 537409 outcome TRUE reported 3206_4_LYVE1_LYVE1
LYVE1 chr11 10506171 0.028024 5.43e-33 35373 TRUE reported
textfile 2 1 TRUE
5141 rs7856589 A G A G 0.0494 0.0063 0.37907 NA FALSE FALSE FALSE
GCST006061 0.0075 0.4013 537409 outcome TRUE reported 3206_4_LYVE1_LYVE1
LYVE1 chr9 137109469 0.008634 1.055e-08 35259 TRUE reported
textfile 2 1 TRUE
5142 rs7865362 T C T C 0.1446 0.0022 0.34624 NA FALSE FALSE FALSE
GCST006061 0.0075 0.7726 537409 outcome TRUE reported 3206_4_LYVE1_LYVE1
LYVE1 chr9 33117967 0.008741 1.801e-61 35349 TRUE reported
textfile 2 1 TRUE
5143 rs9826842 C T C T -0.0549 0.0082 0.35038 NA FALSE FALSE FALSE
GCST006061 0.0076 0.2775 537409 outcome TRUE reported 3206_4_LYVE1_LYVE1
LYVE1 chr3 186162411 0.008752 3.541e-10 35361 TRUE reported
textfile 2 1 TRUE
5144 rs9832668 A G A G 0.0557 -0.0283 0.23903 NA FALSE FALSE FALSE
GCST006061 0.008 0.0004262 537409 outcome TRUE reported 3206_4_LYVE1_LYVE1
LYVE1 chr3 72349942 0.009921 1.971e-08 35345 TRUE reported
textfile 2 1 TRUE
5145 rs10769263 C A C A -0.0557 0.0086 0.36683 NA FALSE FALSE FALSE
GCST006061 0.0075 0.2513 537409 outcome TRUE reported 16914_104_CD14_sCD14
CD14 chr11 47395632 0.008524 6.387e-11 35376 TRUE reported
textfile 2 1 TRUE
5146 rs114928388 A G A G 0.2585 0.0446 0.03808 NA FALSE FALSE FALSE
GCST006061 0.0296 0.1317 537409 outcome TRUE reported 16914_104_CD14_sCD14
CD14 chr5 140383553 0.021339 8.888e-34 35363 TRUE reported
textfile 2 1 TRUE
5147 rs1250258 T C T C -0.0684 0.0164 0.32665 NA FALSE FALSE FALSE
GCST006061 0.0083 0.04845 537409 outcome TRUE reported 16914_104_CD14_sCD14
CD14 chr2 215435462 0.008719 4.337e-15 35355 TRUE reported
textfile 2 1 TRUE
5148 rs13209376 T G T G 0.0741 -0.0321 0.1057 NA FALSE FALSE FALSE
GCST006061 0.0131 0.01421 537409 outcome TRUE reported 16914_104_CD14_sCD14
CD14 chr6 95622197 0.013308 2.574e-08 35343 TRUE reported
textfile 2 1 TRUE
5149 rs192900523 A G A G 0.1272 0.019 0.05256 NA FALSE FALSE FALSE
GCST006061 0.0283 0.5023 537409 outcome TRUE reported 16914_104_CD14_sCD14
CD14 chr5 140024507 0.01835 4.15e-12 35360 TRUE reported textfile

2 1 TRUE
5150 rs2948711 G A G A 0.047 5e-04 0.46902 NA FALSE FALSE FALSE GCST006061 0.0076 0.9427 537409 outcome TRUE reported 16914_104_CD14_sCD14 CD14 chr10 17734454 0.008216 1.06e-08 35355 TRUE reported textfile 2 1 TRUE
5151 rs4760 G A G A -0.0716 -0.0025 0.12052 NA FALSE FALSE FALSE GCST006061 0.0109 0.8165 537409 outcome TRUE reported 16914_104_CD14_sCD14 CD14 chr19 43648948 0.012599 1.324e-08 35369 TRUE reported textfile 2 1 TRUE
5152 rs56365312 A G A G -0.1191 -0.0113 0.04375 NA FALSE FALSE FALSE GCST006061 0.0208 0.587 537409 outcome TRUE reported 16914_104_CD14_sCD14 CD14 chr9 79885828 0.020128 3.278e-09 35354 TRUE reported textfile 2 1 TRUE
5153 rs5744454 G T G T -0.2387 -0.0282 0.30745 NA FALSE FALSE FALSE GCST006061 0.0082 0.00057 537409 outcome TRUE reported 16914_104_CD14_sCD14 CD14 chr5 140633982 0.008824 3.582e-161 35363 TRUE reported textfile 2 1 TRUE
5154 rs635634 C T C T 0.0867 0.0285 0.12995 NA FALSE FALSE FALSE GCST006061 0.0089 0.001351 537409 outcome TRUE reported 16914_104_CD14_sCD14 CD14 chr9 133279427 0.012209 1.237e-12 35371 TRUE reported textfile 2 1 TRUE
5155 rs78656209 A G A G 0.115 0.001 0.04859 NA FALSE FALSE FALSE GCST006061 0.019 0.9564 537409 outcome TRUE reported 16914_104_CD14_sCD14 CD14 chr5 139853212 0.019141 1.877e-09 35357 TRUE reported textfile 2 1 TRUE
5156 rs9425316 T C T C 0.0809 0.0042 0.46888 NA FALSE FALSE FALSE GCST006061 0.0072 0.5574 537409 outcome TRUE reported 16914_104_CD14_sCD14 CD14 chr1 172431720 0.008212 6.732e-23 35350 TRUE reported textfile 2 1 TRUE
5157 rs9878990 C T C T -0.0666 -0.0081 0.30458 NA FALSE FALSE FALSE GCST006061 0.0074 0.2732 537409 outcome TRUE reported 16914_104_CD14_sCD14 CD14 chr3 165821334 0.008903 7.394e-14 35339 TRUE reported textfile 2 1 TRUE
5158 rs10770 C T C T -0.0853 -0.0088 0.13964 NA FALSE FALSE FALSE GCST006061 0.0109 0.4198 537409 outcome TRUE reported 7228_2_ST6GALNAC6_SIA7F ST6GALNAC6 chr3 186671770 0.011698 3.056e-13 35368 TRUE reported textfile 2 1 TRUE
5159 rs72616671 T C T C -0.1849 0.0354 0.01743 NA FALSE FALSE FALSE GCST006061 0.0287 0.2174 537409 outcome TRUE reported 7228_2_ST6GALNAC6_SIA7F ST6GALNAC6 chr9 127873856 0.032179 9.142e-09 35357 TRUE reported textfile 2 1 TRUE
5160 rs72769820 C A C A 0.1856 0.0608 0.04356 NA FALSE FALSE FALSE GCST006061 0.0227 0.007438 537409 outcome TRUE reported 7228_2_ST6GALNAC6_SIA7F ST6GALNAC6 chr9 127622891 0.020068 2.279e-20 35365 TRUE reported textfile 2 1 TRUE
5161 rs77089732 A G A G 0.1998 -0.0357 0.01855 NA FALSE FALSE FALSE GCST006061 0.0397 0.3678 537409 outcome TRUE reported 7228_2_ST6GALNAC6_SIA7F ST6GALNAC6 chr9 123140768 0.030763 8.309e-11 35362 TRUE reported textfile 2 1 TRUE
5162 rs10772424 A G A G -0.1646 0.0083 0.03697 NA FALSE FALSE FALSE GCST006061 0.017 0.6241 537409 outcome TRUE reported 2942_50_CYCS_Cytochrome_c CYCS chr12 7490731 0.020873 3.129e-15 35388 TRUE reported textfile 2 1 TRUE
5163 rs12975366 C T C T -0.0644 -0.0089 0.4155 NA FALSE FALSE FALSE GCST006061 0.0081 0.2688 537409 outcome TRUE reported 2942_50_CYCS_Cytochrome_c CYCS chr19 54255498 0.008008 8.84e-16 35370 TRUE reported textfile 2 1 TRUE
5164 rs10773540 A G A G -0.0909 -0.0158 0.10683 NA FALSE FALSE FALSE GCST006061 0.0108 0.1428 537409 outcome TRUE reported 7173_141_TMEM132C_T132C TMEM132C chr12 128421140 0.013737 3.667e-11 35380 TRUE reported textfile 2 1 TRUE
5165 rs1328899 T C T C 0.2547 -0.0028 0.01081 NA FALSE FALSE FALSE GCST006061 0.026 0.9129 537409 outcome TRUE reported 7173_141_TMEM132C_T132C TMEM132C chr9 33151417 0.041483 8.263e-10 35349 TRUE reported textfile 2 1 TRUE
5166 rs1356014 T C T C -0.0843 -0.0103 0.38333 NA FALSE FALSE FALSE

GCST006061 0.0075 0.1712 537409 outcome TRUE reported 7173_141_TMEM132C_T132C TMEM132C chr12 128159988 0.00871 3.736e-22 35380 TRUE reported textfile 2 1 TRUE
5167 rs143501783 C T C T 0.2719 -0.0333 0.01116 NA FALSE FALSE FALSE GCST006061 0.0443 0.4525 537409 outcome TRUE reported 7173_141_TMEM132C_T132C TMEM132C chr17 7897001 0.038336 1.317e-12 35373 TRUE reported textfile 2 1 TRUE
5168 rs1683696 T G T G 0.1053 0.0025 0.48002 NA FALSE FALSE FALSE GCST006061 0.0073 0.7286 537409 outcome TRUE reported 7173_141_TMEM132C_T132C TMEM132C chr12 128389739 0.008527 4.974e-35 35377 TRUE reported textfile 2 1 TRUE
5169 rs2008174 T C T C -0.125 0.0184 0.23503 NA FALSE FALSE FALSE GCST006061 0.0087 0.03407 537409 outcome TRUE reported 7173_141_TMEM132C_T132C TMEM132C chr22 39464125 0.009918 2.015e-36 35364 TRUE reported textfile 2 1 TRUE
5170 rs3827380 G A G A 0.0575 -0.013 0.20147 NA FALSE FALSE FALSE GCST006061 0.0088 0.1398 537409 outcome TRUE reported 7173_141_TMEM132C_T132C TMEM132C chr22 39491681 0.010514 4.532e-08 35368 TRUE reported textfile 2 1 TRUE
5171 rs41556717 T C T C 0.2045 -0.0483 0.01931 NA FALSE FALSE FALSE GCST006061 0.0325 0.1371 537409 outcome TRUE reported 7173_141_TMEM132C_T132C TMEM132C chr17 7496000 0.029523 4.307e-12 35374 TRUE reported textfile 2 1 TRUE
5172 rs56115403 A G A G 0.0885 0.0012 0.21597 NA FALSE FALSE FALSE GCST006061 0.01 0.901 537409 outcome TRUE reported 7173_141_TMEM132C_T132C TMEM132C chr17 7160579 0.010309 9.079e-18 35371 TRUE reported textfile 2 1 TRUE
5173 rs73021419 T C T C -0.1504 0.0022 0.10279 NA FALSE FALSE FALSE GCST006061 0.01 0.8256 537409 outcome TRUE reported 7173_141_TMEM132C_T132C TMEM132C chr11 126380752 0.013856 1.902e-27 35376 TRUE reported textfile 2 1 TRUE
5174 rs77542162 G A G A 0.1864 0.0177 0.025 NA FALSE FALSE FALSE GCST006061 0.0302 0.557 537409 outcome TRUE reported 7173_141_TMEM132C_T132C TMEM132C chr17 69085137 0.026591 2.384e-12 35371 TRUE reported textfile 2 1 TRUE
5175 rs7973997 T C T C -0.2551 0.0013 0.32791 NA FALSE FALSE FALSE GCST006061 0.0078 0.8636 537409 outcome TRUE reported 7173_141_TMEM132C_T132C TMEM132C chr12 128272393 0.009025 9.376e-176 35381 TRUE reported textfile 2 1 TRUE
5176 rs10774378 C T C T -0.0853 0.001 0.30375 NA FALSE FALSE FALSE GCST006061 0.008 0.9042 537409 outcome TRUE reported 9823_2_DHFR_DYR DHFR chr12 5840605 0.008934 1.323e-21 35383 TRUE reported textfile 2 1 TRUE
5177 rs11539945 T C T C -0.081 -0.0231 0.11318 NA FALSE FALSE FALSE GCST006061 0.0126 0.06777 537409 outcome TRUE reported 9823_2_DHFR_DYR DHFR chr12 6200432 0.013107 6.409e-10 35383 TRUE reported textfile 2 1 TRUE
5178 rs1355538 G A G A -0.0602 -0.0076 0.39847 NA FALSE FALSE FALSE GCST006061 0.0072 0.2923 537409 outcome TRUE reported 9823_2_DHFR_DYR DHFR chr3 165787389 0.008283 3.647e-13 35338 TRUE reported textfile 2 1 TRUE
5179 rs483082 T G T G -0.0529 2e-04 0.23213 NA FALSE FALSE FALSE GCST006061 0.0088 0.9858 537409 outcome TRUE reported 9823_2_DHFR_DYR DHFR chr19 44912921 0.009699 4.921e-08 35366 TRUE reported textfile 2 1 TRUE
5180 rs542993 T C T C -0.3193 0.003 0.47995 NA FALSE FALSE FALSE GCST006061 0.0074 0.6895 537409 outcome TRUE reported 9823_2_DHFR_DYR DHFR chr12 6011265 0.006726 1e-200 35376 TRUE reported textfile 2 1 TRUE
5181 rs113896482 T C T C 0.1251 0.008 0.03508 NA FALSE FALSE FALSE GCST006061 0.0271 0.7692 537409 outcome TRUE reported 5063_12_CD244_CD244 CD244 chr1 160862395 0.021461 5.574e-09 35365 TRUE reported textfile 2 1 TRUE
5182 rs114594620 C T C T -0.2645 0.0099 0.02348 NA FALSE FALSE FALSE GCST006061 0.0468 0.833 537409 outcome TRUE reported 5063_12_CD244_CD244 CD244 chr1 160731600 0.02646 1.584e-23 35360 TRUE reported textfile 2 1 TRUE

5183 rs266723 C A C A 0.0743 0.0025 0.44674 NA FALSE FALSE FALSE
GCST006061 0.0071 0.7224 537409 outcome TRUE reported 9942_2_NXT1_NXT1
NXT1 chr3 186729258 0.008046 2.602e-20 35368 TRUE reported
textfile 2 1 TRUE
5184 rs7626301 C T C T 0.2339 1e-04 0.4424 NA FALSE FALSE FALSE
GCST006061 0.0072 0.9914 537409 outcome TRUE reported 9942_2_NXT1_NXT1
NXT1 chr3 186676096 0.007966 1.586e-189 35368 TRUE reported
textfile 2 1 TRUE
5185 rs7632706 T C T C 0.0859 -0.0277 0.12703 NA FALSE FALSE FALSE
GCST006061 0.0097 0.004357 537409 outcome TRUE reported 9942_2_NXT1_NXT1
NXT1 chr3 186416692 0.012029 9.248e-13 35365 TRUE reported
textfile 2 1 TRUE
5186 rs79480970 A G A G 0.0673 0.0064 0.13103 NA FALSE FALSE FALSE
GCST006061 0.0117 0.5851 537409 outcome TRUE reported 9942_2_NXT1_NXT1
NXT1 chr3 187187426 0.012025 2.184e-08 35370 TRUE reported
textfile 2 1 TRUE
5187 rs10775412 C A C A -0.0873 -0.0055 0.34151 NA FALSE FALSE FALSE
GCST006061 0.0076 0.4655 537409 outcome TRUE reported 13501_10_SLC35G2_S35G2
SLC35G2 chr17 27542007 0.008617 4.032e-24 35353 TRUE reported
textfile 2 1 TRUE
5188 rs11869913 C T C T -0.0664 0.0074 0.1834 NA FALSE FALSE FALSE
GCST006061 0.0099 0.4506 537409 outcome TRUE reported 13501_10_SLC35G2_S35G2
SLC35G2 chr17 33035903 0.010689 5.225e-10 35366 TRUE reported
textfile 2 1 TRUE
5189 rs12478841 G A G A 0.062 0.0049 0.21569 NA FALSE FALSE FALSE
GCST006061 0.008 0.5413 537409 outcome TRUE reported 13501_10_SLC35G2_S35G2
SLC35G2 chr2 27588855 0.009646 1.295e-10 35367 TRUE reported
textfile 2 1 TRUE
5190 rs12941530 T C T C 0.1421 -0.0275 0.04125 NA FALSE FALSE FALSE
GCST006061 0.0232 0.2364 537409 outcome TRUE reported 13501_10_SLC35G2_S35G2
SLC35G2 chr17 28494781 0.020518 4.341e-12 35355 TRUE reported
textfile 2 1 TRUE
5191 rs1697422 C T C T -0.0534 0.0053 0.48923 NA FALSE FALSE FALSE
GCST006061 0.0072 0.4547 537409 outcome TRUE reported 13501_10_SLC35G2_S35G2
SLC35G2 chr1 21495404 0.008032 2.96e-11 35368 TRUE reported
textfile 2 1 TRUE
5192 rs3760457 C T C T 0.1849 0.01 0.04832 NA FALSE FALSE FALSE
GCST006061 0.0142 0.4816 537409 outcome TRUE reported 13501_10_SLC35G2_S35G2
SLC35G2 chr17 29264963 0.019183 5.491e-22 35355 TRUE reported
textfile 2 1 TRUE
5193 rs56147019 T C T C -0.2637 -0.0158 0.01164 NA FALSE FALSE FALSE
GCST006061 0.0373 0.6714 537409 outcome TRUE reported 13501_10_SLC35G2_S35G2
SLC35G2 chr17 28664803 0.03747 1.955e-12 35353 TRUE reported textfile
2 1 TRUE
5194 rs7222245 G A G A -0.1638 -9.00E-04 0.16341 NA FALSE FALSE FALSE
GCST006061 0.01 0.9306 537409 outcome TRUE reported 13501_10_SLC35G2_S35G2
SLC35G2 chr17 27942387 0.011123 4.385e-49 35353 TRUE reported
textfile 2 1 TRUE
5195 rs8068072 G A G A -0.2498 0.0127 0.42347 NA FALSE FALSE FALSE
GCST006061 0.0073 0.07984 537409 outcome TRUE reported 13501_10_SLC35G2_S35G2
SLC35G2 chr17 28206276 0.008236 1e-200 35354 TRUE reported textfile
2 1 TRUE
5196 rs10775412 C A C A -0.0909 -0.0055 0.34151 NA FALSE FALSE FALSE
GCST006061 0.0076 0.4655 537409 outcome TRUE reported 12529_32_FKBP6_FKBP6
FKBP6 chr17 27542007 0.00859 3.592e-26 35350 TRUE reported textfile
2 1 TRUE
5197 rs1242299 A G A G 0.2656 -0.0121 0.42604 NA FALSE FALSE FALSE
GCST006061 0.0072 0.09315 537409 outcome TRUE reported 12529_32_FKBP6_FKBP6
FKBP6 chr17 28189182 0.008189 1e-200 35351 TRUE reported textfile
2 1 TRUE
5198 rs16964949 A G A G 0.1713 0.0115 0.04832 NA FALSE FALSE FALSE
GCST006061 0.0142 0.4169 537409 outcome TRUE reported 12529_32_FKBP6_FKBP6
FKBP6 chr17 29255560 0.01913 3.406e-19 35352 TRUE reported textfile
2 1 TRUE
5199 rs3741298 T C T C -0.0572 -0.0014 0.19216 NA FALSE FALSE FALSE
GCST006061 0.0086 0.8685 537409 outcome TRUE reported 12529_32_FKBP6_FKBP6

FKBP6 chr11 116786845 0.010027 1.166e-08 35367 TRUE reported
textfile 2 1 TRUE
5200 rs62294359 A G A G 0.1092 0.0164 0.24788 NA FALSE FALSE FALSE
GCST006061 0.0081 0.04287 537409 outcome TRUE reported 12529_32_FKBP6_FKBP6
FKBP6 chr3 165744429 0.009195 1.578e-32 35330 TRUE reported
textfile 2 1 TRUE
5201 rs662 C T C T 0.0807 -0.0141 0.28559 NA FALSE FALSE FALSE GCST006061
0.0078 0.06938 537409 outcome TRUE reported 12529_32_FKBP6_FKBP6 FKBP6
chr7 95308134 0.008818 5.591e-20 35340 TRUE reported textfile 2
1 TRUE
5202 rs7222245 G A G A -0.1596 -9.00E-04 0.16341 NA FALSE FALSE FALSE
GCST006061 0.01 0.9306 537409 outcome TRUE reported 12529_32_FKBP6_FKBP6
FKBP6 chr17 27942387 0.011088 5.625e-47 35350 TRUE reported
textfile 2 1 TRUE
5203 rs73045691 A G A G 0.3273 0.0102 0.26022 NA FALSE FALSE FALSE
GCST006061 0.0083 0.2192 537409 outcome TRUE reported 12529_32_FKBP6_FKBP6
FKBP6 chr19 44937272 0.009046 1e-200 35359 TRUE reported textfile
2 1 TRUE
5204 rs77660932 C T C T -0.0764 -0.0044 0.18053 NA FALSE FALSE FALSE
GCST006061 0.0102 0.6647 537409 outcome TRUE reported 12529_32_FKBP6_FKBP6
FKBP6 chr19 45091584 0.010522 3.841e-13 35362 TRUE reported
textfile 2 1 TRUE
5205 rs919791 A G A G -0.0532 -0.0081 0.2132 NA FALSE FALSE FALSE
GCST006061 0.0088 0.3607 537409 outcome TRUE reported 12529_32_FKBP6_FKBP6
FKBP6 chr19 15466006 0.009719 4.404e-08 35369 TRUE reported
textfile 2 1 TRUE
5206 rs10776701 A G A G 0.0707 -0.0056 0.40845 NA FALSE FALSE FALSE
GCST006061 0.0075 0.4571 537409 outcome TRUE reported 9748_31_GSTM3_GSTM3_3
GSTM3 chr1 109846704 0.008955 2.894e-15 35343 TRUE reported
textfile 2 1 TRUE
5207 rs11102126 A G A G -0.0576 -0.0023 0.30212 NA FALSE FALSE FALSE
GCST006061 0.0078 0.7645 537409 outcome TRUE reported 9748_31_GSTM3_GSTM3_3
GSTM3 chr1 110541634 0.009607 2.027e-09 35349 TRUE reported
textfile 2 1 TRUE
5208 rs112591404 C T C T -0.0742 -0.0674 0.17436 NA FALSE FALSE FALSE
GCST006061 0.0307 0.02825 537409 outcome TRUE reported 9748_31_GSTM3_GSTM3_3
GSTM3 chr1 120440667 0.012152 1.023e-09 35358 TRUE reported
textfile 2 1 TRUE
5209 rs112852158 G A G A -0.5014 -0.0026 0.09729 NA FALSE FALSE FALSE
GCST006061 0.0203 0.8997 537409 outcome TRUE reported 9748_31_GSTM3_GSTM3_3
GSTM3 chr1 109703211 0.014476 1e-200 35343 TRUE reported textfile
2 1 TRUE
5210 rs115450622 C T C T 0.803 -0.0206 0.03028 NA FALSE FALSE FALSE
GCST006061 0.0242 0.3943 537409 outcome TRUE reported 9748_31_GSTM3_GSTM3_3
GSTM3 chr1 109653279 0.025718 1e-200 35346 TRUE reported textfile
2 1 TRUE
5211 rs116276872 T C T C -0.3773 0.0112 0.02038 NA FALSE FALSE FALSE
GCST006061 0.0298 0.7071 537409 outcome TRUE reported 9748_31_GSTM3_GSTM3_3
GSTM3 chr1 109525669 0.031452 3.733e-33 35345 TRUE reported
textfile 2 1 TRUE
5212 rs116532364 T C T C 0.861 -0.0664 0.02052 NA FALSE FALSE FALSE
GCST006061 0.027 0.014 537409 outcome TRUE reported 9748_31_GSTM3_GSTM3_3
GSTM3 chr1 109636098 0.030979 5.254e-170 35346 TRUE reported
textfile 2 1 TRUE
5213 rs143379394 A G A G -0.2809 -0.0194 0.01921 NA FALSE FALSE FALSE
GCST006061 0.0467 0.6787 537409 outcome TRUE reported 9748_31_GSTM3_GSTM3_3
GSTM3 chr1 109284844 0.032209 2.754e-18 35344 TRUE reported
textfile 2 1 TRUE
5214 rs148631275 C T C T 0.1883 -0.0134 0.02084 NA FALSE FALSE FALSE
GCST006061 0.0318 0.674 537409 outcome TRUE reported 9748_31_GSTM3_GSTM3_3
GSTM3 chr1 109385002 0.030478 6.482e-10 35345 TRUE reported
textfile 2 1 TRUE
5215 rs1707654 T C T C 0.0797 0.0142 0.25158 NA FALSE FALSE FALSE
GCST006061 0.0079 0.07116 537409 outcome TRUE reported 9748_31_GSTM3_GSTM3_3
GSTM3 chr3 165758405 0.009364 1.714e-17 35339 TRUE reported
textfile 2 1 TRUE

5216 rs187542824 T G T G 0.6345 0.0575 0.01477 NA FALSE FALSE FALSE GCST006061 0.047 0.2216 537409 outcome TRUE reported 9748_31_GSTM3_GSTM3_3 GSTM3 chr1 109086421 0.036692 5.37e-67 35342 TRUE reported textfile 2 1 TRUE

5217 rs72705270 T C T C -0.2006 -0.0096 0.15339 NA FALSE FALSE FALSE GCST006061 0.0099 0.3315 537409 outcome TRUE reported 9748_31_GSTM3_GSTM3_3 GSTM3 chr1 109788605 0.012133 2.11e-61 35340 TRUE reported textfile 2 1 TRUE

5218 rs10776914 C T C T -0.2978 -8.00E-04 0.29991 NA FALSE FALSE FALSE GCST006061 0.0077 0.9208 537409 outcome TRUE reported 15582_25_FCN1_FCN1 FCN1 chr9 134948963 0.009015 1e-200 35330 TRUE reported textfile 2 1 TRUE

5219 rs11103734 T C T C -0.096 -0.008 0.09268 NA FALSE FALSE FALSE GCST006061 0.0149 0.5935 537409 outcome TRUE reported 15582_25_FCN1_FCN1 FCN1 chr9 135190355 0.014472 3.276e-11 35323 TRUE reported textfile 2 1 TRUE

5220 rs142369618 C T C T -0.223 0.0094 0.02432 NA FALSE FALSE FALSE GCST006061 0.0338 0.7816 537409 outcome TRUE reported 15582_25_FCN1_FCN1 FCN1 chr9 135427776 0.026883 1.083e-16 35308 TRUE reported textfile 2 1 TRUE

5221 rs149892510 T C T C -0.2325 0.0197 0.01404 NA FALSE FALSE FALSE GCST006061 0.0496 0.6912 537409 outcome TRUE reported 15582_25_FCN1_FCN1 FCN1 chr9 135184742 0.036305 1.513e-10 35323 TRUE reported textfile 2 1 TRUE

5222 rs2344873 A G A G 0.0535 0.0081 0.44804 NA FALSE FALSE FALSE GCST006061 0.0075 0.2771 537409 outcome TRUE reported 15582_25_FCN1_FCN1 FCN1 chr17 73072214 0.008287 1.078e-10 35363 TRUE reported textfile 2 1 TRUE

5223 rs5030049 C T C T 0.0868 0.0098 0.12203 NA FALSE FALSE FALSE GCST006061 0.0116 0.3976 537409 outcome TRUE reported 15582_25_FCN1_FCN1 FCN1 chr3 186733074 0.012571 5.023e-12 35368 TRUE reported textfile 2 1 TRUE

5224 rs7037264 A G A G 0.2388 -0.0095 0.3846 NA FALSE FALSE FALSE GCST006061 0.0073 0.1887 537409 outcome TRUE reported 15582_25_FCN1_FCN1 FCN1 chr9 134883366 0.008487 3.546e-174 35333 TRUE reported textfile 2 1 TRUE

5225 rs705016 T C T C 0.0542 0.0182 0.30763 NA FALSE FALSE FALSE GCST006061 0.0075 0.01538 537409 outcome TRUE reported 15582_25_FCN1_FCN1 FCN1 chr3 165725994 0.008944 1.362e-09 35339 TRUE reported textfile 2 1 TRUE

5226 rs74467699 C T C T -0.1521 -6.00E-04 0.02782 NA FALSE FALSE FALSE GCST006061 0.0299 0.9845 537409 outcome TRUE reported 15582_25_FCN1_FCN1 FCN1 chr9 134781604 0.025055 1.274e-09 35341 TRUE reported textfile 2 1 TRUE

5227 rs75446386 T C T C 0.114 -0.0045 0.0431 NA FALSE FALSE FALSE GCST006061 0.0246 0.8533 537409 outcome TRUE reported 15582_25_FCN1_FCN1 FCN1 chr9 135151528 0.020849 4.551e-08 35323 TRUE reported textfile 2 1 TRUE

5228 rs10922098 T C T C -0.1604 -0.009 0.40011 NA FALSE FALSE FALSE GCST006061 0.0075 0.2275 537409 outcome TRUE reported 9038_12_JTB_JTB JTB chr1 196695521 0.008196 2.823e-85 35338 TRUE reported textfile 2 1 TRUE

5229 rs1707652 T C T C 0.0878 0.0081 0.3102 NA FALSE FALSE FALSE GCST006061 0.0074 0.2701 537409 outcome TRUE reported 9038_12_JTB_JTB JTB chr3 165761011 0.008686 5.076e-24 35339 TRUE reported textfile 2 1 TRUE

5230 rs2256952 T C T C -0.0862 0.0028 0.25749 NA FALSE FALSE FALSE GCST006061 0.0084 0.7392 537409 outcome TRUE reported 9038_12_JTB_JTB JTB chr20 45391167 0.009098 2.67e-21 35368 TRUE reported textfile 2 1 TRUE

5231 rs1077782 C T C T -0.6012 0.0184 0.02255 NA FALSE FALSE FALSE GCST006061 0.0333 0.5809 537409 outcome TRUE reported 9754_33_NQO2_Quinone_reductase_2 NQO2 chr6 3026939 0.029347 2.891e-93 35382 TRUE reported textfile 2 1 TRUE

5232 rs114141799 T C T C 0.5098 -0.0118 0.01004 NA FALSE FALSE FALSE GCST006061 0.0463 0.7995 537409 outcome TRUE reported

9754_33_NQO2_Quinone_reductase_2 NQO2 chr6 2945983 0.044753 4.621e-30
35363 TRUE reported textfile 2 1 TRUE
5233 rs115311554 C T C T 1.0995 -0.0566 0.01847 NA FALSE FALSE FALSE
GCST006061 0.0393 0.1491 537409 outcome TRUE reported
9754_33_NQO2_Quinone_reductase_2 NQO2 chr6 2937060 0.03233 1e-200 35362
TRUE reported textfile 2 1 TRUE
5234 rs143586648 A C A C 0.2666 -0.0769 0.01031 NA FALSE FALSE FALSE
GCST006061 0.0545 0.1586 537409 outcome TRUE reported
9754_33_NQO2_Quinone_reductase_2 NQO2 chr6 1778755 0.043717 1.072e-09
35323 TRUE reported textfile 2 1 TRUE
5235 rs146932567 T C T C 0.3495 0.1046 0.01232 NA FALSE FALSE FALSE
GCST006061 0.0621 0.0919 537409 outcome TRUE reported
9754_33_NQO2_Quinone_reductase_2 NQO2 chr6 3008656 0.039787 1.574e-18
35363 TRUE reported textfile 2 1 TRUE
5236 rs17135727 T C T C -0.1506 -0.0025 0.29156 NA FALSE FALSE FALSE
GCST006061 0.0083 0.7644 537409 outcome TRUE reported
9754_33_NQO2_Quinone_reductase_2 NQO2 chr6 2786329 0.009683 1.534e-54
35362 TRUE reported textfile 2 1 TRUE
5237 rs17548252 T C T C -0.1813 0.0482 0.02217 NA FALSE FALSE FALSE
GCST006061 0.0327 0.1404 537409 outcome TRUE reported
9754_33_NQO2_Quinone_reductase_2 NQO2 chr6 3098696 0.030325 2.25e-09
35375 TRUE reported textfile 2 1 TRUE
5238 rs56336390 T C T C -0.5724 -0.0116 0.05893 NA FALSE FALSE FALSE
GCST006061 0.0157 0.4609 537409 outcome TRUE reported
9754_33_NQO2_Quinone_reductase_2 NQO2 chr6 2993984 0.018786 1e-200 35363
TRUE reported textfile 2 1 TRUE
5239 rs62391549 C T C T -0.6704 -0.0263 0.11159 NA FALSE FALSE FALSE
GCST006061 0.0145 0.0686 537409 outcome TRUE reported
9754_33_NQO2_Quinone_reductase_2 NQO2 chr6 2991774 0.009579 1e-200 35363
TRUE reported textfile 2 1 TRUE
5240 rs79087434 A G A G -0.2978 -0.0253 0.01964 NA FALSE FALSE FALSE
GCST006061 0.0315 0.4216 537409 outcome TRUE reported
9754_33_NQO2_Quinone_reductase_2 NQO2 chr6 3349864 0.031687 5.559e-21
35382 TRUE reported textfile 2 1 TRUE
5241 rs10778149 A G A G 0.114 -0.0131 0.27262 NA FALSE FALSE FALSE
GCST006061 0.0078 0.09187 537409 outcome TRUE reported 3198_4_IDS_IDS IDS
chr12 101771564 0.008938 2.967e-37 35364 TRUE reported textfile 2
1 TRUE
5242 rs10922098 T C T C -0.0529 -0.009 0.40011 NA FALSE FALSE FALSE
GCST006061 0.0075 0.2275 537409 outcome TRUE reported 3198_4_IDS_IDS IDS
chr1 196695521 0.008156 8.831e-11 35338 TRUE reported textfile 2
1 TRUE
5243 rs12132412 G A G A -0.0534 0.0055 0.34943 NA FALSE FALSE FALSE
GCST006061 0.0077 0.4794 537409 outcome TRUE reported 3198_4_IDS_IDS IDS
chr1 21493549 0.008398 2.032e-10 35374 TRUE reported textfile 2
1 TRUE
5244 rs9346804 A G A G -0.0918 0.0225 0.09534 NA FALSE FALSE FALSE
GCST006061 0.0108 0.03742 537409 outcome TRUE reported 3198_4_IDS_IDS IDS
chr6 159979996 0.013415 7.758e-12 35388 TRUE reported textfile 2
1 TRUE
5245 rs9365124 T C T C 0.0691 -0.0043 0.17446 NA FALSE FALSE FALSE
GCST006061 0.0098 0.6597 537409 outcome TRUE reported 3198_4_IDS_IDS IDS
chr6 160019851 0.010556 5.902e-11 35390 TRUE reported textfile 2
1 TRUE
5246 rs10778152 G A G A -0.118 0.0057 0.27867 NA FALSE FALSE FALSE
GCST006061 0.0078 0.4668 537409 outcome TRUE reported 4771_10_SMPDL3A_ASM3A
SMPDL3A chr12 101831973 0.009078 1.244e-38 35364 TRUE reported
textfile 2 1 TRUE
5247 rs140624338 C T C T 0.2527 0.0021 0.01047 NA FALSE FALSE FALSE
GCST006061 0.0539 0.9695 537409 outcome TRUE reported 4771_10_SMPDL3A_ASM3A
SMPDL3A chr12 101963654 0.039748 2.049e-10 35362 TRUE reported
textfile 2 1 TRUE
5248 rs146457388 G A G A 0.1363 -0.0375 0.03747 NA FALSE FALSE FALSE
GCST006061 0.0273 0.1701 537409 outcome TRUE reported 4771_10_SMPDL3A_ASM3A
SMPDL3A chr19 10181296 0.0215 2.306e-10 35375 TRUE reported textfile
2 1 TRUE

5249 rs28385609 T C T C -0.3023 0.0038 0.17027 NA FALSE FALSE FALSE
GCST006061 0.0108 0.7205 537409 outcome TRUE reported 4771_10_SMPDL3A_ASM3A
SMPDL3A chr6 122801319 0.010746 4.14e-174 35348 TRUE reported
textfile 2 1 TRUE
5250 rs523118 G T G T 0.0628 -0.0288 0.23079 NA FALSE FALSE FALSE
GCST006061 0.0085 0.0006911 537409 outcome TRUE reported
4771_10_SMPDL3A_ASM3A SMPDL3A chr3 136247046 0.009637 7.208e-11 35361
TRUE reported textfile 2 1 TRUE
5251 rs6542680 T C T C -0.2162 -0.0012 0.18362 NA FALSE FALSE FALSE
GCST006061 0.0091 0.8935 537409 outcome TRUE reported 4771_10_SMPDL3A_ASM3A
SMPDL3A chr2 3592552 0.010402 5.943e-96 35332 TRUE reported textfile
2 1 TRUE
5252 rs9401605 A G A G -0.0464 0.0166 0.42431 NA FALSE FALSE FALSE
GCST006061 0.0072 0.02143 537409 outcome TRUE reported 4771_10_SMPDL3A_ASM3A
SMPDL3A chr6 122875195 0.008232 1.737e-08 35351 TRUE reported
textfile 2 1 TRUE
5253 rs10778161 G A G A -0.0645 -0.0125 0.23651 NA FALSE FALSE FALSE
GCST006061 0.008 0.1199 537409 outcome TRUE reported 15426_5_NEU1_NEUR1
NEU1 chr12 101973606 0.009617 1.986e-11 35362 TRUE reported
textfile 2 1 TRUE
5254 rs112829508 G A G A 0.2204 0.0324 0.01055 NA FALSE FALSE FALSE
GCST006061 0.0319 0.3084 537409 outcome TRUE reported 15426_5_NEU1_NEUR1
NEU1 chr12 101226350 0.038346 9.049e-09 35360 TRUE reported
textfile 2 1 TRUE
5255 rs174564 G A G A 0.0572 0.0107 0.39357 NA FALSE FALSE FALSE
GCST006061 0.0075 0.1516 537409 outcome TRUE reported 15426_5_NEU1_NEUR1
NEU1 chr11 61820833 0.008283 5.002e-12 35367 TRUE reported
textfile 2 1 TRUE
5256 rs9346805 T C T C -0.0928 0.0233 0.1021 NA FALSE FALSE FALSE
GCST006061 0.0108 0.03134 537409 outcome TRUE reported 15426_5_NEU1_NEUR1
NEU1 chr6 160001183 0.013198 2.042e-12 35389 TRUE reported
textfile 2 1 TRUE
5257 rs1077834 C T C T -0.0639 0.0019 0.19627 NA FALSE FALSE FALSE
GCST006061 0.0084 0.825 537409 outcome TRUE reported 9416_77_CPM_CBPM CPM
chr15 58431280 0.010384 7.565e-10 35365 TRUE reported textfile 2
1 TRUE
5258 rs10822143 T C T C 0.0457 -0.0483 0.45345 NA FALSE FALSE FALSE
GCST006061 0.0071 1.255e-11 537409 outcome TRUE reported 9416_77_CPM_CBPM
CPM chr10 63128096 0.00835 4.425e-08 35350 TRUE reported textfile 2
1 TRUE
5259 rs11087520 T C T C -0.063 0.0023 0.43124 NA FALSE FALSE FALSE
GCST006061 0.0074 0.7572 537409 outcome TRUE reported 9416_77_CPM_CBPM CPM
chr20 25456191 0.008396 6.223e-14 35365 TRUE reported textfile 2
1 TRUE
5260 rs1129555 G A G A -0.0545 0.0056 0.24626 NA FALSE FALSE FALSE
GCST006061 0.0079 0.4809 537409 outcome TRUE reported 9416_77_CPM_CBPM CPM
chr10 112150963 0.00965 1.628e-08 35372 TRUE reported textfile 2 1
TRUE
5261 rs2244608 G A G A 0.0545 -0.0109 0.31579 NA FALSE FALSE FALSE
GCST006061 0.0076 0.1491 537409 outcome TRUE reported 9416_77_CPM_CBPM CPM
chr12 120979185 0.008926 1.025e-09 35365 TRUE reported textfile 2
1 TRUE
5262 rs2954021 G A G A -0.0936 -0.0016 0.45781 NA FALSE FALSE FALSE
GCST006061 0.0071 0.8249 537409 outcome TRUE reported 9416_77_CPM_CBPM CPM
chr8 125469835 0.008311 2.032e-29 35368 TRUE reported textfile 2
1 TRUE
5263 rs487766 T C T C 0.0604 0.0091 0.23742 NA FALSE FALSE FALSE
GCST006061 0.0086 0.2882 537409 outcome TRUE reported 9416_77_CPM_CBPM CPM
chr15 58401661 0.009697 4.708e-10 35364 TRUE reported textfile 2
1 TRUE
5264 rs760904 A G A G 0.0909 0.0098 0.19033 NA FALSE FALSE FALSE
GCST006061 0.0086 0.2519 537409 outcome TRUE reported 9416_77_CPM_CBPM CPM
chr6 24476325 0.010556 7.249e-18 35380 TRUE reported textfile 2
1 TRUE
5265 rs79391862 C A C A -0.1614 0.0467 0.06536 NA FALSE FALSE FALSE
GCST006061 0.0414 0.2586 537409 outcome TRUE reported 9416_77_CPM_CBPM CPM

chr15 53447229 0.016681 3.836e-22 35362 TRUE reported textfile 2
1 TRUE
5266 rs9379685 G A G A -0.0567 -0.012 0.25823 NA FALSE FALSE FALSE
GCST006061 0.0085 0.159 537409 outcome TRUE reported 9416_77_CPM_CBPM CPM
chr6 24711816 0.009525 2.638e-09 35380 TRUE reported textfile 2
1 TRUE
5267 rs10784951 C T C T -0.0621 -0.0144 0.13724 NA FALSE FALSE FALSE
GCST006061 0.0103 0.1616 537409 outcome TRUE reported 3435_53_FN1_FN1_4 FN1
chr12 40926689 0.011318 4.087e-08 35352 TRUE reported textfile 2
1 TRUE
5268 rs10788007 A G A G 0.0714 0.0013 0.12004 NA FALSE FALSE FALSE
GCST006061 0.0105 0.9049 537409 outcome TRUE reported 3519_3_CCL17_TARC
CCL17 chr10 119973718 0.012815 2.524e-08 35380 TRUE reported
textfile 2 1 TRUE
5269 rs10886430 G A G A -0.4028 0.0071 0.12424 NA FALSE FALSE FALSE
GCST006061 0.0118 0.5496 537409 outcome TRUE reported 3519_3_CCL17_TARC
CCL17 chr10 119250744 0.01251 1e-200 35379 TRUE reported textfile 2
1 TRUE
5270 rs111265267 T C T C 0.2063 -0.0781 0.02798 NA FALSE FALSE FALSE
GCST006061 0.0518 0.1315 537409 outcome TRUE reported 3519_3_CCL17_TARC
CCL17 chr3 42107320 0.025876 1.552e-15 35352 TRUE reported
textfile 2 1 TRUE
5271 rs115952894 G A G A -0.4867 -0.0016 0.06768 NA FALSE FALSE FALSE
GCST006061 0.0172 0.9281 537409 outcome TRUE reported 3519_3_CCL17_TARC
CCL17 chr3 16908867 0.016461 3.986e-192 35371 TRUE reported
textfile 2 1 TRUE
5272 rs11718659 T C T C -0.0506 0.0061 0.34775 NA FALSE FALSE FALSE
GCST006061 0.0076 0.4215 537409 outcome TRUE reported 3519_3_CCL17_TARC
CCL17 chr3 42386995 0.008925 1.431e-08 35350 TRUE reported
textfile 2 1 TRUE
5273 rs13078687 A G A G 0.1189 0.0208 0.14626 NA FALSE FALSE FALSE
GCST006061 0.0114 0.06872 537409 outcome TRUE reported 3519_3_CCL17_TARC
CCL17 chr3 18181950 0.011946 2.453e-23 35369 TRUE reported
textfile 2 1 TRUE
5274 rs139617453 A G A G 0.146 -0.0159 0.03324 NA FALSE FALSE FALSE
GCST006061 0.0314 0.613 537409 outcome TRUE reported 3519_3_CCL17_TARC
CCL17 chr3 16928113 0.023779 8.253e-10 35371 TRUE reported
textfile 2 1 TRUE
5275 rs148393806 A G A G -0.16 0.003 0.02493 NA FALSE FALSE FALSE
GCST006061 0.0311 0.9232 537409 outcome TRUE reported 3519_3_CCL17_TARC
CCL17 chr3 16241844 0.027209 4.095e-09 35368 TRUE reported
textfile 2 1 TRUE
5276 rs150210110 T C T C 0.1237 -0.0033 0.04064 NA FALSE FALSE FALSE
GCST006061 0.035 0.9244 537409 outcome TRUE reported 3519_3_CCL17_TARC
CCL17 chr3 16273310 0.021234 5.695e-09 35369 TRUE reported
textfile 2 1 TRUE
5277 rs17098260 C T C T -0.0927 0.0087 0.11952 NA FALSE FALSE FALSE
GCST006061 0.012 0.4708 537409 outcome TRUE reported 3519_3_CCL17_TARC
CCL17 chr10 118957262 0.01302 1.082e-12 35379 TRUE reported textfile
2 1 TRUE
5278 rs2228467 C T C T 0.4553 0.0048 0.05811 NA FALSE FALSE FALSE
GCST006061 0.0154 0.7554 537409 outcome TRUE reported 3519_3_CCL17_TARC
CCL17 chr3 42864624 0.017893 7.941e-143 35353 TRUE reported
textfile 2 1 TRUE
5279 rs223895 C T C T -0.1425 0.0088 0.38537 NA FALSE FALSE FALSE
GCST006061 0.0074 0.2356 537409 outcome TRUE reported 3519_3_CCL17_TARC
CCL17 chr16 57406984 0.008644 4.65e-61 35373 TRUE reported
textfile 2 1 TRUE
5280 rs28695417 A G A G 0.3985 -0.0037 0.07461 NA FALSE FALSE FALSE
GCST006061 0.0131 0.7749 537409 outcome TRUE reported 3519_3_CCL17_TARC
CCL17 chr16 57410006 0.015945 7.567e-138 35373 TRUE reported
textfile 2 1 TRUE
5281 rs35216408 T C T C 0.1483 -0.0124 0.22431 NA FALSE FALSE FALSE
GCST006061 0.0092 0.1768 537409 outcome TRUE reported 3519_3_CCL17_TARC
CCL17 chr3 17093803 0.010166 3.357e-48 35371 TRUE reported
textfile 2 1 TRUE

5282 rs35574803 C T C T -0.1417 -0.037 0.03889 NA FALSE FALSE FALSE
GCST006061 0.0252 0.1424 537409 outcome TRUE reported 3519_3_CCL17_TARC
CCL17 chr3 32491754 0.021804 8.1e-11 35351 TRUE reported textfile
2 1 TRUE
5283 rs3785155 A G A G -0.0697 -0.0103 0.14526 NA FALSE FALSE FALSE
GCST006061 0.011 0.3506 537409 outcome TRUE reported 3519_3_CCL17_TARC
CCL17 chr16 55688478 0.011902 4.731e-09 35371 TRUE reported
textfile 2 1 TRUE
5284 rs4343578 C T C T -0.0568 0.021 0.23344 NA FALSE FALSE FALSE
GCST006061 0.0088 0.01712 537409 outcome TRUE reported 3519_3_CCL17_TARC
CCL17 chr3 33011258 0.010009 1.387e-08 35353 TRUE reported
textfile 2 1 TRUE
5285 rs4683159 G A G A -0.0504 0.0067 0.4011 NA FALSE FALSE FALSE
GCST006061 0.0077 0.3841 537409 outcome TRUE reported 3519_3_CCL17_TARC
CCL17 chr3 45983521 0.008661 5.907e-09 35346 TRUE reported
textfile 2 1 TRUE
5286 rs74597484 G A G A 0.1047 -0.0027 0.07014 NA FALSE FALSE FALSE
GCST006061 0.0196 0.8887 537409 outcome TRUE reported 3519_3_CCL17_TARC
CCL17 chr3 43216074 0.016611 2.921e-10 35352 TRUE reported
textfile 2 1 TRUE
5287 rs10789520 C T C T 0.1811 -0.0276 0.10507 NA FALSE FALSE FALSE
GCST006061 0.0104 0.00799 537409 outcome TRUE reported 2828_82_SPINT1_HAI_1
SPINT1 chr1 38923572 0.013592 1.68e-40 35376 TRUE reported
textfile 2 1 TRUE
5288 rs10922098 T C T C -0.1106 -0.009 0.40011 NA FALSE FALSE FALSE
GCST006061 0.0075 0.2275 537409 outcome TRUE reported 2828_82_SPINT1_HAI_1
SPINT1 chr1 196695521 0.008568 4.031e-38 35338 TRUE reported
textfile 2 1 TRUE
5289 rs10991614 G A G A 0.0958 0.0049 0.08197 NA FALSE FALSE FALSE
GCST006061 0.0128 0.7035 537409 outcome TRUE reported 2828_82_SPINT1_HAI_1
SPINT1 chr9 105305190 0.015011 1.748e-10 35362 TRUE reported
textfile 2 1 TRUE
5290 rs138965969 C T C T -0.1383 -0.0374 0.0358 NA FALSE FALSE FALSE
GCST006061 0.0305 0.2198 537409 outcome TRUE reported 2828_82_SPINT1_HAI_1
SPINT1 chr1 41008613 0.022985 1.778e-09 35373 TRUE reported
textfile 2 1 TRUE
5291 rs17658212 T C T C -0.3977 -0.0236 0.04753 NA FALSE FALSE FALSE
GCST006061 0.0167 0.1577 537409 outcome TRUE reported 2828_82_SPINT1_HAI_1
SPINT1 chr15 40853721 0.019338 5.606e-94 35364 TRUE reported
textfile 2 1 TRUE
5292 rs2419900 T C T C 0.0647 -0.0095 0.45218 NA FALSE FALSE FALSE
GCST006061 0.0072 0.1872 537409 outcome TRUE reported 2828_82_SPINT1_HAI_1
SPINT1 chr2 60287229 0.008266 5.003e-15 35348 TRUE reported
textfile 2 1 TRUE
5293 rs41277680 A G A G -0.1462 -0.0218 0.04032 NA FALSE FALSE FALSE
GCST006061 0.0238 0.36 537409 outcome TRUE reported 2828_82_SPINT1_HAI_1
SPINT1 chr15 40297623 0.020832 2.249e-12 35361 TRUE reported
textfile 2 1 TRUE
5294 rs62061425 G A G A 0.0718 0.001 0.21627 NA FALSE FALSE FALSE
GCST006061 0.0097 0.9151 537409 outcome TRUE reported 2828_82_SPINT1_HAI_1
SPINT1 chr17 7169772 0.010052 9.149e-13 35370 TRUE reported textfile
2 1 TRUE
5295 rs6445035 A G A G 0.0617 0.0154 0.1904 NA FALSE FALSE FALSE
GCST006061 0.0089 0.08256 537409 outcome TRUE reported 2828_82_SPINT1_HAI_1
SPINT1 chr3 165762312 0.010471 3.81e-09 35339 TRUE reported
textfile 2 1 TRUE
5296 rs708686 T C T C 0.0779 -4.00E-04 0.23081 NA FALSE FALSE FALSE
GCST006061 0.0081 0.9647 537409 outcome TRUE reported 2828_82_SPINT1_HAI_1
SPINT1 chr19 5840608 0.009745 1.31e-15 35362 TRUE reported textfile
2 1 TRUE
5297 rs76769476 C T C T -0.0924 -0.0039 0.09791 NA FALSE FALSE FALSE
GCST006061 0.0141 0.783 537409 outcome TRUE reported 2828_82_SPINT1_HAI_1
SPINT1 chr1 38580633 0.013936 3.353e-11 35371 TRUE reported
textfile 2 1 TRUE
5298 rs10791612 T C T C -0.1157 -0.0043 0.10143 NA FALSE FALSE FALSE
GCST006061 0.0116 0.7093 537409 outcome TRUE reported 9341_1_PDGFD_PDGFD

PDGFD chr11 103325056 0.013665 2.515e-17 35364 TRUE reported
textfile 2 1 TRUE
5299 rs146414450 A G A G -0.1326 0.0033 0.04874 NA FALSE FALSE FALSE
GCST006061 0.0192 0.8645 537409 outcome TRUE reported 9341_1_PDGFD_PDGFD
PDGFD chr11 102814919 0.018882 2.177e-12 35364 TRUE reported
textfile 2 1 TRUE
5300 rs34377578 C A C A -0.0578 0.0017 0.19239 NA FALSE FALSE FALSE
GCST006061 0.0087 0.8446 537409 outcome TRUE reported 9341_1_PDGFD_PDGFD
PDGFD chr10 102576669 0.010506 3.76e-08 35370 TRUE reported
textfile 2 1 TRUE
5301 rs7104359 C T C T -0.1294 0.0012 0.19973 NA FALSE FALSE FALSE
GCST006061 0.0099 0.9045 537409 outcome TRUE reported 9341_1_PDGFD_PDGFD
PDGFD chr11 103826689 0.010352 7.45e-36 35364 TRUE reported
textfile 2 1 TRUE
5302 rs7927668 A G A G -0.0498 -0.0187 0.31394 NA FALSE FALSE FALSE
GCST006061 0.0077 0.01579 537409 outcome TRUE reported 9341_1_PDGFD_PDGFD
PDGFD chr11 102263369 0.008971 2.834e-08 35362 TRUE reported
textfile 2 1 TRUE
5303 rs9807368 C T C T 0.0577 -0.0106 0.21308 NA FALSE FALSE FALSE
GCST006061 0.0085 0.2154 537409 outcome TRUE reported 9341_1_PDGFD_PDGFD
PDGFD chr18 9620161 0.010055 9.571e-09 35369 TRUE reported textfile
2 1 TRUE
5304 rs10794998 C T C T 0.0604 -0.0067 0.19432 NA FALSE FALSE FALSE
GCST006061 0.0099 0.4977 537409 outcome TRUE reported 5690_49_TUFT1_TUFT1
TUFT1 chr10 3058556 0.010064 1.951e-09 35264 TRUE reported textfile
2 1 TRUE
5305 rs1706435 G A G A 0.0492 0.0117 0.36981 NA FALSE FALSE FALSE
GCST006061 0.0072 0.1047 537409 outcome TRUE reported 5690_49_TUFT1_TUFT1
TUFT1 chr3 165743688 0.008205 2.014e-09 35339 TRUE reported
textfile 2 1 TRUE
5306 rs10796982 T C T C 0.0449 -0.0044 0.46537 NA FALSE FALSE FALSE
GCST006061 0.0072 0.5352 537409 outcome TRUE reported 12479_50_PRKAR1B_KAP1
PRKAR1B chr1 157673406 0.007856 1.094e-08 35356 TRUE reported
textfile 2 1 TRUE
5307 rs10922098 T C T C -0.0601 -0.009 0.40011 NA FALSE FALSE FALSE
GCST006061 0.0075 0.2275 537409 outcome TRUE reported 12479_50_PRKAR1B_KAP1
PRKAR1B chr1 196695521 0.008015 6.45e-14 35329 TRUE reported
textfile 2 1 TRUE
5308 rs12897483 T G T G -0.0572 0.0072 0.25403 NA FALSE FALSE FALSE
GCST006061 0.0138 0.6038 537409 outcome TRUE reported 12479_50_PRKAR1B_KAP1
PRKAR1B chr14 106048187 0.009302 7.777e-10 34957 TRUE reported
textfile 2 1 TRUE
5309 rs1079783 A G A G 0.0563 -0.0077 0.27994 NA FALSE FALSE FALSE
GCST006061 0.0079 0.331 537409 outcome TRUE reported 6379_62_ADAMTSL2_ATL2
ADAMTSL2 chr9 133597741 0.009376 1.919e-09 35371 TRUE reported
textfile 2 1 TRUE
5310 rs11534419 T C T C 0.1118 0.0154 0.39288 NA FALSE FALSE FALSE
GCST006061 0.0081 0.05784 537409 outcome TRUE reported 6379_62_ADAMTSL2_ATL2
ADAMTSL2 chr9 133523101 0.008717 1.176e-37 35360 TRUE reported
textfile 2 1 TRUE
5311 rs2027962 A C A C -0.0687 0.0058 0.40411 NA FALSE FALSE FALSE
GCST006061 0.0074 0.4358 537409 outcome TRUE reported 6379_62_ADAMTSL2_ATL2
ADAMTSL2 chr9 133720519 0.008612 1.491e-15 35370 TRUE reported
textfile 2 1 TRUE
5312 rs217184 C T C T 0.0677 0.0134 0.18348 NA FALSE FALSE FALSE
GCST006061 0.0091 0.1398 537409 outcome TRUE reported 6379_62_ADAMTSL2_ATL2
ADAMTSL2 chr16 72072066 0.010951 6.328e-10 35375 TRUE reported
textfile 2 1 TRUE
5313 rs56254331 C A C A 0.0909 9e-04 0.18291 NA FALSE FALSE FALSE
GCST006061 0.0108 0.9297 537409 outcome TRUE reported 6379_62_ADAMTSL2_ATL2
ADAMTSL2 chr19 41320115 0.010928 8.949e-17 35375 TRUE reported
textfile 2 1 TRUE
5314 rs61830291 C A C A 0.1328 -0.0236 0.10488 NA FALSE FALSE FALSE
GCST006061 0.0132 0.07315 537409 outcome TRUE reported 6379_62_ADAMTSL2_ATL2
ADAMTSL2 chr1 220827800 0.013809 6.781e-22 35353 TRUE reported
textfile 2 1 TRUE

5315 rs77262773 T C T C 0.2174 0.014 0.02263 NA FALSE FALSE FALSE
GCST006061 0.0283 0.6205 537409 outcome TRUE reported 6379_62_ADAMTSL2_ATL2
ADAMTSL2 chr17 69253570 0.027953 7.402e-15 35371 TRUE reported
textfile 2 1 TRUE
5316 rs10800448 G A G A -0.0798 -4.00E-04 0.12252 NA FALSE FALSE FALSE
GCST006061 0.0092 0.9646 537409 outcome TRUE reported 10008_43_GUCA1A_GUC1A
GUCA1A chr1 169491168 0.012035 3.342e-11 35351 TRUE reported
textfile 2 1 TRUE
5317 rs115977231 A G A G -0.2245 0.0568 0.01907 NA FALSE FALSE FALSE
GCST006061 0.0352 0.1065 537409 outcome TRUE reported 10008_43_GUCA1A_GUC1A
GUCA1A chr1 169097491 0.028396 2.657e-15 35349 TRUE reported
textfile 2 1 TRUE
5318 rs12765810 A G A G 0.0738 -0.0148 0.28052 NA FALSE FALSE FALSE
GCST006061 0.0078 0.05666 537409 outcome TRUE reported 10008_43_GUCA1A_GUC1A
GUCA1A chr10 19927245 0.008796 4.866e-17 35349 TRUE reported
textfile 2 1 TRUE
5319 rs184815320 G A G A -0.2109 -0.0256 0.01124 NA FALSE FALSE FALSE
GCST006061 0.0525 0.6257 537409 outcome TRUE reported 10008_43_GUCA1A_GUC1A
GUCA1A chr1 168215004 0.038254 3.525e-08 35356 TRUE reported
textfile 2 1 TRUE
5320 rs2239853 C T C T -0.077 0.0039 0.44202 NA FALSE FALSE FALSE
GCST006061 0.0073 0.5928 537409 outcome TRUE reported 10008_43_GUCA1A_GUC1A
GUCA1A chr1 169556528 0.008014 7.37e-22 35341 TRUE reported
textfile 2 1 TRUE
5321 rs61806985 T C T C -0.456 -0.0247 0.07704 NA FALSE FALSE FALSE
GCST006061 0.0227 0.2752 537409 outcome TRUE reported 10008_43_GUCA1A_GUC1A
GUCA1A chr1 169330603 0.014532 1e-200 35352 TRUE reported textfile
2 1 TRUE
5322 rs62140549 G A G A 0.0526 -3.00E-04 0.30898 NA FALSE FALSE FALSE
GCST006061 0.008 0.9672 537409 outcome TRUE reported 10008_43_GUCA1A_GUC1A
GUCA1A chr2 46914826 0.008538 7.252e-10 35346 TRUE reported
textfile 2 1 TRUE
5323 rs704 A G A G 0.0546 -0.0155 0.49189 NA FALSE FALSE FALSE GCST006061
0.0072 0.03144 537409 outcome TRUE reported 12503_5_GPR68_SPR1 GPR68 chr17
28367840 0.008002 8.903e-12 35351 TRUE reported textfile 2 1 TRUE
5324 rs10800475 T C T C -0.0726 0.0255 0.14298 NA FALSE FALSE FALSE
GCST006061 0.0094 0.006453 537409 outcome TRUE reported 8034_6_DRGX_DRGX
DRGX chr1 169775177 0.011761 6.712e-10 35363 TRUE reported
textfile 2 1 TRUE
5325 rs111605355 G T G T 0.2258 -0.0247 0.01228 NA FALSE FALSE FALSE
GCST006061 0.0379 0.514 537409 outcome TRUE reported 8034_6_DRGX_DRGX
DRGX chr1 168896600 0.036465 5.931e-10 35360 TRUE reported
textfile 2 1 TRUE
5326 rs12734872 A G A G 0.058 -0.0065 0.20997 NA FALSE FALSE FALSE
GCST006061 0.0101 0.5174 537409 outcome TRUE reported 8034_6_DRGX_DRGX
DRGX chr1 182304341 0.009932 5.238e-09 35361 TRUE reported
textfile 2 1 TRUE
5327 rs1602244 T C T C -0.0821 0.0067 0.25048 NA FALSE FALSE FALSE
GCST006061 0.0081 0.4108 537409 outcome TRUE reported 8034_6_DRGX_DRGX
DRGX chr1 171629800 0.009318 1.24e-18 35362 TRUE reported
textfile 2 1 TRUE
5328 rs2050151 G A G A 0.1434 0.0275 0.3573 NA FALSE FALSE FALSE
GCST006061 0.0074 0.0002073 537409 outcome TRUE reported 8034_6_DRGX_DRGX
DRGX chr1 170989323 0.008495 6.207e-64 35358 TRUE reported
textfile 2 1 TRUE
5329 rs61801570 G A G A 0.1861 0.0321 0.02959 NA FALSE FALSE FALSE
GCST006061 0.0241 0.1839 537409 outcome TRUE reported 8034_6_DRGX_DRGX
DRGX chr1 168694375 0.023728 4.395e-15 35362 TRUE reported
textfile 2 1 TRUE
5330 rs61826205 G T G T 0.4537 0.081 0.10901 NA FALSE FALSE FALSE
GCST006061 0.0141 8.698e-09 537409 outcome TRUE reported 8034_6_DRGX_DRGX
DRGX chr1 170590485 0.012861 1e-200 35359 TRUE reported textfile
2 1 TRUE
5331 rs6427276 A G A G 0.0908 0.0311 0.45246 NA FALSE FALSE FALSE
GCST006061 0.0073 1.99e-05 537409 outcome TRUE reported 8034_6_DRGX_DRGX
DRGX chr1 170938207 0.008158 8.881e-29 35359 TRUE reported

textfile 2 1 TRUE
5332 rs7546274 A G A G 0.1017 0.0852 0.0499 NA FALSE FALSE FALSE
GCST006061 0.0368 0.02061 537409 outcome TRUE reported 8034_6_DRGX_DRGX
DRGX chr1 167545611 0.018632 4.806e-08 35360 TRUE reported
textfile 2 1 TRUE
5333 rs10801559 A G A G -0.088 -8.00E-04 0.38662 NA FALSE FALSE FALSE
GCST006061 0.0072 0.9119 537409 outcome TRUE reported 5803_24_C3_C3d C3
chr1 196735074 0.008258 1.634e-26 35339 TRUE reported textfile 2
1 TRUE
5334 rs10801660 A G A G 0.0776 -0.0119 0.11574 NA FALSE FALSE FALSE
GCST006061 0.0108 0.2735 537409 outcome TRUE reported
16021_30_CDH8_Cadherin_8 CDH8 chr1 198272225 0.012557 6.413e-10 35345
TRUE reported textfile 2 1 TRUE
5335 rs111696008 A G A G -0.1761 -0.0259 0.02443 NA FALSE FALSE FALSE
GCST006061 0.0401 0.519 537409 outcome TRUE reported
16021_30_CDH8_Cadherin_8 CDH8 chr2 216622986 0.026715 4.342e-11 35359
TRUE reported textfile 2 1 TRUE
5336 rs1329424 G T G T -0.2959 -0.0095 0.39735 NA FALSE FALSE FALSE
GCST006061 0.0075 0.2071 537409 outcome TRUE reported
16021_30_CDH8_Cadherin_8 CDH8 chr1 196677046 0.008141 1e-200 35338
TRUE reported textfile 2 1 TRUE
5337 rs144202860 A G A G -0.1985 -0.0629 0.01274 NA FALSE FALSE FALSE
GCST006061 0.0538 0.2423 537409 outcome TRUE reported
16021_30_CDH8_Cadherin_8 CDH8 chr2 216625740 0.035765 2.855e-08 35359
TRUE reported textfile 2 1 TRUE
5338 rs2686395 C T C T -0.1544 -0.0077 0.3101 NA FALSE FALSE FALSE
GCST006061 0.0074 0.2965 537409 outcome TRUE reported
16021_30_CDH8_Cadherin_8 CDH8 chr3 165767231 0.008652 3.161e-71 35339
TRUE reported textfile 2 1 TRUE
5339 rs429358 C T C T 0.0607 0.005 0.17407 NA FALSE FALSE FALSE
GCST006061 0.0103 0.6319 537409 outcome TRUE reported
16021_30_CDH8_Cadherin_8 CDH8 chr19 44908684 0.010712 1.456e-08 35368
TRUE reported textfile 2 1 TRUE
5340 rs72748420 T C T C 0.1009 0.0095 0.07417 NA FALSE FALSE FALSE
GCST006061 0.0134 0.4784 537409 outcome TRUE reported
16021_30_CDH8_Cadherin_8 CDH8 chr1 196141224 0.015344 4.837e-11 35337
TRUE reported textfile 2 1 TRUE
5341 rs73169117 T C T C 0.0714 0.0051 0.11405 NA FALSE FALSE FALSE
GCST006061 0.0122 0.6755 537409 outcome TRUE reported
16021_30_CDH8_Cadherin_8 CDH8 chr3 167217620 0.01274 2.092e-08 35354
TRUE reported textfile 2 1 TRUE
5342 rs10801660 A G A G 0.0841 -0.0119 0.11574 NA FALSE FALSE FALSE
GCST006061 0.0108 0.2735 537409 outcome TRUE reported 18270_10_TAF10_TAF10
TAF10 chr1 198272225 0.01261 2.568e-11 35345 TRUE reported textfile
2 1 TRUE
5343 rs111696008 A G A G -0.1779 -0.0259 0.02443 NA FALSE FALSE FALSE
GCST006061 0.0401 0.519 537409 outcome TRUE reported 18270_10_TAF10_TAF10
TAF10 chr2 216622986 0.026777 3.06e-11 35359 TRUE reported
textfile 2 1 TRUE
5344 rs1329424 G T G T -0.3121 -0.0095 0.39735 NA FALSE FALSE FALSE
GCST006061 0.0075 0.2071 537409 outcome TRUE reported 18270_10_TAF10_TAF10
TAF10 chr1 196677046 0.006833 1e-200 35338 TRUE reported textfile
2 1 TRUE
5345 rs1707652 T C T C 0.1569 0.0081 0.3102 NA FALSE FALSE FALSE
GCST006061 0.0074 0.2701 537409 outcome TRUE reported 18270_10_TAF10_TAF10
TAF10 chr3 165761011 0.008674 3.869e-73 35339 TRUE reported
textfile 2 1 TRUE
5346 rs72748420 T C T C 0.1052 0.0095 0.07417 NA FALSE FALSE FALSE
GCST006061 0.0134 0.4784 537409 outcome TRUE reported 18270_10_TAF10_TAF10
TAF10 chr1 196141224 0.015396 8.321e-12 35337 TRUE reported
textfile 2 1 TRUE
5347 rs73169117 T C T C 0.0809 0.0051 0.11405 NA FALSE FALSE FALSE
GCST006061 0.0122 0.6755 537409 outcome TRUE reported 18270_10_TAF10_TAF10
TAF10 chr3 167217620 0.012775 2.41e-10 35354 TRUE reported
textfile 2 1 TRUE
5348 rs10801660 A G A G 0.0714 -0.0119 0.11574 NA FALSE FALSE FALSE

GCST006061 0.0108 0.2735 537409 outcome TRUE reported 9581_4_PRPF6_PRP6
PRPF6 chr1 198272225 0.012459 1e-08 35345 TRUE reported textfile
2 1 TRUE
5349 rs111696008 A G A G -0.1745 -0.0259 0.02443 NA FALSE FALSE FALSE
GCST006061 0.0401 0.519 537409 outcome TRUE reported 9581_4_PRPF6_PRP6
PRPF6 chr2 216622986 0.026572 5.13e-11 35359 TRUE reported
textfile 2 1 TRUE
5350 rs1329424 G T G T -0.2457 -0.0095 0.39735 NA FALSE FALSE FALSE
GCST006061 0.0075 0.2071 537409 outcome TRUE reported 9581_4_PRPF6_PRP6
PRPF6 chr1 196677046 0.008117 1e-200 35338 TRUE reported textfile
2 1 TRUE
5351 rs10801660 A G A G 0.0759 -0.0119 0.11574 NA FALSE FALSE FALSE
GCST006061 0.0108 0.2735 537409 outcome TRUE reported 15668_19_BMP8B_BMP_8
BMP8B chr1 198272225 0.012476 1.176e-09 35345 TRUE reported
textfile 2 1 TRUE
5352 rs111696008 A G A G -0.1472 -0.0259 0.02443 NA FALSE FALSE FALSE
GCST006061 0.0401 0.519 537409 outcome TRUE reported 15668_19_BMP8B_BMP_8
BMP8B chr2 216622986 0.026552 2.959e-08 35359 TRUE reported
textfile 2 1 TRUE
5353 rs1329424 G T G T -0.2786 -0.0095 0.39735 NA FALSE FALSE FALSE
GCST006061 0.0075 0.2071 537409 outcome TRUE reported 15668_19_BMP8B_BMP_8
BMP8B chr1 196677046 0.008097 1e-200 35338 TRUE reported textfile
2 1 TRUE
5354 rs2686395 C T C T -0.1141 -0.0077 0.3101 NA FALSE FALSE FALSE
GCST006061 0.0074 0.2965 537409 outcome TRUE reported 15668_19_BMP8B_BMP_8
BMP8B chr3 165767231 0.00861 4.426e-40 35339 TRUE reported textfile
2 1 TRUE
5355 rs10801660 A G A G 0.0775 -0.0119 0.11574 NA FALSE FALSE FALSE
GCST006061 0.0108 0.2735 537409 outcome TRUE reported
6020_52_UTS2_Urotensin_II UTS2 chr1 198272225 0.012594 7.565e-10 35345
TRUE reported textfile 2 1 TRUE
5356 rs1329424 G T G T -0.2929 -0.0095 0.39735 NA FALSE FALSE FALSE
GCST006061 0.0075 0.2071 537409 outcome TRUE reported
6020_52_UTS2_Urotensin_II UTS2 chr1 196677046 0.00817 1e-200 35338 TRUE
reported textfile 2 1 TRUE
5357 rs2686395 C T C T -0.1352 -0.0077 0.3101 NA FALSE FALSE FALSE
GCST006061 0.0074 0.2965 537409 outcome TRUE reported
6020_52_UTS2_Urotensin_II UTS2 chr3 165767231 0.008682 1.109e-54 35339
TRUE reported textfile 2 1 TRUE
5358 rs72748420 T C T C 0.1019 0.0095 0.07417 NA FALSE FALSE FALSE
GCST006061 0.0134 0.4784 537409 outcome TRUE reported
6020_52_UTS2_Urotensin_II UTS2 chr1 196141224 0.01539 3.562e-11 35337
TRUE reported textfile 2 1 TRUE
5359 rs10801660 A G A G 0.084 -0.0119 0.11574 NA FALSE FALSE FALSE
GCST006061 0.0108 0.2735 537409 outcome TRUE reported
17155_1_VPS28_VPS28_protein_homolog VPS28 chr1 198272225 0.01264 3.02e-11
35345 TRUE reported textfile 2 1 TRUE
5360 rs111696008 A G A G -0.1794 -0.0259 0.02443 NA FALSE FALSE FALSE
GCST006061 0.0401 0.519 537409 outcome TRUE reported
17155_1_VPS28_VPS28_protein_homolog VPS28 chr2 216622986 0.026899 2.569e-11
35359 TRUE reported textfile 2 1 TRUE
5361 rs12242 C T C T -0.1037 0.0149 0.07496 NA FALSE FALSE FALSE GCST006061
0.0166 0.3695 537409 outcome TRUE reported 17155_1_VPS28_VPS28_protein_homolog
VPS28 chr20 24983922 0.015393 1.62e-11 35366 TRUE reported
textfile 2 1 TRUE
5362 rs1329424 G T G T -0.2863 -0.0095 0.39735 NA FALSE FALSE FALSE
GCST006061 0.0075 0.2071 537409 outcome TRUE reported
17155_1_VPS28_VPS28_protein_homolog VPS28 chr1 196677046 0.008207 1e-200
35338 TRUE reported textfile 2 1 TRUE
5363 rs1707652 T C T C 0.1593 0.0081 0.3102 NA FALSE FALSE FALSE
GCST006061 0.0074 0.2701 537409 outcome TRUE reported
17155_1_VPS28_VPS28_protein_homolog VPS28 chr3 165761011 0.008713 1.117e-74
35339 TRUE reported textfile 2 1 TRUE
5364 rs61821527 T G T G -0.0476 -0.006 0.33477 NA FALSE FALSE FALSE
GCST006061 0.0076 0.4251 537409 outcome TRUE reported
17155_1_VPS28_VPS28_protein_homolog VPS28 chr1 201978878 0.008614 3.284e-08

35355 TRUE reported textfile 2 1 TRUE
5365 rs72748420 T C T C 0.1038 0.0095 0.07417 NA FALSE FALSE FALSE
GCST006061 0.0134 0.4784 537409 outcome TRUE reported
17155_1_VPS28_VPS28_protein_homolog VPS28 chr1 196141224 0.015442 1.796e-11
35337 TRUE reported textfile 2 1 TRUE
5366 rs73169117 T C T C 0.0841 0.0051 0.11405 NA FALSE FALSE FALSE
GCST006061 0.0122 0.6755 537409 outcome TRUE reported
17155_1_VPS28_VPS28_protein_homolog VPS28 chr3 167217620 0.012841 5.77e-11
35354 TRUE reported textfile 2 1 TRUE
5367 rs10801660 A G A G 0.0785 -0.0119 0.11574 NA FALSE FALSE FALSE
GCST006061 0.0108 0.2735 537409 outcome TRUE reported 19327_31_HAT1_Hat1
HAT1 chr1 198272225 0.012727 6.914e-10 35345 TRUE reported
textfile 2 1 TRUE
5368 rs111696008 A G A G -0.1496 -0.0259 0.02443 NA FALSE FALSE FALSE
GCST006061 0.0401 0.519 537409 outcome TRUE reported 19327_31_HAT1_Hat1
HAT1 chr2 216622986 0.027051 3.196e-08 35359 TRUE reported
textfile 2 1 TRUE
5369 rs1329424 G T G T -0.284 -0.0095 0.39735 NA FALSE FALSE FALSE
GCST006061 0.0075 0.2071 537409 outcome TRUE reported 19327_31_HAT1_Hat1
HAT1 chr1 196677046 0.00826 1e-200 35338 TRUE reported textfile 2
1 TRUE
5370 rs1367117 A G A G -0.0491 0.0055 0.35065 NA FALSE FALSE FALSE
GCST006061 0.0079 0.483 537409 outcome TRUE reported 19327_31_HAT1_Hat1
HAT1 chr2 21041028 0.008566 9.937e-09 35373 TRUE reported
textfile 2 1 TRUE
5371 rs1707652 T C T C 0.1227 0.0081 0.3102 NA FALSE FALSE FALSE
GCST006061 0.0074 0.2701 537409 outcome TRUE reported 19327_31_HAT1_Hat1
HAT1 chr3 165761011 0.008767 1.642e-44 35339 TRUE reported
textfile 2 1 TRUE
5372 rs72748418 A G A G 0.0941 0.0089 0.07164 NA FALSE FALSE FALSE
GCST006061 0.0129 0.4914 537409 outcome TRUE reported 19327_31_HAT1_Hat1
HAT1 chr1 196122755 0.01578 2.474e-09 35337 TRUE reported textfile
2 1 TRUE
5373 rs10801660 A G A G 0.082 -0.0119 0.11574 NA FALSE FALSE FALSE
GCST006061 0.0108 0.2735 537409 outcome TRUE reported 10734_339_GAL3ST2_G3ST2
GAL3ST2 chr1 198272225 0.012584 7.206e-11 35333 TRUE reported
textfile 2 1 TRUE
5374 rs111696008 A G A G -0.1615 -0.0259 0.02443 NA FALSE FALSE FALSE
GCST006061 0.0401 0.519 537409 outcome TRUE reported 10734_339_GAL3ST2_G3ST2
GAL3ST2 chr2 216622986 0.026773 1.618e-09 35347 TRUE reported
textfile 2 1 TRUE
5375 rs1329424 G T G T -0.2716 -0.0095 0.39735 NA FALSE FALSE FALSE
GCST006061 0.0075 0.2071 537409 outcome TRUE reported 10734_339_GAL3ST2_G3ST2
GAL3ST2 chr1 196677046 0.008175 1e-200 35326 TRUE reported textfile
2 1 TRUE
5376 rs144202860 A G A G -0.1954 -0.0629 0.01274 NA FALSE FALSE FALSE
GCST006061 0.0538 0.2423 537409 outcome TRUE reported 10734_339_GAL3ST2_G3ST2
GAL3ST2 chr2 216625740 0.035836 4.962e-08 35347 TRUE reported
textfile 2 1 TRUE
5377 rs2686395 C T C T -0.1455 -0.0077 0.3101 NA FALSE FALSE FALSE
GCST006061 0.0074 0.2965 537409 outcome TRUE reported 10734_339_GAL3ST2_G3ST2
GAL3ST2 chr3 165767231 0.008674 3.765e-63 35327 TRUE reported
textfile 2 1 TRUE
5378 rs72748420 T C T C 0.0917 0.0095 0.07417 NA FALSE FALSE FALSE
GCST006061 0.0134 0.4784 537409 outcome TRUE reported 10734_339_GAL3ST2_G3ST2
GAL3ST2 chr1 196141224 0.015358 2.362e-09 35325 TRUE reported
textfile 2 1 TRUE
5379 rs10801660 A G A G 0.0811 -0.0119 0.11574 NA FALSE FALSE FALSE
GCST006061 0.0108 0.2735 537409 outcome TRUE reported 8696_15_GAL3ST1_G3ST1
GAL3ST1 chr1 198272225 0.012634 1.369e-10 35345 TRUE reported
textfile 2 1 TRUE
5380 rs111696008 A G A G -0.1748 -0.0259 0.02443 NA FALSE FALSE FALSE
GCST006061 0.0401 0.519 537409 outcome TRUE reported 8696_15_GAL3ST1_G3ST1
GAL3ST1 chr2 216622986 0.026801 6.932e-11 35359 TRUE reported
textfile 2 1 TRUE
5381 rs1329424 G T G T -0.3127 -0.0095 0.39735 NA FALSE FALSE FALSE

GCST006061 0.0075 0.2071 537409 outcome TRUE reported 8696_15_GAL3ST1_G3ST1
GAL3ST1 chr1 196677046 0.006695 1e-200 35338 TRUE reported textfile
2 1 TRUE
5382 rs144202860 A G A G -0.1959 -0.0629 0.01274 NA FALSE FALSE FALSE
GCST006061 0.0538 0.2423 537409 outcome TRUE reported 8696_15_GAL3ST1_G3ST1
GAL3ST1 chr2 216625740 0.035867 4.714e-08 35359 TRUE reported
textfile 2 1 TRUE
5383 rs2686395 C T C T -0.1693 -0.0077 0.3101 NA FALSE FALSE FALSE
GCST006061 0.0074 0.2965 537409 outcome TRUE reported 8696_15_GAL3ST1_G3ST1
GAL3ST1 chr3 165767231 0.008681 1.045e-84 35339 TRUE reported
textfile 2 1 TRUE
5384 rs61821527 T G T G -0.0514 -0.006 0.33477 NA FALSE FALSE FALSE
GCST006061 0.0076 0.4251 537409 outcome TRUE reported 8696_15_GAL3ST1_G3ST1
GAL3ST1 chr1 201978878 0.008599 2.271e-09 35355 TRUE reported
textfile 2 1 TRUE
5385 rs704 A G A G 0.1665 -0.0155 0.49189 NA FALSE FALSE FALSE GCST006061
0.0072 0.03144 537409 outcome TRUE reported 8696_15_GAL3ST1_G3ST1 GAL3ST1
chr17 28367840 0.008048 4.516e-95 35360 TRUE reported textfile 2
1 TRUE
5386 rs72748420 T C T C 0.1125 0.0095 0.07417 NA FALSE FALSE FALSE
GCST006061 0.0134 0.4784 537409 outcome TRUE reported 8696_15_GAL3ST1_G3ST1
GAL3ST1 chr1 196141224 0.01541 2.87e-13 35337 TRUE reported textfile
2 1 TRUE
5387 rs73169117 T C T C 0.0806 0.0051 0.11405 NA FALSE FALSE FALSE
GCST006061 0.0122 0.6755 537409 outcome TRUE reported 8696_15_GAL3ST1_G3ST1
GAL3ST1 chr3 167217620 0.012802 3.059e-10 35354 TRUE reported
textfile 2 1 TRUE
5388 rs76315187 A C A C 0.1769 0.0164 0.01665 NA FALSE FALSE FALSE
GCST006061 0.0459 0.7211 537409 outcome TRUE reported 8696_15_GAL3ST1_G3ST1
GAL3ST1 chr3 165254582 0.031567 2.096e-08 35340 TRUE reported
textfile 2 1 TRUE
5389 rs10801660 A G A G 0.0801 -0.0119 0.11574 NA FALSE FALSE FALSE
GCST006061 0.0108 0.2735 537409 outcome TRUE reported 18449_33_PPIG_PPIG
PPIG chr1 198272225 0.012569 1.855e-10 35345 TRUE reported
textfile 2 1 TRUE
5390 rs111696008 A G A G -0.159 -0.0259 0.02443 NA FALSE FALSE FALSE
GCST006061 0.0401 0.519 537409 outcome TRUE reported 18449_33_PPIG_PPIG
PPIG chr2 216622986 0.026738 2.736e-09 35359 TRUE reported
textfile 2 1 TRUE
5391 rs1329424 G T G T -0.2891 -0.0095 0.39735 NA FALSE FALSE FALSE
GCST006061 0.0075 0.2071 537409 outcome TRUE reported 18449_33_PPIG_PPIG
PPIG chr1 196677046 0.008152 1e-200 35338 TRUE reported textfile
2 1 TRUE
5392 rs2686395 C T C T -0.1297 -0.0077 0.3101 NA FALSE FALSE FALSE
GCST006061 0.0074 0.2965 537409 outcome TRUE reported 18449_33_PPIG_PPIG
PPIG chr3 165767231 0.008666 1.2e-50 35339 TRUE reported textfile
2 1 TRUE
5393 rs72748420 T C T C 0.0921 0.0095 0.07417 NA FALSE FALSE FALSE
GCST006061 0.0134 0.4784 537409 outcome TRUE reported 18449_33_PPIG_PPIG
PPIG chr1 196141224 0.015351 1.978e-09 35337 TRUE reported
textfile 2 1 TRUE
5394 rs10802089 G A G A 0.2542 -7.00E-04 0.01477 NA FALSE FALSE FALSE
GCST006061 0.0293 0.9801 537409 outcome TRUE reported 15602_43_IL6R_IL_6_sRa
IL6R chr1 119310261 0.03567 1.03e-12 35360 TRUE reported textfile
2 1 TRUE
5395 rs111671159 G T G T 0.4464 0.0272 0.01384 NA FALSE FALSE FALSE
GCST006061 0.0611 0.6564 537409 outcome TRUE reported 15602_43_IL6R_IL_6_sRa
IL6R chr1 153633590 0.035563 3.864e-36 35361 TRUE reported
textfile 2 1 TRUE
5396 rs116103231 A G A G 0.3594 0.0268 0.01145 NA FALSE FALSE FALSE
GCST006061 0.0428 0.5322 537409 outcome TRUE reported 15602_43_IL6R_IL_6_sRa
IL6R chr1 119406313 0.040203 3.903e-19 35360 TRUE reported
textfile 2 1 TRUE
5397 rs12121180 T C T C 0.4319 -0.0057 0.14261 NA FALSE FALSE FALSE
GCST006061 0.011 0.6036 537409 outcome TRUE reported 15602_43_IL6R_IL_6_sRa
IL6R chr1 154309746 0.011704 1e-200 35363 TRUE reported textfile

2 1 TRUE
5398 rs142768042 T C T C -0.5482 -0.023 0.02836 NA FALSE FALSE FALSE
GCST006061 0.0247 0.352 537409 outcome TRUE reported 15602_43_IL6R_IL_6_sRa
IL6R chr1 154636279 0.024729 6.923e-109 35363 TRUE reported
textfile 2 1 TRUE
5399 rs145134231 A G A G 0.2169 0.0705 0.02038 NA FALSE FALSE FALSE
GCST006061 0.0286 0.01363 537409 outcome TRUE reported 15602_43_IL6R_IL_6_sRa
IL6R chr1 119726926 0.03029 8.019e-13 35362 TRUE reported textfile
2 1 TRUE
5400 rs148882162 G A G A 0.5783 -0.0297 0.02209 NA FALSE FALSE FALSE
GCST006061 0.0477 0.5344 537409 outcome TRUE reported 15602_43_IL6R_IL_6_sRa
IL6R chr1 154279712 0.02814 7.623e-94 35363 TRUE reported textfile
2 1 TRUE
5401 rs3184504 C T C T -0.0434 0.0074 0.37862 NA FALSE FALSE FALSE
GCST006061 0.0075 0.3235 537409 outcome TRUE reported 15602_43_IL6R_IL_6_sRa
IL6R chr12 111446804 0.007202 1.677e-09 35371 TRUE reported
textfile 2 1 TRUE
5402 rs34569656 G A G A -0.227 -0.0195 0.06567 NA FALSE FALSE FALSE
GCST006061 0.0176 0.266 537409 outcome TRUE reported 15602_43_IL6R_IL_6_sRa
IL6R chr1 154697660 0.01652 5.795e-43 35363 TRUE reported textfile
2 1 TRUE
5403 rs55953243 A G A G 0.2629 0.0633 0.03594 NA FALSE FALSE FALSE
GCST006061 0.0333 0.05755 537409 outcome TRUE reported 15602_43_IL6R_IL_6_sRa
IL6R chr1 153766423 0.022399 8.201e-32 35363 TRUE reported
textfile 2 1 TRUE
5404 rs57569414 A C A C 0.5269 0.0198 0.09706 NA FALSE FALSE FALSE
GCST006061 0.0106 0.06194 537409 outcome TRUE reported 15602_43_IL6R_IL_6_sRa
IL6R chr1 154407943 0.014032 1e-200 35364 TRUE reported textfile
2 1 TRUE
5405 rs72695200 T C T C -0.1622 -0.0038 0.04213 NA FALSE FALSE FALSE
GCST006061 0.0205 0.8546 537409 outcome TRUE reported 15602_43_IL6R_IL_6_sRa
IL6R chr1 119807763 0.020968 1.029e-14 35362 TRUE reported
textfile 2 1 TRUE
5406 rs10807291 G A G A -0.0849 0.0046 0.12116 NA FALSE FALSE FALSE
GCST006061 0.0105 0.6651 537409 outcome TRUE reported 8231_122_FLT1_VEGF_sR1
FLT1 chr6 43814181 0.013019 6.984e-11 35361 TRUE reported
textfile 2 1 TRUE
5407 rs114694170 C T C T -0.0971 0.0053 0.08378 NA FALSE FALSE FALSE
GCST006061 0.0175 0.7624 537409 outcome TRUE reported 8231_122_FLT1_VEGF_sR1
FLT1 chr5 88884379 0.015219 1.77e-10 35344 TRUE reported
textfile 2 1 TRUE
5408 rs13212731 T C T C -0.0949 0.0273 0.08377 NA FALSE FALSE FALSE
GCST006061 0.013 0.03528 537409 outcome TRUE reported 8231_122_FLT1_VEGF_sR1
FLT1 chr6 43846460 0.015487 8.91e-10 35362 TRUE reported
textfile 2 1 TRUE
5409 rs4426996 A G A G 0.1326 0.0013 0.1105 NA FALSE FALSE FALSE
GCST006061 0.012 0.9142 537409 outcome TRUE reported 8231_122_FLT1_VEGF_sR1
FLT1 chr6 43920200 0.013497 8.843e-23 35361 TRUE reported
textfile 2 1 TRUE
5410 rs56728557 A G A G -0.069 -0.0038 0.14126 NA FALSE FALSE FALSE
GCST006061 0.0104 0.7123 537409 outcome TRUE reported 8231_122_FLT1_VEGF_sR1
FLT1 chr13 28455294 0.012046 1.016e-08 35365 TRUE reported
textfile 2 1 TRUE
5411 rs7098181 T G T G -0.0566 -0.0471 0.43567 NA FALSE FALSE FALSE
GCST006061 0.0072 5.552e-11 537409 outcome TRUE reported
8231_122_FLT1_VEGF_sR1 FLT1 chr10 63267383 0.008451 2.117e-11 35351
TRUE reported textfile 2 1 TRUE
5412 rs7739450 A G A G 0.3108 0.0086 0.4652 NA FALSE FALSE FALSE
GCST006061 0.0074 0.2475 537409 outcome TRUE reported 8231_122_FLT1_VEGF_sR1
FLT1 chr6 43943861 0.008356 1e-200 35363 TRUE reported textfile
2 1 TRUE
5413 rs9472175 C T C T -0.0832 0.0073 0.16328 NA FALSE FALSE FALSE
GCST006061 0.0104 0.4807 537409 outcome TRUE reported 8231_122_FLT1_VEGF_sR1
FLT1 chr6 43967045 0.011422 3.24e-13 35362 TRUE reported
textfile 2 1 TRUE
5414 rs10813948 A G A G -0.0596 0.0081 0.2872 NA FALSE FALSE FALSE

GCST006061 0.0076 0.2907 537409 outcome TRUE reported 3440_7_GZMA_granzyme_A
GZMA chr9 33121572 0.008899 2.127e-11 35349 TRUE reported
textfile 2 1 TRUE
5415 rs10982456 C T C T -0.0573 -0.0162 0.46279 NA FALSE FALSE FALSE
GCST006061 0.0072 0.02384 537409 outcome TRUE reported 3440_7_GZMA_granzyme_A
GZMA chr9 114928478 0.008128 1.798e-12 35362 TRUE reported
textfile 2 1 TRUE
5416 rs116818570 A G A G -0.2106 -0.0142 0.02112 NA FALSE FALSE FALSE
GCST006061 0.0336 0.6729 537409 outcome TRUE reported 3440_7_GZMA_granzyme_A
GZMA chr5 53676913 0.028417 1.252e-13 35366 TRUE reported
textfile 2 1 TRUE
5417 rs12151752 C T C T -0.0562 -0.0065 0.21988 NA FALSE FALSE FALSE
GCST006061 0.0086 0.4509 537409 outcome TRUE reported 3440_7_GZMA_granzyme_A
GZMA chr2 85707529 0.009775 8.971e-09 35344 TRUE reported
textfile 2 1 TRUE
5418 rs139196635 A G A G 0.2042 0.0242 0.02061 NA FALSE FALSE FALSE
GCST006061 0.0312 0.4387 537409 outcome TRUE reported 3440_7_GZMA_granzyme_A
GZMA chr12 101863758 0.02887 1.516e-12 35364 TRUE reported textfile
2 1 TRUE
5419 rs139268409 A C A C -1.0049 0.0626 0.01041 NA FALSE FALSE FALSE
GCST006061 0.0504 0.2146 537409 outcome TRUE reported 3440_7_GZMA_granzyme_A
GZMA chr5 55030849 0.041617 8.121e-129 35361 TRUE reported
textfile 2 1 TRUE
5420 rs142021463 A G A G -0.3347 -0.0161 0.02004 NA FALSE FALSE FALSE
GCST006061 0.0297 0.5877 537409 outcome TRUE reported 3440_7_GZMA_granzyme_A
GZMA chr5 58068337 0.028809 3.347e-31 35369 TRUE reported
textfile 2 1 TRUE
5421 rs142390300 T C T C -0.2088 0.0124 0.03051 NA FALSE FALSE FALSE
GCST006061 0.0308 0.687 537409 outcome TRUE reported 3440_7_GZMA_granzyme_A
GZMA chr5 58581146 0.023962 2.938e-18 35370 TRUE reported
textfile 2 1 TRUE
5422 rs17710827 T G T G 0.1084 -0.0052 0.05471 NA FALSE FALSE FALSE
GCST006061 0.0213 0.8072 537409 outcome TRUE reported 3440_7_GZMA_granzyme_A
GZMA chr6 28088472 0.017856 1.274e-09 35444 TRUE reported
textfile 2 1 TRUE
5423 rs1860545 A G A G -0.046 -0.0017 0.40257 NA FALSE FALSE FALSE
GCST006061 0.0074 0.8175 537409 outcome TRUE reported 3440_7_GZMA_granzyme_A
GZMA chr12 6337611 0.008261 2.576e-08 35377 TRUE reported textfile
2 1 TRUE
5424 rs79634769 C T C T -0.1642 -0.0286 0.03204 NA FALSE FALSE FALSE
GCST006061 0.0347 0.4093 537409 outcome TRUE reported 3440_7_GZMA_granzyme_A
GZMA chr12 101745050 0.0238 5.234e-12 35364 TRUE reported textfile
2 1 TRUE
5425 rs80166608 G A G A -0.1598 -0.0167 0.05268 NA FALSE FALSE FALSE
GCST006061 0.0265 0.5295 537409 outcome TRUE reported 3440_7_GZMA_granzyme_A
GZMA chr5 55265301 0.018457 4.805e-18 35363 TRUE reported
textfile 2 1 TRUE
5426 rs9264638 A G A G 0.2368 0.0207 0.34219 NA FALSE FALSE FALSE
GCST006061 0.0082 0.01184 537409 outcome TRUE reported 3440_7_GZMA_granzyme_A
GZMA chr6 31270541 0.008452 1.019e-172 35439 TRUE reported
textfile 2 1 TRUE
5427 rs10813948 A G A G -0.0685 0.0081 0.2872 NA FALSE FALSE FALSE
GCST006061 0.0076 0.2907 537409 outcome TRUE reported 13427_66_MAN1C1_MA1C1
MAN1C1 chr9 33121572 0.009042 3.562e-14 35343 TRUE reported
textfile 2 1 TRUE
5428 rs111903631 C T C T 0.1871 0.0078 0.01817 NA FALSE FALSE FALSE
GCST006061 0.04 0.8455 537409 outcome TRUE reported 13427_66_MAN1C1_MA1C1
MAN1C1 chr19 48825936 0.030185 5.703e-10 35371 TRUE reported
textfile 2 1 TRUE
5429 rs11247595 C T C T -0.1905 0.0049 0.45473 NA FALSE FALSE FALSE
GCST006061 0.0075 0.5087 537409 outcome TRUE reported 13427_66_MAN1C1_MA1C1
MAN1C1 chr1 25682168 0.008274 2.754e-117 35363 TRUE reported
textfile 2 1 TRUE
5430 rs143794981 A G A G 0.2165 0.0293 0.01432 NA FALSE FALSE FALSE
GCST006061 0.0491 0.5504 537409 outcome TRUE reported 13427_66_MAN1C1_MA1C1
MAN1C1 chr19 47631841 0.034025 1.979e-10 35365 TRUE reported

textfile 2 1 TRUE
5431 rs333947 A G A G -0.0789 -0.0071 0.14937 NA FALSE FALSE FALSE
GCST006061 0.01 0.4808 537409 outcome TRUE reported 13427_66_MAN1C1_MA1C1
MAN1C1 chr1 109928142 0.011659 1.314e-11 35343 TRUE reported
textfile 2 1 TRUE
5432 rs34136392 G T G T -0.0507 0.0075 0.43179 NA FALSE FALSE FALSE
GCST006061 0.0085 0.3754 537409 outcome TRUE reported 13427_66_MAN1C1_MA1C1
MAN1C1 chr1 25558006 0.008382 1.461e-09 35361 TRUE reported
textfile 2 1 TRUE
5433 rs4704043 T C T C 0.054 0.0081 0.30863 NA FALSE FALSE FALSE
GCST006061 0.0077 0.2949 537409 outcome TRUE reported 13427_66_MAN1C1_MA1C1
MAN1C1 chr5 72863352 0.008884 1.213e-09 35348 TRUE reported
textfile 2 1 TRUE
5434 rs4801902 T C T C 0.1288 -0.0168 0.0647 NA FALSE FALSE FALSE
GCST006061 0.0221 0.4455 537409 outcome TRUE reported 13427_66_MAN1C1_MA1C1
MAN1C1 chr19 51936045 0.016603 8.668e-15 35378 TRUE reported
textfile 2 1 TRUE
5435 rs6589598 G A G A -0.0873 0.0061 0.23247 NA FALSE FALSE FALSE
GCST006061 0.0086 0.4778 537409 outcome TRUE reported 13427_66_MAN1C1_MA1C1
MAN1C1 chr11 117131687 0.009718 2.629e-19 35372 TRUE reported
textfile 2 1 TRUE
5436 rs10813949 A G A G -0.0714 0.0069 0.29174 NA FALSE FALSE FALSE
GCST006061 0.0076 0.3637 537409 outcome TRUE reported
5722_78_PRCP_Prolylcarboxypeptidase PRCP chr9 33123466 0.009039 2.818e-15
35349 TRUE reported textfile 2 1 TRUE
5437 rs11142435 G A G A 0.0655 -0.0021 0.18024 NA FALSE FALSE FALSE
GCST006061 0.0098 0.8333 537409 outcome TRUE reported
5722_78_PRCP_Prolylcarboxypeptidase PRCP chr9 70442697 0.010684 8.747e-10
35352 TRUE reported textfile 2 1 TRUE
5438 rs11233045 C A C A 0.0813 -0.0115 0.10355 NA FALSE FALSE FALSE
GCST006061 0.0136 0.3971 537409 outcome TRUE reported
5722_78_PRCP_Prolylcarboxypeptidase PRCP chr11 82237444 0.013564 2.052e-09
35343 TRUE reported textfile 2 1 TRUE
5439 rs140624338 C T C T 0.5615 0.0021 0.01047 NA FALSE FALSE FALSE
GCST006061 0.0539 0.9695 537409 outcome TRUE reported
5722_78_PRCP_Prolylcarboxypeptidase PRCP chr12 101963654 0.040839 5.159e-43
35362 TRUE reported textfile 2 1 TRUE
5440 rs28698773 C A C A 0.0662 -0.0092 0.14857 NA FALSE FALSE FALSE
GCST006061 0.0111 0.4079 537409 outcome TRUE reported
5722_78_PRCP_Prolylcarboxypeptidase PRCP chr5 39436142 0.01175 1.76e-08
35361 TRUE reported textfile 2 1 TRUE
5441 rs6924387 G A G A -0.0638 -0.0026 0.38794 NA FALSE FALSE FALSE
GCST006061 0.0072 0.7204 537409 outcome TRUE reported
5722_78_PRCP_Prolylcarboxypeptidase PRCP chr6 136761810 0.00847 4.976e-14
35364 TRUE reported textfile 2 1 TRUE
5442 rs80300795 A G A G 0.158 0.0198 0.03634 NA FALSE FALSE FALSE
GCST006061 0.0205 0.3354 537409 outcome TRUE reported
5722_78_PRCP_Prolylcarboxypeptidase PRCP chr12 101467581 0.022355 1.575e-12
35354 TRUE reported textfile 2 1 TRUE
5443 rs825038 A C A C 0.1349 0.0218 0.05118 NA FALSE FALSE FALSE
GCST006061 0.0239 0.3624 537409 outcome TRUE reported
5722_78_PRCP_Prolylcarboxypeptidase PRCP chr12 101570303 0.018853 8.334e-13
35363 TRUE reported textfile 2 1 TRUE
5444 rs9298506 G A G A 0.1724 -0.0112 0.24048 NA FALSE FALSE FALSE
GCST006061 0.0088 0.2016 537409 outcome TRUE reported
5722_78_PRCP_Prolylcarboxypeptidase PRCP chr8 54524964 0.009593 3.294e-72
35355 TRUE reported textfile 2 1 TRUE
5445 rs10814104 C T C T 0.0976 -0.0158 0.20887 NA FALSE FALSE FALSE
GCST006061 0.0088 0.07172 537409 outcome TRUE reported 19207_119_ADK_ADK ADK
chr9 34360289 0.00991 6.945e-23 35350 TRUE reported textfile 2 1
TRUE
5446 rs1354034 C T C T 0.0618 0.0088 0.38119 NA FALSE FALSE FALSE
GCST006061 0.0073 0.2248 537409 outcome TRUE reported 19207_119_ADK_ADK ADK
chr3 56815721 0.008294 9.246e-14 35356 TRUE reported textfile 2
1 TRUE
5447 rs4746172 T C T C 0.0561 -3.00E-04 0.25297 NA FALSE FALSE FALSE

GCST006061 0.0081 0.9739 537409 outcome TRUE reported 19207_119_ADK_ADK ADK
chr10 74096084 0.009233 1.232e-09 35369 TRUE reported textfile 2
1 TRUE
5448 rs586689 T C T C 0.053 -0.0108 0.30367 NA FALSE FALSE FALSE
GCST006061 0.0077 0.1604 537409 outcome TRUE reported 19207_119_ADK_ADK ADK
chr11 88186313 0.008752 1.4e-09 35351 TRUE reported textfile 2 1
TRUE
5449 rs10814111 A C A C 0.1822 0.0103 0.07849 NA FALSE FALSE FALSE
GCST006061 0.0159 0.5177 537409 outcome TRUE reported 16583_8_NUDT2_AP4A
NUDT2 chr9 34457854 0.014889 1.974e-34 35350 TRUE reported
textfile 2 1 TRUE
5450 rs111577519 A G A G -0.0729 0.0075 0.14021 NA FALSE FALSE FALSE
GCST006061 0.0119 0.5313 537409 outcome TRUE reported 16583_8_NUDT2_AP4A
NUDT2 chr9 33499936 0.011523 2.506e-10 35348 TRUE reported
textfile 2 1 TRUE
5451 rs1354034 C T C T 0.0476 0.0088 0.38119 NA FALSE FALSE FALSE
GCST006061 0.0073 0.2248 537409 outcome TRUE reported 16583_8_NUDT2_AP4A
NUDT2 chr3 56815721 0.008181 5.945e-09 35356 TRUE reported
textfile 2 1 TRUE
5452 rs7025269 T C T C 0.2999 -0.0152 0.20745 NA FALSE FALSE FALSE
GCST006061 0.0087 0.08281 537409 outcome TRUE reported 16583_8_NUDT2_AP4A
NUDT2 chr9 34336268 0.009776 1e-200 35350 TRUE reported textfile
2 1 TRUE
5453 rs10814194 C T C T 0.0643 -0.0031 0.21818 NA FALSE FALSE FALSE
GCST006061 0.0083 0.7084 537409 outcome TRUE reported 2516_57_CCL21_6Ckine
CCL21 chr9 35053111 0.009789 5.088e-11 35351 TRUE reported
textfile 2 1 TRUE
5454 rs10972211 A G A G 0.1461 0.004 0.14707 NA FALSE FALSE FALSE
GCST006061 0.0105 0.701 537409 outcome TRUE reported 2516_57_CCL21_6Ckine
CCL21 chr9 34729993 0.011399 1.309e-37 35349 TRUE reported
textfile 2 1 TRUE
5455 rs113349704 A G A G 0.3517 -0.0051 0.02689 NA FALSE FALSE FALSE
GCST006061 0.0283 0.8576 537409 outcome TRUE reported 2516_57_CCL21_6Ckine
CCL21 chr3 131431490 0.025049 8.833e-45 35358 TRUE reported
textfile 2 1 TRUE
5456 rs114194685 G A G A 0.1385 0.0074 0.03034 NA FALSE FALSE FALSE
GCST006061 0.0313 0.8139 537409 outcome TRUE reported 2516_57_CCL21_6Ckine
CCL21 chr3 134303269 0.023956 7.406e-09 35360 TRUE reported
textfile 2 1 TRUE
5457 rs116356943 A G A G 0.5709 -0.0044 0.02474 NA FALSE FALSE FALSE
GCST006061 0.0399 0.9116 537409 outcome TRUE reported 2516_57_CCL21_6Ckine
CCL21 chr3 133026780 0.026807 1.221e-100 35358 TRUE reported
textfile 2 1 TRUE
5458 rs11914615 C T C T -0.4509 -0.0095 0.12539 NA FALSE FALSE FALSE
GCST006061 0.0109 0.3818 537409 outcome TRUE reported 2516_57_CCL21_6Ckine
CCL21 chr3 132404638 0.012311 1e-200 35356 TRUE reported textfile
2 1 TRUE
5459 rs1264347 T C T C 0.0841 -0.0214 0.07994 NA FALSE FALSE FALSE
GCST006061 0.0118 0.07131 537409 outcome TRUE reported 2516_57_CCL21_6Ckine
CCL21 chr6 30830920 0.014821 1.393e-08 35439 TRUE reported
textfile 2 1 TRUE
5460 rs141300831 C T C T 0.1867 -0.0168 0.01884 NA FALSE FALSE FALSE
GCST006061 0.0454 0.7113 537409 outcome TRUE reported 2516_57_CCL21_6Ckine
CCL21 chr3 127814750 0.030729 1.234e-09 35360 TRUE reported
textfile 2 1 TRUE
5461 rs148360766 T G T G 0.4072 0.0392 0.02248 NA FALSE FALSE FALSE
GCST006061 0.0337 0.2451 537409 outcome TRUE reported 2516_57_CCL21_6Ckine
CCL21 chr3 131135924 0.027758 1.008e-48 35364 TRUE reported
textfile 2 1 TRUE
5462 rs17764730 T C T C -0.0811 0.0055 0.18947 NA FALSE FALSE FALSE
GCST006061 0.0084 0.5079 537409 outcome TRUE reported 2516_57_CCL21_6Ckine
CCL21 chr5 128021834 0.010257 2.638e-15 35348 TRUE reported
textfile 2 1 TRUE
5463 rs2600961 G A G A -0.1425 -0.0036 0.08805 NA FALSE FALSE FALSE
GCST006061 0.0142 0.7996 537409 outcome TRUE reported 2516_57_CCL21_6Ckine
CCL21 chr3 132895738 0.01447 6.989e-23 35358 TRUE reported textfile

2 1 TRUE
5464 rs6017250 C T C T 0.0524 0.0143 0.34146 NA FALSE FALSE FALSE
GCST006061 0.0082 0.0814 537409 outcome TRUE reported 2516_57_CCL21_6Ckine
CCL21 chr20 44032864 0.008483 6.536e-10 35369 TRUE reported
textfile 2 1 TRUE
5465 rs6077870 A G A G 0.0521 -0.019 0.2414 NA FALSE FALSE FALSE
GCST006061 0.0088 0.03119 537409 outcome TRUE reported 2516_57_CCL21_6Ckine
CCL21 chr20 10672061 0.009429 3.29e-08 35379 TRUE reported
textfile 2 1 TRUE
5466 rs6715061 A G A G -0.067 0.0126 0.36578 NA FALSE FALSE FALSE
GCST006061 0.0076 0.09709 537409 outcome TRUE reported 2516_57_CCL21_6Ckine
CCL21 chr2 65363202 0.008337 9.262e-16 35353 TRUE reported
textfile 2 1 TRUE
5467 rs677106 T G T G 0.0447 2e-04 0.49553 NA FALSE FALSE FALSE
GCST006061 0.0072 0.979 537409 outcome TRUE reported 2516_57_CCL21_6Ckine
CCL21 chr11 75358827 0.008028 2.576e-08 35371 TRUE reported
textfile 2 1 TRUE
5468 rs6871748 C T C T -0.0538 0.0017 0.32803 NA FALSE FALSE FALSE
GCST006061 0.0081 0.837 537409 outcome TRUE reported 2516_57_CCL21_6Ckine
CCL21 chr5 35885880 0.008614 4.212e-10 35380 TRUE reported
textfile 2 1 TRUE
5469 rs7212344 C A C A -0.0572 2e-04 0.20559 NA FALSE FALSE FALSE
GCST006061 0.0086 0.9831 537409 outcome TRUE reported 2516_57_CCL21_6Ckine
CCL21 chr17 49376575 0.009995 1.046e-08 35371 TRUE reported
textfile 2 1 TRUE
5470 rs74765117 T C T C 0.2886 0.0132 0.01669 NA FALSE FALSE FALSE
GCST006061 0.0395 0.7388 537409 outcome TRUE reported 2516_57_CCL21_6Ckine
CCL21 chr3 129371423 0.031801 1.134e-19 35358 TRUE reported
textfile 2 1 TRUE
5471 rs80252447 T C T C 0.1236 -0.0013 0.04507 NA FALSE FALSE FALSE
GCST006061 0.0208 0.9501 537409 outcome TRUE reported 2516_57_CCL21_6Ckine
CCL21 chr3 128478584 0.019524 2.442e-10 35361 TRUE reported
textfile 2 1 TRUE
5472 rs9551444 T C T C 0.0569 0.0014 0.2206 NA FALSE FALSE FALSE
GCST006061 0.0084 0.8694 537409 outcome TRUE reported 2516_57_CCL21_6Ckine
CCL21 chr13 28109027 0.009709 4.621e-09 35359 TRUE reported
textfile 2 1 TRUE
5473 rs111520336 C T C T -0.1759 0.0079 0.03387 NA FALSE FALSE FALSE
GCST006061 0.0339 0.8147 537409 outcome TRUE reported 4775_34_GSN_Gelsolin
GSN chr9 119356923 0.02293 1.703e-14 35368 TRUE reported textfile 2
1 TRUE
5474 rs139301169 A G A G -0.3613 0.0948 0.01959 NA FALSE FALSE FALSE
GCST006061 0.0459 0.039 537409 outcome TRUE reported 4775_34_GSN_Gelsolin
GSN chr9 121791334 0.029803 7.988e-34 35371 TRUE reported textfile
2 1 TRUE
5475 rs148425777 T C T C -0.2009 -0.0278 0.0248 NA FALSE FALSE FALSE
GCST006061 0.0522 0.5945 537409 outcome TRUE reported 4775_34_GSN_Gelsolin
GSN chr9 124102537 0.026776 6.233e-14 35360 TRUE reported textfile
2 1 TRUE
5476 rs16910132 A G A G -0.1426 0.0157 0.02785 NA FALSE FALSE FALSE
GCST006061 0.0191 0.4094 537409 outcome TRUE reported 4775_34_GSN_Gelsolin
GSN chr9 120776363 0.024899 1.021e-08 35385 TRUE reported textfile
2 1 TRUE
5477 rs10819316 A G A G -0.1121 0.0115 0.04464 NA FALSE FALSE FALSE
GCST006061 0.0172 0.5046 537409 outcome TRUE reported 4908_6_ENG_Endoglin ENG
chr9 127902228 0.020394 3.868e-08 35356 TRUE reported textfile 2
1 TRUE
5478 rs111888671 T G T G 0.2048 -0.0071 0.01917 NA FALSE FALSE FALSE
GCST006061 0.0483 0.8826 537409 outcome TRUE reported 4908_6_ENG_Endoglin ENG
chr9 130174704 0.030745 2.716e-11 35367 TRUE reported textfile 2
1 TRUE
5479 rs112564808 C T C T 0.1795 0.0497 0.01893 NA FALSE FALSE FALSE
GCST006061 0.0423 0.2402 537409 outcome TRUE reported 4908_6_ENG_Endoglin ENG
chr11 126177295 0.02934 9.483e-10 35368 TRUE reported textfile 2 1
TRUE
5480 rs113599685 T C T C 0.2132 0.0087 0.031 NA FALSE FALSE FALSE

GCST006061 0.0228 0.7024 537409 outcome TRUE reported 4908_6_ENG_Endoglin ENG
chr9 123280729 0.024206 1.275e-18 35362 TRUE reported textfile 2
1 TRUE
5481 rs117603193 G A G A -0.2068 -0.0554 0.01687 NA FALSE FALSE FALSE
GCST006061 0.0387 0.1516 537409 outcome TRUE reported 4908_6_ENG_Endoglin ENG
chr11 126390969 0.032316 1.56e-10 35375 TRUE reported textfile 2
1 TRUE
5482 rs12378537 T C T C 0.5027 0.0068 0.07663 NA FALSE FALSE FALSE
GCST006061 0.0125 0.5833 537409 outcome TRUE reported 4908_6_ENG_Endoglin ENG
chr9 133190139 0.015806 1e-200 35368 TRUE reported textfile 2 1
TRUE
5483 rs139562936 T G T G -0.2178 -0.0301 0.0211 NA FALSE FALSE FALSE
GCST006061 0.034 0.3765 537409 outcome TRUE reported 4908_6_ENG_Endoglin ENG
chr9 134215558 0.02947 1.463e-13 35372 TRUE reported textfile 2 1
TRUE
5484 rs149024872 T C T C 0.3417 -0.0109 0.02236 NA FALSE FALSE FALSE
GCST006061 0.0208 0.601 537409 outcome TRUE reported 4908_6_ENG_Endoglin ENG
chr9 126077700 0.028972 4.19e-32 35372 TRUE reported textfile 2
1 TRUE
5485 rs3094379 C T C T 0.4165 0.0023 0.0951 NA FALSE FALSE FALSE
GCST006061 0.0105 0.8286 537409 outcome TRUE reported 4908_6_ENG_Endoglin ENG
chr9 133469788 0.014285 6.94e-187 35369 TRUE reported textfile 2
1 TRUE
5486 rs34250484 A G A G 0.1302 0.0154 0.07182 NA FALSE FALSE FALSE
GCST006061 0.0166 0.3557 537409 outcome TRUE reported 4908_6_ENG_Endoglin ENG
chr9 133761397 0.016352 1.691e-15 35367 TRUE reported textfile 2
1 TRUE
5487 rs35166255 A G A G 0.2915 0.0222 0.0367 NA FALSE FALSE FALSE
GCST006061 0.0229 0.3329 537409 outcome TRUE reported 4908_6_ENG_Endoglin ENG
chr11 126431861 0.020858 2.199e-44 35371 TRUE reported textfile 2
1 TRUE
5488 rs4760 G A G A 0.2979 -0.0025 0.12052 NA FALSE FALSE FALSE GCST006061
0.0109 0.8165 537409 outcome TRUE reported 4908_6_ENG_Endoglin ENG chr19
43648948 0.012401 1.613e-127 35369 TRUE reported textfile 2 1 TRUE
5489 rs55714927 T C T C 0.0691 1e-04 0.21166 NA FALSE FALSE FALSE
GCST006061 0.0098 0.9926 537409 outcome TRUE reported 4908_6_ENG_Endoglin ENG
chr17 7176997 0.010004 4.947e-12 35370 TRUE reported textfile 2 1
TRUE
5490 rs6141486 A G A G -0.0543 -0.0116 0.35673 NA FALSE FALSE FALSE
GCST006061 0.0075 0.1223 537409 outcome TRUE reported 4908_6_ENG_Endoglin ENG
chr20 34590328 0.008472 1.464e-10 35366 TRUE reported textfile 2
1 TRUE
5491 rs9402686 A G A G 0.0693 -0.0044 0.27859 NA FALSE FALSE FALSE
GCST006061 0.0079 0.5774 537409 outcome TRUE reported 4908_6_ENG_Endoglin ENG
chr6 135106679 0.009065 2.091e-14 35372 TRUE reported textfile 2
1 TRUE
5492 rs10820918 G A G A 0.1481 -0.012 0.31376 NA FALSE FALSE FALSE
GCST006061 0.0075 0.1106 537409 outcome TRUE reported 7861_9_ROR2_ROR2
ROR2 chr9 91924171 0.008581 9.61e-67 35358 TRUE reported
textfile 2 1 TRUE
5493 rs34654815 C T C T -0.1143 -0.01 0.05656 NA FALSE FALSE FALSE
GCST006061 0.014 0.4764 537409 outcome TRUE reported 7861_9_ROR2_ROR2
ROR2 chr9 93014747 0.017442 5.635e-11 35356 TRUE reported
textfile 2 1 TRUE
5494 rs72704449 C T C T 0.1162 0.013 0.04582 NA FALSE FALSE FALSE
GCST006061 0.0199 0.5128 537409 outcome TRUE reported 7861_9_ROR2_ROR2
ROR2 chr1 179504138 0.018968 9.014e-10 35358 TRUE reported
textfile 2 1 TRUE
5495 rs76590235 C T C T 0.2209 -0.0123 0.02238 NA FALSE FALSE FALSE
GCST006061 0.0226 0.5876 537409 outcome TRUE reported 7861_9_ROR2_ROR2
ROR2 chr9 92202202 0.027306 5.979e-16 35359 TRUE reported
textfile 2 1 TRUE
5496 rs1147707 T C T C -0.1507 0.0034 0.37455 NA FALSE FALSE FALSE
GCST006061 0.008 0.6737 537409 outcome TRUE reported 13112_179_FSTL1_FSTL1
FSTL1 chr3 120450401 0.008488 1.602e-70 35352 TRUE reported
textfile 2 1 TRUE

5497 rs763665 T C T C 0.106 0.0096 0.18297 NA FALSE FALSE FALSE GCST006061 0.01 0.3355 537409 outcome TRUE reported 13112_179_FSTL1_FSTL1 FSTL1 chr16 72044144 0.010491 5.3e-24 35366 TRUE reported textfile 2 1 TRUE

5498 rs77542162 G A G A 0.1516 0.0177 0.025 NA FALSE FALSE FALSE GCST006061 0.0302 0.557 537409 outcome TRUE reported 13112_179_FSTL1_FSTL1 FSTL1 chr17 69085137 0.025813 4.278e-09 35362 TRUE reported textfile 2 1 TRUE

5499 rs780094 C T C T 0.0537 -0.0076 0.33917 NA FALSE FALSE FALSE GCST006061 0.0073 0.2951 537409 outcome TRUE reported 13112_179_FSTL1_FSTL1 FSTL1 chr2 27518370 0.00867 5.862e-10 35364 TRUE reported textfile 2 1 TRUE

5500 rs10821074 T C T C 0.0496 0.0063 0.46842 NA FALSE FALSE FALSE GCST006061 0.0072 0.3791 537409 outcome TRUE reported 19273_3_GSR_Glutathione_reductase GSR chr9 93133514 0.008107 9.471e-10 35357 TRUE reported textfile 2 1 TRUE

5501 rs12680844 T C T C -0.1593 0.016 0.02447 NA FALSE FALSE FALSE GCST006061 0.0148 0.2818 537409 outcome TRUE reported 19273_3_GSR_Glutathione_reductase GSR chr8 32133899 0.026328 1.443e-09 35355 TRUE reported textfile 2 1 TRUE

5502 rs4733505 G A G A 0.113 -0.0085 0.339 NA FALSE FALSE FALSE GCST006061 0.0076 0.262 537409 outcome TRUE reported 19273_3_GSR_Glutathione_reductase GSR chr8 30695913 0.008578 1.256e-39 35358 TRUE reported textfile 2 1 TRUE

5503 rs8815 G T G T 0.072 0.0125 0.36414 NA FALSE FALSE FALSE GCST006061 0.008 0.1175 537409 outcome TRUE reported 19273_3_GSR_Glutathione_reductase GSR chr12 7355130 0.008417 1.191e-17 35390 TRUE reported textfile 2 1 TRUE

5504 rs10822143 T C T C 0.0515 -0.0483 0.45345 NA FALSE FALSE FALSE GCST006061 0.0071 1.255e-11 537409 outcome TRUE reported 19639_53_IAPP_IAPP IAPP chr10 63128096 0.008124 2.313e-10 35350 TRUE reported textfile 2 1 TRUE

5505 rs11111306 A G A G -0.0447 0.0105 0.44375 NA FALSE FALSE FALSE GCST006061 0.0075 0.1608 537409 outcome TRUE reported 19639_53_IAPP_IAPP IAPP chr12 102569869 0.008122 3.722e-08 35361 TRUE reported textfile 2 1 TRUE

5506 rs116908055 T C T C -0.1359 0.0087 0.0396 NA FALSE FALSE FALSE GCST006061 0.0254 0.7324 537409 outcome TRUE reported 19639_53_IAPP_IAPP IAPP chr11 65864171 0.021259 1.631e-10 35366 TRUE reported textfile 2 1 TRUE

5507 rs117025069 G A G A -0.3798 0.0121 0.01183 NA FALSE FALSE FALSE GCST006061 0.0479 0.8012 537409 outcome TRUE reported 19639_53_IAPP_IAPP IAPP chr11 68006283 0.037136 1.498e-24 35364 TRUE reported textfile 2 1 TRUE

5508 rs1260326 C T C T -0.0804 -0.0069 0.34132 NA FALSE FALSE FALSE GCST006061 0.0073 0.3401 537409 outcome TRUE reported 19639_53_IAPP_IAPP IAPP chr2 27508073 0.008525 4.059e-21 35373 TRUE reported textfile 2 1 TRUE

5509 rs1355538 G A G A -0.0569 -0.0076 0.39847 NA FALSE FALSE FALSE GCST006061 0.0072 0.2923 537409 outcome TRUE reported 19639_53_IAPP_IAPP IAPP chr3 165787389 0.008219 4.411e-12 35338 TRUE reported textfile 2 1 TRUE

5510 rs140624338 C T C T 0.4335 0.0021 0.01047 NA FALSE FALSE FALSE GCST006061 0.0539 0.9695 537409 outcome TRUE reported 19639_53_IAPP_IAPP IAPP chr12 101963654 0.039926 1.838e-27 35362 TRUE reported textfile 2 1 TRUE

5511 rs174560 C T C T -0.0503 0.0142 0.35311 NA FALSE FALSE FALSE GCST006061 0.0076 0.06271 537409 outcome TRUE reported 19639_53_IAPP_IAPP IAPP chr11 61814292 0.008451 2.654e-09 35367 TRUE reported textfile 2 1 TRUE

5512 rs2242663 C T C T 0.2623 -9.00E-04 0.23289 NA FALSE FALSE FALSE GCST006061 0.0083 0.915 537409 outcome TRUE reported 19639_53_IAPP_IAPP IAPP chr11 66567837 0.009461 3.669e-169 35364 TRUE reported textfile 2 1 TRUE

5513 rs41280463 A G A G -0.0679 0.0238 0.18512 NA FALSE FALSE FALSE GCST006061 0.0101 0.0178 537409 outcome TRUE reported 19639_53_IAPP_IAPP

IAPP chr4 153270074 0.010371 5.87e-11 35376 TRUE reported
textfile 2 1 TRUE
5514 rs480989 G A G A 0.0993 0.0143 0.11706 NA FALSE FALSE FALSE
GCST006061 0.0104 0.1691 537409 outcome TRUE reported 19639_53_IAPP_IAPP
IAPP chr11 65726521 0.012608 3.389e-15 35366 TRUE reported
textfile 2 1 TRUE
5515 rs55707100 T C T C 0.2456 0.0383 0.02732 NA FALSE FALSE FALSE
GCST006061 0.0246 0.1192 537409 outcome TRUE reported 19639_53_IAPP_IAPP
IAPP chr15 43528519 0.024578 1.64e-23 35363 TRUE reported
textfile 2 1 TRUE
5516 rs645040 T G T G 0.0611 -0.0287 0.22729 NA FALSE FALSE FALSE
GCST006061 0.0085 0.0007847 537409 outcome TRUE reported 19639_53_IAPP_IAPP
IAPP chr3 136207780 0.009647 2.395e-10 35361 TRUE reported
textfile 2 1 TRUE
5517 rs704 A G A G -0.0761 -0.0155 0.49189 NA FALSE FALSE FALSE GCST006061
0.0072 0.03144 537409 outcome TRUE reported 19639_53_IAPP_IAPP IAPP chr17
28367840 0.008055 3.474e-21 35360 TRUE reported textfile 2 1 TRUE
5518 rs80300795 A G A G 0.1433 0.0198 0.03634 NA FALSE FALSE FALSE
GCST006061 0.0205 0.3354 537409 outcome TRUE reported 19639_53_IAPP_IAPP
IAPP chr12 101467581 0.021847 5.411e-11 35354 TRUE reported
textfile 2 1 TRUE
5519 rs825038 A C A C 0.1719 0.0218 0.05118 NA FALSE FALSE FALSE
GCST006061 0.0239 0.3624 537409 outcome TRUE reported 19639_53_IAPP_IAPP
IAPP chr12 101570303 0.018412 9.989e-21 35363 TRUE reported
textfile 2 1 TRUE
5520 rs956886 T C T C -0.0535 -0.0104 0.3139 NA FALSE FALSE FALSE
GCST006061 0.0078 0.1823 537409 outcome TRUE reported 19639_53_IAPP_IAPP
IAPP chr12 104560803 0.008752 9.781e-10 35360 TRUE reported
textfile 2 1 TRUE
5521 rs9639523 T G T G 0.0508 0.0021 0.33286 NA FALSE FALSE FALSE
GCST006061 0.0075 0.7843 537409 outcome TRUE reported 19639_53_IAPP_IAPP
IAPP chr7 25952357 0.008523 2.517e-09 35371 TRUE reported
textfile 2 1 TRUE
5522 rs10822155 A C A C 0.0437 -0.0472 0.43479 NA FALSE FALSE FALSE
GCST006061 0.0072 4.622e-11 537409 outcome TRUE reported
5340_24_CASP10_Caspase_10 CASP10 chr10 63311455 0.007989 4.496e-08 35351
TRUE reported textfile 2 1 TRUE
5523 rs1619994 G A G A 0.0656 0.0168 0.2807 NA FALSE FALSE FALSE
GCST006061 0.0078 0.03057 537409 outcome TRUE reported
5340_24_CASP10_Caspase_10 CASP10 chr3 165744047 0.008827 1.073e-13 35339
TRUE reported textfile 2 1 TRUE
5524 rs66489920 C T C T -0.0607 0.0189 0.25071 NA FALSE FALSE FALSE
GCST006061 0.0078 0.01607 537409 outcome TRUE reported
5340_24_CASP10_Caspase_10 CASP10 chr8 105580311 0.009171 3.625e-11 35362
TRUE reported textfile 2 1 TRUE
5525 rs10822155 A C A C 0.0843 -0.0472 0.43479 NA FALSE FALSE FALSE
GCST006061 0.0072 4.622e-11 537409 outcome TRUE reported
9345_436_WFDC13_WFD13 WFDC13 chr10 63311455 0.008183 6.932e-25 35351
TRUE reported textfile 2 1 TRUE
5526 rs1398015 T C T C -0.1133 0.0026 0.05914 NA FALSE FALSE FALSE
GCST006061 0.0215 0.9043 537409 outcome TRUE reported 9345_436_WFDC13_WFD13
WFDC13 chr4 73891519 0.01739 7.26e-11 35349 TRUE reported textfile
2 1 TRUE
5527 rs16850360 G A G A -0.1765 0.0094 0.02454 NA FALSE FALSE FALSE
GCST006061 0.0182 0.6046 537409 outcome TRUE reported 9345_436_WFDC13_WFD13
WFDC13 chr4 74006728 0.027087 7.222e-11 35351 TRUE reported
textfile 2 1 TRUE
5528 rs10822155 A C A C 0.0512 -0.0472 0.43479 NA FALSE FALSE FALSE
GCST006061 0.0072 4.622e-11 537409 outcome TRUE reported 18160_2_PCMT1_PIMT
PCMT1 chr10 63311455 0.008017 1.7e-10 35351 TRUE reported textfile
2 1 TRUE
5529 rs11574452 A C A C -0.148 0.0147 0.02727 NA FALSE FALSE FALSE
GCST006061 0.0173 0.3972 537409 outcome TRUE reported 18160_2_PCMT1_PIMT
PCMT1 chr4 73980944 0.025062 3.519e-09 35351 TRUE reported
textfile 2 1 TRUE
5530 rs572515 G A G A -0.0501 -0.0113 0.3973 NA FALSE FALSE FALSE

GCST006061 0.0075 0.1355 537409 outcome TRUE reported 18160_2_PCMT1_PIMT
PCMT1 chr1 196677131 0.008141 7.543e-10 35338 TRUE reported
textfile 2 1 TRUE
5531 rs10822155 A C A C 0.0603 -0.0472 0.43479 NA FALSE FALSE FALSE
GCST006061 0.0072 4.622e-11 537409 outcome TRUE reported 12392_30_ARL1_ARL1
ARL1 chr10 63311455 0.008126 1.166e-13 35342 TRUE reported
textfile 2 1 TRUE
5532 rs1706435 G A G A 0.0541 0.0117 0.36981 NA FALSE FALSE FALSE
GCST006061 0.0072 0.1047 537409 outcome TRUE reported 12392_30_ARL1_ARL1
ARL1 chr3 165743688 0.008344 8.953e-11 35330 TRUE reported
textfile 2 1 TRUE
5533 rs429358 C T C T -0.1812 0.005 0.17407 NA FALSE FALSE FALSE
GCST006061 0.0103 0.6319 537409 outcome TRUE reported 12392_30_ARL1_ARL1
ARL1 chr19 44908684 0.010721 4.442e-64 35359 TRUE reported
textfile 2 1 TRUE
5534 rs10822155 A C A C 0.0478 -0.0472 0.43479 NA FALSE FALSE FALSE
GCST006061 0.0072 4.622e-11 537409 outcome TRUE reported 8778_3_NOG_Noggin
NOG chr10 63311455 0.008423 1.385e-08 35351 TRUE reported textfile
2 1 TRUE
5535 rs11187019 G A G A -0.051 -0.009 0.43932 NA FALSE FALSE FALSE
GCST006061 0.0074 0.2217 537409 outcome TRUE reported 8778_3_NOG_Noggin NOG
chr10 92488553 0.008439 1.512e-09 35373 TRUE reported textfile 2
1 TRUE
5536 rs114694170 C T C T 0.2838 0.0053 0.08378 NA FALSE FALSE FALSE
GCST006061 0.0175 0.7624 537409 outcome TRUE reported 8778_3_NOG_Noggin NOG
chr5 88884379 0.015117 1.256e-78 35344 TRUE reported textfile 2
1 TRUE
5537 rs114968084 A G A G -0.1623 -0.0043 0.03399 NA FALSE FALSE FALSE
GCST006061 0.0281 0.8795 537409 outcome TRUE reported 8778_3_NOG_Noggin NOG
chr9 133147567 0.023196 2.617e-12 35367 TRUE reported textfile 2
1 TRUE
5538 rs35979828 T C T C -0.1624 -0.0044 0.08648 NA FALSE FALSE FALSE
GCST006061 0.0156 0.7763 537409 outcome TRUE reported 8778_3_NOG_Noggin NOG
chr12 54292096 0.015048 3.738e-27 35375 TRUE reported textfile 2
1 TRUE
5539 rs4141420 A C A C -0.0947 -0.0035 0.07326 NA FALSE FALSE FALSE
GCST006061 0.0109 0.7483 537409 outcome TRUE reported 8778_3_NOG_Noggin NOG
chr1 54385891 0.015806 2.082e-09 35363 TRUE reported textfile 2
1 TRUE
5540 rs62428833 T C T C -0.0683 3e-04 0.21442 NA FALSE FALSE FALSE
GCST006061 0.0092 0.9775 537409 outcome TRUE reported 8778_3_NOG_Noggin NOG
chr6 142942133 0.010216 2.295e-11 35350 TRUE reported textfile 2
1 TRUE
5541 rs71387447 T C T C 0.165 -0.0192 0.02613 NA FALSE FALSE FALSE
GCST006061 0.0231 0.4063 537409 outcome TRUE reported 8778_3_NOG_Noggin NOG
chr17 56252438 0.02661 5.619e-10 35372 TRUE reported textfile 2 1
TRUE
5542 rs73000929 A G A G 0.1667 0.0131 0.04815 NA FALSE FALSE FALSE
GCST006061 0.0245 0.593 537409 outcome TRUE reported 8778_3_NOG_Noggin NOG
chr11 114082900 0.019458 1.061e-17 35365 TRUE reported textfile 2
1 TRUE
5543 rs7475335 A C A C 0.1539 0.0058 0.27899 NA FALSE FALSE FALSE
GCST006061 0.0078 0.4553 537409 outcome TRUE reported 8778_3_NOG_Noggin NOG
chr10 102583671 0.009366 1.122e-60 35370 TRUE reported textfile 2
1 TRUE
5544 rs76164057 G A G A 1.2598 0.0409 0.01406 NA FALSE FALSE FALSE
GCST006061 0.0476 0.39 537409 outcome TRUE reported 8778_3_NOG_Noggin NOG
chr17 56763503 0.03543 1e-200 35365 TRUE reported textfile 2 1 TRUE
5545 rs7622284 C T C T 0.0819 -0.0084 0.11107 NA FALSE FALSE FALSE
GCST006061 0.0107 0.4359 537409 outcome TRUE reported 8778_3_NOG_Noggin NOG
chr3 39199068 0.013294 7.249e-10 35355 TRUE reported textfile 2
1 TRUE
5546 rs78909033 A G A G -0.12 -0.0167 0.16064 NA FALSE FALSE FALSE
GCST006061 0.0117 0.1526 537409 outcome TRUE reported 8778_3_NOG_Noggin NOG
chr2 240571486 0.01139 5.941e-26 35270 TRUE reported textfile 2 1
TRUE

5547 rs10822155 A C A C 0.1426 -0.0472 0.43479 NA FALSE FALSE FALSE
GCST006061 0.0072 4.622e-11 537409 outcome TRUE reported
14094_29_HBEGF_HB_EGF HBEGF chr10 63311455 0.008367 3.953e-65 35351
TRUE reported textfile 2 1 TRUE
5548 rs11553699 G A G A 0.1687 -0.0209 0.14277 NA FALSE FALSE FALSE
GCST006061 0.012 0.08291 537409 outcome TRUE reported 14094_29_HBEGF_HB_EGF
HBEGF chr12 121779004 0.011886 1.012e-45 35366 TRUE reported
textfile 2 1 TRUE
5549 rs116574109 A G A G 0.1887 0.0475 0.01685 NA FALSE FALSE FALSE
GCST006061 0.0512 0.3534 537409 outcome TRUE reported 14094_29_HBEGF_HB_EGF
HBEGF chr6 26575558 0.032929 1.001e-08 35377 TRUE reported
textfile 2 1 TRUE
5550 rs13207171 T C T C 0.0817 0.003 0.14938 NA FALSE FALSE FALSE
GCST006061 0.0112 0.7869 537409 outcome TRUE reported 14094_29_HBEGF_HB_EGF
HBEGF chr6 41198081 0.011578 1.709e-12 35371 TRUE reported
textfile 2 1 TRUE
5551 rs1354034 C T C T -0.0805 0.0088 0.38119 NA FALSE FALSE FALSE
GCST006061 0.0073 0.2248 537409 outcome TRUE reported 14094_29_HBEGF_HB_EGF
HBEGF chr3 56815721 0.008562 5.361e-21 35356 TRUE reported
textfile 2 1 TRUE
5552 rs2073775 C T C T -0.0524 -0.0018 0.38698 NA FALSE FALSE FALSE
GCST006061 0.0073 0.8008 537409 outcome TRUE reported 14094_29_HBEGF_HB_EGF
HBEGF chr22 19040963 0.00857 9.677e-10 35346 TRUE reported textfile
2 1 TRUE
5553 rs269767 G A G A -0.0502 -0.0137 0.43885 NA FALSE FALSE FALSE
GCST006061 0.0073 0.05932 537409 outcome TRUE reported 14094_29_HBEGF_HB_EGF
HBEGF chr5 140157337 0.008416 2.448e-09 35362 TRUE reported
textfile 2 1 TRUE
5554 rs3811444 T C T C -0.073 0.0103 0.34232 NA FALSE FALSE FALSE
GCST006061 0.0077 0.1812 537409 outcome TRUE reported 14094_29_HBEGF_HB_EGF
HBEGF chr1 247876149 0.008835 1.428e-16 35156 TRUE reported
textfile 2 1 TRUE
5555 rs6479631 T C T C -0.0712 -0.01 0.28404 NA FALSE FALSE FALSE
GCST006061 0.008 0.2146 537409 outcome TRUE reported 14094_29_HBEGF_HB_EGF
HBEGF chr9 134017699 0.009263 1.511e-14 35366 TRUE reported
textfile 2 1 TRUE
5556 rs77906229 T C T C 0.0749 -0.0141 0.12172 NA FALSE FALSE FALSE
GCST006061 0.0128 0.2707 537409 outcome TRUE reported 14094_29_HBEGF_HB_EGF
HBEGF chr12 64672764 0.012744 4.17e-09 35368 TRUE reported
textfile 2 1 TRUE
5557 rs10822155 A C A C 0.0558 -0.0472 0.43479 NA FALSE FALSE FALSE
GCST006061 0.0072 4.622e-11 537409 outcome TRUE reported 8269_327_ARSK_ARSK
ARSK chr10 63311455 0.008175 8.775e-12 35351 TRUE reported
textfile 2 1 TRUE
5558 rs10922098 T C T C -0.0728 -0.009 0.40011 NA FALSE FALSE FALSE
GCST006061 0.0075 0.2275 537409 outcome TRUE reported 8269_327_ARSK_ARSK
ARSK chr1 196695521 0.008285 1.536e-18 35338 TRUE reported
textfile 2 1 TRUE
5559 rs114300458 C T C T -0.1962 0.048 0.01465 NA FALSE FALSE FALSE
GCST006061 0.0354 0.1754 537409 outcome TRUE reported 8269_327_ARSK_ARSK
ARSK chr5 91213844 0.034116 8.876e-09 35340 TRUE reported
textfile 2 1 TRUE
5560 rs12813762 C A C A 0.1127 0.0012 0.39661 NA FALSE FALSE FALSE
GCST006061 0.0075 0.8705 537409 outcome TRUE reported 8269_327_ARSK_ARSK
ARSK chr12 8009775 0.008353 1.749e-41 35378 TRUE reported textfile
2 1 TRUE
5561 rs1354034 C T C T -0.0626 0.0088 0.38119 NA FALSE FALSE FALSE
GCST006061 0.0073 0.2248 537409 outcome TRUE reported 8269_327_ARSK_ARSK
ARSK chr3 56815721 0.008346 6.367e-14 35356 TRUE reported
textfile 2 1 TRUE
5562 rs1355538 G A G A -0.051 -0.0076 0.39847 NA FALSE FALSE FALSE
GCST006061 0.0072 0.2923 537409 outcome TRUE reported 8269_327_ARSK_ARSK
ARSK chr3 165787389 0.008278 7.247e-10 35338 TRUE reported
textfile 2 1 TRUE
5563 rs139102439 C T C T -0.2177 0.1141 0.01222 NA FALSE FALSE FALSE
GCST006061 0.0587 0.05212 537409 outcome TRUE reported 8269_327_ARSK_ARSK

ARSK chr5 97644336 0.036721 3.056e-09 35344 TRUE reported
textfile 2 1 TRUE
5564 rs140624338 C T C T 0.2451 0.0021 0.01047 NA FALSE FALSE FALSE
GCST006061 0.0539 0.9695 537409 outcome TRUE reported 8269_327_ARSK_ARSK
ARSK chr12 101963654 0.039958 8.574e-10 35362 TRUE reported
textfile 2 1 TRUE
5565 rs34762726 A G A G -0.052 -0.0047 0.30713 NA FALSE FALSE FALSE
GCST006061 0.008 0.5543 537409 outcome TRUE reported 8269_327_ARSK_ARSK
ARSK chr3 49651777 0.008779 3.155e-09 35343 TRUE reported
textfile 2 1 TRUE
5566 rs4883201 G A G A 0.0811 -0.0182 0.12502 NA FALSE FALSE FALSE
GCST006061 0.0114 0.1094 537409 outcome TRUE reported 8269_327_ARSK_ARSK
ARSK chr12 8929985 0.012285 4.074e-11 35387 TRUE reported textfile
2 1 TRUE
5567 rs10822155 A C A C 0.0643 -0.0472 0.43479 NA FALSE FALSE FALSE
GCST006061 0.0072 4.622e-11 537409 outcome TRUE reported
5001_6_SCGB2A1_Mammaglobin_2 SCGB2A1 chr10 63311455 0.008106 2.152e-15
35351 TRUE reported textfile 2 1 TRUE
5568 rs1706435 G A G A 0.0576 0.0117 0.36981 NA FALSE FALSE FALSE
GCST006061 0.0072 0.1047 537409 outcome TRUE reported
5001_6_SCGB2A1_Mammaglobin_2 SCGB2A1 chr3 165743688 0.008318 4.367e-12
35339 TRUE reported textfile 2 1 TRUE
5569 rs10822155 A C A C 0.1105 -0.0472 0.43479 NA FALSE FALSE FALSE
GCST006061 0.0072 4.622e-11 537409 outcome TRUE reported 15678_71_DKK2_DKK2
DKK2 chr10 63311455 0.008349 5.484e-40 35351 TRUE reported
textfile 2 1 TRUE
5570 rs10888208 G T G T 0.4573 0.0632 0.01397 NA FALSE FALSE FALSE
GCST006061 0.0409 0.1227 537409 outcome TRUE reported 15678_71_DKK2_DKK2
DKK2 chr10 47096668 0.035169 1.175e-38 35333 TRUE reported
textfile 2 1 TRUE
5571 rs117565303 T C T C 0.1697 -0.0061 0.03105 NA FALSE FALSE FALSE
GCST006061 0.0281 0.8278 537409 outcome TRUE reported 15678_71_DKK2_DKK2
DKK2 chr10 55217204 0.023664 7.432e-13 35358 TRUE reported
textfile 2 1 TRUE
5572 rs141099634 C T C T 0.6858 -0.1632 0.0118 NA FALSE FALSE FALSE
GCST006061 0.1635 0.3183 537409 outcome TRUE reported 15678_71_DKK2_DKK2
DKK2 chr10 50347786 0.038014 9.368e-73 35353 TRUE reported
textfile 2 1 TRUE
5573 rs145591269 G A G A 0.1856 0.0025 0.01489 NA FALSE FALSE FALSE
GCST006061 0.0441 0.9541 537409 outcome TRUE reported 15678_71_DKK2_DKK2
DKK2 chr10 61660551 0.033792 3.965e-08 35354 TRUE reported
textfile 2 1 TRUE
5574 rs150513777 G A G A 0.4518 -0.0025 0.0321 NA FALSE FALSE FALSE
GCST006061 0.0255 0.9229 537409 outcome TRUE reported 15678_71_DKK2_DKK2
DKK2 chr10 52474587 0.02323 2.95e-84 35355 TRUE reported textfile
2 1 TRUE
5575 rs34416903 C T C T 0.0512 -0.001 0.30061 NA FALSE FALSE FALSE
GCST006061 0.0084 0.9015 537409 outcome TRUE reported 15678_71_DKK2_DKK2
DKK2 chr16 88492837 0.008935 1.003e-08 35318 TRUE reported
textfile 2 1 TRUE
5576 rs4648825 C T C T 0.0575 0.0069 0.19892 NA FALSE FALSE FALSE
GCST006061 0.0099 0.4849 537409 outcome TRUE reported 15678_71_DKK2_DKK2
DKK2 chr1 2304900 0.010387 3.103e-08 35328 TRUE reported textfile
2 1 TRUE
5577 rs7101062 G A G A -0.2734 -0.036 0.03623 NA FALSE FALSE FALSE
GCST006061 0.0364 0.3234 537409 outcome TRUE reported 15678_71_DKK2_DKK2
DKK2 chr10 51837325 0.021951 1.311e-35 35358 TRUE reported
textfile 2 1 TRUE
5578 rs75580312 A G A G 0.7185 0.0425 0.01464 NA FALSE FALSE FALSE
GCST006061 0.0478 0.3745 537409 outcome TRUE reported 15678_71_DKK2_DKK2
DKK2 chr10 51875766 0.033058 9.629e-105 35362 TRUE reported
textfile 2 1 TRUE
5579 rs10822155 A C A C 0.05 -0.0472 0.43479 NA FALSE FALSE FALSE
GCST006061 0.0072 4.622e-11 537409 outcome TRUE reported
4184_43_EIF4EBP2_4EBP2 EIF4EBP2 chr10 63311455 0.008024 4.63e-10 35351
TRUE reported textfile 2 1 TRUE

5580 rs10822155 A C A C 0.0463 -0.0472 0.43479 NA FALSE FALSE FALSE GCST006061 0.0072 4.622e-11 537409 outcome TRUE reported 15674_3_CD79A_CD79A CD79A chr10 63311455 0.008012 7.509e-09 35351 TRUE reported textfile 2 1 TRUE

5581 rs4734879 G A G A -0.0573 0.0194 0.21866 NA FALSE FALSE FALSE GCST006061 0.0079 0.01412 537409 outcome TRUE reported 15674_3_CD79A_CD79A CD79A chr8 105570896 0.009638 2.764e-09 35365 TRUE reported textfile 2 1 TRUE

5582 rs572515 G A G A -0.049 -0.0113 0.3973 NA FALSE FALSE FALSE GCST006061 0.0075 0.1355 537409 outcome TRUE reported 15674_3_CD79A_CD79A CD79A chr1 196677131 0.00812 1.596e-09 35338 TRUE reported textfile 2 1 TRUE

5583 rs10822155 A C A C 0.0998 -0.0472 0.43479 NA FALSE FALSE FALSE GCST006061 0.0072 4.622e-11 537409 outcome TRUE reported 16317_20_DSG3_Desmoglein_3 DSG3 chr10 63311455 0.008169 2.515e-34 35351 TRUE reported textfile 2 1 TRUE

5584 rs6780053 G A G A 0.0506 0.0106 0.26289 NA FALSE FALSE FALSE GCST006061 0.008 0.1829 537409 outcome TRUE reported 16317_20_DSG3_Desmoglein_3 DSG3 chr3 151149276 0.009251 4.511e-08 35348 TRUE reported textfile 2 1 TRUE

5585 rs10822155 A C A C 0.045 -0.0472 0.43479 NA FALSE FALSE FALSE GCST006061 0.0072 4.622e-11 537409 outcome TRUE reported 18213_30_UBE2F_UBE2F UBE2F chr10 63311455 0.008035 2.139e-08 35351 TRUE reported textfile 2 1 TRUE

5586 rs1354034 C T C T 0.0646 0.0088 0.38119 NA FALSE FALSE FALSE GCST006061 0.0073 0.2248 537409 outcome TRUE reported 18213_30_UBE2F_UBE2F UBE2F chr3 56815721 0.008207 3.517e-15 35356 TRUE reported textfile 2 1 TRUE

5587 rs1619994 G A G A 0.0545 0.0168 0.2807 NA FALSE FALSE FALSE GCST006061 0.0078 0.03057 537409 outcome TRUE reported 18213_30_UBE2F_UBE2F UBE2F chr3 165744047 0.008886 8.599e-10 35339 TRUE reported textfile 2 1 TRUE

5588 rs4980320 C T C T 0.0451 0.0041 0.46986 NA FALSE FALSE FALSE GCST006061 0.0076 0.5858 537409 outcome TRUE reported 18213_30_UBE2F_UBE2F UBE2F chr11 198986 0.008006 1.769e-08 35352 TRUE reported textfile 2 1 TRUE

5589 rs61751507 T C T C 0.2438 0.0047 0.04162 NA FALSE FALSE FALSE GCST006061 0.018 0.794 537409 outcome TRUE reported 18213_30_UBE2F_UBE2F UBE2F chr10 100069757 0.019852 1.145e-34 35368 TRUE reported textfile 2 1 TRUE

5590 rs704 A G A G 0.0448 -0.0155 0.49189 NA FALSE FALSE FALSE GCST006061 0.0072 0.03144 537409 outcome TRUE reported 18213_30_UBE2F_UBE2F UBE2F chr17 28367840 0.007995 2.1e-08 35360 TRUE reported textfile 2 1 TRUE

5591 rs10822155 A C A C 0.077 -0.0472 0.43479 NA FALSE FALSE FALSE GCST006061 0.0072 4.622e-11 537409 outcome TRUE reported 4499_21_PDGFA_PDGF_AA PDGFA chr10 63311455 0.008303 1.8e-20 35351 TRUE reported textfile 2 1 TRUE

5592 rs12468189 G A G A 0.0476 -0.0052 0.40103 NA FALSE FALSE FALSE GCST006061 0.0074 0.4799 537409 outcome TRUE reported 4499_21_PDGFA_PDGF_AA PDGFA chr2 31255954 0.008443 1.722e-08 35369 TRUE reported textfile 2 1 TRUE

5593 rs145793648 G A G A -0.1502 -0.0116 0.02697 NA FALSE FALSE FALSE GCST006061 0.0428 0.7866 537409 outcome TRUE reported 4499_21_PDGFA_PDGF_AA PDGFA chr7 589908 0.026577 1.59e-08 35323 TRUE reported textfile 2 1 TRUE

5594 rs3811444 T C T C -0.0727 0.0103 0.34232 NA FALSE FALSE FALSE GCST006061 0.0077 0.1812 537409 outcome TRUE reported 4499_21_PDGFA_PDGF_AA PDGFA chr1 247876149 0.00875 9.671e-17 35156 TRUE reported textfile 2 1 TRUE

5595 rs10822155 A C A C 0.1043 -0.0472 0.43479 NA FALSE FALSE FALSE GCST006061 0.0072 4.622e-11 537409 outcome TRUE reported 3038_9_CXCL11_I_TAC CXCL11 chr10 63311455 0.008041 1.783e-38 35351 TRUE reported textfile 2 1 TRUE

5596 rs111425801 A G A G -0.1363 0.032 0.03319 NA FALSE FALSE FALSE GCST006061 0.0223 0.1516 537409 outcome TRUE reported 3038_9_CXCL11_I_TAC

CXCL11 chr9 133094804 0.022346 1.064e-09 35372 TRUE reported
textfile 2 1 TRUE
5597 rs114694170 C T C T -0.0999 0.0053 0.08378 NA FALSE FALSE FALSE
GCST006061 0.0175 0.7624 537409 outcome TRUE reported 3038_9_CXCL11_I_TAC
CXCL11 chr5 88884379 0.01448 5.23e-12 35344 TRUE reported textfile
2 1 TRUE
5598 rs117150187 G A G A -0.1357 0.0354 0.02674 NA FALSE FALSE FALSE
GCST006061 0.03 0.2389 537409 outcome TRUE reported 3038_9_CXCL11_I_TAC
CXCL11 chr9 133786984 0.023938 1.438e-08 35371 TRUE reported
textfile 2 1 TRUE
5599 rs11729174 T C T C -0.0571 -0.0113 0.24336 NA FALSE FALSE FALSE
GCST006061 0.0089 0.2027 537409 outcome TRUE reported 3038_9_CXCL11_I_TAC
CXCL11 chr4 75626583 0.009394 1.216e-09 35354 TRUE reported
textfile 2 1 TRUE
5600 rs12075 A G A G -0.0716 5e-04 0.42429 NA FALSE FALSE FALSE GCST006061
0.0074 0.9502 537409 outcome TRUE reported 3038_9_CXCL11_I_TAC CXCL11 chr1
159205564 0.008093 8.993e-19 35358 TRUE reported textfile 2 1 TRUE
5601 rs12767683 A C A C -0.1412 0.0038 0.26443 NA FALSE FALSE FALSE
GCST006061 0.0079 0.6284 537409 outcome TRUE reported 3038_9_CXCL11_I_TAC
CXCL11 chr10 102556824 0.009086 1.847e-54 35370 TRUE reported
textfile 2 1 TRUE
5602 rs3184504 C T C T -0.1011 0.0074 0.37862 NA FALSE FALSE FALSE
GCST006061 0.0075 0.3235 537409 outcome TRUE reported 3038_9_CXCL11_I_TAC
CXCL11 chr12 111446804 0.008232 1.145e-34 35371 TRUE reported
textfile 2 1 TRUE
5603 rs342287 C T C T 0.0506 -0.0103 0.31982 NA FALSE FALSE FALSE
GCST006061 0.008 0.1978 537409 outcome TRUE reported 3038_9_CXCL11_I_TAC
CXCL11 chr7 106724921 0.00856 3.397e-09 35359 TRUE reported textfile
2 1 TRUE
5604 rs35140383 C T C T -0.254 0.0133 0.01155 NA FALSE FALSE FALSE
GCST006061 0.0296 0.6519 537409 outcome TRUE reported 3038_9_CXCL11_I_TAC
CXCL11 chr9 132646885 0.037879 2.006e-11 35373 TRUE reported
textfile 2 1 TRUE
5605 rs4783187 C T C T 0.094 -0.0124 0.12255 NA FALSE FALSE FALSE
GCST006061 0.0108 0.2492 537409 outcome TRUE reported 3038_9_CXCL11_I_TAC
CXCL11 chr16 85382232 0.012148 1.012e-14 35368 TRUE reported
textfile 2 1 TRUE
5606 rs595585 T C T C 0.054 -0.0076 0.39833 NA FALSE FALSE FALSE
GCST006061 0.0073 0.2965 537409 outcome TRUE reported 3038_9_CXCL11_I_TAC
CXCL11 chr11 128734115 0.00813 3.1e-11 35359 TRUE reported textfile 2
1 TRUE
5607 rs6580981 A G A G -0.0521 4e-04 0.47567 NA FALSE FALSE FALSE
GCST006061 0.0072 0.9564 537409 outcome TRUE reported 3038_9_CXCL11_I_TAC
CXCL11 chr12 54329244 0.007999 7.371e-11 35372 TRUE reported
textfile 2 1 TRUE
5608 rs6822200 C T C T -0.0881 0.0059 0.13781 NA FALSE FALSE FALSE
GCST006061 0.0093 0.5264 537409 outcome TRUE reported 3038_9_CXCL11_I_TAC
CXCL11 chr4 76427904 0.011652 4.002e-14 35357 TRUE reported
textfile 2 1 TRUE
5609 rs10822155 A C A C 0.0445 -0.0472 0.43479 NA FALSE FALSE FALSE
GCST006061 0.0072 4.622e-11 537409 outcome TRUE reported
12704_26_SH2D3C_SH2D3 SH2D3C chr10 63311455 0.008128 4.371e-08 35342
TRUE reported textfile 2 1 TRUE
5610 rs1706435 G A G A 0.0842 0.0117 0.36981 NA FALSE FALSE FALSE
GCST006061 0.0072 0.1047 537409 outcome TRUE reported 12704_26_SH2D3C_SH2D3
SH2D3C chr3 165743688 0.008338 5.609e-24 35330 TRUE reported
textfile 2 1 TRUE
5611 rs438811 T C T C -0.1085 -5.00E-04 0.23201 NA FALSE FALSE FALSE
GCST006061 0.0088 0.9556 537409 outcome TRUE reported 12704_26_SH2D3C_SH2D3
SH2D3C chr19 44913484 0.009622 1.716e-29 35357 TRUE reported
textfile 2 1 TRUE
5612 rs704 A G A G 0.0474 -0.0155 0.49189 NA FALSE FALSE FALSE GCST006061
0.0072 0.03144 537409 outcome TRUE reported 12704_26_SH2D3C_SH2D3 SH2D3C
chr17 28367840 0.00807 4.269e-09 35351 TRUE reported textfile 2 1
TRUE
5613 rs10822155 A C A C 0.0518 -0.0472 0.43479 NA FALSE FALSE FALSE

GCST006061 0.0072 4.622e-11 537409 outcome TRUE reported 11649_3_SMAP1_SMAP1
SMAP1 chr10 63311455 0.008124 1.819e-10 35342 TRUE reported
textfile 2 1 TRUE
5614 rs10944798 A G A G -0.0498 0.0039 0.30173 NA FALSE FALSE FALSE
GCST006061 0.0079 0.6229 537409 outcome TRUE reported 11649_3_SMAP1_SMAP1
SMAP1 chr6 65423849 0.00888 2.048e-08 35328 TRUE reported textfile
2 1 TRUE
5615 rs12203753 T C T C 0.1906 -9.00E-04 0.03938 NA FALSE FALSE FALSE
GCST006061 0.0174 0.9585 537409 outcome TRUE reported 11649_3_SMAP1_SMAP1
SMAP1 chr6 71221481 0.021176 2.246e-19 35323 TRUE reported
textfile 2 1 TRUE
5616 rs1354034 C T C T 0.0937 0.0088 0.38119 NA FALSE FALSE FALSE
GCST006061 0.0073 0.2248 537409 outcome TRUE reported 11649_3_SMAP1_SMAP1
SMAP1 chr3 56815721 0.008288 1.232e-29 35347 TRUE reported
textfile 2 1 TRUE
5617 rs138174879 A G A G 0.1181 -0.0139 0.04852 NA FALSE FALSE FALSE
GCST006061 0.0237 0.5565 537409 outcome TRUE reported 11649_3_SMAP1_SMAP1
SMAP1 chr6 56300137 0.019011 5.226e-10 35340 TRUE reported
textfile 2 1 TRUE
5618 rs147236212 G A G A 0.2081 -0.0322 0.04201 NA FALSE FALSE FALSE
GCST006061 0.035 0.3566 537409 outcome TRUE reported 11649_3_SMAP1_SMAP1
SMAP1 chr6 70944004 0.020295 1.137e-24 35324 TRUE reported
textfile 2 1 TRUE
5619 rs2691477 A G A G -0.312 0.0024 0.2473 NA FALSE FALSE FALSE
GCST006061 0.008 0.7645 537409 outcome TRUE reported 11649_3_SMAP1_SMAP1
SMAP1 chr6 70618140 0.009388 1e-200 35324 TRUE reported textfile
2 1 TRUE
5620 rs4980320 C T C T 0.0612 0.0041 0.46986 NA FALSE FALSE FALSE
GCST006061 0.0076 0.5858 537409 outcome TRUE reported 11649_3_SMAP1_SMAP1
SMAP1 chr11 198986 0.008088 3.819e-14 35343 TRUE reported textfile
2 1 TRUE
5621 rs6454314 A G A G 0.0506 -0.0025 0.31959 NA FALSE FALSE FALSE
GCST006061 0.0078 0.749 537409 outcome TRUE reported 11649_3_SMAP1_SMAP1
SMAP1 chr6 83030681 0.008714 6.371e-09 35326 TRUE reported
textfile 2 1 TRUE
5622 rs10822155 A C A C 0.0584 -0.0472 0.43479 NA FALSE FALSE FALSE
GCST006061 0.0072 4.622e-11 537409 outcome TRUE reported
13434_172_PARVA_PARVA PARVA chr10 63311455 0.008251 1.461e-12 35345
TRUE reported textfile 2 1 TRUE
5623 rs10822155 A C A C 0.0943 -0.0472 0.43479 NA FALSE FALSE FALSE
GCST006061 0.0072 4.622e-11 537409 outcome TRUE reported
5335_73_ANXA6_annexin_VI ANXA6 chr10 63311455 0.008239 2.473e-30 35351
TRUE reported textfile 2 1 TRUE
5624 rs11574452 A C A C -0.2139 0.0147 0.02727 NA FALSE FALSE FALSE
GCST006061 0.0173 0.3972 537409 outcome TRUE reported
5335_73_ANXA6_annexin_VI ANXA6 chr4 73980944 0.025738 9.523e-17 35351
TRUE reported textfile 2 1 TRUE
5625 rs1398015 T C T C -0.1324 0.0026 0.05914 NA FALSE FALSE FALSE
GCST006061 0.0215 0.9043 537409 outcome TRUE reported
5335_73_ANXA6_annexin_VI ANXA6 chr4 73891519 0.017501 3.873e-14 35349
TRUE reported textfile 2 1 TRUE
5626 rs10822155 A C A C 0.0722 -0.0472 0.43479 NA FALSE FALSE FALSE
GCST006061 0.0072 4.622e-11 537409 outcome TRUE reported
4149_8_PDGFB_PDGF_BB PDGFB chr10 63311455 0.008228 1.717e-18 35351
TRUE reported textfile 2 1 TRUE
5627 rs4965869 T C T C 0.0621 0.0139 0.23581 NA FALSE FALSE FALSE
GCST006061 0.008 0.08436 537409 outcome TRUE reported 4149_8_PDGFB_PDGF_BB
PDGFB chr15 101450115 0.00966 1.289e-10 35307 TRUE reported textfile
2 1 TRUE
5628 rs68066031 C T C T 0.1044 -0.0076 0.19513 NA FALSE FALSE FALSE
GCST006061 0.0091 0.4038 537409 outcome TRUE reported 4149_8_PDGFB_PDGF_BB
PDGFB chr2 224015781 0.010299 3.78e-24 35366 TRUE reported
textfile 2 1 TRUE
5629 rs10822155 A C A C 0.0472 -0.0472 0.43479 NA FALSE FALSE FALSE
GCST006061 0.0072 4.622e-11 537409 outcome TRUE reported
9196_8_LGALS7_Galectin_7 LGALS7 chr10 63311455 0.008015 3.88e-09 35351

TRUE reported textfile 2 1 TRUE
5630 rs1619994 G A G A 0.0548 0.0168 0.2807 NA FALSE FALSE FALSE
GCST006061 0.0078 0.03057 537409 outcome TRUE reported
9196_8_LGALS7_Galectin_7 LGALS7 chr3 165744047 0.008864 6.318e-10 35339
TRUE reported textfile 2 1 TRUE
5631 rs34762726 A G A G -0.0502 -0.0047 0.30713 NA FALSE FALSE FALSE
GCST006061 0.008 0.5543 537409 outcome TRUE reported
9196_8_LGALS7_Galectin_7 LGALS7 chr3 49651777 0.008606 5.441e-09 35343
TRUE reported textfile 2 1 TRUE
5632 rs533617 C T C T 0.13 -0.0316 0.04482 NA FALSE FALSE FALSE
GCST006061 0.0198 0.1103 537409 outcome TRUE reported
9196_8_LGALS7_Galectin_7 LGALS7 chr2 21011100 0.018885 5.833e-12 35373
TRUE reported textfile 2 1 TRUE
5633 rs10822155 A C A C 0.0517 -0.0472 0.43479 NA FALSE FALSE FALSE
GCST006061 0.0072 4.622e-11 537409 outcome TRUE reported 18921_30_PHEX_PHEX
PHEX chr10 63311455 0.00806 1.412e-10 35351 TRUE reported textfile
2 1 TRUE
5634 rs7187856 A G A G -0.0906 -0.0041 0.30703 NA FALSE FALSE FALSE
GCST006061 0.008 0.6118 537409 outcome TRUE reported 18921_30_PHEX_PHEX
PHEX chr16 78193642 0.008649 1.122e-25 35369 TRUE reported
textfile 2 1 TRUE
5635 rs9332701 G A G A -0.0844 -0.0115 0.07189 NA FALSE FALSE FALSE
GCST006061 0.0196 0.5575 537409 outcome TRUE reported 18921_30_PHEX_PHEX
PHEX chr1 169515529 0.015371 3.998e-08 35364 TRUE reported
textfile 2 1 TRUE
5636 rs10822155 A C A C 0.0643 -0.0472 0.43479 NA FALSE FALSE FALSE
GCST006061 0.0072 4.622e-11 537409 outcome TRUE reported
11370_20_INPP5E_INP5E INPP5E chr10 63311455 0.008137 2.736e-15 35342
TRUE reported textfile 2 1 TRUE
5637 rs1398015 T C T C -0.1083 0.0026 0.05914 NA FALSE FALSE FALSE
GCST006061 0.0215 0.9043 537409 outcome TRUE reported 11370_20_INPP5E_INP5E
INPP5E chr4 73891519 0.017283 3.701e-10 35340 TRUE reported
textfile 2 1 TRUE
5638 rs16850360 G A G A -0.1667 0.0094 0.02454 NA FALSE FALSE FALSE
GCST006061 0.0182 0.6046 537409 outcome TRUE reported 11370_20_INPP5E_INP5E
INPP5E chr4 74006728 0.026935 6.06e-10 35342 TRUE reported
textfile 2 1 TRUE
5639 rs6542680 T C T C -0.0697 -0.0012 0.18362 NA FALSE FALSE FALSE
GCST006061 0.0091 0.8935 537409 outcome TRUE reported 11370_20_INPP5E_INP5E
INPP5E chr2 3592552 0.010456 2.63e-11 35323 TRUE reported textfile
2 1 TRUE
5640 rs10822155 A C A C 0.0635 -0.0472 0.43479 NA FALSE FALSE FALSE
GCST006061 0.0072 4.622e-11 537409 outcome TRUE reported
10499_1_TMEM106A_T106A TMEM106A chr10 63311455 0.008018 2.372e-15 35339
TRUE reported textfile 2 1 TRUE
5641 rs10922098 T C T C -0.0485 -0.009 0.40011 NA FALSE FALSE FALSE
GCST006061 0.0075 0.2275 537409 outcome TRUE reported 10499_1_TMEM106A_T106A
TMEM106A chr1 196695521 0.008135 2.489e-09 35326 TRUE reported
textfile 2 1 TRUE
5642 rs2071040 G A G A -0.1001 -0.0191 0.22348 NA FALSE FALSE FALSE
GCST006061 0.0083 0.02178 537409 outcome TRUE reported 10499_1_TMEM106A_T106A
TMEM106A chr3 52830844 0.00952 7.392e-26 35335 TRUE reported
textfile 2 1 TRUE
5643 rs7157038 C T C T 0.061 0.0153 0.29162 NA FALSE FALSE FALSE
GCST006061 0.0078 0.04843 537409 outcome TRUE reported 10499_1_TMEM106A_T106A
TMEM106A chr14 92735683 0.008756 3.242e-12 35349 TRUE reported
textfile 2 1 TRUE
5644 rs79391435 C T C T 0.0587 0.0034 0.30087 NA FALSE FALSE FALSE
GCST006061 0.0075 0.6484 537409 outcome TRUE reported 10499_1_TMEM106A_T106A
TMEM106A chr17 43321215 0.008722 1.691e-11 35362 TRUE reported
textfile 2 1 TRUE
5645 rs10822155 A C A C 0.0736 -0.0472 0.43479 NA FALSE FALSE FALSE
GCST006061 0.0072 4.622e-11 537409 outcome TRUE reported 2979_8_CXCL5_ENA_78
CXCL5 chr10 63311455 0.008025 4.656e-20 35351 TRUE reported
textfile 2 1 TRUE
5646 rs1619994 G A G A 0.0608 0.0168 0.2807 NA FALSE FALSE FALSE

GCST006061 0.0078 0.03057 537409 outcome TRUE reported 2979_8_CXCL5_ENA_78
CXCL5 chr3 165744047 0.008872 7.227e-12 35339 TRUE reported
textfile 2 1 TRUE
5647 rs352045 G T G T -0.1975 0.0186 0.15324 NA FALSE FALSE FALSE
GCST006061 0.0115 0.1045 537409 outcome TRUE reported 2979_8_CXCL5_ENA_78
CXCL5 chr4 73998970 0.010906 2.724e-73 35351 TRUE reported
textfile 2 1 TRUE
5648 rs572515 G A G A -0.1268 -0.0113 0.3973 NA FALSE FALSE FALSE
GCST006061 0.0075 0.1355 537409 outcome TRUE reported 2979_8_CXCL5_ENA_78
CXCL5 chr1 196677131 0.008138 9.631e-55 35338 TRUE reported
textfile 2 1 TRUE
5649 rs10822155 A C A C 0.0524 -0.0472 0.43479 NA FALSE FALSE FALSE
GCST006061 0.0072 4.622e-11 537409 outcome TRUE reported
8458_111_SNCA_a_Synuclein SNCA chr10 63311455 0.008107 1.022e-10 35351
TRUE reported textfile 2 1 TRUE
5650 rs1354034 C T C T 0.0496 0.0088 0.38119 NA FALSE FALSE FALSE
GCST006061 0.0073 0.2248 537409 outcome TRUE reported
8458_111_SNCA_a_Synuclein SNCA chr3 56815721 0.008275 2.052e-09 35356
TRUE reported textfile 2 1 TRUE
5651 rs2119785 C A C A 0.0461 -0.001 0.47647 NA FALSE FALSE FALSE
GCST006061 0.0071 0.8876 537409 outcome TRUE reported
8458_111_SNCA_a_Synuclein SNCA chr4 89915368 0.008068 1.105e-08 35345
TRUE reported textfile 2 1 TRUE
5652 rs3811444 T C T C -0.0656 0.0103 0.34232 NA FALSE FALSE FALSE
GCST006061 0.0077 0.1812 537409 outcome TRUE reported
8458_111_SNCA_a_Synuclein SNCA chr1 247876149 0.008541 1.588e-14 35156
TRUE reported textfile 2 1 TRUE
5653 rs10822155 A C A C 0.0442 -0.0472 0.43479 NA FALSE FALSE FALSE
GCST006061 0.0072 4.622e-11 537409 outcome TRUE reported 4956_2_EREG_EPI
EREG chr10 63311455 0.008019 3.556e-08 35351 TRUE reported
textfile 2 1 TRUE
5654 rs1354034 C T C T 0.0636 0.0088 0.38119 NA FALSE FALSE FALSE
GCST006061 0.0073 0.2248 537409 outcome TRUE reported 4956_2_EREG_EPI EREG
chr3 56815721 0.00818 7.569e-15 35356 TRUE reported textfile 2 1
TRUE
5655 rs10822155 A C A C 0.05 -0.0472 0.43479 NA FALSE FALSE FALSE
GCST006061 0.0072 4.622e-11 537409 outcome TRUE reported
17164_15_ANXA4_annexin_IV ANXA4 chr10 63311455 0.008017 4.462e-10 35351
TRUE reported textfile 2 1 TRUE
5656 rs148712348 A G A G 0.1541 -0.0223 0.02309 NA FALSE FALSE FALSE
GCST006061 0.0215 0.2977 537409 outcome TRUE reported
17164_15_ANXA4_annexin_IV ANXA4 chr2 69928688 0.02647 5.823e-09 35363
TRUE reported textfile 2 1 TRUE
5657 rs17037076 A G A G -0.2876 0.0743 0.22091 NA FALSE FALSE FALSE
GCST006061 0.0089 7.115e-17 537409 outcome TRUE reported
17164_15_ANXA4_annexin_IV ANXA4 chr2 69806517 0.009525 2.695e-200 35364
TRUE reported textfile 2 1 TRUE
5658 rs10822155 A C A C 0.0547 -0.0472 0.43479 NA FALSE FALSE FALSE
GCST006061 0.0072 4.622e-11 537409 outcome TRUE reported
5939_42_TNFSF12_TWEAK TNFSF12 chr10 63311455 0.008182 2.306e-11 35351
TRUE reported textfile 2 1 TRUE
5659 rs8073033 C T C T 0.0596 0.0052 0.44746 NA FALSE FALSE FALSE
GCST006061 0.0072 0.4678 537409 outcome TRUE reported 5939_42_TNFSF12_TWEAK
TNFSF12 chr17 7989856 0.008266 5.578e-13 35372 TRUE reported textfile
2 1 TRUE
5660 rs10822155 A C A C 0.0474 -0.0472 0.43479 NA FALSE FALSE FALSE
GCST006061 0.0072 4.622e-11 537409 outcome TRUE reported
18243_9_CMPK1_Cytidylate_kinase CMPK1 chr10 63311455 0.007966 2.681e-09
35351 TRUE reported textfile 2 1 TRUE
5661 rs203687 T C T C -0.0485 -0.0146 0.4315 NA FALSE FALSE FALSE
GCST006061 0.0075 0.05005 537409 outcome TRUE reported
18243_9_CMPK1_Cytidylate_kinase CMPK1 chr1 196705140 0.00798 1.217e-09 35339
TRUE reported textfile 2 1 TRUE
5662 rs35687416 T G T G -0.1668 0.0057 0.06493 NA FALSE FALSE FALSE
GCST006061 0.0176 0.7465 537409 outcome TRUE reported
18243_9_CMPK1_Cytidylate_kinase CMPK1 chr1 47368537 0.015955 1.399e-25

35367 TRUE reported textfile 2 1 TRUE
5663 rs10822155 A C A C 0.0619 -0.0472 0.43479 NA FALSE FALSE FALSE
GCST006061 0.0072 4.622e-11 537409 outcome TRUE reported 15416_54_LYG2_LYG2
LYG2 chr10 63311455 0.008124 2.551e-14 35351 TRUE reported
textfile 2 1 TRUE
5664 rs10922098 T C T C -0.0722 -0.009 0.40011 NA FALSE FALSE FALSE
GCST006061 0.0075 0.2275 537409 outcome TRUE reported 15416_54_LYG2_LYG2
LYG2 chr1 196695521 0.008232 1.769e-18 35338 TRUE reported
textfile 2 1 TRUE
5665 rs13412535 A G A G -0.06 -0.0072 0.19379 NA FALSE FALSE FALSE
GCST006061 0.0091 0.43 537409 outcome TRUE reported 15416_54_LYG2_LYG2
LYG2 chr2 224010157 0.010197 4.006e-09 35366 TRUE reported
textfile 2 1 TRUE
5666 rs7172696 G A G A -0.049 0.0115 0.34251 NA FALSE FALSE FALSE
GCST006061 0.0077 0.1371 537409 outcome TRUE reported 15416_54_LYG2_LYG2
LYG2 chr15 101451106 0.008463 7.048e-09 35308 TRUE reported
textfile 2 1 TRUE
5667 rs10822155 A C A C 0.0741 -0.0472 0.43479 NA FALSE FALSE FALSE
GCST006061 0.0072 4.622e-11 537409 outcome TRUE reported 6213_10_CGB2_CGB2
CGB2 chr10 63311455 0.00822 1.969e-19 35351 TRUE reported textfile
2 1 TRUE
5668 rs1398015 T C T C -0.1049 0.0026 0.05914 NA FALSE FALSE FALSE
GCST006061 0.0215 0.9043 537409 outcome TRUE reported 6213_10_CGB2_CGB2
CGB2 chr4 73891519 0.017452 1.845e-09 35349 TRUE reported
textfile 2 1 TRUE
5669 rs10822155 A C A C 0.0461 -0.0472 0.43479 NA FALSE FALSE FALSE
GCST006061 0.0072 4.622e-11 537409 outcome TRUE reported 9110_2_SYT17_SYT17
SYT17 chr10 63311455 0.008033 9.532e-09 35351 TRUE reported
textfile 2 1 TRUE
5670 rs572515 G A G A -0.0551 -0.0113 0.3973 NA FALSE FALSE FALSE
GCST006061 0.0075 0.1355 537409 outcome TRUE reported 9110_2_SYT17_SYT17
SYT17 chr1 196677131 0.008141 1.304e-11 35338 TRUE reported
textfile 2 1 TRUE
5671 rs10822155 A C A C 0.0688 -0.0472 0.43479 NA FALSE FALSE FALSE
GCST006061 0.0072 4.622e-11 537409 outcome TRUE reported 6326_20_BMP4_BMP_4
BMP4 chr10 63311455 0.008138 2.817e-17 35351 TRUE reported
textfile 2 1 TRUE
5672 rs1398015 T C T C -0.1014 0.0026 0.05914 NA FALSE FALSE FALSE
GCST006061 0.0215 0.9043 537409 outcome TRUE reported 6326_20_BMP4_BMP_4
BMP4 chr4 73891519 0.017294 4.533e-09 35349 TRUE reported
textfile 2 1 TRUE
5673 rs16850360 G A G A -0.1715 0.0094 0.02454 NA FALSE FALSE FALSE
GCST006061 0.0182 0.6046 537409 outcome TRUE reported 6326_20_BMP4_BMP_4
BMP4 chr4 74006728 0.026936 1.928e-10 35351 TRUE reported
textfile 2 1 TRUE
5674 rs188400754 T C T C 0.1777 0.1002 0.01919 NA FALSE FALSE FALSE
GCST006061 0.0424 0.01825 537409 outcome TRUE reported 6326_20_BMP4_BMP_4
BMP4 chr2 211734716 0.02996 3.008e-09 35356 TRUE reported textfile
2 1 TRUE
5675 rs10822155 A C A C 0.0643 -0.0472 0.43479 NA FALSE FALSE FALSE
GCST006061 0.0072 4.622e-11 537409 outcome TRUE reported
18385_4_PSMD10_PSD10 PSMD10 chr10 63311455 0.008066 1.567e-15 35351
TRUE reported textfile 2 1 TRUE
5676 rs10922098 T C T C -0.0772 -0.009 0.40011 NA FALSE FALSE FALSE
GCST006061 0.0075 0.2275 537409 outcome TRUE reported 18385_4_PSMD10_PSD10
PSMD10 chr1 196695521 0.008171 3.454e-21 35338 TRUE reported
textfile 2 1 TRUE
5677 rs1355538 G A G A -0.0557 -0.0076 0.39847 NA FALSE FALSE FALSE
GCST006061 0.0072 0.2923 537409 outcome TRUE reported 18385_4_PSMD10_PSD10
PSMD10 chr3 165787389 0.008168 9.168e-12 35338 TRUE reported
textfile 2 1 TRUE
5678 rs10822155 A C A C 0.1122 -0.0472 0.43479 NA FALSE FALSE FALSE
GCST006061 0.0072 4.622e-11 537409 outcome TRUE reported 3365_7_DKK4_Dkk_4
DKK4 chr10 63311455 0.008325 2.112e-41 35351 TRUE reported
textfile 2 1 TRUE
5679 rs10888208 G T G T 0.455 0.0632 0.01397 NA FALSE FALSE FALSE

GCST006061 0.0409 0.1227 537409 outcome TRUE reported 3365_7_DKK4_Dkk_4
DKK4 chr10 47096668 0.035064 1.67e-38 35333 TRUE reported
textfile 2 1 TRUE
5680 rs117565303 T C T C 0.1663 -0.0061 0.03105 NA FALSE FALSE FALSE
GCST006061 0.0281 0.8278 537409 outcome TRUE reported 3365_7_DKK4_Dkk_4
DKK4 chr10 55217204 0.023592 1.802e-12 35358 TRUE reported
textfile 2 1 TRUE
5681 rs12468189 G A G A 0.048 -0.0052 0.40103 NA FALSE FALSE FALSE
GCST006061 0.0074 0.4799 537409 outcome TRUE reported 3365_7_DKK4_Dkk_4
DKK4 chr2 31255954 0.00842 1.191e-08 35369 TRUE reported textfile
2 1 TRUE
5682 rs141099634 C T C T 0.6889 -0.1632 0.0118 NA FALSE FALSE FALSE
GCST006061 0.1635 0.3183 537409 outcome TRUE reported 3365_7_DKK4_Dkk_4
DKK4 chr10 50347786 0.0379 7.89e-74 35353 TRUE reported textfile
2 1 TRUE
5683 rs145591269 G A G A 0.1922 0.0025 0.01489 NA FALSE FALSE FALSE
GCST006061 0.0441 0.9541 537409 outcome TRUE reported 3365_7_DKK4_Dkk_4
DKK4 chr10 61660551 0.033697 1.172e-08 35354 TRUE reported
textfile 2 1 TRUE
5684 rs150513777 G A G A 0.4478 -0.0025 0.0321 NA FALSE FALSE FALSE
GCST006061 0.0255 0.9229 537409 outcome TRUE reported 3365_7_DKK4_Dkk_4
DKK4 chr10 52474587 0.023163 2.851e-83 35355 TRUE reported
textfile 2 1 TRUE
5685 rs34416903 C T C T 0.0504 -0.001 0.30061 NA FALSE FALSE FALSE
GCST006061 0.0084 0.9015 537409 outcome TRUE reported 3365_7_DKK4_Dkk_4
DKK4 chr16 88492837 0.008914 1.57e-08 35318 TRUE reported
textfile 2 1 TRUE
5686 rs7101062 G A G A -0.2769 -0.036 0.03623 NA FALSE FALSE FALSE
GCST006061 0.0364 0.3234 537409 outcome TRUE reported 3365_7_DKK4_Dkk_4
DKK4 chr10 51837325 0.02189 1.124e-36 35358 TRUE reported textfile
2 1 TRUE
5687 rs75580312 A G A G 0.7221 0.0425 0.01464 NA FALSE FALSE FALSE
GCST006061 0.0478 0.3745 537409 outcome TRUE reported 3365_7_DKK4_Dkk_4
DKK4 chr10 51875766 0.032954 1.987e-106 35362 TRUE reported
textfile 2 1 TRUE
5688 rs9905408 C A C A 0.3183 -0.0496 0.05389 NA FALSE FALSE FALSE
GCST006061 0.0152 0.00106 537409 outcome TRUE reported 3365_7_DKK4_Dkk_4
DKK4 chr17 66194566 0.018468 1.454e-66 35370 TRUE reported
textfile 2 1 TRUE
5689 rs10822155 A C A C 0.0687 -0.0472 0.43479 NA FALSE FALSE FALSE
GCST006061 0.0072 4.622e-11 537409 outcome TRUE reported
15545_13_PPP3R1_Calcineurin_B_a PPP3R1 chr10 63311455 0.008108 2.385e-17
35351 TRUE reported textfile 2 1 TRUE
5690 rs13129744 C T C T 0.0623 -0.0045 0.24543 NA FALSE FALSE FALSE
GCST006061 0.008 0.5733 537409 outcome TRUE reported
15545_13_PPP3R1_Calcineurin_B_a PPP3R1 chr4 101835574 0.009384 3.162e-11
35339 TRUE reported textfile 2 1 TRUE
5691 rs1355538 G A G A -0.045 -0.0076 0.39847 NA FALSE FALSE FALSE
GCST006061 0.0072 0.2923 537409 outcome TRUE reported
15545_13_PPP3R1_Calcineurin_B_a PPP3R1 chr3 165787389 0.008211 4.245e-08
35338 TRUE reported textfile 2 1 TRUE
5692 rs4980320 C T C T 0.0463 0.0041 0.46986 NA FALSE FALSE FALSE
GCST006061 0.0076 0.5858 537409 outcome TRUE reported
15545_13_PPP3R1_Calcineurin_B_a PPP3R1 chr11 198986 0.008074 9.784e-09 35352
TRUE reported textfile 2 1 TRUE
5693 rs68066031 C T C T 0.0676 -0.0076 0.19513 NA FALSE FALSE FALSE
GCST006061 0.0091 0.4038 537409 outcome TRUE reported
15545_13_PPP3R1_Calcineurin_B_a PPP3R1 chr2 224015781 0.010144 2.662e-11
35366 TRUE reported textfile 2 1 TRUE
5694 rs10822155 A C A C 0.0905 -0.0472 0.43479 NA FALSE FALSE FALSE
GCST006061 0.0072 4.622e-11 537409 outcome TRUE reported 2790_54_PPBP_NAP_2
PPBP chr10 63311455 0.008237 4.437e-28 35351 TRUE reported
textfile 2 1 TRUE
5695 rs12468189 G A G A 0.0459 -0.0052 0.40103 NA FALSE FALSE FALSE
GCST006061 0.0074 0.4799 537409 outcome TRUE reported 2790_54_PPBP_NAP_2
PPBP chr2 31255954 0.008392 4.513e-08 35369 TRUE reported

textfile 2 1 TRUE
5696 rs7172696 G A G A -0.0499 0.0115 0.34251 NA FALSE FALSE FALSE
GCST006061 0.0077 0.1371 537409 outcome TRUE reported 2790_54_PPBP_NAP_2
PPBP chr15 101451106 0.008573 5.873e-09 35308 TRUE reported
textfile 2 1 TRUE
5697 rs10822155 A C A C 0.0721 -0.0472 0.43479 NA FALSE FALSE FALSE
GCST006061 0.0072 4.622e-11 537409 outcome TRUE reported
6400_33_TP53I13_P5I13 TP53I13 chr10 63311455 0.008337 5.221e-18 35351
TRUE reported textfile 2 1 TRUE
5698 rs1434282 T C T C 0.0898 -0.0021 0.26524 NA FALSE FALSE FALSE
GCST006061 0.008 0.7916 537409 outcome TRUE reported 6400_33_TP53I13_P5I13
TP53I13 chr1 199041592 0.009488 2.934e-21 35343 TRUE reported
textfile 2 1 TRUE
5699 rs342299 T C T C -0.0805 1e-04 0.45394 NA FALSE FALSE FALSE
GCST006061 0.0074 0.9852 537409 outcome TRUE reported 6400_33_TP53I13_P5I13
TP53I13 chr7 106733272 0.008283 2.519e-22 35354 TRUE reported
textfile 2 1 TRUE
5700 rs7776054 G A G A -0.0524 -0.0032 0.27214 NA FALSE FALSE FALSE
GCST006061 0.008 0.6938 537409 outcome TRUE reported 6400_33_TP53I13_P5I13
TP53I13 chr6 135097778 0.009308 1.806e-08 35372 TRUE reported
textfile 2 1 TRUE
5701 rs79880068 T C T C 0.0837 0.0029 0.0927 NA FALSE FALSE FALSE
GCST006061 0.0123 0.8157 537409 outcome TRUE reported 6400_33_TP53I13_P5I13
TP53I13 chr12 54256194 0.014274 4.523e-09 35374 TRUE reported
textfile 2 1 TRUE
5702 rs10822155 A C A C 0.0533 -0.0472 0.43479 NA FALSE FALSE FALSE
GCST006061 0.0072 4.622e-11 537409 outcome TRUE reported
8907_11_UGT2A1_UD2A1 UGT2A1 chr10 63311455 0.00817 6.867e-11 35351 TRUE
reported textfile 2 1 TRUE
5703 rs13412535 A G A G -0.1074 -0.0072 0.19379 NA FALSE FALSE FALSE
GCST006061 0.0091 0.43 537409 outcome TRUE reported 8907_11_UGT2A1_UD2A1
UGT2A1 chr2 224010157 0.010255 1.152e-25 35366 TRUE reported
textfile 2 1 TRUE
5704 rs368465 C T C T -0.0506 -0.012 0.39961 NA FALSE FALSE FALSE
GCST006061 0.0075 0.1119 537409 outcome TRUE reported 8907_11_UGT2A1_UD2A1
UGT2A1 chr1 196702851 0.00829 1.035e-09 35339 TRUE reported textfile
2 1 TRUE
5705 rs704 A G A G 0.0457 -0.0155 0.49189 NA FALSE FALSE FALSE GCST006061
0.0072 0.03144 537409 outcome TRUE reported 8907_11_UGT2A1_UD2A1 UGT2A1
chr17 28367840 0.008128 1.883e-08 35360 TRUE reported textfile 2
1 TRUE
5706 rs10822156 C T C T 0.0538 -0.0527 0.4811 NA FALSE FALSE FALSE
GCST006061 0.0071 7.899e-14 537409 outcome TRUE reported
7891_45_UGT1A6_UGT_1A6 UGT1A6 chr10 63320967 0.007905 1.002e-11 35351
TRUE reported textfile 2 1 TRUE
5707 rs112963922 T C T C 0.2722 0.0542 0.01119 NA FALSE FALSE FALSE
GCST006061 0.0399 0.1741 537409 outcome TRUE reported 7891_45_UGT1A6_UGT_1A6
UGT1A6 chr14 94411531 0.037036 1.987e-13 35371 TRUE reported
textfile 2 1 TRUE
5708 rs58817302 T C T C 0.1488 -0.0314 0.03812 NA FALSE FALSE FALSE
GCST006061 0.0231 0.1735 537409 outcome TRUE reported 7891_45_UGT1A6_UGT_1A6
UGT1A6 chr2 233472784 0.020916 1.125e-12 35374 TRUE reported
textfile 2 1 TRUE
5709 rs7139079 A G A G -0.0579 0.0044 0.38321 NA FALSE FALSE FALSE
GCST006061 0.0072 0.5391 537409 outcome TRUE reported 7891_45_UGT1A6_UGT_1A6
UGT1A6 chr12 120977490 0.008127 1.047e-12 35365 TRUE reported
textfile 2 1 TRUE
5710 rs7238784 T G T G 0.0446 -0.0122 0.39885 NA FALSE FALSE FALSE
GCST006061 0.0078 0.1151 537409 outcome TRUE reported 7891_45_UGT1A6_UGT_1A6
UGT1A6 chr18 74260617 0.008072 3.293e-08 35376 TRUE reported
textfile 2 1 TRUE
5711 rs887829 T C T C -0.2321 0.0019 0.31955 NA FALSE FALSE FALSE
GCST006061 0.0077 0.8072 537409 outcome TRUE reported 7891_45_UGT1A6_UGT_1A6
UGT1A6 chr2 233759924 0.008441 1.89e-166 35372 TRUE reported
textfile 2 1 TRUE
5712 rs10822159 T C T C 0.0485 -0.0466 0.43566 NA FALSE FALSE FALSE

GCST006061 0.0071 6.851e-11 537409 outcome TRUE reported
6207_10_PSAP_prosaposin PSAP chr10 63336490 0.008323 5.633e-09 35351
TRUE reported textfile 2 1 TRUE
5713 rs11111026 T G T G -0.1677 0.0017 0.19802 NA FALSE FALSE FALSE
GCST006061 0.0091 0.8502 537409 outcome TRUE reported 6207_10_PSAP_prosaposin
PSAP chr12 101799025 0.010314 1.905e-59 35364 TRUE reported
textfile 2 1 TRUE
5714 rs113654122 T C T C 0.3161 0.0105 0.03521 NA FALSE FALSE FALSE
GCST006061 0.0202 0.6009 537409 outcome TRUE reported 6207_10_PSAP_prosaposin
PSAP chr10 71933122 0.022237 7.42e-46 35370 TRUE reported
textfile 2 1 TRUE
5715 rs1194179 C T C T 0.0524 -0.0129 0.48178 NA FALSE FALSE FALSE
GCST006061 0.0072 0.07169 537409 outcome TRUE reported 6207_10_PSAP_prosaposin
PSAP chr7 80603657 0.008118 1.083e-10 35347 TRUE reported
textfile 2 1 TRUE
5716 rs1293316 G A G A 0.1134 -0.0145 0.2613 NA FALSE FALSE FALSE
GCST006061 0.0084 0.08191 537409 outcome TRUE reported 6207_10_PSAP_prosaposin
PSAP chr8 11833406 0.009302 3.497e-34 35378 TRUE reported
textfile 2 1 TRUE
5717 rs1530804 G A G A 0.0601 -0.0055 0.28785 NA FALSE FALSE FALSE
GCST006061 0.0078 0.4832 537409 outcome TRUE reported 6207_10_PSAP_prosaposin
PSAP chr10 72067534 0.009093 3.862e-11 35373 TRUE reported
textfile 2 1 TRUE
5718 rs174584 A G A G 0.0468 0.012 0.39181 NA FALSE FALSE FALSE
GCST006061 0.0074 0.1059 537409 outcome TRUE reported 6207_10_PSAP_prosaposin
PSAP chr11 61843278 0.008342 2.018e-08 35367 TRUE reported
textfile 2 1 TRUE
5719 rs2127868 A G A G 0.0671 -0.0083 0.17112 NA FALSE FALSE FALSE
GCST006061 0.0085 0.3283 537409 outcome TRUE reported 6207_10_PSAP_prosaposin
PSAP chr14 65327609 0.010775 4.746e-10 35359 TRUE reported
textfile 2 1 TRUE
5720 rs2868346 T C T C -0.0797 0.0118 0.26325 NA FALSE FALSE FALSE
GCST006061 0.0082 0.1511 537409 outcome TRUE reported 6207_10_PSAP_prosaposin
PSAP chr20 45919331 0.009231 5.916e-18 35370 TRUE reported
textfile 2 1 TRUE
5721 rs4883201 G A G A 0.0959 -0.0182 0.12502 NA FALSE FALSE FALSE
GCST006061 0.0114 0.1094 537409 outcome TRUE reported 6207_10_PSAP_prosaposin
PSAP chr12 8929985 0.012353 8.272e-15 35387 TRUE reported textfile
2 1 TRUE
5722 rs56255430 C A C A 0.0883 -0.0034 0.0795 NA FALSE FALSE FALSE
GCST006061 0.0132 0.7997 537409 outcome TRUE reported 6207_10_PSAP_prosaposin
PSAP chr19 19367068 0.015167 5.824e-09 35353 TRUE reported
textfile 2 1 TRUE
5723 rs825038 A C A C 0.114 0.0218 0.05118 NA FALSE FALSE FALSE
GCST006061 0.0239 0.3624 537409 outcome TRUE reported 6207_10_PSAP_prosaposin
PSAP chr12 101570303 0.018526 7.583e-10 35363 TRUE reported
textfile 2 1 TRUE
5724 rs10822186 G A G A 0.0465 -0.0493 0.4663 NA FALSE FALSE FALSE
GCST006061 0.0071 2.964e-12 537409 outcome TRUE reported 8356_88_OXT_NEU1
OXT chr10 63590623 0.008099 9.378e-09 35351 TRUE reported textfile
2 1 TRUE
5725 rs11087565 C T C T -0.0752 -0.0109 0.1956 NA FALSE FALSE FALSE
GCST006061 0.0097 0.2612 537409 outcome TRUE reported 8356_88_OXT_NEU1 OXT
chr20 3103947 0.010255 2.255e-13 35373 TRUE reported textfile 2 1
TRUE
5726 rs6047800 C T C T -0.0669 9e-04 0.1496 NA FALSE FALSE FALSE
GCST006061 0.0098 0.929 537409 outcome TRUE reported 8356_88_OXT_NEU1 OXT
chr20 2272093 0.011454 5.198e-09 35373 TRUE reported textfile 2 1
TRUE
5727 rs6115677 G A G A 0.1531 0.0021 0.17233 NA FALSE FALSE FALSE
GCST006061 0.0093 0.8217 537409 outcome TRUE reported 8356_88_OXT_NEU1 OXT
chr20 2809471 0.010756 5.651e-46 35376 TRUE reported textfile 2 1
TRUE
5728 rs6115776 C A C A 0.3183 -0.0043 0.40241 NA FALSE FALSE FALSE
GCST006061 0.0082 0.5979 537409 outcome TRUE reported 8356_88_OXT_NEU1 OXT
chr20 3067658 0.006773 1e-200 35374 TRUE reported textfile 2 1 TRUE

5729 rs10823375 G A G A 0.0887 -0.0109 0.44471 NA FALSE FALSE FALSE
GCST006061 0.0073 0.1352 537409 outcome TRUE reported 5090_49_LILRB1_ILT_2
LILRB1 chr10 69442226 0.007666 5.81e-31 35365 TRUE reported
textfile 2 1 TRUE
5730 rs117587884 A G A G 0.2037 -0.0377 0.01831 NA FALSE FALSE FALSE
GCST006061 0.0325 0.2464 537409 outcome TRUE reported 5090_49_LILRB1_ILT_2
LILRB1 chr16 57058087 0.028495 8.761e-13 35371 TRUE reported
textfile 2 1 TRUE
5731 rs117868499 A G A G -0.2234 0.0089 0.04197 NA FALSE FALSE FALSE
GCST006061 0.0252 0.7252 537409 outcome TRUE reported 5090_49_LILRB1_ILT_2
LILRB1 chr19 55454908 0.021226 6.626e-26 35381 TRUE reported
textfile 2 1 TRUE
5732 rs117886241 T C T C -0.7499 0.0128 0.04457 NA FALSE FALSE FALSE
GCST006061 0.0254 0.6136 537409 outcome TRUE reported 5090_49_LILRB1_ILT_2
LILRB1 chr19 54454030 0.020214 1e-200 35371 TRUE reported textfile
2 1 TRUE
5733 rs143501783 C T C T 0.2189 -0.0333 0.01116 NA FALSE FALSE FALSE
GCST006061 0.0443 0.4525 537409 outcome TRUE reported 5090_49_LILRB1_ILT_2
LILRB1 chr17 7897001 0.034613 2.545e-10 35373 TRUE reported textfile
2 1 TRUE
5734 rs154042 A G A G -0.0606 -0.0037 0.40743 NA FALSE FALSE FALSE
GCST006061 0.0074 0.6137 537409 outcome TRUE reported 5090_49_LILRB1_ILT_2
LILRB1 chr16 57081891 0.00779 7.325e-15 35371 TRUE reported textfile
2 1 TRUE
5735 rs289747 T C T C 0.0422 -0.0104 0.43265 NA FALSE FALSE FALSE
GCST006061 0.0074 0.163 537409 outcome TRUE reported 5090_49_LILRB1_ILT_2
LILRB1 chr16 56990026 0.007719 4.578e-08 35370 TRUE reported
textfile 2 1 TRUE
5736 rs35064159 C T C T 0.0685 -0.0321 0.10573 NA FALSE FALSE FALSE
GCST006061 0.013 0.01389 537409 outcome TRUE reported 5090_49_LILRB1_ILT_2
LILRB1 chr6 95634240 0.012555 4.872e-08 35343 TRUE reported
textfile 2 1 TRUE
5737 rs56115403 A G A G 0.0812 0.0012 0.21597 NA FALSE FALSE FALSE
GCST006061 0.01 0.901 537409 outcome TRUE reported 5090_49_LILRB1_ILT_2
LILRB1 chr17 7160579 0.009298 2.477e-18 35371 TRUE reported textfile
2 1 TRUE
5738 rs62165726 A C A C -0.2108 -0.0263 0.03053 NA FALSE FALSE FALSE
GCST006061 0.0212 0.2141 537409 outcome TRUE reported 5090_49_LILRB1_ILT_2
LILRB1 chr2 134208991 0.021974 8.559e-22 35391 TRUE reported
textfile 2 1 TRUE
5739 rs73021415 T C T C -0.1062 0.0074 0.05905 NA FALSE FALSE FALSE
GCST006061 0.0143 0.6056 537409 outcome TRUE reported 5090_49_LILRB1_ILT_2
LILRB1 chr11 126378312 0.016099 4.205e-11 35376 TRUE reported
textfile 2 1 TRUE
5740 rs7865362 T C T C 0.0484 0.0022 0.34624 NA FALSE FALSE FALSE
GCST006061 0.0075 0.7726 537409 outcome TRUE reported 5090_49_LILRB1_ILT_2
LILRB1 chr9 33117967 0.007959 1.192e-09 35349 TRUE reported
textfile 2 1 TRUE
5741 rs9263773 C T C T 0.1315 4e-04 0.43963 NA FALSE FALSE FALSE
GCST006061 0.0077 0.956 537409 outcome TRUE reported 5090_49_LILRB1_ILT_2
LILRB1 chr6 31152463 0.007789 6.002e-64 35438 TRUE reported
textfile 2 1 TRUE
5742 rs10823375 G A G A 0.063 -0.0109 0.44471 NA FALSE FALSE FALSE
GCST006061 0.0073 0.1352 537409 outcome TRUE reported 5091_28_LILRB2_ILT_4
LILRB2 chr10 69442226 0.008725 5.185e-13 35365 TRUE reported
textfile 2 1 TRUE
5743 rs117421142 G A G A 0.2403 -0.0037 0.02216 NA FALSE FALSE FALSE
GCST006061 0.028 0.895 537409 outcome TRUE reported 5091_28_LILRB2_ILT_4
LILRB2 chr19 54252383 0.031086 1.074e-14 35370 TRUE reported
textfile 2 1 TRUE
5744 rs55714927 T C T C 0.0734 1e-04 0.21166 NA FALSE FALSE FALSE
GCST006061 0.0098 0.9926 537409 outcome TRUE reported 5091_28_LILRB2_ILT_4
LILRB2 chr17 7176997 0.010668 5.963e-12 35370 TRUE reported textfile
2 1 TRUE
5745 rs6688272 T G T G -0.0764 8e-04 0.38664 NA FALSE FALSE FALSE
GCST006061 0.0072 0.9072 537409 outcome TRUE reported 5091_28_LILRB2_ILT_4

```
      LILRB2  chr1    196715262   0.008886    8.111e-18   35339   TRUE    reported
      textfile    2   1   TRUE
5746  rs9267084   A   G   A   G   0.1528  -0.0071 0.14154 NA  FALSE   FALSE   FALSE
      GCST006061  0.0111  0.5238  537409  outcome TRUE    reported    5091_28_LILRB2_ILT_4
      LILRB2  chr6    31441828    0.012468    1.58e-34    35439   TRUE    reported
      textfile    2   1   TRUE
5747  rs10823375  G   A   G   A   0.0688  -0.0109 0.44471 NA  FALSE   FALSE   FALSE
      GCST006061  0.0073  0.1352  537409  outcome TRUE    reported    3166_92_CD33_Siglec_3
      CD33    chr10   69442226    0.007984    6.875e-18   35365   TRUE    reported
      textfile    2   1   TRUE
5748  rs117711212 T   C   T   C   -0.3711 -0.0328 0.01096 NA  FALSE   FALSE   FALSE
      GCST006061  0.0361  0.3636  537409  outcome TRUE    reported    3166_92_CD33_Siglec_3
      CD33    chr19   50506164    0.045787    5.277e-16   35374   TRUE    reported
      textfile    2   1   TRUE
5749  rs16983230  G   T   G   T   0.2093  0.01    0.05579 NA  FALSE   FALSE   FALSE
      GCST006061  0.0209  0.633   537409  outcome TRUE    reported    3166_92_CD33_Siglec_3
      CD33    chr19   51915442    0.02124 6.587e-23   35385   TRUE    reported    textfile
      2   1   TRUE
5750  rs1846934   T   C   T   C   0.332   -0.0167 0.4809  NA  FALSE   FALSE   FALSE
      GCST006061  0.0081  0.03888 537409  outcome TRUE    reported    3166_92_CD33_Siglec_3
      CD33    chr19   51145440    0.009675    1e-200  35372   TRUE    reported    textfile
      2   1   TRUE
5751  rs2459147   G   A   G   A   -0.3914 0.0036  0.42022 NA  FALSE   FALSE   FALSE
      GCST006061  0.0073  0.6172  537409  outcome TRUE    reported    3166_92_CD33_Siglec_3
      CD33    chr19   51262922    0.005813    1e-200  35379   TRUE    reported    textfile
      2   1   TRUE
5752  rs55714927  T   C   T   C   0.1081  1e-04   0.21166 NA  FALSE   FALSE   FALSE
      GCST006061  0.0098  0.9926  537409  outcome TRUE    reported    3166_92_CD33_Siglec_3
      CD33    chr17   7176997 0.00978 2.111e-28   35370   TRUE    reported    textfile    2
      1   TRUE
5753  rs62114983  C   A   C   A   0.1698  0.0386  0.03645 NA  FALSE   FALSE   FALSE
      GCST006061  0.0371  0.2981  537409  outcome TRUE    reported    3166_92_CD33_Siglec_3
      CD33    chr19   51594347    0.026437    1.337e-10   35383   TRUE    reported
      textfile    2   1   TRUE
5754  rs67621164  A   G   A   G   -0.1959 -0.0154 0.16767 NA  FALSE   FALSE   FALSE
      GCST006061  0.0104  0.1383  537409  outcome TRUE    reported    3166_92_CD33_Siglec_3
      CD33    chr19   51132484    0.01304 5.217e-51   35372   TRUE    reported    textfile
      2   1   TRUE
5755  rs73021419  T   C   T   C   -0.0775 0.0022  0.10279 NA  FALSE   FALSE   FALSE
      GCST006061  0.01    0.8256  537409  outcome TRUE    reported    3166_92_CD33_Siglec_3
      CD33    chr11   126380752   0.013022    2.657e-09   35376   TRUE    reported
      textfile    2   1   TRUE
5756  rs76920575  T   G   T   G   -0.1969 -0.0532 0.02615 NA  FALSE   FALSE   FALSE
      GCST006061  0.0227  0.01903 537409  outcome TRUE    reported    3166_92_CD33_Siglec_3
      CD33    chr19   51793132    0.03071 1.44e-10    35384   TRUE    reported    textfile
      2   1   TRUE
5757  rs10823655  G   A   G   A   0.0722  -0.0115 0.12425 NA  FALSE   FALSE   FALSE
      GCST006061  0.0103  0.2658  537409  outcome TRUE    reported    15394_79_UNC5B_UNC5B
      UNC5B   chr10   70990912    0.012395    5.72e-09    35371   TRUE    reported
      textfile    2   1   TRUE
5758  rs10999762  A   G   A   G   -0.1125 0.0023  0.22104 NA  FALSE   FALSE   FALSE
      GCST006061  0.0084  0.7834  537409  outcome TRUE    reported    15394_79_UNC5B_UNC5B
      UNC5B   chr10   71284917    0.009842    2.957e-30   35374   TRUE    reported
      textfile    2   1   TRUE
5759  rs2837995   T   C   T   C   0.1243  0.0104  0.03452 NA  FALSE   FALSE   FALSE
      GCST006061  0.0175  0.5544  537409  outcome TRUE    reported    15394_79_UNC5B_UNC5B
      UNC5B   chr21   41252543    0.021938    1.462e-08   35368   TRUE    reported
      textfile    2   1   TRUE
5760  rs72704449  C   T   C   T   0.1726  0.013   0.04582 NA  FALSE   FALSE   FALSE
      GCST006061  0.0199  0.5128  537409  outcome TRUE    reported    15394_79_UNC5B_UNC5B
      UNC5B   chr1    179504138   0.019481    8.006e-19   35358   TRUE    reported
      textfile    2   1   TRUE
5761  rs77924615  A   G   A   G   -0.0588 0.0136  0.19701 NA  FALSE   FALSE   FALSE
      GCST006061  0.0092  0.1375  537409  outcome TRUE    reported    15394_79_UNC5B_UNC5B
      UNC5B   chr16   20381010    0.010352    1.348e-08   35355   TRUE    reported
      textfile    2   1   TRUE
```

5762 rs780094 C T C T 0.0548 -0.0076 0.33917 NA FALSE FALSE FALSE
GCST006061 0.0073 0.2951 537409 outcome TRUE reported 15394_79_UNC5B_UNC5B
UNC5B chr2 27518370 0.00866 2.482e-10 35373 TRUE reported textfile
2 1 TRUE
5763 rs8176743 T C T C 0.2497 0.017 0.06488 NA FALSE FALSE FALSE
GCST006061 0.0128 0.1834 537409 outcome TRUE reported 15394_79_UNC5B_UNC5B
UNC5B chr9 133256028 0.016644 7.049e-51 35370 TRUE reported
textfile 2 1 TRUE
5764 rs10828722 G A G A -0.054 0.0013 0.35928 NA FALSE FALSE FALSE
GCST006061 0.0077 0.8662 537409 outcome TRUE reported 11573_3_SRSF6_SRSF6
SRSF6 chr10 24911022 0.008366 1.082e-10 35353 TRUE reported
textfile 2 1 TRUE
5765 rs10832616 A G A G 0.0629 0.0048 0.22532 NA FALSE FALSE FALSE
GCST006061 0.0087 0.5778 537409 outcome TRUE reported 18830_1_ITLN1_Omentin
ITLN1 chr11 16484543 0.010005 3.238e-10 35370 TRUE reported
textfile 2 1 TRUE
5766 rs10922098 T C T C -0.1631 -0.009 0.40011 NA FALSE FALSE FALSE
GCST006061 0.0075 0.2275 537409 outcome TRUE reported 18830_1_ITLN1_Omentin
ITLN1 chr1 196695521 0.00854 2.563e-81 35338 TRUE reported textfile
2 1 TRUE
5767 rs141410828 T C T C 0.2489 -0.0074 0.01144 NA FALSE FALSE FALSE
GCST006061 0.0276 0.7877 537409 outcome TRUE reported 18830_1_ITLN1_Omentin
ITLN1 chr1 160875666 0.038786 1.388e-10 35365 TRUE reported
textfile 2 1 TRUE
5768 rs1707652 T C T C 0.0831 0.0081 0.3102 NA FALSE FALSE FALSE
GCST006061 0.0074 0.2701 537409 outcome TRUE reported 18830_1_ITLN1_Omentin
ITLN1 chr3 165761011 0.009024 3.31e-20 35339 TRUE reported
textfile 2 1 TRUE
5769 rs35209896 A C A C 0.0807 0.0267 0.09263 NA FALSE FALSE FALSE
GCST006061 0.0355 0.4521 537409 outcome TRUE reported 18830_1_ITLN1_Omentin
ITLN1 chr6 32519009 0.014325 1.765e-08 35439 TRUE reported
textfile 2 1 TRUE
5770 rs4722168 A G A G 0.0473 0.0086 0.46999 NA FALSE FALSE FALSE
GCST006061 0.0076 0.258 537409 outcome TRUE reported 18830_1_ITLN1_Omentin
ITLN1 chr7 22739383 0.008425 1.971e-08 35372 TRUE reported
textfile 2 1 TRUE
5771 rs62193065 A G A G -0.0711 0.0082 0.18195 NA FALSE FALSE FALSE
GCST006061 0.0109 0.45 537409 outcome TRUE reported 18830_1_ITLN1_Omentin
ITLN1 chr2 241789202 0.010965 8.914e-11 35114 TRUE reported
textfile 2 1 TRUE
5772 rs6768262 G A G A 0.0497 0.0044 0.33979 NA FALSE FALSE FALSE
GCST006061 0.0078 0.5667 537409 outcome TRUE reported 18830_1_ITLN1_Omentin
ITLN1 chr3 167088625 0.008833 1.837e-08 35354 TRUE reported
textfile 2 1 TRUE
5773 rs10863962 T C T C 0.0738 0.008 0.25986 NA FALSE FALSE FALSE
GCST006061 0.0081 0.3226 537409 outcome TRUE reported 10603_1_HTN3_HIS3
HTN3 chr1 206955921 0.009323 2.451e-15 35362 TRUE reported
textfile 2 1 TRUE
5774 rs115000417 T C T C -0.442 -0.0458 0.0322 NA FALSE FALSE FALSE
GCST006061 0.03 0.1262 537409 outcome TRUE reported 10603_1_HTN3_HIS3
HTN3 chr4 70014802 0.02342 1.912e-79 35345 TRUE reported textfile
2 1 TRUE
5775 rs12517853 A G A G -0.0913 -0.0284 0.08551 NA FALSE FALSE FALSE
GCST006061 0.0136 0.03669 537409 outcome TRUE reported 10603_1_HTN3_HIS3
HTN3 chr5 96785443 0.014597 3.984e-10 35337 TRUE reported
textfile 2 1 TRUE
5776 rs13357659 G A G A -0.0877 0.0014 0.39638 NA FALSE FALSE FALSE
GCST006061 0.0072 0.8426 537409 outcome TRUE reported 10603_1_HTN3_HIS3
HTN3 chr5 44432783 0.008361 9.625e-26 35348 TRUE reported
textfile 2 1 TRUE
5777 rs150022487 A G A G 0.1348 -0.0148 0.07224 NA FALSE FALSE FALSE
GCST006061 0.0187 0.4295 537409 outcome TRUE reported 10603_1_HTN3_HIS3
HTN3 chr4 127900150 0.015766 1.232e-17 35351 TRUE reported
textfile 2 1 TRUE
5778 rs3749751 T C T C -0.0943 0.0026 0.38867 NA FALSE FALSE FALSE
GCST006061 0.0073 0.7172 537409 outcome TRUE reported 10603_1_HTN3_HIS3

HTN3 chr5 135173987 0.008389 2.555e-29 35348 TRUE reported
textfile 2 1 TRUE
5779 rs61762319 G A G A 0.2029 -0.0455 0.02082 NA FALSE FALSE FALSE
GCST006061 0.0273 0.09563 537409 outcome TRUE reported 10603_1_HTN3_HIS3
HTN3 chr3 155084189 0.028655 1.434e-12 35328 TRUE reported
textfile 2 1 TRUE
5780 rs61930005 A G A G 0.0917 -0.0109 0.12195 NA FALSE FALSE FALSE
GCST006061 0.011 0.3199 537409 outcome TRUE reported 10603_1_HTN3_HIS3
HTN3 chr12 11214367 0.012452 1.784e-13 35363 TRUE reported
textfile 2 1 TRUE
5781 rs681343 T C T C 0.1264 0.0054 0.3954 NA FALSE FALSE FALSE
GCST006061 0.0076 0.4742 537409 outcome TRUE reported 10603_1_HTN3_HIS3
HTN3 chr19 48703205 0.008367 1.469e-51 35365 TRUE reported
textfile 2 1 TRUE
5782 rs848097 C T C T 0.0469 -0.0121 0.39216 NA FALSE FALSE FALSE
GCST006061 0.0072 0.09641 537409 outcome TRUE reported 10603_1_HTN3_HIS3
HTN3 chr14 36792277 0.008388 2.251e-08 35348 TRUE reported
textfile 2 1 TRUE
5783 rs10833712 G T G T 0.0959 -0.0242 0.3971 NA FALSE FALSE FALSE
GCST006061 0.0074 0.001125 537409 outcome TRUE reported
13534_20_MYOM2_MYOM2 MYOM2 chr11 22179461 0.008329 1.129e-30 35354
TRUE reported textfile 2 1 TRUE
5784 rs12463674 G A G A 0.0586 0.0097 0.28112 NA FALSE FALSE FALSE
GCST006061 0.008 0.2259 537409 outcome TRUE reported 13534_20_MYOM2_MYOM2
MYOM2 chr2 178567458 0.009079 1.085e-10 35355 TRUE reported
textfile 2 1 TRUE
5785 rs12473286 T G T G -0.05 0.0178 0.27749 NA FALSE FALSE FALSE
GCST006061 0.0081 0.0278 537409 outcome TRUE reported 13534_20_MYOM2_MYOM2
MYOM2 chr2 219491798 0.009098 3.896e-08 35366 TRUE reported
textfile 2 1 TRUE
5786 rs17617337 T C T C 0.0586 -0.0028 0.2412 NA FALSE FALSE FALSE
GCST006061 0.0091 0.7591 537409 outcome TRUE reported 13534_20_MYOM2_MYOM2
MYOM2 chr10 119667372 0.009535 7.973e-10 35372 TRUE reported
textfile 2 1 TRUE
5787 rs1799938 A G A G 0.0958 0.0048 0.1061 NA FALSE FALSE FALSE
GCST006061 0.0112 0.6678 537409 outcome TRUE reported 13534_20_MYOM2_MYOM2
MYOM2 chr17 67056188 0.013373 7.862e-13 35361 TRUE reported
textfile 2 1 TRUE
5788 rs7211380 G A G A 0.1052 -0.0418 0.06768 NA FALSE FALSE FALSE
GCST006061 0.0136 0.002092 537409 outcome TRUE reported
13534_20_MYOM2_MYOM2 MYOM2 chr17 66210650 0.016475 1.708e-10 35364
TRUE reported textfile 2 1 TRUE
5789 rs73184720 T C T C -0.1513 0.0038 0.0266 NA FALSE FALSE FALSE
GCST006061 0.0333 0.9093 537409 outcome TRUE reported 13534_20_MYOM2_MYOM2
MYOM2 chr8 1973246 0.025454 2.78e-09 35277 TRUE reported textfile
2 1 TRUE
5790 rs7318906 A G A G -0.0718 0.0063 0.46975 NA FALSE FALSE FALSE
GCST006061 0.0072 0.3803 537409 outcome TRUE reported 13534_20_MYOM2_MYOM2
MYOM2 chr13 73531994 0.008213 2.286e-18 35351 TRUE reported
textfile 2 1 TRUE
5791 rs74455617 T C T C -0.2778 -0.0539 0.01605 NA FALSE FALSE FALSE
GCST006061 0.0538 0.3168 537409 outcome TRUE reported 13534_20_MYOM2_MYOM2
MYOM2 chr8 2164884 0.032461 1.148e-17 35283 TRUE reported textfile
2 1 TRUE
5792 rs10833719 C T C T 0.1161 -0.0226 0.41016 NA FALSE FALSE FALSE
GCST006061 0.0075 0.002572 537409 outcome TRUE reported
18376_19_MYL3_Myosin_light_chain_1 MYL3 chr11 22205338 0.008447 5.515e-43
35360 TRUE reported textfile 2 1 TRUE
5793 rs113785232 A G A G 0.1471 0.0322 0.03583 NA FALSE FALSE FALSE
GCST006061 0.0234 0.1686 537409 outcome TRUE reported
18376_19_MYL3_Myosin_light_chain_1 MYL3 chr1 228474243 0.023059 1.779e-10
35359 TRUE reported textfile 2 1 TRUE
5794 rs12463674 G A G A 0.0655 0.0097 0.28112 NA FALSE FALSE FALSE
GCST006061 0.008 0.2259 537409 outcome TRUE reported
18376_19_MYL3_Myosin_light_chain_1 MYL3 chr2 178567458 0.009238 1.338e-12
35361 TRUE reported textfile 2 1 TRUE

5795 rs17617337 T C T C 0.065 -0.0028 0.2412 NA FALSE FALSE FALSE
GCST006061 0.0091 0.7591 537409 outcome TRUE reported
18376_19_MYL3_Myosin_light_chain_1 MYL3 chr10 119667372 0.009681 1.888e-11
35378 TRUE reported textfile 2 1 TRUE
5796 rs6431221 A C A C 0.0461 4e-04 0.4821 NA FALSE FALSE FALSE
GCST006061 0.0072 0.956 537409 outcome TRUE reported
18376_19_MYL3_Myosin_light_chain_1 MYL3 chr2 127127018 0.00836 3.497e-08
35368 TRUE reported textfile 2 1 TRUE
5797 rs7318906 A G A G -0.098 0.0063 0.46975 NA FALSE FALSE FALSE
GCST006061 0.0072 0.3803 537409 outcome TRUE reported
18376_19_MYL3_Myosin_light_chain_1 MYL3 chr13 73531994 0.008336 6.532e-32
35357 TRUE reported textfile 2 1 TRUE
5798 rs10833719 C T C T 0.1038 -0.0226 0.41016 NA FALSE FALSE FALSE
GCST006061 0.0075 0.002572 537409 outcome TRUE reported
9844_138_ACTN2_ACTN2 ACTN2 chr11 22205338 0.008338 1.418e-35 35360
TRUE reported textfile 2 1 TRUE
5799 rs12463674 G A G A 0.0662 0.0097 0.28112 NA FALSE FALSE FALSE
GCST006061 0.008 0.2259 537409 outcome TRUE reported 9844_138_ACTN2_ACTN2
ACTN2 chr2 178567458 0.009122 3.963e-13 35361 TRUE reported
textfile 2 1 TRUE
5800 rs138536547 T C T C -0.0961 0.0382 0.05783 NA FALSE FALSE FALSE
GCST006061 0.0157 0.01483 537409 outcome TRUE reported 9844_138_ACTN2_ACTN2
ACTN2 chr19 38487596 0.017522 4.144e-08 35375 TRUE reported
textfile 2 1 TRUE
5801 rs17617337 T C T C 0.0714 -0.0028 0.2412 NA FALSE FALSE FALSE
GCST006061 0.0091 0.7591 537409 outcome TRUE reported 9844_138_ACTN2_ACTN2
ACTN2 chr10 119667372 0.009559 8.059e-14 35378 TRUE reported
textfile 2 1 TRUE
5802 rs1799938 A G A G 0.0982 0.0048 0.1061 NA FALSE FALSE FALSE
GCST006061 0.0112 0.6678 537409 outcome TRUE reported 9844_138_ACTN2_ACTN2
ACTN2 chr17 67056188 0.013425 2.583e-13 35367 TRUE reported
textfile 2 1 TRUE
5803 rs2800280 G A G A 0.0472 -0.0283 0.38146 NA FALSE FALSE FALSE
GCST006061 0.0074 0.0001356 537409 outcome TRUE reported
9844_138_ACTN2_ACTN2 ACTN2 chr9 100570530 0.008436 2.208e-08 35343
TRUE reported textfile 2 1 TRUE
5804 rs523206 C T C T 0.0482 -0.0052 0.31746 NA FALSE FALSE FALSE
GCST006061 0.0079 0.5124 537409 outcome TRUE reported 9844_138_ACTN2_ACTN2
ACTN2 chr6 117305139 0.008797 4.269e-08 35351 TRUE reported
textfile 2 1 TRUE
5805 rs61752479 A G A G 0.069 -0.0059 0.12457 NA FALSE FALSE FALSE
GCST006061 0.0113 0.5997 537409 outcome TRUE reported 9844_138_ACTN2_ACTN2
ACTN2 chr1 114688633 0.012555 3.893e-08 35363 TRUE reported
textfile 2 1 TRUE
5806 rs7211380 G A G A 0.0986 -0.0418 0.06768 NA FALSE FALSE FALSE
GCST006061 0.0136 0.002092 537409 outcome TRUE reported
9844_138_ACTN2_ACTN2 ACTN2 chr17 66210650 0.01654 2.502e-09 35370 TRUE
reported textfile 2 1 TRUE
5807 rs9318187 G T G T 0.0764 0.0021 0.48793 NA FALSE FALSE FALSE
GCST006061 0.0073 0.7769 537409 outcome TRUE reported 9844_138_ACTN2_ACTN2
ACTN2 chr13 73536275 0.008224 1.542e-20 35358 TRUE reported
textfile 2 1 TRUE
5808 rs10833719 C T C T 0.0492 -0.0226 0.41016 NA FALSE FALSE FALSE
GCST006061 0.0075 0.002572 537409 outcome TRUE reported
5440_26_TNNI2_Troponin_I__skeletal__fast_twitch TNNI2 chr11 22205338 0.008164
1.675e-09 35360 TRUE reported textfile 2 1 TRUE
5809 rs11599353 A G A G -0.0463 0.0039 0.40717 NA FALSE FALSE FALSE
GCST006061 0.0074 0.5943 537409 outcome TRUE reported
5440_26_TNNI2_Troponin_I__skeletal__fast_twitch TNNI2 chr10 68242630 0.008143
1.299e-08 35363 TRUE reported textfile 2 1 TRUE
5810 rs11651658 C T C T 0.0917 -0.0419 0.06769 NA FALSE FALSE FALSE
GCST006061 0.0135 0.001949 537409 outcome TRUE reported
5440_26_TNNI2_Troponin_I__skeletal__fast_twitch TNNI2 chr17 66202522 0.016164
1.403e-08 35370 TRUE reported textfile 2 1 TRUE
5811 rs1799938 A G A G 0.0857 0.0048 0.1061 NA FALSE FALSE FALSE
GCST006061 0.0112 0.6678 537409 outcome TRUE reported

5440_26_TNNI2_Troponin_I__skeletal__fast_twitch TNNI2 chr17 67056188 0.013117
6.415e-11 35367 TRUE reported textfile 2 1 TRUE
5812 rs1815739 T C T C -0.048 -0.0031 0.44258 NA FALSE FALSE FALSE
GCST006061 0.0072 0.6614 537409 outcome TRUE reported
5440_26_TNNI2_Troponin_I__skeletal__fast_twitch TNNI2 chr11 66560624 0.008101
3.121e-09 35364 TRUE reported textfile 2 1 TRUE
5813 rs583104 T G T G 0.1023 -0.0173 0.20819 NA FALSE FALSE FALSE
GCST006061 0.0087 0.04629 537409 outcome TRUE reported
5440_26_TNNI2_Troponin_I__skeletal__fast_twitch TNNI2 chr1 109278685 0.009899
4.926e-25 35344 TRUE reported textfile 2 1 TRUE
5814 rs10836121 T C T C -0.0712 -0.0017 0.48319 NA FALSE FALSE FALSE
GCST006061 0.008 0.8322 537409 outcome TRUE reported 11514_196_CD59_CD59
CD59 chr11 33848167 0.008107 1.604e-18 35357 TRUE reported
textfile 2 1 TRUE
5815 rs10922098 T C T C -0.0885 -0.009 0.40011 NA FALSE FALSE FALSE
GCST006061 0.0075 0.2275 537409 outcome TRUE reported 11514_196_CD59_CD59
CD59 chr1 196695521 0.00826 8.735e-27 35329 TRUE reported textfile
2 1 TRUE
5816 rs4756046 A C A C 0.092 0.0073 0.08105 NA FALSE FALSE FALSE
GCST006061 0.013 0.5728 537409 outcome TRUE reported 11514_196_CD59_CD59
CD59 chr11 33821025 0.014961 7.773e-10 35357 TRUE reported
textfile 2 1 TRUE
5817 rs77924615 A G A G -0.0737 0.0136 0.19701 NA FALSE FALSE FALSE
GCST006061 0.0092 0.1375 537409 outcome TRUE reported 11514_196_CD59_CD59
CD59 chr16 20381010 0.010252 6.538e-13 35346 TRUE reported
textfile 2 1 TRUE
5818 rs831630 T C T C -0.1828 0.0029 0.32663 NA FALSE FALSE FALSE
GCST006061 0.0076 0.7043 537409 outcome TRUE reported 11514_196_CD59_CD59
CD59 chr11 33721473 0.008567 5.102e-101 35356 TRUE reported
textfile 2 1 TRUE
5819 rs9992101 A G A G 0.046 0.0224 0.39832 NA FALSE FALSE FALSE
GCST006061 0.0075 0.00273 537409 outcome TRUE reported 11514_196_CD59_CD59
CD59 chr4 76439278 0.008253 2.492e-08 35347 TRUE reported
textfile 2 1 TRUE
5820 rs10836317 A G A G 0.0735 -0.0045 0.34309 NA FALSE FALSE FALSE
GCST006061 0.0077 0.5585 537409 outcome TRUE reported
14076_74_CST4_Cystatin_S CST4 chr11 34863975 0.008554 8.541e-18 35378
TRUE reported textfile 2 1 TRUE
5821 rs111257030 T C T C -0.4363 -0.0223 0.08651 NA FALSE FALSE FALSE
GCST006061 0.0124 0.07195 537409 outcome TRUE reported
14076_74_CST4_Cystatin_S CST4 chr20 23692416 0.014418 1e-200 35363
TRUE reported textfile 2 1 TRUE
5822 rs145979311 C T C T -0.1378 0.0114 0.03232 NA FALSE FALSE FALSE
GCST006061 0.0273 0.6767 537409 outcome TRUE reported
14076_74_CST4_Cystatin_S CST4 chr20 35749478 0.023796 6.998e-09 35366
TRUE reported textfile 2 1 TRUE
5823 rs1998082 C T C T -0.0999 -0.0162 0.06787 NA FALSE FALSE FALSE
GCST006061 0.0141 0.2498 537409 outcome TRUE reported
14076_74_CST4_Cystatin_S CST4 chr20 23077041 0.016342 9.771e-10 35368
TRUE reported textfile 2 1 TRUE
5824 rs34223888 T C T C 0.1713 -0.0468 0.0209 NA FALSE FALSE FALSE
GCST006061 0.0299 0.1177 537409 outcome TRUE reported
14076_74_CST4_Cystatin_S CST4 chr4 70050252 0.028006 9.559e-10 35357
TRUE reported textfile 2 1 TRUE
5825 rs3749751 T C T C -0.1586 0.0026 0.38867 NA FALSE FALSE FALSE
GCST006061 0.0073 0.7172 537409 outcome TRUE reported
14076_74_CST4_Cystatin_S CST4 chr5 135173987 0.008357 2.562e-80 35360
TRUE reported textfile 2 1 TRUE
5826 rs6113759 T C T C -0.1118 -0.0088 0.06049 NA FALSE FALSE FALSE
GCST006061 0.0188 0.6372 537409 outcome TRUE reported
14076_74_CST4_Cystatin_S CST4 chr20 22670023 0.017297 1.022e-10 35369
TRUE reported textfile 2 1 TRUE
5827 rs6138249 T G T G -0.1451 -0.0012 0.06306 NA FALSE FALSE FALSE
GCST006061 0.0149 0.9337 537409 outcome TRUE reported
14076_74_CST4_Cystatin_S CST4 chr20 24250886 0.016933 1.045e-17 35366
TRUE reported textfile 2 1 TRUE

5828 rs61880808 G A G A -0.065 -0.0089 0.17811 NA FALSE FALSE FALSE
GCST006061 0.0094 0.3433 537409 outcome TRUE reported
14076_74_CST4_Cystatin_S CST4 chr11 34393829 0.010632 9.751e-10 35369
TRUE reported textfile 2 1 TRUE
5829 rs62216860 A G A G -0.34 -0.1424 0.01234 NA FALSE FALSE FALSE
GCST006061 0.0426 0.0008408 537409 outcome TRUE reported
14076_74_CST4_Cystatin_S CST4 chr20 24695146 0.038501 1.038e-18 35366
TRUE reported textfile 2 1 TRUE
5830 rs6427943 A G A G -0.1049 0.0238 0.4103 NA FALSE FALSE FALSE
GCST006061 0.0073 0.001097 537409 outcome TRUE reported
14076_74_CST4_Cystatin_S CST4 chr1 202217817 0.008287 9.983e-37 35361
TRUE reported textfile 2 1 TRUE
5831 rs67020211 G T G T -0.0736 1e-04 0.4901 NA FALSE FALSE FALSE
GCST006061 0.0073 0.9882 537409 outcome TRUE reported
14076_74_CST4_Cystatin_S CST4 chr15 63347445 0.008126 1.336e-19 35362
TRUE reported textfile 2 1 TRUE
5832 rs10836486 T G T G 0.0497 0.0133 0.35858 NA FALSE FALSE FALSE
GCST006061 0.0075 0.07835 537409 outcome TRUE reported 3448_13_INSR_IR INSR
chr11 36058225 0.008699 1.109e-08 35368 TRUE reported textfile 2
1 TRUE
5833 rs10901256 T C T C -0.1642 -0.0015 0.28085 NA FALSE FALSE FALSE
GCST006061 0.008 0.8548 537409 outcome TRUE reported 3448_13_INSR_IR INSR
chr9 133287875 0.009504 7.045e-67 35370 TRUE reported textfile 2
1 TRUE
5834 rs10962668 C T C T 0.0683 0.0314 0.39835 NA FALSE FALSE FALSE
GCST006061 0.0073 1.743e-05 537409 outcome TRUE reported 3448_13_INSR_IR
INSR chr9 16894142 0.008731 5.166e-15 35365 TRUE reported
textfile 2 1 TRUE
5835 rs11605349 C T C T 0.1133 0.0065 0.08574 NA FALSE FALSE FALSE
GCST006061 0.0124 0.6026 537409 outcome TRUE reported 3448_13_INSR_IR INSR
chr11 126435860 0.014669 1.13e-14 35371 TRUE reported textfile 2
1 TRUE
5836 rs143501783 C T C T 0.2376 -0.0333 0.01116 NA FALSE FALSE FALSE
GCST006061 0.0443 0.4525 537409 outcome TRUE reported 3448_13_INSR_IR INSR
chr17 7897001 0.038125 4.6e-10 35373 TRUE reported textfile 2 1 TRUE
5837 rs150960915 G A G A -0.226 -0.0149 0.0149 NA FALSE FALSE FALSE
GCST006061 0.0474 0.7531 537409 outcome TRUE reported 3448_13_INSR_IR INSR
chr1 161604637 0.034462 5.454e-11 35351 TRUE reported textfile 2
1 TRUE
5838 rs17710008 A G A G 0.0736 0.0084 0.16527 NA FALSE FALSE FALSE
GCST006061 0.0096 0.3817 537409 outcome TRUE reported 3448_13_INSR_IR INSR
chr6 152721900 0.011221 5.405e-11 35377 TRUE reported textfile 2
1 TRUE
5839 rs2502742 T G T G -0.0601 0.0034 0.49931 NA FALSE FALSE FALSE
GCST006061 0.0072 0.6413 537409 outcome TRUE reported 3448_13_INSR_IR INSR
chr9 133697989 0.008622 3.157e-12 35369 TRUE reported textfile 2
1 TRUE
5840 rs387057 T C T C -0.0763 0.0124 0.3718 NA FALSE FALSE FALSE
GCST006061 0.0075 0.09678 537409 outcome TRUE reported 3448_13_INSR_IR INSR
chr19 16301309 0.008732 2.365e-18 35363 TRUE reported textfile 2
1 TRUE
5841 rs4458292 G T G T -0.0891 -0.0076 0.25155 NA FALSE FALSE FALSE
GCST006061 0.0147 0.6065 537409 outcome TRUE reported 3448_13_INSR_IR INSR
chr21 13894619 0.010369 8.499e-18 35271 TRUE reported textfile 2
1 TRUE
5842 rs4804368 G A G A 0.0715 -0.0096 0.3392 NA FALSE FALSE FALSE
GCST006061 0.0074 0.1933 537409 outcome TRUE reported 3448_13_INSR_IR INSR
chr19 7190279 0.008858 6.92e-16 35374 TRUE reported textfile 2 1
TRUE
5843 rs55714927 T C T C 0.0828 1e-04 0.21166 NA FALSE FALSE FALSE
GCST006061 0.0098 0.9926 537409 outcome TRUE reported 3448_13_INSR_IR INSR
chr17 7176997 0.010321 1.039e-15 35370 TRUE reported textfile 2 1
TRUE
5844 rs58745116 A G A G -0.0848 0.0099 0.42203 NA FALSE FALSE FALSE
GCST006061 0.0075 0.1874 537409 outcome TRUE reported 3448_13_INSR_IR INSR
chr17 35476573 0.008506 2.077e-23 35366 TRUE reported textfile 2

1 TRUE
5845 rs7080169 A G A G 0.056 0.0211 0.43914 NA FALSE FALSE FALSE
GCST006061 0.0074 0.004466 537409 outcome TRUE reported 3448_13_INSR_IR
INSR chr10 80472950 0.008465 3.701e-11 35370 TRUE reported
textfile 2 1 TRUE
5846 rs760462 C T C T 0.1931 -0.0203 0.23025 NA FALSE FALSE FALSE
GCST006061 0.0095 0.03196 537409 outcome TRUE reported 3448_13_INSR_IR INSR
chr21 44908184 0.009974 1.67e-83 35259 TRUE reported textfile 2
1 TRUE
5847 rs9936992 G A G A -0.1005 2e-04 0.35128 NA FALSE FALSE FALSE
GCST006061 0.0074 0.9732 537409 outcome TRUE reported 3448_13_INSR_IR INSR
chr16 31398790 0.008825 4.768e-30 35364 TRUE reported textfile 2
1 TRUE
5848 rs10838389 C T C T 0.0612 -0.0026 0.2121 NA FALSE FALSE FALSE
GCST006061 0.0088 0.7676 537409 outcome TRUE reported 16300_4_TREM2_TREM2
TREM2 chr11 44923073 0.00989 6.085e-10 35362 TRUE reported textfile
2 1 TRUE
5849 rs11038540 A G A G 0.0891 -0.0062 0.19148 NA FALSE FALSE FALSE
GCST006061 0.0091 0.4926 537409 outcome TRUE reported 16300_4_TREM2_TREM2
TREM2 chr11 45554263 0.010367 8.335e-18 35369 TRUE reported
textfile 2 1 TRUE
5850 rs111266377 T G T G 0.1363 0.037 0.02883 NA FALSE FALSE FALSE
GCST006061 0.0258 0.1519 537409 outcome TRUE reported 16300_4_TREM2_TREM2
TREM2 chr3 167239870 0.023443 6.093e-09 35348 TRUE reported
textfile 2 1 TRUE
5851 rs112474179 T C T C 0.1758 0.0782 0.02869 NA FALSE FALSE FALSE
GCST006061 0.036 0.03 537409 outcome TRUE reported 16300_4_TREM2_TREM2
TREM2 chr11 59493198 0.023984 2.305e-13 35366 TRUE reported
textfile 2 1 TRUE
5852 rs114431690 A G A G -0.1878 0.0134 0.03974 NA FALSE FALSE FALSE
GCST006061 0.0233 0.5645 537409 outcome TRUE reported 16300_4_TREM2_TREM2
TREM2 chr6 41760329 0.020547 6.251e-20 35361 TRUE reported
textfile 2 1 TRUE
5853 rs116319373 G A G A -0.3687 -0.0102 0.02207 NA FALSE FALSE FALSE
GCST006061 0.0332 0.7588 537409 outcome TRUE reported 16300_4_TREM2_TREM2
TREM2 chr6 39981247 0.027357 2.119e-41 35356 TRUE reported
textfile 2 1 TRUE
5854 rs117327140 T C T C -0.1791 -0.0312 0.03797 NA FALSE FALSE FALSE
GCST006061 0.0267 0.2426 537409 outcome TRUE reported 16300_4_TREM2_TREM2
TREM2 chr11 60516607 0.020855 8.859e-18 35363 TRUE reported
textfile 2 1 TRUE
5855 rs138508553 T C T C -0.3657 -0.0208 0.01668 NA FALSE FALSE FALSE
GCST006061 0.0497 0.6758 537409 outcome TRUE reported 16300_4_TREM2_TREM2
TREM2 chr11 60327946 0.031718 9.338e-31 35368 TRUE reported
textfile 2 1 TRUE
5856 rs140378210 G A G A -0.3519 -0.0333 0.01948 NA FALSE FALSE FALSE
GCST006061 0.0415 0.4228 537409 outcome TRUE reported 16300_4_TREM2_TREM2
TREM2 chr6 39624613 0.027983 2.884e-36 35355 TRUE reported
textfile 2 1 TRUE
5857 rs141393619 T C T C -0.2114 -0.0394 0.01136 NA FALSE FALSE FALSE
GCST006061 0.047 0.402 537409 outcome TRUE reported 16300_4_TREM2_TREM2
TREM2 chr11 60690095 0.037141 1.257e-08 35361 TRUE reported
textfile 2 1 TRUE
5858 rs143329063 C T C T -0.2424 -0.0629 0.01559 NA FALSE FALSE FALSE
GCST006061 0.0486 0.1958 537409 outcome TRUE reported 16300_4_TREM2_TREM2
TREM2 chr6 44913727 0.032757 1.362e-13 35358 TRUE reported
textfile 2 1 TRUE
5859 rs144782411 T G T G -0.3022 -0.0076 0.02148 NA FALSE FALSE FALSE
GCST006061 0.0455 0.8673 537409 outcome TRUE reported 16300_4_TREM2_TREM2
TREM2 chr6 38859146 0.027649 8.297e-28 35355 TRUE reported
textfile 2 1 TRUE
5860 rs146936090 T C T C -0.6281 0.0612 0.01673 NA FALSE FALSE FALSE
GCST006061 0.0559 0.2733 537409 outcome TRUE reported 16300_4_TREM2_TREM2
TREM2 chr6 41433547 0.031473 1.311e-88 35358 TRUE reported
textfile 2 1 TRUE
5861 rs147931202 C T C T -0.2826 0.0122 0.01529 NA FALSE FALSE FALSE

GCST006061 0.0333 0.714 537409 outcome TRUE reported 16300_4_TREM2_TREM2
TREM2 chr11 60976134 0.033129 1.459e-17 35362 TRUE reported
textfile 2 1 TRUE
5862 rs149069407 T G T G 0.1534 -0.0201 0.05374 NA FALSE FALSE FALSE
GCST006061 0.0221 0.362 537409 outcome TRUE reported 16300_4_TREM2_TREM2
TREM2 chr11 60690967 0.017983 1.46e-17 35361 TRUE reported
textfile 2 1 TRUE
5863 rs150271427 C A C A -0.4223 0.1118 0.02575 NA FALSE FALSE FALSE
GCST006061 0.0403 0.005585 537409 outcome TRUE reported 16300_4_TREM2_TREM2
TREM2 chr11 60426702 0.025194 4.643e-63 35368 TRUE reported
textfile 2 1 TRUE
5864 rs188904277 A G A G -1.1852 6e-04 0.01016 NA FALSE FALSE FALSE
GCST006061 0.0552 0.9914 537409 outcome TRUE reported 16300_4_TREM2_TREM2
TREM2 chr6 41053109 0.038561 1e-200 35366 TRUE reported textfile
2 1 TRUE
5865 rs261290 C T C T -0.0546 0.0145 0.36352 NA FALSE FALSE FALSE
GCST006061 0.0074 0.05053 537409 outcome TRUE reported 16300_4_TREM2_TREM2
TREM2 chr15 58386521 0.008178 2.447e-11 35358 TRUE reported
textfile 2 1 TRUE
5866 rs432157 T C T C 0.0719 0.0089 0.13042 NA FALSE FALSE FALSE
GCST006061 0.011 0.419 537409 outcome TRUE reported 16300_4_TREM2_TREM2
TREM2 chr19 46711154 0.011723 8.622e-10 35366 TRUE reported
textfile 2 1 TRUE
5867 rs55756397 A G A G 0.1788 0.034 0.01852 NA FALSE FALSE FALSE
GCST006061 0.0305 0.2649 537409 outcome TRUE reported 16300_4_TREM2_TREM2
TREM2 chr11 60518091 0.029773 1.908e-09 35363 TRUE reported
textfile 2 1 TRUE
5868 rs62414730 A G A G -0.5113 -0.0174 0.01348 NA FALSE FALSE FALSE
GCST006061 0.0328 0.5962 537409 outcome TRUE reported 16300_4_TREM2_TREM2
TREM2 chr6 42203020 0.034353 4.198e-50 35360 TRUE reported
textfile 2 1 TRUE
5869 rs72912764 A G A G -0.3569 0.0166 0.02589 NA FALSE FALSE FALSE
GCST006061 0.0344 0.6294 537409 outcome TRUE reported 16300_4_TREM2_TREM2
TREM2 chr11 59975502 0.025143 9.828e-46 35362 TRUE reported
textfile 2 1 TRUE
5870 rs74730442 A G A G -0.2236 -0.0196 0.02359 NA FALSE FALSE FALSE
GCST006061 0.0226 0.3845 537409 outcome TRUE reported 16300_4_TREM2_TREM2
TREM2 chr6 44041534 0.02618 1.334e-17 35358 TRUE reported textfile
2 1 TRUE
5871 rs77705101 G A G A -0.5257 -0.0128 0.02453 NA FALSE FALSE FALSE
GCST006061 0.0188 0.4967 537409 outcome TRUE reported 16300_4_TREM2_TREM2
TREM2 chr6 40794563 0.025903 1.434e-91 35360 TRUE reported
textfile 2 1 TRUE
5872 rs77790943 A G A G -0.1317 -0.0037 0.085 NA FALSE FALSE FALSE
GCST006061 0.0163 0.8222 537409 outcome TRUE reported 16300_4_TREM2_TREM2
TREM2 chr6 41103202 0.014186 1.639e-20 35366 TRUE reported
textfile 2 1 TRUE
5873 rs78053849 T C T C -0.1511 -0.0104 0.03595 NA FALSE FALSE FALSE
GCST006061 0.0265 0.6944 537409 outcome TRUE reported 16300_4_TREM2_TREM2
TREM2 chr11 61392099 0.022274 1.171e-11 35359 TRUE reported
textfile 2 1 TRUE
5874 rs79289342 C T C T -0.2404 -0.0264 0.01319 NA FALSE FALSE FALSE
GCST006061 0.0328 0.4207 537409 outcome TRUE reported 16300_4_TREM2_TREM2
TREM2 chr6 51282858 0.035387 1.095e-11 35365 TRUE reported
textfile 2 1 TRUE
5875 rs9939224 G T G T 0.062 0.0125 0.2164 NA FALSE FALSE FALSE
GCST006061 0.0089 0.1583 537409 outcome TRUE reported 16300_4_TREM2_TREM2
TREM2 chr16 56968820 0.009571 9.303e-11 35363 TRUE reported
textfile 2 1 TRUE
5876 rs1354034 C T C T 0.0713 0.0088 0.38119 NA FALSE FALSE FALSE
GCST006061 0.0073 0.2248 537409 outcome TRUE reported 7898_29_MDM1_MDM1
MDM1 chr3 56815721 0.008095 1.276e-18 35356 TRUE reported
textfile 2 1 TRUE
5877 rs2294081 C T C T 0.0552 0.0074 0.47719 NA FALSE FALSE FALSE
GCST006061 0.0091 0.4156 537409 outcome TRUE reported 7898_29_MDM1_MDM1
MDM1 chr11 193863 0.008526 9.508e-11 35352 TRUE reported textfile

2 1 TRUE
5878 rs45488900 T G T G -0.0718 0.0081 0.10749 NA FALSE FALSE FALSE
GCST006061 0.0116 0.4843 537409 outcome TRUE reported 7898_29_MDM1_MDM1
MDM1 chr2 219251787 0.012806 2.061e-08 35369 TRUE reported
textfile 2 1 TRUE
5879 rs10842789 T G T G -0.0521 7e-04 0.35198 NA FALSE FALSE FALSE
GCST006061 0.0077 0.9266 537409 outcome TRUE reported 19488_1_PDCD6_PDCD6
PDCD6 chr12 9057603 0.008474 7.852e-10 35387 TRUE reported textfile
2 1 TRUE
5880 rs1707652 T C T C 0.0566 0.0081 0.3102 NA FALSE FALSE FALSE
GCST006061 0.0074 0.2701 537409 outcome TRUE reported 19488_1_PDCD6_PDCD6
PDCD6 chr3 165761011 0.00871 8.125e-11 35339 TRUE reported textfile
2 1 TRUE
5881 rs34511054 C A C A -0.1843 -0.0025 0.05685 NA FALSE FALSE FALSE
GCST006061 0.018 0.8877 537409 outcome TRUE reported 19488_1_PDCD6_PDCD6
PDCD6 chr5 264041 0.01771 2.321e-25 35291 TRUE reported textfile 2
1 TRUE
5882 rs607268 C T C T -0.0959 0.0088 0.33888 NA FALSE FALSE FALSE
GCST006061 0.0075 0.2411 537409 outcome TRUE reported 19488_1_PDCD6_PDCD6
PDCD6 chr1 56913999 0.00848 1.193e-29 35369 TRUE reported textfile
2 1 TRUE
5883 rs62346174 A C A C 0.1433 -0.029 0.04249 NA FALSE FALSE FALSE
GCST006061 0.0172 0.09126 537409 outcome TRUE reported 19488_1_PDCD6_PDCD6
PDCD6 chr5 296193 0.020548 3.081e-12 35291 TRUE reported textfile
2 1 TRUE
5884 rs72705167 T C T C -0.3896 0.0554 0.01099 NA FALSE FALSE FALSE
GCST006061 0.0393 0.1584 537409 outcome TRUE reported 19488_1_PDCD6_PDCD6
PDCD6 chr5 988097 0.039947 1.793e-22 35283 TRUE reported textfile
2 1 TRUE
5885 rs10843188 G A G A -0.0725 -0.0019 0.34507 NA FALSE FALSE FALSE
GCST006061 0.0077 0.8039 537409 outcome TRUE reported 9578_263_MANSC4_MANS4
MANSC4 chr12 28504200 0.008705 8.196e-17 35356 TRUE reported
textfile 2 1 TRUE
5886 rs11049245 G A G A 0.1413 0.0112 0.09094 NA FALSE FALSE FALSE
GCST006061 0.0116 0.3346 537409 outcome TRUE reported 9578_263_MANSC4_MANS4
MANSC4 chr12 27973346 0.014393 9.489e-23 35353 TRUE reported
textfile 2 1 TRUE
5887 rs145142574 T C T C 0.1795 -0.1154 0.01529 NA FALSE FALSE FALSE
GCST006061 0.1238 0.3511 537409 outcome TRUE reported 9578_263_MANSC4_MANS4
MANSC4 chr12 39209959 0.032719 4.11e-08 35350 TRUE reported
textfile 2 1 TRUE
5888 rs203688 A G A G -0.0524 -0.012 0.39961 NA FALSE FALSE FALSE
GCST006061 0.0075 0.1106 537409 outcome TRUE reported 9578_263_MANSC4_MANS4
MANSC4 chr1 196703255 0.00827 2.358e-10 35339 TRUE reported textfile
2 1 TRUE
5889 rs34793466 C T C T 0.0628 0.0074 0.16907 NA FALSE FALSE FALSE
GCST006061 0.0121 0.5398 537409 outcome TRUE reported 9578_263_MANSC4_MANS4
MANSC4 chr14 106540249 0.011029 1.241e-08 34966 TRUE reported
textfile 2 1 TRUE
5890 rs4439576 G A G A 0.0536 -0.011 0.25598 NA FALSE FALSE FALSE
GCST006061 0.0079 0.1622 537409 outcome TRUE reported 9578_263_MANSC4_MANS4
MANSC4 chr12 42682379 0.009494 1.643e-08 35358 TRUE reported
textfile 2 1 TRUE
5891 rs7307323 C T C T -0.0963 -0.0121 0.29098 NA FALSE FALSE FALSE
GCST006061 0.0083 0.1413 537409 outcome TRUE reported 9578_263_MANSC4_MANS4
MANSC4 chr12 28959043 0.009069 2.451e-26 35355 TRUE reported
textfile 2 1 TRUE
5892 rs73294067 G T G T 0.2662 -0.0192 0.02456 NA FALSE FALSE FALSE
GCST006061 0.0259 0.4582 537409 outcome TRUE reported 9578_263_MANSC4_MANS4
MANSC4 chr12 27646075 0.027193 1.252e-22 35354 TRUE reported
textfile 2 1 TRUE
5893 rs10843403 C T C T 0.0723 -0.0063 0.36436 NA FALSE FALSE FALSE
GCST006061 0.0073 0.384 537409 outcome TRUE reported 4437_56_ENTPD5_ENTP5
ENTPD5 chr12 29387376 0.008377 6.12e-18 35359 TRUE reported
textfile 2 1 TRUE
5894 rs10922098 T C T C -0.0828 -0.009 0.40011 NA FALSE FALSE FALSE

GCST006061 0.0075 0.2275 537409 outcome TRUE reported 4437_56_ENTPD5_ENTP5
ENTPD5 chr1 196695521 0.008272 1.383e-23 35338 TRUE reported
textfile 2 1 TRUE
5895 rs112109204 T C T C -0.2036 0.0131 0.11029 NA FALSE FALSE FALSE
GCST006061 0.0206 0.5267 537409 outcome TRUE reported 4437_56_ENTPD5_ENTP5
ENTPD5 chr14 73635053 0.013042 6.128e-55 35351 TRUE reported
textfile 2 1 TRUE
5896 rs112963922 T C T C 0.2364 0.0542 0.01119 NA FALSE FALSE FALSE
GCST006061 0.0399 0.1741 537409 outcome TRUE reported 4437_56_ENTPD5_ENTP5
ENTPD5 chr14 94411531 0.038663 9.693e-10 35371 TRUE reported
textfile 2 1 TRUE
5897 rs12433287 C T C T 0.115 0.011 0.04754 NA FALSE FALSE FALSE
GCST006061 0.0215 0.6094 537409 outcome TRUE reported 4437_56_ENTPD5_ENTP5
ENTPD5 chr14 75230677 0.019388 3.004e-09 35347 TRUE reported
textfile 2 1 TRUE
5898 rs145561224 A G A G -0.3595 -0.1042 0.01761 NA FALSE FALSE FALSE
GCST006061 0.0684 0.1277 537409 outcome TRUE reported 4437_56_ENTPD5_ENTP5
ENTPD5 chr14 72603150 0.032118 4.415e-29 35352 TRUE reported
textfile 2 1 TRUE
5899 rs149062332 C T C T 0.7654 0.0424 0.02045 NA FALSE FALSE FALSE
GCST006061 0.0435 0.33 537409 outcome TRUE reported 4437_56_ENTPD5_ENTP5
ENTPD5 chr3 186623236 0.02766 1.511e-168 35367 TRUE reported textfile
2 1 TRUE
5900 rs150185151 C T C T 0.2929 0.0717 0.01427 NA FALSE FALSE FALSE
GCST006061 0.0521 0.1689 537409 outcome TRUE reported 4437_56_ENTPD5_ENTP5
ENTPD5 chr14 73805057 0.034056 7.938e-18 35351 TRUE reported
textfile 2 1 TRUE
5901 rs150243994 G A G A 0.174 0.0226 0.03126 NA FALSE FALSE FALSE
GCST006061 0.0293 0.4403 537409 outcome TRUE reported 4437_56_ENTPD5_ENTP5
ENTPD5 chr14 73127057 0.023474 1.241e-13 35352 TRUE reported
textfile 2 1 TRUE
5902 rs16860640 C T C T -0.0713 0.0118 0.10753 NA FALSE FALSE FALSE
GCST006061 0.012 0.3285 537409 outcome TRUE reported 4437_56_ENTPD5_ENTP5
ENTPD5 chr3 186252384 0.013018 4.329e-08 35358 TRUE reported
textfile 2 1 TRUE
5903 rs1707652 T C T C 0.0579 0.0081 0.3102 NA FALSE FALSE FALSE
GCST006061 0.0074 0.2701 537409 outcome TRUE reported 4437_56_ENTPD5_ENTP5
ENTPD5 chr3 165761011 0.008745 3.567e-11 35339 TRUE reported
textfile 2 1 TRUE
5904 rs2244608 G A G A 0.0705 -0.0109 0.31579 NA FALSE FALSE FALSE
GCST006061 0.0076 0.1491 537409 outcome TRUE reported 4437_56_ENTPD5_ENTP5
ENTPD5 chr12 120979185 0.008722 6.322e-16 35365 TRUE reported
textfile 2 1 TRUE
5905 rs28458356 A G A G -0.4189 0.0022 0.11237 NA FALSE FALSE FALSE
GCST006061 0.0111 0.8438 537409 outcome TRUE reported 4437_56_ENTPD5_ENTP5
ENTPD5 chr14 73831862 0.012961 1e-200 35351 TRUE reported textfile
2 1 TRUE
5906 rs33951980 T C T C 0.0708 -0.0097 0.12207 NA FALSE FALSE FALSE
GCST006061 0.0109 0.3705 537409 outcome TRUE reported 4437_56_ENTPD5_ENTP5
ENTPD5 chr7 73615107 0.012325 9.229e-09 35354 TRUE reported
textfile 2 1 TRUE
5907 rs4806498 C T C T -0.0455 -0.0037 0.40548 NA FALSE FALSE FALSE
GCST006061 0.0074 0.6205 537409 outcome TRUE reported 4437_56_ENTPD5_ENTP5
ENTPD5 chr19 54171009 0.008283 3.955e-08 35371 TRUE reported
textfile 2 1 TRUE
5908 rs55663055 A C A C 0.0923 0.0149 0.17033 NA FALSE FALSE FALSE
GCST006061 0.0112 0.1856 537409 outcome TRUE reported 4437_56_ENTPD5_ENTP5
ENTPD5 chr3 186426705 0.010736 8.139e-18 35366 TRUE reported
textfile 2 1 TRUE
5909 rs7236989 C T C T -0.054 -0.0046 0.37181 NA FALSE FALSE FALSE
GCST006061 0.0073 0.5285 537409 outcome TRUE reported 4437_56_ENTPD5_ENTP5
ENTPD5 chr18 59343221 0.008385 1.196e-10 35367 TRUE reported
textfile 2 1 TRUE
5910 rs8178824 T C T C 0.2883 -0.0854 0.01066 NA FALSE FALSE FALSE
GCST006061 0.023 0.0002035 537409 outcome TRUE reported
4437_56_ENTPD5_ENTP5 ENTPD5 chr17 66228657 0.039053 1.555e-13 35369

TRUE reported textfile 2 1 TRUE
5911 rs9302635 C T C T 0.0659 0.0086 0.19843 NA FALSE FALSE FALSE
GCST006061 0.0095 0.367 537409 outcome TRUE reported 4437_56_ENTPD5_ENTP5
ENTPD5 chr16 72110275 0.010046 5.377e-11 35375 TRUE reported
textfile 2 1 TRUE
5912 rs10845139 A G A G -0.0985 -0.0065 0.11302 NA FALSE FALSE FALSE
GCST006061 0.0113 0.5637 537409 outcome TRUE reported 13939_14_UGP2_UGPA1
UGP2 chr12 7427058 0.012607 5.58e-15 35389 TRUE reported textfile
2 1 TRUE
5913 rs11046893 T C T C 0.0788 0.015 0.38897 NA FALSE FALSE FALSE
GCST006061 0.0076 0.05011 537409 outcome TRUE reported 13939_14_UGP2_UGPA1
UGP2 chr12 7250980 0.008124 3.011e-22 35390 TRUE reported textfile
2 1 TRUE
5914 rs71309976 T C T C 0.1701 -0.0352 0.01647 NA FALSE FALSE FALSE
GCST006061 0.0567 0.5346 537409 outcome TRUE reported 13939_14_UGP2_UGPA1
UGP2 chr3 56694053 0.030643 2.84e-08 35359 TRUE reported
textfile 2 1 TRUE
5915 rs10846742 A G A G -0.0657 0.0168 0.12656 NA FALSE FALSE FALSE
GCST006061 0.0097 0.08316 537409 outcome TRUE reported 9017_58_LCT_LPH LCT
chr12 124824136 0.011317 6.422e-09 35380 TRUE reported textfile 2
1 TRUE
5916 rs11195909 G A G A -0.0434 0.0058 0.33515 NA FALSE FALSE FALSE
GCST006061 0.0076 0.4454 537409 outcome TRUE reported 9017_58_LCT_LPH LCT
chr10 112349749 0.007901 3.955e-08 35373 TRUE reported textfile 2
1 TRUE
5917 rs11564538 A G A G -0.0967 -0.0348 0.04928 NA FALSE FALSE FALSE
GCST006061 0.0165 0.03549 537409 outcome TRUE reported 9017_58_LCT_LPH LCT
chr19 48098255 0.017721 4.851e-08 35375 TRUE reported textfile 2
1 TRUE
5918 rs12614226 G T G T -0.4809 -0.0017 0.01899 NA FALSE FALSE FALSE
GCST006061 0.0221 0.9377 537409 outcome TRUE reported 9017_58_LCT_LPH LCT
chr2 134834976 0.03041 2.489e-56 35392 TRUE reported textfile 2 1
TRUE
5919 rs13426918 G A G A -0.4444 0.0293 0.03443 NA FALSE FALSE FALSE
GCST006061 0.016 0.06704 537409 outcome TRUE reported 9017_58_LCT_LPH LCT
chr2 136278219 0.022222 5.737e-89 35387 TRUE reported textfile 2
1 TRUE
5920 rs139040889 G T G T -0.3149 -0.0064 0.01173 NA FALSE FALSE FALSE
GCST006061 0.0328 0.8452 537409 outcome TRUE reported 9017_58_LCT_LPH LCT
chr2 137219098 0.037446 4.116e-17 35392 TRUE reported textfile 2
1 TRUE
5921 rs141079659 G A G A -0.1044 0.0337 0.04316 NA FALSE FALSE FALSE
GCST006061 0.03 0.2617 537409 outcome TRUE reported 9017_58_LCT_LPH LCT
chr19 47394619 0.018469 1.58e-08 35368 TRUE reported textfile 2
1 TRUE
5922 rs150543594 T C T C -0.2246 0.0485 0.01327 NA FALSE FALSE FALSE
GCST006061 0.0471 0.3027 537409 outcome TRUE reported 9017_58_LCT_LPH LCT
chr2 140013745 0.034816 1.111e-10 35354 TRUE reported textfile 2
1 TRUE
5923 rs2216812 T C T C 0.1232 -0.0017 0.02837 NA FALSE FALSE FALSE
GCST006061 0.0198 0.9327 537409 outcome TRUE reported 9017_58_LCT_LPH LCT
chr18 32143828 0.022484 4.268e-08 35348 TRUE reported textfile 2
1 TRUE
5924 rs368565 T C T C 0.2554 -0.0092 0.35428 NA FALSE FALSE FALSE
GCST006061 0.0076 0.2281 537409 outcome TRUE reported 9017_58_LCT_LPH LCT
chr19 48697960 0.007838 1e-200 35377 TRUE reported textfile 2 1
TRUE
5925 rs41307428 T C T C 0.1952 0.0291 0.01987 NA FALSE FALSE FALSE
GCST006061 0.023 0.206 537409 outcome TRUE reported 9017_58_LCT_LPH LCT
chr9 133471682 0.026731 2.827e-13 35369 TRUE reported textfile 2
1 TRUE
5926 rs479980 A C A C -0.2876 -0.0073 0.41407 NA FALSE FALSE FALSE
GCST006061 0.0077 0.3427 537409 outcome TRUE reported 9017_58_LCT_LPH LCT
chr2 134526467 0.008234 1e-200 35385 TRUE reported textfile 2 1
TRUE
5927 rs616114 T C T C 0.16 0.01 0.39746 NA FALSE FALSE FALSE

GCST006061 0.0073 0.1691 537409 outcome TRUE reported 9017_58_LCT_LPH LCT chr18 32217958 0.00762 6.958e-98 35350 TRUE reported textfile 2 1 TRUE

5928 rs635634 C T C T 0.2159 0.0285 0.12995 NA FALSE FALSE FALSE GCST006061 0.0089 0.001351 537409 outcome TRUE reported 9017_58_LCT_LPH LCT chr9 133279427 0.011119 5.474e-84 35371 TRUE reported textfile 2 1 TRUE

5929 rs77854115 C A C A -0.886 -0.0559 0.01295 NA FALSE FALSE FALSE GCST006061 0.0371 0.1318 537409 outcome TRUE reported 9017_58_LCT_LPH LCT chr2 135459099 0.035343 1.091e-138 35390 TRUE reported textfile 2 1 TRUE

5930 rs78918822 T C T C -0.905 0.0386 0.01168 NA FALSE FALSE FALSE GCST006061 0.026 0.1368 537409 outcome TRUE reported 9017_58_LCT_LPH LCT chr2 136107122 0.038871 6.701e-120 35392 TRUE reported textfile 2 1 TRUE

5931 rs10849448 G A G A -0.0532 0.0056 0.28422 NA FALSE FALSE FALSE GCST006061 0.009 0.532 537409 outcome TRUE reported 3487_32_CXCL13_BLC CXCL13 chr12 6384185 0.008893 2.197e-09 35380 TRUE reported textfile 2 1 TRUE

5932 rs12166583 A C A C -0.0747 0.0019 0.26524 NA FALSE FALSE FALSE GCST006061 0.0085 0.826 537409 outcome TRUE reported 3487_32_CXCL13_BLC CXCL13 chr22 30087975 0.009109 2.391e-16 35366 TRUE reported textfile 2 1 TRUE

5933 rs10849448 G A G A 0.0461 0.0056 0.28422 NA FALSE FALSE FALSE GCST006061 0.009 0.532 537409 outcome TRUE reported 5487_7_SLAMF7_SLAF7 SLAMF7 chr12 6384185 0.008272 2.506e-08 35380 TRUE reported textfile 2 1 TRUE

5934 rs11160704 G A G A -0.0656 -0.0132 0.15284 NA FALSE FALSE FALSE GCST006061 0.0102 0.1959 537409 outcome TRUE reported 5487_7_SLAMF7_SLAF7 SLAMF7 chr14 102815003 0.010436 3.265e-10 35375 TRUE reported textfile 2 1 TRUE

5935 rs115877710 T C T C -0.5047 -0.0238 0.02935 NA FALSE FALSE FALSE GCST006061 0.0354 0.5009 537409 outcome TRUE reported 5487_7_SLAMF7_SLAF7 SLAMF7 chr1 161817286 0.025557 8.365e-87 35361 TRUE reported textfile 2 1 TRUE

5936 rs116205652 A G A G -0.4181 -0.0045 0.03593 NA FALSE FALSE FALSE GCST006061 0.0279 0.8712 537409 outcome TRUE reported 5487_7_SLAMF7_SLAF7 SLAMF7 chr1 161689669 0.023171 8.749e-73 35351 TRUE reported textfile 2 1 TRUE

5937 rs11710456 A G A G -0.0902 -0.0045 0.28702 NA FALSE FALSE FALSE GCST006061 0.0078 0.5685 537409 outcome TRUE reported 5487_7_SLAMF7_SLAF7 SLAMF7 chr3 187008099 0.008287 1.372e-27 35363 TRUE reported textfile 2 1 TRUE

5938 rs12079579 A G A G 0.1434 0.0037 0.06927 NA FALSE FALSE FALSE GCST006061 0.0124 0.7655 537409 outcome TRUE reported 5487_7_SLAMF7_SLAF7 SLAMF7 chr1 161910692 0.016884 2.006e-17 35361 TRUE reported textfile 2 1 TRUE

5939 rs13297246 A G A G 0.0605 -0.0063 0.17783 NA FALSE FALSE FALSE GCST006061 0.0098 0.5199 537409 outcome TRUE reported 5487_7_SLAMF7_SLAF7 SLAMF7 chr9 33128619 0.009736 5.163e-10 35349 TRUE reported textfile 2 1 TRUE

5940 rs1448187 C T C T -0.0578 -0.0041 0.29843 NA FALSE FALSE FALSE GCST006061 0.0097 0.6721 537409 outcome TRUE reported 5487_7_SLAMF7_SLAF7 SLAMF7 chr2 111462782 0.00826 2.602e-12 35345 TRUE reported textfile 2 1 TRUE

5941 rs1512226 G A G A -0.0416 0.0045 0.47251 NA FALSE FALSE FALSE GCST006061 0.0072 0.534 537409 outcome TRUE reported 5487_7_SLAMF7_SLAF7 SLAMF7 chr2 60324666 0.007438 2.234e-08 35346 TRUE reported textfile 2 1 TRUE

5942 rs16830188 T C T C -0.3205 0.0318 0.01152 NA FALSE FALSE FALSE GCST006061 0.0224 0.1559 537409 outcome TRUE reported 5487_7_SLAMF7_SLAF7 SLAMF7 chr1 24972350 0.041125 6.526e-15 35371 TRUE reported textfile 2 1 TRUE

5943 rs190841461 T C T C 0.2791 0.0277 0.01172 NA FALSE FALSE FALSE GCST006061 0.053 0.6011 537409 outcome TRUE reported 5487_7_SLAMF7_SLAF7 SLAMF7 chr1 161968366 0.039125 9.788e-13 35360 TRUE reported

textfile 2 1 TRUE
5944 rs2067583 C T C T 0.05 -0.003 0.22673 NA FALSE FALSE FALSE
GCST006061 0.0088 0.7347 537409 outcome TRUE reported 5487_7_SLAMF7_SLAF7
SLAMF7 chr9 114849783 0.008939 2.227e-08 35362 TRUE reported
textfile 2 1 TRUE
5945 rs34158391 C A C A -0.3431 0.015 0.21871 NA FALSE FALSE FALSE
GCST006061 0.0085 0.07603 537409 outcome TRUE reported 5487_7_SLAMF7_SLAF7
SLAMF7 chr1 160737276 0.010311 1e-200 35360 TRUE reported textfile
2 1 TRUE
5946 rs352686 A G A G -0.2835 -0.0014 0.47225 NA FALSE FALSE FALSE
GCST006061 0.008 0.86 537409 outcome TRUE reported 5487_7_SLAMF7_SLAF7
SLAMF7 chr1 160713760 0.008505 1e-200 35352 TRUE reported textfile
2 1 TRUE
5947 rs3807307 C T C T 0.0462 -0.0074 0.45902 NA FALSE FALSE FALSE
GCST006061 0.0072 0.3034 537409 outcome TRUE reported 5487_7_SLAMF7_SLAF7
SLAMF7 chr7 128939148 0.007477 6.466e-10 35351 TRUE reported
textfile 2 1 TRUE
5948 rs5997579 C T C T -0.063 -0.003 0.42299 NA FALSE FALSE FALSE
GCST006061 0.0072 0.6758 537409 outcome TRUE reported 5487_7_SLAMF7_SLAF7
SLAMF7 chr22 30141537 0.007551 7.258e-17 35366 TRUE reported
textfile 2 1 TRUE
5949 rs61764072 C A C A 0.1911 -0.0676 0.01124 NA FALSE FALSE FALSE
GCST006061 0.0411 0.1003 537409 outcome TRUE reported 5487_7_SLAMF7_SLAF7
SLAMF7 chr17 7849205 0.034009 1.92e-08 35374 TRUE reported textfile
2 1 TRUE
5950 rs62447171 A G A G -0.0591 3e-04 0.20053 NA FALSE FALSE FALSE
GCST006061 0.008 0.9667 537409 outcome TRUE reported 5487_7_SLAMF7_SLAF7
SLAMF7 chr7 50270294 0.009289 1.989e-10 35337 TRUE reported
textfile 2 1 TRUE
5951 rs6580642 C T C T -0.0613 -0.0208 0.14153 NA FALSE FALSE FALSE
GCST006061 0.0107 0.05235 537409 outcome TRUE reported 5487_7_SLAMF7_SLAF7
SLAMF7 chr12 47876813 0.01067 9.197e-09 35355 TRUE reported textfile
2 1 TRUE
5952 rs75213764 T C T C -0.333 0.0048 0.14523 NA FALSE FALSE FALSE
GCST006061 0.0102 0.6364 537409 outcome TRUE reported 5487_7_SLAMF7_SLAF7
SLAMF7 chr1 161265807 0.012218 1.458e-163 35358 TRUE reported
textfile 2 1 TRUE
5953 rs7754000 A G A G -0.0652 0.0092 0.15419 NA FALSE FALSE FALSE
GCST006061 0.0273 0.7369 537409 outcome TRUE reported 5487_7_SLAMF7_SLAF7
SLAMF7 chr6 303017 0.010941 2.538e-09 35308 TRUE reported textfile
2 1 TRUE
5954 rs9901675 A G A G 0.0793 -0.0272 0.07646 NA FALSE FALSE FALSE
GCST006061 0.0161 0.0905 537409 outcome TRUE reported 5487_7_SLAMF7_SLAF7
SLAMF7 chr17 7581494 0.014048 1.654e-08 35379 TRUE reported textfile
2 1 TRUE
5955 rs10849546 A G A G -0.0962 -0.0036 0.11875 NA FALSE FALSE FALSE
GCST006061 0.0105 0.7312 537409 outcome TRUE reported
14115_34_ADM_Adrenomedullin ADM chr12 7068900 0.012672 3.154e-14 35389 TRUE
reported textfile 2 1 TRUE
5956 rs2304456 G T G T -0.1801 0.0072 0.12203 NA FALSE FALSE FALSE
GCST006061 0.0114 0.5273 537409 outcome TRUE reported
14115_34_ADM_Adrenomedullin ADM chr3 186727263 0.012266 8.374e-49 35368
TRUE reported textfile 2 1 TRUE
5957 rs2731672 C T C T 0.0688 -0.0025 0.25436 NA FALSE FALSE FALSE
GCST006061 0.0083 0.7666 537409 outcome TRUE reported
14115_34_ADM_Adrenomedullin ADM chr5 177415473 0.009302 1.401e-13 35362
TRUE reported textfile 2 1 TRUE
5958 rs10849546 A G A G -0.0909 -0.0036 0.11875 NA FALSE FALSE FALSE
GCST006061 0.0105 0.7312 537409 outcome TRUE reported 14100_63_C1QC_C1QC
C1QC chr12 7068900 0.01287 1.631e-12 35389 TRUE reported textfile 2
1 TRUE
5959 rs144444914 A C A C 0.4429 -0.0208 0.01134 NA FALSE FALSE FALSE
GCST006061 0.0291 0.4754 537409 outcome TRUE reported 14100_63_C1QC_C1QC
C1QC chr1 22554246 0.039416 2.696e-29 35369 TRUE reported
textfile 2 1 TRUE
5960 rs184316561 T C T C -0.1332 -0.0737 0.03635 NA FALSE FALSE FALSE

GCST006061 0.0318 0.02031 537409 outcome TRUE reported 14100_63_C1QC_C1QC
C1QC chr1 64759626 0.022534 3.401e-09 35343 TRUE reported
textfile 2 1 TRUE
5961 rs2744700 T C T C -0.0887 -0.0072 0.4644 NA FALSE FALSE FALSE
GCST006061 0.0072 0.3193 537409 outcome TRUE reported 14100_63_C1QC_C1QC
C1QC chr1 22171881 0.008514 2.043e-25 35373 TRUE reported
textfile 2 1 TRUE
5962 rs292004 A G A G -0.2517 -0.004 0.0752 NA FALSE FALSE FALSE
GCST006061 0.0174 0.8192 537409 outcome TRUE reported 14100_63_C1QC_C1QC
C1QC chr1 22667221 0.015932 3.208e-56 35369 TRUE reported
textfile 2 1 TRUE
5963 rs2987530 C T C T 0.0626 0.0124 0.17832 NA FALSE FALSE FALSE
GCST006061 0.0096 0.1946 537409 outcome TRUE reported 14100_63_C1QC_C1QC
C1QC chr13 49033278 0.010748 5.737e-09 35363 TRUE reported
textfile 2 1 TRUE
5964 rs41266007 T C T C -0.1682 -0.0199 0.04003 NA FALSE FALSE FALSE
GCST006061 0.0281 0.4794 537409 outcome TRUE reported 14100_63_C1QC_C1QC
C1QC chr1 21842832 0.021472 4.741e-15 35375 TRUE reported
textfile 2 1 TRUE
5965 rs4934470 C T C T 0.1365 -0.0225 0.26361 NA FALSE FALSE FALSE
GCST006061 0.0085 0.008204 537409 outcome TRUE reported 14100_63_C1QC_C1QC
C1QC chr10 89307681 0.009307 1.049e-48 35355 TRUE reported
textfile 2 1 TRUE
5966 rs6532770 A C A C -0.0824 -0.0031 0.24489 NA FALSE FALSE FALSE
GCST006061 0.0083 0.7144 537409 outcome TRUE reported 14100_63_C1QC_C1QC
C1QC chr4 98862518 0.009555 6.457e-18 35344 TRUE reported
textfile 2 1 TRUE
5967 rs77482787 T G T G -0.2793 -0.0419 0.02309 NA FALSE FALSE FALSE
GCST006061 0.0329 0.2026 537409 outcome TRUE reported 14100_63_C1QC_C1QC
C1QC chr1 22616057 0.027989 1.888e-23 35369 TRUE reported
textfile 2 1 TRUE
5968 rs10849546 A G A G -0.1708 -0.0036 0.11875 NA FALSE FALSE FALSE
GCST006061 0.0105 0.7312 537409 outcome TRUE reported 9251_28_MAN2B2_MA2B2
MAN2B2 chr12 7068900 0.013426 4.497e-37 35389 TRUE reported textfile
2 1 TRUE
5969 rs10922098 T C T C -0.086 -0.009 0.40011 NA FALSE FALSE FALSE
GCST006061 0.0075 0.2275 537409 outcome TRUE reported 9251_28_MAN2B2_MA2B2
MAN2B2 chr1 196695521 0.008709 5.333e-23 35338 TRUE reported
textfile 2 1 TRUE
5970 rs11155297 T G T G -0.1301 7e-04 0.2561 NA FALSE FALSE FALSE
GCST006061 0.0085 0.931 537409 outcome TRUE reported 9251_28_MAN2B2_MA2B2
MAN2B2 chr6 143503967 0.009787 2.542e-40 35356 TRUE reported
textfile 2 1 TRUE
5971 rs4277850 A G A G -0.0934 2e-04 0.12791 NA FALSE FALSE FALSE
GCST006061 0.0132 0.9877 537409 outcome TRUE reported 9251_28_MAN2B2_MA2B2
MAN2B2 chr4 6456946 0.013409 3.276e-12 35377 TRUE reported textfile
2 1 TRUE
5972 rs4346716 C T C T -0.3595 -0.0127 0.43283 NA FALSE FALSE FALSE
GCST006061 0.0075 0.09244 537409 outcome TRUE reported 9251_28_MAN2B2_MA2B2
MAN2B2 chr4 6561722 0.006773 1e-200 35372 TRUE reported textfile 2
1 TRUE
5973 rs483465 G A G A 0.0584 -0.0204 0.24663 NA FALSE FALSE FALSE
GCST006061 0.0083 0.01415 537409 outcome TRUE reported 9251_28_MAN2B2_MA2B2
MAN2B2 chr3 136329135 0.009942 4.252e-09 35361 TRUE reported
textfile 2 1 TRUE
5974 rs57410633 C A C A 0.1874 -0.0069 0.03189 NA FALSE FALSE FALSE
GCST006061 0.0172 0.6879 537409 outcome TRUE reported 9251_28_MAN2B2_MA2B2
MAN2B2 chr4 6476965 0.025533 2.144e-13 35377 TRUE reported textfile
2 1 TRUE
5975 rs62297711 G A G A 0.1258 -0.0112 0.03963 NA FALSE FALSE FALSE
GCST006061 0.0197 0.5719 537409 outcome TRUE reported 9251_28_MAN2B2_MA2B2
MAN2B2 chr4 5817062 0.022856 3.711e-08 35376 TRUE reported textfile
2 1 TRUE
5976 rs67321746 T C T C -0.1223 -0.0161 0.08 NA FALSE FALSE FALSE
GCST006061 0.0143 0.2609 537409 outcome TRUE reported 9251_28_MAN2B2_MA2B2
MAN2B2 chr4 6944000 0.016373 8.04e-14 35381 TRUE reported textfile

2 1 TRUE
5977 rs73199928 A G A G 0.3277 0.0391 0.02229 NA FALSE FALSE FALSE GCST006061 0.0417 0.3482 537409 outcome TRUE reported 9251_28_MAN2B2_MA2B2 MAN2B2 chr4 6873486 0.030714 1.415e-26 35376 TRUE reported textfile 2 1 TRUE
5978 rs73213615 A G A G 0.1855 -0.0204 0.16692 NA FALSE FALSE FALSE GCST006061 0.0091 0.02563 537409 outcome TRUE reported 9251_28_MAN2B2_MA2B2 MAN2B2 chr4 6743075 0.011954 2.612e-54 35378 TRUE reported textfile 2 1 TRUE
5979 rs79493386 T G T G 0.4935 0.0635 0.02701 NA FALSE FALSE FALSE GCST006061 0.0279 0.023 537409 outcome TRUE reported 9251_28_MAN2B2_MA2B2 MAN2B2 chr4 6574712 0.027703 5.519e-71 35375 TRUE reported textfile 2 1 TRUE
5980 rs10876168 T C T C 0.0998 0.0066 0.36623 NA FALSE FALSE FALSE GCST006061 0.0075 0.3747 537409 outcome TRUE reported 8397_147_QSOX2_QSOX2 QSOX2 chr12 51386852 0.008095 6.336e-35 35370 TRUE reported textfile 2 1 TRUE
5981 rs111589804 A G A G -0.3295 0.0176 0.0106 NA FALSE FALSE FALSE GCST006061 0.0301 0.5598 537409 outcome TRUE reported 8397_147_QSOX2_QSOX2 QSOX2 chr9 135522294 0.041895 3.694e-15 35303 TRUE reported textfile 2 1 TRUE
5982 rs1179011 G A G A 0.2817 -8.00E-04 0.33449 NA FALSE FALSE FALSE GCST006061 0.0077 0.9187 537409 outcome TRUE reported 8397_147_QSOX2_QSOX2 QSOX2 chr9 133319548 0.00918 1e-200 35371 TRUE reported textfile 2 1 TRUE
5983 rs139562936 T G T G 0.3638 -0.0301 0.0211 NA FALSE FALSE FALSE GCST006061 0.034 0.3765 537409 outcome TRUE reported 8397_147_QSOX2_QSOX2 QSOX2 chr9 134215558 0.030464 7.148e-33 35372 TRUE reported textfile 2 1 TRUE
5984 rs17476364 C T C T 0.0729 -1.00E-04 0.09492 NA FALSE FALSE FALSE GCST006061 0.0127 0.9964 537409 outcome TRUE reported 8397_147_QSOX2_QSOX2 QSOX2 chr10 69334748 0.013138 2.876e-08 35359 TRUE reported textfile 2 1 TRUE
5985 rs1886296 G A G A -0.2962 -0.0359 0.29991 NA FALSE FALSE FALSE GCST006061 0.0078 4.119e-06 537409 outcome TRUE reported 8397_147_QSOX2_QSOX2 QSOX2 chr9 136200434 0.009508 1e-200 35266 TRUE reported textfile 2 1 TRUE
5986 rs2923444 T C T C 0.0619 -0.0083 0.3596 NA FALSE FALSE FALSE GCST006061 0.0074 0.2577 537409 outcome TRUE reported 8397_147_QSOX2_QSOX2 QSOX2 chr8 42541861 0.008082 1.88e-14 35353 TRUE reported textfile 2 1 TRUE
5987 rs34130413 A G A G -0.0728 -0.0053 0.17818 NA FALSE FALSE FALSE GCST006061 0.0095 0.5756 537409 outcome TRUE reported 8397_147_QSOX2_QSOX2 QSOX2 chr2 62083167 0.010097 5.6e-13 35353 TRUE reported textfile 2 1 TRUE
5988 rs61858556 A C A C -0.0679 0.01 0.16504 NA FALSE FALSE FALSE GCST006061 0.0105 0.3396 537409 outcome TRUE reported 8397_147_QSOX2_QSOX2 QSOX2 chr10 96580719 0.010405 6.774e-11 35368 TRUE reported textfile 2 1 TRUE
5989 rs6511291 C T C T 0.0476 -0.0056 0.45866 NA FALSE FALSE FALSE GCST006061 0.0073 0.4461 537409 outcome TRUE reported 8397_147_QSOX2_QSOX2 QSOX2 chr19 21767600 0.007767 8.888e-10 35356 TRUE reported textfile 2 1 TRUE
5990 rs7044834 A C A C -0.1367 0.0152 0.18881 NA FALSE FALSE FALSE GCST006061 0.0109 0.1627 537409 outcome TRUE reported 8397_147_QSOX2_QSOX2 QSOX2 chr9 133165512 0.011141 1.319e-34 35366 TRUE reported textfile 2 1 TRUE
5991 rs76518888 C A C A 0.4204 0.0073 0.01702 NA FALSE FALSE FALSE GCST006061 0.0321 0.8212 537409 outcome TRUE reported 8397_147_QSOX2_QSOX2 QSOX2 chr9 132657117 0.033541 4.867e-36 35373 TRUE reported textfile 2 1 TRUE
5992 rs7849542 A G A G 0.3219 -0.0038 0.29202 NA FALSE FALSE FALSE GCST006061 0.0081 0.6378 537409 outcome TRUE reported 8397_147_QSOX2_QSOX2 QSOX2 chr9 133167143 0.009525 1e-200 35364 TRUE reported textfile 2 1 TRUE
5993 rs8887 C T C T -0.0445 0.0035 0.39121 NA FALSE FALSE FALSE GCST006061

0.0075 0.6388 537409 outcome TRUE reported 8397_147_QSOX2_QSOX2 QSOX2
chr19 4502189 0.007965 2.312e-08 35355 TRUE reported textfile 2 1
TRUE
5994 rs9392385 G A G A -0.0454 0.01 0.4049 NA FALSE FALSE FALSE
GCST006061 0.0073 0.1729 537409 outcome TRUE reported 8397_147_QSOX2_QSOX2
QSOX2 chr6 2399924 0.007891 8.75e-09 35335 TRUE reported textfile
2 1 TRUE
5995 rs10849939 A G A G -0.0913 0.0057 0.27096 NA FALSE FALSE FALSE
GCST006061 0.009 0.527 537409 outcome TRUE reported 18381_16_ALDH2_ALDH_E2
ALDH2 chr12 111373800 0.009144 1.77e-23 35370 TRUE reported
textfile 2 1 TRUE
5996 rs116859940 A G A G -0.2259 0.006 0.01164 NA FALSE FALSE FALSE
GCST006061 0.0609 0.9216 537409 outcome TRUE reported 18381_16_ALDH2_ALDH_E2
ALDH2 chr12 108295526 0.036833 8.616e-10 35374 TRUE reported
textfile 2 1 TRUE
5997 rs13030345 T G T G 0.0654 0.0092 0.14944 NA FALSE FALSE FALSE
GCST006061 0.0093 0.3221 537409 outcome TRUE reported 18381_16_ALDH2_ALDH_E2
ALDH2 chr2 27780307 0.011267 6.449e-09 35371 TRUE reported
textfile 2 1 TRUE
5998 rs4835265 A C A C 0.0764 -0.0033 0.16265 NA FALSE FALSE FALSE
GCST006061 0.0094 0.7227 537409 outcome TRUE reported 18381_16_ALDH2_ALDH_E2
ALDH2 chr4 145900258 0.010975 3.369e-12 35360 TRUE reported
textfile 2 1 TRUE
5999 rs4940691 G T G T -0.0533 0.0041 0.22 NA FALSE FALSE FALSE
GCST006061 0.0088 0.6399 537409 outcome TRUE reported 18381_16_ALDH2_ALDH_E2
ALDH2 chr18 58428146 0.009775 4.955e-08 35367 TRUE reported
textfile 2 1 TRUE
6000 rs58118359 A G A G -0.1571 0.0271 0.02298 NA FALSE FALSE FALSE
GCST006061 0.026 0.2975 537409 outcome TRUE reported 18381_16_ALDH2_ALDH_E2
ALDH2 chr12 109822046 0.026917 5.333e-09 35367 TRUE reported
textfile 2 1 TRUE
6001 rs7519043 T C T C 0.0572 0.0013 0.43222 NA FALSE FALSE FALSE
GCST006061 0.0073 0.8569 537409 outcome TRUE reported 18381_16_ALDH2_ALDH_E2
ALDH2 chr1 16174028 0.008214 3.312e-12 35386 TRUE reported
textfile 2 1 TRUE
6002 rs17349460 G A G A -0.2493 -0.0332 0.0443 NA FALSE FALSE FALSE
GCST006061 0.0193 0.085 537409 outcome TRUE reported 4154_57_SELP_P_Selectin
SELP chr1 169184350 0.020206 5.668e-35 35363 TRUE reported
textfile 2 1 TRUE
6003 rs635634 C T C T 0.2302 0.0285 0.12995 NA FALSE FALSE FALSE
GCST006061 0.0089 0.001351 537409 outcome TRUE reported
4154_57_SELP_P_Selectin SELP chr9 133279427 0.012073 4.671e-81 35371
TRUE reported textfile 2 1 TRUE
6004 rs655029 A G A G 0.0518 -0.0036 0.28471 NA FALSE FALSE FALSE
GCST006061 0.0082 0.6598 537409 outcome TRUE reported 4154_57_SELP_P_Selectin
SELP chr2 31254972 0.009087 1.197e-08 35369 TRUE reported
textfile 2 1 TRUE
6005 rs9332575 C T C T -0.3856 -0.0099 0.12705 NA FALSE FALSE FALSE
GCST006061 0.0125 0.4284 537409 outcome TRUE reported 4154_57_SELP_P_Selectin
SELP chr1 169557863 0.012213 1e-200 35353 TRUE reported textfile
2 1 TRUE
6006 rs13342837 T C T C 0.321 3e-04 0.32419 NA FALSE FALSE FALSE
GCST006061 0.0083 0.9726 537409 outcome TRUE reported 5129_12_SCARF1_SREC_I
SCARF1 chr17 1628679 0.008955 1e-200 35356 TRUE reported textfile 2
1 TRUE
6007 rs141910927 C T C T -0.115 -0.0061 0.07529 NA FALSE FALSE FALSE
GCST006061 0.0218 0.7808 537409 outcome TRUE reported 5129_12_SCARF1_SREC_I
SCARF1 chr17 1595133 0.016349 2.003e-12 35350 TRUE reported textfile
2 1 TRUE
6008 rs35340377 A G A G -0.0532 0.0075 0.25094 NA FALSE FALSE FALSE
GCST006061 0.0082 0.3585 537409 outcome TRUE reported 5129_12_SCARF1_SREC_I
SCARF1 chr1 247874908 0.009741 4.721e-08 35156 TRUE reported
textfile 2 1 TRUE
6009 rs75179845 C T C T 0.1022 0.0169 0.06486 NA FALSE FALSE FALSE
GCST006061 0.0134 0.2077 537409 outcome TRUE reported 5129_12_SCARF1_SREC_I
SCARF1 chr9 133257567 0.017062 2.099e-09 35371 TRUE reported

textfile 2 1 TRUE
6010 rs75695549 C T C T 0.104 0.0023 0.06222 NA FALSE FALSE FALSE
GCST006061 0.0174 0.8934 537409 outcome TRUE reported 5129_12_SCARF1_SREC_I
SCARF1 chr17 2370433 0.017374 2.153e-09 35364 TRUE reported textfile
2 1 TRUE
6011 rs10853015 C A C A 0.0462 -0.0189 0.21747 NA FALSE FALSE FALSE
GCST006061 0.0084 0.02452 537409 outcome TRUE reported 6247_9_SIRPB1_SIRB1
SIRPB1 chr17 59700978 0.00835 3.145e-08 35370 TRUE reported textfile
2 1 TRUE
6012 rs113484100 C A C A -0.1818 -0.0063 0.02643 NA FALSE FALSE FALSE
GCST006061 0.0225 0.779 537409 outcome TRUE reported 6247_9_SIRPB1_SIRB1
SIRPB1 chr20 2065937 0.028089 9.649e-11 35377 TRUE reported textfile
2 1 TRUE
6013 rs117149764 C T C T -0.3423 0.0144 0.01745 NA FALSE FALSE FALSE
GCST006061 0.0393 0.714 537409 outcome TRUE reported 6247_9_SIRPB1_SIRB1
SIRPB1 chr20 2044443 0.03354 1.869e-24 35375 TRUE reported textfile 2
1 TRUE
6014 rs117245515 T C T C 0.2608 -0.058 0.02172 NA FALSE FALSE FALSE
GCST006061 0.0309 0.06039 537409 outcome TRUE reported 6247_9_SIRPB1_SIRB1
SIRPB1 chr20 2676365 0.030915 3.285e-17 35380 TRUE reported textfile
2 1 TRUE
6015 rs117284274 T G T G -0.8836 0.0119 0.04172 NA FALSE FALSE FALSE
GCST006061 0.0224 0.5966 537409 outcome TRUE reported 6247_9_SIRPB1_SIRB1
SIRPB1 chr20 1616224 0.013227 1e-200 35374 TRUE reported textfile 2
1 TRUE
6016 rs17791824 T C T C 0.8653 0.0493 0.03152 NA FALSE FALSE FALSE
GCST006061 0.0322 0.1262 537409 outcome TRUE reported 6247_9_SIRPB1_SIRB1
SIRPB1 chr20 1376802 0.025077 1e-200 35373 TRUE reported textfile 2
1 TRUE
6017 rs183218916 G A G A 0.2136 -0.0577 0.04467 NA FALSE FALSE FALSE
GCST006061 0.0254 0.02302 537409 outcome TRUE reported 6247_9_SIRPB1_SIRB1
SIRPB1 chr20 964702 0.02172 8.009e-23 35368 TRUE reported textfile 2
1 TRUE
6018 rs62187494 A G A G 0.5229 0.0037 0.05528 NA FALSE FALSE FALSE
GCST006061 0.0194 0.8508 537409 outcome TRUE reported 6247_9_SIRPB1_SIRB1
SIRPB1 chr20 1291036 0.019934 1.156e-151 35373 TRUE reported textfile
2 1 TRUE
6019 rs73081790 A G A G -0.4147 -0.0148 0.14203 NA FALSE FALSE FALSE
GCST006061 0.011 0.1774 537409 outcome TRUE reported 6247_9_SIRPB1_SIRB1
SIRPB1 chr20 1405421 0.012675 1e-200 35373 TRUE reported textfile 2
1 TRUE
6020 rs76137931 A G A G 0.2261 0.0302 0.02837 NA FALSE FALSE FALSE
GCST006061 0.0295 0.3067 537409 outcome TRUE reported 6247_9_SIRPB1_SIRB1
SIRPB1 chr20 734129 0.027099 7.214e-17 35361 TRUE reported textfile
2 1 TRUE
6021 rs79039084 A G A G 0.3388 0.0771 0.02432 NA FALSE FALSE FALSE
GCST006061 0.0444 0.08266 537409 outcome TRUE reported 6247_9_SIRPB1_SIRB1
SIRPB1 chr20 1629294 0.029077 2.25e-31 35374 TRUE reported textfile
2 1 TRUE
6022 rs10853721 G A G A -0.0787 0.0188 0.12994 NA FALSE FALSE FALSE
GCST006061 0.0107 0.08021 537409 outcome TRUE reported 12662_82_ECH1_ECH1
ECH1 chr19 38821856 0.011774 2.32e-11 35366 TRUE reported
textfile 2 1 TRUE
6023 rs4802890 A G A G 0.4475 -8.00E-04 0.08781 NA FALSE FALSE FALSE
GCST006061 0.0115 0.9431 537409 outcome TRUE reported 12662_82_ECH1_ECH1
ECH1 chr19 38813762 0.013784 1e-200 35366 TRUE reported textfile
2 1 TRUE
6024 rs73544031 C T C T 0.2006 0.0117 0.01294 NA FALSE FALSE FALSE
GCST006061 0.0275 0.6717 537409 outcome TRUE reported 12662_82_ECH1_ECH1
ECH1 chr19 39280627 0.035106 1.103e-08 35359 TRUE reported
textfile 2 1 TRUE
6025 rs10858824 G T G T 0.0857 0.0179 0.19935 NA FALSE FALSE FALSE
GCST006061 0.0087 0.03925 537409 outcome TRUE reported 5630_48_CD300A_CM35H
CD300A chr12 89089498 0.01045 2.381e-16 35356 TRUE reported textfile
2 1 TRUE
6026 rs10858852 C T C T -0.2861 0.0079 0.25558 NA FALSE FALSE FALSE

GCST006061 0.0087 0.3604 537409 outcome TRUE reported 5630_48_CD300A_CM35H
CD300A chr12 89439561 0.009393 1e-200 35357 TRUE reported textfile
2 1 TRUE
6027 rs11495145 T C T C 0.1715 -0.0701 0.01886 NA FALSE FALSE FALSE
GCST006061 0.0421 0.09555 537409 outcome TRUE reported 5630_48_CD300A_CM35H
CD300A chr12 91298527 0.030114 1.233e-08 35358 TRUE reported
textfile 2 1 TRUE
6028 rs11571364 A G A G 0.1261 -2.00E-04 0.04967 NA FALSE FALSE FALSE
GCST006061 0.0193 0.9922 537409 outcome TRUE reported 5630_48_CD300A_CM35H
CD300A chr17 7008372 0.019364 7.41e-11 35374 TRUE reported textfile
2 1 TRUE
6029 rs115931 T C T C -0.0507 -0.0065 0.49749 NA FALSE FALSE FALSE
GCST006061 0.0072 0.3657 537409 outcome TRUE reported 5630_48_CD300A_CM35H
CD300A chr3 98750945 0.008189 5.975e-10 35331 TRUE reported
textfile 2 1 TRUE
6030 rs117162506 A G A G -0.3234 -0.0405 0.02242 NA FALSE FALSE FALSE
GCST006061 0.0276 0.1426 537409 outcome TRUE reported 5630_48_CD300A_CM35H
CD300A chr17 75176080 0.028783 2.722e-29 35381 TRUE reported
textfile 2 1 TRUE
6031 rs11888817 C T C T 0.1122 0.0137 0.32877 NA FALSE FALSE FALSE
GCST006061 0.0077 0.07488 537409 outcome TRUE reported 5630_48_CD300A_CM35H
CD300A chr2 62303845 0.008697 4.472e-38 35352 TRUE reported
textfile 2 1 TRUE
6032 rs12601859 T C T C 0.0561 -0.0047 0.28919 NA FALSE FALSE FALSE
GCST006061 0.0082 0.5664 537409 outcome TRUE reported 5630_48_CD300A_CM35H
CD300A chr17 6836215 0.00944 2.798e-09 35370 TRUE reported textfile 2
1 TRUE
6033 rs1320040 C T C T -0.1159 0.0057 0.22043 NA FALSE FALSE FALSE
GCST006061 0.0087 0.5064 537409 outcome TRUE reported 5630_48_CD300A_CM35H
CD300A chr17 74374262 0.010306 2.416e-29 35372 TRUE reported
textfile 2 1 TRUE
6034 rs13205436 C T C T 0.0808 -0.0345 0.10488 NA FALSE FALSE FALSE
GCST006061 0.013 0.008041 537409 outcome TRUE reported
5630_48_CD300A_CM35H CD300A chr6 95586174 0.013329 1.344e-09 35343
TRUE reported textfile 2 1 TRUE
6035 rs1838095 T C T C 0.0477 0.0015 0.34532 NA FALSE FALSE FALSE
GCST006061 0.0075 0.839 537409 outcome TRUE reported 5630_48_CD300A_CM35H
CD300A chr11 60379874 0.008636 3.322e-08 35374 TRUE reported
textfile 2 1 TRUE
6036 rs1996710 G A G A -0.0641 0.0175 0.18788 NA FALSE FALSE FALSE
GCST006061 0.0098 0.0757 537409 outcome TRUE reported 5630_48_CD300A_CM35H
CD300A chr17 74865023 0.010916 4.299e-09 35378 TRUE reported
textfile 2 1 TRUE
6037 rs2706525 A G A G -0.5624 -0.0163 0.17963 NA FALSE FALSE FALSE
GCST006061 0.0096 0.08738 537409 outcome TRUE reported 5630_48_CD300A_CM35H
CD300A chr17 74449620 0.007948 1e-200 35377 TRUE reported textfile
2 1 TRUE
6038 rs2722224 G A G A -0.1057 0.0205 0.446 NA FALSE FALSE FALSE
GCST006061 0.0073 0.004698 537409 outcome TRUE reported
5630_48_CD300A_CM35H CD300A chr12 89837533 0.008369 1.439e-36 35357
TRUE reported textfile 2 1 TRUE
6039 rs59728170 A G A G 0.3654 0.0033 0.06257 NA FALSE FALSE FALSE
GCST006061 0.0137 0.8114 537409 outcome TRUE reported 5630_48_CD300A_CM35H
CD300A chr17 74569905 0.017389 4.914e-98 35380 TRUE reported
textfile 2 1 TRUE
6040 rs62624498 A G A G 0.1453 -0.006 0.03051 NA FALSE FALSE FALSE
GCST006061 0.0272 0.825 537409 outcome TRUE reported 5630_48_CD300A_CM35H
CD300A chr17 76000464 0.02468 3.924e-09 35382 TRUE reported textfile
2 1 TRUE
6041 rs6501688 G A G A 0.0953 -0.001 0.10525 NA FALSE FALSE FALSE
GCST006061 0.0118 0.9346 537409 outcome TRUE reported 5630_48_CD300A_CM35H
CD300A chr17 74055879 0.013995 9.781e-12 35371 TRUE reported
textfile 2 1 TRUE
6042 rs7225955 C T C T -0.4124 0.0149 0.01359 NA FALSE FALSE FALSE
GCST006061 0.0211 0.4786 537409 outcome TRUE reported 5630_48_CD300A_CM35H
CD300A chr17 74434720 0.036586 1.805e-29 35374 TRUE reported

textfile 2 1 TRUE
6043 rs9797243 G A G A -0.0732 -0.0145 0.26437 NA FALSE FALSE FALSE GCST006061 0.0082 0.07818 537409 outcome TRUE reported 5630_48_CD300A_CM35H CD300A chr17 74266521 0.009635 3.024e-14 35380 TRUE reported textfile 2 1 TRUE
6044 rs10858851 T C T C 0.0984 0.0082 0.25242 NA FALSE FALSE FALSE GCST006061 0.0088 0.3488 537409 outcome TRUE reported 3710_49_PLG_Angiostatin PLG chr12 89437093 0.009498 3.788e-25 35357 TRUE reported textfile 2 1 TRUE
6045 rs11751347 T C T C -0.3754 0.0519 0.07953 NA FALSE FALSE FALSE GCST006061 0.0129 6.02e-05 537409 outcome TRUE reported 3710_49_PLG_Angiostatin PLG chr6 160671406 0.015346 3.678e-132 35388 TRUE reported textfile 2 1 TRUE
6046 rs1260326 C T C T -0.0699 -0.0069 0.34132 NA FALSE FALSE FALSE GCST006061 0.0073 0.3401 537409 outcome TRUE reported 3710_49_PLG_Angiostatin PLG chr2 27508073 0.008766 1.541e-15 35373 TRUE reported textfile 2 1 TRUE
6047 rs142126734 A G A G 0.2504 -0.0113 0.03808 NA FALSE FALSE FALSE GCST006061 0.0183 0.5366 537409 outcome TRUE reported 3710_49_PLG_Angiostatin PLG chr6 160521894 0.021957 3.983e-30 35390 TRUE reported textfile 2 1 TRUE
6048 rs17088106 A G A G 0.315 -0.0398 0.01234 NA FALSE FALSE FALSE GCST006061 0.0195 0.04114 537409 outcome TRUE reported 3710_49_PLG_Angiostatin PLG chr6 157092754 0.038582 3.229e-16 35364 TRUE reported textfile 2 1 TRUE
6049 rs34778552 A G A G 0.1989 0.0346 0.01912 NA FALSE FALSE FALSE GCST006061 0.0337 0.3037 537409 outcome TRUE reported 3710_49_PLG_Angiostatin PLG chr6 158036098 0.030498 6.949e-11 35365 TRUE reported textfile 2 1 TRUE
6050 rs9898 T C T C 0.0602 -0.0066 0.30049 NA FALSE FALSE FALSE GCST006061 0.0075 0.3765 537409 outcome TRUE reported 3710_49_PLG_Angiostatin PLG chr3 186672838 0.00899 2.135e-11 35368 TRUE reported textfile 2 1 TRUE
6051 rs10858853 T C T C -0.0481 0.0016 0.31807 NA FALSE FALSE FALSE GCST006061 0.0076 0.829 537409 outcome TRUE reported 6086_15_CHRDL2_CRDL2 CHRDL2 chr12 89441037 0.008732 3.623e-08 35357 TRUE reported textfile 2 1 TRUE
6052 rs117464057 C T C T -0.1449 -0.0043 0.02748 NA FALSE FALSE FALSE GCST006061 0.0328 0.896 537409 outcome TRUE reported 6086_15_CHRDL2_CRDL2 CHRDL2 chr11 74539707 0.024897 5.882e-09 35371 TRUE reported textfile 2 1 TRUE
6053 rs140198958 A G A G 0.1754 -0.0196 0.02427 NA FALSE FALSE FALSE GCST006061 0.0308 0.5246 537409 outcome TRUE reported 6086_15_CHRDL2_CRDL2 CHRDL2 chr11 74784531 0.025943 1.371e-11 35369 TRUE reported textfile 2 1 TRUE
6054 rs34695217 C T C T -0.6217 0.0311 0.05037 NA FALSE FALSE FALSE GCST006061 0.0194 0.1102 537409 outcome TRUE reported 6086_15_CHRDL2_CRDL2 CHRDL2 chr11 74702798 0.018367 1e-200 35363 TRUE reported textfile 2 1 TRUE
6055 rs6592590 T C T C 0.149 -0.0029 0.44399 NA FALSE FALSE FALSE GCST006061 0.0072 0.6881 537409 outcome TRUE reported 6086_15_CHRDL2_CRDL2 CHRDL2 chr11 74669984 0.008187 5.242e-74 35370 TRUE reported textfile 2 1 TRUE
6056 rs72837687 A G A G 0.0601 9e-04 0.21627 NA FALSE FALSE FALSE GCST006061 0.0097 0.9226 537409 outcome TRUE reported 6086_15_CHRDL2_CRDL2 CHRDL2 chr17 7170428 0.009902 1.283e-09 35370 TRUE reported textfile 2 1 TRUE
6057 rs80086566 C A C A -0.238 -0.0142 0.01833 NA FALSE FALSE FALSE GCST006061 0.0339 0.6755 537409 outcome TRUE reported 6086_15_CHRDL2_CRDL2 CHRDL2 chr11 74173358 0.030917 1.383e-14 35372 TRUE reported textfile 2 1 TRUE
6058 rs10860794 A C A C -0.2278 0.0049 0.27829 NA FALSE FALSE FALSE GCST006061 0.0078 0.526 537409 outcome TRUE reported 9370_69_GGH_GGH GGH chr12 101823942 0.009002 2.781e-141 35364 TRUE reported textfile 2 1 TRUE
6059 rs12547350 T C T C 0.1591 -0.0125 0.06507 NA FALSE FALSE FALSE GCST006061 0.0123 0.3088 537409 outcome TRUE reported 9370_69_GGH_GGH GGH

chr8 61296995 0.016471 4.49e-22 35367 TRUE reported textfile 2
1 TRUE
6060 rs12975366 C T C T -0.0818 -0.0089 0.4155 NA FALSE FALSE FALSE
GCST006061 0.0081 0.2688 537409 outcome TRUE reported 9370_69_GGH_GGH GGH
chr19 54255498 0.008142 9.512e-24 35370 TRUE reported textfile 2
1 TRUE
6061 rs147233090 T C T C 0.1615 0.0375 0.02693 NA FALSE FALSE FALSE
GCST006061 0.0254 0.1396 537409 outcome TRUE reported 9370_69_GGH_GGH GGH
chr15 43735849 0.024653 5.715e-11 35362 TRUE reported textfile 2
1 TRUE
6062 rs28654257 C T C T -0.0452 3e-04 0.41236 NA FALSE FALSE FALSE
GCST006061 0.0074 0.9677 537409 outcome TRUE reported 9370_69_GGH_GGH GGH
chr18 62491793 0.008169 3.146e-08 35367 TRUE reported textfile 2
1 TRUE
6063 rs4883263 C T C T -0.1803 0.003 0.03698 NA FALSE FALSE FALSE
GCST006061 0.0169 0.857 537409 outcome TRUE reported 9370_69_GGH_GGH GGH
chr12 7496888 0.021323 2.774e-17 35388 TRUE reported textfile 2 1
TRUE
6064 rs7314249 T C T C -0.0656 0.005 0.19044 NA FALSE FALSE FALSE
GCST006061 0.0089 0.5684 537409 outcome TRUE reported 9370_69_GGH_GGH GGH
chr12 7580515 0.01037 2.518e-10 35389 TRUE reported textfile 2 1 TRUE
6065 rs7459882 G A G A 0.0989 0.0054 0.4966 NA FALSE FALSE FALSE
GCST006061 0.0072 0.4567 537409 outcome TRUE reported 9370_69_GGH_GGH GGH
chr8 63298664 0.008136 5.358e-34 35342 TRUE reported textfile 2
1 TRUE
6066 rs75127467 C A C A -0.0639 -0.0078 0.25139 NA FALSE FALSE FALSE
GCST006061 0.0081 0.3323 537409 outcome TRUE reported 9370_69_GGH_GGH GGH
chr12 101947099 0.00931 6.721e-12 35363 TRUE reported textfile 2 1
TRUE
6067 rs76279395 G T G T 0.1149 -0.0092 0.05348 NA FALSE FALSE FALSE
GCST006061 0.0225 0.6825 537409 outcome TRUE reported 9370_69_GGH_GGH GGH
chr8 61813819 0.017914 1.417e-10 35345 TRUE reported textfile 2
1 TRUE
6068 rs77291996 C T C T 0.5901 -0.003 0.05876 NA FALSE FALSE FALSE
GCST006061 0.0135 0.825 537409 outcome TRUE reported 9370_69_GGH_GGH GGH
chr8 62830418 0.017003 1e-200 35344 TRUE reported textfile 2 1
TRUE
6069 rs825038 A C A C 0.1234 0.0218 0.05118 NA FALSE FALSE FALSE
GCST006061 0.0239 0.3624 537409 outcome TRUE reported 9370_69_GGH_GGH GGH
chr12 101570303 0.018264 1.413e-11 35363 TRUE reported textfile 2
1 TRUE
6070 rs10861032 C T C T 0.0697 0.0049 0.15546 NA FALSE FALSE FALSE
GCST006061 0.0096 0.609 537409 outcome TRUE reported 15667_39_BMP4_BMP_4
BMP4 chr12 103518728 0.011531 1.497e-09 35367 TRUE reported
textfile 2 1 TRUE
6071 rs117029746 A G A G -0.1689 -0.0099 0.0193 NA FALSE FALSE FALSE
GCST006061 0.0295 0.736 537409 outcome TRUE reported 15667_39_BMP4_BMP_4
BMP4 chr11 74259828 0.030564 3.274e-08 35371 TRUE reported
textfile 2 1 TRUE
6072 rs1355538 G A G A -0.0494 -0.0076 0.39847 NA FALSE FALSE FALSE
GCST006061 0.0072 0.2923 537409 outcome TRUE reported 15667_39_BMP4_BMP_4
BMP4 chr3 165787389 0.008461 5.259e-09 35338 TRUE reported
textfile 2 1 TRUE
6073 rs143501783 C T C T 0.2654 -0.0333 0.01116 NA FALSE FALSE FALSE
GCST006061 0.0443 0.4525 537409 outcome TRUE reported 15667_39_BMP4_BMP_4
BMP4 chr17 7897001 0.037773 2.122e-12 35373 TRUE reported textfile
2 1 TRUE
6074 rs35887873 C T C T 0.0831 -0.0114 0.26311 NA FALSE FALSE FALSE
GCST006061 0.0091 0.2089 537409 outcome TRUE reported 15667_39_BMP4_BMP_4
BMP4 chr11 126349501 0.009429 1.213e-18 35374 TRUE reported
textfile 2 1 TRUE
6075 rs55714927 T C T C 0.1114 1e-04 0.21166 NA FALSE FALSE FALSE
GCST006061 0.0098 0.9926 537409 outcome TRUE reported 15667_39_BMP4_BMP_4
BMP4 chr17 7176997 0.010225 1.222e-27 35370 TRUE reported textfile
2 1 TRUE
6076 rs6123685 A G A G 0.0776 0.0027 0.23171 NA FALSE FALSE FALSE

GCST006061 0.0085 0.7537 537409 outcome TRUE reported 15667_39_BMP4_BMP_4
BMP4 chr20 57260984 0.009871 3.791e-15 35388 TRUE reported
textfile 2 1 TRUE
6077 rs61389091 T C T C -0.4997 0.029 0.05062 NA FALSE FALSE FALSE
GCST006061 0.019 0.127 537409 outcome TRUE reported 15667_39_BMP4_BMP_4
BMP4 chr11 74716876 0.018791 8.257e-156 35367 TRUE reported
textfile 2 1 TRUE
6078 rs6592590 T C T C 0.1076 -0.0029 0.44399 NA FALSE FALSE FALSE
GCST006061 0.0072 0.6881 537409 outcome TRUE reported 15667_39_BMP4_BMP_4
BMP4 chr11 74669984 0.008385 1.078e-37 35370 TRUE reported
textfile 2 1 TRUE
6079 rs10861032 C T C T 0.0825 0.0049 0.15546 NA FALSE FALSE FALSE
GCST006061 0.0096 0.609 537409 outcome TRUE reported 15491_20_CD248_CD248
CD248 chr12 103518728 0.011657 1.47e-12 35367 TRUE reported
textfile 2 1 TRUE
6080 rs11039216 T C T C 0.0486 0.0126 0.48532 NA FALSE FALSE FALSE
GCST006061 0.0074 0.08661 537409 outcome TRUE reported 15491_20_CD248_CD248
CD248 chr11 47385041 0.008384 6.759e-09 35376 TRUE reported
textfile 2 1 TRUE
6081 rs116884770 T C T C 0.1167 0.0111 0.04934 NA FALSE FALSE FALSE
GCST006061 0.0258 0.6678 537409 outcome TRUE reported 15491_20_CD248_CD248
CD248 chr11 66904366 0.019407 1.819e-09 35363 TRUE reported
textfile 2 1 TRUE
6082 rs11768171 T C T C 0.065 -0.0038 0.48524 NA FALSE FALSE FALSE
GCST006061 0.0073 0.6014 537409 outcome TRUE reported 15491_20_CD248_CD248
CD248 chr7 100135692 0.008403 1.034e-14 35368 TRUE reported
textfile 2 1 TRUE
6083 rs12544863 T C T C -0.0477 0.0019 0.37043 NA FALSE FALSE FALSE
GCST006061 0.0076 0.7974 537409 outcome TRUE reported 15491_20_CD248_CD248
CD248 chr8 141226958 0.008667 3.727e-08 35307 TRUE reported
textfile 2 1 TRUE
6084 rs1260326 C T C T 0.0692 -0.0069 0.34132 NA FALSE FALSE FALSE
GCST006061 0.0073 0.3401 537409 outcome TRUE reported 15491_20_CD248_CD248
CD248 chr2 27508073 0.00889 7.043e-15 35373 TRUE reported textfile
2 1 TRUE
6085 rs141359357 A G A G 0.192 -0.022 0.03367 NA FALSE FALSE FALSE
GCST006061 0.0238 0.355 537409 outcome TRUE reported 15491_20_CD248_CD248
CD248 chr3 53161104 0.023582 3.898e-16 35343 TRUE reported
textfile 2 1 TRUE
6086 rs143840518 A C A C 0.2167 -0.0013 0.02183 NA FALSE FALSE FALSE
GCST006061 0.0404 0.9738 537409 outcome TRUE reported 15491_20_CD248_CD248
CD248 chr3 53540401 0.028968 7.395e-14 35350 TRUE reported
textfile 2 1 TRUE
6087 rs145928297 C A C A 0.2031 0.0697 0.01307 NA FALSE FALSE FALSE
GCST006061 0.0937 0.4571 537409 outcome TRUE reported 15491_20_CD248_CD248
CD248 chr11 67259085 0.036968 3.932e-08 35361 TRUE reported
textfile 2 1 TRUE
6088 rs150844304 C A C A -0.1412 0.0439 0.0276 NA FALSE FALSE FALSE
GCST006061 0.0244 0.07203 537409 outcome TRUE reported 15491_20_CD248_CD248
CD248 chr15 43434427 0.02551 3.11e-08 35363 TRUE reported textfile
2 1 TRUE
6089 rs186189054 T C T C 0.2554 0.0157 0.01482 NA FALSE FALSE FALSE
GCST006061 0.0561 0.7794 537409 outcome TRUE reported 15491_20_CD248_CD248
CD248 chr3 52075762 0.034386 1.107e-13 35336 TRUE reported
textfile 2 1 TRUE
6090 rs2142306 C T C T 0.0487 -0.0024 0.43618 NA FALSE FALSE FALSE
GCST006061 0.0073 0.7446 537409 outcome TRUE reported 15491_20_CD248_CD248
CD248 chr8 133458388 0.00844 7.916e-09 35374 TRUE reported textfile
2 1 TRUE
6091 rs3199918 C T C T 0.0771 6e-04 0.20519 NA FALSE FALSE FALSE
GCST006061 0.0097 0.9503 537409 outcome TRUE reported 15491_20_CD248_CD248
CD248 chr3 52547419 0.010378 1.095e-13 35352 TRUE reported
textfile 2 1 TRUE
6092 rs333947 A G A G -0.0741 -0.0071 0.14937 NA FALSE FALSE FALSE
GCST006061 0.01 0.4808 537409 outcome TRUE reported 15491_20_CD248_CD248
CD248 chr1 109928142 0.011875 4.381e-10 35349 TRUE reported

textfile 2 1 TRUE
6093 rs62292950 G T G T -0.0722 -0.0034 0.15819 NA FALSE FALSE FALSE
GCST006061 0.0104 0.7442 537409 outcome TRUE reported 15491_20_CD248_CD248
CD248 chr3 132479151 0.011552 4.103e-10 35358 TRUE reported
textfile 2 1 TRUE
6094 rs62446919 A G A G 0.0591 0.002 0.4195 NA FALSE FALSE FALSE
GCST006061 0.0072 0.7768 537409 outcome TRUE reported 15491_20_CD248_CD248
CD248 chr7 17984310 0.008486 3.291e-12 35366 TRUE reported
textfile 2 1 TRUE
6095 rs74814272 G A G A 0.2113 0.0247 0.01224 NA FALSE FALSE FALSE
GCST006061 0.0362 0.4957 537409 outcome TRUE reported 15491_20_CD248_CD248
CD248 chr3 41080294 0.037499 1.752e-08 35353 TRUE reported
textfile 2 1 TRUE
6096 rs10863437 C T C T 0.0834 0.0025 0.4265 NA FALSE FALSE FALSE
GCST006061 0.0073 0.7277 537409 outcome TRUE reported 5069_9_CD55_DAF CD55
chr1 207644267 0.008448 5.479e-23 35370 TRUE reported textfile 2
1 TRUE
6097 rs13108218 G A G A 0.0477 -0.01 0.36204 NA FALSE FALSE FALSE
GCST006061 0.0074 0.1774 537409 outcome TRUE reported 5069_9_CD55_DAF CD55
chr4 3442204 0.008546 2.381e-08 35280 TRUE reported textfile 2 1
TRUE
6098 rs143501783 C T C T 0.2782 -0.0333 0.01116 NA FALSE FALSE FALSE
GCST006061 0.0443 0.4525 537409 outcome TRUE reported 5069_9_CD55_DAF CD55
chr17 7897001 0.037506 1.193e-13 35373 TRUE reported textfile 2 1
TRUE
6099 rs148327582 C T C T -0.1669 -0.0562 0.02752 NA FALSE FALSE FALSE
GCST006061 0.0461 0.2236 537409 outcome TRUE reported 5069_9_CD55_DAF CD55
chr1 208186642 0.025601 7.068e-11 35367 TRUE reported textfile 2
1 TRUE
6100 rs148838761 T G T G -0.1538 0.018 0.03459 NA FALSE FALSE FALSE
GCST006061 0.0302 0.5509 537409 outcome TRUE reported 5069_9_CD55_DAF CD55
chr1 207061657 0.022591 9.897e-12 35374 TRUE reported textfile 2
1 TRUE
6101 rs2142306 C T C T 0.0637 -0.0024 0.43618 NA FALSE FALSE FALSE
GCST006061 0.0073 0.7446 537409 outcome TRUE reported 5069_9_CD55_DAF CD55
chr8 133458388 0.008288 1.519e-14 35374 TRUE reported textfile 2
1 TRUE
6102 rs2978006 G A G A 0.0627 -0.0029 0.36615 NA FALSE FALSE FALSE
GCST006061 0.0075 0.7034 537409 outcome TRUE reported 5069_9_CD55_DAF CD55
chr8 133521519 0.008531 1.982e-13 35373 TRUE reported textfile 2
1 TRUE
6103 rs6666604 G T G T -0.3504 -0.0065 0.1604 NA FALSE FALSE FALSE
GCST006061 0.0097 0.5049 537409 outcome TRUE reported 5069_9_CD55_DAF CD55
chr1 207172474 0.011266 1e-200 35369 TRUE reported textfile 2 1
TRUE
6104 rs72704449 C T C T 0.1442 0.013 0.04582 NA FALSE FALSE FALSE
GCST006061 0.0199 0.5128 537409 outcome TRUE reported 5069_9_CD55_DAF CD55
chr1 179504138 0.019894 4.218e-13 35358 TRUE reported textfile 2
1 TRUE
6105 rs10864508 G T G T 0.0465 -0.008 0.48014 NA FALSE FALSE FALSE
GCST006061 0.0073 0.2768 537409 outcome TRUE reported
7655_11_NPPB_N_terminal_pro_BNP NPPB chr1 11430687 0.008224 1.569e-08
35388 TRUE reported textfile 2 1 TRUE
6106 rs198379 C T C T 0.2574 -0.0081 0.39648 NA FALSE FALSE FALSE
GCST006061 0.0074 0.2768 537409 outcome TRUE reported
7655_11_NPPB_N_terminal_pro_BNP NPPB chr1 11855410 0.008282 1e-200 35387
TRUE reported textfile 2 1 TRUE
6107 rs2233019 C A C A 0.2134 -0.0276 0.0152 NA FALSE FALSE FALSE
GCST006061 0.0454 0.5438 537409 outcome TRUE reported
7655_11_NPPB_N_terminal_pro_BNP NPPB chr1 11676807 0.033392 1.65e-10
35381 TRUE reported textfile 2 1 TRUE
6108 rs2234962 C T C T 0.0777 -0.0024 0.24167 NA FALSE FALSE FALSE
GCST006061 0.0091 0.7907 537409 outcome TRUE reported
7655_11_NPPB_N_terminal_pro_BNP NPPB chr10 119670121 0.0095 2.861e-16 35378
TRUE reported textfile 2 1 TRUE
6109 rs3740293 C A C A -0.0911 0.1 0.14467 NA FALSE FALSE FALSE GCST006061

0.0101 3.008e-23 537409 outcome TRUE reported 7655_11_NPPB_N_terminal_pro_BNP NPPB chr10 73646383 0.011581 3.643e-15 35370 TRUE reported textfile 2 1 TRUE

6110 rs760189 T C T C 0.114 0.0012 0.19695 NA FALSE FALSE FALSE GCST006061 0.0091 0.8938 537409 outcome TRUE reported 7655_11_NPPB_N_terminal_pro_BNP NPPB chr1 11935503 0.010334 2.703e-28 35387 TRUE reported textfile 2 1 TRUE

6111 rs10868235 T C T C -0.0701 0.0105 0.46309 NA FALSE FALSE FALSE GCST006061 0.0072 0.1472 537409 outcome TRUE reported 4145_58_NTF3_Neurotrophin_3 NTF3 chr9 84878840 0.008026 2.467e-18 35362 TRUE reported textfile 2 1 TRUE

6112 rs6542680 T C T C -0.066 -0.0012 0.18362 NA FALSE FALSE FALSE GCST006061 0.0091 0.8935 537409 outcome TRUE reported 4145_58_NTF3_Neurotrophin_3 NTF3 chr2 3592552 0.010299 1.473e-10 35332 TRUE reported textfile 2 1 TRUE

6113 rs10869516 C T C T 0.0473 0.0115 0.3724 NA FALSE FALSE FALSE GCST006061 0.0076 0.1294 537409 outcome TRUE reported 10710_23_ZPBP2_ZPBP2 ZPBP2 chr9 75152655 0.007973 2.987e-09 35342 TRUE reported textfile 2 1 TRUE

6114 rs10922098 T C T C -0.0504 -0.009 0.40011 NA FALSE FALSE FALSE GCST006061 0.0075 0.2275 537409 outcome TRUE reported 10710_23_ZPBP2_ZPBP2 ZPBP2 chr1 196695521 0.007911 1.875e-10 35326 TRUE reported textfile 2 1 TRUE

6115 rs10876550 A G A G 0.0542 -1.00E-04 0.43699 NA FALSE FALSE FALSE GCST006061 0.0073 0.991 537409 outcome TRUE reported 15604_18_MAPK9_JNK2 MAPK9 chr12 54318524 0.00813 2.614e-11 35374 TRUE reported textfile 2 1 TRUE

6116 rs1354034 C T C T 0.0477 0.0088 0.38119 NA FALSE FALSE FALSE GCST006061 0.0073 0.2248 537409 outcome TRUE reported 15604_18_MAPK9_JNK2 MAPK9 chr3 56815721 0.008266 7.886e-09 35356 TRUE reported textfile 2 1 TRUE

6117 rs145843394 T C T C 0.3074 0.0165 0.01186 NA FALSE FALSE FALSE GCST006061 0.0428 0.6993 537409 outcome TRUE reported 15604_18_MAPK9_JNK2 MAPK9 chr5 180142991 0.037008 9.866e-17 35336 TRUE reported textfile 2 1 TRUE

6118 rs4980320 C T C T 0.0494 0.0041 0.46986 NA FALSE FALSE FALSE GCST006061 0.0076 0.5858 537409 outcome TRUE reported 15604_18_MAPK9_JNK2 MAPK9 chr11 198986 0.008065 9.045e-10 35352 TRUE reported textfile 2 1 TRUE

6119 rs10876550 A G A G 0.0504 -1.00E-04 0.43699 NA FALSE FALSE FALSE GCST006061 0.0073 0.991 537409 outcome TRUE reported 6923_1_PLOD2_PLOD2 PLOD2 chr12 54318524 0.008178 7.131e-10 35374 TRUE reported textfile 2 1 TRUE

6120 rs10876550 A G A G 0.046 -1.00E-04 0.43699 NA FALSE FALSE FALSE GCST006061 0.0073 0.991 537409 outcome TRUE reported 9869_28_NFKB1_NF_kappa_B_p105 NFKB1 chr12 54318524 0.00806 1.148e-08 35374 TRUE reported textfile 2 1 TRUE

6121 rs11601507 A C A C -0.1018 -0.0076 0.06035 NA FALSE FALSE FALSE GCST006061 0.0145 0.601 537409 outcome TRUE reported 9869_28_NFKB1_NF_kappa_B_p105 NFKB1 chr11 5679844 0.016766 1.266e-09 35378 TRUE reported textfile 2 1 TRUE

6122 rs144883301 T C T C -0.1413 -0.0297 0.02437 NA FALSE FALSE FALSE GCST006061 0.0242 0.2194 537409 outcome TRUE reported 9869_28_NFKB1_NF_kappa_B_p105 NFKB1 chr9 32156024 0.025864 4.674e-08 35355 TRUE reported textfile 2 1 TRUE

6123 rs230539 A G A G -0.0657 0.024 0.35127 NA FALSE FALSE FALSE GCST006061 0.0075 0.001401 537409 outcome TRUE reported 9869_28_NFKB1_NF_kappa_B_p105 NFKB1 chr4 102574375 0.008307 2.597e-15 35343 TRUE reported textfile 2 1 TRUE

6124 rs10876550 A G A G 0.0522 -1.00E-04 0.43699 NA FALSE FALSE FALSE GCST006061 0.0073 0.991 537409 outcome TRUE reported 11454_87_EIF3G_EIF3G EIF3G chr12 54318524 0.008235 2.31e-10 35365 TRUE reported textfile 2 1 TRUE

6125 rs1354034 C T C T 0.0715 0.0088 0.38119 NA FALSE FALSE FALSE GCST006061 0.0073 0.2248 537409 outcome TRUE reported 11454_87_EIF3G_EIF3G EIF3G chr3 56815721 0.008381 1.445e-17 35347 TRUE reported

textfile	2	1	TRUE
6126	rs3748136	A	G	A	G	0.1248	-0.0088	0.17278	NA	FALSE	FALSE	FALSE
GCST006061	0.0093	0.3444	537409	outcome	TRUE	reported	11454_87_EIF3G_EIF3G
EIF3G	chr8	9172650	0.010752	3.784e-31	35375	TRUE	reported	textfile
2	1	TRUE
6127	rs4980320	C	T	C	T	0.0534	0.0041	0.46986	NA	FALSE	FALSE	FALSE
GCST006061	0.0076	0.5858	537409	outcome	TRUE	reported	11454_87_EIF3G_EIF3G
EIF3G	chr11	198986	0.008173	6.43e-11	35343	TRUE	reported	textfile
2	1	TRUE
6128	rs10876550	A	G	A	G	0.0508	-1.00E-04	0.43699	NA	FALSE	FALSE	FALSE
GCST006061	0.0073	0.991	537409	outcome	TRUE	reported	11211_7_TBCE_TBCE
TBCE	chr12	54318524	0.008228	6.66e-10	35365	TRUE	reported
textfile	2	1	TRUE
6129	rs1109867	T	G	T	G	0.0877	-0.0072	0.10541	NA	FALSE	FALSE	FALSE
GCST006061	0.0116	0.5345	537409	outcome	TRUE	reported	11211_7_TBCE_TBCE
TBCE	chr2	219218731	0.013351	5.076e-11	35359	TRUE	reported
textfile	2	1	TRUE
6130	rs1354034	C	T	C	T	0.0703	0.0088	0.38119	NA	FALSE	FALSE	FALSE
GCST006061	0.0073	0.2248	537409	outcome	TRUE	reported	11211_7_TBCE_TBCE
TBCE	chr3	56815721	0.00837	4.493e-17	35347	TRUE	reported	textfile
2	1	TRUE
6131	rs34099250	G	A	G	A	-0.0554	-0.006	0.34948	NA	FALSE	FALSE	FALSE
GCST006061	0.0083	0.4713	537409	outcome	TRUE	reported	11211_7_TBCE_TBCE
TBCE	chr1	235056162	0.008512	7.604e-11	35349	TRUE	reported
textfile	2	1	TRUE
6132	rs4980320	C	T	C	T	0.0528	0.0041	0.46986	NA	FALSE	FALSE	FALSE
GCST006061	0.0076	0.5858	537409	outcome	TRUE	reported	11211_7_TBCE_TBCE
TBCE	chr11	198986	0.008148	9.171e-11	35343	TRUE	reported	textfile
2	1	TRUE
6133	rs903347	T	C	T	C	-0.1006	-0.0027	0.38663	NA	FALSE	FALSE	FALSE
GCST006061	0.0072	0.7046	537409	outcome	TRUE	reported	11211_7_TBCE_TBCE
TBCE	chr1	235423298	0.008345	1.813e-33	35356	TRUE	reported
textfile	2	1	TRUE
6134	rs10876550	A	G	A	G	0.0461	-1.00E-04	0.43699	NA	FALSE	FALSE	FALSE
GCST006061	0.0073	0.991	537409	outcome	TRUE	reported	12593_33_PDRG1_PDRG1
PDRG1	chr12	54318524	0.00806	1.068e-08	35365	TRUE	reported	textfile
2	1	TRUE
6135	rs1354034	C	T	C	T	0.0883	0.0088	0.38119	NA	FALSE	FALSE	FALSE
GCST006061	0.0073	0.2248	537409	outcome	TRUE	reported	12593_33_PDRG1_PDRG1
PDRG1	chr3	56815721	0.008193	4.415e-27	35347	TRUE	reported
textfile	2	1	TRUE
6136	rs4980320	C	T	C	T	0.0467	0.0041	0.46986	NA	FALSE	FALSE	FALSE
GCST006061	0.0076	0.5858	537409	outcome	TRUE	reported	12593_33_PDRG1_PDRG1
PDRG1	chr11	198986	0.007991	5.1e-09	35343	TRUE	reported	textfile	2
1	TRUE
6137	rs5742938	A	G	A	G	0.0575	0.0143	0.27025	NA	FALSE	FALSE	FALSE
GCST006061	0.008	0.07323	537409	outcome	TRUE	reported	12593_33_PDRG1_PDRG1
PDRG1	chr2	189785232	0.008994	1.628e-10	35361	TRUE	reported
textfile	2	1	TRUE
6138	rs10876550	A	G	A	G	0.0485	-1.00E-04	0.43699	NA	FALSE	FALSE	FALSE
GCST006061	0.0073	0.991	537409	outcome	TRUE	reported	5885_55_EIF4H_eIF_4H
EIF4H	chr12	54318524	0.008155	2.721e-09	35374	TRUE	reported
textfile	2	1	TRUE
6139	rs1354034	C	T	C	T	0.0791	0.0088	0.38119	NA	FALSE	FALSE	FALSE
GCST006061	0.0073	0.2248	537409	outcome	TRUE	reported	5885_55_EIF4H_eIF_4H
EIF4H	chr3	56815721	0.008294	1.477e-21	35356	TRUE	reported
textfile	2	1	TRUE
6140	rs4980320	C	T	C	T	0.0473	0.0041	0.46986	NA	FALSE	FALSE	FALSE
GCST006061	0.0076	0.5858	537409	outcome	TRUE	reported	5885_55_EIF4H_eIF_4H
EIF4H	chr11	198986	0.008093	5.077e-09	35352	TRUE	reported	textfile
2	1	TRUE
6141	rs10876550	A	G	A	G	0.0447	-1.00E-04	0.43699	NA	FALSE	FALSE	FALSE
GCST006061	0.0073	0.991	537409	outcome	TRUE	reported	12859_33_ECI2_PECI
ECI2	chr12	54318524	0.008024	2.536e-08	35365	TRUE	reported
textfile	2	1	TRUE
6142	rs1354034	C	T	C	T	0.0574	0.0088	0.38119	NA	FALSE	FALSE	FALSE

GCST006061 0.0073 0.2248 537409 outcome TRUE reported 12859_33_ECI2_PECI
ECI2 chr3 56815721 0.00818 2.262e-12 35347 TRUE reported textfile
2 1 TRUE
6143 rs4544959 G A G A 0.0907 0.009 0.44045 NA FALSE FALSE FALSE
GCST006061 0.0073 0.2183 537409 outcome TRUE reported 12859_33_ECI2_PECI
ECI2 chr6 4242217 0.008096 3.929e-29 35373 TRUE reported textfile
2 1 TRUE
6144 rs675073 A G A G 0.2691 0.0151 0.4264 NA FALSE FALSE FALSE
GCST006061 0.0076 0.04607 537409 outcome TRUE reported 12859_33_ECI2_PECI
ECI2 chr6 4067285 0.007997 1e-200 35378 TRUE reported textfile 2
1 TRUE
6145 rs74770429 C T C T -0.1117 0.0364 0.04468 NA FALSE FALSE FALSE
GCST006061 0.0335 0.2774 537409 outcome TRUE reported 12859_33_ECI2_PECI
ECI2 chr22 40814137 0.019338 7.645e-09 35362 TRUE reported
textfile 2 1 TRUE
6146 rs77482745 G A G A -0.1925 -0.0055 0.02242 NA FALSE FALSE FALSE
GCST006061 0.0314 0.8607 537409 outcome TRUE reported 12859_33_ECI2_PECI
ECI2 chr6 3882812 0.02652 3.912e-13 35375 TRUE reported textfile 2
1 TRUE
6147 rs10876550 A G A G 0.0456 -1.00E-04 0.43699 NA FALSE FALSE FALSE
GCST006061 0.0073 0.991 537409 outcome TRUE reported 12842_43_STX10_STX10
STX10 chr12 54318524 0.007965 1.033e-08 35365 TRUE reported
textfile 2 1 TRUE
6148 rs2057681 G A G A 0.1373 -0.0136 0.28541 NA FALSE FALSE FALSE
GCST006061 0.0078 0.07965 537409 outcome TRUE reported 12842_43_STX10_STX10
STX10 chr7 95308945 0.008728 9.356e-56 35340 TRUE reported
textfile 2 1 TRUE
6149 rs10876550 A G A G 0.051 -1.00E-04 0.43699 NA FALSE FALSE FALSE
GCST006061 0.0073 0.991 537409 outcome TRUE reported 12471_47_STAU1_STAU1
STAU1 chr12 54318524 0.008084 2.811e-10 35365 TRUE reported
textfile 2 1 TRUE
6150 rs10922098 T C T C -0.0484 -0.009 0.40011 NA FALSE FALSE FALSE
GCST006061 0.0075 0.2275 537409 outcome TRUE reported 12471_47_STAU1_STAU1
STAU1 chr1 196695521 0.008165 3.077e-09 35329 TRUE reported
textfile 2 1 TRUE
6151 rs11553699 G A G A 0.072 -0.0209 0.14277 NA FALSE FALSE FALSE
GCST006061 0.012 0.08291 537409 outcome TRUE reported 12471_47_STAU1_STAU1
STAU1 chr12 121779004 0.011448 3.186e-10 35357 TRUE reported
textfile 2 1 TRUE
6152 rs1354034 C T C T 0.1067 0.0088 0.38119 NA FALSE FALSE FALSE
GCST006061 0.0073 0.2248 537409 outcome TRUE reported 12471_47_STAU1_STAU1
STAU1 chr3 56815721 0.00822 1.582e-38 35347 TRUE reported textfile
2 1 TRUE
6153 rs1355538 G A G A -0.0571 -0.0076 0.39847 NA FALSE FALSE FALSE
GCST006061 0.0072 0.2923 537409 outcome TRUE reported 12471_47_STAU1_STAU1
STAU1 chr3 165787389 0.008155 2.533e-12 35329 TRUE reported
textfile 2 1 TRUE
6154 rs9894057 A G A G 0.1001 -0.0013 0.15123 NA FALSE FALSE FALSE
GCST006061 0.009 0.8863 537409 outcome TRUE reported 12471_47_STAU1_STAU1
STAU1 chr17 35562904 0.011133 2.435e-19 35362 TRUE reported
textfile 2 1 TRUE
6155 rs10876550 A G A G 0.0448 -1.00E-04 0.43699 NA FALSE FALSE FALSE
GCST006061 0.0073 0.991 537409 outcome TRUE reported 2998_53_JAM3_JAM_C
JAM3 chr12 54318524 0.008186 4.439e-08 35374 TRUE reported
textfile 2 1 TRUE
6156 rs1354034 C T C T 0.0519 0.0088 0.38119 NA FALSE FALSE FALSE
GCST006061 0.0073 0.2248 537409 outcome TRUE reported 2998_53_JAM3_JAM_C
JAM3 chr3 56815721 0.008325 4.54e-10 35356 TRUE reported
textfile 2 1 TRUE
6157 rs4980320 C T C T 0.0514 0.0041 0.46986 NA FALSE FALSE FALSE
GCST006061 0.0076 0.5858 537409 outcome TRUE reported 2998_53_JAM3_JAM_C
JAM3 chr11 198986 0.008121 2.464e-10 35352 TRUE reported textfile
2 1 TRUE
6158 rs10876550 A G A G 0.0516 -1.00E-04 0.43699 NA FALSE FALSE FALSE
GCST006061 0.0073 0.991 537409 outcome TRUE reported 7713_102_FMR1_FMR1
FMR1 chr12 54318524 0.008128 2.18e-10 35374 TRUE reported

textfile 2 1 TRUE
6159 rs11601665 T C T C 0.0568 -0.0045 0.26367 NA FALSE FALSE FALSE
GCST006061 0.0088 0.6088 537409 outcome TRUE reported 7713_102_FMR1_FMR1
FMR1 chr11 262930 0.009099 4.301e-10 35352 TRUE reported textfile
2 1 TRUE
6160 rs1354034 C T C T 0.0786 0.0088 0.38119 NA FALSE FALSE FALSE
GCST006061 0.0073 0.2248 537409 outcome TRUE reported 7713_102_FMR1_FMR1
FMR1 chr3 56815721 0.008278 2.194e-21 35356 TRUE reported
textfile 2 1 TRUE
6161 rs185009887 A G A G -0.1462 -0.0595 0.02519 NA FALSE FALSE FALSE
GCST006061 0.0496 0.2302 537409 outcome TRUE reported 7713_102_FMR1_FMR1
FMR1 chr17 5848332 0.026084 2.084e-08 35374 TRUE reported textfile
2 1 TRUE
6162 rs10876550 A G A G 0.051 -1.00E-04 0.43699 NA FALSE FALSE FALSE
GCST006061 0.0073 0.991 537409 outcome TRUE reported 13976_9_ARHGEF1_ARHG1
ARHGEF1 chr12 54318524 0.0081 3.041e-10 35374 TRUE reported textfile
2 1 TRUE
6163 rs1354034 C T C T 0.0455 0.0088 0.38119 NA FALSE FALSE FALSE
GCST006061 0.0073 0.2248 537409 outcome TRUE reported 13976_9_ARHGEF1_ARHG1
ARHGEF1 chr3 56815721 0.008255 3.546e-08 35356 TRUE reported
textfile 2 1 TRUE
6164 rs4980320 C T C T 0.0479 0.0041 0.46986 NA FALSE FALSE FALSE
GCST006061 0.0076 0.5858 537409 outcome TRUE reported 13976_9_ARHGEF1_ARHG1
ARHGEF1 chr11 198986 0.008043 2.592e-09 35352 TRUE reported textfile
2 1 TRUE
6165 rs10876550 A G A G 0.0516 -1.00E-04 0.43699 NA FALSE FALSE FALSE
GCST006061 0.0073 0.991 537409 outcome TRUE reported 19131_184_TIAL1_TIAR
TIAL1 chr12 54318524 0.008098 1.865e-10 35374 TRUE reported
textfile 2 1 TRUE
6166 rs1354034 C T C T 0.0745 0.0088 0.38119 NA FALSE FALSE FALSE
GCST006061 0.0073 0.2248 537409 outcome TRUE reported 19131_184_TIAL1_TIAR
TIAL1 chr3 56815721 0.008241 1.568e-19 35356 TRUE reported
textfile 2 1 TRUE
6167 rs185009887 A G A G -0.1541 -0.0595 0.02519 NA FALSE FALSE FALSE
GCST006061 0.0496 0.2302 537409 outcome TRUE reported 19131_184_TIAL1_TIAR
TIAL1 chr17 5848332 0.025999 3.084e-09 35374 TRUE reported textfile
2 1 TRUE
6168 rs4980320 C T C T 0.0518 0.0041 0.46986 NA FALSE FALSE FALSE
GCST006061 0.0076 0.5858 537409 outcome TRUE reported 19131_184_TIAL1_TIAR
TIAL1 chr11 198986 0.008038 1.16e-10 35352 TRUE reported textfile
2 1 TRUE
6169 rs10876550 A G A G 0.06 -1.00E-04 0.43699 NA FALSE FALSE FALSE
GCST006061 0.0073 0.991 537409 outcome TRUE reported 12724_81_CIRBP_CIRBP
CIRBP chr12 54318524 0.008228 3.043e-13 35365 TRUE reported
textfile 2 1 TRUE
6170 rs1354034 C T C T 0.1177 0.0088 0.38119 NA FALSE FALSE FALSE
GCST006061 0.0073 0.2248 537409 outcome TRUE reported 12724_81_CIRBP_CIRBP
CIRBP chr3 56815721 0.008365 5.774e-45 35347 TRUE reported
textfile 2 1 TRUE
6171 rs4980320 C T C T 0.053 0.0041 0.46986 NA FALSE FALSE FALSE
GCST006061 0.0076 0.5858 537409 outcome TRUE reported 12724_81_CIRBP_CIRBP
CIRBP chr11 198986 0.008162 8.385e-11 35343 TRUE reported textfile
2 1 TRUE
6172 rs10876550 A G A G 0.0448 -1.00E-04 0.43699 NA FALSE FALSE FALSE
GCST006061 0.0073 0.991 537409 outcome TRUE reported 13570_43_HDLBP_VIGLN
HDLBP chr12 54318524 0.008137 3.671e-08 35368 TRUE reported
textfile 2 1 TRUE
6173 rs1354034 C T C T 0.0768 0.0088 0.38119 NA FALSE FALSE FALSE
GCST006061 0.0073 0.2248 537409 outcome TRUE reported 13570_43_HDLBP_VIGLN
HDLBP chr3 56815721 0.008282 1.802e-20 35350 TRUE reported
textfile 2 1 TRUE
6174 rs4980320 C T C T 0.0519 0.0041 0.46986 NA FALSE FALSE FALSE
GCST006061 0.0076 0.5858 537409 outcome TRUE reported 13570_43_HDLBP_VIGLN
HDLBP chr11 198986 0.008074 1.29e-10 35346 TRUE reported textfile
2 1 TRUE
6175 rs10876550 A G A G 0.0694 -1.00E-04 0.43699 NA FALSE FALSE FALSE

GCST006061 0.0073 0.991 537409 outcome TRUE reported 11540_37_FOXO3_FOXO3A
FOXO3 chr12 54318524 0.008123 1.304e-17 35365 TRUE reported
textfile 2 1 TRUE
6176 rs1354034 C T C T -0.064 0.0088 0.38119 NA FALSE FALSE FALSE
GCST006061 0.0073 0.2248 537409 outcome TRUE reported 11540_37_FOXO3_FOXO3A
FOXO3 chr3 56815721 0.008267 9.842e-15 35347 TRUE reported
textfile 2 1 TRUE
6177 rs3813498 T C T C -0.0634 0.0082 0.18042 NA FALSE FALSE FALSE
GCST006061 0.0091 0.3625 537409 outcome TRUE reported 11540_37_FOXO3_FOXO3A
FOXO3 chr6 108622962 0.010373 9.835e-10 35365 TRUE reported
textfile 2 1 TRUE
6178 rs10876550 A G A G 0.0449 -1.00E-04 0.43699 NA FALSE FALSE FALSE
GCST006061 0.0073 0.991 537409 outcome TRUE reported 3887_90_KPNB1_IMB1
KPNB1 chr12 54318524 0.008066 2.6e-08 35374 TRUE reported textfile
2 1 TRUE
6179 rs1354034 C T C T 0.0643 0.0088 0.38119 NA FALSE FALSE FALSE
GCST006061 0.0073 0.2248 537409 outcome TRUE reported 3887_90_KPNB1_IMB1
KPNB1 chr3 56815721 0.008211 4.851e-15 35356 TRUE reported
textfile 2 1 TRUE
6180 rs4980320 C T C T 0.0475 0.0041 0.46986 NA FALSE FALSE FALSE
GCST006061 0.0076 0.5858 537409 outcome TRUE reported 3887_90_KPNB1_IMB1
KPNB1 chr11 198986 0.008 2.897e-09 35352 TRUE reported textfile 2
1 TRUE
6181 rs10876550 A G A G 0.0454 -1.00E-04 0.43699 NA FALSE FALSE FALSE
GCST006061 0.0073 0.991 537409 outcome TRUE reported 8035_6_C1orf198_CA198
C1orf198 chr12 54318524 0.00814 2.441e-08 35374 TRUE reported
textfile 2 1 TRUE
6182 rs4414033 A G A G 0.047 -0.0207 0.39306 NA FALSE FALSE FALSE
GCST006061 0.0075 0.005726 537409 outcome TRUE reported
8035_6_C1orf198_CA198 C1orf198 chr1 156437061 0.008222 1.09e-08 35358
TRUE reported textfile 2 1 TRUE
6183 rs56148170 G A G A 0.1438 0.0121 0.12962 NA FALSE FALSE FALSE
GCST006061 0.0098 0.2158 537409 outcome TRUE reported 8035_6_C1orf198_CA198
C1orf198 chr1 230872285 0.011993 3.994e-33 35365 TRUE reported
textfile 2 1 TRUE
6184 rs10876550 A G A G 0.049 -1.00E-04 0.43699 NA FALSE FALSE FALSE
GCST006061 0.0073 0.991 537409 outcome TRUE reported 12720_71_UBQLN4_UBQL4
UBQLN4 chr12 54318524 0.008116 1.566e-09 35365 TRUE reported
textfile 2 1 TRUE
6185 rs1354034 C T C T 0.0653 0.0088 0.38119 NA FALSE FALSE FALSE
GCST006061 0.0073 0.2248 537409 outcome TRUE reported 12720_71_UBQLN4_UBQL4
UBQLN4 chr3 56815721 0.008263 2.724e-15 35347 TRUE reported
textfile 2 1 TRUE
6186 rs185009887 A G A G -0.1434 -0.0595 0.02519 NA FALSE FALSE FALSE
GCST006061 0.0496 0.2302 537409 outcome TRUE reported 12720_71_UBQLN4_UBQL4
UBQLN4 chr17 5848332 0.026045 3.673e-08 35365 TRUE reported textfile
2 1 TRUE
6187 rs4980320 C T C T 0.0503 0.0041 0.46986 NA FALSE FALSE FALSE
GCST006061 0.0076 0.5858 537409 outcome TRUE reported 12720_71_UBQLN4_UBQL4
UBQLN4 chr11 198986 0.008051 4.169e-10 35343 TRUE reported textfile
2 1 TRUE
6188 rs10876550 A G A G 0.0447 -1.00E-04 0.43699 NA FALSE FALSE FALSE
GCST006061 0.0073 0.991 537409 outcome TRUE reported 9842_2_CTNNB1_b_Catenin
CTNNB1 chr12 54318524 0.007968 2.024e-08 35374 TRUE reported
textfile 2 1 TRUE
6189 rs116148318 C T C T -0.2174 0.0273 0.01241 NA FALSE FALSE FALSE
GCST006061 0.0396 0.4912 537409 outcome TRUE reported 9842_2_CTNNB1_b_Catenin
CTNNB1 chr5 30067405 0.035978 1.517e-09 35369 TRUE reported
textfile 2 1 TRUE
6190 rs1276194 A G A G -0.0965 0.0031 0.33301 NA FALSE FALSE FALSE
GCST006061 0.0078 0.6884 537409 outcome TRUE reported 9842_2_CTNNB1_b_Catenin
CTNNB1 chr5 30916140 0.008701 1.393e-28 35373 TRUE reported
textfile 2 1 TRUE
6191 rs1347640 A C A C 0.2777 -0.0026 0.05554 NA FALSE FALSE FALSE
GCST006061 0.0168 0.8772 537409 outcome TRUE reported 9842_2_CTNNB1_b_Catenin
CTNNB1 chr5 30661018 0.017734 2.89e-55 35371 TRUE reported

textfile 2 1 TRUE
6192 rs149683334 G T G T 0.1847 -0.0624 0.03697 NA FALSE FALSE FALSE
GCST006061 0.0345 0.07033 537409 outcome TRUE reported 9842_2_CTNNB1_b_Catenin
CTNNB1 chr5 29620160 0.022135 7.173e-17 35364 TRUE reported
textfile 2 1 TRUE
6193 rs150195415 A G A G 0.132 0.0352 0.04043 NA FALSE FALSE FALSE
GCST006061 0.0295 0.233 537409 outcome TRUE reported 9842_2_CTNNB1_b_Catenin
CTNNB1 chr5 23768318 0.020952 2.975e-10 35373 TRUE reported
textfile 2 1 TRUE
6194 rs185992068 A C A C -0.2 0.0066 0.0152 NA FALSE FALSE FALSE
GCST006061 0.0571 0.9077 537409 outcome TRUE reported 9842_2_CTNNB1_b_Catenin
CTNNB1 chr5 20003742 0.034354 5.825e-09 35369 TRUE reported
textfile 2 1 TRUE
6195 rs2018523 A G A G 0.3208 0.0106 0.44024 NA FALSE FALSE FALSE
GCST006061 0.0078 0.1768 537409 outcome TRUE reported 9842_2_CTNNB1_b_Catenin
CTNNB1 chr5 30593275 0.006331 1e-200 35371 TRUE reported textfile
2 1 TRUE
6196 rs35340377 A G A G -0.0526 0.0075 0.25094 NA FALSE FALSE FALSE
GCST006061 0.0082 0.3585 537409 outcome TRUE reported 9842_2_CTNNB1_b_Catenin
CTNNB1 chr1 247874908 0.009162 9.412e-09 35156 TRUE reported
textfile 2 1 TRUE
6197 rs62356994 C T C T -0.3593 -0.0144 0.08407 NA FALSE FALSE FALSE
GCST006061 0.0115 0.2113 537409 outcome TRUE reported 9842_2_CTNNB1_b_Catenin
CTNNB1 chr5 30841905 0.014723 1.572e-131 35373 TRUE reported
textfile 2 1 TRUE
6198 rs79780354 T C T C -0.2669 0.0294 0.01313 NA FALSE FALSE FALSE
GCST006061 0.0561 0.6 537409 outcome TRUE reported 9842_2_CTNNB1_b_Catenin
CTNNB1 chr5 29544408 0.03654 2.785e-13 35366 TRUE reported textfile
2 1 TRUE
6199 rs10876550 A G A G 0.0491 -1.00E-04 0.43699 NA FALSE FALSE FALSE
GCST006061 0.0073 0.991 537409 outcome TRUE reported 19121_3_UBQLN2_UBQL2
UBQLN2 chr12 54318524 0.008125 1.513e-09 35374 TRUE reported
textfile 2 1 TRUE
6200 rs1354034 C T C T 0.0639 0.0088 0.38119 NA FALSE FALSE FALSE
GCST006061 0.0073 0.2248 537409 outcome TRUE reported 19121_3_UBQLN2_UBQL2
UBQLN2 chr3 56815721 0.008272 1.123e-14 35356 TRUE reported
textfile 2 1 TRUE
6201 rs4980320 C T C T 0.052 0.0041 0.46986 NA FALSE FALSE FALSE
GCST006061 0.0076 0.5858 537409 outcome TRUE reported 19121_3_UBQLN2_UBQL2
UBQLN2 chr11 198986 0.008061 1.111e-10 35352 TRUE reported textfile
2 1 TRUE
6202 rs10876550 A G A G 0.0571 -1.00E-04 0.43699 NA FALSE FALSE FALSE
GCST006061 0.0073 0.991 537409 outcome TRUE reported 19281_86_MAFG_MAFG
MAFG chr12 54318524 0.008042 1.243e-12 35374 TRUE reported
textfile 2 1 TRUE
6203 rs11594179 T C T C 0.063 0.0011 0.18128 NA FALSE FALSE FALSE
GCST006061 0.0089 0.9 537409 outcome TRUE reported 19281_86_MAFG_MAFG MAFG
chr10 102632823 0.010392 1.341e-09 35370 TRUE reported textfile 2
1 TRUE
6204 rs342290 C A C A -0.0566 -5.00E-04 0.45126 NA FALSE FALSE FALSE
GCST006061 0.0073 0.949 537409 outcome TRUE reported 19281_86_MAFG_MAFG
MAFG chr7 106727158 0.007962 1.173e-12 35357 TRUE reported
textfile 2 1 TRUE
6205 rs10876550 A G A G 0.0472 -1.00E-04 0.43699 NA FALSE FALSE FALSE
GCST006061 0.0073 0.991 537409 outcome TRUE reported 17826_341_EEF1D_EF1D
EEF1D chr12 54318524 0.008225 9.534e-09 35374 TRUE reported
textfile 2 1 TRUE
6206 rs1354034 C T C T 0.0632 0.0088 0.38119 NA FALSE FALSE FALSE
GCST006061 0.0073 0.2248 537409 outcome TRUE reported 17826_341_EEF1D_EF1D
EEF1D chr3 56815721 0.008364 4.161e-14 35356 TRUE reported
textfile 2 1 TRUE
6207 rs3811444 T C T C -0.104 0.0103 0.34232 NA FALSE FALSE FALSE
GCST006061 0.0077 0.1812 537409 outcome TRUE reported 17826_341_EEF1D_EF1D
EEF1D chr1 247876149 0.00863 1.902e-33 35156 TRUE reported textfile
2 1 TRUE
6208 rs4980320 C T C T 0.0541 0.0041 0.46986 NA FALSE FALSE FALSE

GCST006061 0.0076 0.5858 537409 outcome TRUE reported 17826_341_EEF1D_EF1D
EEF1D chr11 198986 0.008158 3.317e-11 35352 TRUE reported textfile
2 1 TRUE
6209 rs10876550 A G A G 0.0469 -1.00E-04 0.43699 NA FALSE FALSE FALSE
GCST006061 0.0073 0.991 537409 outcome TRUE reported 13076_4_FXR1_FXR1
FXR1 chr12 54318524 0.008143 8.425e-09 35365 TRUE reported
textfile 2 1 TRUE
6210 rs10922098 T C T C -0.0936 -0.009 0.40011 NA FALSE FALSE FALSE
GCST006061 0.0075 0.2275 537409 outcome TRUE reported 13076_4_FXR1_FXR1
FXR1 chr1 196695521 0.008219 4.792e-30 35329 TRUE reported
textfile 2 1 TRUE
6211 rs1354034 C T C T 0.06 0.0088 0.38119 NA FALSE FALSE FALSE
GCST006061 0.0073 0.2248 537409 outcome TRUE reported 13076_4_FXR1_FXR1
FXR1 chr3 56815721 0.008278 4.23e-13 35347 TRUE reported
textfile 2 1 TRUE
6212 rs1355538 G A G A -0.0538 -0.0076 0.39847 NA FALSE FALSE FALSE
GCST006061 0.0072 0.2923 537409 outcome TRUE reported 13076_4_FXR1_FXR1
FXR1 chr3 165787389 0.008212 5.714e-11 35329 TRUE reported
textfile 2 1 TRUE
6213 rs3847647 A C A C 0.0471 0.0034 0.33648 NA FALSE FALSE FALSE
GCST006061 0.0079 0.6636 537409 outcome TRUE reported 13076_4_FXR1_FXR1
FXR1 chr11 218141 0.008554 3.661e-08 35343 TRUE reported textfile
2 1 TRUE
6214 rs10876550 A G A G 0.0733 -1.00E-04 0.43699 NA FALSE FALSE FALSE
GCST006061 0.0073 0.991 537409 outcome TRUE reported 10056_5_FOXM1_FOXM1
FOXM1 chr12 54318524 0.008172 2.964e-19 35362 TRUE reported
textfile 2 1 TRUE
6215 rs117828578 A G A G 0.1983 -0.0385 0.02463 NA FALSE FALSE FALSE
GCST006061 0.0207 0.06343 537409 outcome TRUE reported 10056_5_FOXM1_FOXM1
FOXM1 chr13 40645935 0.025474 7.009e-15 35348 TRUE reported
textfile 2 1 TRUE
6216 rs1354034 C T C T -0.0612 0.0088 0.38119 NA FALSE FALSE FALSE
GCST006061 0.0073 0.2248 537409 outcome TRUE reported 10056_5_FOXM1_FOXM1
FOXM1 chr3 56815721 0.00832 1.894e-13 35344 TRUE reported textfile
2 1 TRUE
6217 rs10876550 A G A G 0.0678 -1.00E-04 0.43699 NA FALSE FALSE FALSE
GCST006061 0.0073 0.991 537409 outcome TRUE reported 12747_89_RBM3_RBM3
RBM3 chr12 54318524 0.008176 1.109e-16 35365 TRUE reported
textfile 2 1 TRUE
6218 rs114694170 C T C T 0.0863 0.0053 0.08378 NA FALSE FALSE FALSE
GCST006061 0.0175 0.7624 537409 outcome TRUE reported 12747_89_RBM3_RBM3
RBM3 chr5 88884379 0.014672 4.056e-09 35335 TRUE reported
textfile 2 1 TRUE
6219 rs1354034 C T C T 0.1125 0.0088 0.38119 NA FALSE FALSE FALSE
GCST006061 0.0073 0.2248 537409 outcome TRUE reported 12747_89_RBM3_RBM3
RBM3 chr3 56815721 0.008312 9.681e-42 35347 TRUE reported
textfile 2 1 TRUE
6220 rs4980320 C T C T 0.0533 0.0041 0.46986 NA FALSE FALSE FALSE
GCST006061 0.0076 0.5858 537409 outcome TRUE reported 12747_89_RBM3_RBM3
RBM3 chr11 198986 0.008107 4.875e-11 35343 TRUE reported textfile
2 1 TRUE
6221 rs10876550 A G A G 0.0487 -1.00E-04 0.43699 NA FALSE FALSE FALSE
GCST006061 0.0073 0.991 537409 outcome TRUE reported 13488_3_ARFIP1_ARFP1
ARFIP1 chr12 54318524 0.008228 3.24e-09 35368 TRUE reported
textfile 2 1 TRUE
6222 rs1354034 C T C T 0.074 0.0088 0.38119 NA FALSE FALSE FALSE
GCST006061 0.0073 0.2248 537409 outcome TRUE reported 13488_3_ARFIP1_ARFP1
ARFIP1 chr3 56815721 0.008381 1.049e-18 35350 TRUE reported
textfile 2 1 TRUE
6223 rs4619875 T C T C 0.2215 -0.0138 0.37238 NA FALSE FALSE FALSE
GCST006061 0.0072 0.0568 537409 outcome TRUE reported 13488_3_ARFIP1_ARFP1
ARFIP1 chr4 152779978 0.008385 8.572e-154 35371 TRUE reported
textfile 2 1 TRUE
6224 rs4980320 C T C T 0.0545 0.0041 0.46986 NA FALSE FALSE FALSE
GCST006061 0.0076 0.5858 537409 outcome TRUE reported 13488_3_ARFIP1_ARFP1
ARFIP1 chr11 198986 0.008164 2.455e-11 35346 TRUE reported textfile

2 1 TRUE
6225 rs77450465 A G A G -0.0814 0.0114 0.11819 NA FALSE FALSE FALSE
GCST006061 0.0143 0.4234 537409 outcome TRUE reported 13488_3_ARFIP1_ARFP1
ARFIP1 chr4 152485987 0.012618 1.109e-10 35368 TRUE reported
textfile 2 1 TRUE
6226 rs10876550 A G A G 0.0664 -1.00E-04 0.43699 NA FALSE FALSE FALSE
GCST006061 0.0073 0.991 537409 outcome TRUE reported 18183_3_LDLRAP1_ARH
LDLRAP1 chr12 54318524 0.008302 1.26e-15 35374 TRUE reported
textfile 2 1 TRUE
6227 rs11248997 C T C T 0.049 0.0051 0.37628 NA FALSE FALSE FALSE
GCST006061 0.0078 0.5116 537409 outcome TRUE reported 18183_3_LDLRAP1_ARH
LDLRAP1 chr1 25193792 0.008529 9.191e-09 35370 TRUE reported
textfile 2 1 TRUE
6228 rs35340377 A G A G -0.0538 0.0075 0.25094 NA FALSE FALSE FALSE
GCST006061 0.0082 0.3585 537409 outcome TRUE reported 18183_3_LDLRAP1_ARH
LDLRAP1 chr1 247874908 0.009573 1.907e-08 35156 TRUE reported
textfile 2 1 TRUE
6229 rs72673751 C T C T -0.0764 0.0202 0.14705 NA FALSE FALSE FALSE
GCST006061 0.0093 0.0294 537409 outcome TRUE reported 18183_3_LDLRAP1_ARH
LDLRAP1 chr8 105566712 0.011656 5.588e-11 35365 TRUE reported
textfile 2 1 TRUE
6230 rs75446219 T C T C 0.2673 -0.0209 0.03208 NA FALSE FALSE FALSE
GCST006061 0.0252 0.4073 537409 outcome TRUE reported 18183_3_LDLRAP1_ARH
LDLRAP1 chr1 25551001 0.023894 4.727e-29 35369 TRUE reported
textfile 2 1 TRUE
6231 rs10876550 A G A G 0.0454 -1.00E-04 0.43699 NA FALSE FALSE FALSE
GCST006061 0.0073 0.991 537409 outcome TRUE reported 4487_1_FGF7_FGF7
FGF7 chr12 54318524 0.008207 3.171e-08 35374 TRUE reported
textfile 2 1 TRUE
6232 rs1354034 C T C T 0.0503 0.0088 0.38119 NA FALSE FALSE FALSE
GCST006061 0.0073 0.2248 537409 outcome TRUE reported 4487_1_FGF7_FGF7
FGF7 chr3 56815721 0.008347 1.68e-09 35356 TRUE reported
textfile 2 1 TRUE
6233 rs4980320 C T C T 0.0497 0.0041 0.46986 NA FALSE FALSE FALSE
GCST006061 0.0076 0.5858 537409 outcome TRUE reported 4487_1_FGF7_FGF7
FGF7 chr11 198986 0.008131 9.833e-10 35352 TRUE reported textfile
2 1 TRUE
6234 rs10876550 A G A G 0.0478 -1.00E-04 0.43699 NA FALSE FALSE FALSE
GCST006061 0.0073 0.991 537409 outcome TRUE reported 14326_4_UBE2E1_UB2E1
UBE2E1 chr12 54318524 0.008135 4.201e-09 35374 TRUE reported
textfile 2 1 TRUE
6235 rs1354034 C T C T 0.1668 0.0088 0.38119 NA FALSE FALSE FALSE
GCST006061 0.0073 0.2248 537409 outcome TRUE reported 14326_4_UBE2E1_UB2E1
UBE2E1 chr3 56815721 0.008254 8.287e-91 35356 TRUE reported
textfile 2 1 TRUE
6236 rs4980320 C T C T 0.0711 0.0041 0.46986 NA FALSE FALSE FALSE
GCST006061 0.0076 0.5858 537409 outcome TRUE reported 14326_4_UBE2E1_UB2E1
UBE2E1 chr11 198986 0.008069 1.239e-18 35352 TRUE reported textfile
2 1 TRUE
6237 rs10876550 A G A G 0.0491 -1.00E-04 0.43699 NA FALSE FALSE FALSE
GCST006061 0.0073 0.991 537409 outcome TRUE reported 13044_5_TSG101_TS101
TSG101 chr12 54318524 0.008111 1.419e-09 35365 TRUE reported
textfile 2 1 TRUE
6238 rs1395319 G T G T 0.1473 0.0097 0.32694 NA FALSE FALSE FALSE
GCST006061 0.0075 0.1992 537409 outcome TRUE reported 13044_5_TSG101_TS101
TSG101 chr11 18526636 0.008558 2.14e-66 35374 TRUE reported
textfile 2 1 TRUE
6239 rs185009887 A G A G -0.1472 -0.0595 0.02519 NA FALSE FALSE FALSE
GCST006061 0.0496 0.2302 537409 outcome TRUE reported 13044_5_TSG101_TS101
TSG101 chr17 5848332 0.026033 1.565e-08 35365 TRUE reported textfile
2 1 TRUE
6240 rs4980320 C T C T 0.0498 0.0041 0.46986 NA FALSE FALSE FALSE
GCST006061 0.0076 0.5858 537409 outcome TRUE reported 13044_5_TSG101_TS101
TSG101 chr11 198986 0.008052 6.208e-10 35343 TRUE reported textfile
2 1 TRUE
6241 rs10876550 A G A G 0.0532 -1.00E-04 0.43699 NA FALSE FALSE FALSE

GCST006061 0.0073 0.991 537409 outcome TRUE reported 19266_35_G3BP1_G3BP1
G3BP1 chr12 54318524 0.008066 4.247e-11 35374 TRUE reported
textfile 2 1 TRUE
6242 rs1354034 C T C T 0.0769 0.0088 0.38119 NA FALSE FALSE FALSE
GCST006061 0.0073 0.2248 537409 outcome TRUE reported 19266_35_G3BP1_G3BP1
G3BP1 chr3 56815721 0.008211 7.606e-21 35356 TRUE reported
textfile 2 1 TRUE
6243 rs185009887 A G A G -0.1467 -0.0595 0.02519 NA FALSE FALSE FALSE
GCST006061 0.0496 0.2302 537409 outcome TRUE reported 19266_35_G3BP1_G3BP1
G3BP1 chr17 5848332 0.025893 1.464e-08 35374 TRUE reported textfile
2 1 TRUE
6244 rs4980320 C T C T 0.0515 0.0041 0.46986 NA FALSE FALSE FALSE
GCST006061 0.0076 0.5858 537409 outcome TRUE reported 19266_35_G3BP1_G3BP1
G3BP1 chr11 198986 0.008 1.214e-10 35352 TRUE reported textfile 2
1 TRUE
6245 rs10876550 A G A G 0.0454 -1.00E-04 0.43699 NA FALSE FALSE FALSE
GCST006061 0.0073 0.991 537409 outcome TRUE reported 9960_2_TBC1D5_TBCD5
TBC1D5 chr12 54318524 0.008083 1.946e-08 35374 TRUE reported
textfile 2 1 TRUE
6246 rs12465887 C A C A -0.0634 0.0026 0.14744 NA FALSE FALSE FALSE
GCST006061 0.0094 0.7793 537409 outcome TRUE reported 9960_2_TBC1D5_TBCD5
TBC1D5 chr2 239575191 0.011401 2.683e-08 35290 TRUE reported
textfile 2 1 TRUE
6247 rs1354034 C T C T 0.0481 0.0088 0.38119 NA FALSE FALSE FALSE
GCST006061 0.0073 0.2248 537409 outcome TRUE reported 9960_2_TBC1D5_TBCD5
TBC1D5 chr3 56815721 0.008232 5.12e-09 35356 TRUE reported
textfile 2 1 TRUE
6248 rs1619994 G A G A 0.063 0.0168 0.2807 NA FALSE FALSE FALSE
GCST006061 0.0078 0.03057 537409 outcome TRUE reported 9960_2_TBC1D5_TBCD5
TBC1D5 chr3 165744047 0.008901 1.465e-12 35339 TRUE reported
textfile 2 1 TRUE
6249 rs4980320 C T C T 0.0482 0.0041 0.46986 NA FALSE FALSE FALSE
GCST006061 0.0076 0.5858 537409 outcome TRUE reported 9960_2_TBC1D5_TBCD5
TBC1D5 chr11 198986 0.008022 1.872e-09 35352 TRUE reported textfile
2 1 TRUE
6250 rs10876550 A G A G 0.055 -1.00E-04 0.43699 NA FALSE FALSE FALSE
GCST006061 0.0073 0.991 537409 outcome TRUE reported 13450_49_USP8_UBP8
USP8 chr12 54318524 0.00811 1.19e-11 35368 TRUE reported textfile
2 1 TRUE
6251 rs1354034 C T C T 0.0742 0.0088 0.38119 NA FALSE FALSE FALSE
GCST006061 0.0073 0.2248 537409 outcome TRUE reported 13450_49_USP8_UBP8
USP8 chr3 56815721 0.008259 2.6e-19 35350 TRUE reported textfile
2 1 TRUE
6252 rs185009887 A G A G -0.1471 -0.0595 0.02519 NA FALSE FALSE FALSE
GCST006061 0.0496 0.2302 537409 outcome TRUE reported 13450_49_USP8_UBP8
USP8 chr17 5848332 0.026058 1.65e-08 35368 TRUE reported textfile
2 1 TRUE
6253 rs4980320 C T C T 0.0507 0.0041 0.46986 NA FALSE FALSE FALSE
GCST006061 0.0076 0.5858 537409 outcome TRUE reported 13450_49_USP8_UBP8
USP8 chr11 198986 0.008052 3.039e-10 35346 TRUE reported textfile
2 1 TRUE
6254 rs62019074 C T C T 0.1017 0.0114 0.16136 NA FALSE FALSE FALSE
GCST006061 0.01 0.2562 537409 outcome TRUE reported 13450_49_USP8_UBP8
USP8 chr15 50433826 0.010906 1.104e-20 35355 TRUE reported
textfile 2 1 TRUE
6255 rs78909033 A G A G -0.1969 -0.0167 0.16064 NA FALSE FALSE FALSE
GCST006061 0.0117 0.1526 537409 outcome TRUE reported 13450_49_USP8_UBP8
USP8 chr2 240571486 0.010914 9.344e-73 35264 TRUE reported
textfile 2 1 TRUE
6256 rs10876550 A G A G 0.0562 -1.00E-04 0.43699 NA FALSE FALSE FALSE
GCST006061 0.0073 0.991 537409 outcome TRUE reported 13943_38_DPY30_DPY30
DPY30 chr12 54318524 0.008021 2.437e-12 35374 TRUE reported
textfile 2 1 TRUE
6257 rs1355538 G A G A -0.0598 -0.0076 0.39847 NA FALSE FALSE FALSE
GCST006061 0.0072 0.2923 537409 outcome TRUE reported 13943_38_DPY30_DPY30
DPY30 chr3 165787389 0.008094 1.488e-13 35338 TRUE reported

textfile 2 1 TRUE
6258 rs221205 C T C T -0.1017 0.0249 0.11518 NA FALSE FALSE FALSE
GCST006061 0.0119 0.03693 537409 outcome TRUE reported 13943_38_DPY30_DPY30
DPY30 chr2 31861868 0.0123 1.358e-16 35363 TRUE reported textfile
2 1 TRUE
6259 rs4610723 G A G A -0.0583 -6.00E-04 0.17937 NA FALSE FALSE FALSE
GCST006061 0.01 0.9548 537409 outcome TRUE reported 13943_38_DPY30_DPY30
DPY30 chr8 140517207 0.010321 1.617e-08 35325 TRUE reported
textfile 2 1 TRUE
6260 rs72806804 G A G A 0.1161 -0.0015 0.04023 NA FALSE FALSE FALSE
GCST006061 0.0256 0.9522 537409 outcome TRUE reported 13943_38_DPY30_DPY30
DPY30 chr5 141226874 0.0206 1.742e-08 35365 TRUE reported textfile
2 1 TRUE
6261 rs10876550 A G A G 0.054 -1.00E-04 0.43699 NA FALSE FALSE FALSE
GCST006061 0.0073 0.991 537409 outcome TRUE reported 19620_16_DLG2_DLG2
DLG2 chr12 54318524 0.008093 2.519e-11 35374 TRUE reported
textfile 2 1 TRUE
6262 rs11601665 T C T C 0.0508 -0.0045 0.26367 NA FALSE FALSE FALSE
GCST006061 0.0088 0.6088 537409 outcome TRUE reported 19620_16_DLG2_DLG2
DLG2 chr11 262930 0.009054 2.014e-08 35352 TRUE reported textfile
2 1 TRUE
6263 rs1354034 C T C T 0.0652 0.0088 0.38119 NA FALSE FALSE FALSE
GCST006061 0.0073 0.2248 537409 outcome TRUE reported 19620_16_DLG2_DLG2
DLG2 chr3 56815721 0.008248 2.673e-15 35356 TRUE reported
textfile 2 1 TRUE
6264 rs185009887 A G A G -0.1498 -0.0595 0.02519 NA FALSE FALSE FALSE
GCST006061 0.0496 0.2302 537409 outcome TRUE reported 19620_16_DLG2_DLG2
DLG2 chr17 5848332 0.025991 8.237e-09 35374 TRUE reported textfile
2 1 TRUE
6265 rs78909033 A G A G -0.1161 -0.0167 0.16064 NA FALSE FALSE FALSE
GCST006061 0.0117 0.1526 537409 outcome TRUE reported 19620_16_DLG2_DLG2
DLG2 chr2 240571486 0.010908 1.874e-26 35270 TRUE reported
textfile 2 1 TRUE
6266 rs10876550 A G A G 0.0505 -1.00E-04 0.43699 NA FALSE FALSE FALSE
GCST006061 0.0073 0.991 537409 outcome TRUE reported 4212_5_EPS15L1_EP15R
EPS15L1 chr12 54318524 0.008141 5.529e-10 35374 TRUE reported
textfile 2 1 TRUE
6267 rs1354034 C T C T 0.0635 0.0088 0.38119 NA FALSE FALSE FALSE
GCST006061 0.0073 0.2248 537409 outcome TRUE reported 4212_5_EPS15L1_EP15R
EPS15L1 chr3 56815721 0.008284 1.785e-14 35356 TRUE reported
textfile 2 1 TRUE
6268 rs185009887 A G A G -0.1436 -0.0595 0.02519 NA FALSE FALSE FALSE
GCST006061 0.0496 0.2302 537409 outcome TRUE reported 4212_5_EPS15L1_EP15R
EPS15L1 chr17 5848332 0.026113 3.816e-08 35374 TRUE reported textfile
2 1 TRUE
6269 rs4980320 C T C T 0.0523 0.0041 0.46986 NA FALSE FALSE FALSE
GCST006061 0.0076 0.5858 537409 outcome TRUE reported 4212_5_EPS15L1_EP15R
EPS15L1 chr11 198986 0.008069 9.083e-11 35352 TRUE reported textfile
2 1 TRUE
6270 rs10876550 A G A G 0.0515 -1.00E-04 0.43699 NA FALSE FALSE FALSE
GCST006061 0.0073 0.991 537409 outcome TRUE reported 11307_33_WWP1_WWP1
WWP1 chr12 54318524 0.008173 2.95e-10 35365 TRUE reported
textfile 2 1 TRUE
6271 rs1354034 C T C T 0.0891 0.0088 0.38119 NA FALSE FALSE FALSE
GCST006061 0.0073 0.2248 537409 outcome TRUE reported 11307_33_WWP1_WWP1
WWP1 chr3 56815721 0.00832 9.186e-27 35347 TRUE reported textfile
2 1 TRUE
6272 rs4980320 C T C T 0.06 0.0041 0.46986 NA FALSE FALSE FALSE
GCST006061 0.0076 0.5858 537409 outcome TRUE reported 11307_33_WWP1_WWP1
WWP1 chr11 198986 0.008105 1.336e-13 35343 TRUE reported textfile
2 1 TRUE
6273 rs7073753 T C T C 0.0455 -0.0472 0.4356 NA FALSE FALSE FALSE
GCST006061 0.0072 5.02e-11 537409 outcome TRUE reported 11307_33_WWP1_WWP1
WWP1 chr10 63303060 0.008157 2.431e-08 35342 TRUE reported
textfile 2 1 TRUE
6274 rs10876550 A G A G 0.0734 -1.00E-04 0.43699 NA FALSE FALSE FALSE

GCST006061 0.0073 0.991 537409 outcome TRUE reported 19238_12_GLUL_GLNA
GLUL chr12 54318524 0.008153 2.201e-19 35374 TRUE reported
textfile 2 1 TRUE
6275 rs114694170 C T C T 0.0902 0.0053 0.08378 NA FALSE FALSE FALSE
GCST006061 0.0175 0.7624 537409 outcome TRUE reported 19238_12_GLUL_GLNA
GLUL chr5 88884379 0.01464 7.214e-10 35344 TRUE reported textfile
2 1 TRUE
6276 rs10876550 A G A G 0.0456 -1.00E-04 0.43699 NA FALSE FALSE FALSE
GCST006061 0.0073 0.991 537409 outcome TRUE reported 2855_49_MAPK3_ERK_1
MAPK3 chr12 54318524 0.008191 2.587e-08 35374 TRUE reported
textfile 2 1 TRUE
6277 rs117198781 A C A C 0.1047 -0.022 0.05929 NA FALSE FALSE FALSE
GCST006061 0.0174 0.2059 537409 outcome TRUE reported 2855_49_MAPK3_ERK_1
MAPK3 chr16 28512616 0.017046 8.134e-10 35386 TRUE reported
textfile 2 1 TRUE
6278 rs10876550 A G A G 0.0464 -1.00E-04 0.43699 NA FALSE FALSE FALSE
GCST006061 0.0073 0.991 537409 outcome TRUE reported 9913_4_RRBP1_RRBP1
RRBP1 chr12 54318524 0.008022 7.278e-09 35374 TRUE reported
textfile 2 1 TRUE
6279 rs11227136 A G A G -0.0868 -0.0129 0.25007 NA FALSE FALSE FALSE
GCST006061 0.0087 0.1386 537409 outcome TRUE reported 9913_4_RRBP1_RRBP1
RRBP1 chr11 65164493 0.009175 3.061e-21 35363 TRUE reported
textfile 2 1 TRUE
6280 rs1354034 C T C T 0.0752 0.0088 0.38119 NA FALSE FALSE FALSE
GCST006061 0.0073 0.2248 537409 outcome TRUE reported 9913_4_RRBP1_RRBP1
RRBP1 chr3 56815721 0.008165 3.263e-20 35356 TRUE reported
textfile 2 1 TRUE
6281 rs1707652 T C T C 0.0718 0.0081 0.3102 NA FALSE FALSE FALSE
GCST006061 0.0074 0.2701 537409 outcome TRUE reported 17706_4_PPP1R1A_PPR1A
PPP1R1A chr3 165761011 0.008743 2.163e-16 35339 TRUE reported
textfile 2 1 TRUE
6282 rs10878824 G A G A 0.0545 -0.0036 0.44744 NA FALSE FALSE FALSE
GCST006061 0.0073 0.6222 537409 outcome TRUE reported 15686_49_INHBC_INHBC
INHBC chr12 68414668 0.008546 1.807e-10 35362 TRUE reported
textfile 2 1 TRUE
6283 rs113953523 A G A G -0.8216 0.033 0.02879 NA FALSE FALSE FALSE
GCST006061 0.0374 0.3779 537409 outcome TRUE reported 15686_49_INHBC_INHBC
INHBC chr12 57146051 0.025456 1e-200 35365 TRUE reported textfile
2 1 TRUE
6284 rs117176626 A G A G -0.6443 -0.0454 0.02982 NA FALSE FALSE FALSE
GCST006061 0.0336 0.1763 537409 outcome TRUE reported 15686_49_INHBC_INHBC
INHBC chr12 58091429 0.024731 1.273e-149 35365 TRUE reported
textfile 2 1 TRUE
6285 rs117325986 T C T C -0.7239 2e-04 0.03741 NA FALSE FALSE FALSE
GCST006061 0.0384 0.9948 537409 outcome TRUE reported 15686_49_INHBC_INHBC
INHBC chr12 56603971 0.02176 1e-200 35369 TRUE reported textfile 2
1 TRUE
6286 rs12816210 A C A C -0.2029 -0.0105 0.03649 NA FALSE FALSE FALSE
GCST006061 0.0251 0.6766 537409 outcome TRUE reported 15686_49_INHBC_INHBC
INHBC chr12 58578453 0.022379 1.228e-19 35366 TRUE reported
textfile 2 1 TRUE
6287 rs147618567 T C T C -0.1891 -0.0031 0.01714 NA FALSE FALSE FALSE
GCST006061 0.0485 0.9495 537409 outcome TRUE reported 15686_49_INHBC_INHBC
INHBC chr12 69915396 0.032481 5.819e-09 35366 TRUE reported
textfile 2 1 TRUE
6288 rs149717212 C A C A -0.2768 -0.0028 0.03037 NA FALSE FALSE FALSE
GCST006061 0.0434 0.9477 537409 outcome TRUE reported 15686_49_INHBC_INHBC
INHBC chr12 55609104 0.024103 1.591e-30 35379 TRUE reported
textfile 2 1 TRUE
6289 rs150291370 C T C T -0.3192 -0.0309 0.01553 NA FALSE FALSE FALSE
GCST006061 0.0349 0.3755 537409 outcome TRUE reported 15686_49_INHBC_INHBC
INHBC chr12 58500711 0.033299 9.161e-22 35367 TRUE reported
textfile 2 1 TRUE
6290 rs183310037 A C A C 0.3637 0.0211 0.03362 NA FALSE FALSE FALSE
GCST006061 0.0336 0.5306 537409 outcome TRUE reported 15686_49_INHBC_INHBC
INHBC chr12 56654023 0.023718 4.513e-53 35369 TRUE reported

textfile 2 1 TRUE
6291 rs34633617 G A G A 0.3825 -0.0084 0.04605 NA FALSE FALSE FALSE
GCST006061 0.0246 0.7341 537409 outcome TRUE reported 15686_49_INHBC_INHBC
INHBC chr12 57909704 0.020082 7.014e-81 35366 TRUE reported
textfile 2 1 TRUE
6292 rs76182087 G A G A -0.1309 0.0015 0.06537 NA FALSE FALSE FALSE
GCST006061 0.0163 0.9264 537409 outcome TRUE reported 15686_49_INHBC_INHBC
INHBC chr12 69036629 0.016951 1.142e-14 35363 TRUE reported
textfile 2 1 TRUE
6293 rs76545764 C A C A -0.5217 0.0168 0.06949 NA FALSE FALSE FALSE
GCST006061 0.0196 0.3898 537409 outcome TRUE reported 15686_49_INHBC_INHBC
INHBC chr12 56859027 0.016409 1e-200 35369 TRUE reported textfile
2 1 TRUE
6294 rs78188443 T C T C 0.2315 -0.0224 0.01316 NA FALSE FALSE FALSE
GCST006061 0.0405 0.5802 537409 outcome TRUE reported 15686_49_INHBC_INHBC
INHBC chr12 56138509 0.037578 7.254e-10 35367 TRUE reported
textfile 2 1 TRUE
6295 rs79245439 C T C T 0.4087 0.0527 0.01234 NA FALSE FALSE FALSE
GCST006061 0.0304 0.08352 537409 outcome TRUE reported 15686_49_INHBC_INHBC
INHBC chr12 57277839 0.037677 2.05e-27 35367 TRUE reported
textfile 2 1 TRUE
6296 rs10882283 C A C A -0.0855 0.0122 0.35748 NA FALSE FALSE FALSE
GCST006061 0.008 0.1295 537409 outcome TRUE reported 15633_6_RBP4_RBP
RBP4 chr10 93601207 0.008568 1.891e-23 35369 TRUE reported
textfile 2 1 TRUE
6297 rs1667244 G A G A 0.0739 0.0031 0.4113 NA FALSE FALSE FALSE
GCST006061 0.0073 0.6712 537409 outcome TRUE reported 15633_6_RBP4_RBP
RBP4 chr18 31587942 0.00837 1.052e-18 35349 TRUE reported textfile
2 1 TRUE
6298 rs2126263 A G A G 0.0901 -0.0133 0.07874 NA FALSE FALSE FALSE
GCST006061 0.0124 0.2821 537409 outcome TRUE reported 15633_6_RBP4_RBP
RBP4 chr8 9324101 0.015261 3.547e-09 35385 TRUE reported textfile
2 1 TRUE
6299 rs4243177 G A G A 0.0473 -0.0055 0.34021 NA FALSE FALSE FALSE
GCST006061 0.0073 0.4525 537409 outcome TRUE reported 15633_6_RBP4_RBP
RBP4 chr16 79669932 0.00866 4.717e-08 35371 TRUE reported textfile
2 1 TRUE
6300 rs780093 C T C T -0.054 -0.0074 0.34157 NA FALSE FALSE FALSE
GCST006061 0.0073 0.3116 537409 outcome TRUE reported 15633_6_RBP4_RBP
RBP4 chr2 27519736 0.008689 5.139e-10 35373 TRUE reported
textfile 2 1 TRUE
6301 rs10883451 C T C T -0.0469 0.0055 0.49527 NA FALSE FALSE FALSE
GCST006061 0.0074 0.4551 537409 outcome TRUE reported 8229_1_GXYLT1_GXLT1
GXYLT1 chr10 100164661 0.007911 3.062e-09 35368 TRUE reported
textfile 2 1 TRUE
6302 rs113355147 A C A C -0.1931 -0.0496 0.02084 NA FALSE FALSE FALSE
GCST006061 0.0473 0.2945 537409 outcome TRUE reported 8229_1_GXYLT1_GXLT1
GXYLT1 chr12 41851140 0.02883 2.114e-11 35364 TRUE reported textfile
2 1 TRUE
6303 rs117903281 T C T C 0.3231 -0.0395 0.04765 NA FALSE FALSE FALSE
GCST006061 0.0256 0.1229 537409 outcome TRUE reported 8229_1_GXYLT1_GXLT1
GXYLT1 chr12 41276395 0.018854 7.908e-66 35354 TRUE reported
textfile 2 1 TRUE
6304 rs118141779 A G A G 0.3737 -0.0671 0.01447 NA FALSE FALSE FALSE
GCST006061 0.0557 0.2286 537409 outcome TRUE reported 8229_1_GXYLT1_GXLT1
GXYLT1 chr12 32344773 0.034021 4.539e-28 35361 TRUE reported
textfile 2 1 TRUE
6305 rs139485922 T C T C 0.3436 -0.0304 0.01483 NA FALSE FALSE FALSE
GCST006061 0.043 0.4792 537409 outcome TRUE reported 8229_1_GXYLT1_GXLT1
GXYLT1 chr12 40228156 0.033891 3.728e-24 35351 TRUE reported
textfile 2 1 TRUE
6306 rs139974673 C T C T 0.2225 0.0351 0.02734 NA FALSE FALSE FALSE
GCST006061 0.0241 0.1443 537409 outcome TRUE reported 8229_1_GXYLT1_GXLT1
GXYLT1 chr15 43735687 0.024181 3.527e-20 35362 TRUE reported
textfile 2 1 TRUE
6307 rs140624338 C T C T 0.432 0.0021 0.01047 NA FALSE FALSE FALSE

GCST006061 0.0539 0.9695 537409 outcome TRUE reported 8229_1_GXYLT1_GXLT1
GXYLT1 chr12 101963654 0.040121 4.912e-27 35362 TRUE reported
textfile 2 1 TRUE
6308 rs144355501 G T G T 0.3349 0.0234 0.02038 NA FALSE FALSE FALSE
GCST006061 0.0409 0.5668 537409 outcome TRUE reported 8229_1_GXYLT1_GXLT1
GXYLT1 chr12 40847044 0.028857 3.872e-31 35353 TRUE reported
textfile 2 1 TRUE
6309 rs1711171 C T C T 0.0516 -0.0217 0.23991 NA FALSE FALSE FALSE
GCST006061 0.0083 0.009298 537409 outcome TRUE reported 8229_1_GXYLT1_GXLT1
GXYLT1 chr3 136301699 0.009325 3.142e-08 35361 TRUE reported
textfile 2 1 TRUE
6310 rs2706333 C T C T -0.1646 0.0149 0.02368 NA FALSE FALSE FALSE
GCST006061 0.0222 0.5033 537409 outcome TRUE reported 8229_1_GXYLT1_GXLT1
GXYLT1 chr12 43161712 0.026798 8.134e-10 35359 TRUE reported
textfile 2 1 TRUE
6311 rs351973 G A G A 0.0457 0.0022 0.39647 NA FALSE FALSE FALSE
GCST006061 0.0091 0.8119 537409 outcome TRUE reported 8229_1_GXYLT1_GXLT1
GXYLT1 chr19 795457 0.00811 1.751e-08 35305 TRUE reported textfile 2
1 TRUE
6312 rs4764822 T C T C -0.3135 0.0045 0.2787 NA FALSE FALSE FALSE
GCST006061 0.0078 0.564 537409 outcome TRUE reported 8229_1_GXYLT1_GXLT1
GXYLT1 chr12 101822895 0.00905 1e-200 35364 TRUE reported textfile 2
1 TRUE
6313 rs56272941 C T C T -0.1143 -0.0107 0.0513 NA FALSE FALSE FALSE
GCST006061 0.0261 0.6813 537409 outcome TRUE reported 8229_1_GXYLT1_GXLT1
GXYLT1 chr12 102702636 0.018506 6.561e-10 35363 TRUE reported
textfile 2 1 TRUE
6314 rs77827196 C T C T 0.1845 0.0126 0.0506 NA FALSE FALSE FALSE
GCST006061 0.0232 0.5862 537409 outcome TRUE reported 8229_1_GXYLT1_GXLT1
GXYLT1 chr12 43692000 0.018197 3.701e-24 35357 TRUE reported
textfile 2 1 TRUE
6315 rs7960537 T C T C 0.156 0.0121 0.04474 NA FALSE FALSE FALSE
GCST006061 0.0194 0.5331 537409 outcome TRUE reported 8229_1_GXYLT1_GXLT1
GXYLT1 chr12 41979063 0.019717 2.535e-15 35364 TRUE reported
textfile 2 1 TRUE
6316 rs825038 A C A C 0.1845 0.0218 0.05118 NA FALSE FALSE FALSE
GCST006061 0.0239 0.3624 537409 outcome TRUE reported 8229_1_GXYLT1_GXLT1
GXYLT1 chr12 101570303 0.01849 1.893e-23 35363 TRUE reported textfile
2 1 TRUE
6317 rs891488 C T C T 0.0625 0.0134 0.15634 NA FALSE FALSE FALSE
GCST006061 0.0089 0.1336 537409 outcome TRUE reported 8229_1_GXYLT1_GXLT1
GXYLT1 chr18 79877424 0.010899 9.785e-09 34950 TRUE reported
textfile 2 1 TRUE
6318 rs956886 T C T C -0.0558 -0.0104 0.3139 NA FALSE FALSE FALSE
GCST006061 0.0078 0.1823 537409 outcome TRUE reported 8229_1_GXYLT1_GXLT1
GXYLT1 chr12 104560803 0.008794 2.222e-10 35360 TRUE reported
textfile 2 1 TRUE
6319 rs97384 C T C T 0.0525 -0.0095 0.41277 NA FALSE FALSE FALSE GCST006061
0.0075 0.205 537409 outcome TRUE reported 8229_1_GXYLT1_GXLT1 GXYLT1 chr11
61856709 0.008062 7.398e-11 35366 TRUE reported textfile 2 1 TRUE
6320 rs10883974 C T C T -0.4293 -0.0219 0.25464 NA FALSE FALSE FALSE
GCST006061 0.0084 0.009012 537409 outcome TRUE reported
12436_84_GSTO1_GST_omega_1 GSTO1 chr10 104167148 0.006732 1e-200 35364
TRUE reported textfile 2 1 TRUE
6321 rs113474709 A G A G -0.2493 -0.0231 0.03091 NA FALSE FALSE FALSE
GCST006061 0.0237 0.3294 537409 outcome TRUE reported
12436_84_GSTO1_GST_omega_1 GSTO1 chr10 101131923 0.023491 2.601e-26 35358
TRUE reported textfile 2 1 TRUE
6322 rs11817165 C A C A 0.2028 -0.0234 0.0293 NA FALSE FALSE FALSE
GCST006061 0.0173 0.1756 537409 outcome TRUE reported
12436_84_GSTO1_GST_omega_1 GSTO1 chr10 103227159 0.024196 5.216e-17 35361
TRUE reported textfile 2 1 TRUE
6323 rs140911275 C T C T -0.5101 -0.0349 0.01348 NA FALSE FALSE FALSE
GCST006061 0.0543 0.5208 537409 outcome TRUE reported
12436_84_GSTO1_GST_omega_1 GSTO1 chr10 105943325 0.0338 1.836e-51 35358
TRUE reported textfile 2 1 TRUE

6324 rs142707907 A C A C -0.1843 -0.0355 0.02837 NA FALSE FALSE FALSE
GCST006061 0.0335 0.2888 537409 outcome TRUE reported
12436_84_GSTO1_GST_omega_1 GSTO1 chr10 103275967 0.024665 7.897e-14 35361
TRUE reported textfile 2 1 TRUE
6325 rs146191686 T C T C -0.2131 -0.0295 0.02355 NA FALSE FALSE FALSE
GCST006061 0.0319 0.3553 537409 outcome TRUE reported
12436_84_GSTO1_GST_omega_1 GSTO1 chr10 102094866 0.027047 3.301e-15 35360
TRUE reported textfile 2 1 TRUE
6326 rs1490171 G A G A -0.0983 -0.0035 0.46187 NA FALSE FALSE FALSE
GCST006061 0.0073 0.632 537409 outcome TRUE reported
12436_84_GSTO1_GST_omega_1 GSTO1 chr10 104790142 0.008151 1.726e-33 35362
TRUE reported textfile 2 1 TRUE
6327 rs185795781 A G A G -0.187 0.0078 0.03226 NA FALSE FALSE FALSE
GCST006061 0.0218 0.7208 537409 outcome TRUE reported
12436_84_GSTO1_GST_omega_1 GSTO1 chr10 106197828 0.022731 1.924e-16 35356
TRUE reported textfile 2 1 TRUE
6328 rs1881451 G A G A 0.0489 -0.0119 0.30166 NA FALSE FALSE FALSE
GCST006061 0.0087 0.1722 537409 outcome TRUE reported
12436_84_GSTO1_GST_omega_1 GSTO1 chr20 26170355 0.00817 2.157e-09 35356
TRUE reported textfile 2 1 TRUE
6329 rs3788375 A G A G 0.0568 -0.0046 0.33453 NA FALSE FALSE FALSE
GCST006061 0.0077 0.5509 537409 outcome TRUE reported
12436_84_GSTO1_GST_omega_1 GSTO1 chr22 24600471 0.007969 1.018e-12 35358
TRUE reported textfile 2 1 TRUE
6330 rs4917404 C T C T 0.1137 0.018 0.41303 NA FALSE FALSE FALSE
GCST006061 0.0073 0.01395 537409 outcome TRUE reported
12436_84_GSTO1_GST_omega_1 GSTO1 chr10 103871368 0.0082 1.012e-43 35360
TRUE reported textfile 2 1 TRUE
6331 rs61870857 T C T C -0.2132 0.032 0.02005 NA FALSE FALSE FALSE
GCST006061 0.0337 0.3414 537409 outcome TRUE reported
12436_84_GSTO1_GST_omega_1 GSTO1 chr10 103206162 0.029109 2.405e-13 35361
TRUE reported textfile 2 1 TRUE
6332 rs61871328 A G A G -0.1816 -0.0418 0.02263 NA FALSE FALSE FALSE
GCST006061 0.0251 0.09603 537409 outcome TRUE reported
12436_84_GSTO1_GST_omega_1 GSTO1 chr10 111657591 0.027577 4.543e-11 35364
TRUE reported textfile 2 1 TRUE
6333 rs75950986 T G T G -0.2029 -0.0667 0.03052 NA FALSE FALSE FALSE
GCST006061 0.0281 0.01773 537409 outcome TRUE reported
12436_84_GSTO1_GST_omega_1 GSTO1 chr10 102655933 0.023053 1.35e-18 35361
TRUE reported textfile 2 1 TRUE
6334 rs78837000 T C T C 0.2196 -0.0073 0.01708 NA FALSE FALSE FALSE
GCST006061 0.0282 0.7949 537409 outcome TRUE reported
12436_84_GSTO1_GST_omega_1 GSTO1 chr10 102593155 0.030818 1.036e-12 35361
TRUE reported textfile 2 1 TRUE
6335 rs805670 T C T C -0.2443 -0.0094 0.43989 NA FALSE FALSE FALSE
GCST006061 0.0073 0.1997 537409 outcome TRUE reported
12436_84_GSTO1_GST_omega_1 GSTO1 chr10 103979110 0.008064 1e-200 35362
TRUE reported textfile 2 1 TRUE
6336 rs10886437 T C T C -0.0884 -0.0056 0.18137 NA FALSE FALSE FALSE
GCST006061 0.0097 0.5597 537409 outcome TRUE reported 3508_78_CCL22_MDC
CCL22 chr10 119277642 0.010629 9.044e-17 35379 TRUE reported
textfile 2 1 TRUE
6337 rs11129978 T C T C -0.0767 0.0158 0.32875 NA FALSE FALSE FALSE
GCST006061 0.0076 0.03609 537409 outcome TRUE reported 3508_78_CCL22_MDC
CCL22 chr3 42842084 0.008752 1.89e-18 35353 TRUE reported
textfile 2 1 TRUE
6338 rs1123507 T C T C -0.093 -0.0138 0.13183 NA FALSE FALSE FALSE
GCST006061 0.012 0.2504 537409 outcome TRUE reported 3508_78_CCL22_MDC
CCL22 chr16 56871338 0.012296 3.927e-14 35369 TRUE reported
textfile 2 1 TRUE
6339 rs11697497 A G A G -0.0752 0.0113 0.13186 NA FALSE FALSE FALSE
GCST006061 0.014 0.4164 537409 outcome TRUE reported 3508_78_CCL22_MDC
CCL22 chr20 63757025 0.012318 1.03e-09 35247 TRUE reported
textfile 2 1 TRUE
6340 rs192297333 A G A G 0.1323 0.0242 0.03761 NA FALSE FALSE FALSE
GCST006061 0.0325 0.4575 537409 outcome TRUE reported 3508_78_CCL22_MDC

CCL22 chr16 57476427 0.021947 1.657e-09 35372 TRUE reported
textfile 2 1 TRUE
6341 rs289750 C T C T -0.0741 -0.0092 0.33473 NA FALSE FALSE FALSE
GCST006061 0.008 0.249 537409 outcome TRUE reported 3508_78_CCL22_MDC
CCL22 chr16 56992852 0.008823 4.507e-17 35371 TRUE reported
textfile 2 1 TRUE
6342 rs60066732 G A G A -0.0544 -0.0045 0.34847 NA FALSE FALSE FALSE
GCST006061 0.0076 0.5583 537409 outcome TRUE reported 3508_78_CCL22_MDC
CCL22 chr6 90225664 0.008681 3.695e-10 35344 TRUE reported
textfile 2 1 TRUE
6343 rs62037104 A C A C -0.651 -0.0226 0.02622 NA FALSE FALSE FALSE
GCST006061 0.0293 0.4394 537409 outcome TRUE reported 3508_78_CCL22_MDC
CCL22 chr16 57401753 0.025649 4.027e-142 35373 TRUE reported
textfile 2 1 TRUE
6344 rs6808893 T C T C -0.0591 -0.0123 0.45934 NA FALSE FALSE FALSE
GCST006061 0.0074 0.09503 537409 outcome TRUE reported 3508_78_CCL22_MDC
CCL22 chr3 188415651 0.008234 7.082e-13 35373 TRUE reported
textfile 2 1 TRUE
6345 rs71375907 G T G T -0.2383 3e-04 0.08794 NA FALSE FALSE FALSE
GCST006061 0.0139 0.9856 537409 outcome TRUE reported 3508_78_CCL22_MDC
CCL22 chr16 57286010 0.014839 4.933e-58 35373 TRUE reported
textfile 2 1 TRUE
6346 rs77542162 G A G A 0.5502 0.0177 0.025 NA FALSE FALSE FALSE
GCST006061 0.0302 0.557 537409 outcome TRUE reported 3508_78_CCL22_MDC
CCL22 chr17 69085137 0.025822 9.636e-101 35371 TRUE reported
textfile 2 1 TRUE
6347 rs9921051 A G A G 0.266 0.0063 0.05923 NA FALSE FALSE FALSE
GCST006061 0.015 0.6753 537409 outcome TRUE reported 3508_78_CCL22_MDC
CCL22 chr16 57340522 0.017403 9.723e-53 35373 TRUE reported
textfile 2 1 TRUE
6348 rs10889352 C T C T -0.2189 -0.025 0.33773 NA FALSE FALSE FALSE
GCST006061 0.0076 0.0009663 537409 outcome TRUE reported
10391_1_ANGPTL3_ANGL3 ANGPTL3 chr1 62633352 0.008507 5.121e-146 35346
TRUE reported textfile 2 1 TRUE
6349 rs113939762 G T G T -0.158 -0.0092 0.04279 NA FALSE FALSE FALSE
GCST006061 0.0221 0.677 537409 outcome TRUE reported 10391_1_ANGPTL3_ANGL3
ANGPTL3 chr1 63583261 0.020156 4.549e-15 35347 TRUE reported
textfile 2 1 TRUE
6350 rs115365379 C T C T -0.6289 -0.0666 0.01178 NA FALSE FALSE FALSE
GCST006061 0.05 0.1828 537409 outcome TRUE reported 10391_1_ANGPTL3_ANGL3
ANGPTL3 chr1 63830105 0.03726 6.453e-64 35341 TRUE reported textfile
2 1 TRUE
6351 rs116843064 A G A G 0.1472 0.0449 0.02426 NA FALSE FALSE FALSE
GCST006061 0.0321 0.1613 537409 outcome TRUE reported 10391_1_ANGPTL3_ANGL3
ANGPTL3 chr19 8364439 0.02579 1.146e-08 35363 TRUE reported textfile 2
1 TRUE
6352 rs12130644 T C T C -0.0711 -0.0243 0.12561 NA FALSE FALSE FALSE
GCST006061 0.0115 0.03421 537409 outcome TRUE reported 10391_1_ANGPTL3_ANGL3
ANGPTL3 chr1 73428961 0.01214 4.726e-09 35339 TRUE reported textfile
2 1 TRUE
6353 rs138617180 G T G T -0.3843 -0.0514 0.01967 NA FALSE FALSE FALSE
GCST006061 0.0386 0.1825 537409 outcome TRUE reported 10391_1_ANGPTL3_ANGL3
ANGPTL3 chr1 61058312 0.029336 3.298e-39 35348 TRUE reported
textfile 2 1 TRUE
6354 rs144996885 G A G A -0.2094 0.0401 0.01765 NA FALSE FALSE FALSE
GCST006061 0.0404 0.3201 537409 outcome TRUE reported 10391_1_ANGPTL3_ANGL3
ANGPTL3 chr1 63494584 0.030016 3.031e-12 35347 TRUE reported
textfile 2 1 TRUE
6355 rs17122460 T G T G -0.0952 -0.0097 0.08477 NA FALSE FALSE FALSE
GCST006061 0.0114 0.3943 537409 outcome TRUE reported 10391_1_ANGPTL3_ANGL3
ANGPTL3 chr1 61594615 0.014526 5.615e-11 35349 TRUE reported
textfile 2 1 TRUE
6356 rs17123220 A G A G -0.7903 -0.03 0.01048 NA FALSE FALSE FALSE
GCST006061 0.0282 0.2867 537409 outcome TRUE reported 10391_1_ANGPTL3_ANGL3
ANGPTL3 chr1 62238014 0.038998 2.612e-91 35345 TRUE reported
textfile 2 1 TRUE

6357 rs2119690 A G A G 0.0522 0.0127 0.29756 NA FALSE FALSE FALSE
GCST006061 0.0079 0.1069 537409 outcome TRUE reported 10391_1_ANGPTL3_ANGL3
ANGPTL3 chr8 20002028 0.008733 2.267e-09 35356 TRUE reported
textfile 2 1 TRUE
6358 rs10889353 C A C A -0.05 -0.0244 0.34024 NA FALSE FALSE FALSE
GCST006061 0.0076 0.001249 537409 outcome TRUE reported
13022_20_TP53I11_P5I11 TP53I11 chr1 62652525 0.008793 1.3e-08 35349 TRUE
reported textfile 2 1 TRUE
6359 rs141469619 G A G A 0.2345 -0.0376 0.01201 NA FALSE FALSE FALSE
GCST006061 0.0531 0.4786 537409 outcome TRUE reported 13022_20_TP53I11_P5I11
TP53I11 chr11 116843577 0.037165 2.796e-10 35368 TRUE reported
textfile 2 1 TRUE
6360 rs1532085 G A G A -0.0571 0.0148 0.41111 NA FALSE FALSE FALSE
GCST006061 0.0073 0.04221 537409 outcome TRUE reported 13022_20_TP53I11_P5I11
TP53I11 chr15 58391167 0.008458 1.469e-11 35355 TRUE reported
textfile 2 1 TRUE
6361 rs1707652 T C T C 0.1018 0.0081 0.3102 NA FALSE FALSE FALSE
GCST006061 0.0074 0.2701 537409 outcome TRUE reported 13022_20_TP53I11_P5I11
TP53I11 chr3 165761011 0.008976 8.191e-30 35330 TRUE reported
textfile 2 1 TRUE
6362 rs261332 G A G A -0.0607 -0.0047 0.18171 NA FALSE FALSE FALSE
GCST006061 0.0087 0.587 537409 outcome TRUE reported 13022_20_TP53I11_P5I11
TP53I11 chr15 58435126 0.010759 1.683e-08 35357 TRUE reported
textfile 2 1 TRUE
6363 rs2678379 G A G A 0.06 0.0099 0.23322 NA FALSE FALSE FALSE
GCST006061 0.0085 0.2464 537409 outcome TRUE reported 13022_20_TP53I11_P5I11
TP53I11 chr2 21003688 0.009893 1.321e-09 35364 TRUE reported
textfile 2 1 TRUE
6364 rs283815 G A G A -0.3093 5e-04 0.21988 NA FALSE FALSE FALSE
GCST006061 0.0088 0.9539 537409 outcome TRUE reported 13022_20_TP53I11_P5I11
TP53I11 chr19 44887076 0.01014 1e-200 35359 TRUE reported textfile 2
1 TRUE
6365 rs2954038 A C A C -0.0585 0.0046 0.31787 NA FALSE FALSE FALSE
GCST006061 0.0078 0.5552 537409 outcome TRUE reported 13022_20_TP53I11_P5I11
TP53I11 chr8 125495147 0.008939 5.966e-11 35358 TRUE reported
textfile 2 1 TRUE
6366 rs62120574 T C T C 0.0888 0.0023 0.09184 NA FALSE FALSE FALSE
GCST006061 0.0151 0.8787 537409 outcome TRUE reported 13022_20_TP53I11_P5I11
TP53I11 chr19 44711506 0.014623 1.259e-09 35360 TRUE reported
textfile 2 1 TRUE
6367 rs66777718 G A G A -0.0856 -0.0121 0.16753 NA FALSE FALSE FALSE
GCST006061 0.0098 0.2151 537409 outcome TRUE reported 13022_20_TP53I11_P5I11
TP53I11 chr19 45898634 0.011387 5.596e-14 35362 TRUE reported
textfile 2 1 TRUE
6368 rs780093 C T C T -0.0755 -0.0074 0.34157 NA FALSE FALSE FALSE
GCST006061 0.0073 0.3116 537409 outcome TRUE reported 13022_20_TP53I11_P5I11
TP53I11 chr2 27519736 0.008798 9.334e-18 35364 TRUE reported
textfile 2 1 TRUE
6369 rs78810414 T C T C -0.0856 0.0257 0.08751 NA FALSE FALSE FALSE
GCST006061 0.012 0.03151 537409 outcome TRUE reported 13022_20_TP53I11_P5I11
TP53I11 chr8 20013602 0.014664 5.301e-09 35359 TRUE reported
textfile 2 1 TRUE
6370 rs10892301 A G A G -0.0504 0.0019 0.43417 NA FALSE FALSE FALSE
GCST006061 0.0072 0.7949 537409 outcome TRUE reported
10514_5_PTGDS_PGD2_synthase PTGDS chr11 118864767 0.008263 1.065e-09 35369
TRUE reported textfile 2 1 TRUE
6371 rs28611065 C T C T 0.0649 -0.0149 0.16415 NA FALSE FALSE FALSE
GCST006061 0.0097 0.1231 537409 outcome TRUE reported
10514_5_PTGDS_PGD2_synthase PTGDS chr17 2230277 0.011119 5.325e-09 35353
TRUE reported textfile 2 1 TRUE
6372 rs7040970 C T C T 0.141 0.0015 0.4943 NA FALSE FALSE FALSE
GCST006061 0.0073 0.834 537409 outcome TRUE reported
10514_5_PTGDS_PGD2_synthase PTGDS chr9 136964561 0.008163 7.433e-67 35250
TRUE reported textfile 2 1 TRUE
6373 rs72704449 C T C T 0.1066 0.013 0.04582 NA FALSE FALSE FALSE
GCST006061 0.0199 0.5128 537409 outcome TRUE reported

10514_5_PTGDS_PGD2_synthase PTGDS chr1 179504138 0.019492 4.526e-08 35346
TRUE reported textfile 2 1 TRUE
6374 rs77924615 A G A G -0.0805 0.0136 0.19701 NA FALSE FALSE FALSE
GCST006061 0.0092 0.1375 537409 outcome TRUE reported
10514_5_PTGDS_PGD2_synthase PTGDS chr16 20381010 0.01035 7.382e-15 35343
TRUE reported textfile 2 1 TRUE
6375 rs10893501 G A G A -0.1378 0.0045 0.10283 NA FALSE FALSE FALSE
GCST006061 0.0097 0.6441 537409 outcome TRUE reported 4564_2_PLXNC1_PLXC1
PLXNC1 chr11 126381138 0.013584 3.51e-24 35376 TRUE reported
textfile 2 1 TRUE
6376 rs11107427 A G A G 0.4018 -0.0019 0.03628 NA FALSE FALSE FALSE
GCST006061 0.027 0.9447 537409 outcome TRUE reported 4564_2_PLXNC1_PLXC1
PLXNC1 chr12 94179118 0.023136 1.461e-67 35368 TRUE reported
textfile 2 1 TRUE
6377 rs11108494 A G A G -0.1891 -0.0048 0.03816 NA FALSE FALSE FALSE
GCST006061 0.0218 0.8247 537409 outcome TRUE reported 4564_2_PLXNC1_PLXC1
PLXNC1 chr12 96412177 0.022742 9.166e-17 35359 TRUE reported
textfile 2 1 TRUE
6378 rs115651556 A G A G -2.0436 0.0337 0.01483 NA FALSE FALSE FALSE
GCST006061 0.0444 0.448 537409 outcome TRUE reported 4564_2_PLXNC1_PLXC1
PLXNC1 chr12 94220122 0.02548 1e-200 35368 TRUE reported textfile 2
1 TRUE
6379 rs116852327 T G T G 0.2854 -0.0213 0.01186 NA FALSE FALSE FALSE
GCST006061 0.0385 0.5806 537409 outcome TRUE reported 4564_2_PLXNC1_PLXC1
PLXNC1 chr17 7721481 0.035741 1.402e-15 35377 TRUE reported textfile
2 1 TRUE
6380 rs117077998 G A G A 0.1097 0.0015 0.05357 NA FALSE FALSE FALSE
GCST006061 0.0288 0.9585 537409 outcome TRUE reported 4564_2_PLXNC1_PLXC1
PLXNC1 chr12 96605997 0.019429 1.64e-08 35360 TRUE reported
textfile 2 1 TRUE
6381 rs117633773 A G A G 0.1313 0.0067 0.04197 NA FALSE FALSE FALSE
GCST006061 0.0224 0.7662 537409 outcome TRUE reported 4564_2_PLXNC1_PLXC1
PLXNC1 chr17 6953365 0.020425 1.289e-10 35374 TRUE reported textfile
2 1 TRUE
6382 rs117699264 A G A G 0.2407 -0.0028 0.02545 NA FALSE FALSE FALSE
GCST006061 0.0306 0.9266 537409 outcome TRUE reported 4564_2_PLXNC1_PLXC1
PLXNC1 chr12 94225784 0.028095 1.058e-17 35368 TRUE reported
textfile 2 1 TRUE
6383 rs12302104 A G A G -0.7202 -0.013 0.01235 NA FALSE FALSE FALSE
GCST006061 0.0399 0.7449 537409 outcome TRUE reported 4564_2_PLXNC1_PLXC1
PLXNC1 chr12 94935299 0.038889 1.446e-76 35368 TRUE reported
textfile 2 1 TRUE
6384 rs141186127 T C T C 0.2637 -0.0436 0.0128 NA FALSE FALSE FALSE
GCST006061 0.0375 0.2459 537409 outcome TRUE reported 4564_2_PLXNC1_PLXC1
PLXNC1 chr12 94573868 0.037508 2.059e-12 35367 TRUE reported
textfile 2 1 TRUE
6385 rs270862 G A G A -0.4053 -0.0225 0.02497 NA FALSE FALSE FALSE
GCST006061 0.0244 0.3558 537409 outcome TRUE reported 4564_2_PLXNC1_PLXC1
PLXNC1 chr12 93960209 0.027462 2.7e-49 35369 TRUE reported textfile
2 1 TRUE
6386 rs55714927 T C T C 0.1134 1e-04 0.21166 NA FALSE FALSE FALSE
GCST006061 0.0098 0.9926 537409 outcome TRUE reported 4564_2_PLXNC1_PLXC1
PLXNC1 chr17 7176997 0.010201 1.044e-28 35370 TRUE reported textfile
2 1 TRUE
6387 rs10893507 C A C A 0.0658 0.0104 0.48067 NA FALSE FALSE FALSE
GCST006061 0.0072 0.1524 537409 outcome TRUE reported 7185_29_GP5_GPV GP5
chr11 126416693 0.008074 3.663e-16 35370 TRUE reported textfile 2
1 TRUE
6388 rs115202389 A G A G -0.128 -0.0595 0.03854 NA FALSE FALSE FALSE
GCST006061 0.0229 0.009495 537409 outcome TRUE reported 7185_29_GP5_GPV GP5
chr3 194852972 0.020972 1.039e-09 35245 TRUE reported textfile 2
1 TRUE
6389 rs1466733 G A G A -0.1173 0.0035 0.23222 NA FALSE FALSE FALSE
GCST006061 0.0083 0.6781 537409 outcome TRUE reported 7185_29_GP5_GPV GP5
chr3 194400269 0.00959 2.126e-34 35296 TRUE reported textfile 2 1
TRUE

6390 rs1555405 A G A G -0.0494 -0.0047 0.30381 NA FALSE FALSE FALSE
GCST006061 0.0081 0.5592 537409 outcome TRUE reported 7185_29_GP5_GPV GP5
chr14 100710432 0.008817 2.109e-08 35380 TRUE reported textfile 2
1 TRUE
6391 rs3792386 A G A G -0.0904 0.0089 0.35198 NA FALSE FALSE FALSE
GCST006061 0.0073 0.2182 537409 outcome TRUE reported 7185_29_GP5_GPV GP5
chr3 123134833 0.008475 1.461e-26 35367 TRUE reported textfile 2
1 TRUE
6392 rs838136 C T C T -0.058 0.0096 0.23057 NA FALSE FALSE FALSE
GCST006061 0.0082 0.2437 537409 outcome TRUE reported 7185_29_GP5_GPV GP5
chr19 48753131 0.009569 1.353e-09 35376 TRUE reported textfile 2
1 TRUE
6393 rs10894353 T C T C 0.2708 0.0095 0.46651 NA FALSE FALSE FALSE
GCST006061 0.0076 0.2088 537409 outcome TRUE reported 8428_102_NTM_NTRI NTM
chr11 131255185 0.008502 1e-200 35369 TRUE reported textfile 2 1
TRUE
6394 rs1793768 T C T C 0.0587 -0.0069 0.43214 NA FALSE FALSE FALSE
GCST006061 0.0073 0.3443 537409 outcome TRUE reported 8428_102_NTM_NTRI NTM
chr11 131170695 0.008667 1.266e-11 35370 TRUE reported textfile 2
1 TRUE
6395 rs487303 T C T C 0.0526 0.0141 0.30391 NA FALSE FALSE FALSE
GCST006061 0.0081 0.08124 537409 outcome TRUE reported 8428_102_NTM_NTRI NTM
chr11 131918323 0.009406 2.242e-08 35371 TRUE reported textfile 2
1 TRUE
6396 rs7111143 A G A G 0.0709 0.0091 0.329 NA FALSE FALSE FALSE
GCST006061 0.0075 0.2274 537409 outcome TRUE reported 8428_102_NTM_NTRI NTM
chr11 130889029 0.009174 1.089e-14 35371 TRUE reported textfile 2
1 TRUE
6397 rs10896415 A G A G -0.0577 0.0011 0.37201 NA FALSE FALSE FALSE
GCST006061 0.0078 0.8924 537409 outcome TRUE reported
10754_113_PROK2_Prokineticin_2 PROK2 chr11 69071866 0.008611 2.075e-11
35354 TRUE reported textfile 2 1 TRUE
6398 rs116198731 C T C T 0.1893 0.0349 0.01599 NA FALSE FALSE FALSE
GCST006061 0.044 0.4272 537409 outcome TRUE reported
10754_113_PROK2_Prokineticin_2 PROK2 chr3 69474148 0.034111 2.864e-08
35340 TRUE reported textfile 2 1 TRUE
6399 rs138866676 T C T C 0.1832 0.0233 0.02635 NA FALSE FALSE FALSE
GCST006061 0.0244 0.3392 537409 outcome TRUE reported
10754_113_PROK2_Prokineticin_2 PROK2 chr3 70273106 0.026856 9.001e-12
35342 TRUE reported textfile 2 1 TRUE
6400 rs17009077 G A G A -0.1229 -7.00E-04 0.28852 NA FALSE FALSE FALSE
GCST006061 0.0084 0.9336 537409 outcome TRUE reported
10754_113_PROK2_Prokineticin_2 PROK2 chr3 71892003 0.00955 6.729e-38 35335
TRUE reported textfile 2 1 TRUE
6401 rs2686274 T C T C -0.0963 -0.018 0.2316 NA FALSE FALSE FALSE
GCST006061 0.0085 0.03413 537409 outcome TRUE reported
10754_113_PROK2_Prokineticin_2 PROK2 chr3 71262190 0.010238 5.163e-21
35339 TRUE reported textfile 2 1 TRUE
6402 rs3809627 A C A C 0.0479 0.0105 0.42241 NA FALSE FALSE FALSE
GCST006061 0.0073 0.1543 537409 outcome TRUE reported
10754_113_PROK2_Prokineticin_2 PROK2 chr16 30091839 0.008451 1.444e-08
35360 TRUE reported textfile 2 1 TRUE
6403 rs56318703 T C T C 0.081 -0.0127 0.24229 NA FALSE FALSE FALSE
GCST006061 0.0088 0.1508 537409 outcome TRUE reported
10754_113_PROK2_Prokineticin_2 PROK2 chr10 909566 0.009696 6.591e-17 35167
TRUE reported textfile 2 1 TRUE
6404 rs9809964 A C A C 0.1303 -0.017 0.04747 NA FALSE FALSE FALSE
GCST006061 0.0213 0.4235 537409 outcome TRUE reported
10754_113_PROK2_Prokineticin_2 PROK2 chr3 72628421 0.019962 6.689e-11
35335 TRUE reported textfile 2 1 TRUE
6405 rs9898547 T G T G -0.1002 0.013 0.44771 NA FALSE FALSE FALSE
GCST006061 0.0071 0.06951 537409 outcome TRUE reported
10754_113_PROK2_Prokineticin_2 PROK2 chr17 39979773 0.008386 6.661e-33
35367 TRUE reported textfile 2 1 TRUE
6406 rs10896631 C T C T -0.1219 -0.0054 0.24211 NA FALSE FALSE FALSE
GCST006061 0.0082 0.5057 537409 outcome TRUE reported 13575_40_DVL2_DVL2

DVL2 chr11 57613790 0.009595 5.591e-37 35358 TRUE reported
textfile 2 1 TRUE
6407 rs11849582 G A G A 0.1632 -0.0016 0.40798 NA FALSE FALSE FALSE
GCST006061 0.0108 0.8834 537409 outcome TRUE reported 13575_40_DVL2_DVL2
DVL2 chr14 105728918 0.008387 2.424e-84 35092 TRUE reported
textfile 2 1 TRUE
6408 rs12058824 A G A G -0.103 0.0033 0.09736 NA FALSE FALSE FALSE
GCST006061 0.0136 0.8082 537409 outcome TRUE reported 13575_40_DVL2_DVL2
DVL2 chr1 22636557 0.013844 1.005e-13 35363 TRUE reported
textfile 2 1 TRUE
6409 rs145271606 T G T G -0.2055 0.045 0.01442 NA FALSE FALSE FALSE
GCST006061 0.0567 0.4274 537409 outcome TRUE reported 13575_40_DVL2_DVL2
DVL2 chr12 7777303 0.03533 6.005e-09 35372 TRUE reported textfile 2
1 TRUE
6410 rs7962629 G A G A -0.3978 -5.00E-04 0.13882 NA FALSE FALSE FALSE
GCST006061 0.0103 0.9618 537409 outcome TRUE reported 13575_40_DVL2_DVL2
DVL2 chr12 7059466 0.011929 1e-200 35384 TRUE reported textfile 2
1 TRUE
6411 rs8010223 C T C T -0.1505 0.0276 0.03367 NA FALSE FALSE FALSE
GCST006061 0.0405 0.4963 537409 outcome TRUE reported 13575_40_DVL2_DVL2
DVL2 chr14 105245754 0.022968 5.653e-11 35103 TRUE reported
textfile 2 1 TRUE
6412 rs10900219 G A G A 0.1046 0.0072 0.20674 NA FALSE FALSE FALSE
GCST006061 0.0083 0.3859 537409 outcome TRUE reported 6895_1_TFRC_TR TFRC
chr10 45476479 0.009988 1.154e-25 35328 TRUE reported textfile 2
1 TRUE
6413 rs12210538 G A G A 0.0649 0.0047 0.22584 NA FALSE FALSE FALSE
GCST006061 0.009 0.604 537409 outcome TRUE reported 6895_1_TFRC_TR TFRC
chr6 110438805 0.00972 2.44e-11 35373 TRUE reported textfile 2 1
TRUE
6414 rs12461546 T C T C 0.1273 -0.0172 0.08577 NA FALSE FALSE FALSE
GCST006061 0.0139 0.2164 537409 outcome TRUE reported 6895_1_TFRC_TR TFRC
chr19 11187815 0.014567 2.356e-18 35382 TRUE reported textfile 2
1 TRUE
6415 rs1574539 A G A G -0.0508 -0.002 0.28622 NA FALSE FALSE FALSE
GCST006061 0.0082 0.8103 537409 outcome TRUE reported 6895_1_TFRC_TR TFRC
chr1 31548894 0.008979 1.533e-08 35373 TRUE reported textfile 2
1 TRUE
6416 rs2413450 C T C T -0.0487 -0.002 0.42498 NA FALSE FALSE FALSE
GCST006061 0.0072 0.7817 537409 outcome TRUE reported 6895_1_TFRC_TR TFRC
chr22 37074184 0.008227 3.228e-09 35373 TRUE reported textfile 2
1 TRUE
6417 rs3741983 T C T C -0.0543 0.0089 0.23511 NA FALSE FALSE FALSE
GCST006061 0.0095 0.3472 537409 outcome TRUE reported 6895_1_TFRC_TR TFRC
chr12 112502049 0.00963 1.712e-08 35370 TRUE reported textfile 2 1
TRUE
6418 rs8047587 T G T G 0.0518 -0.0194 0.43917 NA FALSE FALSE FALSE
GCST006061 0.0073 0.00763 537409 outcome TRUE reported 6895_1_TFRC_TR TFRC
chr16 53764710 0.008142 1.993e-10 35366 TRUE reported textfile 2
1 TRUE
6419 rs10901246 T G T G 0.3535 0.002 0.19344 NA FALSE FALSE FALSE
GCST006061 0.0087 0.8143 537409 outcome TRUE reported 5680_54_OBP2B_OBP2B
OBP2B chr9 133205098 0.011307 1e-200 35369 TRUE reported textfile
2 1 TRUE
6420 rs11103374 T C T C -0.131 -0.0408 0.11491 NA FALSE FALSE FALSE
GCST006061 0.0105 0.0001067 537409 outcome TRUE reported 5680_54_OBP2B_OBP2B
OBP2B chr9 136195675 0.014231 3.406e-20 35266 TRUE reported
textfile 2 1 TRUE
6421 rs147980161 T C T C 0.3585 -0.0478 0.01136 NA FALSE FALSE FALSE
GCST006061 0.0571 0.4025 537409 outcome TRUE reported 5680_54_OBP2B_OBP2B
OBP2B chr9 135403570 0.042283 2.281e-17 35309 TRUE reported
textfile 2 1 TRUE
6422 rs615364 G A G A -0.7535 0.0394 0.0488 NA FALSE FALSE FALSE
GCST006061 0.0263 0.1332 537409 outcome TRUE reported 5680_54_OBP2B_OBP2B
OBP2B chr9 135545821 0.020926 1e-200 35302 TRUE reported textfile
2 1 TRUE

6423 rs76523956 C A C A 0.2394 -0.012 0.01464 NA FALSE FALSE FALSE GCST006061 0.0327 0.7127 537409 outcome TRUE reported 5680_54_OBP2B_OBP2B OBP2B chr9 135530290 0.036961 9.355e-11 35303 TRUE reported textfile 2 1 TRUE
6424 rs7856882 G A G A 0.1185 -0.0054 0.46282 NA FALSE FALSE FALSE GCST006061 0.0076 0.4836 537409 outcome TRUE reported 5680_54_OBP2B_OBP2B OBP2B chr9 135771333 0.009129 1.582e-38 35291 TRUE reported textfile 2 1 TRUE
6425 rs7857413 A C A C -0.3671 3e-04 0.26436 NA FALSE FALSE FALSE GCST006061 0.0092 0.9727 537409 outcome TRUE reported 5680_54_OBP2B_OBP2B OBP2B chr9 135537409 0.010091 1e-200 35300 TRUE reported textfile 2 1 TRUE
6426 rs11956300 T G T G 0.0722 -0.0024 0.11195 NA FALSE FALSE FALSE GCST006061 0.0113 0.8307 537409 outcome TRUE reported 7945_10_SEMA6A_Semaphorin_6A SEMA6A chr5 116515541 0.013145 3.964e-08 35360 TRUE reported textfile 2 1 TRUE
6427 rs3094379 C T C T 0.1853 0.0023 0.0951 NA FALSE FALSE FALSE GCST006061 0.0105 0.8286 537409 outcome TRUE reported 7945_10_SEMA6A_Semaphorin_6A SEMA6A chr9 133469788 0.014147 3.36e-39 35369 TRUE reported textfile 2 1 TRUE
6428 rs35166255 A G A G 0.1197 0.0222 0.0367 NA FALSE FALSE FALSE GCST006061 0.0229 0.3329 537409 outcome TRUE reported 7945_10_SEMA6A_Semaphorin_6A SEMA6A chr11 126431861 0.021278 1.85e-08 35371 TRUE reported textfile 2 1 TRUE
6429 rs4857414 T C T C -0.0654 0.0036 0.43729 NA FALSE FALSE FALSE GCST006061 0.0072 0.6214 537409 outcome TRUE reported 7945_10_SEMA6A_Semaphorin_6A SEMA6A chr3 98713715 0.008316 3.704e-15 35330 TRUE reported textfile 2 1 TRUE
6430 rs55714927 T C T C 0.0638 1e-04 0.21166 NA FALSE FALSE FALSE GCST006061 0.0098 0.9926 537409 outcome TRUE reported 7945_10_SEMA6A_Semaphorin_6A SEMA6A chr17 7176997 0.010193 3.864e-10 35370 TRUE reported textfile 2 1 TRUE
6431 rs7369864 A G A G -0.0672 0.0044 0.41682 NA FALSE FALSE FALSE GCST006061 0.0072 0.5432 537409 outcome TRUE reported 7945_10_SEMA6A_Semaphorin_6A SEMA6A chr2 109478679 0.008394 1.188e-15 35354 TRUE reported textfile 2 1 TRUE
6432 rs117221168 A G A G 0.1926 -0.0261 0.04753 NA FALSE FALSE FALSE GCST006061 0.0233 0.264 537409 outcome TRUE reported 16610_13_LRP10_LRP10 LRP10 chr14 22937095 0.018943 2.779e-24 35379 TRUE reported textfile 2 1 TRUE
6433 rs117411993 C T C T -0.2433 -0.0371 0.0127 NA FALSE FALSE FALSE GCST006061 0.0348 0.2857 537409 outcome TRUE reported 16610_13_LRP10_LRP10 LRP10 chr14 23165687 0.037058 5.192e-11 35377 TRUE reported textfile 2 1 TRUE
6434 rs1805248 G A G A 0.0558 0.0232 0.22071 NA FALSE FALSE FALSE GCST006061 0.0086 0.007041 537409 outcome TRUE reported 16610_13_LRP10_LRP10 LRP10 chr12 8948541 0.009888 1.668e-08 35387 TRUE reported textfile 2 1 TRUE
6435 rs599839 A G A G 0.0684 -0.0163 0.20777 NA FALSE FALSE FALSE GCST006061 0.0087 0.06014 537409 outcome TRUE reported 16610_13_LRP10_LRP10 LRP10 chr1 109279544 0.010076 1.135e-11 35344 TRUE reported textfile 2 1 TRUE
6436 rs77924615 A G A G -0.0563 0.0136 0.19701 NA FALSE FALSE FALSE GCST006061 0.0092 0.1375 537409 outcome TRUE reported 16610_13_LRP10_LRP10 LRP10 chr16 20381010 0.010322 4.918e-08 35355 TRUE reported textfile 2 1 TRUE
6437 rs1260326 C T C T 0.0611 -0.0069 0.34132 NA FALSE FALSE FALSE GCST006061 0.0073 0.3401 537409 outcome TRUE reported 5107_7_NOTCH1_Notch_1 NOTCH1 chr2 27508073 0.008686 2.003e-12 35373 TRUE reported textfile 2 1 TRUE
6438 rs3184504 C T C T -0.0577 0.0074 0.37862 NA FALSE FALSE FALSE GCST006061 0.0075 0.3235 537409 outcome TRUE reported 5107_7_NOTCH1_Notch_1 NOTCH1 chr12 111446804 0.008467 9.438e-12 35371 TRUE reported textfile 2 1 TRUE
6439 rs35166255 A G A G 0.1383 0.0222 0.0367 NA FALSE FALSE FALSE GCST006061 0.0229 0.3329 537409 outcome TRUE reported 5107_7_NOTCH1_Notch_1

NOTCH1 chr11 126431861 0.021082 5.374e-11 35371 TRUE reported textfile 2 1 TRUE
6440 rs62576042 T C T C 0.0856 -0.0019 0.19144 NA FALSE FALSE FALSE GCST006061 0.0109 0.8608 537409 outcome TRUE reported 5107_7_NOTCH1_Notch_1 NOTCH1 chr9 133513605 0.010511 3.823e-16 35360 TRUE reported textfile 2 1 TRUE
6441 rs9411207 T C T C 0.0968 0.0065 0.32159 NA FALSE FALSE FALSE GCST006061 0.008 0.4192 537409 outcome TRUE reported 5107_7_NOTCH1_Notch_1 NOTCH1 chr9 136514099 0.008839 6.496e-28 35270 TRUE reported textfile 2 1 TRUE
6442 rs10922098 T C T C -0.17 -0.009 0.40011 NA FALSE FALSE FALSE GCST006061 0.0075 0.2275 537409 outcome TRUE reported 2611_72_TYRO3_Dtk TYRO3 chr1 196695521 0.008237 1.25e-94 35338 TRUE reported textfile 2 1 TRUE
6443 rs1707652 T C T C 0.0807 0.0081 0.3102 NA FALSE FALSE FALSE GCST006061 0.0074 0.2701 537409 outcome TRUE reported 2611_72_TYRO3_Dtk TYRO3 chr3 165761011 0.008725 2.251e-20 35339 TRUE reported textfile 2 1 TRUE
6444 rs704 A G A G 0.0468 -0.0155 0.49189 NA FALSE FALSE FALSE GCST006061 0.0072 0.03144 537409 outcome TRUE reported 2611_72_TYRO3_Dtk TYRO3 chr17 28367840 0.008112 7.976e-09 35360 TRUE reported textfile 2 1 TRUE
6445 rs1355538 G A G A -0.0525 -0.0076 0.39847 NA FALSE FALSE FALSE GCST006061 0.0072 0.2923 537409 outcome TRUE reported 7950_142_BTNL9_BTNL9 BTNL9 chr3 165787389 0.008061 7.372e-11 35338 TRUE reported textfile 2 1 TRUE
6446 rs113020163 A G A G -0.245 0.0811 0.02193 NA FALSE FALSE FALSE GCST006061 0.0393 0.03894 537409 outcome TRUE reported 5132_71_IL27RA_TCCR IL27RA chr19 14514283 0.031622 9.344e-15 35374 TRUE reported textfile 2 1 TRUE
6447 rs11667546 T C T C -0.5714 -0.024 0.17284 NA FALSE FALSE FALSE GCST006061 0.0098 0.01433 537409 outcome TRUE reported 5132_71_IL27RA_TCCR IL27RA chr19 13957898 0.008624 1e-200 35372 TRUE reported textfile 2 1 TRUE
6448 rs141425154 T C T C 0.2556 -0.0504 0.01295 NA FALSE FALSE FALSE GCST006061 0.0423 0.2337 537409 outcome TRUE reported 5132_71_IL27RA_TCCR IL27RA chr19 13896226 0.040227 2.098e-10 35373 TRUE reported textfile 2 1 TRUE
6449 rs3184504 C T C T -0.0951 0.0074 0.37862 NA FALSE FALSE FALSE GCST006061 0.0075 0.3235 537409 outcome TRUE reported 5132_71_IL27RA_TCCR IL27RA chr12 111446804 0.008821 4.202e-27 35371 TRUE reported textfile 2 1 TRUE
6450 rs34317732 T C T C -0.7632 -0.0094 0.02221 NA FALSE FALSE FALSE GCST006061 0.0353 0.7903 537409 outcome TRUE reported 5132_71_IL27RA_TCCR IL27RA chr19 14061996 0.031257 1.131e-131 35369 TRUE reported textfile 2 1 TRUE
6451 rs56361194 T C T C 0.212 -0.0046 0.0258 NA FALSE FALSE FALSE GCST006061 0.0303 0.8795 537409 outcome TRUE reported 5132_71_IL27RA_TCCR IL27RA chr19 14054786 0.028659 1.389e-13 35369 TRUE reported textfile 2 1 TRUE
6452 rs62576042 T C T C 0.1043 -0.0019 0.19144 NA FALSE FALSE FALSE GCST006061 0.0109 0.8608 537409 outcome TRUE reported 5132_71_IL27RA_TCCR IL27RA chr9 133513605 0.010972 1.978e-21 35360 TRUE reported textfile 2 1 TRUE
6453 rs6511928 T C T C 0.2453 0.0041 0.06235 NA FALSE FALSE FALSE GCST006061 0.0159 0.7968 537409 outcome TRUE reported 5132_71_IL27RA_TCCR IL27RA chr19 14312415 0.018752 4.232e-39 35374 TRUE reported textfile 2 1 TRUE
6454 rs72759422 T C T C 0.1975 0.0056 0.01571 NA FALSE FALSE FALSE GCST006061 0.029 0.8459 537409 outcome TRUE reported 5132_71_IL27RA_TCCR IL27RA chr9 132853065 0.034883 1.498e-08 35373 TRUE reported textfile 2 1 TRUE
6455 rs1427407 G T G T -0.0731 0.0124 0.134 NA FALSE FALSE FALSE GCST006061 0.0096 0.1972 537409 outcome TRUE reported 7019_13_SEMA7A_Semaphorin_7A SEMA7A chr2 60490908 0.012169 1.89e-09 35346 TRUE reported textfile 2 1 TRUE
6456 rs281377 T C T C -0.0549 -0.0091 0.3353 NA FALSE FALSE FALSE

GCST006061 0.0075 0.2214 537409 outcome TRUE reported
7019_13_SEMA7A_Semaphorin_7A SEMA7A chr19 48703346 0.008968 9.265e-10
35377 TRUE reported textfile 2 1 TRUE
6457 rs3094379 C T C T 0.0816 0.0023 0.0951 NA FALSE FALSE FALSE
GCST006061 0.0105 0.8286 537409 outcome TRUE reported
7019_13_SEMA7A_Semaphorin_7A SEMA7A chr9 133469788 0.014215 9.434e-09
35369 TRUE reported textfile 2 1 TRUE
6458 rs3864105 G A G A -0.1264 0.0015 0.43496 NA FALSE FALSE FALSE
GCST006061 0.0072 0.8407 537409 outcome TRUE reported
7019_13_SEMA7A_Semaphorin_7A SEMA7A chr3 186938746 0.008405 4.136e-51
35368 TRUE reported textfile 2 1 TRUE
6459 rs4686841 A G A G 0.0483 0.0029 0.37454 NA FALSE FALSE FALSE
GCST006061 0.0073 0.6881 537409 outcome TRUE reported
7019_13_SEMA7A_Semaphorin_7A SEMA7A chr3 187037955 0.008616 2.075e-08
35363 TRUE reported textfile 2 1 TRUE
6460 rs55714927 T C T C 0.0894 1e-04 0.21166 NA FALSE FALSE FALSE
GCST006061 0.0098 0.9926 537409 outcome TRUE reported
7019_13_SEMA7A_Semaphorin_7A SEMA7A chr17 7176997 0.010244 2.612e-18 35370
TRUE reported textfile 2 1 TRUE
6461 rs78994380 A C A C -0.127 0.0195 0.11252 NA FALSE FALSE FALSE
GCST006061 0.0126 0.1213 537409 outcome TRUE reported
7019_13_SEMA7A_Semaphorin_7A SEMA7A chr15 74400071 0.013212 7.086e-22
35359 TRUE reported textfile 2 1 TRUE
6462 rs10922098 T C T C -0.1077 -0.009 0.40011 NA FALSE FALSE FALSE
GCST006061 0.0075 0.2275 537409 outcome TRUE reported 2997_8_JAM2_JAM_B
JAM2 chr1 196695521 0.008272 9.521e-39 35338 TRUE reported
textfile 2 1 TRUE
6463 rs1355538 G A G A -0.0565 -0.0076 0.39847 NA FALSE FALSE FALSE
GCST006061 0.0072 0.2923 537409 outcome TRUE reported 2997_8_JAM2_JAM_B
JAM2 chr3 165787389 0.008273 8.542e-12 35338 TRUE reported
textfile 2 1 TRUE
6464 rs11111630 T C T C 0.1027 -0.0192 0.08669 NA FALSE FALSE FALSE
GCST006061 0.0165 0.2441 537409 outcome TRUE reported
13499_30_F8_Coagulation_Factor_VIII F8 chr12 103535806 0.014786 3.764e-12
35361 TRUE reported textfile 2 1 TRUE
6465 rs11243569 T C T C -0.0673 -0.0066 0.14913 NA FALSE FALSE FALSE
GCST006061 0.011 0.5493 537409 outcome TRUE reported
13499_30_F8_Coagulation_Factor_VIII F8 chr9 131940362 0.011595 6.465e-09
35366 TRUE reported textfile 2 1 TRUE
6466 rs11603123 A G A G 0.1871 0.0206 0.03671 NA FALSE FALSE FALSE
GCST006061 0.023 0.3702 537409 outcome TRUE reported
13499_30_F8_Coagulation_Factor_VIII F8 chr11 126435600 0.020925 3.84e-19
35365 TRUE reported textfile 2 1 TRUE
6467 rs139562936 T G T G 0.1751 -0.0301 0.0211 NA FALSE FALSE FALSE
GCST006061 0.034 0.3765 537409 outcome TRUE reported
13499_30_F8_Coagulation_Factor_VIII F8 chr9 134215558 0.028889 1.352e-09
35366 TRUE reported textfile 2 1 TRUE
6468 rs146437644 A G A G 0.1815 -0.0439 0.02499 NA FALSE FALSE FALSE
GCST006061 0.0299 0.1414 537409 outcome TRUE reported
13499_30_F8_Coagulation_Factor_VIII F8 chr9 132784976 0.026339 5.543e-12
35366 TRUE reported textfile 2 1 TRUE
6469 rs188547348 G A G A 0.1586 0.0654 0.02666 NA FALSE FALSE FALSE
GCST006061 0.0352 0.06349 537409 outcome TRUE reported
13499_30_F8_Coagulation_Factor_VIII F8 chr12 104682338 0.025581 5.649e-10
35347 TRUE reported textfile 2 1 TRUE
6470 rs2277998 A G A G -0.056 0.0058 0.28867 NA FALSE FALSE FALSE
GCST006061 0.0082 0.4751 537409 outcome TRUE reported
13499_30_F8_Coagulation_Factor_VIII F8 chr19 7766742 0.009015 5.231e-10 35368
TRUE reported textfile 2 1 TRUE
6471 rs2283335 C T C T 0.1282 -0.0106 0.40778 NA FALSE FALSE FALSE
GCST006061 0.0076 0.1641 537409 outcome TRUE reported
13499_30_F8_Coagulation_Factor_VIII F8 chr12 6048228 0.00832 1.439e-53 35375
TRUE reported textfile 2 1 TRUE
6472 rs2362482 A G A G 0.1743 -0.0189 0.0737 NA FALSE FALSE FALSE
GCST006061 0.0155 0.2213 537409 outcome TRUE reported
13499_30_F8_Coagulation_Factor_VIII F8 chr12 5949747 0.015758 1.945e-28 35377

TRUE reported textfile 2 1 TRUE
6473 rs306535 T C T C -0.1327 -0.0019 0.03847 NA FALSE FALSE FALSE
GCST006061 0.0171 0.9098 537409 outcome TRUE reported
13499_30_F8_Coagulation_Factor_VIII F8 chr9 132642278 0.021545 7.308e-10
35367 TRUE reported textfile 2 1 TRUE
6474 rs3124761 C T C T -0.3009 0.0014 0.09484 NA FALSE FALSE FALSE
GCST006061 0.0106 0.8981 537409 outcome TRUE reported
13499_30_F8_Coagulation_Factor_VIII F8 chr9 133474633 0.014095 4.099e-101
35363 TRUE reported textfile 2 1 TRUE
6475 rs4981022 A G A G 0.0731 -5.00E-04 0.3206 NA FALSE FALSE FALSE
GCST006061 0.0078 0.9508 537409 outcome TRUE reported
13499_30_F8_Coagulation_Factor_VIII F8 chr12 103756096 0.008729 5.555e-17
35355 TRUE reported textfile 2 1 TRUE
6476 rs6928919 G A G A 0.0479 -0.0148 0.45671 NA FALSE FALSE FALSE
GCST006061 0.0072 0.03937 537409 outcome TRUE reported
13499_30_F8_Coagulation_Factor_VIII F8 chr6 147310065 0.008192 4.995e-09
35347 TRUE reported textfile 2 1 TRUE
6477 rs78004438 T C T C 0.1668 -0.0132 0.02042 NA FALSE FALSE FALSE
GCST006061 0.0377 0.7269 537409 outcome TRUE reported
13499_30_F8_Coagulation_Factor_VIII F8 chr12 103706537 0.028275 3.651e-09
35354 TRUE reported textfile 2 1 TRUE
6478 rs9751083 C T C T 0.0582 0.0073 0.19825 NA FALSE FALSE FALSE
GCST006061 0.0087 0.4006 537409 outcome TRUE reported
13499_30_F8_Coagulation_Factor_VIII F8 chr2 62375785 0.010138 9.438e-09
35343 TRUE reported textfile 2 1 TRUE
6479 rs11085824 G A G A 0.0699 -0.0089 0.40391 NA FALSE FALSE FALSE
GCST006061 0.0076 0.2396 537409 outcome TRUE reported 2816_50_BCAM_BCAM
BCAM chr19 12890733 0.008608 4.65e-16 35381 TRUE reported
textfile 2 1 TRUE
6480 rs112382785 C T C T 0.2052 -0.0343 0.02739 NA FALSE FALSE FALSE
GCST006061 0.0292 0.2396 537409 outcome TRUE reported 2816_50_BCAM_BCAM
BCAM chr19 45751984 0.025456 7.573e-16 35369 TRUE reported
textfile 2 1 TRUE
6481 rs117446824 A G A G 0.0893 0.0113 0.07195 NA FALSE FALSE FALSE
GCST006061 0.0189 0.5488 537409 outcome TRUE reported 2816_50_BCAM_BCAM
BCAM chr9 133781764 0.016073 2.764e-08 35372 TRUE reported
textfile 2 1 TRUE
6482 rs117644736 T C T C 0.2222 0.008 0.04055 NA FALSE FALSE FALSE
GCST006061 0.0373 0.8301 537409 outcome TRUE reported 2816_50_BCAM_BCAM
BCAM chr19 45011597 0.020709 7.381e-27 35367 TRUE reported
textfile 2 1 TRUE
6483 rs12461127 T C T C 0.0684 0.0062 0.48367 NA FALSE FALSE FALSE
GCST006061 0.0073 0.3974 537409 outcome TRUE reported 2816_50_BCAM_BCAM
BCAM chr19 44549282 0.008421 4.57e-16 35369 TRUE reported
textfile 2 1 TRUE
6484 rs139645865 A G A G -0.1578 9e-04 0.03264 NA FALSE FALSE FALSE
GCST006061 0.0385 0.9817 537409 outcome TRUE reported 2816_50_BCAM_BCAM
BCAM chr19 45858717 0.023601 2.289e-11 35369 TRUE reported
textfile 2 1 TRUE
6485 rs150908522 T C T C 0.307 0.0087 0.03563 NA FALSE FALSE FALSE
GCST006061 0.024 0.7159 537409 outcome TRUE reported 2816_50_BCAM_BCAM
BCAM chr19 45293071 0.022396 9.119e-43 35373 TRUE reported
textfile 2 1 TRUE
6486 rs62576042 T C T C 0.0757 -0.0019 0.19144 NA FALSE FALSE FALSE
GCST006061 0.0109 0.8608 537409 outcome TRUE reported 2816_50_BCAM_BCAM
BCAM chr9 133513605 0.010603 9.387e-13 35360 TRUE reported
textfile 2 1 TRUE
6487 rs6592965 A G A G -0.0479 -0.0032 0.40057 NA FALSE FALSE FALSE
GCST006061 0.0072 0.656 537409 outcome TRUE reported 2816_50_BCAM_BCAM
BCAM chr7 50360284 0.008459 1.493e-08 35336 TRUE reported
textfile 2 1 TRUE
6488 rs746063 T C T C 0.0537 0.0059 0.4015 NA FALSE FALSE FALSE
GCST006061 0.0076 0.4429 537409 outcome TRUE reported 2816_50_BCAM_BCAM
BCAM chr21 41209498 0.008468 2.275e-10 35362 TRUE reported
textfile 2 1 TRUE
6489 rs7610449 A G A G -0.0548 -0.0296 0.23597 NA FALSE FALSE FALSE

GCST006061 0.0082 0.0002971 537409 outcome TRUE reported 2816_50_BCAM_BCAM
BCAM chr3 58334231 0.009805 2.282e-08 35357 TRUE reported
textfile 2 1 TRUE
6490 rs78675029 A G A G 0.1713 -0.009 0.03704 NA FALSE FALSE FALSE
GCST006061 0.0271 0.7389 537409 outcome TRUE reported 2816_50_BCAM_BCAM
BCAM chr19 45100162 0.022333 1.717e-14 35371 TRUE reported
textfile 2 1 TRUE
6491 rs11125916 A C A C -0.086 0.0084 0.19143 NA FALSE FALSE FALSE
GCST006061 0.0088 0.341 537409 outcome TRUE reported 2991_9_IL1R1_IL_1_sRI
IL1R1 chr2 62382243 0.010297 6.692e-17 35349 TRUE reported
textfile 2 1 TRUE
6492 rs11885528 G A G A 0.0798 -0.0039 0.48828 NA FALSE FALSE FALSE
GCST006061 0.0076 0.6124 537409 outcome TRUE reported 2991_9_IL1R1_IL_1_sRI
IL1R1 chr2 102072035 0.00818 1.747e-22 35366 TRUE reported textfile
2 1 TRUE
6493 rs3094379 C T C T 0.0954 0.0023 0.0951 NA FALSE FALSE FALSE
GCST006061 0.0105 0.8286 537409 outcome TRUE reported 2991_9_IL1R1_IL_1_sRI
IL1R1 chr9 133469788 0.013837 5.4e-12 35369 TRUE reported textfile
2 1 TRUE
6494 rs4580257 G T G T -0.0604 -0.0122 0.38675 NA FALSE FALSE FALSE
GCST006061 0.0072 0.09157 537409 outcome TRUE reported 2991_9_IL1R1_IL_1_sRI
IL1R1 chr17 78406947 0.008319 3.869e-13 35378 TRUE reported
textfile 2 1 TRUE
6495 rs55709272 C T C T 0.1774 0.0013 0.48871 NA FALSE FALSE FALSE
GCST006061 0.0074 0.8665 537409 outcome TRUE reported 2991_9_IL1R1_IL_1_sRI
IL1R1 chr2 113109711 0.008092 1.567e-106 35356 TRUE reported
textfile 2 1 TRUE
6496 rs61830291 C A C A -0.1059 -0.0236 0.10488 NA FALSE FALSE FALSE
GCST006061 0.0132 0.07315 537409 outcome TRUE reported 2991_9_IL1R1_IL_1_sRI
IL1R1 chr1 220827800 0.013256 1.362e-15 35353 TRUE reported
textfile 2 1 TRUE
6497 rs6728769 C T C T 0.1136 -5.00E-04 0.30847 NA FALSE FALSE FALSE
GCST006061 0.008 0.9491 537409 outcome TRUE reported 2991_9_IL1R1_IL_1_sRI
IL1R1 chr2 113050934 0.008876 1.664e-37 35360 TRUE reported
textfile 2 1 TRUE
6498 rs9483788 C T C T 0.0577 -0.005 0.25867 NA FALSE FALSE FALSE
GCST006061 0.0081 0.5365 537409 outcome TRUE reported 2991_9_IL1R1_IL_1_sRI
IL1R1 chr6 135114363 0.009239 4.224e-10 35372 TRUE reported
textfile 2 1 TRUE
6499 rs9864515 G T G T 0.0675 -0.0023 0.16393 NA FALSE FALSE FALSE
GCST006061 0.0115 0.8412 537409 outcome TRUE reported 2991_9_IL1R1_IL_1_sRI
IL1R1 chr3 97782304 0.01096 7.342e-10 35339 TRUE reported textfile
2 1 TRUE
6500 rs11072526 C T C T 0.0579 -0.0194 0.3141 NA FALSE FALSE FALSE
GCST006061 0.0081 0.01632 537409 outcome TRUE reported
4930_21_STC1_Stanniocalcin_1 STC1 chr15 75140176 0.00878 4.261e-11 35362
TRUE reported textfile 2 1 TRUE
6501 rs13262489 T C T C 0.1255 -0.0108 0.10108 NA FALSE FALSE FALSE
GCST006061 0.0114 0.3425 537409 outcome TRUE reported
4930_21_STC1_Stanniocalcin_1 STC1 chr8 23835137 0.013654 3.885e-20
35370 TRUE reported textfile 2 1 TRUE
6502 rs646776 T C T C 0.1411 -0.0142 0.20804 NA FALSE FALSE FALSE
GCST006061 0.0087 0.1014 537409 outcome TRUE reported
4930_21_STC1_Stanniocalcin_1 STC1 chr1 109275908 0.01006 1.089e-44 35344
TRUE reported textfile 2 1 TRUE
6503 rs77797953 G A G A 0.0835 -0.0094 0.09768 NA FALSE FALSE FALSE
GCST006061 0.0147 0.5207 537409 outcome TRUE reported
4930_21_STC1_Stanniocalcin_1 STC1 chr12 571510 0.013791 1.406e-09 35314
TRUE reported textfile 2 1 TRUE
6504 rs10919615 T C T C 0.0579 -5.00E-04 0.24671 NA FALSE FALSE FALSE
GCST006061 0.0083 0.9543 537409 outcome TRUE reported 5740_17_ROBO1_ROBO1
ROBO1 chr1 199005775 0.009715 2.524e-09 35342 TRUE reported
textfile 2 1 TRUE
6505 rs11121245 C T C T 0.0728 -0.0058 0.445 NA FALSE FALSE FALSE
GCST006061 0.0073 0.4262 537409 outcome TRUE reported 5740_17_ROBO1_ROBO1
ROBO1 chr1 8856165 0.008361 3.107e-18 35388 TRUE reported textfile

2 1 TRUE
6506 rs2686395 C T C T -0.0796 -0.0077 0.3101 NA FALSE FALSE FALSE
GCST006061 0.0074 0.2965 537409 outcome TRUE reported 5740_17_ROBO1_ROBO1
ROBO1 chr3 165767231 0.008978 7.567e-19 35339 TRUE reported
textfile 2 1 TRUE
6507 rs3773233 T C T C 0.223 -0.0054 0.21279 NA FALSE FALSE FALSE
GCST006061 0.0087 0.5331 537409 outcome TRUE reported 5740_17_ROBO1_ROBO1
ROBO1 chr3 78725134 0.010121 1.405e-107 35345 TRUE reported
textfile 2 1 TRUE
6508 rs73103884 G A G A -0.2104 -0.0071 0.01772 NA FALSE FALSE FALSE
GCST006061 0.0365 0.8466 537409 outcome TRUE reported 5740_17_ROBO1_ROBO1
ROBO1 chr3 77848463 0.031332 1.878e-11 35344 TRUE reported
textfile 2 1 TRUE
6509 rs78539764 C T C T 0.1663 0.0126 0.01805 NA FALSE FALSE FALSE
GCST006061 0.026 0.6287 537409 outcome TRUE reported 5740_17_ROBO1_ROBO1
ROBO1 chr12 12338047 0.030504 4.99e-08 35376 TRUE reported
textfile 2 1 TRUE
6510 rs11075299 G T G T 0.0741 -0.0143 0.2517 NA FALSE FALSE FALSE
GCST006061 0.008 0.07532 537409 outcome TRUE reported 5451_1_ALCAM_ALCAM
ALCAM chr16 16152307 0.009475 5.251e-15 35375 TRUE reported
textfile 2 1 TRUE
6511 rs143501783 C T C T 0.2295 -0.0333 0.01116 NA FALSE FALSE FALSE
GCST006061 0.0443 0.4525 537409 outcome TRUE reported 5451_1_ALCAM_ALCAM
ALCAM chr17 7897001 0.03747 9.072e-10 35373 TRUE reported textfile 2
1 TRUE
6512 rs281377 T C T C -0.0742 -0.0091 0.3353 NA FALSE FALSE FALSE
GCST006061 0.0075 0.2214 537409 outcome TRUE reported 5451_1_ALCAM_ALCAM
ALCAM chr19 48703346 0.008886 6.822e-17 35377 TRUE reported
textfile 2 1 TRUE
6513 rs35067598 G A G A -0.1241 -0.0036 0.0452 NA FALSE FALSE FALSE
GCST006061 0.0184 0.8435 537409 outcome TRUE reported 5451_1_ALCAM_ALCAM
ALCAM chr3 105502588 0.019536 2.12e-10 35326 TRUE reported
textfile 2 1 TRUE
6514 rs3747207 A G A G 0.0603 0.0153 0.22927 NA FALSE FALSE FALSE
GCST006061 0.0085 0.07308 537409 outcome TRUE reported 5451_1_ALCAM_ALCAM
ALCAM chr22 43928975 0.009809 7.877e-10 35372 TRUE reported
textfile 2 1 TRUE
6515 rs77262773 T C T C 0.1978 0.014 0.02263 NA FALSE FALSE FALSE
GCST006061 0.0283 0.6205 537409 outcome TRUE reported 5451_1_ALCAM_ALCAM
ALCAM chr17 69253570 0.027354 4.79e-13 35371 TRUE reported
textfile 2 1 TRUE
6516 rs9834384 C T C T -0.1488 -0.0198 0.04368 NA FALSE FALSE FALSE
GCST006061 0.0243 0.4156 537409 outcome TRUE reported 5451_1_ALCAM_ALCAM
ALCAM chr3 105446365 0.020123 1.419e-13 35326 TRUE reported
textfile 2 1 TRUE
6517 rs10901256 T C T C -0.0819 -0.0015 0.28085 NA FALSE FALSE FALSE
GCST006061 0.008 0.8548 537409 outcome TRUE reported
17441_4_ALPI_Alkaline_phosphatase__intestine ALPI chr9 133287875 0.009011
9.998e-20 35370 TRUE reported textfile 2 1 TRUE
6518 rs10922098 T C T C -0.0726 -0.009 0.40011 NA FALSE FALSE FALSE
GCST006061 0.0075 0.2275 537409 outcome TRUE reported
17441_4_ALPI_Alkaline_phosphatase__intestine ALPI chr1 196695521 0.008255
1.433e-18 35338 TRUE reported textfile 2 1 TRUE
6519 rs579232 G T G T 0.0587 0.0028 0.35766 NA FALSE FALSE FALSE
GCST006061 0.0076 0.7102 537409 outcome TRUE reported
17441_4_ALPI_Alkaline_phosphatase__intestine ALPI chr9 133734641 0.008496
4.864e-12 35368 TRUE reported textfile 2 1 TRUE
6520 rs10901262 A G A G 0.0644 -0.0035 0.24707 NA FALSE FALSE FALSE
GCST006061 0.0085 0.6814 537409 outcome TRUE reported 7810_20_C1QTNF5_C1QTNF5
C1QTNF5 chr9 133325278 0.009636 2.339e-11 35371 TRUE reported
textfile 2 1 TRUE
6521 rs11039297 A G A G 0.0708 0.0095 0.26112 NA FALSE FALSE FALSE
GCST006061 0.0079 0.2302 537409 outcome TRUE reported 7810_20_C1QTNF5_C1QTNF5
C1QTNF5 chr11 47559891 0.009531 1.1e-13 35377 TRUE reported textfile
2 1 TRUE
6522 rs117981338 T C T C 0.2733 -0.0865 0.02918 NA FALSE FALSE FALSE

GCST006061 0.0383 0.0237 537409 outcome TRUE reported 7810_20_C1QTNF5_C1QTNF5
C1QTNF5 chr17 73092704 0.024745 2.326e-28 35365 TRUE reported
textfile 2 1 TRUE
6523 rs2089855 G A G A -0.1626 0.008 0.41937 NA FALSE FALSE FALSE
GCST006061 0.0075 0.2828 537409 outcome TRUE reported 7810_20_C1QTNF5_C1QTNF5
C1QTNF5 chr5 132237836 0.008444 1.269e-82 35352 TRUE reported
textfile 2 1 TRUE
6524 rs27289 T G T G -0.1073 0.0154 0.45961 NA FALSE FALSE FALSE GCST006061
0.0072 0.0309 537409 outcome TRUE reported 7810_20_C1QTNF5_C1QTNF5 C1QTNF5
chr5 97010574 0.008336 6.432e-38 35348 TRUE reported textfile 2
1 TRUE
6525 rs4876 C T C T 0.0532 -0.0036 0.44575 NA FALSE FALSE FALSE GCST006061
0.0072 0.6156 537409 outcome TRUE reported 7810_20_C1QTNF5_C1QTNF5 C1QTNF5
chr11 32103432 0.00839 2.281e-10 35366 TRUE reported textfile 2 1
TRUE
6526 rs546388 T C T C -0.0589 -0.0186 0.47687 NA FALSE FALSE FALSE
GCST006061 0.0073 0.01099 537409 outcome TRUE reported 7810_20_C1QTNF5_C1QTNF5
C1QTNF5 chr5 172901130 0.008386 2.167e-12 35366 TRUE reported
textfile 2 1 TRUE
6527 rs587985 T C T C -0.0889 -0.0051 0.32914 NA FALSE FALSE FALSE
GCST006061 0.0076 0.5029 537409 outcome TRUE reported 7810_20_C1QTNF5_C1QTNF5
C1QTNF5 chr11 119373385 0.008852 9.891e-24 35377 TRUE reported
textfile 2 1 TRUE
6528 rs792975 G A G A -0.1092 0.0154 0.15679 NA FALSE FALSE FALSE
GCST006061 0.0095 0.1049 537409 outcome TRUE reported 7810_20_C1QTNF5_C1QTNF5
C1QTNF5 chr5 172844004 0.01152 2.561e-21 35365 TRUE reported textfile
2 1 TRUE
6529 rs1257205 A G A G -0.0539 -0.0073 0.30974 NA FALSE FALSE FALSE
GCST006061 0.0076 0.3357 537409 outcome TRUE reported 2967_8_VCAM1_VCAM_1
VCAM1 chr2 134222961 0.009039 2.474e-09 35392 TRUE reported
textfile 2 1 TRUE
6530 rs1375493 A G A G -0.0527 -0.0121 0.42591 NA FALSE FALSE FALSE
GCST006061 0.0071 0.0882 537409 outcome TRUE reported 2967_8_VCAM1_VCAM_1
VCAM1 chr2 181459039 0.008454 4.545e-10 35353 TRUE reported
textfile 2 1 TRUE
6531 rs187099314 T C T C 0.1483 0.0287 0.06588 NA FALSE FALSE FALSE
GCST006061 0.016 0.07216 537409 outcome TRUE reported 2967_8_VCAM1_VCAM_1
VCAM1 chr9 133255354 0.016766 9.109e-19 35370 TRUE reported
textfile 2 1 TRUE
6532 rs3184504 C T C T -0.1333 0.0074 0.37862 NA FALSE FALSE FALSE
GCST006061 0.0075 0.3235 537409 outcome TRUE reported 2967_8_VCAM1_VCAM_1
VCAM1 chr12 111446804 0.0086 3.454e-54 35371 TRUE reported textfile
2 1 TRUE
6533 rs55971447 T C T C 0.0714 -0.0176 0.13302 NA FALSE FALSE FALSE
GCST006061 0.0121 0.1457 537409 outcome TRUE reported 2967_8_VCAM1_VCAM_1
VCAM1 chr1 161545536 0.012263 5.794e-09 35351 TRUE reported
textfile 2 1 TRUE
6534 rs62061425 G A G A 0.0755 0.001 0.21627 NA FALSE FALSE FALSE
GCST006061 0.0097 0.9151 537409 outcome TRUE reported 2967_8_VCAM1_VCAM_1
VCAM1 chr17 7169772 0.010177 1.185e-13 35370 TRUE reported textfile
2 1 TRUE
6535 rs62165726 A C A C -0.312 -0.0263 0.03053 NA FALSE FALSE FALSE
GCST006061 0.0212 0.2141 537409 outcome TRUE reported 2967_8_VCAM1_VCAM_1
VCAM1 chr2 134208991 0.024048 1.719e-38 35391 TRUE reported
textfile 2 1 TRUE
6536 rs9358948 A G A G 0.0695 -0.0098 0.15195 NA FALSE FALSE FALSE
GCST006061 0.0092 0.2868 537409 outcome TRUE reported 2967_8_VCAM1_VCAM_1
VCAM1 chr6 26483578 0.011644 2.389e-09 35376 TRUE reported
textfile 2 1 TRUE
6537 rs10903015 A C A C -0.2266 -0.0214 0.47479 NA FALSE FALSE FALSE
GCST006061 0.0072 0.002774 537409 outcome TRUE reported 6919_3_HBZ_HBAZ HBZ
chr16 330425 0.008724 9.668e-149 35318 TRUE reported textfile 2 1
TRUE
6538 rs117082297 T C T C 0.16 0.0265 0.04658 NA FALSE FALSE FALSE
GCST006061 0.0246 0.2807 537409 outcome TRUE reported 6919_3_HBZ_HBAZ HBZ
chr16 449213 0.02119 4.323e-14 35319 TRUE reported textfile 2 1 TRUE

6539 rs59548273 A G A G -0.1991 0.0184 0.01614 NA FALSE FALSE FALSE
GCST006061 0.0456 0.6864 537409 outcome TRUE reported 6919_3_HBZ_HBAZ HBZ
chr16 1213949 0.034418 7.264e-09 35316 TRUE reported textfile 2 1
TRUE

6540 rs62108438 C T C T -0.0511 -0.01 0.41675 NA FALSE FALSE FALSE
GCST006061 0.0076 0.184 537409 outcome TRUE reported 6919_3_HBZ_HBAZ HBZ
chr19 12889433 0.008487 1.73e-09 35381 TRUE reported textfile 2
1 TRUE

6541 rs635243 C A C A -0.0646 -0.0023 0.47636 NA FALSE FALSE FALSE
GCST006061 0.0073 0.7566 537409 outcome TRUE reported 6919_3_HBZ_HBAZ HBZ
chr6 139522213 0.008352 1.033e-14 35354 TRUE reported textfile 2
1 TRUE

6542 rs704 A G A G 0.2053 -0.0155 0.49189 NA FALSE FALSE FALSE GCST006061
0.0072 0.03144 537409 outcome TRUE reported 6919_3_HBZ_HBAZ HBZ chr17
28367840 0.008302 5.348e-135 35360 TRUE reported textfile 2 1 TRUE

6543 rs78369087 A G A G -0.3012 -0.0065 0.0227 NA FALSE FALSE FALSE
GCST006061 0.0187 0.7276 537409 outcome TRUE reported 6919_3_HBZ_HBAZ HBZ
chr16 194786 0.029208 6.214e-25 35317 TRUE reported textfile 2 1
TRUE

6544 rs8642 A G A G -0.1303 -0.011 0.27373 NA FALSE FALSE FALSE GCST006061
0.0085 0.1981 537409 outcome TRUE reported 6919_3_HBZ_HBAZ HBZ chr16 583764
0.009924 2.205e-39 35311 TRUE reported textfile 2 1 TRUE

6545 rs10904861 T C T C -0.0583 -0.0155 0.37165 NA FALSE FALSE FALSE
GCST006061 0.0076 0.04065 537409 outcome TRUE reported 10880_38_FAM163B_F163B
FAM163B chr10 16988776 0.008213 1.26e-12 35354 TRUE reported
textfile 2 1 TRUE

6546 rs1797077 T C T C -0.2344 0.0046 0.47532 NA FALSE FALSE FALSE
GCST006061 0.0073 0.5262 537409 outcome TRUE reported 10880_38_FAM163B_F163B
FAM163B chr10 16811253 0.007884 2.946e-194 35351 TRUE reported
textfile 2 1 TRUE

6547 rs61751507 T C T C 0.3289 0.0047 0.04162 NA FALSE FALSE FALSE
GCST006061 0.018 0.794 537409 outcome TRUE reported 10880_38_FAM163B_F163B
FAM163B chr10 100069757 0.019833 9.235e-62 35356 TRUE reported
textfile 2 1 TRUE

6548 rs704 A G A G 0.0551 -0.0155 0.49189 NA FALSE FALSE FALSE GCST006061
0.0072 0.03144 537409 outcome TRUE reported 10880_38_FAM163B_F163B FAM163B
chr17 28367840 0.007961 4.483e-12 35348 TRUE reported textfile 2
1 TRUE

6549 rs7634194 C T C T 0.0476 0.0096 0.34569 NA FALSE FALSE FALSE
GCST006061 0.008 0.2339 537409 outcome TRUE reported 10880_38_FAM163B_F163B
FAM163B chr3 194343888 0.008422 1.587e-08 35287 TRUE reported
textfile 2 1 TRUE

6550 rs10904881 A C A C 0.2471 -0.0046 0.40794 NA FALSE FALSE FALSE
GCST006061 0.0074 0.5331 537409 outcome TRUE reported 12635_9_TRDMT1_TRDMT
TRDMT1 chr10 17130953 0.008171 1e-200 35354 TRUE reported textfile
2 1 TRUE

6551 rs1354034 C T C T 0.0595 0.0088 0.38119 NA FALSE FALSE FALSE
GCST006061 0.0073 0.2248 537409 outcome TRUE reported 12635_9_TRDMT1_TRDMT
TRDMT1 chr3 56815721 0.008268 6.2e-13 35347 TRUE reported textfile
2 1 TRUE

6552 rs4311960 T C T C 0.0661 0.0041 0.17848 NA FALSE FALSE FALSE
GCST006061 0.0095 0.6631 537409 outcome TRUE reported 12635_9_TRDMT1_TRDMT
TRDMT1 chr10 19988154 0.010546 3.66e-10 35350 TRUE reported
textfile 2 1 TRUE

6553 rs10904915 C T C T 0.0559 -2.00E-04 0.27842 NA FALSE FALSE FALSE
GCST006061 0.0081 0.9808 537409 outcome TRUE reported 15540_6_VIM_Vimentin
VIM chr10 17270691 0.008837 2.516e-10 35360 TRUE reported textfile
2 1 TRUE

6554 rs114694170 C T C T 0.0852 0.0053 0.08378 NA FALSE FALSE FALSE
GCST006061 0.0175 0.7624 537409 outcome TRUE reported 15540_6_VIM_Vimentin
VIM chr5 88884379 0.014498 4.187e-09 35344 TRUE reported textfile
2 1 TRUE

6555 rs3902042 C T C T 0.0446 -0.0015 0.40655 NA FALSE FALSE FALSE
GCST006061 0.0073 0.8426 537409 outcome TRUE reported 15540_6_VIM_Vimentin
VIM chr10 17023068 0.008123 4.008e-08 35365 TRUE reported textfile
2 1 TRUE

6556 rs438811 T C T C -0.0957 -5.00E-04 0.23201 NA FALSE FALSE FALSE
GCST006061 0.0088 0.9556 537409 outcome TRUE reported 15540_6_VIM_Vimentin
VIM chr19 44913484 0.009533 1.034e-23 35366 TRUE reported textfile
2 1 TRUE
6557 rs4639017 C T C T -0.0521 -0.0061 0.39283 NA FALSE FALSE FALSE
GCST006061 0.0073 0.4022 537409 outcome TRUE reported 15540_6_VIM_Vimentin
VIM chr3 165833296 0.00816 1.712e-10 35339 TRUE reported textfile 2
1 TRUE
6558 rs796667 T G T G -0.0763 0.0011 0.42958 NA FALSE FALSE FALSE
GCST006061 0.0077 0.8878 537409 outcome TRUE reported 15540_6_VIM_Vimentin
VIM chr10 16904820 0.008063 2.989e-21 35365 TRUE reported textfile
2 1 TRUE
6559 rs114694170 C T C T -0.0957 0.0053 0.08378 NA FALSE FALSE FALSE
GCST006061 0.0175 0.7624 537409 outcome TRUE reported
3903_49_SNX4_Sorting_nexin_4 SNX4 chr5 88884379 0.014848 1.154e-10
35344 TRUE reported textfile 2 1 TRUE
6560 rs1354034 C T C T 0.0733 0.0088 0.38119 NA FALSE FALSE FALSE
GCST006061 0.0073 0.2248 537409 outcome TRUE reported
3903_49_SNX4_Sorting_nexin_4 SNX4 chr3 56815721 0.008409 2.862e-18
35356 TRUE reported textfile 2 1 TRUE
6561 rs2645984 C T C T -0.1063 -0.0237 0.42441 NA FALSE FALSE FALSE
GCST006061 0.0074 0.001352 537409 outcome TRUE reported
3903_49_SNX4_Sorting_nexin_4 SNX4 chr9 112740762 0.008266 7.616e-38
35371 TRUE reported textfile 2 1 TRUE
6562 rs8193003 C T C T -0.0527 0.005 0.4212 NA FALSE FALSE FALSE
GCST006061 0.0073 0.4876 537409 outcome TRUE reported
3903_49_SNX4_Sorting_nexin_4 SNX4 chr9 132990109 0.008251 1.69e-10
35367 TRUE reported textfile 2 1 TRUE
6563 rs11190141 T C T C 0.0477 -0.0152 0.36345 NA FALSE FALSE FALSE
GCST006061 0.0073 0.03695 537409 outcome TRUE reported 4924_32_MMP1_MMP_1
MMP1 chr10 99532633 0.008448 1.642e-08 35371 TRUE reported
textfile 2 1 TRUE
6564 rs11243920 T C T C 0.1332 -0.0275 0.03308 NA FALSE FALSE FALSE
GCST006061 0.0265 0.2997 537409 outcome TRUE reported 4924_32_MMP1_MMP_1
MMP1 chr9 132853740 0.023081 7.876e-09 35373 TRUE reported
textfile 2 1 TRUE
6565 rs114694170 C T C T -0.1016 0.0053 0.08378 NA FALSE FALSE FALSE
GCST006061 0.0175 0.7624 537409 outcome TRUE reported 4924_32_MMP1_MMP_1
MMP1 chr5 88884379 0.01473 5.294e-12 35344 TRUE reported textfile
2 1 TRUE
6566 rs117342740 T C T C 0.2558 -0.0199 0.01843 NA FALSE FALSE FALSE
GCST006061 0.0231 0.3894 537409 outcome TRUE reported 4924_32_MMP1_MMP_1
MMP1 chr11 103409586 0.032132 1.708e-15 35364 TRUE reported
textfile 2 1 TRUE
6567 rs117695939 A G A G -0.2516 0.0293 0.04585 NA FALSE FALSE FALSE
GCST006061 0.029 0.3129 537409 outcome TRUE reported 4924_32_MMP1_MMP_1
MMP1 chr11 103595802 0.020403 6.127e-35 35362 TRUE reported
textfile 2 1 TRUE
6568 rs12767683 A C A C -0.0611 0.0038 0.26443 NA FALSE FALSE FALSE
GCST006061 0.0079 0.6284 537409 outcome TRUE reported 4924_32_MMP1_MMP_1
MMP1 chr10 102556824 0.009249 3.937e-11 35370 TRUE reported
textfile 2 1 TRUE
6569 rs138804703 T C T C 0.3049 -0.0488 0.01115 NA FALSE FALSE FALSE
GCST006061 0.0495 0.3243 537409 outcome TRUE reported 4924_32_MMP1_MMP_1
MMP1 chr11 99482791 0.040747 7.277e-14 35354 TRUE reported
textfile 2 1 TRUE
6570 rs1434282 T C T C 0.0567 -0.0021 0.26524 NA FALSE FALSE FALSE
GCST006061 0.008 0.7916 537409 outcome TRUE reported 4924_32_MMP1_MMP_1
MMP1 chr1 199041592 0.009322 1.185e-09 35343 TRUE reported
textfile 2 1 TRUE
6571 rs144817684 T C T C -0.2269 0.0349 0.01538 NA FALSE FALSE FALSE
GCST006061 0.0256 0.1727 537409 outcome TRUE reported 4924_32_MMP1_MMP_1
MMP1 chr11 100036027 0.034921 8.168e-11 35352 TRUE reported
textfile 2 1 TRUE
6572 rs2846703 A G A G -0.1423 -0.0078 0.26963 NA FALSE FALSE FALSE
GCST006061 0.0081 0.3349 537409 outcome TRUE reported 4924_32_MMP1_MMP_1

MMP1 chr11 102701510 0.009659 3.994e-49 35365 TRUE reported
textfile 2 1 TRUE
6573 rs35340377 A G A G -0.0647 0.0075 0.25094 NA FALSE FALSE FALSE
GCST006061 0.0082 0.3585 537409 outcome TRUE reported 4924_32_MMP1_MMP_1
MMP1 chr1 247874908 0.009458 7.884e-12 35156 TRUE reported
textfile 2 1 TRUE
6574 rs514921 G A G A -0.303 0.005 0.33497 NA FALSE FALSE FALSE
GCST006061 0.0083 0.5486 537409 outcome TRUE reported 4924_32_MMP1_MMP_1
MMP1 chr11 102798499 0.008974 1e-200 35363 TRUE reported textfile
2 1 TRUE
6575 rs6591003 C T C T -0.3138 4e-04 0.02699 NA FALSE FALSE FALSE
GCST006061 0.0183 0.9835 537409 outcome TRUE reported 4924_32_MMP1_MMP_1
MMP1 chr11 103042350 0.026405 1.433e-32 35364 TRUE reported
textfile 2 1 TRUE
6576 rs7774377 A G A G 0.0716 0.0143 0.44972 NA FALSE FALSE FALSE
GCST006061 0.0071 0.04378 537409 outcome TRUE reported 4924_32_MMP1_MMP_1
MMP1 chr6 109281240 0.008202 2.559e-18 35380 TRUE reported
textfile 2 1 TRUE
6577 rs7938172 A G A G 0.054 -0.0176 0.35166 NA FALSE FALSE FALSE
GCST006061 0.0072 0.01508 537409 outcome TRUE reported 4924_32_MMP1_MMP_1
MMP1 chr11 102123149 0.009023 2.167e-09 35363 TRUE reported
textfile 2 1 TRUE
6578 rs10922098 T C T C -0.0527 -0.009 0.40011 NA FALSE FALSE FALSE
GCST006061 0.0075 0.2275 537409 outcome TRUE reported 7897_75_DLG3_DLG3
DLG3 chr1 196695521 0.008403 3.582e-10 35338 TRUE reported
textfile 2 1 TRUE
6579 rs111432261 G A G A -0.1123 0.0336 0.04427 NA FALSE FALSE FALSE
GCST006061 0.0235 0.1517 537409 outcome TRUE reported 7897_75_DLG3_DLG3
DLG3 chr5 92709189 0.020339 3.362e-08 35340 TRUE reported
textfile 2 1 TRUE
6580 rs114694170 C T C T -0.2855 0.0053 0.08378 NA FALSE FALSE FALSE
GCST006061 0.0175 0.7624 537409 outcome TRUE reported 7897_75_DLG3_DLG3
DLG3 chr5 88884379 0.014883 5.136e-82 35344 TRUE reported
textfile 2 1 TRUE
6581 rs342295 T C T C -0.0545 -0.0019 0.4541 NA FALSE FALSE FALSE
GCST006061 0.0073 0.7921 537409 outcome TRUE reported 7897_75_DLG3_DLG3
DLG3 chr7 106732314 0.008225 3.437e-11 35357 TRUE reported
textfile 2 1 TRUE
6582 rs34826779 T G T G 0.0944 0.0171 0.21632 NA FALSE FALSE FALSE
GCST006061 0.008 0.03314 537409 outcome TRUE reported 7897_75_DLG3_DLG3
DLG3 chr8 105558736 0.010018 4.4e-21 35365 TRUE reported textfile
2 1 TRUE
6583 rs74360467 G A G A -0.1823 -0.0227 0.01872 NA FALSE FALSE FALSE
GCST006061 0.0259 0.3803 537409 outcome TRUE reported 7897_75_DLG3_DLG3
DLG3 chr5 89396922 0.030474 2.201e-09 35344 TRUE reported
textfile 2 1 TRUE
6584 rs9494145 C T C T 0.0545 -0.0073 0.24334 NA FALSE FALSE FALSE
GCST006061 0.0083 0.3799 537409 outcome TRUE reported 7897_75_DLG3_DLG3
DLG3 chr6 135111414 0.009597 1.355e-08 35372 TRUE reported
textfile 2 1 TRUE
6585 rs114547897 C T C T -0.7547 0.0627 0.01444 NA FALSE FALSE FALSE
GCST006061 0.0496 0.2057 537409 outcome TRUE reported 3292_75_CD48_CD48
CD48 chr1 160597708 0.034322 3.707e-107 35358 TRUE reported
textfile 2 1 TRUE
6586 rs1260326 C T C T 0.0512 -0.0069 0.34132 NA FALSE FALSE FALSE
GCST006061 0.0073 0.3401 537409 outcome TRUE reported 3292_75_CD48_CD48
CD48 chr2 27508073 0.008884 8.268e-09 35373 TRUE reported
textfile 2 1 TRUE
6587 rs13108218 G A G A 0.0475 -0.01 0.36204 NA FALSE FALSE FALSE
GCST006061 0.0074 0.1774 537409 outcome TRUE reported 3292_75_CD48_CD48
CD48 chr4 3442204 0.008695 4.676e-08 35280 TRUE reported textfile
2 1 TRUE
6588 rs143501783 C T C T 0.2674 -0.0333 0.01116 NA FALSE FALSE FALSE
GCST006061 0.0443 0.4525 537409 outcome TRUE reported 3292_75_CD48_CD48
CD48 chr17 7897001 0.038172 2.467e-12 35373 TRUE reported textfile
2 1 TRUE

6589 rs188662236 A C A C -0.2274 -0.0161 0.01259 NA FALSE FALSE FALSE
GCST006061 0.0453 0.7214 537409 outcome TRUE reported 3292_75_CD48_CD48
CD48 chr1 159273728 0.037093 8.76e-10 35358 TRUE reported
textfile 2 1 TRUE
6590 rs1980606 A G A G -0.2807 -0.003 0.18289 NA FALSE FALSE FALSE
GCST006061 0.0088 0.7378 537409 outcome TRUE reported 3292_75_CD48_CD48
CD48 chr1 160684996 0.010909 5.147e-146 35356 TRUE reported
textfile 2 1 TRUE
6591 rs2659005 T C T C 0.0692 -0.0028 0.44601 NA FALSE FALSE FALSE
GCST006061 0.0073 0.7016 537409 outcome TRUE reported 3292_75_CD48_CD48
CD48 chr17 81244914 0.008481 3.364e-16 35133 TRUE reported
textfile 2 1 TRUE
6592 rs3184504 C T C T -0.1075 0.0074 0.37862 NA FALSE FALSE FALSE
GCST006061 0.0075 0.3235 537409 outcome TRUE reported 3292_75_CD48_CD48
CD48 chr12 111446804 0.00865 1.836e-35 35371 TRUE reported textfile
2 1 TRUE
6593 rs41266895 T C T C -0.3579 0.0199 0.01726 NA FALSE FALSE FALSE
GCST006061 0.0456 0.6631 537409 outcome TRUE reported 3292_75_CD48_CD48
CD48 chr1 160426376 0.033078 2.769e-27 35359 TRUE reported
textfile 2 1 TRUE
6594 rs6427541 T C T C 0.0881 -0.0097 0.33835 NA FALSE FALSE FALSE
GCST006061 0.0084 0.2475 537409 outcome TRUE reported 3292_75_CD48_CD48
CD48 chr1 160714121 0.008961 8.23e-23 35352 TRUE reported
textfile 2 1 TRUE
6595 rs10909012 G T G T 0.1029 0.0125 0.38697 NA FALSE FALSE FALSE
GCST006061 0.0079 0.114 537409 outcome TRUE reported 3220_40_RET_RET RET
chr10 41803558 0.008452 4.272e-34 35331 TRUE reported textfile 2
1 TRUE
6596 rs116883658 A G A G -0.1896 0.0522 0.02659 NA FALSE FALSE FALSE
GCST006061 0.0339 0.1238 537409 outcome TRUE reported 3220_40_RET_RET RET
chr10 35964729 0.025074 3.982e-14 35338 TRUE reported textfile 2
1 TRUE
6597 rs2795496 A G A G -0.3522 -0.0171 0.17774 NA FALSE FALSE FALSE
GCST006061 0.0096 0.07433 537409 outcome TRUE reported 3220_40_RET_RET RET
chr10 42842394 0.010612 1e-200 35331 TRUE reported textfile 2 1
TRUE
6598 rs2946992 C T C T -0.0715 0.0311 0.11101 NA FALSE FALSE FALSE
GCST006061 0.0111 0.005075 537409 outcome TRUE reported 3220_40_RET_RET RET
chr10 43358831 0.01307 4.484e-08 35328 TRUE reported textfile 2 1
TRUE
6599 rs4278528 C T C T 0.0558 -0.0015 0.49208 NA FALSE FALSE FALSE
GCST006061 0.0071 0.8347 537409 outcome TRUE reported 3220_40_RET_RET RET
chr11 126388627 0.008082 5.042e-12 35375 TRUE reported textfile 2
1 TRUE
6600 rs752978 T C T C 0.151 0.0036 0.43338 NA FALSE FALSE FALSE
GCST006061 0.0072 0.6196 537409 outcome TRUE reported 3220_40_RET_RET RET
chr10 43092981 0.008262 1.265e-74 35330 TRUE reported textfile 2
1 TRUE
6601 rs12039758 A G A G 0.3019 -0.0087 0.21638 NA FALSE FALSE FALSE
GCST006061 0.0095 0.3628 537409 outcome TRUE reported 8973_23_FCRL4_FCRL4
FCRL4 chr1 157510525 0.009862 1e-200 35371 TRUE reported textfile
2 1 TRUE
6602 rs139610741 A G A G -0.2312 -0.0087 0.01377 NA FALSE FALSE FALSE
GCST006061 0.0343 0.7998 537409 outcome TRUE reported 8973_23_FCRL4_FCRL4
FCRL4 chr1 119265390 0.034145 1.278e-11 35360 TRUE reported
textfile 2 1 TRUE
6603 rs1432296 T C T C -0.058 0.0315 0.16194 NA FALSE FALSE FALSE
GCST006061 0.0106 0.00285 537409 outcome TRUE reported 8973_23_FCRL4_FCRL4
FCRL4 chr2 60841032 0.010002 6.681e-09 35357 TRUE reported
textfile 2 1 TRUE
6604 rs148317837 A C A C 0.1385 0.0075 0.02914 NA FALSE FALSE FALSE
GCST006061 0.0422 0.8596 537409 outcome TRUE reported 8973_23_FCRL4_FCRL4
FCRL4 chr8 22701068 0.021975 2.924e-10 35369 TRUE reported
textfile 2 1 TRUE
6605 rs2511739 C T C T -0.0461 -0.021 0.29997 NA FALSE FALSE FALSE
GCST006061 0.0084 0.01267 537409 outcome TRUE reported 8973_23_FCRL4_FCRL4

FCRL4 chr8 102569746 0.007979 7.584e-09 35360 TRUE reported
textfile 2 1 TRUE
6606 rs34296440 C T C T -0.1878 0.0122 0.09905 NA FALSE FALSE FALSE
GCST006061 0.0124 0.3245 537409 outcome TRUE reported 8973_23_FCRL4_FCRL4
FCRL4 chr1 157360707 0.013631 3.469e-43 35366 TRUE reported
textfile 2 1 TRUE
6607 rs58036957 A C A C 0.0682 -0.0015 0.10105 NA FALSE FALSE FALSE
GCST006061 0.0104 0.8842 537409 outcome TRUE reported 8973_23_FCRL4_FCRL4
FCRL4 chr15 56080858 0.012136 1.911e-08 35361 TRUE reported
textfile 2 1 TRUE
6608 rs61751627 G A G A -0.508 0.0805 0.01059 NA FALSE FALSE FALSE
GCST006061 0.0652 0.217 537409 outcome TRUE reported 8973_23_FCRL4_FCRL4
FCRL4 chr1 156065926 0.039177 1.887e-38 35361 TRUE reported
textfile 2 1 TRUE
6609 rs77328788 T C T C -0.1065 -0.0115 0.12995 NA FALSE FALSE FALSE
GCST006061 0.0116 0.3227 537409 outcome TRUE reported 8973_23_FCRL4_FCRL4
FCRL4 chr9 36994015 0.010741 3.574e-23 35353 TRUE reported
textfile 2 1 TRUE
6610 rs78269692 C T C T 0.0844 0.0188 0.07622 NA FALSE FALSE FALSE
GCST006061 0.0181 0.3 537409 outcome TRUE reported 8973_23_FCRL4_FCRL4 FCRL4
chr19 13047463 0.013613 5.645e-10 35379 TRUE reported textfile 2
1 TRUE
6611 rs857680 G A G A 0.0648 -0.0018 0.42286 NA FALSE FALSE FALSE
GCST006061 0.0072 0.8016 537409 outcome TRUE reported 8973_23_FCRL4_FCRL4
FCRL4 chr1 158666062 0.008306 6.092e-15 35360 TRUE reported
textfile 2 1 TRUE
6612 rs9419741 G A G A 0.0421 0.0087 0.47923 NA FALSE FALSE FALSE
GCST006061 0.0072 0.2262 537409 outcome TRUE reported 8973_23_FCRL4_FCRL4
FCRL4 chr10 92670768 0.007343 9.867e-09 35372 TRUE reported
textfile 2 1 TRUE
6613 rs10915471 G T G T 0.046 -0.0043 0.48853 NA FALSE FALSE FALSE
GCST006061 0.0072 0.5514 537409 outcome TRUE reported 11264_33_XDH_XDH XDH
chr1 4207829 0.007807 3.809e-09 35343 TRUE reported textfile 2 1
TRUE
6614 rs10922098 T C T C -0.0435 -0.009 0.40011 NA FALSE FALSE FALSE
GCST006061 0.0075 0.2275 537409 outcome TRUE reported 11264_33_XDH_XDH XDH
chr1 196695521 0.007938 4.259e-08 35329 TRUE reported textfile 2
1 TRUE
6615 rs10915983 T C T C -0.0493 0.0132 0.43513 NA FALSE FALSE FALSE
GCST006061 0.0076 0.08339 537409 outcome TRUE reported 7251_64_C1QTNF3_C1QT3
C1QTNF3 chr1 226347052 0.008177 1.648e-09 35355 TRUE reported
textfile 2 1 TRUE
6616 rs11190970 A G A G 0.0652 0.0378 0.21108 NA FALSE FALSE FALSE
GCST006061 0.0092 4.064e-05 537409 outcome TRUE reported
7251_64_C1QTNF3_C1QT3 C1QTNF3 chr10 101368575 0.009921 4.978e-11 35369
TRUE reported textfile 2 1 TRUE
6617 rs11900031 G A G A -0.0736 -0.0234 0.11083 NA FALSE FALSE FALSE
GCST006061 0.0113 0.03943 537409 outcome TRUE reported 7251_64_C1QTNF3_C1QT3
C1QTNF3 chr2 17749607 0.012862 1.051e-08 35376 TRUE reported
textfile 2 1 TRUE
6618 rs506317 T C T C -0.0479 -0.0159 0.43587 NA FALSE FALSE FALSE
GCST006061 0.0075 0.03311 537409 outcome TRUE reported 7251_64_C1QTNF3_C1QT3
C1QTNF3 chr1 196748023 0.008163 4.417e-09 35339 TRUE reported
textfile 2 1 TRUE
6619 rs602633 G T G T 0.0595 -0.0162 0.20391 NA FALSE FALSE FALSE
GCST006061 0.0088 0.06452 537409 outcome TRUE reported 7251_64_C1QTNF3_C1QT3
C1QTNF3 chr1 109278889 0.010089 3.686e-09 35344 TRUE reported
textfile 2 1 TRUE
6620 rs77262773 T C T C 0.1526 0.014 0.02263 NA FALSE FALSE FALSE
GCST006061 0.0283 0.6205 537409 outcome TRUE reported 7251_64_C1QTNF3_C1QT3
C1QTNF3 chr17 69253570 0.026878 1.366e-08 35371 TRUE reported
textfile 2 1 TRUE
6621 rs840390 A G A G -0.1854 -0.0039 0.13114 NA FALSE FALSE FALSE
GCST006061 0.0107 0.7156 537409 outcome TRUE reported 7251_64_C1QTNF3_C1QT3
C1QTNF3 chr5 34018518 0.011973 4.423e-54 35383 TRUE reported
textfile 2 1 TRUE

6622 rs10918270 A G A G -0.0493 -0.0142 0.38885 NA FALSE FALSE FALSE GCST006061 0.0074 0.0547 537409 outcome TRUE reported 11277_23_ATF6_ATF6A ATF6 chr1 161945711 0.008385 4.115e-09 35352 TRUE reported textfile 2 1 TRUE
6623 rs147456212 A G A G 0.2198 0 0.01225 NA FALSE FALSE FALSE GCST006061 0.028 0.9997 537409 outcome TRUE reported 11277_23_ATF6_ATF6A ATF6 chr10 15318139 0.037563 4.871e-09 35358 TRUE reported textfile 2 1 TRUE
6624 rs149072800 C T C T 0.1341 0.0238 0.03494 NA FALSE FALSE FALSE GCST006061 0.0255 0.3513 537409 outcome TRUE reported 11277_23_ATF6_ATF6A ATF6 chr1 91980163 0.02271 3.528e-09 35343 TRUE reported textfile 2 1 TRUE
6625 rs163279 T C T C 0.0524 -0.0187 0.38774 NA FALSE FALSE FALSE GCST006061 0.0076 0.01364 537409 outcome TRUE reported 11277_23_ATF6_ATF6A ATF6 chr5 16500772 0.008425 4.974e-10 35361 TRUE reported textfile 2 1 TRUE
6626 rs2273967 T C T C 0.0953 0.0115 0.24296 NA FALSE FALSE FALSE GCST006061 0.0082 0.1615 537409 outcome TRUE reported 11277_23_ATF6_ATF6A ATF6 chr1 230279547 0.009568 2.281e-23 35351 TRUE reported textfile 2 1 TRUE
6627 rs2583223 A G A G 0.081 -0.0043 0.32004 NA FALSE FALSE FALSE GCST006061 0.0074 0.5592 537409 outcome TRUE reported 11277_23_ATF6_ATF6A ATF6 chr12 103923063 0.008771 2.585e-20 35352 TRUE reported textfile 2 1 TRUE
6628 rs4796383 A G A G -0.0477 -0.0031 0.47118 NA FALSE FALSE FALSE GCST006061 0.0072 0.6671 537409 outcome TRUE reported 11277_23_ATF6_ATF6A ATF6 chr17 7118971 0.008226 6.685e-09 35367 TRUE reported textfile 2 1 TRUE
6629 rs572553 A G A G 0.063 0.0028 0.39505 NA FALSE FALSE FALSE GCST006061 0.0077 0.718 537409 outcome TRUE reported 11277_23_ATF6_ATF6A ATF6 chr4 55394851 0.008351 4.551e-14 35351 TRUE reported textfile 2 1 TRUE
6630 rs72706230 A C A C 0.1103 -0.0064 0.04535 NA FALSE FALSE FALSE GCST006061 0.0178 0.7215 537409 outcome TRUE reported 11277_23_ATF6_ATF6A ATF6 chr14 94361316 0.019923 3.089e-08 35363 TRUE reported textfile 2 1 TRUE
6631 rs737908 A G A G 0.066 0.0094 0.3706 NA FALSE FALSE FALSE GCST006061 0.0074 0.2028 537409 outcome TRUE reported 11277_23_ATF6_ATF6A ATF6 chr22 29992313 0.008455 5.914e-15 35360 TRUE reported textfile 2 1 TRUE
6632 rs77262773 T C T C 0.1933 0.014 0.02263 NA FALSE FALSE FALSE GCST006061 0.0283 0.6205 537409 outcome TRUE reported 11277_23_ATF6_ATF6A ATF6 chr17 69253570 0.027131 1.043e-12 35362 TRUE reported textfile 2 1 TRUE
6633 rs79688957 C T C T 0.2308 -0.0394 0.02868 NA FALSE FALSE FALSE GCST006061 0.0316 0.2117 537409 outcome TRUE reported 11277_23_ATF6_ATF6A ATF6 chr10 14138082 0.02459 6.233e-21 35358 TRUE reported textfile 2 1 TRUE
6634 rs8066387 C T C T -0.046 -0.0076 0.4393 NA FALSE FALSE FALSE GCST006061 0.0072 0.292 537409 outcome TRUE reported 11277_23_ATF6_ATF6A ATF6 chr17 18284307 0.008258 2.538e-08 35349 TRUE reported textfile 2 1 TRUE
6635 rs8176743 T C T C 0.112 0.017 0.06488 NA FALSE FALSE FALSE GCST006061 0.0128 0.1834 537409 outcome TRUE reported 11277_23_ATF6_ATF6A ATF6 chr9 133256028 0.01668 1.885e-11 35361 TRUE reported textfile 2 1 TRUE
6636 rs9333273 A G A G 0.2223 0.0496 0.01641 NA FALSE FALSE FALSE GCST006061 0.0337 0.1407 537409 outcome TRUE reported 11277_23_ATF6_ATF6A ATF6 chr10 15576891 0.032315 6.021e-12 35356 TRUE reported textfile 2 1 TRUE
6637 rs9825233 T C T C 0.0593 -0.0611 0.37766 NA FALSE FALSE FALSE GCST006061 0.0074 1.07e-16 537409 outcome TRUE reported 11277_23_ATF6_ATF6A ATF6 chr3 12801869 0.008466 2.477e-12 35360 TRUE reported textfile 2 1 TRUE
6638 rs10918341 G A G A 0.2459 -0.0158 0.303 NA FALSE FALSE FALSE GCST006061 0.0078 0.04392 537409 outcome TRUE reported 15388_24_FCGR3A_FcRIIIa FCGR3A chr1 161980609 0.009874 6.623e-137 35360 TRUE reported

textfile 2 1 TRUE
6639 rs12095544 C T C T -0.23 0.0128 0.07008 NA FALSE FALSE FALSE
GCST006061 0.013 0.3215 537409 outcome TRUE reported 15388_24_FCGR3A_FcRIIIa
FCGR3A chr1 162062119 0.017753 2.189e-38 35359 TRUE reported
textfile 2 1 TRUE
6640 rs143426473 T C T C 0.4728 0.0033 0.03664 NA FALSE FALSE FALSE
GCST006061 0.022 0.8822 537409 outcome TRUE reported 15388_24_FCGR3A_FcRIIIa
FCGR3A chr1 160687337 0.024347 5.33e-84 35356 TRUE reported
textfile 2 1 TRUE
6641 rs1466933 G T G T -0.2385 0.0076 0.03463 NA FALSE FALSE FALSE
GCST006061 0.0139 0.5837 537409 outcome TRUE reported 15388_24_FCGR3A_FcRIIIa
FCGR3A chr1 160579090 0.025163 2.583e-21 35358 TRUE reported
textfile 2 1 TRUE
6642 rs16849374 C T C T 0.201 0.0065 0.0304 NA FALSE FALSE FALSE
GCST006061 0.0182 0.721 537409 outcome TRUE reported 15388_24_FCGR3A_FcRIIIa
FCGR3A chr1 165680478 0.026448 2.966e-14 35357 TRUE reported
textfile 2 1 TRUE
6643 rs1935992 C T C T 0.0734 0.0192 0.18943 NA FALSE FALSE FALSE
GCST006061 0.0086 0.02601 537409 outcome TRUE reported 15388_24_FCGR3A_FcRIIIa
FCGR3A chr1 118150594 0.011665 3.126e-10 35366 TRUE reported
textfile 2 1 TRUE
6644 rs278373 T C T C -0.0643 -0.0072 0.48917 NA FALSE FALSE FALSE
GCST006061 0.0071 0.3111 537409 outcome TRUE reported 15388_24_FCGR3A_FcRIIIa
FCGR3A chr3 98735107 0.006806 3.459e-21 35330 TRUE reported
textfile 2 1 TRUE
6645 rs35118879 A C A C 0.0923 0.0093 0.08908 NA FALSE FALSE FALSE
GCST006061 0.0146 0.526 537409 outcome TRUE reported 15388_24_FCGR3A_FcRIIIa
FCGR3A chr1 150628204 0.016134 1.061e-08 35358 TRUE reported
textfile 2 1 TRUE
6646 rs4657041 C T C T -0.4515 0.0163 0.44469 NA FALSE FALSE FALSE
GCST006061 0.0072 0.02419 537409 outcome TRUE reported 15388_24_FCGR3A_FcRIIIa
FCGR3A chr1 161509069 0.005024 1e-200 35351 TRUE reported textfile
2 1 TRUE
6647 rs4657260 G T G T 0.3795 -0.005 0.01342 NA FALSE FALSE FALSE
GCST006061 0.0289 0.8615 537409 outcome TRUE reported 15388_24_FCGR3A_FcRIIIa
FCGR3A chr1 163316131 0.039485 7.173e-22 35359 TRUE reported
textfile 2 1 TRUE
6648 rs56115403 A G A G 0.0496 0.0012 0.21597 NA FALSE FALSE FALSE
GCST006061 0.01 0.901 537409 outcome TRUE reported 15388_24_FCGR3A_FcRIIIa
FCGR3A chr17 7160579 0.008314 2.432e-09 35371 TRUE reported textfile
2 1 TRUE
6649 rs653178 T C T C -0.0551 0.0076 0.38124 NA FALSE FALSE FALSE
GCST006061 0.0075 0.3129 537409 outcome TRUE reported 15388_24_FCGR3A_FcRIIIa
FCGR3A chr12 111569952 0.007008 3.768e-15 35371 TRUE reported
textfile 2 1 TRUE
6650 rs7047770 A G A G -0.0463 0.0017 0.34125 NA FALSE FALSE FALSE
GCST006061 0.0077 0.828 537409 outcome TRUE reported 15388_24_FCGR3A_FcRIIIa
FCGR3A chr9 137036493 0.00717 1.062e-10 35261 TRUE reported textfile
2 1 TRUE
6651 rs7147722 T G T G -0.0372 -0.0014 0.45254 NA FALSE FALSE FALSE
GCST006061 0.0074 0.8486 537409 outcome TRUE reported 15388_24_FCGR3A_FcRIIIa
FCGR3A chr14 49645994 0.00682 4.907e-08 35362 TRUE reported textfile
2 1 TRUE
6652 rs75609405 A G A G 0.1681 -0.0051 0.04705 NA FALSE FALSE FALSE
GCST006061 0.0232 0.8241 537409 outcome TRUE reported 15388_24_FCGR3A_FcRIIIa
FCGR3A chr1 159835150 0.021787 1.204e-14 35351 TRUE reported
textfile 2 1 TRUE
6653 rs10920636 A G A G 0.2496 0.0074 0.4918 NA FALSE FALSE FALSE
GCST006061 0.0073 0.3105 537409 outcome TRUE reported 10758_2_KERA_KERA
KERA chr1 203402861 0.008231 1e-200 35357 TRUE reported textfile
2 1 TRUE
6654 rs1362245 A G A G 0.0523 0.007 0.28531 NA FALSE FALSE FALSE
GCST006061 0.008 0.3791 537409 outcome TRUE reported 10758_2_KERA_KERA
KERA chr2 229265954 0.009181 1.224e-08 35347 TRUE reported
textfile 2 1 TRUE
6655 rs186033146 T C T C -0.1666 -0.0045 0.0428 NA FALSE FALSE FALSE

GCST006061 0.0413 0.9129 537409 outcome TRUE reported 10758_2_KERA_KERA
KERA chr12 104377858 0.020307 2.324e-16 35348 TRUE reported
textfile 2 1 TRUE
6656 rs2686395 C T C T -0.0662 -0.0077 0.3101 NA FALSE FALSE FALSE
GCST006061 0.0074 0.2965 537409 outcome TRUE reported 10758_2_KERA_KERA
KERA chr3 165767231 0.008929 1.226e-13 35327 TRUE reported
textfile 2 1 TRUE
6657 rs1355538 G A G A -0.0691 -0.0076 0.39847 NA FALSE FALSE FALSE
GCST006061 0.0072 0.2923 537409 outcome TRUE reported
14093_10_FLT3LG_Flt3_ligand FLT3LG chr3 165787389 0.008032 7.775e-18 35338
TRUE reported textfile 2 1 TRUE
6658 rs76428106 C T C T 0.2362 -0.0446 0.01359 NA FALSE FALSE FALSE
GCST006061 0.0438 0.3085 537409 outcome TRUE reported
14093_10_FLT3LG_Flt3_ligand FLT3LG chr13 28029870 0.034219 5.106e-12 35363
TRUE reported textfile 2 1 TRUE
6659 rs111696008 A G A G -0.1588 -0.0259 0.02443 NA FALSE FALSE FALSE
GCST006061 0.0401 0.519 537409 outcome TRUE reported 19616_100_EFHD1_EFHD1
EFHD1 chr2 216622986 0.027336 6.278e-09 35359 TRUE reported
textfile 2 1 TRUE
6660 rs117827303 T C T C 0.1517 -0.0047 0.03794 NA FALSE FALSE FALSE
GCST006061 0.0216 0.8283 537409 outcome TRUE reported 19616_100_EFHD1_EFHD1
EFHD1 chr12 7123080 0.021179 7.898e-13 35391 TRUE reported textfile
2 1 TRUE
6661 rs12146727 A G A G -0.1886 -0.0037 0.11875 NA FALSE FALSE FALSE
GCST006061 0.0105 0.7269 537409 outcome TRUE reported 19616_100_EFHD1_EFHD1
EFHD1 chr12 7063032 0.012922 3.01e-48 35390 TRUE reported textfile
2 1 TRUE
6662 rs141622900 A G A G 0.1443 -0.0024 0.04063 NA FALSE FALSE FALSE
GCST006061 0.0174 0.8886 537409 outcome TRUE reported 19616_100_EFHD1_EFHD1
EFHD1 chr19 44923535 0.020706 3.19e-12 35368 TRUE reported
textfile 2 1 TRUE
6663 rs144202860 A G A G -0.2022 -0.0629 0.01274 NA FALSE FALSE FALSE
GCST006061 0.0538 0.2423 537409 outcome TRUE reported 19616_100_EFHD1_EFHD1
EFHD1 chr2 216625740 0.036592 3.279e-08 35359 TRUE reported
textfile 2 1 TRUE
6664 rs1707652 T C T C 0.1302 0.0081 0.3102 NA FALSE FALSE FALSE
GCST006061 0.0074 0.2701 537409 outcome TRUE reported 19616_100_EFHD1_EFHD1
EFHD1 chr3 165761011 0.008859 6.711e-49 35339 TRUE reported
textfile 2 1 TRUE
6665 rs74615535 A G A G -0.0865 0.0211 0.08965 NA FALSE FALSE FALSE
GCST006061 0.0119 0.07636 537409 outcome TRUE reported 19616_100_EFHD1_EFHD1
EFHD1 chr8 20023725 0.014314 1.514e-09 35369 TRUE reported
textfile 2 1 TRUE
6666 rs1707652 T C T C 0.0909 0.0081 0.3102 NA FALSE FALSE FALSE
GCST006061 0.0074 0.2701 537409 outcome TRUE reported 12347_29_CCM2_CCM2
CCM2 chr3 165761011 0.008641 7.025e-26 35330 TRUE reported
textfile 2 1 TRUE
6667 rs6958168 G T G T 0.0729 -0.0146 0.17184 NA FALSE FALSE FALSE
GCST006061 0.0087 0.09624 537409 outcome TRUE reported 12347_29_CCM2_CCM2
CCM2 chr7 44980215 0.01072 1.045e-11 35343 TRUE reported textfile
2 1 TRUE
6668 rs6542680 T C T C -0.1115 -0.0012 0.18362 NA FALSE FALSE FALSE
GCST006061 0.0091 0.8935 537409 outcome TRUE reported 9872_23_CTNNA2_CTNA2
CTNNA2 chr2 3592552 0.010243 1.345e-27 35332 TRUE reported textfile
2 1 TRUE
6669 rs1355538 G A G A -0.0636 -0.0076 0.39847 NA FALSE FALSE FALSE
GCST006061 0.0072 0.2923 537409 outcome TRUE reported 11542_11_TMEM230_TM230
TMEM230 chr3 165787389 0.008135 5.384e-15 35329 TRUE reported
textfile 2 1 TRUE
6670 rs16881504 T C T C -0.0722 0.0109 0.09636 NA FALSE FALSE FALSE
GCST006061 0.0134 0.418 537409 outcome TRUE reported 12462_20_SETMAR_SETMR
SETMAR chr6 18935483 0.013169 4.188e-08 35366 TRUE reported
textfile 2 1 TRUE
6671 rs4977155 C T C T -0.0639 -0.0069 0.16663 NA FALSE FALSE FALSE
GCST006061 0.0127 0.5868 537409 outcome TRUE reported 12462_20_SETMAR_SETMR
SETMAR chr14 106473508 0.010775 3.023e-09 34957 TRUE reported

textfile 2 1 TRUE
6672 rs112930619 C A C A -0.7717 0.0129 0.02581 NA FALSE FALSE FALSE
GCST006061 0.0298 0.6654 537409 outcome TRUE reported 7795_14_KLRC3_NKG2E
KLRC3 chr17 35961002 0.025513 1e-200 35366 TRUE reported textfile
2 1 TRUE
6673 rs117517474 C T C T -0.2272 0.0943 0.01071 NA FALSE FALSE FALSE
GCST006061 0.0504 0.06137 537409 outcome TRUE reported 7795_14_KLRC3_NKG2E
KLRC3 chr17 35556493 0.039973 1.317e-08 35370 TRUE reported
textfile 2 1 TRUE
6674 rs140748737 T C T C 0.1287 0.0228 0.0382 NA FALSE FALSE FALSE
GCST006061 0.0384 0.5533 537409 outcome TRUE reported 7795_14_KLRC3_NKG2E
KLRC3 chr11 71444219 0.021043 9.591e-10 35376 TRUE reported
textfile 2 1 TRUE
6675 rs150951362 A G A G -0.717 -0.0092 0.03577 NA FALSE FALSE FALSE
GCST006061 0.0156 0.5574 537409 outcome TRUE reported 7795_14_KLRC3_NKG2E
KLRC3 chr17 35977228 0.021361 1e-200 35366 TRUE reported textfile
2 1 TRUE
6676 rs41324748 G A G A -0.5515 0.0219 0.01015 NA FALSE FALSE FALSE
GCST006061 0.0463 0.6357 537409 outcome TRUE reported 7795_14_KLRC3_NKG2E
KLRC3 chr17 35498384 0.041034 3.515e-41 35363 TRUE reported
textfile 2 1 TRUE
6677 rs4251784 A G A G -0.242 0.0162 0.04233 NA FALSE FALSE FALSE
GCST006061 0.0321 0.6139 537409 outcome TRUE reported 7795_14_KLRC3_NKG2E
KLRC3 chr17 35844448 0.020203 4.616e-33 35370 TRUE reported
textfile 2 1 TRUE
6678 rs59779517 G A G A -0.0831 -7.00E-04 0.12476 NA FALSE FALSE FALSE
GCST006061 0.0116 0.9553 537409 outcome TRUE reported 7795_14_KLRC3_NKG2E
KLRC3 chr17 34646039 0.012505 3.023e-11 35364 TRUE reported
textfile 2 1 TRUE
6679 rs77508077 A C A C 0.1246 0.035 0.11086 NA FALSE FALSE FALSE
GCST006061 0.0131 0.007712 537409 outcome TRUE reported 7795_14_KLRC3_NKG2E
KLRC3 chr11 72152689 0.013008 9.799e-22 35373 TRUE reported
textfile 2 1 TRUE
6680 rs1707652 T C T C 0.0639 0.0081 0.3102 NA FALSE FALSE FALSE
GCST006061 0.0074 0.2701 537409 outcome TRUE reported 4468_21_SPHK2_SPHK2
SPHK2 chr3 165761011 0.008655 1.547e-13 35339 TRUE reported
textfile 2 1 TRUE
6681 rs6542680 T C T C -0.0596 -0.0012 0.18362 NA FALSE FALSE FALSE
GCST006061 0.0091 0.8935 537409 outcome TRUE reported 11596_47_ZNF75D_ZN75D
ZNF75D chr2 3592552 0.010186 4.887e-09 35323 TRUE reported textfile
2 1 TRUE
6682 rs1707652 T C T C 0.0858 0.0081 0.3102 NA FALSE FALSE FALSE
GCST006061 0.0074 0.2701 537409 outcome TRUE reported 12580_7_PSMB5_PSB5
PSMB5 chr3 165761011 0.008569 1.331e-23 35330 TRUE reported
textfile 2 1 TRUE
6683 rs35209896 A C A C 0.0894 0.0267 0.09263 NA FALSE FALSE FALSE
GCST006061 0.0355 0.4521 537409 outcome TRUE reported 12580_7_PSMB5_PSB5
PSMB5 chr6 32519009 0.013598 4.886e-11 35430 TRUE reported
textfile 2 1 TRUE
6684 rs12146727 A G A G -0.1613 -0.0037 0.11875 NA FALSE FALSE FALSE
GCST006061 0.0105 0.7269 537409 outcome TRUE reported 12873_11_SPN_LEUK SPN
chr12 7063032 0.012499 4.215e-38 35381 TRUE reported textfile 2 1
TRUE
6685 rs1706435 G A G A 0.0826 0.0117 0.36981 NA FALSE FALSE FALSE
GCST006061 0.0072 0.1047 537409 outcome TRUE reported 12873_11_SPN_LEUK SPN
chr3 165743688 0.008225 9.872e-24 35330 TRUE reported textfile 2
1 TRUE
6686 rs12146727 A G A G -0.0989 -0.0037 0.11875 NA FALSE FALSE FALSE
GCST006061 0.0105 0.7269 537409 outcome TRUE reported 9607_39_PRR27_CD040
PRR27 chr12 7063032 0.012513 2.709e-15 35390 TRUE reported textfile
2 1 TRUE
6687 rs13469 T C T C -0.055 -0.0177 0.45244 NA FALSE FALSE FALSE GCST006061
0.0071 0.01333 537409 outcome TRUE reported 9607_39_PRR27_CD040 PRR27 chr17
28349109 0.007989 5.813e-12 35360 TRUE reported textfile 2 1 TRUE
6688 rs1707652 T C T C 0.0775 0.0081 0.3102 NA FALSE FALSE FALSE
GCST006061 0.0074 0.2701 537409 outcome TRUE reported 9607_39_PRR27_CD040

PRR27 chr3 165761011 0.008571 1.543e-19 35339 TRUE reported
textfile 2 1 TRUE
6689 rs111265231 T C T C 0.1099 -0.0158 0.07157 NA FALSE FALSE FALSE
GCST006061 0.0186 0.3957 537409 outcome TRUE reported 11239_49_TMPRSS6_TMPS6
TMPRSS6 chr17 14360389 0.016185 1.119e-11 35346 TRUE reported
textfile 2 1 TRUE
6690 rs117348742 C T C T -0.48 -0.0428 0.01221 NA FALSE FALSE FALSE
GCST006061 0.0344 0.2137 537409 outcome TRUE reported 11239_49_TMPRSS6_TMPS6
TMPRSS6 chr17 28414292 0.037808 6.233e-37 35352 TRUE reported
textfile 2 1 TRUE
6691 rs117451747 G A G A 0.4604 -0.0385 0.02581 NA FALSE FALSE FALSE
GCST006061 0.024 0.1093 537409 outcome TRUE reported 11239_49_TMPRSS6_TMPS6
TMPRSS6 chr17 28395871 0.025655 5.156e-72 35352 TRUE reported
textfile 2 1 TRUE
6692 rs12478841 G A G A 0.0608 0.0049 0.21569 NA FALSE FALSE FALSE
GCST006061 0.008 0.5413 537409 outcome TRUE reported 11239_49_TMPRSS6_TMPS6
TMPRSS6 chr2 27588855 0.008977 1.262e-11 35364 TRUE reported
textfile 2 1 TRUE
6693 rs137940962 T C T C 0.1537 0.0152 0.04516 NA FALSE FALSE FALSE
GCST006061 0.0216 0.4808 537409 outcome TRUE reported 11239_49_TMPRSS6_TMPS6
TMPRSS6 chr17 31642390 0.020308 3.782e-14 35352 TRUE reported
textfile 2 1 TRUE
6694 rs140390732 A C A C -0.3479 0.0526 0.01316 NA FALSE FALSE FALSE
GCST006061 0.0456 0.2487 537409 outcome TRUE reported 11239_49_TMPRSS6_TMPS6
TMPRSS6 chr17 27292324 0.036795 3.225e-21 35352 TRUE reported
textfile 2 1 TRUE
6695 rs142216432 A G A G -0.2357 0.0585 0.02132 NA FALSE FALSE FALSE
GCST006061 0.0397 0.1405 537409 outcome TRUE reported 11239_49_TMPRSS6_TMPS6
TMPRSS6 chr17 29539856 0.029041 4.813e-16 35352 TRUE reported
textfile 2 1 TRUE
6696 rs144924964 A G A G -0.4301 -0.0117 0.09574 NA FALSE FALSE FALSE
GCST006061 0.0159 0.4587 537409 outcome TRUE reported 11239_49_TMPRSS6_TMPS6
TMPRSS6 chr17 28093061 0.01409 1e-200 35351 TRUE reported textfile 2
1 TRUE
6697 rs147804221 T C T C -0.2165 -0.0204 0.02403 NA FALSE FALSE FALSE
GCST006061 0.0321 0.5263 537409 outcome TRUE reported 11239_49_TMPRSS6_TMPS6
TMPRSS6 chr17 15132253 0.027105 1.378e-15 35346 TRUE reported
textfile 2 1 TRUE
6698 rs16964261 C T C T -0.5032 0.0201 0.05153 NA FALSE FALSE FALSE
GCST006061 0.0174 0.2487 537409 outcome TRUE reported 11239_49_TMPRSS6_TMPS6
TMPRSS6 chr17 28416775 0.019051 9.695e-154 35351 TRUE reported
textfile 2 1 TRUE
6699 rs190324353 G T G T 0.2395 -0.0495 0.01217 NA FALSE FALSE FALSE
GCST006061 0.0389 0.2033 537409 outcome TRUE reported 11239_49_TMPRSS6_TMPS6
TMPRSS6 chr17 15380062 0.037733 2.193e-10 35343 TRUE reported
textfile 2 1 TRUE
6700 rs2004584 T C T C 0.2037 -0.0051 0.14725 NA FALSE FALSE FALSE
GCST006061 0.0097 0.6024 537409 outcome TRUE reported 11239_49_TMPRSS6_TMPS6
TMPRSS6 chr17 27581275 0.011883 7.212e-66 35350 TRUE reported
textfile 2 1 TRUE
6701 rs4239214 A G A G 0.4948 0.0011 0.09756 NA FALSE FALSE FALSE
GCST006061 0.0113 0.9243 537409 outcome TRUE reported 11239_49_TMPRSS6_TMPS6
TMPRSS6 chr17 28000866 0.014013 1e-200 35351 TRUE reported textfile
2 1 TRUE
6702 rs78119120 A G A G 0.3426 -0.0172 0.01409 NA FALSE FALSE FALSE
GCST006061 0.0572 0.7635 537409 outcome TRUE reported 11239_49_TMPRSS6_TMPS6
TMPRSS6 chr17 30381853 0.035222 2.317e-22 35353 TRUE reported
textfile 2 1 TRUE
6703 rs2894038 A C A C -0.2465 0.0196 0.02944 NA FALSE FALSE FALSE
GCST006061 0.0193 0.3101 537409 outcome TRUE reported 12692_56_DOT1L_DOT1L
DOT1L chr6 25144779 0.023462 8.091e-26 35369 TRUE reported
textfile 2 1 TRUE
6704 rs6445035 A G A G 0.0554 0.0154 0.1904 NA FALSE FALSE FALSE
GCST006061 0.0089 0.08256 537409 outcome TRUE reported 12692_56_DOT1L_DOT1L
DOT1L chr3 165762312 0.009936 2.469e-08 35330 TRUE reported
textfile 2 1 TRUE

6705 rs9348630 A G A G 0.1532 -0.0091 0.08059 NA FALSE FALSE FALSE
GCST006061 0.0115 0.4263 537409 outcome TRUE reported 12692_56_DOT1L_DOT1L
DOT1L chr6 24071449 0.014359 1.416e-26 35367 TRUE reported
textfile 2 1 TRUE
6706 rs1706435 G A G A 0.0451 0.0117 0.36981 NA FALSE FALSE FALSE
GCST006061 0.0072 0.1047 537409 outcome TRUE reported
2839_2_TNFSF4_OX40_Ligand TNFSF4 chr3 165743688 0.008154 3.182e-08 35339
TRUE reported textfile 2 1 TRUE
6707 rs1707652 T C T C 0.0635 0.0081 0.3102 NA FALSE FALSE FALSE
GCST006061 0.0074 0.2701 537409 outcome TRUE reported 12683_156_DPYSL5_DPYL5
DPYSL5 chr3 165761011 0.008494 7.692e-14 35330 TRUE reported
textfile 2 1 TRUE
6708 rs1260326 C T C T -0.069 -0.0069 0.34132 NA FALSE FALSE FALSE
GCST006061 0.0073 0.3401 537409 outcome TRUE reported 6433_57_FAM20A_FA20A
FAM20A chr2 27508073 0.008666 1.69e-15 35373 TRUE reported
textfile 2 1 TRUE
6709 rs12941038 T C T C -0.1576 -0.0023 0.21873 NA FALSE FALSE FALSE
GCST006061 0.0084 0.7865 537409 outcome TRUE reported 6433_57_FAM20A_FA20A
FAM20A chr17 68513002 0.009862 1.74e-57 35368 TRUE reported
textfile 2 1 TRUE
6710 rs150231592 A G A G -0.188 -0.0342 0.03706 NA FALSE FALSE FALSE
GCST006061 0.0255 0.1805 537409 outcome TRUE reported 6433_57_FAM20A_FA20A
FAM20A chr7 259877 0.021735 5.174e-18 35323 TRUE reported textfile
2 1 TRUE
6711 rs2622935 G A G A -0.0846 0.0082 0.23255 NA FALSE FALSE FALSE
GCST006061 0.0086 0.3419 537409 outcome TRUE reported 6433_57_FAM20A_FA20A
FAM20A chr11 117140042 0.009683 2.397e-18 35378 TRUE reported
textfile 2 1 TRUE
6712 rs72654473 A C A C 0.0926 0.0046 0.08728 NA FALSE FALSE FALSE
GCST006061 0.0121 0.7062 537409 outcome TRUE reported 6433_57_FAM20A_FA20A
FAM20A chr19 44911142 0.014435 1.408e-10 35367 TRUE reported
textfile 2 1 TRUE
6713 rs929477 A G A G -0.2017 -0.0025 0.08687 NA FALSE FALSE FALSE
GCST006061 0.0125 0.8406 537409 outcome TRUE reported 6433_57_FAM20A_FA20A
FAM20A chr17 68659675 0.014526 7.742e-44 35368 TRUE reported
textfile 2 1 TRUE
6714 rs1354034 C T C T 0.066 0.0088 0.38119 NA FALSE FALSE FALSE
GCST006061 0.0073 0.2248 537409 outcome TRUE reported 14287_6_RAB5C_RAB5C
RAB5C chr3 56815721 0.008232 1.083e-15 35356 TRUE reported
textfile 2 1 TRUE
6715 rs1355538 G A G A -0.049 -0.0076 0.39847 NA FALSE FALSE FALSE
GCST006061 0.0072 0.2923 537409 outcome TRUE reported 14287_6_RAB5C_RAB5C
RAB5C chr3 165787389 0.008157 1.888e-09 35338 TRUE reported
textfile 2 1 TRUE
6716 rs185009887 A G A G -0.15 -0.0595 0.02519 NA FALSE FALSE FALSE
GCST006061 0.0496 0.2302 537409 outcome TRUE reported 14287_6_RAB5C_RAB5C
RAB5C chr17 5848332 0.025975 7.708e-09 35374 TRUE reported textfile
2 1 TRUE
6717 rs4980320 C T C T 0.0493 0.0041 0.46986 NA FALSE FALSE FALSE
GCST006061 0.0076 0.5858 537409 outcome TRUE reported 14287_6_RAB5C_RAB5C
RAB5C chr11 198986 0.008034 8.447e-10 35352 TRUE reported textfile
2 1 TRUE
6718 rs1355538 G A G A -0.06 -0.0076 0.39847 NA FALSE FALSE FALSE
GCST006061 0.0072 0.2923 537409 outcome TRUE reported 10425_3_B4GALT5_B4GT5
B4GALT5 chr3 165787389 0.008079 1.116e-13 35326 TRUE reported
textfile 2 1 TRUE
6719 rs13389363 G A G A 0.0718 -0.0027 0.11269 NA FALSE FALSE FALSE
GCST006061 0.0108 0.805 537409 outcome TRUE reported 12813_18_EHBP1_EHBP1
EHBP1 chr2 62499374 0.01258 1.147e-08 35341 TRUE reported textfile
2 1 TRUE
6720 rs2142760 G A G A -0.0447 -0.0042 0.4482 NA FALSE FALSE FALSE
GCST006061 0.0074 0.573 537409 outcome TRUE reported 12813_18_EHBP1_EHBP1
EHBP1 chr1 169608095 0.007946 1.847e-08 35338 TRUE reported
textfile 2 1 TRUE
6721 rs9332701 G A G A -0.2345 -0.0115 0.07189 NA FALSE FALSE FALSE
GCST006061 0.0196 0.5575 537409 outcome TRUE reported 12813_18_EHBP1_EHBP1

EHBP1 chr1 169515529 0.01512 3.024e-54 35355 TRUE reported textfile
2 1 TRUE
6722 rs4566588 T C T C 0.0532 0.0164 0.24788 NA FALSE FALSE FALSE
GCST006061 0.0081 0.04301 537409 outcome TRUE reported 12636_113_SMYD2_SMYD2
SMYD2 chr3 165744780 0.009081 4.674e-09 35330 TRUE reported
textfile 2 1 TRUE
6723 rs2686395 C T C T -0.0603 -0.0077 0.3101 NA FALSE FALSE FALSE
GCST006061 0.0074 0.2965 537409 outcome TRUE reported 10342_55_PIAS4_PIAS4
PIAS4 chr3 165767231 0.008504 1.335e-12 35327 TRUE reported
textfile 2 1 TRUE
6724 rs2293772 G A G A 0.0792 0.0057 0.40397 NA FALSE FALSE FALSE
GCST006061 0.0073 0.4346 537409 outcome TRUE reported
10737_96_SERPINB1_Serpin_B1 SERPINB1 chr6 2835597 0.008228 6.234e-22 35348
TRUE reported textfile 2 1 TRUE
6725 rs9971407 T C T C -0.0691 -0.0126 0.24858 NA FALSE FALSE FALSE
GCST006061 0.0086 0.1427 537409 outcome TRUE reported
10737_96_SERPINB1_Serpin_B1 SERPINB1 chr11 65130754 0.009334 1.329e-13
35351 TRUE reported textfile 2 1 TRUE
6726 rs117557731 G A G A 0.2732 -0.0064 0.04516 NA FALSE FALSE FALSE
GCST006061 0.0225 0.7749 537409 outcome TRUE reported 9172_69_MMP8_MMP_8
MMP8 chr11 102676761 0.019535 1.929e-44 35366 TRUE reported
textfile 2 1 TRUE
6727 rs1465528 T C T C -0.0603 0.0026 0.33979 NA FALSE FALSE FALSE
GCST006061 0.0075 0.7279 537409 outcome TRUE reported 9172_69_MMP8_MMP_8
MMP8 chr12 40369814 0.008596 2.298e-12 35351 TRUE reported
textfile 2 1 TRUE
6728 rs307736 A G A G -0.0666 -0.0088 0.39257 NA FALSE FALSE FALSE
GCST006061 0.0076 0.2448 537409 outcome TRUE reported 9172_69_MMP8_MMP_8
MMP8 chr8 141169703 0.008399 2.202e-15 35310 TRUE reported
textfile 2 1 TRUE
6729 rs331 A G A G -0.0511 0.0154 0.27558 NA FALSE FALSE FALSE GCST006061
0.008 0.05524 537409 outcome TRUE reported 9172_69_MMP8_MMP_8 MMP8 chr8
19962894 0.009118 2.094e-08 35368 TRUE reported textfile 2 1 TRUE
6730 rs35231465 A G A G -0.6843 0.0343 0.02267 NA FALSE FALSE FALSE
GCST006061 0.0264 0.1948 537409 outcome TRUE reported 9172_69_MMP8_MMP_8
MMP8 chr11 102713404 0.027199 1.127e-139 35365 TRUE reported
textfile 2 1 TRUE
6731 rs429358 C T C T 0.2367 0.005 0.17407 NA FALSE FALSE FALSE
GCST006061 0.0103 0.6319 537409 outcome TRUE reported 9172_69_MMP8_MMP_8
MMP8 chr19 44908684 0.010851 1.733e-105 35368 TRUE reported
textfile 2 1 TRUE
6732 rs4961341 A C A C -0.0498 0.0017 0.30893 NA FALSE FALSE FALSE
GCST006061 0.0078 0.8269 537409 outcome TRUE reported 9172_69_MMP8_MMP_8
MMP8 chr8 141225055 0.008874 1.998e-08 35308 TRUE reported
textfile 2 1 TRUE
6733 rs66595754 G A G A -0.1367 0.0171 0.06783 NA FALSE FALSE FALSE
GCST006061 0.0166 0.3043 537409 outcome TRUE reported 9172_69_MMP8_MMP_8
MMP8 chr7 49024312 0.016352 6.293e-17 35345 TRUE reported
textfile 2 1 TRUE
6734 rs6960617 G A G A -0.1295 -0.0064 0.36408 NA FALSE FALSE FALSE
GCST006061 0.0077 0.4093 537409 outcome TRUE reported 9172_69_MMP8_MMP_8
MMP8 chr7 48282854 0.008535 5.373e-52 35350 TRUE reported
textfile 2 1 TRUE
6735 rs78425657 G A G A -0.192 -0.0291 0.01605 NA FALSE FALSE FALSE
GCST006061 0.0269 0.2788 537409 outcome TRUE reported 9172_69_MMP8_MMP_8
MMP8 chr11 103861752 0.031955 1.872e-09 35364 TRUE reported
textfile 2 1 TRUE
6736 rs8075668 T C T C -0.0734 0.0133 0.44948 NA FALSE FALSE FALSE
GCST006061 0.0071 0.0614 537409 outcome TRUE reported 9172_69_MMP8_MMP_8
MMP8 chr17 39981370 0.008224 4.443e-19 35379 TRUE reported
textfile 2 1 TRUE
6737 rs1619994 G A G A 0.0814 0.0168 0.2807 NA FALSE FALSE FALSE
GCST006061 0.0078 0.03057 537409 outcome TRUE reported 2714_78_AIMP1_EMAP_2
AIMP1 chr3 165744047 0.008891 5.417e-20 35339 TRUE reported
textfile 2 1 TRUE
6738 rs71352239 T C T C 0.0688 -0.0041 0.30263 NA FALSE FALSE FALSE

GCST006061 0.0087 0.6372 537409 outcome TRUE reported 2714_78_AIMP1_EMAP_2
AIMP1 chr19 44926286 0.008718 2.986e-15 35368 TRUE reported
textfile 2 1 TRUE
6739 rs780093 C T C T -0.0523 -0.0074 0.34157 NA FALSE FALSE FALSE
GCST006061 0.0073 0.3116 537409 outcome TRUE reported 2714_78_AIMP1_EMAP_2
AIMP1 chr2 27519736 0.008427 5.431e-10 35373 TRUE reported
textfile 2 1 TRUE
6740 rs117265578 A G A G -0.0792 0.0127 0.08612 NA FALSE FALSE FALSE
GCST006061 0.0183 0.4854 537409 outcome TRUE reported 12705_9_HERC1_HERC1
HERC1 chr14 49522779 0.01397 1.434e-08 35353 TRUE reported textfile
2 1 TRUE
6741 rs8099840 C A C A -0.0519 -0.0055 0.3775 NA FALSE FALSE FALSE
GCST006061 0.0074 0.4583 537409 outcome TRUE reported 7737_76_ITGB6_ITB6
ITGB6 chr19 15470214 0.008013 9.375e-11 35378 TRUE reported
textfile 2 1 TRUE
6742 rs6445035 A G A G 0.0856 0.0154 0.1904 NA FALSE FALSE FALSE
GCST006061 0.0089 0.08256 537409 outcome TRUE reported 13441_30_DIXDC1_DIXC1
DIXDC1 chr3 165762312 0.009974 9.287e-18 35333 TRUE reported
textfile 2 1 TRUE
6743 rs8106813 G A G A 0.0448 -0.0037 0.46477 NA FALSE FALSE FALSE
GCST006061 0.0086 0.672 537409 outcome TRUE reported 13441_30_DIXDC1_DIXC1
DIXDC1 chr19 44928401 0.007924 1.572e-08 35362 TRUE reported
textfile 2 1 TRUE
6744 rs1355538 G A G A -0.0465 -0.0076 0.39847 NA FALSE FALSE FALSE
GCST006061 0.0072 0.2923 537409 outcome TRUE reported
2431_17_CTSG_Cathepsin_G CTSG chr3 165787389 0.00817 1.257e-08 35338
TRUE reported textfile 2 1 TRUE
6745 rs180977001 C A C A -0.089 -0.0093 0.07051 NA FALSE FALSE FALSE
GCST006061 0.0156 0.5527 537409 outcome TRUE reported
2431_17_CTSG_Cathepsin_G CTSG chr3 58332737 0.015777 1.69e-08 35357
TRUE reported textfile 2 1 TRUE
6746 rs6542680 T C T C -0.1083 -0.0012 0.18362 NA FALSE FALSE FALSE
GCST006061 0.0091 0.8935 537409 outcome TRUE reported
2431_17_CTSG_Cathepsin_G CTSG chr2 3592552 0.010351 1.276e-25 35332
TRUE reported textfile 2 1 TRUE
6747 rs12478841 G A G A 0.056 0.0049 0.21569 NA FALSE FALSE FALSE
GCST006061 0.008 0.5413 537409 outcome TRUE reported
16312_45_CDH6_Cadherin_6 CDH6 chr2 27588855 0.009499 3.737e-09 35373
TRUE reported textfile 2 1 TRUE
6748 rs2686395 C T C T -0.0768 -0.0077 0.3101 NA FALSE FALSE FALSE
GCST006061 0.0074 0.2965 537409 outcome TRUE reported
16312_45_CDH6_Cadherin_6 CDH6 chr3 165767231 0.008499 1.624e-19 35339
TRUE reported textfile 2 1 TRUE
6749 rs1706435 G A G A 0.0747 0.0117 0.36981 NA FALSE FALSE FALSE
GCST006061 0.0072 0.1047 537409 outcome TRUE reported 3005_5_PTPN1_PTP_1B
PTPN1 chr3 165743688 0.008301 2.277e-19 35339 TRUE reported
textfile 2 1 TRUE
6750 rs185009887 A G A G -0.1446 -0.0595 0.02519 NA FALSE FALSE FALSE
GCST006061 0.0496 0.2302 537409 outcome TRUE reported 3005_5_PTPN1_PTP_1B
PTPN1 chr17 5848332 0.026037 2.799e-08 35374 TRUE reported textfile
2 1 TRUE
6751 rs1355538 G A G A -0.0485 -0.0076 0.39847 NA FALSE FALSE FALSE
GCST006061 0.0072 0.2923 537409 outcome TRUE reported 9859_180_SAT1_SSAT_1
SAT1 chr3 165787389 0.007943 1.022e-09 35338 TRUE reported
textfile 2 1 TRUE
6752 rs1355538 G A G A -0.0443 -0.0076 0.39847 NA FALSE FALSE FALSE
GCST006061 0.0072 0.2923 537409 outcome TRUE reported 13513_174_PIAS3_PIAS3
PIAS3 chr3 165787389 0.008044 3.641e-08 35332 TRUE reported
textfile 2 1 TRUE
6753 rs116614403 T C T C 0.2215 0.0122 0.01638 NA FALSE FALSE FALSE
GCST006061 0.0353 0.7299 537409 outcome TRUE reported 10938_13_CD58_sLFA_3
CD58 chr1 118591986 0.031178 1.209e-12 35361 TRUE reported
textfile 2 1 TRUE
6754 rs1706435 G A G A 0.0744 0.0117 0.36981 NA FALSE FALSE FALSE
GCST006061 0.0072 0.1047 537409 outcome TRUE reported 10938_13_CD58_sLFA_3
CD58 chr3 165743688 0.00824 1.735e-19 35330 TRUE reported textfile

2 1 TRUE
6755 rs61789227 G A G A 0.6656 0.0129 0.02109 NA FALSE FALSE FALSE
GCST006061 0.0371 0.7288 537409 outcome TRUE reported 10938_13_CD58_sLFA_3
CD58 chr1 116537006 0.027448 6.699e-130 35344 TRUE reported
textfile 2 1 TRUE
6756 rs1354034 C T C T 0.0455 0.0088 0.38119 NA FALSE FALSE FALSE
GCST006061 0.0073 0.2248 537409 outcome TRUE reported 19338_3_LZIC_LZIC
LZIC chr3 56815721 0.008194 2.815e-08 35356 TRUE reported
textfile 2 1 TRUE
6757 rs1619994 G A G A 0.0577 0.0168 0.2807 NA FALSE FALSE FALSE
GCST006061 0.0078 0.03057 537409 outcome TRUE reported 19338_3_LZIC_LZIC
LZIC chr3 165744047 0.008867 7.671e-11 35339 TRUE reported
textfile 2 1 TRUE
6758 rs4265433 G A G A 0.1684 -0.0303 0.02238 NA FALSE FALSE FALSE
GCST006061 0.0329 0.3565 537409 outcome TRUE reported 19338_3_LZIC_LZIC
LZIC chr1 10065497 0.026857 3.604e-10 35391 TRUE reported
textfile 2 1 TRUE
6759 rs4665985 C A C A 0.0507 0.0065 0.23221 NA FALSE FALSE FALSE
GCST006061 0.0082 0.424 537409 outcome TRUE reported 13515_8_RPRD1A_RPR1A
RPRD1A chr2 27531011 0.009241 4.103e-08 35367 TRUE reported
textfile 2 1 TRUE
6760 rs9534312 C T C T 0.1108 0.0059 0.34021 NA FALSE FALSE FALSE
GCST006061 0.0076 0.4359 537409 outcome TRUE reported 13515_8_RPRD1A_RPR1A
RPRD1A chr13 46076084 0.008307 1.376e-40 35355 TRUE reported
textfile 2 1 TRUE
6761 rs112963922 T C T C 0.2718 0.0542 0.01119 NA FALSE FALSE FALSE
GCST006061 0.0399 0.1741 537409 outcome TRUE reported 15455_40_ADAM15_ADA15
ADAM15 chr14 94411531 0.036708 1.317e-13 35371 TRUE reported
textfile 2 1 TRUE
6762 rs1355538 G A G A -0.053 -0.0076 0.39847 NA FALSE FALSE FALSE
GCST006061 0.0072 0.2923 537409 outcome TRUE reported 15455_40_ADAM15_ADA15
ADAM15 chr3 165787389 0.007982 3.134e-11 35338 TRUE reported
textfile 2 1 TRUE
6763 rs16914574 C T C T 0.1149 -0.0098 0.04057 NA FALSE FALSE FALSE
GCST006061 0.0233 0.6742 537409 outcome TRUE reported 15455_40_ADAM15_ADA15
ADAM15 chr11 6511260 0.01996 8.592e-09 35380 TRUE reported textfile 2
1 TRUE
6764 rs1706435 G A G A 0.0593 0.0117 0.36981 NA FALSE FALSE FALSE
GCST006061 0.0072 0.1047 537409 outcome TRUE reported 5590_11_ZPLD1_ZPLD1
ZPLD1 chr3 165743688 0.008211 5.113e-13 35339 TRUE reported
textfile 2 1 TRUE
6765 rs1355538 G A G A -0.0458 -0.0076 0.39847 NA FALSE FALSE FALSE
GCST006061 0.0072 0.2923 537409 outcome TRUE reported 11657_86_SOCS7_SOCS7
SOCS7 chr3 165787389 0.007998 1.026e-08 35329 TRUE reported
textfile 2 1 TRUE
6766 rs6542680 T C T C -0.0575 -0.0012 0.18362 NA FALSE FALSE FALSE
GCST006061 0.0091 0.8935 537409 outcome TRUE reported 11657_86_SOCS7_SOCS7
SOCS7 chr2 3592552 0.010151 1.476e-08 35323 TRUE reported textfile
2 1 TRUE
6767 rs2106003 G A G A 0.0472 -0.0218 0.36634 NA FALSE FALSE FALSE
GCST006061 0.0124 0.07867 537409 outcome TRUE reported 4453_83_LRRTM3_LRRT3
LRRTM3 chr14 106418055 0.008323 1.419e-08 34966 TRUE reported
textfile 2 1 TRUE
6768 rs211161 G T G T 0.1749 -0.0176 0.01526 NA FALSE FALSE FALSE
GCST006061 0.0295 0.5516 537409 outcome TRUE reported 4453_83_LRRTM3_LRRT3
LRRTM3 chr6 96614917 0.03115 1.969e-08 35343 TRUE reported textfile
2 1 TRUE
6769 rs6542680 T C T C -0.0986 -0.0012 0.18362 NA FALSE FALSE FALSE
GCST006061 0.0091 0.8935 537409 outcome TRUE reported 4453_83_LRRTM3_LRRT3
LRRTM3 chr2 3592552 0.010189 3.785e-22 35332 TRUE reported textfile
2 1 TRUE
6770 rs1260326 C T C T -0.0699 -0.0069 0.34132 NA FALSE FALSE FALSE
GCST006061 0.0073 0.3401 537409 outcome TRUE reported 11457_53_GALE_GALE
GALE chr2 27508073 0.008574 3.57e-16 35364 TRUE reported
textfile 2 1 TRUE
6771 rs148023052 A G A G -0.0914 0.0018 0.10601 NA FALSE FALSE FALSE

GCST006061 0.0153 0.9053 537409 outcome TRUE reported 11457_53_GALE_GALE
GALE chr19 45195332 0.013201 4.397e-12 35361 TRUE reported
textfile 2 1 TRUE
6772 rs1706435 G A G A 0.0714 0.0117 0.36981 NA FALSE FALSE FALSE
GCST006061 0.0072 0.1047 537409 outcome TRUE reported 11457_53_GALE_GALE
GALE chr3 165743688 0.008366 1.405e-17 35330 TRUE reported
textfile 2 1 TRUE
6773 rs59007384 T G T G -0.0826 0.0033 0.21351 NA FALSE FALSE FALSE
GCST006061 0.009 0.7119 537409 outcome TRUE reported 11457_53_GALE_GALE
GALE chr19 44893408 0.009938 9.465e-17 35359 TRUE reported
textfile 2 1 TRUE
6774 rs704 A G A G 0.0486 -0.0155 0.49189 NA FALSE FALSE FALSE GCST006061
0.0072 0.03144 537409 outcome TRUE reported 11457_53_GALE_GALE GALE chr17
28367840 0.0081 1.974e-09 35351 TRUE reported textfile 2 1 TRUE
6775 rs8106813 G A G A 0.152 -0.0037 0.46477 NA FALSE FALSE FALSE
GCST006061 0.0086 0.672 537409 outcome TRUE reported 11457_53_GALE_GALE
GALE chr19 44928401 0.00814 8.186e-78 35359 TRUE reported textfile
2 1 TRUE
6776 rs113747960 A G A G -0.0676 -0.0034 0.12384 NA FALSE FALSE FALSE
GCST006061 0.0107 0.7473 537409 outcome TRUE reported 12960_9_GCK_HXK4 GCK
chr12 7074644 0.012336 4.254e-08 35380 TRUE reported textfile 2 1
TRUE
6777 rs12995461 C A C A 0.0655 0.0086 0.21643 NA FALSE FALSE FALSE
GCST006061 0.008 0.2842 537409 outcome TRUE reported 12960_9_GCK_HXK4 GCK
chr2 27555300 0.009641 1.094e-11 35364 TRUE reported textfile 2
1 TRUE
6778 rs17732416 T C T C -0.1081 -0.0036 0.04293 NA FALSE FALSE FALSE
GCST006061 0.0161 0.8254 537409 outcome TRUE reported 12960_9_GCK_HXK4 GCK
chr9 21638408 0.019791 4.707e-08 35344 TRUE reported textfile 2
1 TRUE
6779 rs704 A G A G 0.0461 -0.0155 0.49189 NA FALSE FALSE FALSE GCST006061
0.0072 0.03144 537409 outcome TRUE reported 12960_9_GCK_HXK4 GCK chr17
28367840 0.008012 8.724e-09 35351 TRUE reported textfile 2 1 TRUE
6780 rs1706435 G A G A 0.0618 0.0117 0.36981 NA FALSE FALSE FALSE
GCST006061 0.0072 0.1047 537409 outcome TRUE reported 19123_6_NFKBID_IKBD
NFKBID chr3 165743688 0.008311 1.041e-13 35339 TRUE reported
textfile 2 1 TRUE
6781 rs2239165 C T C T 0.0578 -0.0132 0.28884 NA FALSE FALSE FALSE
GCST006061 0.0078 0.09017 537409 outcome TRUE reported 19123_6_NFKBID_IKBD
NFKBID chr12 6914702 0.008875 7.378e-11 35390 TRUE reported textfile
2 1 TRUE
6782 rs6445035 A G A G 0.0591 0.0154 0.1904 NA FALSE FALSE FALSE
GCST006061 0.0089 0.08256 537409 outcome TRUE reported 9923_6_SOS1_SOS1
SOS1 chr3 165762312 0.009996 3.369e-09 35339 TRUE reported
textfile 2 1 TRUE
6783 rs6542680 T C T C -0.0644 -0.0012 0.18362 NA FALSE FALSE FALSE
GCST006061 0.0091 0.8935 537409 outcome TRUE reported 7782_34_EDDM3B_EP3B
EDDM3B chr2 3592552 0.010064 1.565e-10 35332 TRUE reported textfile
2 1 TRUE
6784 rs11906988 G T G T -0.2123 -0.0389 0.01085 NA FALSE FALSE FALSE
GCST006061 0.0283 0.1697 537409 outcome TRUE reported 13961_18_KIF3A_KIF3A
KIF3A chr20 38383368 0.038407 3.246e-08 35372 TRUE reported
textfile 2 1 TRUE
6785 rs12478841 G A G A 0.0635 0.0049 0.21569 NA FALSE FALSE FALSE
GCST006061 0.008 0.5413 537409 outcome TRUE reported 13961_18_KIF3A_KIF3A
KIF3A chr2 27588855 0.009763 7.807e-11 35373 TRUE reported
textfile 2 1 TRUE
6786 rs17732416 T C T C -0.1174 -0.0036 0.04293 NA FALSE FALSE FALSE
GCST006061 0.0161 0.8254 537409 outcome TRUE reported 13961_18_KIF3A_KIF3A
KIF3A chr9 21638408 0.020014 4.465e-09 35353 TRUE reported
textfile 2 1 TRUE
6787 rs2232613 T C T C -0.1849 0.0151 0.0989 NA FALSE FALSE FALSE
GCST006061 0.0141 0.2834 537409 outcome TRUE reported 13961_18_KIF3A_KIF3A
KIF3A chr20 38369011 0.013345 1.175e-43 35375 TRUE reported
textfile 2 1 TRUE
6788 rs3917538 A G A G 0.0632 -0.0137 0.21967 NA FALSE FALSE FALSE

GCST006061 0.0082 0.09535 537409 outcome TRUE reported 13961_18_KIF3A_KIF3A
KIF3A chr7 95308581 0.009785 1.053e-10 35349 TRUE reported
textfile 2 1 TRUE
6789 rs6445035 A G A G 0.1406 0.0154 0.1904 NA FALSE FALSE FALSE
GCST006061 0.0089 0.08256 537409 outcome TRUE reported 13961_18_KIF3A_KIF3A
KIF3A chr3 165762312 0.01028 1.395e-42 35339 TRUE reported textfile
2 1 TRUE
6790 rs111278137 A G A G 0.3717 -0.0296 0.01948 NA FALSE FALSE FALSE
GCST006061 0.0311 0.3413 537409 outcome TRUE reported 14318_1_VPS29_VPS29
VPS29 chr19 44711810 0.033168 3.792e-29 35369 TRUE reported
textfile 2 1 TRUE
6791 rs118004808 T C T C 0.2468 -5.00E-04 0.03208 NA FALSE FALSE FALSE
GCST006061 0.0399 0.9892 537409 outcome TRUE reported 14318_1_VPS29_VPS29
VPS29 chr19 44936241 0.025511 3.876e-22 35368 TRUE reported
textfile 2 1 TRUE
6792 rs118170342 C T C T -0.271 0.0162 0.04525 NA FALSE FALSE FALSE
GCST006061 0.0216 0.453 537409 outcome TRUE reported 14318_1_VPS29_VPS29
VPS29 chr19 44898611 0.022031 8.933e-35 35368 TRUE reported
textfile 2 1 TRUE
6793 rs12691088 A G A G -0.3843 -0.0225 0.05447 NA FALSE FALSE FALSE
GCST006061 0.0301 0.4558 537409 outcome TRUE reported 14318_1_VPS29_VPS29
VPS29 chr19 44915229 0.02005 6.986e-82 35368 TRUE reported textfile
2 1 TRUE
6794 rs1706435 G A G A 0.0698 0.0117 0.36981 NA FALSE FALSE FALSE
GCST006061 0.0072 0.1047 537409 outcome TRUE reported 14318_1_VPS29_VPS29
VPS29 chr3 165743688 0.008936 5.652e-15 35339 TRUE reported
textfile 2 1 TRUE
6795 rs4251945 A G A G 0.3179 0.0223 0.02184 NA FALSE FALSE FALSE
GCST006061 0.0424 0.5993 537409 outcome TRUE reported 14318_1_VPS29_VPS29
VPS29 chr19 43656997 0.031049 1.331e-24 35368 TRUE reported
textfile 2 1 TRUE
6796 rs62117160 A G A G 0.9679 -0.0012 0.03283 NA FALSE FALSE FALSE
GCST006061 0.0206 0.9534 537409 outcome TRUE reported 14318_1_VPS29_VPS29
VPS29 chr19 44728895 0.019038 1e-200 35368 TRUE reported textfile
2 1 TRUE
6797 rs7254892 A G A G 1.4862 -0.013 0.01741 NA FALSE FALSE FALSE
GCST006061 0.0198 0.5114 537409 outcome TRUE reported 14318_1_VPS29_VPS29
VPS29 chr19 44886339 0.025113 1e-200 35368 TRUE reported textfile
2 1 TRUE
6798 rs111696008 A G A G -0.1752 -0.0259 0.02443 NA FALSE FALSE FALSE
GCST006061 0.0401 0.519 537409 outcome TRUE reported 5345_51_CKAP2_CKAP2
CKAP2 chr2 216622986 0.027325 1.438e-10 35359 TRUE reported
textfile 2 1 TRUE
6799 rs12026141 T C T C -0.0491 0.0094 0.31246 NA FALSE FALSE FALSE
GCST006061 0.0076 0.2137 537409 outcome TRUE reported 5345_51_CKAP2_CKAP2
CKAP2 chr1 201980465 0.008939 3.95e-08 35357 TRUE reported
textfile 2 1 TRUE
6800 rs144202860 A G A G -0.2101 -0.0629 0.01274 NA FALSE FALSE FALSE
GCST006061 0.0538 0.2423 537409 outcome TRUE reported 5345_51_CKAP2_CKAP2
CKAP2 chr2 216625740 0.036579 9.265e-09 35359 TRUE reported
textfile 2 1 TRUE
6801 rs1618069 G A G A 0.0531 -0.02 0.24647 NA FALSE FALSE FALSE
GCST006061 0.0083 0.01621 537409 outcome TRUE reported 5345_51_CKAP2_CKAP2
CKAP2 chr3 136308303 0.009598 3.163e-08 35361 TRUE reported
textfile 2 1 TRUE
6802 rs2057681 G A G A 0.1239 -0.0136 0.28541 NA FALSE FALSE FALSE
GCST006061 0.0078 0.07965 537409 outcome TRUE reported 5345_51_CKAP2_CKAP2
CKAP2 chr7 95308945 0.009134 6.475e-42 35349 TRUE reported
textfile 2 1 TRUE
6803 rs41290120 A G A G 0.1195 -0.008 0.04078 NA FALSE FALSE FALSE
GCST006061 0.0191 0.6753 537409 outcome TRUE reported 5345_51_CKAP2_CKAP2
CKAP2 chr19 44879418 0.020983 1.234e-08 35367 TRUE reported
textfile 2 1 TRUE
6804 rs4666000 C T C T 0.0668 0.007 0.22594 NA FALSE FALSE FALSE
GCST006061 0.0078 0.3727 537409 outcome TRUE reported 5345_51_CKAP2_CKAP2
CKAP2 chr2 27616502 0.009775 8.266e-12 35373 TRUE reported

textfile 2 1 TRUE
6805 rs6445035 A G A G 0.1662 0.0154 0.1904 NA FALSE FALSE FALSE
GCST006061 0.0089 0.08256 537409 outcome TRUE reported 5345_51_CKAP2_CKAP2
CKAP2 chr3 165762312 0.010428 3.429e-57 35339 TRUE reported
textfile 2 1 TRUE
6806 rs73169103 T C T C 0.1338 0.0178 0.03563 NA FALSE FALSE FALSE
GCST006061 0.0249 0.4756 537409 outcome TRUE reported 5345_51_CKAP2_CKAP2
CKAP2 chr3 167190675 0.022169 1.586e-09 35354 TRUE reported
textfile 2 1 TRUE
6807 rs78810414 T C T C -0.0914 0.0257 0.08751 NA FALSE FALSE FALSE
GCST006061 0.012 0.03151 537409 outcome TRUE reported 5345_51_CKAP2_CKAP2
CKAP2 chr8 20013602 0.01447 2.672e-10 35368 TRUE reported textfile
2 1 TRUE
6808 rs8106813 G A G A 0.0797 -0.0037 0.46477 NA FALSE FALSE FALSE
GCST006061 0.0086 0.672 537409 outcome TRUE reported 5345_51_CKAP2_CKAP2
CKAP2 chr19 44928401 0.00828 6.203e-22 35368 TRUE reported textfile
2 1 TRUE
6809 rs16559 C T C T -0.0517 0.0082 0.22433 NA FALSE FALSE FALSE GCST006061
0.0086 0.3417 537409 outcome TRUE reported 9220_7_EPHB3_EPHB3 EPHB3 chr17
33038811 0.009365 3.384e-08 35372 TRUE reported textfile 2 1 TRUE
6810 rs1707652 T C T C 0.0791 0.0081 0.3102 NA FALSE FALSE FALSE
GCST006061 0.0074 0.2701 537409 outcome TRUE reported 6578_29_TNMD_TNMD
TNMD chr3 165761011 0.008552 2.256e-20 35339 TRUE reported
textfile 2 1 TRUE
6811 rs1123176 C T C T -0.1865 -0.0109 0.15665 NA FALSE FALSE FALSE
GCST006061 0.0093 0.2436 537409 outcome TRUE reported 8786_6_FAM171B_F171B
FAM171B chr2 186742744 0.010802 8.649e-67 35364 TRUE reported
textfile 2 1 TRUE
6812 rs12216891 T C T C 0.1018 0.0175 0.05689 NA FALSE FALSE FALSE
GCST006061 0.0134 0.1937 537409 outcome TRUE reported 8786_6_FAM171B_F171B
FAM171B chr9 133251979 0.01703 2.263e-09 35370 TRUE reported textfile
2 1 TRUE
6813 rs4917 C T C T 0.0709 -6.00E-04 0.36516 NA FALSE FALSE FALSE
GCST006061 0.0075 0.9407 537409 outcome TRUE reported 8786_6_FAM171B_F171B
FAM171B chr3 186619924 0.008176 4.253e-18 35367 TRUE reported
textfile 2 1 TRUE
6814 rs1354034 C T C T 0.054 0.0088 0.38119 NA FALSE FALSE FALSE
GCST006061 0.0073 0.2248 537409 outcome TRUE reported 16883_57_RAB4A_RAB4A
RAB4A chr3 56815721 0.008193 4.365e-11 35356 TRUE reported
textfile 2 1 TRUE
6815 rs12622141 A G A G -0.061 0.0059 0.21384 NA FALSE FALSE FALSE
GCST006061 0.0089 0.5083 537409 outcome TRUE reported 5227_60_PDK1_PDK1
PDK1 chr2 172865958 0.009699 3.192e-10 35363 TRUE reported
textfile 2 1 TRUE
6816 rs1706435 G A G A 0.0542 0.0117 0.36981 NA FALSE FALSE FALSE
GCST006061 0.0072 0.1047 537409 outcome TRUE reported 5227_60_PDK1_PDK1
PDK1 chr3 165743688 0.008215 4.176e-11 35339 TRUE reported
textfile 2 1 TRUE
6817 rs35783685 T C T C -0.0557 -0.0035 0.23407 NA FALSE FALSE FALSE
GCST006061 0.0081 0.6661 537409 outcome TRUE reported 5227_60_PDK1_PDK1
PDK1 chr2 172458199 0.009363 2.694e-09 35366 TRUE reported
textfile 2 1 TRUE
6818 rs704 A G A G 0.055 -0.0155 0.49189 NA FALSE FALSE FALSE GCST006061
0.0072 0.03144 537409 outcome TRUE reported 5227_60_PDK1_PDK1 PDK1 chr17
28367840 0.007943 4.382e-12 35360 TRUE reported textfile 2 1 TRUE
6819 rs1354034 C T C T 0.0647 0.0088 0.38119 NA FALSE FALSE FALSE
GCST006061 0.0073 0.2248 537409 outcome TRUE reported 15331_47_RBBP4_RBBP4
RBBP4 chr3 56815721 0.008142 1.925e-15 35356 TRUE reported
textfile 2 1 TRUE
6820 rs4639017 C T C T -0.0446 -0.0061 0.39283 NA FALSE FALSE FALSE
GCST006061 0.0073 0.4022 537409 outcome TRUE reported 15331_47_RBBP4_RBBP4
RBBP4 chr3 165833296 0.008081 3.406e-08 35339 TRUE reported
textfile 2 1 TRUE
6821 rs6542680 T C T C -0.0623 -0.0012 0.18362 NA FALSE FALSE FALSE
GCST006061 0.0091 0.8935 537409 outcome TRUE reported 17776_15_PECR_PECR
PECR chr2 3592552 0.010286 1.388e-09 35332 TRUE reported textfile

2 1 TRUE
6822 rs73072196 C T C T -0.1544 0.0106 0.10387 NA FALSE FALSE FALSE
GCST006061 0.0123 0.3889 537409 outcome TRUE reported 17776_15_PECR_PECR
PECR chr2 216008671 0.013046 2.568e-32 35353 TRUE reported
textfile 2 1 TRUE
6823 rs1355538 G A G A -0.0613 -0.0076 0.39847 NA FALSE FALSE FALSE
GCST006061 0.0072 0.2923 537409 outcome TRUE reported 12534_10_CALCOCO2_CACO2
CALCOCO2 chr3 165787389 0.008171 6.295e-14 35329 TRUE reported
textfile 2 1 TRUE
6824 rs4646787 G A G A 0.1267 0.015 0.33581 NA FALSE FALSE FALSE
GCST006061 0.0078 0.05343 537409 outcome TRUE reported
11480_1_ALDH3A1_Aldehyde_dehydrogenase__class_3 ALDH3A1 chr17 19745936 0.008277
6.817e-53 35353 TRUE reported textfile 2 1 TRUE
6825 rs1706435 G A G A 0.0478 0.0117 0.36981 NA FALSE FALSE FALSE
GCST006061 0.0072 0.1047 537409 outcome TRUE reported 15402_2_ID1_ID_1 ID1
chr3 165743688 0.00811 3.768e-09 35339 TRUE reported textfile 2 1
TRUE
6826 rs111696008 A G A G -0.1617 -0.0259 0.02443 NA FALSE FALSE FALSE
GCST006061 0.0401 0.519 537409 outcome TRUE reported 19496_1_NPL_NPL NPL
chr2 216622986 0.026787 1.574e-09 35359 TRUE reported textfile 2
1 TRUE
6827 rs12138972 T C T C 0.1376 -0.0343 0.03354 NA FALSE FALSE FALSE
GCST006061 0.0239 0.1525 537409 outcome TRUE reported 19496_1_NPL_NPL NPL
chr1 182904296 0.022482 9.339e-10 35359 TRUE reported textfile 2
1 TRUE
6828 rs74646646 C T C T 0.4351 -0.0075 0.0233 NA FALSE FALSE FALSE
GCST006061 0.0208 0.7191 537409 outcome TRUE reported 19496_1_NPL_NPL NPL
chr1 182909529 0.026566 2.731e-60 35359 TRUE reported textfile 2
1 TRUE
6829 rs6445035 A G A G 0.1096 0.0154 0.1904 NA FALSE FALSE FALSE
GCST006061 0.0089 0.08256 537409 outcome TRUE reported 18205_123_MAGEA6_MAGE_6
MAGEA6 chr3 165762312 0.010143 3.243e-27 35339 TRUE reported
textfile 2 1 TRUE
6830 rs704 A G A G 0.0631 -0.0155 0.49189 NA FALSE FALSE FALSE GCST006061
0.0072 0.03144 537409 outcome TRUE reported 18205_123_MAGEA6_MAGE_6 MAGEA6
chr17 28367840 0.007991 2.872e-15 35360 TRUE reported textfile 2
1 TRUE
6831 rs1354034 C T C T 0.0474 0.0088 0.38119 NA FALSE FALSE FALSE
GCST006061 0.0073 0.2248 537409 outcome TRUE reported 9836_20_DCK_DCK DCK
chr3 56815721 0.008128 5.491e-09 35356 TRUE reported textfile 2
1 TRUE
6832 rs11569415 A G A G -0.0751 0.0011 0.24276 NA FALSE FALSE FALSE
GCST006061 0.0097 0.9129 537409 outcome TRUE reported 17771_35_CRCP_RPC9
CRCP chr19 6716268 0.009249 4.662e-16 35372 TRUE reported textfile
2 1 TRUE
6833 rs1707652 T C T C 0.0877 0.0081 0.3102 NA FALSE FALSE FALSE
GCST006061 0.0074 0.2701 537409 outcome TRUE reported 17771_35_CRCP_RPC9
CRCP chr3 165761011 0.008584 1.674e-24 35339 TRUE reported
textfile 2 1 TRUE
6834 rs704 A G A G 0.0676 -0.0155 0.49189 NA FALSE FALSE FALSE GCST006061
0.0072 0.03144 537409 outcome TRUE reported 17771_35_CRCP_RPC9 CRCP chr17
28367840 0.007975 2.321e-17 35360 TRUE reported textfile 2 1 TRUE
6835 rs1707652 T C T C 0.0614 0.0081 0.3102 NA FALSE FALSE FALSE
GCST006061 0.0074 0.2701 537409 outcome TRUE reported 12581_39_IMPA2_IMPA2
IMPA2 chr3 165761011 0.00844 3.47e-13 35330 TRUE reported textfile
2 1 TRUE
6836 rs2237582 G A G A 0.107 -0.0128 0.28548 NA FALSE FALSE FALSE
GCST006061 0.0078 0.0999 537409 outcome TRUE reported 12490_92_LAMTOR3_LTOR3
LAMTOR3 chr7 95304888 0.008848 1.157e-33 35340 TRUE reported
textfile 2 1 TRUE
6837 rs7620024 A G A G 0.2179 0.001 0.23792 NA FALSE FALSE FALSE
GCST006061 0.0084 0.9069 537409 outcome TRUE reported 12490_92_LAMTOR3_LTOR3
LAMTOR3 chr3 49414258 0.009342 2.499e-120 35335 TRUE reported
textfile 2 1 TRUE
6838 rs1355538 G A G A -0.045 -0.0076 0.39847 NA FALSE FALSE FALSE
GCST006061 0.0072 0.2923 537409 outcome TRUE reported 7995_16_VWC2L_VWC2L

VWC2L chr3 165787389 0.007995 1.816e-08 35338 TRUE reported
textfile 2 1 TRUE
6839 rs1260326 C T C T -0.0529 -0.0069 0.34132 NA FALSE FALSE FALSE
GCST006061 0.0073 0.3401 537409 outcome TRUE reported 8106_15_SSR1_SSRA
SSR1 chr2 27508073 0.008561 6.452e-10 35373 TRUE reported
textfile 2 1 TRUE
6840 rs1707652 T C T C 0.0896 0.0081 0.3102 NA FALSE FALSE FALSE
GCST006061 0.0074 0.2701 537409 outcome TRUE reported 7069_9_TSNARE1_TSNA1
TSNARE1 chr3 165761011 0.00858 1.587e-25 35339 TRUE reported textfile
2 1 TRUE
6841 rs704 A G A G 0.0584 -0.0155 0.49189 NA FALSE FALSE FALSE GCST006061
0.0072 0.03144 537409 outcome TRUE reported 7069_9_TSNARE1_TSNA1 TSNARE1
chr17 28367840 0.007979 2.492e-13 35360 TRUE reported textfile 2
1 TRUE
6842 rs7625980 C T C T 0.0843 0.0011 0.23642 NA FALSE FALSE FALSE
GCST006061 0.0083 0.895 537409 outcome TRUE reported 3520_58_TGFB3_TGF_b3
TGFB3 chr3 186675758 0.009283 1.075e-19 35368 TRUE reported
textfile 2 1 TRUE
6843 rs9902706 T C T C -0.1104 -0.05 0.05353 NA FALSE FALSE FALSE
GCST006061 0.0152 0.0009747 537409 outcome TRUE reported
3520_58_TGFB3_TGF_b3 TGFB3 chr17 66190870 0.017896 6.879e-10 35370
TRUE reported textfile 2 1 TRUE
6844 rs1707652 T C T C 0.064 0.0081 0.3102 NA FALSE FALSE FALSE
GCST006061 0.0074 0.2701 537409 outcome TRUE reported 6357_83_CELA3B_ELA3B
CELA3B chr3 165761011 0.008494 4.889e-14 35339 TRUE reported
textfile 2 1 TRUE
6845 rs11536949 T G T G -0.0763 0.0148 0.1003 NA FALSE FALSE FALSE
GCST006061 0.0141 0.2939 537409 outcome TRUE reported 5936_53_TNF_TNF_a TNF
chr20 38357231 0.012946 3.779e-09 35375 TRUE reported textfile 2
1 TRUE
6846 rs12085435 A G A G 0.1304 -0.0136 0.05177 NA FALSE FALSE FALSE
GCST006061 0.017 0.4252 537409 outcome TRUE reported 8932_1_ENTPD6_ENTP6
ENTPD6 chr1 56949637 0.01856 2.126e-12 35371 TRUE reported textfile
2 1 TRUE
6847 rs1706435 G A G A 0.0653 0.0117 0.36981 NA FALSE FALSE FALSE
GCST006061 0.0072 0.1047 537409 outcome TRUE reported 8932_1_ENTPD6_ENTP6
ENTPD6 chr3 165743688 0.008323 4.313e-15 35339 TRUE reported
textfile 2 1 TRUE
6848 rs6050446 G A G A 0.3418 -0.0245 0.03163 NA FALSE FALSE FALSE
GCST006061 0.024 0.3082 537409 outcome TRUE reported 8932_1_ENTPD6_ENTP6
ENTPD6 chr20 25214873 0.023645 2.305e-47 35365 TRUE reported
textfile 2 1 TRUE
6849 rs3184504 C T C T -0.052 0.0074 0.37862 NA FALSE FALSE FALSE
GCST006061 0.0075 0.3235 537409 outcome TRUE reported 6221_1_ADPGK_ADPGK
ADPGK chr12 111446804 0.008484 8.825e-10 35371 TRUE reported
textfile 2 1 TRUE
6850 rs11216161 C T C T -0.0992 0.003 0.20203 NA FALSE FALSE FALSE
GCST006061 0.0093 0.7434 537409 outcome TRUE reported
2558_51_POMC_b_Endorphin POMC chr11 116850485 0.009871 9.21e-24 35377
TRUE reported textfile 2 1 TRUE
6851 rs12721028 G A G A -0.1448 -0.0033 0.12278 NA FALSE FALSE FALSE
GCST006061 0.0095 0.7264 537409 outcome TRUE reported
2558_51_POMC_b_Endorphin POMC chr11 116834874 0.012048 2.83e-33 35377
TRUE reported textfile 2 1 TRUE
6852 rs7412 T C T C 0.1533 -0.0027 0.05941 NA FALSE FALSE FALSE GCST006061
0.0138 0.8459 537409 outcome TRUE reported 2558_51_POMC_b_Endorphin POMC
chr19 44908822 0.016738 5.236e-20 35367 TRUE reported textfile 2
1 TRUE
6853 rs75275400 C T C T 0.136 -0.0015 0.03947 NA FALSE FALSE FALSE
GCST006061 0.0256 0.9533 537409 outcome TRUE reported
2558_51_POMC_b_Endorphin POMC chr11 117658961 0.020388 2.549e-11 35376
TRUE reported textfile 2 1 TRUE
6854 rs1355538 G A G A -0.0688 -0.0076 0.39847 NA FALSE FALSE FALSE
GCST006061 0.0072 0.2923 537409 outcome TRUE reported 13067_5_PRKG1_KGP1B
PRKG1 chr3 165787389 0.008191 4.466e-17 35329 TRUE reported
textfile 2 1 TRUE

6855 rs2057681 G A G A 0.0996 -0.0136 0.28541 NA FALSE FALSE FALSE GCST006061 0.0078 0.07965 537409 outcome TRUE reported 13067_5_PRKG1_KGP1B PRKG1 chr7 95308945 0.008926 6.486e-29 35340 TRUE reported textfile 2 1 TRUE
6856 rs4666000 C T C T 0.0609 0.007 0.22594 NA FALSE FALSE FALSE GCST006061 0.0078 0.3727 537409 outcome TRUE reported 13067_5_PRKG1_KGP1B PRKG1 chr2 27616502 0.009547 1.779e-10 35364 TRUE reported textfile 2 1 TRUE
6857 rs4795734 A G A G -0.0517 0.012 0.23628 NA FALSE FALSE FALSE GCST006061 0.0084 0.1516 537409 outcome TRUE reported 10575_31_PUF60_PUF60 PUF60 chr17 33030407 0.009316 2.865e-08 35360 TRUE reported textfile 2 1 TRUE
6858 rs4801902 T C T C 0.098 -0.0168 0.0647 NA FALSE FALSE FALSE GCST006061 0.0221 0.4455 537409 outcome TRUE reported 10575_31_PUF60_PUF60 PUF60 chr19 51936045 0.016013 9.353e-10 35372 TRUE reported textfile 2 1 TRUE
6859 rs1619994 G A G A 0.0518 0.0168 0.2807 NA FALSE FALSE FALSE GCST006061 0.0078 0.03057 537409 outcome TRUE reported 13481_24_TCEA2_TCEA2 TCEA2 chr3 165744047 0.0089 5.883e-09 35333 TRUE reported textfile 2 1 TRUE
6860 rs6011275 G T G T -0.1453 0.002 0.32709 NA FALSE FALSE FALSE GCST006061 0.0077 0.8004 537409 outcome TRUE reported 13481_24_TCEA2_TCEA2 TCEA2 chr20 64068875 0.008573 1.986e-64 34864 TRUE reported textfile 2 1 TRUE
6861 rs12478841 G A G A 0.0535 0.0049 0.21569 NA FALSE FALSE FALSE GCST006061 0.008 0.5413 537409 outcome TRUE reported 12616_45_NRBP1_NRBP NRBP1 chr2 27588855 0.009683 3.288e-08 35364 TRUE reported textfile 2 1 TRUE
6862 rs1707652 T C T C 0.0954 0.0081 0.3102 NA FALSE FALSE FALSE GCST006061 0.0074 0.2701 537409 outcome TRUE reported 12616_45_NRBP1_NRBP NRBP1 chr3 165761011 0.008656 3.011e-28 35330 TRUE reported textfile 2 1 TRUE
6863 rs429358 C T C T 0.0835 0.005 0.17407 NA FALSE FALSE FALSE GCST006061 0.0103 0.6319 537409 outcome TRUE reported 12616_45_NRBP1_NRBP NRBP1 chr19 44908684 0.010702 6.081e-15 35359 TRUE reported textfile 2 1 TRUE
6864 rs9971407 T C T C -0.0696 -0.0126 0.24858 NA FALSE FALSE FALSE GCST006061 0.0086 0.1427 537409 outcome TRUE reported 12616_45_NRBP1_NRBP NRBP1 chr11 65130754 0.009303 7.357e-14 35354 TRUE reported textfile 2 1 TRUE
6865 rs1707652 T C T C 0.1113 0.0081 0.3102 NA FALSE FALSE FALSE GCST006061 0.0074 0.2701 537409 outcome TRUE reported 8535_102_DMKN_Dermokine DMKN chr3 165761011 0.008519 5.176e-39 35339 TRUE reported textfile 2 1 TRUE
6866 rs12137007 G A G A -0.2124 0.0073 0.17654 NA FALSE FALSE FALSE GCST006061 0.0089 0.4135 537409 outcome TRUE reported 5128_53_SLAMF6_SLAF6 SLAMF6 chr1 160493616 0.010478 2.341e-91 35350 TRUE reported textfile 2 1 TRUE
6867 rs12945886 G A G A 0.0582 0 0.24063 NA FALSE FALSE FALSE GCST006061 0.0094 0.9973 537409 outcome TRUE reported 5128_53_SLAMF6_SLAF6 SLAMF6 chr17 7169695 0.009352 4.872e-10 35370 TRUE reported textfile 2 1 TRUE
6868 rs3184504 C T C T -0.0625 0.0074 0.37862 NA FALSE FALSE FALSE GCST006061 0.0075 0.3235 537409 outcome TRUE reported 5128_53_SLAMF6_SLAF6 SLAMF6 chr12 111446804 0.008231 3.113e-14 35371 TRUE reported textfile 2 1 TRUE
6869 rs6542680 T C T C -0.0586 -0.0012 0.18362 NA FALSE FALSE FALSE GCST006061 0.0091 0.8935 537409 outcome TRUE reported 11336_9_FANCF_FANCF FANCF chr2 3592552 0.010086 6.241e-09 35323 TRUE reported textfile 2 1 TRUE
6870 rs112692814 A G A G -0.146 -0.0195 0.03232 NA FALSE FALSE FALSE GCST006061 0.0282 0.4885 537409 outcome TRUE reported 17138_8_GSTA1_GST_A1_1 GSTA1 chr6 53221120 0.022234 5.153e-11 35369 TRUE reported textfile 2 1 TRUE
6871 rs1553331 A G A G 0.0581 -0.0064 0.34236 NA FALSE FALSE FALSE GCST006061 0.0075 0.3953 537409 outcome TRUE reported 17138_8_GSTA1_GST_A1_1

GSTA1 chr6 55416335 0.008385 4.245e-12 35350 TRUE reported
textfile 2 1 TRUE
6872 rs1706435 G A G A 0.0474 0.0117 0.36981 NA FALSE FALSE FALSE
GCST006061 0.0072 0.1047 537409 outcome TRUE reported 17138_8_GSTA1_GST_A1_1
GSTA1 chr3 165743688 0.008241 8.839e-09 35339 TRUE reported
textfile 2 1 TRUE
6873 rs2144693 C T C T 0.2501 0.0049 0.36859 NA FALSE FALSE FALSE
GCST006061 0.0077 0.5235 537409 outcome TRUE reported 17138_8_GSTA1_GST_A1_1
GSTA1 chr6 52739150 0.008231 1e-200 35365 TRUE reported textfile
2 1 TRUE
6874 rs3747207 A G A G 0.0644 0.0153 0.22927 NA FALSE FALSE FALSE
GCST006061 0.0085 0.07308 537409 outcome TRUE reported 17138_8_GSTA1_GST_A1_1
GSTA1 chr22 43928975 0.009486 1.128e-11 35372 TRUE reported
textfile 2 1 TRUE
6875 rs4712001 C A C A -0.0678 -0.0132 0.32352 NA FALSE FALSE FALSE
GCST006061 0.0077 0.08596 537409 outcome TRUE reported 17138_8_GSTA1_GST_A1_1
GSTA1 chr6 52357141 0.0086 3.177e-15 35367 TRUE reported textfile
2 1 TRUE
6876 rs7752387 A G A G -0.1693 -0.0211 0.04539 NA FALSE FALSE FALSE
GCST006061 0.0276 0.4439 537409 outcome TRUE reported 17138_8_GSTA1_GST_A1_1
GSTA1 chr6 53039089 0.0196 5.729e-18 35368 TRUE reported textfile
2 1 TRUE
6877 rs3782677 T G T G 0.0475 -0.0042 0.4529 NA FALSE FALSE FALSE
GCST006061 0.0071 0.5588 537409 outcome TRUE reported 4703_87_LTA_TNF_b LTA
chr12 9164807 0.007955 2.36e-09 35387 TRUE reported textfile 2 1
TRUE
6878 rs6542680 T C T C -0.074 -0.0012 0.18362 NA FALSE FALSE FALSE
GCST006061 0.0091 0.8935 537409 outcome TRUE reported 4703_87_LTA_TNF_b LTA
chr2 3592552 0.010239 4.934e-13 35332 TRUE reported textfile 2 1
TRUE
6879 rs111602331 C T C T -0.0552 -0.0114 0.21885 NA FALSE FALSE FALSE
GCST006061 0.0096 0.2329 537409 outcome TRUE reported 4967_1_C1QBP_C1QBP
C1QBP chr20 45928835 0.009764 1.574e-08 35370 TRUE reported
textfile 2 1 TRUE
6880 rs111696008 A G A G -0.1604 -0.0259 0.02443 NA FALSE FALSE FALSE
GCST006061 0.0401 0.519 537409 outcome TRUE reported 4967_1_C1QBP_C1QBP
C1QBP chr2 216622986 0.026857 2.338e-09 35359 TRUE reported
textfile 2 1 TRUE
6881 rs12146727 A G A G -0.0928 -0.0037 0.11875 NA FALSE FALSE FALSE
GCST006061 0.0105 0.7269 537409 outcome TRUE reported 4967_1_C1QBP_C1QBP
C1QBP chr12 7063032 0.01271 2.845e-13 35390 TRUE reported textfile 2
1 TRUE
6882 rs1707652 T C T C 0.111 0.0081 0.3102 NA FALSE FALSE FALSE
GCST006061 0.0074 0.2701 537409 outcome TRUE reported 4967_1_C1QBP_C1QBP
C1QBP chr3 165761011 0.008705 3.095e-37 35339 TRUE reported
textfile 2 1 TRUE
6883 rs73400343 G A G A 0.1021 0.0065 0.051 NA FALSE FALSE FALSE
GCST006061 0.0146 0.6562 537409 outcome TRUE reported 4967_1_C1QBP_C1QBP
C1QBP chr11 5602460 0.018409 2.92e-08 35378 TRUE reported textfile
2 1 TRUE
6884 rs1355538 G A G A -0.0531 -0.0076 0.39847 NA FALSE FALSE FALSE
GCST006061 0.0072 0.2923 537409 outcome TRUE reported 9875_107_THOC1_THOC1
THOC1 chr3 165787389 0.008098 5.488e-11 35338 TRUE reported
textfile 2 1 TRUE
6885 rs704 A G A G 0.0456 -0.0155 0.49189 NA FALSE FALSE FALSE GCST006061
0.0072 0.03144 537409 outcome TRUE reported 9875_107_THOC1_THOC1 THOC1
chr17 28367840 0.007955 9.918e-09 35360 TRUE reported textfile 2
1 TRUE
6886 rs72637119 G T G T -0.1436 0.0055 0.04345 NA FALSE FALSE FALSE
GCST006061 0.0288 0.8472 537409 outcome TRUE reported 9875_107_THOC1_THOC1
THOC1 chr14 105853387 0.019946 6.042e-13 34967 TRUE reported
textfile 2 1 TRUE
6887 rs1451915 A C A C 0.0635 -0.0107 0.13641 NA FALSE FALSE FALSE
GCST006061 0.0124 0.3895 537409 outcome TRUE reported 17725_37_USH1C_USH1C
USH1C chr1 196058002 0.011531 3.654e-08 35335 TRUE reported
textfile 2 1 TRUE

6888 rs2686395 C T C T -0.0978 -0.0077 0.3101 NA FALSE FALSE FALSE
GCST006061 0.0074 0.2965 537409 outcome TRUE reported 17725_37_USH1C_USH1C
USH1C chr3 165767231 0.008572 3.74e-30 35339 TRUE reported
textfile 2 1 TRUE
6889 rs16876270 C A C A 0.2768 -0.0575 0.01224 NA FALSE FALSE FALSE
GCST006061 0.0204 0.004922 537409 outcome TRUE reported 17404_5_ARL5B_ARL5B
ARL5B chr5 52962521 0.037901 2.81e-13 35361 TRUE reported
textfile 2 1 TRUE
6890 rs190444444 C T C T 0.3591 -0.0038 0.01091 NA FALSE FALSE FALSE
GCST006061 0.0472 0.935 537409 outcome TRUE reported 17404_5_ARL5B_ARL5B
ARL5B chr5 41344224 0.039514 1.01e-19 35355 TRUE reported
textfile 2 1 TRUE
6891 rs62358178 G T G T 0.6173 0.0095 0.01272 NA FALSE FALSE FALSE
GCST006061 0.0318 0.7659 537409 outcome TRUE reported 17404_5_ARL5B_ARL5B
ARL5B chr5 39783951 0.036099 1.482e-65 35365 TRUE reported
textfile 2 1 TRUE
6892 rs62358228 G A G A 0.1174 -0.0312 0.05488 NA FALSE FALSE FALSE
GCST006061 0.018 0.08372 537409 outcome TRUE reported 17404_5_ARL5B_ARL5B
ARL5B chr5 40295026 0.018455 2e-10 35358 TRUE reported textfile
2 1 TRUE
6893 rs62359169 A G A G 0.0902 -0.0138 0.07461 NA FALSE FALSE FALSE
GCST006061 0.0172 0.4232 537409 outcome TRUE reported 17404_5_ARL5B_ARL5B
ARL5B chr5 41504783 0.016344 3.415e-08 35355 TRUE reported
textfile 2 1 TRUE
6894 rs6864541 G A G A 0.1925 -0.0109 0.07613 NA FALSE FALSE FALSE
GCST006061 0.0135 0.4202 537409 outcome TRUE reported 17404_5_ARL5B_ARL5B
ARL5B chr5 39364372 0.015688 1.3e-34 35365 TRUE reported textfile
2 1 TRUE
6895 rs752721 T C T C 0.1764 -0.0059 0.0336 NA FALSE FALSE FALSE
GCST006061 0.0173 0.7313 537409 outcome TRUE reported 17404_5_ARL5B_ARL5B
ARL5B chr5 38470165 0.023173 2.692e-14 35382 TRUE reported
textfile 2 1 TRUE
6896 rs853484 G A G A 0.0531 -0.0077 0.30994 NA FALSE FALSE FALSE
GCST006061 0.0074 0.2977 537409 outcome TRUE reported 17404_5_ARL5B_ARL5B
ARL5B chr3 165780046 0.008956 3.043e-09 35338 TRUE reported
textfile 2 1 TRUE
6897 rs1355538 G A G A -0.0478 -0.0076 0.39847 NA FALSE FALSE FALSE
GCST006061 0.0072 0.2923 537409 outcome TRUE reported 9783_75_DNAJC11_DJC11
DNAJC11 chr3 165787389 0.00809 3.45e-09 35338 TRUE reported textfile
2 1 TRUE
6898 rs6542680 T C T C -0.104 -0.0012 0.18362 NA FALSE FALSE FALSE
GCST006061 0.0091 0.8935 537409 outcome TRUE reported 9783_75_DNAJC11_DJC11
DNAJC11 chr2 3592552 0.010253 3.563e-24 35332 TRUE reported textfile
2 1 TRUE
6899 rs1555405 A G A G -0.048 -0.0047 0.30381 NA FALSE FALSE FALSE
GCST006061 0.0081 0.5592 537409 outcome TRUE reported 7009_8_CD72_CD72
CD72 chr14 100710432 0.008752 4.148e-08 35380 TRUE reported
textfile 2 1 TRUE
6900 rs3184504 C T C T -0.0542 0.0074 0.37862 NA FALSE FALSE FALSE
GCST006061 0.0075 0.3235 537409 outcome TRUE reported 7009_8_CD72_CD72
CD72 chr12 111446804 0.008277 5.817e-11 35371 TRUE reported
textfile 2 1 TRUE
6901 rs34791102 A G A G 0.2264 0.0154 0.06974 NA FALSE FALSE FALSE
GCST006061 0.0162 0.3397 537409 outcome TRUE reported 7009_8_CD72_CD72
CD72 chr9 35612981 0.015734 6.05e-47 35351 TRUE reported
textfile 2 1 TRUE
6902 rs1355538 G A G A -0.055 -0.0076 0.39847 NA FALSE FALSE FALSE
GCST006061 0.0072 0.2923 537409 outcome TRUE reported 11407_57_PLSCR3_PLS3
PLSCR3 chr3 165787389 0.008003 6.326e-12 35329 TRUE reported
textfile 2 1 TRUE
6903 rs1706435 G A G A 0.0516 0.0117 0.36981 NA FALSE FALSE FALSE
GCST006061 0.0072 0.1047 537409 outcome TRUE reported 7981_230_B3GALT6_B3GT6
B3GALT6 chr3 165743688 0.008161 2.57e-10 35339 TRUE reported
textfile 2 1 TRUE
6904 rs12403425 A C A C 0.0496 -0.0064 0.2662 NA FALSE FALSE FALSE
GCST006061 0.009 0.4754 537409 outcome TRUE reported 2333_72_TGFB1_TGF_b1

TGFB1 chr1 197845693 0.009011 3.699e-08 35337 TRUE reported textfile 2 1 TRUE
6905 rs1706435 G A G A 0.0621 0.0117 0.36981 NA FALSE FALSE FALSE GCST006061 0.0072 0.1047 537409 outcome TRUE reported 2333_72_TGFB1_TGF_b1 TGFB1 chr3 165743688 0.008266 5.8e-14 35339 TRUE reported textfile 2 1 TRUE
6906 rs6542680 T C T C -0.085 -0.0012 0.18362 NA FALSE FALSE FALSE GCST006061 0.0091 0.8935 537409 outcome TRUE reported 2333_72_TGFB1_TGF_b1 TGFB1 chr2 3592552 0.010341 2.044e-16 35332 TRUE reported textfile 2 1 TRUE
6907 rs1355538 G A G A -0.0542 -0.0076 0.39847 NA FALSE FALSE FALSE GCST006061 0.0072 0.2923 537409 outcome TRUE reported 13991_47_EIF4G3_IF4G3 EIF4G3 chr3 165787389 0.008035 1.521e-11 35338 TRUE reported textfile 2 1 TRUE
6908 rs704 A G A G 0.0765 -0.0155 0.49189 NA FALSE FALSE FALSE GCST006061 0.0072 0.03144 537409 outcome TRUE reported 13991_47_EIF4G3_IF4G3 EIF4G3 chr17 28367840 0.007885 2.975e-22 35360 TRUE reported textfile 2 1 TRUE
6909 rs1355538 G A G A -0.0442 -0.0076 0.39847 NA FALSE FALSE FALSE GCST006061 0.0072 0.2923 537409 outcome TRUE reported 7748_11_NDUFV2_NDUV2 NDUFV2 chr3 165787389 0.008041 3.866e-08 35338 TRUE reported textfile 2 1 TRUE
6910 rs111696008 A G A G -0.1504 -0.0259 0.02443 NA FALSE FALSE FALSE GCST006061 0.0401 0.519 537409 outcome TRUE reported 17137_160_HBB_Beta_globin HBB chr2 216622986 0.026568 1.505e-08 35359 TRUE reported textfile 2 1 TRUE
6911 rs117051919 C T C T 0.4072 -0.0176 0.01969 NA FALSE FALSE FALSE GCST006061 0.0357 0.6206 537409 outcome TRUE reported 17137_160_HBB_Beta_globin HBB chr16 72487217 0.029002 8.825e-45 35372 TRUE reported textfile 2 1 TRUE
6912 rs118107049 T C T C 0.2597 0.0336 0.01125 NA FALSE FALSE FALSE GCST006061 0.0471 0.4764 537409 outcome TRUE reported 17137_160_HBB_Beta_globin HBB chr16 73934544 0.038619 1.76e-11 35369 TRUE reported textfile 2 1 TRUE
6913 rs2461286 G A G A -0.0754 -0.0165 0.32244 NA FALSE FALSE FALSE GCST006061 0.008 0.03841 537409 outcome TRUE reported 17137_160_HBB_Beta_globin HBB chr16 153255 0.008616 2.11e-18 35315 TRUE reported textfile 2 1 TRUE
6914 rs2668202 G A G A -0.1305 -0.0076 0.30991 NA FALSE FALSE FALSE GCST006061 0.0074 0.3015 537409 outcome TRUE reported 17137_160_HBB_Beta_globin HBB chr3 165772680 0.008619 8.58e-52 35339 TRUE reported textfile 2 1 TRUE
6915 rs34924625 G A G A 0.3028 0.0035 0.01967 NA FALSE FALSE FALSE GCST006061 0.0378 0.9265 537409 outcome TRUE reported 17137_160_HBB_Beta_globin HBB chr16 69432734 0.028728 5.644e-26 35374 TRUE reported textfile 2 1 TRUE
6916 rs5030049 C T C T 0.1127 0.0098 0.12203 NA FALSE FALSE FALSE GCST006061 0.0116 0.3976 537409 outcome TRUE reported 17137_160_HBB_Beta_globin HBB chr3 186733074 0.0122 2.517e-20 35368 TRUE reported textfile 2 1 TRUE
6917 rs7113917 G A G A 0.0578 -0.001 0.22046 NA FALSE FALSE FALSE GCST006061 0.0083 0.9027 537409 outcome TRUE reported 17137_160_HBB_Beta_globin HBB chr11 34461600 0.009719 2.734e-09 35374 TRUE reported textfile 2 1 TRUE
6918 rs12478841 G A G A 0.0536 0.0049 0.21569 NA FALSE FALSE FALSE GCST006061 0.008 0.5413 537409 outcome TRUE reported 7860_9_HERC4_HERC4 HERC4 chr2 27588855 0.009676 3.029e-08 35373 TRUE reported textfile 2 1 TRUE
6919 rs1555405 A G A G -0.048 -0.0047 0.30381 NA FALSE FALSE FALSE GCST006061 0.0081 0.5592 537409 outcome TRUE reported 7860_9_HERC4_HERC4 HERC4 chr14 100710432 0.008766 4.356e-08 35380 TRUE reported textfile 2 1 TRUE
6920 rs1706435 G A G A 0.0787 0.0117 0.36981 NA FALSE FALSE FALSE GCST006061 0.0072 0.1047 537409 outcome TRUE reported 7860_9_HERC4_HERC4 HERC4 chr3 165743688 0.008304 2.6e-21 35339 TRUE reported textfile 2 1 TRUE

6921 rs1706435 G A G A 0.0738 0.0117 0.36981 NA FALSE FALSE FALSE
GCST006061 0.0072 0.1047 537409 outcome TRUE reported 11671_19_BRPF1_BRPF1
BRPF1 chr3 165743688 0.00817 1.664e-19 35330 TRUE reported textfile
2 1 TRUE
6922 rs34295758 C T C T 0.0733 0.0035 0.1212 NA FALSE FALSE FALSE
GCST006061 0.0135 0.7975 537409 outcome TRUE reported
3581_53_AHSG_a2_HS_Glycoprotein AHSG chr3 186369064 0.012785 9.863e-09
35363 TRUE reported textfile 2 1 TRUE
6923 rs3775327 C T C T -0.0525 0.0109 0.2552 NA FALSE FALSE FALSE
GCST006061 0.0082 0.1851 537409 outcome TRUE reported
3581_53_AHSG_a2_HS_Glycoprotein AHSG chr4 148064569 0.009498 3.246e-08
35362 TRUE reported textfile 2 1 TRUE
6924 rs7647776 A C A C 0.3559 -0.0075 0.29369 NA FALSE FALSE FALSE
GCST006061 0.0081 0.3533 537409 outcome TRUE reported
3581_53_AHSG_a2_HS_Glycoprotein AHSG chr3 186602713 0.007571 1e-200 35367
TRUE reported textfile 2 1 TRUE
6925 rs78963288 G A G A 0.1227 -0.0154 0.0798 NA FALSE FALSE FALSE
GCST006061 0.0216 0.4767 537409 outcome TRUE reported
3581_53_AHSG_a2_HS_Glycoprotein AHSG chr3 186330219 0.01518 6.327e-16 35358
TRUE reported textfile 2 1 TRUE
6926 rs80069596 T C T C 0.107 -0.0135 0.05488 NA FALSE FALSE FALSE
GCST006061 0.0202 0.5028 537409 outcome TRUE reported
3581_53_AHSG_a2_HS_Glycoprotein AHSG chr3 185928266 0.018238 4.438e-09
35358 TRUE reported textfile 2 1 TRUE
6927 rs822395 A C A C 0.0858 0.0018 0.31255 NA FALSE FALSE FALSE
GCST006061 0.0078 0.8151 537409 outcome TRUE reported
3581_53_AHSG_a2_HS_Glycoprotein AHSG chr3 186849018 0.009061 2.825e-21
35364 TRUE reported textfile 2 1 TRUE
6928 rs139011716 A G A G 0.2271 0.0073 0.01914 NA FALSE FALSE FALSE
GCST006061 0.0357 0.8367 537409 outcome TRUE reported 9398_30_GALP_GALP
GALP chr19 56178262 0.028703 2.529e-15 35278 TRUE reported
textfile 2 1 TRUE
6929 rs2956634 C T C T -0.2354 -0.0169 0.02769 NA FALSE FALSE FALSE
GCST006061 0.0197 0.3905 537409 outcome TRUE reported 6441_62_ADAMTS6_ATS6
ADAMTS6 chr11 18226152 0.023755 3.788e-23 35383 TRUE reported
textfile 2 1 TRUE
6930 rs4639017 C T C T -0.0456 -0.0061 0.39283 NA FALSE FALSE FALSE
GCST006061 0.0073 0.4022 537409 outcome TRUE reported 6441_62_ADAMTS6_ATS6
ADAMTS6 chr3 165833296 0.008084 1.692e-08 35339 TRUE reported
textfile 2 1 TRUE
6931 rs144274836 T C T C 0.3663 0.0195 0.13196 NA FALSE FALSE FALSE
GCST006061 0.0114 0.08685 537409 outcome TRUE reported 12423_38_A1CF_A1CF
A1CF chr3 186704578 0.0116 1e-200 35359 TRUE reported textfile 2
1 TRUE
6932 rs186023627 T C T C 0.1566 -0.0167 0.03347 NA FALSE FALSE FALSE
GCST006061 0.0227 0.4621 537409 outcome TRUE reported 12423_38_A1CF_A1CF
A1CF chr3 186491105 0.022439 2.976e-12 35357 TRUE reported
textfile 2 1 TRUE
6933 rs6542680 T C T C -0.0594 -0.0012 0.18362 NA FALSE FALSE FALSE
GCST006061 0.0091 0.8935 537409 outcome TRUE reported 7262_191_CHST14_CHSTE
CHST14 chr2 3592552 0.010212 5.995e-09 35332 TRUE reported textfile
2 1 TRUE
6934 rs1355538 G A G A -0.0485 -0.0076 0.39847 NA FALSE FALSE FALSE
GCST006061 0.0072 0.2923 537409 outcome TRUE reported
6651_74_UBB_PolyUbiquitin_K48 UBB chr3 165787389 0.008197 3.284e-09 35338
TRUE reported textfile 2 1 TRUE
6935 rs34253288 C T C T -0.0442 0.0048 0.46407 NA FALSE FALSE FALSE
GCST006061 0.0077 0.5291 537409 outcome TRUE reported
6651_74_UBB_PolyUbiquitin_K48 UBB chr16 17514498 0.008061 4.181e-08 35378
TRUE reported textfile 2 1 TRUE
6936 rs11244079 A G A G 0.1019 0.0148 0.05622 NA FALSE FALSE FALSE
GCST006061 0.0161 0.3575 537409 outcome TRUE reported 11229_16_ERN1_ERN1
ERN1 chr9 133317691 0.017146 2.795e-09 35362 TRUE reported
textfile 2 1 TRUE
6937 rs507855 G A G A -0.0455 0.0063 0.37052 NA FALSE FALSE FALSE
GCST006061 0.0076 0.408 537409 outcome TRUE reported 11229_16_ERN1_ERN1

ERN1 chr19 48705244 0.008189 2.757e-08 35368 TRUE reported
textfile 2 1 TRUE
6938 rs6542680 T C T C -0.148 -0.0012 0.18362 NA FALSE FALSE FALSE
GCST006061 0.0091 0.8935 537409 outcome TRUE reported 3738_54_CSF1_CSF_1
CSF1 chr2 3592552 0.010167 5.283e-48 35332 TRUE reported textfile
2 1 TRUE
6939 rs11677932 A G A G -0.0691 -0.0032 0.29364 NA FALSE FALSE FALSE
GCST006061 0.0075 0.6682 537409 outcome TRUE reported
11196_31_COL6A3_Collagen_alpha_3_VI_ COL6A3 chr2 237315312 0.008988
1.498e-14 35365 TRUE reported textfile 2 1 TRUE
6940 rs3088026 T C T C -0.0811 -0.0111 0.09272 NA FALSE FALSE FALSE
GCST006061 0.0131 0.3954 537409 outcome TRUE reported
11196_31_COL6A3_Collagen_alpha_3_VI_ COL6A3 chr21 46129699 0.01419 1.095e-08
34726 TRUE reported textfile 2 1 TRUE
6941 rs115028961 G A G A 0.2036 -0.052 0.02067 NA FALSE FALSE FALSE
GCST006061 0.0356 0.1441 537409 outcome TRUE reported
14011_17_S100A11_S100A11 S100A11 chr3 38459686 0.028406 7.632e-13 35358
TRUE reported textfile 2 1 TRUE
6942 rs3827295 A G A G 0.1386 -0.0398 0.06037 NA FALSE FALSE FALSE
GCST006061 0.0147 0.006739 537409 outcome TRUE reported
14011_17_S100A11_S100A11 S100A11 chr22 17119607 0.017381 1.534e-15 35338
TRUE reported textfile 2 1 TRUE
6943 rs653178 T C T C -0.0488 0.0076 0.38124 NA FALSE FALSE FALSE
GCST006061 0.0075 0.3129 537409 outcome TRUE reported
14011_17_S100A11_S100A11 S100A11 chr12 111569952 0.008356 5.219e-09 35371
TRUE reported textfile 2 1 TRUE
6944 rs78094070 G T G T 0.1848 0.0862 0.01814 NA FALSE FALSE FALSE
GCST006061 0.0641 0.1783 537409 outcome TRUE reported
14011_17_S100A11_S100A11 S100A11 chr3 40875933 0.029418 3.343e-10 35347
TRUE reported textfile 2 1 TRUE
6945 rs9880223 A G A G 0.2522 0.0059 0.10482 NA FALSE FALSE FALSE
GCST006061 0.0117 0.6106 537409 outcome TRUE reported
14011_17_S100A11_S100A11 S100A11 chr3 38252413 0.013195 1.939e-81 35357
TRUE reported textfile 2 1 TRUE
6946 rs1706435 G A G A 0.0707 0.0117 0.36981 NA FALSE FALSE FALSE
GCST006061 0.0072 0.1047 537409 outcome TRUE reported 17813_21_BPHL_BPHL
BPHL chr3 165743688 0.008177 5.315e-18 35339 TRUE reported
textfile 2 1 TRUE
6947 rs9503407 C T C T 0.2067 0.0046 0.07547 NA FALSE FALSE FALSE
GCST006061 0.0169 0.7835 537409 outcome TRUE reported 17813_21_BPHL_BPHL
BPHL chr6 3143886 0.015216 4.934e-42 35376 TRUE reported textfile
2 1 TRUE
6948 rs1706435 G A G A 0.0488 0.0117 0.36981 NA FALSE FALSE FALSE
GCST006061 0.0072 0.1047 537409 outcome TRUE reported 8589_13_CDCP1_CDCP1
CDCP1 chr3 165743688 0.008248 3.28e-09 35339 TRUE reported
textfile 2 1 TRUE
6949 rs35004449 T G T G 0.0584 -0.0241 0.26814 NA FALSE FALSE FALSE
GCST006061 0.0079 0.002283 537409 outcome TRUE reported 8589_13_CDCP1_CDCP1
CDCP1 chr3 52818881 0.008991 8.298e-11 35351 TRUE reported
textfile 2 1 TRUE
6950 rs704 A G A G -0.0911 -0.0155 0.49189 NA FALSE FALSE FALSE GCST006061
0.0072 0.03144 537409 outcome TRUE reported 8589_13_CDCP1_CDCP1 CDCP1 chr17
28367840 0.007981 3.535e-30 35360 TRUE reported textfile 2 1 TRUE
6951 rs2686395 C T C T -0.0639 -0.0077 0.3101 NA FALSE FALSE FALSE
GCST006061 0.0074 0.2965 537409 outcome TRUE reported 9012_1_PRPF6_PRP6
PRPF6 chr3 165767231 0.008543 7.455e-14 35339 TRUE reported
textfile 2 1 TRUE
6952 rs1355538 G A G A -0.0591 -0.0076 0.39847 NA FALSE FALSE FALSE
GCST006061 0.0072 0.2923 537409 outcome TRUE reported 12669_30_HECW1_HECW1
HECW1 chr3 165787389 0.008066 2.357e-13 35329 TRUE reported
textfile 2 1 TRUE
6953 rs11601665 T C T C 0.0502 -0.0045 0.26367 NA FALSE FALSE FALSE
GCST006061 0.0088 0.6088 537409 outcome TRUE reported 17367_5_STMN1_Stathmin
STMN1 chr11 262930 0.009058 2.993e-08 35352 TRUE reported textfile
2 1 TRUE
6954 rs1354034 C T C T 0.089 0.0088 0.38119 NA FALSE FALSE FALSE

GCST006061 0.0073 0.2248 537409 outcome TRUE reported 17367_5_STMN1_Stathmin
STMN1 chr3 56815721 0.008228 2.875e-27 35356 TRUE reported
textfile 2 1 TRUE
6955 rs4759076 C T C T 0.0721 -0.0011 0.4756 NA FALSE FALSE FALSE
GCST006061 0.0073 0.8799 537409 outcome TRUE reported 17367_5_STMN1_Stathmin
STMN1 chr12 54336088 0.008026 2.64e-19 35372 TRUE reported
textfile 2 1 TRUE
6956 rs6436128 T C T C -0.0825 -0.0053 0.10496 NA FALSE FALSE FALSE
GCST006061 0.0114 0.64 537409 outcome TRUE reported 17367_5_STMN1_Stathmin
STMN1 chr2 219264316 0.013139 3.408e-10 35369 TRUE reported
textfile 2 1 TRUE
6957 rs117827303 T C T C 0.1302 -0.0047 0.03794 NA FALSE FALSE FALSE
GCST006061 0.0216 0.8283 537409 outcome TRUE reported 11263_57_CASQ1_CASQ1
CASQ1 chr12 7123080 0.021045 6.146e-10 35382 TRUE reported textfile
2 1 TRUE
6958 rs11838267 C T C T 0.2003 -3.00E-04 0.11963 NA FALSE FALSE FALSE
GCST006061 0.0105 0.9793 537409 outcome TRUE reported 11263_57_CASQ1_CASQ1
CASQ1 chr12 7068568 0.012776 2.125e-55 35380 TRUE reported textfile
2 1 TRUE
6959 rs12100587 G T G T 0.079 0.0098 0.11567 NA FALSE FALSE FALSE
GCST006061 0.0137 0.4743 537409 outcome TRUE reported 11263_57_CASQ1_CASQ1
CASQ1 chr14 105537986 0.012984 1.169e-09 35091 TRUE reported
textfile 2 1 TRUE
6960 rs1355538 G A G A -0.072 -0.0076 0.39847 NA FALSE FALSE FALSE
GCST006061 0.0072 0.2923 537409 outcome TRUE reported 11263_57_CASQ1_CASQ1
CASQ1 chr3 165787389 0.008324 5.181e-18 35329 TRUE reported
textfile 2 1 TRUE
6961 rs72713385 T C T C -0.0975 -3.00E-04 0.15427 NA FALSE FALSE FALSE
GCST006061 0.0185 0.986 537409 outcome TRUE reported 11263_57_CASQ1_CASQ1
CASQ1 chr14 105732642 0.011474 1.937e-17 35089 TRUE reported
textfile 2 1 TRUE
6962 rs1355538 G A G A -0.0623 -0.0076 0.39847 NA FALSE FALSE FALSE
GCST006061 0.0072 0.2923 537409 outcome TRUE reported 13985_12_SMURF2_SMUF2
SMURF2 chr3 165787389 0.008064 1.113e-14 35338 TRUE reported
textfile 2 1 TRUE
6963 rs1355538 G A G A -0.0653 -0.0076 0.39847 NA FALSE FALSE FALSE
GCST006061 0.0072 0.2923 537409 outcome TRUE reported 13447_42_SPRN_SPRN
SPRN chr3 165787389 0.007977 2.709e-16 35332 TRUE reported
textfile 2 1 TRUE
6964 rs17090888 C T C T 0.0652 0.0122 0.13227 NA FALSE FALSE FALSE
GCST006061 0.0101 0.2294 537409 outcome TRUE reported 13447_42_SPRN_SPRN
SPRN chr14 94446899 0.0115 1.43e-08 35366 TRUE reported textfile
2 1 TRUE
6965 rs2073333 T C T C 0.2679 0.0103 0.20754 NA FALSE FALSE FALSE
GCST006061 0.0081 0.2059 537409 outcome TRUE reported 13447_42_SPRN_SPRN
SPRN chr14 94378225 0.009601 2.441e-171 35365 TRUE reported
textfile 2 1 TRUE
6966 rs2248747 C T C T 0.1125 0.0346 0.06109 NA FALSE FALSE FALSE
GCST006061 0.0315 0.272 537409 outcome TRUE reported 13447_42_SPRN_SPRN
SPRN chr2 89160700 0.016336 5.721e-12 35344 TRUE reported
textfile 2 1 TRUE
6967 rs2736894 G A G A 0.0509 0.0027 0.34843 NA FALSE FALSE FALSE
GCST006061 0.0078 0.7346 537409 outcome TRUE reported 13447_42_SPRN_SPRN
SPRN chr14 94333039 0.008227 6.142e-10 35366 TRUE reported
textfile 2 1 TRUE
6968 rs78111684 A G A G 0.2302 0.0214 0.0124 NA FALSE FALSE FALSE
GCST006061 0.037 0.564 537409 outcome TRUE reported 13447_42_SPRN_SPRN
SPRN chr14 94186307 0.035861 1.37e-10 35366 TRUE reported
textfile 2 1 TRUE
6969 rs1355538 G A G A -0.0449 -0.0076 0.39847 NA FALSE FALSE FALSE
GCST006061 0.0072 0.2923 537409 outcome TRUE reported 4132_27_FST_FST FST
chr3 165787389 0.008178 4.018e-08 35338 TRUE reported textfile 2
1 TRUE
6970 rs831429 A G A G 0.0508 -0.005 0.3045 NA FALSE FALSE FALSE
GCST006061 0.0084 0.55 537409 outcome TRUE reported 5357_60_NLGN4X_NLGNX
NLGN4X chr12 103668053 0.008661 4.489e-09 35364 TRUE reported

textfile 2 1 TRUE
6971 rs75475328 G A G A 0.125 0.0124 0.08035 NA FALSE FALSE FALSE
GCST006061 0.0146 0.3945 537409 outcome TRUE reported 4133_54_GZMB_Granzyme_B
GZMB chr6 29585114 0.014464 5.515e-18 35448 TRUE reported
textfile 2 1 TRUE
6972 rs78564162 G A G A 0.1596 0.017 0.06551 NA FALSE FALSE FALSE
GCST006061 0.0138 0.2191 537409 outcome TRUE reported 4133_54_GZMB_Granzyme_B
GZMB chr14 24689939 0.016026 2.307e-23 35378 TRUE reported
textfile 2 1 TRUE
6973 rs6542680 T C T C -0.0707 -0.0012 0.18362 NA FALSE FALSE FALSE
GCST006061 0.0091 0.8935 537409 outcome TRUE reported 6119_14_MAMDC2_MAMC2
MAMDC2 chr2 3592552 0.010245 5.171e-12 35332 TRUE reported textfile
2 1 TRUE
6974 rs6445035 A G A G 0.0647 0.0154 0.1904 NA FALSE FALSE FALSE
GCST006061 0.0089 0.08256 537409 outcome TRUE reported 11163_7_FAM162B_F162B
FAM162B chr3 165762312 0.01001 1.022e-10 35330 TRUE reported textfile
2 1 TRUE
6975 rs6542680 T C T C -0.0592 -0.0012 0.18362 NA FALSE FALSE FALSE
GCST006061 0.0091 0.8935 537409 outcome TRUE reported 11163_7_FAM162B_F162B
FAM162B chr2 3592552 0.010197 6.404e-09 35323 TRUE reported textfile
2 1 TRUE
6976 rs1451915 A C A C 0.0629 -0.0107 0.13641 NA FALSE FALSE FALSE
GCST006061 0.0124 0.3895 537409 outcome TRUE reported 13946_8_DDX23_DDX23
DDX23 chr1 196058002 0.011524 4.815e-08 35335 TRUE reported
textfile 2 1 TRUE
6977 rs6445035 A G A G 0.0943 0.0154 0.1904 NA FALSE FALSE FALSE
GCST006061 0.0089 0.08256 537409 outcome TRUE reported 13946_8_DDX23_DDX23
DDX23 chr3 165762312 0.01011 1.087e-20 35339 TRUE reported textfile
2 1 TRUE
6978 rs704 A G A G 0.1096 -0.0155 0.49189 NA FALSE FALSE FALSE GCST006061
0.0072 0.03144 537409 outcome TRUE reported 13946_8_DDX23_DDX23 DDX23 chr17
28367840 0.007958 3.698e-43 35360 TRUE reported textfile 2 1 TRUE
6979 rs1355538 G A G A -0.0578 -0.0076 0.39847 NA FALSE FALSE FALSE
GCST006061 0.0072 0.2923 537409 outcome TRUE reported 9040_144_EPDR1_EPDR1
EPDR1 chr3 165787389 0.008135 1.199e-12 35338 TRUE reported
textfile 2 1 TRUE
6980 rs62079523 A G A G 0.1273 0.0087 0.35228 NA FALSE FALSE FALSE
GCST006061 0.0079 0.2703 537409 outcome TRUE reported
4278_14_P4HB_Protein_disulfide_isomerase P4HB chr17 82394427 0.008342
1.425e-52 35019 TRUE reported textfile 2 1 TRUE
6981 rs74090714 A G A G 0.0816 -0.0102 0.14697 NA FALSE FALSE FALSE
GCST006061 0.0107 0.34 537409 outcome TRUE reported
4278_14_P4HB_Protein_disulfide_isomerase P4HB chr14 106691225 0.011505
1.314e-12 34968 TRUE reported textfile 2 1 TRUE
6982 rs1354034 C T C T 0.059 0.0088 0.38119 NA FALSE FALSE FALSE
GCST006061 0.0073 0.2248 537409 outcome TRUE reported 9808_41_TNRC6B_TNR6B
TNRC6B chr3 56815721 0.008183 5.588e-13 35356 TRUE reported
textfile 2 1 TRUE
6983 rs4980320 C T C T 0.0458 0.0041 0.46986 NA FALSE FALSE FALSE
GCST006061 0.0076 0.5858 537409 outcome TRUE reported 9808_41_TNRC6B_TNR6B
TNRC6B chr11 198986 0.007968 9.031e-09 35352 TRUE reported textfile
2 1 TRUE
6984 rs2686395 C T C T -0.0621 -0.0077 0.3101 NA FALSE FALSE FALSE
GCST006061 0.0074 0.2965 537409 outcome TRUE reported 2505_49_GFRA3_GFRa_3
GFRA3 chr3 165767231 0.008633 6.341e-13 35339 TRUE reported
textfile 2 1 TRUE
6985 rs6542680 T C T C -0.105 -0.0012 0.18362 NA FALSE FALSE FALSE
GCST006061 0.0091 0.8935 537409 outcome TRUE reported 2505_49_GFRA3_GFRa_3
GFRA3 chr2 3592552 0.010341 3.176e-24 35332 TRUE reported textfile
2 1 TRUE
6986 rs1355538 G A G A -0.0443 -0.0076 0.39847 NA FALSE FALSE FALSE
GCST006061 0.0072 0.2923 537409 outcome TRUE reported 7983_1_PCDHGC5_PCDGM
PCDHGC5 chr3 165787389 0.00804 3.595e-08 35338 TRUE reported textfile
2 1 TRUE
6987 rs4639017 C T C T -0.0556 -0.0061 0.39283 NA FALSE FALSE FALSE
GCST006061 0.0073 0.4022 537409 outcome TRUE reported 11138_16_RUNX3_RUNX3

RUNX3 chr3 165833296 0.008036 4.539e-12 35330 TRUE reported
textfile 2 1 TRUE
6988 rs1355538 G A G A -0.0505 -0.0076 0.39847 NA FALSE FALSE FALSE
GCST006061 0.0072 0.2923 537409 outcome TRUE reported 7132_55_C1QL4_C1QL4
C1QL4 chr3 165787389 0.008058 3.679e-10 35338 TRUE reported
textfile 2 1 TRUE
6989 rs35209896 A C A C 0.0779 0.0267 0.09263 NA FALSE FALSE FALSE
GCST006061 0.0355 0.4521 537409 outcome TRUE reported 7132_55_C1QL4_C1QL4
C1QL4 chr6 32519009 0.013507 8.054e-09 35439 TRUE reported
textfile 2 1 TRUE
6990 rs111266377 T G T G 0.1753 0.037 0.02883 NA FALSE FALSE FALSE
GCST006061 0.0258 0.1519 537409 outcome TRUE reported 13066_42_EDC4_EDC4
EDC4 chr3 167239870 0.023983 2.683e-13 35345 TRUE reported
textfile 2 1 TRUE
6991 rs111696008 A G A G -0.2136 -0.0259 0.02443 NA FALSE FALSE FALSE
GCST006061 0.0401 0.519 537409 outcome TRUE reported 13066_42_EDC4_EDC4
EDC4 chr2 216622986 0.026732 1.345e-15 35350 TRUE reported
textfile 2 1 TRUE
6992 rs12485327 T G T G 0.0895 0.0109 0.07334 NA FALSE FALSE FALSE
GCST006061 0.0118 0.3548 537409 outcome TRUE reported 13066_42_EDC4_EDC4
EDC4 chr3 162196930 0.015574 9.102e-09 35331 TRUE reported
textfile 2 1 TRUE
6993 rs144202860 A G A G -0.2377 -0.0629 0.01274 NA FALSE FALSE FALSE
GCST006061 0.0538 0.2423 537409 outcome TRUE reported 13066_42_EDC4_EDC4
EDC4 chr2 216625740 0.035783 3.077e-11 35350 TRUE reported
textfile 2 1 TRUE
6994 rs148077724 G A G A 0.1665 -0.0095 0.02611 NA FALSE FALSE FALSE
GCST006061 0.0214 0.6574 537409 outcome TRUE reported 13066_42_EDC4_EDC4
EDC4 chr3 165952844 0.025038 2.931e-11 35331 TRUE reported
textfile 2 1 TRUE
6995 rs2232613 T C T C -0.0742 0.0151 0.0989 NA FALSE FALSE FALSE
GCST006061 0.0141 0.2834 537409 outcome TRUE reported 13066_42_EDC4_EDC4
EDC4 chr20 38369011 0.013256 2.175e-08 35366 TRUE reported
textfile 2 1 TRUE
6996 rs55881728 G A G A -0.0449 -0.0072 0.41277 NA FALSE FALSE FALSE
GCST006061 0.0074 0.3268 537409 outcome TRUE reported 13066_42_EDC4_EDC4
EDC4 chr2 203441633 0.008219 4.681e-08 35356 TRUE reported
textfile 2 1 TRUE
6997 rs62087820 T G T G 0.0543 0.0031 0.21738 NA FALSE FALSE FALSE
GCST006061 0.0088 0.7278 537409 outcome TRUE reported 13066_42_EDC4_EDC4
EDC4 chr17 68902274 0.009845 3.473e-08 35363 TRUE reported
textfile 2 1 TRUE
6998 rs62122484 A G A G 0.1307 0.0026 0.16998 NA FALSE FALSE FALSE
GCST006061 0.0095 0.7806 537409 outcome TRUE reported 13066_42_EDC4_EDC4
EDC4 chr2 21000700 0.010706 2.812e-34 35364 TRUE reported
textfile 2 1 TRUE
6999 rs6445035 A G A G 0.2327 0.0154 0.1904 NA FALSE FALSE FALSE
GCST006061 0.0089 0.08256 537409 outcome TRUE reported 13066_42_EDC4_EDC4
EDC4 chr3 165762312 0.010185 1.592e-115 35330 TRUE reported
textfile 2 1 TRUE
7000 rs75327431 C T C T -0.097 0.0083 0.05347 NA FALSE FALSE FALSE
GCST006061 0.0222 0.7083 537409 outcome TRUE reported 13066_42_EDC4_EDC4
EDC4 chr12 42802980 0.017641 3.832e-08 35352 TRUE reported
textfile 2 1 TRUE
7001 rs6542680 T C T C -0.0881 -0.0012 0.18362 NA FALSE FALSE FALSE
GCST006061 0.0091 0.8935 537409 outcome TRUE reported 11645_9_P4HA1_P4HA1
P4HA1 chr2 3592552 0.010245 8.038e-18 35323 TRUE reported textfile
2 1 TRUE
7002 rs12146727 A G A G -0.1286 -0.0037 0.11875 NA FALSE FALSE FALSE
GCST006061 0.0105 0.7269 537409 outcome TRUE reported 12895_28_DGKB_DGKB
DGKB chr12 7063032 0.012721 5.034e-24 35381 TRUE reported textfile
2 1 TRUE
7003 rs2057681 G A G A 0.1154 -0.0136 0.28541 NA FALSE FALSE FALSE
GCST006061 0.0078 0.07965 537409 outcome TRUE reported 12895_28_DGKB_DGKB
DGKB chr7 95308945 0.00898 8.576e-38 35340 TRUE reported textfile
2 1 TRUE

7004 rs57516848 G A G A 0.1334 -0.0186 0.03033 NA FALSE FALSE FALSE
GCST006061 0.0229 0.4172 537409 outcome TRUE reported 12895_28_DGKB_DGKB
DGKB chr12 7376960 0.023928 2.474e-08 35380 TRUE reported textfile
2 1 TRUE
7005 rs1706435 G A G A 0.0612 0.0117 0.36981 NA FALSE FALSE FALSE
GCST006061 0.0072 0.1047 537409 outcome TRUE reported 12355_223_MRRF_RRFM
MRRF chr3 165743688 0.00818 7.347e-14 35330 TRUE reported textfile
2 1 TRUE
7006 rs10947428 C T C T -0.0743 -0.0088 0.20569 NA FALSE FALSE FALSE
GCST006061 0.009 0.3268 537409 outcome TRUE reported 5749_53_CLPS_COL
CLPS chr6 33679281 0.010177 2.864e-13 35360 TRUE reported
textfile 2 1 TRUE
7007 rs115985672 G A G A 0.1352 0.0235 0.03003 NA FALSE FALSE FALSE
GCST006061 0.0344 0.4932 537409 outcome TRUE reported 5749_53_CLPS_COL
CLPS chr6 38009654 0.023721 1.201e-08 35365 TRUE reported
textfile 2 1 TRUE
7008 rs2451340 A G A G -0.2214 -0.0043 0.02414 NA FALSE FALSE FALSE
GCST006061 0.0225 0.8476 537409 outcome TRUE reported 5749_53_CLPS_COL
CLPS chr6 34108351 0.026482 6.256e-17 35349 TRUE reported
textfile 2 1 TRUE
7009 rs2467071 T G T G 0.2106 -0.0669 0.02842 NA FALSE FALSE FALSE
GCST006061 0.015 8.059e-06 537409 outcome TRUE reported 5749_53_CLPS_COL
CLPS chr8 17879884 0.023726 6.918e-19 35377 TRUE reported
textfile 2 1 TRUE
7010 rs2766575 C T C T 0.3339 -0.0102 0.41021 NA FALSE FALSE FALSE
GCST006061 0.0072 0.16 537409 outcome TRUE reported 5749_53_CLPS_COL
CLPS chr6 35773900 0.006667 1e-200 35354 TRUE reported textfile
2 1 TRUE
7011 rs7763946 A G A G -0.2384 -0.008 0.15927 NA FALSE FALSE FALSE
GCST006061 0.0088 0.3646 537409 outcome TRUE reported 5749_53_CLPS_COL
CLPS chr6 35223049 0.01114 1.305e-101 35352 TRUE reported textfile
2 1 TRUE
7012 rs79476859 T C T C -0.222 0.0015 0.03197 NA FALSE FALSE FALSE
GCST006061 0.0231 0.9486 537409 outcome TRUE reported 5749_53_CLPS_COL
CLPS chr6 35657861 0.022947 3.87e-22 35353 TRUE reported
textfile 2 1 TRUE
7013 rs8051363 G A G A 0.0651 -0.005 0.26103 NA FALSE FALSE FALSE
GCST006061 0.0083 0.5493 537409 outcome TRUE reported 5749_53_CLPS_COL
CLPS chr16 75221319 0.00908 7.521e-13 35362 TRUE reported textfile
2 1 TRUE
7014 rs1355538 G A G A -0.0701 -0.0076 0.39847 NA FALSE FALSE FALSE
GCST006061 0.0072 0.2923 537409 outcome TRUE reported 14070_56_ITSN1_ITSN1
ITSN1 chr3 165787389 0.008147 7.661e-18 35338 TRUE reported
textfile 2 1 TRUE
7015 rs704 A G A G 0.0581 -0.0155 0.49189 NA FALSE FALSE FALSE GCST006061
0.0072 0.03144 537409 outcome TRUE reported 14070_56_ITSN1_ITSN1 ITSN1
chr17 28367840 0.008004 3.893e-13 35360 TRUE reported textfile 2
1 TRUE
7016 rs12478841 G A G A 0.0585 0.0049 0.21569 NA FALSE FALSE FALSE
GCST006061 0.008 0.5413 537409 outcome TRUE reported 7881_244_PARP16_PAR16
PARP16 chr2 27588855 0.009726 1.803e-09 35373 TRUE reported
textfile 2 1 TRUE
7017 rs1619994 G A G A 0.0833 0.0168 0.2807 NA FALSE FALSE FALSE
GCST006061 0.0078 0.03057 537409 outcome TRUE reported 7881_244_PARP16_PAR16
PARP16 chr3 165744047 0.008991 1.962e-20 35339 TRUE reported
textfile 2 1 TRUE
7018 rs5030049 C T C T 0.098 0.0098 0.12203 NA FALSE FALSE FALSE
GCST006061 0.0116 0.3976 537409 outcome TRUE reported 7881_244_PARP16_PAR16
PARP16 chr3 186733074 0.01231 1.71e-15 35368 TRUE reported textfile
2 1 TRUE
7019 rs704 A G A G 0.0536 -0.0155 0.49189 NA FALSE FALSE FALSE GCST006061
0.0072 0.03144 537409 outcome TRUE reported 7881_244_PARP16_PAR16 PARP16
chr17 28367840 0.008082 3.314e-11 35360 TRUE reported textfile 2
1 TRUE
7020 rs1707652 T C T C 0.0816 0.0081 0.3102 NA FALSE FALSE FALSE
GCST006061 0.0074 0.2701 537409 outcome TRUE reported 16612_28_SDC3_SDC3

SDC3 chr3 165761011 0.008838 2.637e-20 35339 TRUE reported
textfile 2 1 TRUE
7021 rs4665985 C A C A 0.0649 0.0065 0.23221 NA FALSE FALSE FALSE
GCST006061 0.0082 0.424 537409 outcome TRUE reported 16612_28_SDC3_SDC3
SDC3 chr2 27531011 0.009654 1.785e-11 35373 TRUE reported
textfile 2 1 TRUE
7022 rs71352239 T C T C 0.0895 -0.0041 0.30263 NA FALSE FALSE FALSE
GCST006061 0.0087 0.6372 537409 outcome TRUE reported 7202_107_SEMA6C_SEMA6C
SEMA6C chr19 44926286 0.008697 7.769e-25 35368 TRUE reported
textfile 2 1 TRUE
7023 rs1706435 G A G A 0.0547 0.0117 0.36981 NA FALSE FALSE FALSE
GCST006061 0.0072 0.1047 537409 outcome TRUE reported 11300_32_SORT1_SORT
SORT1 chr3 165743688 0.008059 1.14e-11 35330 TRUE reported
textfile 2 1 TRUE
7024 rs4977155 C T C T -0.0776 -0.0069 0.16663 NA FALSE FALSE FALSE
GCST006061 0.0127 0.5868 537409 outcome TRUE reported 11300_32_SORT1_SORT
SORT1 chr14 106473508 0.010688 3.851e-13 34957 TRUE reported
textfile 2 1 TRUE
7025 rs12371 G A G A 0.1617 -0.0098 0.10848 NA FALSE FALSE FALSE GCST006061
0.0132 0.4598 537409 outcome TRUE reported 12743_18_BAG5_BAG5 BAG5 chr1
180194255 0.01272 5.025e-37 35346 TRUE reported textfile 2 1 TRUE
7026 rs1355538 G A G A -0.0451 -0.0076 0.39847 NA FALSE FALSE FALSE
GCST006061 0.0072 0.2923 537409 outcome TRUE reported 12743_18_BAG5_BAG5
BAG5 chr3 165787389 0.008049 2.105e-08 35329 TRUE reported
textfile 2 1 TRUE
7027 rs2686395 C T C T -0.0773 -0.0077 0.3101 NA FALSE FALSE FALSE
GCST006061 0.0074 0.2965 537409 outcome TRUE reported 8962_48_RPS6KB1_KS6B1
RPS6KB1 chr3 165767231 0.008752 1.025e-18 35339 TRUE reported
textfile 2 1 TRUE
7028 rs6512033 A C A C -0.2973 -0.0068 0.2144 NA FALSE FALSE FALSE
GCST006061 0.0088 0.4398 537409 outcome TRUE reported 8962_48_RPS6KB1_KS6B1
RPS6KB1 chr19 15460527 0.009767 1e-200 35377 TRUE reported textfile
2 1 TRUE
7029 rs4977155 C T C T -0.106 -0.0069 0.16663 NA FALSE FALSE FALSE
GCST006061 0.0127 0.5868 537409 outcome TRUE reported 9774_59_RNF150_RN150
RNF150 chr14 106473508 0.010675 3.077e-23 34966 TRUE reported
textfile 2 1 TRUE
7030 rs1707652 T C T C 0.0817 0.0081 0.3102 NA FALSE FALSE FALSE
GCST006061 0.0074 0.2701 537409 outcome TRUE reported 14249_68_MID2_TRIM1
MID2 chr3 165761011 0.008429 3.255e-22 35339 TRUE reported
textfile 2 1 TRUE
7031 rs11130032 A G A G -0.0512 -0.0023 0.40661 NA FALSE FALSE FALSE
GCST006061 0.0074 0.7615 537409 outcome TRUE reported 16773_29_SCUBE3_SCUB3
SCUBE3 chr3 44662591 0.008135 3.1e-10 35346 TRUE reported textfile
2 1 TRUE
7032 rs3800403 G A G A 0.2734 -0.0036 0.13796 NA FALSE FALSE FALSE
GCST006061 0.0095 0.7038 537409 outcome TRUE reported 16773_29_SCUBE3_SCUB3
SCUBE3 chr6 35170473 0.011634 4.04e-122 35353 TRUE reported
textfile 2 1 TRUE
7033 rs6445035 A G A G 0.0564 0.0154 0.1904 NA FALSE FALSE FALSE
GCST006061 0.0089 0.08256 537409 outcome TRUE reported
9907_216_TNKS_Tankyrase_1 TNKS chr3 165762312 0.010003 1.717e-08 35339
TRUE reported textfile 2 1 TRUE
7034 rs6445035 A G A G 0.0763 0.0154 0.1904 NA FALSE FALSE FALSE
GCST006061 0.0089 0.08256 537409 outcome TRUE reported 9379_248_SMIM10_CX069
SMIM10 chr3 165762312 0.009863 1.025e-14 35339 TRUE reported
textfile 2 1 TRUE
7035 rs704 A G A G -0.0813 -0.0155 0.49189 NA FALSE FALSE FALSE GCST006061
0.0072 0.03144 537409 outcome TRUE reported 11162_37_EQTN_EQTN EQTN chr17
28367840 0.007728 6.997e-26 35351 TRUE reported textfile 2 1 TRUE
7036 rs1706435 G A G A 0.0632 0.0117 0.36981 NA FALSE FALSE FALSE
GCST006061 0.0072 0.1047 537409 outcome TRUE reported 18339_207_PSMB3_PSB3
PSMB3 chr3 165743688 0.008305 2.747e-14 35339 TRUE reported
textfile 2 1 TRUE
7037 rs3917538 A G A G 0.0612 -0.0137 0.21967 NA FALSE FALSE FALSE
GCST006061 0.0082 0.09535 537409 outcome TRUE reported 18339_207_PSMB3_PSB3

PSMB3 chr7 95308581 0.009704 2.856e-10 35349 TRUE reported
textfile 2 1 TRUE
7038 rs1706435 G A G A 0.0881 0.0117 0.36981 NA FALSE FALSE FALSE
GCST006061 0.0072 0.1047 537409 outcome TRUE reported 13468_5_NEURL4_NEUL4
NEURL4 chr3 165743688 0.008182 4.878e-27 35333 TRUE reported
textfile 2 1 TRUE
7039 rs438811 T C T C -0.0905 -5.00E-04 0.23201 NA FALSE FALSE FALSE
GCST006061 0.0088 0.9556 537409 outcome TRUE reported 13468_5_NEURL4_NEUL4
NEURL4 chr19 44913484 0.009457 1.073e-21 35360 TRUE reported
textfile 2 1 TRUE
7040 rs112963922 T C T C 0.6397 0.0542 0.01119 NA FALSE FALSE FALSE
GCST006061 0.0399 0.1741 537409 outcome TRUE reported 13453_2_MRPL33_RM33
MRPL33 chr14 94411531 0.037784 2.678e-64 35365 TRUE reported
textfile 2 1 TRUE
7041 rs116922373 G A G A 0.4077 -0.0161 0.01544 NA FALSE FALSE FALSE
GCST006061 0.0282 0.5685 537409 outcome TRUE reported 13453_2_MRPL33_RM33
MRPL33 chr14 94373319 0.032604 7.052e-36 35365 TRUE reported
textfile 2 1 TRUE
7042 rs118168154 A G A G 0.145 -0.0538 0.029 NA FALSE FALSE FALSE
GCST006061 0.0228 0.01804 537409 outcome TRUE reported 13453_2_MRPL33_RM33
MRPL33 chr14 94602983 0.023664 8.934e-10 35366 TRUE reported
textfile 2 1 TRUE
7043 rs12590834 A G A G 0.4118 -2.00E-04 0.11533 NA FALSE FALSE FALSE
GCST006061 0.0103 0.9841 537409 outcome TRUE reported 13453_2_MRPL33_RM33
MRPL33 chr14 94317456 0.012348 1e-200 35366 TRUE reported textfile
2 1 TRUE
7044 rs1355538 G A G A -0.0464 -0.0076 0.39847 NA FALSE FALSE FALSE
GCST006061 0.0072 0.2923 537409 outcome TRUE reported 13453_2_MRPL33_RM33
MRPL33 chr3 165787389 0.008141 1.2e-08 35332 TRUE reported textfile
2 1 TRUE
7045 rs139421381 T G T G 0.2177 0.0087 0.02168 NA FALSE FALSE FALSE
GCST006061 0.0287 0.7628 537409 outcome TRUE reported 13453_2_MRPL33_RM33
MRPL33 chr14 94459937 0.028144 1.032e-14 35366 TRUE reported
textfile 2 1 TRUE
7046 rs145115766 C T C T -0.1915 -0.1001 0.01612 NA FALSE FALSE FALSE
GCST006061 0.0532 0.05992 537409 outcome TRUE reported 13453_2_MRPL33_RM33
MRPL33 chr14 94295051 0.032405 3.429e-09 35366 TRUE reported
textfile 2 1 TRUE
7047 rs150331426 G A G A -0.1299 0.0212 0.03221 NA FALSE FALSE FALSE
GCST006061 0.0397 0.5929 537409 outcome TRUE reported 13453_2_MRPL33_RM33
MRPL33 chr14 94297718 0.022802 1.22e-08 35366 TRUE reported
textfile 2 1 TRUE
7048 rs72706301 A G A G 0.3078 0.0178 0.02593 NA FALSE FALSE FALSE
GCST006061 0.0268 0.5065 537409 outcome TRUE reported 13453_2_MRPL33_RM33
MRPL33 chr14 94385882 0.025931 1.692e-32 35366 TRUE reported
textfile 2 1 TRUE
7049 rs75285293 C T C T 0.3244 0.009 0.02122 NA FALSE FALSE FALSE
GCST006061 0.0264 0.7324 537409 outcome TRUE reported 13453_2_MRPL33_RM33
MRPL33 chr14 94386459 0.02798 4.421e-31 35366 TRUE reported textfile
2 1 TRUE
7050 rs79247904 A G A G -0.1777 -0.0019 0.03579 NA FALSE FALSE FALSE
GCST006061 0.0233 0.9334 537409 outcome TRUE reported 13453_2_MRPL33_RM33
MRPL33 chr14 94309697 0.021745 3.031e-16 35366 TRUE reported
textfile 2 1 TRUE
7051 rs1706435 G A G A 0.0527 0.0117 0.36981 NA FALSE FALSE FALSE
GCST006061 0.0072 0.1047 537409 outcome TRUE reported 12892_10_SYTL1_SYTL1
SYTL1 chr3 165743688 0.008233 1.542e-10 35330 TRUE reported
textfile 2 1 TRUE
7052 rs11846959 A G A G 0.1455 0.0105 0.20756 NA FALSE FALSE FALSE
GCST006061 0.0081 0.1966 537409 outcome TRUE reported 11402_17_KMT2C_KMT2C
KMT2C chr14 94379979 0.009664 3.141e-51 35362 TRUE reported
textfile 2 1 TRUE
7053 rs1706435 G A G A 0.0704 0.0117 0.36981 NA FALSE FALSE FALSE
GCST006061 0.0072 0.1047 537409 outcome TRUE reported 11402_17_KMT2C_KMT2C
KMT2C chr3 165743688 0.008094 3.387e-18 35330 TRUE reported
textfile 2 1 TRUE

7054 rs1260326 C T C T -0.0628 -0.0069 0.34132 NA FALSE FALSE FALSE
GCST006061 0.0073 0.3401 537409 outcome TRUE reported
15513_108_PRSS8_Prostasin PRSS8 chr2 27508073 0.008638 3.597e-13 35373
TRUE reported textfile 2 1 TRUE

7055 rs147398533 G A G A 0.1044 0.0057 0.04744 NA FALSE FALSE FALSE
GCST006061 0.0401 0.8861 537409 outcome TRUE reported
15513_108_PRSS8_Prostasin PRSS8 chr16 32943400 0.018996 3.888e-08 35364
TRUE reported textfile 2 1 TRUE

7056 rs1706435 G A G A 0.0611 0.0117 0.36981 NA FALSE FALSE FALSE
GCST006061 0.0072 0.1047 537409 outcome TRUE reported
15513_108_PRSS8_Prostasin PRSS8 chr3 165743688 0.008443 4.582e-13 35339
TRUE reported textfile 2 1 TRUE

7057 rs55667375 T C T C 0.0597 -0.0076 0.28217 NA FALSE FALSE FALSE
GCST006061 0.0083 0.3607 537409 outcome TRUE reported
15513_108_PRSS8_Prostasin PRSS8 chr16 31144137 0.009041 4.015e-11 35367
TRUE reported textfile 2 1 TRUE

7058 rs76045563 T C T C 0.1537 -0.0163 0.03011 NA FALSE FALSE FALSE
GCST006061 0.0296 0.5829 537409 outcome TRUE reported
15513_108_PRSS8_Prostasin PRSS8 chr16 33797169 0.023872 1.206e-10 35365
TRUE reported textfile 2 1 TRUE

7059 rs2057681 G A G A 0.2424 -0.0136 0.28541 NA FALSE FALSE FALSE
GCST006061 0.0078 0.07965 537409 outcome TRUE reported 18842_24_UBE2D2_UB2D2
UBE2D2 chr7 95308945 0.008856 6.174e-165 35349 TRUE reported
textfile 2 1 TRUE

7060 rs6445035 A G A G 0.1246 0.0154 0.1904 NA FALSE FALSE FALSE
GCST006061 0.0089 0.08256 537409 outcome TRUE reported 18842_24_UBE2D2_UB2D2
UBE2D2 chr3 165762312 0.010176 1.807e-34 35339 TRUE reported
textfile 2 1 TRUE

7061 rs1355538 G A G A -0.0571 -0.0076 0.39847 NA FALSE FALSE FALSE
GCST006061 0.0072 0.2923 537409 outcome TRUE reported 13452_113_SMIM13_SIM13
SMIM13 chr3 165787389 0.008052 1.326e-12 35332 TRUE reported
textfile 2 1 TRUE

7062 rs6542680 T C T C -0.0828 -0.0012 0.18362 NA FALSE FALSE FALSE
GCST006061 0.0091 0.8935 537409 outcome TRUE reported 6549_60_VSTM2L_VTM2L
VSTM2L chr2 3592552 0.010161 3.68e-16 35332 TRUE reported textfile
2 1 TRUE

7063 rs12366520 A C A C -0.0908 -0.0053 0.12386 NA FALSE FALSE FALSE
GCST006061 0.0106 0.6159 537409 outcome TRUE reported 3030_3_CLEC4M_DC_SIGNR
CLEC4M chr12 7073858 0.012351 1.959e-13 35389 TRUE reported textfile
2 1 TRUE

7064 rs1707652 T C T C 0.1033 0.0081 0.3102 NA FALSE FALSE FALSE
GCST006061 0.0074 0.2701 537409 outcome TRUE reported 3030_3_CLEC4M_DC_SIGNR
CLEC4M chr3 165761011 0.008628 4.958e-33 35339 TRUE reported
textfile 2 1 TRUE

7065 rs1355538 G A G A -0.0445 -0.0076 0.39847 NA FALSE FALSE FALSE
GCST006061 0.0072 0.2923 537409 outcome TRUE reported 10876_300_BRAT1_BRAT1
BRAT1 chr3 165787389 0.008016 2.835e-08 35326 TRUE reported
textfile 2 1 TRUE

7066 rs6542680 T C T C -0.0853 -0.0012 0.18362 NA FALSE FALSE FALSE
GCST006061 0.0091 0.8935 537409 outcome TRUE reported 4284_18_RBM39_RBM39
RBM39 chr2 3592552 0.010351 1.715e-16 35332 TRUE reported textfile
2 1 TRUE

7067 rs1355538 G A G A -0.0488 -0.0076 0.39847 NA FALSE FALSE FALSE
GCST006061 0.0072 0.2923 537409 outcome TRUE reported 6456_17_PSG6_PSG6
PSG6 chr3 165787389 0.00801 1.11e-09 35338 TRUE reported textfile
2 1 TRUE

7068 rs35209896 A C A C 0.0749 0.0267 0.09263 NA FALSE FALSE FALSE
GCST006061 0.0355 0.4521 537409 outcome TRUE reported 6456_17_PSG6_PSG6
PSG6 chr6 32519009 0.013409 2.326e-08 35439 TRUE reported
textfile 2 1 TRUE

7069 rs1706435 G A G A 0.0695 0.0117 0.36981 NA FALSE FALSE FALSE
GCST006061 0.0072 0.1047 537409 outcome TRUE reported 19126_4_RPRD1B_C20orf77
RPRD1B chr3 165743688 0.00819 2.143e-17 35339 TRUE reported textfile
2 1 TRUE

7070 rs6542680 T C T C -0.1334 -0.0012 0.18362 NA FALSE FALSE FALSE
GCST006061 0.0091 0.8935 537409 outcome TRUE reported 3065_65_FGF5_FGF_5

FGF5 chr2 3592552 0.010158 2.143e-39 35332 TRUE reported textfile
2 1 TRUE
7071 rs7131073 G A G A 0.0731 -0.0154 0.35239 NA FALSE FALSE FALSE
GCST006061 0.0074 0.03752 537409 outcome TRUE reported 10440_26_CLMP_ACAM
CLMP chr11 123145655 0.008629 2.427e-17 35359 TRUE reported
textfile 2 1 TRUE
7072 rs77924615 A G A G -0.066 0.0136 0.19701 NA FALSE FALSE FALSE
GCST006061 0.0092 0.1375 537409 outcome TRUE reported 10440_26_CLMP_ACAM
CLMP chr16 20381010 0.010382 2.055e-10 35343 TRUE reported
textfile 2 1 TRUE
7073 rs7946718 G A G A 0.2113 0.0026 0.4079 NA FALSE FALSE FALSE
GCST006061 0.0075 0.7342 537409 outcome TRUE reported 10440_26_CLMP_ACAM
CLMP chr11 123197533 0.008322 3.116e-142 35355 TRUE reported
textfile 2 1 TRUE
7074 rs1707652 T C T C 0.0884 0.0081 0.3102 NA FALSE FALSE FALSE
GCST006061 0.0074 0.2701 537409 outcome TRUE reported 19635_69_FGL2_FGL2
FGL2 chr3 165761011 0.008783 7.907e-24 35339 TRUE reported
textfile 2 1 TRUE
7075 rs66471312 C T C T -0.0717 -0.0071 0.16445 NA FALSE FALSE FALSE
GCST006061 0.0096 0.4569 537409 outcome TRUE reported 19635_69_FGL2_FGL2
FGL2 chr7 77221788 0.010971 6.338e-11 35351 TRUE reported
textfile 2 1 TRUE
7076 rs6542680 T C T C -0.061 -0.0012 0.18362 NA FALSE FALSE FALSE
GCST006061 0.0091 0.8935 537409 outcome TRUE reported 7003_4_GALNT10_GLT10
GALNT10 chr2 3592552 0.010088 1.479e-09 35332 TRUE reported textfile
2 1 TRUE
7077 rs1129264 G A G A -0.0787 0.0186 0.3185 NA FALSE FALSE FALSE
GCST006061 0.0079 0.01791 537409 outcome TRUE reported 17737_7_IVD_IVD IVD
chr15 40331623 0.008496 1.98e-20 35361 TRUE reported textfile 2
1 TRUE
7078 rs12902310 C T C T 0.1105 0.0122 0.36207 NA FALSE FALSE FALSE
GCST006061 0.0074 0.09885 537409 outcome TRUE reported 17737_7_IVD_IVD IVD
chr15 40423228 0.008218 3.219e-41 35363 TRUE reported textfile 2
1 TRUE
7079 rs13394512 C T C T -0.045 -0.0026 0.37013 NA FALSE FALSE FALSE
GCST006061 0.0075 0.7325 537409 outcome TRUE reported 17737_7_IVD_IVD IVD
chr2 31057272 0.008194 3.971e-08 35364 TRUE reported textfile 2
1 TRUE
7080 rs1706435 G A G A 0.048 0.0117 0.36981 NA FALSE FALSE FALSE
GCST006061 0.0072 0.1047 537409 outcome TRUE reported 17737_7_IVD_IVD IVD
chr3 165743688 0.008186 4.526e-09 35339 TRUE reported textfile 2
1 TRUE
7081 rs2393778 T G T G -0.0791 0.0017 0.22691 NA FALSE FALSE FALSE
GCST006061 0.008 0.8261 537409 outcome TRUE reported 17737_7_IVD_IVD IVD
chr12 120702516 0.009422 4.661e-17 35368 TRUE reported textfile 2
1 TRUE
7082 rs1707652 T C T C 0.11 0.0081 0.3102 NA FALSE FALSE FALSE
GCST006061 0.0074 0.2701 537409 outcome TRUE reported 13073_14_ANP32A_AN32A
ANP32A chr3 165761011 0.008614 2.413e-37 35330 TRUE reported
textfile 2 1 TRUE
7083 rs2232613 T C T C -0.1108 0.0151 0.0989 NA FALSE FALSE FALSE
GCST006061 0.0141 0.2834 537409 outcome TRUE reported 13073_14_ANP32A_AN32A
ANP32A chr20 38369011 0.013192 4.501e-17 35366 TRUE reported
textfile 2 1 TRUE
7084 rs1706420 C T C T 0.0751 0.0122 0.36627 NA FALSE FALSE FALSE
GCST006061 0.0072 0.0907 537409 outcome TRUE reported 12775_6_HMGB3_HMGB3
HMGB3 chr3 165732681 0.008226 6.891e-20 35330 TRUE reported
textfile 2 1 TRUE
7085 rs35209896 A C A C 0.0759 0.0267 0.09263 NA FALSE FALSE FALSE
GCST006061 0.0355 0.4521 537409 outcome TRUE reported 12775_6_HMGB3_HMGB3
HMGB3 chr6 32519009 0.013558 2.167e-08 35430 TRUE reported
textfile 2 1 TRUE
7086 rs12589344 C A C A -0.0453 0.0057 0.4215 NA FALSE FALSE FALSE
GCST006061 0.0073 0.4387 537409 outcome TRUE reported 2754_50_C3_C3 C3
chr14 85491326 0.008238 3.818e-08 35355 TRUE reported textfile 2
1 TRUE

7087 rs6870182 A G A G 0.0506 0.0046 0.24923 NA FALSE FALSE FALSE
GCST006061 0.008 0.5663 537409 outcome TRUE reported 14309_8_HNRNPH1_HNRH1
HNRNPH1 chr5 36383678 0.009252 4.525e-08 35379 TRUE reported
textfile 2 1 TRUE
7088 rs78385612 A G A G -0.0628 -0.0041 0.22671 NA FALSE FALSE FALSE
GCST006061 0.0083 0.6175 537409 outcome TRUE reported 14309_8_HNRNPH1_HNRH1
HNRNPH1 chr3 186462462 0.009613 6.453e-11 35366 TRUE reported
textfile 2 1 TRUE
7089 rs113747960 A G A G -0.0726 -0.0034 0.12384 NA FALSE FALSE FALSE
GCST006061 0.0107 0.7473 537409 outcome TRUE reported 17152_10_KIR2DS4_KI2S4
KIR2DS4 chr12 7074644 0.012315 3.737e-09 35389 TRUE reported textfile
2 1 TRUE
7090 rs1355538 G A G A -0.0532 -0.0076 0.39847 NA FALSE FALSE FALSE
GCST006061 0.0072 0.2923 537409 outcome TRUE reported 17152_10_KIR2DS4_KI2S4
KIR2DS4 chr3 165787389 0.008137 6.241e-11 35338 TRUE reported
textfile 2 1 TRUE
7091 rs9902706 T C T C -0.2228 -0.05 0.05353 NA FALSE FALSE FALSE
GCST006061 0.0152 0.0009747 537409 outcome TRUE reported
17152_10_KIR2DS4_KI2S4 KIR2DS4 chr17 66190870 0.018001 3.482e-35 35370
TRUE reported textfile 2 1 TRUE
7092 rs1354034 C T C T 0.0502 0.0088 0.38119 NA FALSE FALSE FALSE
GCST006061 0.0073 0.2248 537409 outcome TRUE reported 9064_12_RNF215_RN215
RNF215 chr3 56815721 0.00813 6.626e-10 35356 TRUE reported textfile
2 1 TRUE
7093 rs185009887 A G A G -0.1662 -0.0595 0.02519 NA FALSE FALSE FALSE
GCST006061 0.0496 0.2302 537409 outcome TRUE reported 9064_12_RNF215_RN215
RNF215 chr17 5848332 0.025614 8.656e-11 35374 TRUE reported textfile
2 1 TRUE
7094 rs4980320 C T C T 0.0459 0.0041 0.46986 NA FALSE FALSE FALSE
GCST006061 0.0076 0.5858 537409 outcome TRUE reported 9064_12_RNF215_RN215
RNF215 chr11 198986 0.00793 7.123e-09 35352 TRUE reported textfile 2
1 TRUE
7095 rs1706435 G A G A 0.0504 0.0117 0.36981 NA FALSE FALSE FALSE
GCST006061 0.0072 0.1047 537409 outcome TRUE reported
7922_5_ADM_Adrenomedullin ADM chr3 165743688 0.008181 7.252e-10 35339
TRUE reported textfile 2 1 TRUE
7096 rs10971512 A C A C 0.1274 -0.0111 0.17277 NA FALSE FALSE FALSE
GCST006061 0.0091 0.2242 537409 outcome TRUE reported 10974_20_SPINK7_ISK7
SPINK7 chr9 33437284 0.010934 2.251e-31 35339 TRUE reported
textfile 2 1 TRUE
7097 rs1355538 G A G A -0.0537 -0.0076 0.39847 NA FALSE FALSE FALSE
GCST006061 0.0072 0.2923 537409 outcome TRUE reported 10974_20_SPINK7_ISK7
SPINK7 chr3 165787389 0.0084 1.626e-10 35329 TRUE reported textfile
2 1 TRUE
7098 rs2727270 T C T C -0.0619 -0.0038 0.16553 NA FALSE FALSE FALSE
GCST006061 0.0104 0.7143 537409 outcome TRUE reported 10974_20_SPINK7_ISK7
SPINK7 chr11 61835765 0.011104 2.481e-08 35358 TRUE reported
textfile 2 1 TRUE
7099 rs322372 G A G A -0.1007 0.0068 0.05848 NA FALSE FALSE FALSE
GCST006061 0.0166 0.6828 537409 outcome TRUE reported 10974_20_SPINK7_ISK7
SPINK7 chr5 172749458 0.017618 1.092e-08 35355 TRUE reported
textfile 2 1 TRUE
7100 rs34155263 T C T C -0.0464 0.003 0.4112 NA FALSE FALSE FALSE
GCST006061 0.0073 0.6801 537409 outcome TRUE reported 10974_20_SPINK7_ISK7
SPINK7 chr2 72036095 0.008427 3.663e-08 35341 TRUE reported
textfile 2 1 TRUE
7101 rs34529371 A G A G -0.0639 -0.0066 0.15843 NA FALSE FALSE FALSE
GCST006061 0.0094 0.4834 537409 outcome TRUE reported 10974_20_SPINK7_ISK7
SPINK7 chr14 23280869 0.011221 1.237e-08 35368 TRUE reported
textfile 2 1 TRUE
7102 rs61816766 C T C T 0.1718 -0.0108 0.01942 NA FALSE FALSE FALSE
GCST006061 0.0264 0.682 537409 outcome TRUE reported 10974_20_SPINK7_ISK7
SPINK7 chr1 152347096 0.030331 1.478e-08 35355 TRUE reported
textfile 2 1 TRUE
7103 rs77924615 A G A G -0.0717 0.0136 0.19701 NA FALSE FALSE FALSE
GCST006061 0.0092 0.1375 537409 outcome TRUE reported 10974_20_SPINK7_ISK7

SPINK7 chr16 20381010 0.010423 6.037e-12 35346 TRUE reported
textfile 2 1 TRUE
7104 rs9784645 T G T G 0.1322 0.0021 0.25293 NA FALSE FALSE FALSE
GCST006061 0.008 0.7935 537409 outcome TRUE reported 10974_20_SPINK7_ISK7
SPINK7 chr5 148337418 0.009459 2.184e-44 35349 TRUE reported
textfile 2 1 TRUE
7105 rs12378445 C T C T -0.0712 -7.00E-04 0.27922 NA FALSE FALSE FALSE
GCST006061 0.0081 0.9331 537409 outcome TRUE reported 9199_6_UBE2G2_UB2G2
UBE2G2 chr9 114067500 0.009289 1.785e-14 35362 TRUE reported
textfile 2 1 TRUE
7106 rs429358 C T C T -0.0769 0.005 0.17407 NA FALSE FALSE FALSE
GCST006061 0.0103 0.6319 537409 outcome TRUE reported 9199_6_UBE2G2_UB2G2
UBE2G2 chr19 44908684 0.011057 3.531e-12 35368 TRUE reported
textfile 2 1 TRUE
7107 rs9273325 A G A G -0.0929 -0.0031 0.14149 NA FALSE FALSE FALSE
GCST006061 0.0114 0.7866 537409 outcome TRUE reported 9199_6_UBE2G2_UB2G2
UBE2G2 chr6 32655416 0.012054 1.286e-14 35439 TRUE reported
textfile 2 1 TRUE
7108 rs112930619 C A C A -0.7924 0.0129 0.02581 NA FALSE FALSE FALSE
GCST006061 0.0298 0.6654 537409 outcome TRUE reported 10572_65_CST8_CST8
CST8 chr17 35961002 0.026145 1e-200 35354 TRUE reported textfile
2 1 TRUE
7109 rs117517474 C T C T -0.2423 0.0943 0.01071 NA FALSE FALSE FALSE
GCST006061 0.0504 0.06137 537409 outcome TRUE reported 10572_65_CST8_CST8
CST8 chr17 35556493 0.040959 3.304e-09 35358 TRUE reported
textfile 2 1 TRUE
7110 rs150951362 A G A G -0.7549 -0.0092 0.03577 NA FALSE FALSE FALSE
GCST006061 0.0156 0.5574 537409 outcome TRUE reported 10572_65_CST8_CST8
CST8 chr17 35977228 0.021873 1e-200 35354 TRUE reported textfile
2 1 TRUE
7111 rs1707652 T C T C 0.065 0.0081 0.3102 NA FALSE FALSE FALSE
GCST006061 0.0074 0.2701 537409 outcome TRUE reported 10572_65_CST8_CST8
CST8 chr3 165761011 0.009007 5.333e-13 35327 TRUE reported
textfile 2 1 TRUE
7112 rs41324748 G A G A -0.5803 0.0219 0.01015 NA FALSE FALSE FALSE
GCST006061 0.0463 0.6357 537409 outcome TRUE reported 10572_65_CST8_CST8
CST8 chr17 35498384 0.042042 2.45e-43 35351 TRUE reported
textfile 2 1 TRUE
7113 rs4251784 A G A G -0.2631 0.0162 0.04233 NA FALSE FALSE FALSE
GCST006061 0.0321 0.6139 537409 outcome TRUE reported 10572_65_CST8_CST8
CST8 chr17 35844448 0.02071 5.624e-37 35358 TRUE reported textfile
2 1 TRUE
7114 rs1619994 G A G A 0.0667 0.0168 0.2807 NA FALSE FALSE FALSE
GCST006061 0.0078 0.03057 537409 outcome TRUE reported 18863_176_CHST2_CHST2
CHST2 chr3 165744047 0.008677 1.502e-14 35339 TRUE reported
textfile 2 1 TRUE
7115 rs2232613 T C T C -0.0751 0.0151 0.0989 NA FALSE FALSE FALSE
GCST006061 0.0141 0.2834 537409 outcome TRUE reported 18863_176_CHST2_CHST2
CHST2 chr20 38369011 0.012863 5.273e-09 35375 TRUE reported
textfile 2 1 TRUE
7116 rs1260326 C T C T -0.0755 -0.0069 0.34132 NA FALSE FALSE FALSE
GCST006061 0.0073 0.3401 537409 outcome TRUE reported
15487_164_CES1_carboxylesterase__liver CES1 chr2 27508073 0.008543
9.814e-19 35373 TRUE reported textfile 2 1 TRUE
7117 rs1707652 T C T C 0.0941 0.0081 0.3102 NA FALSE FALSE FALSE
GCST006061 0.0074 0.2701 537409 outcome TRUE reported
15487_164_CES1_carboxylesterase__liver CES1 chr3 165761011 0.008695
2.688e-27 35339 TRUE reported textfile 2 1 TRUE
7118 rs3859101 A G A G -0.1116 -0.0151 0.04384 NA FALSE FALSE FALSE
GCST006061 0.0211 0.4747 537409 outcome TRUE reported
15487_164_CES1_carboxylesterase__liver CES1 chr16 55833669 0.019939
2.181e-08 35367 TRUE reported textfile 2 1 TRUE
7119 rs4240624 A G A G -0.0977 -0.016 0.07464 NA FALSE FALSE FALSE
GCST006061 0.0128 0.2119 537409 outcome TRUE reported
15487_164_CES1_carboxylesterase__liver CES1 chr8 9326721 0.015308 1.746e-10
35385 TRUE reported textfile 2 1 TRUE

7120 rs1706435 G A G A 0.0548 0.0117 0.36981 NA FALSE FALSE FALSE
GCST006061 0.0072 0.1047 537409 outcome TRUE reported 5062_60_CD226_CD226
CD226 chr3 165743688 0.008171 1.99e-11 35339 TRUE reported
textfile 2 1 TRUE
7121 rs2232613 T C T C -0.1475 0.0151 0.0989 NA FALSE FALSE FALSE
GCST006061 0.0141 0.2834 537409 outcome TRUE reported 5062_60_CD226_CD226
CD226 chr20 38369011 0.013025 9.928e-30 35375 TRUE reported
textfile 2 1 TRUE
7122 rs6542680 T C T C -0.1271 -0.0012 0.18362 NA FALSE FALSE FALSE
GCST006061 0.0091 0.8935 537409 outcome TRUE reported 5062_60_CD226_CD226
CD226 chr2 3592552 0.010213 1.501e-35 35332 TRUE reported textfile
2 1 TRUE
7123 rs2686395 C T C T -0.0669 -0.0077 0.3101 NA FALSE FALSE FALSE
GCST006061 0.0074 0.2965 537409 outcome TRUE reported 9930_48_GABBR2_GABR2
GABBR2 chr3 165767231 0.008442 2.289e-15 35339 TRUE reported
textfile 2 1 TRUE
7124 rs3820897 C T C T -0.0642 -0.0012 0.18633 NA FALSE FALSE FALSE
GCST006061 0.0091 0.893 537409 outcome TRUE reported
11617_1_ITGAL_LFA_1_alpha_L_chain ITGAL chr2 3594771 0.010151 2.544e-10
35324 TRUE reported textfile 2 1 TRUE
7125 rs1706435 G A G A 0.0567 0.0117 0.36981 NA FALSE FALSE FALSE
GCST006061 0.0072 0.1047 537409 outcome TRUE reported 19206_20_NUDT1_8ODP
NUDT1 chr3 165743688 0.008312 8.989e-12 35339 TRUE reported
textfile 2 1 TRUE
7126 rs4693545 G T G T -0.0607 0.0286 0.15993 NA FALSE FALSE FALSE
GCST006061 0.0093 0.002024 537409 outcome TRUE reported 19206_20_NUDT1_8ODP
NUDT1 chr4 83034489 0.010932 2.819e-08 35355 TRUE reported
textfile 2 1 TRUE
7127 rs1706435 G A G A 0.0809 0.0117 0.36981 NA FALSE FALSE FALSE
GCST006061 0.0072 0.1047 537409 outcome TRUE reported 19280_29_UBE2D3_UB2D3
UBE2D3 chr3 165743688 0.008297 1.832e-22 35339 TRUE reported
textfile 2 1 TRUE
7128 rs73262897 A G A G -0.087 -0.011 0.09123 NA FALSE FALSE FALSE
GCST006061 0.0134 0.41 537409 outcome TRUE reported 19280_29_UBE2D3_UB2D3
UBE2D3 chr12 6961967 0.01399 5.011e-10 35391 TRUE reported textfile 2
1 TRUE
7129 rs1180658 A C A C -0.0591 -0.0051 0.36209 NA FALSE FALSE FALSE
GCST006061 0.0076 0.502 537409 outcome TRUE reported 9522_3_AIFM1_AIF
AIFM1 chr10 59886187 0.008309 1.14e-12 35352 TRUE reported
textfile 2 1 TRUE
7130 rs704 A G A G 0.0485 -0.0155 0.49189 NA FALSE FALSE FALSE GCST006061
0.0072 0.03144 537409 outcome TRUE reported 9522_3_AIFM1_AIF AIFM1 chr17
28367840 0.008012 1.417e-09 35360 TRUE reported textfile 2 1 TRUE
7131 rs1707652 T C T C 0.0809 0.0081 0.3102 NA FALSE FALSE FALSE
GCST006061 0.0074 0.2701 537409 outcome TRUE reported 13610_9_MAGEA10_MAGE_10
MAGEA10 chr3 165761011 0.008801 3.854e-20 35333 TRUE reported
textfile 2 1 TRUE
7132 rs2388595 T C T C 0.2175 -0.0059 0.3753 NA FALSE FALSE FALSE
GCST006061 0.0074 0.4204 537409 outcome TRUE reported 13610_9_MAGEA10_MAGE_10
MAGEA10 chr10 3095817 0.008487 7.331e-145 35307 TRUE reported textfile
2 1 TRUE
7133 rs62269916 A G A G 0.1304 -0.0234 0.03471 NA FALSE FALSE FALSE
GCST006061 0.0238 0.3259 537409 outcome TRUE reported 17356_34_IL1F10_IL1FA
IL1F10 chr3 74348841 0.021589 1.541e-09 35343 TRUE reported
textfile 2 1 TRUE
7134 rs1706435 G A G A 0.0518 0.0117 0.36981 NA FALSE FALSE FALSE
GCST006061 0.0072 0.1047 537409 outcome TRUE reported 14002_18_DOCK9_DOCK9
DOCK9 chr3 165743688 0.008228 3.07e-10 35339 TRUE reported
textfile 2 1 TRUE
7135 rs34762726 A G A G -0.048 -0.0047 0.30713 NA FALSE FALSE FALSE
GCST006061 0.008 0.5543 537409 outcome TRUE reported 14002_18_DOCK9_DOCK9
DOCK9 chr3 49651777 0.008603 2.416e-08 35343 TRUE reported
textfile 2 1 TRUE
7136 rs12132412 G A G A -0.0516 0.0055 0.34943 NA FALSE FALSE FALSE
GCST006061 0.0077 0.4794 537409 outcome TRUE reported
13113_7_SPP1_Osteopontin SPP1 chr1 21493549 0.008487 1.201e-09 35365

TRUE reported textfile 2 1 TRUE
7137 rs149063279 G A G A -0.2228 -0.034 0.0109 NA FALSE FALSE FALSE
GCST006061 0.0445 0.4449 537409 outcome TRUE reported
13113_7_SPP1_Osteopontin SPP1 chr4 83784891 0.038689 8.477e-09 35352
TRUE reported textfile 2 1 TRUE
7138 rs181830063 A G A G -0.3072 0.0262 0.01462 NA FALSE FALSE FALSE
GCST006061 0.0562 0.641 537409 outcome TRUE reported
13113_7_SPP1_Osteopontin SPP1 chr4 85489313 0.032709 5.895e-21 35346
TRUE reported textfile 2 1 TRUE
7139 rs9996125 A G A G -0.109 -0.0017 0.08546 NA FALSE FALSE FALSE
GCST006061 0.0146 0.907 537409 outcome TRUE reported
13113_7_SPP1_Osteopontin SPP1 chr4 88364077 0.014523 6.129e-14 35336
TRUE reported textfile 2 1 TRUE
7140 rs6542680 T C T C -0.0625 -0.0012 0.18362 NA FALSE FALSE FALSE
GCST006061 0.0091 0.8935 537409 outcome TRUE reported 12553_5_VRK1_VRK1
VRK1 chr2 3592552 0.010216 9.5e-10 35323 TRUE reported textfile 2
1 TRUE
7141 rs12075 A G A G 0.0679 5e-04 0.42429 NA FALSE FALSE FALSE GCST006061
0.0074 0.9502 537409 outcome TRUE reported 3447_64_CXCL8_IL_8 CXCL8 chr1
159205564 0.008138 7.204e-17 35358 TRUE reported textfile 2 1 TRUE
7142 rs1706435 G A G A 0.0504 0.0117 0.36981 NA FALSE FALSE FALSE
GCST006061 0.0072 0.1047 537409 outcome TRUE reported 3447_64_CXCL8_IL_8
CXCL8 chr3 165743688 0.008296 1.241e-09 35339 TRUE reported
textfile 2 1 TRUE
7143 rs79237872 T G T G -0.1407 -0.0055 0.02388 NA FALSE FALSE FALSE
GCST006061 0.0171 0.748 537409 outcome TRUE reported 3447_64_CXCL8_IL_8
CXCL8 chr4 73696328 0.025686 4.311e-08 35347 TRUE reported
textfile 2 1 TRUE
7144 rs1355538 G A G A -0.045 -0.0076 0.39847 NA FALSE FALSE FALSE
GCST006061 0.0072 0.2923 537409 outcome TRUE reported 11692_21_SHC4_SHC4
SHC4 chr3 165787389 0.008028 2.078e-08 35329 TRUE reported
textfile 2 1 TRUE
7145 rs1555405 A G A G -0.0469 -0.0047 0.30381 NA FALSE FALSE FALSE
GCST006061 0.0081 0.5592 537409 outcome TRUE reported 11692_21_SHC4_SHC4
SHC4 chr14 100710432 0.008587 4.721e-08 35371 TRUE reported
textfile 2 1 TRUE
7146 rs6542680 T C T C -0.06 -0.0012 0.18362 NA FALSE FALSE FALSE
GCST006061 0.0091 0.8935 537409 outcome TRUE reported 11692_21_SHC4_SHC4
SHC4 chr2 3592552 0.010182 3.804e-09 35323 TRUE reported textfile
2 1 TRUE
7147 rs11569415 A G A G -0.0569 0.0011 0.24276 NA FALSE FALSE FALSE
GCST006061 0.0097 0.9129 537409 outcome TRUE reported 9229_9_CTRL_CTRL
CTRL chr19 6716268 0.009194 6.074e-10 35372 TRUE reported textfile
2 1 TRUE
7148 rs1354034 C T C T 0.088 0.0088 0.38119 NA FALSE FALSE FALSE
GCST006061 0.0073 0.2248 537409 outcome TRUE reported 9229_9_CTRL_CTRL
CTRL chr3 56815721 0.008148 3.459e-27 35356 TRUE reported
textfile 2 1 TRUE
7149 rs1707652 T C T C 0.0821 0.0081 0.3102 NA FALSE FALSE FALSE
GCST006061 0.0074 0.2701 537409 outcome TRUE reported 9229_9_CTRL_CTRL
CTRL chr3 165761011 0.008536 6.721e-22 35339 TRUE reported
textfile 2 1 TRUE
7150 rs1707652 T C T C 0.0513 0.0081 0.3102 NA FALSE FALSE FALSE
GCST006061 0.0074 0.2701 537409 outcome TRUE reported 19506_6_GLYAT_GLYAT
GLYAT chr3 165761011 0.008722 4.063e-09 35339 TRUE reported
textfile 2 1 TRUE
7151 rs1355538 G A G A -0.0577 -0.0076 0.39847 NA FALSE FALSE FALSE
GCST006061 0.0072 0.2923 537409 outcome TRUE reported 12881_17_RDH16_RDH16
RDH16 chr3 165787389 0.008132 1.293e-12 35329 TRUE reported
textfile 2 1 TRUE
7152 rs429358 C T C T -0.1596 0.005 0.17407 NA FALSE FALSE FALSE
GCST006061 0.0103 0.6319 537409 outcome TRUE reported 12881_17_RDH16_RDH16
RDH16 chr19 44908684 0.010605 3.449e-51 35359 TRUE reported
textfile 2 1 TRUE
7153 rs704 A G A G 0.057 -0.0155 0.49189 NA FALSE FALSE FALSE GCST006061
0.0072 0.03144 537409 outcome TRUE reported 12881_17_RDH16_RDH16 RDH16

chr17 28367840 0.007994 1.003e-12 35351 TRUE reported textfile 2
1 TRUE
7154 rs704 A G A G -0.0992 -0.0155 0.49189 NA FALSE FALSE FALSE GCST006061
0.0072 0.03144 537409 outcome TRUE reported 11107_25_TENM3_TEN3 TENM3 chr17
28367840 0.007832 9.207e-37 35351 TRUE reported textfile 2 1 TRUE
7155 rs34931250 T C T C 0.1241 0.0028 0.08188 NA FALSE FALSE FALSE
GCST006061 0.0174 0.8738 537409 outcome TRUE reported 9321_400_NMB_NMB NMB
chr17 68883786 0.014691 2.978e-17 35372 TRUE reported textfile 2
1 TRUE
7156 rs77262773 T C T C 0.1877 0.014 0.02263 NA FALSE FALSE FALSE
GCST006061 0.0283 0.6205 537409 outcome TRUE reported 9321_400_NMB_NMB NMB
chr17 69253570 0.026619 1.772e-12 35371 TRUE reported textfile 2
1 TRUE
7157 rs1706435 G A G A 0.0517 0.0117 0.36981 NA FALSE FALSE FALSE
GCST006061 0.0072 0.1047 537409 outcome TRUE reported 18945_11_RGS1_RGS1
RGS1 chr3 165743688 0.008189 2.733e-10 35339 TRUE reported
textfile 2 1 TRUE
7158 rs6542680 T C T C -0.1473 -0.0012 0.18362 NA FALSE FALSE FALSE
GCST006061 0.0091 0.8935 537409 outcome TRUE reported 18945_11_RGS1_RGS1
RGS1 chr2 3592552 0.010214 3.769e-47 35332 TRUE reported textfile
2 1 TRUE
7159 rs1355538 G A G A -0.0689 -0.0076 0.39847 NA FALSE FALSE FALSE
GCST006061 0.0072 0.2923 537409 outcome TRUE reported 10761_5_TMED2_TMED2
TMED2 chr3 165787389 0.008042 1.06e-17 35326 TRUE reported
textfile 2 1 TRUE
7160 rs6542680 T C T C -0.0571 -0.0012 0.18362 NA FALSE FALSE FALSE
GCST006061 0.0091 0.8935 537409 outcome TRUE reported 8872_1_SEC61G_SC61G
SEC61G chr2 3592552 0.010145 1.82e-08 35332 TRUE reported textfile
2 1 TRUE
7161 rs1355538 G A G A -0.0451 -0.0076 0.39847 NA FALSE FALSE FALSE
GCST006061 0.0072 0.2923 537409 outcome TRUE reported 8935_22_ABCC6_MRP6
ABCC6 chr3 165787389 0.008038 2.01e-08 35338 TRUE reported
textfile 2 1 TRUE
7162 rs6542680 T C T C -0.0576 -0.0012 0.18362 NA FALSE FALSE FALSE
GCST006061 0.0091 0.8935 537409 outcome TRUE reported 8935_22_ABCC6_MRP6
ABCC6 chr2 3592552 0.010198 1.621e-08 35332 TRUE reported textfile
2 1 TRUE
7163 rs1555405 A G A G -0.0487 -0.0047 0.30381 NA FALSE FALSE FALSE
GCST006061 0.0081 0.5592 537409 outcome TRUE reported
9874_28_CDKN2B_p15_INK4b CDKN2B chr14 100710432 0.008731 2.436e-08 35380
TRUE reported textfile 2 1 TRUE
7164 rs1707652 T C T C 0.0793 0.0081 0.3102 NA FALSE FALSE FALSE
GCST006061 0.0074 0.2701 537409 outcome TRUE reported
9874_28_CDKN2B_p15_INK4b CDKN2B chr3 165761011 0.008622 3.65e-20 35339
TRUE reported textfile 2 1 TRUE
7165 rs2232710 C T C T 0.3743 -0.0163 0.0257 NA FALSE FALSE FALSE
GCST006061 0.042 0.6983 537409 outcome TRUE reported
9874_28_CDKN2B_p15_INK4b CDKN2B chr14 94284149 0.024856 3.037e-51 35372
TRUE reported textfile 2 1 TRUE
7166 rs111696008 A G A G -0.1681 -0.0259 0.02443 NA FALSE FALSE FALSE
GCST006061 0.0401 0.519 537409 outcome TRUE reported 17820_170_MLF1_MLF1
MLF1 chr2 216622986 0.026726 3.179e-10 35359 TRUE reported
textfile 2 1 TRUE
7167 rs117827303 T C T C 0.1158 -0.0047 0.03794 NA FALSE FALSE FALSE
GCST006061 0.0216 0.8283 537409 outcome TRUE reported 17820_170_MLF1_MLF1
MLF1 chr12 7123080 0.020698 2.21e-08 35391 TRUE reported textfile
2 1 TRUE
7168 rs12146727 A G A G -0.1459 -0.0037 0.11875 NA FALSE FALSE FALSE
GCST006061 0.0105 0.7269 537409 outcome TRUE reported 17820_170_MLF1_MLF1
MLF1 chr12 7063032 0.012638 7.872e-31 35390 TRUE reported textfile
2 1 TRUE
7169 rs17164545 C T C T 0.0889 -0.0132 0.10827 NA FALSE FALSE FALSE
GCST006061 0.0117 0.258 537409 outcome TRUE reported 17820_170_MLF1_MLF1
MLF1 chr7 88818843 0.013138 1.317e-11 35349 TRUE reported
textfile 2 1 TRUE
7170 rs1801701 T C T C 0.1096 0.0099 0.12795 NA FALSE FALSE FALSE

GCST006061 0.0129 0.443 537409 outcome TRUE reported 17820_170_MLF1_MLF1 MLF1 chr2 21005955 0.011922 3.81e-20 35373 TRUE reported textfile 2 1 TRUE
7171 rs62290472 A G A G 0.0892 0.011 0.08266 NA FALSE FALSE FALSE GCST006061 0.0106 0.3013 537409 outcome TRUE reported 17820_170_MLF1_MLF1 MLF1 chr3 162193597 0.014722 1.37e-09 35340 TRUE reported textfile 2 1 TRUE
7172 rs6445035 A G A G 0.1585 0.0154 0.1904 NA FALSE FALSE FALSE GCST006061 0.0089 0.08256 537409 outcome TRUE reported 17820_170_MLF1_MLF1 MLF1 chr3 165762312 0.010206 2.153e-54 35339 TRUE reported textfile 2 1 TRUE
7173 rs7412 T C T C -0.0947 -0.0027 0.05941 NA FALSE FALSE FALSE GCST006061 0.0138 0.8459 537409 outcome TRUE reported 17820_170_MLF1_MLF1 MLF1 chr19 44908822 0.016997 2.523e-08 35367 TRUE reported textfile 2 1 TRUE
7174 rs76342676 T C T C 0.2562 -0.0765 0.02104 NA FALSE FALSE FALSE GCST006061 0.038 0.04431 537409 outcome TRUE reported 17820_170_MLF1_MLF1 MLF1 chr7 94660581 0.028951 8.794e-19 35352 TRUE reported textfile 2 1 TRUE
7175 rs854547 A G A G -0.2621 0 0.40137 NA FALSE FALSE FALSE GCST006061 0.0073 0.9958 537409 outcome TRUE reported 17820_170_MLF1_MLF1 MLF1 chr7 95294544 0.008229 1e-200 35349 TRUE reported textfile 2 1 TRUE
7176 rs11569415 A G A G -0.0587 0.0011 0.24276 NA FALSE FALSE FALSE GCST006061 0.0097 0.9129 537409 outcome TRUE reported 6315_58_PLBD1_PLBL1 PLBD1 chr19 6716268 0.00924 2.113e-10 35372 TRUE reported textfile 2 1 TRUE
7177 rs1474868 T C T C -0.051 0.01 0.45394 NA FALSE FALSE FALSE GCST006061 0.0071 0.1619 537409 outcome TRUE reported 6315_58_PLBD1_PLBL1 PLBD1 chr1 11984107 0.007972 1.577e-10 35387 TRUE reported textfile 2 1 TRUE
7178 rs1707652 T C T C 0.0468 0.0081 0.3102 NA FALSE FALSE FALSE GCST006061 0.0074 0.2701 537409 outcome TRUE reported 11406_82_ACAD8_ACAD8 ACAD8 chr3 165761011 0.008531 4.113e-08 35330 TRUE reported textfile 2 1 TRUE
7179 rs1355538 G A G A -0.059 -0.0076 0.39847 NA FALSE FALSE FALSE GCST006061 0.0072 0.2923 537409 outcome TRUE reported 18179_56_SMS_SPSY SMS chr3 165787389 0.008069 2.637e-13 35338 TRUE reported textfile 2 1 TRUE
7180 rs62467349 T C T C 0.0663 -0.0056 0.15309 NA FALSE FALSE FALSE GCST006061 0.0108 0.6067 537409 outcome TRUE reported 18179_56_SMS_SPSY SMS chr7 95317483 0.011009 1.718e-09 35349 TRUE reported textfile 2 1 TRUE
7181 rs6542680 T C T C -0.184 -0.0012 0.18362 NA FALSE FALSE FALSE GCST006061 0.0091 0.8935 537409 outcome TRUE reported 5534_49_TNFRSF10B_TRAIL_R2 TNFRSF10B chr2 3592552 0.010261 6.677e-72 35332 TRUE reported textfile 2 1 TRUE
7182 rs1355538 G A G A -0.0751 -0.0076 0.39847 NA FALSE FALSE FALSE GCST006061 0.0072 0.2923 537409 outcome TRUE reported 10842_7_GCNT4_GCNT4 GCNT4 chr3 165787389 0.008119 2.243e-20 35326 TRUE reported textfile 2 1 TRUE
7183 rs1555405 A G A G -0.0479 -0.0047 0.30381 NA FALSE FALSE FALSE GCST006061 0.0081 0.5592 537409 outcome TRUE reported 10842_7_GCNT4_GCNT4 GCNT4 chr14 100710432 0.008696 3.621e-08 35368 TRUE reported textfile 2 1 TRUE
7184 rs6542680 T C T C -0.0703 -0.0012 0.18362 NA FALSE FALSE FALSE GCST006061 0.0091 0.8935 537409 outcome TRUE reported 15319_226_CCNA1_CCNA1 CCNA1 chr2 3592552 0.010195 5.358e-12 35332 TRUE reported textfile 2 1 TRUE
7185 rs6542680 T C T C -0.059 -0.0012 0.18362 NA FALSE FALSE FALSE GCST006061 0.0091 0.8935 537409 outcome TRUE reported 9027_10_NLGN2_NLGN2 NLGN2 chr2 3592552 0.010172 6.614e-09 35332 TRUE reported textfile 2 1 TRUE
7186 rs1706435 G A G A 0.0472 0.0117 0.36981 NA FALSE FALSE FALSE GCST006061 0.0072 0.1047 537409 outcome TRUE reported 9574_11_BIN1_BIN1 BIN1 chr3 165743688 0.008239 1.013e-08 35339 TRUE reported textfile 2 1 TRUE
7187 rs2404176 A G A G 0.0454 -0.0055 0.47375 NA FALSE FALSE FALSE

GCST006061 0.0072 0.4447 537409 outcome TRUE reported 9574_11_BIN1_BIN1
BIN1 chr2 127027827 0.007976 1.255e-08 35369 TRUE reported
textfile 2 1 TRUE
7188 rs35103166 C T C T 0.1306 0.0126 0.34953 NA FALSE FALSE FALSE
GCST006061 0.0074 0.08924 537409 outcome TRUE reported 9574_11_BIN1_BIN1
BIN1 chr2 127124606 0.008389 1.197e-54 35368 TRUE reported
textfile 2 1 TRUE
7189 rs1354034 C T C T 0.0871 0.0088 0.38119 NA FALSE FALSE FALSE
GCST006061 0.0073 0.2248 537409 outcome TRUE reported 2524_56_HMGB1_HMG_1
HMGB1 chr3 56815721 0.008174 1.65e-26 35356 TRUE reported
textfile 2 1 TRUE
7190 rs1707652 T C T C 0.0734 0.0081 0.3102 NA FALSE FALSE FALSE
GCST006061 0.0074 0.2701 537409 outcome TRUE reported 2524_56_HMGB1_HMG_1
HMGB1 chr3 165761011 0.008572 1.103e-17 35339 TRUE reported
textfile 2 1 TRUE
7191 rs35209896 A C A C 0.0816 0.0267 0.09263 NA FALSE FALSE FALSE
GCST006061 0.0355 0.4521 537409 outcome TRUE reported 2524_56_HMGB1_HMG_1
HMGB1 chr6 32519009 0.013582 1.877e-09 35439 TRUE reported
textfile 2 1 TRUE
7192 rs12478841 G A G A 0.0648 0.0049 0.21569 NA FALSE FALSE FALSE
GCST006061 0.008 0.5413 537409 outcome TRUE reported 10990_21_LRRK2_LRRK2
LRRK2 chr2 27588855 0.009677 2.133e-11 35364 TRUE reported
textfile 2 1 TRUE
7193 rs1291921 G A G A 0.0503 -0.0157 0.2763 NA FALSE FALSE FALSE
GCST006061 0.0081 0.05326 537409 outcome TRUE reported 10990_21_LRRK2_LRRK2
LRRK2 chr3 136317384 0.009015 2.414e-08 35352 TRUE reported
textfile 2 1 TRUE
7194 rs6445035 A G A G 0.1146 0.0154 0.1904 NA FALSE FALSE FALSE
GCST006061 0.0089 0.08256 537409 outcome TRUE reported 10990_21_LRRK2_LRRK2
LRRK2 chr3 165762312 0.010209 3.061e-29 35330 TRUE reported
textfile 2 1 TRUE
7195 rs704 A G A G 0.0964 -0.0155 0.49189 NA FALSE FALSE FALSE GCST006061
0.0072 0.03144 537409 outcome TRUE reported 10990_21_LRRK2_LRRK2 LRRK2
chr17 28367840 0.008036 3.727e-33 35351 TRUE reported textfile 2
1 TRUE
7196 rs71352239 T C T C 0.0596 -0.0041 0.30263 NA FALSE FALSE FALSE
GCST006061 0.0087 0.6372 537409 outcome TRUE reported 10990_21_LRRK2_LRRK2
LRRK2 chr19 44926286 0.008768 1.067e-11 35359 TRUE reported
textfile 2 1 TRUE
7197 rs1706435 G A G A 0.0587 0.0117 0.36981 NA FALSE FALSE FALSE
GCST006061 0.0072 0.1047 537409 outcome TRUE reported 13509_5_SCAMP5_SCAM5
SCAMP5 chr3 165743688 0.008128 5.125e-13 35333 TRUE reported
textfile 2 1 TRUE
7198 rs1619994 G A G A 0.0886 0.0168 0.2807 NA FALSE FALSE FALSE
GCST006061 0.0078 0.03057 537409 outcome TRUE reported 13078_3_CETN2_CETN2
CETN2 chr3 165744047 0.00891 2.673e-23 35330 TRUE reported textfile
2 1 TRUE
7199 rs6796 C T C T 0.0521 0.0015 0.23682 NA FALSE FALSE FALSE GCST006061
0.0081 0.857 537409 outcome TRUE reported 8394_56_RNASE2_RNase_2 RNASE2
chr7 6462736 0.009236 1.692e-08 35364 TRUE reported textfile 2 1
TRUE
7200 rs1707652 T C T C 0.0754 0.0081 0.3102 NA FALSE FALSE FALSE
GCST006061 0.0074 0.2701 537409 outcome TRUE reported 13512_28_HNRNPR_HNRPR
HNRNPR chr3 165761011 0.008494 6.859e-19 35333 TRUE reported
textfile 2 1 TRUE
7201 rs1707652 T C T C 0.078 0.0081 0.3102 NA FALSE FALSE FALSE
GCST006061 0.0074 0.2701 537409 outcome TRUE reported 6360_7_DEFB128_DB128
DEFB128 chr3 165761011 0.008534 6.276e-20 35339 TRUE reported
textfile 2 1 TRUE
7202 rs11856676 T C T C -0.1011 -0.0143 0.44644 NA FALSE FALSE FALSE
GCST006061 0.0072 0.04638 537409 outcome TRUE reported 9504_19_RAB27A_RB27A
RAB27A chr15 80530206 0.008031 2.459e-36 35356 TRUE reported
textfile 2 1 TRUE
7203 rs8039725 G A G A 0.0677 0.0018 0.18034 NA FALSE FALSE FALSE
GCST006061 0.0092 0.8483 537409 outcome TRUE reported 9504_19_RAB27A_RB27A
RAB27A chr15 80582585 0.010351 6.137e-11 35356 TRUE reported

textfile 2 1 TRUE
7204 rs115886838 T G T G 0.1971 -0.0885 0.016 NA FALSE FALSE FALSE
GCST006061 0.0479 0.06426 537409 outcome TRUE reported 8913_22_SIGLEC11_SIG11
SIGLEC11 chr1 170278470 0.032523 1.359e-09 35361 TRUE reported
textfile 2 1 TRUE
7205 rs12127364 A C A C 0.2631 -0.006 0.3095 NA FALSE FALSE FALSE
GCST006061 0.0081 0.4569 537409 outcome TRUE reported 8913_22_SIGLEC11_SIG11
SIGLEC11 chr1 169499904 0.008442 1e-200 35363 TRUE reported
textfile 2 1 TRUE
7206 rs1355538 G A G A -0.0489 -0.0076 0.39847 NA FALSE FALSE FALSE
GCST006061 0.0072 0.2923 537409 outcome TRUE reported 8913_22_SIGLEC11_SIG11
SIGLEC11 chr3 165787389 0.008041 1.19e-09 35338 TRUE reported
textfile 2 1 TRUE
7207 rs6542680 T C T C -0.0619 -0.0012 0.18362 NA FALSE FALSE FALSE
GCST006061 0.0091 0.8935 537409 outcome TRUE reported 8913_22_SIGLEC11_SIG11
SIGLEC11 chr2 3592552 0.010191 1.25e-09 35332 TRUE reported
textfile 2 1 TRUE
7208 rs11645522 A G A G 0.058 -0.012 0.19819 NA FALSE FALSE FALSE
GCST006061 0.0099 0.2272 537409 outcome TRUE reported
11287_14_CYB5A_Cytochrome_b5 CYB5A chr16 80469448 0.00991 4.83e-09 35370
TRUE reported textfile 2 1 TRUE
7209 rs17806420 T C T C -0.1014 -0.0109 0.19046 NA FALSE FALSE FALSE
GCST006061 0.0098 0.2643 537409 outcome TRUE reported
11287_14_CYB5A_Cytochrome_b5 CYB5A chr18 74316132 0.01007 7.542e-24 35369
TRUE reported textfile 2 1 TRUE
7210 rs7669911 T C T C 0.0607 -0.0023 0.16277 NA FALSE FALSE FALSE
GCST006061 0.0091 0.8029 537409 outcome TRUE reported
11287_14_CYB5A_Cytochrome_b5 CYB5A chr4 145868333 0.010646 1.186e-08
35351 TRUE reported textfile 2 1 TRUE
7211 rs1706435 G A G A 0.0775 0.0117 0.36981 NA FALSE FALSE FALSE
GCST006061 0.0072 0.1047 537409 outcome TRUE reported 14132_21_HHLA2_HHLA2
HHLA2 chr3 165743688 0.008177 2.583e-21 35339 TRUE reported
textfile 2 1 TRUE
7212 rs4541868 A C A C -0.0535 0.0211 0.21915 NA FALSE FALSE FALSE
GCST006061 0.0082 0.01029 537409 outcome TRUE reported 14132_21_HHLA2_HHLA2
HHLA2 chr8 105578477 0.009576 2.312e-08 35364 TRUE reported
textfile 2 1 TRUE
7213 rs1707652 T C T C 0.0747 0.0081 0.3102 NA FALSE FALSE FALSE
GCST006061 0.0074 0.2701 537409 outcome TRUE reported 9028_5_MTPAP_PAPD1
MTPAP chr3 165761011 0.008603 3.845e-18 35339 TRUE reported
textfile 2 1 TRUE
7214 rs704 A G A G 0.0793 -0.0155 0.49189 NA FALSE FALSE FALSE GCST006061
0.0072 0.03144 537409 outcome TRUE reported 9028_5_MTPAP_PAPD1 MTPAP chr17
28367840 0.007988 3.159e-23 35360 TRUE reported textfile 2 1 TRUE
7215 rs1706435 G A G A 0.0627 0.0117 0.36981 NA FALSE FALSE FALSE
GCST006061 0.0072 0.1047 537409 outcome TRUE reported 7738_299_STX2_STX2
STX2 chr3 165743688 0.008254 3.041e-14 35339 TRUE reported
textfile 2 1 TRUE
7216 rs2104066 T C T C 0.0754 -0.0072 0.41997 NA FALSE FALSE FALSE
GCST006061 0.0073 0.3259 537409 outcome TRUE reported 7738_299_STX2_STX2
STX2 chr14 94458975 0.008188 3.312e-20 35372 TRUE reported
textfile 2 1 TRUE
7217 rs72704666 G A G A 0.2458 0.0397 0.02478 NA FALSE FALSE FALSE
GCST006061 0.029 0.1714 537409 outcome TRUE reported 7738_299_STX2_STX2
STX2 chr14 94968526 0.026333 1.015e-20 35367 TRUE reported
textfile 2 1 TRUE
7218 rs12403425 A C A C 0.0491 -0.0064 0.2662 NA FALSE FALSE FALSE
GCST006061 0.009 0.4754 537409 outcome TRUE reported 11228_37_RIC3_RIC3
RIC3 chr1 197845693 0.008986 4.658e-08 35328 TRUE reported
textfile 2 1 TRUE
7219 rs1355538 G A G A -0.0785 -0.0076 0.39847 NA FALSE FALSE FALSE
GCST006061 0.0072 0.2923 537409 outcome TRUE reported 11228_37_RIC3_RIC3
RIC3 chr3 165787389 0.008109 3.634e-22 35329 TRUE reported
textfile 2 1 TRUE
7220 rs704 A G A G 0.0509 -0.0155 0.49189 NA FALSE FALSE FALSE GCST006061
0.0072 0.03144 537409 outcome TRUE reported 11228_37_RIC3_RIC3 RIC3 chr17

28367840 0.007959 1.601e-10 35351 TRUE reported textfile 2 1 TRUE
7221 rs941758 A C A C -0.0481 0.0022 0.28938 NA FALSE FALSE FALSE
GCST006061 0.0079 0.7754 537409 outcome TRUE reported 11228_37_RIC3_RIC3
RIC3 chr4 73853224 0.008735 3.664e-08 35339 TRUE reported
textfile 2 1 TRUE
7222 rs1707652 T C T C 0.0576 0.0081 0.3102 NA FALSE FALSE FALSE
GCST006061 0.0074 0.2701 537409 outcome TRUE reported 19491_11_HSD17B8_DHB8
HSD17B8 chr3 165761011 0.00865 2.76e-11 35339 TRUE reported textfile
2 1 TRUE
7223 rs6857 T C T C -0.0767 0.0052 0.19663 NA FALSE FALSE FALSE GCST006061
0.0098 0.5973 537409 outcome TRUE reported 19491_11_HSD17B8_DHB8 HSD17B8
chr19 44888997 0.010171 4.669e-14 35368 TRUE reported textfile 2
1 TRUE
7224 rs780093 C T C T -0.0518 -0.0074 0.34157 NA FALSE FALSE FALSE
GCST006061 0.0073 0.3116 537409 outcome TRUE reported 19491_11_HSD17B8_DHB8
HSD17B8 chr2 27519736 0.008479 1e-09 35373 TRUE reported textfile
2 1 TRUE
7225 rs8106813 G A G A 0.124 -0.0037 0.46477 NA FALSE FALSE FALSE
GCST006061 0.0086 0.672 537409 outcome TRUE reported 19491_11_HSD17B8_DHB8
HSD17B8 chr19 44928401 0.008074 3.098e-53 35368 TRUE reported
textfile 2 1 TRUE
7226 rs1355538 G A G A -0.0666 -0.0076 0.39847 NA FALSE FALSE FALSE
GCST006061 0.0072 0.2923 537409 outcome TRUE reported 18386_36_GLRX_GLRX1
GLRX chr3 165787389 0.008051 1.316e-16 35338 TRUE reported
textfile 2 1 TRUE
7227 rs6556885 A G A G -0.0845 0.0026 0.25611 NA FALSE FALSE FALSE
GCST006061 0.0086 0.7598 537409 outcome TRUE reported 18386_36_GLRX_GLRX1
GLRX chr5 95825737 0.00908 1.33e-20 35349 TRUE reported textfile
2 1 TRUE
7228 rs1707652 T C T C 0.0593 0.0081 0.3102 NA FALSE FALSE FALSE
GCST006061 0.0074 0.2701 537409 outcome TRUE reported 7789_182_PRDX4_PRDX4
PRDX4 chr3 165761011 0.008587 5.005e-12 35339 TRUE reported
textfile 2 1 TRUE
7229 rs6542680 T C T C -0.1255 -0.0012 0.18362 NA FALSE FALSE FALSE
GCST006061 0.0091 0.8935 537409 outcome TRUE reported 7789_182_PRDX4_PRDX4
PRDX4 chr2 3592552 0.010283 2.935e-34 35332 TRUE reported textfile
2 1 TRUE
7230 rs12478841 G A G A 0.0581 0.0049 0.21569 NA FALSE FALSE FALSE
GCST006061 0.008 0.5413 537409 outcome TRUE reported 11262_39_TRABD_TRABD
TRABD chr2 27588855 0.00954 1.127e-09 35364 TRUE reported textfile
2 1 TRUE
7231 rs1707652 T C T C 0.0698 0.0081 0.3102 NA FALSE FALSE FALSE
GCST006061 0.0074 0.2701 537409 outcome TRUE reported 11262_39_TRABD_TRABD
TRABD chr3 165761011 0.008527 2.696e-16 35330 TRUE reported
textfile 2 1 TRUE
7232 rs71352239 T C T C 0.0739 -0.0041 0.30263 NA FALSE FALSE FALSE
GCST006061 0.0087 0.6372 537409 outcome TRUE reported 11262_39_TRABD_TRABD
TRABD chr19 44926286 0.008626 1.062e-17 35359 TRUE reported
textfile 2 1 TRUE
7233 rs1707652 T C T C 0.0806 0.0081 0.3102 NA FALSE FALSE FALSE
GCST006061 0.0074 0.2701 537409 outcome TRUE reported 10078_5_BAG3_BAG3
BAG3 chr3 165761011 0.008509 2.744e-21 35327 TRUE reported
textfile 2 1 TRUE
7234 rs1619994 G A G A 0.0627 0.0168 0.2807 NA FALSE FALSE FALSE
GCST006061 0.0078 0.03057 537409 outcome TRUE reported 7851_30_ANKRD46_ANR46
ANKRD46 chr3 165744047 0.00877 8.74e-13 35339 TRUE reported textfile
2 1 TRUE
7235 rs6542680 T C T C -0.0589 -0.0012 0.18362 NA FALSE FALSE FALSE
GCST006061 0.0091 0.8935 537409 outcome TRUE reported 7890_68_DPP10_DPP10
DPP10 chr2 3592552 0.01025 9.131e-09 35332 TRUE reported textfile 2
1 TRUE
7236 rs144040165 T G T G 0.1187 -0.018 0.03711 NA FALSE FALSE FALSE
GCST006061 0.0372 0.6291 537409 outcome TRUE reported 19579_5_DEFA1_HNP_1
DEFA1 chr6 26079793 0.021113 1.885e-08 35376 TRUE reported
textfile 2 1 TRUE
7237 rs2686395 C T C T -0.0632 -0.0077 0.3101 NA FALSE FALSE FALSE

GCST006061 0.0074 0.2965 537409 outcome TRUE reported 19579_5_DEFA1_HNP_1
DEFA1 chr3 165767231 0.008611 2.148e-13 35339 TRUE reported
textfile 2 1 TRUE
7238 rs1354034 C T C T 0.0738 0.0088 0.38119 NA FALSE FALSE FALSE
GCST006061 0.0073 0.2248 537409 outcome TRUE reported 7765_15_ITM2A_ITM2A
ITM2A chr3 56815721 0.008223 2.83e-19 35356 TRUE reported
textfile 2 1 TRUE
7239 rs1707652 T C T C 0.0749 0.0081 0.3102 NA FALSE FALSE FALSE
GCST006061 0.0074 0.2701 537409 outcome TRUE reported 7765_15_ITM2A_ITM2A
ITM2A chr3 165761011 0.008614 3.463e-18 35339 TRUE reported
textfile 2 1 TRUE
7240 rs11752007 C T C T 0.0742 -0.0053 0.11286 NA FALSE FALSE FALSE
GCST006061 0.0116 0.651 537409 outcome TRUE reported 9391_60_PCSK1N_PCSK1
PCSK1N chr6 7242372 0.012449 2.518e-09 35374 TRUE reported textfile
2 1 TRUE
7241 rs118111537 A G A G 0.4217 -0.008 0.04519 NA FALSE FALSE FALSE
GCST006061 0.0269 0.7657 537409 outcome TRUE reported 9391_60_PCSK1N_PCSK1
PCSK1N chr11 65944156 0.01947 5.064e-104 35366 TRUE reported textfile
2 1 TRUE
7242 rs1355538 G A G A -0.0553 -0.0076 0.39847 NA FALSE FALSE FALSE
GCST006061 0.0072 0.2923 537409 outcome TRUE reported 9391_60_PCSK1N_PCSK1
PCSK1N chr3 165787389 0.008079 7.673e-12 35338 TRUE reported
textfile 2 1 TRUE
7243 rs140172352 C T C T 1.4518 0.06 0.01286 NA FALSE FALSE FALSE
GCST006061 0.0923 0.5153 537409 outcome TRUE reported 9391_60_PCSK1N_PCSK1
PCSK1N chr11 67210607 0.029694 1e-200 35361 TRUE reported textfile
2 1 TRUE
7244 rs141454471 G A G A 0.4382 0.0121 0.0424 NA FALSE FALSE FALSE
GCST006061 0.021 0.5646 537409 outcome TRUE reported 9391_60_PCSK1N_PCSK1
PCSK1N chr11 62320550 0.019603 1.121e-110 35364 TRUE reported
textfile 2 1 TRUE
7245 rs7108689 T G T G 0.6242 -0.0072 0.0169 NA FALSE FALSE FALSE
GCST006061 0.0307 0.8139 537409 outcome TRUE reported 9391_60_PCSK1N_PCSK1
PCSK1N chr11 61475269 0.032093 2.927e-84 35365 TRUE reported
textfile 2 1 TRUE
7246 rs72904397 C T C T 0.1953 -0.0313 0.01605 NA FALSE FALSE FALSE
GCST006061 0.0376 0.4047 537409 outcome TRUE reported 9391_60_PCSK1N_PCSK1
PCSK1N chr11 46083755 0.032891 2.89e-09 35376 TRUE reported
textfile 2 1 TRUE
7247 rs72922318 T C T C 0.8734 0.0307 0.02385 NA FALSE FALSE FALSE
GCST006061 0.0354 0.3858 537409 outcome TRUE reported 9391_60_PCSK1N_PCSK1
PCSK1N chr11 63148800 0.026627 1e-200 35362 TRUE reported textfile
2 1 TRUE
7248 rs78724679 C T C T 0.2422 -0.0013 0.01324 NA FALSE FALSE FALSE
GCST006061 0.0243 0.9582 537409 outcome TRUE reported 9391_60_PCSK1N_PCSK1
PCSK1N chr11 73020594 0.035368 7.488e-12 35370 TRUE reported
textfile 2 1 TRUE
7249 rs697357 G A G A 0.0504 0.0156 0.38884 NA FALSE FALSE FALSE
GCST006061 0.0072 0.03066 537409 outcome TRUE reported 11202_70_TBX5_TBX5
TBX5 chr3 165718484 0.008053 3.886e-10 35330 TRUE reported
textfile 2 1 TRUE
7250 rs7137828 T C T C -0.0449 0.0076 0.38049 NA FALSE FALSE FALSE
GCST006061 0.0075 0.3144 537409 outcome TRUE reported 11202_70_TBX5_TBX5
TBX5 chr12 111494996 0.008118 3.188e-08 35362 TRUE reported
textfile 2 1 TRUE
7251 rs1706435 G A G A 0.0496 0.0117 0.36981 NA FALSE FALSE FALSE
GCST006061 0.0072 0.1047 537409 outcome TRUE reported 9767_22_RIPPLY1_RIPP1
RIPPLY1 chr3 165743688 0.00813 1.056e-09 35339 TRUE reported textfile
2 1 TRUE
7252 rs6445035 A G A G 0.1191 0.0154 0.1904 NA FALSE FALSE FALSE
GCST006061 0.0089 0.08256 537409 outcome TRUE reported 18308_30_STXBP6_STXB6
STXBP6 chr3 165762312 0.010083 3.386e-32 35339 TRUE reported
textfile 2 1 TRUE
7253 rs6736362 T C T C -0.2429 0.0027 0.46386 NA FALSE FALSE FALSE
GCST006061 0.0072 0.7057 537409 outcome TRUE reported 18308_30_STXBP6_STXB6
STXBP6 chr2 218250385 0.007914 1e-200 35368 TRUE reported textfile

2 1 TRUE
7254 rs71352239 T C T C 0.1049 -0.0041 0.30263 NA FALSE FALSE FALSE
GCST006061 0.0087 0.6372 537409 outcome TRUE reported 18308_30_STXBP6_STXB6
STXBP6 chr19 44926286 0.008667 1.008e-33 35368 TRUE reported
textfile 2 1 TRUE
7255 rs2686395 C T C T -0.0694 -0.0077 0.3101 NA FALSE FALSE FALSE
GCST006061 0.0074 0.2965 537409 outcome TRUE reported 8067_21_KCNE3_KCNE3
KCNE3 chr3 165767231 0.008413 1.589e-16 35339 TRUE reported
textfile 2 1 TRUE
7256 rs1291921 G A G A 0.0499 -0.0157 0.2763 NA FALSE FALSE FALSE
GCST006061 0.0081 0.05326 537409 outcome TRUE reported 17742_2_RRAS_RRAS
RRAS chr3 136317384 0.008907 2.116e-08 35361 TRUE reported
textfile 2 1 TRUE
7257 rs4955716 C T C T 0.0476 0.003 0.32947 NA FALSE FALSE FALSE
GCST006061 0.0076 0.6983 537409 outcome TRUE reported 17742_2_RRAS_RRAS
RRAS chr3 167239055 0.00844 1.705e-08 35354 TRUE reported textfile
2 1 TRUE
7258 rs211161 G T G T 0.1785 -0.0176 0.01526 NA FALSE FALSE FALSE
GCST006061 0.0295 0.5516 537409 outcome TRUE reported 5822_22_GDNF_GDNF
GDNF chr6 96614917 0.031397 1.306e-08 35343 TRUE reported
textfile 2 1 TRUE
7259 rs12478841 G A G A 0.0638 0.0049 0.21569 NA FALSE FALSE FALSE
GCST006061 0.008 0.5413 537409 outcome TRUE reported
17505_125_NAALADL1_NALDL NAALADL1 chr2 27588855 0.009755 6.155e-11
35373 TRUE reported textfile 2 1 TRUE
7260 rs1707652 T C T C 0.119 0.0081 0.3102 NA FALSE FALSE FALSE
GCST006061 0.0074 0.2701 537409 outcome TRUE reported
17505_125_NAALADL1_NALDL NAALADL1 chr3 165761011 0.008725 2.349e-42
35339 TRUE reported textfile 2 1 TRUE
7261 rs429358 C T C T -0.1005 0.005 0.17407 NA FALSE FALSE FALSE
GCST006061 0.0103 0.6319 537409 outcome TRUE reported
17505_125_NAALADL1_NALDL NAALADL1 chr19 44908684 0.010793 1.26e-20
35368 TRUE reported textfile 2 1 TRUE
7262 rs704 A G A G 0.0777 -0.0155 0.49189 NA FALSE FALSE FALSE GCST006061
0.0072 0.03144 537409 outcome TRUE reported 17505_125_NAALADL1_NALDL
NAALADL1 chr17 28367840 0.008105 9.136e-22 35360 TRUE reported
textfile 2 1 TRUE
7263 rs76004643 T G T G 0.0522 -0.0069 0.2662 NA FALSE FALSE FALSE
GCST006061 0.009 0.4422 537409 outcome TRUE reported
17505_125_NAALADL1_NALDL NAALADL1 chr1 197847653 0.009162 1.216e-08
35337 TRUE reported textfile 2 1 TRUE
7264 rs111696008 A G A G -0.1691 -0.0259 0.02443 NA FALSE FALSE FALSE
GCST006061 0.0401 0.519 537409 outcome TRUE reported 12041_33_HSPA1L_HS71L
HSPA1L chr2 216622986 0.0269 3.251e-10 35350 TRUE reported textfile
2 1 TRUE
7265 rs144704969 T C T C 0.1071 -0.0238 0.04536 NA FALSE FALSE FALSE
GCST006061 0.0154 0.1235 537409 outcome TRUE reported 12041_33_HSPA1L_HS71L
HSPA1L chr1 196158214 0.019352 3.124e-08 35328 TRUE reported
textfile 2 1 TRUE
7266 rs4955716 C T C T 0.0536 0.003 0.32947 NA FALSE FALSE FALSE
GCST006061 0.0076 0.6983 537409 outcome TRUE reported 12041_33_HSPA1L_HS71L
HSPA1L chr3 167239055 0.008611 4.825e-10 35345 TRUE reported
textfile 2 1 TRUE
7267 rs704 A G A G 0.1489 -0.0155 0.49189 NA FALSE FALSE FALSE GCST006061
0.0072 0.03144 537409 outcome TRUE reported 12041_33_HSPA1L_HS71L HSPA1L
chr17 28367840 0.008081 7.991e-76 35351 TRUE reported textfile 2
1 TRUE
7268 rs12478841 G A G A 0.0606 0.0049 0.21569 NA FALSE FALSE FALSE
GCST006061 0.008 0.5413 537409 outcome TRUE reported
12618_50_AKR1C1_Aldo_keto_reductase_1C1 AKR1C1 chr2 27588855 0.009528
2.013e-10 35364 TRUE reported textfile 2 1 TRUE
7269 rs1619994 G A G A 0.0599 0.0168 0.2807 NA FALSE FALSE FALSE
GCST006061 0.0078 0.03057 537409 outcome TRUE reported
12618_50_AKR1C1_Aldo_keto_reductase_1C1 AKR1C1 chr3 165744047 0.0088 9.979e-12
35330 TRUE reported textfile 2 1 TRUE
7270 rs11569415 A G A G -0.0609 0.0011 0.24276 NA FALSE FALSE FALSE

GCST006061 0.0097 0.9129 537409 outcome TRUE reported 5726_49_SPAG11A_SG11A SPAG11A chr19 6716268 0.009227 4.112e-11 35372 TRUE reported textfile 2 1 TRUE
7271 rs1451915 A C A C 0.0722 -0.0107 0.13641 NA FALSE FALSE FALSE GCST006061 0.0124 0.3895 537409 outcome TRUE reported 5726_49_SPAG11A_SG11A SPAG11A chr1 196058002 0.011532 3.832e-10 35335 TRUE reported textfile 2 1 TRUE
7272 rs1707652 T C T C 0.0935 0.0081 0.3102 NA FALSE FALSE FALSE GCST006061 0.0074 0.2701 537409 outcome TRUE reported 5726_49_SPAG11A_SG11A SPAG11A chr3 165761011 0.008565 9.665e-28 35339 TRUE reported textfile 2 1 TRUE
7273 rs35209896 A C A C 0.0925 0.0267 0.09263 NA FALSE FALSE FALSE GCST006061 0.0355 0.4521 537409 outcome TRUE reported 5726_49_SPAG11A_SG11A SPAG11A chr6 32519009 0.013591 1.003e-11 35439 TRUE reported textfile 2 1 TRUE
7274 rs1355538 G A G A -0.0555 -0.0076 0.39847 NA FALSE FALSE FALSE GCST006061 0.0072 0.2923 537409 outcome TRUE reported 16754_40_CASK_CSKP CASK chr3 165787389 0.007966 3.23e-12 35338 TRUE reported textfile 2 1 TRUE
7275 rs12378445 C T C T -0.052 -7.00E-04 0.27922 NA FALSE FALSE FALSE GCST006061 0.0081 0.9331 537409 outcome TRUE reported 13465_5_RCAN1_CCP1 RCAN1 chr9 114067500 0.009122 1.195e-08 35356 TRUE reported textfile 2 1 TRUE
7276 rs1707652 T C T C 0.0896 0.0081 0.3102 NA FALSE FALSE FALSE GCST006061 0.0074 0.2701 537409 outcome TRUE reported 13465_5_RCAN1_CCP1 RCAN1 chr3 165761011 0.008772 1.714e-24 35333 TRUE reported textfile 2 1 TRUE
7277 rs41313005 C T C T 0.2176 -0.0482 0.01828 NA FALSE FALSE FALSE GCST006061 0.0321 0.1334 537409 outcome TRUE reported 13465_5_RCAN1_CCP1 RCAN1 chr21 34514913 0.03009 4.769e-13 35373 TRUE reported textfile 2 1 TRUE
7278 rs72847562 G T G T -0.1083 -0.0406 0.09317 NA FALSE FALSE FALSE GCST006061 0.0168 0.01535 537409 outcome TRUE reported 13465_5_RCAN1_CCP1 RCAN1 chr2 88878601 0.014025 1.147e-14 35343 TRUE reported textfile 2 1 TRUE
7279 rs1354034 C T C T 0.0568 0.0088 0.38119 NA FALSE FALSE FALSE GCST006061 0.0073 0.2248 537409 outcome TRUE reported 12653_13_CSNK1G2_CSNK1G2 CSNK1G2 chr3 56815721 0.008141 3.006e-12 35347 TRUE reported textfile 2 1 TRUE
7280 rs1355538 G A G A -0.0459 -0.0076 0.39847 NA FALSE FALSE FALSE GCST006061 0.0072 0.2923 537409 outcome TRUE reported 12653_13_CSNK1G2_CSNK1G2 CSNK1G2 chr3 165787389 0.008058 1.226e-08 35329 TRUE reported textfile 2 1 TRUE
7281 rs12292693 C A C A -0.1235 -0.0128 0.26371 NA FALSE FALSE FALSE GCST006061 0.0086 0.1354 537409 outcome TRUE reported 5009_11_MSN_Moesin MSN chr11 65169248 0.008947 2.405e-43 35363 TRUE reported textfile 2 1 TRUE
7282 rs1355538 G A G A -0.0544 -0.0076 0.39847 NA FALSE FALSE FALSE GCST006061 0.0072 0.2923 537409 outcome TRUE reported 5009_11_MSN_Moesin MSN chr3 165787389 0.008056 1.447e-11 35338 TRUE reported textfile 2 1 TRUE
7283 rs3811444 T C T C -0.0941 0.0103 0.34232 NA FALSE FALSE FALSE GCST006061 0.0077 0.1812 537409 outcome TRUE reported 19257_11_CLTA_CLCA CLTA chr1 247876149 0.008387 3.26e-29 35156 TRUE reported textfile 2 1 TRUE
7284 rs1555405 A G A G -0.0511 -0.0047 0.30381 NA FALSE FALSE FALSE GCST006061 0.0081 0.5592 537409 outcome TRUE reported 10798_4_CD300E_CLM2 CD300E chr14 100710432 0.00861 2.934e-09 35368 TRUE reported textfile 2 1 TRUE
7285 rs1706435 G A G A 0.0697 0.0117 0.36981 NA FALSE FALSE FALSE GCST006061 0.0072 0.1047 537409 outcome TRUE reported 10798_4_CD300E_CLM2 CD300E chr3 165743688 0.00815 1.21e-17 35327 TRUE reported textfile 2 1 TRUE
7286 rs182482308 G A G A 0.1949 -0.0045 0.01861 NA FALSE FALSE FALSE GCST006061 0.0295 0.8784 537409 outcome TRUE reported 11872_9_PCDHGB1_PCDGD PCDHGB1 chr3 123083767 0.030234 1.145e-10 35359 TRUE reported

textfile 2 1 TRUE
7287 rs2232613 T C T C -0.1696 0.0151 0.0989 NA FALSE FALSE FALSE
GCST006061 0.0141 0.2834 537409 outcome TRUE reported 11872_9_PCDHGB1_PCDGD
PCDHGB1 chr20 38369011 0.013037 1.087e-38 35366 TRUE reported
textfile 2 1 TRUE
7288 rs6445035 A G A G 0.1163 0.0154 0.1904 NA FALSE FALSE FALSE
GCST006061 0.0089 0.08256 537409 outcome TRUE reported 11872_9_PCDHGB1_PCDGD
PCDHGB1 chr3 165762312 0.010054 6.037e-31 35330 TRUE reported
textfile 2 1 TRUE
7289 rs6542680 T C T C -0.0655 -0.0012 0.18362 NA FALSE FALSE FALSE
GCST006061 0.0091 0.8935 537409 outcome TRUE reported 8878_48_YTHDC1_YTDC1
YTHDC1 chr2 3592552 0.01024 1.593e-10 35332 TRUE reported textfile 2
1 TRUE
7290 rs1619994 G A G A 0.0537 0.0168 0.2807 NA FALSE FALSE FALSE
GCST006061 0.0078 0.03057 537409 outcome TRUE reported 12556_7_UBE2C_UBE2C
UBE2C chr3 165744047 0.008799 1.043e-09 35330 TRUE reported
textfile 2 1 TRUE
7291 rs36051332 A G A G -0.1375 0.0055 0.36616 NA FALSE FALSE FALSE
GCST006061 0.0073 0.4534 537409 outcome TRUE reported 12556_7_UBE2C_UBE2C
UBE2C chr20 45789372 0.008146 6.294e-64 35362 TRUE reported
textfile 2 1 TRUE
7292 rs1355538 G A G A -0.0644 -0.0076 0.39847 NA FALSE FALSE FALSE
GCST006061 0.0072 0.2923 537409 outcome TRUE reported 10048_7_CBFB_PEBB
CBFB chr3 165787389 0.008096 1.804e-15 35326 TRUE reported
textfile 2 1 TRUE
7293 rs16919431 G T G T 0.0528 0.0053 0.23714 NA FALSE FALSE FALSE
GCST006061 0.008 0.5042 537409 outcome TRUE reported 10048_7_CBFB_PEBB
CBFB chr10 19853771 0.009365 1.721e-08 35345 TRUE reported
textfile 2 1 TRUE
7294 rs7535409 G A G A -0.0629 -7.00E-04 0.33792 NA FALSE FALSE FALSE
GCST006061 0.0075 0.9311 537409 outcome TRUE reported 10048_7_CBFB_PEBB
CBFB chr1 169537003 0.008376 5.947e-14 35352 TRUE reported
textfile 2 1 TRUE
7295 rs1354034 C T C T 0.0457 0.0088 0.38119 NA FALSE FALSE FALSE
GCST006061 0.0073 0.2248 537409 outcome TRUE reported 2849_49_AIF1_AIF1
AIF1 chr3 56815721 0.008215 2.651e-08 35356 TRUE reported
textfile 2 1 TRUE
7296 rs2261033 G A G A -0.1252 0.0151 0.45141 NA FALSE FALSE FALSE
GCST006061 0.0074 0.04091 537409 outcome TRUE reported 2849_49_AIF1_AIF1
AIF1 chr6 31635814 0.008006 3.938e-55 35439 TRUE reported
textfile 2 1 TRUE
7297 rs4631576 T C T C 0.0558 -0.0052 0.35116 NA FALSE FALSE FALSE
GCST006061 0.0075 0.4837 537409 outcome TRUE reported 2849_49_AIF1_AIF1
AIF1 chr9 97942928 0.008322 2.015e-11 35345 TRUE reported
textfile 2 1 TRUE
7298 rs4639017 C T C T -0.0458 -0.0061 0.39283 NA FALSE FALSE FALSE
GCST006061 0.0073 0.4022 537409 outcome TRUE reported 8040_9_LEMD1_LEMD1
LEMD1 chr3 165833296 0.008056 1.304e-08 35339 TRUE reported
textfile 2 1 TRUE
7299 rs80182784 C T C T 0.2524 0.007 0.01542 NA FALSE FALSE FALSE
GCST006061 0.0318 0.8263 537409 outcome TRUE reported 8040_9_LEMD1_LEMD1
LEMD1 chr1 151130606 0.032674 1.121e-14 35362 TRUE reported
textfile 2 1 TRUE
7300 rs113370836 A G A G 0.2172 0.0199 0.02185 NA FALSE FALSE FALSE
GCST006061 0.0254 0.4344 537409 outcome TRUE reported 7753_21_HTATIP2_HTAI2
HTATIP2 chr14 64274445 0.028178 1.278e-14 35358 TRUE reported
textfile 2 1 TRUE
7301 rs1706435 G A G A 0.0559 0.0117 0.36981 NA FALSE FALSE FALSE
GCST006061 0.0072 0.1047 537409 outcome TRUE reported 7753_21_HTATIP2_HTAI2
HTATIP2 chr3 165743688 0.008285 1.512e-11 35339 TRUE reported
textfile 2 1 TRUE
7302 rs72719202 T C T C 0.3764 -0.0361 0.02601 NA FALSE FALSE FALSE
GCST006061 0.0232 0.1194 537409 outcome TRUE reported 7753_21_HTATIP2_HTAI2
HTATIP2 chr14 67510599 0.025978 1.419e-47 35365 TRUE reported
textfile 2 1 TRUE
7303 rs75141912 A C A C 0.1816 -0.0118 0.01801 NA FALSE FALSE FALSE

GCST006061 0.0291 0.6848 537409 outcome TRUE reported 7753_21_HTATIP2_HTAI2
HTATIP2 chr14 68654085 0.030609 2.977e-09 35358 TRUE reported
textfile 2 1 TRUE
7304 rs75475095 G A G A -0.1341 0.0087 0.06594 NA FALSE FALSE FALSE
GCST006061 0.0183 0.6355 537409 outcome TRUE reported 7753_21_HTATIP2_HTAI2
HTATIP2 chr14 65467015 0.01647 3.882e-16 35360 TRUE reported textfile
2 1 TRUE
7305 rs79836542 A G A G 0.1933 0.0344 0.0261 NA FALSE FALSE FALSE
GCST006061 0.0202 0.08813 537409 outcome TRUE reported 7753_21_HTATIP2_HTAI2
HTATIP2 chr14 64097847 0.025998 1.044e-13 35355 TRUE reported
textfile 2 1 TRUE
7306 rs1355538 G A G A -0.0615 -0.0076 0.39847 NA FALSE FALSE FALSE
GCST006061 0.0072 0.2923 537409 outcome TRUE reported
16074_12_GRAP2_GRB2_related_adapter_protein_2 GRAP2 chr3 165787389 0.008243
8.585e-14 35338 TRUE reported textfile 2 1 TRUE
7307 rs148328786 G A G A 0.1496 0.0085 0.04808 NA FALSE FALSE FALSE
GCST006061 0.0409 0.8356 537409 outcome TRUE reported
16074_12_GRAP2_GRB2_related_adapter_protein_2 GRAP2 chr22 39946989 0.018677
1.149e-15 35366 TRUE reported textfile 2 1 TRUE
7308 rs704 A G A G 0.0675 -0.0155 0.49189 NA FALSE FALSE FALSE GCST006061
0.0072 0.03144 537409 outcome TRUE reported
16074_12_GRAP2_GRB2_related_adapter_protein_2 GRAP2 chr17 28367840 0.008103
8.05e-17 35360 TRUE reported textfile 2 1 TRUE
7309 rs9971407 T C T C -0.0883 -0.0126 0.24858 NA FALSE FALSE FALSE
GCST006061 0.0086 0.1427 537409 outcome TRUE reported
16074_12_GRAP2_GRB2_related_adapter_protein_2 GRAP2 chr11 65130754 0.009364
4.114e-21 35363 TRUE reported textfile 2 1 TRUE
7310 rs28384482 T C T C -0.0746 0.0242 0.11777 NA FALSE FALSE FALSE
GCST006061 0.0118 0.04046 537409 outcome TRUE reported
13580_2_UAP1_Sperm_associated_antigen_2 UAP1 chr1 162561114 0.012179
9.051e-10 35352 TRUE reported textfile 2 1 TRUE
7311 rs12146727 A G A G -0.1862 -0.0037 0.11875 NA FALSE FALSE FALSE
GCST006061 0.0105 0.7269 537409 outcome TRUE reported 11320_29_CHFR_CHFR
CHFR chr12 7063032 0.012631 3.489e-49 35381 TRUE reported textfile
2 1 TRUE
7312 rs1355538 G A G A -0.0686 -0.0076 0.39847 NA FALSE FALSE FALSE
GCST006061 0.0072 0.2923 537409 outcome TRUE reported 11320_29_CHFR_CHFR
CHFR chr3 165787389 0.008196 5.753e-17 35329 TRUE reported
textfile 2 1 TRUE
7313 rs61984785 T C T C 0.0818 0.0074 0.09245 NA FALSE FALSE FALSE
GCST006061 0.0295 0.803 537409 outcome TRUE reported 11320_29_CHFR_CHFR
CHFR chr14 105668556 0.014258 9.624e-09 35089 TRUE reported
textfile 2 1 TRUE
7314 rs1707652 T C T C 0.0566 0.0081 0.3102 NA FALSE FALSE FALSE
GCST006061 0.0074 0.2701 537409 outcome TRUE reported 10445_20_APOM_ApoM
APOM chr3 165761011 0.008837 1.502e-10 35327 TRUE reported
textfile 2 1 TRUE
7315 rs1800961 T C T C -0.2149 -4.00E-04 0.04601 NA FALSE FALSE FALSE
GCST006061 0.0219 0.9854 537409 outcome TRUE reported 10445_20_APOM_ApoM
APOM chr20 44413724 0.019723 1.209e-27 35359 TRUE reported
textfile 2 1 TRUE
7316 rs3782735 A G A G -0.0483 -0.01 0.38274 NA FALSE FALSE FALSE
GCST006061 0.0076 0.1867 537409 outcome TRUE reported 10445_20_APOM_ApoM
APOM chr12 6775910 0.00846 1.135e-08 35376 TRUE reported textfile 2
1 TRUE
7317 rs72786786 A G A G 0.0762 0.0062 0.31073 NA FALSE FALSE FALSE
GCST006061 0.0081 0.4475 537409 outcome TRUE reported 10445_20_APOM_ApoM
APOM chr16 56951602 0.008864 8.243e-18 35357 TRUE reported
textfile 2 1 TRUE
7318 rs780093 C T C T -0.0578 -0.0074 0.34157 NA FALSE FALSE FALSE
GCST006061 0.0073 0.3116 537409 outcome TRUE reported 10445_20_APOM_ApoM
APOM chr2 27519736 0.008647 2.313e-11 35361 TRUE reported
textfile 2 1 TRUE
7319 rs6542680 T C T C -0.1246 -0.0012 0.18362 NA FALSE FALSE FALSE
GCST006061 0.0091 0.8935 537409 outcome TRUE reported 3083_71_EDA2R_XEDAR
EDA2R chr2 3592552 0.010153 1.282e-34 35332 TRUE reported textfile

2 1 TRUE
7320 rs1355538 G A G A -0.0783 -0.0076 0.39847 NA FALSE FALSE FALSE
GCST006061 0.0072 0.2923 537409 outcome TRUE reported 10064_12_RBBP9_RBBP9
RBBP9 chr3 165787389 0.008172 9.605e-22 35326 TRUE reported
textfile 2 1 TRUE
7321 rs1355538 G A G A -0.0692 -0.0076 0.39847 NA FALSE FALSE FALSE
GCST006061 0.0072 0.2923 537409 outcome TRUE reported 6411_58_DEFB135_DB135
DEFB135 chr3 165787389 0.008252 5.054e-17 35338 TRUE reported
textfile 2 1 TRUE
7322 rs1706435 G A G A 0.0482 0.0117 0.36981 NA FALSE FALSE FALSE
GCST006061 0.0072 0.1047 537409 outcome TRUE reported 18172_71_ASF1A_ASF1A
ASF1A chr3 165743688 0.008207 4.286e-09 35339 TRUE reported
textfile 2 1 TRUE
7323 rs185009887 A G A G -0.1403 -0.0595 0.02519 NA FALSE FALSE FALSE
GCST006061 0.0496 0.2302 537409 outcome TRUE reported 18172_71_ASF1A_ASF1A
ASF1A chr17 5848332 0.025733 4.977e-08 35374 TRUE reported textfile
2 1 TRUE
7324 rs6489859 G A G A -0.0442 0.0117 0.47634 NA FALSE FALSE FALSE
GCST006061 0.0072 0.1054 537409 outcome TRUE reported 18172_71_ASF1A_ASF1A
ASF1A chr12 112774128 0.007972 2.948e-08 35366 TRUE reported
textfile 2 1 TRUE
7325 rs1706435 G A G A 0.0713 0.0117 0.36981 NA FALSE FALSE FALSE
GCST006061 0.0072 0.1047 537409 outcome TRUE reported 7854_38_NCR3LG1_NR3L1
NCR3LG1 chr3 165743688 0.00814 1.965e-18 35339 TRUE reported textfile
2 1 TRUE
7326 rs58068569 G A G A -0.0536 -0.0152 0.20781 NA FALSE FALSE FALSE
GCST006061 0.0098 0.1209 537409 outcome TRUE reported 5623_11_CD300LF_CLM1
CD300LF chr12 3991507 0.009756 3.927e-08 35377 TRUE reported textfile
2 1 TRUE
7327 rs1355538 G A G A -0.06 -0.0076 0.39847 NA FALSE FALSE FALSE
GCST006061 0.0072 0.2923 537409 outcome TRUE reported 8748_45_CD74_HG2A
CD74 chr3 165787389 0.008025 7.601e-14 35338 TRUE reported
textfile 2 1 TRUE
7328 rs28645249 C T C T 0.0473 0.019 0.26162 NA FALSE FALSE FALSE
GCST006061 0.0115 0.09815 537409 outcome TRUE reported 8748_45_CD74_HG2A
CD74 chr6 32557458 0.008623 4.127e-08 35438 TRUE reported
textfile 2 1 TRUE
7329 rs1135816 G A G A -0.2296 -0.0048 0.37818 NA FALSE FALSE FALSE
GCST006061 0.0072 0.5052 537409 outcome TRUE reported 10809_14_KLRB1_KLRB1
KLRB1 chr12 9598073 0.008094 5.361e-177 35372 TRUE reported textfile
2 1 TRUE
7330 rs118021813 C T C T -0.0966 -0.0176 0.06111 NA FALSE FALSE FALSE
GCST006061 0.0186 0.3429 537409 outcome TRUE reported 10809_14_KLRB1_KLRB1
KLRB1 chr12 9889577 0.016561 5.447e-09 35373 TRUE reported textfile
2 1 TRUE
7331 rs139517772 C A C A -0.1543 -0.0055 0.0209 NA FALSE FALSE FALSE
GCST006061 0.033 0.8678 537409 outcome TRUE reported 10809_14_KLRB1_KLRB1
KLRB1 chr12 9125550 0.027023 1.13e-08 35375 TRUE reported textfile
2 1 TRUE
7332 rs713452 C T C T 0.0695 0.013 0.29838 NA FALSE FALSE FALSE
GCST006061 0.0077 0.09355 537409 outcome TRUE reported 10809_14_KLRB1_KLRB1
KLRB1 chr1 56920602 0.008553 4.448e-16 35357 TRUE reported
textfile 2 1 TRUE
7333 rs7862602 T G T G 0.1026 -3.00E-04 0.43654 NA FALSE FALSE FALSE
GCST006061 0.0074 0.9625 537409 outcome TRUE reported 10809_14_KLRB1_KLRB1
KLRB1 chr9 136946019 0.007935 3.067e-38 35248 TRUE reported
textfile 2 1 TRUE
7334 rs13319916 G A G A 0.0507 0.0109 0.38361 NA FALSE FALSE FALSE
GCST006061 0.0076 0.1493 537409 outcome TRUE reported 9068_17_JAKMIP3_JKIP3
JAKMIP3 chr3 54204031 0.008012 2.485e-10 35346 TRUE reported
textfile 2 1 TRUE
7335 rs216311 C T C T -0.0531 0.0064 0.41422 NA FALSE FALSE FALSE
GCST006061 0.0076 0.4034 537409 outcome TRUE reported 9068_17_JAKMIP3_JKIP3
JAKMIP3 chr12 6019277 0.007889 1.686e-11 35376 TRUE reported textfile
2 1 TRUE
7336 rs3774558 T C T C 0.0666 -0.0138 0.44899 NA FALSE FALSE FALSE

GCST006061 0.0076 0.07016 537409 outcome TRUE reported 9068_17_JAKMIP3_JKIP3
JAKMIP3 chr3 53741712 0.007825 1.714e-17 35349 TRUE reported
textfile 2 1 TRUE
7337 rs6802320 C T C T 0.2534 0.0043 0.37345 NA FALSE FALSE FALSE
GCST006061 0.0075 0.5634 537409 outcome TRUE reported 9068_17_JAKMIP3_JKIP3
JAKMIP3 chr3 52207819 0.007966 1e-200 35347 TRUE reported textfile
2 1 TRUE
7338 rs1354034 C T C T 0.0592 0.0088 0.38119 NA FALSE FALSE FALSE
GCST006061 0.0073 0.2248 537409 outcome TRUE reported 15669_7_BRAF_BRAF1
BRAF chr3 56815721 0.008204 5.343e-13 35356 TRUE reported
textfile 2 1 TRUE
7339 rs1619994 G A G A 0.051 0.0168 0.2807 NA FALSE FALSE FALSE
GCST006061 0.0078 0.03057 537409 outcome TRUE reported 15669_7_BRAF_BRAF1
BRAF chr3 165744047 0.008876 9.155e-09 35339 TRUE reported
textfile 2 1 TRUE
7340 rs4980320 C T C T 0.0453 0.0041 0.46986 NA FALSE FALSE FALSE
GCST006061 0.0076 0.5858 537409 outcome TRUE reported 15669_7_BRAF_BRAF1
BRAF chr11 198986 0.007992 1.442e-08 35352 TRUE reported textfile
2 1 TRUE
7341 rs704 A G A G 0.0591 -0.0155 0.49189 NA FALSE FALSE FALSE GCST006061
0.0072 0.03144 537409 outcome TRUE reported 15669_7_BRAF_BRAF1 BRAF chr17
28367840 0.007985 1.346e-13 35360 TRUE reported textfile 2 1 TRUE
7342 rs7332311 C T C T 0.0579 0.0048 0.20458 NA FALSE FALSE FALSE
GCST006061 0.0084 0.5671 537409 outcome TRUE reported 15669_7_BRAF_BRAF1
BRAF chr13 46099790 0.009966 6.258e-09 35361 TRUE reported
textfile 2 1 TRUE
7343 rs1354034 C T C T 0.045 0.0088 0.38119 NA FALSE FALSE FALSE
GCST006061 0.0073 0.2248 537409 outcome TRUE reported 18405_117_AP1S2_AP1S2
AP1S2 chr3 56815721 0.008119 2.984e-08 35356 TRUE reported
textfile 2 1 TRUE
7344 rs1619994 G A G A 0.0753 0.0168 0.2807 NA FALSE FALSE FALSE
GCST006061 0.0078 0.03057 537409 outcome TRUE reported 18405_117_AP1S2_AP1S2
AP1S2 chr3 165744047 0.008787 1.04e-17 35339 TRUE reported
textfile 2 1 TRUE
7345 rs147604653 A G A G 0.1507 0.0332 0.02654 NA FALSE FALSE FALSE
GCST006061 0.0429 0.4393 537409 outcome TRUE reported 11117_2_SPATA20_SPT20
SPATA20 chr17 49734100 0.025619 4.047e-09 35357 TRUE reported
textfile 2 1 TRUE
7346 rs17714829 C T C T 0.1255 -0.0047 0.0781 NA FALSE FALSE FALSE
GCST006061 0.0148 0.7497 537409 outcome TRUE reported 11117_2_SPATA20_SPT20
SPATA20 chr17 50375120 0.015247 1.852e-16 35357 TRUE reported
textfile 2 1 TRUE
7347 rs4666000 C T C T 0.0534 0.007 0.22594 NA FALSE FALSE FALSE
GCST006061 0.0078 0.3727 537409 outcome TRUE reported 11117_2_SPATA20_SPT20
SPATA20 chr2 27616502 0.009653 3.171e-08 35364 TRUE reported
textfile 2 1 TRUE
7348 rs77405995 A G A G 0.1222 0.0029 0.04892 NA FALSE FALSE FALSE
GCST006061 0.0226 0.8962 537409 outcome TRUE reported 11117_2_SPATA20_SPT20
SPATA20 chr17 50284012 0.018545 4.412e-11 35350 TRUE reported
textfile 2 1 TRUE
7349 rs9890200 C A C A -0.3204 0.0094 0.40963 NA FALSE FALSE FALSE
GCST006061 0.0076 0.2155 537409 outcome TRUE reported 11117_2_SPATA20_SPT20
SPATA20 chr17 50547162 0.007116 1e-200 35353 TRUE reported textfile
2 1 TRUE
7350 rs1707652 T C T C 0.0632 0.0081 0.3102 NA FALSE FALSE FALSE
GCST006061 0.0074 0.2701 537409 outcome TRUE reported 10035_6_DUSP4_DUS4
DUSP4 chr3 165761011 0.008547 1.421e-13 35327 TRUE reported
textfile 2 1 TRUE
7351 rs6542680 T C T C -0.0748 -0.0012 0.18362 NA FALSE FALSE FALSE
GCST006061 0.0091 0.8935 537409 outcome TRUE reported 10035_6_DUSP4_DUS4
DUSP4 chr2 3592552 0.010246 2.863e-13 35320 TRUE reported textfile
2 1 TRUE
7352 rs1706435 G A G A 0.063 0.0117 0.36981 NA FALSE FALSE FALSE
GCST006061 0.0072 0.1047 537409 outcome TRUE reported 13573_5_ARPC3_p21_ARC
ARPC3 chr3 165743688 0.008215 1.733e-14 35333 TRUE reported
textfile 2 1 TRUE

7353 rs1707652 T C T C 0.1069 0.0081 0.3102 NA FALSE FALSE FALSE GCST006061 0.0074 0.2701 537409 outcome TRUE reported 12957_62_UQCRB_UCR6 UQCRB chr3 165761011 0.008554 7.684e-36 35330 TRUE reported textfile 2 1 TRUE

7354 rs1354034 C T C T 0.0574 0.0088 0.38119 NA FALSE FALSE FALSE GCST006061 0.0073 0.2248 537409 outcome TRUE reported 11226_16_UBE3A_UBE3A UBE3A chr3 56815721 0.008301 4.684e-12 35347 TRUE reported textfile 2 1 TRUE

7355 rs1355538 G A G A -0.0453 -0.0076 0.39847 NA FALSE FALSE FALSE GCST006061 0.0072 0.2923 537409 outcome TRUE reported 11226_16_UBE3A_UBE3A UBE3A chr3 165787389 0.008224 3.627e-08 35329 TRUE reported textfile 2 1 TRUE

7356 rs4980320 C T C T 0.0516 0.0041 0.46986 NA FALSE FALSE FALSE GCST006061 0.0076 0.5858 537409 outcome TRUE reported 11226_16_UBE3A_UBE3A UBE3A chr11 198986 0.008094 1.826e-10 35343 TRUE reported textfile 2 1 TRUE

7357 rs34843303 C T C T -0.0872 -0.0345 0.10898 NA FALSE FALSE FALSE GCST006061 0.0119 0.003659 537409 outcome TRUE reported 15457_14_ANPEP_Aminopeptidase_N ANPEP chr15 78942128 0.012733 7.474e-12 35358 TRUE reported textfile 2 1 TRUE

7358 rs1707652 T C T C 0.0933 0.0081 0.3102 NA FALSE FALSE FALSE GCST006061 0.0074 0.2701 537409 outcome TRUE reported 15460_9_DPP4_CD26 DPP4 chr3 165761011 0.008533 7.917e-28 35339 TRUE reported textfile 2 1 TRUE

7359 rs1260326 C T C T -0.0659 -0.0069 0.34132 NA FALSE FALSE FALSE GCST006061 0.0073 0.3401 537409 outcome TRUE reported 15606_19_KRT19_Keratin_19 KRT19 chr2 27508073 0.008719 4.099e-14 35373 TRUE reported textfile 2 1 TRUE

7360 rs438811 T C T C -0.2758 -5.00E-04 0.23201 NA FALSE FALSE FALSE GCST006061 0.0088 0.9556 537409 outcome TRUE reported 15606_19_KRT19_Keratin_19 KRT19 chr19 44913484 0.009787 1.009e-174 35366 TRUE reported textfile 2 1 TRUE

7361 rs73940367 T C T C 0.0599 0.0022 0.24386 NA FALSE FALSE FALSE GCST006061 0.0084 0.7981 537409 outcome TRUE reported 15606_19_KRT19_Keratin_19 KRT19 chr19 45898685 0.009621 4.79e-10 35371 TRUE reported textfile 2 1 TRUE

7362 rs1355538 G A G A -0.0477 -0.0076 0.39847 NA FALSE FALSE FALSE GCST006061 0.0072 0.2923 537409 outcome TRUE reported 4304_18_LCORL_transcription_factor_MLR1__isoform_CRA_b LCORL chr3 165787389 0.008059 3.235e-09 35338 TRUE reported textfile 2 1 TRUE

7363 rs1355538 G A G A -0.0569 -0.0076 0.39847 NA FALSE FALSE FALSE GCST006061 0.0072 0.2923 537409 outcome TRUE reported 8749_194_CD1D_CD1D CD1D chr3 165787389 0.008031 1.387e-12 35338 TRUE reported textfile 2 1 TRUE

7364 rs16964821 C T C T 0.1157 0.0125 0.04905 NA FALSE FALSE FALSE GCST006061 0.0155 0.4204 537409 outcome TRUE reported 16302_11_BPIFA2_SPLC2 BPIFA2 chr17 29119140 0.019093 1.362e-09 35361 TRUE reported textfile 2 1 TRUE

7365 rs1707652 T C T C 0.0984 0.0081 0.3102 NA FALSE FALSE FALSE GCST006061 0.0074 0.2701 537409 outcome TRUE reported 16302_11_BPIFA2_SPLC2 BPIFA2 chr3 165761011 0.008813 6.012e-29 35339 TRUE reported textfile 2 1 TRUE

7366 rs28676336 G T G T 0.0509 0.0049 0.33317 NA FALSE FALSE FALSE GCST006061 0.0077 0.521 537409 outcome TRUE reported 16302_11_BPIFA2_SPLC2 BPIFA2 chr5 44281701 0.008633 3.717e-09 35361 TRUE reported textfile 2 1 TRUE

7367 rs3818222 A G A G -0.0768 -0.006 0.4925 NA FALSE FALSE FALSE GCST006061 0.0074 0.4159 537409 outcome TRUE reported 16302_11_BPIFA2_SPLC2 BPIFA2 chr20 33225117 0.008193 7.002e-21 35367 TRUE reported textfile 2 1 TRUE

7368 rs67086575 G A G A 0.0767 0.0024 0.12471 NA FALSE FALSE FALSE GCST006061 0.0103 0.8161 537409 outcome TRUE reported 16302_11_BPIFA2_SPLC2 BPIFA2 chr2 27463613 0.012433 6.862e-10 35374 TRUE reported textfile 2 1 TRUE

7369 rs704 A G A G 0.2457 -0.0155 0.49189 NA FALSE FALSE FALSE GCST006061 0.0072 0.03144 537409 outcome TRUE reported 16302_11_BPIFA2_SPLC2 BPIFA2

chr17 28367840 0.008137 2.591e-200 35360 TRUE reported textfile 2 1 TRUE

7370 rs71352239 T C T C 0.1128 -0.0041 0.30263 NA FALSE FALSE FALSE GCST006061 0.0087 0.6372 537409 outcome TRUE reported 16302_11_BPIFA2_SPLC2 BPIFA2 chr19 44926286 0.00897 2.876e-36 35368 TRUE reported textfile 2 1 TRUE

7371 rs7217335 A G A G 0.0591 -0.0193 0.19396 NA FALSE FALSE FALSE GCST006061 0.0093 0.0382 537409 outcome TRUE reported 16302_11_BPIFA2_SPLC2 BPIFA2 chr17 27771755 0.010408 1.361e-08 35358 TRUE reported textfile 2 1 TRUE

7372 rs1707652 T C T C 0.059 0.0081 0.3102 NA FALSE FALSE FALSE GCST006061 0.0074 0.2701 537409 outcome TRUE reported 14056_4_CGA_Glycoprotein_hormones_a_chain CGA chr3 165761011 0.008513 4.192e-12 35339 TRUE reported textfile 2 1 TRUE

7373 rs1706435 G A G A 0.0808 0.0117 0.36981 NA FALSE FALSE FALSE GCST006061 0.0072 0.1047 537409 outcome TRUE reported 16887_29_PTRH2_PTH2 PTRH2 chr3 165743688 0.00817 4.594e-23 35339 TRUE reported textfile 2 1 TRUE

7374 rs1355538 G A G A -0.0519 -0.0076 0.39847 NA FALSE FALSE FALSE GCST006061 0.0072 0.2923 537409 outcome TRUE reported 7803_4_CHST1_CHST1 CHST1 chr3 165787389 0.008059 1.194e-10 35338 TRUE reported textfile 2 1 TRUE

7375 rs1707652 T C T C 0.0679 0.0081 0.3102 NA FALSE FALSE FALSE GCST006061 0.0074 0.2701 537409 outcome TRUE reported 2878_66_YES1_YES YES1 chr3 165761011 0.00859 2.693e-15 35339 TRUE reported textfile 2 1 TRUE

7376 rs11121817 A G A G -0.0456 0.0067 0.35149 NA FALSE FALSE FALSE GCST006061 0.0079 0.3967 537409 outcome TRUE reported 19347_37_CA12_Carbonic_Anhydrase_XII CA12 chr1 11741030 0.008339 4.544e-08 35381 TRUE reported textfile 2 1 TRUE

7377 rs7183733 T C T C -0.0489 -0.0017 0.32215 NA FALSE FALSE FALSE GCST006061 0.0075 0.8191 537409 outcome TRUE reported 19347_37_CA12_Carbonic_Anhydrase_XII CA12 chr15 63373648 0.008438 6.819e-09 35362 TRUE reported textfile 2 1 TRUE

7378 rs34762726 A G A G -0.0772 -0.0047 0.30713 NA FALSE FALSE FALSE GCST006061 0.008 0.5543 537409 outcome TRUE reported 8261_51_STOM_STOM STOM chr3 49651777 0.008585 2.407e-19 35343 TRUE reported textfile 2 1 TRUE

7379 rs438811 T C T C -0.0668 -5.00E-04 0.23201 NA FALSE FALSE FALSE GCST006061 0.0088 0.9556 537409 outcome TRUE reported 8261_51_STOM_STOM STOM chr19 44913484 0.009487 1.908e-12 35366 TRUE reported textfile 2 1 TRUE

7380 rs12478841 G A G A 0.0631 0.0049 0.21569 NA FALSE FALSE FALSE GCST006061 0.008 0.5413 537409 outcome TRUE reported 12572_236_EFS_EFS EFS chr2 27588855 0.009852 1.504e-10 35364 TRUE reported textfile 2 1 TRUE

7381 rs150687841 C T C T 0.2081 -0.0212 0.0124 NA FALSE FALSE FALSE GCST006061 0.0381 0.5774 537409 outcome TRUE reported 12572_236_EFS_EFS EFS chr2 240741404 0.03789 3.97e-08 35258 TRUE reported textfile 2 1 TRUE

7382 rs1706435 G A G A 0.0938 0.0117 0.36981 NA FALSE FALSE FALSE GCST006061 0.0072 0.1047 537409 outcome TRUE reported 12572_236_EFS_EFS EFS chr3 165743688 0.00846 1.447e-28 35330 TRUE reported textfile 2 1 TRUE

7383 rs71352239 T C T C 0.0593 -0.0041 0.30263 NA FALSE FALSE FALSE GCST006061 0.0087 0.6372 537409 outcome TRUE reported 12572_236_EFS_EFS EFS chr19 44926286 0.008925 3.042e-11 35359 TRUE reported textfile 2 1 TRUE

7384 rs78810414 T C T C -0.0884 0.0257 0.08751 NA FALSE FALSE FALSE GCST006061 0.012 0.03151 537409 outcome TRUE reported 12572_236_EFS_EFS EFS chr8 20013602 0.014396 8.216e-10 35359 TRUE reported textfile 2 1 TRUE

7385 rs28720282 C T C T 0.0733 -0.0063 0.09507 NA FALSE FALSE FALSE GCST006061 0.0118 0.5926 537409 outcome TRUE reported 10917_40_GNGT2_GBGT2 GNGT2 chr11 61323484 0.013259 3.233e-08 35354 TRUE reported textfile 2 1 TRUE

7386 rs113747960 A G A G -0.0724 -0.0034 0.12384 NA FALSE FALSE FALSE GCST006061 0.0107 0.7473 537409 outcome TRUE reported 6054_6_NXPH3_NXPH3 NXPH3 chr12 7074644 0.012442 5.915e-09 35389 TRUE reported textfile 2 1 TRUE
7387 rs1260326 C T C T -0.0606 -0.0069 0.34132 NA FALSE FALSE FALSE GCST006061 0.0073 0.3401 537409 outcome TRUE reported 6054_6_NXPH3_NXPH3 NXPH3 chr2 27508073 0.008551 1.369e-12 35373 TRUE reported textfile 2 1 TRUE
7388 rs1355538 G A G A -0.0744 -0.0076 0.39847 NA FALSE FALSE FALSE GCST006061 0.0072 0.2923 537409 outcome TRUE reported 6054_6_NXPH3_NXPH3 NXPH3 chr3 165787389 0.008228 1.539e-19 35338 TRUE reported textfile 2 1 TRUE
7389 rs78810414 T C T C -0.1234 0.0257 0.08751 NA FALSE FALSE FALSE GCST006061 0.012 0.03151 537409 outcome TRUE reported 6054_6_NXPH3_NXPH3 NXPH3 chr8 20013602 0.014203 3.682e-18 35368 TRUE reported textfile 2 1 TRUE
7390 rs11227136 A G A G -0.0664 -0.0129 0.25007 NA FALSE FALSE FALSE GCST006061 0.0087 0.1386 537409 outcome TRUE reported 18198_51_HBQ1_HBAT HBQ1 chr11 65164493 0.009144 3.834e-13 35363 TRUE reported textfile 2 1 TRUE
7391 rs138620872 G A G A 0.2444 -0.0106 0.01929 NA FALSE FALSE FALSE GCST006061 0.0388 0.7847 537409 outcome TRUE reported 18198_51_HBQ1_HBAT HBQ1 chr16 72672741 0.029014 3.655e-17 35372 TRUE reported textfile 2 1 TRUE
7392 rs2287997 A G A G 0.0715 -0.011 0.14857 NA FALSE FALSE FALSE GCST006061 0.0088 0.2105 537409 outcome TRUE reported 18198_51_HBQ1_HBAT HBQ1 chr16 72106654 0.011241 2.008e-10 35375 TRUE reported textfile 2 1 TRUE
7393 rs34924625 G A G A 0.1793 0.0035 0.01967 NA FALSE FALSE FALSE GCST006061 0.0378 0.9265 537409 outcome TRUE reported 18198_51_HBQ1_HBAT HBQ1 chr16 69432734 0.028422 2.816e-10 35374 TRUE reported textfile 2 1 TRUE
7394 rs6445035 A G A G 0.1289 0.0154 0.1904 NA FALSE FALSE FALSE GCST006061 0.0089 0.08256 537409 outcome TRUE reported 18198_51_HBQ1_HBAT HBQ1 chr3 165762312 0.010041 1.016e-37 35339 TRUE reported textfile 2 1 TRUE
7395 rs10982054 A G A G -0.0642 -0.0026 0.27709 NA FALSE FALSE FALSE GCST006061 0.0081 0.7491 537409 outcome TRUE reported 10036_201_ZHX3_ZHX3 ZHX3 chr9 114071373 0.009128 2.012e-12 35350 TRUE reported textfile 2 1 TRUE
7396 rs62068364 T C T C -0.058 -0.0019 0.33982 NA FALSE FALSE FALSE GCST006061 0.0076 0.7999 537409 outcome TRUE reported 10036_201_ZHX3_ZHX3 ZHX3 chr16 89690791 0.008576 1.35e-11 35311 TRUE reported textfile 2 1 TRUE
7397 rs1355538 G A G A -0.0657 -0.0076 0.39847 NA FALSE FALSE FALSE GCST006061 0.0072 0.2923 537409 outcome TRUE reported 11270_17_CHCHD10_CHC10 CHCHD10 chr3 165787389 0.008026 2.701e-16 35329 TRUE reported textfile 2 1 TRUE
7398 rs6542680 T C T C -0.0567 -0.0012 0.18362 NA FALSE FALSE FALSE GCST006061 0.0091 0.8935 537409 outcome TRUE reported 8998_15_HS3ST4_HS3S4 HS3ST4 chr2 3592552 0.010263 3.302e-08 35332 TRUE reported textfile 2 1 TRUE
7399 rs1355538 G A G A -0.061 -0.0076 0.39847 NA FALSE FALSE FALSE GCST006061 0.0072 0.2923 537409 outcome TRUE reported 7146_16_PDIA4_PDIA4 PDIA4 chr3 165787389 0.008095 4.861e-14 35338 TRUE reported textfile 2 1 TRUE
7400 rs6542680 T C T C -0.0708 -0.0012 0.18362 NA FALSE FALSE FALSE GCST006061 0.0091 0.8935 537409 outcome TRUE reported 7146_16_PDIA4_PDIA4 PDIA4 chr2 3592552 0.010278 5.647e-12 35332 TRUE reported textfile 2 1 TRUE
7401 rs1355538 G A G A -0.0439 -0.0076 0.39847 NA FALSE FALSE FALSE GCST006061 0.0072 0.2923 537409 outcome TRUE reported 16536_3_HMGA1_HMGA1 HMGA1 chr3 165787389 0.008009 4.215e-08 35338 TRUE reported textfile 2 1 TRUE
7402 rs34475883 C T C T 0.0571 -0.01 0.1796 NA FALSE FALSE FALSE GCST006061 0.0104 0.3376 537409 outcome TRUE reported 16536_3_HMGA1_HMGA1

HMGA1 chr20 45092742 0.010256 2.587e-08 35369 TRUE reported
textfile 2 1 TRUE
7403 rs1354034 C T C T 0.0614 0.0088 0.38119 NA FALSE FALSE FALSE
GCST006061 0.0073 0.2248 537409 outcome TRUE reported 6371_50_ANGPTL7_ANGL7
ANGPTL7 chr3 56815721 0.008099 3.431e-14 35356 TRUE reported
textfile 2 1 TRUE
7404 rs3917538 A G A G 0.0607 -0.0137 0.21967 NA FALSE FALSE FALSE
GCST006061 0.0082 0.09535 537409 outcome TRUE reported 6371_50_ANGPTL7_ANGL7
ANGPTL7 chr7 95308581 0.009498 1.65e-10 35349 TRUE reported
textfile 2 1 TRUE
7405 rs4980320 C T C T 0.044 0.0041 0.46986 NA FALSE FALSE FALSE
GCST006061 0.0076 0.5858 537409 outcome TRUE reported 6371_50_ANGPTL7_ANGL7
ANGPTL7 chr11 198986 0.007901 2.563e-08 35352 TRUE reported textfile
2 1 TRUE
7406 rs1209879 T C T C -0.0978 0.0052 0.33625 NA FALSE FALSE FALSE
GCST006061 0.0075 0.4877 537409 outcome TRUE reported 2823_7_COMMD7_COMMD7
COMMD7 chr8 144851323 0.008511 1.465e-30 34534 TRUE reported
textfile 2 1 TRUE
7407 rs1354034 C T C T 0.0734 0.0088 0.38119 NA FALSE FALSE FALSE
GCST006061 0.0073 0.2248 537409 outcome TRUE reported 2823_7_COMMD7_COMMD7
COMMD7 chr3 56815721 0.008197 3.423e-19 35356 TRUE reported
textfile 2 1 TRUE
7408 rs1355538 G A G A -0.0614 -0.0076 0.39847 NA FALSE FALSE FALSE
GCST006061 0.0072 0.2923 537409 outcome TRUE reported 2823_7_COMMD7_COMMD7
COMMD7 chr3 165787389 0.008125 4.122e-14 35338 TRUE reported
textfile 2 1 TRUE
7409 rs4980320 C T C T 0.0533 0.0041 0.46986 NA FALSE FALSE FALSE
GCST006061 0.0076 0.5858 537409 outcome TRUE reported 2823_7_COMMD7_COMMD7
COMMD7 chr11 198986 0.007991 2.561e-11 35352 TRUE reported textfile
2 1 TRUE
7410 rs7113761 A G A G -0.0559 -0.0146 0.24918 NA FALSE FALSE FALSE
GCST006061 0.0087 0.09269 537409 outcome TRUE reported 2823_7_COMMD7_COMMD7
COMMD7 chr11 65159251 0.009227 1.376e-09 35363 TRUE reported
textfile 2 1 TRUE
7411 rs12044086 C T C T -0.1717 0.0068 0.025 NA FALSE FALSE FALSE
GCST006061 0.0258 0.7914 537409 outcome TRUE reported
17836_17_S100A16_S100A16 S100A16 chr1 153270357 0.027107 2.385e-10 35362
TRUE reported textfile 2 1 TRUE
7412 rs9726753 A G A G 0.1865 -0.023 0.43164 NA FALSE FALSE FALSE
GCST006061 0.0073 0.001579 537409 outcome TRUE reported
17836_17_S100A16_S100A16 S100A16 chr1 153619176 0.008285 3.221e-112 35362
TRUE reported textfile 2 1 TRUE
7413 rs1355538 G A G A -0.0541 -0.0076 0.39847 NA FALSE FALSE FALSE
GCST006061 0.0072 0.2923 537409 outcome TRUE reported 15437_11_RAMP3_RAMP3
RAMP3 chr3 165787389 0.00799 1.277e-11 35338 TRUE reported textfile
2 1 TRUE
7414 rs6542680 T C T C -0.0888 -0.0012 0.18362 NA FALSE FALSE FALSE
GCST006061 0.0091 0.8935 537409 outcome TRUE reported 15437_11_RAMP3_RAMP3
RAMP3 chr2 3592552 0.010129 1.833e-18 35332 TRUE reported textfile
2 1 TRUE
7415 rs1619994 G A G A 0.0631 0.0168 0.2807 NA FALSE FALSE FALSE
GCST006061 0.0078 0.03057 537409 outcome TRUE reported
12577_100_FEN1_FLAP_endonuclease_1 FEN1 chr3 165744047 0.008739 5.166e-13
35330 TRUE reported textfile 2 1 TRUE
7416 rs4977155 C T C T -0.0681 -0.0069 0.16663 NA FALSE FALSE FALSE
GCST006061 0.0127 0.5868 537409 outcome TRUE reported 9865_40_UBE2B_UBE2B
UBE2B chr14 106473508 0.010727 2.172e-10 34966 TRUE reported
textfile 2 1 TRUE
7417 rs1355538 G A G A -0.0615 -0.0076 0.39847 NA FALSE FALSE FALSE
GCST006061 0.0072 0.2923 537409 outcome TRUE reported 14192_31_PDCL2_PDCL2
PDCL2 chr3 165787389 0.008109 3.342e-14 35338 TRUE reported
textfile 2 1 TRUE
7418 rs281377 T C T C -0.0591 -0.0091 0.3353 NA FALSE FALSE FALSE
GCST006061 0.0075 0.2214 537409 outcome TRUE reported 11708_2_LCN1_LCN1
LCN1 chr19 48703346 0.00856 5.035e-12 35368 TRUE reported textfile
2 1 TRUE

7419 rs56307603 C T C T -0.0917 -5.00E-04 0.15132 NA FALSE FALSE FALSE
GCST006061 0.0104 0.9638 537409 outcome TRUE reported 11708_2_LCN1_LCN1
LCN1 chr9 135514751 0.011035 9.566e-17 35293 TRUE reported
textfile 2 1 TRUE
7420 rs621089 T C T C 0.0531 0.0016 0.23583 NA FALSE FALSE FALSE
GCST006061 0.0086 0.8572 537409 outcome TRUE reported 11708_2_LCN1_LCN1
LCN1 chr8 108139814 0.009276 1.038e-08 35334 TRUE reported
textfile 2 1 TRUE
7421 rs114537924 A G A G 0.1163 -0.0088 0.06291 NA FALSE FALSE FALSE
GCST006061 0.0243 0.7172 537409 outcome TRUE reported 5704_74_GZMM_Granzyme_M
GZMM chr19 548612 0.016794 4.356e-12 35308 TRUE reported textfile
2 1 TRUE
7422 rs3184504 C T C T -0.0547 0.0074 0.37862 NA FALSE FALSE FALSE
GCST006061 0.0075 0.3235 537409 outcome TRUE reported 5704_74_GZMM_Granzyme_M
GZMM chr12 111446804 0.008247 3.288e-11 35371 TRUE reported
textfile 2 1 TRUE
7423 rs3917538 A G A G 0.1038 -0.0137 0.21967 NA FALSE FALSE FALSE
GCST006061 0.0082 0.09535 537409 outcome TRUE reported
15383_200_EDN3_Endothelin_3 EDN3 chr7 95308581 0.009523 1.156e-27 35349
TRUE reported textfile 2 1 TRUE
7424 rs4735839 T C T C 0.051 -0.0012 0.25086 NA FALSE FALSE FALSE
GCST006061 0.0078 0.8755 537409 outcome TRUE reported
15383_200_EDN3_Endothelin_3 EDN3 chr8 1112631 0.009157 2.552e-08 35271
TRUE reported textfile 2 1 TRUE
7425 rs704 A G A G -0.1078 -0.0155 0.49189 NA FALSE FALSE FALSE GCST006061
0.0072 0.03144 537409 outcome TRUE reported 15383_200_EDN3_Endothelin_3 EDN3
chr17 28367840 0.007884 1.461e-42 35360 TRUE reported textfile 2
1 TRUE
7426 rs705379 A G A G -0.11 -8.00E-04 0.38289 NA FALSE FALSE FALSE
GCST006061 0.0072 0.9068 537409 outcome TRUE reported
15383_200_EDN3_Endothelin_3 EDN3 chr7 95324583 0.008141 1.327e-41 35349
TRUE reported textfile 2 1 TRUE
7427 rs111696008 A G A G -0.157 -0.0259 0.02443 NA FALSE FALSE FALSE
GCST006061 0.0401 0.519 537409 outcome TRUE reported 7766_25_ZP4_ZP4 ZP4
chr2 216622986 0.026739 4.314e-09 35359 TRUE reported textfile 2
1 TRUE
7428 rs1707652 T C T C 0.1144 0.0081 0.3102 NA FALSE FALSE FALSE
GCST006061 0.0074 0.2701 537409 outcome TRUE reported 7766_25_ZP4_ZP4 ZP4
chr3 165761011 0.008673 9.921e-40 35339 TRUE reported textfile 2
1 TRUE
7429 rs12478841 G A G A 0.072 0.0049 0.21569 NA FALSE FALSE FALSE
GCST006061 0.008 0.5413 537409 outcome TRUE reported 11556_19_ARFGAP1_ARFG1
ARFGAP1 chr2 27588855 0.009877 3.106e-13 35364 TRUE reported
textfile 2 1 TRUE
7430 rs1707652 T C T C 0.1021 0.0081 0.3102 NA FALSE FALSE FALSE
GCST006061 0.0074 0.2701 537409 outcome TRUE reported 11556_19_ARFGAP1_ARFG1
ARFGAP1 chr3 165761011 0.008831 6.475e-31 35330 TRUE reported
textfile 2 1 TRUE
7431 rs71352239 T C T C 0.1155 -0.0041 0.30263 NA FALSE FALSE FALSE
GCST006061 0.0087 0.6372 537409 outcome TRUE reported 11556_19_ARFGAP1_ARFG1
ARFGAP1 chr19 44926286 0.008935 3.198e-38 35359 TRUE reported
textfile 2 1 TRUE
7432 rs1707652 T C T C 0.0868 0.0081 0.3102 NA FALSE FALSE FALSE
GCST006061 0.0074 0.2701 537409 outcome TRUE reported 9800_20_NDUFB8_NDUB8
NDUFB8 chr3 165761011 0.008543 2.982e-24 35339 TRUE reported
textfile 2 1 TRUE
7433 rs111305232 C T C T 0.1903 0.064 0.02323 NA FALSE FALSE FALSE
GCST006061 0.0474 0.1767 537409 outcome TRUE reported 15333_11_SDF2_SDF2
SDF2 chr6 19605610 0.027853 8.353e-12 35375 TRUE reported
textfile 2 1 TRUE
7434 rs113969967 G A G A 0.3481 0.0015 0.04652 NA FALSE FALSE FALSE
GCST006061 0.0256 0.9535 537409 outcome TRUE reported 15333_11_SDF2_SDF2
SDF2 chr6 25115349 0.019325 1.534e-72 35378 TRUE reported
textfile 2 1 TRUE
7435 rs114173559 G A G A 0.2688 0.0493 0.02452 NA FALSE FALSE FALSE
GCST006061 0.0378 0.1921 537409 outcome TRUE reported 15333_11_SDF2_SDF2

SDF2 chr6 23175531 0.026581 4.862e-24 35364 TRUE reported
textfile 2 1 TRUE
7436 rs114746521 A G A G 0.2703 -0.0024 0.04971 NA FALSE FALSE FALSE
GCST006061 0.0321 0.9401 537409 outcome TRUE reported 15333_11_SDF2_SDF2
SDF2 chr6 33752282 0.018975 4.821e-46 35360 TRUE reported
textfile 2 1 TRUE
7437 rs115000510 A G A G 0.3107 -0.0261 0.02352 NA FALSE FALSE FALSE
GCST006061 0.047 0.5781 537409 outcome TRUE reported 15333_11_SDF2_SDF2
SDF2 chr6 22473837 0.027119 2.169e-30 35365 TRUE reported
textfile 2 1 TRUE
7438 rs115103442 T C T C 1.0305 -0.0057 0.01681 NA FALSE FALSE FALSE
GCST006061 0.0239 0.8103 537409 outcome TRUE reported 15333_11_SDF2_SDF2
SDF2 chr6 25497668 0.032216 1e-200 35375 TRUE reported textfile
2 1 TRUE
7439 rs143425782 A G A G 0.239 -0.0449 0.01921 NA FALSE FALSE FALSE
GCST006061 0.0284 0.1133 537409 outcome TRUE reported 15333_11_SDF2_SDF2
SDF2 chr6 34920277 0.030216 2.58e-15 35352 TRUE reported
textfile 2 1 TRUE
7440 rs148513395 C T C T 0.6212 0.047 0.01542 NA FALSE FALSE FALSE
GCST006061 0.0473 0.3209 537409 outcome TRUE reported 15333_11_SDF2_SDF2
SDF2 chr6 36310265 0.034156 6.522e-74 35353 TRUE reported
textfile 2 1 TRUE
7441 rs1706435 G A G A 0.0467 0.0117 0.36981 NA FALSE FALSE FALSE
GCST006061 0.0072 0.1047 537409 outcome TRUE reported 15333_11_SDF2_SDF2
SDF2 chr3 165743688 0.008228 1.382e-08 35339 TRUE reported
textfile 2 1 TRUE
7442 rs17360657 C T C T 0.4227 0.0369 0.02738 NA FALSE FALSE FALSE
GCST006061 0.0245 0.1316 537409 outcome TRUE reported 15333_11_SDF2_SDF2
SDF2 chr6 36548250 0.025464 6.951e-62 35355 TRUE reported
textfile 2 1 TRUE
7443 rs1863622 C T C T -0.0599 9e-04 0.23639 NA FALSE FALSE FALSE
GCST006061 0.0083 0.9099 537409 outcome TRUE reported 15333_11_SDF2_SDF2
SDF2 chr3 186678827 0.00932 1.304e-10 35368 TRUE reported textfile
2 1 TRUE
7444 rs2237193 A C A C 0.1438 0.0054 0.02818 NA FALSE FALSE FALSE
GCST006061 0.0184 0.7698 537409 outcome TRUE reported 15333_11_SDF2_SDF2
SDF2 chr6 16479900 0.025325 1.362e-08 35380 TRUE reported
textfile 2 1 TRUE
7445 rs55870921 T C T C -0.0549 -0.0076 0.33388 NA FALSE FALSE FALSE
GCST006061 0.0078 0.3291 537409 outcome TRUE reported 15333_11_SDF2_SDF2
SDF2 chr17 27461032 0.008438 7.703e-11 35359 TRUE reported
textfile 2 1 TRUE
7446 rs704 A G A G 0.2352 -0.0155 0.49189 NA FALSE FALSE FALSE GCST006061
0.0072 0.03144 537409 outcome TRUE reported 15333_11_SDF2_SDF2 SDF2 chr17
28367840 0.007919 7.266e-194 35360 TRUE reported textfile 2 1 TRUE
7447 rs72834371 A G A G 0.1136 0.0034 0.04452 NA FALSE FALSE FALSE
GCST006061 0.0236 0.8852 537409 outcome TRUE reported 15333_11_SDF2_SDF2
SDF2 chr6 21052452 0.019714 8.291e-09 35375 TRUE reported
textfile 2 1 TRUE
7448 rs9462399 C A C A 0.0812 -0.044 0.10455 NA FALSE FALSE FALSE
GCST006061 0.0118 0.0001836 537409 outcome TRUE reported 15333_11_SDF2_SDF2
SDF2 chr6 38173546 0.013411 1.405e-09 35364 TRUE reported
textfile 2 1 TRUE
7449 rs1707652 T C T C 0.0706 0.0081 0.3102 NA FALSE FALSE FALSE
GCST006061 0.0074 0.2701 537409 outcome TRUE reported 7225_51_MMGT1_MMGT1
MMGT1 chr3 165761011 0.008569 1.741e-16 35339 TRUE reported
textfile 2 1 TRUE
7450 rs1706435 G A G A 0.0479 0.0117 0.36981 NA FALSE FALSE FALSE
GCST006061 0.0072 0.1047 537409 outcome TRUE reported 9898_161_ARID1A_ARI1A
ARID1A chr3 165743688 0.008201 5.202e-09 35339 TRUE reported
textfile 2 1 TRUE
7451 rs7321465 G T G T 0.044 0.0192 0.41374 NA FALSE FALSE FALSE
GCST006061 0.0073 0.00857 537409 outcome TRUE reported 9898_161_ARID1A_ARI1A
ARID1A chr13 73363150 0.008037 4.389e-08 35357 TRUE reported
textfile 2 1 TRUE
7452 rs111696008 A G A G -0.1486 -0.0259 0.02443 NA FALSE FALSE FALSE

GCST006061 0.0401 0.519 537409 outcome TRUE reported 13514_121_RAB35_Rab_1C
RAB35 chr2 216622986 0.026601 2.319e-08 35353 TRUE reported
textfile 2 1 TRUE
7453 rs144704969 T C T C 0.1077 -0.0238 0.04536 NA FALSE FALSE FALSE
GCST006061 0.0154 0.1235 537409 outcome TRUE reported 13514_121_RAB35_Rab_1C
RAB35 chr1 196158214 0.019131 1.807e-08 35331 TRUE reported
textfile 2 1 TRUE
7454 rs1707652 T C T C 0.1129 0.0081 0.3102 NA FALSE FALSE FALSE
GCST006061 0.0074 0.2701 537409 outcome TRUE reported 13514_121_RAB35_Rab_1C
RAB35 chr3 165761011 0.008623 3.636e-39 35333 TRUE reported
textfile 2 1 TRUE
7455 rs704 A G A G 0.1517 -0.0155 0.49189 NA FALSE FALSE FALSE GCST006061
0.0072 0.03144 537409 outcome TRUE reported 13514_121_RAB35_Rab_1C RAB35
chr17 28367840 0.007989 2.156e-80 35354 TRUE reported textfile 2
1 TRUE
7456 rs1354034 C T C T 0.0497 0.0088 0.38119 NA FALSE FALSE FALSE
GCST006061 0.0073 0.2248 537409 outcome TRUE reported 9045_3_SLC27A2_S27A2
SLC27A2 chr3 56815721 0.008144 1.043e-09 35356 TRUE reported
textfile 2 1 TRUE
7457 rs12992267 T C T C -0.1322 0.0026 0.17923 NA FALSE FALSE FALSE
GCST006061 0.0089 0.7697 537409 outcome TRUE reported 8918_64_PTK2B_FAK2
PTK2B chr2 20992773 0.010561 5.928e-36 35373 TRUE reported
textfile 2 1 TRUE
7458 rs3816117 C T C T -0.0447 0.0137 0.49421 NA FALSE FALSE FALSE
GCST006061 0.0071 0.05455 537409 outcome TRUE reported 8918_64_PTK2B_FAK2
PTK2B chr16 56962246 0.008129 3.828e-08 35369 TRUE reported
textfile 2 1 TRUE
7459 rs4665985 C A C A 0.0611 0.0065 0.23221 NA FALSE FALSE FALSE
GCST006061 0.0082 0.424 537409 outcome TRUE reported 8918_64_PTK2B_FAK2
PTK2B chr2 27531011 0.009561 1.656e-10 35373 TRUE reported
textfile 2 1 TRUE
7460 rs74615535 A G A G -0.0902 0.0211 0.08965 NA FALSE FALSE FALSE
GCST006061 0.0119 0.07636 537409 outcome TRUE reported 8918_64_PTK2B_FAK2
PTK2B chr8 20023725 0.014125 1.703e-10 35369 TRUE reported
textfile 2 1 TRUE
7461 rs1355538 G A G A -0.0691 -0.0076 0.39847 NA FALSE FALSE FALSE
GCST006061 0.0072 0.2923 537409 outcome TRUE reported 12700_9_ACLY_ACLY
ACLY chr3 165787389 0.008022 7.089e-18 35329 TRUE reported
textfile 2 1 TRUE
7462 rs6542680 T C T C -0.0585 -0.0012 0.18362 NA FALSE FALSE FALSE
GCST006061 0.0091 0.8935 537409 outcome TRUE reported 12700_9_ACLY_ACLY
ACLY chr2 3592552 0.010193 9.508e-09 35323 TRUE reported textfile
2 1 TRUE
7463 rs113747960 A G A G -0.0919 -0.0034 0.12384 NA FALSE FALSE FALSE
GCST006061 0.0107 0.7473 537409 outcome TRUE reported 11371_1_GPR101_GP101
GPR101 chr12 7074644 0.012128 3.527e-14 35380 TRUE reported textfile
2 1 TRUE
7464 rs28624101 T C T C 0.0437 0.0242 0.36922 NA FALSE FALSE FALSE
GCST006061 0.0144 0.09211 537409 outcome TRUE reported 11371_1_GPR101_GP101
GPR101 chr6 32557514 0.007789 2.02e-08 35421 TRUE reported
textfile 2 1 TRUE
7465 rs10922098 T C T C -0.1381 -0.009 0.40011 NA FALSE FALSE FALSE
GCST006061 0.0075 0.2275 537409 outcome TRUE reported
12923_51_KRT17_Keratin_17 KRT17 chr1 196695521 0.008035 3.261e-66 35329
TRUE reported textfile 2 1 TRUE
7466 rs2686395 C T C T -0.0834 -0.0077 0.3101 NA FALSE FALSE FALSE
GCST006061 0.0074 0.2965 537409 outcome TRUE reported
12923_51_KRT17_Keratin_17 KRT17 chr3 165767231 0.008495 9.502e-23 35330
TRUE reported textfile 2 1 TRUE
7467 rs10922098 T C T C -0.0907 -0.009 0.40011 NA FALSE FALSE FALSE
GCST006061 0.0075 0.2275 537409 outcome TRUE reported 2734_49_NCR2_NKp44
NCR2 chr1 196695521 0.008065 2.406e-29 35338 TRUE reported
textfile 2 1 TRUE
7468 rs1355538 G A G A -0.0504 -0.0076 0.39847 NA FALSE FALSE FALSE
GCST006061 0.0072 0.2923 537409 outcome TRUE reported 2734_49_NCR2_NKp44
NCR2 chr3 165787389 0.008061 4.039e-10 35338 TRUE reported

textfile 2 1 TRUE
7469 rs1555405 A G A G -0.0479 -0.0047 0.30381 NA FALSE FALSE FALSE
GCST006061 0.0081 0.5592 537409 outcome TRUE reported 2734_49_NCR2_NKp44
NCR2 chr14 100710432 0.008637 2.922e-08 35380 TRUE reported
textfile 2 1 TRUE
7470 rs10922098 T C T C -0.1268 -0.009 0.40011 NA FALSE FALSE FALSE
GCST006061 0.0075 0.2275 537409 outcome TRUE reported 10024_44_HOGA1_HOGA1
HOGA1 chr1 196695521 0.007999 1.347e-56 35326 TRUE reported
textfile 2 1 TRUE
7471 rs1707652 T C T C 0.0568 0.0081 0.3102 NA FALSE FALSE FALSE
GCST006061 0.0074 0.2701 537409 outcome TRUE reported 10024_44_HOGA1_HOGA1
HOGA1 chr3 165761011 0.008477 2.074e-11 35327 TRUE reported
textfile 2 1 TRUE
7472 rs10922098 T C T C -0.0453 -0.009 0.40011 NA FALSE FALSE FALSE
GCST006061 0.0075 0.2275 537409 outcome TRUE reported 7193_98_PCDHAC1_PCDC1
PCDHAC1 chr1 196695521 0.008049 1.82e-08 35338 TRUE reported
textfile 2 1 TRUE
7473 rs1355538 G A G A -0.0462 -0.0076 0.39847 NA FALSE FALSE FALSE
GCST006061 0.0072 0.2923 537409 outcome TRUE reported 7193_98_PCDHAC1_PCDC1
PCDHAC1 chr3 165787389 0.008025 8.571e-09 35338 TRUE reported
textfile 2 1 TRUE
7474 rs2851584 G T G T 0.1275 0.0035 0.07745 NA FALSE FALSE FALSE
GCST006061 0.0121 0.77 537409 outcome TRUE reported 7193_98_PCDHAC1_PCDC1
PCDHAC1 chr11 94729990 0.014749 5.407e-18 35354 TRUE reported
textfile 2 1 TRUE
7475 rs10922098 T C T C -0.1502 -0.009 0.40011 NA FALSE FALSE FALSE
GCST006061 0.0075 0.2275 537409 outcome TRUE reported 7755_37_ITGB5_ITB5
ITGB5 chr1 196695521 0.008028 4.122e-78 35338 TRUE reported
textfile 2 1 TRUE
7476 rs6445035 A G A G 0.0817 0.0154 0.1904 NA FALSE FALSE FALSE
GCST006061 0.0089 0.08256 537409 outcome TRUE reported 7755_37_ITGB5_ITB5
ITGB5 chr3 165762312 0.010009 3.283e-16 35339 TRUE reported
textfile 2 1 TRUE
7477 rs10922098 T C T C -0.0756 -0.009 0.40011 NA FALSE FALSE FALSE
GCST006061 0.0075 0.2275 537409 outcome TRUE reported 13479_8_FAM171A2_F1712
FAM171A2 chr1 196695521 0.008194 2.795e-20 35332 TRUE reported
textfile 2 1 TRUE
7478 rs12478841 G A G A 0.0527 0.0049 0.21569 NA FALSE FALSE FALSE
GCST006061 0.008 0.5413 537409 outcome TRUE reported 13479_8_FAM171A2_F1712
FAM171A2 chr2 27588855 0.009667 4.99e-08 35367 TRUE reported
textfile 2 1 TRUE
7479 rs1706435 G A G A 0.0621 0.0117 0.36981 NA FALSE FALSE FALSE
GCST006061 0.0072 0.1047 537409 outcome TRUE reported 13479_8_FAM171A2_F1712
FAM171A2 chr3 165743688 0.008305 7.56e-14 35333 TRUE reported
textfile 2 1 TRUE
7480 rs704 A G A G 0.0751 -0.0155 0.49189 NA FALSE FALSE FALSE GCST006061
0.0072 0.03144 537409 outcome TRUE reported 13479_8_FAM171A2_F1712 FAM171A2
chr17 28367840 0.008028 8.338e-21 35354 TRUE reported textfile 2
1 TRUE
7481 rs10922098 T C T C -0.0837 -0.009 0.40011 NA FALSE FALSE FALSE
GCST006061 0.0075 0.2275 537409 outcome TRUE reported 4880_21_GDF2_GDF2
GDF2 chr1 196695521 0.008217 2.276e-24 35338 TRUE reported
textfile 2 1 TRUE
7482 rs1956713 A G A G -0.1091 0.0027 0.15334 NA FALSE FALSE FALSE
GCST006061 0.0101 0.7898 537409 outcome TRUE reported 4880_21_GDF2_GDF2
GDF2 chr14 94466582 0.011082 7.22e-23 35372 TRUE reported
textfile 2 1 TRUE
7483 rs2229498 A G A G -0.0683 0.0047 0.16514 NA FALSE FALSE FALSE
GCST006061 0.0092 0.6113 537409 outcome TRUE reported 4880_21_GDF2_GDF2
GDF2 chr10 69097096 0.010901 3.713e-10 35362 TRUE reported
textfile 2 1 TRUE
7484 rs35887873 C T C T 0.0556 -0.0114 0.26311 NA FALSE FALSE FALSE
GCST006061 0.0091 0.2089 537409 outcome TRUE reported 4880_21_GDF2_GDF2
GDF2 chr11 126349501 0.009083 9.275e-10 35374 TRUE reported
textfile 2 1 TRUE
7485 rs68066031 C T C T 0.0721 -0.0076 0.19513 NA FALSE FALSE FALSE

GCST006061 0.0091 0.4038 537409 outcome TRUE reported 4880_21_GDF2_GDF2
GDF2 chr2 224015781 0.01014 1.155e-12 35366 TRUE reported textfile
2 1 TRUE

7486 rs7080386 A C A C 0.0523 -0.0468 0.43502 NA FALSE FALSE FALSE
GCST006061 0.0072 7.735e-11 537409 outcome TRUE reported 4880_21_GDF2_GDF2
GDF2 chr10 63288546 0.008106 1.102e-10 35351 TRUE reported
textfile 2 1 TRUE

7487 rs7848055 C A C A -0.1341 0.0197 0.36834 NA FALSE FALSE FALSE
GCST006061 0.0076 0.009839 537409 outcome TRUE reported 4880_21_GDF2_GDF2
GDF2 chr9 92353210 0.008322 2.029e-58 35359 TRUE reported
textfile 2 1 TRUE

7488 rs10922098 T C T C -0.0821 -0.009 0.40011 NA FALSE FALSE FALSE
GCST006061 0.0075 0.2275 537409 outcome TRUE reported 14329_4_DIDO1_DIDO1
DIDO1 chr1 196695521 0.008089 3.337e-24 35338 TRUE reported
textfile 2 1 TRUE

7489 rs10922098 T C T C -0.0659 -0.009 0.40011 NA FALSE FALSE FALSE
GCST006061 0.0075 0.2275 537409 outcome TRUE reported 18878_15_GREM1_GREM1
GREM1 chr1 196695521 0.008309 2.166e-15 35338 TRUE reported
textfile 2 1 TRUE

7490 rs11071897 G A G A -0.4539 -0.0238 0.01329 NA FALSE FALSE FALSE
GCST006061 0.0259 0.3582 537409 outcome TRUE reported 18878_15_GREM1_GREM1
GREM1 chr15 32678947 0.037369 5.993e-34 35398 TRUE reported
textfile 2 1 TRUE

7491 rs114922365 A G A G 0.0959 0.0019 0.09078 NA FALSE FALSE FALSE
GCST006061 0.0138 0.8904 537409 outcome TRUE reported 18878_15_GREM1_GREM1
GREM1 chr3 186409123 0.013945 6.109e-12 35365 TRUE reported
textfile 2 1 TRUE

7492 rs117880751 G T G T -0.1269 -0.0087 0.05721 NA FALSE FALSE FALSE
GCST006061 0.0186 0.6419 537409 outcome TRUE reported 18878_15_GREM1_GREM1
GREM1 chr15 33119694 0.01843 5.762e-12 35380 TRUE reported textfile
2 1 TRUE

7493 rs144674978 T C T C 0.4937 -0.0172 0.01392 NA FALSE FALSE FALSE
GCST006061 0.0464 0.7113 537409 outcome TRUE reported 18878_15_GREM1_GREM1
GREM1 chr15 32857550 0.036706 3.079e-41 35396 TRUE reported
textfile 2 1 TRUE

7494 rs149062332 C T C T 0.4545 0.0424 0.02045 NA FALSE FALSE FALSE
GCST006061 0.0435 0.33 537409 outcome TRUE reported 18878_15_GREM1_GREM1
GREM1 chr3 186623236 0.027939 1.672e-59 35367 TRUE reported
textfile 2 1 TRUE

7495 rs17816447 T C T C 0.3411 -0.0237 0.03025 NA FALSE FALSE FALSE
GCST006061 0.0261 0.3622 537409 outcome TRUE reported 18878_15_GREM1_GREM1
GREM1 chr15 32843327 0.024928 1.277e-42 35396 TRUE reported
textfile 2 1 TRUE

7496 rs4779584 C T C T -0.5988 -0.0181 0.18567 NA FALSE FALSE FALSE
GCST006061 0.009 0.04427 537409 outcome TRUE reported 18878_15_GREM1_GREM1
GREM1 chr15 32702555 0.008403 1e-200 35398 TRUE reported textfile
2 1 TRUE

7497 rs56031155 T C T C 0.1448 0.0061 0.14736 NA FALSE FALSE FALSE
GCST006061 0.0106 0.5658 537409 outcome TRUE reported 18878_15_GREM1_GREM1
GREM1 chr15 29972087 0.011931 6.75e-34 35397 TRUE reported
textfile 2 1 TRUE

7498 rs56111084 T C T C 0.139 -0.0157 0.02785 NA FALSE FALSE FALSE
GCST006061 0.0265 0.5533 537409 outcome TRUE reported 18878_15_GREM1_GREM1
GREM1 chr15 27774981 0.02517 3.343e-08 35393 TRUE reported textfile
2 1 TRUE

7499 rs79404501 C T C T 0.1479 0.006 0.02476 NA FALSE FALSE FALSE
GCST006061 0.0292 0.8382 537409 outcome TRUE reported 18878_15_GREM1_GREM1
GREM1 chr15 29680923 0.026665 2.913e-08 35396 TRUE reported
textfile 2 1 TRUE

7500 rs10922098 T C T C -0.0901 -0.009 0.40011 NA FALSE FALSE FALSE
GCST006061 0.0075 0.2275 537409 outcome TRUE reported 13595_20_THTPA_THTPA
THTPA chr1 196695521 0.008035 3.52e-29 35332 TRUE reported
textfile 2 1 TRUE

7501 rs6445035 A G A G 0.1434 0.0154 0.1904 NA FALSE FALSE FALSE
GCST006061 0.0089 0.08256 537409 outcome TRUE reported 13595_20_THTPA_THTPA
THTPA chr3 165762312 0.009989 9.736e-47 35333 TRUE reported

textfile 2 1 TRUE
7502 rs10922098 T C T C -0.0989 -0.009 0.40011 NA FALSE FALSE FALSE
GCST006061 0.0075 0.2275 537409 outcome TRUE reported 4237_70_LCMT1_LCMT1
LCMT1 chr1 196695521 0.0083 9.826e-33 35338 TRUE reported textfile
2 1 TRUE
7503 rs4980320 C T C T 0.0464 0.0041 0.46986 NA FALSE FALSE FALSE
GCST006061 0.0076 0.5858 537409 outcome TRUE reported 4237_70_LCMT1_LCMT1
LCMT1 chr11 198986 0.008164 1.321e-08 35352 TRUE reported textfile
2 1 TRUE
7504 rs5030044 G A G A 0.1978 0.009 0.12205 NA FALSE FALSE FALSE
GCST006061 0.0115 0.4352 537409 outcome TRUE reported 4237_70_LCMT1_LCMT1
LCMT1 chr3 186731333 0.012381 1.861e-57 35368 TRUE reported
textfile 2 1 TRUE
7505 rs10922098 T C T C -0.0682 -0.009 0.40011 NA FALSE FALSE FALSE
GCST006061 0.0075 0.2275 537409 outcome TRUE reported 12515_45_UCK2_UCK2
UCK2 chr1 196695521 0.007953 9.832e-18 35329 TRUE reported
textfile 2 1 TRUE
7506 rs6445035 A G A G 0.0867 0.0154 0.1904 NA FALSE FALSE FALSE
GCST006061 0.0089 0.08256 537409 outcome TRUE reported 12515_45_UCK2_UCK2
UCK2 chr3 165762312 0.009908 2.117e-18 35330 TRUE reported
textfile 2 1 TRUE
7507 rs704 A G A G -0.0512 -0.0155 0.49189 NA FALSE FALSE FALSE GCST006061
0.0072 0.03144 537409 outcome TRUE reported 12515_45_UCK2_UCK2 UCK2 chr17
28367840 0.007804 5.349e-11 35351 TRUE reported textfile 2 1 TRUE
7508 rs10922098 T C T C -0.1952 -0.009 0.40011 NA FALSE FALSE FALSE
GCST006061 0.0075 0.2275 537409 outcome TRUE reported 14286_2_NFATC1_NF_ATc1
NFATC1 chr1 196695521 0.008119 9.938e-128 35338 TRUE reported
textfile 2 1 TRUE
7509 rs12478841 G A G A 0.0581 0.0049 0.21569 NA FALSE FALSE FALSE
GCST006061 0.008 0.5413 537409 outcome TRUE reported 14286_2_NFATC1_NF_ATc1
NFATC1 chr2 27588855 0.009629 1.602e-09 35373 TRUE reported
textfile 2 1 TRUE
7510 rs1451915 A C A C 0.0669 -0.0107 0.13641 NA FALSE FALSE FALSE
GCST006061 0.0124 0.3895 537409 outcome TRUE reported 14286_2_NFATC1_NF_ATc1
NFATC1 chr1 196058002 0.011589 7.802e-09 35335 TRUE reported
textfile 2 1 TRUE
7511 rs1707652 T C T C 0.0921 0.0081 0.3102 NA FALSE FALSE FALSE
GCST006061 0.0074 0.2701 537409 outcome TRUE reported 14286_2_NFATC1_NF_ATc1
NFATC1 chr3 165761011 0.008606 1.002e-26 35339 TRUE reported
textfile 2 1 TRUE
7512 rs10922098 T C T C -0.1744 -0.009 0.40011 NA FALSE FALSE FALSE
GCST006061 0.0075 0.2275 537409 outcome TRUE reported 14208_3_RBP7_RET7
RBP7 chr1 196695521 0.008171 4.433e-101 35338 TRUE reported
textfile 2 1 TRUE
7513 rs1707652 T C T C 0.0868 0.0081 0.3102 NA FALSE FALSE FALSE
GCST006061 0.0074 0.2701 537409 outcome TRUE reported 14208_3_RBP7_RET7
RBP7 chr3 165761011 0.00865 1.068e-23 35339 TRUE reported textfile
2 1 TRUE
7514 rs62143198 A G A G 0.0502 -0.0043 0.26852 NA FALSE FALSE FALSE
GCST006061 0.0099 0.6616 537409 outcome TRUE reported 14208_3_RBP7_RET7
RBP7 chr19 53817685 0.009086 3.29e-08 35363 TRUE reported
textfile 2 1 TRUE
7515 rs7513100 C T C T 0.0668 0 0.14871 NA FALSE FALSE FALSE GCST006061
0.0098 0.9981 537409 outcome TRUE reported 14208_3_RBP7_RET7 RBP7 chr1
198065935 0.011247 2.863e-09 35343 TRUE reported textfile 2 1 TRUE
7516 rs10922098 T C T C -0.1051 -0.009 0.40011 NA FALSE FALSE FALSE
GCST006061 0.0075 0.2275 537409 outcome TRUE reported 5359_65_PIM1_PIM1
PIM1 chr1 196695521 0.008043 5.119e-39 35338 TRUE reported
textfile 2 1 TRUE
7517 rs112963922 T C T C 0.3027 0.0542 0.01119 NA FALSE FALSE FALSE
GCST006061 0.0399 0.1741 537409 outcome TRUE reported 5359_65_PIM1_PIM1
PIM1 chr14 94411531 0.036978 2.703e-16 35371 TRUE reported
textfile 2 1 TRUE
7518 rs1355538 G A G A -0.0535 -0.0076 0.39847 NA FALSE FALSE FALSE
GCST006061 0.0072 0.2923 537409 outcome TRUE reported 5359_65_PIM1_PIM1
PIM1 chr3 165787389 0.008046 2.945e-11 35338 TRUE reported

textfile 2 1 TRUE
7519 rs33972313 T C T C -0.1421 0.0068 0.03423 NA FALSE FALSE FALSE GCST006061 0.0217 0.7549 537409 outcome TRUE reported 5359_65_PIM1_PIM1 PIM1 chr5 139379813 0.021559 4.365e-11 35358 TRUE reported textfile 2 1 TRUE
7520 rs10922098 T C T C -0.1151 -0.009 0.40011 NA FALSE FALSE FALSE GCST006061 0.0075 0.2275 537409 outcome TRUE reported 5639_49_NETO1_NETO1 NETO1 chr1 196695521 0.008148 2.618e-45 35338 TRUE reported textfile 2 1 TRUE
7521 rs6445035 A G A G 0.066 0.0154 0.1904 NA FALSE FALSE FALSE GCST006061 0.0089 0.08256 537409 outcome TRUE reported 5639_49_NETO1_NETO1 NETO1 chr3 165762312 0.010151 7.95e-11 35339 TRUE reported textfile 2 1 TRUE
7522 rs10922098 T C T C -0.2002 -0.009 0.40011 NA FALSE FALSE FALSE GCST006061 0.0075 0.2275 537409 outcome TRUE reported 17449_23_CD9_CD9 CD9 chr1 196695521 0.008144 1.9e-133 35338 TRUE reported textfile 2 1 TRUE
7523 rs12403425 A C A C 0.0526 -0.0064 0.2662 NA FALSE FALSE FALSE GCST006061 0.009 0.4754 537409 outcome TRUE reported 17449_23_CD9_CD9 CD9 chr1 197845693 0.009051 6.196e-09 35337 TRUE reported textfile 2 1 TRUE
7524 rs1707652 T C T C 0.1115 0.0081 0.3102 NA FALSE FALSE FALSE GCST006061 0.0074 0.2701 537409 outcome TRUE reported 17449_23_CD9_CD9 CD9 chr3 165761011 0.008638 4.041e-38 35339 TRUE reported textfile 2 1 TRUE
7525 rs10922098 T C T C -0.0659 -0.009 0.40011 NA FALSE FALSE FALSE GCST006061 0.0075 0.2275 537409 outcome TRUE reported 8336_267_GRAMD1C_GRM1C GRAMD1C chr1 196695521 0.008162 6.786e-16 35338 TRUE reported textfile 2 1 TRUE
7526 rs1355538 G A G A -0.0542 -0.0076 0.39847 NA FALSE FALSE FALSE GCST006061 0.0072 0.2923 537409 outcome TRUE reported 8336_267_GRAMD1C_GRM1C GRAMD1C chr3 165787389 0.008159 3.078e-11 35338 TRUE reported textfile 2 1 TRUE
7527 rs148931761 C A C A 0.1618 -0.0564 0.02775 NA FALSE FALSE FALSE GCST006061 0.0367 0.1249 537409 outcome TRUE reported 8336_267_GRAMD1C_GRM1C GRAMD1C chr3 108009106 0.024382 3.223e-11 35352 TRUE reported textfile 2 1 TRUE
7528 rs1697422 C T C T -0.0443 0.0053 0.48923 NA FALSE FALSE FALSE GCST006061 0.0072 0.4547 537409 outcome TRUE reported 8336_267_GRAMD1C_GRM1C GRAMD1C chr1 21495404 0.008015 3.253e-08 35374 TRUE reported textfile 2 1 TRUE
7529 rs4795730 G A G A 0.0594 -0.0027 0.15942 NA FALSE FALSE FALSE GCST006061 0.011 0.8084 537409 outcome TRUE reported 8336_267_GRAMD1C_GRM1C GRAMD1C chr17 27555748 0.010885 4.839e-08 35359 TRUE reported textfile 2 1 TRUE
7530 rs56209585 A G A G 0.3398 -0.0318 0.01397 NA FALSE FALSE FALSE GCST006061 0.0361 0.3784 537409 outcome TRUE reported 8336_267_GRAMD1C_GRM1C GRAMD1C chr3 114839696 0.034252 3.385e-23 35365 TRUE reported textfile 2 1 TRUE
7531 rs58358607 G T G T 0.1746 0.0123 0.02932 NA FALSE FALSE FALSE GCST006061 0.0262 0.6384 537409 outcome TRUE reported 8336_267_GRAMD1C_GRM1C GRAMD1C chr3 114326820 0.023165 4.797e-14 35363 TRUE reported textfile 2 1 TRUE
7532 rs704 A G A G -0.1984 -0.0155 0.49189 NA FALSE FALSE FALSE GCST006061 0.0072 0.03144 537409 outcome TRUE reported 8336_267_GRAMD1C_GRM1C GRAMD1C chr17 28367840 0.007955 2.634e-137 35360 TRUE reported textfile 2 1 TRUE
7533 rs78441035 A G A G 0.3256 0.0073 0.01415 NA FALSE FALSE FALSE GCST006061 0.0269 0.7865 537409 outcome TRUE reported 8336_267_GRAMD1C_GRM1C GRAMD1C chr3 112775423 0.033185 1.004e-22 35362 TRUE reported textfile 2 1 TRUE
7534 rs10922098 T C T C -0.2162 -0.009 0.40011 NA FALSE FALSE FALSE GCST006061 0.0075 0.2275 537409 outcome TRUE reported 19518_12_HK3_HXK3 HK3 chr1 196695521 0.008038 2.285e-159 35338 TRUE reported textfile 2 1 TRUE
7535 rs2686395 C T C T -0.0917 -0.0077 0.3101 NA FALSE FALSE FALSE

GCST006061 0.0074 0.2965 537409 outcome TRUE reported 19518_12_HK3_HXK3 HK3
chr3 165767231 0.008547 7.459e-27 35339 TRUE reported textfile 2
1 TRUE
7536 rs10922098 T C T C -0.1587 -0.009 0.40011 NA FALSE FALSE FALSE
GCST006061 0.0075 0.2275 537409 outcome TRUE reported 10933_107_CALN1_CABP8
CALN1 chr1 196695521 0.008097 1.543e-85 35329 TRUE reported
textfile 2 1 TRUE
7537 rs11569415 A G A G -0.0517 0.0011 0.24276 NA FALSE FALSE FALSE
GCST006061 0.0097 0.9129 537409 outcome TRUE reported 10933_107_CALN1_CABP8
CALN1 chr19 6716268 0.00924 2.206e-08 35363 TRUE reported textfile 2
1 TRUE
7538 rs1355538 G A G A -0.0698 -0.0076 0.39847 NA FALSE FALSE FALSE
GCST006061 0.0072 0.2923 537409 outcome TRUE reported 10933_107_CALN1_CABP8
CALN1 chr3 165787389 0.008103 7.064e-18 35329 TRUE reported
textfile 2 1 TRUE
7539 rs10922098 T C T C -0.1385 -0.009 0.40011 NA FALSE FALSE FALSE
GCST006061 0.0075 0.2275 537409 outcome TRUE reported 13386_248_RNF34_RNF34
RNF34 chr1 196695521 0.008082 7.905e-66 35329 TRUE reported
textfile 2 1 TRUE
7540 rs1706435 G A G A 0.0613 0.0117 0.36981 NA FALSE FALSE FALSE
GCST006061 0.0072 0.1047 537409 outcome TRUE reported 13386_248_RNF34_RNF34
RNF34 chr3 165743688 0.008204 7.909e-14 35330 TRUE reported
textfile 2 1 TRUE
7541 rs10922098 T C T C -0.0955 -0.009 0.40011 NA FALSE FALSE FALSE
GCST006061 0.0075 0.2275 537409 outcome TRUE reported 8059_1_THPO_Tpo THPO
chr1 196695521 0.008151 1.056e-31 35338 TRUE reported textfile 2
1 TRUE
7542 rs1707652 T C T C 0.0815 0.0081 0.3102 NA FALSE FALSE FALSE
GCST006061 0.0074 0.2701 537409 outcome TRUE reported 8059_1_THPO_Tpo THPO
chr3 165761011 0.008608 2.866e-21 35339 TRUE reported textfile 2
1 TRUE
7543 rs10922098 T C T C -0.1404 -0.009 0.40011 NA FALSE FALSE FALSE
GCST006061 0.0075 0.2275 537409 outcome TRUE reported 8929_7_BDP1_BDP1
BDP1 chr1 196695521 0.008077 1.123e-67 35338 TRUE reported
textfile 2 1 TRUE
7544 rs12995461 C A C A 0.0557 0.0086 0.21643 NA FALSE FALSE FALSE
GCST006061 0.008 0.2842 537409 outcome TRUE reported 8929_7_BDP1_BDP1
BDP1 chr2 27555300 0.009543 5.319e-09 35373 TRUE reported
textfile 2 1 TRUE
7545 rs1355538 G A G A -0.058 -0.0076 0.39847 NA FALSE FALSE FALSE
GCST006061 0.0072 0.2923 537409 outcome TRUE reported 8929_7_BDP1_BDP1
BDP1 chr3 165787389 0.00808 7.064e-13 35338 TRUE reported textfile
2 1 TRUE
7546 rs10922098 T C T C -0.043 -0.009 0.40011 NA FALSE FALSE FALSE
GCST006061 0.0075 0.2275 537409 outcome TRUE reported 19141_22_DAP_DAP1 DAP
chr1 196695521 0.007472 8.685e-09 35338 TRUE reported textfile 2
1 TRUE
7547 rs140915195 A G A G 0.6497 0.0069 0.01693 NA FALSE FALSE FALSE
GCST006061 0.0586 0.9062 537409 outcome TRUE reported 19141_22_DAP_DAP1 DAP
chr3 186964310 0.035939 4.744e-73 35366 TRUE reported textfile 2
1 TRUE
7548 rs28412033 G A G A -0.1301 -0.0146 0.08208 NA FALSE FALSE FALSE
GCST006061 0.0141 0.3 537409 outcome TRUE reported 19141_22_DAP_DAP1 DAP
chr3 187031664 0.017236 4.408e-14 35362 TRUE reported textfile 2
1 TRUE
7549 rs58624989 T C T C 0.1614 -0.0031 0.07744 NA FALSE FALSE FALSE
GCST006061 0.0129 0.8094 537409 outcome TRUE reported 19141_22_DAP_DAP1 DAP
chr3 188262648 0.017384 1.626e-20 35372 TRUE reported textfile 2
1 TRUE
7550 rs6797321 C T C T 0.6789 9e-04 0.42142 NA FALSE FALSE FALSE
GCST006061 0.0072 0.8976 537409 outcome TRUE reported 19141_22_DAP_DAP1 DAP
chr3 186645795 0.005369 1e-200 35368 TRUE reported textfile 2 1
TRUE
7551 rs7611640 A G A G 0.0821 -0.008 0.19126 NA FALSE FALSE FALSE
GCST006061 0.009 0.3724 537409 outcome TRUE reported 19141_22_DAP_DAP1 DAP
chr3 187360614 0.011763 2.968e-12 35366 TRUE reported textfile 2

1 TRUE
7552 rs78311875 G A G A -0.2574 0.018 0.02675 NA FALSE FALSE FALSE GCST006061 0.0356 0.613 537409 outcome TRUE reported 19141_22_DAP_DAP1 DAP chr3 186307863 0.02901 7.138e-19 35358 TRUE reported textfile 2 1 TRUE
7553 rs9852488 T C T C -0.2263 -0.0135 0.28938 NA FALSE FALSE FALSE GCST006061 0.0079 0.08878 537409 outcome TRUE reported 19141_22_DAP_DAP1 DAP chr3 186753538 0.010194 3.445e-109 35366 TRUE reported textfile 2 1 TRUE
7554 rs9858594 G A G A -0.3738 0.0162 0.18479 NA FALSE FALSE FALSE GCST006061 0.0097 0.09381 537409 outcome TRUE reported 19141_22_DAP_DAP1 DAP chr3 186566163 0.011799 1e-200 35367 TRUE reported textfile 2 1 TRUE
7555 rs10922098 T C T C -0.124 -0.009 0.40011 NA FALSE FALSE FALSE GCST006061 0.0075 0.2275 537409 outcome TRUE reported 12756_3_BACH2_BACH2 BACH2 chr1 196695521 0.007998 3.298e-54 35329 TRUE reported textfile 2 1 TRUE
7556 rs2686395 C T C T -0.0625 -0.0077 0.3101 NA FALSE FALSE FALSE GCST006061 0.0074 0.2965 537409 outcome TRUE reported 12756_3_BACH2_BACH2 BACH2 chr3 165767231 0.008462 1.517e-13 35330 TRUE reported textfile 2 1 TRUE
7557 rs3917538 A G A G 0.0871 -0.0137 0.21967 NA FALSE FALSE FALSE GCST006061 0.0082 0.09535 537409 outcome TRUE reported 12756_3_BACH2_BACH2 BACH2 chr7 95308581 0.009479 3.975e-20 35340 TRUE reported textfile 2 1 TRUE
7558 rs10922098 T C T C -0.2212 -0.009 0.40011 NA FALSE FALSE FALSE GCST006061 0.0075 0.2275 537409 outcome TRUE reported 4712_28_APOD_Apo_D APOD chr1 196695521 0.00819 1.21e-160 35338 TRUE reported textfile 2 1 TRUE
7559 rs1707652 T C T C 0.1045 0.0081 0.3102 NA FALSE FALSE FALSE GCST006061 0.0074 0.2701 537409 outcome TRUE reported 4712_28_APOD_Apo_D APOD chr3 165761011 0.008686 2.452e-33 35339 TRUE reported textfile 2 1 TRUE
7560 rs34967494 A G A G 0.0578 -0.0033 0.23758 NA FALSE FALSE FALSE GCST006061 0.0091 0.7133 537409 outcome TRUE reported 4712_28_APOD_Apo_D APOD chr1 197820974 0.009496 1.15e-09 35337 TRUE reported textfile 2 1 TRUE
7561 rs35351085 T C T C 0.1157 0.0061 0.04877 NA FALSE FALSE FALSE GCST006061 0.022 0.7825 537409 outcome TRUE reported 4712_28_APOD_Apo_D APOD chr1 197992799 0.018646 5.465e-10 35343 TRUE reported textfile 2 1 TRUE
7562 rs10922098 T C T C -0.0464 -0.009 0.40011 NA FALSE FALSE FALSE GCST006061 0.0075 0.2275 537409 outcome TRUE reported 8899_75_UGT1A8_UD18 UGT1A8 chr1 196695521 0.008012 6.996e-09 35338 TRUE reported textfile 2 1 TRUE
7563 rs80167373 A G A G -0.1295 -0.0132 0.03467 NA FALSE FALSE FALSE GCST006061 0.0168 0.4315 537409 outcome TRUE reported 8899_75_UGT1A8_UD18 UGT1A8 chr10 7724618 0.021744 2.591e-09 35361 TRUE reported textfile 2 1 TRUE
7564 rs10922098 T C T C -0.0511 -0.009 0.40011 NA FALSE FALSE FALSE GCST006061 0.0075 0.2275 537409 outcome TRUE reported 11667_29_TNS2_TENC1 TNS2 chr1 196695521 0.007999 1.679e-10 35329 TRUE reported textfile 2 1 TRUE
7565 rs9852529 A G A G -0.1005 -0.0031 0.30753 NA FALSE FALSE FALSE GCST006061 0.0081 0.7037 537409 outcome TRUE reported 11667_29_TNS2_TENC1 TNS2 chr3 49693192 0.008463 1.588e-32 35335 TRUE reported textfile 2 1 TRUE
7566 rs10922098 T C T C -0.127 -0.009 0.40011 NA FALSE FALSE FALSE GCST006061 0.0075 0.2275 537409 outcome TRUE reported 11563_51_SYTL4_SYTL4 SYTL4 chr1 196695521 0.008137 6.502e-55 35329 TRUE reported textfile 2 1 TRUE
7567 rs1707652 T C T C 0.0873 0.0081 0.3102 NA FALSE FALSE FALSE GCST006061 0.0074 0.2701 537409 outcome TRUE reported 11563_51_SYTL4_SYTL4 SYTL4 chr3 165761011 0.0086 3.258e-24 35330 TRUE reported textfile 2 1 TRUE
7568 rs4665985 C A C A 0.0582 0.0065 0.23221 NA FALSE FALSE FALSE

GCST006061 0.0082 0.424 537409 outcome TRUE reported 11563_51_SYTL4_SYTL4 SYTL4 chr2 27531011 0.00939 5.706e-10 35364 TRUE reported textfile 2 1 TRUE

7569 rs704 A G A G 0.0734 -0.0155 0.49189 NA FALSE FALSE FALSE GCST006061 0.0072 0.03144 537409 outcome TRUE reported 11563_51_SYTL4_SYTL4 SYTL4 chr17 28367840 0.007985 3.828e-20 35351 TRUE reported textfile 2 1 TRUE

7570 rs71352239 T C T C 0.0648 -0.0041 0.30263 NA FALSE FALSE FALSE GCST006061 0.0087 0.6372 537409 outcome TRUE reported 11563_51_SYTL4_SYTL4 SYTL4 chr19 44926286 0.008712 1.02e-13 35359 TRUE reported textfile 2 1 TRUE

7571 rs10922098 T C T C -0.0572 -0.009 0.40011 NA FALSE FALSE FALSE GCST006061 0.0075 0.2275 537409 outcome TRUE reported 8055_33_DGCR2_IDD DGCR2 chr1 196695521 0.008046 1.17e-12 35338 TRUE reported textfile 2 1 TRUE

7572 rs34240868 G A G A 0.0591 0.0055 0.1681 NA FALSE FALSE FALSE GCST006061 0.0121 0.6477 537409 outcome TRUE reported 8055_33_DGCR2_IDD DGCR2 chr14 106550964 0.01078 4.199e-08 34966 TRUE reported textfile 2 1 TRUE

7573 rs6542680 T C T C -0.0609 -0.0012 0.18362 NA FALSE FALSE FALSE GCST006061 0.0091 0.8935 537409 outcome TRUE reported 8055_33_DGCR2_IDD DGCR2 chr2 3592552 0.010194 2.314e-09 35332 TRUE reported textfile 2 1 TRUE

7574 rs10922098 T C T C -0.1325 -0.009 0.40011 NA FALSE FALSE FALSE GCST006061 0.0075 0.2275 537409 outcome TRUE reported 13025_4_FRS2_FRS2 FRS2 chr1 196695521 0.008095 3.241e-60 35329 TRUE reported textfile 2 1 TRUE

7575 rs1355538 G A G A -0.0716 -0.0076 0.39847 NA FALSE FALSE FALSE GCST006061 0.0072 0.2923 537409 outcome TRUE reported 13025_4_FRS2_FRS2 FRS2 chr3 165787389 0.008104 9.973e-19 35329 TRUE reported textfile 2 1 TRUE

7576 rs704 A G A G 0.0505 -0.0155 0.49189 NA FALSE FALSE FALSE GCST006061 0.0072 0.03144 537409 outcome TRUE reported 13025_4_FRS2_FRS2 FRS2 chr17 28367840 0.00795 2.119e-10 35351 TRUE reported textfile 2 1 TRUE

7577 rs10922098 T C T C -0.0572 -0.009 0.40011 NA FALSE FALSE FALSE GCST006061 0.0075 0.2275 537409 outcome TRUE reported 14614_41_NCMAP_CA130 NCMAP chr1 196695521 0.008176 2.627e-12 35338 TRUE reported textfile 2 1 TRUE

7578 rs112095134 A G A G -0.2321 0.0076 0.18992 NA FALSE FALSE FALSE GCST006061 0.0093 0.4105 537409 outcome TRUE reported 14614_41_NCMAP_CA130 NCMAP chr9 92546517 0.010431 1.111e-109 35359 TRUE reported textfile 2 1 TRUE

7579 rs116853338 T C T C -0.1778 -0.0578 0.01608 NA FALSE FALSE FALSE GCST006061 0.0373 0.1207 537409 outcome TRUE reported 14614_41_NCMAP_CA130 NCMAP chr15 41371800 0.031784 2.219e-08 35364 TRUE reported textfile 2 1 TRUE

7580 rs117475080 A G A G 0.262 -0.0082 0.02864 NA FALSE FALSE FALSE GCST006061 0.0335 0.8056 537409 outcome TRUE reported 14614_41_NCMAP_CA130 NCMAP chr9 92054782 0.023708 2.161e-28 35358 TRUE reported textfile 2 1 TRUE

7581 rs140989966 G A G A -0.3752 0.0587 0.01203 NA FALSE FALSE FALSE GCST006061 0.0427 0.1687 537409 outcome TRUE reported 14614_41_NCMAP_CA130 NCMAP chr9 95519248 0.035956 1.714e-25 35345 TRUE reported textfile 2 1 TRUE

7582 rs150844304 C A C A -0.2539 0.0439 0.0276 NA FALSE FALSE FALSE GCST006061 0.0244 0.07203 537409 outcome TRUE reported 14614_41_NCMAP_CA130 NCMAP chr15 43434427 0.024292 1.432e-25 35363 TRUE reported textfile 2 1 TRUE

7583 rs17282057 C T C T -0.0993 -0.0212 0.09691 NA FALSE FALSE FALSE GCST006061 0.0109 0.05271 537409 outcome TRUE reported 14614_41_NCMAP_CA130 NCMAP chr3 124727230 0.013442 1.498e-13 35369 TRUE reported textfile 2 1 TRUE

7584 rs34971098 A G A G -0.3263 -0.042 0.01782 NA FALSE FALSE FALSE GCST006061 0.0359 0.242 537409 outcome TRUE reported 14614_41_NCMAP_CA130 NCMAP chr9 91185142 0.031561 4.71e-25 35358 TRUE reported textfile 2 1 TRUE

7585 rs3858097 G A G A -0.3149 0.0149 0.21817 NA FALSE FALSE FALSE
GCST006061 0.009 0.09665 537409 outcome TRUE reported 14614_41_NCMAP_CA130
NCMAP chr9 92189537 0.009712 1e-200 35359 TRUE reported textfile
2 1 TRUE
7586 rs4921915 A G A G -0.0741 0.0167 0.18763 NA FALSE FALSE FALSE
GCST006061 0.0083 0.04427 537409 outcome TRUE reported 14614_41_NCMAP_CA130
NCMAP chr8 18414956 0.010321 7.003e-13 35377 TRUE reported
textfile 2 1 TRUE
7587 rs61937764 T C T C 0.0826 -0.0243 0.08854 NA FALSE FALSE FALSE
GCST006061 0.0165 0.1405 537409 outcome TRUE reported 14614_41_NCMAP_CA130
NCMAP chr12 103542547 0.014337 8.352e-09 35367 TRUE reported
textfile 2 1 TRUE
7588 rs7139079 A G A G -0.0667 0.0044 0.38321 NA FALSE FALSE FALSE
GCST006061 0.0072 0.5391 537409 outcome TRUE reported 14614_41_NCMAP_CA130
NCMAP chr12 120977490 0.00822 4.878e-16 35365 TRUE reported textfile
2 1 TRUE
7589 rs72713764 C T C T -0.1875 -0.0161 0.01481 NA FALSE FALSE FALSE
GCST006061 0.0316 0.6106 537409 outcome TRUE reported 14614_41_NCMAP_CA130
NCMAP chr15 42755696 0.033064 1.421e-08 35363 TRUE reported
textfile 2 1 TRUE
7590 rs10922098 T C T C -0.2043 -0.009 0.40011 NA FALSE FALSE FALSE
GCST006061 0.0075 0.2275 537409 outcome TRUE reported 2708_54_TNFSF18_TNFSF18
TNFSF18 chr1 196695521 0.008112 6.012e-140 35338 TRUE reported
textfile 2 1 TRUE
7591 rs2686395 C T C T -0.1031 -0.0077 0.3101 NA FALSE FALSE FALSE
GCST006061 0.0074 0.2965 537409 outcome TRUE reported 2708_54_TNFSF18_TNFSF18
TNFSF18 chr3 165767231 0.008608 4.658e-33 35339 TRUE reported
textfile 2 1 TRUE
7592 rs10922098 T C T C -0.0549 -0.009 0.40011 NA FALSE FALSE FALSE
GCST006061 0.0075 0.2275 537409 outcome TRUE reported 4124_24_HSPA1A_HSP_70
HSPA1A chr1 196695521 0.008246 2.786e-11 35338 TRUE reported
textfile 2 1 TRUE
7593 rs11646715 A G A G 0.0483 -0.0204 0.47149 NA FALSE FALSE FALSE
GCST006061 0.0073 0.005107 537409 outcome TRUE reported
4124_24_HSPA1A_HSP_70 HSPA1A chr16 53790095 0.008082 2.284e-09 35367
TRUE reported textfile 2 1 TRUE
7594 rs72801474 A G A G -0.0737 0.0088 0.17476 NA FALSE FALSE FALSE
GCST006061 0.0138 0.522 537409 outcome TRUE reported 4124_24_HSPA1A_HSP_70
HSPA1A chr5 133108436 0.0107 5.653e-12 35347 TRUE reported textfile
2 1 TRUE
7595 rs10922098 T C T C -0.0912 -0.009 0.40011 NA FALSE FALSE FALSE
GCST006061 0.0075 0.2275 537409 outcome TRUE reported 3499_77_IL17B_IL_17B
IL17B chr1 196695521 0.008075 1.398e-29 35338 TRUE reported
textfile 2 1 TRUE
7596 rs6887452 G T G T 0.0717 0.0024 0.23656 NA FALSE FALSE FALSE
GCST006061 0.0077 0.7536 537409 outcome TRUE reported 3499_77_IL17B_IL_17B
IL17B chr5 149404204 0.0093 1.258e-14 35362 TRUE reported textfile
2 1 TRUE
7597 rs10922098 T C T C -0.1137 -0.009 0.40011 NA FALSE FALSE FALSE
GCST006061 0.0075 0.2275 537409 outcome TRUE reported 18947_3_LRRC3B_LRC3B
LRRC3B chr1 196695521 0.008016 1.163e-45 35338 TRUE reported
textfile 2 1 TRUE
7598 rs1707652 T C T C 0.0622 0.0081 0.3102 NA FALSE FALSE FALSE
GCST006061 0.0074 0.2701 537409 outcome TRUE reported 18947_3_LRRC3B_LRC3B
LRRC3B chr3 165761011 0.008486 2.303e-13 35339 TRUE reported
textfile 2 1 TRUE
7599 rs71352239 T C T C 0.0517 -0.0041 0.30263 NA FALSE FALSE FALSE
GCST006061 0.0087 0.6372 537409 outcome TRUE reported 18947_3_LRRC3B_LRC3B
LRRC3B chr19 44926286 0.008587 1.734e-09 35368 TRUE reported
textfile 2 1 TRUE
7600 rs10922098 T C T C -0.2157 -0.009 0.40011 NA FALSE FALSE FALSE
GCST006061 0.0075 0.2275 537409 outcome TRUE reported 12001_7_TJP1_ZO1
TJP1 chr1 196695521 0.008105 4.752e-156 35329 TRUE reported
textfile 2 1 TRUE
7601 rs111696008 A G A G -0.1457 -0.0259 0.02443 NA FALSE FALSE FALSE
GCST006061 0.0401 0.519 537409 outcome TRUE reported 12001_7_TJP1_ZO1

TJP1 chr2 216622986 0.026547 4.055e-08 35350 TRUE reported
textfile 2 1 TRUE
7602 rs1707652 T C T C 0.1106 0.0081 0.3102 NA FALSE FALSE FALSE
GCST006061 0.0074 0.2701 537409 outcome TRUE reported 12001_7_TJP1_ZO1
TJP1 chr3 165761011 0.008603 7.985e-38 35330 TRUE reported
textfile 2 1 TRUE
7603 rs10922098 T C T C -0.2208 -0.009 0.40011 NA FALSE FALSE FALSE
GCST006061 0.0075 0.2275 537409 outcome TRUE reported 11464_9_RELB_RELB
RELB chr1 196695521 0.008146 8.551e-162 35329 TRUE reported
textfile 2 1 TRUE
7604 rs114009540 A G A G -0.1214 0.0322 0.03606 NA FALSE FALSE FALSE
GCST006061 0.0299 0.2809 537409 outcome TRUE reported 11464_9_RELB_RELB
RELB chr1 196158413 0.021744 2.363e-08 35328 TRUE reported
textfile 2 1 TRUE
7605 rs704 A G A G 0.1623 -0.0155 0.49189 NA FALSE FALSE FALSE GCST006061
0.0072 0.03144 537409 outcome TRUE reported 11464_9_RELB_RELB RELB chr17
28367840 0.008005 2.173e-91 35351 TRUE reported textfile 2 1 TRUE
7606 rs10922098 T C T C -0.0935 -0.009 0.40011 NA FALSE FALSE FALSE
GCST006061 0.0075 0.2275 537409 outcome TRUE reported 12848_9_ARHGEF2_ARHG2
ARHGEF2 chr1 196695521 0.008094 7.251e-31 35329 TRUE reported
textfile 2 1 TRUE
7607 rs12995461 C A C A 0.0542 0.0086 0.21643 NA FALSE FALSE FALSE
GCST006061 0.008 0.2842 537409 outcome TRUE reported 12848_9_ARHGEF2_ARHG2
ARHGEF2 chr2 27555300 0.009552 1.394e-08 35364 TRUE reported
textfile 2 1 TRUE
7608 rs10922098 T C T C -0.0646 -0.009 0.40011 NA FALSE FALSE FALSE
GCST006061 0.0075 0.2275 537409 outcome TRUE reported 12018_84_TPK1_TPK1
TPK1 chr1 196695521 0.008114 1.704e-15 35329 TRUE reported
textfile 2 1 TRUE
7609 rs10922098 T C T C -0.0444 -0.009 0.40011 NA FALSE FALSE FALSE
GCST006061 0.0075 0.2275 537409 outcome TRUE reported 12650_43_GNAI3_GNAI3
GNAI3 chr1 196695521 0.008011 2.989e-08 35329 TRUE reported
textfile 2 1 TRUE
7610 rs1706435 G A G A 0.0527 0.0117 0.36981 NA FALSE FALSE FALSE
GCST006061 0.0072 0.1047 537409 outcome TRUE reported 12650_43_GNAI3_GNAI3
GNAI3 chr3 165743688 0.008128 8.934e-11 35330 TRUE reported
textfile 2 1 TRUE
7611 rs185009887 A G A G -0.1534 -0.0595 0.02519 NA FALSE FALSE FALSE
GCST006061 0.0496 0.2302 537409 outcome TRUE reported 12650_43_GNAI3_GNAI3
GNAI3 chr17 5848332 0.025456 1.679e-09 35365 TRUE reported textfile
2 1 TRUE
7612 rs7412 T C T C 0.2402 -0.0027 0.05941 NA FALSE FALSE FALSE GCST006061
0.0138 0.8459 537409 outcome TRUE reported 12650_43_GNAI3_GNAI3 GNAI3
chr19 44908822 0.016565 1.207e-47 35358 TRUE reported textfile 2
1 TRUE
7613 rs10922098 T C T C -0.0502 -0.009 0.40011 NA FALSE FALSE FALSE
GCST006061 0.0075 0.2275 537409 outcome TRUE reported 5615_62_FAM172A_F172A
FAM172A chr1 196695521 0.008118 6.248e-10 35338 TRUE reported
textfile 2 1 TRUE
7614 rs13165400 A G A G 0.0968 -0.0282 0.09572 NA FALSE FALSE FALSE
GCST006061 0.011 0.01031 537409 outcome TRUE reported 5615_62_FAM172A_F172A
FAM172A chr5 93755721 0.013653 1.343e-12 35342 TRUE reported
textfile 2 1 TRUE
7615 rs10922098 T C T C -0.077 -0.009 0.40011 NA FALSE FALSE FALSE
GCST006061 0.0075 0.2275 537409 outcome TRUE reported 19242_21_PFDN1_PFD1
PFDN1 chr1 196695521 0.008116 2.372e-21 35338 TRUE reported
textfile 2 1 TRUE
7616 rs10922098 T C T C -0.1206 -0.009 0.40011 NA FALSE FALSE FALSE
GCST006061 0.0075 0.2275 537409 outcome TRUE reported 7842_52_SERF1A_SERF1
SERF1A chr1 196695521 0.008127 8.147e-50 35338 TRUE reported
textfile 2 1 TRUE
7617 rs1355538 G A G A -0.0574 -0.0076 0.39847 NA FALSE FALSE FALSE
GCST006061 0.0072 0.2923 537409 outcome TRUE reported 7842_52_SERF1A_SERF1
SERF1A chr3 165787389 0.008139 1.755e-12 35338 TRUE reported
textfile 2 1 TRUE
7618 rs10922098 T C T C -0.1489 -0.009 0.40011 NA FALSE FALSE FALSE

GCST006061 0.0075 0.2275 537409 outcome TRUE reported 7767_1_CLPSL2_CF126
CLPSL2 chr1 196695521 0.00812 4.1e-75 35338 TRUE reported textfile 2
1 TRUE
7619 rs12478841 G A G A 0.0624 0.0049 0.21569 NA FALSE FALSE FALSE
GCST006061 0.008 0.5413 537409 outcome TRUE reported 7767_1_CLPSL2_CF126
CLPSL2 chr2 27588855 0.009613 8.504e-11 35373 TRUE reported
textfile 2 1 TRUE
7620 rs1707652 T C T C 0.0734 0.0081 0.3102 NA FALSE FALSE FALSE
GCST006061 0.0074 0.2701 537409 outcome TRUE reported 7767_1_CLPSL2_CF126
CLPSL2 chr3 165761011 0.00859 1.282e-17 35339 TRUE reported textfile
2 1 TRUE
7621 rs10922098 T C T C -0.1677 -0.009 0.40011 NA FALSE FALSE FALSE
GCST006061 0.0075 0.2275 537409 outcome TRUE reported 17743_14_UBE2A_UBE2A
UBE2A chr1 196695521 0.008041 1.372e-96 35338 TRUE reported
textfile 2 1 TRUE
7622 rs111696008 A G A G -0.1466 -0.0259 0.02443 NA FALSE FALSE FALSE
GCST006061 0.0401 0.519 537409 outcome TRUE reported 17743_14_UBE2A_UBE2A
UBE2A chr2 216622986 0.026263 2.378e-08 35359 TRUE reported
textfile 2 1 TRUE
7623 rs2686395 C T C T -0.1036 -0.0077 0.3101 NA FALSE FALSE FALSE
GCST006061 0.0074 0.2965 537409 outcome TRUE reported 17743_14_UBE2A_UBE2A
UBE2A chr3 165767231 0.008516 4.721e-34 35339 TRUE reported
textfile 2 1 TRUE
7624 rs10922098 T C T C -0.1507 -0.009 0.40011 NA FALSE FALSE FALSE
GCST006061 0.0075 0.2275 537409 outcome TRUE reported 7221_56_CKAP4_CKAP4
CKAP4 chr1 196695521 0.008055 4.188e-78 35338 TRUE reported
textfile 2 1 TRUE
7625 rs35209896 A C A C 0.0815 0.0267 0.09263 NA FALSE FALSE FALSE
GCST006061 0.0355 0.4521 537409 outcome TRUE reported 7221_56_CKAP4_CKAP4
CKAP4 chr6 32519009 0.013543 1.768e-09 35439 TRUE reported
textfile 2 1 TRUE
7626 rs6445035 A G A G 0.0758 0.0154 0.1904 NA FALSE FALSE FALSE
GCST006061 0.0089 0.08256 537409 outcome TRUE reported 7221_56_CKAP4_CKAP4
CKAP4 chr3 165762312 0.010053 4.693e-14 35339 TRUE reported
textfile 2 1 TRUE
7627 rs10922098 T C T C -0.165 -0.009 0.40011 NA FALSE FALSE FALSE
GCST006061 0.0075 0.2275 537409 outcome TRUE reported 8700_325_GALNT11_GLT11
GALNT11 chr1 196695521 0.008144 2.91e-91 35338 TRUE reported
textfile 2 1 TRUE
7628 rs1707652 T C T C 0.0801 0.0081 0.3102 NA FALSE FALSE FALSE
GCST006061 0.0074 0.2701 537409 outcome TRUE reported 8700_325_GALNT11_GLT11
GALNT11 chr3 165761011 0.00863 1.669e-20 35339 TRUE reported textfile
2 1 TRUE
7629 rs10922098 T C T C -0.0823 -0.009 0.40011 NA FALSE FALSE FALSE
GCST006061 0.0075 0.2275 537409 outcome TRUE reported 5610_32_FAM171A1_CJ038
FAM171A1 chr1 196695521 0.00803 1.189e-24 35338 TRUE reported
textfile 2 1 TRUE
7630 rs12411755 C T C T 0.1745 -0.0503 0.01768 NA FALSE FALSE FALSE
GCST006061 0.0336 0.1346 537409 outcome TRUE reported 5610_32_FAM171A1_CJ038
FAM171A1 chr10 15335971 0.030561 1.131e-08 35367 TRUE reported
textfile 2 1 TRUE
7631 rs10922098 T C T C -0.1647 -0.009 0.40011 NA FALSE FALSE FALSE
GCST006061 0.0075 0.2275 537409 outcome TRUE reported 11185_145_GCH1_GCH1
GCH1 chr1 196695521 0.008043 3.44e-93 35329 TRUE reported
textfile 2 1 TRUE
7632 rs2686395 C T C T -0.077 -0.0077 0.3101 NA FALSE FALSE FALSE
GCST006061 0.0074 0.2965 537409 outcome TRUE reported 11185_145_GCH1_GCH1
GCH1 chr3 165767231 0.008526 1.704e-19 35330 TRUE reported
textfile 2 1 TRUE
7633 rs35209896 A C A C 0.0857 0.0267 0.09263 NA FALSE FALSE FALSE
GCST006061 0.0355 0.4521 537409 outcome TRUE reported 11185_145_GCH1_GCH1
GCH1 chr6 32519009 0.013518 2.304e-10 35430 TRUE reported
textfile 2 1 TRUE
7634 rs10922098 T C T C -0.0898 -0.009 0.40011 NA FALSE FALSE FALSE
GCST006061 0.0075 0.2275 537409 outcome TRUE reported
10507_166_ACRV1_sperm_protein_10 ACRV1 chr1 196695521 0.008057 7.565e-29

35326 TRUE reported textfile 2 1 TRUE
7635 rs13379369 G A G A -0.1442 0.0108 0.15348 NA FALSE FALSE FALSE GCST006061 0.0182 0.5534 537409 outcome TRUE reported 10507_166_ACRV1_sperm_protein_10 ACRV1 chr14 105863077 0.011189 5.255e-38 34955 TRUE reported textfile 2 1 TRUE
7636 rs1355538 G A G A -0.0506 -0.0076 0.39847 NA FALSE FALSE FALSE GCST006061 0.0072 0.2923 537409 outcome TRUE reported 10507_166_ACRV1_sperm_protein_10 ACRV1 chr3 165787389 0.008054 3.325e-10 35326 TRUE reported textfile 2 1 TRUE
7637 rs10922098 T C T C -0.0869 -0.009 0.40011 NA FALSE FALSE FALSE GCST006061 0.0075 0.2275 537409 outcome TRUE reported 18894_1_GPX2_GPX2 GPX2 chr1 196695521 0.00801 2.028e-27 35338 TRUE reported textfile 2 1 TRUE
7638 rs10922098 T C T C -0.1216 -0.009 0.40011 NA FALSE FALSE FALSE GCST006061 0.0075 0.2275 537409 outcome TRUE reported 12445_50_ANKRD27_ANR27 ANKRD27 chr1 196695521 0.00811 8.006e-51 35329 TRUE reported textfile 2 1 TRUE
7639 rs1707652 T C T C 0.0873 0.0081 0.3102 NA FALSE FALSE FALSE GCST006061 0.0074 0.2701 537409 outcome TRUE reported 12445_50_ANKRD27_ANR27 ANKRD27 chr3 165761011 0.00857 2.27e-24 35330 TRUE reported textfile 2 1 TRUE
7640 rs17693106 G A G A 0.0522 0.0053 0.40227 NA FALSE FALSE FALSE GCST006061 0.0073 0.4692 537409 outcome TRUE reported 12445_50_ANKRD27_ANR27 ANKRD27 chr19 32634149 0.00812 1.289e-10 35360 TRUE reported textfile 2 1 TRUE
7641 rs9971407 T C T C -0.0532 -0.0126 0.24858 NA FALSE FALSE FALSE GCST006061 0.0086 0.1427 537409 outcome TRUE reported 12445_50_ANKRD27_ANR27 ANKRD27 chr11 65130754 0.009221 7.953e-09 35354 TRUE reported textfile 2 1 TRUE
7642 rs10922098 T C T C -0.0892 -0.009 0.40011 NA FALSE FALSE FALSE GCST006061 0.0075 0.2275 537409 outcome TRUE reported 4542_24_CLU_Clusterin CLU chr1 196695521 0.008392 2.191e-26 35338 TRUE reported textfile 2 1 TRUE
7643 rs2668202 G A G A -0.0641 -0.0076 0.30991 NA FALSE FALSE FALSE GCST006061 0.0074 0.3015 537409 outcome TRUE reported 4542_24_CLU_Clusterin CLU chr3 165772680 0.008864 4.768e-13 35339 TRUE reported textfile 2 1 TRUE
7644 rs602633 G T G T 0.0559 -0.0162 0.20391 NA FALSE FALSE FALSE GCST006061 0.0088 0.06452 537409 outcome TRUE reported 4542_24_CLU_Clusterin CLU chr1 109278889 0.010226 4.598e-08 35344 TRUE reported textfile 2 1 TRUE
7645 rs6073971 T C T C -0.0836 -0.0096 0.21924 NA FALSE FALSE FALSE GCST006061 0.0097 0.32 537409 outcome TRUE reported 4542_24_CLU_Clusterin CLU chr20 45961331 0.009914 3.392e-17 35370 TRUE reported textfile 2 1 TRUE
7646 rs704 A G A G 0.1051 -0.0155 0.49189 NA FALSE FALSE FALSE GCST006061 0.0072 0.03144 537409 outcome TRUE reported 4542_24_CLU_Clusterin CLU chr17 28367840 0.008216 1.816e-37 35360 TRUE reported textfile 2 1 TRUE
7647 rs75849644 G A G A -0.1299 -0.0239 0.05109 NA FALSE FALSE FALSE GCST006061 0.0169 0.1571 537409 outcome TRUE reported 4542_24_CLU_Clusterin CLU chr12 7052508 0.018844 5.45e-12 35390 TRUE reported textfile 2 1 TRUE
7648 rs10922098 T C T C -0.0996 -0.009 0.40011 NA FALSE FALSE FALSE GCST006061 0.0075 0.2275 537409 outcome TRUE reported 8815_1_OLFML1_OLFL1 OLFML1 chr1 196695521 0.008095 8.709e-35 35338 TRUE reported textfile 2 1 TRUE
7649 rs1355538 G A G A -0.0568 -0.0076 0.39847 NA FALSE FALSE FALSE GCST006061 0.0072 0.2923 537409 outcome TRUE reported 8815_1_OLFML1_OLFL1 OLFML1 chr3 165787389 0.008102 2.368e-12 35338 TRUE reported textfile 2 1 TRUE
7650 rs10922098 T C T C -0.0793 -0.009 0.40011 NA FALSE FALSE FALSE GCST006061 0.0075 0.2275 537409 outcome TRUE reported 12507_16_ITPKC_IP3KC ITPKC chr1 196695521 0.008089 1.086e-22 35329 TRUE reported textfile 2 1 TRUE
7651 rs10922098 T C T C -0.1126 -0.009 0.40011 NA FALSE FALSE FALSE GCST006061 0.0075 0.2275 537409 outcome TRUE reported 7199_3_SYN3_SYN3

SYN3 chr1 196695521 0.008147 1.893e-43 35338 TRUE reported
textfile 2 1 TRUE
7652 rs10922098 T C T C -0.1381 -0.009 0.40011 NA FALSE FALSE FALSE
GCST006061 0.0075 0.2275 537409 outcome TRUE reported 10643_16_OLFM3_NOE3
OLFM3 chr1 196695521 0.008011 1.354e-66 35326 TRUE reported
textfile 2 1 TRUE
7653 rs1707652 T C T C 0.0701 0.0081 0.3102 NA FALSE FALSE FALSE
GCST006061 0.0074 0.2701 537409 outcome TRUE reported 10643_16_OLFM3_NOE3
OLFM3 chr3 165761011 0.008484 1.424e-16 35327 TRUE reported
textfile 2 1 TRUE
7654 rs2384656 G A G A 0.0518 0.0053 0.21641 NA FALSE FALSE FALSE
GCST006061 0.0079 0.5066 537409 outcome TRUE reported 10643_16_OLFM3_NOE3
OLFM3 chr2 27609188 0.00946 4.36e-08 35361 TRUE reported textfile
2 1 TRUE
7655 rs704 A G A G 0.0668 -0.0155 0.49189 NA FALSE FALSE FALSE GCST006061
0.0072 0.03144 537409 outcome TRUE reported 10643_16_OLFM3_NOE3 OLFM3 chr17
28367840 0.007869 2.085e-17 35348 TRUE reported textfile 2 1 TRUE
7656 rs72749892 G A G A -0.1523 -0.0267 0.02117 NA FALSE FALSE FALSE
GCST006061 0.0185 0.1492 537409 outcome TRUE reported 10643_16_OLFM3_NOE3
OLFM3 chr15 92525256 0.027138 1.999e-08 35363 TRUE reported
textfile 2 1 TRUE
7657 rs10922098 T C T C -0.0638 -0.009 0.40011 NA FALSE FALSE FALSE
GCST006061 0.0075 0.2275 537409 outcome TRUE reported 10605_22_APMAP_APMAP
APMAP chr1 196695521 0.00808 2.873e-15 35326 TRUE reported textfile
2 1 TRUE
7658 rs1355538 G A G A -0.0715 -0.0076 0.39847 NA FALSE FALSE FALSE
GCST006061 0.0072 0.2923 537409 outcome TRUE reported 10605_22_APMAP_APMAP
APMAP chr3 165787389 0.008063 7.438e-19 35326 TRUE reported
textfile 2 1 TRUE
7659 rs6036977 T C T C -0.1558 0.0163 0.11921 NA FALSE FALSE FALSE
GCST006061 0.0112 0.1449 537409 outcome TRUE reported 10605_22_APMAP_APMAP
APMAP chr20 24921032 0.01203 2.32e-38 35354 TRUE reported textfile
2 1 TRUE
7660 rs6049886 T G T G -0.0727 0.0051 0.31953 NA FALSE FALSE FALSE
GCST006061 0.0076 0.5071 537409 outcome TRUE reported 10605_22_APMAP_APMAP
APMAP chr20 24702918 0.00853 1.559e-17 35354 TRUE reported textfile
2 1 TRUE
7661 rs10922098 T C T C -0.1678 -0.009 0.40011 NA FALSE FALSE FALSE
GCST006061 0.0075 0.2275 537409 outcome TRUE reported 12864_9_NIPAL4_NIPA4
NIPAL4 chr1 196695521 0.008081 9.047e-96 35329 TRUE reported
textfile 2 1 TRUE
7662 rs10922098 T C T C -0.0691 -0.009 0.40011 NA FALSE FALSE FALSE
GCST006061 0.0075 0.2275 537409 outcome TRUE reported 12363_70_TRIB2_TRIB2
TRIB2 chr1 196695521 0.008079 1.194e-17 35329 TRUE reported
textfile 2 1 TRUE
7663 rs697357 G A G A 0.054 0.0156 0.38884 NA FALSE FALSE FALSE
GCST006061 0.0072 0.03066 537409 outcome TRUE reported 12363_70_TRIB2_TRIB2
TRIB2 chr3 165718484 0.008106 2.702e-11 35330 TRUE reported
textfile 2 1 TRUE
7664 rs10922098 T C T C -0.0863 -0.009 0.40011 NA FALSE FALSE FALSE
GCST006061 0.0075 0.2275 537409 outcome TRUE reported 9532_5_WBP1L_WBP1L
WBP1L chr1 196695521 0.008016 5.008e-27 35338 TRUE reported
textfile 2 1 TRUE
7665 rs7651933 A G A G 0.0567 0.0169 0.24788 NA FALSE FALSE FALSE
GCST006061 0.0081 0.0377 537409 outcome TRUE reported 9532_5_WBP1L_WBP1L
WBP1L chr3 165750184 0.009115 4.947e-10 35339 TRUE reported
textfile 2 1 TRUE
7666 rs10922098 T C T C -0.0556 -0.009 0.40011 NA FALSE FALSE FALSE
GCST006061 0.0075 0.2275 537409 outcome TRUE reported 2939_10_ARTN_Artemin
ARTN chr1 196695521 0.008083 6.039e-12 35338 TRUE reported
textfile 2 1 TRUE
7667 rs7080536 A G A G -0.1322 -0.0145 0.04142 NA FALSE FALSE FALSE
GCST006061 0.0203 0.4753 537409 outcome TRUE reported 2939_10_ARTN_Artemin
ARTN chr10 113588287 0.019752 2.188e-11 35387 TRUE reported
textfile 2 1 TRUE
7668 rs77065856 G A G A 0.1242 0.0219 0.03371 NA FALSE FALSE FALSE

GCST006061 0.0248 0.3767 537409 outcome TRUE reported 2939_10_ARTN_Artemin
ARTN chr10 114017128 0.021649 9.645e-09 35384 TRUE reported
textfile 2 1 TRUE
7669 rs77774564 A G A G 0.3163 0.0637 0.01388 NA FALSE FALSE FALSE
GCST006061 0.0403 0.1143 537409 outcome TRUE reported 2939_10_ARTN_Artemin
ARTN chr10 113637445 0.033238 1.795e-21 35387 TRUE reported
textfile 2 1 TRUE
7670 rs10922098 T C T C -0.061 -0.009 0.40011 NA FALSE FALSE FALSE
GCST006061 0.0075 0.2275 537409 outcome TRUE reported
14713_46_AZU1_Azurocidin AZU1 chr1 196695521 0.008045 3.394e-14 35338
TRUE reported textfile 2 1 TRUE
7671 rs16881504 T C T C -0.0752 0.0109 0.09636 NA FALSE FALSE FALSE
GCST006061 0.0134 0.418 537409 outcome TRUE reported
14713_46_AZU1_Azurocidin AZU1 chr6 18935483 0.013218 1.277e-08 35375
TRUE reported textfile 2 1 TRUE
7672 rs62294359 A G A G 0.0641 0.0164 0.24788 NA FALSE FALSE FALSE
GCST006061 0.0081 0.04287 537409 outcome TRUE reported
14713_46_AZU1_Azurocidin AZU1 chr3 165744429 0.009131 2.219e-12 35339
TRUE reported textfile 2 1 TRUE
7673 rs10922098 T C T C -0.0661 -0.009 0.40011 NA FALSE FALSE FALSE
GCST006061 0.0075 0.2275 537409 outcome TRUE reported 3280_49_ACAN_Aggrecan
ACAN chr1 196695521 0.008191 7.013e-16 35338 TRUE reported
textfile 2 1 TRUE
7674 rs11144945 A C A C -0.0541 -0.0043 0.27054 NA FALSE FALSE FALSE
GCST006061 0.0083 0.607 537409 outcome TRUE reported 3280_49_ACAN_Aggrecan
ACAN chr9 76535950 0.008963 1.579e-09 35350 TRUE reported
textfile 2 1 TRUE
7675 rs12153751 T C T C -0.0543 0.0115 0.3298 NA FALSE FALSE FALSE
GCST006061 0.0076 0.1301 537409 outcome TRUE reported 3280_49_ACAN_Aggrecan
ACAN chr5 83697010 0.00856 2.245e-10 35342 TRUE reported textfile
2 1 TRUE
7676 rs34949187 A G A G -0.1415 -5.00E-04 0.16378 NA FALSE FALSE FALSE
GCST006061 0.01 0.9592 537409 outcome TRUE reported 3280_49_ACAN_Aggrecan
ACAN chr15 88843421 0.010752 1.491e-39 35355 TRUE reported
textfile 2 1 TRUE
7677 rs10922098 T C T C -0.1641 -0.009 0.40011 NA FALSE FALSE FALSE
GCST006061 0.0075 0.2275 537409 outcome TRUE reported 13596_3_RAB27B_RB27B
RAB27B chr1 196695521 0.008115 6.375e-91 35332 TRUE reported
textfile 2 1 TRUE
7678 rs1555405 A G A G -0.0485 -0.0047 0.30381 NA FALSE FALSE FALSE
GCST006061 0.0081 0.5592 537409 outcome TRUE reported 13596_3_RAB27B_RB27B
RAB27B chr14 100710432 0.00871 2.576e-08 35374 TRUE reported textfile
2 1 TRUE
7679 rs1706435 G A G A 0.0764 0.0117 0.36981 NA FALSE FALSE FALSE
GCST006061 0.0072 0.1047 537409 outcome TRUE reported 13596_3_RAB27B_RB27B
RAB27B chr3 165743688 0.008241 1.841e-20 35333 TRUE reported
textfile 2 1 TRUE
7680 rs10922098 T C T C -0.0627 -0.009 0.40011 NA FALSE FALSE FALSE
GCST006061 0.0075 0.2275 537409 outcome TRUE reported 5015_15_PLA2G7_PAFAH
PLA2G7 chr1 196695521 0.008654 4.328e-13 35338 TRUE reported
textfile 2 1 TRUE
7681 rs11057840 C A C A 0.0759 -0.0135 0.1158 NA FALSE FALSE FALSE
GCST006061 0.0103 0.1915 537409 outcome TRUE reported 5015_15_PLA2G7_PAFAH
PLA2G7 chr12 124831509 0.013295 1.137e-08 35380 TRUE reported
textfile 2 1 TRUE
7682 rs111266377 T G T G 0.2384 0.037 0.02883 NA FALSE FALSE FALSE
GCST006061 0.0258 0.1519 537409 outcome TRUE reported 5015_15_PLA2G7_PAFAH
PLA2G7 chr3 167239870 0.025401 6.257e-21 35354 TRUE reported
textfile 2 1 TRUE
7683 rs111696008 A G A G -0.2971 -0.0259 0.02443 NA FALSE FALSE FALSE
GCST006061 0.0401 0.519 537409 outcome TRUE reported 5015_15_PLA2G7_PAFAH
PLA2G7 chr2 216622986 0.028132 4.527e-26 35359 TRUE reported
textfile 2 1 TRUE
7684 rs11571943 C T C T -0.1562 -0.0198 0.02381 NA FALSE FALSE FALSE
GCST006061 0.0266 0.4568 537409 outcome TRUE reported 5015_15_PLA2G7_PAFAH
PLA2G7 chr6 44403953 0.027521 1.382e-08 35372 TRUE reported

textfile 2 1 TRUE
7685 rs11591147 T G T G -0.3413 0.0511 0.01208 NA FALSE FALSE FALSE
GCST006061 0.0356 0.1511 537409 outcome TRUE reported 5015_15_PLA2G7_PAFAH
PLA2G7 chr1 55039974 0.040074 1.64e-17 35363 TRUE reported
textfile 2 1 TRUE
7686 rs116824493 T G T G -0.459 0.0063 0.01249 NA FALSE FALSE FALSE
GCST006061 0.0479 0.8959 537409 outcome TRUE reported 5015_15_PLA2G7_PAFAH
PLA2G7 chr6 48287292 0.037994 1.333e-33 35359 TRUE reported
textfile 2 1 TRUE
7687 rs12026141 T C T C -0.0715 0.0094 0.31246 NA FALSE FALSE FALSE
GCST006061 0.0076 0.2137 537409 outcome TRUE reported 5015_15_PLA2G7_PAFAH
PLA2G7 chr1 201980465 0.009197 7.578e-15 35357 TRUE reported
textfile 2 1 TRUE
7688 rs139434889 T C T C -0.4692 -0.0044 0.01187 NA FALSE FALSE FALSE
GCST006061 0.0289 0.8795 537409 outcome TRUE reported 5015_15_PLA2G7_PAFAH
PLA2G7 chr6 45298410 0.040259 2.174e-31 35362 TRUE reported
textfile 2 1 TRUE
7689 rs143372081 G A G A -0.2078 0.0032 0.01578 NA FALSE FALSE FALSE
GCST006061 0.039 0.9354 537409 outcome TRUE reported 5015_15_PLA2G7_PAFAH
PLA2G7 chr6 54402010 0.034256 1.311e-09 35357 TRUE reported
textfile 2 1 TRUE
7690 rs144202860 A G A G -0.323 -0.0629 0.01274 NA FALSE FALSE FALSE
GCST006061 0.0538 0.2423 537409 outcome TRUE reported 5015_15_PLA2G7_PAFAH
PLA2G7 chr2 216625740 0.037685 1.026e-17 35359 TRUE reported
textfile 2 1 TRUE
7691 rs144311893 T C T C -0.3637 -0.0064 0.02146 NA FALSE FALSE FALSE
GCST006061 0.0312 0.8373 537409 outcome TRUE reported 5015_15_PLA2G7_PAFAH
PLA2G7 chr19 44920687 0.029465 5.291e-35 35368 TRUE reported
textfile 2 1 TRUE
7692 rs147033786 C A C A 0.3673 0.0651 0.01101 NA FALSE FALSE FALSE
GCST006061 0.05 0.1932 537409 outcome TRUE reported 5015_15_PLA2G7_PAFAH
PLA2G7 chr3 168506251 0.041572 9.989e-19 35354 TRUE reported
textfile 2 1 TRUE
7693 rs148077724 G A G A 0.3073 -0.0095 0.02611 NA FALSE FALSE FALSE
GCST006061 0.0214 0.6574 537409 outcome TRUE reported 5015_15_PLA2G7_PAFAH
PLA2G7 chr3 165952844 0.026504 4.392e-31 35340 TRUE reported
textfile 2 1 TRUE
7694 rs17145750 T C T C 0.0665 -0.0143 0.15023 NA FALSE FALSE FALSE
GCST006061 0.01 0.1504 537409 outcome TRUE reported 5015_15_PLA2G7_PAFAH
PLA2G7 chr7 73612048 0.011822 1.853e-08 35354 TRUE reported
textfile 2 1 TRUE
7695 rs17248720 T C T C -0.1481 0.0109 0.08884 NA FALSE FALSE FALSE
GCST006061 0.0116 0.347 537409 outcome TRUE reported 5015_15_PLA2G7_PAFAH
PLA2G7 chr19 11087511 0.014886 2.557e-23 35379 TRUE reported
textfile 2 1 TRUE
7696 rs174576 A C A C -0.0632 0.0105 0.3905 NA FALSE FALSE FALSE
GCST006061 0.0074 0.155 537409 outcome TRUE reported 5015_15_PLA2G7_PAFAH
PLA2G7 chr11 61836038 0.008653 2.805e-13 35367 TRUE reported
textfile 2 1 TRUE
7697 rs429358 C T C T 0.2526 0.005 0.17407 NA FALSE FALSE FALSE
GCST006061 0.0103 0.6319 537409 outcome TRUE reported 5015_15_PLA2G7_PAFAH
PLA2G7 chr19 44908684 0.011238 7.024e-112 35368 TRUE reported
textfile 2 1 TRUE
7698 rs4803776 C T C T -0.0549 0.0081 0.46804 NA FALSE FALSE FALSE
GCST006061 0.0074 0.2693 537409 outcome TRUE reported 5015_15_PLA2G7_PAFAH
PLA2G7 chr19 44949078 0.008727 3.164e-10 35366 TRUE reported
textfile 2 1 TRUE
7699 rs599839 A G A G 0.1 -0.0163 0.20777 NA FALSE FALSE FALSE GCST006061
0.0087 0.06014 537409 outcome TRUE reported 5015_15_PLA2G7_PAFAH PLA2G7
chr1 109279544 0.010462 1.197e-21 35344 TRUE reported textfile 2
1 TRUE
7700 rs62182252 A G A G -0.0748 -0.0018 0.33568 NA FALSE FALSE FALSE
GCST006061 0.0076 0.8157 537409 outcome TRUE reported 5015_15_PLA2G7_PAFAH
PLA2G7 chr2 203189874 0.009006 9.917e-17 35364 TRUE reported
textfile 2 1 TRUE
7701 rs6556405 C T C T 0.0649 -0.0069 0.2263 NA FALSE FALSE FALSE

GCST006061 0.0079 0.3844 537409 outcome TRUE reported 5015_15_PLA2G7_PAFAH
PLA2G7 chr5 159208094 0.010147 1.593e-10 35368 TRUE reported
textfile 2 1 TRUE
7702 rs71566584 C A C A 0.1739 -0.0026 0.0478 NA FALSE FALSE FALSE
GCST006061 0.0207 0.8986 537409 outcome TRUE reported 5015_15_PLA2G7_PAFAH
PLA2G7 chr6 46633844 0.019828 1.779e-18 35359 TRUE reported
textfile 2 1 TRUE
7703 rs934197 A G A G 0.0691 0.0055 0.35055 NA FALSE FALSE FALSE
GCST006061 0.0079 0.4819 537409 outcome TRUE reported 5015_15_PLA2G7_PAFAH
PLA2G7 chr2 21044589 0.008916 9.183e-15 35373 TRUE reported
textfile 2 1 TRUE
7704 rs9881140 C T C T 0.1239 -0.0034 0.05228 NA FALSE FALSE FALSE
GCST006061 0.0155 0.8262 537409 outcome TRUE reported 5015_15_PLA2G7_PAFAH
PLA2G7 chr3 169738610 0.019361 1.558e-10 35359 TRUE reported
textfile 2 1 TRUE
7705 rs10922098 T C T C -0.063 -0.009 0.40011 NA FALSE FALSE FALSE
GCST006061 0.0075 0.2275 537409 outcome TRUE reported 6525_17_DUSP13_DUSP13
DUSP13 chr1 196695521 0.008165 1.201e-14 35338 TRUE reported
textfile 2 1 TRUE
7706 rs1707652 T C T C 0.0619 0.0081 0.3102 NA FALSE FALSE FALSE
GCST006061 0.0074 0.2701 537409 outcome TRUE reported 6525_17_DUSP13_DUSP13
DUSP13 chr3 165761011 0.008608 6.417e-13 35339 TRUE reported
textfile 2 1 TRUE
7707 rs2299258 A G A G 0.1899 -0.0059 0.15543 NA FALSE FALSE FALSE
GCST006061 0.0108 0.5866 537409 outcome TRUE reported 6525_17_DUSP13_DUSP13
DUSP13 chr7 95313605 0.011039 2.529e-66 35349 TRUE reported
textfile 2 1 TRUE
7708 rs10922098 T C T C -0.1968 -0.009 0.40011 NA FALSE FALSE FALSE
GCST006061 0.0075 0.2275 537409 outcome TRUE reported 9786_310_IER3_IEX1
IER3 chr1 196695521 0.008073 2.894e-131 35338 TRUE reported
textfile 2 1 TRUE
7709 rs11569415 A G A G -0.0548 0.0011 0.24276 NA FALSE FALSE FALSE
GCST006061 0.0097 0.9129 537409 outcome TRUE reported 9786_310_IER3_IEX1
IER3 chr19 6716268 0.009226 2.857e-09 35372 TRUE reported textfile
2 1 TRUE
7710 rs1707652 T C T C 0.0896 0.0081 0.3102 NA FALSE FALSE FALSE
GCST006061 0.0074 0.2701 537409 outcome TRUE reported 9786_310_IER3_IEX1
IER3 chr3 165761011 0.008565 1.306e-25 35339 TRUE reported
textfile 2 1 TRUE
7711 rs35351085 T C T C 0.1024 0.0061 0.04877 NA FALSE FALSE FALSE
GCST006061 0.022 0.7825 537409 outcome TRUE reported 9786_310_IER3_IEX1
IER3 chr1 197992799 0.018351 2.405e-08 35343 TRUE reported
textfile 2 1 TRUE
7712 rs10922098 T C T C -0.1222 -0.009 0.40011 NA FALSE FALSE FALSE
GCST006061 0.0075 0.2275 537409 outcome TRUE reported 11537_12_TFR2_TFR2
TFR2 chr1 196695521 0.007995 9.816e-53 35329 TRUE reported
textfile 2 1 TRUE
7713 rs113099710 A C A C 0.1763 -0.0013 0.01668 NA FALSE FALSE FALSE
GCST006061 0.0338 0.9698 537409 outcome TRUE reported 11537_12_TFR2_TFR2
TFR2 chr5 39328054 0.030096 4.688e-09 35357 TRUE reported
textfile 2 1 TRUE
7714 rs1355538 G A G A -0.0556 -0.0076 0.39847 NA FALSE FALSE FALSE
GCST006061 0.0072 0.2923 537409 outcome TRUE reported 11537_12_TFR2_TFR2
TFR2 chr3 165787389 0.007997 3.585e-12 35329 TRUE reported
textfile 2 1 TRUE
7715 rs60805730 G A G A 0.1557 -0.0079 0.0212 NA FALSE FALSE FALSE
GCST006061 0.0315 0.8023 537409 outcome TRUE reported 11537_12_TFR2_TFR2
TFR2 chr5 39424061 0.027483 1.467e-08 35355 TRUE reported
textfile 2 1 TRUE
7716 rs10922098 T C T C -0.0914 -0.009 0.40011 NA FALSE FALSE FALSE
GCST006061 0.0075 0.2275 537409 outcome TRUE reported 4158_54_PLAU_uPA
PLAU chr1 196695521 0.008429 2.139e-27 35338 TRUE reported
textfile 2 1 TRUE
7717 rs12570183 T C T C -0.1664 0.0071 0.02866 NA FALSE FALSE FALSE
GCST006061 0.0223 0.7493 537409 outcome TRUE reported 4158_54_PLAU_uPA
PLAU chr10 70884567 0.02487 2.219e-11 35376 TRUE reported textfile

2 1 TRUE
7718 rs143501783 C T C T 0.2129 -0.0333 0.01116 NA FALSE FALSE FALSE
GCST006061 0.0443 0.4525 537409 outcome TRUE reported 4158_54_PLAU_uPA
PLAU chr17 7897001 0.037557 1.439e-08 35373 TRUE reported textfile
2 1 TRUE
7719 rs147611419 C T C T -0.4593 -0.016 0.02834 NA FALSE FALSE FALSE
GCST006061 0.0442 0.7176 537409 outcome TRUE reported 4158_54_PLAU_uPA
PLAU chr10 72054316 0.025305 1.264e-73 35373 TRUE reported
textfile 2 1 TRUE
7720 rs150026080 T C T C -0.4302 0.0368 0.04294 NA FALSE FALSE FALSE
GCST006061 0.0381 0.3344 537409 outcome TRUE reported 4158_54_PLAU_uPA
PLAU chr10 73596323 0.020705 6.851e-96 35370 TRUE reported
textfile 2 1 TRUE
7721 rs192913987 A G A G 0.261 -0.023 0.03149 NA FALSE FALSE FALSE
GCST006061 0.0417 0.5805 537409 outcome TRUE reported 4158_54_PLAU_uPA
PLAU chr10 74110955 0.023685 3.078e-28 35369 TRUE reported
textfile 2 1 TRUE
7722 rs2302524 C T C T -0.1797 0.0208 0.17537 NA FALSE FALSE FALSE
GCST006061 0.0098 0.03386 537409 outcome TRUE reported 4158_54_PLAU_uPA
PLAU chr19 43652320 0.010902 4.812e-61 35369 TRUE reported
textfile 2 1 TRUE
7723 rs2470854 G A G A -0.0493 -0.0016 0.45355 NA FALSE FALSE FALSE
GCST006061 0.0073 0.8314 537409 outcome TRUE reported 4158_54_PLAU_uPA
PLAU chr3 98985392 0.008325 3.179e-09 35331 TRUE reported
textfile 2 1 TRUE
7724 rs36229204 T C T C -0.3555 0 0.02865 NA FALSE FALSE FALSE GCST006061
0.0222 0.9997 537409 outcome TRUE reported 4158_54_PLAU_uPA PLAU chr19
43671830 0.024523 1.273e-47 35368 TRUE reported textfile 2 1 TRUE
7725 rs55714927 T C T C 0.0943 1e-04 0.21166 NA FALSE FALSE FALSE
GCST006061 0.0098 0.9926 537409 outcome TRUE reported 4158_54_PLAU_uPA
PLAU chr17 7176997 0.010167 1.769e-20 35370 TRUE reported textfile
2 1 TRUE
7726 rs75350427 A G A G -0.2084 0.0044 0.02318 NA FALSE FALSE FALSE
GCST006061 0.0327 0.8939 537409 outcome TRUE reported 4158_54_PLAU_uPA
PLAU chr10 70915073 0.028352 1.976e-13 35375 TRUE reported
textfile 2 1 TRUE
7727 rs78290332 G A G A -0.2952 -0.0546 0.01325 NA FALSE FALSE FALSE
GCST006061 0.0312 0.07951 537409 outcome TRUE reported 4158_54_PLAU_uPA
PLAU chr10 75250009 0.036635 7.768e-16 35364 TRUE reported
textfile 2 1 TRUE
7728 rs8176743 T C T C 0.1079 0.017 0.06488 NA FALSE FALSE FALSE
GCST006061 0.0128 0.1834 537409 outcome TRUE reported 4158_54_PLAU_uPA
PLAU chr9 133256028 0.016821 1.411e-10 35370 TRUE reported
textfile 2 1 TRUE
7729 rs9948409 G A G A 0.0744 -0.0013 0.34814 NA FALSE FALSE FALSE
GCST006061 0.0079 0.8709 537409 outcome TRUE reported 4158_54_PLAU_uPA
PLAU chr18 27181964 0.008634 6.856e-18 35367 TRUE reported
textfile 2 1 TRUE
7730 rs10922098 T C T C -0.1739 -0.009 0.40011 NA FALSE FALSE FALSE
GCST006061 0.0075 0.2275 537409 outcome TRUE reported 2760_2_FAM107A_DRR1
FAM107A chr1 196695521 0.008166 1.225e-100 35338 TRUE reported
textfile 2 1 TRUE
7731 rs1706435 G A G A 0.0728 0.0117 0.36981 NA FALSE FALSE FALSE
GCST006061 0.0072 0.1047 537409 outcome TRUE reported 2760_2_FAM107A_DRR1
FAM107A chr3 165743688 0.008298 1.742e-18 35339 TRUE reported
textfile 2 1 TRUE
7732 rs10922098 T C T C -0.1314 -0.009 0.40011 NA FALSE FALSE FALSE
GCST006061 0.0075 0.2275 537409 outcome TRUE reported 17432_25_AK4_KAD4 AK4
chr1 196695521 0.008144 1.481e-58 35338 TRUE reported textfile 2
1 TRUE
7733 rs11569415 A G A G -0.06 0.0011 0.24276 NA FALSE FALSE FALSE
GCST006061 0.0097 0.9129 537409 outcome TRUE reported 17432_25_AK4_KAD4 AK4
chr19 6716268 0.009273 9.761e-11 35372 TRUE reported textfile 2 1
TRUE
7734 rs1555405 A G A G -0.0515 -0.0047 0.30381 NA FALSE FALSE FALSE
GCST006061 0.0081 0.5592 537409 outcome TRUE reported 17432_25_AK4_KAD4 AK4

chr14 100710432 0.008736 3.742e-09 35380 TRUE reported textfile 2
1 TRUE
7735 rs1707652 T C T C 0.1085 0.0081 0.3102 NA FALSE FALSE FALSE
GCST006061 0.0074 0.2701 537409 outcome TRUE reported 17432_25_AK4_KAD4 AK4
chr3 165761011 0.008609 2.021e-36 35339 TRUE reported textfile 2
1 TRUE
7736 rs10922098 T C T C -0.0531 -0.009 0.40011 NA FALSE FALSE FALSE
GCST006061 0.0075 0.2275 537409 outcome TRUE reported 17510_7_SRP14_SRP14
SRP14 chr1 196695521 0.008009 3.354e-11 35338 TRUE reported
textfile 2 1 TRUE
7737 rs185009887 A G A G -0.1474 -0.0595 0.02519 NA FALSE FALSE FALSE
GCST006061 0.0496 0.2302 537409 outcome TRUE reported 17510_7_SRP14_SRP14
SRP14 chr17 5848332 0.025444 6.907e-09 35374 TRUE reported textfile
2 1 TRUE
7738 rs62269916 A G A G 0.119 -0.0234 0.03471 NA FALSE FALSE FALSE
GCST006061 0.0238 0.3259 537409 outcome TRUE reported 17510_7_SRP14_SRP14
SRP14 chr3 74348841 0.021494 3.088e-08 35343 TRUE reported
textfile 2 1 TRUE
7739 rs79740491 T C T C -0.1587 -0.0794 0.01941 NA FALSE FALSE FALSE
GCST006061 0.0405 0.04973 537409 outcome TRUE reported 17510_7_SRP14_SRP14
SRP14 chr12 118347982 0.028989 4.387e-08 35375 TRUE reported
textfile 2 1 TRUE
7740 rs10922098 T C T C -0.0461 -0.009 0.40011 NA FALSE FALSE FALSE
GCST006061 0.0075 0.2275 537409 outcome TRUE reported 15504_39_BIRC7_Livin_B
BIRC7 chr1 196695521 0.008167 1.658e-08 35338 TRUE reported
textfile 2 1 TRUE
7741 rs146457388 G A G A 0.1262 -0.0375 0.03747 NA FALSE FALSE FALSE
GCST006061 0.0273 0.1701 537409 outcome TRUE reported 15504_39_BIRC7_Livin_B
BIRC7 chr19 10181296 0.021306 3.155e-09 35375 TRUE reported
textfile 2 1 TRUE
7742 rs6542680 T C T C -0.2601 -0.0012 0.18362 NA FALSE FALSE FALSE
GCST006061 0.0091 0.8935 537409 outcome TRUE reported 15504_39_BIRC7_Livin_B
BIRC7 chr2 3592552 0.010282 3.395e-141 35332 TRUE reported textfile
2 1 TRUE
7743 rs10922098 T C T C -0.0679 -0.009 0.40011 NA FALSE FALSE FALSE
GCST006061 0.0075 0.2275 537409 outcome TRUE reported 19170_25_POLE3_DPOE3
POLE3 chr1 196695521 0.008064 3.773e-17 35338 TRUE reported
textfile 2 1 TRUE
7744 rs34481430 G A G A -0.2137 -0.0053 0.02819 NA FALSE FALSE FALSE
GCST006061 0.0193 0.7839 537409 outcome TRUE reported 19170_25_POLE3_DPOE3
POLE3 chr8 140518126 0.023909 3.959e-19 35324 TRUE reported
textfile 2 1 TRUE
7745 rs6445035 A G A G 0.1113 0.0154 0.1904 NA FALSE FALSE FALSE
GCST006061 0.0089 0.08256 537409 outcome TRUE reported 19170_25_POLE3_DPOE3
POLE3 chr3 165762312 0.010031 1.31e-28 35339 TRUE reported
textfile 2 1 TRUE
7746 rs10922098 T C T C -0.1071 -0.009 0.40011 NA FALSE FALSE FALSE
GCST006061 0.0075 0.2275 537409 outcome TRUE reported 14149_9_IL36B_IL_1F8
IL36B chr1 196695521 0.008168 2.833e-39 35338 TRUE reported
textfile 2 1 TRUE
7747 rs1355538 G A G A -0.057 -0.0076 0.39847 NA FALSE FALSE FALSE
GCST006061 0.0072 0.2923 537409 outcome TRUE reported 14149_9_IL36B_IL_1F8
IL36B chr3 165787389 0.008166 2.947e-12 35338 TRUE reported
textfile 2 1 TRUE
7748 rs10922098 T C T C -0.0761 -0.009 0.40011 NA FALSE FALSE FALSE
GCST006061 0.0075 0.2275 537409 outcome TRUE reported 6622_90_APLN_APEL
APLN chr1 196695521 0.008186 1.455e-20 35338 TRUE reported
textfile 2 1 TRUE
7749 rs2806894 T C T C -0.0498 -0.0031 0.2839 NA FALSE FALSE FALSE
GCST006061 0.0077 0.6856 537409 outcome TRUE reported 6622_90_APLN_APEL
APLN chr13 45897526 0.008895 2.164e-08 35360 TRUE reported
textfile 2 1 TRUE
7750 rs9562634 A C A C 0.1771 0.0057 0.34012 NA FALSE FALSE FALSE
GCST006061 0.0076 0.4536 537409 outcome TRUE reported 6622_90_APLN_APEL
APLN chr13 46068729 0.008471 4.595e-97 35361 TRUE reported
textfile 2 1 TRUE

7751 rs10922098 T C T C -0.1101 -0.009 0.40011 NA FALSE FALSE FALSE GCST006061 0.0075 0.2275 537409 outcome TRUE reported 2993_1_IL18RAP_IL_18_Rb IL18RAP chr1 196695521 0.008143 1.168e-41 35338 TRUE reported textfile 2 1 TRUE
7752 rs1355538 G A G A -0.0519 -0.0076 0.39847 NA FALSE FALSE FALSE GCST006061 0.0072 0.2923 537409 outcome TRUE reported 2993_1_IL18RAP_IL_18_Rb IL18RAP chr3 165787389 0.008145 1.87e-10 35338 TRUE reported textfile 2 1 TRUE
7753 rs10922098 T C T C -0.0864 -0.009 0.40011 NA FALSE FALSE FALSE GCST006061 0.0075 0.2275 537409 outcome TRUE reported 5089_11_IL7R_IL_7_Ra IL7R chr1 196695521 0.007992 3.048e-27 35338 TRUE reported textfile 2 1 TRUE
7754 rs1706435 G A G A 0.0549 0.0117 0.36981 NA FALSE FALSE FALSE GCST006061 0.0072 0.1047 537409 outcome TRUE reported 5089_11_IL7R_IL_7_Ra IL7R chr3 165743688 0.008105 1.259e-11 35339 TRUE reported textfile 2 1 TRUE
7755 rs6542680 T C T C -0.0584 -0.0012 0.18362 NA FALSE FALSE FALSE GCST006061 0.0091 0.8935 537409 outcome TRUE reported 5089_11_IL7R_IL_7_Ra IL7R chr2 3592552 0.010132 8.226e-09 35332 TRUE reported textfile 2 1 TRUE
7756 rs10922098 T C T C -0.0507 -0.009 0.40011 NA FALSE FALSE FALSE GCST006061 0.0075 0.2275 537409 outcome TRUE reported 3622_33_LGMN_LGMN LGMN chr1 196695521 0.008391 1.522e-09 35338 TRUE reported textfile 2 1 TRUE
7757 rs11111000 T C T C -0.1077 0.0219 0.11118 NA FALSE FALSE FALSE GCST006061 0.0123 0.07411 537409 outcome TRUE reported 3622_33_LGMN_LGMN LGMN chr12 101756445 0.013279 5.035e-16 35364 TRUE reported textfile 2 1 TRUE
7758 rs117317284 T C T C 0.2727 0.0155 0.01706 NA FALSE FALSE FALSE GCST006061 0.0327 0.6358 537409 outcome TRUE reported 3622_33_LGMN_LGMN LGMN chr12 101665112 0.032415 4.006e-17 35363 TRUE reported textfile 2 1 TRUE
7759 rs148659834 A G A G -0.5082 -0.0458 0.01341 NA FALSE FALSE FALSE GCST006061 0.0382 0.2301 537409 outcome TRUE reported 3622_33_LGMN_LGMN LGMN chr14 92709697 0.037531 8.971e-42 35362 TRUE reported textfile 2 1 TRUE
7760 rs2250644 T C T C 0.0495 -0.0046 0.37467 NA FALSE FALSE FALSE GCST006061 0.0075 0.5456 537409 outcome TRUE reported 3622_33_LGMN_LGMN LGMN chr10 89249122 0.008479 5.295e-09 35353 TRUE reported textfile 2 1 TRUE
7761 rs35036246 C A C A 0.0656 -0.0158 0.14785 NA FALSE FALSE FALSE GCST006061 0.0101 0.1194 537409 outcome TRUE reported 3622_33_LGMN_LGMN LGMN chr16 73171973 0.011488 1.127e-08 35368 TRUE reported textfile 2 1 TRUE
7762 rs4561280 A C A C -0.0487 -0.0047 0.3343 NA FALSE FALSE FALSE GCST006061 0.0093 0.615 537409 outcome TRUE reported 3622_33_LGMN_LGMN LGMN chr12 8345474 0.008751 2.625e-08 35376 TRUE reported textfile 2 1 TRUE
7763 rs4883201 G A G A 0.2331 -0.0182 0.12502 NA FALSE FALSE FALSE GCST006061 0.0114 0.1094 537409 outcome TRUE reported 3622_33_LGMN_LGMN LGMN chr12 8929985 0.012409 9.944e-79 35387 TRUE reported textfile 2 1 TRUE
7764 rs75747979 A G A G -0.1963 -0.0083 0.01837 NA FALSE FALSE FALSE GCST006061 0.0358 0.816 537409 outcome TRUE reported 3622_33_LGMN_LGMN LGMN chr14 93178752 0.031113 2.803e-10 35366 TRUE reported textfile 2 1 TRUE
7765 rs10922098 T C T C -0.045 -0.009 0.40011 NA FALSE FALSE FALSE GCST006061 0.0075 0.2275 537409 outcome TRUE reported 8360_169_NCR1_NKp46 NCR1 chr1 196695521 0.008186 3.852e-08 35338 TRUE reported textfile 2 1 TRUE
7766 rs196904 T C T C 0.0645 -0.0054 0.15941 NA FALSE FALSE FALSE GCST006061 0.0094 0.5616 537409 outcome TRUE reported 8360_169_NCR1_NKp46 NCR1 chr17 64042195 0.011007 4.628e-09 35365 TRUE reported textfile 2 1 TRUE
7767 rs3184504 C T C T -0.1005 0.0074 0.37862 NA FALSE FALSE FALSE GCST006061 0.0075 0.3235 537409 outcome TRUE reported 8360_169_NCR1_NKp46

NCR1 chr12 111446804 0.008262 4.834e-34 35371 TRUE reported
textfile 2 1 TRUE
7768 rs875063 C A C A -0.0846 -5.00E-04 0.28107 NA FALSE FALSE FALSE
GCST006061 0.0076 0.9486 537409 outcome TRUE reported 8360_169_NCR1_NKp46
NCR1 chr2 111055508 0.008896 1.905e-21 35359 TRUE reported
textfile 2 1 TRUE
7769 rs10922098 T C T C -0.1379 -0.009 0.40011 NA FALSE FALSE FALSE
GCST006061 0.0075 0.2275 537409 outcome TRUE reported 18177_49_NAE1_ULA1
NAE1 chr1 196695521 0.008112 8.207e-65 35338 TRUE reported
textfile 2 1 TRUE
7770 rs10922098 T C T C -0.2199 -0.009 0.40011 NA FALSE FALSE FALSE
GCST006061 0.0075 0.2275 537409 outcome TRUE reported 14197_2_PRDM1_PRDM1
PRDM1 chr1 196695521 0.008136 6.746e-161 35338 TRUE reported
textfile 2 1 TRUE
7771 rs1707652 T C T C 0.114 0.0081 0.3102 NA FALSE FALSE FALSE
GCST006061 0.0074 0.2701 537409 outcome TRUE reported 14197_2_PRDM1_PRDM1
PRDM1 chr3 165761011 0.008635 8.489e-40 35339 TRUE reported
textfile 2 1 TRUE
7772 rs35351085 T C T C 0.1023 0.0061 0.04877 NA FALSE FALSE FALSE
GCST006061 0.022 0.7825 537409 outcome TRUE reported 14197_2_PRDM1_PRDM1
PRDM1 chr1 197992799 0.018525 3.346e-08 35343 TRUE reported
textfile 2 1 TRUE
7773 rs10922098 T C T C -0.16 -0.009 0.40011 NA FALSE FALSE FALSE
GCST006061 0.0075 0.2275 537409 outcome TRUE reported 14334_3_RCVRN_RECO
RCVRN chr1 196695521 0.008086 3.861e-87 35338 TRUE reported
textfile 2 1 TRUE
7774 rs111696008 A G A G -0.1492 -0.0259 0.02443 NA FALSE FALSE FALSE
GCST006061 0.0401 0.519 537409 outcome TRUE reported 14334_3_RCVRN_RECO
RCVRN chr2 216622986 0.026396 1.582e-08 35359 TRUE reported
textfile 2 1 TRUE
7775 rs1707652 T C T C 0.0979 0.0081 0.3102 NA FALSE FALSE FALSE
GCST006061 0.0074 0.2701 537409 outcome TRUE reported 14334_3_RCVRN_RECO
RCVRN chr3 165761011 0.008568 3.076e-30 35339 TRUE reported
textfile 2 1 TRUE
7776 rs2232613 T C T C -0.092 0.0151 0.0989 NA FALSE FALSE FALSE
GCST006061 0.0141 0.2834 537409 outcome TRUE reported 14334_3_RCVRN_RECO
RCVRN chr20 38369011 0.013108 2.238e-12 35375 TRUE reported
textfile 2 1 TRUE
7777 rs10922098 T C T C -0.1584 -0.009 0.40011 NA FALSE FALSE FALSE
GCST006061 0.0075 0.2275 537409 outcome TRUE reported 14006_36_GNMT_GNMT
GNMT chr1 196695521 0.008049 3.306e-86 35338 TRUE reported
textfile 2 1 TRUE
7778 rs1355538 G A G A -0.0651 -0.0076 0.39847 NA FALSE FALSE FALSE
GCST006061 0.0072 0.2923 537409 outcome TRUE reported 14006_36_GNMT_GNMT
GNMT chr3 165787389 0.008058 6.545e-16 35338 TRUE reported
textfile 2 1 TRUE
7779 rs6940814 G A G A -0.2195 0.0109 0.3708 NA FALSE FALSE FALSE
GCST006061 0.0073 0.1335 537409 outcome TRUE reported 14006_36_GNMT_GNMT
GNMT chr6 42957194 0.0081 1.033e-161 35368 TRUE reported textfile
2 1 TRUE
7780 rs9472021 T C T C -0.2133 0.0103 0.03386 NA FALSE FALSE FALSE
GCST006061 0.0266 0.6974 537409 outcome TRUE reported 14006_36_GNMT_GNMT
GNMT chr6 43170986 0.022629 4.266e-21 35367 TRUE reported
textfile 2 1 TRUE
7781 rs10922098 T C T C -0.1316 -0.009 0.40011 NA FALSE FALSE FALSE
GCST006061 0.0075 0.2275 537409 outcome TRUE reported
15688_30_LMAN1_Mannose_binding_lectin LMAN1 chr1 196695521 0.008137
7.713e-59 35338 TRUE reported textfile 2 1 TRUE
7782 rs12133641 G A G A 0.0755 0.0382 0.41476 NA FALSE FALSE FALSE
GCST006061 0.0073 1.459e-07 537409 outcome TRUE reported
15688_30_LMAN1_Mannose_binding_lectin LMAN1 chr1 154455807 0.008152
2.011e-20 35364 TRUE reported textfile 2 1 TRUE
7783 rs1706435 G A G A 0.074 0.0117 0.36981 NA FALSE FALSE FALSE
GCST006061 0.0072 0.1047 537409 outcome TRUE reported
15688_30_LMAN1_Mannose_binding_lectin LMAN1 chr3 165743688 0.008258
3.226e-19 35339 TRUE reported textfile 2 1 TRUE

7784 rs10922098 T C T C -0.0952 -0.009 0.40011 NA FALSE FALSE FALSE GCST006061 0.0075 0.2275 537409 outcome TRUE reported 6597_24_FAM174A_TM157 FAM174A chr1 196695521 0.00827 1.155e-30 35338 TRUE reported textfile 2 1 TRUE
7785 rs112291863 C T C T -0.2244 -0.0619 0.01928 NA FALSE FALSE FALSE GCST006061 0.0475 0.1928 537409 outcome TRUE reported 6597_24_FAM174A_TM157 FAM174A chr10 86130155 0.030247 1.181e-13 35364 TRUE reported textfile 2 1 TRUE
7786 rs117342095 C T C T -0.1641 -0.0235 0.03742 NA FALSE FALSE FALSE GCST006061 0.0479 0.6238 537409 outcome TRUE reported 6597_24_FAM174A_TM157 FAM174A chr10 88451781 0.022028 9.354e-14 35357 TRUE reported textfile 2 1 TRUE
7787 rs12254574 A G A G -0.6177 -0.0145 0.08697 NA FALSE FALSE FALSE GCST006061 0.013 0.266 537409 outcome TRUE reported 6597_24_FAM174A_TM157 FAM174A chr10 88672181 0.012385 1e-200 35357 TRUE reported textfile 2 1 TRUE
7788 rs1706435 G A G A 0.0658 0.0117 0.36981 NA FALSE FALSE FALSE GCST006061 0.0072 0.1047 537409 outcome TRUE reported 6597_24_FAM174A_TM157 FAM174A chr3 165743688 0.008396 4.602e-15 35339 TRUE reported textfile 2 1 TRUE
7789 rs4933164 G A G A 0.2559 0.037 0.02548 NA FALSE FALSE FALSE GCST006061 0.0327 0.258 537409 outcome TRUE reported 6597_24_FAM174A_TM157 FAM174A chr10 89871489 0.026206 1.592e-22 35360 TRUE reported textfile 2 1 TRUE
7790 rs74611276 G A G A -0.176 0.0045 0.0305 NA FALSE FALSE FALSE GCST006061 0.0267 0.8652 537409 outcome TRUE reported 6597_24_FAM174A_TM157 FAM174A chr10 85756870 0.024134 3.043e-13 35365 TRUE reported textfile 2 1 TRUE
7791 rs77835145 A C A C -0.189 -0.0045 0.03517 NA FALSE FALSE FALSE GCST006061 0.0298 0.8811 537409 outcome TRUE reported 6597_24_FAM174A_TM157 FAM174A chr10 89139988 0.022305 2.384e-17 35355 TRUE reported textfile 2 1 TRUE
7792 rs78932638 C T C T -0.2659 -0.0233 0.03278 NA FALSE FALSE FALSE GCST006061 0.025 0.3514 537409 outcome TRUE reported 6597_24_FAM174A_TM157 FAM174A chr10 88158549 0.023173 1.767e-30 35356 TRUE reported textfile 2 1 TRUE
7793 rs10922098 T C T C -0.1124 -0.009 0.40011 NA FALSE FALSE FALSE GCST006061 0.0075 0.2275 537409 outcome TRUE reported 3299_29_CNTN5_Contactin_5 CNTN5 chr1 196695521 0.008102 9.275e-44 35338 TRUE reported textfile 2 1 TRUE
7794 rs11223396 T C T C 0.0897 0.0059 0.23052 NA FALSE FALSE FALSE GCST006061 0.0086 0.4965 537409 outcome TRUE reported 3299_29_CNTN5_Contactin_5 CNTN5 chr11 100248127 0.00945 2.275e-21 35351 TRUE reported textfile 2 1 TRUE
7795 rs4325882 C A C A 0.0452 4e-04 0.43261 NA FALSE FALSE FALSE GCST006061 0.0072 0.9553 537409 outcome TRUE reported 3299_29_CNTN5_Contactin_5 CNTN5 chr3 166124518 0.008007 1.651e-08 35340 TRUE reported textfile 2 1 TRUE
7796 rs4528296 T C T C -0.4257 0.0172 0.07442 NA FALSE FALSE FALSE GCST006061 0.0144 0.2299 537409 outcome TRUE reported 3299_29_CNTN5_Contactin_5 CNTN5 chr11 99222947 0.015189 7.616e-173 35355 TRUE reported textfile 2 1 TRUE
7797 rs4754290 C T C T 0.0723 -0.0036 0.32611 NA FALSE FALSE FALSE GCST006061 0.0075 0.626 537409 outcome TRUE reported 3299_29_CNTN5_Contactin_5 CNTN5 chr11 97733281 0.008536 2.462e-17 35364 TRUE reported textfile 2 1 TRUE
7798 rs10922098 T C T C -0.1918 -0.009 0.40011 NA FALSE FALSE FALSE GCST006061 0.0075 0.2275 537409 outcome TRUE reported 11372_2_ZNF18_ZNF18 ZNF18 chr1 196695521 0.008085 2.126e-124 35329 TRUE reported textfile 2 1 TRUE
7799 rs1707652 T C T C 0.124 0.0081 0.3102 NA FALSE FALSE FALSE GCST006061 0.0074 0.2701 537409 outcome TRUE reported 11372_2_ZNF18_ZNF18 ZNF18 chr3 165761011 0.008568 1.829e-47 35330 TRUE reported textfile 2 1 TRUE
7800 rs3926704 A G A G 0.0475 7e-04 0.32404 NA FALSE FALSE FALSE GCST006061 0.0078 0.9316 537409 outcome TRUE reported 11372_2_ZNF18_ZNF18

ZNF18 chr3 167076696 0.008505 2.337e-08 35345 TRUE reported textfile 2 1 TRUE
7801 rs10922098 T C T C -0.0659 -0.009 0.40011 NA FALSE FALSE FALSE GCST006061 0.0075 0.2275 537409 outcome TRUE reported 18859_7_EIF4EBP3_4EBP3 EIF4EBP3 chr1 196695521 0.007917 8.51e-17 35338 TRUE reported textfile 2 1 TRUE
7802 rs28645249 C T C T 0.0547 0.019 0.26162 NA FALSE FALSE FALSE GCST006061 0.0115 0.09815 537409 outcome TRUE reported 18859_7_EIF4EBP3_4EBP3 EIF4EBP3 chr6 32557458 0.008498 1.218e-10 35438 TRUE reported textfile 2 1 TRUE
7803 rs10922098 T C T C -0.2172 -0.009 0.40011 NA FALSE FALSE FALSE GCST006061 0.0075 0.2275 537409 outcome TRUE reported 6527_1_TRIL_TRIL TRIL chr1 196695521 0.008254 1.332e-152 35338 TRUE reported textfile 2 1 TRUE
7804 rs1707652 T C T C 0.1121 0.0081 0.3102 NA FALSE FALSE FALSE GCST006061 0.0074 0.2701 537409 outcome TRUE reported 6527_1_TRIL_TRIL TRIL chr3 165761011 0.008753 1.497e-37 35339 TRUE reported textfile 2 1 TRUE
7805 rs221187 G A G A -0.1064 0.0088 0.11233 NA FALSE FALSE FALSE GCST006061 0.0103 0.3934 537409 outcome TRUE reported 6527_1_TRIL_TRIL TRIL chr7 28972654 0.012859 1.294e-16 35370 TRUE reported textfile 2 1 TRUE
7806 rs704 A G A G 0.1308 -0.0155 0.49189 NA FALSE FALSE FALSE GCST006061 0.0072 0.03144 537409 outcome TRUE reported 6527_1_TRIL_TRIL TRIL chr17 28367840 0.008114 1.856e-58 35360 TRUE reported textfile 2 1 TRUE
7807 rs7630163 A C A C 0.0462 0.0184 0.44201 NA FALSE FALSE FALSE GCST006061 0.0075 0.01463 537409 outcome TRUE reported 6527_1_TRIL_TRIL TRIL chr3 194343569 0.008216 1.875e-08 35299 TRUE reported textfile 2 1 TRUE
7808 rs10922098 T C T C -0.0549 -0.009 0.40011 NA FALSE FALSE FALSE GCST006061 0.0075 0.2275 537409 outcome TRUE reported 15574_37_CCNA2_Cyclin_A CCNA2 chr1 196695521 0.008061 9.738e-12 35338 TRUE reported textfile 2 1 TRUE
7809 rs6542680 T C T C -0.0988 -0.0012 0.18362 NA FALSE FALSE FALSE GCST006061 0.0091 0.8935 537409 outcome TRUE reported 15574_37_CCNA2_Cyclin_A CCNA2 chr2 3592552 0.010212 3.849e-22 35332 TRUE reported textfile 2 1 TRUE
7810 rs10922098 T C T C -0.0642 -0.009 0.40011 NA FALSE FALSE FALSE GCST006061 0.0075 0.2275 537409 outcome TRUE reported 9916_146_LRRC4B_LRC4B LRRC4B chr1 196695521 0.007982 8.74e-16 35338 TRUE reported textfile 2 1 TRUE
7811 rs12974169 A G A G 0.0502 0.0028 0.46846 NA FALSE FALSE FALSE GCST006061 0.008 0.7295 537409 outcome TRUE reported 9916_146_LRRC4B_LRC4B LRRC4B chr19 50553597 0.00784 1.526e-10 35372 TRUE reported textfile 2 1 TRUE
7812 rs181596081 A C A C -0.2514 0.0705 0.01921 NA FALSE FALSE FALSE GCST006061 0.0529 0.1823 537409 outcome TRUE reported 9916_146_LRRC4B_LRC4B LRRC4B chr19 50547802 0.028274 6.032e-19 35373 TRUE reported textfile 2 1 TRUE
7813 rs2686395 C T C T -0.0517 -0.0077 0.3101 NA FALSE FALSE FALSE GCST006061 0.0074 0.2965 537409 outcome TRUE reported 9916_146_LRRC4B_LRC4B LRRC4B chr3 165767231 0.008435 8.811e-10 35339 TRUE reported textfile 2 1 TRUE
7814 rs10922098 T C T C -0.0583 -0.009 0.40011 NA FALSE FALSE FALSE GCST006061 0.0075 0.2275 537409 outcome TRUE reported 16057_6_IGF2R_IGF_II_receptor IGF2R chr1 196695521 0.008597 1.193e-11 35338 TRUE reported textfile 2 1 TRUE
7815 rs117534432 T C T C 0.248 -0.029 0.03547 NA FALSE FALSE FALSE GCST006061 0.0247 0.241 537409 outcome TRUE reported 16057_6_IGF2R_IGF_II_receptor IGF2R chr6 160756411 0.023554 6.337e-26 35390 TRUE reported textfile 2 1 TRUE
7816 rs12984330 C A C A -0.0518 0.0102 0.44602 NA FALSE FALSE FALSE GCST006061 0.0073 0.1644 537409 outcome TRUE reported 16057_6_IGF2R_IGF_II_receptor IGF2R chr19 18165166 0.008471 9.673e-10 35353 TRUE reported textfile 2 1 TRUE
7817 rs148234538 A G A G 0.265 -0.0084 0.01393 NA FALSE FALSE FALSE

GCST006061 0.0387 0.829 537409 outcome TRUE reported
16057_6_IGF2R_IGF_II_receptor IGF2R chr6 160949921 0.036769 5.714e-13
35387 TRUE reported textfile 2 1 TRUE
7818 rs1805248 G A G A 0.0821 0.0232 0.22071 NA FALSE FALSE FALSE
GCST006061 0.0086 0.007041 537409 outcome TRUE reported
16057_6_IGF2R_IGF_II_receptor IGF2R chr12 8948541 0.010188 7.717e-16 35387
TRUE reported textfile 2 1 TRUE
7819 rs2065168 C T C T -0.0549 -0.0215 0.28352 NA FALSE FALSE FALSE
GCST006061 0.0079 0.006283 537409 outcome TRUE reported
16057_6_IGF2R_IGF_II_receptor IGF2R chr6 125870587 0.009549 8.972e-09
35358 TRUE reported textfile 2 1 TRUE
7820 rs76889468 A G A G -0.2373 -0.0176 0.03205 NA FALSE FALSE FALSE
GCST006061 0.0377 0.6406 537409 outcome TRUE reported
16057_6_IGF2R_IGF_II_receptor IGF2R chr12 101764986 0.024732 8.394e-22
35364 TRUE reported textfile 2 1 TRUE
7821 rs7978801 C T C T 0.0606 -0.0073 0.1881 NA FALSE FALSE FALSE
GCST006061 0.0088 0.4057 537409 outcome TRUE reported
16057_6_IGF2R_IGF_II_receptor IGF2R chr12 101916338 0.010754 1.748e-08
35364 TRUE reported textfile 2 1 TRUE
7822 rs10922098 T C T C -0.0475 -0.009 0.40011 NA FALSE FALSE FALSE
GCST006061 0.0075 0.2275 537409 outcome TRUE reported 6276_16_FURIN_Furin
FURIN chr1 196695521 0.008152 5.655e-09 35338 TRUE reported
textfile 2 1 TRUE
7823 rs4639017 C T C T -0.0469 -0.0061 0.39283 NA FALSE FALSE FALSE
GCST006061 0.0073 0.4022 537409 outcome TRUE reported 6276_16_FURIN_Furin
FURIN chr3 165833296 0.008146 8.533e-09 35339 TRUE reported
textfile 2 1 TRUE
7824 rs10922098 T C T C -0.0514 -0.009 0.40011 NA FALSE FALSE FALSE
GCST006061 0.0075 0.2275 537409 outcome TRUE reported 14227_21_MYL6B_MYL6B
MYL6B chr1 196695521 0.008292 5.698e-10 35338 TRUE reported
textfile 2 1 TRUE
7825 rs115975731 A G A G 0.2394 0.005 0.02486 NA FALSE FALSE FALSE
GCST006061 0.0239 0.836 537409 outcome TRUE reported 14227_21_MYL6B_MYL6B
MYL6B chr2 88831873 0.026061 4.07e-20 35350 TRUE reported
textfile 2 1 TRUE
7826 rs1355538 G A G A -0.0658 -0.0076 0.39847 NA FALSE FALSE FALSE
GCST006061 0.0072 0.2923 537409 outcome TRUE reported 14227_21_MYL6B_MYL6B
MYL6B chr3 165787389 0.008271 1.784e-15 35338 TRUE reported
textfile 2 1 TRUE
7827 rs10922098 T C T C -0.0578 -0.009 0.40011 NA FALSE FALSE FALSE
GCST006061 0.0075 0.2275 537409 outcome TRUE reported 15466_30_COL9A1_CO9A1
COL9A1 chr1 196695521 0.007938 3.308e-13 35338 TRUE reported
textfile 2 1 TRUE
7828 rs3806095 A G A G -0.0729 -0.0094 0.27104 NA FALSE FALSE FALSE
GCST006061 0.0078 0.2301 537409 outcome TRUE reported 15466_30_COL9A1_CO9A1
COL9A1 chr6 70278273 0.00867 4.154e-17 35338 TRUE reported textfile
2 1 TRUE
7829 rs6455357 A G A G -0.0767 0.0019 0.0887 NA FALSE FALSE FALSE
GCST006061 0.0107 0.8578 537409 outcome TRUE reported 15466_30_COL9A1_CO9A1
COL9A1 chr6 70242278 0.013723 2.284e-08 35338 TRUE reported
textfile 2 1 TRUE
7830 rs10922098 T C T C -0.0769 -0.009 0.40011 NA FALSE FALSE FALSE
GCST006061 0.0075 0.2275 537409 outcome TRUE reported 2906_55_IL4_IL_4 IL4
chr1 196695521 0.008142 3.57e-21 35338 TRUE reported textfile 2
1 TRUE
7831 rs6542680 T C T C -0.0612 -0.0012 0.18362 NA FALSE FALSE FALSE
GCST006061 0.0091 0.8935 537409 outcome TRUE reported 2906_55_IL4_IL_4 IL4
chr2 3592552 0.010313 2.95e-09 35332 TRUE reported textfile 2 1
TRUE
7832 rs10922098 T C T C -0.1554 -0.009 0.40011 NA FALSE FALSE FALSE
GCST006061 0.0075 0.2275 537409 outcome TRUE reported 11164_7_TENM2_TEN2
TENM2 chr1 196695521 0.008063 8.823e-83 35329 TRUE reported
textfile 2 1 TRUE
7833 rs1355538 G A G A -0.0749 -0.0076 0.39847 NA FALSE FALSE FALSE
GCST006061 0.0072 0.2923 537409 outcome TRUE reported 11164_7_TENM2_TEN2
TENM2 chr3 165787389 0.008068 1.641e-20 35329 TRUE reported

textfile 2 1 TRUE
7834 rs35209896 A C A C 0.0861 0.0267 0.09263 NA FALSE FALSE FALSE
GCST006061 0.0355 0.4521 537409 outcome TRUE reported 11164_7_TENM2_TEN2
TENM2 chr6 32519009 0.013547 2.077e-10 35430 TRUE reported
textfile 2 1 TRUE
7835 rs10922098 T C T C -0.063 -0.009 0.40011 NA FALSE FALSE FALSE
GCST006061 0.0075 0.2275 537409 outcome TRUE reported 2618_10_ERBB4_ERBB4
ERBB4 chr1 196695521 0.007973 2.747e-15 35338 TRUE reported
textfile 2 1 TRUE
7836 rs117265578 A G A G -0.0814 0.0127 0.08612 NA FALSE FALSE FALSE
GCST006061 0.0183 0.4854 537409 outcome TRUE reported 2618_10_ERBB4_ERBB4
ERBB4 chr14 49522779 0.013799 3.657e-09 35362 TRUE reported
textfile 2 1 TRUE
7837 rs9902706 T C T C -0.103 -0.05 0.05353 NA FALSE FALSE FALSE
GCST006061 0.0152 0.0009747 537409 outcome TRUE reported 2618_10_ERBB4_ERBB4
ERBB4 chr17 66190870 0.017627 5.12e-09 35370 TRUE reported
textfile 2 1 TRUE
7838 rs10922098 T C T C -0.0974 -0.009 0.40011 NA FALSE FALSE FALSE
GCST006061 0.0075 0.2275 537409 outcome TRUE reported 17462_19_IRF1_IRF1
IRF1 chr1 196695521 0.008061 1.304e-33 35338 TRUE reported
textfile 2 1 TRUE
7839 rs10922098 T C T C -0.0737 -0.009 0.40011 NA FALSE FALSE FALSE
GCST006061 0.0075 0.2275 537409 outcome TRUE reported 14670_1_SKIL_SKIL
SKIL chr1 196695521 0.008255 4.328e-19 35338 TRUE reported
textfile 2 1 TRUE
7840 rs1707652 T C T C 0.0728 0.0081 0.3102 NA FALSE FALSE FALSE
GCST006061 0.0074 0.2701 537409 outcome TRUE reported 14670_1_SKIL_SKIL
SKIL chr3 165761011 0.008716 6.684e-17 35339 TRUE reported
textfile 2 1 TRUE
7841 rs10922098 T C T C -0.1395 -0.009 0.40011 NA FALSE FALSE FALSE
GCST006061 0.0075 0.2275 537409 outcome TRUE reported 8366_19_C1orf115_CA115
C1orf115 chr1 196695521 0.008131 5.514e-66 35338 TRUE reported
textfile 2 1 TRUE
7842 rs10922098 T C T C -0.1876 -0.009 0.40011 NA FALSE FALSE FALSE
GCST006061 0.0075 0.2275 537409 outcome TRUE reported 2649_77_ICAM3_sICAM_3
ICAM3 chr1 196695521 0.008096 8.971e-119 35338 TRUE reported
textfile 2 1 TRUE
7843 rs11569415 A G A G -0.0713 0.0011 0.24276 NA FALSE FALSE FALSE
GCST006061 0.0097 0.9129 537409 outcome TRUE reported 2649_77_ICAM3_sICAM_3
ICAM3 chr19 6716268 0.009244 1.227e-14 35372 TRUE reported textfile
2 1 TRUE
7844 rs2686395 C T C T -0.0736 -0.0077 0.3101 NA FALSE FALSE FALSE
GCST006061 0.0074 0.2965 537409 outcome TRUE reported 2649_77_ICAM3_sICAM_3
ICAM3 chr3 165767231 0.008585 1.011e-17 35339 TRUE reported
textfile 2 1 TRUE
7845 rs10922098 T C T C -0.099 -0.009 0.40011 NA FALSE FALSE FALSE
GCST006061 0.0075 0.2275 537409 outcome TRUE reported 10074_128_BRD2_BRD2
BRD2 chr1 196695521 0.008064 1.206e-34 35326 TRUE reported
textfile 2 1 TRUE
7846 rs10922098 T C T C -0.0615 -0.009 0.40011 NA FALSE FALSE FALSE
GCST006061 0.0075 0.2275 537409 outcome TRUE reported 7790_21_LIF_LIF LIF
chr1 196695521 0.00806 2.343e-14 35338 TRUE reported textfile 2 1
TRUE
7847 rs6542680 T C T C -0.1044 -0.0012 0.18362 NA FALSE FALSE FALSE
GCST006061 0.0091 0.8935 537409 outcome TRUE reported 7790_21_LIF_LIF LIF
chr2 3592552 0.010205 1.448e-24 35332 TRUE reported textfile 2 1
TRUE
7848 rs10922098 T C T C -0.0506 -0.009 0.40011 NA FALSE FALSE FALSE
GCST006061 0.0075 0.2275 537409 outcome TRUE reported 7045_4_BNIP3_BNIP3
BNIP3 chr1 196695521 0.008033 2.99e-10 35338 TRUE reported
textfile 2 1 TRUE
7849 rs1354034 C T C T 0.0549 0.0088 0.38119 NA FALSE FALSE FALSE
GCST006061 0.0073 0.2248 537409 outcome TRUE reported 7045_4_BNIP3_BNIP3
BNIP3 chr3 56815721 0.008082 1.102e-11 35356 TRUE reported
textfile 2 1 TRUE
7850 rs6444156 A G A G 0.0873 -0.0028 0.25196 NA FALSE FALSE FALSE

GCST006061 0.0081 0.732 537409 outcome TRUE reported 7045_4_BNIP3_BNIP3
BNIP3 chr3 186679685 0.009024 3.895e-22 35368 TRUE reported
textfile 2 1 TRUE
7851 rs10922098 T C T C -0.1425 -0.009 0.40011 NA FALSE FALSE FALSE
GCST006061 0.0075 0.2275 537409 outcome TRUE reported 18255_6_CYB5R1_NB5R1
CYB5R1 chr1 196695521 0.008045 3.333e-70 35338 TRUE reported
textfile 2 1 TRUE
7852 rs1706435 G A G A 0.0731 0.0117 0.36981 NA FALSE FALSE FALSE
GCST006061 0.0072 0.1047 537409 outcome TRUE reported 18255_6_CYB5R1_NB5R1
CYB5R1 chr3 165743688 0.008174 3.797e-19 35339 TRUE reported
textfile 2 1 TRUE
7853 rs6542680 T C T C -0.0621 -0.0012 0.18362 NA FALSE FALSE FALSE
GCST006061 0.0091 0.8935 537409 outcome TRUE reported 18255_6_CYB5R1_NB5R1
CYB5R1 chr2 3592552 0.010235 1.301e-09 35332 TRUE reported textfile
2 1 TRUE
7854 rs10922098 T C T C -0.0541 -0.009 0.40011 NA FALSE FALSE FALSE
GCST006061 0.0075 0.2275 537409 outcome TRUE reported 7224_11_TRAT1_TRAT1
TRAT1 chr1 196695521 0.008118 2.665e-11 35338 TRUE reported
textfile 2 1 TRUE
7855 rs11574452 A C A C -0.1426 0.0147 0.02727 NA FALSE FALSE FALSE
GCST006061 0.0173 0.3972 537409 outcome TRUE reported 7224_11_TRAT1_TRAT1
TRAT1 chr4 73980944 0.025051 1.253e-08 35351 TRUE reported
textfile 2 1 TRUE
7856 rs7080386 A C A C 0.0504 -0.0468 0.43502 NA FALSE FALSE FALSE
GCST006061 0.0072 7.735e-11 537409 outcome TRUE reported 7224_11_TRAT1_TRAT1
TRAT1 chr10 63288546 0.008016 3.222e-10 35351 TRUE reported
textfile 2 1 TRUE
7857 rs10922098 T C T C -0.1218 -0.009 0.40011 NA FALSE FALSE FALSE
GCST006061 0.0075 0.2275 537409 outcome TRUE reported 2952_75_IGF1_IGF_I
IGF1 chr1 196695521 0.00828 5.601e-49 35338 TRUE reported textfile
2 1 TRUE
7858 rs1113480 C A C A 0.0503 -0.001 0.27584 NA FALSE FALSE FALSE
GCST006061 0.0082 0.902 537409 outcome TRUE reported 2952_75_IGF1_IGF_I
IGF1 chr11 48036761 0.009027 2.514e-08 35366 TRUE reported
textfile 2 1 TRUE
7859 rs17205365 G A G A -0.0497 0.0137 0.30458 NA FALSE FALSE FALSE
GCST006061 0.0083 0.09632 537409 outcome TRUE reported 2952_75_IGF1_IGF_I
IGF1 chr15 62047927 0.008841 1.893e-08 35376 TRUE reported
textfile 2 1 TRUE
7860 rs2686395 C T C T -0.0588 -0.0077 0.3101 NA FALSE FALSE FALSE
GCST006061 0.0074 0.2965 537409 outcome TRUE reported 2952_75_IGF1_IGF_I
IGF1 chr3 165767231 0.008755 1.869e-11 35339 TRUE reported
textfile 2 1 TRUE
7861 rs74004837 G A G A -0.378 0.0019 0.01114 NA FALSE FALSE FALSE
GCST006061 0.0437 0.9656 537409 outcome TRUE reported 2952_75_IGF1_IGF_I
IGF1 chr16 1168321 0.039336 7.295e-22 35311 TRUE reported textfile
2 1 TRUE
7862 rs924140 T C T C 0.0533 0.0181 0.38261 NA FALSE FALSE FALSE
GCST006061 0.0072 0.01196 537409 outcome TRUE reported 2952_75_IGF1_IGF_I
IGF1 chr7 45923515 0.008353 1.757e-10 35347 TRUE reported
textfile 2 1 TRUE
7863 rs9806893 G A G A -0.212 0.0034 0.02061 NA FALSE FALSE FALSE
GCST006061 0.022 0.8782 537409 outcome TRUE reported 2952_75_IGF1_IGF_I
IGF1 chr16 1903648 0.027626 1.667e-14 35321 TRUE reported textfile
2 1 TRUE
7864 rs9874429 C T C T -0.0492 0.0189 0.30877 NA FALSE FALSE FALSE
GCST006061 0.0079 0.01615 537409 outcome TRUE reported 2952_75_IGF1_IGF_I
IGF1 chr3 172469758 0.00881 2.339e-08 35358 TRUE reported textfile
2 1 TRUE
7865 rs9930725 A G A G 0.1375 -0.0403 0.08527 NA FALSE FALSE FALSE
GCST006061 0.0124 0.001184 537409 outcome TRUE reported 2952_75_IGF1_IGF_I
IGF1 chr16 1794175 0.014662 6.743e-21 35321 TRUE reported textfile
2 1 TRUE
7866 rs10922098 T C T C -0.1019 -0.009 0.40011 NA FALSE FALSE FALSE
GCST006061 0.0075 0.2275 537409 outcome TRUE reported 7815_49_IGFBPL1_IBPL1
IGFBPL1 chr1 196695521 0.008043 8.76e-37 35338 TRUE reported

textfile 2 1 TRUE
7867 rs1355538 G A G A -0.044 -0.0076 0.39847 NA FALSE FALSE FALSE
GCST006061 0.0072 0.2923 537409 outcome TRUE reported 7815_49_IGFBPL1_IBPL1
IGFBPL1 chr3 165787389 0.008043 4.481e-08 35338 TRUE reported
textfile 2 1 TRUE
7868 rs10922098 T C T C -0.1311 -0.009 0.40011 NA FALSE FALSE FALSE
GCST006061 0.0075 0.2275 537409 outcome TRUE reported 12738_43_NISCH_NISCH
NISCH chr1 196695521 0.008158 4.147e-58 35329 TRUE reported
textfile 2 1 TRUE
7869 rs1355538 G A G A -0.0629 -0.0076 0.39847 NA FALSE FALSE FALSE
GCST006061 0.0072 0.2923 537409 outcome TRUE reported 12738_43_NISCH_NISCH
NISCH chr3 165787389 0.008157 1.248e-14 35329 TRUE reported
textfile 2 1 TRUE
7870 rs10922098 T C T C -0.1598 -0.009 0.40011 NA FALSE FALSE FALSE
GCST006061 0.0075 0.2275 537409 outcome TRUE reported 7142_5_CPN1_CBPN
CPN1 chr1 196695521 0.008067 2.448e-87 35338 TRUE reported
textfile 2 1 TRUE
7871 rs1707652 T C T C 0.0833 0.0081 0.3102 NA FALSE FALSE FALSE
GCST006061 0.0074 0.2701 537409 outcome TRUE reported 7142_5_CPN1_CBPN
CPN1 chr3 165761011 0.008551 1.995e-22 35339 TRUE reported
textfile 2 1 TRUE
7872 rs10922098 T C T C -0.1365 -0.009 0.40011 NA FALSE FALSE FALSE
GCST006061 0.0075 0.2275 537409 outcome TRUE reported 11312_40_PMS2_PMS2
PMS2 chr1 196695521 0.008086 6.188e-64 35329 TRUE reported
textfile 2 1 TRUE
7873 rs1707652 T C T C 0.074 0.0081 0.3102 NA FALSE FALSE FALSE
GCST006061 0.0074 0.2701 537409 outcome TRUE reported 11312_40_PMS2_PMS2
PMS2 chr3 165761011 0.008559 5.345e-18 35330 TRUE reported
textfile 2 1 TRUE
7874 rs10922098 T C T C -0.0677 -0.009 0.40011 NA FALSE FALSE FALSE
GCST006061 0.0075 0.2275 537409 outcome TRUE reported 11173_29_MILR1_MILR1
MILR1 chr1 196695521 0.00809 5.827e-17 35329 TRUE reported textfile
2 1 TRUE
7875 rs1555405 A G A G -0.0497 -0.0047 0.30381 NA FALSE FALSE FALSE
GCST006061 0.0081 0.5592 537409 outcome TRUE reported 11173_29_MILR1_MILR1
MILR1 chr14 100710432 0.008644 8.944e-09 35371 TRUE reported
textfile 2 1 TRUE
7876 rs1926446 A C A C -0.069 -0.0038 0.24321 NA FALSE FALSE FALSE
GCST006061 0.0079 0.6325 537409 outcome TRUE reported 11173_29_MILR1_MILR1
MILR1 chr13 46055860 0.009285 1.075e-13 35352 TRUE reported
textfile 2 1 TRUE
7877 rs2057681 G A G A 0.077 -0.0136 0.28541 NA FALSE FALSE FALSE
GCST006061 0.0078 0.07965 537409 outcome TRUE reported 11173_29_MILR1_MILR1
MILR1 chr7 95308945 0.008796 2.059e-18 35340 TRUE reported
textfile 2 1 TRUE
7878 rs10922098 T C T C -0.1312 -0.009 0.40011 NA FALSE FALSE FALSE
GCST006061 0.0075 0.2275 537409 outcome TRUE reported 7247_1_GORAB_GORAB
GORAB chr1 196695521 0.008145 2.221e-58 35338 TRUE reported
textfile 2 1 TRUE
7879 rs1707652 T C T C 0.0782 0.0081 0.3102 NA FALSE FALSE FALSE
GCST006061 0.0074 0.2701 537409 outcome TRUE reported 7247_1_GORAB_GORAB
GORAB chr3 165761011 0.00861 1.061e-19 35339 TRUE reported textfile
2 1 TRUE
7880 rs2232613 T C T C -0.0916 0.0151 0.0989 NA FALSE FALSE FALSE
GCST006061 0.0141 0.2834 537409 outcome TRUE reported 7247_1_GORAB_GORAB
GORAB chr20 38369011 0.013178 3.634e-12 35375 TRUE reported
textfile 2 1 TRUE
7881 rs10922098 T C T C -0.0549 -0.009 0.40011 NA FALSE FALSE FALSE
GCST006061 0.0075 0.2275 537409 outcome TRUE reported 9853_3_IFIT2_IFIT2
IFIT2 chr1 196695521 0.008133 1.477e-11 35338 TRUE reported
textfile 2 1 TRUE
7882 rs144202860 A G A G -0.1996 -0.0629 0.01274 NA FALSE FALSE FALSE
GCST006061 0.0538 0.2423 537409 outcome TRUE reported 9853_3_IFIT2_IFIT2
IFIT2 chr2 216625740 0.035397 1.711e-08 35359 TRUE reported
textfile 2 1 TRUE
7883 rs71352239 T C T C 0.1264 -0.0041 0.30263 NA FALSE FALSE FALSE

GCST006061 0.0087 0.6372 537409 outcome TRUE reported 9853_3_IFIT2_IFIT2
IFIT2 chr19 44926286 0.008676 4.417e-48 35368 TRUE reported
textfile 2 1 TRUE
7884 rs10922098 T C T C -0.0883 -0.009 0.40011 NA FALSE FALSE FALSE
GCST006061 0.0075 0.2275 537409 outcome TRUE reported 7257_18_PTH2_TIP39
PTH2 chr1 196695521 0.007995 2.323e-28 35338 TRUE reported
textfile 2 1 TRUE
7885 rs6445035 A G A G 0.0618 0.0154 0.1904 NA FALSE FALSE FALSE
GCST006061 0.0089 0.08256 537409 outcome TRUE reported 7257_18_PTH2_TIP39
PTH2 chr3 165762312 0.009956 5.388e-10 35339 TRUE reported
textfile 2 1 TRUE
7886 rs10922098 T C T C -0.0782 -0.009 0.40011 NA FALSE FALSE FALSE
GCST006061 0.0075 0.2275 537409 outcome TRUE reported 2247_20_PROK1_EG_VEGF
PROK1 chr1 196695521 0.008 1.445e-22 35338 TRUE reported textfile
2 1 TRUE
7887 rs10922098 T C T C -0.0683 -0.009 0.40011 NA FALSE FALSE FALSE
GCST006061 0.0075 0.2275 537409 outcome TRUE reported 6553_68_CFAP45_CCD19
CFAP45 chr1 196695521 0.008016 1.591e-17 35338 TRUE reported
textfile 2 1 TRUE
7888 rs2232613 T C T C -0.1054 0.0151 0.0989 NA FALSE FALSE FALSE
GCST006061 0.0141 0.2834 537409 outcome TRUE reported 6553_68_CFAP45_CCD19
CFAP45 chr20 38369011 0.012954 4.075e-16 35375 TRUE reported
textfile 2 1 TRUE
7889 rs10922098 T C T C -0.2139 -0.009 0.40011 NA FALSE FALSE FALSE
GCST006061 0.0075 0.2275 537409 outcome TRUE reported 15670_15_C1QL2_C1QL2
C1QL2 chr1 196695521 0.008148 6.533e-152 35338 TRUE reported
textfile 2 1 TRUE
7890 rs12740041 C T C T 0.0582 -0.0091 0.26633 NA FALSE FALSE FALSE
GCST006061 0.0089 0.3093 537409 outcome TRUE reported 15670_15_C1QL2_C1QL2
C1QL2 chr1 197845477 0.009065 1.358e-10 35337 TRUE reported
textfile 2 1 TRUE
7891 rs704 A G A G 0.093 -0.0155 0.49189 NA FALSE FALSE FALSE GCST006061
0.0072 0.03144 537409 outcome TRUE reported 15670_15_C1QL2_C1QL2 C1QL2
chr17 28367840 0.008025 4.697e-31 35360 TRUE reported textfile 2
1 TRUE
7892 rs10922098 T C T C -0.1612 -0.009 0.40011 NA FALSE FALSE FALSE
GCST006061 0.0075 0.2275 537409 outcome TRUE reported 13502_2_SLC26A11_S2611
SLC26A11 chr1 196695521 0.008135 2.161e-87 35332 TRUE reported
textfile 2 1 TRUE
7893 rs1555405 A G A G -0.0477 -0.0047 0.30381 NA FALSE FALSE FALSE
GCST006061 0.0081 0.5592 537409 outcome TRUE reported 13502_2_SLC26A11_S2611
SLC26A11 chr14 100710432 0.008738 4.79e-08 35374 TRUE reported
textfile 2 1 TRUE
7894 rs1707652 T C T C 0.0986 0.0081 0.3102 NA FALSE FALSE FALSE
GCST006061 0.0074 0.2701 537409 outcome TRUE reported 13502_2_SLC26A11_S2611
SLC26A11 chr3 165761011 0.008611 2.336e-30 35333 TRUE reported
textfile 2 1 TRUE
7895 rs704 A G A G -0.0779 -0.0155 0.49189 NA FALSE FALSE FALSE GCST006061
0.0072 0.03144 537409 outcome TRUE reported 13502_2_SLC26A11_S2611 SLC26A11
chr17 28367840 0.008004 2.182e-22 35354 TRUE reported textfile 2
1 TRUE
7896 rs10922098 T C T C -0.0753 -0.009 0.40011 NA FALSE FALSE FALSE
GCST006061 0.0075 0.2275 537409 outcome TRUE reported 5255_22_PDE4D_PDE4D
PDE4D chr1 196695521 0.008011 5.45e-21 35338 TRUE reported
textfile 2 1 TRUE
7897 rs1619994 G A G A 0.0543 0.0168 0.2807 NA FALSE FALSE FALSE
GCST006061 0.0078 0.03057 537409 outcome TRUE reported 5255_22_PDE4D_PDE4D
PDE4D chr3 165744047 0.00873 4.964e-10 35339 TRUE reported textfile
2 1 TRUE
7898 rs10922098 T C T C -0.121 -0.009 0.40011 NA FALSE FALSE FALSE
GCST006061 0.0075 0.2275 537409 outcome TRUE reported 8889_5_CCPG1_CCPG1
CCPG1 chr1 196695521 0.008052 4.889e-51 35338 TRUE reported
textfile 2 1 TRUE
7899 rs1354034 C T C T 0.0477 0.0088 0.38119 NA FALSE FALSE FALSE
GCST006061 0.0073 0.2248 537409 outcome TRUE reported 8889_5_CCPG1_CCPG1
CCPG1 chr3 56815721 0.008123 4.306e-09 35356 TRUE reported

textfile 2 1 TRUE
7900 rs1355538 G A G A -0.0598 -0.0076 0.39847 NA FALSE FALSE FALSE
GCST006061 0.0072 0.2923 537409 outcome TRUE reported 8889_5_CCPG1_CCPG1
CCPG1 chr3 165787389 0.008051 1.104e-13 35338 TRUE reported
textfile 2 1 TRUE
7901 rs10922098 T C T C -0.1081 -0.009 0.40011 NA FALSE FALSE FALSE
GCST006061 0.0075 0.2275 537409 outcome TRUE reported 16049_43_OLR1_OLR1
OLR1 chr1 196695521 0.008077 7.557e-41 35338 TRUE reported
textfile 2 1 TRUE
7902 rs1355538 G A G A -0.054 -0.0076 0.39847 NA FALSE FALSE FALSE
GCST006061 0.0072 0.2923 537409 outcome TRUE reported 16049_43_OLR1_OLR1
OLR1 chr3 165787389 0.008083 2.383e-11 35338 TRUE reported
textfile 2 1 TRUE
7903 rs10922098 T C T C -0.0938 -0.009 0.40011 NA FALSE FALSE FALSE
GCST006061 0.0075 0.2275 537409 outcome TRUE reported 9365_91_EYS_EYS EYS
chr1 196695521 0.007999 9.342e-32 35338 TRUE reported textfile 2
1 TRUE
7904 rs1355538 G A G A -0.0459 -0.0076 0.39847 NA FALSE FALSE FALSE
GCST006061 0.0072 0.2923 537409 outcome TRUE reported 9365_91_EYS_EYS EYS
chr3 165787389 0.007995 9.408e-09 35338 TRUE reported textfile 2
1 TRUE
7905 rs10922098 T C T C -0.2041 -0.009 0.40011 NA FALSE FALSE FALSE
GCST006061 0.0075 0.2275 537409 outcome TRUE reported 19310_81_COPE_COPE
COPE chr1 196695521 0.008073 5.084e-141 35338 TRUE reported
textfile 2 1 TRUE
7906 rs12629202 A C A C 0.0469 0.0052 0.31116 NA FALSE FALSE FALSE
GCST006061 0.0081 0.5213 537409 outcome TRUE reported 19310_81_COPE_COPE
COPE chr3 167083477 0.008584 4.658e-08 35354 TRUE reported
textfile 2 1 TRUE
7907 rs1707652 T C T C 0.1057 0.0081 0.3102 NA FALSE FALSE FALSE
GCST006061 0.0074 0.2701 537409 outcome TRUE reported 19310_81_COPE_COPE
COPE chr3 165761011 0.008568 5.756e-35 35339 TRUE reported
textfile 2 1 TRUE
7908 rs2808466 C T C T 0.1321 -0.0081 0.11154 NA FALSE FALSE FALSE
GCST006061 0.0104 0.4395 537409 outcome TRUE reported 19310_81_COPE_COPE
COPE chr1 207100406 0.012707 2.58e-25 35369 TRUE reported
textfile 2 1 TRUE
7909 rs7598430 T C T C 0.0445 0.0042 0.46402 NA FALSE FALSE FALSE
GCST006061 0.0072 0.5584 537409 outcome TRUE reported 19310_81_COPE_COPE
COPE chr2 218329240 0.00801 2.764e-08 35368 TRUE reported textfile
2 1 TRUE
7910 rs10922098 T C T C -0.096 -0.009 0.40011 NA FALSE FALSE FALSE
GCST006061 0.0075 0.2275 537409 outcome TRUE reported 3797_1_CDH2_Cadherin_2
CDH2 chr1 196695521 0.008082 1.547e-32 35338 TRUE reported
textfile 2 1 TRUE
7911 rs10922098 T C T C -0.0572 -0.009 0.40011 NA FALSE FALSE FALSE
GCST006061 0.0075 0.2275 537409 outcome TRUE reported 6455_52_HAPLN4_HPLN4
HAPLN4 chr1 196695521 0.008039 1.114e-12 35338 TRUE reported
textfile 2 1 TRUE
7912 rs6542680 T C T C -0.0583 -0.0012 0.18362 NA FALSE FALSE FALSE
GCST006061 0.0091 0.8935 537409 outcome TRUE reported 6455_52_HAPLN4_HPLN4
HAPLN4 chr2 3592552 0.010189 1.053e-08 35332 TRUE reported textfile
2 1 TRUE
7913 rs10922098 T C T C -0.2057 -0.009 0.40011 NA FALSE FALSE FALSE
GCST006061 0.0075 0.2275 537409 outcome TRUE reported 16551_14_STAT5B_STA5B
STAT5B chr1 196695521 0.008147 1.163e-140 35338 TRUE reported
textfile 2 1 TRUE
7914 rs1291921 G A G A 0.0492 -0.0157 0.2763 NA FALSE FALSE FALSE
GCST006061 0.0081 0.05326 537409 outcome TRUE reported 16551_14_STAT5B_STA5B
STAT5B chr3 136317384 0.008995 4.509e-08 35361 TRUE reported
textfile 2 1 TRUE
7915 rs35351085 T C T C 0.1084 0.0061 0.04877 NA FALSE FALSE FALSE
GCST006061 0.022 0.7825 537409 outcome TRUE reported 16551_14_STAT5B_STA5B
STAT5B chr1 197992799 0.018521 4.832e-09 35343 TRUE reported
textfile 2 1 TRUE
7916 rs60946795 T C T C 0.0482 0.0013 0.33204 NA FALSE FALSE FALSE

GCST006061 0.0076 0.8653 537409 outcome TRUE reported 16551_14_STAT5B_STA5B
STAT5B chr3 167050200 0.008539 1.652e-08 35354 TRUE reported
textfile 2 1 TRUE
7917 rs10922098 T C T C -0.1413 -0.009 0.40011 NA FALSE FALSE FALSE
GCST006061 0.0075 0.2275 537409 outcome TRUE reported 18259_15_MEOX2_MEOX2
MEOX2 chr1 196695521 0.008111 5.736e-68 35338 TRUE reported
textfile 2 1 TRUE
7918 rs12146727 A G A G -0.1441 -0.0037 0.11875 NA FALSE FALSE FALSE
GCST006061 0.0105 0.7269 537409 outcome TRUE reported 18259_15_MEOX2_MEOX2
MEOX2 chr12 7063032 0.012521 1.193e-30 35390 TRUE reported textfile
2 1 TRUE
7919 rs1355538 G A G A -0.0666 -0.0076 0.39847 NA FALSE FALSE FALSE
GCST006061 0.0072 0.2923 537409 outcome TRUE reported 18259_15_MEOX2_MEOX2
MEOX2 chr3 165787389 0.008117 2.307e-16 35338 TRUE reported
textfile 2 1 TRUE
7920 rs28362944 C T C T 0.1027 0.0094 0.05754 NA FALSE FALSE FALSE
GCST006061 0.0194 0.629 537409 outcome TRUE reported 18259_15_MEOX2_MEOX2
MEOX2 chr11 57598250 0.0173 2.913e-09 35364 TRUE reported textfile
2 1 TRUE
7921 rs10922098 T C T C -0.1624 -0.009 0.40011 NA FALSE FALSE FALSE
GCST006061 0.0075 0.2275 537409 outcome TRUE reported 18297_8_MRAS_RASM
MRAS chr1 196695521 0.008094 1.532e-89 35338 TRUE reported
textfile 2 1 TRUE
7922 rs1707652 T C T C 0.0844 0.0081 0.3102 NA FALSE FALSE FALSE
GCST006061 0.0074 0.2701 537409 outcome TRUE reported 18297_8_MRAS_RASM
MRAS chr3 165761011 0.008574 7.279e-23 35339 TRUE reported
textfile 2 1 TRUE
7923 rs10922098 T C T C -0.0514 -0.009 0.40011 NA FALSE FALSE FALSE
GCST006061 0.0075 0.2275 537409 outcome TRUE reported 8222_49_CDHR3_CDHR3
CDHR3 chr1 196695521 0.007996 1.288e-10 35338 TRUE reported
textfile 2 1 TRUE
7924 rs112130428 T C T C 0.1811 0.065 0.02272 NA FALSE FALSE FALSE
GCST006061 0.0381 0.08786 537409 outcome TRUE reported 8222_49_CDHR3_CDHR3
CDHR3 chr10 7988387 0.025735 1.964e-12 35361 TRUE reported textfile
2 1 TRUE
7925 rs2284351 C T C T -0.0697 -0.0171 0.39076 NA FALSE FALSE FALSE
GCST006061 0.0073 0.01963 537409 outcome TRUE reported 8222_49_CDHR3_CDHR3
CDHR3 chr3 52788773 0.00801 3.284e-18 35354 TRUE reported textfile
2 1 TRUE
7926 rs10922098 T C T C -0.0671 -0.009 0.40011 NA FALSE FALSE FALSE
GCST006061 0.0075 0.2275 537409 outcome TRUE reported 14283_12_RAB14_RAB14
RAB14 chr1 196695521 0.008093 1.125e-16 35338 TRUE reported
textfile 2 1 TRUE
7927 rs438811 T C T C -0.1537 -5.00E-04 0.23201 NA FALSE FALSE FALSE
GCST006061 0.0088 0.9556 537409 outcome TRUE reported 14283_12_RAB14_RAB14
RAB14 chr19 44913484 0.009446 1.565e-59 35366 TRUE reported
textfile 2 1 TRUE
7928 rs10922098 T C T C -0.069 -0.009 0.40011 NA FALSE FALSE FALSE
GCST006061 0.0075 0.2275 537409 outcome TRUE reported 5663_18_PF4V1_PF4V
PF4V1 chr1 196695521 0.008203 4.038e-17 35338 TRUE reported
textfile 2 1 TRUE
7929 rs74706296 T C T C 0.0793 -0.0187 0.1082 NA FALSE FALSE FALSE
GCST006061 0.0129 0.1475 537409 outcome TRUE reported 5663_18_PF4V1_PF4V
PF4V1 chr4 71799255 0.013087 1.365e-09 35352 TRUE reported
textfile 2 1 TRUE
7930 rs75038538 A G A G 0.1723 0.0101 0.03775 NA FALSE FALSE FALSE
GCST006061 0.0197 0.6096 537409 outcome TRUE reported 5663_18_PF4V1_PF4V
PF4V1 chr4 73187658 0.020914 1.745e-16 35355 TRUE reported
textfile 2 1 TRUE
7931 rs7658970 C T C T -0.3218 0.0019 0.28703 NA FALSE FALSE FALSE
GCST006061 0.0079 0.807 537409 outcome TRUE reported 5663_18_PF4V1_PF4V
PF4V1 chr4 73848233 0.008748 1e-200 35349 TRUE reported textfile
2 1 TRUE
7932 rs10922098 T C T C -0.058 -0.009 0.40011 NA FALSE FALSE FALSE
GCST006061 0.0075 0.2275 537409 outcome TRUE reported 10716_35_PSMD5_PSMD5
PSMD5 chr1 196695521 0.007986 3.787e-13 35326 TRUE reported

textfile 2 1 TRUE
7933 rs2284350 C T C T -0.0521 -0.0167 0.39076 NA FALSE FALSE FALSE
GCST006061 0.0073 0.0219 537409 outcome TRUE reported 10716_35_PSMD5_PSMD5
PSMD5 chr3 52788840 0.007994 7.166e-11 35342 TRUE reported
textfile 2 1 TRUE
7934 rs3917550 A G A G 0.0677 -0.0068 0.15554 NA FALSE FALSE FALSE
GCST006061 0.0107 0.5243 537409 outcome TRUE reported 10716_35_PSMD5_PSMD5
PSMD5 chr7 95305261 0.010806 3.727e-10 35337 TRUE reported
textfile 2 1 TRUE
7935 rs10922098 T C T C -0.1831 -0.009 0.40011 NA FALSE FALSE FALSE
GCST006061 0.0075 0.2275 537409 outcome TRUE reported
18290_6_ANXA8_Annexin_A8 ANXA8 chr1 196695521 0.008093 2.452e-113 35338
TRUE reported textfile 2 1 TRUE
7936 rs1707652 T C T C 0.0859 0.0081 0.3102 NA FALSE FALSE FALSE
GCST006061 0.0074 0.2701 537409 outcome TRUE reported
18290_6_ANXA8_Annexin_A8 ANXA8 chr3 165761011 0.008582 1.387e-23 35339
TRUE reported textfile 2 1 TRUE
7937 rs28645249 C T C T 0.0631 0.019 0.26162 NA FALSE FALSE FALSE
GCST006061 0.0115 0.09815 537409 outcome TRUE reported
18290_6_ANXA8_Annexin_A8 ANXA8 chr6 32557458 0.008725 4.756e-13 35438
TRUE reported textfile 2 1 TRUE
7938 rs10922098 T C T C -0.0624 -0.009 0.40011 NA FALSE FALSE FALSE
GCST006061 0.0075 0.2275 537409 outcome TRUE reported 11155_16_COL6A5_CO6A5
COL6A5 chr1 196695521 0.007997 6.056e-15 35329 TRUE reported
textfile 2 1 TRUE
7939 rs10922098 T C T C -0.0765 -0.009 0.40011 NA FALSE FALSE FALSE
GCST006061 0.0075 0.2275 537409 outcome TRUE reported 6237_11_FGFRL1_FGRL1
FGFRL1 chr1 196695521 0.008055 2.154e-21 35338 TRUE reported
textfile 2 1 TRUE
7940 rs704 A G A G 0.057 -0.0155 0.49189 NA FALSE FALSE FALSE GCST006061
0.0072 0.03144 537409 outcome TRUE reported 6237_11_FGFRL1_FGRL1 FGFRL1
chr17 28367840 0.007902 5.441e-13 35360 TRUE reported textfile 2
1 TRUE
7941 rs10922098 T C T C -0.1412 -0.009 0.40011 NA FALSE FALSE FALSE
GCST006061 0.0075 0.2275 537409 outcome TRUE reported 10539_30_CD99L2_C99L2
CD99L2 chr1 196695521 0.008081 2.296e-68 35326 TRUE reported
textfile 2 1 TRUE
7942 rs1355538 G A G A -0.0605 -0.0076 0.39847 NA FALSE FALSE FALSE
GCST006061 0.0072 0.2923 537409 outcome TRUE reported 10539_30_CD99L2_C99L2
CD99L2 chr3 165787389 0.008091 7.572e-14 35326 TRUE reported
textfile 2 1 TRUE
7943 rs704 A G A G 0.0642 -0.0155 0.49189 NA FALSE FALSE FALSE GCST006061
0.0072 0.03144 537409 outcome TRUE reported 10539_30_CD99L2_C99L2 CD99L2
chr17 28367840 0.007937 6.019e-16 35348 TRUE reported textfile 2
1 TRUE
7944 rs10922098 T C T C -0.1054 -0.009 0.40011 NA FALSE FALSE FALSE
GCST006061 0.0075 0.2275 537409 outcome TRUE reported 10892_8_OSMR_OSMR
OSMR chr1 196695521 0.008138 2.32e-38 35327 TRUE reported
textfile 2 1 TRUE
7945 rs2106009 A C A C -0.0648 0.0213 0.42058 NA FALSE FALSE FALSE
GCST006061 0.0122 0.08103 537409 outcome TRUE reported 10892_8_OSMR_OSMR
OSMR chr14 106135717 0.008205 2.841e-15 34955 TRUE reported
textfile 2 1 TRUE
7946 rs10922098 T C T C -0.0453 -0.009 0.40011 NA FALSE FALSE FALSE
GCST006061 0.0075 0.2275 537409 outcome TRUE reported 10506_53_PGRMC2_PGRC2
PGRMC2 chr1 196695521 0.008026 1.658e-08 35326 TRUE reported
textfile 2 1 TRUE
7947 rs1355538 G A G A -0.052 -0.0076 0.39847 NA FALSE FALSE FALSE
GCST006061 0.0072 0.2923 537409 outcome TRUE reported 10506_53_PGRMC2_PGRC2
PGRMC2 chr3 165787389 0.008013 8.603e-11 35326 TRUE reported
textfile 2 1 TRUE
7948 rs7311982 C T C T 0.0922 -0.0049 0.30107 NA FALSE FALSE FALSE
GCST006061 0.0078 0.5309 537409 outcome TRUE reported 10506_53_PGRMC2_PGRC2
PGRMC2 chr12 9162261 0.008582 6.357e-27 35375 TRUE reported textfile
2 1 TRUE
7949 rs10922098 T C T C -0.0979 -0.009 0.40011 NA FALSE FALSE FALSE

GCST006061 0.0075 0.2275 537409 outcome TRUE reported 12970_35_STAU2_STAU2
STAU2 chr1 196695521 0.007977 1.265e-34 35329 TRUE reported
textfile 2 1 TRUE

7950 rs11601665 T C T C 0.0511 -0.0045 0.26367 NA FALSE FALSE FALSE
GCST006061 0.0088 0.6088 537409 outcome TRUE reported 12970_35_STAU2_STAU2
STAU2 chr11 262930 0.008849 7.71e-09 35343 TRUE reported textfile
2 1 TRUE

7951 rs1354034 C T C T 0.0817 0.0088 0.38119 NA FALSE FALSE FALSE
GCST006061 0.0073 0.2248 537409 outcome TRUE reported 12970_35_STAU2_STAU2
STAU2 chr3 56815721 0.008044 3.1e-24 35347 TRUE reported textfile
2 1 TRUE

7952 rs1355538 G A G A -0.0464 -0.0076 0.39847 NA FALSE FALSE FALSE
GCST006061 0.0072 0.2923 537409 outcome TRUE reported 12970_35_STAU2_STAU2
STAU2 chr3 165787389 0.00797 5.823e-09 35329 TRUE reported textfile
2 1 TRUE

7953 rs7080536 A G A G -0.1122 -0.0145 0.04142 NA FALSE FALSE FALSE
GCST006061 0.0203 0.4753 537409 outcome TRUE reported 12970_35_STAU2_STAU2
STAU2 chr10 113588287 0.019535 9.271e-09 35378 TRUE reported
textfile 2 1 TRUE

7954 rs10922098 T C T C -0.111 -0.009 0.40011 NA FALSE FALSE FALSE
GCST006061 0.0075 0.2275 537409 outcome TRUE reported 13574_50_RND1_RND1
RND1 chr1 196695521 0.007976 5.063e-44 35332 TRUE reported
textfile 2 1 TRUE

7955 rs111696008 A G A G -0.1569 -0.0259 0.02443 NA FALSE FALSE FALSE
GCST006061 0.0401 0.519 537409 outcome TRUE reported 13574_50_RND1_RND1
RND1 chr2 216622986 0.025981 1.551e-09 35353 TRUE reported
textfile 2 1 TRUE

7956 rs10922098 T C T C -0.1322 -0.009 0.40011 NA FALSE FALSE FALSE
GCST006061 0.0075 0.2275 537409 outcome TRUE reported 12945_33_RAB18_RAB18
RAB18 chr1 196695521 0.008093 5.583e-60 35329 TRUE reported
textfile 2 1 TRUE

7957 rs10922098 T C T C -0.0675 -0.009 0.40011 NA FALSE FALSE FALSE
GCST006061 0.0075 0.2275 537409 outcome TRUE reported 6626_81_CHST12_CHSTC
CHST12 chr1 196695521 0.008292 3.931e-16 35338 TRUE reported
textfile 2 1 TRUE

7958 rs111696008 A G A G 0.167 -0.0259 0.02443 NA FALSE FALSE FALSE
GCST006061 0.0401 0.519 537409 outcome TRUE reported 6626_81_CHST12_CHSTC
CHST12 chr2 216622986 0.026954 5.804e-10 35359 TRUE reported
textfile 2 1 TRUE

7959 rs17713196 T C T C -0.1805 0.015 0.1903 NA FALSE FALSE FALSE
GCST006061 0.0089 0.09205 537409 outcome TRUE reported 6626_81_CHST12_CHSTC
CHST12 chr3 165771936 0.010294 7.815e-69 35339 TRUE reported
textfile 2 1 TRUE

7960 rs3184504 C T C T -0.0572 0.0074 0.37862 NA FALSE FALSE FALSE
GCST006061 0.0075 0.3235 537409 outcome TRUE reported 6626_81_CHST12_CHSTC
CHST12 chr12 111446804 0.008378 8.668e-12 35371 TRUE reported
textfile 2 1 TRUE

7961 rs4720987 C T C T -0.188 -0.0051 0.02068 NA FALSE FALSE FALSE
GCST006061 0.0354 0.8848 537409 outcome TRUE reported 6626_81_CHST12_CHSTC
CHST12 chr7 1741786 0.029131 1.092e-10 35325 TRUE reported textfile
2 1 TRUE

7962 rs635634 C T C T -0.1921 0.0285 0.12995 NA FALSE FALSE FALSE
GCST006061 0.0089 0.001351 537409 outcome TRUE reported
6626_81_CHST12_CHSTC CHST12 chr9 133279427 0.012054 3.498e-57 35371
TRUE reported textfile 2 1 TRUE

7963 rs6952433 G A G A -0.2047 -1.00E-04 0.21383 NA FALSE FALSE FALSE
GCST006061 0.0088 0.992 537409 outcome TRUE reported 6626_81_CHST12_CHSTC
CHST12 chr7 2357003 0.009862 1.051e-95 35334 TRUE reported textfile
2 1 TRUE

7964 rs704 A G A G 0.0485 -0.0155 0.49189 NA FALSE FALSE FALSE GCST006061
0.0072 0.03144 537409 outcome TRUE reported 6626_81_CHST12_CHSTC CHST12
chr17 28367840 0.008114 2.267e-09 35360 TRUE reported textfile 2
1 TRUE

7965 rs10922098 T C T C -0.13 -0.009 0.40011 NA FALSE FALSE FALSE
GCST006061 0.0075 0.2275 537409 outcome TRUE reported 3445_53_IL15RA_IL_15_Ra
IL15RA chr1 196695521 0.008088 3.975e-58 35338 TRUE reported

textfile 2 1 TRUE
7966 rs1355538 G A G A -0.0551 -0.0076 0.39847 NA FALSE FALSE FALSE
GCST006061 0.0072 0.2923 537409 outcome TRUE reported 3445_53_IL15RA_IL_15_Ra
IL15RA chr3 165787389 0.008101 1.037e-11 35338 TRUE reported
textfile 2 1 TRUE
7967 rs10922098 T C T C -0.1059 -0.009 0.40011 NA FALSE FALSE FALSE
GCST006061 0.0075 0.2275 537409 outcome TRUE reported
3189_61_TMPRSS15_Enterokinase TMPRSS15 chr1 196695521 0.00806 1.987e-39
35338 TRUE reported textfile 2 1 TRUE
7968 rs1355538 G A G A -0.0465 -0.0076 0.39847 NA FALSE FALSE FALSE
GCST006061 0.0072 0.2923 537409 outcome TRUE reported
3189_61_TMPRSS15_Enterokinase TMPRSS15 chr3 165787389 0.008059 7.942e-09
35338 TRUE reported textfile 2 1 TRUE
7969 rs6542680 T C T C -0.0836 -0.0012 0.18362 NA FALSE FALSE FALSE
GCST006061 0.0091 0.8935 537409 outcome TRUE reported
3189_61_TMPRSS15_Enterokinase TMPRSS15 chr2 3592552 0.010226 2.955e-16
35332 TRUE reported textfile 2 1 TRUE
7970 rs10922098 T C T C -0.1039 -0.009 0.40011 NA FALSE FALSE FALSE
GCST006061 0.0075 0.2275 537409 outcome TRUE reported 5867_60_ARG1_ARGI1
ARG1 chr1 196695521 0.008044 3.669e-38 35338 TRUE reported
textfile 2 1 TRUE
7971 rs2668202 G A G A -0.0535 -0.0076 0.30991 NA FALSE FALSE FALSE
GCST006061 0.0074 0.3015 537409 outcome TRUE reported 5867_60_ARG1_ARGI1
ARG1 chr3 165772680 0.008511 3.258e-10 35339 TRUE reported
textfile 2 1 TRUE
7972 rs2781668 T C T C -0.1115 -0.0107 0.1932 NA FALSE FALSE FALSE
GCST006061 0.0094 0.2576 537409 outcome TRUE reported 5867_60_ARG1_ARGI1
ARG1 chr6 131576138 0.009949 3.744e-29 35366 TRUE reported
textfile 2 1 TRUE
7973 rs7756633 T C T C 0.1732 0.0299 0.02146 NA FALSE FALSE FALSE
GCST006061 0.0247 0.2268 537409 outcome TRUE reported 5867_60_ARG1_ARGI1
ARG1 chr6 131726686 0.027354 2.423e-10 35363 TRUE reported
textfile 2 1 TRUE
7974 rs8014529 A G A G -0.1371 -0.0034 0.41692 NA FALSE FALSE FALSE
GCST006061 0.0073 0.6455 537409 outcome TRUE reported 5867_60_ARG1_ARGI1
ARG1 chr14 106784065 0.008144 1.35e-63 34965 TRUE reported
textfile 2 1 TRUE
7975 rs10922098 T C T C -0.1039 -0.009 0.40011 NA FALSE FALSE FALSE
GCST006061 0.0075 0.2275 537409 outcome TRUE reported 2447_7_PLA2G2E_GIIE
PLA2G2E chr1 196695521 0.008014 1.945e-38 35338 TRUE reported
textfile 2 1 TRUE
7976 rs1355538 G A G A -0.0462 -0.0076 0.39847 NA FALSE FALSE FALSE
GCST006061 0.0072 0.2923 537409 outcome TRUE reported 2447_7_PLA2G2E_GIIE
PLA2G2E chr3 165787389 0.008016 8.257e-09 35338 TRUE reported
textfile 2 1 TRUE
7977 rs10922098 T C T C -0.1465 -0.009 0.40011 NA FALSE FALSE FALSE
GCST006061 0.0075 0.2275 537409 outcome TRUE reported 9444_70_DGCR6_DGCR6
DGCR6 chr1 196695521 0.008072 1.283e-73 35338 TRUE reported
textfile 2 1 TRUE
7978 rs2686395 C T C T -0.0733 -0.0077 0.3101 NA FALSE FALSE FALSE
GCST006061 0.0074 0.2965 537409 outcome TRUE reported 9444_70_DGCR6_DGCR6
DGCR6 chr3 165767231 0.00855 1.005e-17 35339 TRUE reported textfile
2 1 TRUE
7979 rs10922098 T C T C -0.1174 -0.009 0.40011 NA FALSE FALSE FALSE
GCST006061 0.0075 0.2275 537409 outcome TRUE reported 19215_7_NDRG1_NDRG1
NDRG1 chr1 196695521 0.0079 6.007e-50 35338 TRUE reported textfile
2 1 TRUE
7980 rs1355538 G A G A -0.0719 -0.0076 0.39847 NA FALSE FALSE FALSE
GCST006061 0.0072 0.2923 537409 outcome TRUE reported 19215_7_NDRG1_NDRG1
NDRG1 chr3 165787389 0.007909 9.813e-20 35338 TRUE reported
textfile 2 1 TRUE
7981 rs10922098 T C T C -0.0666 -0.009 0.40011 NA FALSE FALSE FALSE
GCST006061 0.0075 0.2275 537409 outcome TRUE reported 12936_38_RLBP1_RLBP1
RLBP1 chr1 196695521 0.008043 1.225e-16 35329 TRUE reported
textfile 2 1 TRUE
7982 rs704 A G A G 0.158 -0.0155 0.49189 NA FALSE FALSE FALSE GCST006061

0.0072 0.03144 537409 outcome TRUE reported 12936_38_RLBP1_RLBP1 RLBP1
chr17 28367840 0.007859 6.928e-90 35351 TRUE reported textfile 2
1 TRUE
7983 rs872914 A G A G -0.0479 0.0019 0.28935 NA FALSE FALSE FALSE
GCST006061 0.0079 0.8076 537409 outcome TRUE reported 12936_38_RLBP1_RLBP1
RLBP1 chr4 73852384 0.008661 3.197e-08 35339 TRUE reported
textfile 2 1 TRUE
7984 rs10922098 T C T C -0.0527 -0.009 0.40011 NA FALSE FALSE FALSE
GCST006061 0.0075 0.2275 537409 outcome TRUE reported 17828_3_S100A14_S100A14
S100A14 chr1 196695521 0.008158 1.046e-10 35338 TRUE reported
textfile 2 1 TRUE
7985 rs1355538 G A G A -0.05 -0.0076 0.39847 NA FALSE FALSE FALSE
GCST006061 0.0072 0.2923 537409 outcome TRUE reported 17828_3_S100A14_S100A14
S100A14 chr3 165787389 0.00815 8.516e-10 35338 TRUE reported textfile
2 1 TRUE
7986 rs2020934 G A G A 0.0551 0.0029 0.44309 NA FALSE FALSE FALSE
GCST006061 0.0076 0.7055 537409 outcome TRUE reported 17828_3_S100A14_S100A14
S100A14 chr17 30234442 0.008045 7.427e-12 35360 TRUE reported
textfile 2 1 TRUE
7987 rs9330298 A C A C -0.0887 -0.023 0.43025 NA FALSE FALSE FALSE
GCST006061 0.0073 0.001624 537409 outcome TRUE reported
17828_3_S100A14_S100A14 S100A14 chr1 153617778 0.008097 6.283e-28 35362
TRUE reported textfile 2 1 TRUE
7988 rs10922098 T C T C -0.1466 -0.009 0.40011 NA FALSE FALSE FALSE
GCST006061 0.0075 0.2275 537409 outcome TRUE reported 7064_2_VAMP8_VAMP8
VAMP8 chr1 196695521 0.00804 2.733e-74 35338 TRUE reported textfile
2 1 TRUE
7989 rs1706435 G A G A 0.0769 0.0117 0.36981 NA FALSE FALSE FALSE
GCST006061 0.0072 0.1047 537409 outcome TRUE reported 7064_2_VAMP8_VAMP8
VAMP8 chr3 165743688 0.008169 4.788e-21 35339 TRUE reported
textfile 2 1 TRUE
7990 rs10922098 T C T C -0.1255 -0.009 0.40011 NA FALSE FALSE FALSE
GCST006061 0.0075 0.2275 537409 outcome TRUE reported 7117_21_SERPINI2_SPI2
SERPINI2 chr1 196695521 0.008081 2.156e-54 35338 TRUE reported
textfile 2 1 TRUE
7991 rs1706435 G A G A 0.0681 0.0117 0.36981 NA FALSE FALSE FALSE
GCST006061 0.0072 0.1047 537409 outcome TRUE reported 7117_21_SERPINI2_SPI2
SERPINI2 chr3 165743688 0.008193 9.389e-17 35339 TRUE reported
textfile 2 1 TRUE
7992 rs10922098 T C T C -0.0845 -0.009 0.40011 NA FALSE FALSE FALSE
GCST006061 0.0075 0.2275 537409 outcome TRUE reported 7903_18_VAMP3_VAMP3
VAMP3 chr1 196695521 0.008058 9.994e-26 35338 TRUE reported
textfile 2 1 TRUE
7993 rs10922098 T C T C -0.079 -0.009 0.40011 NA FALSE FALSE FALSE
GCST006061 0.0075 0.2275 537409 outcome TRUE reported 8321_27_ZNF843_ZN843
ZNF843 chr1 196695521 0.008008 5.878e-23 35338 TRUE reported
textfile 2 1 TRUE
7994 rs1355538 G A G A -0.0456 -0.0076 0.39847 NA FALSE FALSE FALSE
GCST006061 0.0072 0.2923 537409 outcome TRUE reported 8321_27_ZNF843_ZN843
ZNF843 chr3 165787389 0.008006 1.23e-08 35338 TRUE reported
textfile 2 1 TRUE
7995 rs211161 G T G T 0.172 -0.0176 0.01526 NA FALSE FALSE FALSE
GCST006061 0.0295 0.5516 537409 outcome TRUE reported 8321_27_ZNF843_ZN843
ZNF843 chr6 96614917 0.030985 2.839e-08 35343 TRUE reported
textfile 2 1 TRUE
7996 rs10922098 T C T C -0.1511 -0.009 0.40011 NA FALSE FALSE FALSE
GCST006061 0.0075 0.2275 537409 outcome TRUE reported 9044_1_CNTNAP5_CNTP5
CNTNAP5 chr1 196695521 0.008021 3.71e-79 35338 TRUE reported
textfile 2 1 TRUE
7997 rs1707652 T C T C 0.0717 0.0081 0.3102 NA FALSE FALSE FALSE
GCST006061 0.0074 0.2701 537409 outcome TRUE reported 9044_1_CNTNAP5_CNTP5
CNTNAP5 chr3 165761011 0.008501 3.325e-17 35339 TRUE reported
textfile 2 1 TRUE
7998 rs4795434 T G T G 0.0458 -0.0164 0.44864 NA FALSE FALSE FALSE
GCST006061 0.0071 0.02188 537409 outcome TRUE reported 9044_1_CNTNAP5_CNTP5
CNTNAP5 chr17 28389898 0.007916 7.222e-09 35361 TRUE reported

textfile 2 1 TRUE
7999 rs10922098 T C T C -0.0638 -0.009 0.40011 NA FALSE FALSE FALSE
GCST006061 0.0075 0.2275 537409 outcome TRUE reported 12633_3_PTPN9_PTN9
PTPN9 chr1 196695521 0.00807 2.654e-15 35329 TRUE reported textfile
2 1 TRUE
8000 rs111647176 A G A G -0.0702 -0.0214 0.27386 NA FALSE FALSE FALSE
GCST006061 0.0086 0.01227 537409 outcome TRUE reported 12633_3_PTPN9_PTN9
PTPN9 chr15 75459420 0.008811 1.626e-15 35352 TRUE reported
textfile 2 1 TRUE
8001 rs1355538 G A G A -0.0597 -0.0076 0.39847 NA FALSE FALSE FALSE
GCST006061 0.0072 0.2923 537409 outcome TRUE reported 12633_3_PTPN9_PTN9
PTPN9 chr3 165787389 0.008057 1.269e-13 35329 TRUE reported
textfile 2 1 TRUE
8002 rs10922098 T C T C -0.1377 -0.009 0.40011 NA FALSE FALSE FALSE
GCST006061 0.0075 0.2275 537409 outcome TRUE reported 6600_70_RELL2_RELL2
RELL2 chr1 196695521 0.008043 1.063e-65 35338 TRUE reported
textfile 2 1 TRUE
8003 rs1707652 T C T C 0.0696 0.0081 0.3102 NA FALSE FALSE FALSE
GCST006061 0.0074 0.2701 537409 outcome TRUE reported 6600_70_RELL2_RELL2
RELL2 chr3 165761011 0.008513 2.933e-16 35339 TRUE reported
textfile 2 1 TRUE
8004 rs2073453 T C T C -0.0995 -0.0115 0.3832 NA FALSE FALSE FALSE
GCST006061 0.0074 0.1174 537409 outcome TRUE reported 6600_70_RELL2_RELL2
RELL2 chr22 22162605 0.008174 4.343e-34 35364 TRUE reported
textfile 2 1 TRUE
8005 rs10922098 T C T C -0.204 -0.009 0.40011 NA FALSE FALSE FALSE
GCST006061 0.0075 0.2275 537409 outcome TRUE reported
11647_6_FZD10_Frizzled_10 FZD10 chr1 196695521 0.008038 4.12e-142 35329
TRUE reported textfile 2 1 TRUE
8006 rs11569415 A G A G -0.0675 0.0011 0.24276 NA FALSE FALSE FALSE
GCST006061 0.0097 0.9129 537409 outcome TRUE reported
11647_6_FZD10_Frizzled_10 FZD10 chr19 6716268 0.009186 2.004e-13 35363
TRUE reported textfile 2 1 TRUE
8007 rs2686395 C T C T -0.0878 -0.0077 0.3101 NA FALSE FALSE FALSE
GCST006061 0.0074 0.2965 537409 outcome TRUE reported
11647_6_FZD10_Frizzled_10 FZD10 chr3 165767231 0.00853 7.602e-25 35330
TRUE reported textfile 2 1 TRUE
8008 rs35351085 T C T C 0.1013 0.0061 0.04877 NA FALSE FALSE FALSE
GCST006061 0.022 0.7825 537409 outcome TRUE reported
11647_6_FZD10_Frizzled_10 FZD10 chr1 197992799 0.018278 2.987e-08 35334
TRUE reported textfile 2 1 TRUE
8009 rs10922098 T C T C -0.1443 -0.009 0.40011 NA FALSE FALSE FALSE
GCST006061 0.0075 0.2275 537409 outcome TRUE reported 12427_8_CDC25B_MPIP2
CDC25B chr1 196695521 0.00814 2.535e-70 35329 TRUE reported textfile
2 1 TRUE
8010 rs1706435 G A G A 0.0946 0.0117 0.36981 NA FALSE FALSE FALSE
GCST006061 0.0072 0.1047 537409 outcome TRUE reported 12427_8_CDC25B_MPIP2
CDC25B chr3 165743688 0.00826 2.271e-30 35330 TRUE reported textfile
2 1 TRUE
8011 rs429358 C T C T 0.0612 0.005 0.17407 NA FALSE FALSE FALSE
GCST006061 0.0103 0.6319 537409 outcome TRUE reported 12427_8_CDC25B_MPIP2
CDC25B chr19 44908684 0.010649 9.075e-09 35359 TRUE reported
textfile 2 1 TRUE
8012 rs704 A G A G 0.0682 -0.0155 0.49189 NA FALSE FALSE FALSE GCST006061
0.0072 0.03144 537409 outcome TRUE reported 12427_8_CDC25B_MPIP2 CDC25B
chr17 28367840 0.007999 1.518e-17 35351 TRUE reported textfile 2
1 TRUE
8013 rs10922098 T C T C -0.1899 -0.009 0.40011 NA FALSE FALSE FALSE
GCST006061 0.0075 0.2275 537409 outcome TRUE reported 4359_87_DYRK3_DYRK3
DYRK3 chr1 196695521 0.008132 1.3e-120 35338 TRUE reported
textfile 2 1 TRUE
8014 rs1451915 A C A C 0.0635 -0.0107 0.13641 NA FALSE FALSE FALSE
GCST006061 0.0124 0.3895 537409 outcome TRUE reported 4359_87_DYRK3_DYRK3
DYRK3 chr1 196058002 0.011594 4.327e-08 35335 TRUE reported
textfile 2 1 TRUE
8015 rs6445035 A G A G 0.0958 0.0154 0.1904 NA FALSE FALSE FALSE

GCST006061 0.0089 0.08256 537409 outcome TRUE reported 4359_87_DYRK3_DYRK3
DYRK3 chr3 165762312 0.010164 4.274e-21 35339 TRUE reported
textfile 2 1 TRUE
8016 rs10922098 T C T C -0.0977 -0.009 0.40011 NA FALSE FALSE FALSE
GCST006061 0.0075 0.2275 537409 outcome TRUE reported 7932_23_SYT8_SYT8
SYT8 chr1 196695521 0.008 2.656e-34 35338 TRUE reported textfile
2 1 TRUE
8017 rs1706435 G A G A 0.0599 0.0117 0.36981 NA FALSE FALSE FALSE
GCST006061 0.0072 0.1047 537409 outcome TRUE reported 7932_23_SYT8_SYT8
SYT8 chr3 165743688 0.008117 1.587e-13 35339 TRUE reported
textfile 2 1 TRUE
8018 rs10922098 T C T C -0.0513 -0.009 0.40011 NA FALSE FALSE FALSE
GCST006061 0.0075 0.2275 537409 outcome TRUE reported 6941_11_SUMF1_SUMF1
SUMF1 chr1 196695521 0.008511 1.666e-09 35338 TRUE reported
textfile 2 1 TRUE
8019 rs10982050 G A G A -0.0843 -7.00E-04 0.27663 NA FALSE FALSE FALSE
GCST006061 0.0081 0.934 537409 outcome TRUE reported 6941_11_SUMF1_SUMF1
SUMF1 chr9 114066493 0.009344 1.848e-19 35362 TRUE reported
textfile 2 1 TRUE
8020 rs149062332 C T C T 0.404 0.0424 0.02045 NA FALSE FALSE FALSE
GCST006061 0.0435 0.33 537409 outcome TRUE reported 6941_11_SUMF1_SUMF1
SUMF1 chr3 186623236 0.028536 1.674e-45 35367 TRUE reported
textfile 2 1 TRUE
8021 rs1707652 T C T C 0.0698 0.0081 0.3102 NA FALSE FALSE FALSE
GCST006061 0.0074 0.2701 537409 outcome TRUE reported 6941_11_SUMF1_SUMF1
SUMF1 chr3 165761011 0.008965 6.937e-15 35339 TRUE reported
textfile 2 1 TRUE
8022 rs2106727 G A G A 0.0634 -0.0022 0.35982 NA FALSE FALSE FALSE
GCST006061 0.0074 0.7654 537409 outcome TRUE reported 6941_11_SUMF1_SUMF1
SUMF1 chr7 17248374 0.00865 2.307e-13 35363 TRUE reported textfile
2 1 TRUE
8023 rs2472297 T C T C 0.0501 0.0015 0.29113 NA FALSE FALSE FALSE
GCST006061 0.0088 0.8653 537409 outcome TRUE reported 6941_11_SUMF1_SUMF1
SUMF1 chr15 74735539 0.009132 4.103e-08 35359 TRUE reported
textfile 2 1 TRUE
8024 rs682479 A C A C 0.1361 0.0133 0.29837 NA FALSE FALSE FALSE
GCST006061 0.0078 0.08774 537409 outcome TRUE reported 6941_11_SUMF1_SUMF1
SUMF1 chr1 56910045 0.009028 2.382e-51 35369 TRUE reported
textfile 2 1 TRUE
8025 rs7862602 T G T G 0.1463 -3.00E-04 0.43654 NA FALSE FALSE FALSE
GCST006061 0.0074 0.9625 537409 outcome TRUE reported 6941_11_SUMF1_SUMF1
SUMF1 chr9 136946019 0.008377 2.647e-68 35260 TRUE reported
textfile 2 1 TRUE
8026 rs10922098 T C T C -0.0752 -0.009 0.40011 NA FALSE FALSE FALSE
GCST006061 0.0075 0.2275 537409 outcome TRUE reported
15589_1_GC_Gc_Globulin__Mixed_Type GC chr1 196695521 0.008284 1.104e-19
35338 TRUE reported textfile 2 1 TRUE
8027 rs2954021 G A G A -0.0488 -0.0016 0.45781 NA FALSE FALSE FALSE
GCST006061 0.0071 0.8249 537409 outcome TRUE reported
15589_1_GC_Gc_Globulin__Mixed_Type GC chr8 125469835 0.008172 2.352e-09
35368 TRUE reported textfile 2 1 TRUE
8028 rs72729623 T C T C -0.0608 0.024 0.16311 NA FALSE FALSE FALSE
GCST006061 0.0107 0.02466 537409 outcome TRUE reported
15589_1_GC_Gc_Globulin__Mixed_Type GC chr4 153287126 0.011047 3.718e-08
35376 TRUE reported textfile 2 1 TRUE
8029 rs7697091 C A C A -0.1219 -0.0053 0.35008 NA FALSE FALSE FALSE
GCST006061 0.0075 0.4777 537409 outcome TRUE reported
15589_1_GC_Gc_Globulin__Mixed_Type GC chr4 71759380 0.008532 2.623e-46
35353 TRUE reported textfile 2 1 TRUE
8030 rs10922098 T C T C -0.082 -0.009 0.40011 NA FALSE FALSE FALSE
GCST006061 0.0075 0.2275 537409 outcome TRUE reported 19108_50_MECP2_MECP2
MECP2 chr1 196695521 0.008033 1.817e-24 35338 TRUE reported
textfile 2 1 TRUE
8031 rs144744495 A G A G 0.1191 0.0149 0.03437 NA FALSE FALSE FALSE
GCST006061 0.0154 0.3351 537409 outcome TRUE reported 19108_50_MECP2_MECP2
MECP2 chr2 23889671 0.02119 1.903e-08 35370 TRUE reported textfile

2 1 TRUE
8032 rs10922098 T C T C -0.0946 -0.009 0.40011 NA FALSE FALSE FALSE
GCST006061 0.0075 0.2275 537409 outcome TRUE reported 7092_7_PDILT_PDILT
PDILT chr1 196695521 0.00811 1.919e-31 35338 TRUE reported textfile
2 1 TRUE
8033 rs6445035 A G A G 0.0639 0.0154 0.1904 NA FALSE FALSE FALSE
GCST006061 0.0089 0.08256 537409 outcome TRUE reported 7092_7_PDILT_PDILT
PDILT chr3 165762312 0.010109 2.599e-10 35339 TRUE reported
textfile 2 1 TRUE
8034 rs10922098 T C T C -0.137 -0.009 0.40011 NA FALSE FALSE FALSE
GCST006061 0.0075 0.2275 537409 outcome TRUE reported 6536_54_PLBD2_LAML2
PLBD2 chr1 196695521 0.008197 1.044e-62 35338 TRUE reported
textfile 2 1 TRUE
8035 rs1706435 G A G A 0.07 0.0117 0.36981 NA FALSE FALSE FALSE
GCST006061 0.0072 0.1047 537409 outcome TRUE reported 6536_54_PLBD2_LAML2
PLBD2 chr3 165743688 0.008314 3.791e-17 35339 TRUE reported
textfile 2 1 TRUE
8036 rs10922098 T C T C -0.1353 -0.009 0.40011 NA FALSE FALSE FALSE
GCST006061 0.0075 0.2275 537409 outcome TRUE reported 9723_105_MMRN2_EMILIN_3
MMRN2 chr1 196695521 0.008168 1.256e-61 35338 TRUE reported
textfile 2 1 TRUE
8037 rs1707652 T C T C 0.1003 0.0081 0.3102 NA FALSE FALSE FALSE
GCST006061 0.0074 0.2701 537409 outcome TRUE reported 9723_105_MMRN2_EMILIN_3
MMRN2 chr3 165761011 0.008632 3.27e-31 35339 TRUE reported
textfile 2 1 TRUE
8038 rs4821102 A C A C -0.0595 0.0144 0.22639 NA FALSE FALSE FALSE
GCST006061 0.008 0.07137 537409 outcome TRUE reported 9723_105_MMRN2_EMILIN_3
MMRN2 chr22 32770241 0.009595 5.611e-10 35375 TRUE reported
textfile 2 1 TRUE
8039 rs10922098 T C T C -0.1527 -0.009 0.40011 NA FALSE FALSE FALSE
GCST006061 0.0075 0.2275 537409 outcome TRUE reported
19237_17_DDT_D_dopachrome_decarboxylase DDT chr1 196695521 0.00821 3.249e-77
35338 TRUE reported textfile 2 1 TRUE
8040 rs117689831 G A G A 0.3156 0.0115 0.02088 NA FALSE FALSE FALSE
GCST006061 0.0287 0.6902 537409 outcome TRUE reported
19237_17_DDT_D_dopachrome_decarboxylase DDT chr16 1710485 0.028296 6.883e-29
35320 TRUE reported textfile 2 1 TRUE
8041 rs118130999 C T C T 0.2328 -0.0235 0.0237 NA FALSE FALSE FALSE
GCST006061 0.0374 0.5299 537409 outcome TRUE reported
19237_17_DDT_D_dopachrome_decarboxylase DDT chr16 806052 0.026891 4.833e-18
35301 TRUE reported textfile 2 1 TRUE
8042 rs148117344 C T C T 0.1597 0.0138 0.02928 NA FALSE FALSE FALSE
GCST006061 0.0269 0.6084 537409 outcome TRUE reported
19237_17_DDT_D_dopachrome_decarboxylase DDT chr16 577359 0.023819 2.016e-11
35311 TRUE reported textfile 2 1 TRUE
8043 rs1707652 T C T C 0.0801 0.0081 0.3102 NA FALSE FALSE FALSE
GCST006061 0.0074 0.2701 537409 outcome TRUE reported
19237_17_DDT_D_dopachrome_decarboxylase DDT chr3 165761011 0.008691 3.08e-20
35339 TRUE reported textfile 2 1 TRUE
8044 rs41277305 C A C A 0.3432 0.0064 0.03485 NA FALSE FALSE FALSE
GCST006061 0.0235 0.7861 537409 outcome TRUE reported
19237_17_DDT_D_dopachrome_decarboxylase DDT chr22 24005171 0.022527 2.059e-52
35369 TRUE reported textfile 2 1 TRUE
8045 rs58596209 C T C T 0.4486 0.0203 0.01125 NA FALSE FALSE FALSE
GCST006061 0.0353 0.5642 537409 outcome TRUE reported
19237_17_DDT_D_dopachrome_decarboxylase DDT chr16 769008 0.037699 1.189e-32
35305 TRUE reported textfile 2 1 TRUE
8046 rs7187649 T G T G 0.1152 -0.0335 0.03987 NA FALSE FALSE FALSE
GCST006061 0.019 0.07674 537409 outcome TRUE reported
19237_17_DDT_D_dopachrome_decarboxylase DDT chr16 3982020 0.020498 1.908e-08
35351 TRUE reported textfile 2 1 TRUE
8047 rs10922098 T C T C -0.1262 -0.009 0.40011 NA FALSE FALSE FALSE
GCST006061 0.0075 0.2275 537409 outcome TRUE reported 5249_31_STK17B_DRAK2
STK17B chr1 196695521 0.008084 6.092e-55 35338 TRUE reported
textfile 2 1 TRUE
8048 rs6445035 A G A G 0.0618 0.0154 0.1904 NA FALSE FALSE FALSE

GCST006061 0.0089 0.08256 537409 outcome TRUE reported 5249_31_STK17B_DRAK2
STK17B chr3 165762312 0.010085 8.908e-10 35339 TRUE reported
textfile 2 1 TRUE
8049 rs10922098 T C T C -0.1014 -0.009 0.40011 NA FALSE FALSE FALSE
GCST006061 0.0075 0.2275 537409 outcome TRUE reported 10014_31_SNAI2_SLUG
SNAI2 chr1 196695521 0.008057 2.547e-36 35326 TRUE reported
textfile 2 1 TRUE
8050 rs1706435 G A G A 0.0541 0.0117 0.36981 NA FALSE FALSE FALSE
GCST006061 0.0072 0.1047 537409 outcome TRUE reported 10014_31_SNAI2_SLUG
SNAI2 chr3 165743688 0.008176 3.664e-11 35327 TRUE reported
textfile 2 1 TRUE
8051 rs10922098 T C T C -0.0538 -0.009 0.40011 NA FALSE FALSE FALSE
GCST006061 0.0075 0.2275 537409 outcome TRUE reported 12758_47_GRID2_GRID2
GRID2 chr1 196695521 0.008745 7.641e-10 35329 TRUE reported
textfile 2 1 TRUE
8052 rs117894413 A G A G 0.1462 -0.0281 0.02872 NA FALSE FALSE FALSE
GCST006061 0.0279 0.3153 537409 outcome TRUE reported 12758_47_GRID2_GRID2
GRID2 chr20 3835918 0.025674 1.238e-08 35362 TRUE reported textfile
2 1 TRUE
8053 rs12798553 A G A G 0.0638 0.0037 0.21904 NA FALSE FALSE FALSE
GCST006061 0.0091 0.6835 537409 outcome TRUE reported 12758_47_GRID2_GRID2
GRID2 chr11 123482985 0.01044 9.905e-10 35352 TRUE reported textfile
2 1 TRUE
8054 rs142279279 A G A G -0.1141 0.0057 0.08356 NA FALSE FALSE FALSE
GCST006061 0.014 0.6839 537409 outcome TRUE reported 12758_47_GRID2_GRID2
GRID2 chr2 118958929 0.015605 2.639e-13 35350 TRUE reported
textfile 2 1 TRUE
8055 rs146335091 A G A G 0.3116 0.0258 0.01066 NA FALSE FALSE FALSE
GCST006061 0.0469 0.5823 537409 outcome TRUE reported 12758_47_GRID2_GRID2
GRID2 chr14 104684180 0.042146 1.432e-13 35101 TRUE reported
textfile 2 1 TRUE
8056 rs147424791 A G A G 0.1409 -0.0612 0.03257 NA FALSE FALSE FALSE
GCST006061 0.038 0.1072 537409 outcome TRUE reported 12758_47_GRID2_GRID2
GRID2 chr20 3812584 0.024866 1.459e-08 35367 TRUE reported textfile
2 1 TRUE
8057 rs148813665 T C T C -0.1333 -0.0026 0.07095 NA FALSE FALSE FALSE
GCST006061 0.0186 0.8893 537409 outcome TRUE reported 12758_47_GRID2_GRID2
GRID2 chr6 261343 0.016994 4.369e-15 35300 TRUE reported textfile
2 1 TRUE
8058 rs17578693 C T C T 0.0758 0.0085 0.11123 NA FALSE FALSE FALSE
GCST006061 0.0131 0.5155 537409 outcome TRUE reported 12758_47_GRID2_GRID2
GRID2 chr1 234566161 0.0137 3.15e-08 35352 TRUE reported textfile
2 1 TRUE
8059 rs2295616 A G A G -0.05 0.0018 0.33112 NA FALSE FALSE FALSE
GCST006061 0.0078 0.8148 537409 outcome TRUE reported 12758_47_GRID2_GRID2
GRID2 chr1 160740367 0.009078 3.63e-08 35352 TRUE reported
textfile 2 1 TRUE
8060 rs2511737 C A C A -0.0812 -0.0263 0.27692 NA FALSE FALSE FALSE
GCST006061 0.0084 0.001647 537409 outcome TRUE reported
12758_47_GRID2_GRID2 GRID2 chr8 102568299 0.009572 2.197e-17 35351
TRUE reported textfile 2 1 TRUE
8061 rs3810560 C T C T -0.0786 -0.0033 0.43172 NA FALSE FALSE FALSE
GCST006061 0.0075 0.661 537409 outcome TRUE reported 12758_47_GRID2_GRID2
GRID2 chr20 3227173 0.008664 1.165e-19 35369 TRUE reported textfile
2 1 TRUE
8062 rs6679677 A C A C -0.0817 -0.0086 0.13318 NA FALSE FALSE FALSE
GCST006061 0.0124 0.487 537409 outcome TRUE reported 12758_47_GRID2_GRID2
GRID2 chr1 113761186 0.012691 1.212e-10 35341 TRUE reported
textfile 2 1 TRUE
8063 rs6891054 G A G A -0.0577 -0.0176 0.25565 NA FALSE FALSE FALSE
GCST006061 0.0081 0.03003 537409 outcome TRUE reported 12758_47_GRID2_GRID2
GRID2 chr5 125005010 0.009831 4.385e-09 35345 TRUE reported
textfile 2 1 TRUE
8064 rs76106586 G A G A 0.2351 -0.0133 0.02363 NA FALSE FALSE FALSE
GCST006061 0.0244 0.5858 537409 outcome TRUE reported 12758_47_GRID2_GRID2
GRID2 chr6 483593 0.02777 2.539e-17 35306 TRUE reported textfile 2

1 TRUE
8065 rs7785014 C T C T 0.0639 0.008 0.2888 NA FALSE FALSE FALSE GCST006061 0.0078 0.3034 537409 outcome TRUE reported 12758_47_GRID2_GRID2 GRID2 chr7 138196944 0.009436 1.271e-11 35365 TRUE reported textfile 2 1 TRUE
8066 rs79395339 C T C T -0.1673 -0.0409 0.02357 NA FALSE FALSE FALSE GCST006061 0.0273 0.1349 537409 outcome TRUE reported 12758_47_GRID2_GRID2 GRID2 chr20 3755220 0.028578 4.796e-09 35366 TRUE reported textfile 2 1 TRUE
8067 rs9302635 C T C T -0.0671 0.0086 0.19843 NA FALSE FALSE FALSE GCST006061 0.0095 0.367 537409 outcome TRUE reported 12758_47_GRID2_GRID2 GRID2 chr16 72110275 0.010595 2.405e-10 35366 TRUE reported textfile 2 1 TRUE
8068 rs10922098 T C T C -0.1522 -0.009 0.40011 NA FALSE FALSE FALSE GCST006061 0.0075 0.2275 537409 outcome TRUE reported 18285_6_SVIP_SVIP SVIP chr1 196695521 0.008111 1.5e-78 35338 TRUE reported textfile 2 1 TRUE
8069 rs1706435 G A G A 0.075 0.0117 0.36981 NA FALSE FALSE FALSE GCST006061 0.0072 0.1047 537409 outcome TRUE reported 18285_6_SVIP_SVIP SVIP chr3 165743688 0.008235 8.406e-20 35339 TRUE reported textfile 2 1 TRUE
8070 rs75380382 T C T C 0.1989 -0.0511 0.01283 NA FALSE FALSE FALSE GCST006061 0.0494 0.301 537409 outcome TRUE reported 18285_6_SVIP_SVIP SVIP chr6 23858567 0.034413 7.481e-09 35369 TRUE reported textfile 2 1 TRUE
8071 rs10922098 T C T C -0.1467 -0.009 0.40011 NA FALSE FALSE FALSE GCST006061 0.0075 0.2275 537409 outcome TRUE reported 8380_244_DLK1_DLK1 DLK1 chr1 196695521 0.008134 1.033e-72 35338 TRUE reported textfile 2 1 TRUE
8072 rs1707652 T C T C 0.0839 0.0081 0.3102 NA FALSE FALSE FALSE GCST006061 0.0074 0.2701 537409 outcome TRUE reported 8380_244_DLK1_DLK1 DLK1 chr3 165761011 0.008604 1.821e-22 35339 TRUE reported textfile 2 1 TRUE
8073 rs10922098 T C T C -0.0694 -0.009 0.40011 NA FALSE FALSE FALSE GCST006061 0.0075 0.2275 537409 outcome TRUE reported 17357_33_SEPSECS_SPCS SEPSECS chr1 196695521 0.008117 1.236e-17 35338 TRUE reported textfile 2 1 TRUE
8074 rs61923082 C A C A -0.0899 0.0038 0.0616 NA FALSE FALSE FALSE GCST006061 0.0164 0.8176 537409 outcome TRUE reported 17357_33_SEPSECS_SPCS SEPSECS chr12 46179199 0.016485 4.941e-08 35361 TRUE reported textfile 2 1 TRUE
8075 rs6448333 T C T C -0.1556 -0.008 0.03656 NA FALSE FALSE FALSE GCST006061 0.0166 0.629 537409 outcome TRUE reported 17357_33_SEPSECS_SPCS SEPSECS chr4 25115419 0.020634 4.663e-14 35363 TRUE reported textfile 2 1 TRUE
8076 rs10922098 T C T C -0.1068 -0.009 0.40011 NA FALSE FALSE FALSE GCST006061 0.0075 0.2275 537409 outcome TRUE reported 7246_4_FREM2_FREM2 FREM2 chr1 196695521 0.008039 2.835e-40 35338 TRUE reported textfile 2 1 TRUE
8077 rs1706435 G A G A 0.0678 0.0117 0.36981 NA FALSE FALSE FALSE GCST006061 0.0072 0.1047 537409 outcome TRUE reported 7246_4_FREM2_FREM2 FREM2 chr3 165743688 0.00815 8.88e-17 35339 TRUE reported textfile 2 1 TRUE
8078 rs10922098 T C T C -0.1414 -0.009 0.40011 NA FALSE FALSE FALSE GCST006061 0.0075 0.2275 537409 outcome TRUE reported 6624_94_WNT11_WNT11 WNT11 chr1 196695521 0.008112 4.744e-68 35338 TRUE reported textfile 2 1 TRUE
8079 rs1706435 G A G A 0.0725 0.0117 0.36981 NA FALSE FALSE FALSE GCST006061 0.0072 0.1047 537409 outcome TRUE reported 6624_94_WNT11_WNT11 WNT11 chr3 165743688 0.00823 1.26e-18 35339 TRUE reported textfile 2 1 TRUE
8080 rs10922098 T C T C -0.0613 -0.009 0.40011 NA FALSE FALSE FALSE GCST006061 0.0075 0.2275 537409 outcome TRUE reported 10512_13_CSF2RB_IL3RB CSF2RB chr1 196695521 0.008461 4.334e-13 35326 TRUE reported textfile 2 1 TRUE
8081 rs113908050 C T C T 0.1512 -0.0319 0.03576 NA FALSE FALSE FALSE

GCST006061 0.0236 0.1759 537409 outcome TRUE reported 10512_13_CSF2RB_IL3RB
CSF2RB chr22 38291687 0.023045 5.342e-11 35348 TRUE reported
textfile 2 1 TRUE

8082 rs145143750 A C A C -0.1731 0.0444 0.018 NA FALSE FALSE FALSE
GCST006061 0.0421 0.2919 537409 outcome TRUE reported 10512_13_CSF2RB_IL3RB
CSF2RB chr22 37508521 0.031375 3.446e-08 35356 TRUE reported
textfile 2 1 TRUE

8083 rs2284031 C T C T -0.3277 -0.0071 0.40547 NA FALSE FALSE FALSE
GCST006061 0.0077 0.3529 537409 outcome TRUE reported 10512_13_CSF2RB_IL3RB
CSF2RB chr22 36925038 0.007129 1e-200 35356 TRUE reported textfile
2 1 TRUE

8084 rs2858236 C A C A -0.0958 -0.0116 0.38867 NA FALSE FALSE FALSE
GCST006061 0.0077 0.1321 537409 outcome TRUE reported 10512_13_CSF2RB_IL3RB
CSF2RB chr22 36971924 0.008724 4.705e-28 35360 TRUE reported
textfile 2 1 TRUE

8085 rs77818863 G A G A 0.2203 -0.0084 0.03052 NA FALSE FALSE FALSE
GCST006061 0.0213 0.6922 537409 outcome TRUE reported 10512_13_CSF2RB_IL3RB
CSF2RB chr22 36916259 0.024858 7.834e-19 35355 TRUE reported
textfile 2 1 TRUE

8086 rs78469 T G T G 0.0501 -0.0028 0.43385 NA FALSE FALSE FALSE GCST006061
0.0077 0.7138 537409 outcome TRUE reported 10512_13_CSF2RB_IL3RB CSF2RB
chr22 36263142 0.008624 6.259e-09 35356 TRUE reported textfile 2
1 TRUE

8087 rs10922098 T C T C -0.1858 -0.009 0.40011 NA FALSE FALSE FALSE
GCST006061 0.0075 0.2275 537409 outcome TRUE reported 11656_110_EVL_EVL EVL
chr1 196695521 0.00813 1.351e-115 35329 TRUE reported textfile 2 1
TRUE

8088 rs117136557 C T C T 0.1671 0.0221 0.0354 NA FALSE FALSE FALSE
GCST006061 0.0348 0.5245 537409 outcome TRUE reported 11656_110_EVL_EVL EVL
chr14 99822278 0.021457 6.822e-15 35373 TRUE reported textfile 2
1 TRUE

8089 rs144704969 T C T C 0.1074 -0.0238 0.04536 NA FALSE FALSE FALSE
GCST006061 0.0154 0.1235 537409 outcome TRUE reported 11656_110_EVL_EVL EVL
chr1 196158214 0.019071 1.785e-08 35328 TRUE reported textfile 2
1 TRUE

8090 rs1707652 T C T C 0.0992 0.0081 0.3102 NA FALSE FALSE FALSE
GCST006061 0.0074 0.2701 537409 outcome TRUE reported 11656_110_EVL_EVL EVL
chr3 165761011 0.008616 1.123e-30 35330 TRUE reported textfile 2
1 TRUE

8091 rs17099126 A G A G 0.1573 -0.0108 0.03319 NA FALSE FALSE FALSE
GCST006061 0.0258 0.677 537409 outcome TRUE reported 11656_110_EVL_EVL EVL
chr14 99846720 0.022331 1.866e-12 35374 TRUE reported textfile 2
1 TRUE

8092 rs2767355 A G A G -0.1023 -2.00E-04 0.16134 NA FALSE FALSE FALSE
GCST006061 0.0101 0.9831 537409 outcome TRUE reported 11656_110_EVL_EVL EVL
chr14 100016044 0.010836 3.704e-21 35375 TRUE reported textfile 2
1 TRUE

8093 rs10922098 T C T C -0.1221 -0.009 0.40011 NA FALSE FALSE FALSE
GCST006061 0.0075 0.2275 537409 outcome TRUE reported 7895_108_MRAP_MRAP
MRAP chr1 196695521 0.008094 2.033e-51 35338 TRUE reported
textfile 2 1 TRUE

8094 rs6542680 T C T C -0.0657 -0.0012 0.18362 NA FALSE FALSE FALSE
GCST006061 0.0091 0.8935 537409 outcome TRUE reported 7895_108_MRAP_MRAP
MRAP chr2 3592552 0.010278 1.633e-10 35332 TRUE reported textfile
2 1 TRUE

8095 rs10922098 T C T C -0.0483 -0.009 0.40011 NA FALSE FALSE FALSE
GCST006061 0.0075 0.2275 537409 outcome TRUE reported 2944_66_NBL1_DAN
NBL1 chr1 196695521 0.008202 3.886e-09 35338 TRUE reported
textfile 2 1 TRUE

8096 rs117042741 T C T C -0.1374 0.031 0.05224 NA FALSE FALSE FALSE
GCST006061 0.0194 0.1103 537409 outcome TRUE reported 2944_66_NBL1_DAN
NBL1 chr11 74917698 0.017966 2.042e-14 35369 TRUE reported
textfile 2 1 TRUE

8097 rs12408663 C T C T -0.0602 0.002 0.21403 NA FALSE FALSE FALSE
GCST006061 0.0084 0.81 537409 outcome TRUE reported 2944_66_NBL1_DAN
NBL1 chr1 19641564 0.009845 9.671e-10 35379 TRUE reported

textfile 2 1 TRUE
8098 rs4002471 T C T C -0.0508 0.0025 0.36751 NA FALSE FALSE FALSE
GCST006061 0.0076 0.74 537409 outcome TRUE reported 2944_66_NBL1_DAN
NBL1 chr19 48711838 0.008366 1.259e-09 35378 TRUE reported
textfile 2 1 TRUE
8099 rs4911994 A C A C -0.0534 -0.012 0.36807 NA FALSE FALSE FALSE
GCST006061 0.0074 0.1032 537409 outcome TRUE reported 2944_66_NBL1_DAN
NBL1 chr1 19435099 0.008324 1.403e-10 35379 TRUE reported
textfile 2 1 TRUE
8100 rs62061425 G A G A 0.0654 0.001 0.21627 NA FALSE FALSE FALSE
GCST006061 0.0097 0.9151 537409 outcome TRUE reported 2944_66_NBL1_DAN
NBL1 chr17 7169772 0.009807 2.583e-11 35370 TRUE reported textfile
2 1 TRUE
8101 rs72704449 C T C T 0.1054 0.013 0.04582 NA FALSE FALSE FALSE
GCST006061 0.0199 0.5128 537409 outcome TRUE reported 2944_66_NBL1_DAN
NBL1 chr1 179504138 0.019139 3.647e-08 35358 TRUE reported
textfile 2 1 TRUE
8102 rs77924615 A G A G -0.0682 0.0136 0.19701 NA FALSE FALSE FALSE
GCST006061 0.0092 0.1375 537409 outcome TRUE reported 2944_66_NBL1_DAN
NBL1 chr16 20381010 0.010166 1.968e-11 35355 TRUE reported
textfile 2 1 TRUE
8103 rs10922098 T C T C -0.0661 -0.009 0.40011 NA FALSE FALSE FALSE
GCST006061 0.0075 0.2275 537409 outcome TRUE reported
12976_49_SORBS3_Vinexin_b SORBS3 chr1 196695521 0.007969 1.09e-16 35329
TRUE reported textfile 2 1 TRUE
8104 rs1355538 G A G A -0.0494 -0.0076 0.39847 NA FALSE FALSE FALSE
GCST006061 0.0072 0.2923 537409 outcome TRUE reported
12976_49_SORBS3_Vinexin_b SORBS3 chr3 165787389 0.007967 5.63e-10 35329
TRUE reported textfile 2 1 TRUE
8105 rs10922098 T C T C -0.1733 -0.009 0.40011 NA FALSE FALSE FALSE
GCST006061 0.0075 0.2275 537409 outcome TRUE reported 13059_33_RFK_RIFK RFK
chr1 196695521 0.008037 4.128e-103 35329 TRUE reported textfile 2
1 TRUE
8106 rs11144910 T G T G -0.0745 -0.0204 0.18124 NA FALSE FALSE FALSE
GCST006061 0.0106 0.05346 537409 outcome TRUE reported 13059_33_RFK_RIFK RFK
chr9 76457209 0.01025 3.634e-13 35343 TRUE reported textfile 2 1
TRUE
8107 rs28645249 C T C T 0.0666 0.019 0.26162 NA FALSE FALSE FALSE
GCST006061 0.0115 0.09815 537409 outcome TRUE reported 13059_33_RFK_RIFK RFK
chr6 32557458 0.008664 1.509e-14 35429 TRUE reported textfile 2
1 TRUE
8108 rs591411 T C T C 0.129 0.0024 0.23986 NA FALSE FALSE FALSE
GCST006061 0.0087 0.7832 537409 outcome TRUE reported 13059_33_RFK_RIFK RFK
chr9 76698613 0.009253 3.566e-44 35343 TRUE reported textfile 2
1 TRUE
8109 rs10922098 T C T C -0.1718 -0.009 0.40011 NA FALSE FALSE FALSE
GCST006061 0.0075 0.2275 537409 outcome TRUE reported 9495_10_CXCL17_VCC1
CXCL17 chr1 196695521 0.008114 1.664e-99 35338 TRUE reported
textfile 2 1 TRUE
8110 rs1707652 T C T C 0.0978 0.0081 0.3102 NA FALSE FALSE FALSE
GCST006061 0.0074 0.2701 537409 outcome TRUE reported 9495_10_CXCL17_VCC1
CXCL17 chr3 165761011 0.008598 5.604e-30 35339 TRUE reported
textfile 2 1 TRUE
8111 rs10922098 T C T C -0.1144 -0.009 0.40011 NA FALSE FALSE FALSE
GCST006061 0.0075 0.2275 537409 outcome TRUE reported 17703_40_GMNN_GEMI
GMNN chr1 196695521 0.008064 1.11e-45 35338 TRUE reported
textfile 2 1 TRUE
8112 rs1355538 G A G A -0.0561 -0.0076 0.39847 NA FALSE FALSE FALSE
GCST006061 0.0072 0.2923 537409 outcome TRUE reported 17703_40_GMNN_GEMI
GMNN chr3 165787389 0.008073 3.678e-12 35338 TRUE reported
textfile 2 1 TRUE
8113 rs10922098 T C T C -0.0452 -0.009 0.40011 NA FALSE FALSE FALSE
GCST006061 0.0075 0.2275 537409 outcome TRUE reported 3191_50_WFIKKN1_WFKN1
WFIKKN1 chr1 196695521 0.008261 4.465e-08 35338 TRUE reported
textfile 2 1 TRUE
8114 rs116914290 T C T C 0.3123 0.0317 0.01924 NA FALSE FALSE FALSE

GCST006061 0.0402 0.4301 537409 outcome TRUE reported 3191_50_WFIKKN1_WFKN1
WFIKKN1 chr16 700179 0.028551 7.557e-28 35311 TRUE reported textfile
2 1 TRUE
8115 rs3184504 C T C T -0.0573 0.0074 0.37862 NA FALSE FALSE FALSE
GCST006061 0.0075 0.3235 537409 outcome TRUE reported 3191_50_WFIKKN1_WFKN1
WFIKKN1 chr12 111446804 0.008349 6.72e-12 35371 TRUE reported
textfile 2 1 TRUE
8116 rs4432538 A G A G 0.0613 0.0193 0.4912 NA FALSE FALSE FALSE
GCST006061 0.0072 0.007157 537409 outcome TRUE reported
3191_50_WFIKKN1_WFKN1 WFIKKN1 chr20 8626746 0.008092 3.583e-14 35369 TRUE
reported textfile 2 1 TRUE
8117 rs55798945 G T G T 0.1487 -8.00E-04 0.36015 NA FALSE FALSE FALSE
GCST006061 0.0086 0.9223 537409 outcome TRUE reported 3191_50_WFIKKN1_WFKN1
WFIKKN1 chr16 619708 0.008403 4.497e-70 35313 TRUE reported textfile
2 1 TRUE
8118 rs10922098 T C T C -0.1915 -0.009 0.40011 NA FALSE FALSE FALSE
GCST006061 0.0075 0.2275 537409 outcome TRUE reported 17209_27_SULT2B1_ST2B1
SULT2B1 chr1 196695521 0.008135 1.541e-122 35338 TRUE reported
textfile 2 1 TRUE
8119 rs11569415 A G A G -0.0557 0.0011 0.24276 NA FALSE FALSE FALSE
GCST006061 0.0097 0.9129 537409 outcome TRUE reported 17209_27_SULT2B1_ST2B1
SULT2B1 chr19 6716268 0.00929 2.03e-09 35372 TRUE reported textfile 2
1 TRUE
8120 rs2232613 T C T C -0.1125 0.0151 0.0989 NA FALSE FALSE FALSE
GCST006061 0.0141 0.2834 537409 outcome TRUE reported 17209_27_SULT2B1_ST2B1
SULT2B1 chr20 38369011 0.013198 1.543e-17 35375 TRUE reported
textfile 2 1 TRUE
8121 rs2686395 C T C T -0.1201 -0.0077 0.3101 NA FALSE FALSE FALSE
GCST006061 0.0074 0.2965 537409 outcome TRUE reported 17209_27_SULT2B1_ST2B1
SULT2B1 chr3 165767231 0.008616 3.688e-44 35339 TRUE reported
textfile 2 1 TRUE
8122 rs10922098 T C T C -0.0935 -0.009 0.40011 NA FALSE FALSE FALSE
GCST006061 0.0075 0.2275 537409 outcome TRUE reported 12681_63_USP21_UBP21
USP21 chr1 196695521 0.008062 4.235e-31 35329 TRUE reported
textfile 2 1 TRUE
8123 rs1354034 C T C T 0.051 0.0088 0.38119 NA FALSE FALSE FALSE
GCST006061 0.0073 0.2248 537409 outcome TRUE reported 12681_63_USP21_UBP21
USP21 chr3 56815721 0.008126 3.469e-10 35347 TRUE reported
textfile 2 1 TRUE
8124 rs1706435 G A G A 0.0783 0.0117 0.36981 NA FALSE FALSE FALSE
GCST006061 0.0072 0.1047 537409 outcome TRUE reported 12681_63_USP21_UBP21
USP21 chr3 165743688 0.00817 9.298e-22 35330 TRUE reported textfile
2 1 TRUE
8125 rs10922098 T C T C -0.0809 -0.009 0.40011 NA FALSE FALSE FALSE
GCST006061 0.0075 0.2275 537409 outcome TRUE reported 3143_3_CD4_sCD4 CD4
chr1 196695521 0.008103 1.782e-23 35338 TRUE reported textfile 2
1 TRUE
8126 rs61916271 A G A G -0.0607 0.0051 0.20984 NA FALSE FALSE FALSE
GCST006061 0.0095 0.5925 537409 outcome TRUE reported 3143_3_CD4_sCD4 CD4
chr12 6781356 0.009746 4.715e-10 35391 TRUE reported textfile 2 1
TRUE
8127 rs7137828 T C T C -0.0453 0.0076 0.38049 NA FALSE FALSE FALSE
GCST006061 0.0075 0.3144 537409 outcome TRUE reported 3143_3_CD4_sCD4 CD4
chr12 111494996 0.008185 3.123e-08 35371 TRUE reported textfile 2
1 TRUE
8128 rs10922098 T C T C -0.1399 -0.009 0.40011 NA FALSE FALSE FALSE
GCST006061 0.0075 0.2275 537409 outcome TRUE reported 3809_1_FGFR3_FGFR_3
FGFR3 chr1 196695521 0.008109 1.092e-66 35338 TRUE reported
textfile 2 1 TRUE
8129 rs11569415 A G A G -0.0521 0.0011 0.24276 NA FALSE FALSE FALSE
GCST006061 0.0097 0.9129 537409 outcome TRUE reported 3809_1_FGFR3_FGFR_3
FGFR3 chr19 6716268 0.00924 1.717e-08 35372 TRUE reported textfile 2
1 TRUE
8130 rs1355538 G A G A -0.0696 -0.0076 0.39847 NA FALSE FALSE FALSE
GCST006061 0.0072 0.2923 537409 outcome TRUE reported 3809_1_FGFR3_FGFR_3
FGFR3 chr3 165787389 0.008115 9.804e-18 35338 TRUE reported

textfile 2 1 TRUE
8131 rs10922098 T C T C -0.2203 -0.009 0.40011 NA FALSE FALSE FALSE GCST006061 0.0075 0.2275 537409 outcome TRUE reported 14028_22_PIK3C2A_P3C2A PIK3C2A chr1 196695521 0.008186 1.653e-159 35338 TRUE reported textfile 2 1 TRUE
8132 rs2686395 C T C T -0.1051 -0.0077 0.3101 NA FALSE FALSE FALSE GCST006061 0.0074 0.2965 537409 outcome TRUE reported 14028_22_PIK3C2A_P3C2A PIK3C2A chr3 165767231 0.008693 1.199e-33 35339 TRUE reported textfile 2 1 TRUE
8133 rs35351085 T C T C 0.1095 0.0061 0.04877 NA FALSE FALSE FALSE GCST006061 0.022 0.7825 537409 outcome TRUE reported 14028_22_PIK3C2A_P3C2A PIK3C2A chr1 197992799 0.018637 4.214e-09 35343 TRUE reported textfile 2 1 TRUE
8134 rs10922098 T C T C -0.1041 -0.009 0.40011 NA FALSE FALSE FALSE GCST006061 0.0075 0.2275 537409 outcome TRUE reported 13451_2_FNDC4_FNDC4 FNDC4 chr1 196695521 0.008031 2.02e-38 35332 TRUE reported textfile 2 1 TRUE
8135 rs1706435 G A G A 0.0516 0.0117 0.36981 NA FALSE FALSE FALSE GCST006061 0.0072 0.1047 537409 outcome TRUE reported 13451_2_FNDC4_FNDC4 FNDC4 chr3 165743688 0.008147 2.4e-10 35333 TRUE reported textfile 2 1 TRUE
8136 rs10922098 T C T C -0.0661 -0.009 0.40011 NA FALSE FALSE FALSE GCST006061 0.0075 0.2275 537409 outcome TRUE reported 16298_84_PLA2G2D_sPLA_2__IID PLA2G2D chr1 196695521 0.008019 1.684e-16 35338 TRUE reported textfile 2 1 TRUE
8137 rs4977155 C T C T -0.0601 -0.0069 0.16663 NA FALSE FALSE FALSE GCST006061 0.0127 0.5868 537409 outcome TRUE reported 16298_84_PLA2G2D_sPLA_2__IID PLA2G2D chr14 106473508 0.010769 2.396e-08 34966 TRUE reported textfile 2 1 TRUE
8138 rs10922098 T C T C -0.1105 -0.009 0.40011 NA FALSE FALSE FALSE GCST006061 0.0075 0.2275 537409 outcome TRUE reported 13977_28_BARD1_BARD1 BARD1 chr1 196695521 0.008054 7.639e-43 35338 TRUE reported textfile 2 1 TRUE
8139 rs1707652 T C T C 0.0674 0.0081 0.3102 NA FALSE FALSE FALSE GCST006061 0.0074 0.2701 537409 outcome TRUE reported 13977_28_BARD1_BARD1 BARD1 chr3 165761011 0.008523 2.608e-15 35339 TRUE reported textfile 2 1 TRUE
8140 rs41446546 T C T C 0.1245 -0.0088 0.03118 NA FALSE FALSE FALSE GCST006061 0.0262 0.7371 537409 outcome TRUE reported 13977_28_BARD1_BARD1 BARD1 chr18 48197233 0.022829 4.938e-08 35366 TRUE reported textfile 2 1 TRUE
8141 rs10922098 T C T C -0.1831 -0.009 0.40011 NA FALSE FALSE FALSE GCST006061 0.0075 0.2275 537409 outcome TRUE reported 11709_29_CPT1B_CPT1B CPT1B chr1 196695521 0.008142 5.306e-112 35329 TRUE reported textfile 2 1 TRUE
8142 rs111696008 A G A G -0.3249 -0.0259 0.02443 NA FALSE FALSE FALSE GCST006061 0.0401 0.519 537409 outcome TRUE reported 11709_29_CPT1B_CPT1B CPT1B chr2 216622986 0.026554 2.015e-34 35350 TRUE reported textfile 2 1 TRUE
8143 rs115041442 T C T C -0.1404 -0.0276 0.02598 NA FALSE FALSE FALSE GCST006061 0.0475 0.5604 537409 outcome TRUE reported 11709_29_CPT1B_CPT1B CPT1B chr1 152992214 0.025545 3.879e-08 35353 TRUE reported textfile 2 1 TRUE
8144 rs117192879 T C T C 0.1252 0.0485 0.03114 NA FALSE FALSE FALSE GCST006061 0.0335 0.1485 537409 outcome TRUE reported 11709_29_CPT1B_CPT1B CPT1B chr11 116283303 0.022609 3.067e-08 35366 TRUE reported textfile 2 1 TRUE
8145 rs12026141 T C T C -0.0744 0.0094 0.31246 NA FALSE FALSE FALSE GCST006061 0.0076 0.2137 537409 outcome TRUE reported 11709_29_CPT1B_CPT1B CPT1B chr1 201980465 0.008695 1.165e-17 35348 TRUE reported textfile 2 1 TRUE
8146 rs12485327 T G T G 0.109 0.0109 0.07334 NA FALSE FALSE FALSE GCST006061 0.0118 0.3548 537409 outcome TRUE reported 11709_29_CPT1B_CPT1B CPT1B chr3 162196930 0.015572 2.565e-12 35331 TRUE reported textfile 2 1 TRUE
8147 rs142437389 T C T C 0.1799 -0.0259 0.01603 NA FALSE FALSE FALSE

GCST006061 0.0431 0.5485 537409 outcome TRUE reported 11709_29_CPT1B_CPT1B
CPT1B chr11 119018348 0.031928 1.756e-08 35369 TRUE reported
textfile 2 1 TRUE
8148 rs144202860 A G A G -0.3141 -0.0629 0.01274 NA FALSE FALSE FALSE
GCST006061 0.0538 0.2423 537409 outcome TRUE reported 11709_29_CPT1B_CPT1B
CPT1B chr2 216625740 0.035572 1.047e-18 35350 TRUE reported
textfile 2 1 TRUE
8149 rs483465 G A G A 0.0576 -0.0204 0.24663 NA FALSE FALSE FALSE
GCST006061 0.0083 0.01415 537409 outcome TRUE reported 11709_29_CPT1B_CPT1B
CPT1B chr3 136329135 0.009397 8.818e-10 35352 TRUE reported
textfile 2 1 TRUE
8150 rs55881728 G A G A -0.071 -0.0072 0.41277 NA FALSE FALSE FALSE
GCST006061 0.0074 0.3268 537409 outcome TRUE reported 11709_29_CPT1B_CPT1B
CPT1B chr2 203441633 0.008166 3.467e-18 35356 TRUE reported
textfile 2 1 TRUE
8151 rs72939784 C T C T -0.1079 0.0087 0.04841 NA FALSE FALSE FALSE
GCST006061 0.0184 0.6379 537409 outcome TRUE reported 11709_29_CPT1B_CPT1B
CPT1B chr6 105399298 0.018796 9.43e-09 35344 TRUE reported
textfile 2 1 TRUE
8152 rs73015723 C T C T 0.1331 0.0244 0.0347 NA FALSE FALSE FALSE
GCST006061 0.0242 0.3139 537409 outcome TRUE reported 11709_29_CPT1B_CPT1B
CPT1B chr11 118069105 0.021545 6.497e-10 35370 TRUE reported
textfile 2 1 TRUE
8153 rs74683816 A G A G 0.1407 -0.0057 0.03162 NA FALSE FALSE FALSE
GCST006061 0.0215 0.7901 537409 outcome TRUE reported 11709_29_CPT1B_CPT1B
CPT1B chr11 117855388 0.022594 4.741e-10 35365 TRUE reported
textfile 2 1 TRUE
8154 rs77368797 T G T G 0.1871 -0.015 0.0231 NA FALSE FALSE FALSE
GCST006061 0.0286 0.6004 537409 outcome TRUE reported 11709_29_CPT1B_CPT1B
CPT1B chr11 116505867 0.026586 1.957e-12 35364 TRUE reported
textfile 2 1 TRUE
8155 rs77998935 A G A G 0.1415 -0.014 0.02293 NA FALSE FALSE FALSE
GCST006061 0.0434 0.7476 537409 outcome TRUE reported 11709_29_CPT1B_CPT1B
CPT1B chr11 117912877 0.025902 4.685e-08 35365 TRUE reported
textfile 2 1 TRUE
8156 rs79330559 T C T C 0.2399 0.0142 0.01152 NA FALSE FALSE FALSE
GCST006061 0.0409 0.7273 537409 outcome TRUE reported 11709_29_CPT1B_CPT1B
CPT1B chr3 165020966 0.037712 2e-10 35334 TRUE reported textfile
2 1 TRUE
8157 rs10922098 T C T C -0.0925 -0.009 0.40011 NA FALSE FALSE FALSE
GCST006061 0.0075 0.2275 537409 outcome TRUE reported 9583_17_RNF24_RNF24
RNF24 chr1 196695521 0.008219 2.214e-29 35338 TRUE reported
textfile 2 1 TRUE
8158 rs111696008 A G A G -0.1623 -0.0259 0.02443 NA FALSE FALSE FALSE
GCST006061 0.0401 0.519 537409 outcome TRUE reported 9583_17_RNF24_RNF24
RNF24 chr2 216622986 0.026747 1.295e-09 35359 TRUE reported
textfile 2 1 TRUE
8159 rs11906988 G T G T -0.2969 -0.0389 0.01085 NA FALSE FALSE FALSE
GCST006061 0.0283 0.1697 537409 outcome TRUE reported 9583_17_RNF24_RNF24
RNF24 chr20 38383368 0.038276 8.712e-15 35372 TRUE reported
textfile 2 1 TRUE
8160 rs2232613 T C T C -0.4023 0.0151 0.0989 NA FALSE FALSE FALSE
GCST006061 0.0141 0.2834 537409 outcome TRUE reported 9583_17_RNF24_RNF24
RNF24 chr20 38369011 0.01317 1e-200 35375 TRUE reported textfile 2
1 TRUE
8161 rs56317765 T C T C 0.0743 -0.0069 0.15401 NA FALSE FALSE FALSE
GCST006061 0.0104 0.5072 537409 outcome TRUE reported 9583_17_RNF24_RNF24
RNF24 chr20 37489853 0.011195 3.21e-11 35369 TRUE reported
textfile 2 1 TRUE
8162 rs6029138 A C A C -0.0679 -0.0122 0.18997 NA FALSE FALSE FALSE
GCST006061 0.0101 0.2256 537409 outcome TRUE reported 9583_17_RNF24_RNF24
RNF24 chr20 40484266 0.010294 4.215e-11 35373 TRUE reported
textfile 2 1 TRUE
8163 rs10922098 T C T C -0.1102 -0.009 0.40011 NA FALSE FALSE FALSE
GCST006061 0.0075 0.2275 537409 outcome TRUE reported 16877_19_MSRB2_MSRB2
MSRB2 chr1 196695521 0.008108 4.47e-42 35338 TRUE reported

textfile 2 1 TRUE
8164 rs3917538 A G A G 0.0963 -0.0137 0.21967 NA FALSE FALSE FALSE GCST006061 0.0082 0.09535 537409 outcome TRUE reported 16877_19_MSRB2_MSRB2 MSRB2 chr7 95308581 0.009585 9.487e-24 35349 TRUE reported textfile 2 1 TRUE
8165 rs10922098 T C T C -0.0525 -0.009 0.40011 NA FALSE FALSE FALSE GCST006061 0.0075 0.2275 537409 outcome TRUE reported 18918_86_PDE4A_PDE4A PDE4A chr1 196695521 0.008095 8.865e-11 35338 TRUE reported textfile 2 1 TRUE
8166 rs11667876 G A G A 0.1558 -0.0119 0.02301 NA FALSE FALSE FALSE GCST006061 0.0339 0.7262 537409 outcome TRUE reported 18918_86_PDE4A_PDE4A PDE4A chr19 10104536 0.02645 3.853e-09 35369 TRUE reported textfile 2 1 TRUE
8167 rs138570417 G T G T 0.2459 -0.0135 0.01231 NA FALSE FALSE FALSE GCST006061 0.0455 0.7659 537409 outcome TRUE reported 18918_86_PDE4A_PDE4A PDE4A chr19 9760047 0.037144 3.587e-11 35380 TRUE reported textfile 2 1 TRUE
8168 rs146704942 A C A C 0.2471 0.0396 0.01114 NA FALSE FALSE FALSE GCST006061 0.0491 0.4206 537409 outcome TRUE reported 18918_86_PDE4A_PDE4A PDE4A chr19 12541989 0.03838 1.209e-10 35380 TRUE reported textfile 2 1 TRUE
8169 rs62269916 A G A G 0.1236 -0.0234 0.03471 NA FALSE FALSE FALSE GCST006061 0.0238 0.3259 537409 outcome TRUE reported 18918_86_PDE4A_PDE4A PDE4A chr3 74348841 0.021732 1.29e-08 35343 TRUE reported textfile 2 1 TRUE
8170 rs77897491 T C T C 0.2451 0.1195 0.01181 NA FALSE FALSE FALSE GCST006061 0.0635 0.05961 537409 outcome TRUE reported 18918_86_PDE4A_PDE4A PDE4A chr19 11596519 0.037319 5.11e-11 35383 TRUE reported textfile 2 1 TRUE
8171 rs79803848 A G A G 0.1895 4e-04 0.01586 NA FALSE FALSE FALSE GCST006061 0.0276 0.9894 537409 outcome TRUE reported 18918_86_PDE4A_PDE4A PDE4A chr19 10071236 0.032496 5.495e-09 35373 TRUE reported textfile 2 1 TRUE
8172 rs10922098 T C T C -0.0588 -0.009 0.40011 NA FALSE FALSE FALSE GCST006061 0.0075 0.2275 537409 outcome TRUE reported 11557_3_SMURF1_SMUF1 SMURF1 chr1 196695521 0.00811 4.148e-13 35329 TRUE reported textfile 2 1 TRUE
8173 rs1355538 G A G A -0.0485 -0.0076 0.39847 NA FALSE FALSE FALSE GCST006061 0.0072 0.2923 537409 outcome TRUE reported 11557_3_SMURF1_SMUF1 SMURF1 chr3 165787389 0.008102 2.148e-09 35329 TRUE reported textfile 2 1 TRUE
8174 rs4419136 G A G A -0.1317 6e-04 0.23952 NA FALSE FALSE FALSE GCST006061 0.0086 0.9447 537409 outcome TRUE reported 11557_3_SMURF1_SMUF1 SMURF1 chr18 58056398 0.009273 8.805e-46 35350 TRUE reported textfile 2 1 TRUE
8175 rs10922098 T C T C -0.1099 -0.009 0.40011 NA FALSE FALSE FALSE GCST006061 0.0075 0.2275 537409 outcome TRUE reported 3361_26_CD207_CLC4K CD207 chr1 196695521 0.008057 2.284e-42 35338 TRUE reported textfile 2 1 TRUE
8176 rs1355538 G A G A -0.0448 -0.0076 0.39847 NA FALSE FALSE FALSE GCST006061 0.0072 0.2923 537409 outcome TRUE reported 3361_26_CD207_CLC4K CD207 chr3 165787389 0.008062 2.743e-08 35338 TRUE reported textfile 2 1 TRUE
8177 rs10922098 T C T C -0.1735 -0.009 0.40011 NA FALSE FALSE FALSE GCST006061 0.0075 0.2275 537409 outcome TRUE reported 10527_22_DHX9_DHX9 DHX9 chr1 196695521 0.008125 3.666e-101 35326 TRUE reported textfile 2 1 TRUE
8178 rs11569415 A G A G -0.0586 0.0011 0.24276 NA FALSE FALSE FALSE GCST006061 0.0097 0.9129 537409 outcome TRUE reported 10527_22_DHX9_DHX9 DHX9 chr19 6716268 0.009267 2.559e-10 35360 TRUE reported textfile 2 1 TRUE
8179 rs1707652 T C T C 0.1031 0.0081 0.3102 NA FALSE FALSE FALSE GCST006061 0.0074 0.2701 537409 outcome TRUE reported 10527_22_DHX9_DHX9 DHX9 chr3 165761011 0.008603 4.322e-33 35327 TRUE reported textfile 2 1 TRUE
8180 rs10922098 T C T C -0.0875 -0.009 0.40011 NA FALSE FALSE FALSE

GCST006061 0.0075 0.2275 537409 outcome TRUE reported 6404_20_C1QL1_C1QRF
C1QL1 chr1 196695521 0.008341 9.509e-26 35338 TRUE reported
textfile 2 1 TRUE
8181 rs12766994 C T C T 0.0516 0.002 0.27487 NA FALSE FALSE FALSE
GCST006061 0.0077 0.7924 537409 outcome TRUE reported 6404_20_C1QL1_C1QRF
C1QL1 chr10 19851879 0.009176 1.876e-08 35357 TRUE reported
textfile 2 1 TRUE
8182 rs1706435 G A G A 0.05 0.0117 0.36981 NA FALSE FALSE FALSE
GCST006061 0.0072 0.1047 537409 outcome TRUE reported 6404_20_C1QL1_C1QRF
C1QL1 chr3 165743688 0.008436 3.09e-09 35339 TRUE reported
textfile 2 1 TRUE
8183 rs2808466 C T C T 0.1015 -0.0081 0.11154 NA FALSE FALSE FALSE
GCST006061 0.0104 0.4395 537409 outcome TRUE reported 6404_20_C1QL1_C1QRF
C1QL1 chr1 207100406 0.013039 6.993e-15 35369 TRUE reported
textfile 2 1 TRUE
8184 rs34497316 A G A G -0.1421 0.0291 0.14369 NA FALSE FALSE FALSE
GCST006061 0.0105 0.00569 537409 outcome TRUE reported 6404_20_C1QL1_C1QRF
C1QL1 chr17 45110419 0.011673 4.299e-34 35367 TRUE reported
textfile 2 1 TRUE
8185 rs3784535 A G A G -0.0504 -0.0027 0.29214 NA FALSE FALSE FALSE
GCST006061 0.0083 0.7452 537409 outcome TRUE reported 6404_20_C1QL1_C1QRF
C1QL1 chr15 79004668 0.008983 2.013e-08 35355 TRUE reported
textfile 2 1 TRUE
8186 rs4266889 A G A G -0.15 -0.0168 0.32236 NA FALSE FALSE FALSE
GCST006061 0.0075 0.02485 537409 outcome TRUE reported 6404_20_C1QL1_C1QRF
C1QL1 chr1 207132325 0.008715 2.133e-66 35369 TRUE reported
textfile 2 1 TRUE
8187 rs71373512 T C T C 0.1213 -0.0066 0.04683 NA FALSE FALSE FALSE
GCST006061 0.0246 0.7878 537409 outcome TRUE reported 6404_20_C1QL1_C1QRF
C1QL1 chr17 44598433 0.019569 5.7e-10 35370 TRUE reported textfile
2 1 TRUE
8188 rs7225162 T C T C -0.202 0.0034 0.48076 NA FALSE FALSE FALSE
GCST006061 0.0071 0.6298 537409 outcome TRUE reported 6404_20_C1QL1_C1QRF
C1QL1 chr17 44958892 0.008123 1.627e-136 35368 TRUE reported
textfile 2 1 TRUE
8189 rs10922098 T C T C -0.1271 -0.009 0.40011 NA FALSE FALSE FALSE
GCST006061 0.0075 0.2275 537409 outcome TRUE reported 6604_59_NDNF_CD031
NDNF chr1 196695521 0.008147 7.112e-55 35338 TRUE reported
textfile 2 1 TRUE
8190 rs1355538 G A G A -0.0581 -0.0076 0.39847 NA FALSE FALSE FALSE
GCST006061 0.0072 0.2923 537409 outcome TRUE reported 6604_59_NDNF_CD031
NDNF chr3 165787389 0.008151 1.016e-12 35338 TRUE reported
textfile 2 1 TRUE
8191 rs10922098 T C T C -0.0595 -0.009 0.40011 NA FALSE FALSE FALSE
GCST006061 0.0075 0.2275 537409 outcome TRUE reported 7982_10_MRPL32_RM32
MRPL32 chr1 196695521 0.00795 7.214e-14 35338 TRUE reported textfile
2 1 TRUE
8192 rs10922098 T C T C -0.1034 -0.009 0.40011 NA FALSE FALSE FALSE
GCST006061 0.0075 0.2275 537409 outcome TRUE reported 11425_31_RUFY1_RUFY1
RUFY1 chr1 196695521 0.008068 1.327e-37 35329 TRUE reported
textfile 2 1 TRUE
8193 rs12995461 C A C A 0.0631 0.0086 0.21643 NA FALSE FALSE FALSE
GCST006061 0.008 0.2842 537409 outcome TRUE reported 11425_31_RUFY1_RUFY1
RUFY1 chr2 27555300 0.009521 3.419e-11 35364 TRUE reported
textfile 2 1 TRUE
8194 rs1697422 C T C T -0.0466 0.0053 0.48923 NA FALSE FALSE FALSE
GCST006061 0.0072 0.4547 537409 outcome TRUE reported 11425_31_RUFY1_RUFY1
RUFY1 chr1 21495404 0.007954 4.671e-09 35365 TRUE reported
textfile 2 1 TRUE
8195 rs1706435 G A G A 0.0634 0.0117 0.36981 NA FALSE FALSE FALSE
GCST006061 0.0072 0.1047 537409 outcome TRUE reported 11425_31_RUFY1_RUFY1
RUFY1 chr3 165743688 0.008182 9.308e-15 35330 TRUE reported
textfile 2 1 TRUE
8196 rs10922098 T C T C -0.0513 -0.009 0.40011 NA FALSE FALSE FALSE
GCST006061 0.0075 0.2275 537409 outcome TRUE reported 16845_15_MPZL1_MPZL1
MPZL1 chr1 196695521 0.008088 2.261e-10 35338 TRUE reported

textfile 2 1 TRUE
8197 rs6542680 T C T C -0.0569 -0.0012 0.18362 NA FALSE FALSE FALSE
GCST006061 0.0091 0.8935 537409 outcome TRUE reported 16845_15_MPZL1_MPZL1
MPZL1 chr2 3592552 0.010251 2.843e-08 35332 TRUE reported textfile
2 1 TRUE
8198 rs77303550 T C T C 0.0807 0.0134 0.18195 NA FALSE FALSE FALSE
GCST006061 0.0091 0.1409 537409 outcome TRUE reported 16845_15_MPZL1_MPZL1
MPZL1 chr16 72045758 0.010319 5.266e-15 35375 TRUE reported
textfile 2 1 TRUE
8199 rs10922098 T C T C -0.0504 -0.009 0.40011 NA FALSE FALSE FALSE
GCST006061 0.0075 0.2275 537409 outcome TRUE reported 4831_4_SELL_sL_Selectin
SELL chr1 196695521 0.008494 2.961e-09 35338 TRUE reported
textfile 2 1 TRUE
8200 rs11125915 C A C A 0.0744 0.0139 0.32861 NA FALSE FALSE FALSE
GCST006061 0.0077 0.07187 537409 outcome TRUE reported 4831_4_SELL_sL_Selectin
SELL chr2 62308853 0.008672 9.563e-18 35352 TRUE reported
textfile 2 1 TRUE
8201 rs139621801 A G A G -0.1856 0.0195 0.01996 NA FALSE FALSE FALSE
GCST006061 0.0351 0.5798 537409 outcome TRUE reported 4831_4_SELL_sL_Selectin
SELL chr1 168808632 0.029084 1.753e-10 35361 TRUE reported
textfile 2 1 TRUE
8202 rs143501783 C T C T 0.2254 -0.0333 0.01116 NA FALSE FALSE FALSE
GCST006061 0.0443 0.4525 537409 outcome TRUE reported 4831_4_SELL_sL_Selectin
SELL chr17 7897001 0.037072 1.201e-09 35373 TRUE reported textfile
2 1 TRUE
8203 rs17350144 C T C T -0.1183 -0.0522 0.09956 NA FALSE FALSE FALSE
GCST006061 0.0123 2.252e-05 537409 outcome TRUE reported
4831_4_SELL_sL_Selectin SELL chr1 170259154 0.013874 1.504e-17 35361
TRUE reported textfile 2 1 TRUE
8204 rs1982094 T C T C -0.1188 -0.012 0.05192 NA FALSE FALSE FALSE
GCST006061 0.0164 0.464 537409 outcome TRUE reported 4831_4_SELL_sL_Selectin
SELL chr8 129612570 0.018157 6.027e-11 35372 TRUE reported
textfile 2 1 TRUE
8205 rs218265 C T C T 0.0641 0.0057 0.13571 NA FALSE FALSE FALSE
GCST006061 0.0098 0.5597 537409 outcome TRUE reported 4831_4_SELL_sL_Selectin
SELL chr4 54542832 0.011755 4.954e-08 35363 TRUE reported
textfile 2 1 TRUE
8206 rs240537 T G T G 0.0485 -0.0111 0.32826 NA FALSE FALSE FALSE
GCST006061 0.0081 0.1702 537409 outcome TRUE reported 4831_4_SELL_sL_Selectin
SELL chr11 126343446 0.008653 2.079e-08 35373 TRUE reported
textfile 2 1 TRUE
8207 rs34890930 C T C T -0.0595 -0.0029 0.2924 NA FALSE FALSE FALSE
GCST006061 0.008 0.7115 537409 outcome TRUE reported 4831_4_SELL_sL_Selectin
SELL chr3 128610380 0.008956 3.055e-11 35363 TRUE reported
textfile 2 1 TRUE
8208 rs36084354 A G A G 0.0722 -0.0423 0.14122 NA FALSE FALSE FALSE
GCST006061 0.0141 0.002636 537409 outcome TRUE reported
4831_4_SELL_sL_Selectin SELL chr19 1079960 0.011687 6.496e-10 35324 TRUE
reported textfile 2 1 TRUE
8209 rs4649425 A G A G -0.0583 -0.002 0.192 NA FALSE FALSE FALSE
GCST006061 0.0085 0.8155 537409 outcome TRUE reported 4831_4_SELL_sL_Selectin
SELL chr1 233019717 0.010631 4.154e-08 35363 TRUE reported
textfile 2 1 TRUE
8210 rs6136 G T G T -0.1729 -0.0141 0.12391 NA FALSE FALSE FALSE GCST006061
0.0122 0.2504 537409 outcome TRUE reported 4831_4_SELL_sL_Selectin SELL
chr1 169594713 0.012651 1.61e-42 35360 TRUE reported textfile 2
1 TRUE
8211 rs62246503 A G A G 0.067 0.0039 0.1395 NA FALSE FALSE FALSE
GCST006061 0.0113 0.7308 537409 outcome TRUE reported 4831_4_SELL_sL_Selectin
SELL chr3 22222601 0.011718 1.079e-08 35371 TRUE reported
textfile 2 1 TRUE
8212 rs6796 C T C T 0.0624 0.0015 0.23682 NA FALSE FALSE FALSE GCST006061
0.0081 0.857 537409 outcome TRUE reported 4831_4_SELL_sL_Selectin SELL
chr7 6462736 0.009559 6.669e-11 35364 TRUE reported textfile 2 1
TRUE
8213 rs75776028 G A G A -0.4017 0.0041 0.30423 NA FALSE FALSE FALSE

GCST006061 0.0085 0.6268 537409 outcome TRUE reported 4831_4_SELL_sL_Selectin
SELL chr1 169684654 0.006718 1e-200 35362 TRUE reported textfile
2 1 TRUE
8214 rs76428106 C T C T 0.3753 -0.0446 0.01359 NA FALSE FALSE FALSE
GCST006061 0.0438 0.3085 537409 outcome TRUE reported 4831_4_SELL_sL_Selectin
SELL chr13 28029870 0.03536 2.57e-26 35363 TRUE reported textfile
2 1 TRUE
8215 rs9898547 T G T G -0.0786 0.013 0.44771 NA FALSE FALSE FALSE
GCST006061 0.0071 0.06951 537409 outcome TRUE reported 4831_4_SELL_sL_Selectin
SELL chr17 39979773 0.008209 1.023e-21 35379 TRUE reported
textfile 2 1 TRUE
8216 rs10922098 T C T C -0.1194 -0.009 0.40011 NA FALSE FALSE FALSE
GCST006061 0.0075 0.2275 537409 outcome TRUE reported 16858_384_RCN3_RCN3
RCN3 chr1 196695521 0.008136 9.248e-49 35338 TRUE reported
textfile 2 1 TRUE
8217 rs1707652 T C T C 0.0751 0.0081 0.3102 NA FALSE FALSE FALSE
GCST006061 0.0074 0.2701 537409 outcome TRUE reported 16858_384_RCN3_RCN3
RCN3 chr3 165761011 0.008614 2.819e-18 35339 TRUE reported
textfile 2 1 TRUE
8218 rs223046 C A C A -0.132 0.0087 0.2363 NA FALSE FALSE FALSE
GCST006061 0.0078 0.2625 537409 outcome TRUE reported 16858_384_RCN3_RCN3
RCN3 chr11 32091022 0.009288 7.689e-46 35366 TRUE reported
textfile 2 1 TRUE
8219 rs3842763 T G T G 0.0706 0.0131 0.192 NA FALSE FALSE FALSE
GCST006061 0.0084 0.1208 537409 outcome TRUE reported 16858_384_RCN3_RCN3
RCN3 chr11 2157974 0.010206 4.594e-12 35355 TRUE reported textfile
2 1 TRUE
8220 rs10922098 T C T C -0.1508 -0.009 0.40011 NA FALSE FALSE FALSE
GCST006061 0.0075 0.2275 537409 outcome TRUE reported 11168_3_MEGF10_MEG10
MEGF10 chr1 196695521 0.007967 6.845e-80 35329 TRUE reported
textfile 2 1 TRUE
8221 rs1355538 G A G A -0.0646 -0.0076 0.39847 NA FALSE FALSE FALSE
GCST006061 0.0072 0.2923 537409 outcome TRUE reported 11168_3_MEGF10_MEG10
MEGF10 chr3 165787389 0.007986 6.017e-16 35329 TRUE reported
textfile 2 1 TRUE
8222 rs10922098 T C T C -0.0851 -0.009 0.40011 NA FALSE FALSE FALSE
GCST006061 0.0075 0.2275 537409 outcome TRUE reported 6955_68_SNX1_SNX1
SNX1 chr1 196695521 0.008076 5.77e-26 35338 TRUE reported
textfile 2 1 TRUE
8223 rs1354034 C T C T 0.0618 0.0088 0.38119 NA FALSE FALSE FALSE
GCST006061 0.0073 0.2248 537409 outcome TRUE reported 6955_68_SNX1_SNX1
SNX1 chr3 56815721 0.008139 3.113e-14 35356 TRUE reported
textfile 2 1 TRUE
8224 rs1619994 G A G A 0.0493 0.0168 0.2807 NA FALSE FALSE FALSE
GCST006061 0.0078 0.03057 537409 outcome TRUE reported 6955_68_SNX1_SNX1
SNX1 chr3 165744047 0.008819 2.264e-08 35339 TRUE reported
textfile 2 1 TRUE
8225 rs3748136 A G A G 0.1215 -0.0088 0.17278 NA FALSE FALSE FALSE
GCST006061 0.0093 0.3444 537409 outcome TRUE reported 6955_68_SNX1_SNX1
SNX1 chr8 9172650 0.010449 2.98e-31 35384 TRUE reported textfile
2 1 TRUE
8226 rs77404765 C T C T 0.4359 -4.00E-04 0.01934 NA FALSE FALSE FALSE
GCST006061 0.0307 0.9897 537409 outcome TRUE reported 6955_68_SNX1_SNX1
SNX1 chr15 64300071 0.029517 2.362e-49 35362 TRUE reported
textfile 2 1 TRUE
8227 rs10922098 T C T C -0.089 -0.009 0.40011 NA FALSE FALSE FALSE
GCST006061 0.0075 0.2275 537409 outcome TRUE reported 17702_53_UGT1A1_UGT_1A1
UGT1A1 chr1 196695521 0.008091 3.837e-28 35338 TRUE reported
textfile 2 1 TRUE
8228 rs13001875 C T C T -0.0711 0.0093 0.24428 NA FALSE FALSE FALSE
GCST006061 0.0085 0.2787 537409 outcome TRUE reported 17702_53_UGT1A1_UGT_1A1
UGT1A1 chr2 233361711 0.00924 1.418e-14 35374 TRUE reported textfile
2 1 TRUE
8229 rs1355538 G A G A -0.0679 -0.0076 0.39847 NA FALSE FALSE FALSE
GCST006061 0.0072 0.2923 537409 outcome TRUE reported 17702_53_UGT1A1_UGT_1A1
UGT1A1 chr3 165787389 0.008086 4.555e-17 35338 TRUE reported

textfile 2 1 TRUE
8230 rs6742078 T G T G -0.2225 0.002 0.31953 NA FALSE FALSE FALSE
GCST006061 0.0077 0.7998 537409 outcome TRUE reported 17702_53_UGT1A1_UGT_1A1
UGT1A1 chr2 233763993 0.008466 3.15e-152 35372 TRUE reported
textfile 2 1 TRUE
8231 rs704 A G A G -0.1207 -0.0155 0.49189 NA FALSE FALSE FALSE GCST006061
0.0072 0.03144 537409 outcome TRUE reported 17702_53_UGT1A1_UGT_1A1 UGT1A1
chr17 28367840 0.007927 2.372e-52 35360 TRUE reported textfile 2
1 TRUE
8232 rs10922098 T C T C -0.0709 -0.009 0.40011 NA FALSE FALSE FALSE
GCST006061 0.0075 0.2275 537409 outcome TRUE reported 16320_139_CD320_CD320
CD320 chr1 196695521 0.007916 3.351e-19 35338 TRUE reported
textfile 2 1 TRUE
8233 rs10922098 T C T C -0.0993 -0.009 0.40011 NA FALSE FALSE FALSE
GCST006061 0.0075 0.2275 537409 outcome TRUE reported 18277_28_TRIAP1_TRIA1
TRIAP1 chr1 196695521 0.008099 1.48e-34 35338 TRUE reported
textfile 2 1 TRUE
8234 rs1355538 G A G A -0.0494 -0.0076 0.39847 NA FALSE FALSE FALSE
GCST006061 0.0072 0.2923 537409 outcome TRUE reported 18277_28_TRIAP1_TRIA1
TRIAP1 chr3 165787389 0.008095 1.043e-09 35338 TRUE reported
textfile 2 1 TRUE
8235 rs2057681 G A G A 0.1294 -0.0136 0.28541 NA FALSE FALSE FALSE
GCST006061 0.0078 0.07965 537409 outcome TRUE reported 18277_28_TRIAP1_TRIA1
TRIAP1 chr7 95308945 0.008816 8.983e-49 35349 TRUE reported
textfile 2 1 TRUE
8236 rs10922098 T C T C -0.1845 -0.009 0.40011 NA FALSE FALSE FALSE
GCST006061 0.0075 0.2275 537409 outcome TRUE reported 7805_52_GOSR1_GOS_28
GOSR1 chr1 196695521 0.008129 4.725e-114 35338 TRUE reported
textfile 2 1 TRUE
8237 rs144704969 T C T C 0.109 -0.0238 0.04536 NA FALSE FALSE FALSE
GCST006061 0.0154 0.1235 537409 outcome TRUE reported 7805_52_GOSR1_GOS_28
GOSR1 chr1 196158214 0.019053 1.059e-08 35337 TRUE reported
textfile 2 1 TRUE
8238 rs1555405 A G A G -0.0485 -0.0047 0.30381 NA FALSE FALSE FALSE
GCST006061 0.0081 0.5592 537409 outcome TRUE reported 7805_52_GOSR1_GOS_28
GOSR1 chr14 100710432 0.008736 2.829e-08 35380 TRUE reported
textfile 2 1 TRUE
8239 rs1707652 T C T C 0.0994 0.0081 0.3102 NA FALSE FALSE FALSE
GCST006061 0.0074 0.2701 537409 outcome TRUE reported 7805_52_GOSR1_GOS_28
GOSR1 chr3 165761011 0.008614 8.323e-31 35339 TRUE reported
textfile 2 1 TRUE
8240 rs72952553 A G A G -0.0473 -0.0042 0.31481 NA FALSE FALSE FALSE
GCST006061 0.0085 0.6159 537409 outcome TRUE reported 7805_52_GOSR1_GOS_28
GOSR1 chr2 216616793 0.008647 4.492e-08 35359 TRUE reported
textfile 2 1 TRUE
8241 rs10922098 T C T C -0.1991 -0.009 0.40011 NA FALSE FALSE FALSE
GCST006061 0.0075 0.2275 537409 outcome TRUE reported 6395_58_GPHA2_GPHA2
GPHA2 chr1 196695521 0.008102 2.414e-133 35338 TRUE reported
textfile 2 1 TRUE
8242 rs1707652 T C T C 0.0825 0.0081 0.3102 NA FALSE FALSE FALSE
GCST006061 0.0074 0.2701 537409 outcome TRUE reported 6395_58_GPHA2_GPHA2
GPHA2 chr3 165761011 0.008595 8.093e-22 35339 TRUE reported
textfile 2 1 TRUE
8243 rs10922098 T C T C -0.1185 -0.009 0.40011 NA FALSE FALSE FALSE
GCST006061 0.0075 0.2275 537409 outcome TRUE reported 14135_3_RXFP1_RXFP1
RXFP1 chr1 196695521 0.00812 3.077e-48 35338 TRUE reported textfile
2 1 TRUE
8244 rs1706435 G A G A 0.0591 0.0117 0.36981 NA FALSE FALSE FALSE
GCST006061 0.0072 0.1047 537409 outcome TRUE reported 14135_3_RXFP1_RXFP1
RXFP1 chr3 165743688 0.00824 7.374e-13 35339 TRUE reported textfile
2 1 TRUE
8245 rs6542680 T C T C -0.0766 -0.0012 0.18362 NA FALSE FALSE FALSE
GCST006061 0.0091 0.8935 537409 outcome TRUE reported 14135_3_RXFP1_RXFP1
RXFP1 chr2 3592552 0.010304 1.056e-13 35332 TRUE reported textfile
2 1 TRUE
8246 rs10922098 T C T C -0.1123 -0.009 0.40011 NA FALSE FALSE FALSE

GCST006061 0.0075 0.2275 537409 outcome TRUE reported 12845_18_SNX17_SNX17
SNX17 chr1 196695521 0.00823 2.159e-42 35329 TRUE reported textfile
2 1 TRUE
8247 rs1354034 C T C T 0.0463 0.0088 0.38119 NA FALSE FALSE FALSE
GCST006061 0.0073 0.2248 537409 outcome TRUE reported 12845_18_SNX17_SNX17
SNX17 chr3 56815721 0.008306 2.484e-08 35347 TRUE reported
textfile 2 1 TRUE
8248 rs1706435 G A G A 0.0575 0.0117 0.36981 NA FALSE FALSE FALSE
GCST006061 0.0072 0.1047 537409 outcome TRUE reported 12845_18_SNX17_SNX17
SNX17 chr3 165743688 0.008345 5.569e-12 35330 TRUE reported
textfile 2 1 TRUE
8249 rs10922098 T C T C -0.1036 -0.009 0.40011 NA FALSE FALSE FALSE
GCST006061 0.0075 0.2275 537409 outcome TRUE reported 9350_3_FSTL4_FSTL4
FSTL4 chr1 196695521 0.00814 4.139e-37 35338 TRUE reported textfile
2 1 TRUE
8250 rs1355538 G A G A -0.0462 -0.0076 0.39847 NA FALSE FALSE FALSE
GCST006061 0.0072 0.2923 537409 outcome TRUE reported 9350_3_FSTL4_FSTL4
FSTL4 chr3 165787389 0.008145 1.411e-08 35338 TRUE reported
textfile 2 1 TRUE
8251 rs17642974 C T C T 0.0544 -0.0032 0.29899 NA FALSE FALSE FALSE
GCST006061 0.0081 0.6957 537409 outcome TRUE reported 9350_3_FSTL4_FSTL4
FSTL4 chr5 133606103 0.008708 4.19e-10 35350 TRUE reported
textfile 2 1 TRUE
8252 rs10922098 T C T C -0.1278 -0.009 0.40011 NA FALSE FALSE FALSE
GCST006061 0.0075 0.2275 537409 outcome TRUE reported 9363_11_CT55_CX048
CT55 chr1 196695521 0.008063 1.42e-56 35338 TRUE reported
textfile 2 1 TRUE
8253 rs10922098 T C T C -0.0612 -0.009 0.40011 NA FALSE FALSE FALSE
GCST006061 0.0075 0.2275 537409 outcome TRUE reported 10908_2_GALNT13_GLT13
GALNT13 chr1 196695521 0.008245 1.146e-13 35327 TRUE reported
textfile 2 1 TRUE
8254 rs12975366 C T C T -0.061 -0.0089 0.4155 NA FALSE FALSE FALSE
GCST006061 0.0081 0.2688 537409 outcome TRUE reported 10908_2_GALNT13_GLT13
GALNT13 chr19 54255498 0.008196 9.857e-14 35359 TRUE reported
textfile 2 1 TRUE
8255 rs16834610 T C T C 0.1275 -0.0037 0.04952 NA FALSE FALSE FALSE
GCST006061 0.0168 0.8277 537409 outcome TRUE reported 10908_2_GALNT13_GLT13
GALNT13 chr2 153873982 0.018824 1.258e-11 35338 TRUE reported
textfile 2 1 TRUE
8256 rs10922098 T C T C -0.1049 -0.009 0.40011 NA FALSE FALSE FALSE
GCST006061 0.0075 0.2275 537409 outcome TRUE reported 19196_73_HOPX_HOP
HOPX chr1 196695521 0.008017 3.995e-39 35338 TRUE reported
textfile 2 1 TRUE
8257 rs1697422 C T C T -0.0447 0.0053 0.48923 NA FALSE FALSE FALSE
GCST006061 0.0072 0.4547 537409 outcome TRUE reported 19196_73_HOPX_HOP
HOPX chr1 21495404 0.007902 1.544e-08 35374 TRUE reported
textfile 2 1 TRUE
8258 rs56270003 T C T C 0.09 0.0134 0.26081 NA FALSE FALSE FALSE
GCST006061 0.0079 0.09085 537409 outcome TRUE reported 19196_73_HOPX_HOP
HOPX chr4 7804267 0.008936 7.357e-24 35382 TRUE reported textfile
2 1 TRUE
8259 rs10922098 T C T C -0.1966 -0.009 0.40011 NA FALSE FALSE FALSE
GCST006061 0.0075 0.2275 537409 outcome TRUE reported 3391_10_PIK3CG_PK3CG
PIK3CG chr1 196695521 0.008301 5.229e-124 35338 TRUE reported
textfile 2 1 TRUE
8260 rs11569415 A G A G -0.0592 0.0011 0.24276 NA FALSE FALSE FALSE
GCST006061 0.0097 0.9129 537409 outcome TRUE reported 3391_10_PIK3CG_PK3CG
PIK3CG chr19 6716268 0.009474 4.132e-10 35372 TRUE reported textfile
2 1 TRUE
8261 rs146453555 C T C T -0.3155 -0.0872 0.01325 NA FALSE FALSE FALSE
GCST006061 0.0596 0.1434 537409 outcome TRUE reported 3391_10_PIK3CG_PK3CG
PIK3CG chr17 8828612 0.034871 1.46e-19 35373 TRUE reported textfile
2 1 TRUE
8262 rs1707652 T C T C 0.0954 0.0081 0.3102 NA FALSE FALSE FALSE
GCST006061 0.0074 0.2701 537409 outcome TRUE reported 3391_10_PIK3CG_PK3CG
PIK3CG chr3 165761011 0.008791 1.954e-27 35339 TRUE reported

textfile 2 1 TRUE
8263 rs342294 C T C T -0.0484 -0.0016 0.45409 NA FALSE FALSE FALSE
GCST006061 0.0073 0.8241 537409 outcome TRUE reported 3391_10_PIK3CG_PK3CG
PIK3CG chr7 106732176 0.00817 3.134e-09 35354 TRUE reported textfile
2 1 TRUE
8264 rs4541868 A C A C -0.0715 0.0211 0.21915 NA FALSE FALSE FALSE
GCST006061 0.0082 0.01029 537409 outcome TRUE reported 3391_10_PIK3CG_PK3CG
PIK3CG chr8 105578477 0.009883 4.66e-13 35364 TRUE reported
textfile 2 1 TRUE
8265 rs72748418 A G A G 0.0879 0.0089 0.07164 NA FALSE FALSE FALSE
GCST006061 0.0129 0.4914 537409 outcome TRUE reported 3391_10_PIK3CG_PK3CG
PIK3CG chr1 196122755 0.015746 2.371e-08 35337 TRUE reported
textfile 2 1 TRUE
8266 rs10922098 T C T C -0.1263 -0.009 0.40011 NA FALSE FALSE FALSE
GCST006061 0.0075 0.2275 537409 outcome TRUE reported 11560_76_NFATC4_NFAC4
NFATC4 chr1 196695521 0.00806 2.414e-55 35329 TRUE reported textfile
2 1 TRUE
8267 rs10922098 T C T C -0.0683 -0.009 0.40011 NA FALSE FALSE FALSE
GCST006061 0.0075 0.2275 537409 outcome TRUE reported
3298_52_CNTN4_Contactin_4 CNTN4 chr1 196695521 0.00871 4.45e-15 35338
TRUE reported textfile 2 1 TRUE
8268 rs115370013 T C T C 0.2332 0.0261 0.06581 NA FALSE FALSE FALSE
GCST006061 0.0168 0.1191 537409 outcome TRUE reported
3298_52_CNTN4_Contactin_4 CNTN4 chr3 2159855 0.017356 3.714e-41 35261
TRUE reported textfile 2 1 TRUE
8269 rs13071423 A C A C 0.2532 -0.0032 0.18783 NA FALSE FALSE FALSE
GCST006061 0.0099 0.7486 537409 outcome TRUE reported
3298_52_CNTN4_Contactin_4 CNTN4 chr3 2098694 0.011067 7.379e-116 35250
TRUE reported textfile 2 1 TRUE
8270 rs1499126 G A G A -0.4912 -0.0023 0.01463 NA FALSE FALSE FALSE
GCST006061 0.0234 0.922 537409 outcome TRUE reported
3298_52_CNTN4_Contactin_4 CNTN4 chr3 2993909 0.035303 5.234e-44 35372
TRUE reported textfile 2 1 TRUE
8271 rs1850507 G T G T 0.0686 -0.0133 0.18809 NA FALSE FALSE FALSE
GCST006061 0.0087 0.1288 537409 outcome TRUE reported
3298_52_CNTN4_Contactin_4 CNTN4 chr4 16027243 0.010921 3.356e-10 35359
TRUE reported textfile 2 1 TRUE
8272 rs1949342 T G T G -0.0816 0.026 0.09203 NA FALSE FALSE FALSE
GCST006061 0.0149 0.08032 537409 outcome TRUE reported
3298_52_CNTN4_Contactin_4 CNTN4 chr3 2696709 0.014874 4.113e-08 35367
TRUE reported textfile 2 1 TRUE
8273 rs35887873 C T C T 0.1187 -0.0114 0.26311 NA FALSE FALSE FALSE
GCST006061 0.0091 0.2089 537409 outcome TRUE reported
3298_52_CNTN4_Contactin_4 CNTN4 chr11 126349501 0.009607 4.568e-35 35374
TRUE reported textfile 2 1 TRUE
8274 rs55714927 T C T C 0.074 1e-04 0.21166 NA FALSE FALSE FALSE
GCST006061 0.0098 0.9926 537409 outcome TRUE reported
3298_52_CNTN4_Contactin_4 CNTN4 chr17 7176997 0.010494 1.768e-12 35370
TRUE reported textfile 2 1 TRUE
8275 rs975334 A G A G 0.0738 0.0129 0.34311 NA FALSE FALSE FALSE
GCST006061 0.0075 0.08626 537409 outcome TRUE reported
3298_52_CNTN4_Contactin_4 CNTN4 chr3 2804632 0.009116 5.692e-16 35373
TRUE reported textfile 2 1 TRUE
8276 rs9878112 G T G T -0.1063 0.0047 0.15636 NA FALSE FALSE FALSE
GCST006061 0.01 0.6349 537409 outcome TRUE reported
3298_52_CNTN4_Contactin_4 CNTN4 chr3 2032831 0.012034 1.018e-18 35249
TRUE reported textfile 2 1 TRUE
8277 rs10922098 T C T C -0.1223 -0.009 0.40011 NA FALSE FALSE FALSE
GCST006061 0.0075 0.2275 537409 outcome TRUE reported 13439_6_IQCF3_IQCF3
IQCF3 chr1 196695521 0.00812 2.869e-51 35332 TRUE reported textfile
2 1 TRUE
8278 rs1355538 G A G A -0.0529 -0.0076 0.39847 NA FALSE FALSE FALSE
GCST006061 0.0072 0.2923 537409 outcome TRUE reported 13439_6_IQCF3_IQCF3
IQCF3 chr3 165787389 0.008123 7.406e-11 35332 TRUE reported
textfile 2 1 TRUE
8279 rs10922098 T C T C -0.0567 -0.009 0.40011 NA FALSE FALSE FALSE

GCST006061 0.0075 0.2275 537409 outcome TRUE reported 14134_49_AMIGO2_AMGO2
AMIGO2 chr1 196695521 0.008365 1.214e-11 35338 TRUE reported
textfile 2 1 TRUE
8280 rs12493830 C T C T -0.0585 -0.0049 0.42563 NA FALSE FALSE FALSE
GCST006061 0.0072 0.4985 537409 outcome TRUE reported 14134_49_AMIGO2_AMGO2
AMIGO2 chr3 98687950 0.008282 1.621e-12 35330 TRUE reported
textfile 2 1 TRUE
8281 rs1354034 C T C T -0.1488 0.0088 0.38119 NA FALSE FALSE FALSE
GCST006061 0.0073 0.2248 537409 outcome TRUE reported 14134_49_AMIGO2_AMGO2
AMIGO2 chr3 56815721 0.00842 6.98e-70 35356 TRUE reported textfile
2 1 TRUE
8282 rs1993484 C T C T 0.0568 0.0174 0.21898 NA FALSE FALSE FALSE
GCST006061 0.0086 0.04311 537409 outcome TRUE reported 14134_49_AMIGO2_AMGO2
AMIGO2 chr10 80462942 0.009922 1.036e-08 35370 TRUE reported
textfile 2 1 TRUE
8283 rs2127868 A G A G 0.0599 -0.0083 0.17112 NA FALSE FALSE FALSE
GCST006061 0.0085 0.3283 537409 outcome TRUE reported 14134_49_AMIGO2_AMGO2
AMIGO2 chr14 65327609 0.010841 3.292e-08 35359 TRUE reported
textfile 2 1 TRUE
8284 rs215397 A C A C 0.051 0.005 0.48269 NA FALSE FALSE FALSE
GCST006061 0.0071 0.4868 537409 outcome TRUE reported 14134_49_AMIGO2_AMGO2
AMIGO2 chr12 47372913 0.008241 6.081e-10 35367 TRUE reported
textfile 2 1 TRUE
8285 rs55714927 T C T C 0.0746 1e-04 0.21166 NA FALSE FALSE FALSE
GCST006061 0.0098 0.9926 537409 outcome TRUE reported 14134_49_AMIGO2_AMGO2
AMIGO2 chr17 7176997 0.010092 1.444e-13 35370 TRUE reported textfile
2 1 TRUE
8286 rs635634 C T C T 0.1361 0.0285 0.12995 NA FALSE FALSE FALSE
GCST006061 0.0089 0.001351 537409 outcome TRUE reported
14134_49_AMIGO2_AMGO2 AMIGO2 chr9 133279427 0.012182 5.6e-29 35371 TRUE
reported textfile 2 1 TRUE
8287 rs79609374 G A G A -0.1032 0.0022 0.2848 NA FALSE FALSE FALSE
GCST006061 0.0078 0.7739 537409 outcome TRUE reported 14134_49_AMIGO2_AMGO2
AMIGO2 chr12 47079943 0.009145 1.553e-29 35365 TRUE reported
textfile 2 1 TRUE
8288 rs9494145 C T C T 0.0577 -0.0073 0.24334 NA FALSE FALSE FALSE
GCST006061 0.0083 0.3799 537409 outcome TRUE reported 14134_49_AMIGO2_AMGO2
AMIGO2 chr6 135111414 0.009555 1.553e-09 35372 TRUE reported
textfile 2 1 TRUE
8289 rs10922098 T C T C -0.1919 -0.009 0.40011 NA FALSE FALSE FALSE
GCST006061 0.0075 0.2275 537409 outcome TRUE reported 10748_216_PCDHB2_PCDB2
PCDHB2 chr1 196695521 0.007991 1.952e-127 35326 TRUE reported
textfile 2 1 TRUE
8290 rs11064498 G A G A -0.0815 -8.00E-04 0.1306 NA FALSE FALSE FALSE
GCST006061 0.0102 0.939 537409 outcome TRUE reported 10748_216_PCDHB2_PCDB2
PCDHB2 chr12 7064203 0.011848 6.041e-12 35378 TRUE reported textfile
2 1 TRUE
8291 rs56317765 T C T C 0.0617 -0.0069 0.15401 NA FALSE FALSE FALSE
GCST006061 0.0104 0.5072 537409 outcome TRUE reported 10748_216_PCDHB2_PCDB2
PCDHB2 chr20 37489853 0.0109 1.511e-08 35357 TRUE reported textfile
2 1 TRUE
8292 rs10922098 T C T C -0.0535 -0.009 0.40011 NA FALSE FALSE FALSE
GCST006061 0.0075 0.2275 537409 outcome TRUE reported 18932_84_NMNAT1_NMNA1
NMNAT1 chr1 196695521 0.00796 1.807e-11 35338 TRUE reported textfile
2 1 TRUE
8293 rs10922098 T C T C -0.1527 -0.009 0.40011 NA FALSE FALSE FALSE
GCST006061 0.0075 0.2275 537409 outcome TRUE reported 10023_32_VDR_VDR VDR
chr1 196695521 0.008104 3.381e-79 35326 TRUE reported textfile 2
1 TRUE
8294 rs13469 T C T C -0.0439 -0.0177 0.45244 NA FALSE FALSE FALSE GCST006061
0.0071 0.01333 537409 outcome TRUE reported 10023_32_VDR_VDR VDR chr17
28349109 0.007996 4.008e-08 35348 TRUE reported textfile 2 1 TRUE
8295 rs2686395 C T C T -0.0904 -0.0077 0.3101 NA FALSE FALSE FALSE
GCST006061 0.0074 0.2965 537409 outcome TRUE reported 10023_32_VDR_VDR VDR
chr3 165767231 0.00858 5.853e-26 35327 TRUE reported textfile 2 1
TRUE

8296 rs10922098 T C T C -0.1291 -0.009 0.40011 NA FALSE FALSE FALSE GCST006061 0.0075 0.2275 537409 outcome TRUE reported 10848_137_BTNL3_BTNL3 BTNL3 chr1 196695521 0.008111 4.803e-57 35326 TRUE reported textfile 2 1 TRUE

8297 rs35209896 A C A C 0.0908 0.0267 0.09263 NA FALSE FALSE FALSE GCST006061 0.0355 0.4521 537409 outcome TRUE reported 10848_137_BTNL3_BTNL3 BTNL3 chr6 32519009 0.013605 2.489e-11 35427 TRUE reported textfile 2 1 TRUE

8298 rs6445035 A G A G 0.1067 0.0154 0.1904 NA FALSE FALSE FALSE GCST006061 0.0089 0.08256 537409 outcome TRUE reported 10848_137_BTNL3_BTNL3 BTNL3 chr3 165762312 0.010105 4.599e-26 35327 TRUE reported textfile 2 1 TRUE

8299 rs10922098 T C T C -0.1113 -0.009 0.40011 NA FALSE FALSE FALSE GCST006061 0.0075 0.2275 537409 outcome TRUE reported 8587_21_SPINK14_ISK52 SPINK14 chr1 196695521 0.00825 1.78e-41 35338 TRUE reported textfile 2 1 TRUE

8300 rs1707652 T C T C 0.0751 0.0081 0.3102 NA FALSE FALSE FALSE GCST006061 0.0074 0.2701 537409 outcome TRUE reported 8587_21_SPINK14_ISK52 SPINK14 chr3 165761011 0.00872 7.137e-18 35339 TRUE reported textfile 2 1 TRUE

8301 rs704 A G A G 0.0461 -0.0155 0.49189 NA FALSE FALSE FALSE GCST006061 0.0072 0.03144 537409 outcome TRUE reported 8587_21_SPINK14_ISK52 SPINK14 chr17 28367840 0.008106 1.29e-08 35360 TRUE reported textfile 2 1 TRUE

8302 rs10922098 T C T C -0.0614 -0.009 0.40011 NA FALSE FALSE FALSE GCST006061 0.0075 0.2275 537409 outcome TRUE reported 3835_11_TLR2_TLR2 TLR2 chr1 196695521 0.008077 2.925e-14 35338 TRUE reported textfile 2 1 TRUE

8303 rs112595928 A G A G -0.2091 0.1287 0.01366 NA FALSE FALSE FALSE GCST006061 0.0556 0.02069 537409 outcome TRUE reported 3835_11_TLR2_TLR2 TLR2 chr4 153914154 0.03293 2.155e-10 35381 TRUE reported textfile 2 1 TRUE

8304 rs10922098 T C T C -0.0879 -0.009 0.40011 NA FALSE FALSE FALSE GCST006061 0.0075 0.2275 537409 outcome TRUE reported 9322_15_RCN1_RCN1 RCN1 chr1 196695521 0.007998 4.27e-28 35338 TRUE reported textfile 2 1 TRUE

8305 rs3842763 T G T G 0.0626 0.0131 0.192 NA FALSE FALSE FALSE GCST006061 0.0084 0.1208 537409 outcome TRUE reported 9322_15_RCN1_RCN1 RCN1 chr11 2157974 0.010014 4.065e-10 35355 TRUE reported textfile 2 1 TRUE

8306 rs531576 A G A G -0.1735 -0.0085 0.13047 NA FALSE FALSE FALSE GCST006061 0.01 0.395 537409 outcome TRUE reported 9322_15_RCN1_RCN1 RCN1 chr11 32089877 0.011538 4.211e-51 35366 TRUE reported textfile 2 1 TRUE

8307 rs10922098 T C T C -0.0997 -0.009 0.40011 NA FALSE FALSE FALSE GCST006061 0.0075 0.2275 537409 outcome TRUE reported 10955_4_CLEC10A_CLC10 CLEC10A chr1 196695521 0.008099 7.99e-35 35329 TRUE reported textfile 2 1 TRUE

8308 rs1706435 G A G A 0.0602 0.0117 0.36981 NA FALSE FALSE FALSE GCST006061 0.0072 0.1047 537409 outcome TRUE reported 10955_4_CLEC10A_CLC10 CLEC10A chr3 165743688 0.008211 2.272e-13 35330 TRUE reported textfile 2 1 TRUE

8309 rs9469017 A G A G -0.087 0.0066 0.07464 NA FALSE FALSE FALSE GCST006061 0.015 0.659 537409 outcome TRUE reported 10955_4_CLEC10A_CLC10 CLEC10A chr6 31496662 0.015136 9.028e-09 35431 TRUE reported textfile 2 1 TRUE

8310 rs10922098 T C T C -0.0922 -0.009 0.40011 NA FALSE FALSE FALSE GCST006061 0.0075 0.2275 537409 outcome TRUE reported 9956_7_ADAM15_ADA15 ADAM15 chr1 196695521 0.008068 3.035e-30 35338 TRUE reported textfile 2 1 TRUE

8311 rs1706435 G A G A 0.0514 0.0117 0.36981 NA FALSE FALSE FALSE GCST006061 0.0072 0.1047 537409 outcome TRUE reported 9956_7_ADAM15_ADA15 ADAM15 chr3 165743688 0.008177 3.267e-10 35339 TRUE reported textfile 2 1 TRUE

8312 rs10922098 T C T C -0.136 -0.009 0.40011 NA FALSE FALSE FALSE GCST006061 0.0075 0.2275 537409 outcome TRUE reported 13711_10_CLTC_CLH1

CLTC chr1 196695521 0.008021 1.76e-64 35338 TRUE reported
textfile 2 1 TRUE
8313 rs1706435 G A G A 0.0759 0.0117 0.36981 NA FALSE FALSE FALSE
GCST006061 0.0072 0.1047 537409 outcome TRUE reported 13711_10_CLTC_CLH1
CLTC chr3 165743688 0.008145 1.181e-20 35339 TRUE reported
textfile 2 1 TRUE
8314 rs10922098 T C T C -0.0645 -0.009 0.40011 NA FALSE FALSE FALSE
GCST006061 0.0075 0.2275 537409 outcome TRUE reported 2732_58_NANOG_NANOG
NANOG chr1 196695521 0.007937 4.41e-16 35338 TRUE reported
textfile 2 1 TRUE
8315 rs11651658 C T C T -0.0901 -0.0419 0.06769 NA FALSE FALSE FALSE
GCST006061 0.0135 0.001949 537409 outcome TRUE reported 2732_58_NANOG_NANOG
NANOG chr17 66202522 0.015664 8.813e-09 35370 TRUE reported
textfile 2 1 TRUE
8316 rs10922098 T C T C -0.1927 -0.009 0.40011 NA FALSE FALSE FALSE
GCST006061 0.0075 0.2275 537409 outcome TRUE reported 16749_79_GDF10_BMP_3b
GDF10 chr1 196695521 0.008098 3.599e-125 35338 TRUE reported
textfile 2 1 TRUE
8317 rs11569415 A G A G -0.0549 0.0011 0.24276 NA FALSE FALSE FALSE
GCST006061 0.0097 0.9129 537409 outcome TRUE reported 16749_79_GDF10_BMP_3b
GDF10 chr19 6716268 0.009246 2.885e-09 35372 TRUE reported textfile
2 1 TRUE
8318 rs10922098 T C T C -0.2058 -0.009 0.40011 NA FALSE FALSE FALSE
GCST006061 0.0075 0.2275 537409 outcome TRUE reported 11459_81_ELL_ELL ELL
chr1 196695521 0.008111 5.05e-142 35329 TRUE reported textfile 2
1 TRUE
8319 rs144704969 T C T C 0.104 -0.0238 0.04536 NA FALSE FALSE FALSE
GCST006061 0.0154 0.1235 537409 outcome TRUE reported 11459_81_ELL_ELL ELL
chr1 196158214 0.019039 4.698e-08 35328 TRUE reported textfile 2
1 TRUE
8320 rs1707652 T C T C 0.1119 0.0081 0.3102 NA FALSE FALSE FALSE
GCST006061 0.0074 0.2701 537409 outcome TRUE reported 11459_81_ELL_ELL ELL
chr3 165761011 0.008601 1.073e-38 35330 TRUE reported textfile 2
1 TRUE
8321 rs704 A G A G 0.0464 -0.0155 0.49189 NA FALSE FALSE FALSE GCST006061
0.0072 0.03144 537409 outcome TRUE reported 11459_81_ELL_ELL ELL chr17
28367840 0.007997 6.536e-09 35351 TRUE reported textfile 2 1 TRUE
8322 rs10922098 T C T C -0.0859 -0.009 0.40011 NA FALSE FALSE FALSE
GCST006061 0.0075 0.2275 537409 outcome TRUE reported 8372_29_CDHR1_CDHR1
CDHR1 chr1 196695521 0.008057 1.548e-26 35338 TRUE reported
textfile 2 1 TRUE
8323 rs10922098 T C T C -0.1064 -0.009 0.40011 NA FALSE FALSE FALSE
GCST006061 0.0075 0.2275 537409 outcome TRUE reported 18304_19_BCCIP_BCCIP
BCCIP chr1 196695521 0.008129 3.846e-39 35338 TRUE reported
textfile 2 1 TRUE
8324 rs1706435 G A G A 0.0625 0.0117 0.36981 NA FALSE FALSE FALSE
GCST006061 0.0072 0.1047 537409 outcome TRUE reported 18304_19_BCCIP_BCCIP
BCCIP chr3 165743688 0.008247 3.501e-14 35339 TRUE reported
textfile 2 1 TRUE
8325 rs704 A G A G 0.1123 -0.0155 0.49189 NA FALSE FALSE FALSE GCST006061
0.0072 0.03144 537409 outcome TRUE reported 18304_19_BCCIP_BCCIP BCCIP
chr17 28367840 0.007968 4.181e-45 35360 TRUE reported textfile 2
1 TRUE
8326 rs10922098 T C T C -0.1644 -0.009 0.40011 NA FALSE FALSE FALSE
GCST006061 0.0075 0.2275 537409 outcome TRUE reported 2704_74_TNFRSF13B_TACI
TNFRSF13B chr1 196695521 0.00815 1.771e-90 35338 TRUE reported
textfile 2 1 TRUE
8327 rs1707652 T C T C 0.0729 0.0081 0.3102 NA FALSE FALSE FALSE
GCST006061 0.0074 0.2701 537409 outcome TRUE reported 2704_74_TNFRSF13B_TACI
TNFRSF13B chr3 165761011 0.008639 3.22e-17 35339 TRUE reported
textfile 2 1 TRUE
8328 rs10922098 T C T C -0.1087 -0.009 0.40011 NA FALSE FALSE FALSE
GCST006061 0.0075 0.2275 537409 outcome TRUE reported
8002_27_TMPRSS5_Spinesin TMPRSS5 chr1 196695521 0.008101 4.771e-41 35338
TRUE reported textfile 2 1 TRUE
8329 rs1355538 G A G A -0.0504 -0.0076 0.39847 NA FALSE FALSE FALSE

GCST006061 0.0072 0.2923 537409 outcome TRUE reported
8002_27_TMPRSS5_Spinesin TMPRSS5 chr3 165787389 0.008102 4.959e-10 35338
TRUE reported textfile 2 1 TRUE
8330 rs10922098 T C T C -0.1483 -0.009 0.40011 NA FALSE FALSE FALSE
GCST006061 0.0075 0.2275 537409 outcome TRUE reported 15346_31_IFNG_IFN_g
IFNG chr1 196695521 0.008085 3.836e-75 35338 TRUE reported
textfile 2 1 TRUE
8331 rs1707652 T C T C 0.0721 0.0081 0.3102 NA FALSE FALSE FALSE
GCST006061 0.0074 0.2701 537409 outcome TRUE reported 15346_31_IFNG_IFN_g
IFNG chr3 165761011 0.008557 3.59e-17 35339 TRUE reported
textfile 2 1 TRUE
8332 rs10922098 T C T C -0.0489 -0.009 0.40011 NA FALSE FALSE FALSE
GCST006061 0.0075 0.2275 537409 outcome TRUE reported 17752_24_ARG2_Arginase
ARG2 chr1 196695521 0.008046 1.223e-09 35338 TRUE reported
textfile 2 1 TRUE
8333 rs9558305 G T G T 0.052 -0.0113 0.22519 NA FALSE FALSE FALSE
GCST006061 0.009 0.2071 537409 outcome TRUE reported 17752_24_ARG2_Arginase
ARG2 chr13 104505041 0.009422 3.405e-08 35363 TRUE reported
textfile 2 1 TRUE
8334 rs10922098 T C T C -0.0504 -0.009 0.40011 NA FALSE FALSE FALSE
GCST006061 0.0075 0.2275 537409 outcome TRUE reported 18400_52_ALKBH2_ALKB2
ALKBH2 chr1 196695521 0.007913 1.904e-10 35338 TRUE reported
textfile 2 1 TRUE
8335 rs10922098 T C T C -0.16 -0.009 0.40011 NA FALSE FALSE FALSE
GCST006061 0.0075 0.2275 537409 outcome TRUE reported 13378_80_C5orf63_YD286
C5orf63 chr1 196695521 0.008102 8.446e-87 35329 TRUE reported
textfile 2 1 TRUE
8336 rs12478841 G A G A 0.0525 0.0049 0.21569 NA FALSE FALSE FALSE
GCST006061 0.008 0.5413 537409 outcome TRUE reported 13378_80_C5orf63_YD286
C5orf63 chr2 27588855 0.009591 4.396e-08 35364 TRUE reported
textfile 2 1 TRUE
8337 rs139160896 A G A G 0.2302 0.0527 0.01243 NA FALSE FALSE FALSE
GCST006061 0.0392 0.1791 537409 outcome TRUE reported 13378_80_C5orf63_YD286
C5orf63 chr3 133841736 0.03606 1.728e-10 35349 TRUE reported textfile
2 1 TRUE
8338 rs1707652 T C T C 0.0762 0.0081 0.3102 NA FALSE FALSE FALSE
GCST006061 0.0074 0.2701 537409 outcome TRUE reported 13378_80_C5orf63_YD286
C5orf63 chr3 165761011 0.008586 6.98e-19 35330 TRUE reported
textfile 2 1 TRUE
8339 rs28645249 C T C T 0.0598 0.019 0.26162 NA FALSE FALSE FALSE
GCST006061 0.0115 0.09815 537409 outcome TRUE reported 13378_80_C5orf63_YD286
C5orf63 chr6 32557458 0.008736 7.63e-12 35429 TRUE reported
textfile 2 1 TRUE
8340 rs10922098 T C T C -0.1619 -0.009 0.40011 NA FALSE FALSE FALSE
GCST006061 0.0075 0.2275 537409 outcome TRUE reported 12456_5_PFKFB3_F263
PFKFB3 chr1 196695521 0.00818 3.557e-87 35329 TRUE reported textfile
2 1 TRUE
8341 rs1564879 A G A G -0.0585 -0.0281 0.4658 NA FALSE FALSE FALSE
GCST006061 0.0072 0.0001006 537409 outcome TRUE reported 12456_5_PFKFB3_F263
PFKFB3 chr1 207078173 0.008093 4.891e-13 35363 TRUE reported
textfile 2 1 TRUE
8342 rs1706435 G A G A 0.0816 0.0117 0.36981 NA FALSE FALSE FALSE
GCST006061 0.0072 0.1047 537409 outcome TRUE reported 12456_5_PFKFB3_F263
PFKFB3 chr3 165743688 0.008304 8.631e-23 35330 TRUE reported
textfile 2 1 TRUE
8343 rs704 A G A G 0.0612 -0.0155 0.49189 NA FALSE FALSE FALSE GCST006061
0.0072 0.03144 537409 outcome TRUE reported 12456_5_PFKFB3_F263 PFKFB3 chr17
28367840 0.008037 2.634e-14 35351 TRUE reported textfile 2 1 TRUE
8344 rs74175068 C T C T 0.0541 0.0049 0.21639 NA FALSE FALSE FALSE
GCST006061 0.0079 0.541 537409 outcome TRUE reported 12456_5_PFKFB3_F263
PFKFB3 chr2 27610635 0.009668 2.197e-08 35364 TRUE reported
textfile 2 1 TRUE
8345 rs10922098 T C T C -0.1207 -0.009 0.40011 NA FALSE FALSE FALSE
GCST006061 0.0075 0.2275 537409 outcome TRUE reported 13960_15_AGAP3_AGAP3
AGAP3 chr1 196695521 0.007992 1.544e-51 35338 TRUE reported
textfile 2 1 TRUE

8346 rs1355538 G A G A -0.0599 -0.0076 0.39847 NA FALSE FALSE FALSE
GCST006061 0.0072 0.2923 537409 outcome TRUE reported 13960_15_AGAP3_AGAP3
AGAP3 chr3 165787389 0.008002 7.096e-14 35338 TRUE reported
textfile 2 1 TRUE
8347 rs10922098 T C T C -0.2261 -0.009 0.40011 NA FALSE FALSE FALSE
GCST006061 0.0075 0.2275 537409 outcome TRUE reported 9374_24_NNAT_NNAT
NNAT chr1 196695521 0.008046 9.589e-174 35338 TRUE reported
textfile 2 1 TRUE
8348 rs1451915 A C A C 0.0722 -0.0107 0.13641 NA FALSE FALSE FALSE
GCST006061 0.0124 0.3895 537409 outcome TRUE reported 9374_24_NNAT_NNAT
NNAT chr1 196058002 0.011511 3.562e-10 35335 TRUE reported
textfile 2 1 TRUE
8349 rs1707652 T C T C 0.093 0.0081 0.3102 NA FALSE FALSE FALSE
GCST006061 0.0074 0.2701 537409 outcome TRUE reported 9374_24_NNAT_NNAT
NNAT chr3 165761011 0.008548 1.435e-27 35339 TRUE reported
textfile 2 1 TRUE
8350 rs7513100 C T C T 0.069 0 0.14871 NA FALSE FALSE FALSE GCST006061
0.0098 0.9981 537409 outcome TRUE reported 9374_24_NNAT_NNAT NNAT chr1
198065935 0.011122 5.517e-10 35343 TRUE reported textfile 2 1 TRUE
8351 rs10922098 T C T C -0.064 -0.009 0.40011 NA FALSE FALSE FALSE
GCST006061 0.0075 0.2275 537409 outcome TRUE reported 12437_18_ULK3_ULK3
ULK3 chr1 196695521 0.008042 1.75e-15 35329 TRUE reported
textfile 2 1 TRUE
8352 rs1619994 G A G A 0.0499 0.0168 0.2807 NA FALSE FALSE FALSE
GCST006061 0.0078 0.03057 537409 outcome TRUE reported 12437_18_ULK3_ULK3
ULK3 chr3 165744047 0.008779 1.314e-08 35330 TRUE reported
textfile 2 1 TRUE
8353 rs2731672 C T C T 0.0617 -0.0025 0.25436 NA FALSE FALSE FALSE
GCST006061 0.0083 0.7666 537409 outcome TRUE reported 12437_18_ULK3_ULK3
ULK3 chr5 177415473 0.009105 1.229e-11 35353 TRUE reported
textfile 2 1 TRUE
8354 rs936228 C T C T -0.0869 -0.0127 0.25656 NA FALSE FALSE FALSE
GCST006061 0.0078 0.1011 537409 outcome TRUE reported 12437_18_ULK3_ULK3
ULK3 chr15 74839823 0.009018 5.612e-22 35352 TRUE reported
textfile 2 1 TRUE
8355 rs10922098 T C T C -0.1212 -0.009 0.40011 NA FALSE FALSE FALSE
GCST006061 0.0075 0.2275 537409 outcome TRUE reported 9233_71_TFPI2_TFPI__2
TFPI2 chr1 196695521 0.008169 8.458e-50 35338 TRUE reported
textfile 2 1 TRUE
8356 rs11039122 A G A G -0.0515 0.0062 0.45755 NA FALSE FALSE FALSE
GCST006061 0.0073 0.3992 537409 outcome TRUE reported 9233_71_TFPI2_TFPI__2
TFPI2 chr11 47191566 0.00806 1.664e-10 35377 TRUE reported textfile
2 1 TRUE
8357 rs1873400 A G A G -0.0811 -0.001 0.31071 NA FALSE FALSE FALSE
GCST006061 0.0077 0.9007 537409 outcome TRUE reported 9233_71_TFPI2_TFPI__2
TFPI2 chr3 126538393 0.00866 7.65e-21 35371 TRUE reported textfile
2 1 TRUE
8358 rs62466701 C T C T 0.1089 0.02 0.06581 NA FALSE FALSE FALSE
GCST006061 0.0186 0.2817 537409 outcome TRUE reported 9233_71_TFPI2_TFPI__2
TFPI2 chr7 93956351 0.016163 1.612e-11 35348 TRUE reported
textfile 2 1 TRUE
8359 rs6445035 A G A G 0.1262 0.0154 0.1904 NA FALSE FALSE FALSE
GCST006061 0.0089 0.08256 537409 outcome TRUE reported 9233_71_TFPI2_TFPI__2
TFPI2 chr3 165762312 0.010167 2.221e-35 35339 TRUE reported
textfile 2 1 TRUE
8360 rs704 A G A G -0.0686 -0.0155 0.49189 NA FALSE FALSE FALSE GCST006061
0.0072 0.03144 537409 outcome TRUE reported 9233_71_TFPI2_TFPI__2 TFPI2
chr17 28367840 0.008012 1.112e-17 35360 TRUE reported textfile 2
1 TRUE
8361 rs962656 T C T C -0.08 0.0186 0.09903 NA FALSE FALSE FALSE
GCST006061 0.0107 0.08152 537409 outcome TRUE reported 9233_71_TFPI2_TFPI__2
TFPI2 chr12 25051419 0.013372 2.197e-09 35364 TRUE reported
textfile 2 1 TRUE
8362 rs10922098 T C T C -0.0815 -0.009 0.40011 NA FALSE FALSE FALSE
GCST006061 0.0075 0.2275 537409 outcome TRUE reported 6547_83_TMPRSS11D_TM11D
TMPRSS11D chr1 196695521 0.008063 5.095e-24 35338 TRUE reported

textfile 2 1 TRUE
8363 rs62251293 T C T C -0.0448 -0.0029 0.37125 NA FALSE FALSE FALSE
GCST006061 0.0078 0.7106 537409 outcome TRUE reported 6547_83_TMPRSS11D_TM11D
TMPRSS11D chr3 44213973 0.008171 4.187e-08 35346 TRUE reported
textfile 2 1 TRUE
8364 rs73089342 A G A G 0.0674 -0.0054 0.12473 NA FALSE FALSE FALSE
GCST006061 0.0119 0.6513 537409 outcome TRUE reported 6547_83_TMPRSS11D_TM11D
TMPRSS11D chr3 45117930 0.012009 1.993e-08 35341 TRUE reported
textfile 2 1 TRUE
8365 rs10922098 T C T C -0.1865 -0.009 0.40011 NA FALSE FALSE FALSE
GCST006061 0.0075 0.2275 537409 outcome TRUE reported 13658_31_PDGFC_PDGF_CC
PDGFC chr1 196695521 0.008067 2.926e-118 35338 TRUE reported
textfile 2 1 TRUE
8366 rs10922098 T C T C -0.1718 -0.009 0.40011 NA FALSE FALSE FALSE
GCST006061 0.0075 0.2275 537409 outcome TRUE reported 5343_74_CPE_CBPE CPE
chr1 196695521 0.008163 2.505e-98 35338 TRUE reported textfile 2
1 TRUE
8367 rs11569415 A G A G -0.0631 0.0011 0.24276 NA FALSE FALSE FALSE
GCST006061 0.0097 0.9129 537409 outcome TRUE reported 5343_74_CPE_CBPE CPE
chr19 6716268 0.009321 1.291e-11 35372 TRUE reported textfile 2 1
TRUE
8368 rs1707652 T C T C 0.1071 0.0081 0.3102 NA FALSE FALSE FALSE
GCST006061 0.0074 0.2701 537409 outcome TRUE reported 5343_74_CPE_CBPE CPE
chr3 165761011 0.008645 3.008e-35 35339 TRUE reported textfile 2
1 TRUE
8369 rs429358 C T C T 0.1125 0.005 0.17407 NA FALSE FALSE FALSE
GCST006061 0.0103 0.6319 537409 outcome TRUE reported 5343_74_CPE_CBPE CPE
chr19 44908684 0.010687 6.482e-26 35368 TRUE reported textfile 2
1 TRUE
8370 rs10922098 T C T C -0.1326 -0.009 0.40011 NA FALSE FALSE FALSE
GCST006061 0.0075 0.2275 537409 outcome TRUE reported 15315_64_CUTC_CUTC
CUTC chr1 196695521 0.007996 9.27e-62 35338 TRUE reported
textfile 2 1 TRUE
8371 rs10922098 T C T C -0.1195 -0.009 0.40011 NA FALSE FALSE FALSE
GCST006061 0.0075 0.2275 537409 outcome TRUE reported 8671_378_TRAPPC5_TPPC5
TRAPPC5 chr1 196695521 0.008144 9.425e-49 35338 TRUE reported
textfile 2 1 TRUE
8372 rs10922098 T C T C -0.0576 -0.009 0.40011 NA FALSE FALSE FALSE
GCST006061 0.0075 0.2275 537409 outcome TRUE reported 9053_16_CRIP2_CRIP2
CRIP2 chr1 196695521 0.00811 1.23e-12 35338 TRUE reported textfile
2 1 TRUE
8373 rs188530181 C A C A -0.4166 -0.0578 0.0126 NA FALSE FALSE FALSE
GCST006061 0.0522 0.2683 537409 outcome TRUE reported 9053_16_CRIP2_CRIP2
CRIP2 chr1 223691121 0.035722 1.986e-31 35361 TRUE reported
textfile 2 1 TRUE
8374 rs26515 T C T C 0.0847 -0.0015 0.49919 NA FALSE FALSE FALSE GCST006061
0.0071 0.8327 537409 outcome TRUE reported 9053_16_CRIP2_CRIP2 CRIP2 chr5
96719133 0.007957 1.845e-26 35349 TRUE reported textfile 2 1 TRUE
8375 rs10922098 T C T C -0.0922 -0.009 0.40011 NA FALSE FALSE FALSE
GCST006061 0.0075 0.2275 537409 outcome TRUE reported 18414_26_ARL5A_ARL5A
ARL5A chr1 196695521 0.008081 3.732e-30 35338 TRUE reported
textfile 2 1 TRUE
8376 rs1706435 G A G A 0.0657 0.0117 0.36981 NA FALSE FALSE FALSE
GCST006061 0.0072 0.1047 537409 outcome TRUE reported 18414_26_ARL5A_ARL5A
ARL5A chr3 165743688 0.008186 1.006e-15 35339 TRUE reported
textfile 2 1 TRUE
8377 rs10922098 T C T C -0.1207 -0.009 0.40011 NA FALSE FALSE FALSE
GCST006061 0.0075 0.2275 537409 outcome TRUE reported 7821_6_C1D_C1D C1D
chr1 196695521 0.008144 1.087e-49 35338 TRUE reported textfile 2
1 TRUE
8378 rs1355538 G A G A -0.0575 -0.0076 0.39847 NA FALSE FALSE FALSE
GCST006061 0.0072 0.2923 537409 outcome TRUE reported 7821_6_C1D_C1D C1D
chr3 165787389 0.008149 1.709e-12 35338 TRUE reported textfile 2
1 TRUE
8379 rs10922098 T C T C -0.1105 -0.009 0.40011 NA FALSE FALSE FALSE
GCST006061 0.0075 0.2275 537409 outcome TRUE reported 8908_14_KCNE5_KCE1L

KCNE5 chr1 196695521 0.008512 1.564e-38 35338 TRUE reported textfile 2 1 TRUE
8380 rs117801489 C T C T 0.1292 -0.0141 0.04343 NA FALSE FALSE FALSE GCST006061 0.034 0.6772 537409 outcome TRUE reported 8908_14_KCNE5_KCE1L KCNE5 chr12 104015054 0.020357 2.2e-10 35361 TRUE reported textfile 2 1 TRUE
8381 rs150849265 T C T C -0.0942 0.0014 0.06174 NA FALSE FALSE FALSE GCST006061 0.0146 0.9236 537409 outcome TRUE reported 8908_14_KCNE5_KCE1L KCNE5 chr19 49520198 0.017262 4.838e-08 35375 TRUE reported textfile 2 1 TRUE
8382 rs2233726 G A G A -0.2186 -0.0057 0.01139 NA FALSE FALSE FALSE GCST006061 0.0431 0.8957 537409 outcome TRUE reported 8908_14_KCNE5_KCE1L KCNE5 chr1 203483653 0.037913 8.125e-09 35367 TRUE reported textfile 2 1 TRUE
8383 rs2794457 T C T C 0.1181 -0.0236 0.04695 NA FALSE FALSE FALSE GCST006061 0.0145 0.1024 537409 outcome TRUE reported 8908_14_KCNE5_KCE1L KCNE5 chr1 203461532 0.019557 1.552e-09 35368 TRUE reported textfile 2 1 TRUE
8384 rs10922098 T C T C -0.055 -0.009 0.40011 NA FALSE FALSE FALSE GCST006061 0.0075 0.2275 537409 outcome TRUE reported 10953_14_CLEC2A_CLC2A CLEC2A chr1 196695521 0.008144 1.445e-11 35329 TRUE reported textfile 2 1 TRUE
8385 rs11196979 G A G A -0.0745 0.0172 0.11452 NA FALSE FALSE FALSE GCST006061 0.0102 0.0914 537409 outcome TRUE reported 10953_14_CLEC2A_CLC2A CLEC2A chr10 114904626 0.01257 3.091e-09 35373 TRUE reported textfile 2 1 TRUE
8386 rs485305 A C A C -0.1994 -0.0376 0.02166 NA FALSE FALSE FALSE GCST006061 0.0245 0.1243 537409 outcome TRUE reported 10953_14_CLEC2A_CLC2A CLEC2A chr10 114288286 0.026955 1.389e-13 35368 TRUE reported textfile 2 1 TRUE
8387 rs7080536 A G A G -0.6371 -0.0145 0.04142 NA FALSE FALSE FALSE GCST006061 0.0203 0.4753 537409 outcome TRUE reported 10953_14_CLEC2A_CLC2A CLEC2A chr10 113588287 0.019699 1e-200 35378 TRUE reported textfile 2 1 TRUE
8388 rs77774564 A G A G -0.3431 0.0637 0.01388 NA FALSE FALSE FALSE GCST006061 0.0403 0.1143 537409 outcome TRUE reported 10953_14_CLEC2A_CLC2A CLEC2A chr10 113637445 0.033605 1.79e-24 35378 TRUE reported textfile 2 1 TRUE
8389 rs10922098 T C T C -0.0863 -0.009 0.40011 NA FALSE FALSE FALSE GCST006061 0.0075 0.2275 537409 outcome TRUE reported 6469_62_LY6G6D_LY66D LY6G6D chr1 196695521 0.008093 1.511e-26 35338 TRUE reported textfile 2 1 TRUE
8390 rs10922098 T C T C -0.0745 -0.009 0.40011 NA FALSE FALSE FALSE GCST006061 0.0075 0.2275 537409 outcome TRUE reported 7825_7_ABHD12_ABD12 ABHD12 chr1 196695521 0.00811 4.059e-20 35338 TRUE reported textfile 2 1 TRUE
8391 rs10922098 T C T C -0.0516 -0.009 0.40011 NA FALSE FALSE FALSE GCST006061 0.0075 0.2275 537409 outcome TRUE reported 8378_3_LDOC1_LDOC1 LDOC1 chr1 196695521 0.008085 1.744e-10 35338 TRUE reported textfile 2 1 TRUE
8392 rs2956479 T C T C 0.0604 0.0103 0.16565 NA FALSE FALSE FALSE GCST006061 0.0107 0.3331 537409 outcome TRUE reported 8378_3_LDOC1_LDOC1 LDOC1 chr14 106588438 0.010939 3.361e-08 34966 TRUE reported textfile 2 1 TRUE
8393 rs919791 A G A G -0.1252 -0.0081 0.2132 NA FALSE FALSE FALSE GCST006061 0.0088 0.3607 537409 outcome TRUE reported 8378_3_LDOC1_LDOC1 LDOC1 chr19 15466006 0.009586 5.549e-39 35378 TRUE reported textfile 2 1 TRUE
8394 rs10922098 T C T C -0.1876 -0.009 0.40011 NA FALSE FALSE FALSE GCST006061 0.0075 0.2275 537409 outcome TRUE reported 4280_47_PSMA2_PSA2 PSMA2 chr1 196695521 0.008075 2.098e-119 35338 TRUE reported textfile 2 1 TRUE
8395 rs11569415 A G A G -0.0567 0.0011 0.24276 NA FALSE FALSE FALSE GCST006061 0.0097 0.9129 537409 outcome TRUE reported 4280_47_PSMA2_PSA2 PSMA2 chr19 6716268 0.009217 7.682e-10 35372 TRUE reported textfile 2 1 TRUE

8396 rs1451915 A C A C 0.0631 -0.0107 0.13641 NA FALSE FALSE FALSE
GCST006061 0.0124 0.3895 537409 outcome TRUE reported 4280_47_PSMA2_PSA2
PSMA2 chr1 196058002 0.011513 4.234e-08 35335 TRUE reported
textfile 2 1 TRUE
8397 rs2686395 C T C T -0.0908 -0.0077 0.3101 NA FALSE FALSE FALSE
GCST006061 0.0074 0.2965 537409 outcome TRUE reported 4280_47_PSMA2_PSA2
PSMA2 chr3 165767231 0.008568 3.047e-26 35339 TRUE reported
textfile 2 1 TRUE
8398 rs10922098 T C T C -0.0817 -0.009 0.40011 NA FALSE FALSE FALSE
GCST006061 0.0075 0.2275 537409 outcome TRUE reported 11110_4_TMEM119_TM119
TMEM119 chr1 196695521 0.008189 1.927e-23 35329 TRUE reported
textfile 2 1 TRUE
8399 rs1355538 G A G A -0.0481 -0.0076 0.39847 NA FALSE FALSE FALSE
GCST006061 0.0072 0.2923 537409 outcome TRUE reported 11110_4_TMEM119_TM119
TMEM119 chr3 165787389 0.008177 4.053e-09 35329 TRUE reported
textfile 2 1 TRUE
8400 rs802171 G A G A 0.0633 0.0054 0.47349 NA FALSE FALSE FALSE
GCST006061 0.0073 0.459 537409 outcome TRUE reported 11110_4_TMEM119_TM119
TMEM119 chr10 16814686 0.008018 2.902e-15 35354 TRUE reported
textfile 2 1 TRUE
8401 rs10922098 T C T C -0.103 -0.009 0.40011 NA FALSE FALSE FALSE
GCST006061 0.0075 0.2275 537409 outcome TRUE reported 6921_24_B4GALT3_B4GT3
B4GALT3 chr1 196695521 0.008105 5.322e-37 35338 TRUE reported
textfile 2 1 TRUE
8402 rs6542680 T C T C -0.0718 -0.0012 0.18362 NA FALSE FALSE FALSE
GCST006061 0.0091 0.8935 537409 outcome TRUE reported 6921_24_B4GALT3_B4GT3
B4GALT3 chr2 3592552 0.010285 2.933e-12 35332 TRUE reported textfile
2 1 TRUE
8403 rs10922098 T C T C -0.0763 -0.009 0.40011 NA FALSE FALSE FALSE
GCST006061 0.0075 0.2275 537409 outcome TRUE reported 6240_70_RSPO1_RSPO1
RSPO1 chr1 196695521 0.008086 3.871e-21 35338 TRUE reported
textfile 2 1 TRUE
8404 rs1355538 G A G A -0.0444 -0.0076 0.39847 NA FALSE FALSE FALSE
GCST006061 0.0072 0.2923 537409 outcome TRUE reported 6240_70_RSPO1_RSPO1
RSPO1 chr3 165787389 0.008077 3.867e-08 35338 TRUE reported
textfile 2 1 TRUE
8405 rs10922098 T C T C -0.062 -0.009 0.40011 NA FALSE FALSE FALSE
GCST006061 0.0075 0.2275 537409 outcome TRUE reported 16859_100_SH3GLB1_SHLB1
SH3GLB1 chr1 196695521 0.008076 1.626e-14 35338 TRUE reported
textfile 2 1 TRUE
8406 rs11679004 A C A C 0.0941 0.0149 0.06532 NA FALSE FALSE FALSE
GCST006061 0.0283 0.5978 537409 outcome TRUE reported 16859_100_SH3GLB1_SHLB1
SH3GLB1 chr2 89150383 0.015882 3.122e-09 35350 TRUE reported
textfile 2 1 TRUE
8407 rs1619994 G A G A 0.0541 0.0168 0.2807 NA FALSE FALSE FALSE
GCST006061 0.0078 0.03057 537409 outcome TRUE reported 16859_100_SH3GLB1_SHLB1
SH3GLB1 chr3 165744047 0.008803 7.979e-10 35339 TRUE reported
textfile 2 1 TRUE
8408 rs10922098 T C T C -0.0999 -0.009 0.40011 NA FALSE FALSE FALSE
GCST006061 0.0075 0.2275 537409 outcome TRUE reported 12794_6_NLRP4_NALP4
NLRP4 chr1 196695521 0.008109 7.117e-35 35329 TRUE reported
textfile 2 1 TRUE
8409 rs6542680 T C T C -0.0714 -0.0012 0.18362 NA FALSE FALSE FALSE
GCST006061 0.0091 0.8935 537409 outcome TRUE reported 12794_6_NLRP4_NALP4
NLRP4 chr2 3592552 0.010275 3.69e-12 35323 TRUE reported textfile
2 1 TRUE
8410 rs10922098 T C T C -0.0384 -0.009 0.40011 NA FALSE FALSE FALSE
GCST006061 0.0075 0.2275 537409 outcome TRUE reported 9253_52_ABO_BGAT ABO
chr1 196695521 0.006337 1.365e-09 35338 TRUE reported textfile 2
1 TRUE
8411 rs11522324 C T C T -0.2593 0.0236 0.10009 NA FALSE FALSE FALSE
GCST006061 0.0115 0.0396 537409 outcome TRUE reported 9253_52_ABO_BGAT ABO
chr9 133543821 0.014293 1.478e-73 35365 TRUE reported textfile 2
1 TRUE
8412 rs139562936 T G T G 0.3691 -0.0301 0.0211 NA FALSE FALSE FALSE
GCST006061 0.034 0.3765 537409 outcome TRUE reported 9253_52_ABO_BGAT ABO

chr9 134215558 0.029787 2.916e-35 35372 TRUE reported textfile 2
1 TRUE
8413 rs176694 G T G T 0.4763 -0.0345 0.09036 NA FALSE FALSE FALSE
GCST006061 0.0125 0.005629 537409 outcome TRUE reported 9253_52_ABO_BGAT
ABO chr9 133289021 0.014845 1e-200 35370 TRUE reported textfile 2
1 TRUE
8414 rs2313155 G A G A -0.2319 0.0039 0.02148 NA FALSE FALSE FALSE
GCST006061 0.0327 0.9048 537409 outcome TRUE reported 9253_52_ABO_BGAT ABO
chr9 134347416 0.029905 8.86e-15 35364 TRUE reported textfile 2
1 TRUE
8415 rs4962160 A G A G -0.283 -0.0144 0.15836 NA FALSE FALSE FALSE
GCST006061 0.012 0.2313 537409 outcome TRUE reported 9253_52_ABO_BGAT ABO
chr9 133501627 0.01164 1.451e-130 35364 TRUE reported textfile 2 1
TRUE
8416 rs56296048 A G A G -0.2644 0.0386 0.01639 NA FALSE FALSE FALSE
GCST006061 0.0589 0.5118 537409 outcome TRUE reported 9253_52_ABO_BGAT ABO
chr9 133788429 0.033937 6.659e-15 35371 TRUE reported textfile 2
1 TRUE
8417 rs56302081 T C T C -0.3592 0.0108 0.49193 NA FALSE FALSE FALSE
GCST006061 0.0073 0.138 537409 outcome TRUE reported 9253_52_ABO_BGAT ABO
chr9 133193023 0.004888 1e-200 35368 TRUE reported textfile 2 1
TRUE
8418 rs7033492 C T C T 0.2707 0.0017 0.4994 NA FALSE FALSE FALSE
GCST006061 0.0079 0.8288 537409 outcome TRUE reported 9253_52_ABO_BGAT ABO
chr9 133165599 0.008423 1e-200 35366 TRUE reported textfile 2 1
TRUE
8419 rs787891 T C T C 0.0832 0.0092 0.4191 NA FALSE FALSE FALSE
GCST006061 0.0075 0.2166 537409 outcome TRUE reported 9253_52_ABO_BGAT ABO
chr9 132469773 0.008714 1.329e-21 35372 TRUE reported textfile 2
1 TRUE
8420 rs10922098 T C T C -0.1198 -0.009 0.40011 NA FALSE FALSE FALSE
GCST006061 0.0075 0.2275 537409 outcome TRUE reported
13631_1_RBBP5_RB_binding_protein_5 RBBP5 chr1 196695521 0.00809 1.281e-49
35338 TRUE reported textfile 2 1 TRUE
8421 rs1355538 G A G A -0.0552 -0.0076 0.39847 NA FALSE FALSE FALSE
GCST006061 0.0072 0.2923 537409 outcome TRUE reported
13631_1_RBBP5_RB_binding_protein_5 RBBP5 chr3 165787389 0.008098 9.336e-12
35338 TRUE reported textfile 2 1 TRUE
8422 rs10922098 T C T C -0.072 -0.009 0.40011 NA FALSE FALSE FALSE
GCST006061 0.0075 0.2275 537409 outcome TRUE reported 11201_19_PUS1_TRUA
PUS1 chr1 196695521 0.008024 2.873e-19 35329 TRUE reported
textfile 2 1 TRUE
8423 rs10922098 T C T C -0.162 -0.009 0.40011 NA FALSE FALSE FALSE
GCST006061 0.0075 0.2275 537409 outcome TRUE reported 9241_40_SIRPG_SIRPG
SIRPG chr1 196695521 0.008165 1.344e-87 35338 TRUE reported
textfile 2 1 TRUE
8424 rs1707652 T C T C 0.0929 0.0081 0.3102 NA FALSE FALSE FALSE
GCST006061 0.0074 0.2701 537409 outcome TRUE reported 9241_40_SIRPG_SIRPG
SIRPG chr3 165761011 0.008648 6.443e-27 35339 TRUE reported
textfile 2 1 TRUE
8425 rs6043409 G A G A 0.1514 0.0045 0.31777 NA FALSE FALSE FALSE
GCST006061 0.0077 0.5553 537409 outcome TRUE reported 9241_40_SIRPG_SIRPG
SIRPG chr20 1635560 0.008573 8.456e-70 35374 TRUE reported textfile
2 1 TRUE
8426 rs10922098 T C T C -0.0509 -0.009 0.40011 NA FALSE FALSE FALSE
GCST006061 0.0075 0.2275 537409 outcome TRUE reported 13947_371_NUDT12_NUD12
NUDT12 chr1 196695521 0.008139 3.996e-10 35338 TRUE reported
textfile 2 1 TRUE
8427 rs13155553 A G A G -0.1631 -0.0178 0.03572 NA FALSE FALSE FALSE
GCST006061 0.0227 0.4336 537409 outcome TRUE reported 13947_371_NUDT12_NUD12
NUDT12 chr5 100740817 0.021324 2.032e-14 35348 TRUE reported
textfile 2 1 TRUE
8428 rs140216035 A G A G -0.1936 -0.0113 0.02518 NA FALSE FALSE FALSE
GCST006061 0.0353 0.7484 537409 outcome TRUE reported 13947_371_NUDT12_NUD12
NUDT12 chr5 103795063 0.025164 1.43e-14 35344 TRUE reported
textfile 2 1 TRUE

8429 rs1477619 A G A G 0.0844 -0.0079 0.21701 NA FALSE FALSE FALSE
GCST006061 0.0083 0.3393 537409 outcome TRUE reported 13947_371_NUDT12_NUD12
NUDT12 chr5 102096794 0.009743 4.623e-18 35344 TRUE reported
textfile 2 1 TRUE
8430 rs10922098 T C T C -0.1239 -0.009 0.40011 NA FALSE FALSE FALSE
GCST006061 0.0075 0.2275 537409 outcome TRUE reported 16308_14_BTLA_BTLA
BTLA chr1 196695521 0.008099 7.895e-53 35338 TRUE reported
textfile 2 1 TRUE
8431 rs10922098 T C T C -0.057 -0.009 0.40011 NA FALSE FALSE FALSE
GCST006061 0.0075 0.2275 537409 outcome TRUE reported 13936_24_PGK2_PGK2
PGK2 chr1 196695521 0.008044 1.378e-12 35338 TRUE reported
textfile 2 1 TRUE
8432 rs62205397 T C T C -0.1018 0.0211 0.18823 NA FALSE FALSE FALSE
GCST006061 0.0099 0.03316 537409 outcome TRUE reported 13936_24_PGK2_PGK2
PGK2 chr20 59021956 0.010012 2.756e-24 35381 TRUE reported
textfile 2 1 TRUE
8433 rs10922098 T C T C -0.0757 -0.009 0.40011 NA FALSE FALSE FALSE
GCST006061 0.0075 0.2275 537409 outcome TRUE reported 5494_52_SNRPF_RUXF
SNRPF chr1 196695521 0.00822 3.289e-20 35338 TRUE reported textfile
2 1 TRUE
8434 rs429358 C T C T -0.2643 0.005 0.17407 NA FALSE FALSE FALSE
GCST006061 0.0103 0.6319 537409 outcome TRUE reported 5494_52_SNRPF_RUXF
SNRPF chr19 44908684 0.010671 2.002e-135 35368 TRUE reported
textfile 2 1 TRUE
8435 rs72952553 A G A G -0.0489 -0.0042 0.31481 NA FALSE FALSE FALSE
GCST006061 0.0085 0.6159 537409 outcome TRUE reported 5494_52_SNRPF_RUXF
SNRPF chr2 216616793 0.008695 1.864e-08 35359 TRUE reported
textfile 2 1 TRUE
8436 rs73169103 T C T C 0.1225 0.0178 0.03563 NA FALSE FALSE FALSE
GCST006061 0.0249 0.4756 537409 outcome TRUE reported 5494_52_SNRPF_RUXF
SNRPF chr3 167190675 0.021702 1.655e-08 35354 TRUE reported
textfile 2 1 TRUE
8437 rs10922098 T C T C -0.065 -0.009 0.40011 NA FALSE FALSE FALSE
GCST006061 0.0075 0.2275 537409 outcome TRUE reported 15641_20_TMEFF1_TEFF1
TMEFF1 chr1 196695521 0.008292 4.547e-15 35338 TRUE reported
textfile 2 1 TRUE
8438 rs1555405 A G A G -0.0499 -0.0047 0.30381 NA FALSE FALSE FALSE
GCST006061 0.0081 0.5592 537409 outcome TRUE reported 15641_20_TMEFF1_TEFF1
TMEFF1 chr14 100710432 0.008874 1.872e-08 35380 TRUE reported
textfile 2 1 TRUE
8439 rs3983768 C T C T 0.0755 0.009 0.14187 NA FALSE FALSE FALSE
GCST006061 0.0106 0.3925 537409 outcome TRUE reported 15641_20_TMEFF1_TEFF1
TMEFF1 chr9 100484832 0.011525 5.706e-11 35343 TRUE reported
textfile 2 1 TRUE
8440 rs7073753 T C T C 0.1003 -0.0472 0.4356 NA FALSE FALSE FALSE
GCST006061 0.0072 5.02e-11 537409 outcome TRUE reported
15641_20_TMEFF1_TEFF1 TMEFF1 chr10 63303060 0.00817 1.21e-34 35351 TRUE
reported textfile 2 1 TRUE
8441 rs10922098 T C T C -0.1075 -0.009 0.40011 NA FALSE FALSE FALSE
GCST006061 0.0075 0.2275 537409 outcome TRUE reported 16932_5_SULT1E1_SULT_1E
SULT1E1 chr1 196695521 0.007979 2.269e-41 35338 TRUE reported
textfile 2 1 TRUE
8442 rs1706435 G A G A 0.0583 0.0117 0.36981 NA FALSE FALSE FALSE
GCST006061 0.0072 0.1047 537409 outcome TRUE reported 16932_5_SULT1E1_SULT_1E
SULT1E1 chr3 165743688 0.0081 6.127e-13 35339 TRUE reported textfile
2 1 TRUE
8443 rs10922098 T C T C -0.1029 -0.009 0.40011 NA FALSE FALSE FALSE
GCST006061 0.0075 0.2275 537409 outcome TRUE reported 18276_34_TCL1B_TCL1B
TCL1B chr1 196695521 0.008101 5.73e-37 35338 TRUE reported
textfile 2 1 TRUE
8444 rs1355538 G A G A -0.0476 -0.0076 0.39847 NA FALSE FALSE FALSE
GCST006061 0.0072 0.2923 537409 outcome TRUE reported 18276_34_TCL1B_TCL1B
TCL1B chr3 165787389 0.008103 4.241e-09 35338 TRUE reported
textfile 2 1 TRUE
8445 rs6542680 T C T C -0.0694 -0.0012 0.18362 NA FALSE FALSE FALSE
GCST006061 0.0091 0.8935 537409 outcome TRUE reported 18276_34_TCL1B_TCL1B

TCL1B chr2 3592552 0.010276 1.439e-11 35332 TRUE reported textfile 2 1 TRUE
8446 rs10922098 T C T C -0.1605 -0.009 0.40011 NA FALSE FALSE FALSE GCST006061 0.0075 0.2275 537409 outcome TRUE reported 4128_27_CCL24_Eotaxin_2 CCL24 chr1 196695521 0.008084 1.01e-87 35338 TRUE reported textfile 2 1 TRUE
8447 rs1355538 G A G A -0.0573 -0.0076 0.39847 NA FALSE FALSE FALSE GCST006061 0.0072 0.2923 537409 outcome TRUE reported 4128_27_CCL24_Eotaxin_2 CCL24 chr3 165787389 0.008107 1.571e-12 35338 TRUE reported textfile 2 1 TRUE
8448 rs10922098 T C T C -0.1622 -0.009 0.40011 NA FALSE FALSE FALSE GCST006061 0.0075 0.2275 537409 outcome TRUE reported 8946_38_NR1H4_NR1H4 NR1H4 chr1 196695521 0.008099 3.255e-89 35338 TRUE reported textfile 2 1 TRUE
8449 rs1707652 T C T C 0.1167 0.0081 0.3102 NA FALSE FALSE FALSE GCST006061 0.0074 0.2701 537409 outcome TRUE reported 8946_38_NR1H4_NR1H4 NR1H4 chr3 165761011 0.008572 3.275e-42 35339 TRUE reported textfile 2 1 TRUE
8450 rs10922098 T C T C -0.0589 -0.009 0.40011 NA FALSE FALSE FALSE GCST006061 0.0075 0.2275 537409 outcome TRUE reported 5100_53_SCARB2_LIMP_II SCARB2 chr1 196695521 0.008039 2.35e-13 35338 TRUE reported textfile 2 1 TRUE
8451 rs16881504 T C T C -0.0723 0.0109 0.09636 NA FALSE FALSE FALSE GCST006061 0.0134 0.418 537409 outcome TRUE reported 5100_53_SCARB2_LIMP_II SCARB2 chr6 18935483 0.013214 4.463e-08 35375 TRUE reported textfile 2 1 TRUE
8452 rs6542680 T C T C -0.0671 -0.0012 0.18362 NA FALSE FALSE FALSE GCST006061 0.0091 0.8935 537409 outcome TRUE reported 5100_53_SCARB2_LIMP_II SCARB2 chr2 3592552 0.010186 4.466e-11 35332 TRUE reported textfile 2 1 TRUE
8453 rs10922098 T C T C -0.1366 -0.009 0.40011 NA FALSE FALSE FALSE GCST006061 0.0075 0.2275 537409 outcome TRUE reported 12754_14_CSDC2_CSDC2 CSDC2 chr1 196695521 0.008128 2.176e-63 35329 TRUE reported textfile 2 1 TRUE
8454 rs1555405 A G A G -0.049 -0.0047 0.30381 NA FALSE FALSE FALSE GCST006061 0.0081 0.5592 537409 outcome TRUE reported 12754_14_CSDC2_CSDC2 CSDC2 chr14 100710432 0.008706 1.822e-08 35371 TRUE reported textfile 2 1 TRUE
8455 rs1707652 T C T C 0.0956 0.0081 0.3102 NA FALSE FALSE FALSE GCST006061 0.0074 0.2701 537409 outcome TRUE reported 12754_14_CSDC2_CSDC2 CSDC2 chr3 165761011 0.0086 1.048e-28 35330 TRUE reported textfile 2 1 TRUE
8456 rs7412 T C T C -0.1524 -0.0027 0.05941 NA FALSE FALSE FALSE GCST006061 0.0138 0.8459 537409 outcome TRUE reported 12754_14_CSDC2_CSDC2 CSDC2 chr19 44908822 0.016862 1.596e-19 35358 TRUE reported textfile 2 1 TRUE
8457 rs10922098 T C T C -0.083 -0.009 0.40011 NA FALSE FALSE FALSE GCST006061 0.0075 0.2275 537409 outcome TRUE reported 7878_2_SEC61B_SC61B SEC61B chr1 196695521 0.008048 6.185e-25 35338 TRUE reported textfile 2 1 TRUE
8458 rs10922098 T C T C -0.1445 -0.009 0.40011 NA FALSE FALSE FALSE GCST006061 0.0075 0.2275 537409 outcome TRUE reported 3423_59_CMA1_Chymase CMA1 chr1 196695521 0.00816 3.61e-70 35338 TRUE reported textfile 2 1 TRUE
8459 rs11569415 A G A G -0.0512 0.0011 0.24276 NA FALSE FALSE FALSE GCST006061 0.0097 0.9129 537409 outcome TRUE reported 3423_59_CMA1_Chymase CMA1 chr19 6716268 0.009296 3.63e-08 35372 TRUE reported textfile 2 1 TRUE
8460 rs1706435 G A G A 0.0612 0.0117 0.36981 NA FALSE FALSE FALSE GCST006061 0.0072 0.1047 537409 outcome TRUE reported 3423_59_CMA1_Chymase CMA1 chr3 165743688 0.008291 1.567e-13 35339 TRUE reported textfile 2 1 TRUE
8461 rs10922098 T C T C -0.0849 -0.009 0.40011 NA FALSE FALSE FALSE GCST006061 0.0075 0.2275 537409 outcome TRUE reported 17835_28_ANXA13_ANX13 ANXA13 chr1 196695521 0.008095 9.776e-26 35338 TRUE reported textfile 2 1 TRUE

8462 rs10922098 T C T C -0.1481 -0.009 0.40011 NA FALSE FALSE FALSE
GCST006061 0.0075 0.2275 537409 outcome TRUE reported 7747_47_NDUFB11_NDUBB
NDUFB11 chr1 196695521 0.008187 3.802e-73 35338 TRUE reported
textfile 2 1 TRUE
8463 rs11569415 A G A G -0.0528 0.0011 0.24276 NA FALSE FALSE FALSE
GCST006061 0.0097 0.9129 537409 outcome TRUE reported 7747_47_NDUFB11_NDUBB
NDUFB11 chr19 6716268 0.009329 1.518e-08 35372 TRUE reported textfile
2 1 TRUE
8464 rs12478841 G A G A 0.0629 0.0049 0.21569 NA FALSE FALSE FALSE
GCST006061 0.008 0.5413 537409 outcome TRUE reported 7747_47_NDUFB11_NDUBB
NDUFB11 chr2 27588855 0.009686 8.351e-11 35373 TRUE reported
textfile 2 1 TRUE
8465 rs1555405 A G A G -0.0503 -0.0047 0.30381 NA FALSE FALSE FALSE
GCST006061 0.0081 0.5592 537409 outcome TRUE reported 7747_47_NDUFB11_NDUBB
NDUFB11 chr14 100710432 0.008766 9.574e-09 35380 TRUE reported
textfile 2 1 TRUE
8466 rs1697422 C T C T -0.0481 0.0053 0.48923 NA FALSE FALSE FALSE
GCST006061 0.0072 0.4547 537409 outcome TRUE reported 7747_47_NDUFB11_NDUBB
NDUFB11 chr1 21495404 0.008079 2.626e-09 35374 TRUE reported
textfile 2 1 TRUE
8467 rs1707652 T C T C 0.0707 0.0081 0.3102 NA FALSE FALSE FALSE
GCST006061 0.0074 0.2701 537409 outcome TRUE reported 7747_47_NDUFB11_NDUBB
NDUFB11 chr3 165761011 0.008661 3.26e-16 35339 TRUE reported
textfile 2 1 TRUE
8468 rs6444156 A G A G 0.0547 -0.0028 0.25196 NA FALSE FALSE FALSE
GCST006061 0.0081 0.732 537409 outcome TRUE reported 7747_47_NDUFB11_NDUBB
NDUFB11 chr3 186679685 0.009236 3.177e-09 35368 TRUE reported
textfile 2 1 TRUE
8469 rs704 A G A G 0.0655 -0.0155 0.49189 NA FALSE FALSE FALSE GCST006061
0.0072 0.03144 537409 outcome TRUE reported 7747_47_NDUFB11_NDUBB NDUFB11
chr17 28367840 0.008045 3.891e-16 35360 TRUE reported textfile 2
1 TRUE
8470 rs10922098 T C T C -0.12 -0.009 0.40011 NA FALSE FALSE FALSE
GCST006061 0.0075 0.2275 537409 outcome TRUE reported 13039_1_PPP1R8_PP1R8
PPP1R8 chr1 196695521 0.008151 4.679e-49 35329 TRUE reported
textfile 2 1 TRUE
8471 rs111696008 A G A G -0.165 -0.0259 0.02443 NA FALSE FALSE FALSE
GCST006061 0.0401 0.519 537409 outcome TRUE reported 13039_1_PPP1R8_PP1R8
PPP1R8 chr2 216622986 0.026562 5.234e-10 35350 TRUE reported
textfile 2 1 TRUE
8472 rs483465 G A G A 0.0539 -0.0204 0.24663 NA FALSE FALSE FALSE
GCST006061 0.0083 0.01415 537409 outcome TRUE reported 13039_1_PPP1R8_PP1R8
PPP1R8 chr3 136329135 0.009333 7.679e-09 35352 TRUE reported
textfile 2 1 TRUE
8473 rs10922098 T C T C -0.0648 -0.009 0.40011 NA FALSE FALSE FALSE
GCST006061 0.0075 0.2275 537409 outcome TRUE reported 11654_77_NRSN1_NRSN1
NRSN1 chr1 196695521 0.008018 6.386e-16 35329 TRUE reported
textfile 2 1 TRUE
8474 rs704 A G A G 0.0617 -0.0155 0.49189 NA FALSE FALSE FALSE GCST006061
0.0072 0.03144 537409 outcome TRUE reported 11654_77_NRSN1_NRSN1 NRSN1
chr17 28367840 0.007859 4.134e-15 35351 TRUE reported textfile 2
1 TRUE
8475 rs10922098 T C T C -0.0737 -0.009 0.40011 NA FALSE FALSE FALSE
GCST006061 0.0075 0.2275 537409 outcome TRUE reported 16922_53_PLXNB3_PLXB3
PLXNB3 chr1 196695521 0.008072 6.797e-20 35338 TRUE reported
textfile 2 1 TRUE
8476 rs41446546 T C T C 0.1249 -0.0088 0.03118 NA FALSE FALSE FALSE
GCST006061 0.0262 0.7371 537409 outcome TRUE reported 16922_53_PLXNB3_PLXB3
PLXNB3 chr18 48197233 0.022868 4.713e-08 35366 TRUE reported
textfile 2 1 TRUE
8477 rs9635249 G A G A 0.1221 -0.0086 0.1706 NA FALSE FALSE FALSE
GCST006061 0.0085 0.3087 537409 outcome TRUE reported 16922_53_PLXNB3_PLXB3
PLXNB3 chr14 65331066 0.010464 1.838e-31 35359 TRUE reported
textfile 2 1 TRUE
8478 rs10922098 T C T C -0.1749 -0.009 0.40011 NA FALSE FALSE FALSE
GCST006061 0.0075 0.2275 537409 outcome TRUE reported 7082_2_B3GNT6_B3GN6

B3GNT6 chr1 196695521 0.008157 5.478e-102 35338 TRUE reported
textfile 2 1 TRUE
8479 rs10922098 T C T C -0.0702 -0.009 0.40011 NA FALSE FALSE FALSE
GCST006061 0.0075 0.2275 537409 outcome TRUE reported 9333_59_ERP27_ERP27
ERP27 chr1 196695521 0.007906 6.725e-19 35338 TRUE reported
textfile 2 1 TRUE
8480 rs8176743 T C T C 0.0874 0.017 0.06488 NA FALSE FALSE FALSE
GCST006061 0.0128 0.1834 537409 outcome TRUE reported 9333_59_ERP27_ERP27
ERP27 chr9 133256028 0.015785 3.076e-08 35370 TRUE reported
textfile 2 1 TRUE
8481 rs10922098 T C T C -0.1371 -0.009 0.40011 NA FALSE FALSE FALSE
GCST006061 0.0075 0.2275 537409 outcome TRUE reported 18888_37_FABP12_FBP12
FABP12 chr1 196695521 0.007978 3.413e-66 35338 TRUE reported
textfile 2 1 TRUE
8482 rs1355538 G A G A -0.053 -0.0076 0.39847 NA FALSE FALSE FALSE
GCST006061 0.0072 0.2923 537409 outcome TRUE reported 18888_37_FABP12_FBP12
FABP12 chr3 165787389 0.007985 3.19e-11 35338 TRUE reported
textfile 2 1 TRUE
8483 rs1555405 A G A G -0.0481 -0.0047 0.30381 NA FALSE FALSE FALSE
GCST006061 0.0081 0.5592 537409 outcome TRUE reported 18888_37_FABP12_FBP12
FABP12 chr14 100710432 0.00856 1.921e-08 35380 TRUE reported textfile
2 1 TRUE
8484 rs10922098 T C T C -0.0841 -0.009 0.40011 NA FALSE FALSE FALSE
GCST006061 0.0075 0.2275 537409 outcome TRUE reported 13484_69_COL1A1_CO1A1
COL1A1 chr1 196695521 0.007989 6.445e-26 35332 TRUE reported
textfile 2 1 TRUE
8485 rs10922098 T C T C -0.165 -0.009 0.40011 NA FALSE FALSE FALSE
GCST006061 0.0075 0.2275 537409 outcome TRUE reported 6558_5_COLEC10_COL10
COLEC10 chr1 196695521 0.008173 1.233e-90 35338 TRUE reported
textfile 2 1 TRUE
8486 rs111696008 A G A G -0.1958 -0.0259 0.02443 NA FALSE FALSE FALSE
GCST006061 0.0401 0.519 537409 outcome TRUE reported 6558_5_COLEC10_COL10
COLEC10 chr2 216622986 0.02668 2.155e-13 35359 TRUE reported textfile
2 1 TRUE
8487 rs144202860 A G A G -0.2003 -0.0629 0.01274 NA FALSE FALSE FALSE
GCST006061 0.0538 0.2423 537409 outcome TRUE reported 6558_5_COLEC10_COL10
COLEC10 chr2 216625740 0.035714 2.041e-08 35359 TRUE reported
textfile 2 1 TRUE
8488 rs3820897 C T C T 0.1553 -0.0012 0.18633 NA FALSE FALSE FALSE
GCST006061 0.0091 0.893 537409 outcome TRUE reported 6558_5_COLEC10_COL10
COLEC10 chr2 3594771 0.010316 3.23e-51 35333 TRUE reported textfile
2 1 TRUE
8489 rs6445035 A G A G 0.1759 0.0154 0.1904 NA FALSE FALSE FALSE
GCST006061 0.0089 0.08256 537409 outcome TRUE reported 6558_5_COLEC10_COL10
COLEC10 chr3 165762312 0.010184 7.678e-67 35339 TRUE reported
textfile 2 1 TRUE
8490 rs73169103 T C T C 0.121 0.0178 0.03563 NA FALSE FALSE FALSE
GCST006061 0.0249 0.4756 537409 outcome TRUE reported 6558_5_COLEC10_COL10
COLEC10 chr3 167190675 0.021664 2.332e-08 35354 TRUE reported
textfile 2 1 TRUE
8491 rs7844871 A C A C -0.2447 0.0118 0.01345 NA FALSE FALSE FALSE
GCST006061 0.0388 0.7604 537409 outcome TRUE reported 6558_5_COLEC10_COL10
COLEC10 chr8 119047854 0.034846 2.183e-12 35361 TRUE reported
textfile 2 1 TRUE
8492 rs10922098 T C T C -0.2116 -0.009 0.40011 NA FALSE FALSE FALSE
GCST006061 0.0075 0.2275 537409 outcome TRUE reported 12604_16_SCMH1_SCMH1
SCMH1 chr1 196695521 0.008062 8.011e-152 35329 TRUE reported
textfile 2 1 TRUE
8493 rs1706435 G A G A 0.0592 0.0117 0.36981 NA FALSE FALSE FALSE
GCST006061 0.0072 0.1047 537409 outcome TRUE reported 12604_16_SCMH1_SCMH1
SCMH1 chr3 165743688 0.008217 5.829e-13 35330 TRUE reported
textfile 2 1 TRUE
8494 rs35351085 T C T C 0.1085 0.0061 0.04877 NA FALSE FALSE FALSE
GCST006061 0.022 0.7825 537409 outcome TRUE reported 12604_16_SCMH1_SCMH1
SCMH1 chr1 197992799 0.018341 3.304e-09 35334 TRUE reported
textfile 2 1 TRUE

8495 rs10922098 T C T C -0.1609 -0.009 0.40011 NA FALSE FALSE FALSE GCST006061 0.0075 0.2275 537409 outcome TRUE reported 4563_61_PLCG1_PLCG1 PLCG1 chr1 196695521 0.008107 1.178e-87 35338 TRUE reported textfile 2 1 TRUE
8496 rs11569415 A G A G -0.0649 0.0011 0.24276 NA FALSE FALSE FALSE GCST006061 0.0097 0.9129 537409 outcome TRUE reported 4563_61_PLCG1_PLCG1 PLCG1 chr19 6716268 0.009249 2.27e-12 35372 TRUE reported textfile 2 1 TRUE
8497 rs12478841 G A G A 0.0588 0.0049 0.21569 NA FALSE FALSE FALSE GCST006061 0.008 0.5413 537409 outcome TRUE reported 4563_61_PLCG1_PLCG1 PLCG1 chr2 27588855 0.009591 8.75e-10 35373 TRUE reported textfile 2 1 TRUE
8498 rs1706435 G A G A 0.0668 0.0117 0.36981 NA FALSE FALSE FALSE GCST006061 0.0072 0.1047 537409 outcome TRUE reported 4563_61_PLCG1_PLCG1 PLCG1 chr3 165743688 0.008231 4.849e-16 35339 TRUE reported textfile 2 1 TRUE
8499 rs704 A G A G 0.0549 -0.0155 0.49189 NA FALSE FALSE FALSE GCST006061 0.0072 0.03144 537409 outcome TRUE reported 4563_61_PLCG1_PLCG1 PLCG1 chr17 28367840 0.007981 6.021e-12 35360 TRUE reported textfile 2 1 TRUE
8500 rs753381 C T C T 0.0669 0.013 0.47315 NA FALSE FALSE FALSE GCST006061 0.0074 0.07611 537409 outcome TRUE reported 4563_61_PLCG1_PLCG1 PLCG1 chr20 41168825 0.007994 5.799e-17 35372 TRUE reported textfile 2 1 TRUE
8501 rs10922098 T C T C -0.0466 -0.009 0.40011 NA FALSE FALSE FALSE GCST006061 0.0075 0.2275 537409 outcome TRUE reported 17711_13_VAMP1_VAMP1 VAMP1 chr1 196695521 0.007923 4.054e-09 35338 TRUE reported textfile 2 1 TRUE
8502 rs2523451 A G A G 0.0471 -0.0086 0.30714 NA FALSE FALSE FALSE GCST006061 0.0083 0.297 537409 outcome TRUE reported 17711_13_VAMP1_VAMP1 VAMP1 chr6 31401374 0.008403 2.08e-08 35439 TRUE reported textfile 2 1 TRUE
8503 rs10922098 T C T C -0.1031 -0.009 0.40011 NA FALSE FALSE FALSE GCST006061 0.0075 0.2275 537409 outcome TRUE reported 6936_7_PMEPA1_PMEPA PMEPA1 chr1 196695521 0.008167 1.555e-36 35338 TRUE reported textfile 2 1 TRUE
8504 rs10922098 T C T C -0.0715 -0.009 0.40011 NA FALSE FALSE FALSE GCST006061 0.0075 0.2275 537409 outcome TRUE reported 8045_3_KIR3DL3_KI3L3 KIR3DL3 chr1 196695521 0.007963 2.739e-19 35338 TRUE reported textfile 2 1 TRUE
8505 rs1706435 G A G A 0.047 0.0117 0.36981 NA FALSE FALSE FALSE GCST006061 0.0072 0.1047 537409 outcome TRUE reported 8045_3_KIR3DL3_KI3L3 KIR3DL3 chr3 165743688 0.008067 5.666e-09 35339 TRUE reported textfile 2 1 TRUE
8506 rs2317230 T G T G 0.0473 -0.0057 0.39826 NA FALSE FALSE FALSE GCST006061 0.0072 0.4268 537409 outcome TRUE reported 8045_3_KIR3DL3_KI3L3 KIR3DL3 chr1 157705207 0.007934 2.501e-09 35365 TRUE reported textfile 2 1 TRUE
8507 rs55650306 T C T C -0.0449 -0.016 0.35183 NA FALSE FALSE FALSE GCST006061 0.0078 0.04027 537409 outcome TRUE reported 8045_3_KIR3DL3_KI3L3 KIR3DL3 chr3 152500769 0.008159 3.729e-08 35348 TRUE reported textfile 2 1 TRUE
8508 rs10922098 T C T C -0.1232 -0.009 0.40011 NA FALSE FALSE FALSE GCST006061 0.0075 0.2275 537409 outcome TRUE reported 12400_25_UBE2T_UBE2T UBE2T chr1 196695521 0.00809 2.278e-52 35329 TRUE reported textfile 2 1 TRUE
8509 rs10922098 T C T C -0.144 -0.009 0.40011 NA FALSE FALSE FALSE GCST006061 0.0075 0.2275 537409 outcome TRUE reported 4480_59_C3_C3b C3 chr1 196695521 0.00827 6.671e-68 35338 TRUE reported textfile 2 1 TRUE
8510 rs10922098 T C T C -0.1685 -0.009 0.40011 NA FALSE FALSE FALSE GCST006061 0.0075 0.2275 537409 outcome TRUE reported 9092_33_ANGPTL1_ANGL1 ANGPTL1 chr1 196695521 0.008152 6.6e-95 35338 TRUE reported textfile 2 1 TRUE
8511 rs10929302 A G A G 0.0699 4e-04 0.29541 NA FALSE FALSE FALSE GCST006061 0.0079 0.9593 537409 outcome TRUE reported 9092_33_ANGPTL1_ANGL1 ANGPTL1 chr2 233757136 0.008795 1.905e-15 35373 TRUE reported

textfile 2 1 TRUE
8512 rs11581618 A G A G 0.0535 -0.0123 0.38099 NA FALSE FALSE FALSE
GCST006061 0.0074 0.09827 537409 outcome TRUE reported 9092_33_ANGPTL1_ANGL1
ANGPTL1 chr1 178603585 0.00825 8.87e-11 35357 TRUE reported textfile
2 1 TRUE
8513 rs117516437 T C T C 0.1655 0.0362 0.03677 NA FALSE FALSE FALSE
GCST006061 0.0284 0.2031 537409 outcome TRUE reported 9092_33_ANGPTL1_ANGL1
ANGPTL1 chr19 10115903 0.021296 7.771e-15 35375 TRUE reported
textfile 2 1 TRUE
8514 rs2686395 C T C T -0.075 -0.0077 0.3101 NA FALSE FALSE FALSE
GCST006061 0.0074 0.2965 537409 outcome TRUE reported 9092_33_ANGPTL1_ANGL1
ANGPTL1 chr3 165767231 0.008646 4.152e-18 35339 TRUE reported
textfile 2 1 TRUE
8515 rs71630204 C T C T 0.0743 -0.0039 0.38219 NA FALSE FALSE FALSE
GCST006061 0.0077 0.6173 537409 outcome TRUE reported 9092_33_ANGPTL1_ANGL1
ANGPTL1 chr1 178928183 0.008224 1.642e-19 35357 TRUE reported
textfile 2 1 TRUE
8516 rs10922098 T C T C -0.0476 -0.009 0.40011 NA FALSE FALSE FALSE
GCST006061 0.0075 0.2275 537409 outcome TRUE reported 2985_35_CXCL1_Gro_a
CXCL1 chr1 196695521 0.008032 3.092e-09 35338 TRUE reported
textfile 2 1 TRUE
8517 rs114087287 A G A G 0.2328 0.0358 0.01319 NA FALSE FALSE FALSE
GCST006061 0.0476 0.4521 537409 outcome TRUE reported 2985_35_CXCL1_Gro_a
CXCL1 chr4 79532875 0.035662 6.67e-11 35354 TRUE reported
textfile 2 1 TRUE
8518 rs114448914 C T C T 0.3245 -0.0722 0.01323 NA FALSE FALSE FALSE
GCST006061 0.0588 0.2193 537409 outcome TRUE reported 2985_35_CXCL1_Gro_a
CXCL1 chr4 72297546 0.036362 4.489e-19 35350 TRUE reported
textfile 2 1 TRUE
8519 rs115214168 T C T C 1.1245 -0.006 0.01277 NA FALSE FALSE FALSE
GCST006061 0.027 0.8237 537409 outcome TRUE reported 2985_35_CXCL1_Gro_a
CXCL1 chr4 73832401 0.03691 1e-200 35351 TRUE reported textfile 2
1 TRUE
8520 rs11944104 C A C A 0.5807 -0.0016 0.01736 NA FALSE FALSE FALSE
GCST006061 0.0279 0.9554 537409 outcome TRUE reported 2985_35_CXCL1_Gro_a
CXCL1 chr4 73846576 0.032049 2.26e-73 35350 TRUE reported
textfile 2 1 TRUE
8521 rs140569632 A G A G 0.8229 -0.0649 0.01187 NA FALSE FALSE FALSE
GCST006061 0.0347 0.06153 537409 outcome TRUE reported 2985_35_CXCL1_Gro_a
CXCL1 chr4 74137475 0.038165 4.14e-103 35351 TRUE reported
textfile 2 1 TRUE
8522 rs144828812 C T C T 0.2981 -0.0028 0.01192 NA FALSE FALSE FALSE
GCST006061 0.0437 0.9486 537409 outcome TRUE reported 2985_35_CXCL1_Gro_a
CXCL1 chr4 71550144 0.0391 2.457e-14 35351 TRUE reported textfile
2 1 TRUE
8523 rs151107944 A G A G 0.1926 -0.023 0.01405 NA FALSE FALSE FALSE
GCST006061 0.0302 0.4475 537409 outcome TRUE reported 2985_35_CXCL1_Gro_a
CXCL1 chr4 61259516 0.034152 1.705e-08 35349 TRUE reported
textfile 2 1 TRUE
8524 rs16845887 G A G A 0.252 -4.00E-04 0.02774 NA FALSE FALSE FALSE
GCST006061 0.0332 0.9893 537409 outcome TRUE reported 2985_35_CXCL1_Gro_a
CXCL1 chr4 71140106 0.025666 9.389e-23 35347 TRUE reported
textfile 2 1 TRUE
8525 rs62309626 G A G A -0.1857 -0.0237 0.15176 NA FALSE FALSE FALSE
GCST006061 0.0102 0.02067 537409 outcome TRUE reported 2985_35_CXCL1_Gro_a
CXCL1 chr4 72998190 0.011518 1.789e-58 35355 TRUE reported
textfile 2 1 TRUE
8526 rs72651015 G T G T -0.1813 -0.0115 0.03011 NA FALSE FALSE FALSE
GCST006061 0.0332 0.7298 537409 outcome TRUE reported 2985_35_CXCL1_Gro_a
CXCL1 chr4 71921296 0.023702 2.023e-14 35352 TRUE reported
textfile 2 1 TRUE
8527 rs900792 G T G T -0.1892 -0.0079 0.01959 NA FALSE FALSE FALSE
GCST006061 0.0213 0.7091 537409 outcome TRUE reported 2985_35_CXCL1_Gro_a
CXCL1 chr4 67604887 0.029701 1.889e-10 35350 TRUE reported
textfile 2 1 TRUE
8528 rs954645 C T C T 0.0556 -0.019 0.2086 NA FALSE FALSE FALSE

GCST006061 0.0083 0.0221 537409 outcome TRUE reported 2985_35_CXCL1_Gro_a
CXCL1 chr4 61993793 0.010159 4.422e-08 35349 TRUE reported
textfile 2 1 TRUE
8529 rs10922098 T C T C -0.1359 -0.009 0.40011 NA FALSE FALSE FALSE
GCST006061 0.0075 0.2275 537409 outcome TRUE reported
2632_5_IL12RB1_IL_12_Rb1 IL12RB1 chr1 196695521 0.008051 6.37e-64 35338
TRUE reported textfile 2 1 TRUE
8530 rs1707652 T C T C 0.0528 0.0081 0.3102 NA FALSE FALSE FALSE
GCST006061 0.0074 0.2701 537409 outcome TRUE reported
2632_5_IL12RB1_IL_12_Rb1 IL12RB1 chr3 165761011 0.008526 5.911e-10 35339
TRUE reported textfile 2 1 TRUE
8531 rs35209896 A C A C 0.081 0.0267 0.09263 NA FALSE FALSE FALSE
GCST006061 0.0355 0.4521 537409 outcome TRUE reported
2632_5_IL12RB1_IL_12_Rb1 IL12RB1 chr6 32519009 0.013507 2.014e-09 35439
TRUE reported textfile 2 1 TRUE
8532 rs429774 C T C T -0.0791 -0.0068 0.30493 NA FALSE FALSE FALSE
GCST006061 0.0076 0.3717 537409 outcome TRUE reported
2632_5_IL12RB1_IL_12_Rb1 IL12RB1 chr19 18075942 0.008594 3.442e-20 35351
TRUE reported textfile 2 1 TRUE
8533 rs10922098 T C T C -0.0657 -0.009 0.40011 NA FALSE FALSE FALSE
GCST006061 0.0075 0.2275 537409 outcome TRUE reported
4769_10_OLFM4_Olfactomedin_4 OLFM4 chr1 196695521 0.00801 2.358e-16 35338
TRUE reported textfile 2 1 TRUE
8534 rs10922098 T C T C -0.1089 -0.009 0.40011 NA FALSE FALSE FALSE
GCST006061 0.0075 0.2275 537409 outcome TRUE reported 12425_104_ARF6_ARF6
ARF6 chr1 196695521 0.0081 3.286e-41 35329 TRUE reported textfile
2 1 TRUE
8535 rs6445035 A G A G 0.118 0.0154 0.1904 NA FALSE FALSE FALSE
GCST006061 0.0089 0.08256 537409 outcome TRUE reported 12425_104_ARF6_ARF6
ARF6 chr3 165762312 0.010073 1.075e-31 35330 TRUE reported
textfile 2 1 TRUE
8536 rs704 A G A G 0.0486 -0.0155 0.49189 NA FALSE FALSE FALSE GCST006061
0.0072 0.03144 537409 outcome TRUE reported 12425_104_ARF6_ARF6 ARF6 chr17
28367840 0.007945 9.543e-10 35351 TRUE reported textfile 2 1 TRUE
8537 rs10922098 T C T C -0.0824 -0.009 0.40011 NA FALSE FALSE FALSE
GCST006061 0.0075 0.2275 537409 outcome TRUE reported
5017_19_PRDX5_Peroxiredoxin_5 PRDX5 chr1 196695521 0.008038 1.171e-24
35338 TRUE reported textfile 2 1 TRUE
8538 rs10922098 T C T C -0.218 -0.009 0.40011 NA FALSE FALSE FALSE
GCST006061 0.0075 0.2275 537409 outcome TRUE reported 2635_61_LAYN_Layilin
LAYN chr1 196695521 0.008164 4.3e-157 35338 TRUE reported
textfile 2 1 TRUE
8539 rs11569415 A G A G -0.0751 0.0011 0.24276 NA FALSE FALSE FALSE
GCST006061 0.0097 0.9129 537409 outcome TRUE reported 2635_61_LAYN_Layilin
LAYN chr19 6716268 0.009332 8.466e-16 35372 TRUE reported textfile
2 1 TRUE
8540 rs1451915 A C A C 0.0668 -0.0107 0.13641 NA FALSE FALSE FALSE
GCST006061 0.0124 0.3895 537409 outcome TRUE reported 2635_61_LAYN_Layilin
LAYN chr1 196058002 0.011672 1.046e-08 35335 TRUE reported
textfile 2 1 TRUE
8541 rs2686395 C T C T -0.1013 -0.0077 0.3101 NA FALSE FALSE FALSE
GCST006061 0.0074 0.2965 537409 outcome TRUE reported 2635_61_LAYN_Layilin
LAYN chr3 165767231 0.008668 1.484e-31 35339 TRUE reported
textfile 2 1 TRUE
8542 rs4938792 C T C T -0.0523 -0.0016 0.37645 NA FALSE FALSE FALSE
GCST006061 0.0075 0.8329 537409 outcome TRUE reported 2635_61_LAYN_Layilin
LAYN chr11 111550211 0.008322 3.294e-10 35364 TRUE reported
textfile 2 1 TRUE
8543 rs7513100 C T C T 0.0635 0 0.14871 NA FALSE FALSE FALSE GCST006061
0.0098 0.9981 537409 outcome TRUE reported 2635_61_LAYN_Layilin LAYN
chr1 198065935 0.011282 1.816e-08 35343 TRUE reported textfile 2
1 TRUE
8544 rs10922098 T C T C -0.1467 -0.009 0.40011 NA FALSE FALSE FALSE
GCST006061 0.0075 0.2275 537409 outcome TRUE reported 9819_110_NUP210_PO210
NUP210 chr1 196695521 0.00805 3.295e-74 35338 TRUE reported textfile
2 1 TRUE

8545 rs1355538 G A G A -0.0671 -0.0076 0.39847 NA FALSE FALSE FALSE GCST006061 0.0072 0.2923 537409 outcome TRUE reported 9819_110_NUP210_PO210 NUP210 chr3 165787389 0.008056 8.144e-17 35338 TRUE reported textfile 2 1 TRUE
8546 rs10922098 T C T C -0.045 -0.009 0.40011 NA FALSE FALSE FALSE GCST006061 0.0075 0.2275 537409 outcome TRUE reported 10015_119_KCNAB2_KCAB2 KCNAB2 chr1 196695521 0.008042 2.199e-08 35326 TRUE reported textfile 2 1 TRUE
8547 rs806109 A G A G 0.08 -0.0054 0.21513 NA FALSE FALSE FALSE GCST006061 0.0089 0.5444 537409 outcome TRUE reported 10015_119_KCNAB2_KCAB2 KCNAB2 chr1 5992521 0.009576 6.573e-17 35372 TRUE reported textfile 2 1 TRUE
8548 rs10922098 T C T C -0.1544 -0.009 0.40011 NA FALSE FALSE FALSE GCST006061 0.0075 0.2275 537409 outcome TRUE reported 6545_58_PRNP_PRIO PRNP chr1 196695521 0.008134 2.394e-80 35338 TRUE reported textfile 2 1 TRUE
8549 rs1707652 T C T C 0.0747 0.0081 0.3102 NA FALSE FALSE FALSE GCST006061 0.0074 0.2701 537409 outcome TRUE reported 6545_58_PRNP_PRIO PRNP chr3 165761011 0.008606 3.96e-18 35339 TRUE reported textfile 2 1 TRUE
8550 rs10922098 T C T C -0.0472 -0.009 0.40011 NA FALSE FALSE FALSE GCST006061 0.0075 0.2275 537409 outcome TRUE reported 8631_13_ERMAP_ERMAP ERMAP chr1 196695521 0.008052 4.582e-09 35338 TRUE reported textfile 2 1 TRUE
8551 rs2057681 G A G A 0.1863 -0.0136 0.28541 NA FALSE FALSE FALSE GCST006061 0.0078 0.07965 537409 outcome TRUE reported 8631_13_ERMAP_ERMAP ERMAP chr7 95308945 0.008732 5.239e-101 35349 TRUE reported textfile 2 1 TRUE
8552 rs10922098 T C T C -0.1049 -0.009 0.40011 NA FALSE FALSE FALSE GCST006061 0.0075 0.2275 537409 outcome TRUE reported 11094_104_CLC_LPPL CLC chr1 196695521 0.008048 7.727e-39 35329 TRUE reported textfile 2 1 TRUE
8553 rs10922098 T C T C -0.0914 -0.009 0.40011 NA FALSE FALSE FALSE GCST006061 0.0075 0.2275 537409 outcome TRUE reported 7943_16_CUZD1_CUZD1 CUZD1 chr1 196695521 0.008148 3.325e-29 35338 TRUE reported textfile 2 1 TRUE
8554 rs1707652 T C T C 0.0816 0.0081 0.3102 NA FALSE FALSE FALSE GCST006061 0.0074 0.2701 537409 outcome TRUE reported 7943_16_CUZD1_CUZD1 CUZD1 chr3 165761011 0.008607 2.54e-21 35339 TRUE reported textfile 2 1 TRUE
8555 rs429358 C T C T 0.1479 0.005 0.17407 NA FALSE FALSE FALSE GCST006061 0.0103 0.6319 537409 outcome TRUE reported 7943_16_CUZD1_CUZD1 CUZD1 chr19 44908684 0.010621 4.462e-44 35368 TRUE reported textfile 2 1 TRUE
8556 rs74607435 C T C T -0.097 0.0047 0.06148 NA FALSE FALSE FALSE GCST006061 0.0193 0.8088 537409 outcome TRUE reported 7943_16_CUZD1_CUZD1 CUZD1 chr19 44732440 0.016772 7.312e-09 35369 TRUE reported textfile 2 1 TRUE
8557 rs10922098 T C T C -0.1205 -0.009 0.40011 NA FALSE FALSE FALSE GCST006061 0.0075 0.2275 537409 outcome TRUE reported 4862_63_BMPR2_BMP_RII BMPR2 chr1 196695521 0.008038 8.267e-51 35338 TRUE reported textfile 2 1 TRUE
8558 rs1706435 G A G A 0.0781 0.0117 0.36981 NA FALSE FALSE FALSE GCST006061 0.0072 0.1047 537409 outcome TRUE reported 4862_63_BMPR2_BMP_RII BMPR2 chr3 165743688 0.008151 9.57e-22 35339 TRUE reported textfile 2 1 TRUE
8559 rs10922098 T C T C -0.0548 -0.009 0.40011 NA FALSE FALSE FALSE GCST006061 0.0075 0.2275 537409 outcome TRUE reported 7140_1_CELA2A_ELA2A CELA2A chr1 196695521 0.008473 9.968e-11 35338 TRUE reported textfile 2 1 TRUE
8560 rs17032925 C T C T -0.1485 -9.00E-04 0.08221 NA FALSE FALSE FALSE GCST006061 0.0134 0.9475 537409 outcome TRUE reported 7140_1_CELA2A_ELA2A CELA2A chr2 67039351 0.015087 7.333e-23 35359 TRUE reported textfile 2 1 TRUE
8561 rs28365941 C T C T -0.0963 -0.0107 0.10449 NA FALSE FALSE FALSE GCST006061 0.0114 0.3499 537409 outcome TRUE reported 7140_1_CELA2A_ELA2A

CELA2A chr16 283138 0.013691 2.013e-12 35313 TRUE reported textfile
2 1 TRUE
8562 rs533406 G A G A -0.1302 -0.0177 0.41964 NA FALSE FALSE FALSE
GCST006061 0.0075 0.01777 537409 outcome TRUE reported 7140_1_CELA2A_ELA2A
CELA2A chr16 88908452 0.0084 3.448e-54 35321 TRUE reported textfile
2 1 TRUE
8563 rs6812832 G A G A 0.0582 -0.0042 0.48044 NA FALSE FALSE FALSE
GCST006061 0.0073 0.5637 537409 outcome TRUE reported 7140_1_CELA2A_ELA2A
CELA2A chr4 40336703 0.008291 2.225e-12 35362 TRUE reported
textfile 2 1 TRUE
8564 rs7023365 T C T C 0.0638 0.0032 0.34392 NA FALSE FALSE FALSE
GCST006061 0.0096 0.7361 537409 outcome TRUE reported 7140_1_CELA2A_ELA2A
CELA2A chr9 133097020 0.008715 2.471e-13 35372 TRUE reported
textfile 2 1 TRUE
8565 rs75593565 G A G A -0.2719 -0.0252 0.03282 NA FALSE FALSE FALSE
GCST006061 0.0283 0.3743 537409 outcome TRUE reported 7140_1_CELA2A_ELA2A
CELA2A chr12 31962332 0.023092 5.259e-32 35358 TRUE reported
textfile 2 1 TRUE
8566 rs77643237 A G A G 0.1823 -0.0051 0.0815 NA FALSE FALSE FALSE
GCST006061 0.0175 0.7705 537409 outcome TRUE reported 7140_1_CELA2A_ELA2A
CELA2A chr9 133013823 0.015127 1.903e-33 35368 TRUE reported
textfile 2 1 TRUE
8567 rs78880003 T C T C -0.2027 0.0028 0.06114 NA FALSE FALSE FALSE
GCST006061 0.0163 0.8627 537409 outcome TRUE reported 7140_1_CELA2A_ELA2A
CELA2A chr1 15414434 0.017514 5.619e-31 35382 TRUE reported
textfile 2 1 TRUE
8568 rs9482770 C T C T 0.0548 0.002 0.47412 NA FALSE FALSE FALSE
GCST006061 0.0071 0.7769 537409 outcome TRUE reported 7140_1_CELA2A_ELA2A
CELA2A chr6 127121947 0.008297 3.987e-11 35355 TRUE reported
textfile 2 1 TRUE
8569 rs10922098 T C T C -0.0754 -0.009 0.40011 NA FALSE FALSE FALSE
GCST006061 0.0075 0.2275 537409 outcome TRUE reported 9719_145_MMP16_MMP_16
MMP16 chr1 196695521 0.008055 7.887e-21 35338 TRUE reported
textfile 2 1 TRUE
8570 rs1355538 G A G A -0.0461 -0.0076 0.39847 NA FALSE FALSE FALSE
GCST006061 0.0072 0.2923 537409 outcome TRUE reported 9719_145_MMP16_MMP_16
MMP16 chr3 165787389 0.008042 9.895e-09 35338 TRUE reported
textfile 2 1 TRUE
8571 rs35889496 G A G A -0.0699 -0.0167 0.25554 NA FALSE FALSE FALSE
GCST006061 0.0079 0.0349 537409 outcome TRUE reported 9719_145_MMP16_MMP_16
MMP16 chr8 88322681 0.009079 1.367e-14 35349 TRUE reported
textfile 2 1 TRUE
8572 rs58205855 G A G A -0.0509 -0.001 0.23678 NA FALSE FALSE FALSE
GCST006061 0.0085 0.9034 537409 outcome TRUE reported 9719_145_MMP16_MMP_16
MMP16 chr14 25035454 0.009296 4.368e-08 35377 TRUE reported
textfile 2 1 TRUE
8573 rs10922098 T C T C -0.0829 -0.009 0.40011 NA FALSE FALSE FALSE
GCST006061 0.0075 0.2275 537409 outcome TRUE reported 18887_7_ENTPD2_ENTP2
ENTPD2 chr1 196695521 0.007946 1.759e-25 35338 TRUE reported
textfile 2 1 TRUE
8574 rs10922098 T C T C -0.0442 -0.009 0.40011 NA FALSE FALSE FALSE
GCST006061 0.0075 0.2275 537409 outcome TRUE reported 7853_19_SCO1_SCO1
SCO1 chr1 196695521 0.007966 2.88e-08 35338 TRUE reported
textfile 2 1 TRUE
8575 rs150241543 A G A G -0.224 -0.0503 0.01289 NA FALSE FALSE FALSE
GCST006061 0.0343 0.1429 537409 outcome TRUE reported 7853_19_SCO1_SCO1
SCO1 chr4 70405192 0.033725 3.095e-11 35359 TRUE reported
textfile 2 1 TRUE
8576 rs10922098 T C T C -0.0641 -0.009 0.40011 NA FALSE FALSE FALSE
GCST006061 0.0075 0.2275 537409 outcome TRUE reported 12510_3_STAP1_STAP1
STAP1 chr1 196695521 0.008173 4.387e-15 35329 TRUE reported
textfile 2 1 TRUE
8577 rs1355538 G A G A -0.0728 -0.0076 0.39847 NA FALSE FALSE FALSE
GCST006061 0.0072 0.2923 537409 outcome TRUE reported 12510_3_STAP1_STAP1
STAP1 chr3 165787389 0.008162 4.691e-19 35329 TRUE reported
textfile 2 1 TRUE

8578 rs16849384 C T C T 0.0634 -0.0051 0.37488 NA FALSE FALSE FALSE
GCST006061 0.0072 0.477 537409 outcome TRUE reported 12510_3_STAP1_STAP1
STAP1 chr4 73437153 0.008298 2.163e-14 35345 TRUE reported
textfile 2 1 TRUE
8579 rs2232613 T C T C -0.2132 0.0151 0.0989 NA FALSE FALSE FALSE
GCST006061 0.0141 0.2834 537409 outcome TRUE reported 12510_3_STAP1_STAP1
STAP1 chr20 38369011 0.013174 6.609e-59 35366 TRUE reported
textfile 2 1 TRUE
8580 rs10922098 T C T C -0.1258 -0.009 0.40011 NA FALSE FALSE FALSE
GCST006061 0.0075 0.2275 537409 outcome TRUE reported 14662_6_MZF1_MZF1
MZF1 chr1 196695521 0.008299 6.664e-52 35338 TRUE reported
textfile 2 1 TRUE
8581 rs11607339 C T C T 0.0842 -0.0076 0.10687 NA FALSE FALSE FALSE
GCST006061 0.0108 0.4833 537409 outcome TRUE reported 14662_6_MZF1_MZF1
MZF1 chr11 5996951 0.013242 2.034e-10 35379 TRUE reported textfile
2 1 TRUE
8582 rs117983753 G A G A 0.2309 -0.059 0.01454 NA FALSE FALSE FALSE
GCST006061 0.0424 0.1642 537409 outcome TRUE reported 14662_6_MZF1_MZF1
MZF1 chr11 6989764 0.03375 7.836e-12 35380 TRUE reported textfile 2
1 TRUE
8583 rs13019325 G A G A -0.0576 -0.0075 0.44984 NA FALSE FALSE FALSE
GCST006061 0.0071 0.2908 537409 outcome TRUE reported 14662_6_MZF1_MZF1
MZF1 chr2 159772479 0.008168 1.762e-12 35350 TRUE reported
textfile 2 1 TRUE
8584 rs1707652 T C T C 0.1205 0.0081 0.3102 NA FALSE FALSE FALSE
GCST006061 0.0074 0.2701 537409 outcome TRUE reported 14662_6_MZF1_MZF1
MZF1 chr3 165761011 0.008769 5.76e-43 35339 TRUE reported
textfile 2 1 TRUE
8585 rs2035675 G A G A 0.1327 0.0105 0.26609 NA FALSE FALSE FALSE
GCST006061 0.0087 0.2283 537409 outcome TRUE reported 14662_6_MZF1_MZF1
MZF1 chr11 6441858 0.009201 3.78e-47 35375 TRUE reported textfile
2 1 TRUE
8586 rs10922098 T C T C -0.0993 -0.009 0.40011 NA FALSE FALSE FALSE
GCST006061 0.0075 0.2275 537409 outcome TRUE reported 19227_18_BATF_BATF
BATF chr1 196695521 0.008023 3.47e-35 35338 TRUE reported
textfile 2 1 TRUE
8587 rs115341303 A G A G -0.1473 -0.0335 0.02225 NA FALSE FALSE FALSE
GCST006061 0.0259 0.1953 537409 outcome TRUE reported 19227_18_BATF_BATF
BATF chr4 152257448 0.026891 4.312e-08 35374 TRUE reported
textfile 2 1 TRUE
8588 rs35209896 A C A C 0.0736 0.0267 0.09263 NA FALSE FALSE FALSE
GCST006061 0.0355 0.4521 537409 outcome TRUE reported 19227_18_BATF_BATF
BATF chr6 32519009 0.013449 4.435e-08 35439 TRUE reported
textfile 2 1 TRUE
8589 rs6445035 A G A G 0.079 0.0154 0.1904 NA FALSE FALSE FALSE
GCST006061 0.0089 0.08256 537409 outcome TRUE reported 19227_18_BATF_BATF
BATF chr3 165762312 0.009986 2.552e-15 35339 TRUE reported
textfile 2 1 TRUE
8590 rs6542680 T C T C -0.0618 -0.0012 0.18362 NA FALSE FALSE FALSE
GCST006061 0.0091 0.8935 537409 outcome TRUE reported 19227_18_BATF_BATF
BATF chr2 3592552 0.010181 1.279e-09 35332 TRUE reported textfile
2 1 TRUE
8591 rs10922098 T C T C -0.0848 -0.009 0.40011 NA FALSE FALSE FALSE
GCST006061 0.0075 0.2275 537409 outcome TRUE reported 12795_2_ZNF566_ZN566
ZNF566 chr1 196695521 0.008093 1.095e-25 35329 TRUE reported
textfile 2 1 TRUE
8592 rs1355538 G A G A -0.0493 -0.0076 0.39847 NA FALSE FALSE FALSE
GCST006061 0.0072 0.2923 537409 outcome TRUE reported 12795_2_ZNF566_ZN566
ZNF566 chr3 165787389 0.008084 1.071e-09 35329 TRUE reported
textfile 2 1 TRUE
8593 rs10922098 T C T C -0.1835 -0.009 0.40011 NA FALSE FALSE FALSE
GCST006061 0.0075 0.2275 537409 outcome TRUE reported 17710_40_TPD52L1_TPD53
TPD52L1 chr1 196695521 0.008055 6.908e-115 35338 TRUE reported
textfile 2 1 TRUE
8594 rs2686395 C T C T -0.0779 -0.0077 0.3101 NA FALSE FALSE FALSE
GCST006061 0.0074 0.2965 537409 outcome TRUE reported 17710_40_TPD52L1_TPD53

TPD52L1 chr3 165767231 0.008538 7.243e-20 35339 TRUE reported
textfile 2 1 TRUE
8595 rs10922098 T C T C -0.2098 -0.009 0.40011 NA FALSE FALSE FALSE
GCST006061 0.0075 0.2275 537409 outcome TRUE reported 4562_1_NXPH1_NXPH1
NXPH1 chr1 196695521 0.008243 6.96e-143 35338 TRUE reported
textfile 2 1 TRUE
8596 rs116898372 A G A G -0.215 0.0721 0.01173 NA FALSE FALSE FALSE
GCST006061 0.0395 0.06826 537409 outcome TRUE reported 4562_1_NXPH1_NXPH1
NXPH1 chr22 39278271 0.038466 2.279e-08 35361 TRUE reported
textfile 2 1 TRUE
8597 rs12085435 A G A G 0.1707 -0.0136 0.05177 NA FALSE FALSE FALSE
GCST006061 0.017 0.4252 537409 outcome TRUE reported 4562_1_NXPH1_NXPH1
NXPH1 chr1 56949637 0.01875 8.702e-20 35371 TRUE reported textfile
2 1 TRUE
8598 rs13412535 A G A G -0.2009 -0.0072 0.19379 NA FALSE FALSE FALSE
GCST006061 0.0091 0.43 537409 outcome TRUE reported 4562_1_NXPH1_NXPH1
NXPH1 chr2 224010157 0.01021 3.422e-86 35366 TRUE reported textfile
2 1 TRUE
8599 rs1552948 T C T C -0.0886 0.0121 0.25513 NA FALSE FALSE FALSE
GCST006061 0.0078 0.1205 537409 outcome TRUE reported 4562_1_NXPH1_NXPH1
NXPH1 chr15 101453784 0.009332 2.215e-21 35307 TRUE reported
textfile 2 1 TRUE
8600 rs2686395 C T C T -0.0817 -0.0077 0.3101 NA FALSE FALSE FALSE
GCST006061 0.0074 0.2965 537409 outcome TRUE reported 4562_1_NXPH1_NXPH1
NXPH1 chr3 165767231 0.008738 8.78e-21 35339 TRUE reported
textfile 2 1 TRUE
8601 rs587255 G A G A 0.1032 0.0106 0.31019 NA FALSE FALSE FALSE
GCST006061 0.0075 0.1595 537409 outcome TRUE reported 4562_1_NXPH1_NXPH1
NXPH1 chr1 56924756 0.008734 3.241e-32 35369 TRUE reported
textfile 2 1 TRUE
8602 rs10922098 T C T C -0.1096 -0.009 0.40011 NA FALSE FALSE FALSE
GCST006061 0.0075 0.2275 537409 outcome TRUE reported
9412_52_ACVR2B_Activin_RIIB ACVR2B chr1 196695521 0.008101 1.049e-41 35338
TRUE reported textfile 2 1 TRUE
8603 rs6542680 T C T C -0.0586 -0.0012 0.18362 NA FALSE FALSE FALSE
GCST006061 0.0091 0.8935 537409 outcome TRUE reported
9412_52_ACVR2B_Activin_RIIB ACVR2B chr2 3592552 0.010281 1.198e-08 35332
TRUE reported textfile 2 1 TRUE
8604 rs10922098 T C T C -0.2222 -0.009 0.40011 NA FALSE FALSE FALSE
GCST006061 0.0075 0.2275 537409 outcome TRUE reported 2695_25_PECAM1_PECAM_1
PECAM1 chr1 196695521 0.008177 1.298e-162 35338 TRUE reported
textfile 2 1 TRUE
8605 rs11569415 A G A G -0.057 0.0011 0.24276 NA FALSE FALSE FALSE
GCST006061 0.0097 0.9129 537409 outcome TRUE reported 2695_25_PECAM1_PECAM_1
PECAM1 chr19 6716268 0.009356 1.114e-09 35372 TRUE reported textfile
2 1 TRUE
8606 rs2686395 C T C T -0.0984 -0.0077 0.3101 NA FALSE FALSE FALSE
GCST006061 0.0074 0.2965 537409 outcome TRUE reported 2695_25_PECAM1_PECAM_1
PECAM1 chr3 165767231 0.00868 8.708e-30 35339 TRUE reported textfile
2 1 TRUE
8607 rs10922098 T C T C -0.0658 -0.009 0.40011 NA FALSE FALSE FALSE
GCST006061 0.0075 0.2275 537409 outcome TRUE reported 7779_86_CHST11_CHSTB
CHST11 chr1 196695521 0.008352 3.326e-15 35338 TRUE reported
textfile 2 1 TRUE
8608 rs11216161 C T C T -0.0812 0.003 0.20203 NA FALSE FALSE FALSE
GCST006061 0.0093 0.7434 537409 outcome TRUE reported 7779_86_CHST11_CHSTB
CHST11 chr11 116850485 0.010177 1.476e-15 35377 TRUE reported
textfile 2 1 TRUE
8609 rs146776981 A G A G 0.2027 0.0267 0.02099 NA FALSE FALSE FALSE
GCST006061 0.0368 0.469 537409 outcome TRUE reported 7779_86_CHST11_CHSTB
CHST11 chr12 104618354 0.028727 1.713e-12 35356 TRUE reported
textfile 2 1 TRUE
8610 rs1650127 T C T C -0.2756 0.0165 0.31498 NA FALSE FALSE FALSE
GCST006061 0.0078 0.03425 537409 outcome TRUE reported 7779_86_CHST11_CHSTB
CHST11 chr12 104575785 0.008767 1e-200 35360 TRUE reported textfile
2 1 TRUE

8611 rs1983127 T G T G -0.0504 -0.0056 0.32014 NA FALSE FALSE FALSE GCST006061 0.0077 0.4685 537409 outcome TRUE reported 7779_86_CHST11_CHSTB CHST11 chr10 69230145 0.008757 8.631e-09 35362 TRUE reported textfile 2 1 TRUE

8612 rs7295941 T C T C -0.0606 0.0066 0.17199 NA FALSE FALSE FALSE GCST006061 0.0096 0.4917 537409 outcome TRUE reported 7779_86_CHST11_CHSTB CHST11 chr12 104417649 0.010934 2.988e-08 35360 TRUE reported textfile 2 1 TRUE

8613 rs79213114 A G A G 0.197 -0.0546 0.02803 NA FALSE FALSE FALSE GCST006061 0.0315 0.08335 537409 outcome TRUE reported 7779_86_CHST11_CHSTB CHST11 chr12 104505408 0.025515 1.156e-14 35361 TRUE reported textfile 2 1 TRUE

8614 rs856595 C T C T -0.0974 4e-04 0.46732 NA FALSE FALSE FALSE GCST006061 0.0072 0.9568 537409 outcome TRUE reported 7779_86_CHST11_CHSTB CHST11 chr12 104667669 0.008253 3.817e-32 35355 TRUE reported textfile 2 1 TRUE

8615 rs10922098 T C T C -0.0848 -0.009 0.40011 NA FALSE FALSE FALSE GCST006061 0.0075 0.2275 537409 outcome TRUE reported 6543_182_PRLH_PRRP PRLH chr1 196695521 0.008045 5.57e-26 35338 TRUE reported textfile 2 1 TRUE

8616 rs1260326 C T C T -0.0498 -0.0069 0.34132 NA FALSE FALSE FALSE GCST006061 0.0073 0.3401 537409 outcome TRUE reported 6543_182_PRLH_PRRP PRLH chr2 27508073 0.008352 2.484e-09 35373 TRUE reported textfile 2 1 TRUE

8617 rs1355538 G A G A -0.0503 -0.0076 0.39847 NA FALSE FALSE FALSE GCST006061 0.0072 0.2923 537409 outcome TRUE reported 6543_182_PRLH_PRRP PRLH chr3 165787389 0.008034 3.823e-10 35338 TRUE reported textfile 2 1 TRUE

8618 rs10922098 T C T C -0.164 -0.009 0.40011 NA FALSE FALSE FALSE GCST006061 0.0075 0.2275 537409 outcome TRUE reported 8985_13_SERAC1_SRAC1 SERAC1 chr1 196695521 0.008109 5.959e-91 35338 TRUE reported textfile 2 1 TRUE

8619 rs1707652 T C T C 0.0787 0.0081 0.3102 NA FALSE FALSE FALSE GCST006061 0.0074 0.2701 537409 outcome TRUE reported 8985_13_SERAC1_SRAC1 SERAC1 chr3 165761011 0.008596 5.394e-20 35339 TRUE reported textfile 2 1 TRUE

8620 rs704 A G A G 0.0551 -0.0155 0.49189 NA FALSE FALSE FALSE GCST006061 0.0072 0.03144 537409 outcome TRUE reported 8985_13_SERAC1_SRAC1 SERAC1 chr17 28367840 0.007986 5.206e-12 35360 TRUE reported textfile 2 1 TRUE

8621 rs10922098 T C T C -0.105 -0.009 0.40011 NA FALSE FALSE FALSE GCST006061 0.0075 0.2275 537409 outcome TRUE reported 10530_8_PSMB6_PSB6 PSMB6 chr1 196695521 0.00806 8.558e-39 35326 TRUE reported textfile 2 1 TRUE

8622 rs1355538 G A G A -0.0449 -0.0076 0.39847 NA FALSE FALSE FALSE GCST006061 0.0072 0.2923 537409 outcome TRUE reported 10530_8_PSMB6_PSB6 PSMB6 chr3 165787389 0.008068 2.622e-08 35326 TRUE reported textfile 2 1 TRUE

8623 rs5754116 T C T C -0.0541 -0.0037 0.30857 NA FALSE FALSE FALSE GCST006061 0.008 0.6412 537409 outcome TRUE reported 10530_8_PSMB6_PSB6 PSMB6 chr22 32497933 0.008587 2.967e-10 35359 TRUE reported textfile 2 1 TRUE

8624 rs10922098 T C T C -0.0652 -0.009 0.40011 NA FALSE FALSE FALSE GCST006061 0.0075 0.2275 537409 outcome TRUE reported 8569_147_ARL8B_ARL8B ARL8B chr1 196695521 0.00808 7.074e-16 35338 TRUE reported textfile 2 1 TRUE

8625 rs10922098 T C T C -0.1755 -0.009 0.40011 NA FALSE FALSE FALSE GCST006061 0.0075 0.2275 537409 outcome TRUE reported 18190_15_CARD18_ICBR CARD18 chr1 196695521 0.008125 1.832e-103 35338 TRUE reported textfile 2 1 TRUE

8626 rs111696008 A G A G -0.1783 -0.0259 0.02443 NA FALSE FALSE FALSE GCST006061 0.0401 0.519 537409 outcome TRUE reported 18190_15_CARD18_ICBR CARD18 chr2 216622986 0.026526 1.797e-11 35359 TRUE reported textfile 2 1 TRUE

8627 rs3917538 A G A G 0.0646 -0.0137 0.21967 NA FALSE FALSE FALSE GCST006061 0.0082 0.09535 537409 outcome TRUE reported 18190_15_CARD18_ICBR

CARD18 chr7 95308581 0.009654 2.205e-11 35349 TRUE reported
textfile 2 1 TRUE
8628 rs4900225 A C A C 0.049 -0.0031 0.46387 NA FALSE FALSE FALSE
GCST006061 0.0072 0.6691 537409 outcome TRUE reported 18190_15_CARD18_ICBR
CARD18 chr14 94322256 0.008014 9.689e-10 35372 TRUE reported
textfile 2 1 TRUE
8629 rs6445035 A G A G 0.1228 0.0154 0.1904 NA FALSE FALSE FALSE
GCST006061 0.0089 0.08256 537409 outcome TRUE reported 18190_15_CARD18_ICBR
CARD18 chr3 165762312 0.010137 8.853e-34 35339 TRUE reported
textfile 2 1 TRUE
8630 rs10922098 T C T C -0.0633 -0.009 0.40011 NA FALSE FALSE FALSE
GCST006061 0.0075 0.2275 537409 outcome TRUE reported 4140_3_IL7_IL_7 IL7
chr1 196695521 0.008059 4.02e-15 35338 TRUE reported textfile 2
1 TRUE
8631 rs2393326 T C T C -0.053 0.0096 0.2401 NA FALSE FALSE FALSE
GCST006061 0.008 0.2334 537409 outcome TRUE reported 4140_3_IL7_IL_7 IL7
chr12 117815540 0.009247 9.95e-09 35371 TRUE reported textfile 2
1 TRUE
8632 rs10922098 T C T C -0.1213 -0.009 0.40011 NA FALSE FALSE FALSE
GCST006061 0.0075 0.2275 537409 outcome TRUE reported 10703_203_SUDS3_SDS3
SUDS3 chr1 196695521 0.008089 7.859e-51 35326 TRUE reported
textfile 2 1 TRUE
8633 rs11569415 A G A G -0.0556 0.0011 0.24276 NA FALSE FALSE FALSE
GCST006061 0.0097 0.9129 537409 outcome TRUE reported 10703_203_SUDS3_SDS3
SUDS3 chr19 6716268 0.00921 1.573e-09 35360 TRUE reported textfile 2
1 TRUE
8634 rs2634465 G A G A -0.0628 0.0042 0.1413 NA FALSE FALSE FALSE
GCST006061 0.0092 0.6461 537409 outcome TRUE reported 10703_203_SUDS3_SDS3
SUDS3 chr4 55666449 0.011447 4.111e-08 35346 TRUE reported
textfile 2 1 TRUE
8635 rs10922098 T C T C -0.0948 -0.009 0.40011 NA FALSE FALSE FALSE
GCST006061 0.0075 0.2275 537409 outcome TRUE reported 7233_73_P2RX6_P2RX6
P2RX6 chr1 196695521 0.008131 2.048e-31 35338 TRUE reported
textfile 2 1 TRUE
8636 rs1355538 G A G A -0.0541 -0.0076 0.39847 NA FALSE FALSE FALSE
GCST006061 0.0072 0.2923 537409 outcome TRUE reported 7233_73_P2RX6_P2RX6
P2RX6 chr3 165787389 0.008128 2.818e-11 35338 TRUE reported
textfile 2 1 TRUE
8637 rs10922098 T C T C -0.1571 -0.009 0.40011 NA FALSE FALSE FALSE
GCST006061 0.0075 0.2275 537409 outcome TRUE reported 15497_9_HS3ST1_HS3S1
HS3ST1 chr1 196695521 0.008086 4.337e-84 35338 TRUE reported
textfile 2 1 TRUE
8638 rs1707652 T C T C 0.0704 0.0081 0.3102 NA FALSE FALSE FALSE
GCST006061 0.0074 0.2701 537409 outcome TRUE reported 15497_9_HS3ST1_HS3S1
HS3ST1 chr3 165761011 0.008568 2.099e-16 35339 TRUE reported
textfile 2 1 TRUE
8639 rs10922098 T C T C -0.0399 -0.009 0.40011 NA FALSE FALSE FALSE
GCST006061 0.0075 0.2275 537409 outcome TRUE reported 8326_63_SIGIRR_SIGIRR
SIGIRR chr1 196695521 0.007197 2.961e-08 35338 TRUE reported
textfile 2 1 TRUE
8640 rs114076668 T C T C 0.1399 -0.026 0.03049 NA FALSE FALSE FALSE
GCST006061 0.0236 0.2704 537409 outcome TRUE reported 8326_63_SIGIRR_SIGIRR
SIGIRR chr3 40369338 0.022117 2.523e-10 35353 TRUE reported
textfile 2 1 TRUE
8641 rs114541510 A C A C -0.1397 -0.0253 0.02359 NA FALSE FALSE FALSE
GCST006061 0.0302 0.4015 537409 outcome TRUE reported 8326_63_SIGIRR_SIGIRR
SIGIRR chr3 54148395 0.024984 2.25e-08 35347 TRUE reported
textfile 2 1 TRUE
8642 rs12257143 C T C T -0.2227 -0.009 0.03461 NA FALSE FALSE FALSE
GCST006061 0.0174 0.6034 537409 outcome TRUE reported 8326_63_SIGIRR_SIGIRR
SIGIRR chr10 7732765 0.019535 4.179e-30 35363 TRUE reported textfile
2 1 TRUE
8643 rs140750015 A C A C 0.1925 -0.0175 0.01797 NA FALSE FALSE FALSE
GCST006061 0.0276 0.5256 537409 outcome TRUE reported 8326_63_SIGIRR_SIGIRR
SIGIRR chr3 50176870 0.027858 4.844e-12 35343 TRUE reported
textfile 2 1 TRUE

8644 rs143836348 C T C T -0.3087 0.0098 0.02829 NA FALSE FALSE FALSE
GCST006061 0.0303 0.7476 537409 outcome TRUE reported 8326_63_SIGIRR_SIGIRR
SIGIRR chr3 52524221 0.022473 6.135e-43 35352 TRUE reported
textfile 2 1 TRUE

8645 rs146456061 G A G A 0.257 -0.0115 0.02035 NA FALSE FALSE FALSE
GCST006061 0.036 0.7493 537409 outcome TRUE reported 8326_63_SIGIRR_SIGIRR
SIGIRR chr3 51625414 0.026943 1.448e-21 35337 TRUE reported
textfile 2 1 TRUE

8646 rs148645177 A G A G 0.1196 -0.03 0.03118 NA FALSE FALSE FALSE
GCST006061 0.0332 0.3665 537409 outcome TRUE reported 8326_63_SIGIRR_SIGIRR
SIGIRR chr3 32798210 0.021332 2.062e-08 35348 TRUE reported
textfile 2 1 TRUE

8647 rs148834251 C T C T -0.2037 -0.0088 0.03638 NA FALSE FALSE FALSE
GCST006061 0.0245 0.7206 537409 outcome TRUE reported 8326_63_SIGIRR_SIGIRR
SIGIRR chr3 49523775 0.020256 8.627e-24 35344 TRUE reported
textfile 2 1 TRUE

8648 rs150933254 T G T G 0.4567 -0.0174 0.04126 NA FALSE FALSE FALSE
GCST006061 0.0236 0.4596 537409 outcome TRUE reported 8326_63_SIGIRR_SIGIRR
SIGIRR chr3 52283868 0.018842 8.857e-130 35351 TRUE reported
textfile 2 1 TRUE

8649 rs17477867 A G A G 0.4562 -0.027 0.05931 NA FALSE FALSE FALSE
GCST006061 0.0164 0.1008 537409 outcome TRUE reported 8326_63_SIGIRR_SIGIRR
SIGIRR chr3 52761957 0.016035 4.801e-178 35354 TRUE reported
textfile 2 1 TRUE

8650 rs186797403 A G A G -0.1937 0.0086 0.01715 NA FALSE FALSE FALSE
GCST006061 0.0488 0.8597 537409 outcome TRUE reported 8326_63_SIGIRR_SIGIRR
SIGIRR chr3 39957040 0.029258 3.584e-11 35349 TRUE reported
textfile 2 1 TRUE

8651 rs187979386 T C T C -0.1791 0.0045 0.03103 NA FALSE FALSE FALSE
GCST006061 0.0242 0.8509 537409 outcome TRUE reported 8326_63_SIGIRR_SIGIRR
SIGIRR chr3 48401516 0.022036 4.384e-16 35344 TRUE reported
textfile 2 1 TRUE

8652 rs2633726 C T C T 0.1319 -0.0018 0.35583 NA FALSE FALSE FALSE
GCST006061 0.0077 0.8175 537409 outcome TRUE reported 8326_63_SIGIRR_SIGIRR
SIGIRR chr3 53713106 0.007919 2.745e-62 35349 TRUE reported
textfile 2 1 TRUE

8653 rs3802589 G T G T 0.1488 7e-04 0.02587 NA FALSE FALSE FALSE
GCST006061 0.0207 0.9737 537409 outcome TRUE reported 8326_63_SIGIRR_SIGIRR
SIGIRR chr10 7742333 0.021904 1.096e-11 35363 TRUE reported textfile
2 1 TRUE

8654 rs62242377 C T C T 0.2951 -0.0074 0.01066 NA FALSE FALSE FALSE
GCST006061 0.0459 0.8721 537409 outcome TRUE reported 8326_63_SIGIRR_SIGIRR
SIGIRR chr3 45216727 0.036194 3.544e-16 35348 TRUE reported
textfile 2 1 TRUE

8655 rs62261068 T C T C 0.3965 -0.0049 0.12018 NA FALSE FALSE FALSE
GCST006061 0.015 0.741 537409 outcome TRUE reported 8326_63_SIGIRR_SIGIRR
SIGIRR chr3 52155181 0.011442 1e-200 35337 TRUE reported textfile
2 1 TRUE

8656 rs6766712 T G T G 0.1577 -0.0079 0.2674 NA FALSE FALSE FALSE
GCST006061 0.0081 0.327 537409 outcome TRUE reported 8326_63_SIGIRR_SIGIRR
SIGIRR chr3 126541765 0.008514 1.376e-76 35371 TRUE reported
textfile 2 1 TRUE

8657 rs73064834 G A G A 0.1251 0.0178 0.04084 NA FALSE FALSE FALSE
GCST006061 0.0214 0.4058 537409 outcome TRUE reported 8326_63_SIGIRR_SIGIRR
SIGIRR chr3 38317365 0.018823 3.006e-11 35354 TRUE reported
textfile 2 1 TRUE

8658 rs77563378 A C A C 0.3956 0.0126 0.01013 NA FALSE FALSE FALSE
GCST006061 0.0453 0.7805 537409 outcome TRUE reported 8326_63_SIGIRR_SIGIRR
SIGIRR chr3 46954439 0.038015 2.318e-25 35342 TRUE reported
textfile 2 1 TRUE

8659 rs10922098 T C T C -0.1152 -0.009 0.40011 NA FALSE FALSE FALSE
GCST006061 0.0075 0.2275 537409 outcome TRUE reported 8999_19_CEACAM3_CEAM3
CEACAM3 chr1 196695521 0.008155 2.617e-45 35338 TRUE reported
textfile 2 1 TRUE

8660 rs1707652 T C T C 0.092 0.0081 0.3102 NA FALSE FALSE FALSE
GCST006061 0.0074 0.2701 537409 outcome TRUE reported 8999_19_CEACAM3_CEAM3

CEACAM3 chr3 165761011 0.008613 1.243e-26 35339 TRUE reported
textfile 2 1 TRUE
8661 rs10922098 T C T C -0.0789 -0.009 0.40011 NA FALSE FALSE FALSE
GCST006061 0.0075 0.2275 537409 outcome TRUE reported 11146_4_TBX22_TBX22
TBX22 chr1 196695521 0.008071 1.43e-22 35329 TRUE reported
textfile 2 1 TRUE
8662 rs1355538 G A G A -0.0517 -0.0076 0.39847 NA FALSE FALSE FALSE
GCST006061 0.0072 0.2923 537409 outcome TRUE reported 11146_4_TBX22_TBX22
TBX22 chr3 165787389 0.008063 1.436e-10 35329 TRUE reported
textfile 2 1 TRUE
8663 rs10922098 T C T C -0.0938 -0.009 0.40011 NA FALSE FALSE FALSE
GCST006061 0.0075 0.2275 537409 outcome TRUE reported 6933_20_MRPL34_RM34
MRPL34 chr1 196695521 0.008038 1.82e-31 35338 TRUE reported
textfile 2 1 TRUE
8664 rs6542680 T C T C -0.0646 -0.0012 0.18362 NA FALSE FALSE FALSE
GCST006061 0.0091 0.8935 537409 outcome TRUE reported 6933_20_MRPL34_RM34
MRPL34 chr2 3592552 0.010206 2.456e-10 35332 TRUE reported textfile
2 1 TRUE
8665 rs10922098 T C T C -0.0483 -0.009 0.40011 NA FALSE FALSE FALSE
GCST006061 0.0075 0.2275 537409 outcome TRUE reported 8601_167_LRP1_sLRP1
LRP1 chr1 196695521 0.008046 1.934e-09 35338 TRUE reported
textfile 2 1 TRUE
8666 rs704 A G A G 0.092 -0.0155 0.49189 NA FALSE FALSE FALSE GCST006061
0.0072 0.03144 537409 outcome TRUE reported 8601_167_LRP1_sLRP1 LRP1 chr17
28367840 0.007878 1.663e-31 35360 TRUE reported textfile 2 1 TRUE
8667 rs10922098 T C T C -0.0564 -0.009 0.40011 NA FALSE FALSE FALSE
GCST006061 0.0075 0.2275 537409 outcome TRUE reported 18170_46_PYCR1_P5CR1
PYCR1 chr1 196695521 0.008007 1.874e-12 35338 TRUE reported
textfile 2 1 TRUE
8668 rs1126477 T C T C -0.0585 2e-04 0.22827 NA FALSE FALSE FALSE
GCST006061 0.0082 0.984 537409 outcome TRUE reported 18170_46_PYCR1_P5CR1
PYCR1 chr3 46459778 0.009394 4.74e-10 35343 TRUE reported
textfile 2 1 TRUE
8669 rs10922098 T C T C -0.0781 -0.009 0.40011 NA FALSE FALSE FALSE
GCST006061 0.0075 0.2275 537409 outcome TRUE reported 17199_43_HIF1AN_HIF1N
HIF1AN chr1 196695521 0.008103 5.492e-22 35338 TRUE reported
textfile 2 1 TRUE
8670 rs6542680 T C T C -0.0592 -0.0012 0.18362 NA FALSE FALSE FALSE
GCST006061 0.0091 0.8935 537409 outcome TRUE reported 17199_43_HIF1AN_HIF1N
HIF1AN chr2 3592552 0.010271 8.225e-09 35332 TRUE reported textfile
2 1 TRUE
8671 rs10922098 T C T C -0.1715 -0.009 0.40011 NA FALSE FALSE FALSE
GCST006061 0.0075 0.2275 537409 outcome TRUE reported 10550_37_BMPR1B_BMP_RIB
BMPR1B chr1 196695521 0.008179 1.257e-97 35326 TRUE reported
textfile 2 1 TRUE
8672 rs1707652 T C T C 0.085 0.0081 0.3102 NA FALSE FALSE FALSE
GCST006061 0.0074 0.2701 537409 outcome TRUE reported 10550_37_BMPR1B_BMP_RIB
BMPR1B chr3 165761011 0.00867 1.083e-22 35327 TRUE reported textfile
2 1 TRUE
8673 rs10922098 T C T C -0.1406 -0.009 0.40011 NA FALSE FALSE FALSE
GCST006061 0.0075 0.2275 537409 outcome TRUE reported 14687_6_PLIN3_M6PBP
PLIN3 chr1 196695521 0.008013 6.405e-69 35338 TRUE reported
textfile 2 1 TRUE
8674 rs1355538 G A G A -0.0549 -0.0076 0.39847 NA FALSE FALSE FALSE
GCST006061 0.0072 0.2923 537409 outcome TRUE reported 14687_6_PLIN3_M6PBP
PLIN3 chr3 165787389 0.008018 7.547e-12 35338 TRUE reported
textfile 2 1 TRUE
8675 rs10922098 T C T C -0.1211 -0.009 0.40011 NA FALSE FALSE FALSE
GCST006061 0.0075 0.2275 537409 outcome TRUE reported 11988_24_PTPRH_PTPRH
PTPRH chr1 196695521 0.008154 6.853e-50 35329 TRUE reported
textfile 2 1 TRUE
8676 rs1355538 G A G A -0.0586 -0.0076 0.39847 NA FALSE FALSE FALSE
GCST006061 0.0072 0.2923 537409 outcome TRUE reported 11988_24_PTPRH_PTPRH
PTPRH chr3 165787389 0.008157 6.788e-13 35329 TRUE reported
textfile 2 1 TRUE
8677 rs10922098 T C T C -0.1173 -0.009 0.40011 NA FALSE FALSE FALSE

GCST006061 0.0075 0.2275 537409 outcome TRUE reported 11247_20_NAGS_NAGS
NAGS chr1 196695521 0.008092 1.291e-47 35329 TRUE reported
textfile 2 1 TRUE
8678 rs6445035 A G A G 0.0723 0.0154 0.1904 NA FALSE FALSE FALSE
GCST006061 0.0089 0.08256 537409 outcome TRUE reported 11247_20_NAGS_NAGS
NAGS chr3 165762312 0.010084 7.503e-13 35330 TRUE reported
textfile 2 1 TRUE
8679 rs10922098 T C T C -0.1087 -0.009 0.40011 NA FALSE FALSE FALSE
GCST006061 0.0075 0.2275 537409 outcome TRUE reported 7745_3_CISD1_ZCD1
CISD1 chr1 196695521 0.007986 3.398e-42 35338 TRUE reported
textfile 2 1 TRUE
8680 rs1706435 G A G A 0.0663 0.0117 0.36981 NA FALSE FALSE FALSE
GCST006061 0.0072 0.1047 537409 outcome TRUE reported 7745_3_CISD1_ZCD1
CISD1 chr3 165743688 0.008099 2.698e-16 35339 TRUE reported
textfile 2 1 TRUE
8681 rs10922098 T C T C -0.1334 -0.009 0.40011 NA FALSE FALSE FALSE
GCST006061 0.0075 0.2275 537409 outcome TRUE reported 6510_56_RNF128_RN128
RNF128 chr1 196695521 0.00806 1.559e-61 35338 TRUE reported textfile
2 1 TRUE
8682 rs2686395 C T C T -0.0837 -0.0077 0.3101 NA FALSE FALSE FALSE
GCST006061 0.0074 0.2965 537409 outcome TRUE reported 6510_56_RNF128_RN128
RNF128 chr3 165767231 0.008528 9.741e-23 35339 TRUE reported
textfile 2 1 TRUE
8683 rs10922098 T C T C -0.2103 -0.009 0.40011 NA FALSE FALSE FALSE
GCST006061 0.0075 0.2275 537409 outcome TRUE reported 17393_13_ACADS_SCAD
ACADS chr1 196695521 0.008161 1.902e-146 35338 TRUE reported
textfile 2 1 TRUE
8684 rs111696008 A G A G -0.196 -0.0259 0.02443 NA FALSE FALSE FALSE
GCST006061 0.0401 0.519 537409 outcome TRUE reported 17393_13_ACADS_SCAD
ACADS chr2 216622986 0.026686 2.064e-13 35359 TRUE reported
textfile 2 1 TRUE
8685 rs1408868 C T C T -0.0655 0.0058 0.1337 NA FALSE FALSE FALSE
GCST006061 0.0097 0.5484 537409 outcome TRUE reported 17393_13_ACADS_SCAD
ACADS chr1 196183998 0.011784 2.72e-08 35337 TRUE reported
textfile 2 1 TRUE
8686 rs704 A G A G 0.0472 -0.0155 0.49189 NA FALSE FALSE FALSE GCST006061
0.0072 0.03144 537409 outcome TRUE reported 17393_13_ACADS_SCAD ACADS chr17
28367840 0.008051 4.549e-09 35360 TRUE reported textfile 2 1 TRUE
8687 rs73169103 T C T C 0.1281 0.0178 0.03563 NA FALSE FALSE FALSE
GCST006061 0.0249 0.4756 537409 outcome TRUE reported 17393_13_ACADS_SCAD
ACADS chr3 167190675 0.021685 3.48e-09 35354 TRUE reported
textfile 2 1 TRUE
8688 rs10922098 T C T C -0.0714 -0.009 0.40011 NA FALSE FALSE FALSE
GCST006061 0.0075 0.2275 537409 outcome TRUE reported 8839_4_MTX2_Metaxin_2
MTX2 chr1 196695521 0.008047 7.15e-19 35338 TRUE reported
textfile 2 1 TRUE
8689 rs112969279 A G A G -0.1293 0.0202 0.03085 NA FALSE FALSE FALSE
GCST006061 0.0254 0.427 537409 outcome TRUE reported 8839_4_MTX2_Metaxin_2
MTX2 chr2 148490967 0.023433 3.43e-08 35346 TRUE reported
textfile 2 1 TRUE
8690 rs10922098 T C T C -0.0783 -0.009 0.40011 NA FALSE FALSE FALSE
GCST006061 0.0075 0.2275 537409 outcome TRUE reported 11149_3_TLR1_TLR1
TLR1 chr1 196695521 0.008014 1.51e-22 35329 TRUE reported
textfile 2 1 TRUE
8691 rs1555405 A G A G -0.0494 -0.0047 0.30381 NA FALSE FALSE FALSE
GCST006061 0.0081 0.5592 537409 outcome TRUE reported 11149_3_TLR1_TLR1
TLR1 chr14 100710432 0.008564 8e-09 35371 TRUE reported textfile
2 1 TRUE
8692 rs10922098 T C T C -0.1742 -0.009 0.40011 NA FALSE FALSE FALSE
GCST006061 0.0075 0.2275 537409 outcome TRUE reported 19366_8_PEX26_PEX26
PEX26 chr1 196695521 0.008147 1.974e-101 35338 TRUE reported
textfile 2 1 TRUE
8693 rs11569415 A G A G -0.0575 0.0011 0.24276 NA FALSE FALSE FALSE
GCST006061 0.0097 0.9129 537409 outcome TRUE reported 19366_8_PEX26_PEX26
PEX26 chr19 6716268 0.009295 6.159e-10 35372 TRUE reported textfile
2 1 TRUE

8694 rs1707652 T C T C 0.0869 0.0081 0.3102 NA FALSE FALSE FALSE GCST006061 0.0074 0.2701 537409 outcome TRUE reported 19366_8_PEX26_PEX26 PEX26 chr3 165761011 0.008629 7.469e-24 35339 TRUE reported textfile 2 1 TRUE
8695 rs76004643 T G T G 0.0505 -0.0069 0.2662 NA FALSE FALSE FALSE GCST006061 0.009 0.4422 537409 outcome TRUE reported 19366_8_PEX26_PEX26 PEX26 chr1 197847653 0.009042 2.339e-08 35337 TRUE reported textfile 2 1 TRUE
8696 rs10922098 T C T C -0.1055 -0.009 0.40011 NA FALSE FALSE FALSE GCST006061 0.0075 0.2275 537409 outcome TRUE reported 5602_62_RNASE10_RNS10 RNASE10 chr1 196695521 0.008092 7.472e-39 35338 TRUE reported textfile 2 1 TRUE
8697 rs1706435 G A G A 0.0487 0.0117 0.36981 NA FALSE FALSE FALSE GCST006061 0.0072 0.1047 537409 outcome TRUE reported 5602_62_RNASE10_RNS10 RNASE10 chr3 165743688 0.008215 3.06e-09 35339 TRUE reported textfile 2 1 TRUE
8698 rs6542680 T C T C -0.0647 -0.0012 0.18362 NA FALSE FALSE FALSE GCST006061 0.0091 0.8935 537409 outcome TRUE reported 5602_62_RNASE10_RNS10 RNASE10 chr2 3592552 0.010264 2.909e-10 35332 TRUE reported textfile 2 1 TRUE
8699 rs10922098 T C T C -0.0836 -0.009 0.40011 NA FALSE FALSE FALSE GCST006061 0.0075 0.2275 537409 outcome TRUE reported 8318_13_SPRED1_SPRE1 SPRED1 chr1 196695521 0.008042 2.59e-25 35338 TRUE reported textfile 2 1 TRUE
8700 rs4977155 C T C T -0.0619 -0.0069 0.16663 NA FALSE FALSE FALSE GCST006061 0.0127 0.5868 537409 outcome TRUE reported 8318_13_SPRED1_SPRE1 SPRED1 chr14 106473508 0.010812 1.033e-08 34966 TRUE reported textfile 2 1 TRUE
8701 rs7080536 A G A G -0.1487 -0.0145 0.04142 NA FALSE FALSE FALSE GCST006061 0.0203 0.4753 537409 outcome TRUE reported 8318_13_SPRED1_SPRE1 SPRED1 chr10 113588287 0.019663 3.959e-14 35387 TRUE reported textfile 2 1 TRUE
8702 rs10922098 T C T C -0.1035 -0.009 0.40011 NA FALSE FALSE FALSE GCST006061 0.0075 0.2275 537409 outcome TRUE reported 11198_37_CREB3L1_CR3L1 CREB3L1 chr1 196695521 0.007984 1.992e-38 35329 TRUE reported textfile 2 1 TRUE
8703 rs1355538 G A G A -0.057 -0.0076 0.39847 NA FALSE FALSE FALSE GCST006061 0.0072 0.2923 537409 outcome TRUE reported 11198_37_CREB3L1_CR3L1 CREB3L1 chr3 165787389 0.007986 9.534e-13 35329 TRUE reported textfile 2 1 TRUE
8704 rs10922098 T C T C -0.1185 -0.009 0.40011 NA FALSE FALSE FALSE GCST006061 0.0075 0.2275 537409 outcome TRUE reported 11120_49_NAT14_NAT14 NAT14 chr1 196695521 0.008103 1.973e-48 35329 TRUE reported textfile 2 1 TRUE
8705 rs1707652 T C T C 0.0682 0.0081 0.3102 NA FALSE FALSE FALSE GCST006061 0.0074 0.2701 537409 outcome TRUE reported 11120_49_NAT14_NAT14 NAT14 chr3 165761011 0.008564 1.675e-15 35330 TRUE reported textfile 2 1 TRUE
8706 rs35209896 A C A C 0.0743 0.0267 0.09263 NA FALSE FALSE FALSE GCST006061 0.0355 0.4521 537409 outcome TRUE reported 11120_49_NAT14_NAT14 NAT14 chr6 32519009 0.013576 4.429e-08 35430 TRUE reported textfile 2 1 TRUE
8707 rs704 A G A G -0.0997 -0.0155 0.49189 NA FALSE FALSE FALSE GCST006061 0.0072 0.03144 537409 outcome TRUE reported 11120_49_NAT14_NAT14 NAT14 chr17 28367840 0.007945 4.055e-36 35351 TRUE reported textfile 2 1 TRUE
8708 rs10922098 T C T C -0.0623 -0.009 0.40011 NA FALSE FALSE FALSE GCST006061 0.0075 0.2275 537409 outcome TRUE reported 7124_18_IL21_IL_21 IL21 chr1 196695521 0.008253 4.398e-14 35338 TRUE reported textfile 2 1 TRUE
8709 rs11064498 G A G A -0.0996 -8.00E-04 0.1306 NA FALSE FALSE FALSE GCST006061 0.0102 0.939 537409 outcome TRUE reported 7124_18_IL21_IL_21 IL21 chr12 7064203 0.012173 2.783e-16 35390 TRUE reported textfile 2 1 TRUE
8710 rs7651933 A G A G 0.0556 0.0169 0.24788 NA FALSE FALSE FALSE GCST006061 0.0081 0.0377 537409 outcome TRUE reported 7124_18_IL21_IL_21

IL21 chr3 165750184 0.009373 2.996e-09 35339 TRUE reported textfile 2 1 TRUE
8711 rs7887 T G T G -0.2208 -0.0019 0.34968 NA FALSE FALSE FALSE GCST006061 0.0076 0.8063 537409 outcome TRUE reported 7124_18_IL21_IL_21 IL21 chr6 31896770 0.00847 8.314e-150 35441 TRUE reported textfile 2 1 TRUE
8712 rs10922098 T C T C -0.1067 -0.009 0.40011 NA FALSE FALSE FALSE GCST006061 0.0075 0.2275 537409 outcome TRUE reported 5261_13_ABL2_ABL2 ABL2 chr1 196695521 0.008035 3.015e-40 35338 TRUE reported textfile 2 1 TRUE
8713 rs1355538 G A G A -0.054 -0.0076 0.39847 NA FALSE FALSE FALSE GCST006061 0.0072 0.2923 537409 outcome TRUE reported 5261_13_ABL2_ABL2 ABL2 chr3 165787389 0.008038 1.84e-11 35338 TRUE reported textfile 2 1 TRUE
8714 rs10922098 T C T C -0.0452 -0.009 0.40011 NA FALSE FALSE FALSE GCST006061 0.0075 0.2275 537409 outcome TRUE reported 9026_40_BTNL8_BTNL8 BTNL8 chr1 196695521 0.008162 3.065e-08 35339 TRUE reported textfile 2 1 TRUE
8715 rs601338 A G A G -0.0922 0.0056 0.39439 NA FALSE FALSE FALSE GCST006061 0.0076 0.4559 537409 outcome TRUE reported 9026_40_BTNL8_BTNL8 BTNL8 chr19 48703417 0.008208 2.792e-29 35378 TRUE reported textfile 2 1 TRUE
8716 rs6542680 T C T C -0.0579 -0.0012 0.18362 NA FALSE FALSE FALSE GCST006061 0.0091 0.8935 537409 outcome TRUE reported 9026_40_BTNL8_BTNL8 BTNL8 chr2 3592552 0.010347 2.199e-08 35333 TRUE reported textfile 2 1 TRUE
8717 rs76546858 T C T C 0.1137 -0.0057 0.08906 NA FALSE FALSE FALSE GCST006061 0.0169 0.7356 537409 outcome TRUE reported 9026_40_BTNL8_BTNL8 BTNL8 chr5 181070075 0.014023 5.137e-16 35318 TRUE reported textfile 2 1 TRUE
8718 rs10922098 T C T C -0.1264 -0.009 0.40011 NA FALSE FALSE FALSE GCST006061 0.0075 0.2275 537409 outcome TRUE reported 18840_205_SH2D1B_SH21B SH2D1B chr1 196695521 0.008093 5.523e-55 35338 TRUE reported textfile 2 1 TRUE
8719 rs10922098 T C T C -0.2135 -0.009 0.40011 NA FALSE FALSE FALSE GCST006061 0.0075 0.2275 537409 outcome TRUE reported 10012_5_SPDEF_SPDEF SPDEF chr1 196695521 0.008076 5.017e-154 35326 TRUE reported textfile 2 1 TRUE
8720 rs1707652 T C T C 0.0987 0.0081 0.3102 NA FALSE FALSE FALSE GCST006061 0.0074 0.2701 537409 outcome TRUE reported 10012_5_SPDEF_SPDEF SPDEF chr3 165761011 0.008574 1.157e-30 35327 TRUE reported textfile 2 1 TRUE
8721 rs35351085 T C T C 0.1103 0.0061 0.04877 NA FALSE FALSE FALSE GCST006061 0.022 0.7825 537409 outcome TRUE reported 10012_5_SPDEF_SPDEF SPDEF chr1 197992799 0.018372 1.93e-09 35331 TRUE reported textfile 2 1 TRUE
8722 rs10922098 T C T C -0.2127 -0.009 0.40011 NA FALSE FALSE FALSE GCST006061 0.0075 0.2275 537409 outcome TRUE reported 9176_3_MUC1_MUC1 MUC1 chr1 196695521 0.008122 3.631e-151 35338 TRUE reported textfile 2 1 TRUE
8723 rs111696008 A G A G -0.1499 -0.0259 0.02443 NA FALSE FALSE FALSE GCST006061 0.0401 0.519 537409 outcome TRUE reported 9176_3_MUC1_MUC1 MUC1 chr2 216622986 0.026567 1.677e-08 35359 TRUE reported textfile 2 1 TRUE
8724 rs1451915 A C A C 0.0643 -0.0107 0.13641 NA FALSE FALSE FALSE GCST006061 0.0124 0.3895 537409 outcome TRUE reported 9176_3_MUC1_MUC1 MUC1 chr1 196058002 0.011605 3.016e-08 35335 TRUE reported textfile 2 1 TRUE
8725 rs1707652 T C T C 0.112 0.0081 0.3102 NA FALSE FALSE FALSE GCST006061 0.0074 0.2701 537409 outcome TRUE reported 9176_3_MUC1_MUC1 MUC1 chr3 165761011 0.008612 1.15e-38 35339 TRUE reported textfile 2 1 TRUE
8726 rs704 A G A G 0.147 -0.0155 0.49189 NA FALSE FALSE FALSE GCST006061 0.0072 0.03144 537409 outcome TRUE reported 9176_3_MUC1_MUC1 MUC1 chr17 28367840 0.007984 1.051e-75 35360 TRUE reported textfile 2 1 TRUE
8727 rs10922098 T C T C -0.1293 -0.009 0.40011 NA FALSE FALSE FALSE GCST006061 0.0075 0.2275 537409 outcome TRUE reported 18410_26_ARL14_ARL14

ARL14 chr1 196695521 0.007972 3.708e-59 35338 TRUE reported
textfile 2 1 TRUE
8728 rs2686395 C T C T -0.0786 -0.0077 0.3101 NA FALSE FALSE FALSE
GCST006061 0.0074 0.2965 537409 outcome TRUE reported 18410_26_ARL14_ARL14
ARL14 chr3 165767231 0.008439 1.233e-20 35339 TRUE reported
textfile 2 1 TRUE
8729 rs10922098 T C T C -0.0827 -0.009 0.40011 NA FALSE FALSE FALSE
GCST006061 0.0075 0.2275 537409 outcome TRUE reported 7742_11_RNF148_RN148
RNF148 chr1 196695521 0.007991 4.213e-25 35338 TRUE reported
textfile 2 1 TRUE
8730 rs4977155 C T C T -0.0683 -0.0069 0.16663 NA FALSE FALSE FALSE
GCST006061 0.0127 0.5868 537409 outcome TRUE reported 7742_11_RNF148_RN148
RNF148 chr14 106473508 0.010748 2.09e-10 34966 TRUE reported
textfile 2 1 TRUE
8731 rs10922098 T C T C -0.1239 -0.009 0.40011 NA FALSE FALSE FALSE
GCST006061 0.0075 0.2275 537409 outcome TRUE reported 12528_40_WRNIP1_WRIP1
WRNIP1 chr1 196695521 0.008148 3.199e-52 35329 TRUE reported
textfile 2 1 TRUE
8732 rs1706435 G A G A 0.0634 0.0117 0.36981 NA FALSE FALSE FALSE
GCST006061 0.0072 0.1047 537409 outcome TRUE reported 12528_40_WRNIP1_WRIP1
WRNIP1 chr3 165743688 0.008261 1.664e-14 35330 TRUE reported
textfile 2 1 TRUE
8733 rs10922098 T C T C -0.1313 -0.009 0.40011 NA FALSE FALSE FALSE
GCST006061 0.0075 0.2275 537409 outcome TRUE reported 7249_307_BCL2L10_B2L10
BCL2L10 chr1 196695521 0.008139 1.531e-58 35338 TRUE reported
textfile 2 1 TRUE
8734 rs1707652 T C T C 0.0718 0.0081 0.3102 NA FALSE FALSE FALSE
GCST006061 0.0074 0.2701 537409 outcome TRUE reported 7249_307_BCL2L10_B2L10
BCL2L10 chr3 165761011 0.008608 7.333e-17 35339 TRUE reported
textfile 2 1 TRUE
8735 rs10922098 T C T C -0.1163 -0.009 0.40011 NA FALSE FALSE FALSE
GCST006061 0.0075 0.2275 537409 outcome TRUE reported 8104_21_IL10RA_IL_10_Ra
IL10RA chr1 196695521 0.008014 1.005e-47 35338 TRUE reported
textfile 2 1 TRUE
8736 rs10922098 T C T C -0.0764 -0.009 0.40011 NA FALSE FALSE FALSE
GCST006061 0.0075 0.2275 537409 outcome TRUE reported 11678_105_GJD2_CXD2
GJD2 chr1 196695521 0.008056 2.461e-21 35329 TRUE reported
textfile 2 1 TRUE
8737 rs34793466 C T C T 0.0587 0.0074 0.16907 NA FALSE FALSE FALSE
GCST006061 0.0121 0.5398 537409 outcome TRUE reported 11678_105_GJD2_CXD2
GJD2 chr14 106540249 0.010762 4.912e-08 34957 TRUE reported
textfile 2 1 TRUE
8738 rs6445035 A G A G 0.0647 0.0154 0.1904 NA FALSE FALSE FALSE
GCST006061 0.0089 0.08256 537409 outcome TRUE reported 11678_105_GJD2_CXD2
GJD2 chr3 165762312 0.010017 1.056e-10 35330 TRUE reported
textfile 2 1 TRUE
8739 rs8077564 G A G A -0.0645 -0.0051 0.30355 NA FALSE FALSE FALSE
GCST006061 0.0078 0.5175 537409 outcome TRUE reported 11678_105_GJD2_CXD2
GJD2 chr17 66217812 0.008574 5.37e-14 35360 TRUE reported
textfile 2 1 TRUE
8740 rs10922098 T C T C -0.0965 -0.009 0.40011 NA FALSE FALSE FALSE
GCST006061 0.0075 0.2275 537409 outcome TRUE reported 9175_48_DSCAM_DSCAM
DSCAM chr1 196695521 0.008228 9.111e-32 35338 TRUE reported
textfile 2 1 TRUE
8741 rs1355538 G A G A -0.0531 -0.0076 0.39847 NA FALSE FALSE FALSE
GCST006061 0.0072 0.2923 537409 outcome TRUE reported 9175_48_DSCAM_DSCAM
DSCAM chr3 165787389 0.008222 1.058e-10 35338 TRUE reported
textfile 2 1 TRUE
8742 rs6517602 G T G T -0.0588 0.0087 0.36971 NA FALSE FALSE FALSE
GCST006061 0.0073 0.2363 537409 outcome TRUE reported 9175_48_DSCAM_DSCAM
DSCAM chr21 40613822 0.008345 1.838e-12 35365 TRUE reported
textfile 2 1 TRUE
8743 rs78111814 C T C T 0.3257 -0.022 0.05813 NA FALSE FALSE FALSE
GCST006061 0.0188 0.2432 537409 outcome TRUE reported 9175_48_DSCAM_DSCAM
DSCAM chr21 40688572 0.017102 7.311e-81 35363 TRUE reported
textfile 2 1 TRUE

8744 rs10922098 T C T C -0.0988 -0.009 0.40011 NA FALSE FALSE FALSE
GCST006061 0.0075 0.2275 537409 outcome TRUE reported 2962_50_PTHLH_PTHrP
PTHLH chr1 196695521 0.008064 1.65e-34 35338 TRUE reported
textfile 2 1 TRUE
8745 rs11049365 A C A C 0.1225 -0.0142 0.27057 NA FALSE FALSE FALSE
GCST006061 0.008 0.07592 537409 outcome TRUE reported 2962_50_PTHLH_PTHrP
PTHLH chr12 28138401 0.008925 7.177e-43 35354 TRUE reported
textfile 2 1 TRUE
8746 rs1355538 G A G A -0.0444 -0.0076 0.39847 NA FALSE FALSE FALSE
GCST006061 0.0072 0.2923 537409 outcome TRUE reported 2962_50_PTHLH_PTHrP
PTHLH chr3 165787389 0.008059 3.606e-08 35338 TRUE reported
textfile 2 1 TRUE
8747 rs33092 A G A G -0.0518 0.006 0.46132 NA FALSE FALSE FALSE GCST006061
0.0072 0.4047 537409 outcome TRUE reported 2962_50_PTHLH_PTHrP PTHLH chr12
27973459 0.007938 6.792e-11 35353 TRUE reported textfile 2 1 TRUE
8748 rs10922098 T C T C -0.0799 -0.009 0.40011 NA FALSE FALSE FALSE
GCST006061 0.0075 0.2275 537409 outcome TRUE reported 13041_47_TRAF4_TRAF4
TRAF4 chr1 196695521 0.00805 3.214e-23 35329 TRUE reported textfile
2 1 TRUE
8749 rs10922098 T C T C -0.1682 -0.009 0.40011 NA FALSE FALSE FALSE
GCST006061 0.0075 0.2275 537409 outcome TRUE reported 12991_49_CUL9_CUL9
CUL9 chr1 196695521 0.00807 1.798e-96 35329 TRUE reported textfile
2 1 TRUE
8750 rs11569415 A G A G -0.0544 0.0011 0.24276 NA FALSE FALSE FALSE
GCST006061 0.0097 0.9129 537409 outcome TRUE reported 12991_49_CUL9_CUL9
CUL9 chr19 6716268 0.009199 3.351e-09 35363 TRUE reported textfile
2 1 TRUE
8751 rs12403425 A C A C 0.05 -0.0064 0.2662 NA FALSE FALSE FALSE
GCST006061 0.009 0.4754 537409 outcome TRUE reported 12991_49_CUL9_CUL9
CUL9 chr1 197845693 0.008948 2.301e-08 35328 TRUE reported
textfile 2 1 TRUE
8752 rs1355538 G A G A -0.0722 -0.0076 0.39847 NA FALSE FALSE FALSE
GCST006061 0.0072 0.2923 537409 outcome TRUE reported 12991_49_CUL9_CUL9
CUL9 chr3 165787389 0.00808 4.031e-19 35329 TRUE reported textfile
2 1 TRUE
8753 rs10922098 T C T C -0.0461 -0.009 0.40011 NA FALSE FALSE FALSE
GCST006061 0.0075 0.2275 537409 outcome TRUE reported 12350_86_ETS2_ETS2
ETS2 chr1 196695521 0.00833 3.123e-08 35329 TRUE reported textfile
2 1 TRUE
8754 rs141618758 C T C T -0.2552 -0.0803 0.02633 NA FALSE FALSE FALSE
GCST006061 0.0291 0.005819 537409 outcome TRUE reported 12350_86_ETS2_ETS2
ETS2 chr19 11146823 0.025128 3.114e-24 35373 TRUE reported
textfile 2 1 TRUE
8755 rs143973988 T C T C -0.166 0.0912 0.02112 NA FALSE FALSE FALSE
GCST006061 0.0555 0.1003 537409 outcome TRUE reported 12350_86_ETS2_ETS2
ETS2 chr19 8487569 0.028322 4.599e-09 35366 TRUE reported textfile
2 1 TRUE
8756 rs16997771 C T C T -0.3085 -0.0449 0.03104 NA FALSE FALSE FALSE
GCST006061 0.0243 0.06477 537409 outcome TRUE reported 12350_86_ETS2_ETS2
ETS2 chr19 10038037 0.023651 6.902e-39 35365 TRUE reported
textfile 2 1 TRUE
8757 rs6542680 T C T C -0.2418 -0.0012 0.18362 NA FALSE FALSE FALSE
GCST006061 0.0091 0.8935 537409 outcome TRUE reported 12350_86_ETS2_ETS2
ETS2 chr2 3592552 0.010488 1.313e-117 35323 TRUE reported textfile
2 1 TRUE
8758 rs74625263 T C T C -0.3599 0.0133 0.01488 NA FALSE FALSE FALSE
GCST006061 0.0612 0.828 537409 outcome TRUE reported 12350_86_ETS2_ETS2
ETS2 chr19 9968128 0.032999 1.075e-27 35363 TRUE reported textfile
2 1 TRUE
8759 rs10922098 T C T C -0.0731 -0.009 0.40011 NA FALSE FALSE FALSE
GCST006061 0.0075 0.2275 537409 outcome TRUE reported 11934_9_INTS3_INT3
INTS3 chr1 196695521 0.007978 5.045e-20 35329 TRUE reported
textfile 2 1 TRUE
8760 rs13379369 G A G A -0.0833 0.0108 0.15348 NA FALSE FALSE FALSE
GCST006061 0.0182 0.5534 537409 outcome TRUE reported 11934_9_INTS3_INT3
INTS3 chr14 105863077 0.011095 6.011e-14 34957 TRUE reported

textfile 2 1 TRUE
8761 rs10922098 T C T C -0.1036 -0.009 0.40011 NA FALSE FALSE FALSE
GCST006061 0.0075 0.2275 537409 outcome TRUE reported 5410_53_CDH15_CAD15
CDH15 chr1 196695521 0.008097 1.752e-37 35338 TRUE reported
textfile 2 1 TRUE
8762 rs6445035 A G A G 0.0712 0.0154 0.1904 NA FALSE FALSE FALSE
GCST006061 0.0089 0.08256 537409 outcome TRUE reported 5410_53_CDH15_CAD15
CDH15 chr3 165762312 0.010079 1.618e-12 35339 TRUE reported
textfile 2 1 TRUE
8763 rs10922098 T C T C -0.1632 -0.009 0.40011 NA FALSE FALSE FALSE
GCST006061 0.0075 0.2275 537409 outcome TRUE reported 12894_3_PAXIP1_PAXI1
PAXIP1 chr1 196695521 0.008112 5.19e-90 35329 TRUE reported
textfile 2 1 TRUE
8764 rs1291921 G A G A 0.0495 -0.0157 0.2763 NA FALSE FALSE FALSE
GCST006061 0.0081 0.05326 537409 outcome TRUE reported 12894_3_PAXIP1_PAXI1
PAXIP1 chr3 136317384 0.00894 3.074e-08 35352 TRUE reported textfile
2 1 TRUE
8765 rs1555405 A G A G -0.0485 -0.0047 0.30381 NA FALSE FALSE FALSE
GCST006061 0.0081 0.5592 537409 outcome TRUE reported 12894_3_PAXIP1_PAXI1
PAXIP1 chr14 100710432 0.008702 2.495e-08 35371 TRUE reported
textfile 2 1 TRUE
8766 rs1706435 G A G A 0.0781 0.0117 0.36981 NA FALSE FALSE FALSE
GCST006061 0.0072 0.1047 537409 outcome TRUE reported 12894_3_PAXIP1_PAXI1
PAXIP1 chr3 165743688 0.008247 2.806e-21 35330 TRUE reported
textfile 2 1 TRUE
8767 rs10922098 T C T C -0.062 -0.009 0.40011 NA FALSE FALSE FALSE
GCST006061 0.0075 0.2275 537409 outcome TRUE reported 6627_25_PNLIPRP1_LIPR1
PNLIPRP1 chr1 196695521 0.008408 1.656e-13 35338 TRUE reported
textfile 2 1 TRUE
8768 rs111871876 C T C T 0.1321 9e-04 0.04746 NA FALSE FALSE FALSE
GCST006061 0.0274 0.9746 537409 outcome TRUE reported 6627_25_PNLIPRP1_LIPR1
PNLIPRP1 chr10 115073926 0.019569 1.475e-11 35381 TRUE reported
textfile 2 1 TRUE
8769 rs1153021 A G A G -0.0891 0.0023 0.0789 NA FALSE FALSE FALSE
GCST006061 0.0146 0.8721 537409 outcome TRUE reported 6627_25_PNLIPRP1_LIPR1
PNLIPRP1 chr16 75684574 0.015429 7.711e-09 35362 TRUE reported
textfile 2 1 TRUE
8770 rs11724862 C T C T -0.0767 0.0096 0.21985 NA FALSE FALSE FALSE
GCST006061 0.0093 0.298 537409 outcome TRUE reported 6627_25_PNLIPRP1_LIPR1
PNLIPRP1 chr4 185651349 0.009924 1.083e-14 35383 TRUE reported
textfile 2 1 TRUE
8771 rs12249434 T C T C 0.1357 0.0088 0.07689 NA FALSE FALSE FALSE
GCST006061 0.0131 0.5011 537409 outcome TRUE reported 6627_25_PNLIPRP1_LIPR1
PNLIPRP1 chr10 116539194 0.015393 1.192e-18 35376 TRUE reported
textfile 2 1 TRUE
8772 rs144395013 G A G A -0.2105 -0.0112 0.02669 NA FALSE FALSE FALSE
GCST006061 0.0261 0.669 537409 outcome TRUE reported 6627_25_PNLIPRP1_LIPR1
PNLIPRP1 chr16 76541936 0.026307 1.228e-15 35357 TRUE reported
textfile 2 1 TRUE
8773 rs1591805 G A G A -0.0621 -7.00E-04 0.4704 NA FALSE FALSE FALSE
GCST006061 0.0074 0.9288 537409 outcome TRUE reported 6627_25_PNLIPRP1_LIPR1
PNLIPRP1 chr6 126395918 0.008243 4.917e-14 35356 TRUE reported
textfile 2 1 TRUE
8774 rs16950178 T C T C -0.0698 0.0033 0.24286 NA FALSE FALSE FALSE
GCST006061 0.0082 0.6866 537409 outcome TRUE reported 6627_25_PNLIPRP1_LIPR1
PNLIPRP1 chr16 74914280 0.00964 4.459e-13 35363 TRUE reported
textfile 2 1 TRUE
8775 rs2277710 C T C T 0.0891 -0.0077 0.06862 NA FALSE FALSE FALSE
GCST006061 0.0134 0.5632 537409 outcome TRUE reported 6627_25_PNLIPRP1_LIPR1
PNLIPRP1 chr18 44792522 0.016304 4.631e-08 35349 TRUE reported
textfile 2 1 TRUE
8776 rs2915766 T C T C 0.1466 0.0022 0.47392 NA FALSE FALSE FALSE
GCST006061 0.0074 0.7682 537409 outcome TRUE reported 6627_25_PNLIPRP1_LIPR1
PNLIPRP1 chr10 116593741 0.008283 4.352e-70 35379 TRUE reported
textfile 2 1 TRUE
8777 rs507695 T C T C -0.0708 0.0013 0.2959 NA FALSE FALSE FALSE

GCST006061 0.0079 0.8663 537409 outcome TRUE reported 6627_25_PNLIPRP1_LIPR1
PNLIPRP1 chr11 100776205 0.008961 2.777e-15 35356 TRUE reported
textfile 2 1 TRUE
8778 rs66473811 C T C T 0.0685 0.0089 0.1675 NA FALSE FALSE FALSE
GCST006061 0.0097 0.358 537409 outcome TRUE reported 6627_25_PNLIPRP1_LIPR1
PNLIPRP1 chr3 64014788 0.011005 4.839e-10 35358 TRUE reported
textfile 2 1 TRUE
8779 rs72704449 C T C T 0.1194 0.013 0.04582 NA FALSE FALSE FALSE
GCST006061 0.0199 0.5128 537409 outcome TRUE reported 6627_25_PNLIPRP1_LIPR1
PNLIPRP1 chr1 179504138 0.019593 1.102e-09 35358 TRUE reported
textfile 2 1 TRUE
8780 rs72802342 A C A C -0.5856 0.0024 0.06858 NA FALSE FALSE FALSE
GCST006061 0.0147 0.8687 537409 outcome TRUE reported 6627_25_PNLIPRP1_LIPR1
PNLIPRP1 chr16 75200974 0.016343 1e-200 35363 TRUE reported
textfile 2 1 TRUE
8781 rs748208 C T C T -0.0585 -0.0201 0.35761 NA FALSE FALSE FALSE
GCST006061 0.0082 0.01395 537409 outcome TRUE reported 6627_25_PNLIPRP1_LIPR1
PNLIPRP1 chr8 18855003 0.008564 8.45e-12 35375 TRUE reported
textfile 2 1 TRUE
8782 rs7803628 G T G T 0.097 0.0012 0.0731 NA FALSE FALSE FALSE
GCST006061 0.0187 0.9468 537409 outcome TRUE reported 6627_25_PNLIPRP1_LIPR1
PNLIPRP1 chr7 50957086 0.015974 1.259e-09 35339 TRUE reported
textfile 2 1 TRUE
8783 rs8022656 G A G A -0.061 0.0032 0.41088 NA FALSE FALSE FALSE
GCST006061 0.0073 0.6597 537409 outcome TRUE reported 6627_25_PNLIPRP1_LIPR1
PNLIPRP1 chr14 100841205 0.00837 3.151e-13 35378 TRUE reported
textfile 2 1 TRUE
8784 rs10922098 T C T C -0.1055 -0.009 0.40011 NA FALSE FALSE FALSE
GCST006061 0.0075 0.2275 537409 outcome TRUE reported 9287_6_SPR_SPRE SPR
chr1 196695521 0.008008 1.225e-39 35338 TRUE reported textfile 2
1 TRUE
8785 rs1355538 G A G A -0.0667 -0.0076 0.39847 NA FALSE FALSE FALSE
GCST006061 0.0072 0.2923 537409 outcome TRUE reported 9287_6_SPR_SPRE SPR
chr3 165787389 0.008008 8.164e-17 35338 TRUE reported textfile 2
1 TRUE
8786 rs10922098 T C T C -0.137 -0.009 0.40011 NA FALSE FALSE FALSE
GCST006061 0.0075 0.2275 537409 outcome TRUE reported 17817_22_DDIT4_DDIT4
DDIT4 chr1 196695521 0.008095 2.973e-64 35338 TRUE reported
textfile 2 1 TRUE
8787 rs1707652 T C T C 0.0609 0.0081 0.3102 NA FALSE FALSE FALSE
GCST006061 0.0074 0.2701 537409 outcome TRUE reported 17817_22_DDIT4_DDIT4
DDIT4 chr3 165761011 0.008568 1.177e-12 35339 TRUE reported
textfile 2 1 TRUE
8788 rs10922098 T C T C -0.0885 -0.009 0.40011 NA FALSE FALSE FALSE
GCST006061 0.0075 0.2275 537409 outcome TRUE reported 10535_25_COX6A2_CX6A2
COX6A2 chr1 196695521 0.007979 1.372e-28 35326 TRUE reported
textfile 2 1 TRUE
8789 rs11845744 A C A C -0.102 -0.0155 0.11007 NA FALSE FALSE FALSE
GCST006061 0.0192 0.4189 537409 outcome TRUE reported 10535_25_COX6A2_CX6A2
COX6A2 chr14 105921449 0.012889 2.504e-15 34955 TRUE reported
textfile 2 1 TRUE
8790 rs10922098 T C T C -0.1026 -0.009 0.40011 NA FALSE FALSE FALSE
GCST006061 0.0075 0.2275 537409 outcome TRUE reported 7792_58_CCDC90B_CC90B
CCDC90B chr1 196695521 0.008019 1.741e-37 35338 TRUE reported
textfile 2 1 TRUE
8791 rs6542680 T C T C -0.0653 -0.0012 0.18362 NA FALSE FALSE FALSE
GCST006061 0.0091 0.8935 537409 outcome TRUE reported 7792_58_CCDC90B_CC90B
CCDC90B chr2 3592552 0.01017 1.353e-10 35332 TRUE reported textfile 2
1 TRUE
8792 rs10922098 T C T C -0.1115 -0.009 0.40011 NA FALSE FALSE FALSE
GCST006061 0.0075 0.2275 537409 outcome TRUE reported 9964_10_MYZAP_MYZAP
MYZAP chr1 196695521 0.007926 6.003e-45 35338 TRUE reported
textfile 2 1 TRUE
8793 rs1564943 T C T C 0.0451 0.0073 0.33715 NA FALSE FALSE FALSE
GCST006061 0.0075 0.3258 537409 outcome TRUE reported 9964_10_MYZAP_MYZAP
MYZAP chr11 12157560 0.008236 4.344e-08 35376 TRUE reported

textfile 2 1 TRUE
8794 rs1619994 G A G A 0.0627 0.0168 0.2807 NA FALSE FALSE FALSE GCST006061 0.0078 0.03057 537409 outcome TRUE reported 9964_10_MYZAP_MYZAP MYZAP chr3 165744047 0.008654 4.324e-13 35339 TRUE reported textfile 2 1 TRUE
8795 rs10922098 T C T C -0.159 -0.009 0.40011 NA FALSE FALSE FALSE GCST006061 0.0075 0.2275 537409 outcome TRUE reported 2826_53_EDA_EDA EDA chr1 196695521 0.008307 1.186e-81 35338 TRUE reported textfile 2 1 TRUE
8796 rs2686395 C T C T -0.0649 -0.0077 0.3101 NA FALSE FALSE FALSE GCST006061 0.0074 0.2965 537409 outcome TRUE reported 2826_53_EDA_EDA EDA chr3 165767231 0.0088 1.645e-13 35339 TRUE reported textfile 2 1 TRUE
8797 rs10922098 T C T C -0.2001 -0.009 0.40011 NA FALSE FALSE FALSE GCST006061 0.0075 0.2275 537409 outcome TRUE reported 17475_18_PBK_TOPK PBK chr1 196695521 0.008159 8.102e-133 35338 TRUE reported textfile 2 1 TRUE
8798 rs111696008 A G A G -0.1506 -0.0259 0.02443 NA FALSE FALSE FALSE GCST006061 0.0401 0.519 537409 outcome TRUE reported 17475_18_PBK_TOPK PBK chr2 216622986 0.026683 1.662e-08 35359 TRUE reported textfile 2 1 TRUE
8799 rs1707652 T C T C 0.1151 0.0081 0.3102 NA FALSE FALSE FALSE GCST006061 0.0074 0.2701 537409 outcome TRUE reported 17475_18_PBK_TOPK PBK chr3 165761011 0.008651 2.167e-40 35339 TRUE reported textfile 2 1 TRUE
8800 rs704 A G A G 0.0519 -0.0155 0.49189 NA FALSE FALSE FALSE GCST006061 0.0072 0.03144 537409 outcome TRUE reported 17475_18_PBK_TOPK PBK chr17 28367840 0.008044 1.104e-10 35360 TRUE reported textfile 2 1 TRUE
8801 rs10922098 T C T C -0.1265 -0.009 0.40011 NA FALSE FALSE FALSE GCST006061 0.0075 0.2275 537409 outcome TRUE reported 8062_15_EVA1B_F176B EVA1B chr1 196695521 0.008039 8.579e-56 35338 TRUE reported textfile 2 1 TRUE
8802 rs10922098 T C T C -0.1013 -0.009 0.40011 NA FALSE FALSE FALSE GCST006061 0.0075 0.2275 537409 outcome TRUE reported 5714_88_IFNA6_IFNA6 IFNA6 chr1 196695521 0.008134 1.322e-35 35338 TRUE reported textfile 2 1 TRUE
8803 rs10922098 T C T C -0.0438 -0.009 0.40011 NA FALSE FALSE FALSE GCST006061 0.0075 0.2275 537409 outcome TRUE reported 14111_15_THBS2_TSP2 THBS2 chr1 196695521 0.007972 3.922e-08 35338 TRUE reported textfile 2 1 TRUE
8804 rs73041853 A G A G 0.1138 0.0017 0.13852 NA FALSE FALSE FALSE GCST006061 0.0127 0.8923 537409 outcome TRUE reported 14111_15_THBS2_TSP2 THBS2 chr6 169203903 0.011333 1.002e-23 35020 TRUE reported textfile 2 1 TRUE
8805 rs10922098 T C T C -0.0547 -0.009 0.40011 NA FALSE FALSE FALSE GCST006061 0.0075 0.2275 537409 outcome TRUE reported 10464_6_ANTXR1_ANTR1 ANTXR1 chr1 196695521 0.008562 1.67e-10 35326 TRUE reported textfile 2 1 TRUE
8806 rs13329672 T C T C 0.0633 3e-04 0.26556 NA FALSE FALSE FALSE GCST006061 0.0083 0.9682 537409 outcome TRUE reported 10464_6_ANTXR1_ANTR1 ANTXR1 chr15 58407738 0.009478 2.409e-11 35352 TRUE reported textfile 2 1 TRUE
8807 rs17410962 A G A G 0.0927 0.0174 0.09642 NA FALSE FALSE FALSE GCST006061 0.0108 0.1078 537409 outcome TRUE reported 10464_6_ANTXR1_ANTR1 ANTXR1 chr8 19990569 0.014092 4.762e-11 35356 TRUE reported textfile 2 1 TRUE
8808 rs2686395 C T C T -0.098 -0.0077 0.3101 NA FALSE FALSE FALSE GCST006061 0.0074 0.2965 537409 outcome TRUE reported 10464_6_ANTXR1_ANTR1 ANTXR1 chr3 165767231 0.009037 2.125e-27 35327 TRUE reported textfile 2 1 TRUE
8809 rs3764261 A C A C 0.1395 0.0132 0.32148 NA FALSE FALSE FALSE GCST006061 0.0076 0.08361 537409 outcome TRUE reported 10464_6_ANTXR1_ANTR1 ANTXR1 chr16 56959412 0.008936 6.2e-55 35357 TRUE reported textfile 2 1 TRUE
8810 rs7424376 G A G A 0.0474 -0.0128 0.40051 NA FALSE FALSE FALSE GCST006061 0.0074 0.08193 537409 outcome TRUE reported 10464_6_ANTXR1_ANTR1

ANTXR1 chr2 69298791 0.008552 2.98e-08 35350 TRUE reported
textfile 2 1 TRUE
8811 rs10922098 T C T C -0.0697 -0.009 0.40011 NA FALSE FALSE FALSE
GCST006061 0.0075 0.2275 537409 outcome TRUE reported 8825_4_PILRA_PILRA
PILRA chr1 196695521 0.008077 6.143e-18 35338 TRUE reported
textfile 2 1 TRUE
8812 rs115955722 G A G A -0.1705 0.1209 0.01939 NA FALSE FALSE FALSE
GCST006061 0.0435 0.005492 537409 outcome TRUE reported 8825_4_PILRA_PILRA
PILRA chr1 170080428 0.029069 4.48e-09 35361 TRUE reported
textfile 2 1 TRUE
8813 rs12127364 A C A C -0.2707 -0.006 0.3095 NA FALSE FALSE FALSE
GCST006061 0.0081 0.4569 537409 outcome TRUE reported 8825_4_PILRA_PILRA
PILRA chr1 169499904 0.008447 1e-200 35363 TRUE reported textfile
2 1 TRUE
8814 rs10922098 T C T C -0.1372 -0.009 0.40011 NA FALSE FALSE FALSE
GCST006061 0.0075 0.2275 537409 outcome TRUE reported 17855_28_RGS4_RGS4
RGS4 chr1 196695521 0.008026 1.661e-65 35338 TRUE reported
textfile 2 1 TRUE
8815 rs1355538 G A G A -0.0667 -0.0076 0.39847 NA FALSE FALSE FALSE
GCST006061 0.0072 0.2923 537409 outcome TRUE reported 17855_28_RGS4_RGS4
RGS4 chr3 165787389 0.008038 1.058e-16 35338 TRUE reported
textfile 2 1 TRUE
8816 rs10922098 T C T C -0.079 -0.009 0.40011 NA FALSE FALSE FALSE
GCST006061 0.0075 0.2275 537409 outcome TRUE reported 15495_9_FOLR3_FOLR3
FOLR3 chr1 196695521 0.00737 8.246e-27 35338 TRUE reported textfile
2 1 TRUE
8817 rs111490736 C T C T 0.2405 -0.0171 0.01151 NA FALSE FALSE FALSE
GCST006061 0.0267 0.5237 537409 outcome TRUE reported 15495_9_FOLR3_FOLR3
FOLR3 chr11 66879255 0.037346 1.197e-10 35363 TRUE reported
textfile 2 1 TRUE
8818 rs112240808 G A G A 0.4663 0.0132 0.01745 NA FALSE FALSE FALSE
GCST006061 0.0312 0.6729 537409 outcome TRUE reported 15495_9_FOLR3_FOLR3
FOLR3 chr11 75585682 0.030811 9.608e-52 35372 TRUE reported
textfile 2 1 TRUE
8819 rs11230803 T C T C 0.2042 -0.0635 0.02222 NA FALSE FALSE FALSE
GCST006061 0.0318 0.04626 537409 outcome TRUE reported 15495_9_FOLR3_FOLR3
FOLR3 chr11 61832519 0.02773 1.785e-13 35367 TRUE reported textfile
2 1 TRUE
8820 rs117976804 A G A G 0.1373 -0.0028 0.03799 NA FALSE FALSE FALSE
GCST006061 0.0317 0.9305 537409 outcome TRUE reported 15495_9_FOLR3_FOLR3
FOLR3 chr11 69082133 0.021216 9.694e-11 35366 TRUE reported
textfile 2 1 TRUE
8821 rs138312560 C A C A 0.2441 -0.0403 0.01138 NA FALSE FALSE FALSE
GCST006061 0.0435 0.355 537409 outcome TRUE reported 15495_9_FOLR3_FOLR3
FOLR3 chr11 82604216 0.037021 4.294e-11 35342 TRUE reported
textfile 2 1 TRUE
8822 rs1623476 A G A G 0.3494 -0.0071 0.04967 NA FALSE FALSE FALSE
GCST006061 0.017 0.6749 537409 outcome TRUE reported 15495_9_FOLR3_FOLR3
FOLR3 chr11 73870179 0.018572 5.858e-79 35370 TRUE reported
textfile 2 1 TRUE
8823 rs17748438 A G A G 0.1808 -0.0267 0.01744 NA FALSE FALSE FALSE
GCST006061 0.0358 0.4566 537409 outcome TRUE reported 15495_9_FOLR3_FOLR3
FOLR3 chr11 75998091 0.03126 7.305e-09 35375 TRUE reported textfile
2 1 TRUE
8824 rs34300064 G T G T 0.1155 -0.0031 0.05894 NA FALSE FALSE FALSE
GCST006061 0.0178 0.8636 537409 outcome TRUE reported 15495_9_FOLR3_FOLR3
FOLR3 chr11 78241932 0.017309 2.511e-11 35366 TRUE reported
textfile 2 1 TRUE
8825 rs61895676 T C T C 0.5926 -0.0184 0.0152 NA FALSE FALSE FALSE
GCST006061 0.0437 0.6738 537409 outcome TRUE reported 15495_9_FOLR3_FOLR3
FOLR3 chr11 72467660 0.033385 1.701e-70 35371 TRUE reported
textfile 2 1 TRUE
8826 rs704 A G A G 0.0454 -0.0155 0.49189 NA FALSE FALSE FALSE GCST006061
0.0072 0.03144 537409 outcome TRUE reported 15495_9_FOLR3_FOLR3 FOLR3 chr17
28367840 0.007218 3.179e-10 35360 TRUE reported textfile 2 1 TRUE
8827 rs72983223 C T C T -0.1284 -0.0028 0.04078 NA FALSE FALSE FALSE

GCST006061 0.0254 0.9126 537409 outcome TRUE reported 15495_9_FOLR3_FOLR3 FOLR3 chr11 74648235 0.02112 1.205e-09 35370 TRUE reported textfile 2 1 TRUE

8828 rs73504617 C T C T 0.2382 -0.0752 0.01739 NA FALSE FALSE FALSE GCST006061 0.0412 0.06781 537409 outcome TRUE reported 15495_9_FOLR3_FOLR3 FOLR3 chr11 79017024 0.031217 2.341e-14 35365 TRUE reported textfile 2 1 TRUE

8829 rs75926779 A G A G 0.3171 0.0021 0.12292 NA FALSE FALSE FALSE GCST006061 0.0148 0.885 537409 outcome TRUE reported 15495_9_FOLR3_FOLR3 FOLR3 chr11 72601925 0.012357 3.153e-145 35371 TRUE reported textfile 2 1 TRUE

8830 rs77803271 C T C T -0.1573 5e-04 0.03043 NA FALSE FALSE FALSE GCST006061 0.0219 0.9822 537409 outcome TRUE reported 15495_9_FOLR3_FOLR3 FOLR3 chr11 72548556 0.023843 4.189e-11 35373 TRUE reported textfile 2 1 TRUE

8831 rs78672796 C T C T 0.7275 0.0391 0.03056 NA FALSE FALSE FALSE GCST006061 0.0255 0.1253 537409 outcome TRUE reported 15495_9_FOLR3_FOLR3 FOLR3 chr11 71135189 0.023121 1e-200 35378 TRUE reported textfile 2 1 TRUE

8832 rs10922098 T C T C -0.0817 -0.009 0.40011 NA FALSE FALSE FALSE GCST006061 0.0075 0.2275 537409 outcome TRUE reported 12784_10_APBB3_APBB3 APBB3 chr1 196695521 0.007951 9.09e-25 35329 TRUE reported textfile 2 1 TRUE

8833 rs704 A G A G 0.078 -0.0155 0.49189 NA FALSE FALSE FALSE GCST006061 0.0072 0.03144 537409 outcome TRUE reported 12784_10_APBB3_APBB3 APBB3 chr17 28367840 0.007792 1.37e-23 35351 TRUE reported textfile 2 1 TRUE

8834 rs10922098 T C T C -0.0957 -0.009 0.40011 NA FALSE FALSE FALSE GCST006061 0.0075 0.2275 537409 outcome TRUE reported 19302_7_GIMAP6_GIMA6 GIMAP6 chr1 196695521 0.008053 1.444e-32 35338 TRUE reported textfile 2 1 TRUE

8835 rs10922098 T C T C -0.0491 -0.009 0.40011 NA FALSE FALSE FALSE GCST006061 0.0075 0.2275 537409 outcome TRUE reported 9265_10_GLIPR1_GLIP1 GLIPR1 chr1 196695521 0.008093 1.306e-09 35338 TRUE reported textfile 2 1 TRUE

8836 rs12422952 T C T C -0.0859 -0.0039 0.3989 NA FALSE FALSE FALSE GCST006061 0.0072 0.5872 537409 outcome TRUE reported 9265_10_GLIPR1_GLIP1 GLIPR1 chr12 75487238 0.008092 2.529e-26 35371 TRUE reported textfile 2 1 TRUE

8837 rs12608549 T C T C 0.2132 -0.0068 0.01271 NA FALSE FALSE FALSE GCST006061 0.0217 0.7534 537409 outcome TRUE reported 9265_10_GLIPR1_GLIP1 GLIPR1 chr19 57651769 0.034654 7.641e-10 35165 TRUE reported textfile 2 1 TRUE

8838 rs10922098 T C T C -0.0632 -0.009 0.40011 NA FALSE FALSE FALSE GCST006061 0.0075 0.2275 537409 outcome TRUE reported 12664_19_PTPN13_PTN13 PTPN13 chr1 196695521 0.008048 4.054e-15 35329 TRUE reported textfile 2 1 TRUE

8839 rs10922098 T C T C -0.1496 -0.009 0.40011 NA FALSE FALSE FALSE GCST006061 0.0075 0.2275 537409 outcome TRUE reported 7054_87_CLEC2D_CLC2D CLEC2D chr1 196695521 0.008124 1.005e-75 35338 TRUE reported textfile 2 1 TRUE

8840 rs1706435 G A G A 0.0703 0.0117 0.36981 NA FALSE FALSE FALSE GCST006061 0.0072 0.1047 537409 outcome TRUE reported 7054_87_CLEC2D_CLC2D CLEC2D chr3 165743688 0.008252 1.607e-17 35339 TRUE reported textfile 2 1 TRUE

8841 rs10922098 T C T C -0.1324 -0.009 0.40011 NA FALSE FALSE FALSE GCST006061 0.0075 0.2275 537409 outcome TRUE reported 8870_38_NPM1_NPM1 NPM1 chr1 196695521 0.008079 2.29e-60 35338 TRUE reported textfile 2 1 TRUE

8842 rs1707652 T C T C 0.078 0.0081 0.3102 NA FALSE FALSE FALSE GCST006061 0.0074 0.2701 537409 outcome TRUE reported 8870_38_NPM1_NPM1 NPM1 chr3 165761011 0.008551 7.357e-20 35339 TRUE reported textfile 2 1 TRUE

8843 rs704 A G A G 0.0461 -0.0155 0.49189 NA FALSE FALSE FALSE GCST006061 0.0072 0.03144 537409 outcome TRUE reported 8870_38_NPM1_NPM1 NPM1 chr17 28367840 0.007941 6.416e-09 35360 TRUE reported textfile 2 1 TRUE

8844 rs10922098 T C T C -0.2438 -0.009 0.40011 NA FALSE FALSE FALSE
GCST006061 0.0075 0.2275 537409 outcome TRUE reported 6994_19_C3orf18_CC018
C3orf18 chr1 196695521 0.008156 2.373e-196 35338 TRUE reported
textfile 2 1 TRUE

8845 rs111696008 A G A G -0.147 -0.0259 0.02443 NA FALSE FALSE FALSE
GCST006061 0.0401 0.519 537409 outcome TRUE reported 6994_19_C3orf18_CC018
C3orf18 chr2 216622986 0.02673 3.811e-08 35359 TRUE reported textfile
2 1 TRUE

8846 rs2686395 C T C T -0.1235 -0.0077 0.3101 NA FALSE FALSE FALSE
GCST006061 0.0074 0.2965 537409 outcome TRUE reported 6994_19_C3orf18_CC018
C3orf18 chr3 165767231 0.008663 4.097e-46 35339 TRUE reported
textfile 2 1 TRUE

8847 rs10922098 T C T C -0.1102 -0.009 0.40011 NA FALSE FALSE FALSE
GCST006061 0.0075 0.2275 537409 outcome TRUE reported 19567_1_EGFR_EGFRvIII
EGFR chr1 196695521 0.007989 2.742e-43 35338 TRUE reported
textfile 2 1 TRUE

8848 rs13379369 G A G A -0.0845 0.0108 0.15348 NA FALSE FALSE FALSE
GCST006061 0.0182 0.5534 537409 outcome TRUE reported 19567_1_EGFR_EGFRvIII
EGFR chr14 105863077 0.011117 2.942e-14 34966 TRUE reported
textfile 2 1 TRUE

8849 rs1619994 G A G A 0.0491 0.0168 0.2807 NA FALSE FALSE FALSE
GCST006061 0.0078 0.03057 537409 outcome TRUE reported 19567_1_EGFR_EGFRvIII
EGFR chr3 165744047 0.00872 1.797e-08 35339 TRUE reported textfile
2 1 TRUE

8850 rs2753537 T C T C -0.142 -0.0225 0.11088 NA FALSE FALSE FALSE
GCST006061 0.0192 0.2408 537409 outcome TRUE reported 19567_1_EGFR_EGFRvIII
EGFR chr14 105897733 0.012859 2.364e-28 34966 TRUE reported
textfile 2 1 TRUE

8851 rs704 A G A G 0.1273 -0.0155 0.49189 NA FALSE FALSE FALSE GCST006061
0.0072 0.03144 537409 outcome TRUE reported 19567_1_EGFR_EGFRvIII EGFR
chr17 28367840 0.00783 1.942e-59 35360 TRUE reported textfile 2 1
TRUE

8852 rs10922098 T C T C -0.1499 -0.009 0.40011 NA FALSE FALSE FALSE
GCST006061 0.0075 0.2275 537409 outcome TRUE reported 13377_3_CYB561D1_C56D1
CYB561D1 chr1 196695521 0.008204 1.386e-74 35329 TRUE reported
textfile 2 1 TRUE

8853 rs116881261 A G A G 0.1836 -0.0065 0.02647 NA FALSE FALSE FALSE
GCST006061 0.0268 0.8075 537409 outcome TRUE reported 13377_3_CYB561D1_C56D1
CYB561D1 chr6 160769484 0.02585 1.224e-12 35381 TRUE reported
textfile 2 1 TRUE

8854 rs2686395 C T C T -0.0813 -0.0077 0.3101 NA FALSE FALSE FALSE
GCST006061 0.0074 0.2965 537409 outcome TRUE reported 13377_3_CYB561D1_C56D1
CYB561D1 chr3 165767231 0.008685 7.902e-21 35330 TRUE reported
textfile 2 1 TRUE

8855 rs75179845 C T C T 0.1733 0.0169 0.06486 NA FALSE FALSE FALSE
GCST006061 0.0134 0.2077 537409 outcome TRUE reported 13377_3_CYB561D1_C56D1
CYB561D1 chr9 133257567 0.016391 3.992e-26 35362 TRUE reported
textfile 2 1 TRUE

8856 rs10922098 T C T C -0.0667 -0.009 0.40011 NA FALSE FALSE FALSE
GCST006061 0.0075 0.2275 537409 outcome TRUE reported 10571_14_MGAT1_MGAT1
MGAT1 chr1 196695521 0.00801 8.314e-17 35326 TRUE reported textfile
2 1 TRUE

8857 rs634501 G A G A 0.0628 0.0037 0.25799 NA FALSE FALSE FALSE
GCST006061 0.0091 0.686 537409 outcome TRUE reported 10571_14_MGAT1_MGAT1
MGAT1 chr5 180791668 0.008987 2.797e-12 35309 TRUE reported
textfile 2 1 TRUE

8858 rs10922098 T C T C -0.1264 -0.009 0.40011 NA FALSE FALSE FALSE
GCST006061 0.0075 0.2275 537409 outcome TRUE reported 12721_4_TAX1BP1_TAXB1
TAX1BP1 chr1 196695521 0.008029 7.669e-56 35329 TRUE reported
textfile 2 1 TRUE

8859 rs10922098 T C T C -0.1036 -0.009 0.40011 NA FALSE FALSE FALSE
GCST006061 0.0075 0.2275 537409 outcome TRUE reported 9887_40_RBBP6_RBBP6
RBBP6 chr1 196695521 0.008201 1.39e-36 35338 TRUE reported
textfile 2 1 TRUE

8860 rs1355538 G A G A -0.0629 -0.0076 0.39847 NA FALSE FALSE FALSE
GCST006061 0.0072 0.2923 537409 outcome TRUE reported 9887_40_RBBP6_RBBP6

RBBP6 chr3 165787389 0.008192 1.61e-14 35338 TRUE reported
textfile 2 1 TRUE
8861 rs10922098 T C T C -0.1285 -0.009 0.40011 NA FALSE FALSE FALSE
GCST006061 0.0075 0.2275 537409 outcome TRUE reported 10890_135_LCTL_LCTL
LCTL chr1 196695521 0.008126 2.498e-56 35327 TRUE reported
textfile 2 1 TRUE
8862 rs10922098 T C T C -0.112 -0.009 0.40011 NA FALSE FALSE FALSE
GCST006061 0.0075 0.2275 537409 outcome TRUE reported
4143_74_XCL1_Lymphotactin XCL1 chr1 196695521 0.008117 2.624e-43 35338
TRUE reported textfile 2 1 TRUE
8863 rs1706435 G A G A 0.0518 0.0117 0.36981 NA FALSE FALSE FALSE
GCST006061 0.0072 0.1047 537409 outcome TRUE reported
4143_74_XCL1_Lymphotactin XCL1 chr3 165743688 0.008238 3.216e-10 35339
TRUE reported textfile 2 1 TRUE
8864 rs10922098 T C T C -0.0805 -0.009 0.40011 NA FALSE FALSE FALSE
GCST006061 0.0075 0.2275 537409 outcome TRUE reported 18933_4_TGM4_TGM4
TGM4 chr1 196695521 0.008056 1.638e-23 35338 TRUE reported
textfile 2 1 TRUE
8865 rs1995640 T C T C -0.0511 0.0015 0.41121 NA FALSE FALSE FALSE
GCST006061 0.0072 0.8365 537409 outcome TRUE reported 18933_4_TGM4_TGM4
TGM4 chr3 44901577 0.008062 2.324e-10 35346 TRUE reported
textfile 2 1 TRUE
8866 rs10922098 T C T C -0.1441 -0.009 0.40011 NA FALSE FALSE FALSE
GCST006061 0.0075 0.2275 537409 outcome TRUE reported 11129_66_BMP15_BMP15
BMP15 chr1 196695521 0.008015 2.884e-72 35329 TRUE reported
textfile 2 1 TRUE
8867 rs1707652 T C T C 0.0813 0.0081 0.3102 NA FALSE FALSE FALSE
GCST006061 0.0074 0.2701 537409 outcome TRUE reported 11129_66_BMP15_BMP15
BMP15 chr3 165761011 0.008482 9.262e-22 35330 TRUE reported
textfile 2 1 TRUE
8868 rs58068569 G A G A -0.0531 -0.0152 0.20781 NA FALSE FALSE FALSE
GCST006061 0.0098 0.1209 537409 outcome TRUE reported 11129_66_BMP15_BMP15
BMP15 chr12 3991507 0.009708 4.505e-08 35368 TRUE reported textfile
2 1 TRUE
8869 rs10922098 T C T C -0.1115 -0.009 0.40011 NA FALSE FALSE FALSE
GCST006061 0.0075 0.2275 537409 outcome TRUE reported 18324_61_SUOX_SUOX
SUOX chr1 196695521 0.008009 4.653e-44 35338 TRUE reported
textfile 2 1 TRUE
8870 rs1355538 G A G A -0.0736 -0.0076 0.39847 NA FALSE FALSE FALSE
GCST006061 0.0072 0.2923 537409 outcome TRUE reported 18324_61_SUOX_SUOX
SUOX chr3 165787389 0.008003 3.705e-20 35338 TRUE reported
textfile 2 1 TRUE
8871 rs10922098 T C T C -0.1045 -0.009 0.40011 NA FALSE FALSE FALSE
GCST006061 0.0075 0.2275 537409 outcome TRUE reported 15580_2_EPHA7_EPHA7
EPHA7 chr1 196695521 0.008051 1.59e-38 35338 TRUE reported
textfile 2 1 TRUE
8872 rs1355538 G A G A -0.0512 -0.0076 0.39847 NA FALSE FALSE FALSE
GCST006061 0.0072 0.2923 537409 outcome TRUE reported 15580_2_EPHA7_EPHA7
EPHA7 chr3 165787389 0.008053 2.046e-10 35338 TRUE reported
textfile 2 1 TRUE
8873 rs6602268 G A G A 0.2612 -0.0129 0.03404 NA FALSE FALSE FALSE
GCST006061 0.0168 0.4437 537409 outcome TRUE reported 15580_2_EPHA7_EPHA7
EPHA7 chr10 7722270 0.022 1.645e-32 35361 TRUE reported textfile 2
1 TRUE
8874 rs10922098 T C T C -0.0751 -0.009 0.40011 NA FALSE FALSE FALSE
GCST006061 0.0075 0.2275 537409 outcome TRUE reported 4717_55_IL3_IL_3 IL3
chr1 196695521 0.008047 1.034e-20 35338 TRUE reported textfile 2
1 TRUE
8875 rs1355538 G A G A -0.0528 -0.0076 0.39847 NA FALSE FALSE FALSE
GCST006061 0.0072 0.2923 537409 outcome TRUE reported 4717_55_IL3_IL_3 IL3
chr3 165787389 0.008029 4.818e-11 35338 TRUE reported textfile 2
1 TRUE
8876 rs10922098 T C T C -0.0581 -0.009 0.40011 NA FALSE FALSE FALSE
GCST006061 0.0075 0.2275 537409 outcome TRUE reported 5103_30_CD200R1_MO2R1
CD200R1 chr1 196695521 0.008168 1.138e-12 35338 TRUE reported
textfile 2 1 TRUE

8877 rs211161 G T G T 0.1727 -0.0176 0.01526 NA FALSE FALSE FALSE GCST006061 0.0295 0.5516 537409 outcome TRUE reported 5103_30_CD200R1_MO2R1 CD200R1 chr6 96614917 0.031577 4.521e-08 35343 TRUE reported textfile 2 1 TRUE
8878 rs62263730 A G A G 0.1152 -0.0051 0.3474 NA FALSE FALSE FALSE GCST006061 0.008 0.5284 537409 outcome TRUE reported 5103_30_CD200R1_MO2R1 CD200R1 chr3 112859161 0.008424 1.429e-42 35363 TRUE reported textfile 2 1 TRUE
8879 rs7137828 T C T C -0.0464 0.0076 0.38049 NA FALSE FALSE FALSE GCST006061 0.0075 0.3144 537409 outcome TRUE reported 5103_30_CD200R1_MO2R1 CD200R1 chr12 111494996 0.008246 1.836e-08 35371 TRUE reported textfile 2 1 TRUE
8880 rs10922098 T C T C -0.0442 -0.009 0.40011 NA FALSE FALSE FALSE GCST006061 0.0075 0.2275 537409 outcome TRUE reported 14039_33_KLK5_kallikrein_5 KLK5 chr1 196695521 0.007952 2.728e-08 35338 TRUE reported textfile 2 1 TRUE
8881 rs117265578 A G A G -0.0766 0.0127 0.08612 NA FALSE FALSE FALSE GCST006061 0.0183 0.4854 537409 outcome TRUE reported 14039_33_KLK5_kallikrein_5 KLK5 chr14 49522779 0.013784 2.745e-08 35362 TRUE reported textfile 2 1 TRUE
8882 rs10922098 T C T C -0.079 -0.009 0.40011 NA FALSE FALSE FALSE GCST006061 0.0075 0.2275 537409 outcome TRUE reported 14624_51_CTCF_CTCF CTCF chr1 196695521 0.008086 1.521e-22 35338 TRUE reported textfile 2 1 TRUE
8883 rs10922098 T C T C -0.1009 -0.009 0.40011 NA FALSE FALSE FALSE GCST006061 0.0075 0.2275 537409 outcome TRUE reported 13085_18_GLP1R_GLP1R GLP1R chr1 196695521 0.008086 9.73e-36 35329 TRUE reported textfile 2 1 TRUE
8884 rs10922098 T C T C -0.1571 -0.009 0.40011 NA FALSE FALSE FALSE GCST006061 0.0075 0.2275 537409 outcome TRUE reported 5246_64_PDE2A_cGMP_stimulated_PDE PDE2A chr1 196695521 0.007914 1.083e-87 35338 TRUE reported textfile 2 1 TRUE
8885 rs1803274 T C T C 0.0572 0.0149 0.1903 NA FALSE FALSE FALSE GCST006061 0.0089 0.09298 537409 outcome TRUE reported 5246_64_PDE2A_cGMP_stimulated_PDE PDE2A chr3 165773492 0.0099 7.559e-09 35339 TRUE reported textfile 2 1 TRUE
8886 rs10922098 T C T C -0.0601 -0.009 0.40011 NA FALSE FALSE FALSE GCST006061 0.0075 0.2275 537409 outcome TRUE reported 5764_4_GALNT2_GALT2 GALNT2 chr1 196695521 0.008076 9.915e-14 35338 TRUE reported textfile 2 1 TRUE
8887 rs17020993 G A G A 0.0792 -0.001 0.11412 NA FALSE FALSE FALSE GCST006061 0.0102 0.924 537409 outcome TRUE reported 5764_4_GALNT2_GALT2 GALNT2 chr1 207115047 0.012469 2.131e-10 35369 TRUE reported textfile 2 1 TRUE
8888 rs10922098 T C T C -0.0796 -0.009 0.40011 NA FALSE FALSE FALSE GCST006061 0.0075 0.2275 537409 outcome TRUE reported 18332_17_CPLX1_CPLX1 CPLX1 chr1 196695521 0.00812 1.089e-22 35338 TRUE reported textfile 2 1 TRUE
8889 rs1355538 G A G A -0.0531 -0.0076 0.39847 NA FALSE FALSE FALSE GCST006061 0.0072 0.2923 537409 outcome TRUE reported 18332_17_CPLX1_CPLX1 CPLX1 chr3 165787389 0.008105 5.707e-11 35338 TRUE reported textfile 2 1 TRUE
8890 rs6884904 A C A C 0.2201 -0.0233 0.01002 NA FALSE FALSE FALSE GCST006061 0.0315 0.4596 537409 outcome TRUE reported 18332_17_CPLX1_CPLX1 CPLX1 chr5 119581786 0.039885 3.421e-08 35363 TRUE reported textfile 2 1 TRUE
8891 rs7687101 T G T G 0.0568 0.0054 0.433 NA FALSE FALSE FALSE GCST006061 0.0073 0.4607 537409 outcome TRUE reported 18332_17_CPLX1_CPLX1 CPLX1 chr4 818891 0.008011 1.336e-12 35277 TRUE reported textfile 2 1 TRUE
8892 rs10922098 T C T C -0.0652 -0.009 0.40011 NA FALSE FALSE FALSE GCST006061 0.0075 0.2275 537409 outcome TRUE reported 7967_38_POTEM_POTEM POTEM chr1 196695521 0.00784 9.1e-17 35338 TRUE reported textfile 2 1 TRUE
8893 rs10922098 T C T C -0.0746 -0.009 0.40011 NA FALSE FALSE FALSE GCST006061 0.0075 0.2275 537409 outcome TRUE reported 11825_27_PPARGC1A_PRGC1

PPARGC1A chr1 196695521 0.008022 1.418e-20 35329 TRUE reported
textfile 2 1 TRUE
8894 rs112731419 T C T C -0.0701 0.0177 0.10616 NA FALSE FALSE FALSE
GCST006061 0.0116 0.1258 537409 outcome TRUE reported 11825_27_PPARGC1A_PRGC1
PPARGC1A chr14 25041311 0.012741 3.761e-08 35368 TRUE reported
textfile 2 1 TRUE
8895 rs6542680 T C T C -0.0576 -0.0012 0.18362 NA FALSE FALSE FALSE
GCST006061 0.0091 0.8935 537409 outcome TRUE reported 11825_27_PPARGC1A_PRGC1
PPARGC1A chr2 3592552 0.010175 1.505e-08 35323 TRUE reported
textfile 2 1 TRUE
8896 rs10922098 T C T C -0.0486 -0.009 0.40011 NA FALSE FALSE FALSE
GCST006061 0.0075 0.2275 537409 outcome TRUE reported 9970_7_UBXN4_UBXN4
UBXN4 chr1 196695521 0.007996 1.214e-09 35338 TRUE reported
textfile 2 1 TRUE
8897 rs117815264 T C T C -0.0869 -0.0584 0.07055 NA FALSE FALSE FALSE
GCST006061 0.0153 0.0001292 537409 outcome TRUE reported 9970_7_UBXN4_UBXN4
UBXN4 chr9 94598328 0.015264 1.248e-08 35349 TRUE reported
textfile 2 1 TRUE
8898 rs12478841 G A G A 0.0567 0.0049 0.21569 NA FALSE FALSE FALSE
GCST006061 0.008 0.5413 537409 outcome TRUE reported 9970_7_UBXN4_UBXN4
UBXN4 chr2 27588855 0.009437 1.873e-09 35373 TRUE reported
textfile 2 1 TRUE
8899 rs1355538 G A G A -0.0442 -0.0076 0.39847 NA FALSE FALSE FALSE
GCST006061 0.0072 0.2923 537409 outcome TRUE reported 9970_7_UBXN4_UBXN4
UBXN4 chr3 165787389 0.007984 3.092e-08 35338 TRUE reported
textfile 2 1 TRUE
8900 rs6986061 C T C T 0.0471 -0.0155 0.39936 NA FALSE FALSE FALSE
GCST006061 0.0075 0.03819 537409 outcome TRUE reported 9970_7_UBXN4_UBXN4
UBXN4 chr8 17780165 0.008038 4.635e-09 35372 TRUE reported
textfile 2 1 TRUE
8901 rs10922098 T C T C -0.1345 -0.009 0.40011 NA FALSE FALSE FALSE
GCST006061 0.0075 0.2275 537409 outcome TRUE reported 16591_71_CHIA_CHIA
CHIA chr1 196695521 0.008085 3.789e-62 35338 TRUE reported
textfile 2 1 TRUE
8902 rs1706435 G A G A 0.0695 0.0117 0.36981 NA FALSE FALSE FALSE
GCST006061 0.0072 0.1047 537409 outcome TRUE reported 16591_71_CHIA_CHIA
CHIA chr3 165743688 0.00821 2.557e-17 35339 TRUE reported textfile
2 1 TRUE
8903 rs58468211 T C T C 0.104 -0.0206 0.04496 NA FALSE FALSE FALSE
GCST006061 0.0182 0.2557 537409 outcome TRUE reported 16591_71_CHIA_CHIA
CHIA chr1 196206772 0.018976 4.242e-08 35336 TRUE reported
textfile 2 1 TRUE
8904 rs10922098 T C T C -0.1003 -0.009 0.40011 NA FALSE FALSE FALSE
GCST006061 0.0075 0.2275 537409 outcome TRUE reported 19572_10_CEBPG_CEBPG
CEBPG chr1 196695521 0.008012 5.876e-36 35338 TRUE reported
textfile 2 1 TRUE
8905 rs11846452 A G A G 0.0563 0.0094 0.19634 NA FALSE FALSE FALSE
GCST006061 0.0114 0.4108 537409 outcome TRUE reported 19572_10_CEBPG_CEBPG
CEBPG chr14 106538667 0.010131 2.743e-08 34966 TRUE reported
textfile 2 1 TRUE
8906 rs62294359 A G A G 0.0564 0.0164 0.24788 NA FALSE FALSE FALSE
GCST006061 0.0081 0.04287 537409 outcome TRUE reported 19572_10_CEBPG_CEBPG
CEBPG chr3 165744429 0.009102 5.777e-10 35339 TRUE reported
textfile 2 1 TRUE
8907 rs10922098 T C T C -0.2162 -0.009 0.40011 NA FALSE FALSE FALSE
GCST006061 0.0075 0.2275 537409 outcome TRUE reported 8334_65_TCP11_TCP11
TCP11 chr1 196695521 0.008135 1.237e-155 35338 TRUE reported
textfile 2 1 TRUE
8908 rs111696008 A G A G -0.148 -0.0259 0.02443 NA FALSE FALSE FALSE
GCST006061 0.0401 0.519 537409 outcome TRUE reported 8334_65_TCP11_TCP11
TCP11 chr2 216622986 0.026588 2.601e-08 35359 TRUE reported
textfile 2 1 TRUE
8909 rs35351085 T C T C 0.103 0.0061 0.04877 NA FALSE FALSE FALSE
GCST006061 0.022 0.7825 537409 outcome TRUE reported 8334_65_TCP11_TCP11
TCP11 chr1 197992799 0.018516 2.655e-08 35343 TRUE reported
textfile 2 1 TRUE

8910 rs6445035 A G A G 0.1502 0.0154 0.1904 NA FALSE FALSE FALSE
GCST006061 0.0089 0.08256 537409 outcome TRUE reported 8334_65_TCP11_TCP11
TCP11 chr3 165762312 0.01016 1.868e-49 35339 TRUE reported textfile
2 1 TRUE
8911 rs704 A G A G 0.2472 -0.0155 0.49189 NA FALSE FALSE FALSE GCST006061
0.0072 0.03144 537409 outcome TRUE reported 8334_65_TCP11_TCP11 TCP11 chr17
28367840 0.007954 1e-200 35360 TRUE reported textfile 2 1 TRUE
8912 rs73169117 T C T C 0.0713 0.0051 0.11405 NA FALSE FALSE FALSE
GCST006061 0.0122 0.6755 537409 outcome TRUE reported 8334_65_TCP11_TCP11
TCP11 chr3 167217620 0.012682 1.884e-08 35354 TRUE reported
textfile 2 1 TRUE
8913 rs74374003 A G A G 0.1706 0.0113 0.02005 NA FALSE FALSE FALSE
GCST006061 0.0473 0.8116 537409 outcome TRUE reported 8334_65_TCP11_TCP11
TCP11 chr7 94451854 0.02796 1.05e-09 35352 TRUE reported textfile
2 1 TRUE
8914 rs854536 A G A G 0.0883 -0.0085 0.44104 NA FALSE FALSE FALSE
GCST006061 0.0073 0.245 537409 outcome TRUE reported 8334_65_TCP11_TCP11
TCP11 chr7 95280587 0.008076 7.916e-28 35349 TRUE reported
textfile 2 1 TRUE
8915 rs10922098 T C T C -0.1341 -0.009 0.40011 NA FALSE FALSE FALSE
GCST006061 0.0075 0.2275 537409 outcome TRUE reported 9116_28_HEPACAM2_HECA2
HEPACAM2 chr1 196695521 0.008099 1.42e-61 35338 TRUE reported
textfile 2 1 TRUE
8916 rs2686395 C T C T -0.0676 -0.0077 0.3101 NA FALSE FALSE FALSE
GCST006061 0.0074 0.2965 537409 outcome TRUE reported 9116_28_HEPACAM2_HECA2
HEPACAM2 chr3 165767231 0.008566 2.973e-15 35339 TRUE reported
textfile 2 1 TRUE
8917 rs10922098 T C T C -0.1931 -0.009 0.40011 NA FALSE FALSE FALSE
GCST006061 0.0075 0.2275 537409 outcome TRUE reported 10546_2_TSPEAR_TSEAR
TSPEAR chr1 196695521 0.008034 1.202e-127 35326 TRUE reported
textfile 2 1 TRUE
8918 rs1451915 A C A C 0.0661 -0.0107 0.13641 NA FALSE FALSE FALSE
GCST006061 0.0124 0.3895 537409 outcome TRUE reported 10546_2_TSPEAR_TSEAR
TSPEAR chr1 196058002 0.011467 8.205e-09 35323 TRUE reported
textfile 2 1 TRUE
8919 rs2686395 C T C T -0.0876 -0.0077 0.3101 NA FALSE FALSE FALSE
GCST006061 0.0074 0.2965 537409 outcome TRUE reported 10546_2_TSPEAR_TSEAR
TSPEAR chr3 165767231 0.008523 8.849e-25 35327 TRUE reported
textfile 2 1 TRUE
8920 rs28645249 C T C T 0.0742 0.019 0.26162 NA FALSE FALSE FALSE
GCST006061 0.0115 0.09815 537409 outcome TRUE reported 10546_2_TSPEAR_TSEAR
TSPEAR chr6 32557458 0.008678 1.224e-17 35426 TRUE reported
textfile 2 1 TRUE
8921 rs10922098 T C T C -0.0726 -0.009 0.40011 NA FALSE FALSE FALSE
GCST006061 0.0075 0.2275 537409 outcome TRUE reported 3374_49_HCK_HCK HCK
chr1 196695521 0.00799 1.028e-19 35338 TRUE reported textfile 2 1
TRUE
8922 rs10922098 T C T C -0.1603 -0.009 0.40011 NA FALSE FALSE FALSE
GCST006061 0.0075 0.2275 537409 outcome TRUE reported 2859_69_HDAC8_HDAC8
HDAC8 chr1 196695521 0.008198 3.765e-85 35338 TRUE reported
textfile 2 1 TRUE
8923 rs1707652 T C T C 0.1243 0.0081 0.3102 NA FALSE FALSE FALSE
GCST006061 0.0074 0.2701 537409 outcome TRUE reported 2859_69_HDAC8_HDAC8
HDAC8 chr3 165761011 0.00867 1.286e-46 35339 TRUE reported textfile
2 1 TRUE
8924 rs71352239 T C T C 0.0613 -0.0041 0.30263 NA FALSE FALSE FALSE
GCST006061 0.0087 0.6372 537409 outcome TRUE reported 2859_69_HDAC8_HDAC8
HDAC8 chr19 44926286 0.008789 3.061e-12 35368 TRUE reported
textfile 2 1 TRUE
8925 rs72952553 A G A G -0.0477 -0.0042 0.31481 NA FALSE FALSE FALSE
GCST006061 0.0085 0.6159 537409 outcome TRUE reported 2859_69_HDAC8_HDAC8
HDAC8 chr2 216616793 0.008699 4.177e-08 35359 TRUE reported
textfile 2 1 TRUE
8926 rs10922098 T C T C -0.122 -0.009 0.40011 NA FALSE FALSE FALSE
GCST006061 0.0075 0.2275 537409 outcome TRUE reported 12585_39_ERCC1_ERCC1
ERCC1 chr1 196695521 0.008084 1.843e-51 35329 TRUE reported

textfile 2 1 TRUE
8927 rs1707652 T C T C 0.062 0.0081 0.3102 NA FALSE FALSE FALSE
GCST006061 0.0074 0.2701 537409 outcome TRUE reported 12585_39_ERCC1_ERCC1
ERCC1 chr3 165761011 0.008558 4.341e-13 35330 TRUE reported
textfile 2 1 TRUE
8928 rs28624101 T C T C 0.0555 0.0242 0.36922 NA FALSE FALSE FALSE
GCST006061 0.0144 0.09211 537409 outcome TRUE reported 12585_39_ERCC1_ERCC1
ERCC1 chr6 32557514 0.007858 1.626e-12 35421 TRUE reported
textfile 2 1 TRUE
8929 rs3136202 A G A G 0.0499 -0.0215 0.33912 NA FALSE FALSE FALSE
GCST006061 0.0075 0.004101 537409 outcome TRUE reported
12585_39_ERCC1_ERCC1 ERCC1 chr16 13944317 0.008379 2.6e-09 35369 TRUE
reported textfile 2 1 TRUE
8930 rs10922098 T C T C -0.0554 -0.009 0.40011 NA FALSE FALSE FALSE
GCST006061 0.0075 0.2275 537409 outcome TRUE reported 10948_14_PLD3_PLD3
PLD3 chr1 196695521 0.008121 9.001e-12 35329 TRUE reported
textfile 2 1 TRUE
8931 rs4631423 T C T C -0.0564 0.0025 0.20582 NA FALSE FALSE FALSE
GCST006061 0.0088 0.7754 537409 outcome TRUE reported 10948_14_PLD3_PLD3
PLD3 chr8 11841770 0.009882 1.149e-08 35368 TRUE reported
textfile 2 1 TRUE
8932 rs7254808 C T C T 0.1354 0.0158 0.04313 NA FALSE FALSE FALSE
GCST006061 0.0213 0.4586 537409 outcome TRUE reported 10948_14_PLD3_PLD3
PLD3 chr19 40364607 0.019646 5.496e-12 35365 TRUE reported
textfile 2 1 TRUE
8933 rs10922098 T C T C -0.0913 -0.009 0.40011 NA FALSE FALSE FALSE
GCST006061 0.0075 0.2275 537409 outcome TRUE reported 4987_17_FCAR_FCAR
FCAR chr1 196695521 0.008028 5.733e-30 35338 TRUE reported
textfile 2 1 TRUE
8934 rs10922098 T C T C -0.0899 -0.009 0.40011 NA FALSE FALSE FALSE
GCST006061 0.0075 0.2275 537409 outcome TRUE reported 8306_54_PRRG1_TMG1
PRRG1 chr1 196695521 0.008025 3.986e-29 35338 TRUE reported
textfile 2 1 TRUE
8935 rs6445035 A G A G 0.0842 0.0154 0.1904 NA FALSE FALSE FALSE
GCST006061 0.0089 0.08256 537409 outcome TRUE reported 8306_54_PRRG1_TMG1
PRRG1 chr3 165762312 0.009995 3.621e-17 35339 TRUE reported
textfile 2 1 TRUE
8936 rs10922098 T C T C -0.0913 -0.009 0.40011 NA FALSE FALSE FALSE
GCST006061 0.0075 0.2275 537409 outcome TRUE reported 13942_140_SPSB1_SPSB1
SPSB1 chr1 196695521 0.008044 7.351e-30 35338 TRUE reported
textfile 2 1 TRUE
8937 rs12478841 G A G A 0.0524 0.0049 0.21569 NA FALSE FALSE FALSE
GCST006061 0.008 0.5413 537409 outcome TRUE reported 13942_140_SPSB1_SPSB1
SPSB1 chr2 27588855 0.0095 3.473e-08 35373 TRUE reported textfile
2 1 TRUE
8938 rs6542680 T C T C -0.0698 -0.0012 0.18362 NA FALSE FALSE FALSE
GCST006061 0.0091 0.8935 537409 outcome TRUE reported 13942_140_SPSB1_SPSB1
SPSB1 chr2 3592552 0.010206 7.971e-12 35332 TRUE reported textfile
2 1 TRUE
8939 rs10922098 T C T C -0.1271 -0.009 0.40011 NA FALSE FALSE FALSE
GCST006061 0.0075 0.2275 537409 outcome TRUE reported 12760_34_ZNF774_ZN774
ZNF774 chr1 196695521 0.008086 1.126e-55 35329 TRUE reported
textfile 2 1 TRUE
8940 rs10922098 T C T C -0.1781 -0.009 0.40011 NA FALSE FALSE FALSE
GCST006061 0.0075 0.2275 537409 outcome TRUE reported 5723_4_INSL3_INSL3
INSL3 chr1 196695521 0.008098 3.269e-107 35338 TRUE reported
textfile 2 1 TRUE
8941 rs11569415 A G A G -0.066 0.0011 0.24276 NA FALSE FALSE FALSE
GCST006061 0.0097 0.9129 537409 outcome TRUE reported 5723_4_INSL3_INSL3
INSL3 chr19 6716268 0.009238 9.06e-13 35372 TRUE reported textfile
2 1 TRUE
8942 rs1707652 T C T C 0.1038 0.0081 0.3102 NA FALSE FALSE FALSE
GCST006061 0.0074 0.2701 537409 outcome TRUE reported 5723_4_INSL3_INSL3
INSL3 chr3 165761011 0.008581 1.093e-33 35339 TRUE reported
textfile 2 1 TRUE
8943 rs704 A G A G 0.0528 -0.0155 0.49189 NA FALSE FALSE FALSE GCST006061

0.0072 0.03144 537409 outcome TRUE reported 5723_4_INSL3_INSL3 INSL3 chr17
28367840 0.007968 3.442e-11 35360 TRUE reported textfile 2 1 TRUE
8944 rs10922098 T C T C -0.1095 -0.009 0.40011 NA FALSE FALSE FALSE
GCST006061 0.0075 0.2275 537409 outcome TRUE reported 18237_29_NRIP3_NRIP3
NRIP3 chr1 196695521 0.00806 4.892e-42 35338 TRUE reported textfile
2 1 TRUE
8945 rs10922098 T C T C -0.0787 -0.009 0.40011 NA FALSE FALSE FALSE
GCST006061 0.0075 0.2275 537409 outcome TRUE reported 13228_75_MDM2_MDM2
MDM2 chr1 196695521 0.007999 7.695e-23 35329 TRUE reported
textfile 2 1 TRUE
8946 rs10922098 T C T C -0.0788 -0.009 0.40011 NA FALSE FALSE FALSE
GCST006061 0.0075 0.2275 537409 outcome TRUE reported 8066_38_SYT9_SYT9
SYT9 chr1 196695521 0.008008 7.554e-23 35338 TRUE reported
textfile 2 1 TRUE
8947 rs10922098 T C T C -0.1533 -0.009 0.40011 NA FALSE FALSE FALSE
GCST006061 0.0075 0.2275 537409 outcome TRUE reported 9936_27_LNX1_LNX1
LNX1 chr1 196695521 0.008093 5.137e-80 35338 TRUE reported
textfile 2 1 TRUE
8948 rs2686395 C T C T -0.0691 -0.0077 0.3101 NA FALSE FALSE FALSE
GCST006061 0.0074 0.2965 537409 outcome TRUE reported 9936_27_LNX1_LNX1
LNX1 chr3 165767231 0.008571 7.525e-16 35339 TRUE reported
textfile 2 1 TRUE
8949 rs10922098 T C T C -0.1262 -0.009 0.40011 NA FALSE FALSE FALSE
GCST006061 0.0075 0.2275 537409 outcome TRUE reported 13429_3_WFDC10A_WF10A
WFDC10A chr1 196695521 0.008074 4.516e-55 35332 TRUE reported
textfile 2 1 TRUE
8950 rs2668202 G A G A -0.0566 -0.0076 0.30991 NA FALSE FALSE FALSE
GCST006061 0.0074 0.3015 537409 outcome TRUE reported 13429_3_WFDC10A_WF10A
WFDC10A chr3 165772680 0.008548 3.555e-11 35333 TRUE reported
textfile 2 1 TRUE
8951 rs10922098 T C T C -0.0715 -0.009 0.40011 NA FALSE FALSE FALSE
GCST006061 0.0075 0.2275 537409 outcome TRUE reported
10511_10_COL6A3_Collagen_alpha_3_VI_ COL6A3 chr1 196695521 0.007922
1.797e-19 35326 TRUE reported textfile 2 1 TRUE
8952 rs139126374 A G A G -0.1696 -0.061 0.02237 NA FALSE FALSE FALSE
GCST006061 0.0339 0.07193 537409 outcome TRUE reported
10511_10_COL6A3_Collagen_alpha_3_VI_ COL6A3 chr2 237000173 0.026132
8.58e-11 35367 TRUE reported textfile 2 1 TRUE
8953 rs10922098 T C T C -0.1349 -0.009 0.40011 NA FALSE FALSE FALSE
GCST006061 0.0075 0.2275 537409 outcome TRUE reported 7917_17_CPD_CBPD CPD
chr1 196695521 0.008108 3.694e-62 35338 TRUE reported textfile 2
1 TRUE
8954 rs1707652 T C T C 0.0912 0.0081 0.3102 NA FALSE FALSE FALSE
GCST006061 0.0074 0.2701 537409 outcome TRUE reported 7917_17_CPD_CBPD CPD
chr3 165761011 0.008575 2.032e-26 35339 TRUE reported textfile 2
1 TRUE
8955 rs10922098 T C T C -0.0812 -0.009 0.40011 NA FALSE FALSE FALSE
GCST006061 0.0075 0.2275 537409 outcome TRUE reported 15692_300_NODAL_NODAL
NODAL chr1 196695521 0.008079 9.167e-24 35338 TRUE reported
textfile 2 1 TRUE
8956 rs1355538 G A G A -0.0485 -0.0076 0.39847 NA FALSE FALSE FALSE
GCST006061 0.0072 0.2923 537409 outcome TRUE reported 15692_300_NODAL_NODAL
NODAL chr3 165787389 0.008067 1.835e-09 35338 TRUE reported
textfile 2 1 TRUE
8957 rs211161 G T G T 0.1781 -0.0176 0.01526 NA FALSE FALSE FALSE
GCST006061 0.0295 0.5516 537409 outcome TRUE reported 15692_300_NODAL_NODAL
NODAL chr6 96614917 0.031257 1.213e-08 35343 TRUE reported
textfile 2 1 TRUE
8958 rs10922098 T C T C -0.1446 -0.009 0.40011 NA FALSE FALSE FALSE
GCST006061 0.0075 0.2275 537409 outcome TRUE reported 12403_30_RAB39B_RB39B
RAB39B chr1 196695521 0.008121 6.401e-71 35329 TRUE reported
textfile 2 1 TRUE
8959 rs1355538 G A G A -0.0781 -0.0076 0.39847 NA FALSE FALSE FALSE
GCST006061 0.0072 0.2923 537409 outcome TRUE reported 12403_30_RAB39B_RB39B
RAB39B chr3 165787389 0.008133 7.801e-22 35329 TRUE reported
textfile 2 1 TRUE

8960 rs35209896 A C A C 0.0779 0.0267 0.09263 NA FALSE FALSE FALSE GCST006061 0.0355 0.4521 537409 outcome TRUE reported 12403_30_RAB39B_RB39B RAB39B chr6 32519009 0.01364 1.121e-08 35430 TRUE reported textfile 2 1 TRUE
8961 rs10922098 T C T C -0.0744 -0.009 0.40011 NA FALSE FALSE FALSE GCST006061 0.0075 0.2275 537409 outcome TRUE reported 19606_28_IHH_ihh IHH chr1 196695521 0.008432 1.114e-18 35338 TRUE reported textfile 2 1 TRUE
8962 rs114006326 T G T G -0.2654 -0.046 0.01107 NA FALSE FALSE FALSE GCST006061 0.0539 0.3936 537409 outcome TRUE reported 19606_28_IHH_ihh IHH chr2 219330488 0.039937 3.024e-11 35369 TRUE reported textfile 2 1 TRUE
8963 rs118146573 A G A G -0.0747 -0.0173 0.15106 NA FALSE FALSE FALSE GCST006061 0.0115 0.1326 537409 outcome TRUE reported 19606_28_IHH_ihh IHH chr16 56967026 0.011524 9.046e-11 35369 TRUE reported textfile 2 1 TRUE
8964 rs1707652 T C T C 0.0793 0.0081 0.3102 NA FALSE FALSE FALSE GCST006061 0.0074 0.2701 537409 outcome TRUE reported 19606_28_IHH_ihh IHH chr3 165761011 0.008896 4.895e-19 35339 TRUE reported textfile 2 1 TRUE
8965 rs10922098 T C T C -0.0755 -0.009 0.40011 NA FALSE FALSE FALSE GCST006061 0.0075 0.2275 537409 outcome TRUE reported 10885_36_C6orf89_CF089 C6orf89 chr1 196695521 0.008085 9.764e-21 35326 TRUE reported textfile 2 1 TRUE
8966 rs117051919 C T C T 0.182 -0.0176 0.01969 NA FALSE FALSE FALSE GCST006061 0.0357 0.6206 537409 outcome TRUE reported 10885_36_C6orf89_CF089 C6orf89 chr16 72487217 0.028733 2.386e-10 35360 TRUE reported textfile 2 1 TRUE
8967 rs1355538 G A G A -0.0592 -0.0076 0.39847 NA FALSE FALSE FALSE GCST006061 0.0072 0.2923 537409 outcome TRUE reported 10885_36_C6orf89_CF089 C6orf89 chr3 165787389 0.008067 2.164e-13 35326 TRUE reported textfile 2 1 TRUE
8968 rs10922098 T C T C -0.1217 -0.009 0.40011 NA FALSE FALSE FALSE GCST006061 0.0075 0.2275 537409 outcome TRUE reported 19279_42_RBP1_CRBP RBP1 chr1 196695521 0.00806 1.617e-51 35338 TRUE reported textfile 2 1 TRUE
8969 rs1706435 G A G A 0.0756 0.0117 0.36981 NA FALSE FALSE FALSE GCST006061 0.0072 0.1047 537409 outcome TRUE reported 19279_42_RBP1_CRBP RBP1 chr3 165743688 0.00818 2.416e-20 35339 TRUE reported textfile 2 1 TRUE
8970 rs2071387 G A G A 0.0901 0.0078 0.20571 NA FALSE FALSE FALSE GCST006061 0.0091 0.3935 537409 outcome TRUE reported 19279_42_RBP1_CRBP RBP1 chr3 139538761 0.009768 2.86e-20 35364 TRUE reported textfile 2 1 TRUE
8971 rs10922098 T C T C -0.1405 -0.009 0.40011 NA FALSE FALSE FALSE GCST006061 0.0075 0.2275 537409 outcome TRUE reported 14599_18_STAB1_STAB1 STAB1 chr1 196695521 0.008095 1.749e-67 35338 TRUE reported textfile 2 1 TRUE
8972 rs12403425 A C A C 0.0502 -0.0064 0.2662 NA FALSE FALSE FALSE GCST006061 0.009 0.4754 537409 outcome TRUE reported 14599_18_STAB1_STAB1 STAB1 chr1 197845693 0.008963 2.13e-08 35337 TRUE reported textfile 2 1 TRUE
8973 rs151301820 A C A C 0.2517 0.0257 0.03801 NA FALSE FALSE FALSE GCST006061 0.0392 0.5121 537409 outcome TRUE reported 14599_18_STAB1_STAB1 STAB1 chr11 71453724 0.020536 1.545e-34 35376 TRUE reported textfile 2 1 TRUE
8974 rs1707652 T C T C 0.0722 0.0081 0.3102 NA FALSE FALSE FALSE GCST006061 0.0074 0.2701 537409 outcome TRUE reported 14599_18_STAB1_STAB1 STAB1 chr3 165761011 0.00857 3.619e-17 35339 TRUE reported textfile 2 1 TRUE
8975 rs35209896 A C A C 0.0793 0.0267 0.09263 NA FALSE FALSE FALSE GCST006061 0.0355 0.4521 537409 outcome TRUE reported 14599_18_STAB1_STAB1 STAB1 chr6 32519009 0.013593 5.42e-09 35439 TRUE reported textfile 2 1 TRUE
8976 rs76745121 C A C A 0.2427 0.0407 0.11086 NA FALSE FALSE FALSE GCST006061 0.0132 0.002047 537409 outcome TRUE reported

14599_18_STAB1_STAB1 STAB1 chr11 72153923 0.012623 2.223e-82 35373
TRUE reported textfile 2 1 TRUE
8977 rs10922098 T C T C -0.0836 -0.009 0.40011 NA FALSE FALSE FALSE
GCST006061 0.0075 0.2275 537409 outcome TRUE reported 12576_21_MAGEA3_MAGE_3
MAGEA3 chr1 196695521 0.008139 9.471e-25 35329 TRUE reported
textfile 2 1 TRUE
8978 rs1355538 G A G A -0.0733 -0.0076 0.39847 NA FALSE FALSE FALSE
GCST006061 0.0072 0.2923 537409 outcome TRUE reported 12576_21_MAGEA3_MAGE_3
MAGEA3 chr3 165787389 0.008125 1.852e-19 35329 TRUE reported
textfile 2 1 TRUE
8979 rs10922098 T C T C -0.1504 -0.009 0.40011 NA FALSE FALSE FALSE
GCST006061 0.0075 0.2275 537409 outcome TRUE reported 9378_6_FIBCD1_FBCD1
FIBCD1 chr1 196695521 0.008085 3.036e-77 35338 TRUE reported
textfile 2 1 TRUE
8980 rs11569415 A G A G -0.0581 0.0011 0.24276 NA FALSE FALSE FALSE
GCST006061 0.0097 0.9129 537409 outcome TRUE reported 9378_6_FIBCD1_FBCD1
FIBCD1 chr19 6716268 0.009218 2.916e-10 35372 TRUE reported textfile
2 1 TRUE
8981 rs2686395 C T C T -0.0712 -0.0077 0.3101 NA FALSE FALSE FALSE
GCST006061 0.0074 0.2965 537409 outcome TRUE reported 9378_6_FIBCD1_FBCD1
FIBCD1 chr3 165767231 0.008561 9.065e-17 35339 TRUE reported
textfile 2 1 TRUE
8982 rs10922098 T C T C -0.1755 -0.009 0.40011 NA FALSE FALSE FALSE
GCST006061 0.0075 0.2275 537409 outcome TRUE reported 17511_10_RPP30_RPP30
RPP30 chr1 196695521 0.008115 1.036e-103 35338 TRUE reported
textfile 2 1 TRUE
8983 rs1706435 G A G A 0.0771 0.0117 0.36981 NA FALSE FALSE FALSE
GCST006061 0.0072 0.1047 537409 outcome TRUE reported 17511_10_RPP30_RPP30
RPP30 chr3 165743688 0.008248 8.952e-21 35339 TRUE reported
textfile 2 1 TRUE
8984 rs10922098 T C T C -0.084 -0.009 0.40011 NA FALSE FALSE FALSE
GCST006061 0.0075 0.2275 537409 outcome TRUE reported 5262_57_BCAR3_BCAR3
BCAR3 chr1 196695521 0.008018 1.101e-25 35338 TRUE reported
textfile 2 1 TRUE
8985 rs12478841 G A G A 0.0546 0.0049 0.21569 NA FALSE FALSE FALSE
GCST006061 0.008 0.5413 537409 outcome TRUE reported 5262_57_BCAR3_BCAR3
BCAR3 chr2 27588855 0.009471 8.171e-09 35373 TRUE reported
textfile 2 1 TRUE
8986 rs6542680 T C T C -0.0789 -0.0012 0.18362 NA FALSE FALSE FALSE
GCST006061 0.0091 0.8935 537409 outcome TRUE reported 5262_57_BCAR3_BCAR3
BCAR3 chr2 3592552 0.010163 8.252e-15 35332 TRUE reported textfile
2 1 TRUE
8987 rs10922098 T C T C -0.1219 -0.009 0.40011 NA FALSE FALSE FALSE
GCST006061 0.0075 0.2275 537409 outcome TRUE reported
13689_2_ODC1_Ornithine_decarboxylase ODC1 chr1 196695521 0.008296
6.979e-49 35338 TRUE reported textfile 2 1 TRUE
8988 rs11036181 G T G T 0.0878 -0.0254 0.07276 NA FALSE FALSE FALSE
GCST006061 0.0193 0.1878 537409 outcome TRUE reported
13689_2_ODC1_Ornithine_decarboxylase ODC1 chr11 5193272 0.015877 3.201e-08
35365 TRUE reported textfile 2 1 TRUE
8989 rs117983753 G A G A 0.2429 -0.059 0.01454 NA FALSE FALSE FALSE
GCST006061 0.0424 0.1642 537409 outcome TRUE reported
13689_2_ODC1_Ornithine_decarboxylase ODC1 chr11 6989764 0.033727 5.938e-13
35380 TRUE reported textfile 2 1 TRUE
8990 rs12791308 C T C T 0.1062 0.0038 0.06554 NA FALSE FALSE FALSE
GCST006061 0.0173 0.8248 537409 outcome TRUE reported
13689_2_ODC1_Ornithine_decarboxylase ODC1 chr11 6385084 0.016139 4.694e-11
35377 TRUE reported textfile 2 1 TRUE
8991 rs13019325 G A G A -0.0564 -0.0075 0.44984 NA FALSE FALSE FALSE
GCST006061 0.0071 0.2908 537409 outcome TRUE reported
13689_2_ODC1_Ornithine_decarboxylase ODC1 chr2 159772479 0.008162
4.836e-12 35350 TRUE reported textfile 2 1 TRUE
8992 rs16914574 C T C T 0.1918 -0.0098 0.04057 NA FALSE FALSE FALSE
GCST006061 0.0233 0.6742 537409 outcome TRUE reported
13689_2_ODC1_Ornithine_decarboxylase ODC1 chr11 6511260 0.020757 2.455e-20
35380 TRUE reported textfile 2 1 TRUE

8993 rs1707652 T C T C 0.0974 0.0081 0.3102 NA FALSE FALSE FALSE
GCST006061 0.0074 0.2701 537409 outcome TRUE reported
13689_2_ODC1_Ornithine_decarboxylase ODC1 chr3 165761011 0.008764
1.08e-28 35339 TRUE reported textfile 2 1 TRUE
8994 rs17242778 C T C T -0.1366 -0.0205 0.0283 NA FALSE FALSE FALSE
GCST006061 0.041 0.6167 537409 outcome TRUE reported
13689_2_ODC1_Ornithine_decarboxylase ODC1 chr11 6438567 0.024267 1.812e-08
35376 TRUE reported textfile 2 1 TRUE
8995 rs10922098 T C T C -0.0921 -0.009 0.40011 NA FALSE FALSE FALSE
GCST006061 0.0075 0.2275 537409 outcome TRUE reported 14583_49_MSTN_Myostatin
MSTN chr1 196695521 0.008008 1.302e-30 35338 TRUE reported
textfile 2 1 TRUE
8996 rs6445035 A G A G 0.0732 0.0154 0.1904 NA FALSE FALSE FALSE
GCST006061 0.0089 0.08256 537409 outcome TRUE reported 14583_49_MSTN_Myostatin
MSTN chr3 165762312 0.009977 2.187e-13 35339 TRUE reported
textfile 2 1 TRUE
8997 rs10922098 T C T C -0.1158 -0.009 0.40011 NA FALSE FALSE FALSE
GCST006061 0.0075 0.2275 537409 outcome TRUE reported 3605_77_MASP1_MASP3
MASP1 chr1 196695521 0.008262 1.254e-44 35338 TRUE reported
textfile 2 1 TRUE
8998 rs1486236 A C A C 0.1151 0.0029 0.34055 NA FALSE FALSE FALSE
GCST006061 0.0076 0.7053 537409 outcome TRUE reported 3605_77_MASP1_MASP3
MASP1 chr2 179874723 0.008522 1.435e-41 35351 TRUE reported
textfile 2 1 TRUE
8999 rs4938107 A G A G -0.1142 0.0023 0.10328 NA FALSE FALSE FALSE
GCST006061 0.0116 0.8429 537409 outcome TRUE reported 3605_77_MASP1_MASP3
MASP1 chr11 114453386 0.013284 8.177e-18 35371 TRUE reported
textfile 2 1 TRUE
9000 rs6445035 A G A G 0.0672 0.0154 0.1904 NA FALSE FALSE FALSE
GCST006061 0.0089 0.08256 537409 outcome TRUE reported 3605_77_MASP1_MASP3
MASP1 chr3 165762312 0.010297 6.75e-11 35339 TRUE reported
textfile 2 1 TRUE
9001 rs698082 T C T C 0.0825 -0.0045 0.39945 NA FALSE FALSE FALSE
GCST006061 0.0075 0.5422 537409 outcome TRUE reported 3605_77_MASP1_MASP3
MASP1 chr3 187281359 0.008282 2.251e-23 35368 TRUE reported
textfile 2 1 TRUE
9002 rs10922098 T C T C -0.0637 -0.009 0.40011 NA FALSE FALSE FALSE
GCST006061 0.0075 0.2275 537409 outcome TRUE reported 13093_6_SECTM1_SECTM1
SECTM1 chr1 196695521 0.008283 1.464e-14 35329 TRUE reported
textfile 2 1 TRUE
9003 rs112070424 G A G A 0.2516 0.0055 0.01146 NA FALSE FALSE FALSE
GCST006061 0.0438 0.8998 537409 outcome TRUE reported 13093_6_SECTM1_SECTM1
SECTM1 chr12 6805285 0.038846 9.367e-11 35382 TRUE reported textfile
2 1 TRUE
9004 rs180677689 T C T C -0.1152 0.0348 0.03917 NA FALSE FALSE FALSE
GCST006061 0.0407 0.3934 537409 outcome TRUE reported 13093_6_SECTM1_SECTM1
SECTM1 chr17 82556862 0.020931 3.717e-08 35014 TRUE reported
textfile 2 1 TRUE
9005 rs4073961 A G A G 0.0652 3e-04 0.49234 NA FALSE FALSE FALSE
GCST006061 0.0076 0.9653 537409 outcome TRUE reported 13093_6_SECTM1_SECTM1
SECTM1 chr17 82386714 0.008131 1.066e-15 35013 TRUE reported
textfile 2 1 TRUE
9006 rs4639017 C T C T -0.0515 -0.0061 0.39283 NA FALSE FALSE FALSE
GCST006061 0.0073 0.4022 537409 outcome TRUE reported 13093_6_SECTM1_SECTM1
SECTM1 chr3 165833296 0.008279 4.953e-10 35330 TRUE reported
textfile 2 1 TRUE
9007 rs4789763 G A G A 0.1848 0.0126 0.48038 NA FALSE FALSE FALSE
GCST006061 0.0075 0.09217 537409 outcome TRUE reported 13093_6_SECTM1_SECTM1
SECTM1 chr17 82331408 0.008118 1.012e-114 35014 TRUE reported
textfile 2 1 TRUE
9008 rs600038 T C T C 0.082 0.0241 0.15311 NA FALSE FALSE FALSE
GCST006061 0.0085 0.004787 537409 outcome TRUE reported
13093_6_SECTM1_SECTM1 SECTM1 chr9 133276354 0.011257 3.237e-13 35362
TRUE reported textfile 2 1 TRUE
9009 rs601338 A G A G -0.1687 0.0056 0.39439 NA FALSE FALSE FALSE
GCST006061 0.0076 0.4559 537409 outcome TRUE reported 13093_6_SECTM1_SECTM1

SECTM1 chr19 48703417 0.008292 5.113e-92 35368 TRUE reported
textfile 2 1 TRUE
9010 rs10922098 T C T C -0.1109 -0.009 0.40011 NA FALSE FALSE FALSE
GCST006061 0.0075 0.2275 537409 outcome TRUE reported 10618_190_LRP2_megalin
LRP2 chr1 196695521 0.008061 4.56e-43 35326 TRUE reported
textfile 2 1 TRUE
9011 rs1706435 G A G A 0.0606 0.0117 0.36981 NA FALSE FALSE FALSE
GCST006061 0.0072 0.1047 537409 outcome TRUE reported 10618_190_LRP2_megalin
LRP2 chr3 165743688 0.00818 1.275e-13 35327 TRUE reported textfile
2 1 TRUE
9012 rs10922098 T C T C -0.0756 -0.009 0.40011 NA FALSE FALSE FALSE
GCST006061 0.0075 0.2275 537409 outcome TRUE reported 12651_21_PDK2_PDK2
PDK2 chr1 196695521 0.007994 3.159e-21 35329 TRUE reported
textfile 2 1 TRUE
9013 rs1355538 G A G A -0.0452 -0.0076 0.39847 NA FALSE FALSE FALSE
GCST006061 0.0072 0.2923 537409 outcome TRUE reported 12651_21_PDK2_PDK2
PDK2 chr3 165787389 0.007984 1.502e-08 35329 TRUE reported
textfile 2 1 TRUE
9014 rs10922098 T C T C -0.1945 -0.009 0.40011 NA FALSE FALSE FALSE
GCST006061 0.0075 0.2275 537409 outcome TRUE reported
18890_227_FGB_Fibrinogen_B FGB chr1 196695521 0.008237 2.757e-123 35338
TRUE reported textfile 2 1 TRUE
9015 rs111696008 A G A G -0.1806 -0.0259 0.02443 NA FALSE FALSE FALSE
GCST006061 0.0401 0.519 537409 outcome TRUE reported
18890_227_FGB_Fibrinogen_B FGB chr2 216622986 0.026891 1.869e-11 35359
TRUE reported textfile 2 1 TRUE
9016 rs144202860 A G A G -0.2083 -0.0629 0.01274 NA FALSE FALSE FALSE
GCST006061 0.0538 0.2423 537409 outcome TRUE reported
18890_227_FGB_Fibrinogen_B FGB chr2 216625740 0.036003 7.224e-09 35359
TRUE reported textfile 2 1 TRUE
9017 rs62290472 A G A G 0.0911 0.011 0.08266 NA FALSE FALSE FALSE
GCST006061 0.0106 0.3013 537409 outcome TRUE reported
18890_227_FGB_Fibrinogen_B FGB chr3 162193597 0.014824 7.973e-10 35340
TRUE reported textfile 2 1 TRUE
9018 rs6445035 A G A G 0.1794 0.0154 0.1904 NA FALSE FALSE FALSE
GCST006061 0.0089 0.08256 537409 outcome TRUE reported
18890_227_FGB_Fibrinogen_B FGB chr3 165762312 0.010271 2.587e-68 35339
TRUE reported textfile 2 1 TRUE
9019 rs704 A G A G 0.0554 -0.0155 0.49189 NA FALSE FALSE FALSE GCST006061
0.0072 0.03144 537409 outcome TRUE reported 18890_227_FGB_Fibrinogen_B FGB
chr17 28367840 0.008101 7.993e-12 35360 TRUE reported textfile 2
1 TRUE
9020 rs71352239 T C T C 0.0583 -0.0041 0.30263 NA FALSE FALSE FALSE
GCST006061 0.0087 0.6372 537409 outcome TRUE reported
18890_227_FGB_Fibrinogen_B FGB chr19 44926286 0.008838 4.203e-11 35368
TRUE reported textfile 2 1 TRUE
9021 rs73169117 T C T C 0.0802 0.0051 0.11405 NA FALSE FALSE FALSE
GCST006061 0.0122 0.6755 537409 outcome TRUE reported
18890_227_FGB_Fibrinogen_B FGB chr3 167217620 0.012846 4.294e-10 35354
TRUE reported textfile 2 1 TRUE
9022 rs76004643 T G T G 0.0558 -0.0069 0.2662 NA FALSE FALSE FALSE
GCST006061 0.009 0.4422 537409 outcome TRUE reported
18890_227_FGB_Fibrinogen_B FGB chr1 197847653 0.00915 1.071e-09 35337 TRUE
reported textfile 2 1 TRUE
9023 rs10922098 T C T C -0.0562 -0.009 0.40011 NA FALSE FALSE FALSE
GCST006061 0.0075 0.2275 537409 outcome TRUE reported 14250_115_BLMH_BLMH
BLMH chr1 196695521 0.007953 1.591e-12 35338 TRUE reported
textfile 2 1 TRUE
9024 rs2304200 A G A G 0.1239 -0.0064 0.10855 NA FALSE FALSE FALSE
GCST006061 0.0146 0.6624 537409 outcome TRUE reported 14250_115_BLMH_BLMH
BLMH chr19 15469847 0.012566 6.211e-23 35379 TRUE reported
textfile 2 1 TRUE
9025 rs6542680 T C T C -0.0637 -0.0012 0.18362 NA FALSE FALSE FALSE
GCST006061 0.0091 0.8935 537409 outcome TRUE reported 14250_115_BLMH_BLMH
BLMH chr2 3592552 0.010086 2.691e-10 35332 TRUE reported textfile
2 1 TRUE

9026 rs10922098 T C T C -0.1506 -0.009 0.40011 NA FALSE FALSE FALSE
GCST006061 0.0075 0.2275 537409 outcome TRUE reported 12533_135_CYTH2_CYH2
CYTH2 chr1 196695521 0.008131 1.353e-76 35329 TRUE reported
textfile 2 1 TRUE
9027 rs1707652 T C T C 0.0851 0.0081 0.3102 NA FALSE FALSE FALSE
GCST006061 0.0074 0.2701 537409 outcome TRUE reported 12533_135_CYTH2_CYH2
CYTH2 chr3 165761011 0.008609 4.859e-23 35330 TRUE reported
textfile 2 1 TRUE
9028 rs10922098 T C T C -0.1344 -0.009 0.40011 NA FALSE FALSE FALSE
GCST006061 0.0075 0.2275 537409 outcome TRUE reported 8951_162_CSPG4_CSPG4
CSPG4 chr1 196695521 0.008038 9.345e-63 35338 TRUE reported
textfile 2 1 TRUE
9029 rs1355538 G A G A -0.0619 -0.0076 0.39847 NA FALSE FALSE FALSE
GCST006061 0.0072 0.2923 537409 outcome TRUE reported 8951_162_CSPG4_CSPG4
CSPG4 chr3 165787389 0.008044 1.41e-14 35338 TRUE reported
textfile 2 1 TRUE
9030 rs10922098 T C T C -0.0497 -0.009 0.40011 NA FALSE FALSE FALSE
GCST006061 0.0075 0.2275 537409 outcome TRUE reported 4203_50_CFL1_Cofilin_1
CFL1 chr1 196695521 0.008198 1.339e-09 35338 TRUE reported
textfile 2 1 TRUE
9031 rs1706417 G A G A 0.0668 0.0121 0.36627 NA FALSE FALSE FALSE
GCST006061 0.0072 0.09303 537409 outcome TRUE reported 4203_50_CFL1_Cofilin_1
CFL1 chr3 165731121 0.008317 9.617e-16 35339 TRUE reported
textfile 2 1 TRUE
9032 rs10922098 T C T C -0.1637 -0.009 0.40011 NA FALSE FALSE FALSE
GCST006061 0.0075 0.2275 537409 outcome TRUE reported 9941_70_PCDHB1_PCDB1
PCDHB1 chr1 196695521 0.008185 5.424e-89 35338 TRUE reported
textfile 2 1 TRUE
9033 rs11569415 A G A G -0.0519 0.0011 0.24276 NA FALSE FALSE FALSE
GCST006061 0.0097 0.9129 537409 outcome TRUE reported 9941_70_PCDHB1_PCDB1
PCDHB1 chr19 6716268 0.00933 2.654e-08 35372 TRUE reported textfile 2
1 TRUE
9034 rs1707652 T C T C 0.0876 0.0081 0.3102 NA FALSE FALSE FALSE
GCST006061 0.0074 0.2701 537409 outcome TRUE reported 9941_70_PCDHB1_PCDB1
PCDHB1 chr3 165761011 0.00867 5.326e-24 35339 TRUE reported textfile
2 1 TRUE
9035 rs10922098 T C T C -0.0819 -0.009 0.40011 NA FALSE FALSE FALSE
GCST006061 0.0075 0.2275 537409 outcome TRUE reported 7740_33_FATE1_FATE1
FATE1 chr1 196695521 0.008126 6.84e-24 35338 TRUE reported
textfile 2 1 TRUE
9036 rs6542680 T C T C -0.0849 -0.0012 0.18362 NA FALSE FALSE FALSE
GCST006061 0.0091 0.8935 537409 outcome TRUE reported 7740_33_FATE1_FATE1
FATE1 chr2 3592552 0.010295 1.624e-16 35332 TRUE reported textfile
2 1 TRUE
9037 rs10922098 T C T C -0.0569 -0.009 0.40011 NA FALSE FALSE FALSE
GCST006061 0.0075 0.2275 537409 outcome TRUE reported 8065_245_PATE4_PATE4
PATE4 chr1 196695521 0.008022 1.312e-12 35338 TRUE reported
textfile 2 1 TRUE
9038 rs665677 G A G A -0.127 -0.0019 0.25206 NA FALSE FALSE FALSE
GCST006061 0.0078 0.8107 537409 outcome TRUE reported 8065_245_PATE4_PATE4
PATE4 chr11 125882266 0.009066 1.392e-44 35373 TRUE reported
textfile 2 1 TRUE
9039 rs10922098 T C T C -0.1373 -0.009 0.40011 NA FALSE FALSE FALSE
GCST006061 0.0075 0.2275 537409 outcome TRUE reported 3376_49_IL17RD_IL_17_RD
IL17RD chr1 196695521 0.008027 1.353e-65 35338 TRUE reported
textfile 2 1 TRUE
9040 rs112835310 T C T C 0.1791 0.0254 0.05481 NA FALSE FALSE FALSE
GCST006061 0.025 0.3093 537409 outcome TRUE reported 3376_49_IL17RD_IL_17_RD
IL17RD chr3 58608517 0.017393 7.267e-25 35357 TRUE reported
textfile 2 1 TRUE
9041 rs116097686 A C A C 0.1941 -0.007 0.01583 NA FALSE FALSE FALSE
GCST006061 0.0293 0.8098 537409 outcome TRUE reported 3376_49_IL17RD_IL_17_RD
IL17RD chr3 55588735 0.031558 7.719e-10 35348 TRUE reported
textfile 2 1 TRUE
9042 rs142146699 T C T C 0.468 -0.0586 0.02157 NA FALSE FALSE FALSE
GCST006061 0.0356 0.09948 537409 outcome TRUE reported 3376_49_IL17RD_IL_17_RD

IL17RD chr3 57991886 0.027379 1.667e-65 35358 TRUE reported
textfile 2 1 TRUE
9043 rs1707652 T C T C 0.054 0.0081 0.3102 NA FALSE FALSE FALSE
GCST006061 0.0074 0.2701 537409 outcome TRUE reported 3376_49_IL17RD_IL_17_RD
IL17RD chr3 165761011 0.00863 3.922e-10 35339 TRUE reported textfile
2 1 TRUE
9044 rs79257513 A G A G -0.1097 -0.0166 0.07417 NA FALSE FALSE FALSE
GCST006061 0.018 0.3569 537409 outcome TRUE reported 3376_49_IL17RD_IL_17_RD
IL17RD chr3 55828749 0.015372 9.582e-13 35350 TRUE reported
textfile 2 1 TRUE
9045 rs9862503 G A G A 0.2599 0.0012 0.3272 NA FALSE FALSE FALSE
GCST006061 0.0076 0.8797 537409 outcome TRUE reported 3376_49_IL17RD_IL_17_RD
IL17RD chr3 57056332 0.008421 1e-200 35357 TRUE reported textfile
2 1 TRUE
9046 rs10922098 T C T C -0.0553 -0.009 0.40011 NA FALSE FALSE FALSE
GCST006061 0.0075 0.2275 537409 outcome TRUE reported 3003_29_NCR3_NKp30
NCR3 chr1 196695521 0.008154 1.187e-11 35338 TRUE reported
textfile 2 1 TRUE
9047 rs3184504 C T C T -0.0579 0.0074 0.37862 NA FALSE FALSE FALSE
GCST006061 0.0075 0.3235 537409 outcome TRUE reported 3003_29_NCR3_NKp30
NCR3 chr12 111446804 0.008239 2.099e-12 35371 TRUE reported
textfile 2 1 TRUE
9048 rs6922431 C T C T -0.2619 0.0081 0.07464 NA FALSE FALSE FALSE
GCST006061 0.015 0.5901 537409 outcome TRUE reported 3003_29_NCR3_NKp30
NCR3 chr6 31497253 0.015212 2.008e-66 35440 TRUE reported
textfile 2 1 TRUE
9049 rs10922098 T C T C -0.1257 -0.009 0.40011 NA FALSE FALSE FALSE
GCST006061 0.0075 0.2275 537409 outcome TRUE reported 10580_14_SFTA2_SFTA2
SFTA2 chr1 196695521 0.008031 3.217e-55 35326 TRUE reported
[truncated: 490,195 more chars]
